# The long non-coding RNA *HOTAIRM1* promotes tumor aggressiveness and radiotherapy resistance in glioblastoma

**DOI:** 10.1038/s41419-021-04146-0

**Published:** 2021-09-28

**Authors:** Ulvi Ahmadov, Daniel Picard, Jasmin Bartl, Manuela Silginer, Marija Trajkovic-Arsic, Nan Qin, Lena Blümel, Marietta Wolter, Jonathan K. M. Lim, David Pauck, Alina Marie Winkelkotte, Marlen Melcher, Maike Langini, Viktoria Marquardt, Felix Sander, Anja Stefanski, Sascha Steltgens, Christina Hassiepen, Anna Kaufhold, Frauke-Dorothee Meyer, Annette Seibt, Lara Kleinesudeik, Anika Hain, Carsten Münk, Christiane Brigitte Knobbe-Thomsen, Alexander Schramm, Ute Fischer, Gabriel Leprivier, Kai Stühler, Simone Fulda, Jens T. Siveke, Felix Distelmaier, Arndt Borkhardt, Michael Weller, Patrick Roth, Guido Reifenberger, Marc Remke

**Affiliations:** 1 Division of Pediatric Neuro-Oncogenomics, German Cancer Research Center (DKFZ), Heidelberg, Germany; 2 German Consortium for Translational Cancer Research (DKTK), partner site Essen/Düsseldorf, Düsseldorf, Germany; 3 Department of Pediatric Oncology, Hematology, and Clinical Immunology, Medical Faculty, University Hospital Düsseldorf, Düsseldorf, Germany; 4 Department of Neuropathology, Medical Faculty, Heinrich Heine University, Düsseldorf, Germany; 5 Department of Neurology, University Hospital and University of Zurich, Zurich, Switzerland; 6 Bridge Institute of Experimental Tumor Therapy, West German Cancer Center, University Medicine Essen, Essen, Germany; 7 Division of Solid Tumor Translational Oncology, German Cancer Research Center (DKFZ) and German Cancer Consortium (DKTK), partner site Essen, Heidelberg, Germany; 8 Department of General Pediatrics, Neonatology and Pediatric Cardiology, University Hospital Düsseldorf, Düsseldorf, Germany; 9 Institute for Molecular Medicine I, Medical Faculty, Heinrich Heine University, Düsseldorf, Germany; 10 Molecular Proteomics Laboratory (MPL), Biological-Medical Research Center (BMFZ), Heinrich Heine University, Düsseldorf, Germany; 11 Department of Molecular Oncology, West German Cancer Center, University Hospital Essen, Essen, Germany; 12 Institute for Experimental Cancer Research in Pediatrics, Goethe University Frankfurt, Frankfurt, Germany; 13 German Cancer Consortium (DKTK), Partner Site Frankfurt and German Cancer Research Center (DKFZ), Heidelberg, Germany; 14 Clinic for Gastroenterology, Hepatology, and Infectiology, Medical Faculty, Heinrich Heine University, Düsseldorf, Germany

**Keywords:** Cancer genomics, Long non-coding RNAs, Proteomics, Transcriptomics

## Abstract

Glioblastoma is the most common malignant primary brain tumor. To date, clinically relevant biomarkers are restricted to isocitrate dehydrogenase (IDH) gene 1 or 2 mutations and O6-methylguanine DNA methyltransferase (*MGMT*) promoter methylation. Long non-coding RNAs (lncRNAs) have been shown to contribute to glioblastoma pathogenesis and could potentially serve as novel biomarkers. The clinical significance of *HOXA* Transcript Antisense RNA, Myeloid-Specific 1 (*HOTAIRM1*) was determined by analyzing *HOTAIRM1* in multiple glioblastoma gene expression data sets for associations with prognosis, as well as, IDH mutation and *MGMT* promoter methylation status. Finally, the role of *HOTAIRM1* in glioblastoma biology and radiotherapy resistance was characterized in vitro and in vivo. We identified *HOTAIRM1* as a candidate lncRNA whose up-regulation is significantly associated with shorter survival of glioblastoma patients, independent from IDH mutation and *MGMT* promoter methylation. Glioblastoma cell line models uniformly showed reduced cell viability, decreased invasive growth and diminished colony formation capacity upon *HOTAIRM1* down-regulation. Integrated proteogenomic analyses revealed impaired mitochondrial function and determination of reactive oxygen species (ROS) levels confirmed increased ROS levels upon *HOTAIRM1* knock-down. *HOTAIRM1* knock-down decreased expression of transglutaminase 2 (TGM2), a candidate protein implicated in mitochondrial function, and knock-down of *TGM2* mimicked the phenotype of *HOTAIRM1* down-regulation in glioblastoma cells. Moreover, *HOTAIRM1* modulates radiosensitivity of glioblastoma cells both in vitro and in vivo. Our data support a role for *HOTAIRM1* as a driver of biological aggressiveness, radioresistance and poor outcome in glioblastoma. Targeting *HOTAIRM1* may be a promising new therapeutic approach.

## Introduction

Glioblastoma is the most malignant type of astrocytic glioma and the most common malignant primary brain tumor [1]. According to the World Health Organization (WHO) classification of central nervous system tumors, glioblastomas correspond to WHO grade IV and are stratified based on their isocitrate dehydrogenase (IDH) 1 or 2 gene mutation status into two biologically and clinically distinct entities [2]. IDH-wildtype glioblastomas account for more than 90% of the tumors and preferentially manifest *de novo* with short clinical history in patients older than 50 years. In contrast, IDH-mutant glioblastomas, which have recently been re-named as astrocytoma, IDH-mutant, WHO grade 4 [3], are less common, typically occur in patients younger than 50 years and may develop from pre-existing IDH-mutant lower grade astrocytomas [2, 4]. Current standard therapy of glioblastoma consists of surgical resection followed by radiotherapy with concomitant and maintenance temozolomide (TMZ) chemotherapy [5]. Recently, tumor treatment fields (TTF) have been reported and approved as an additional treatment [6]. However, the outcome of IDH-wildtype glioblastoma patients remains poor, with median overall survival limited to 15-18 months and reported five-year survival rate of less than 10% [7]. The majority of glioblastomas demonstrate remarkable resistance to radiation and chemotherapy either upfront or during the course of treatment [8, 9]. Only a few alterations have been established as clinically relevant biomarkers to date, including IDH mutation as a diagnostic and prognostic biomarker, as well as aberrant promoter methylation of the *O6-methylguanine DNA methyltransferase* (*MGMT*) gene as a predictor of benefit from TMZ [10–12]. However, clinically active molecularly targeted therapy approaches are still missing [13].

To further elucidate the molecular pathogenesis of glioblastoma, we evaluated long non-coding RNA (lncRNA) expression profiles in microarray-based gene expression data sets. LncRNAs are non-coding transcripts that are longer than 200 nucleotides [14]. Recent reports have indicated important roles of aberrant lncRNA expression in tumorigenesis, progression and therapy resistance of various cancers [15–17]. We identified the lncRNA *HOXA transcript antisense RNA myeloid-specific 1* (*HOTAIRM1*) as a candidate lncRNA which maps within the *HOXA* gene cluster on the short arm of chromosome 7 and was originally implicated in myelopoiesis through modulation of gene expression in the HOXA cluster [18]. Previous studies have reported that *HOTAIRM1* expression is increased in high-grade gliomas and in recurrent compared to primary glioblastomas [19, 20]. Recently, *HOTAIRM1* has been shown to promote glioma growth and invasion through up-regulation of *HOXA1* expression [21], through long-range chromatin interactions within HOXA cluster genes [22] and regulating *SNAI2* [23]. Moreover, *HOTAIRM1* has been proposed to promote glioma growth by acting as a sponge for several tumor suppressive miRNAs [24–26]. Here, we extend these findings by providing further clinical and functional evidence implicating *HOTAIRM1* as a driver of tumor aggressiveness that contributes to radioresistance and poor outcome of glioblastoma patients.

## Material and methods

### Cell culture

The glioblastoma cell lines T98G, U251MG and U87MG were obtained from American Type Culture Collection (ATCC, Manassas, VA, USA). LN-18 and LN-229 cell lines were provided by Dr. M. Hegi, Lausanne, while the SF126 cell line was obtained from the Japanese Collection of Research Bioresources Cell Bank (JCRB, Osaka, Japan). All lines were authenticated by short tandem repeat (STR) profiling and tested for mycoplasma contamination. Cell lines were grown in Dulbecco’s modified Eagle’s medium (#31966-021, DMEM, Thermo Fischer Scientific, Waltham, MA, USA) supplemented with 10% heat–inactivated fetal bovine serum (FBS, #F9665, Sigma-Aldrich, St. Louis, MO, USA) and 1% penicillin-streptomycin (#P4333, Sigma-Aldrich) in a humidified atmosphere with 5% CO_2_ atmosphere at 37 °C. Stable cells were grown and/or selected with the culture media containing either blasticidin [T98G, LN-18, SF126 and U87MG] (20 µg/ml, #ant-bl-1, InvivoGen, San Diego, CA, USA) or puromycin [LN-229and U251MG] (2 µg/ml, #ant-pr-5, InvivoGen).

### RNA preparation and real-time quantitative PCR

Total RNA extraction was performed by using TRIzol (#15596026, Thermo Fischer Scientific) or by using a Maxwell RSC instrument (#AS1340, RSC simplyRNA Tissue, Promega, Madison, WI, USA). Reverse transcription of total RNA (1 µg) was carried out using the MMLV-RT kit (#M3683, Promega) with random hexamers. Real-time quantitative PCR (RT-qPCR) was subsequently performed with a 1:10 dilution of reverse-transcribed cDNA using a CFX384 Touch™ Real-Time PCR Detection System (Bio-Rad, Hercules, CA, USA). RT-qPCR TagMan Universal Master Mix II (#4440038, Thermo Fischer Scientific) was employed, with TagMan probes specific for *HOTAIRM1*- (exon 1-3, #Hs.PT.58.45434173, Integrated DNA Technologies (IDT), Coralville, IA, USA), *TGM2* (#Hs.PT.58.23141755, IDT) or *PGK1* (#Hs.PT.58 v.606641, IDT). RT-qPCR results were evaluated by 2^−ΔΔ*Ct*^ method [27] using *PGK1* expression levels as a housekeeping gene control.

### Generation of *HOTAIRM1* or *TGM2* knock-down glioblastoma cells

For transient knock-down of *HOTAIRM1* and *TGM2*,T98G, LN-229, LN-18, SF126 or U251 glioblastoma cells were seeded (100,000 cells per well) into 6-well plates the day before knock-down. Specific knock-down of *HOTAIRM1* or *TGM2* and the corresponding non-target negative controls was achieved by using *HOTAIRM1*-specific siPOOLs (#100506311, siTOOLs Technology, Planegg, Martinsried, Germany), *TGM2*-specific siPOOLs (#7052 - TGM2 (human), siTOOLs Technology) and non-target siPOOLs (Neg. control siPOOL 5 nmol, siPOOL Technology), respectively. Transfection of siPOOLs was performed using Lipofectamine RNAiMAX Transfection Reagent (#13778150, Thermo Fischer Scientific). Briefly, 7.5 μl RNAiMAX Transfection Reagent was diluted in 125 μl Opti-MEM (#31985062, Thermo Fischer Scientific) pipetted into a well with 0.5 μl (5 pM) siPOOL in 125 μl Opti-MEM.

Lentivirus-containing *HOTAIRM1* shRNA constructs (sequence is 5′-GGAGACTGGTAGCTTATTAAA-3′) and non-target negative control shRNA constructs (sequence is 5′-CCTAAGGTTAAGTCGCCCTCG-3’) were obtained from IDT and the sequences were obtain from a previous study [28]. HOTAIRM1-pLK0.1-TRC plasmid and third generation lentiviral packing plasmids (pMDL/pRRE, pRSV-Rev and pMD2.G) were transfected into HEK-293T cells using polyethylenimine (#408727, PEI, Sigma-Aldrich). Fresh culture media was added (without antibiotics) after 24 and 48 h post transfection. The virus-containing medium (48 and 72 h after transfection) was stored at −80 °C. Glioblastoma cells (LN-229, LN-18, SF126 and U87MG) were seeded in 6-well plates the day before transduction and were infected with 1.5 ml of the viral suspension containing DMEM, 10% FBS and 2 µg/ml polybrene (#107689, hexadimethrine bromide, Sigma-Aldrich) for stable cell line generation. The virus-containing medium was removed 24 h post transduction and replaced with medium containing puromycin or blasticidin for at least a week.

### Determination of cell viability

Cells were seeded (1000-4000 cells per well) into white-bottom 96-well plates (#136101, Thermo Fischer Scientific) and incubated for 72 h. Afterwards, cells were incubated with sterile 100 μl of 1:1 diluted (with PBS) CellTiter-Glo (#G7570, Promega) for 10 min at RT, shaken for 2 min and the absorbance was measured with a Spark 10 M microplate reader (Tecan, Maennedorf, Switzerland). All experiments were independently repeated at least three times.

### Determination of cell invasion in vitro

Corning BioCoat™ Matrigel Invasion Chambers (#354480, Corning, Bedford, MA, USA) were used to determine invasive capacity of glioblastoma cells in vitro. Transwell membranes were activated by adding 500 μl serum-free medium for 2 h at room temperature. Afterwards, 750 μl medium containing 10 % FBS was added into the lower chamber. 2.5 × 10^5^ cells in 500 μl were resuspended in serum-free medium and were added to the upper chamber. The chambers were removed after 48 h incubation (at 37 °C), the medium was removed and the membrane was washed once with PBS. Next, the cells that had migrated across the polycarbonate membrane were fixed with methanol for 2 min and stained with 1% toluidine blue for 2 min. The membrane was washed 4 times with distilled water and cells remaining in the upper chamber were removed using a cotton swab. Membranes were allowed to air dry for a minimum of one hour before being mounted with vectashield mounting medium (#H-1000, Vector laboratories, Burlingame, CA, USA) on a glass slide. Finally, six random fields were selected and imaged (20X) using an Axiovert 200 microscope (Zeiss, Oberkochen, Germany) and the AxioVision (Version 4.8) software. Invading cells were counted for each membrane. All experiments were independently repeated at least three times.

### Determination of colony formation and in vitro radiosensitivity

Cells were harvested using trypsin, counted with Vi-CELL XR (Beckman Coulter, Brea, CA, USA), plated on culture-treated 100 mm dishes (500 − 1000 cells per plate) and cultured for 21 days. At the end of the incubation period, media was removed from dishes and cells were washed once in PBS and fixed with 10% formalin for 45 min. Cells were then stained with 0.1% crystal violet for 1 h, washed in H_2_O to remove excess dye and were allowed to air dry overnight. The following day, colonies that were visible to the naked eye were counted. All experiments were repeated at least three times.

For the in vitro radiation assay, cells were irradiated at 2 and 4 Gy using a Gulmay RS225 irradiation machine (Gulmay GmbH, Krefeld, Germany). Afterwards, the colony formation assay was performed as described above to evaluate the effect of the irradiation. For determing the efect of radiation on *HOTAIRM1* expression, 4 Gy irradiated cells were seeded (100,000 cells per well) into 6-well plates and samples were harvested 48 h post-seeding. HOTAIRM1 transcript levels were measured using qRT-PCR and results were validated using published data GSE153982 and GSE111247 [29].

### miRNA-175-5p transient over expression

For transient over-expression of miR-17-5p mimic, 2uL of hsa-miR-17-5p mirVana mimic (50μM concentration; #4464066, Ambion) or mirVana miRNA mimic negative control #1 (50μM concentration; #4464058, Ambion) was mixed with 2uL of Lipofectamine 2000 Transfection Reagent (#11668019, Thermo Fischer Scientific) in 100μL of Opti-MEM (#31985062, Thermo Fischer Scientific), and incubated for 15 min.

Each transfection mix was placed into an individual well of a 6-well plate followed by addition of a 2 mL cell suspension of LN-229 glioblastoma cells with a final seeding density of 150,000 cells per well. Transfections were harvested 72 h post-transfection.

### Luciferase reporter assay

Plasmid insert (hg38, chr20:38,137,943-38,138,030), corresponding to the predicted miR-17-5p binding site within the 3’UTR of *TGM2*, was subcloned into psiCHECK-2 vector (Promega, # C8021). The miR-17-5p binding site 5’-gtcctaagCACTTTataaa-3’ was mutated to 5’- gtcctaagAAAAAAataaa-3’. The correctness of insert orientations was confirmed by sequencing. The reporter activity was measured by using Dual-Luciferase Reporter Assay System (Promega, #E1910) according to the manufacturer’s instructions. The 3’UTR- Luciferase reporter gene assays were performed as described previously (Wolter et al., 2016) except for transfecting cells with 50 nM of miRNA mimic hsa-miR-17-5p (ThermoFisher Scientific, # 4464066) and control cells with 50 nM of the miRNA Negative Control #1 (ThermoFisher Scientific, # 4464058).

### In vivo mouse experiments

All animal experiments were performed in accordance with the guidelines of Swiss federal law on animal protection. CD1 Foxn1 nude mice were purchased from Charles River Laboratories (Wilmington, MA, USA). Eleven 6-10-week-old mice per group were used in all experiments. Mice were anaesthetized using an intraperitoneal 3 component injection consisting of fentanyl, midazolam and medetomidin, fixed under a stereotactic device (Stoelting, Wood Dale, IL, USA) and a burr hole was drilled in the skull 2 mm lateral and 1 mm posterior to the bregma. A Hamilton syringe needle was introduced to a depth of 3 mm and LN-229 human glioma cells (75,000) in a volume of 2 µl phosphate-buffered saline (PBS) were injected into the right striatum. Local cranial radiotherapy with a single dose of 12 Gy was performed at day 15 after tumor implantation using a Gulmay 200 kV X-ray unit at 1 Gy/min at room temperature. The mice were observed daily and euthanized when neurological symptoms developed. No blinding was done for mouse experiments.

### Western blot analysis

Cells were lysed and and total proteins were extracted using RIPA lysis buffer (#20-188, Merck Millipore, Burlington, MA, USA) supplemented with protease and phosphatase inhibitor cocktail from Roche (#04693132001 and #04906837001, Sigma-Aldrich). Proteins were quantified with the Bradford method using the Protein Assay Dye Reagent (#500-0006, Bio-Rad). Samples were separated by SDS-PAGE and transferred to a nitrocellulose membrane (#10600002, Sigma-Aldrich) by wet blot using the Mini Gel Tank and Blot Module (#A25977 and #B1000, Thermo Fischer Scientific). The membrane was incubated with rabbit anti-TGM2 (#3557 S, D11A6, 1:1000, Cell Signaling, Danvers, MA, USA), and mouse anti-Actin B (#MAB1501, clone 4, 1:5000, Merck Millipore) primary antibodies overnight at 4 °C. Next, the membrane incubated with species-specific, peroxidase-coupled secondary antibodies (anti-rabbit-HRP, #7074 S, 1:5000, Cell Signaling or anti-mouse-HRP, #H2014, 1:5000, Santa Cruz Biotechnology, Dallas, TX, USA) for an hour at RT. Finally, proteins were visualized using the SuperSignal West Femto Maximum Sensitivity Substrate (#34095, Thermo Fischer Scientific) and detected using the LAS-3000 Imaging System (Fujifilm, Minato, Tokyo, Japan).

### RNA sequencing

Total RNA was isolated from siRNA-mediated *HOTAIRM1* knock-down and control cells of T98G, LN-229 and U251 glioblastoma cell lines 72 h post-transfection using TRIZol reagent. 500 ng total RNA was processed using the TruSeq RNA Sample Preparation v2 kit (low-throughput protocol; Illumina, San Diego, CA, USA) to prepare the barcoded libraries. Libraries were validated and quantified using either DNA 1000 or high-sensitivity chips on a Bioanalyzer (Agilent, Santa Clara, CA, USA). 7.5 pM denatured libraries were input into cBot (Illumina), followed by deep sequencing using HiSeq 2500 (Illumina) for 101 cycles, with an additional seven cycles for index reading. Fastq files were imported into Partek Flow (Partek Incorporated, St. Louis, MO, USA). Quality analysis and quality control were performed on all reads to assess read quality and to determine the amount of trimming required (both ends: 13 bases 5´ and 1 base 3´). Trimmed reads were aligned against the hg38 genome using the STAR v2.4.1d aligner. Unaligned reads were further processed using Bowtie 2 v2.2.5 aligner. Finally, aligned reads were combined before quantifying the expression against the ENSEMBL (release 84) database using the Partek Expectation-Maximization algorithm. Partek Flow default settings were used in all analyses. RNA sequencing data has been deposited in the NCBI GEO dataset repository under the identifyer GSE152147.

### Mass spectrometry-based proteome analyses

For mass spectrometry (MS)-based proteome analyses, proteins were extracted from frozen cell pellets from siRNA-mediated *HOTAIRM1* knock-down and control cells of the T98G, LN-229 and U251 cell lines. In addition, proteins were extracted from shRNA-mediated *HOTAIRM1* knock-down and control cells of LN-229 cells as described. MS-based proteome analyses were performed as described before [30]. Cells were homogenized in urea buffer with a TissueLyser (Qiagen, Hilden, Germany) and subsequent sonication. After centrifugation for 15 min at 14,000 x g and 4 °C, supernatants were collected. Protein concentration was determined via Pierce 660 nm Protein Assay (Thermo Fischer Scientific) and 10 µg protein per sample were desalted through electrophoretic migration at 50 V for 10 min on a 4 −12 % Bis-Tris polyacrylamide gel (#EC62352BOX, Novex NuPAGE, Thermo Fischer Scientific). After silver staining, protein bands were cut out, reduced, alkylated and digested with trypsin before peptide extraction via sonication. Peptides were dissolved and diluted with 0.1 % TFA (v/v).

For MS-based proteome analyses, 15 µL peptide solution per sample was analyzed on a nano-high-performance liquid chromatography electrospray ionization mass spectrometer. The analytical system was composed of an RSLCnano U3000 HPLC coupled to a QExactive Plus mass spectrometer via a nano-electrospray ion source (Thermo Fischer Scientific). Injected peptides were concentrated and desalted at a flow rate of 6 µL/min on a trapping column (Acclaim PepMao C18, 2 cm×100 µm x 3 µm particle size, 100 Å pore size, Thermo Fischer Scientific) with 0.1 % TFA (v/v) for 10 min. Subsequently, peptides were separated at a constant flowrate of 300 nL/min over a 120 min gradient on an analytical column (Acclaim PepMap RSLC C18, 25 cm×75 µm x 2 µm particle size, 100 Å pore size, Thermo Fischer Scientific) at 60 °C. Separation was achieved through a gradient from 4 to 40% solvent B (solvent A: 0.1% (v/v) formic acid in water, solvent B: 0.1% (v/v) formic acid, 84% (v/v) acetonitrile in water). Afterwards, peptides were ionized at a voltage of 1,400 V and introduced into the mass spectrometer operated in positive mode. Mass spectrometry scans were recorded in profile mode in a range from 350-2000 m/z at a resolution of 70,000, while tandem mass spectra were recorded at a resolution of 17,500. Tandem mass spectra were recorded with a data-dependent Top10 method and 30% normalized collision energy. Dynamic exclusion was activated with a repeat count of 1 for 100 s.

Proteome Discoverer (version 1.4.1.14, Thermo Fisher Scientific) was applied for peptide/protein identification with Mascot (version 2.4, Matrix Science, London, UK) as search engine employing the UniProt database (human; including isoforms; date 2016-03-01). A false discovery rate of 1% (*p* ≤ 0.01) on peptide level was set as identification threshold. Proteins were quantified with Progenesis QI for Proteomics (Version 2.0, Nonlinear Dynamics, Waters Corporation, Newcastle upon Tyne, UK). The mass spectrometry proteomics data has been deposited to the ProteomeXchange Consortium via the PRIDE [31] partner repository with the data set identifier PXD020141.

### Determination of superoxide levels

Cellular and mitochondrial superoxide levels were determined by labeling cells with dihydroethidium (HEt, 10 µM, 10 min, and 37 °C; #D11347, Thermo Fischer Scientific) or the mitochondria-targeted variant MitoSOX ™ Red (5 μM, 10 min, 37 °C; #M36008, Thermo Fischer Scientific) as described elsewhere [32]. The staining reactions were stopped by washing the cells three times with PBS. The red fluorescence was documented using an Axio Observer Z1 microscope (Zeiss) with the dihydroethidium filter set (F39-500, F48-515, F47-895; AHF Analysetechnik, Tuebingen, Germany). Images were analyzed and fluorescence intensity was quantified using ImageJ software (Wayne Rasband at the National Institutes of Health; http://rsbweb.nih.gov/ij/).

### Bioinformatic and statistical analyses

Statistical analyses were preformed by using GraphPad Prism (version 5.0) (https://www.graphpad.com/scientific-software/prism/). Experimental data are represented as mean ± SEM based on at least three independent experiments. The two-way ANOVA test was used for statistical analyses of qRT-PCR, viability, invasion, colony formation and reactive oxygen species (ROS) staining assays. Paired T-tests or Mann-Whitney tests (non-parametric t-test) were used for comparisons between two groups for statistical analysis of in vitro radiation, western blotting quantification and N-acetyl cysteine (NAC) assays. Differences between groups were considered statistically significant at *p* < 0.05. Kaplan-Meier survival analysis were calculated using the Log Rank method.

Protein-coding genes were filtered out from the Affymetrix U133 Plus 2 array data leaving a final count of 2858 lncRNAs. Initial identification of lncRNAs in glioblastoma samples from long-term (> 36 months overall survival) versus short-term survivors (< 12 months overall survival) was carried out using GSE53733 data set based on a prospective cohort of the German Glioma Network [33], excluding the data from patients with intermediate overall survival between 12 and 36 months.

Kaplan-Meier survival curves were generated using both the GSE16011 dataset (Affymetrix U133 Plus 2 array filtered for glioblastoma samples, analyzed using R2 platform (Academic Medical Center (AMC) Amsterdam, the Netherlands)) and the TCGA dataset (Affymetrix Human Exon 1.0 ST array, https://www.cancer.gov/tcga, analyzed using IBM SPSS statistics (version 21 IBM Corporation)). The last quartile was used to define high *HOTAIRM1* expression.

Analysis of chromosome 7 gene expression relative to chromosome 7 copy number status was performed by plotting expression fold change according to disomy 7 versus trisomy 7 based on U133 Plus 2 glioblastoma datasets with copy number information (n = 29) taken from GSE7696, GSE36245 and GSE43289. In addition TCGA samples from the Affymetrix Human Exon 1.0 ST array were used in separate analyses with samples of undetermined copy number status being removed.

GeneSet Enrichment Analysis was performed using the t-value from the paired t-test for both RNA sequencing and proteomics data of the siRNA-mediated knockdown and respective controls. Gene sets were comprised of curated pathways from several databases including GO, Reactome, KEGG (March 24 2016 version; http://download.baderlab.org/EM_Genesets/current_release/Human/symbol/) and visualized using Cytoscape (www.cytoscape.org; *p* ≤ 0.001, *q* ≤ 0.05, similarity cutoff 0.5).

MicroRNA (miRNA) predicted to bind to *HOTAIRM1* and either of the 12 candidate proteins that were down-regulated after stable *HOTAIRM1* in LN-229 glioblastoma cells were identified using the miRanda tool (https://omictools.com/miranda-tool). Among these miRNAS, we selected those that showed inverse expression relative to *HOTAIRM1* in the investigated TCGA data set (https://www.cancer.gov/tcga).

*TGM2* promoter methylation status was investigated using a TCGA data set profiled using an illumina 450 K methylation array (https://www.cancer.gov/tcga). Only a small subset of samples have *HOTAIRM1* status since sample overlap was minimal between expression and methylation data.

## Results

### High *HOTAIRM1* expression is associated with shorter survival of glioblastoma patients

**Fig. 1:**
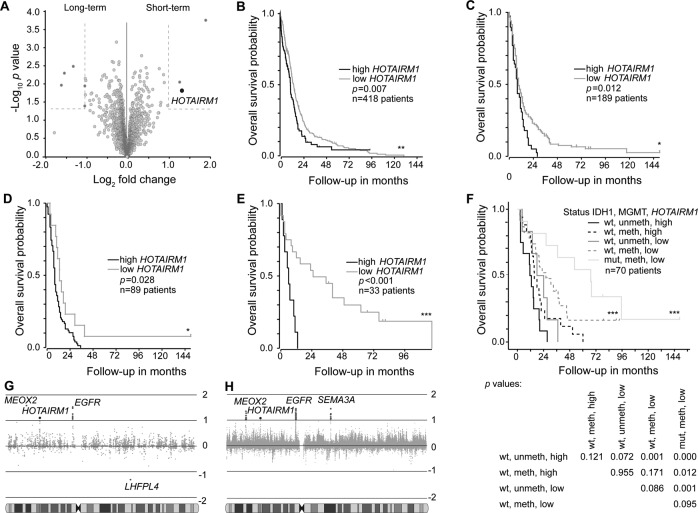
Prognostic role of *HOTAIRM1* expression in glioblastoma patient datasets. **A** Volcano plot showing differential expression of lncRNAs in glioblastomas from patients with long-term (overall survival > 36 months) versus short-term (overall survival < 12 months) survival in the German Glioma Network (GGN) cohort^24^. The black circle highlights *HOTAIRM1* while dark gray circles represent other lncRNAs with differential expression between survival groups (±2-fold change and *p* < 0.05). Light gray circles indicate lncRNAs that are not significant. **B** Overall survival plots of glioblastoma patients from TCGA [34] (https://www.cancer.gov/tcga) and **C** Gravendeel et al. [35] stratified according to high or low *HOTAIRM1* expression levels. Cut-off for high *HOTAIRM1* was determined by upper quartile and log rank statistics were calculated. **D**, **E** Overall survival of glioblastoma patients in the Gravendeel et al. [35] cohort according to *HOTAIRM1* expression in *IDH1-*wildtype (**D**) and *IDH1*-mutant glioblastomas (**E**). **F** Overall survival of glioblastoma patients in the GGN cohort [33] stratified according to *HOTAIRM1* expression, *MGMT* promoter methylation status, and *IDH1* mutation status (wt: wild-type; mut: mutant; meth: methylated; unmeth: unmethylated). The table below the Kaplan-Meier graph lists p-values for the individual subgroups. **G**, **H** Expression of genes mapping to chromosome 7 in glioblastomas stratified according to presence or absence of chromosome 7 gain. **G** Data based on primary glioblastoma [37–39] Affymetrix U133 Plus 2 arrays or (**H**) TCGA [34] Human Exon 1.0 ST array show *HOTAIRM1* as the only lncRNA with significantly increased expression in glioblastomas with chromosome 7 gain in addition to the coding genes *EGFR, MEOX2* and *SEMA3A*. Log rank analysis for Kaplan–Meier survival plots; ***p < 0.001, **p < 0.01, *p < 0.05.

We took advantage of publically available glioma gene expression datasets to identify lncRNAs associated with overall survival of glioblastoma patients. First, we compared lncRNA expression profiles of primary glioblastoma samples from patients with long-term (overall survival > 36 months) versus short-term (overall survival < 12 months) using the German Glioma Network (GGN) cohort [33]. We found three lncRNAs that were significantly upregulated in the population of short-term survivors. Out of these lncRNAs, *HOTAIRM1* was a top candidate based on fold-change expression difference and statistical significance (Fig. 1A, Supplementary Table 1). We validated this observation across the cohort that higher *HOTAIRM1* expression also correlated with shorter overall survival (Supplementary Fig. 1A) and analyzed additional publically available data sets from two independent, non-overlapping patient cohorts published by The Cancer Genome Atlas (TCGA) consortium [34] (https://www.cancer.gov/tcga) and Gravendeel et al. (2009) [35]. Thereby, we confirmed that patients whose tumors show high *HOTAIRM1* expression levels, as defined by the upper quartile, demonstrated shorter overall survival (Fig. 1B, C). We confirmed *IDH1* mutation as a strong prognostic marker of longer survival in the GGN dataset [33] (Supplementary Fig. 1B) and found that the prognostic value of the *HOTAIRM1* expression was independent from *IDH1* mutation (Fig. 1D, E) and the *MGMT* promoter methylation status in this patient cohort (Fig. 1F, Supplementary Fig. 1). Glioblastoma patients whose tumors carried an *IDH1* mutation, *MGMT* promoter methylation and low *HOTAIRM1* expression showed the longest overall survival (Fig. 1F). Consistent with these prognostic associations, *HOTAIRM1* was recently shown by other investigators to be aberrantly expressed in glioblastoma [21] and associated with shorter survival of glioma patients [21, 24, 26].

Since the majority of glioblastomas display copy number gains of chromosome 7, often due to trisomy 7 [36], we evaluated the expression level of *HOTAIRM1* in relation to chromosome 7 copy number status in glioblastomas [37–39]. In addition to the epidermal growth factor receptor (*EGFR)* gene, i.e., the proto-oncogene most commonly amplified and overexpressed in IDH-wildtype glioblastoma [9], *HOTAIRM1* and the protein-coding gene *MEOX2* showed consistently increased expression in tumors with chromosome 7 gain when compared to tumors without this copy number increase (Fig. 1G, H). *EGFR* [40, 41] and *MEOX2* [42] overexpression have been implicated before as drivers of glioblastoma growth. *HOTAIRM1* was the only lncRNA on chromosome 7 that was significantly upregulated in gliomas with chromosome 7 gain.

### *HOTAIRM1* knock-down decreases glioblastoma cell viability, invasion, and clonogenicity

**Fig. 2:**
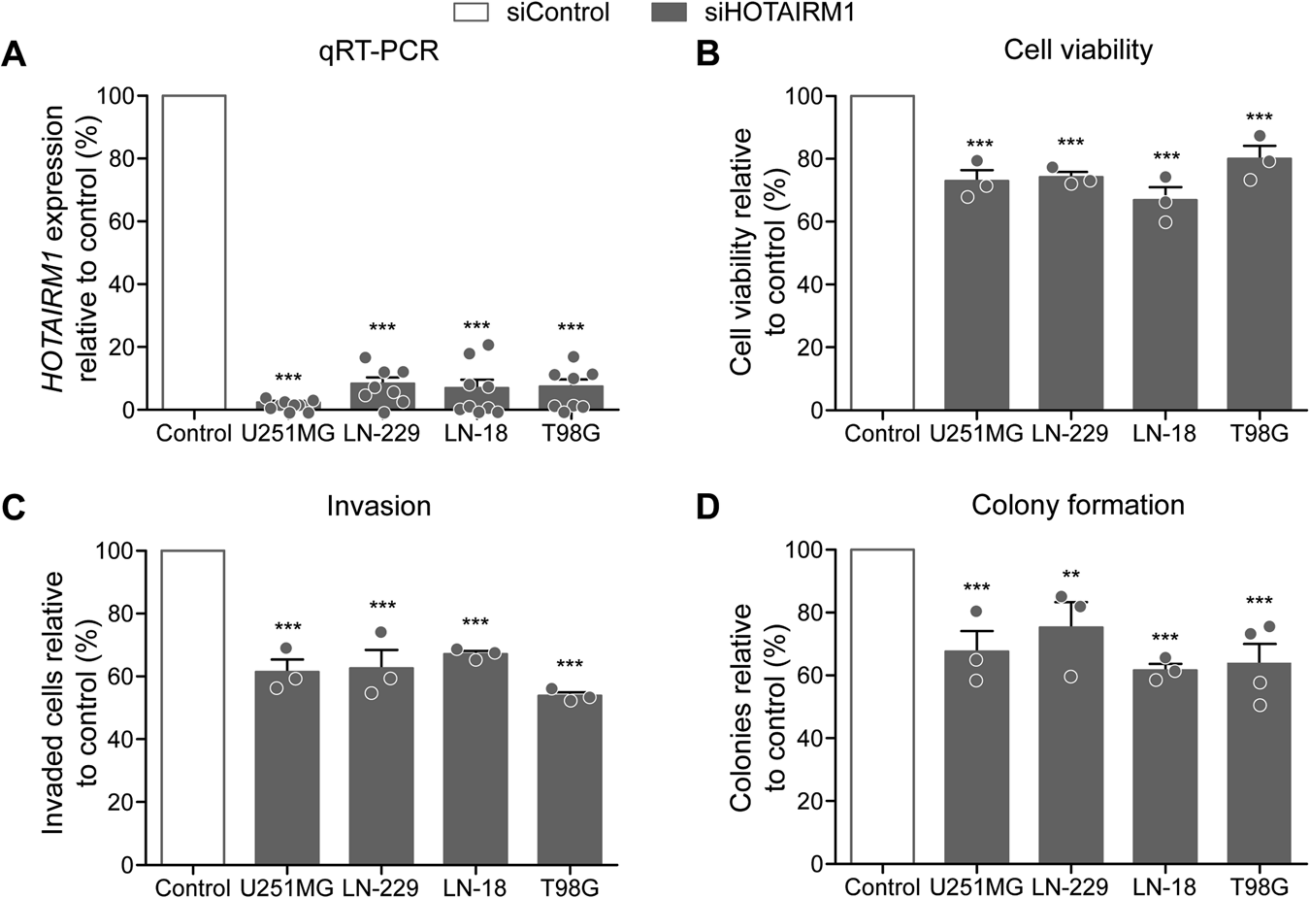
*HOTAIRM1* knock-down decreases oncogenic features in glioblastoma cell lines. siRNA-mediated knock-down was achieved using siPOOLS (siTOOLs Biotech, Planegg, Germany). **A** qRT-PCR was performed using TaqMan probes against *HOTAIRM1* or *phosphoglycerate kinase 1* (*PGK1*) as a housekeeping control gene. Following *HOTAIRM1* knock-down, the four investigated glioma cell lines showed reduced cell viability as determined with the CellTiter-Glo assay (**B**), reduced invasiveness measured in Boyden chamber assays (**C**), and, finally, decreased clonogenicity as determined by colony formation assays after seeding cells at a density of 500 (U251MG and LN-18) to 1000 (LN-229 and T98G) cells per 10 cm dish (**D**). White bars indicate the results of the respective control-transfected cells set to 100%. Filled bars are results obtained with *HOTAIRM1* knock-down cells. siControl: cells transfected with non-target siPOOLS; siHOTAIRM1: cells transfected with siPOOLS against *HOTAIRM1*. Two-way ANOVA was used for statistical analyses; mean ± SEM, ***p < 0.001, **p < 0.01. *n* = 4 independent experiments for the colony formation assays for T98G cell line, *n* = 3 independent experiments per cell line and assay.

To determine effects of genetic knock-down of *HOTAIRM1* in glioma cells, we first performed a transient siRNA-mediated knock-down of *HOTAIRM1* in the four established glioblastoma cell lines U251MG, LN-229, LN-18, and T98G (Fig. 2A–D), which showed an intermediate expression level (Supplementary Fig. 2). Efficiency of *HOTAIRM1* knock-down was greater than 80% in each of the four cell lines (Fig. 2A). *HOTAIRM1* knock-down significantly reduced cell viability of these glioma lines by 20–30% (Fig. 2B). In addition, *HOTAIRM1* knock-down resulted in significant reduction of glioma cell invasiveness by 40–50% (Fig. 2C, Supplementary Fig. 3) and colony formation capacity by 25–40% (Fig. 2D).

To further investigate the phenotypic changes caused by *HOTAIRM1* knock-down, we generated stable knock-down glioma lines using a lentiviral shRNA approach. Twenty-four hours post transduction, glioma cell lines were selected using either clonal selection with puromycin (LN-229) or selection with blasticidin for pooled populations (LN-18, SF126 and U87MG). Efficient stable knock-down was achieved for *HOTAIRM1* in all cell lines (Supplementary Fig. 4A) and the phenotypic characterization was performed as described above for transient models (Supplementary Fig. 4B–D). The stable *HOTAIRM1* knock-down cell lines corroborated the phenotype observed in transient models, i.e., cell viability was decreased by 20–40% (Supplementary Fig. 4B), while cell invasion and colony formation were reduced by 20–70% (Supplementary Fig. 4C) and 15–70% (Supplementary Fig. 4D). These findings are in line with recently published data from other groups [21, 24–26].

### Proteogenomic analyses reveal evidence for mitochondrial dysfunction upon *HOTAIRM1* knock-down

**Fig. 3:**
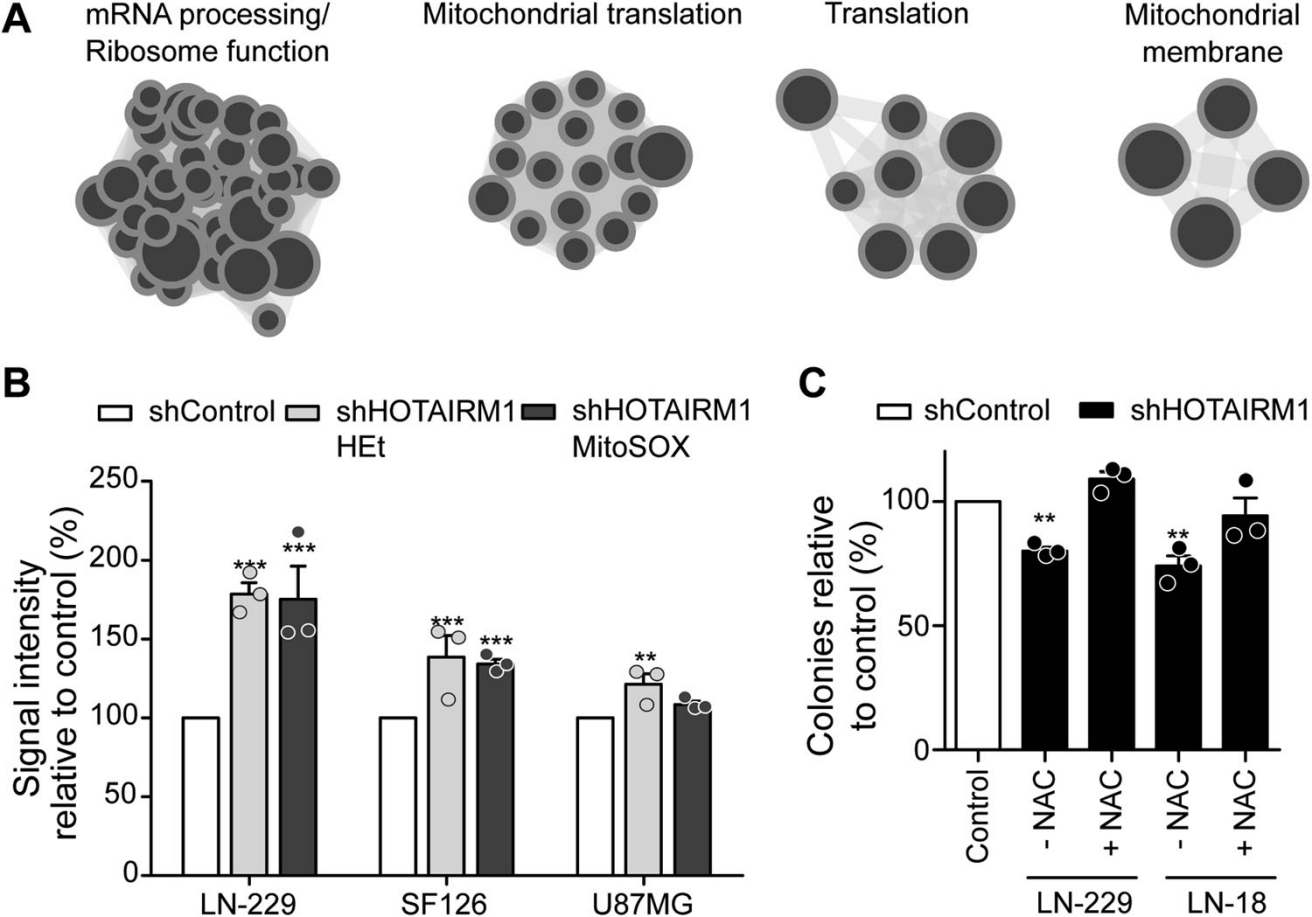
Knock-down of *HOTAIRM1* results in mitochondrial dysfunction and increased reactive oxygen species (ROS). **A** Merged GSEA of RNA sequencing and proteomics data showing overlapping geneset clusters related to mRNA processing/ribosome function, mitochondrial translation, translation and mitochondrial membrane (see supplementary tables 2–5 for the list of the genesets). **B** Quantification of HEt (general superoxide indicator) and MitoSox (mitochondrial superoxide indicator) staining performed on stable LN-229, SF126, and U87MG *HOTAIRM1* knock-down cells and respective controls (*n* = 3). Shown are ROS levels normalized to control cells. **C** Results of colony formation assays 21 days post antioxidant NAC treatment in stable LN-229 and LN-18 *HOTAIRM1* knock-down and control cells (*n* = 3). Two-way ANOVA was used for statistical analyses; mean ± SEM, ***p < 0.001, **p < 0.01, *p < 0.05.

To characterize molecular mechanisms underlying the observed effects of *HOTAIRM1* knock-down on glioma cells in vitro, we performed RNA sequencing and MS-based proteome analyses on three siRNA-mediated *HOTAIRM1* knock-down models (U251, LN-229, and T98G) and their respective control-transfected cell lines (Supplementary Tables 2-3). A proteogenomic approach was taken for integrative bioinformatic evaluation of the RNA and protein data sets. First, preranked GeneSet Enrichment Analysis (GSEA) was performed using the t-statistic from the T-test for both the RNA sequencing data (see Supplementary Table 4 and 5 for the lists of positively or negatively enriched genesets identified by RNA sequencing, respectively) and the mass spectrometry data (see Supplementary Table 6 and 7 for the lists of positively or negatively enriched genesets identified by proteome analyses, respectively). The GSEA output was then visualized in cytoscape. The overlapping nodes that were similarly enriched in both datasets contain genesets involved in mRNA processing and translation, as well as mitochondrial translation and mitochondrial membrane function, suggesting that *HOTAIRM1* knock-down in glioma cells interferes with mitochondrial and translational functions (Fig. 3A, Supplementary Table 8).

To verify potential mitochondrial dysfunction upon *HOTAIRM1* knock-down, we performed immunofluorescent dihydroethidium (HEt) and MitoSOX stainings to measure cytoplasmic and mitochondrial superoxide levels, which is a type of reactive oxygen species (ROS), as an indicator of mitochondrial dysfunction [43]. *HOTAIRM1* knock-down cells showed increased ROS levels compared to control cells (Fig. 3B), a finding in line with the proteogenomic results suggesting deficient mitochondrial function in *HOTAIRM1* knock-down glioma cells. Further support for increased ROS levels as relevant driver of cellular effects caused by *HOTAIRM1* knock-down was obtained by treatment of LN-229 and LN-18 glioma cells with N-acetyl cysteine (NAC), a ROS scavenger, which showed that NAC treatment rescued the decrease in colony formation caused by *HOTAIRM1* knock-down (Fig. 3C, Supplementary Fig. 5).

### *HOTAIRM1* knock-down sensitizes glioblastoma cells to radiation in vitro and in vivo

**Fig. 4:**
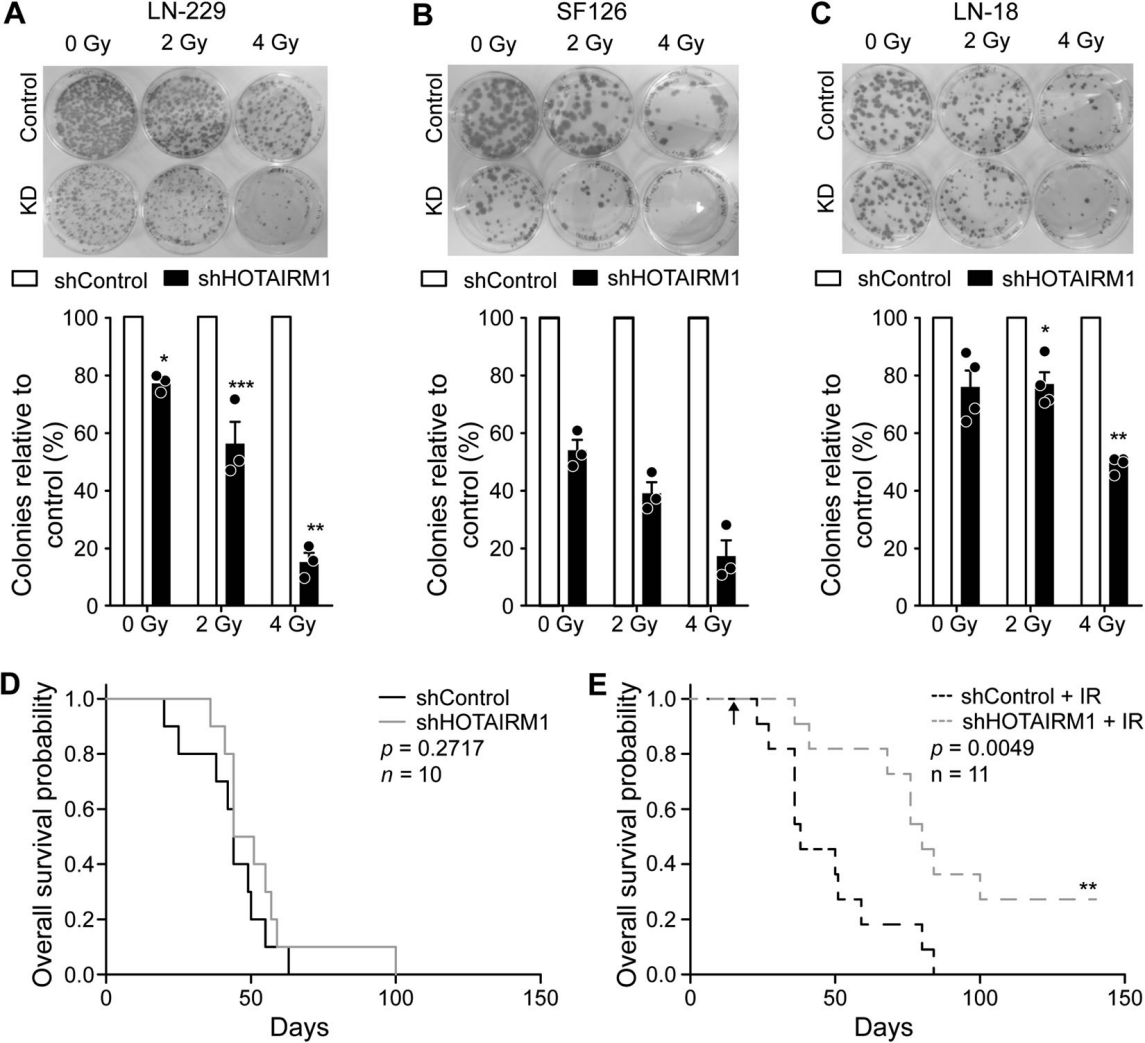
*HOTAIRM1* knock-down sensitizes glioblastoma cells to radiation in vitro and in vivo. **A**, **B**, **C** Representative images and quantification of colony formation assays 21 days post irradiation at indicated doses for stable (**A**) LN-229, (**B**) SF126, and (**C**) LN-18 *HOTAIRM1* knock-down (KD) and control cells. Counts were normalized to the corresponding counts in isogenic controls at respective radiation dose (**D**, **E**) Kaplan–Meier survival plots of mice harboring either *HOTAIRM1* stable knock-down or control transfected LN-229 orthotopic xenografts not treated with radiation (**D**) and treated with 12 Gy radiation at day 15 (arrow) (**E**). Gray and black lines represent *HOTAIRM1* knock-down and controls, respectively. shControl: non-target shRNA; shHOTAIRM1: shRNA against *HOTAIRM1*. Two-way ANOVA was used for colony formation statistical analyses and log rank analysis for Kaplan-Meier survival plots; ***p < 0.001, **p < 0.01, *p < 0.05.

Since radiation sensitivity has been associated with intracellular ROS levels [44] and since radiotherapy is an essential part of glioblastoma treatment, we investigated whether altered levels of *HOTAIRM1* affect radiosensitivity of glioblastoma cells. *HOTAIRM1* knock-down and control LN-229, SF126, and LN-18 cells were irradiated with either 2 or 4 Gy and colony formation capacity was evaluated in relation to non-irradiated cells. After 21 days, surviving colonies revealed a radiation dose-dependent significant decrease in colony formation compared to non-irradiated cells (Fig. 4A–C). *HOTAIRM1* knock-down caused reduced colony formation capacity of glioma cells already in non-irradiated cells (Fig. 2D; Supplementary Fig. 4D). However, we observed an additional, dose-dependent decrease in colony formation of *HOTAIRM1* knock-down glioma cells after irradiation compared to irradiated control-transfected glioma cells (Fig. 4A–C) which was not caused by an alteration in *HOTAIRM1* expression levels (Supplementary Fig. 6).

To validate the radiosensitizing effect of *HOTAIRM1* knock-down in vivo, we investigated an orthotopic xenograft glioma model using LN-229 control and *HOTAIRM1* knock-down cells. Mice inoculated intracerebrally with LN-229 control or *HOTAIRM1* knock-down cells were evaluated for orthotopic tumor growth and survival either with or without single irradiation with 12 Gy at day 15 post tumor cell transplantation. *HOTAIRM1* knock-down did not alter overall survival when mice were not irradiated (Fig. 4D), with control and *HOTAIRM1* knock-down LN-229-bearing mice exhibiting median overall survivals of 43 and 44.5 days, respectively. This finding contrasts with a recent study showing reduced in vivo tumor growth of U87MG glioma cells following *HOTAIRM1* knock-down [21], which might be related to the different models and experimental conditions. Importantly, however, we found that *HOTAIRM1* knock-down LN-229-bearing mice survived significantly longer following radiotherapy when compared to mice transplanted with control-transfected LN-229, as indicated by median overall survivals of 80 versus 38 days, respectively (Fig. 4E).

### *Transglutaminase 2* (*TGM2*) is down-regulated upon *HOTAIRM1* knock-down in glioblastoma cells

**Fig. 5:**
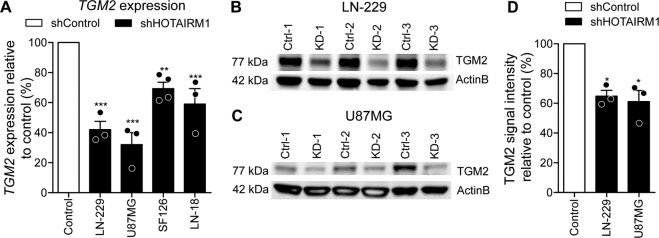
*TGM2* expression is correlated with *HOTAIRM1* expression. **A** qRT-PCR for *TGM2* expression was performed in control and stable *HOTAIRM1* knock-down LN-229, U87MG, SF126, and LN-18 cell lines. White bar indicates the results of the respective control cells set to 100%. Note that *HOTAIRM1* knock-down significantly reduces *TGM2* mRNA levels. **B–D** Western blotting analysis of TGM2 protein expression in control versus stable *HOTAIRM1* knock-down LN-229 (**B**) and U87MG (**C**) cell lines. Shown are three independent experiments. Beta-actin (ActinB) was used as a loading control. **D** Quantification of TGM2 protein expression by western blotting analysis in control versus stable *HOTAIRM1* knock-down LN-229 and U87MG cell lines. White bar indicates the results of the respective control cells set to 100%. Two-way ANOVA was used for statistical calculation for qRT-PCR and Student’s t test was used for statistical analysis. Control: non-target shRNA; KD: shRNA-mediated *HOTAIRM1* knock-down; mean ± SEM, ***p < 0.001, **p < 0.01, *p < 0.05. *n* = 3.

Mass spectrometry-based proteome analyses using the stable LN-229 *HOTAIRM1* knock-down model detected 16 proteins that were upregulated and 12 proteins that were down-regulated upon *HOTAIRM1* knock-down (Supplementary Tables 9–11). Transglutaminase 2 (*TGM2*) was detected as one of the 12 proteins that were down-regulated in *HOTAIRM1* knock-down LN-229 cells and also was significantly correlated with *HOTAIRM1* in the TCGA glioblastoma patient tissues (Supplementary Fig. 7A). Interestingly, *TGM2* has been shown to play a role in mitochondrial function [45] and cancer therapy resistance [46, 47]. Reduced *TGM2* mRNA and protein levels in *HOTAIRM1* stable knock-down LN-229, as well as other glioblastoma cell lines (U87MG, LN-18, and SF126) relative to control-transfected cells, were confirmed by RT-qPCR (Fig. 5A) and Western blotting (Fig. 5B–D). We corroborated reduced TGM2 expression upon siRNA-based knockdown of *HOTAIRM1* in LN-229, T98G, and U87MG, while U251MG showed no detectable expression on protein level of the proposed candidate (Supplementary Fig. 7B).

### siRNA-mediated *TGM2* knock-down mimics the phenotype of *HOTAIRM1* knock-down

**Fig. 6:**
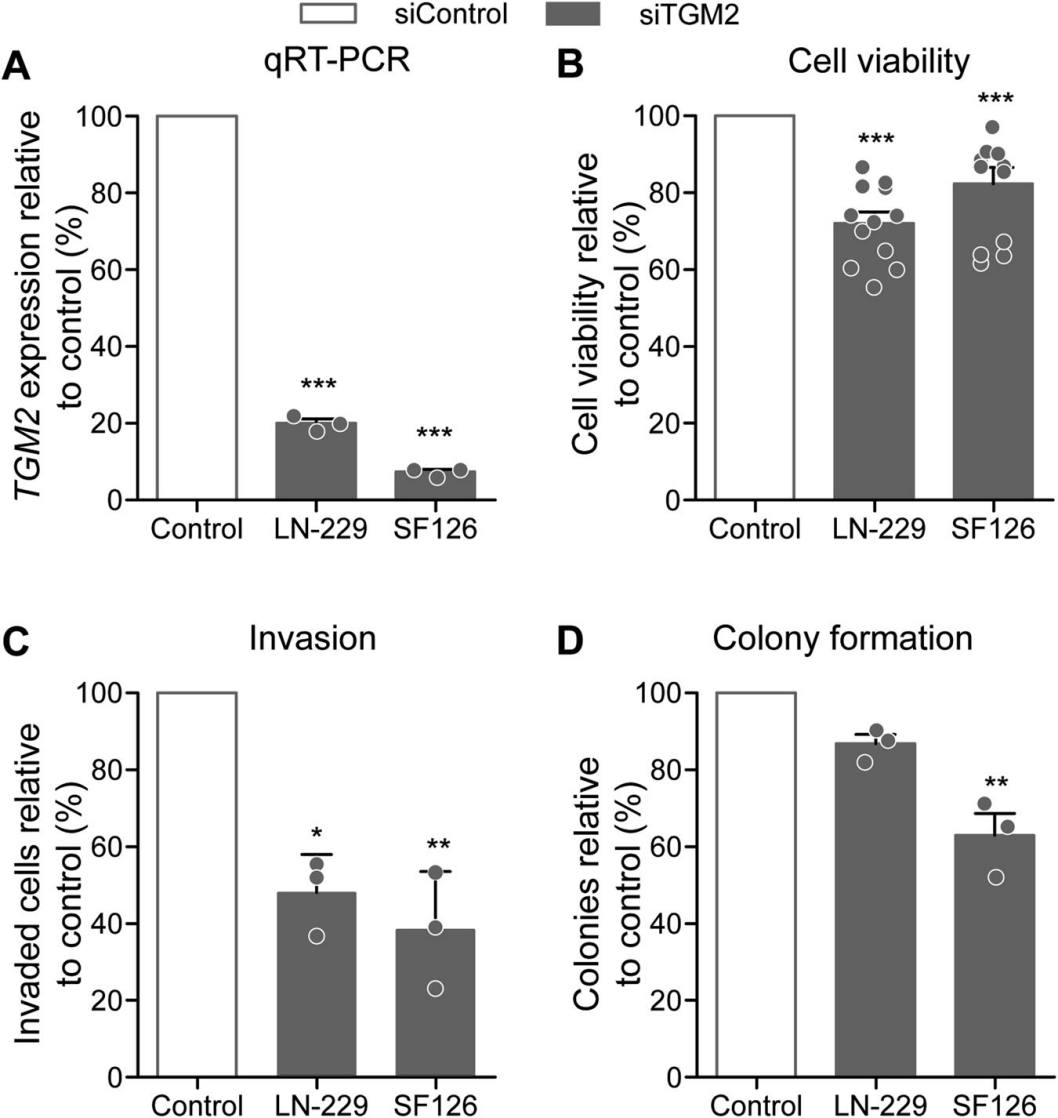
*TGM2* knock-down decreases oncogenic features in glioblastoma cell lines. **A** qRT-PCR analysis confirms siRNA-mediated knock-down of TGM2 in LN-229 and SF126 glioma cells. Shown are *TGM2* mRNA levels normalized to *PGK1* mRNA levels relative to control transfected cells set to 100%. Results of determination of cell viability using CellTiter-Glo assays (**B**), cell invasion using Boyden chamber assays (**C**), and colony formation propensity using colony formation assays (**D**) in control versus stable *HOTAIRM1* knock-down LN-229 and SF126 cell lines. White bars represent control cells normalized to 100%, filled bars represent *TGM2* knock-down cells. Two-way ANOVA was used for statistical analyses; mean ± SEM, ***p < 0.001, **p < 0.01. *n* = 3.

To investigate functional roles of reduced *TGM2* expression in glioblastoma cells, we performed siRNA-mediated knock-down of *TGM2* in LN-229 and SF126 glioma cells (Fig. 6A). Similar to *HOTAIRM1* knock-down, *TGM2* knock-down resulted in significantly reduced cell viability (Fig. 6B), cell invastion (Fig. 6C), and colony formation (Fig. 6D). Although *TGM2* and *HOTAIRM1* expression is positively correlated, we did not observe the same survival benefit for *HOTAIRM1* (Supplementary Fig. 8A–D), as the strongest prognostic indicator observed was *MGMT* promoter methylation status (Supplementary Fig. 8E). The in vitro data are in line with studies reporting on tumor-promoting functions of TGM2 in other cancer models [48, 49] and suggest regulation of *TGM2* by *HOTAIRM1* as a putative mechanism driving glioma aggressiveness However, *TGM2* expression is not regulated by promoter methylation in patient samples, as analysis of glioblastoma methylation data showed that *HOTAIRM1* promoter is unmethylated in all samples, independently of high or low *HOTAIRM1* expression (Supplementary Fig. 7C).

### Potential regulation of *TGM2* by *HOTAIRM1* via sponging of *hsa-miR-17-5p*

*HOTAIRM1* has recently been proposed to function as a sponge for several miRNAs [24–26, 50–54] including *miR-17-5p* [50, 53], *miR-129-5p*, and *miR-495-3p* [24]. Therefore, we performed in silico analyses to determine putative miRNA binding sites that are shared between *HOTAIRM1* and transcripts of the 12 proteins with significantly reduced expression in *HOTAIRM1* knock-down LN-229 cells. The number of candidate miRNAs with putative binding sites in *HOTAIRM1* and any of the 12 candidate gene transcripts was further narrowed down by filtering for those miRNAs whose expression was inversely correlated to the expression of the 12 genes in the TCGA mRNA and miRNA glioblastoma data set (accession: phs000178.v10.p8.c1). Together, these in silico analyses revealed 15 miRNAs targeting *HOTAIRM1* and at least one of the genes of interest (Supplementary Table 12). Interestingly, 14 of the 15 miRNAs had predicted binding sites in both *TGM2* and *HOTAIRM1* (Supplementary Table 12) which includes members of the miR17-92 cluster, specifically *hsa-miR-17-5p* that has been reported to interact with *HOTAIRM1* in gastric and colorectal tumor entities [50, 53]. We found that low levels of *hsa-miR-17-5p* expression are associated with shorter survival of glioblastoma patients in the TCGA cohort (Supplementary Fig. 9A) and that expression of *hsa-miR-17-5p* is inversely correlated with *TGM2* expression in this cohort set (Supplementary Fig. 9B), however, not with *HOTAIRM1* expression (Supplementary Fig. 9C). To support these data, we performed a miR-17-5p overexpression experiment whereby miR-17-5p mimics were transfected in LN-229 cells. We were able to verify that upon increased miR-17-5p expression levels, *TGM2* expression was reduced (Supplementary Fig. 10A). To show that miR-17-5p regulates *TGM2* 3′UTR, we then performed a luciferase assay using either the predicted miR-17-5p binding site of the *TGM2* 3′UTR (17-5 wt) or a corresponding miR17-5p mutant binding site (17-5 mt) fused to Luciferase. This showed that addition of a miR-17-5p mimic led to a reduction of 17-5 wt, but not of 17-5 mt, driven Luciferase (Supplementary Fig. 10B), altogether indicating that *TGM2* transcript is a target of miR-17-5p. Since *HOTAIRM1* was shown to bind to miR-17-5p [50, 53], our data suggest that *HOTAIRM1* regulates *TGM2* expression possibly by controlling the availability of miR-17-5p.

## Discussion

We have collected compelling evidence from several independent data sets that high *HOTAIRM1* expression is linked to clinical aggressiveness and shorter survival of glioblastoma patients and that gene copy number gain is a likely cause of increased *HOTAIRM1* expression levels in glioblastoma. After targeting *HOTAIRM1* expression in glioblastoma cell lines, the oncogenic potential of these cells was diminished and RNA sequencing and mass spectrometry data suggested impaired mitochondrial function. As it has been shown that cells are more sensitive after temozolomide treatment [24], we decided to focus on the novel finding of mitochondiral disfunction induced upon *HOTAIRM1* deficiency. Not only did we validate this finding in vitro, but our data indicate that high expression of *HOTAIRM1* supports radioresistance of glioblastoma cells, which in turn may contribute to shorter patient survival as seen in our in vivo model. Since radiation sensitivity has been associated with intracellular ROS levels [44], the modulation of ROS levels by *HOTAIRM1* shown in our study suggests *HOTAIRM1*-mediated reduction of intracellular ROS as a potential mechanism contributing to glioma radioresistance.

To determine additional factors implicated in the *HOTAIRM1* mode of action, stable control, and *HOTAIRM1* knockdown cell lines have been profiled by proteomic analysis, pointing to *TGM2* as a potential mediator of radioresistance. TGM2 is localized in mitochondria as well as in the cytoplasm, endoplasmic reticulum, and plasma membranes [55]. The function of TGM2 in mitochondria is an emerging field [45] and data indicate that TGM2 plays a role in metabolism and mitochondrial respiration [56]. Our data shows that *HOTAIRM1* promotes *TGM2* expression in glioblastoma cells, which is related to miRNA-mediated mechanisms implicating *hsa-miR-17-5p*. Our analysis predicted binding sites for *hsa-miR-17-5p* in both *HOTAIRM1* and *TGM2* mRNAs. This microRNA, which has been reported to be upregulated by irradiation in glioblastoma [57], has been linked to glioma recurrence [58]. In addition, *TGM2* confers radioresistance in different types of cancer cells [47, 59]. Collectively, these data suggest that *HOTAIRM1* may promote glioma growth and therapy resistance by sponging *hsa-miR-17-5p*, and thereby increasing *TGM2* transcript and protein levels. Thus, in addition to the epigenetic modulation of *HOXA1* and the sponging *hsa-miR-129-5p* and *hsa-miR-495-3p*, sponging of *hsa-miR-17-5p* (and potentially other *TGM2*-binding miRNAs) by *HOTAIRM1* may cause increased *TGM2* mRNA and protein expression in glioblastoma. However, *HOTAIRM1* may also affect other downstream targets as suggested by the absense of TGM2 expresion in U251MG, as the siRNA-based *HOTAIRM1* depletion had similar antitumoral effects in this model. Furthermore, we showed *HOTAIRM1*-mediated TGM2 depletion, which might occur due to hsa-miR-17-5p modulation at the translational level as *TGM2* mRNA levels were not affected in T98G, while a reduction in protein levels was detected.

In summary, we confirm and extend recent data implicating *HOTAIRM1* as an oncogenic lncRNA driving tumor growth, therapy resistance, and poor prognosis of glioblastoma. Moreover, our data suggest a novel role for *HOTAIRM1* in regulating mitochondrial function and ROS levels in glioblastoma cells by modulating expression of *TGM2*, potentially by functioning as a miRNA sponge.

## Supplementary information

Supplementary Figure Legends

Supplementary Figure 1

Supplementary Figure 2

Supplementary Figure 3

Supplementary Figure 4

Supplementary Figure 5

Supplementary Figure 6

Supplementary Figure 7

Supplementary Figure 8

Supplementary Figure 9

Supplementary Figure 10

Supplementary Table 1

Supplementary Table 2

Supplementary Table 3

Supplementary Tables 4-8, 10-12

Supplementary Table 9

author contribution form

CDDis checklist
