# Supplementary material for: The long non-coding RNA HOTAIRM1 promotes tumor aggressiveness and radiotherapy resistance in glioblastoma
Source: Cell Death Dis. 2021 Sep 28;12(10):885. doi: 10.1038/s41419-021-04146-0 (PMC8478910; doi:10.1038/s41419-021-04146-0)
Supplement: Supplementary file 1 — Supplementary Figure Legends [file 41419_2021_4146_MOESM1_ESM.docx]

**Supplementary Figure Legends**

**Supplementary Figure 1**

**High *HOTAIRM1* expression is associated with shorter survival of glioblastoma patients.** (**A**) Kaplan-Meier survival curves of 70 glioblastoma patients from the GGN cohort (1) stratified according to *HOTAIRM1* expression. Black and grey lines indicate patients whose tumors had high or low *HOTAIRM1* expression, respectively. Cut-off was determined by final quartile and log rank statistics were calculated. (**B**) Kaplan-Meier survival plot of the same patient cohort stratified according to *IDH* mutation status; black and grey lines indicate patients with IDH-wildtype or IDH-mutant tumors, respectively. (**C**) Kaplan-Meier survival plot of the patient cohort stratified according to *HOTAIRM1* expression (black and grey lines, high and low *HOTAIRM1*, repsectively) and *MGMT* protomer metylation (full and dashed lines, methylated vs unmethylated MGMT, respectively). (**D**) Kaplan-Meier survival plot of (**C**) after excluding the patients with IDH-mutant tumors.

**Supplementary Figure 2**

***HOTAIRM1* expression levels in various glioma cell lines are similar to those seen in glioblastoma tissue samples.** *HOTAIRM1* expression is shown using the Gravendeel *et al*. dataset (2) for non-neoplastic brain samples (n=8) and glioblastoma tissue samples (n=159), as well as, the Cancer Cell Line Encyclopedia (CCLE, GSE36133) glioma cell lines (n=43) profiled using the Affymetrix U133 Plus 2 array. Arrows and bold names indicate the glioblastoma cell lines used in this study.

**Supplementary Figure 3**

***HOTAIRM1* knock-down decreases invasiveness in established glioblastoma cell lines.** Invasiveness was measured using Boyden chamber assays. Representative images (10X) from one of three replicates of the Boyden chamber assays performed for each cell line are shown. siControl: cells transfected with non-target siRNA pool; siHOTAIRM1: cells transfected with siRNA pool against *HOTAIRM1.*

**Supplementary Figure 4**

**Stable *HOTAIRM1* knock-down decreases oncogenic features in glioblastoma cell lines.** Stable knock-down was achieved using shRNA against *HOTAIRM1*. (**A**) qRT-PCR was performed using TaqMan probes against *HOTAIRM1* or *phosphoglycerate kinase 1* (*PGK1*) (housekeeping gene). (**B**) Cell viability was measured using CellTiter-Glo assays. (**C**) Invasiveness was measured using Boyden chamber assays. (**D**) Colony formation assays were done by seeding cells at a density of 500 (U251MG and LN-18) to 1000 (LN-229, U87MG and SF126) cells into 10 cm dishes. Histogram bars are as follows: outline bars represent results obtained for control-transfected cells set to 100% and filled bars are results obtained for the respective glioma cells after *HOTAIRM1* knock-down relative to the control cells. shControl: cells transduced with non-target shRNA; shHOTAIRM1: cells transduced with shRNA against *HOTAIRM1*. Two-way ANOVA was used for statistical analyses; mean +/-SEM, *** *p* < 0.001, ** *p* < 0.01, * *p* < 0.05. n = 3.

**Supplementary Figure 5**

**Increased reactive oxygen species after antioxidant treatment of *HOTAIRM1* knockdown compared to control.** (**A-B**) Results of colony formation assays 21 days post antioxidant NAC treatment in stable LN-229 (**A**) and LN-18 (**B**) *HOTAIRM1* knock-down and control cells (n = 3) (non-normalized data for Figure 3). Two-way ANOVA was used for statistical analyses; mean +/-SEM, *** *p* < 0.001, ** *p* < 0.01, * *p* < 0.05.

**Supplementary Figure 6**

**Radiation of established glioma cell lines does not alter *HOTAIRM1* expression levels.** Expression of *HOTAIRM1* was measured in gliomablastoma cells lines by qRT-PCR (SF126, LN229 or LN18 (**A)**) or by Affymetrix U133P2 arrays in two separate datasets (HK-374 (**B)** and LNZ308 **(C)**) 48 hours post 2 or 4Gy radiation. Two-way ANOVA was used for statistical analyses; mean +/-SEM (**A**) or Mann-Whitney test, mean +/-SD (**B-C**).

**Supplementary Figure 7**

**HOTAIRM1 and TGM2 expression levels are correlated.** (**A**) Dot plot showing Pearson correlation of *TGM2* and *HOTAIRM1* expression in glioblastomas of the TCGA cohort (3)*.* (**B**) Western blotting analysis of TGM2 protein expression in control versus transient *HOTAIRM1* knock-down of LN-229, U251MG, T98G and U87MG cell lines. Beta-actin (ActinB) was used as a loading control. (**C**) Heatmap showing the methylation status of the *TGM2* gene and probes up to 5kb upstream, and the relationship to *HOTAIRM1* expression, where available. Two-way ANOVA was used for statistical analyses; mean +/-SEM, * *p* < 0.05

**Supplementary Figure 8**

**Prognostic role of *TGM2* expression in glioblastoma patient datasets.** (**A** Overall survival plots of glioblastoma patients from TCGA (<https://www.cancer.gov/tcga>) and **(B)** Gravendeel *et al*. stratified according to high or low *TGM2* expression levels. Cut-off for high *TGM2* was determined by upper quartile and log rank statistics were calculated. (**C,D**) Overall survival of glioblastoma patients in the Gravendeel *et al*. cohort according to *TGM2* expression in *IDH1-*wildtype (**C**) and *IDH1*-mutant glioblastomas (**D**). (**E**) Overall survival of glioblastoma patients in the GGN cohort (1) stratified according to *TGM2* expression, *MGMT* promoter methylation status, and *IDH1* mutation status (wt: wild-type; mut: mutant; meth: methylated; unmeth: unmethylated). The table right of the Kaplan-Meier graph lists p-values for the individual subgroups. Log rank analysis for Kaplan-Meier survival plots**.**

**Supplementary Figure 9**

**Decreased *hsa-miR-17-5p* expression associates with shorter survival of glioblastoma patients.** (**A**) Kaplan-Meier plot illustrating overall survival of IDH-wildtype glioblastoma patients from the TCGA cohort (3) stratified according to *hsa-miR-17-5p* expression. Black and grey lines indicate patients whose tumors showed low and high *hsa-miR-17-5p* expression, respectively. Cut-off was determined by final quartile and log rank statistics were calculated. (**B**-**C**) Dot plots showing Pearson correlations of *hsa-miR-17-5p* and *TGM2* expression (**B**), and *hsa-miR-17-5p* and *HOTAIRM1* expression (**C**) in glioblastomas of the TCGA cohort^3^.

**Supplementary Figure 10**

**TGM2 expression is dependent on *hsa-miR-17-5p*.** (**A**) qRT-PCR of *TGM2* expression levels of LN-229 cells transfected with either a *hsa-miR-17-5p* mimic or a non-target negative control. (**B**) Dual-Glo luciferase assay where LN229 cells were transfected with a *hsa-miR-17-5p* mimic or a non-target negative control, as well as, a construct containing either the *TGM2* *hsa-miR-17-5p* binding site or a *TGM2* *hsa-miR-17-5p* mutated binding. Data is normlized to firefly luciferase for transfection efficiency. Student T-test were used for statistical analyses; mean +/-SEM, *** *p* < 0.001, ** *p* < 0.01, * *p* < 0.05.

**References**

1. Reifenberger G, Weber RG, Riehmer V, Kaulich K, Willscher E, Wirth H, et al. Molecular characterization of long-term survivors of glioblastoma using genome- and transcriptome-wide profiling. Int J Cancer. 2014;135(8):1822-31.

2. Gravendeel LA, Kouwenhoven MC, Gevaert O, de Rooi JJ, Stubbs AP, Duijm JE, et al. Intrinsic gene expression profiles of gliomas are a better predictor of survival than histology. Cancer Res. 2009;69(23):9065-72.

3. Cancer Genome Atlas Research N. Comprehensive genomic characterization defines human glioblastoma genes and core pathways. Nature. 2008;455(7216):1061-8.
