# Supplementary material for: The long non-coding RNA HOTAIRM1 promotes tumor aggressiveness and radiotherapy resistance in glioblastoma
Source: Cell Death Dis. 2021 Sep 28;12(10):885. doi: 10.1038/s41419-021-04146-0 (PMC8478910; doi:10.1038/s41419-021-04146-0)
Supplement: Supplementary file 12 — Supplementary Table 1 [file 41419_2021_4146_MOESM12_ESM.pdf]

Supplementary Table 1. List of lncRNAs used for the Volcano plot

Probe ID	Gene Symbol	Fold Change	*P* Value
229782_at	RMST	3.636649035	0.000168308
238021_s_at	CRNDE	2.913392397	0.008799548
1557051_s_at	HOTAIRM1	2.460288269	0.014781021
238022_at	CRNDE	2.371453092	0.008632333
228642_at	HOTAIRM1	2.334469921	0.025503153
239153_at	HOTAIR	1.948787255	0.038329668
235292_at	FLJ32255	1.842214613	0.008873885
1557050_at	HOTAIRM1	1.822677264	0.025906264
237189_at	HOXB-AS1	1.819193248	0.005389652
234989_at	NEAT1	1.742027557	0.031432957
213340_s_at	TP73-AS1	1.729012422	0.037771583
214077_x_at	MEIS3P1	1.695902792	0.003650158
232090_at	DNM3OS	1.683495182	0.060970009
232752_at	LOXL1-AS1	1.665393029	0.061404607
208816_x_at	ANXA2P2	1.645311079	0.082458646
238320_at	NEAT1	1.629975982	0.084841469
225239_at	NEAT1	1.596143821	0.10593236
231925_at	RP11-38P22.2	1.547676756	0.117320372
1566968_at	SPRY4-IT1	1.540394515	0.150010207
232242_at	PTPRG-AS1	1.524804972	0.013714417
233198_at	GOLGA2P5	1.507360746	0.037839011
235291_s_at	FLJ32255	1.490989736	0.031577727
1555907_at	AGAP2-AS1	1.4758727	0.203940404
217551_at	OR7E14P	1.456449363	0.056282813
1556364_at	ADAMTS9-AS2	1.453308222	0.12348221
214823_at	ZNF204P	1.435975317	0.136763193
241607_at	LOC730102	1.43492702	0.040832792
1557241_a_at	RP11-524D16__A.3	1.434857561	0.089201968
220244_at	LINC00312	1.433609139	0.015961321
228658_at	MIAT	1.429858642	0.302893798
1560431_at	RP11-87H9.3	1.429829313	0.021771783
227062_at	NEAT1	1.42824586	0.233880179
215513_at	HYMAI	1.418865863	0.071077021
1559117_at	RP11-399K21.12	1.413653674	0.023074787
227868_at	LOC154761	1.413146523	0.187498123
220609_at	LOC202181	1.408907267	0.103668107
214740_at	POLR2J4	1.402378742	0.013554623
1558354_s_at	RP1-1J6.2	1.396842462	0.104518574
230595_at	PGM5-AS1	1.390845609	0.005024037
233249_at	LOC100507073	1.389395612	0.036658184
1561759_at	LOC645513	1.386193483	0.006500854
233947_s_at	TBX5-AS1	1.380897357	0.080342577
1556378_a_at	LOC440896	1.376236822	0.063984012
229015_at	LOC286367	1.375719307	0.012065296
230882_at	DLX6-AS1	1.366457302	0.397707447
244571_s_at	RP11-159N11.4	1.365705498	0.003445356
244512_at	HOXB-AS1	1.362344995	0.009225247

1559965_at	ZFHX4-AS1	1.361752393	0.225539098
227168_at	MIAT	1.359944087	0.240388746
203360_s_at	GJA9-MYCBP	1.359465806	0.088566007
222180_at	RP11-769O8.3	1.358659397	0.130640988
243362_s_at	LEF1-AS1	1.355139053	0.015330859
241713_s_at	DYX1C1-CCPG1	1.345984142	0.067497589
207063_at	TTTY14	1.345826917	0.151081631
1562633_at	RMST	1.344302029	0.003667378
236692_at	RP11-686D22.8	1.343186612	0.113371232
239999_at	LINC00478	1.336226264	0.063607131
229543_at	RP1-93H18.6	1.336062024	0.057542325
224566_at	NEAT1	1.334762787	0.216526993
236115_at	HTR7P1	1.332780438	0.063056135
237250_at	RP11-320H14.1	1.331037856	0.456030685
239975_at	HLA-DPB2	1.329677959	0.010412392
230861_at	DKFZP434L187	1.328452413	0.213650729
216950_s_at	FCGR1C	1.322585963	0.190698008
232172_at	CD99P1	1.321922758	0.008435534
238127_at	GAS6-AS1	1.32176548	0.150399469
239675_at	LINC00900	1.317523054	0.057575227
237291_at	PRORSD1P	1.310741836	0.015226251
232878_at	NR2F2-AS1	1.307321049	0.188680104
216333_x_at	TNXA	1.306490718	0.139710144
1559296_at	ADAMTS9-AS2	1.306254006	0.168899132
230505_at	LOC145474	1.306048628	0.202305154
214110_s_at	LOC654342	1.302829496	0.129866584
232309_at	LOC202181	1.299600331	0.044703762
213339_at	TP73-AS1	1.294124204	0.006408052
225214_at	LOC100129034	1.292569275	0.089524605
228158_at	LOC654342	1.292278979	0.098453945
239919_at	TBX5-AS1	1.289907521	0.109292331
213380_x_at	MST1P2	1.285851049	0.064358056
201911_s_at	FARP1-IT1	1.28405992	0.030953199
60815_at	POLR2J4	1.279959323	0.091406024
204961_s_at	NCF1B	1.278926417	0.062447234
239556_at	LOC645513	1.278396659	0.224337657
206093_x_at	TNXA	1.275949205	0.157614282
235469_at	FAM133DP	1.274826028	0.146850769
1553634_a_at	WEE2-AS1	1.272557761	0.013626216
213451_x_at	TNXA	1.269942957	0.193260527
1569973_at	SEPT7P2	1.269598914	0.001604512
239847_at	RP11-33E12.2	1.269594011	0.118228542
243124_at	RRN3P2	1.268650187	0.078637278
240177_at	RP11-218C14.8	1.265518276	0.126004398
211038_s_at	CROCCP2	1.265161706	0.102953375
1569318_at	LINC00663	1.264348507	0.020264804
227437_at	DYNLL1-AS1	1.262364399	0.043015939
1564166_s_at	LOC100630923	1.260952722	0.021066994
1561341_at	RP11-595O22.1	1.260840861	0.35681424
215629_s_at	DLEU2L	1.260181512	0.080402379

238854_at	DYNLL1-AS1	1.258892348	0.039764281
1562904_s_at	FAM86B3P	1.255330629	0.038529126
1568838_at	WASIR1	1.254772783	0.082219711
216336_x_at	MT1P3	1.252864466	0.23202123
1569315_s_at	LINC00894	1.251980029	0.078489836
1559492_at	LOC100506844	1.250652753	0.44973644
1561292_at	RP11-37C7.3	1.248309576	0.004226852
1556003_a_at	RP11-356K23.1	1.24744459	0.069917551
229283_at	LOC728613	1.243460242	0.027797324
201910_at	FARP1-IT1	1.24256798	0.057336834
208609_s_at	TNXA	1.241413696	0.155209845
231436_at	RP11-489D6.2	1.24055253	0.023520968
224565_at	NEAT1	1.240262049	0.350980591
1562938_at	FAHD2CP	1.240208063	0.374525393
243363_at	LEF1-AS1	1.240048875	0.008285821
1559957_a_at	LOC642852	1.239059098	0.177285547
1561692_at	RP11-319G9.3	1.237539049	0.119817714
214084_x_at	NCF1C	1.236733286	0.073314988
230351_at	FGF14-AS2	1.234599534	0.174456283
238085_at	RP11-1191J2.2	1.23268491	0.148829567
227940_at	LOC339803	1.232206312	0.175251013
231470_at	LINC00664	1.230688226	0.057183996
1560019_at	DLGAP1-AS2	1.230047193	0.074951195
214473_x_at	PMS2P3	1.230039872	0.006407421
65585_at	FAM86DP	1.228803369	0.023996013
231366_at	FDPSP2	1.227561287	0.005746967
1561511_at	RP11-510C10.4	1.22250522	0.232049147
221973_at	LOC100506123	1.22244579	0.313748409
216870_x_at	DLEU2	1.22196585	0.06349276
243020_at	FAM13A-AS1	1.218165556	0.071147069
225907_at	LOC728743	1.217671953	0.147432188
236892_s_at	HOXB-AS3	1.2169326	0.048520601
238559_at	RP11-568N6.1	1.216009647	0.108029549
213089_at	LOC100272216	1.215519207	0.305330861
1556026_at	LINC00893	1.215190045	0.013219698
234049_at	FAM86B3P	1.215157818	0.069167545
206685_at	HCG4	1.215048235	0.080698227
213460_x_at	NSUN5P2	1.214669186	0.05079391
234340_at	RP4-614O4.12	1.214618145	0.089091946
1568854_at	LINC00240	1.21453613	0.102863756
242770_at	LOC642236	1.213681036	0.170827369
215667_x_at	PMS2P1	1.213178556	0.016746367
213670_x_at	NSUN5P1	1.212930786	0.046446166
1569974_x_at	SEPT7P2	1.211541644	0.002752464
204060_s_at	PRKY	1.211109259	0.251541098
210109_at	SND1-IT1	1.210417412	0.113300627
216525_x_at	PMS2P3	1.207125617	0.022439346
227996_at	FARP1-IT1	1.205658051	0.372950232
1559901_s_at	LINC00478	1.20409379	0.094898925
242699_at	RP11-642D21.1	1.202640602	0.460014799

213842_x_at	NSUN5P2	1.202251022	0.057566078
1558666_at	PHEX-AS1	1.20214224	0.088307701
226582_at	LOC400043	1.201217223	0.38470809
235482_at	PCBP1-AS1	1.201197448	0.189474213
228913_at	TPT1-AS1	1.200747178	0.162229612
1569794_at	RP4-742J24.2	1.200550339	0.061407319
1560156_at	RP11-705C15.3	1.198558432	0.101574065
240809_at	ZNF295-AS1	1.198415696	0.033800697
1561761_x_at	LOC645513	1.197621983	0.026277747
1562012_at	LOC100506730	1.195067375	0.203581389
1555981_at	ASB16-AS1	1.193862902	0.061947903
1558653_at	MLK7-AS1	1.193351859	0.061066645
1570208_at	LINC00907	1.192270961	0.079469687
1564139_at	A2M-AS1	1.189977655	0.303318001
1558430_at	RP11-680G24.5	1.189171993	0.271503704
214100_x_at	NSUN5P1	1.187783683	0.059173233
229059_at	FAM225B	1.185927835	0.301988567
213893_x_at	PMS2P5	1.185254615	0.02654715
229874_x_at	RP11-108M9.4	1.184902716	0.073110078
227074_at	LOC100131564	1.184426495	0.115258179
235263_at	STAG3L2	1.18393751	0.167837231
215318_at	MINOS1P1	1.183889989	0.250213377
215057_at	LINC00894	1.183287039	0.101573347
233530_at	RP11-314N13.3	1.183066633	0.053844744
224293_at	TTTY10	1.182469202	0.206780303
1554771_at	RP11-690I21.2	1.182464481	0.221726826
223724_s_at	STAG3L2	1.181823412	0.078090013
1559433_at	APCDD1L-AS1	1.181345669	0.017555476
236228_at	LOC100130744	1.179901778	0.048014274
1557262_at	RP11-66N24.3	1.179166946	0.021500707
1558750_a_at	LOC100288637	1.177784371	0.439192948
221191_at	STAG3L1	1.176704962	0.27333549
215920_s_at	PDXDC2P	1.175225946	0.086250788
1560797_s_at	TMLHE-AS1	1.175113987	0.0831058
1557558_s_at	MATN1-AS1	1.174517564	0.181710293
215966_x_at	GK3P	1.172393898	0.04940198
214712_at	SNX29P2	1.171774886	0.143125573
241658_at	RP11-1006G14.4	1.169788747	0.180295951
235242_at	RP11-373L24.1	1.169278473	0.191787585
232881_at	GNAS-AS1	1.168981781	0.180902463
1564372_s_at	CASC2	1.168526332	0.069380604
206742_at	PIR-FIGF	1.167664526	0.037624068
210707_x_at	DTX2P1-UPK3BP1-PMS2P11	1.167574378	0.019532955
236860_at	NPY6R	1.166198652	0.231161702
240219_at	LINC00327	1.166127067	0.129163419
239113_at	LOC100507468	1.165534985	0.099206037
210926_at	POTEKP	1.164880561	0.051103756
229280_s_at	LINC00340	1.162130857	0.36866647
216242_x_at	POLR2J4	1.160858427	0.158076203
208511_at	PTTG3P	1.16082224	0.167525912

230077_at	LOC220729	1.16030001	0.322445527
209805_at	PMS2CL	1.160198586	0.120296421
242528_at	HOXA-AS2	1.159007841	0.180749872
1556931_at	CDC42-IT1	1.158566075	0.31664929
207394_at	ZNF137P	1.15800032	0.150243583
1568593_a_at	NUDT16P1	1.15796359	0.018391015
1556221_a_at	RP11-757F18.5	1.157645305	0.237843657
222208_s_at	POLR2J4	1.156760126	0.106944484
227301_at	CCT6P3	1.156696245	0.194647618
1556630_at	CASC2	1.156536666	0.02633053
215599_at	GUSBP9	1.156271629	0.419423377
220918_at	RUNX1-IT1	1.156023485	0.238109535
230780_at	LINC00886	1.154703758	0.076398355
226546_at	LOC100506844	1.154411634	0.527615549
224517_at	POLR2J4	1.15431927	0.193468657
228198_s_at	FAHD2CP	1.154186715	0.477078788
230121_at	SERTAD4-AS1	1.151704669	0.172615276
205518_s_at	CMAHP	1.15160827	0.495081491
1563969_at	FLJ33360	1.151274362	0.29713219
235691_at	LOC729970	1.15019676	0.273500363
235606_at	LINC00883	1.150011262	0.424487482
239246_at	FARP1-IT1	1.149659886	0.492934647
1554907_a_at	HYDIN2	1.149506172	0.236822369
1561101_at	JAKMIP2-AS1	1.149490994	0.227862572
216111_x_at	PMS2P3	1.148962781	0.051741049
238716_at	LOC100506990	1.147738384	0.235250954
1560536_at	RP11-461A8.4	1.147526091	0.173162246
214756_x_at	PMS2P1	1.146815807	0.037583796
1557845_at	RP11-121L10.2	1.146290595	0.010196622
215412_x_at	PMS2P5	1.146279367	0.090280857
1566557_at	BAIAP2-AS1	1.146156047	0.357130579
1559412_at	LINC00478	1.1459908	0.411677901
209607_x_at	SLX1B-SULT1A4	1.145312574	0.128615312
239313_at	LOC401320	1.144951657	0.230760253
240382_at	RP3-512B11.3	1.144681513	0.303445529
228944_at	RP4-773N10.4	1.144451311	0.131518361
1563897_at	RP11-5O23.2	1.143368165	0.004575387
222312_s_at	RP11-33E12.2	1.14315358	0.12646552
239269_at	NEAT1	1.14286412	0.431021593
1561402_at	LINC00880	1.142028253	0.281826909
228773_at	LOC100506100	1.14199246	0.129884313
244481_at	RP11-483I13.5	1.141692821	0.060947743
1569476_at	DKFZP434L187	1.140985546	0.417219011
1569502_s_at	TP73-AS1	1.139582721	0.164366143
239243_at	ZNF638-IT1	1.138729071	0.334953238
239466_at	LINC00883	1.13837769	0.19562486
236109_at	RP11-50B3.4	1.137820336	0.150007402
222245_s_at	FER1L4	1.137432193	0.238245975
226332_at	FAM133DP	1.136884104	0.07107573
216339_s_at	TNXA	1.136565297	0.307847815

242518_at	RAD51-AS1	1.135311064	0.331816324
1558404_at	LINC00622	1.134838593	0.565016667
240572_s_at	LOC374443	1.134433476	0.312141018
236250_at	AFG3L1P	1.134241269	0.237137943
240365_at	LINC00669	1.133236345	0.139920351
228571_at	RBAKDN	1.1331019	0.110195852
230552_at	LOC284412	1.132664313	0.419882378
235696_at	RP11-11N9.4	1.132023632	0.542267678
223974_at	DLGAP1-AS2	1.131847865	0.231511186
243224_at	LOC100506302	1.131052436	0.309632649
243895_x_at	RP11-380M21.4	1.130655294	0.075050721
226693_at	SDHAP1	1.130039695	0.246425813
237031_at	RP11-21L23.2	1.129985484	0.281325533
210886_x_at	TP53TG1	1.129769984	0.153297957
210580_x_at	SLX1B-SULT1A4	1.129668952	0.208457541
238432_at	DLGAP1-AS1	1.129606013	0.204898516
1552607_at	FAM223A	1.128207404	0.053054151
203245_s_at	LINC00094	1.128193506	0.300108016
216843_x_at	PMS2P1	1.127291736	0.053977259
1567359_at	BDNF-AS	1.126975237	0.005027613
219139_s_at	CROCCP3	1.126422447	0.189764462
215002_at	LOC613037	1.126202239	0.089431266
243048_at	CECR7	1.12589561	0.096452917
1563529_at	HYDIN2	1.124900495	0.417615331
228528_at	C1orf132	1.124577487	0.467252755
244695_at	GHRLOS	1.124548531	0.223092822
242786_at	SBF2-AS1	1.124547345	0.164948204
217485_x_at	PMS2P1	1.124408234	0.06640152
241014_at	FLG-AS1	1.124388453	0.505838519
1559966_a_at	ZFHX4-AS1	1.124312849	0.486746872
229917_at	RP11-571M6.8	1.123612976	0.666190127
232571_at	RP11-27M15.1	1.122986722	0.154555145
239210_at	RP11-477H21.2	1.122733687	0.529836372
222274_at	ZDHHC8P1	1.122613028	0.367020324
1553992_s_at	NBR2	1.121996123	0.331390363
229204_at	RP5-930J4.4	1.12115611	0.190022762
238704_at	RP11-498C9.15	1.120261442	0.405530726
228723_at	NPTN-IT1	1.120086636	0.468729597
210876_at	ANXA2P1	1.119203636	0.316170478
1552793_at	C8orf31	1.118441846	0.563972389
1564371_a_at	CASC2	1.117929691	0.066642421
1560573_at	LINC00944	1.117886841	0.076985592
1568685_at	RP11-229P13.25	1.117717585	0.032331346
236756_at	CENPVP2	1.117491858	0.06979953
232140_at	LOC100132352	1.117249132	0.286283233
206819_at	POM121L9P	1.117161992	0.52031647
213367_at	LOC155060	1.116799976	0.216446161
219442_at	CLUHP3	1.116303984	0.266760419
214526_x_at	PMS2P1	1.115881607	0.137066066
221393_at	TAAR3	1.114887741	0.133678772

239699_s_at	PMS2P5	1.114867645	0.190937611
215247_at	LOC100288570	1.113149625	0.201661921
1558969_a_at	RPL32P3	1.112955804	0.247740758
1559543_at	LINC00441	1.112011964	0.144030578
1557314_at	DPY19L2P3	1.111857168	0.167674705
1558809_s_at	ZNF790-AS1	1.11165415	0.478512522
1559315_s_at	SOCS2-AS1	1.111427889	0.482158438
1559240_at	LOC100507053	1.110594765	0.420175884
237322_at	MIAT	1.109772277	0.678976286
240557_at	RP11-145F16.2	1.109532648	0.396216253
241468_at	RP13-20L14.1	1.109515929	0.227700147
209917_s_at	TP53TG1	1.108468583	0.286668493
237298_at	FLJ26850	1.108339936	0.233572081
222087_at	PVT1	1.107597224	0.3665057
1558849_at	LINC00908	1.107321314	0.046026351
1560637_at	RP11-30L15.6	1.106987653	0.116363999
237357_at	OSTM1-AS1	1.106597105	0.095705758
227591_at	SH3BP5-AS1	1.106507399	0.403851099
1561110_at	RP1-179N16.6	1.105786367	0.053521396
238881_at	RP4-714D9.5	1.10558477	0.119124521
239950_at	HOXA11-AS	1.105529108	0.231249806
229627_at	LOC100499484-C9ORF174	1.105284649	0.460968863
232899_at	RPL23AP7	1.105219947	0.244824792
237065_s_at	LAMTOR5-AS1	1.10476052	0.3549242
1569089_a_at	LINC00957	1.104242087	0.162228178
239625_at	RP11-1109F11.3	1.103877292	0.259986664
224507_s_at	MGC12916	1.103795353	0.466798949
1565715_at	RP11-388M20.6	1.103400985	0.075657553
1562568_at	LOC400541	1.103225257	0.048379386
1568640_at	PCBP1-AS1	1.103223096	0.193741993
232463_at	LINC00685	1.103210815	0.370724513
230666_at	HOXA11-AS	1.102963234	0.366138796
242723_at	RP11-932O9.10	1.10292181	0.336570469
222055_at	FAHD2CP	1.102883378	0.183251716
211241_at	ANXA2P3	1.102853776	0.271144753
1553024_at	DAOA-AS1	1.102681459	0.006391759
230948_at	RP11-95D17.1	1.102504225	0.143752074
219876_s_at	GOLGA2P5	1.102171559	0.294059738
232527_at	PSMD6-AS2	1.102079534	0.37137194
240609_at	LOC100996654	1.101730809	0.381444247
225857_s_at	SNHG17	1.101649673	0.502721655
239521_at	MFI2-AS1	1.101153877	0.302615235
1556004_at	RP11-356K23.1	1.100742145	0.032093993
240890_at	LOC643733	1.099878724	0.137427908
233602_at	RP11-95O2.1	1.099223668	0.418837487
228629_s_at	WWTR1-AS1	1.098658726	0.052883919
240017_at	PCBP1-AS1	1.09858061	0.03189808
237225_at	ZFY-AS1	1.097136428	0.081125201
229041_s_at	ITGB2-AS1	1.095486587	0.448674222
1561319_at	OTX2-AS1	1.094852315	0.318779078

207283_at	RPL23AP32	1.094823857	0.48457268
214682_at	PKD1P1	1.094657618	0.495804417
1559067_a_at	RP11-4O1.2	1.094613599	0.61716373
241366_at	RBAKDN	1.094514721	0.519012851
1556220_at	RP11-757F18.5	1.094197655	0.25400795
1563271_at	RP11-1008C21.1	1.094130119	0.154354168
215009_s_at	THAP9-AS1	1.094016263	0.465158106
228647_at	LOC100049716	1.093908229	0.533721774
243813_at	LINC00968	1.09341324	0.105871601
1558882_at	HTATSF1P2	1.093286938	0.418243248
240912_x_at	LINC00277	1.093208792	0.074667578
232715_at	RP11-1024P17.1	1.093101126	0.011372374
1562681_at	KRTAP5-AS1	1.09298748	0.266627576
244570_at	RP1-151F17.1	1.092980666	0.433320873
238104_at	LINC00106	1.092781526	0.215814324
244705_at	RP11-3B12.1	1.091883258	0.11292418
1562167_a_at	DEFB122	1.091878342	0.034206329
1552868_at	CIRBP-AS1	1.091844388	0.034737349
239151_at	BMS1P5	1.091754603	0.538854883
236950_s_at	LINC00964	1.09162691	0.398821847
220602_s_at	LOC642423	1.091417615	0.263382194
235942_at	FAM224B	1.091150052	0.100015458
231033_at	RP11-408H20.1	1.090941815	0.075177512
215102_at	DPY19L1P1	1.090745882	0.336147507
241434_at	RP11-767N6.7	1.090696514	0.38913672
1570009_at	LOC643711	1.090229365	0.038518264
227306_at	RP11-363E7.4	1.090157762	0.358698452
233130_at	THRB-IT1	1.089728261	0.598019725
242201_at	PMS2P5	1.089718388	0.464631805
1569078_at	LOC100294362	1.089345013	0.324470366
243656_at	LOC642852	1.089336252	0.416608111
230080_at	HOXA-AS2	1.089196326	0.35444404
235776_x_at	LINC00475	1.088504658	0.090829533
238540_at	LOC401320	1.088219731	0.441980468
1557869_at	RP11-520H14.7	1.08814125	0.062451252
238463_at	LOC100506834	1.087704902	0.126997238
242213_at	RP11-727F15.11	1.08719521	0.296249349
235785_at	RP5-991G20.4	1.087178275	0.308379265
234488_s_at	GMCL1P1	1.087134741	0.477310663
226340_x_at	WASH2P	1.086830663	0.464454583
1557261_at	WHAMMP3	1.086768228	0.594990858
239873_at	RP11-334J6.6	1.086563324	0.129929812
233069_at	PPP4R1L	1.086480378	0.090002424
1561760_s_at	LOC645513	1.08632046	0.416282661
1559544_s_at	LINC00441	1.086235549	0.318817996
230653_at	RP11-974F13.6	1.085921986	0.553853983
239993_at	LOC390705	1.085826205	0.092615687
1559375_s_at	RP11-194N12.2	1.085666751	0.258378952
1566761_a_at	LINC00884	1.08547238	0.117336976
240014_at	POLR2J4	1.085321155	0.376615544

244489_at	RP11-263K19.4	1.085033076	0.455014762
229609_at	NUTM2A-AS1	1.084779071	0.339141538
1558290_a_at	PVT1	1.084717531	0.639925079
229699_at	LOC100129550	1.08431219	0.484344037
227969_at	PCBP1-AS1	1.083868415	0.352998274
233172_at	LOC100507073	1.083669426	0.069946756
1568915_at	U73166.2	1.083249296	0.451760292
207458_at	RHPN1-AS1	1.083120251	0.266483196
231950_at	ZNF658B	1.082964418	0.544140154
229006_at	LOC100507487	1.082913689	0.390617206
1556257_at	LOC645513	1.082502457	0.492418487
210241_s_at	TP53TG1	1.082478234	0.305568661
1563745_a_at	ZMIZ1-AS1	1.082419984	0.207838971
234078_at	ADAMTS9-AS2	1.082056487	0.36656792
1558850_s_at	LINC00908	1.081347946	0.231490491
227554_at	MAGI2-AS3	1.081285969	0.586708365
223725_at	LINC00852	1.080990918	0.24443816
1562764_at	RP11-13K12.5	1.080575226	0.087392222
238932_at	RP11-145F16.2	1.080352788	0.588562594
225035_x_at	WASH2P	1.080352157	0.517420043
1553608_a_at	LINC00189	1.079812047	0.381827093
206021_at	SCAND2P	1.079684611	0.441995504
1561037_a_at	RP11-396C23.2	1.07966761	0.168979865
216196_at	HERC2P4	1.079568171	0.085473755
222947_at	LOC100379224	1.078854981	0.622870154
239470_at	C15orf56	1.078844617	0.22583926
241614_at	RP11-471B22.2	1.078617721	0.222560208
242358_at	RASSF8-AS1	1.078230845	0.632589491
231307_at	RP11-382F24.2	1.078014149	0.013806652
238790_at	LOC374443	1.077869953	0.511692248
221194_s_at	TBC1D3P1-DHX40P1	1.077843865	0.605140213
242087_x_at	MFI2-AS1	1.07784151	0.467692117
239612_at	LOC100240734	1.077791143	0.364903631
233770_at	RP1-86D1.3	1.077639568	0.565493104
235878_at	RP11-95D17.1	1.077510115	0.408556724
210818_s_at	GRIK1-AS2	1.077476953	0.51990964
231375_at	RP11-1277A3.1	1.07722764	0.209921273
240063_at	GUSBP5	1.076550811	0.418810124
231260_at	ZNF582-AS1	1.076325998	0.592979581
1561478_at	LINC00560	1.07612888	0.137073199
214657_s_at	NEAT1	1.076013776	0.783664978
1556081_at	RP11-16P6.1	1.07563134	0.700535473
217625_x_at	LINC00963	1.075530915	0.46494987
234913_at	TTTY4B	1.075522832	0.091904547
232396_at	RP11-617F23.2	1.075352962	0.552134446
230631_s_at	IL10RB-AS1	1.075230852	0.297127067
1561666_a_at	PSMG3-AS1	1.075086057	0.406720296
1553881_at	MGC16142	1.074672918	0.47147388
243871_at	LOC100130476	1.074041533	0.150800404
206655_s_at	SEPT5-GP1BB	1.073735029	0.67717871

1557263_s_at	RP11-66N24.3	1.073515314	0.378954176
228549_at	RP11-285F7.2	1.07350947	0.516404774
1556262_at	PWRN1	1.073452006	0.174101808
1556639_at	LOC100996455	1.073307551	0.448422663
222290_at	OR2A9P	1.07317114	0.530607545
218296_x_at	MSTO2P	1.072761789	0.245329622
230491_at	RP11-174G6.5	1.072716953	0.425103714
223945_x_at	RP9P	1.072410383	0.42711608
1562638_at	LOC339874	1.072301695	0.142549078
233176_at	LOC100507642	1.071852378	0.384306194
227390_at	MEG3	1.071822095	0.641141854
217261_at	TTTY2B	1.071789633	0.563540636
1565073_at	RP11-118G23.1	1.070979039	0.124058486
235779_at	ZNF790-AS1	1.070682908	0.663156227
1559651_at	CSNK1A1P1	1.070655522	0.056994593
236632_at	HHIP-AS1	1.070646125	0.465382861
237267_at	LOC100506675	1.070620595	0.090648285
1564224_x_at	C1RL-AS1	1.070462447	0.204837673
242893_at	RP11-3L8.3	1.070018576	0.08700577
232645_at	LOC153684	1.069928429	0.588867238
1559136_s_at	LINC00894	1.06987285	0.271087545
1557190_at	RP11-511H23.2	1.069771814	0.224318548
238553_at	BMS1P5	1.069742315	0.506538056
243629_x_at	MFI2-AS1	1.069561613	0.549463319
1560176_s_at	PPP4R1L	1.069525673	0.096552689
227631_at	RP11-363J17.1	1.069476823	0.258951379
240447_at	HYI-AS1	1.069466449	0.26948481
1555614_at	SUGT1P1	1.069450716	0.149844373
236856_x_at	RP11-458D21.2	1.069441793	0.662422606
228826_at	BZRAP1-AS1	1.069415143	0.56782365
219173_at	MYO15B	1.069104744	0.423279649
1557862_at	LOC654841	1.068899224	0.072945282
230251_at	LINC00473	1.068761622	0.206352519
228055_at	NAPSB	1.068711458	0.789605859
228828_at	BZRAP1-AS1	1.068629815	0.444053421
1569332_at	LINC00488	1.068519377	0.444645905
1567361_at	BDNF-AS	1.068498023	0.3451866
236654_s_at	RP11-362K14.7	1.068479649	0.501446061
1561106_at	LINC00271	1.068401585	0.336680688
1558425_x_at	LINC00265	1.068309321	0.286602729
1560690_at	RP11-159H3.2	1.068150026	0.11838483
242393_x_at	BMS1P5	1.068119103	0.550759653
1566147_a_at	ANKRD20A12P	1.067742892	0.473673847
1560147_at	WDR86-AS1	1.067647628	0.181751931
232241_at	LINC00894	1.067482319	0.112773336
219563_at	LINC00341	1.067467822	0.708632345
1560957_at	RP5-857K21.5	1.067376784	0.269736133
1556222_at	SEPT7P9	1.067134712	0.221044394
1554733_at	LOC728175	1.067041271	0.177845649
243686_at	RP11-438B23.2	1.067031182	0.722185986

1558123_at	LINC00957	1.066947617	0.53085534
212732_at	MEG3	1.066858614	0.87841316
1566108_at	RP11-362K14.5	1.066783221	0.309211929
1556798_a_at	RNF144A-AS1	1.06670715	0.503687352
1566605_at	RP11-48G14.2	1.066661984	0.357028098
1552468_a_at	DSCR10	1.066446488	0.107154132
240194_at	LOC441204	1.066338445	0.603241062
214148_at	LOC100507424	1.066140284	0.548934202
227640_s_at	RP9P	1.066128858	0.486945132
228441_s_at	RP11-164P12.5	1.066037132	0.473803137
1564208_x_at	LINC00957	1.065756921	0.372202234
1553817_at	POM121L10P	1.065675142	0.215421507
233320_at	TCAM1P	1.065652862	0.471528958
1569607_s_at	ANKRD20A11P	1.06514245	0.801285875
237848_at	LOC100996671	1.065127242	0.054065936
244269_at	RP11-321E2.4	1.064846609	0.117834482
236246_x_at	LOC653160	1.064833612	0.20863839
1558660_at	LINC00703	1.064815742	0.225433797
220694_at	ASAP1-IT1	1.064771939	0.638455133
244620_at	SLC8A1-AS1	1.064684681	0.425583343
215288_at	TRPC2	1.06439406	0.202263501
1560306_at	RP11-68I3.11	1.064383274	0.55420979
214123_s_at	NOP14-AS1	1.064167858	0.566914092
235010_at	ZBED5-AS1	1.064030804	0.601448323
235314_at	RPL32P3	1.063887978	0.348001411
1562152_at	RP5-933E2.1	1.063620392	0.167225972
1569785_at	RP11-456K23.1	1.063505043	0.49054474
234611_at	LGALS8-AS1	1.063385501	0.212229306
230743_at	HOXB-AS3	1.063353595	0.137356339
1561327_at	LINC00242	1.063226279	0.302156531
1556229_at	RP11-235E17.4	1.063131118	0.487476769
235428_at	LOC100507316	1.063071807	0.751562506
1552717_s_at	CEP170P1	1.062843043	0.635792311
240423_at	LOC441204	1.062515699	0.7763529
243059_at	FENDRR	1.062122996	0.221112878
1569263_at	RP13-516M14.8	1.062033654	0.518420674
1563913_at	RP11-572M11.3	1.061987946	0.100037457
1569372_at	LOC100507194	1.061984226	0.377088208
1570298_at	RP11-863H1.1	1.06097807	0.105215796
1553518_at	DEFT1P2	1.060898117	0.081688801
242331_x_at	LOC642236	1.060882531	0.198737163
231062_at	DOCK9-AS2	1.060736011	0.252104572
1562738_a_at	USP3-AS1	1.06053969	0.488637897
244242_at	RP11-195F19.9	1.060262921	0.433935132
1561320_at	RP11-133K1.8	1.060179388	0.366662632
1561075_at	LOC101060553	1.060086835	0.267157603
1566967_at	SPRY4-IT1	1.059768285	0.65079305
207229_at	KLRAP1	1.059679441	0.582859261
231061_at	RP11-148B6.1	1.059668763	0.593019918
1556263_s_at	PWRN1	1.059593758	0.265531292

1566832_at	TOP1P2	1.059556278	0.199332983
1569832_at	LOC100131655	1.059356284	0.355252589
1561367_a_at	LINC00540	1.058956511	0.143347314
1564626_at	RP11-81H14.1	1.05892033	0.235453483
229628_s_at	LOC100499484-C9ORF174	1.058828864	0.71240451
65588_at	SNHG17	1.058811653	0.692898992
230433_at	LOC729970	1.058402756	0.586557253
1562098_at	RP11-135F9.4	1.05815953	0.315090621
232664_at	LINC00954	1.058072953	0.222703276
242462_at	LINC00665	1.057902372	0.524232994
238042_at	RP11-417L19.5	1.057881489	0.497037125
1560954_at	RP11-202D1.2	1.057834167	0.082913836
1558148_x_at	BAIAP2-AS1	1.057744409	0.449594509
1558459_s_at	LOC401320	1.057513544	0.577317862
233527_at	RP11-677M14.3	1.057258057	0.174788651
235990_at	LOC100130987	1.057219046	0.593924314
1562348_at	LINC00664	1.057111739	0.305621178
1566558_x_at	BAIAP2-AS1	1.057010276	0.634949704
1556679_at	RP11-567L7.3	1.056629658	0.132225704
1559026_at	PPP1R26-AS1	1.05641004	0.326787132
1565265_at	RP11-587D21.4	1.056335346	0.2059116
1560746_at	NEXN-AS1	1.05596054	0.179011167
1562823_at	RP11-24J23.2	1.055718134	0.382981889
1568687_s_at	ATP8B5P	1.055579445	0.194599471
1561352_at	RP11-245M24.1	1.055519323	0.244958833
228004_at	LINC00261	1.055488189	0.134007132
1553708_at	LINC00525	1.055273798	0.258466099
241947_at	PCED1B-AS1	1.055152199	0.374420932
219043_s_at	PDCL3P4	1.055062391	0.383368845
1570326_at	RP11-359E8.5	1.054931637	0.168373241
237645_at	RP11-413B19.2	1.054902011	0.206713653
234515_at	PCGEM1	1.054600274	0.294464608
239792_at	UBL7-AS1	1.05443919	0.698710249
217338_at	KRT19P2	1.054202614	0.260986816
1570623_at	RP11-96K19.4	1.054162703	0.391506344
240284_x_at	U47924.27	1.05415077	0.361942887
227563_at	FAM27E3	1.053933136	0.666105661
1560161_at	RP11-59H7.3	1.053757883	0.158082413
1563083_s_at	LINC00486	1.053695105	0.048021851
204486_at	KCNQ1OT1	1.053661029	0.27556052
1569958_at	RP4-694A7.4	1.053659163	0.133710765
231989_s_at	LOC613037	1.053566406	0.693666501
1560224_at	AHCTF1P1	1.053121428	0.517422413
1561625_at	RP5-968D22.3	1.053089929	0.359956713
227743_at	MYO15B	1.053080805	0.564249612
1558977_at	LOC100130992	1.052783495	0.331863437
1557895_at	FLJ35934	1.052558789	0.777814977
230963_at	EMX2OS	1.052438594	0.434303939
1570289_at	LOC646736	1.052373536	0.109007352
230267_at	RP11-173M1.8	1.052366618	0.615215244

208045_at	RP11-292D4.4	1.052262598	0.160066467
234262_at	RP11-319G9.1	1.052162334	0.666857969
227406_at	GABPB1-AS1	1.052089426	0.666438935
1562073_at	RP11-378A12.1	1.052061545	0.479038794
1563119_at	HP09025	1.052008795	0.258829845
1564257_at	ACTA2-AS1	1.051977765	0.314226339
1564165_at	LOC100630923	1.051654125	0.243973842
1561880_a_at	SIGLEC16	1.051611973	0.287584384
1557336_at	RP11-81N13.1	1.051530072	0.444827479
243674_at	RP11-374M1.5	1.051430867	0.349615205
234423_x_at	LOC100996255	1.051342683	0.548598474
235077_at	MEG3	1.051296247	0.897174003
1558310_s_at	LOC100132356	1.050972519	0.656100559
1556883_a_at	LOC440896	1.050860541	0.564875062
238944_at	LOC100505715	1.050768208	0.832389434
1560959_a_at	RP1-39J2.1	1.050756314	0.105512865
1553493_a_at	TDH	1.050578957	0.357966222
240519_at	RP11-141M1.1	1.050520919	0.14172018
230313_at	RP11-403P17.3	1.050408554	0.616983991
1568894_at	RP11-678G14.4	1.050298022	0.219994871
1563872_at	LOC284395	1.050168669	0.294163706
1568921_at	RP11-10C8.2	1.04994816	0.234563465
1563260_at	LINC00587	1.04984863	0.30564154
240537_s_at	CDIPT-AS1	1.049827768	0.42500355
231844_at	MGC27345	1.049406962	0.471054316
1561448_at	RP4-630C24.3	1.049325292	0.27523716
237219_at	PHKA1-AS1	1.049249754	0.331502043
230664_at	H2BFXP	1.049234952	0.816859366
1561741_at	RP11-92G12.3	1.048750932	0.687077606
227709_at	TPT1-AS1	1.048700363	0.699004771
1557211_a_at	FAM181A-AS1	1.0486464	0.261926221
210571_s_at	CMAHP	1.048608931	0.694509978
1570375_at	LOC100506585	1.048472065	0.433512559
236205_at	ABCC6P1	1.04839422	0.570773926
1556884_a_at	LINC00558	1.048379745	0.123593901
1561222_at	LINC00485	1.048328642	0.124408087
215105_at	N4BP2L2-IT2	1.048202395	0.408921557
1566843_at	PER4	1.04818739	0.140708483
237767_at	FARP1-IT1	1.048077387	0.334041525
1559497_at	RP11-597K23.2	1.047942287	0.195516498
233551_at	LOC642776	1.047939208	0.233935223
233063_s_at	RP1-80B9.2	1.047884672	0.665584544
236054_at	RP11-635N19.1	1.047802331	0.507723031
214858_at	PP14571	1.047782516	0.399941185
1558458_at	LOC401320	1.047721949	0.644347948
239421_at	DLGAP1-AS1	1.047642087	0.54102428
1558216_at	AFAP1-AS1	1.047424871	0.387369704
1560238_at	LINC00665	1.047330828	0.703334242
1560279_a_at	LOC221122	1.0473198	0.213292877
240708_at	PEX5L-AS2	1.047252771	0.214309386

236506_at	RP11-248J18.3	1.047165903	0.666473686
222054_at	PPIEL	1.046862297	0.6721389
234763_at	RP4-665N4.4	1.046805491	0.191742085
239115_at	RP11-358L4.1	1.046760162	0.290190923
231811_at	LOC100288778	1.046718719	0.516777204
1559501_at	CBR3-AS1	1.046594263	0.360486027
1560432_at	CLRN1-AS1	1.046511688	0.279536204
1570071_at	RP11-258F1.1	1.046384713	0.307579873
1561450_at	RP11-196O2.1	1.04630649	0.120382161
240424_s_at	LOC441204	1.046231249	0.783250871
1556454_a_at	LOC100506274	1.046107146	0.531106653
1553468_at	HYDIN2	1.045845825	0.284192201
235534_at	RP11-134G8.8	1.04579583	0.81599936
224181_at	LINC00470	1.04566266	0.318110867
235126_at	FLVCR1-AS1	1.04564517	0.595992553
1570587_at	MPRIP-AS1	1.04554146	0.171602528
1570388_a_at	LOC440896	1.045469286	0.467578119
238011_at	RP11-473I1.9	1.045466825	0.629972815
1562691_at	RP11-473L15.2	1.045198498	0.24492568
1560750_at	LOC151121	1.045096623	0.361497523
1570049_at	LINC00910	1.044996137	0.30046921
242994_at	RP4-657D16.3	1.044963857	0.459900338
232131_at	LINC00933	1.04490182	0.173937681
239764_at	ITPR1-AS1	1.044753473	0.736785442
1554862_at	CMAHP	1.044667049	0.200687586
1553829_at	CYP1B1-AS1	1.044604793	0.481567098
234666_at	RP11-685B24.1	1.044350162	0.18315861
232853_at	RP11-552M11.8	1.044244416	0.427307803
220157_x_at	PLEKHA8P1	1.044165862	0.614209401
1561036_at	RP11-396C23.2	1.044128219	0.394004022
235137_at	PCBP1-AS1	1.044047117	0.471350134
1561223_at	RP11-677O4.2	1.043924649	0.377361007
1565852_at	RP11-4B16.1	1.043882906	0.533943032
1557881_at	ZNF32-AS3	1.043882119	0.246550395
1563296_at	LINC00572	1.04386696	0.352417644
234189_at	RP11-669C19.1	1.043829525	0.200741087
1563993_at	RP11-770E5.1	1.043741781	0.217392608
1563145_at	LINC00301	1.043536734	0.288187909
233506_at	RP11-469M7.1	1.043521442	0.806359692
239162_at	DAPK1-IT1	1.043519142	0.817919475
1557474_at	LOC284578	1.043367258	0.627890146
241183_at	RP11-457K10.1	1.043347723	0.227126582
215985_at	ZNRD1-AS1	1.043105658	0.759702743
1557446_x_at	TREML3P	1.043033162	0.262730197
1558938_at	RP11-468E2.5	1.042960631	0.538582431
241239_at	EPN2-IT1	1.042881112	0.394226398
223896_at	FKSG29	1.042800657	0.187569288
1562220_at	RP11-195M16.3	1.042783196	0.325987394
1557557_at	MATN1-AS1	1.042587684	0.556517258
232070_at	LOC100506639	1.042450583	0.450935665

1560202_at	CSTF3-AS1	1.042444123	0.162127628
1560950_at	HECW1-IT1	1.042439646	0.260751026
210646_x_at	RPL13AP5	1.042380743	0.608595537
1570423_at	FLG-AS1	1.042183266	0.281962618
214151_s_at	DYX1C1-CCPG1	1.042175787	0.775216065
206302_s_at	NUDT4P1	1.04210202	0.858154263
1560673_at	RP4-777D9.2	1.042066316	0.283888722
235617_x_at	LOC100507537	1.042025393	0.284820254
229811_at	RP11-157P1.4	1.041959507	0.607885162
229716_at	RP3-340N1.5	1.04186672	0.585842154
1561691_at	LINC00326	1.041750826	0.381753424
212868_x_at	MAPKAPK5-AS1	1.041625803	0.317256249
243924_at	LINC00665	1.041484669	0.728975776
232354_at	RP11-463O12.5	1.041457775	0.749044892
213908_at	WHAMMP3	1.041326275	0.713051684
229722_at	LOC100270804	1.04123025	0.641494346
1557604_at	LOC401312	1.040915091	0.286572337
224597_at	LINC00657	1.040728301	0.631348953
241405_at	TOB1-AS1	1.040715324	0.517491346
1553145_at	TAPT1-AS1	1.040577979	0.79909737
1561418_at	RP11-265N7.1	1.040510773	0.430795041
224508_at	MGC12916	1.040508382	0.736088225
243092_at	PAN3-AS1	1.040447001	0.764751055
1557290_at	DPY19L2P4	1.040347882	0.551965219
1556528_at	LINC00692	1.040285198	0.303504423
232284_at	PSMD6-AS2	1.040252947	0.531583605
1556272_a_at	RP11-843A23.1	1.040079126	0.256414428
1561431_at	RP11-284F21.8	1.040007662	0.466723727
1562336_at	CASC2	1.039977927	0.45548035
224457_at	FOXD2-AS1	1.039973872	0.506710484
236579_at	NKX2-1-AS1	1.039829221	0.456550591
1558920_at	SLC8A1-AS1	1.039726264	0.739838797
1559061_at	CACNA1G-AS1	1.03962741	0.440567643
1565563_at	RP11-159D8.2	1.039461329	0.14009255
1553435_at	C18orf15	1.039395899	0.492295459
236626_at	NAGPA-AS1	1.039389517	0.751720681
1570206_at	RP11-500B12.1	1.03934633	0.349858733
228199_at	FAHD2CP	1.039227839	0.797811672
211351_at	KIF25-AS1	1.039158744	0.451314346
241247_at	RP11-167H9.6	1.039144397	0.293128631
222367_at	WHAMMP3	1.038870824	0.498855199
242305_at	LOC645513	1.038825741	0.662111416
235445_at	LOC100508046	1.038810675	0.234580578
1557602_at	LINC00870	1.038766514	0.341989895
236751_at	RP11-214K3.19	1.038719205	0.661229067
233772_at	POU6F2-AS2	1.038668671	0.27970677
229014_at	NR2F1-AS1	1.038575023	0.680536911
1560697_at	TRHDE-AS1	1.038357769	0.639099587
214344_at	LINC00950	1.038288028	0.677735743
1564207_at	LINC00957	1.038204837	0.666626013

241943_at	C18orf61	1.037964454	0.371428076
226382_at	LOC283070	1.037957924	0.845853589
231586_at	SPATA42	1.037921052	0.423135813
242649_x_at	HMGN2P46	1.037778916	0.467658502
1556924_at	CFLAR-AS1	1.037772426	0.371787462
1560340_s_at	RP9P	1.037721703	0.642910216
216276_s_at	ADAM3A	1.037658845	0.20645411
1552287_s_at	AFG3L1P	1.037609065	0.78467378
238924_at	BMS1P5	1.037562044	0.803175363
1556103_at	RP11-480A16.1	1.037553054	0.723039666
1569882_at	NPHP3-AS1	1.037392502	0.343988611
231581_at	LINC00664	1.037318156	0.437148685
220354_at	MCF2L-AS1	1.037292092	0.596156361
220983_s_at	NEAT1	1.037244303	0.737326138
1556820_a_at	DLEU2	1.037220195	0.809554316
1563168_at	NRG1-IT1	1.037121579	0.386044725
236469_at	RP11-1007O24.2	1.037106694	0.62785232
239279_at	LOC730102	1.036808426	0.627160451
1560131_at	LOC100506497	1.036769955	0.683097169
233507_at	RP11-1081M5.2	1.03676892	0.388300764
238097_at	GAS6-AS1	1.036574204	0.412254878
1561560_at	RP11-95H11.1	1.036568562	0.468564607
230139_at	RP11-121C2.2	1.03656618	0.731648451
1562478_at	LINC00659	1.036528401	0.523229247
229493_at	HOXD-AS2	1.036439943	0.673190296
1560241_at	RP11-178L8.5	1.036421144	0.426050803
237088_at	LINC00244	1.036347843	0.380104336
231318_at	DNM1P46	1.03621248	0.455098987
1558144_at	MEG3	1.03611273	0.433072576
1558147_a_at	BAIAP2-AS1	1.035981066	0.570903005
232886_at	RP11-55K13.1	1.035896675	0.65858041
1568849_at	LINC00165	1.035828932	0.496301768
1557380_at	AGAP11	1.035817655	0.472996161
1556624_at	RP11-214K3.20	1.035485919	0.38986161
1563135_at	RP11-955H22.3	1.035326467	0.290243165
1558847_at	LINC00565	1.035245658	0.630582663
1569659_at	LOC100506895	1.035083806	0.33356319
212790_x_at	RPL13AP5	1.035019841	0.686374308
243986_at	LINC00355	1.034810211	0.422164687
215110_at	MBL1P	1.034802006	0.493816529
1556936_at	LOC100506834	1.034684033	0.610030685
1557791_at	RP4-676L2.1	1.034630168	0.408179828
242012_at	RP11-308D16.4	1.034610368	0.619370355
233926_at	RP11-102L12.2	1.034446804	0.520392036
215825_at	RP11-255C15.3	1.034355738	0.564357864
1558628_at	RBFADN	1.034285369	0.567085518
239321_at	LOC441454	1.034171526	0.539133035
239713_at	CASC2	1.03407357	0.452958181
227035_x_at	RP9P	1.033672416	0.676357025
1569577_x_at	RP11-462G12.1	1.033385919	0.827537744

1562965_at	RP11-109E24.1	1.033362562	0.50155653
1555040_at	LINC00612	1.033315421	0.461156162
243996_at	RP11-112J3.16	1.03325357	0.841130128
1564443_at	DLEU2	1.0332373	0.5372771
1569753_at	RP11-64D24.4	1.03316508	0.434220494
231924_at	LINC00958	1.033162356	0.651326723
224271_x_at	FRMD8P1	1.033051167	0.509826445
241312_at	LINC00911	1.032974816	0.519008072
239590_x_at	FAM95B1	1.032957169	0.728014934
1569833_at	RP11-387D10.3	1.032952616	0.504938856
1564338_at	RP4-591N18.2	1.032916701	0.574702252
235403_at	RP11-108K3.2	1.03288244	0.471538666
241904_at	LIPE-AS1	1.032865826	0.558516579
1556879_at	RP11-386M24.6	1.032782739	0.541582681
240100_at	LOC100505920	1.032749066	0.471112517
1561034_at	RP11-510C10.2	1.032641772	0.649475507
1552876_at	LINC00334	1.032538749	0.387015982
241432_at	SLIT2-IT1	1.032472374	0.437595025
240615_at	PTOV1-AS1	1.032195231	0.68538348
1557607_at	LOC284080	1.032136148	0.444394156
228779_at	LOC146880	1.032034828	0.607367147
231028_at	LOC100506082	1.031939426	0.654665862
243171_at	RP11-775D22.2	1.031881019	0.529111853
1569713_at	SEC24B-AS1	1.03184001	0.647240016
1557597_at	LINC00624	1.031653273	0.372623114
1562689_at	LOC151484	1.031640837	0.419054989
215428_at	RP11-680F8.4	1.031551942	0.74215549
242854_x_at	DLEU2	1.031523925	0.720968439
1568832_a_at	RP11-533E19.5	1.031362907	0.597454589
244164_at	FAM223B	1.031362363	0.553721528
1554765_a_at	LINC00301	1.031314692	0.567355362
240755_at	RP11-787B4.2	1.031248571	0.509108508
230328_at	RRN3P1	1.031224854	0.719771895
243983_at	RP11-120K24.5	1.031194767	0.425498023
1563229_at	DLEU2	1.03117381	0.820117458
1561230_at	RP11-171I2.1	1.031020227	0.346121394
225197_at	RP11-473I1.10	1.031015935	0.782226411
238237_at	LOC100130964	1.03090686	0.365189078
1556504_at	RP11-732A21.2	1.030828183	0.365670827
1561468_at	RP1-274L7.1	1.030710469	0.550886914
220846_s_at	RP11-181G12.2	1.030577007	0.646195442
1561850_at	LOC100133669	1.030472268	0.652475009
1564685_a_at	RP1-257A15.1	1.030448394	0.522790993
222328_x_at	MEG3	1.030382056	0.672840569
1559140_at	FAM87B	1.030354342	0.657116706
1570391_at	RP11-661P17.1	1.030268993	0.35276542
1555869_a_at	LOC100507477	1.03024264	0.354977857
1556645_s_at	RP11-875O11.1	1.03012868	0.668185991
243979_at	RP11-506N2.1	1.030101012	0.365223171
236283_x_at	LOC646214	1.030080154	0.71375914

1556821_x_at	DLEU2	1.030029186	0.837948466
1556460_a_at	ARHGAP22-IT1	1.030025849	0.505193187
1554781_at	MGC32805	1.030017623	0.465262697
1566253_at	SH3GL1P2	1.030011706	0.683823299
1560842_a_at	TEX26-AS1	1.029927626	0.682481956
1564309_at	LINC00486	1.029914086	0.535818977
1561021_at	RP11-96H17.1	1.029869392	0.367315842
1563370_at	RP11-672A2.6	1.029862175	0.472843478
1565150_at	RP11-697E2.4	1.029825592	0.614616999
227389_x_at	RP4-781K5.2	1.029805748	0.436440376
231760_at	LINC00029	1.029721957	0.496097723
1559467_at	RP11-81A1.6	1.029592811	0.727757936
237321_at	LOC100506457	1.029550148	0.721361563
233113_at	ADAMTS9-AS2	1.029527731	0.681135468
1562449_s_at	LARGE-AS1	1.029514971	0.40624629
211940_x_at	H3F3AP4	1.029475123	0.794827199
233598_at	C20orf187	1.029408925	0.511708792
240346_at	RP11-834C11.8	1.029389128	0.524926257
1556749_at	RP11-433J8.1	1.029338776	0.685949812
238413_at	RRN3P3	1.029319194	0.689165523
206279_at	PRKY	1.029311556	0.788963433
241256_at	RP11-446F17.3	1.029116263	0.572795467
1566916_at	HPYR1	1.029012138	0.430449369
1562496_at	LOC339539	1.0289225	0.532615881
217492_s_at	PTENP1	1.028896899	0.784002795
229815_at	TMEM161B-AS1	1.028882054	0.883324813
1559542_a_at	RP11-863K10.2	1.028848858	0.392386613
1560081_at	RAD51-AS1	1.028847211	0.72460918
230572_at	WWC2-AS2	1.02878551	0.600178466
1561493_at	RP11-1129I3.1	1.02867888	0.447448382
236832_at	ADCY10P1	1.028645864	0.857428603
1568905_at	RP11-86H7.1	1.028598028	0.634362743
241266_at	RP11-186F10.2	1.028538106	0.382088191
1561699_a_at	ATP11A-AS1	1.028527005	0.513610749
222051_s_at	RP11-219B4.3	1.028520225	0.569434808
1560760_s_at	RP1-13P20.6	1.028489345	0.491546139
233289_at	RP4-635A23.6	1.028450388	0.823068973
244035_at	RP11-28F1.2	1.028408295	0.717664317
1561487_at	RP11-300A12.2	1.028378407	0.521034393
1559459_at	LOC613266	1.02834664	0.574994115
238086_at	LOC100129617	1.028333043	0.500520225
240474_x_at	RP11-313C4.1	1.028278152	0.440178078
228290_at	PLK1S1	1.028204304	0.806361027
1569935_at	RP11-214K3.24	1.02813478	0.599619768
1556333_at	RP11-408H20.3	1.028130287	0.631297555
214107_x_at	LOC440434	1.028104183	0.775995255
1562520_at	RP11-168K9.2	1.027900059	0.508943952
1569750_at	RP11-385H1.1	1.027887687	0.683592419
243703_x_at	LIPE-AS1	1.027810653	0.471582977
1559145_at	RNF144A-AS1	1.027809124	0.684855094

237182_at	MRPL45P2	1.027795011	0.419402184
1557823_s_at	LOC401134	1.027690303	0.389586661
239106_at	CA5BP1	1.027662758	0.795142522
1566277_at	OR5E1P	1.027537374	0.550673096
242207_at	LOC100128361	1.027517633	0.674191063
230294_at	RP5-1125A11.1	1.027452916	0.775113918
1563048_at	RP5-1039K5.16	1.027325719	0.561032165
217588_at	CATSPER2P1	1.027235706	0.736385877
1553607_at	GRIK1-AS2	1.027147751	0.325029049
233740_at	RP11-454F8.4	1.027001403	0.629913751
217039_x_at	ELK2AP	1.026996702	0.595242107
244119_at	LINC00551	1.026996044	0.354492501
223733_s_at	PPP4R1L	1.026964185	0.699658903
1560872_at	RP11-513N24.1	1.026955075	0.593961345
231638_at	PRSS30P	1.0268996	0.71355336
234029_at	PCDHGB8P	1.026894339	0.526956609
1559655_at	MAMDC2-AS1	1.026783031	0.443298121
1556722_a_at	C20orf203	1.026756342	0.646394227
1561098_at	LINC00616	1.026711843	0.493751053
200716_x_at	RPL13AP5	1.02670637	0.772894311
1570015_at	CHIAP2	1.026703199	0.513819638
1562353_x_at	RP11-373E16.3	1.02667949	0.461512537
1557753_at	RP11-519M16.1	1.02649274	0.503623612
233583_at	RP11-560A15.4	1.026401601	0.535966738
1556256_a_at	RP11-61A14.1	1.026391529	0.499517643
242510_at	RP3-476K8.3	1.02638571	0.584306426
1562822_at	RP1-60N8.1	1.026375812	0.475863171
1559766_at	ALDH1L1-AS2	1.026329299	0.860185723
243961_at	LOC100505622	1.026308151	0.481759365
1554978_at	ERICH1-AS1	1.026295315	0.550931835
233591_at	RP11-476D10.1	1.026246699	0.342681474
1561273_at	HOXC-AS3	1.026179375	0.575666785
1556684_at	RPPH1	1.026171895	0.582401472
1560851_at	LINC00619	1.026153108	0.59179988
1559668_s_at	RP11-83N9.5	1.026040489	0.592377418
1562869_at	RP11-111I12.1	1.026027367	0.623596473
232016_at	RP11-540B6.6	1.025811637	0.462695596
1570008_at	GNN	1.025758561	0.543749979
244468_at	PDZK1P1	1.025715226	0.487144207
1557483_at	LOC284788	1.025694226	0.547657529
210909_x_at	LPAL2	1.025691772	0.653557891
1569293_x_at	CROCCP2	1.025647686	0.454719756
238148_s_at	ZNF818P	1.025589867	0.820599604
237015_at	RP11-679B19.1	1.025557433	0.900434263
1562960_at	KCNQ1-AS1	1.025515884	0.586916515
1569839_s_at	LRRC37A5P	1.025503521	0.590038543
232189_at	RP11-108P20.1	1.02542319	0.499547457
1556913_a_at	RP11-837J7.3	1.025412953	0.620208876
230910_s_at	LOC100288181	1.025391592	0.628782519
1560982_at	RP11-452L6.1	1.02534551	0.754007914

1562514_at	RP11-201A3.1	1.025315402	0.454130271
1553482_at	C15orf32	1.025309724	0.472034181
214984_at	SLC7A5P1	1.025306093	0.730511413
244608_at	RP11-384J4.1	1.025304683	0.739831843
230940_at	LOC100288123	1.025280544	0.540144027
1562032_at	RP4-539M6.14	1.02519602	0.581334944
225995_x_at	WASH2P	1.024852609	0.770502453
1559865_at	RP1-149C7.1	1.024779257	0.453573395
233840_at	LOC100130950	1.024591193	0.667537721
239356_at	DOCK9-AS2	1.024415185	0.607285307
235185_s_at	LOC388692	1.024404167	0.616480234
1561335_at	LOC440602	1.024362028	0.606173624
1568633_a_at	RP11-49I11.1	1.024312442	0.573665597
1559374_at	RP11-194N12.2	1.024167713	0.685999281
1561683_at	RP11-410C4.5	1.024135401	0.509028743
1560848_at	RP11-308D13.3	1.0240795	0.621758437
1562480_at	RP11-475A13.2	1.024041579	0.316172881
1561314_at	RP11-752D24.2	1.024037586	0.592776095
1560995_s_at	RP11-867G2.4	1.02402148	0.600530877
1569674_at	LINC00630	1.023741361	0.444295002
1563157_at	FOXP1-AS1	1.023593936	0.589912419
1562469_at	LINC00340	1.023491025	0.521141115
1563038_at	RP11-550P17.5	1.023405781	0.542018193
1560383_at	RP11-752D24.2	1.023335155	0.520945881
1555205_at	RP11-498C9.17	1.023284964	0.620939432
1562943_at	MACROD2-AS1	1.023234159	0.600988522
243440_at	SIX3-AS1	1.023199101	0.687484665
243225_at	FGF14-AS2	1.023187519	0.862895053
237605_at	RP5-1027O15.1	1.023158977	0.586718533
237034_at	RP11-185E8.2	1.023152251	0.892669479
1553311_at	C20orf197	1.023092607	0.740294192
1562839_at	LINC00894	1.022974486	0.782495127
1570291_at	CLDN10-AS1	1.022880326	0.61683699
1563040_s_at	LARGE-AS1	1.022810102	0.431566407
208273_at	ZNF670-ZNF695	1.022768221	0.661211536
1560255_at	CELF2-AS1	1.022636769	0.639913694
1555085_at	RP11-222A5.1	1.02257469	0.604086001
235712_at	GAS5-AS1	1.022529274	0.729974479
216902_s_at	RRN3P1	1.022508936	0.87928874
222669_s_at	SBDSP1	1.0224636	0.796512862
1568899_at	RP13-516M14.4	1.022438795	0.537117336
229171_at	RP11-566K11.5	1.022409273	0.589702838
1564300_at	IQCF5-AS1	1.022382678	0.596503585
1568636_a_at	LINC00354	1.022316532	0.567350502
1558903_at	LINC00907	1.022268856	0.640703988
216548_x_at	HMGB3P1	1.022245692	0.83154468
217499_x_at	OR7E37P	1.022231733	0.818784451
225920_at	LOC148413	1.022203102	0.748188461
215410_at	PMS2P1	1.022099484	0.674176197
1563927_a_at	LOC401463	1.022088318	0.569429951

1561347_a_at	RP11-433M22.2	1.022003749	0.557815795
235397_at	LINC00174	1.021897803	0.709966896
243200_at	RP11-369C8.1	1.021889892	0.514792906
213828_x_at	H3F3AP4	1.021874244	0.830478984
1561909_at	RP11-398G24.2	1.021737268	0.804854792
1562827_at	DIO2-AS1	1.021710634	0.676046396
1557889_at	RP11-1110F20.1	1.021706092	0.893461712
243432_at	CHL1-AS2	1.021705322	0.839794562
231347_at	LOC100996694	1.021655519	0.638138832
239762_at	LOC286437	1.021621786	0.858543953
242382_at	LOC100506207	1.021604468	0.628699293
227106_at	TMEM198B	1.021522613	0.818037206
1555456_at	RP11-185J20.1	1.021515167	0.618380253
1567686_at	CECR9	1.021484017	0.550211402
1559621_at	RP11-584P21.2	1.021389513	0.590329868
235362_at	LOC729970	1.021331146	0.85600614
1564158_a_at	VAC14-AS1	1.021249506	0.905187937
1556387_at	LOC100507389	1.021196763	0.533484162
1562034_at	LINC00163	1.021110229	0.537788606
1554716_s_at	LINC00593	1.020998452	0.680825439
1564610_at	RP11-1109M24.16	1.020943472	0.520010392
220752_at	LOC51145	1.020926415	0.697396837
231141_at	RP11-217B1.2	1.02091282	0.576399715
1557564_at	ALKBH3-AS1	1.020873843	0.631754098
239791_at	HOXB-AS3	1.020869016	0.725897894
240276_at	RP11-108P20.4	1.020855057	0.67798753
1553934_at	LINC00305	1.020828733	0.558960776
1553204_at	C20orf166-AS1	1.020739943	0.738888915
244823_at	LOC100129034	1.020361873	0.704780037
215658_at	LINC00675	1.020342097	0.631578623
1555681_at	RP11-542M13.1	1.020339175	0.651673521
241418_at	LOC344887	1.0203243	0.746240026
231226_at	MED14-AS1	1.02027958	0.654638624
1561442_at	LOC283585	1.020063714	0.650537517
243909_x_at	GUSBP4	1.019935645	0.76706944
1562051_at	LOC729296	1.01986182	0.800772224
243892_at	RP11-171N4.2	1.019720619	0.520287498
234529_at	PCGEM1	1.019644006	0.595810573
1569911_at	RP11-89M16.1	1.019614526	0.683694725
224132_at	RP11-45M22.5	1.019574446	0.719578993
1557610_at	PITRM1-AS1	1.019486052	0.713467812
1561608_at	RP11-472K22.1	1.01943486	0.554138747
233820_at	RP11-399O19.8	1.019377717	0.7699581
1561401_at	LOC285627	1.01932845	0.62754741
243905_at	LINC00944	1.019316431	0.642827145
1553457_at	LINC00269	1.019315529	0.560540215
1559141_s_at	FAM87B	1.01928673	0.861973805
1561676_at	PRICKLE2-AS3	1.019269893	0.645787566
232096_x_at	FOXP1-IT1	1.019246932	0.819444159
1560570_a_at	RP11-111J6.2	1.019229827	0.78434499

232346_at	LOC388692	1.019208114	0.840548022
1562341_at	RP11-475O6.1	1.019101613	0.60670821
1557149_at	RP11-432J24.5	1.019088848	0.690493906
1553067_a_at	GNRHR2	1.019044202	0.62627269
233552_at	RP11-573N10.1	1.018970652	0.658871547
1562865_at	RP11-642A1.1	1.018936662	0.611490691
1556625_a_at	RP11-214K3.20	1.018925588	0.582422924
1566251_at	SH3GL1P1	1.018908085	0.568745372
1556617_a_at	LINC00901	1.018875901	0.559671311
1561078_at	RP11-403N16.3	1.018800579	0.6342729
236394_at	A2MP1	1.018799025	0.698819754
236142_at	RP5-994D16.3	1.018781428	0.810333007
1557386_at	LOC100507600	1.018762527	0.681585108
230917_at	RP11-510J16.3	1.018662826	0.872211712
1560548_at	RP11-501G7.1	1.018595002	0.590464272
1562413_at	LINC00167	1.018569389	0.732985183
237745_at	TSC22D1-AS1	1.018439552	0.917691942
227290_at	RP11-18F14.2	1.018305261	0.927596484
1559079_at	LINC00567	1.018295326	0.680408136
1557486_at	ANKRD44-IT1	1.018138924	0.769308853
236585_at	RP5-894A10.6	1.018031979	0.825904511
1566833_x_at	TOP1P2	1.018030521	0.641469931
243729_at	RP11-747H7.3	1.017835912	0.898539044
1570315_at	HCCAT5	1.017712571	0.681150004
1561491_at	LOC283214	1.017708047	0.688378186
1568888_at	LOC100507283	1.017673351	0.645103185
217003_s_at	ADAM5	1.017640612	0.604608102
231096_at	PCAT4	1.01761786	0.717484689
239887_at	RP11-283G6.4	1.01753577	0.758127858
1562121_at	CHL1-AS1	1.017455736	0.679424999
1562623_at	LOC146513	1.017381247	0.658672845
220152_at	C10orf95	1.017317686	0.687457797
1560940_at	SACS-AS1	1.017274592	0.623442314
235673_at	RP11-95D17.1	1.017144153	0.747569206
240986_at	RP11-17G12.3	1.017114707	0.707836632
235480_at	LOC100506472	1.017108366	0.741513226
236451_at	LOC100996579	1.016897518	0.632260181
1561979_at	LOC100505635	1.016889282	0.696726185
1563581_at	RPL34-AS1	1.016879954	0.693933768
233965_at	TBX5-AS1	1.016863904	0.706655799
1561239_at	RP11-401I19.2	1.016725225	0.726661732
1555822_at	FAM138B	1.016677082	0.682675208
240192_at	GATA3-AS1	1.016617701	0.610155527
1561606_at	RP11-579O24.3	1.016602688	0.682584252
215683_at	RBFADN	1.016541282	0.792014959
230845_at	HOXB-AS5	1.016472182	0.760037727
221511_x_at	DYX1C1-CCPG1	1.01641199	0.910789873
233746_x_at	SERF2-C15ORF63	1.016411607	0.838190967
1560402_at	GAS5	1.016382373	0.804896288
1554996_at	ZNF733P	1.016338573	0.676884404

227941_at	LOC339803	1.016318051	0.901042614
1556445_at	RP11-90P13.1	1.016302565	0.731092047
223977_s_at	LINC00470	1.016247876	0.734564126
1563053_at	LOC729083	1.016208828	0.650076312
215679_at	RP11-6O2.2	1.016205804	0.636127491
1559254_at	LINC00162	1.016170069	0.623433741
1553818_x_at	POM121L10P	1.016117569	0.739384231
230096_at	SAPCD1-AS1	1.016030681	0.785286334
239936_at	DLEU2	1.0160125	0.895093318
1561707_at	LINC00895	1.015985996	0.701331473
216469_at	LOC441666	1.015971509	0.657390821
242354_at	RP11-532F12.5	1.015923899	0.768672499
215738_at	LINC00563	1.015896593	0.732592194
1561650_s_at	LOC285692	1.015802722	0.675099767
231521_at	PMS2P4	1.015766714	0.795049036
207490_at	TUBA4B	1.01574123	0.821218365
213703_at	LINC00342	1.015701034	0.921344454
237224_at	LINC00853	1.015695008	0.802959747
230641_at	LOC100505938	1.015670405	0.884478857
1562617_at	LOC340074	1.015627324	0.691242969
1562932_at	RP11-318G8.4	1.01562214	0.678455949
232251_at	NUDT16P1	1.015622006	0.755584202
1562223_at	LOC642426	1.015526228	0.61290799
215876_at	RP11-217B7.2	1.01548935	0.745385652
239718_at	LOC654342	1.015433882	0.846760338
242812_at	HCG18	1.015424682	0.862591093
1564533_at	LOC643711	1.015332977	0.623590405
227724_at	NUTM2A-AS1	1.015317792	0.884186529
1557565_a_at	ALKBH3-AS1	1.015207931	0.737654863
1559352_a_at	MAFG-AS1	1.015192538	0.753704738
240184_at	SYNPR-AS1	1.015126513	0.72440965
214152_at	DYX1C1-CCPG1	1.015123396	0.916211131
1555221_at	RP11-6N17.1	1.015085175	0.619097359
244325_at	LINC00690	1.01495083	0.755614579
1552952_at	RBMY2FP	1.014941769	0.690521338
1561967_at	FLNB-AS1	1.014781753	0.65696414
1561529_at	RP11-352B15.2	1.014718507	0.748246939
237630_s_at	FAM86B3P	1.014638657	0.788038969
231458_at	LINC00658	1.014617979	0.784433837
238195_at	RP11-506E9.3	1.014602538	0.750525174
1563753_at	LOC149684	1.014552813	0.691221465
239982_at	RP4-612B15.3	1.014494951	0.839800245
221183_at	LOC100507388	1.014420812	0.767984621
215944_at	RP11-170N11.1	1.014324307	0.743810946
240893_at	RP11-502N13.2	1.014205211	0.720193674
208534_s_at	RASA4CP	1.014187484	0.930846332
228632_at	MEG9	1.01412926	0.885975293
224143_at	TTTY8B	1.01412458	0.776316531
1557618_at	LOC285768	1.014109567	0.736922368
223971_at	OR2A9P	1.013992196	0.819567536

243212_at	RP11-225H22.4	1.013967749	0.742110135
217194_at	RP4-765C7.1	1.013944679	0.594989394
1561353_at	RP11-624J12.1	1.013906884	0.731840467
240669_at	RP11-60A24.3	1.013843635	0.757250459
1562893_at	RP11-65D17.1	1.013809854	0.664982037
225786_at	HNRNPU-AS1	1.013773688	0.932232215
1561386_at	RP11-863K10.7	1.013649464	0.745911166
242666_at	RP3-400N23.6	1.013632128	0.825908381
1566082_at	RP11-452K12.7	1.013548832	0.784990834
1559646_a_at	LINC00184	1.013506757	0.798252454
1561331_at	ERVMER61-1	1.013466019	0.657547752
238874_at	LOC100506860	1.013458498	0.781372346
1557783_at	MAP3K14-AS1	1.01335403	0.809201802
220589_s_at	LOC100507424	1.013215639	0.843591087
1561375_at	RP11-806L2.2	1.013215314	0.734317149
224185_at	RP11-199F11.2	1.013212356	0.918879306
224423_x_at	PMCHL2	1.013143502	0.775170587
234234_at	RP5-1120P11.1	1.013099288	0.762524182
229604_at	CMAHP	1.013088048	0.889894596
1562742_at	RP4-601K24.1	1.013080644	0.807833498
1559066_at	RP11-4O1.2	1.013072802	0.767974415
207963_at	KIF25-AS1	1.013065646	0.850140522
1569270_at	LOC100134368	1.013052289	0.770883694
1562558_at	LOC440704	1.013004358	0.723286892
1557167_at	HCG11	1.012942444	0.931811932
1557606_at	RP11-588G21.2	1.012940955	0.678017007
224443_at	LINC00467	1.012934106	0.936166091
238247_at	RP11-761I4.3	1.012827383	0.734560925
235317_at	LOC284454	1.012719298	0.818816269
211942_x_at	RPL13AP5	1.01260554	0.900061233
1566935_at	TYRO3P	1.012532017	0.771081962
237635_at	LOC100128164	1.012506785	0.748750686
232686_at	SIGLEC17P	1.012444959	0.82657282
232965_at	LOC400684	1.012442918	0.794107226
220855_at	CLTC-IT1	1.012350643	0.92457283
1563324_at	LOC100129603	1.012290752	0.755032314
1561553_at	RP11-418J17.1	1.012252085	0.682415229
230432_at	LOC100422737	1.012241846	0.797371664
206097_at	SLC22A18AS	1.012132699	0.82135305
1563369_at	LINC00173	1.012126484	0.793470368
59375_at	MYO15B	1.011931345	0.888385364
222184_at	LINC00965	1.011894293	0.945255483
1561384_a_at	LOC284661	1.011870183	0.75852837
1561069_at	RP11-298H24.1	1.011838545	0.680993584
244553_at	HOTTIP	1.011833552	0.713190605
231421_at	LINC00710	1.01173026	0.746100412
1564017_at	COL18A1-AS1	1.011705525	0.730260553
1564777_at	HYALP1	1.011680734	0.730758404
1559777_at	LOC731424	1.011625817	0.813535434
1561287_at	LINC00561	1.011575953	0.756162598

207631_at	NBR2	1.011517727	0.871933108
233162_at	RP11-333A23.4	1.011493588	0.759362425
237473_at	PPIEL	1.011456742	0.765961049
220577_at	GVINP1	1.011396903	0.881121243
1570316_at	RP11-749H17.2	1.011361699	0.759631238
237651_x_at	LINC00518	1.01125758	0.744451178
220852_at	FOXN3-AS2	1.011215714	0.792455192
238216_at	LOC100506258	1.011205315	0.805190163
234166_at	RP13-487P22.1	1.011198229	0.794523246
242267_x_at	RP5-1154L15.2	1.011166746	0.798909512
1558804_at	RP11-467D6.1	1.011113267	0.776196265
210794_s_at	MEG3	1.011091156	0.956932722
1555890_at	OR2A9P	1.011073635	0.879603824
1562509_at	RP11-402L6.1	1.011065884	0.865851148
1554413_s_at	SNX29P2	1.011007059	0.859367033
1562352_at	RP11-373E16.3	1.010994453	0.73090631
1560978_at	RP11-534L20.5	1.010991216	0.78381698
240068_at	LINC00323	1.010981713	0.844148305
236176_at	RP4-794H19.1	1.010949494	0.852142705
1561084_at	RP13-539F13.3	1.010930662	0.81509677
235284_s_at	LOC388692	1.010658559	0.731469398
1560496_at	RP11-554A11.6	1.010652848	0.823944265
1564790_at	ST7-AS2	1.01061333	0.727737296
1562895_at	RP11-154D17.1	1.010601319	0.805240722
229156_s_at	PRKAG2-AS1	1.010589079	0.936185804
214570_x_at	POM121L1P	1.010527408	0.776925628
236881_at	RP1-135L22.1	1.010505766	0.830249458
1562313_at	BCORP1	1.010375815	0.760018698
244681_at	NUP210P1	1.010328372	0.79046849
1568869_at	RP11-102M11.2	1.010306126	0.776705152
236639_at	RP11-168O10.6	1.010267325	0.823688647
1559208_at	ST7-OT4	1.010259713	0.774443745
217393_x_at	UBE2NL	1.010234577	0.811316675
1561281_a_at	LEMD1-AS1	1.010210449	0.853517566
231330_at	LINC00445	1.010069748	0.825904981
1559813_at	RP11-454K7.3	1.010014329	0.836522958
1565556_at	LINC00628	1.010008831	0.829256381
1561868_at	RP4-584D14.7	1.010007309	0.800206657
1562190_at	RP11-360P21.2	1.009997531	0.712282632
229013_at	LOC145783	1.009966884	0.877525087
1564050_at	LOC100131347	1.00981159	0.879281693
1564909_at	RP11-13E5.2	1.0098079	0.691163909
1557591_at	LOC283038	1.009733914	0.786131597
1552972_at	LOC100507431	1.009689392	0.743842012
220810_at	CLCA3P	1.009617887	0.683708805
1570046_at	RP11-489G11.3	1.009592633	0.775783465
239685_at	LINC00900	1.009480025	0.845238776
1557871_at	LOC253573	1.00945565	0.792485841
217488_x_at	PMS2P3	1.009432054	0.844685293
224421_x_at	PMCHL1	1.009293058	0.828665347

1561311_at	RP11-174G17.2	1.009253384	0.863930609
208245_at	RAB9BP1	1.009044374	0.850437118
1556511_a_at	RP4-668G5.1	1.00898789	0.927602541
1556533_at	LINC00868	1.008977874	0.81522877
243412_at	RP11-1137G4.3	1.008918867	0.810501328
242835_s_at	LOC728730	1.008890742	0.906629709
230256_at	RUSC1-AS1	1.008772455	0.955756809
1553592_x_at	BCRP3	1.008744486	0.865583353
237115_at	RP11-135A1.2	1.008660547	0.901511084
1569765_at	RP11-492E3.2	1.008616908	0.85038898
233661_at	LINC00557	1.008520746	0.841953607
244589_at	RP11-298H24.1	1.008514192	0.853605014
1556734_at	LMO7-AS1	1.00840556	0.814135764
1558786_at	RP11-797A18.4	1.008402198	0.940479838
1559656_a_at	MAMDC2-AS1	1.008364003	0.886197191
231427_at	LOC284648	1.008255976	0.894766132
1570490_at	RP11-646E18.4	1.00823915	0.815736952
228440_at	RP11-164P12.4	1.008197561	0.845416628
231454_at	PLAC4	1.008128212	0.893339528
1569838_at	RP11-386I8.6	1.00809973	0.836267867
1561197_at	LOC442028	1.008090673	0.866349142
1556082_a_at	RP11-16P6.1	1.008065837	0.953617686
243687_at	LINC00881	1.008062229	0.878784932
1562733_at	LINC00092	1.00805247	0.90435927
236669_at	SDCBP2-AS1	1.008031869	0.950548905
233963_at	H2BFXP	1.008002174	0.947019689
243794_at	RP11-474D1.2	1.007978005	0.86165762
1569987_at	DLEU7-AS1	1.00797229	0.884996576
240573_at	LOC374443	1.007922264	0.912548285
1563371_at	RP11-10A14.7	1.007770872	0.796766228
217323_at	HLA-DRB6	1.007763564	0.831117664
1561301_at	MAGI1-IT1	1.007615346	0.871901472
234712_at	LINC00470	1.00759836	0.890641469
1559540_at	LOC100506526	1.007585948	0.876755239
244600_at	LOC100996583	1.007479523	0.842187187
1563235_at	RP11-400N13.1	1.007473432	0.880378312
240441_at	FAM99B	1.007464873	0.861192347
220506_at	GUCY1B2	1.007443601	0.917879647
239760_at	RP11-257O5.2	1.0073966	0.949364866
1562046_at	RP5-952N6.1	1.007336565	0.850141971
231356_at	RP11-862L9.3	1.007211062	0.806737558
1561328_at	RP11-501J20.2	1.007154092	0.831936244
244270_at	RP11-973N13.4	1.007153656	0.924757798
1553898_a_at	DKFZp434L192	1.007097259	0.825782743
1561682_at	TTLL7-IT1	1.007080149	0.896283688
1559017_at	LOC100507351	1.007055281	0.889948366
224238_at	RP11-264L1.2	1.007008449	0.878708872
235941_s_at	FAM224A	1.00697188	0.865340194
236871_s_at	IQCF4	1.006965621	0.873571053
1564485_at	LINC00887	1.006951207	0.849053809

1557338_x_at	RP11-81N13.1	1.006904835	0.926394344
1569294_at	RP5-915N17.11	1.006852055	0.885274144
231051_at	LINC00948	1.006826245	0.884110491
1569486_at	RP11-760D2.5	1.0068007	0.864812949
237845_at	RP11-575L7.4	1.006733457	0.878296393
231557_at	RP11-597D13.8	1.006692196	0.933122107
1563143_at	LOC100507065	1.006668077	0.887391451
232259_s_at	ZBTB11-AS1	1.006603858	0.929225353
228147_at	SEPT7P2	1.006597601	0.967818657
243402_at	LOC643406	1.006485442	0.884543103
1569953_at	RP11-179B15.6	1.006373256	0.903322025
1559470_at	D21S2088E	1.006328996	0.867930318
215185_at	LINC00963	1.006220202	0.947302481
226924_at	LINC00909	1.006183017	0.953008174
1555478_at	C17orf82	1.006160957	0.87043324
1556663_s_at	LOC100506142	1.006139353	0.892127553
1561584_at	RP11-96B5.4	1.006110017	0.859830277
1565132_at	RBMY3AP	1.006096619	0.887099333
233529_at	RP11-472K17.1	1.006085704	0.892001702
220159_at	ABCA11P	1.005992871	0.956085698
229388_at	RP11-152N13.5	1.005955032	0.955313032
1561345_at	RP1-140J1.1	1.005941371	0.875936549
220701_at	LINC00216	1.005740131	0.912768867
1557679_at	ERICH1-AS1	1.00573011	0.896024784
1556827_at	LPP-AS2	1.00572329	0.9599712
207762_at	LPAL2	1.00572132	0.901860236
239241_at	RP11-315O6.1	1.005656157	0.931850175
244864_at	RP11-462L8.1	1.005625642	0.912669399
1559340_at	TTLL11-IT1	1.005534538	0.88525813
1553399_a_at	MGC57346	1.005520845	0.912429951
1562770_at	RP11-309I15.1	1.005481792	0.900283305
1560638_a_at	RP11-30L15.6	1.005455998	0.908055862
1553882_at	LINC00504	1.005439294	0.879410791
1559863_a_at	AFG3L1P	1.005423387	0.90278621
230596_at	RP11-71H17.7	1.005296152	0.981898656
1566526_at	LINC00927	1.005264512	0.899635889
240624_x_at	RP1-170O19.17	1.005255935	0.92631231
1558423_at	LINC00265	1.005140516	0.928034003
243721_at	C18orf61	1.005005462	0.903221514
1553335_x_at	LOC285696	1.004940308	0.943157683
1561598_at	RP11-333O1.1	1.004922061	0.92959051
1562915_at	RP11-278I4.2	1.00487088	0.88986116
1562023_at	RP11-94M14.2	1.004850307	0.904318227
229040_at	ITGB2-AS1	1.004840843	0.925445039
233311_at	LOC145845	1.004756244	0.899058313
234117_at	NPSR1-AS1	1.004749942	0.902946232
1552258_at	LINC00152	1.004715518	0.950169144
1569218_at	RP11-594N15.2	1.004661604	0.902133901
1559285_at	RP11-1017G21.4	1.004458275	0.90827958
217123_x_at	PMCHL1	1.004436593	0.910222134

1570152_at	RP11-360K13.1	1.004288071	0.915522382
230487_at	C6orf99	1.004278177	0.919990983
1555265_at	ABCC13	1.004262742	0.902128349
243354_at	HYDIN2	1.004245775	0.962093187
1559976_at	LOC100506314	1.004211179	0.931353838
1565320_at	RBMY3AP	1.004185341	0.948550125
225458_at	PP7080	1.00417004	0.963260223
1559213_at	RP11-51M18.1	1.004164744	0.877398503
231455_at	LINC00487	1.004159316	0.95398569
1562745_at	RP11-804H8.6	1.004091625	0.956465714
235934_at	NAPA-AS1	1.004051172	0.933830425
1557741_at	LOC100128176	1.004046293	0.897103566
1564539_at	LOC647323	1.00403158	0.921049047
1568662_at	PWRN2	1.003993322	0.89213192
1558411_at	EGFEM1P	1.003958849	0.986343624
244886_at	LOC389641	1.003896769	0.908785773
1557212_at	EIF1B-AS1	1.003872396	0.943413992
238094_at	RP5-968P14.2	1.003870183	0.963475065
234437_at	RP11-111K18.2	1.003765323	0.957307885
244209_at	LBX1-AS1	1.003667751	0.943354826
1569291_at	CROCCP2	1.003667373	0.930808265
1563483_at	LINC00869	1.003637088	0.972906762
1557865_at	LOC100505658	1.003416786	0.913594005
242954_at	LOC101059948	1.003354796	0.941799706
1562606_a_at	LOC440028	1.003350733	0.938641961
1563728_at	LINC00032	1.003318606	0.953740095
1570372_at	RP11-781A6.1	1.003233058	0.933501403
1556555_at	LOC100129461	1.003117173	0.969675526
1558109_x_at	LOC283788	1.003089305	0.970896186
216673_at	TTTY1B	1.003027222	0.952504388
1557506_a_at	RP4-593C16.3	1.002916782	0.980907581
1552877_s_at	LINC00334	1.00291393	0.930748173
1552873_s_at	ASMTL-AS1	1.002876414	0.942685071
224644_at	FGD5-AS1	1.002818009	0.974140431
1561141_at	RP11-417E7.2	1.002765122	0.952935628
1570474_s_at	ANKRD30BP3	1.002739416	0.941415452
1567687_at	CECR9	1.002721285	0.939371392
1561277_at	LOC339298	1.002708669	0.954973723
1562173_a_at	LINC00555	1.002656581	0.951280456
244619_at	LOC646626	1.002654201	0.960273807
1564787_at	LOC643923	1.002638168	0.969748933
224058_s_at	HSD17B7P2	1.002616544	0.972092322
1558765_a_at	RP3-496C20.1	1.002574564	0.950565099
1561626_at	RP5-1033E15.3	1.002528998	0.949476893
1556901_s_at	APCDD1L-AS1	1.002504016	0.942674882
207412_x_at	CELP	1.002491969	0.96289848
234147_at	LOC286059	1.002470141	0.94861107
208755_x_at	H3F3AP4	1.002462947	0.980023091
238290_at	RP11-415J8.5	1.002423617	0.957878953
234940_s_at	RP11-550I24.2	1.002361575	0.952926769

1554764_a_at	LINC00301	1.002340808	0.938242417
1561558_at	PRKG1-AS1	1.002339712	0.946988346
1557021_s_at	LOC100507250	1.002319096	0.985467628
1563878_a_at	LOC338963	1.002305503	0.950269695
226295_at	LOC100507424	1.002249548	0.974807021
1552467_at	DSCR10	1.002136532	0.956262386
1557434_at	LOC100506497	1.002071394	0.975913013
1566860_at	GATM-AS1	1.002045951	0.982856707
240719_at	ISM1-AS1	1.00203425	0.962691956
1558256_at	LINC00662	1.001974157	0.990049952
239880_at	UBE2E2-AS1	1.001927335	0.971924675
240006_at	SCARNA17	1.001892555	0.984871339
233617_at	TSPY26P	1.001870363	0.974458813
236658_at	RPS6KA2-AS1	1.001847454	0.96878533
1561459_at	RP11-379J5.5	1.001720181	0.975567311
231237_x_at	RP11-219B4.3	1.001659032	0.976632609
1562934_at	RP11-756H20.1	1.001557704	0.962747809
237253_at	IGSF11-AS1	1.001500507	0.963618189
1559929_at	RP1-30G7.2	1.001381145	0.97202194
1559792_at	RP11-683O4.1	1.001371695	0.971102151
1557427_at	RP11-799D4.4	1.001237015	0.970322612
1558202_at	LOC145783	1.00111114	0.980475125
1559800_a_at	RP11-709D24.6	1.001094886	0.979683188
1564131_a_at	BSN-AS2	1.001083478	0.9881188
1563287_at	RP11-168E17.1	1.001060908	0.977601628
215827_x_at	CROCCP3	1.0010393	0.980083001
244278_at	RP11-24D15.1	1.001038433	0.982669148
1570269_at	RP11-764E7.1	1.001031739	0.975293188
222225_at	RPL23AP53	1.00094271	0.983916396
210265_x_at	POU5F1P3	1.000874935	0.988257543
1561440_at	RP11-330M19.1	1.000843565	0.985342689
1561215_at	RP11-366L5.1	1.000776268	0.984069139
1557802_at	RP4-782L23.1	1.000756042	0.98575899
1556769_a_at	RP11-874J12.4	1.000729596	0.985639742
220388_at	FER1L4	1.000718701	0.986966008
1555912_at	ST7-AS1	1.000666265	0.996150207
1563190_at	RP11-58G13.1	1.000638408	0.989170443
244283_x_at	RP11-58O3.2	1.000580566	0.984936316
239386_at	HAVCR1P1	1.000558478	0.991536252
1559880_at	LZTS1-AS1	1.000491276	0.992471147
1561044_at	RP11-540K16.1	1.000281254	0.996354646
1560996_at	RP3-507I15.2	1.000241143	0.994535168
1565581_at	RP11-648F7.1	1.000218723	0.996085622
243058_at	RP1-170O19.14	1.000216538	0.996759846
237205_at	LINC00238	1.000197133	0.994971378
1562474_at	RP11-418J17.1	1.0001636	0.998588692
1553080_at	CSN1S2AP	1.000098741	0.99782322
1570447_at	LINC00452	-1.000011603	0.999729379
1552979_at	LINC00471	-1.000095501	0.998580393
1570285_at	RP11-443B7.1	-1.000147248	0.996596679

230642_at	RP11-297L17.2	-1.000266577	0.995548363
1561508_at	RP11-552F3.10	-1.0002779	0.994601102
1560791_at	RP11-630D6.5	-1.000277957	0.994046985
1563062_at	LINC00460	-1.000309497	0.995486661
1565685_at	LOC400940	-1.000352795	0.992789779
1559861_at	RP11-342C23.4	-1.000439755	0.993610243
1564060_at	LINC00934	-1.000509612	0.991267897
226663_at	ANKRD10-IT1	-1.000628588	0.996743687
1561813_at	RP11-16K12.2	-1.000723602	0.980620424
1564819_at	RP11-116D17.1	-1.000729841	0.985222615
243555_at	RP11-752L20.3	-1.000734045	0.997585546
1562924_at	LOC340357	-1.000739549	0.988449796
243017_at	USP27X-AS1	-1.000772536	0.989553005
1560823_at	LOC340017	-1.00089422	0.978142651
208340_at	LOC643733	-1.00099403	0.98071841
1558990_at	RP11-723J4.3	-1.001008001	0.983171448
239968_at	LINC00313	-1.001019276	0.984240782
1561228_at	LOC100506122	-1.001032795	0.983036986
1554591_at	PCAT4	-1.001052574	0.983306833
233432_at	LINC00598	-1.001142725	0.983612133
1568739_at	LOC285692	-1.001169936	0.975709824
222955_s_at	FAM45B	-1.001186852	0.992535883
1558128_at	LOC730202	-1.001200015	0.984519366
1561607_at	RP11-283I3.2	-1.001275261	0.981604899
1568848_at	RP4-813D12.3	-1.00130655	0.988968643
1557702_at	RP5-858B6.3	-1.001353626	0.978370085
1553591_at	BCRP3	-1.001362208	0.977295108
1561525_at	RP11-331K15.1	-1.001387331	0.958872041
239220_at	RP11-736K20.5	-1.001420415	0.982222968
224020_at	LINC00626	-1.001463761	0.973939704
1570006_at	LOC400958	-1.001486416	0.975364219
1558571_at	RP4-569M23.2	-1.001488831	0.976984824
1560514_at	LINC00636	-1.001501696	0.97009797
1568650_a_at	RP11-540O11.1	-1.001550649	0.967880843
232836_at	LINC00687	-1.001602302	0.971288716
1561351_at	RP5-1136A10.1	-1.001622979	0.968243877
238742_x_at	RP11-532F12.5	-1.001776598	0.969450955
231284_at	LOC100506406	-1.001842566	0.962773006
1559344_at	RP11-778J15.1	-1.001855436	0.959290748
240487_at	RP11-619L12.4	-1.001891555	0.955771633
235987_at	PRKXP1	-1.001966872	0.989678061
1561445_at	RP11-461L18.1	-1.00197025	0.949458402
226235_at	LINC00667	-1.002026436	0.984584003
233334_x_at	SLX1B-SULT1A4	-1.002057068	0.96919028
239541_at	LOC100505817	-1.00206592	0.949481693
226407_at	TPT1-AS1	-1.002078623	0.986391421
1562973_at	RP11-707P20.1	-1.002196162	0.955364337
1556467_at	RP11-553L6.2	-1.002216908	0.944888964
1557025_a_at	RP11-744D14.2	-1.00221906	0.979789903
1554976_a_at	LINC00051	-1.002252417	0.944841574

1555979_at	RP11-54O7.3	-1.002260119	0.972492561
1569782_at	RP11-343J18.1	-1.002286473	0.968247621
223443_s_at	AMZ2P1	-1.002488684	0.977870511
231190_at	POM121L10P	-1.002495481	0.961079609
215973_at	HCG4B	-1.002578908	0.964044022
1553880_at	C10orf91	-1.00261239	0.955788526
205703_at	RP11-338K17.10	-1.002612635	0.950598093
1569259_at	RP4-737A23.2	-1.002625194	0.949751501
214896_at	RP11-2E17.1	-1.002627309	0.987688687
217406_at	RP1-149A16.17	-1.00269356	0.960981927
1560939_at	PCOLCE-AS1	-1.002819558	0.942628043
227578_at	TMPO-AS1	-1.00282088	0.976993677
1556740_at	EGFLAM-AS2	-1.00284502	0.947432085
230012_at	LINC00324	-1.0028593	0.957659445
1562854_at	RP1-206D15.3	-1.00290269	0.922609816
1556784_at	RP1-178F10.1	-1.002956642	0.963306517
240169_at	RP4-545K15.5	-1.00296639	0.954205381
1561035_at	RP11-526I2.1	-1.002971075	0.961761112
1557465_at	LINC00282	-1.003040107	0.982732532
1553900_s_at	POM121L1P	-1.003072754	0.940116325
1562640_at	HOXA-AS2	-1.003151297	0.95183812
1562805_at	TLR8-AS1	-1.003214257	0.945114852
1562990_at	LINC00564	-1.003223554	0.927483852
1562750_at	RP11-546O6.4	-1.003317321	0.940188175
1562776_at	LOC339807	-1.003336446	0.94697352
240944_at	RP11-771K4.1	-1.003465625	0.933219048
236668_at	RP5-882C2.2	-1.003475585	0.968885034
1566678_at	RP11-212I21.3	-1.003512481	0.929903162
1560163_at	RP11-75C10.6	-1.003514655	0.95673624
1569341_at	RP3-337H4.8	-1.003550531	0.948043233
1568686_at	ATP8B5P	-1.003577448	0.913058035
1563072_at	RP11-171N4.1	-1.003821873	0.888381563
1564315_at	C8orf49	-1.003865513	0.892855876
244798_at	RP11-664D1.1	-1.0038782	0.91651796
1563742_at	LINC00842	-1.003906034	0.922918977
1560863_a_at	RP11-91I8.1	-1.003919535	0.938633421
239974_at	LOC728095	-1.003921313	0.94474781
1562549_at	RP11-290F20.2	-1.003927912	0.92854322
1556406_at	LINC00879	-1.004189597	0.930678882
1561290_at	LOC339622	-1.00419355	0.893743047
207701_at	C22orf24	-1.004194799	0.923028022
1564323_at	RP11-747D18.1	-1.004270252	0.915040781
1556030_at	RP3-412A9.10	-1.004284741	0.909441988
1553400_a_at	MGC57346	-1.004302769	0.976795615
230355_at	SEPT7P2	-1.004331806	0.97401986
230545_at	PARD3-AS1	-1.004338654	0.971562456
1560958_s_at	RP11-554D15.1	-1.004467791	0.93135009
1560352_at	ENTPD1-AS1	-1.00447254	0.958100936
208529_at	BTF3P11	-1.004484952	0.90445198
1563245_at	CLYBL-AS2	-1.004510513	0.907068685

1567023_at	OR5AK4P	-1.004583038	0.923486269
1569887_a_at	FAM183CP	-1.004599538	0.896675151
1556388_a_at	LOC100507389	-1.004625726	0.900497227
233928_at	ADAMTS9-AS1	-1.004650553	0.916784159
1569430_at	RP11-56D16.2	-1.004718319	0.9178877
1557891_s_at	STXBP5-AS1	-1.004835071	0.938768993
225199_at	RP11-473I1.10	-1.0048596	0.955677946
1562997_a_at	RP11-90K6.1	-1.004924219	0.91084246
1562631_at	TEX26-AS1	-1.00505523	0.899836284
1562100_at	RP11-461F11.2	-1.005056877	0.902924622
1561207_at	RP11-53B2.2	-1.005149093	0.908568509
237540_at	LINC00644	-1.005160168	0.886350127
216319_at	RP11-69I8.2	-1.005185008	0.944279626
240566_at	RP11-266A24.1	-1.005216646	0.932525508
1569784_at	RP11-756P10.2	-1.005244536	0.878816499
234686_at	SUGT1P1	-1.005270079	0.906283129
1557746_at	RP11-254A24.2	-1.005291078	0.918801719
227710_s_at	TPT1-AS1	-1.005338152	0.963549824
1562215_at	RP11-316N24.2	-1.005341901	0.909941292
1552466_x_at	LINC00161	-1.005378543	0.88279139
1557646_at	VWA8-AS1	-1.005391704	0.864114564
1561540_at	LINC00343	-1.005457494	0.899634801
239395_at	ARRDC3-AS1	-1.005514992	0.88611405
1557450_s_at	WHAMMP2	-1.005530561	0.922136696
1564280_x_at	LOC100133920	-1.005695652	0.865760611
238380_s_at	LINC00226	-1.005729011	0.896112225
232107_at	RP11-122G18.5	-1.005731247	0.945203797
1559443_s_at	IL21R-AS1	-1.005743333	0.905696395
1562222_at	RP11-231E4.2	-1.005747481	0.909343711
1559297_at	SNX29P2	-1.005809448	0.891806531
217682_at	RP11-473I1.9	-1.005877046	0.939610166
1562653_at	RP4-697P8.3	-1.005932636	0.885566171
237063_at	RP11-44F14.8	-1.005938926	0.856610487
232572_at	PCA3	-1.00603867	0.87922694
1562029_at	RP11-855O10.2	-1.006115759	0.848296862
239617_at	GHRLOS	-1.006143389	0.892152061
237552_at	LOC100505817	-1.006187073	0.856561076
237699_at	LINC00427	-1.00623489	0.877860955
1561693_at	LOC400794	-1.006354604	0.88992235
240475_at	EGOT	-1.006411794	0.891695402
237738_at	RP11-26M5.2	-1.006430162	0.858632966
1562367_at	C15orf54	-1.006495603	0.853207005
223791_at	FAM27B	-1.006517917	0.944819303
240490_at	RP11-184I16.4	-1.006521064	0.87480674
233755_at	RP11-758M4.4	-1.006548971	0.851407779
239136_at	UNC5B-AS1	-1.006563986	0.89066719
1561516_at	RP11-12L8.1	-1.006601981	0.877833347
227519_at	PLAC4	-1.006626857	0.854657793
1554142_at	SUGT1P3	-1.006714552	0.862383917
1557890_at	STXBP5-AS1	-1.006715102	0.855543328

1563894_at	LOC441178	-1.00675919	0.885824477
1568987_at	MGC57346	-1.006849438	0.911890116
1557724_a_at	LOC285847	-1.006858219	0.870615275
236653_at	LINC00662	-1.006947546	0.938642542
236399_at	RP11-443B7.1	-1.007018104	0.845996699
228078_at	RP11-258F1.1	-1.007043464	0.892344251
212707_s_at	RASA4CP	-1.007048794	0.94660009
1562885_at	RP11-862G15.2	-1.007093598	0.857667335
1562455_at	RP11-379K22.2	-1.00712099	0.839891673
1562765_at	WWTR1-AS1	-1.007132315	0.863276117
234183_at	RP11-673E11.2	-1.007233069	0.840400037
242284_at	LINC00466	-1.007237622	0.845645177
216098_s_at	HTR7P1	-1.007243541	0.891133901
231620_at	RP11-561E1.1	-1.007313247	0.829048948
239125_at	SLC25A5-AS1	-1.007324574	0.879897483
237490_at	RP11-299H22.6	-1.007351877	0.862601064
1562829_at	LOC339568	-1.007362901	0.896277682
1558859_at	LOC401320	-1.007372331	0.95155559
179_at	DTX2P1-UPK3BP1-PMS2P11	-1.007395101	0.865944122
207871_s_at	ST7-OT3	-1.00740066	0.937449314
1563346_at	C21orf91-OT1	-1.00742011	0.858212022
1562724_at	LOC286114	-1.007432216	0.850322492
1563385_at	LINC00307	-1.007437548	0.845826652
213801_x_at	RPSAP9	-1.0075304	0.946069758
1562396_at	RP11-248B24.1	-1.007532146	0.813955564
1568745_at	LOC646268	-1.007537137	0.860260404
233398_at	RP11-319E16.2	-1.007547689	0.842747446
1564028_s_at	LOC154761	-1.007613202	0.932565863
214304_x_at	RP11-654A16.3	-1.007626411	0.881354855
222349_x_at	RNF126P1	-1.007665964	0.889970352
1569923_s_at	LINC00491	-1.007739457	0.854343097
1560646_at	METTL21EP	-1.007744203	0.826302489
230648_at	LINC00926	-1.007768063	0.848746876
1556676_a_at	RP11-425D10.10	-1.007819694	0.864957826
1553320_s_at	CDC14C	-1.007878638	0.797199502
1556048_at	LOC100507564	-1.007933161	0.914858857
243785_at	LOC100272217	-1.007967847	0.946110994
1560560_at	RP11-365O16.3	-1.008051121	0.816000947
234327_at	RP11-1007O24.3	-1.008055071	0.8656357
242292_at	FAM226B	-1.008065305	0.962502139
227901_at	LOC648987	-1.008068476	0.911393264
1569264_at	LOC400655	-1.008104971	0.853619515
1562253_at	SLC7A11-AS1	-1.008132941	0.870588804
1557139_at	DDX11-AS1	-1.008144087	0.869542189
1561587_at	LINC00907	-1.008196422	0.821999297
1568919_at	RP11-341A22.2	-1.008262148	0.759658568
1556248_at	EIF1B-AS1	-1.008294851	0.890667179
1561506_at	RP11-231K24.2	-1.008307614	0.821636539
1553464_at	FLJ40288	-1.008355418	0.838427755
1562923_at	RP11-362A1.1	-1.008420015	0.827656134

240938_at	RP11-18F14.1	-1.008448791	0.792499054
1561323_at	LOC339975	-1.008470122	0.796127577
1564051_at	RP11-410D17.2	-1.008479278	0.783648932
1560246_at	RP11-260E18.1	-1.008506746	0.79581617
224142_s_at	TTTY8B	-1.008583358	0.885594451
241146_at	MAGI2-AS2	-1.008606934	0.81783138
237537_at	RP4-598G3.1	-1.008681085	0.820818976
1564209_at	LINC00700	-1.008745645	0.838924006
240693_at	RP11-63A1.2	-1.008808557	0.875299522
232047_at	LINC00969	-1.008865354	0.889360441
1557850_at	INHBA-AS1	-1.008915769	0.847147716
1570080_at	RP5-1073O3.7	-1.00892949	0.864740024
1570136_at	RP11-483C6.1	-1.008935077	0.947498048
1569963_at	RP11-426A6.5	-1.008938839	0.811227834
1558168_at	H1FX-AS1	-1.008940018	0.795763968
1556400_at	RP11-547D24.1	-1.008943914	0.820654101
1561528_at	RP11-575F12.1	-1.009016663	0.820326814
1556362_at	RP4-809F18.1	-1.00904291	0.803090558
241124_at	RP11-44F21.2	-1.009053268	0.814369444
1569095_at	LOC731424	-1.009056937	0.843698126
1564367_at	CXXC1P1	-1.009100671	0.80021118
224292_at	TTTY13	-1.009158073	0.871793532
229223_at	RP11-96D1.11	-1.009170752	0.855968184
1563092_at	RP11-791M20.1	-1.009228443	0.8830619
1558412_at	LOC113230	-1.009230382	0.911862195
243351_at	FZD10-AS1	-1.009234298	0.878844659
238685_at	STXBP5-AS1	-1.009243879	0.837599628
1564282_a_at	LINC00491	-1.009274713	0.825033481
238691_at	SNHG10	-1.009284408	0.8799595
1553924_at	FMO9P	-1.009383724	0.830469439
233830_at	LOC90246	-1.009406025	0.87086097
1557008_at	LOC340107	-1.009427928	0.854355017
1557848_at	RP11-626I20.3	-1.009431255	0.803960852
224174_at	TTTY11	-1.009453368	0.838553864
1561545_at	EPN2-AS1	-1.00948881	0.847039965
1566689_at	RP11-157B13.7	-1.009514517	0.818400483
215229_at	LOC100129973	-1.009528132	0.924668214
1555580_at	RP11-89M16.1	-1.009550931	0.814983733
1561354_at	RP11-151E14.1	-1.009649417	0.840186016
1563142_at	RP11-521D12.1	-1.009762272	0.850905658
237044_s_at	ZNF503-AS2	-1.009820929	0.90085541
215504_x_at	ANKRD10-IT1	-1.009835081	0.87465661
1560812_at	RP11-899L11.1	-1.009843716	0.78209932
240306_at	LINC00312	-1.009896082	0.849493125
1556492_a_at	RP11-687F6.1	-1.009929352	0.872320333
1564552_at	RP11-678G15.1	-1.009940062	0.857782818
1560581_at	RP11-804F13.1	-1.009988856	0.706212887
1561062_a_at	RP11-259A24.1	-1.010036911	0.814286675
1561383_at	LOC284661	-1.010037044	0.764572262
224110_at	PRO1804	-1.010106943	0.792878717

240917_at	RP11-506B6.6	-1.010119101	0.818857706
1562909_at	LINC00862	-1.010132172	0.774532083
236631_at	LINC00319	-1.01023901	0.811355579
1560260_at	LOC285593	-1.010287857	0.82968034
242507_at	UBXN7-AS1	-1.010443358	0.948597951
1558601_at	TUSC7	-1.010454891	0.842835804
1570484_at	RP11-669I1.1	-1.010462067	0.817612755
236986_at	LINC00582	-1.010520156	0.820607112
216544_at	RBMY2FP	-1.010571453	0.700428963
237617_at	LOC100506675	-1.010600671	0.737802925
1562983_at	LOC441025	-1.010601223	0.794026594
220465_at	CEBPA-AS1	-1.010624256	0.827899794
230488_s_at	DBH-AS1	-1.01063977	0.86942585
236369_at	TSPY26P	-1.01064135	0.898346729
232260_at	ZBTB11-AS1	-1.010671732	0.755405398
216786_at	FAM224A	-1.010738305	0.809269138
1555264_a_at	LINC00598	-1.010857584	0.775615042
1562740_at	DNAJB8-AS1	-1.01087036	0.830434283
1569287_at	LINC00458	-1.010878471	0.778728751
1563255_at	FAM170B-AS1	-1.010879709	0.822549673
1557672_s_at	RSU1P2	-1.01090648	0.80925249
241525_at	LOC200772	-1.01091522	0.819983989
1569600_at	DLEU2	-1.01106657	0.931420332
229990_at	TSC22D1-AS1	-1.011116466	0.801547616
1552961_at	RP11-85I17.2	-1.011226609	0.759358081
237536_at	MAPT-AS1	-1.011338345	0.786108472
1555224_at	CCDC148-AS1	-1.011367166	0.790502053
1569544_at	RP11-460N16.1	-1.011367281	0.699045586
1557567_a_at	LOC100507634	-1.011370652	0.878536382
243425_at	RP11-568J23.6	-1.011387531	0.800767625
1566658_at	RP11-151A6.6	-1.011403266	0.796723811
239120_at	TMEM51-AS1	-1.011404219	0.895083387
1558899_s_at	RGMB-AS1	-1.0114098	0.85318168
242874_at	RP11-747H7.3	-1.011432814	0.954079766
237522_at	FAS-AS1	-1.011509706	0.78787365
220222_at	RBM12B-AS1	-1.011514812	0.818447911
1553299_at	DUSP5P1	-1.011538628	0.94301626
233151_s_at	TTTY7	-1.011542	0.833944617
1561432_at	RP11-539G18.2	-1.011558633	0.884613076
236385_at	RP11-382B18.4	-1.011560215	0.921158268
1553747_at	MGC16025	-1.011567055	0.83330656
1556529_a_at	LINC00692	-1.011570732	0.750901608
1565775_at	RP11-856M7.1	-1.011681572	0.778011036
1558515_at	FTX	-1.011691272	0.94162901
244135_at	RP11-177H2.2	-1.011696112	0.805800976
1561211_at	RP1-18D14.7	-1.011728926	0.806706201
1563825_at	RP11-405M12.2	-1.011765515	0.828203397
1569954_at	RP11-354E11.2	-1.011879464	0.699563815
232900_at	HERC2P7	-1.01191198	0.812070818
235886_at	LOC100505666	-1.01192414	0.820354737

223630_at	C7orf13	-1.011935576	0.930125192
244446_at	RP11-301H24.3	-1.011939846	0.820177282
240407_at	LOC100126784	-1.011974003	0.953203529
241846_at	HCG18	-1.012017787	0.837185828
1562866_at	RP11-184E9.2	-1.012131726	0.669761389
238387_s_at	LINC00226	-1.01217485	0.788827268
228056_s_at	NAPSB	-1.012311459	0.959703283
217308_at	OR1F2P	-1.012315902	0.770212892
240083_at	MEG8	-1.012366889	0.84686544
211458_s_at	GABARAPL3	-1.012417536	0.936387043
211931_s_at	HNRNPA3P1	-1.012460787	0.895699456
238997_at	PSORS1C3	-1.012475757	0.765430719
1557026_at	RP4-781K5.4	-1.012485979	0.791625767
1562873_at	PWRN1	-1.012489068	0.764957645
237260_at	LOC100505978	-1.012528851	0.755606032
1562589_at	RP3-437I16.1	-1.012538024	0.70314488
1555363_s_at	LINC00663	-1.012573803	0.918422393
243248_at	RP11-470M17.2	-1.012575062	0.735662927
1553298_at	C17orf77	-1.012584446	0.718212085
219865_at	LINC00339	-1.012600314	0.918407599
222327_x_at	OR7E156P	-1.012697228	0.852127522
243408_at	RP11-774O3.1	-1.012740948	0.831443668
1561256_at	RP5-991O23.1	-1.012843503	0.726642381
1555485_s_at	LOC100507387	-1.012846365	0.916337805
1562942_at	RP11-982M15.6	-1.012889042	0.776478679
1561318_at	RP11-84D1.2	-1.012903198	0.783642295
1563610_at	LOC157273	-1.012916534	0.688629132
1565152_at	RP11-118G23.2	-1.012941566	0.741775779
1556459_at	ARHGAP22-IT1	-1.012961676	0.730307669
239513_at	ADORA2A-AS1	-1.012973734	0.772532034
233190_at	RP11-266E14.1	-1.012984648	0.7720713
1569023_a_at	LINC00458	-1.013071733	0.7058734
1560870_a_at	RP11-1012E15.1	-1.013088926	0.740990754
237163_x_at	LOC390705	-1.013110394	0.750520096
1563876_at	C1orf220	-1.013131728	0.841654822
1563681_at	RP11-108B14.5	-1.01320081	0.72343835
229820_at	LINC00969	-1.013204474	0.876130729
221415_s_at	GJA9-MYCBP	-1.013270794	0.718472398
237242_at	LINC00661	-1.013295758	0.769291062
238031_at	RP11-347C12.10	-1.013302209	0.789545657
1563099_at	LOC100507053	-1.013377488	0.684602497
239777_at	C14orf182	-1.013415053	0.754333894
1557660_s_at	FAM170B-AS1	-1.013441853	0.748396884
229557_at	MEG3	-1.013458899	0.963702334
240830_at	SCARNA17	-1.013505287	0.913915697
1561371_at	FLJ39080	-1.013528596	0.772999426
1561916_at	RP11-340I6.8	-1.013560172	0.807799748
1564253_at	LOC285766	-1.01359465	0.724474606
216667_at	ECRP	-1.013612234	0.847952144
230854_at	BCAR4	-1.013635144	0.758334797

1557825_at	RP11-1E4.1	-1.013656414	0.851874583
233048_at	RP11-38L15.2	-1.013663058	0.822280794
1557693_at	SMC5-AS1	-1.013784763	0.726865185
1557044_at	LINC00665	-1.013791943	0.916613426
1570405_at	RP11-34F13.2	-1.0137937	0.765197471
1561224_at	RP11-189E14.4	-1.013861586	0.760797719
231518_at	LINC00922	-1.013884903	0.818595979
1560810_at	RP11-669N7.3	-1.014003254	0.724494452
1564337_at	RP11-744N12.3	-1.014009882	0.750555105
1558611_at	MGC57346	-1.014026422	0.75808392
1564291_at	RP11-109D24.1	-1.014092471	0.757259611
1561519_at	RP11-508O18.1	-1.014136901	0.752250236
243776_at	RP11-63A11.1	-1.01414777	0.787299873
1563310_a_at	RP11-7F17.4	-1.014171781	0.644324874
1556496_a_at	RP11-521I2.3	-1.014192336	0.803947074
226708_at	RP11-661A12.9	-1.014241183	0.782446431
1556829_at	TIPARP-AS1	-1.01424487	0.77762049
1562078_at	RP11-24P4.1	-1.014274653	0.770416563
1564072_at	MYH16	-1.014324211	0.694596382
231229_at	HILS1	-1.014423487	0.830180799
235053_at	RP11-257O5.2	-1.014430041	0.907942545
1570020_at	AACSP1	-1.014492925	0.636307645
222278_at	RP1-302G2.5	-1.014510486	0.780949776
1562659_at	LOC400548	-1.014538691	0.680972155
210444_at	NPY6R	-1.0145458	0.801306516
237519_at	RP11-445L13__B.3	-1.01454838	0.749751542
237392_at	FGF14-IT1	-1.014552431	0.822118737
241171_at	RP11-669M16.1	-1.014568388	0.791597552
237312_at	LOC100506272	-1.014577388	0.748793717
1562870_at	RP11-1007G5.2	-1.014594033	0.6793991
226903_s_at	RP11-812E19.3	-1.014666675	0.790838221
234180_at	RP11-674P19.2	-1.014683589	0.806735339
1569831_at	RP11-586K2.1	-1.014688787	0.752690542
1570541_s_at	GBP1P1	-1.014715506	0.798354741
219961_s_at	PLK1S1	-1.014781944	0.917975703
220211_at	FLJ13224	-1.014890326	0.728201672
243485_at	RP5-944M2.2	-1.014918255	0.706042441
1553258_at	FLJ30679	-1.015007227	0.785583234
1567022_at	OR5AK4P	-1.015040723	0.689025162
1570131_at	LOC285847	-1.015047472	0.779323869
243844_at	RP11-138I17.1	-1.015051162	0.709595216
1567241_at	OR2L1P	-1.015136914	0.728233017
234219_at	RP11-30P6.6	-1.015138176	0.805447674
240746_s_at	RP11-245J24.1	-1.015146398	0.693259067
1561050_a_at	NAV2-IT1	-1.015181275	0.71063804
235994_s_at	TINCR	-1.015184009	0.749672034
1566219_at	KRTAP5-AS1	-1.015188274	0.902444874
230222_at	RP11-456H18.2	-1.01522973	0.795410894
1557369_a_at	LINC00698	-1.015300504	0.928763651
237584_at	RP11-684N3.1	-1.015323396	0.943033351

1561555_at	RP11-375F2.2	-1.015332786	0.665974058
236835_at	FUT8-AS1	-1.015390964	0.902103169
1553658_at	LINC00896	-1.015444612	0.733736997
214561_at	LILRP2	-1.015463662	0.785467543
1563983_at	TPT1-AS1	-1.015504575	0.784679083
1562122_at	RP11-214N1.1	-1.015522555	0.635252061
207252_at	INE1	-1.015539445	0.681790129
1568938_at	RP5-1109J22.2	-1.015556431	0.691797394
207524_at	ST7-OT3	-1.015561443	0.821955791
1569925_at	DNAH17-AS1	-1.015566034	0.780005372
243760_at	MIPEPP3	-1.015590633	0.725046185
1568663_a_at	PWRN2	-1.015603087	0.772302846
1558336_at	RP11-560G2.1	-1.015647257	0.747246939
1564352_at	CECR3	-1.015679952	0.602367135
236490_at	RP11-845C23.3	-1.015746587	0.707498659
207377_at	PPP1R2P9	-1.015750202	0.783301748
1567028_s_at	SH3GL1P2	-1.015804195	0.81698924
1562491_at	RP11-79E3.3	-1.015830293	0.640985829
1562903_at	FAM86B3P	-1.015892766	0.836691699
244875_at	ASMTL-AS1	-1.015898985	0.739700487
235929_s_at	ZNF503-AS2	-1.015904018	0.755796816
236598_at	LOC100996579	-1.015938921	0.729739766
1562447_a_at	PPP1R26-AS1	-1.015955588	0.751406451
237980_at	LINC00347	-1.015980829	0.715991942
1556999_at	LOC100271832	-1.016027408	0.722822189
1560563_at	RP11-388C12.1	-1.016065014	0.789291147
241522_at	LINC00454	-1.016123387	0.724566853
1556123_a_at	RAB11B-AS1	-1.016135942	0.82631337
216722_at	VENTXP1	-1.016153876	0.639241231
1569755_at	LINC00276	-1.016173724	0.82525354
239747_s_at	RP11-351M8.1	-1.016201057	0.738917418
1562058_at	RP11-114G22.1	-1.016202913	0.660020665
238527_at	CLUHP3	-1.01622192	0.661856851
1562621_at	RP11-26J3.1	-1.016247838	0.841352161
238113_at	SMIM2-AS1	-1.016311294	0.745188087
1556912_at	RP11-837J7.3	-1.01631472	0.595154856
1557717_at	LINC00550	-1.016365795	0.649214431
1562754_at	LOC339260	-1.016429852	0.754056022
1557161_at	LOC100132735	-1.016431594	0.647181229
215141_at	NOP14-AS1	-1.01643259	0.711992129
1564772_at	RP11-31K23.2	-1.016439444	0.512215283
1561539_at	LOC100506368	-1.016458914	0.76342929
227009_at	RP11-890B15.3	-1.016516104	0.845156402
242683_at	LINC00668	-1.016528109	0.721456486
1564144_at	RP11-274H24.1	-1.016543063	0.712304557
1559522_at	LOC100505918	-1.016588002	0.720316563
216698_x_at	OR7E12P	-1.016710346	0.822291293
232191_at	ERVH48-1	-1.01671431	0.771071308
236785_at	RP3-325F22.3	-1.016775727	0.776732726
1556921_at	RP4-753D4.2	-1.016777738	0.617807237

217317_s_at	HERC2P9	-1.016783387	0.918413899
1559510_at	LINC00630	-1.016794131	0.68927247
220608_s_at	RP11-463I20.1	-1.016796564	0.871597549
232575_at	PCA3	-1.016938738	0.499067882
1561732_at	RP1-69M21.2	-1.016957242	0.617366279
1563165_at	RP11-8L2.1	-1.016978829	0.649534864
233410_at	CYP1B1-AS1	-1.016996567	0.687366473
1559110_at	RP1-212P9.2	-1.017015321	0.793504654
1569496_s_at	LOC100130872	-1.017023999	0.710744693
1560633_a_at	C21orf91-OT1	-1.017026604	0.737263621
212181_s_at	NUDT4P1	-1.017071967	0.90212103
207287_at	BIN3-IT1	-1.017073212	0.760156254
233088_at	RP11-1102P22.1	-1.017127007	0.778081571
244268_x_at	LOC646214	-1.017132409	0.74866419
1563174_at	RP11-271O3.1	-1.017168485	0.684033025
240767_x_at	VPS13A-AS1	-1.017336235	0.636802963
226900_at	GABPB1-AS1	-1.017413825	0.785245826
1562013_a_at	LOC100506730	-1.01742082	0.859697577
208246_x_at	RP11-403P17.4	-1.017495925	0.865313643
1557647_a_at	VWA8-AS1	-1.0175222	0.70916646
1560753_at	RP11-215E13.2	-1.017532933	0.722440548
224139_at	SHANK2-AS3	-1.017548093	0.65718747
1563297_s_at	RP11-643M14.1	-1.017570901	0.682339791
237787_at	LOC100996263	-1.017587442	0.641597501
244505_at	RP11-646I6.6	-1.01764251	0.853166023
231593_at	RP11-960B9.2	-1.01765125	0.733418178
1559316_at	RP11-774D14.1	-1.017719203	0.722942952
78383_at	TOPORS-AS1	-1.017740424	0.803680567
232190_x_at	LOC115110	-1.017778419	0.655647114
1553122_s_at	RBAKDN	-1.017782406	0.902077479
1561590_a_at	CNTFR-AS1	-1.017861372	0.688297971
1562598_at	DLG5-AS1	-1.017862734	0.74429717
1560946_at	RP11-287D1.2	-1.017913598	0.74674119
233948_at	TBX5-AS1	-1.017939654	0.684503182
1569786_at	RP11-572C15.5	-1.017978175	0.636068191
1562054_at	SMEK3P	-1.018041336	0.542499102
1562759_at	RP13-270P17.2	-1.018063848	0.664231288
207894_s_at	TCL6	-1.018073877	0.62095141
208585_at	BTN2A3P	-1.018164679	0.778837171
1561700_at	RP11-489P6.1	-1.018179101	0.677356873
1570473_at	ANKRD30BP3	-1.018223787	0.704017431
224153_s_at	DHRS4-AS1	-1.018238861	0.666556286
1569673_at	RP11-21L23.2	-1.018300083	0.630396201
1558308_at	LINC00942	-1.018300217	0.729610598
220772_at	BPESC1	-1.018317921	0.580290876
236601_at	RP11-276H7.2	-1.018357069	0.750446346
1554666_at	LOC100130950	-1.01838494	0.702013835
238263_at	EPHA1-AS1	-1.018452903	0.734347278
1561339_at	RP11-214L13.1	-1.018470014	0.726347069
215975_x_at	GK-AS1	-1.01850679	0.703480203

1556844_at	LOC100132078	-1.018521542	0.657643259
1562729_at	RP11-893F2.14	-1.01853925	0.684225273
238244_at	LINC00535	-1.018546156	0.686251727
1559627_at	C7orf71	-1.018569255	0.644775193
1564878_at	RP11-231E4.2	-1.018573668	0.690107258
1562566_at	TRAF3IP2-AS1	-1.01860684	0.596921082
237028_at	ENO1-AS1	-1.018686215	0.909304543
233604_at	FLJ22763	-1.018698073	0.482859999
1553931_at	OSTCP1	-1.018710162	0.559968396
1561370_at	LOC100505716	-1.018725761	0.628661
1562044_at	RP11-632K5.3	-1.018729944	0.713604076
207028_at	MYCNOS	-1.018733456	0.592896237
1557692_a_at	RP11-519G16.1	-1.018752395	0.498358249
1563219_at	NADK2-AS1	-1.018776842	0.630739411
238620_at	RP11-846E15.4	-1.018778032	0.89694878
214750_at	PLAC4	-1.018850186	0.687453244
233541_at	LIMD1-AS1	-1.018860145	0.650727667
1561085_at	LOC153910	-1.018914303	0.695830019
1562424_at	LOC285889	-1.018918775	0.632574349
1570268_at	RP11-363G15.2	-1.018937929	0.539164077
237668_at	LOC100507201	-1.018950097	0.564790012
243823_at	RP1-28O17.1	-1.018981208	0.618703987
1555083_at	RPL13AP17	-1.018998252	0.681069339
230817_at	RP11-103H7.5	-1.01905735	0.582900117
217013_at	AZGP1P1	-1.019103955	0.586271049
1569885_at	LINC00882	-1.019196385	0.704474109
1554403_a_at	RP11-574H6.1	-1.019205638	0.638480706
237880_at	LOC100506457	-1.019219479	0.599986209
1563179_at	RP3-400B16.4	-1.019338204	0.528110303
1553919_at	C9orf62	-1.019383458	0.632777614
1561419_at	Z83844.1	-1.019400317	0.658810879
232022_at	RP11-477D19.2	-1.019405347	0.813347185
1557541_at	FAM201A	-1.019425969	0.714753332
244255_at	LOC286114	-1.019436761	0.645864528
238139_at	RP5-968P14.2	-1.019537478	0.838339449
1554839_at	CIDECP	-1.019551689	0.710310684
222072_at	ADD3-AS1	-1.019656432	0.687238939
242135_at	LOC642236	-1.019718526	0.626669591
1568844_at	RP11-716D16.1	-1.019773286	0.685946813
222211_x_at	SCAND2P	-1.019824189	0.686194482
231313_at	FLJ27354	-1.019866103	0.803478941
231024_at	PGM5-AS1	-1.019890999	0.88923636
237789_at	RP11-67L3.4	-1.019898299	0.697441992
1561213_at	RP4-598G3.1	-1.019922255	0.528468316
233524_at	LINC00475	-1.020019485	0.698960426
1555980_a_at	RP11-54O7.3	-1.020068748	0.773798627
221971_x_at	AGAP11	-1.020079718	0.842921709
1560881_a_at	LINC00112	-1.020099682	0.712251493
237598_at	SEC1P	-1.020124046	0.672181251
216830_at	HERC2P10	-1.020134441	0.714723028

234216_at	FLJ21408	-1.020182267	0.629446119
208287_at	HCG9	-1.020197582	0.645938794
241423_at	ZBED3-AS1	-1.020237456	0.74719873
1561984_at	LINC00838	-1.020284865	0.586897202
1563263_at	RP11-960L18.1	-1.020315383	0.714520753
1562826_at	RP11-429H9.4	-1.020325953	0.751123012
1569739_at	LOC221946	-1.020345594	0.615553216
1557466_at	FLJ31104	-1.020381726	0.770660655
1564070_s_at	HOTTIP	-1.020407135	0.637855201
228048_at	ZNF503-AS2	-1.020421799	0.638806719
237754_at	RP11-178L8.5	-1.020449957	0.681473604
1558982_at	ANKRD20A12P	-1.020539145	0.805927452
1570189_at	LINC00671	-1.020573247	0.537710262
1564178_at	LINC00841	-1.020584703	0.652754463
1557382_x_at	AGAP11	-1.020636319	0.725360972
238298_at	LINC00845	-1.020666694	0.635309778
1553780_at	LINC00638	-1.020790028	0.717928674
235416_at	LOC643201	-1.020880861	0.726172284
1557731_at	LOC400620	-1.020886572	0.640033621
242136_x_at	MGC70870	-1.02096651	0.850465275
1553434_at	CYP4Z2P	-1.021091208	0.623218983
1560582_a_at	RP11-804F13.1	-1.021267261	0.610605064
1561978_at	LOC284798	-1.021330107	0.601007903
1563103_at	RP11-429H9.4	-1.021333935	0.638303793
218994_s_at	STAG3L4	-1.02135275	0.854074727
1560619_at	LINC00935	-1.021501429	0.594324289
207161_at	KIAA0087	-1.021542412	0.557463141
1562972_at	LINC00929	-1.021557094	0.609621927
1561593_at	RP11-400D2.2	-1.021582878	0.618819451
1569090_x_at	LINC00957	-1.021601158	0.683387505
230776_at	RNF157-AS1	-1.021618091	0.912162484
1558881_at	LINC00924	-1.02171377	0.86934998
235499_at	LOC728743	-1.021781918	0.699344068
1561083_at	RP11-243E13.1	-1.021802934	0.520332232
1560213_at	HCP5B	-1.02186949	0.635011115
1557424_at	LOC100505878	-1.02187051	0.462474579
1558195_at	LINC00592	-1.021890662	0.688021109
1554187_at	LOC554206	-1.021935761	0.618273832
1568852_x_at	RP11-295M18.2	-1.021969774	0.549394586
215608_at	RP11-229O3.1	-1.021996762	0.790124345
239779_at	RP11-326C3.2	-1.022135909	0.661649716
1562499_at	RP11-314P15.1	-1.022140703	0.387140358
1562616_at	RP11-809F4.3	-1.022151504	0.477095257
239157_at	ZSCAN12P1	-1.022192609	0.769888176
1566844_at	PER4	-1.022204874	0.671779776
1557822_at	LOC401134	-1.022230655	0.611017361
1556366_s_at	LY86-AS1	-1.022238626	0.873998619
240323_at	RP11-728F11.4	-1.022322328	0.640160084
244374_at	TINCR	-1.022374379	0.727649668
237415_at	RP11-493L12.3	-1.022394425	0.563774499

1570085_at	LOC100506679	-1.022416552	0.605595313
1570155_at	RP11-706J10.1	-1.022454163	0.591526993
237771_s_at	RP11-614F17.2	-1.022518623	0.787474509
237170_at	LOC100507384	-1.022591601	0.601695959
236870_at	IQCF4	-1.022635421	0.476954231
240682_at	LINC00293	-1.022707154	0.6338981
1554880_at	DKFZP434K028	-1.022768818	0.594465454
1553443_at	FER1L6-AS1	-1.022824705	0.514773
237957_at	LOC643711	-1.022840657	0.583294486
200080_s_at	H3F3AP4	-1.022873294	0.772721824
1561407_at	RP11-399D2.1	-1.022888514	0.475542979
1568635_at	LINC00354	-1.022912945	0.56828451
225457_s_at	PP7080	-1.022923946	0.811997681
1557009_a_at	LOC340107	-1.022933137	0.622648791
1558967_s_at	HCG18	-1.022942578	0.732586741
1559205_s_at	LOC100996286	-1.022955102	0.582623631
1561469_at	RP11-452H21.4	-1.022969919	0.546245869
240740_at	LOC100507443	-1.023099467	0.44937289
232227_at	RP11-251M1.1	-1.023136757	0.568628578
1557993_at	RP11-510J16.3	-1.023152212	0.545055301
1569763_at	RP11-443A13.5	-1.023153716	0.537118087
222361_at	TUBBP5	-1.02318563	0.73915311
1561198_at	LOC100506127	-1.023216177	0.62204808
234400_at	RP5-968J1.1	-1.023347298	0.665523941
242246_x_at	MEG3	-1.023486417	0.685046231
232684_at	ZNF503-AS1	-1.023548533	0.575967972
1553741_at	LINC00337	-1.023556842	0.578360676
1564287_at	LINC00410	-1.023558963	0.641698472
231378_at	RP11-231D20.2	-1.023690128	0.664336684
211445_x_at	NACAP1	-1.023719437	0.591434465
1569468_at	ZNF876P	-1.023751349	0.506136752
224426_s_at	ACTR3BP5	-1.023824608	0.623076938
1562038_at	RP11-355N15.1	-1.023860033	0.508851226
1557727_at	PCBP1-AS1	-1.023868114	0.636967142
201065_s_at	GTF2IP1	-1.023917871	0.774635263
222156_x_at	DYX1C1-CCPG1	-1.02397411	0.904218737
1561897_at	RP11-932O9.4	-1.023976367	0.553639158
217054_at	RP11-395B7.2	-1.02403178	0.680339594
1569276_at	RP11-394B2.3	-1.024036679	0.6223168
240101_at	RP11-390B4.5	-1.024072614	0.771404768
234058_at	RP11-515O17.3	-1.024140918	0.531190862
235466_s_at	RP11-452F19.3	-1.024218275	0.800335521
239958_at	PSMD5-AS1	-1.024220301	0.627681917
231278_at	RP13-870H17.3	-1.024340688	0.56534323
1555196_at	LINC00421	-1.024362028	0.602322876
1563867_at	LOC283194	-1.024394481	0.669016851
1561003_at	RP11-298D21.2	-1.024401428	0.565335619
1557613_at	KIF9-AS1	-1.02442983	0.479322322
243639_at	RP11-505E24.3	-1.024471954	0.601957092
1562802_at	LINC00210	-1.024498313	0.576272302

1569728_at	LINC00423	-1.024556032	0.576946594
1558477_at	LOC100131496	-1.024637839	0.58843685
1553450_s_at	LINC00304	-1.02466733	0.48477174
213363_at	CA5BP1	-1.024874904	0.769802921
1564760_at	RP11-395N3.1	-1.024880754	0.647819788
1556122_at	RAB11B-AS1	-1.02489251	0.572660957
1569722_s_at	PROSER2-AS1	-1.024896738	0.475677126
236497_at	LOC729683	-1.024916023	0.561727396
236731_at	LRRC2-AS1	-1.024930096	0.617598204
1565911_at	RP11-752G15.10	-1.025040373	0.481051156
1560286_s_at	RP11-1069G10.2	-1.025063117	0.50086363
236893_at	HOXB-AS3	-1.025075126	0.641096274
1553087_at	C18orf12	-1.025107641	0.550591845
1569454_a_at	LOC283352	-1.025146722	0.460520296
239964_at	TCL6	-1.025166263	0.548711915
232288_at	PDXDC2P	-1.025187272	0.774931152
232452_at	GCSAML-AS1	-1.025227612	0.503842291
1557827_at	DNAJC9-AS1	-1.025245764	0.531355337
1565554_at	LINC00628	-1.025252581	0.48111054
1559611_at	TMEM75	-1.025281007	0.457442993
215297_at	LOC441204	-1.02532697	0.910624247
1557034_s_at	LOC100288778	-1.025331006	0.788800518
1562693_at	RP5-971N18.3	-1.025470569	0.562446281
1568689_at	LOC100631378	-1.025504777	0.53581201
1558661_at	LOC100506207	-1.025562997	0.615041436
1560767_at	HCG22	-1.025589345	0.712500297
240814_at	MGC39584	-1.025624543	0.548976637
236493_at	NKAPP1	-1.025652516	0.704015131
1557656_at	LINC00642	-1.025710106	0.609813638
239915_at	HOXA-AS3	-1.025736111	0.606138062
1558794_at	NUTM2A-AS1	-1.025855768	0.642899864
231491_at	LINC00113	-1.02589283	0.461717442
220479_at	CPS1-IT1	-1.025907805	0.552749749
1563005_at	RP11-74C13.3	-1.025941989	0.608634282
230073_at	LOC100131564	-1.025941989	0.609817854
238112_at	LOC283177	-1.025957584	0.559238993
240810_at	MKNK1-AS1	-1.025979459	0.550675043
1564887_at	RP11-440L14.3	-1.025996949	0.52102823
1558436_a_at	RP11-521O16.2	-1.026047736	0.604523787
243499_at	RP11-1103G16.1	-1.026072687	0.492149587
232448_at	LINC00543	-1.026132504	0.664767321
1558250_s_at	RP11-350F4.2	-1.026157843	0.81146871
1563845_at	LOC100507387	-1.026179085	0.703030757
1558711_at	FAM13A-AS1	-1.026328468	0.739982013
1553428_at	LY86-AS1	-1.026334808	0.798494022
216659_at	LOC1720	-1.026397135	0.465645713
1560911_at	LOC100133461	-1.026446725	0.572215817
228247_at	LOC283788	-1.026466639	0.804987863
1557399_at	LOC284009	-1.026530482	0.790899377
1552819_at	LINC00205	-1.026558228	0.600796881

238422_at	LBX2-AS1	-1.026578318	0.686199304
1556744_a_at	RP11-159G9.5	-1.026692098	0.832921738
1553497_at	LINC00615	-1.026729074	0.446539658
1561033_at	RP11-203B9.4	-1.026781658	0.521794055
1560707_at	LOC283856	-1.026797053	0.53356634
241899_at	LOC553103	-1.026808463	0.575318686
1566127_at	RP13-1039J1.3	-1.026817979	0.555258286
235667_at	USP46-AS1	-1.026865442	0.629123785
1569756_at	RP11-70F11.8	-1.026914377	0.428337532
200012_x_at	RPL21P28	-1.026916795	0.858098519
1564446_at	LOC284930	-1.026925325	0.575136884
241327_at	RP11-61G23.1	-1.026931476	0.454734989
238278_at	LINC00415	-1.02696316	0.582124125
1556271_at	RP11-843A23.1	-1.027044812	0.573110672
242139_s_at	ERVK3-1	-1.027064737	0.830375435
1560818_at	LINC00944	-1.027143224	0.539721847
1559514_at	LOC100132077	-1.027179674	0.502888892
1560526_at	MAGI2-IT1	-1.027197145	0.869042737
1559683_at	RP11-507J18.5	-1.027289071	0.542870203
214186_s_at	HCG26	-1.027350526	0.578918594
1561245_at	RP11-45K10.2	-1.027359602	0.501593281
232926_x_at	ANKRD19P	-1.027364324	0.619230636
1568892_at	LOC100996251	-1.02737607	0.585063995
1563043_at	LINC00620	-1.027480668	0.472457284
237909_at	ADAM6	-1.027594239	0.473604312
227502_at	RP5-894A10.2	-1.027601342	0.827210473
1562916_at	LINC00102	-1.027655229	0.323867744
244182_at	RP1-118J21.25	-1.027719455	0.607754441
1567027_at	SH3GL1P2	-1.02773591	0.629338864
1557146_a_at	SSTR5-AS1	-1.02777691	0.541177121
1560771_at	RP11-285A1.1	-1.027777278	0.48770103
1569936_a_at	RP11-214K3.24	-1.027830536	0.407554904
240561_at	RP5-842K24.2	-1.027833652	0.694104435
1560410_at	RP11-449L23.2	-1.02789851	0.533615576
1561279_at	RP11-231E19.1	-1.027931211	0.515169884
230373_at	PRKAG2-AS1	-1.027937252	0.460870242
244405_s_at	RP11-753A21.1	-1.027941589	0.391153043
244831_at	RP11-945A11.2	-1.028034317	0.405291012
235928_at	ZNF503-AS2	-1.028067584	0.640076174
1559712_at	LINC00689	-1.028093978	0.911073549
234281_at	ESPNP	-1.028121244	0.555203496
244372_at	LOC101060019	-1.028192394	0.576989213
1570445_a_at	LOC643201	-1.028197429	0.781675662
213329_at	SRGAP2D	-1.028354795	0.764702051
233170_at	LINC00348	-1.028364305	0.374210337
222226_at	SAA3P	-1.02837248	0.520130075
233513_at	RP11-847H18.2	-1.028386039	0.666226132
215327_at	RP11-414H17.5	-1.028395549	0.57615862
1563800_at	LOC283140	-1.028423869	0.467826665
241126_at	RP11-45L9.1	-1.028462457	0.465203685

1563244_at	RP11-650K20.3	-1.028469004	0.56795054
237806_s_at	LOC729296	-1.028493123	0.562816161
1560038_at	LOC100506071	-1.028503409	0.49504378
1562862_at	RP3-388M5.9	-1.028537157	0.610875737
1557878_at	RP11-10K16.1	-1.028705561	0.492784445
239691_at	C12orf77	-1.028718737	0.526287867
225515_s_at	WAC-AS1	-1.028749798	0.671580936
1562311_at	RP11-521H3.3	-1.028798416	0.458023159
1568722_at	RP11-1078H9.5	-1.028837734	0.60640548
1562048_at	LOC152225	-1.028883217	0.350527727
230227_at	RP11-333I13.1	-1.02888595	0.580031677
215770_at	OR7E2P	-1.028977697	0.434530106
237574_at	RP4-594L9.2	-1.029079919	0.505169202
221804_s_at	FAM45B	-1.029149682	0.748202592
238910_at	CLUHP3	-1.029169493	0.652109722
238893_at	LINC00936	-1.02917463	0.870669931
232732_at	LOC100652999	-1.029183121	0.478019165
1561757_a_at	LOC283352	-1.029270455	0.495741104
1553432_s_at	LOC653786	-1.029278617	0.487746352
1553438_at	C11orf72	-1.029301261	0.399688351
1560175_at	PPP4R1L	-1.029306612	0.531115503
237595_at	RP11-38C18.2	-1.029403709	0.563471987
237154_at	RP1-28O10.1	-1.029408634	0.474942143
1561482_at	RSU1P2	-1.029413733	0.522846153
1570106_at	RP11-439E19.1	-1.029456333	0.486338729
241218_at	RP11-752D24.2	-1.029456546	0.507961963
233774_at	RP11-463O9.2	-1.029490926	0.510414133
1556436_at	EXTL3-AS1	-1.029498314	0.708494896
1561420_a_at	Z83844.1	-1.02955071	0.456154965
243974_at	RP3-331H24.6	-1.029562132	0.855360114
1562036_at	RP11-109G23.3	-1.029655608	0.63984173
1562661_at	RP4-564F22.5	-1.029670541	0.496815215
1561605_at	RP11-394G3.2	-1.02973278	0.470882856
208286_x_at	POU5F1P3	-1.029811179	0.529622675
244727_at	KCNQ1OT1	-1.02982918	0.531517455
234053_at	RP11-442O18.2	-1.02983467	0.463955797
235124_at	EIF3J-AS1	-1.029871836	0.782539784
1561390_at	FAM41AY2	-1.02987492	0.579898324
230310_at	RP11-464F9.20	-1.029900992	0.688387039
240534_at	RP11-38C18.3	-1.02995108	0.528517009
231106_at	BMS1P6	-1.029982799	0.784741964
1557566_at	LOC100507634	-1.030076546	0.541657853
236572_at	RP4-647C14.3	-1.030077749	0.517220367
1560714_at	FLJ37035	-1.030085742	0.536468613
1552401_a_at	GRIK1-AS2	-1.030088187	0.60539319
1558107_at	LOC283788	-1.030093833	0.739436356
224045_x_at	LINC00470	-1.030098955	0.472537165
1553912_at	LINC00955	-1.030120531	0.431286542
233960_s_at	LOC115110	-1.03020255	0.569399961
240427_at	RP11-73B2.2	-1.030204529	0.402933696

1560841_at	LOC389247	-1.030227388	0.398299198
1557389_at	SH3PXD2A-AS1	-1.030412547	0.595632856
220452_x_at	CECR7	-1.030428151	0.621889247
1562068_at	RP11-371I20.2	-1.030479391	0.534151258
208266_at	C8orf17	-1.03048764	0.588508231
1561702_at	RP11-753A21.2	-1.030492861	0.435857965
222801_s_at	STAG3L4	-1.03056206	0.811995762
240827_at	GATA3-AS1	-1.030563147	0.529407276
236614_at	LOC729683	-1.030568349	0.481043669
234097_s_at	ZNRD1-AS1	-1.030603853	0.282536976
241552_at	AA06	-1.03068659	0.385632447
1561544_at	RP11-609L23.1	-1.030747686	0.426192093
238412_at	RRN3P3	-1.030771605	0.781290247
240745_at	RP11-245J24.1	-1.030825037	0.411995452
1560448_at	PRR7-AS1	-1.030831658	0.403062278
1556916_a_at	RP11-2O17.2	-1.030841153	0.513798628
237793_at	C20orf78	-1.030855036	0.537998123
1570345_at	LINC00474	-1.030866336	0.359444092
1566555_at	BAIAP2-AS1	-1.030913579	0.421819297
1560522_at	DLGAP1-AS3	-1.030914336	0.511174839
237563_s_at	RP11-295G20.2	-1.030939482	0.733418452
237406_at	RP11-121A14.3	-1.031088276	0.615183931
1562930_at	SRRM2-AS1	-1.0311096	0.476065872
224456_s_at	FOXD2-AS1	-1.031161243	0.71795729
241475_at	BREA2	-1.031164021	0.470875247
1559372_at	RP1-224A6.3	-1.031213627	0.466023414
1556768_at	LINC00930	-1.031220561	0.542315714
1559667_at	RP11-83N9.5	-1.031231613	0.62093814
240828_at	JARID2-AS1	-1.031502319	0.464009612
231196_x_at	LINC00202-1	-1.031542693	0.57307783
231595_at	MRVI1-AS1	-1.031564766	0.440705682
231637_at	LOC100499194	-1.031640293	0.427668199
1556721_at	C20orf203	-1.031732966	0.674384511
1554615_at	RP11-43D4.3	-1.031836395	0.478055967
1561513_at	RP11-461F11.3	-1.031842536	0.370614277
1561322_at	RP11-165H23.1	-1.031850641	0.315528573
242889_x_at	FUT8-AS1	-1.031886344	0.647761519
1554732_at	LOC728175	-1.031942147	0.54681766
1570105_at	RP5-963E22.5	-1.03196312	0.413059588
1564109_at	LOC284865	-1.032003123	0.560259392
230632_at	IL10RB-AS1	-1.032020404	0.573034184
1561728_a_at	LINC00642	-1.032071082	0.384712661
244175_at	LINC00577	-1.032106949	0.461159056
1556274_at	RP11-480I12.10	-1.032120168	0.534088873
244540_at	RP11-24P14.1	-1.032184596	0.433467761
221206_at	PMS2CL	-1.032187629	0.660130777
1569393_at	MGC15885	-1.032257077	0.429922859
234457_at	ZNRD1-AS1	-1.032279495	0.362410116
240075_at	RP3-467K16.2	-1.032297442	0.472092443
1570182_at	RP11-118E18.4	-1.03231813	0.442090156

237075_at	LOC101060091	-1.032337555	0.43056193
1560282_at	RP11-806K15.1	-1.032424379	0.413936385
1565732_at	RP1-197B17.3	-1.032454716	0.443796712
1560630_at	RP11-109E24.2	-1.032480153	0.453535781
1568851_at	RP11-295M18.2	-1.032494913	0.446853722
1562153_a_at	PVT1	-1.032498919	0.412207859
233418_at	LOC91450	-1.032529511	0.395114838
1561210_at	RP11-478P10.1	-1.032544972	0.387876989
238565_at	RP11-350F4.2	-1.032595248	0.740581464
1568690_a_at	LOC100631378	-1.032598632	0.473284972
1560595_at	LOC100128993	-1.032617421	0.342976106
215126_at	RP11-2E17.1	-1.032622225	0.87645009
238283_at	LINC00635	-1.032669762	0.402661872
1563898_at	RP11-421F16.3	-1.032689466	0.494810275
237730_at	LOC100130700	-1.032690322	0.511085647
1564601_at	LINC00221	-1.032767877	0.487929301
240495_at	RP11-166P13.4	-1.032771047	0.460000468
238456_at	LOC100289230	-1.03277984	0.74680821
1555994_at	DIAPH3-AS1	-1.03280723	0.434075333
242972_at	HCG18	-1.032872966	0.841229103
1562436_at	CARS-AS1	-1.032917946	0.441607153
1559753_at	RP11-76C10.3	-1.032971061	0.430683663
1558930_at	LINC00460	-1.03298182	0.543329397
233556_at	CRYBB2P1	-1.032992813	0.476767469
233775_x_at	LOC100289333	-1.033012115	0.758116052
1570082_x_at	RP5-1073O3.7	-1.033027058	0.582506884
1560988_a_at	LINC00556	-1.033037215	0.34160019
237460_x_at	C14orf182	-1.033044706	0.385798283
1559169_at	RP1-187B23.1	-1.033048092	0.405192793
1555988_a_at	LINC00661	-1.033103705	0.435934399
1560198_at	LINC00523	-1.033194135	0.439297884
1563821_at	LINC00858	-1.033217624	0.473593428
232703_at	GLUD1P7	-1.033309602	0.498153176
1562251_a_at	LOC574538	-1.033332315	0.413658321
238163_at	RP11-273B20.1	-1.033344305	0.284690129
1568974_at	NHEG1	-1.033359525	0.476476876
242888_at	PRRT3-AS1	-1.033523093	0.743229209
1559333_at	SRGAP3-AS2	-1.033545792	0.454186808
1561225_at	ANKRD30BP3	-1.033603592	0.462937247
1565424_at	LINC00529	-1.033615643	0.292644185
1562365_at	LOC286177	-1.033617902	0.327369271
243384_at	LINC00280	-1.033628804	0.424132718
1570600_at	RP13-122B23.8	-1.033700394	0.465331583
1566831_at	SBF2-AS1	-1.033771638	0.496023768
1557208_at	PROSER2-AS1	-1.033787722	0.5557455
231135_at	LOC151174	-1.033826842	0.583158566
231076_at	C16orf82	-1.03385537	0.481193671
1564069_at	HOTTIP	-1.033859595	0.478211884
1559528_at	LOC100129917	-1.033958622	0.734234291
1564721_at	RP11-37N22.1	-1.033990678	0.422121987

1557605_a_at	LOC401312	-1.034014439	0.36025346
1554185_at	LOC554206	-1.034036038	0.628323682
234715_at	GOLGA2P3Y	-1.034067396	0.464174768
243818_at	SFTA1P	-1.034077115	0.443749337
1560207_at	RAD21-AS1	-1.034119577	0.562725891
243971_x_at	LINC00202-2	-1.034135237	0.386967926
241187_at	RP11-5N11.2	-1.034208966	0.519077171
229094_at	ATP6V0E2-AS1	-1.034234563	0.777534566
1570465_at	RP11-153K16.2	-1.034275278	0.426652342
1557024_at	RP11-744D14.2	-1.034306935	0.533421109
1554301_at	LHFPL3-AS1	-1.03432404	0.360507614
1554887_at	RP11-132A1.4	-1.03436772	0.345974109
220377_at	KIAA0125	-1.034428235	0.47475871
1554763_at	UBE2DNL	-1.034512546	0.432750597
1557444_at	TREML3P	-1.03453287	0.531811785
1560819_a_at	LINC00944	-1.034557266	0.456438561
244518_at	LOC100130452	-1.034604522	0.419721521
228207_at	LOC100499489	-1.034622548	0.73542002
1564049_at	LOC339593	-1.034644452	0.397102031
1554222_at	MGC45922	-1.034677095	0.544751909
1562785_at	RP11-10L7.1	-1.034723557	0.754582
1562674_at	RP11-495P10.5	-1.034872741	0.483031295
244296_at	RP11-456O19.2	-1.034876698	0.398435716
231404_at	SPTY2D1-AS1	-1.034913189	0.541414871
1559103_s_at	RP11-73K9.2	-1.034948004	0.752315428
233338_at	FAM230C	-1.034973112	0.442981596
1557498_a_at	RP11-451G4.1	-1.035056375	0.375904833
215697_at	RP11-117L5.3	-1.035112876	0.364177526
227596_at	FLJ37453	-1.035126056	0.44695655
237564_at	RP11-295G20.2	-1.035250319	0.428001515
1556847_s_at	RP11-1006G14.1	-1.035271944	0.443866471
239498_at	RP11-498E2.7	-1.035299478	0.559129654
1556900_at	APCDD1L-AS1	-1.035316209	0.392773203
213356_x_at	HNRNPA1P10	-1.035323425	0.605772508
1557484_at	RP11-634B7.4	-1.035445293	0.447569351
1553832_at	LINC00315	-1.035480282	0.284516877
237249_at	KCNQ1OT1	-1.035560056	0.480845993
214410_at	RP11-348B17.1	-1.035567487	0.425161966
1563316_at	NEGR1-IT1	-1.035592142	0.594208014
242754_at	RP11-314A20.2	-1.035596375	0.475404628
1570270_at	RP11-15K3.1	-1.035628522	0.324828919
236842_at	RP11-290H9.4	-1.035643815	0.380394579
221850_x_at	BMS1P5	-1.035669818	0.739588502
233749_at	RP11-368D24__A.1	-1.035762346	0.361027473
1562703_at	LINC00964	-1.035781309	0.451925219
237523_at	RP11-66N11.7	-1.035840756	0.411505752
1562801_at	RP11-166A12.1	-1.035843819	0.446775863
229216_s_at	RP11-567I13.1	-1.035867427	0.597871635
1556180_at	LINC00847	-1.035894684	0.659461882
1570163_at	EGFLAM-AS4	-1.035904889	0.470231001

219839_x_at	TCL6	-1.035924479	0.524117736
240361_at	RP1-232P20.1	-1.035941006	0.428322536
230548_at	LOC100505942	-1.035944967	0.527461526
239506_s_at	LINC00608	-1.035988403	0.423173715
1554983_at	LINC00317	-1.036022259	0.408088376
1560964_at	RP11-315F22.1	-1.036028074	0.352515632
238180_at	RP11-395B7.4	-1.036086384	0.405537435
1555554_at	BPIFA4P	-1.036305524	0.528649091
1557330_at	RP11-236J17.3	-1.036458528	0.435139129
1569426_at	LOC100996342	-1.036472428	0.392699091
232462_s_at	A1BG-AS1	-1.036514637	0.497872096
244366_at	LOC100506371	-1.036585294	0.45875158
1562103_at	RP11-182I10.3	-1.03661257	0.577237206
216726_at	VENTXP1	-1.03663274	0.48441566
244105_at	WHAMMP3	-1.036856097	0.480528657
240273_at	RP11-39H13.1	-1.036859397	0.444388995
1558685_a_at	LOC158960	-1.03691461	0.562144458
238522_at	RP11-137L10.6	-1.036914961	0.447121
1563265_at	RP5-881L22.4	-1.036922871	0.380657657
240049_at	RP13-39P12.3	-1.037045592	0.30819958
1562871_at	AOX2P	-1.037053542	0.469482098
1555188_at	MTUS2-AS1	-1.037085929	0.395023157
240009_at	LINC00334	-1.037107787	0.321152888
1567036_at	C20orf181	-1.037133397	0.430486805
240999_at	LOC100287704	-1.0371728	0.285332367
216063_at	HBBP1	-1.037218319	0.522671612
1562730_a_at	RP11-893F2.14	-1.037274781	0.693530572
222090_at	NDUFB2-AS1	-1.037321263	0.62655311
224289_s_at	VN1R10P	-1.037350981	0.255272385
1563241_at	RP11-20B24.5	-1.037360751	0.337902722
236657_at	LOC100288911	-1.037405653	0.715194261
235161_at	RASSF8-AS1	-1.037437503	0.639345292
239926_at	RP11-727A23.11	-1.037438793	0.7940438
237888_at	LOC100422737	-1.037451202	0.311385936
1561288_at	LINC00536	-1.037590636	0.404889951
1563568_at	LINC00559	-1.037604394	0.39032212
1569741_at	RP11-653G8.2	-1.03760758	0.294071762
220718_at	HEXA-AS1	-1.037644929	0.501456037
239869_at	RP11-610P16.1	-1.037647665	0.481267379
240692_at	MGC34796	-1.037661542	0.299861476
237749_at	RP11-619L19.1	-1.037741855	0.320283919
1561556_at	RP11-135J2.3	-1.037750221	0.371864819
228675_at	USP30-AS1	-1.037839689	0.33684661
1570297_at	WI2-89031B12.1	-1.037883674	0.410941474
238664_s_at	MGC12916	-1.037911903	0.488986602
207280_at	RNF185-AS1	-1.037963066	0.334110258
1552955_at	LINC00208	-1.037999841	0.429065087
1569849_at	RP1-297M16.2	-1.038039218	0.420894565
231589_at	RP5-937E21.8	-1.038082488	0.318277399
232839_at	STK24-AS1	-1.038104387	0.479395714

1561490_at	NPSR1-AS1	-1.03810775	0.529189843
244179_x_at	GGT3P	-1.03813405	0.402464555
213788_s_at	LINC00094	-1.038142438	0.824605037
211461_at	CSPG4P1Y	-1.038161601	0.390645161
226362_at	RP11-846E15.2	-1.038172982	0.761464345
227887_at	FOXN3-AS1	-1.038180041	0.435494515
1564122_at	LINC00514	-1.038201356	0.379038886
1564263_at	LINC00330	-1.038288419	0.469817667
223779_at	AFAP1-AS1	-1.038365377	0.348364698
1560827_at	RP11-30K9.6	-1.038410401	0.664439681
81737_at	LOC100505915	-1.038426713	0.697438507
1561678_at	RP5-1069C8.2	-1.038459691	0.293147558
1561411_at	RP11-16L9.3	-1.038472757	0.336026456
236803_at	RP11-156P1.3	-1.038498147	0.58066074
240754_at	LOC284933	-1.03856131	0.350534828
211933_s_at	HNRNPA3P1	-1.038612035	0.676569518
1563171_at	RP11-466A17.1	-1.038624849	0.399606918
230374_at	RP11-783K16.13	-1.038650829	0.510556711
1554286_at	FLJ25758	-1.03866734	0.363175823
231232_at	LOC100506125	-1.03872036	0.62096703
1553811_at	FAM222A-AS1	-1.03874057	0.370503064
232248_at	LOC100652768	-1.038742683	0.279348609
235788_at	HCG18	-1.03896401	0.661338718
220445_s_at	CSAG3	-1.03896808	0.420273333
1562850_at	RP11-3B12.2	-1.038976378	0.207554817
1562107_at	XXyac-YR29IB3.1	-1.039066559	0.262806265
232281_at	LINC00662	-1.039123317	0.796160594
1560698_a_at	TRHDE-AS1	-1.039196051	0.756389875
244718_at	MTMR9LP	-1.039446097	0.451206126
244381_at	RP11-385F5.4	-1.039454477	0.596152488
1561408_at	RP11-167H9.3	-1.03945618	0.37487457
1554782_at	KLHL30-AS1	-1.039458294	0.47301452
1564112_at	RP11-338C15.5	-1.039482396	0.338840082
221093_at	BRD7P3	-1.039572915	0.313476602
1556945_a_at	RP11-144L1.4	-1.039575832	0.276173521
231679_at	RP11-161I6.2	-1.039582979	0.516641613
217380_s_at	ADD3-AS1	-1.039595081	0.632217788
237682_at	RP11-742B18.1	-1.039638141	0.304400892
231627_at	LINC00919	-1.039666966	0.339385605
227298_at	TRAM2-AS1	-1.039667456	0.658823569
225225_at	OIP5-AS1	-1.0396851	0.759917952
232230_at	LINC00263	-1.039694989	0.797469684
1563904_at	LOC100129620	-1.039723875	0.397498695
1562081_a_at	LINC00424	-1.039776556	0.305394303
1562671_s_at	RP11-862G15.1	-1.039811085	0.372754774
1555839_a_at	C3orf79	-1.039863301	0.166091113
1561271_at	CCDC144CP	-1.039916773	0.694273324
1564200_at	LINC00607	-1.039922571	0.383422341
240002_at	LINC00937	-1.040014205	0.525311964
1557207_s_at	LOC283177	-1.040043785	0.535322172

233062_at	RP1-80B9.2	-1.040044099	0.272661561
239565_at	DLG5-AS1	-1.040115388	0.307229275
242663_at	LINC00662	-1.040134921	0.735105302
1557896_at	RP11-495P10.3	-1.040163133	0.351825498
1557488_at	CBX3P2	-1.040208646	0.518547854
233934_at	LOC349160	-1.04028657	0.3244976
229643_at	AC078883.4	-1.040368321	0.296039726
1568871_at	CADM2-AS1	-1.040401478	0.402623683
233371_at	ABCC13	-1.040458387	0.431682564
1557068_at	LOC100505782	-1.040467677	0.239720107
240431_at	LINC00459	-1.040497054	0.315652313
242017_at	RP11-159G9.5	-1.040502404	0.610812634
243332_at	RP11-124L9.5	-1.040535977	0.824556388
1561185_at	TTTY7	-1.040545639	0.260735922
211350_s_at	KIF25-AS1	-1.040585525	0.432611864
223757_at	DIO3OS	-1.040588171	0.456179242
224006_at	LOC100507377	-1.040660556	0.51329858
225391_at	LOC93622	-1.04066873	0.684996645
236398_s_at	RP11-443B7.1	-1.040718342	0.411694241
240919_at	RP11-536G4.2	-1.04072587	0.450195439
232718_at	LINC00589	-1.040799676	0.428770884
1567242_at	OR2L1P	-1.040824103	0.443234244
1564670_at	RAMP2-AS1	-1.040883565	0.454338673
242313_at	LOC728730	-1.040933169	0.598333043
237808_at	ST7-AS2	-1.040971892	0.625170067
234012_at	RP11-174G17.3	-1.040985441	0.596357938
1556944_at	RP11-144L1.4	-1.041015735	0.370206105
239262_at	RP11-736K20.4	-1.041134272	0.83498532
220583_at	RP11-864N7.4	-1.041184574	0.258702147
1561530_at	RP11-365O16.6	-1.04120344	0.487651538
237331_s_at	MAPT-AS1	-1.041250117	0.313696583
1557296_at	FLJ12825	-1.041257727	0.467099923
243784_s_at	LOC100272217	-1.04126363	0.520935269
1556518_at	DLG1-AS1	-1.041307073	0.402286025
1569127_at	RP11-876N24.5	-1.041353813	0.489559619
1563074_at	LOC255654	-1.04136262	0.398486217
1558764_at	RP3-496C20.1	-1.041386805	0.258891043
240185_at	LOC100147773	-1.041402066	0.589968396
232586_x_at	LOC100133315	-1.041551036	0.771320384
1569365_a_at	KIAA1656	-1.041605831	0.396961823
235532_at	RP11-226L15.5	-1.041606537	0.665840269
1565729_at	RP13-436F16.1	-1.041627687	0.286783257
1563013_at	LOC646522	-1.041671616	0.380193631
1562803_at	LRRC37A5P	-1.041675599	0.24844917
1561305_at	RP11-24I21.1	-1.041811519	0.428613297
1553020_at	SMCR5	-1.041822135	0.672835172
241317_at	RP11-701I24.3	-1.041840326	0.349626194
1562000_at	LOC400620	-1.041868741	0.46892119
1569789_at	RP11-260O18.1	-1.041882145	0.366102415
1566884_at	RP11-45M22.3	-1.041946809	0.225060539

243749_s_at	LOC100507316	-1.041951382	0.464544549
1562717_at	LINC00299	-1.042025059	0.247586251
1561454_at	RP11-576E20.1	-1.042033734	0.302254301
239182_at	HOXD-AS1	-1.042049515	0.297894879
1557723_at	LOC285847	-1.04205399	0.336277739
1557060_at	RP11-522D2.1	-1.042146656	0.370914793
1569878_at	CCNYL2	-1.042172783	0.6911106
1562760_at	RP11-19D2.2	-1.042230065	0.289957827
240151_at	HOXB-AS3	-1.042232892	0.528711344
1557879_at	LOC100129175	-1.042274451	0.347501001
229754_at	TMEM44-AS1	-1.042288351	0.703185656
213972_at	RP11-79P5.2	-1.042324396	0.382987631
1558493_at	CST13P	-1.042453724	0.220135807
244604_at	RP11-250B2.3	-1.042466939	0.64856824
1554927_at	LINC00598	-1.042657734	0.360820095
1553226_at	LINC00052	-1.042715376	0.271316779
1569858_at	RP11-171A24.2	-1.042789009	0.37124045
1556796_at	KRBOX1-AS1	-1.042827075	0.399198172
227547_at	RP5-1085F17.3	-1.042840962	0.679026561
240897_at	RP11-443C10.1	-1.042870171	0.315173079
243263_at	LINC00482	-1.04289227	0.438567687
1560520_at	LOC401312	-1.042918985	0.267737869
1559324_at	USP32P2	-1.042997112	0.39454824
1563039_at	LARGE-AS1	-1.043002927	0.204861524
1568749_at	RP11-716O23.1	-1.043020726	0.373731886
237236_x_at	LINC00202-2	-1.043113282	0.308846183
1553590_at	FAM27E2	-1.043140494	0.352219219
237494_at	RP11-548M13.1	-1.043214786	0.400483568
230245_s_at	LINC00926	-1.04321626	0.647184377
1564439_a_at	MRGPRG-AS1	-1.043219463	0.255708747
1569193_at	RP11-439E19.10	-1.043243475	0.529926185
1558449_at	RP11-421E14.2	-1.043257171	0.281505686
231652_at	RP6-91H8.2	-1.043352558	0.310540208
244832_at	RP11-752L20.3	-1.043352951	0.767405424
237529_at	LINC00708	-1.043410179	0.228448699
1558011_at	LOC441081	-1.043410612	0.672922509
1557631_at	RP11-386B13.3	-1.043445438	0.201251759
241161_at	RP11-104J23.2	-1.043462851	0.472549081
1563116_at	RP11-240A16.1	-1.043477238	0.322420002
1562716_at	LOC284632	-1.043544065	0.312798838
231529_at	MEG3	-1.043644511	0.433747856
1561253_at	RP11-43N5.1	-1.043691434	0.299619302
1564491_at	SPANXA2-OT1	-1.043771251	0.186101815
229761_at	LOC440173	-1.043931276	0.438486801
216718_at	LINC00302	-1.043936467	0.231462693
1563002_at	RP11-359K18.3	-1.043998977	0.316735913
1557875_at	RP11-879D6.1	-1.04416535	0.314565896
1553484_at	LINC00477	-1.044216703	0.335648754
234866_s_at	ZNRD1-AS1	-1.044261764	0.181798825
243503_at	RP11-676J12.6	-1.044270026	0.34834865

1569418_at	LINC00682	-1.044296934	0.398969502
220399_at	LINC00115	-1.044300494	0.770316949
1557657_a_at	LINC00642	-1.044313083	0.336879257
235406_x_at	TMEM161B-AS1	-1.044514761	0.658622676
1557742_a_at	LOC100128176	-1.044570912	0.138664417
1563254_a_at	FAM170B-AS1	-1.044614218	0.161564182
1561573_at	RP11-127O4.3	-1.044710535	0.143018501
1554715_at	LINC00593	-1.044830379	0.24377778
216998_s_at	ADAM5	-1.044889775	0.422925054
1557431_at	RP4-561L24.3	-1.044897234	0.45585959
231138_at	TRPC5OS	-1.04510186	0.347807471
1569859_at	SUCLA2-AS1	-1.045137884	0.390245722
215003_at	DGCR9	-1.045160798	0.593616264
1560472_at	LINC00705	-1.04516635	0.345547462
1563127_at	RP4-764O22.2	-1.045201156	0.204592487
1570235_at	MGC27382	-1.045230844	0.223327914
220263_at	SMAD5-AS1	-1.045302647	0.314464712
239814_at	LOC100506860	-1.045326766	0.3550367
78047_s_at	MMP24-AS1	-1.045371363	0.513019004
227567_at	LINC00674	-1.04544796	0.622986754
240380_at	LOC728040	-1.045451839	0.384345807
240598_at	RP11-1151B14.3	-1.045566904	0.387148667
1559441_s_at	FLJ38576	-1.045577617	0.343807782
239455_at	RP11-396F22.1	-1.045649759	0.393496001
1564449_at	USP2-AS1	-1.045713968	0.248889261
1552605_s_at	LINC00308	-1.045759113	0.264421408
230460_at	MYLK-AS1	-1.045794845	0.302885873
208073_x_at	TTC3P1	-1.046016413	0.681931398
1563086_at	RP11-702F3.3	-1.046158515	0.298186387
1562737_at	RP11-422J15.1	-1.046167422	0.223149194
1568746_a_at	LOC646268	-1.04617627	0.276285924
224245_at	INGX	-1.046265716	0.329055749
1561562_at	RP11-303E16.7	-1.046315536	0.192469209
240523_at	DPH6-AS1	-1.046348272	0.640210236
228691_at	RP11-843B15.2	-1.046360235	0.30692615
1557590_at	PARD6G-AS1	-1.046380239	0.305025953
1561055_at	LOC100507534	-1.046480425	0.542233589
1562812_at	ZNF32-AS3	-1.046646642	0.364998159
233826_at	RP11-410E4.1	-1.046662039	0.350952444
1556558_s_at	ENTPD3-AS1	-1.046777059	0.534655753
1561340_at	RP11-257I14.1	-1.046796165	0.225246724
1559605_a_at	LOC285043	-1.046808409	0.196383847
235173_at	MBNL1-AS1	-1.046885782	0.487783939
237339_at	RP11-20F24.4	-1.046887951	0.254153994
233590_at	LOC100506457	-1.046928119	0.195928266
1561455_at	LOC284294	-1.046953833	0.328792122
224418_x_at	PMCHL1	-1.047033702	0.295237814
240902_at	LINC00641	-1.047067761	0.420175768
217494_s_at	PTENP1	-1.047153378	0.416628008
1560542_at	MCM3AP-AS1	-1.047169749	0.235705206

1562928_at	RP11-20F18.1	-1.047225629	0.273626256
1566222_at	RP11-264J4.10	-1.047321793	0.271507977
1553478_at	KIRREL3-AS3	-1.047331755	0.452886327
231344_at	RP11-102C16.3	-1.047448565	0.238341031
1566586_at	RP11-397A16.3	-1.047451209	0.212214244
242822_at	MGC39584	-1.047453399	0.40848817
242350_s_at	ST8SIA6-AS1	-1.047461665	0.362848611
239305_at	RP13-638C3.2	-1.047566789	0.16079508
234171_at	RP11-536I6.1	-1.047675298	0.294317733
1559884_at	CDKN2B-AS1	-1.047796134	0.365744441
1556994_at	RP11-1078H9.6	-1.04790964	0.289675008
222659_at	IPO11-LRRC70	-1.047961138	0.643979493
230470_at	DSCR9	-1.047967375	0.301057858
1561373_at	PACRG-AS1	-1.04804896	0.357326431
242902_at	CYMP	-1.048136315	0.268763859
227649_s_at	SRGAP2D	-1.048156591	0.559369455
210905_x_at	POU5F1P4	-1.048164488	0.393667362
1566917_at	HPYR1	-1.048193056	0.249964416
1559538_at	RP11-234B24.2	-1.048253669	0.266557642
232502_at	ENTPD1-AS1	-1.048300129	0.528090219
233075_at	HERC2P7	-1.048351389	0.287001913
239362_at	NAPA-AS1	-1.048436538	0.216919114
233321_x_at	LOC90834	-1.048476825	0.31039139
221072_at	GSN-AS1	-1.048514131	0.335039708
232822_x_at	LINC00629	-1.0485528	0.290904358
1569807_at	RP11-63B19.1	-1.048556079	0.272219474
241233_x_at	ANKRD20A11P	-1.048566566	0.613178325
213502_x_at	GUSBP11	-1.048686556	0.607827473
212700_x_at	PLEKHM1P	-1.04868784	0.247431122
1562527_at	LOC441666	-1.048723376	0.481955281
1566236_at	DGCR12	-1.048791487	0.357018859
1559298_a_at	SPATA41	-1.048861895	0.169249775
240212_at	RP11-10K16.1	-1.048919801	0.392990773
1553871_at	CSNK1G2-AS1	-1.048955186	0.241370201
1557550_at	LINC00906	-1.048957616	0.435704082
1556859_a_at	LOC285740	-1.049031097	0.209980903
239640_at	LOC401320	-1.049118792	0.59206106
1554302_s_at	LHFPL3-AS1	-1.049128751	0.379384129
1557493_x_at	LOC100240728	-1.049128751	0.379782288
1564474_at	ZBED3-AS1	-1.049143038	0.667862136
1553918_at	LINC00479	-1.049201375	0.30783556
1555868_at	LOC100507477	-1.049494411	0.523313717
236271_at	RP11-363E6.3	-1.049498542	0.687977559
240842_at	RP3-417L20.4	-1.049506015	0.315947842
240012_at	RP11-53B5.1	-1.049522758	0.259704109
1569615_at	RP11-220I1.5	-1.0495346	0.193292979
230452_at	FLJ42351	-1.049728705	0.428355357
241095_at	RP11-147C23.1	-1.049761389	0.263451846
216062_at	RP1-68D18.4	-1.04977614	0.299446959
1560463_at	LOC283788	-1.04988021	0.195691309

236854_at	LINC00494	-1.050020227	0.312696131
1563632_at	TMEM72-AS1	-1.050056856	0.293833914
1561531_at	PLCH1-AS1	-1.05011645	0.265058684
234177_at	RP1-256G22.2	-1.050250227	0.304410475
1561891_at	RP11-50C13.2	-1.05027173	0.285826692
240589_at	LINC00313	-1.05030542	0.132776732
231691_at	C3P1	-1.050315787	0.318445276
1556696_s_at	NR2F1-AS1	-1.05045503	0.641206034
238069_at	RP11-98I9.4	-1.050579016	0.729067824
1559170_at	ANKRD20A5P	-1.050581054	0.463639867
1556737_at	LINC00671	-1.050653125	0.354394865
1557402_at	RP1-265C24.8	-1.050662763	0.501792014
231613_at	RP11-322F10.2	-1.050694724	0.332905686
1560533_at	RP11-313A24.1	-1.050751643	0.150349901
233804_at	LINC00544	-1.050774304	0.2585272
1559715_at	LOC100507391	-1.050787327	0.086431717
1557779_at	RP11-346D6.6	-1.050851713	0.362660061
1562927_at	RP11-152L20.3	-1.050903296	0.192786799
1556801_at	LOC400794	-1.051010388	0.272065827
229385_s_at	TINCR	-1.051056139	0.576792586
238511_at	UBL7-AS1	-1.051167266	0.550629399
228493_at	RP3-327A19.5	-1.051229873	0.693876521
1570397_x_at	FAM66C	-1.051262723	0.656889505
207288_at	ASMTL-AS1	-1.051447978	0.265542254
221714_s_at	RRN3P1	-1.051451603	0.29851382
1568683_at	SNAI3-AS1	-1.051454969	0.441450414
1561514_at	LOC400655	-1.051493886	0.203291035
1563042_at	LOC338694	-1.051494382	0.19538412
1561102_at	RP11-756K15.2	-1.051601832	0.201831594
1557451_at	DGCR10	-1.051604862	0.351198966
244152_at	IQCJ-SCHIP1-AS1	-1.05172411	0.279141675
1561348_at	LOC339874	-1.051922484	0.312805491
1562747_at	RP11-350G8.5	-1.051941347	0.303047056
215192_at	PMS2P4	-1.051954781	0.654068936
1557022_at	RP11-179A16.1	-1.05198369	0.258906519
1552895_a_at	ANKRD30BP2	-1.052068302	0.40109761
231808_at	OIP5-AS1	-1.052133678	0.635529613
206478_at	KIAA0125	-1.052165228	0.286345005
223783_s_at	DBIL5P	-1.05226686	0.354949006
1557287_at	RP11-557H15.3	-1.052304598	0.183447304
243576_at	RP11-288L9.1	-1.052321762	0.23222519
243699_at	LOC100507006	-1.052463928	0.383461254
237047_at	FAM230C	-1.052606391	0.241286531
243820_at	LOC401463	-1.052612637	0.318965764
224195_at	TTTY12	-1.052849848	0.330673851
232318_s_at	LINC00284	-1.052858256	0.20409558
242682_at	SOS1-IT1	-1.05304532	0.475080931
1569819_at	PWRN1	-1.053069162	0.137226094
227909_at	LINC00087	-1.053133052	0.807498955
1553436_at	MUC19	-1.053325562	0.27993469

1559220_at	RP5-1157M23.2	-1.053326673	0.288779791
1562655_at	Z99756.1	-1.053393595	0.258009254
1559048_at	RP11-1055B8.6	-1.053558488	0.263384779
224490_s_at	RP11-798G7.6	-1.053584683	0.774951208
224425_x_at	ACTR3BP2	-1.053758518	0.191237828
238096_at	LOC284023	-1.053815325	0.506959248
235267_at	MAGI2-AS3	-1.053848831	0.748738588
1560378_at	GRIK1-AS2	-1.053933752	0.222824079
244829_at	LINC00518	-1.053936015	0.223866506
1569983_at	LINC00566	-1.054032835	0.238939942
1556917_a_at	RP11-2O17.2	-1.054092337	0.145354893
1553756_at	GLIS3-AS1	-1.054116837	0.354247987
1561244_at	RP11-112L7.1	-1.054149558	0.114985907
1561423_at	LINC00535	-1.054171281	0.23001074
1566235_at	DGCR12	-1.054213336	0.250168682
231105_at	RP11-399E6.1	-1.054335422	0.284612085
1561280_at	LEMD1-AS1	-1.054354705	0.156371784
1567624_at	TREML5P	-1.054488426	0.282634857
243419_at	LOC654342	-1.054494822	0.176509954
1561249_a_at	DNM1P35	-1.054507375	0.167863004
244880_at	RP11-1260E13.2	-1.054546206	0.162831692
239894_at	NEBL-AS1	-1.054718054	0.392949067
1569647_at	LOC643623	-1.054797621	0.304754114
1569646_a_at	KIRREL3-AS2	-1.054827125	0.296480063
1557046_x_at	LINC00665	-1.054837953	0.618176532
216099_at	HTR7P1	-1.054884745	0.22548383
1561333_at	RP11-753D20.4	-1.054910237	0.24840015
1560278_at	LOC221122	-1.054952501	0.184562807
221965_at	RP11-546D6.3	-1.054962297	0.637916507
240225_at	EGFLAM-AS2	-1.055012254	0.164267604
230975_at	RP11-109E10.1	-1.055021633	0.202848933
1566720_at	RPS10P7	-1.055033199	0.503726184
1554089_s_at	SBDSP1	-1.05510168	0.643440489
1559321_at	RP11-434D9.1	-1.055203775	0.215299352
1556249_a_at	EIF1B-AS1	-1.055321542	0.184879185
237942_at	SNRK-AS1	-1.055454134	0.669785047
1569416_at	RP11-499E14.1	-1.05548467	0.205255164
1559950_at	FAM66D	-1.05551312	0.469493349
1554057_at	ASH1L-AS1	-1.055515326	0.561377461
217667_at	SEC14L1P1	-1.055524452	0.619128985
1552582_at	ABCC13	-1.055608533	0.181664852
1569234_at	RP11-209D14.2	-1.055674109	0.274292283
239119_at	DNAJC3-AS1	-1.055677808	0.326829974
206808_at	HNRNPA3P1	-1.055703857	0.207468092
1560932_at	FLJ31356	-1.055868813	0.187206948
242643_x_at	RP11-177N22.3	-1.055890372	0.182634187
244707_at	RP11-272D12.1	-1.055909525	0.328591196
1552583_s_at	ABCC13	-1.055965015	0.245340012
1556887_at	NPHP3-AS1	-1.056038967	0.236182335
216908_x_at	RRN3P1	-1.056067313	0.587752881

241587_at	NAALADL2-AS3	-1.056146603	0.102882813
1568853_at	RP11-98D18.9	-1.056193711	0.390466221
222198_at	RP11-930P14.2	-1.056206264	0.127761802
1560772_a_at	RP11-285A1.1	-1.056272793	0.214557551
230439_at	RBAKDN	-1.056316842	0.25886497
1570065_at	C22orf34	-1.056374861	0.359171119
236709_at	RP11-2E17.1	-1.056445838	0.452152659
237287_at	HMGA1P4	-1.056466652	0.362107797
1562475_at	TEX41	-1.056504998	0.217269085
220695_at	RP11-81A1.7	-1.056641002	0.329572013
229363_at	LINC00920	-1.056779034	0.400228056
241249_at	PSMD5-AS1	-1.056903388	0.184771843
1562557_at	RP11-110H1.8	-1.056944119	0.189665046
206954_at	WT1-AS	-1.057143279	0.210838021
1559654_s_at	GRTP1-AS1	-1.057145409	0.4447128
1569698_s_at	RP4-777D9.2	-1.057205784	0.197065579
1562320_at	NAV2-AS5	-1.057224801	0.185934357
220900_at	RP11-549J18.1	-1.057253019	0.438506759
220664_at	SPRR2C	-1.057412083	0.292827552
1558602_a_at	TUSC7	-1.057417042	0.172738465
243164_s_at	RP11-215P8.2	-1.057600693	0.110483518
240584_at	SHANK2-AS2	-1.057690239	0.274793679
1556662_at	LOC100506142	-1.057703209	0.208256378
229209_at	SNHG10	-1.057738153	0.180321583
220209_at	PYY2	-1.057862381	0.256734913
1556970_at	RP11-85A1.3	-1.057886889	0.18659255
1556365_at	LY86-AS1	-1.057975503	0.497376095
1561723_at	RP11-6F2.5	-1.057997722	0.151862854
227919_at	UCA1	-1.058088917	0.366026124
1559952_x_at	FAM66D	-1.05809942	0.474349497
208136_s_at	ZNF205-AS1	-1.058170372	0.2719366
1564475_s_at	ZBED3-AS1	-1.058258292	0.602736839
242679_at	TTN-AS1	-1.058270192	0.582984944
236001_at	LINC00675	-1.058414857	0.214995256
1560654_at	FLJ37201	-1.058486648	0.417830847
224272_at	RACGAP1P	-1.058486847	0.116602816
1562688_at	RP11-5P15.1	-1.058558005	0.160516233
1561289_at	LOC286370	-1.058723966	0.137603222
1556494_at	RP11-236P13.1	-1.058759882	0.255223269
1570262_at	RP11-319E12.1	-1.058769394	0.200123356
1570292_at	RP11-88I21.2	-1.058783195	0.550248219
222021_x_at	SDHAP2	-1.058796417	0.404343065
236770_at	TPTE2P5	-1.058867935	0.277760786
1562543_at	RP5-865N13.1	-1.058869012	0.162343628
1559917_a_at	CBR3-AS1	-1.058890572	0.178014552
209769_s_at	SEPT5-GP1BB	-1.058961438	0.378017538
240818_at	OVCH1-AS1	-1.05898657	0.206209946
1558950_at	RP11-63D14.1	-1.059063048	0.238498684
210645_s_at	TTC3P1	-1.059131552	0.628652375
243710_at	LOC100506175	-1.05914807	0.090534507

237421_at	RP11-768F21.1	-1.059247204	0.309130039
234309_at	TTTY7B	-1.059275675	0.270085744
222279_at	HLA-F-AS1	-1.059314243	0.603265384
228291_s_at	PLK1S1	-1.059427481	0.699239894
243814_at	RP4-569M23.2	-1.059554681	0.157598056
1563524_a_at	ITPK1-AS1	-1.05955969	0.191085476
239141_at	LOC100126784	-1.05966537	0.205718161
1562581_at	LOC254028	-1.059669242	0.265888119
1561059_a_at	LINC00691	-1.059711716	0.109063555
1569816_at	RP11-523L1.2	-1.059715788	0.100938159
1555786_s_at	LINC00520	-1.059727345	0.332547932
244284_at	RP11-805I24.3	-1.059753574	0.316536601
1559471_s_at	D21S2088E	-1.059831704	0.149402716
200626_s_at	SNHG4	-1.059847095	0.61546488
1569104_a_at	RP11-708J19.1	-1.059911417	0.502924079
1562914_a_at	LINC00905	-1.059950647	0.184322333
1559645_at	LINC00184	-1.059989639	0.109432924
1559925_s_at	RP11-474P2.2	-1.060025518	0.285418937
205511_at	FLJ10038	-1.060031348	0.674990465
1568787_at	LOC100506083	-1.060040712	0.571755758
1556911_at	ALMS1-IT1	-1.060063354	0.664884163
1569339_s_at	RP4-647C14.2	-1.060107621	0.238978034
221054_s_at	TCL6	-1.060147957	0.211665688
1561532_at	DACT3-AS1	-1.060232706	0.32872014
1562984_at	RP11-406H4.1	-1.060260425	0.164405614
1562080_at	LINC00424	-1.06030492	0.047870726
240428_at	LOC285000	-1.060701986	0.181951387
1557873_at	RP11-373N22.3	-1.060780746	0.427210746
239389_at	RP11-793H13.11	-1.060834914	0.272816187
1568768_s_at	BRE-AS1	-1.060850999	0.229179599
228430_at	BOLA3-AS1	-1.060896558	0.501658774
240268_at	LOC440117	-1.060960984	0.095160462
237512_at	NAV2-AS2	-1.061009646	0.141696711
235386_at	RP11-774O3.3	-1.061020497	0.430781424
1562586_at	RP13-379O24.2	-1.061068582	0.293671999
1556754_at	LINC00856	-1.061108454	0.127903313
232863_at	ZNF890P	-1.06117731	0.195835618
232105_at	BLACAT1	-1.061199597	0.699921208
243644_at	RP11-350F4.2	-1.061201436	0.218565519
230334_at	LOC100507291	-1.061203515	0.311381902
211460_at	TTTY9B	-1.061224582	0.188859772
1569738_at	RP11-152L7.2	-1.06122962	0.257703488
237368_at	C3orf35	-1.061540851	0.098097087
242042_s_at	HOXD-AS1	-1.06154621	0.258872414
235909_at	PCBP1-AS1	-1.061604876	0.289485112
222314_x_at	EGOT	-1.061607975	0.33446046
1554382_at	LINC00656	-1.061613914	0.162206427
224419_x_at	PMCHL1	-1.061895676	0.194406451
240032_at	RP11-318A15.2	-1.061957422	0.480350986
237389_at	RP11-676J12.4	-1.061983666	0.417418123

1561572_at	RP11-143A12.3	-1.062140161	0.241743222
236905_at	RP5-1068B5.3	-1.062141081	0.4082458
1562670_at	RP11-862G15.1	-1.062188436	0.129548376
213569_at	LOC100506603	-1.06232169	0.594057228
215283_at	LINC00667	-1.062523684	0.462897292
217376_at	LOC100289473	-1.06261731	0.359969739
236788_at	RP11-67C2.2	-1.062637325	0.195799467
220561_at	IGF2-AS	-1.062707741	0.134360326
241283_at	RP11-112J1.2	-1.062724035	0.043678265
1557169_x_at	HCG11	-1.062732522	0.643396058
1561546_at	FGD5-AS1	-1.062817739	0.114963248
240937_at	RP11-17A4.3	-1.062897536	0.226473936
1559483_at	PROX1-AS1	-1.062912051	0.477956418
1562597_at	RP1-167G20.1	-1.062953554	0.060805478
232460_at	RP13-1032I1.7	-1.063056789	0.227708274
241118_at	LINC00462	-1.063080817	0.133220994
1566823_a_at	RP3-449H6.2	-1.063285759	0.232359416
1561294_a_at	LOC100128554	-1.063337372	0.100855777
1562053_at	RP11-10K16.1	-1.063714535	0.549685427
211050_x_at	LOC100288069	-1.06375619	0.28503398
1561424_at	RP11-643M14.1	-1.063772279	0.315881014
1567030_at	SH3GL1P2	-1.063902205	0.397004955
237794_at	ANKRD30BP2	-1.064059905	0.374625984
1569322_at	LINC00857	-1.064063352	0.196699249
1561127_at	ADARB2-AS1	-1.064086882	0.176307734
1561045_a_at	RP11-540K16.1	-1.064119071	0.186507989
1558832_at	SLC2A1-AS1	-1.064146471	0.1091771
236403_at	THAP7-AS1	-1.064207786	0.254976047
238641_at	TMEM51-AS1	-1.06423587	0.196066617
1560941_a_at	SACS-AS1	-1.064284401	0.066054028
1553053_at	LINC00521	-1.064419903	0.115356307
241839_at	DLG3-AS1	-1.064454207	0.295031983
235901_at	RP11-589P10.5	-1.064609642	0.665260319
231564_at	RP11-109P14.9	-1.064674634	0.241652434
1563078_at	RP3-495K2.2	-1.064705558	0.143744597
236071_at	C9orf135-AS1	-1.064712095	0.241808447
207259_at	LINC00483	-1.06477587	0.062424822
1559679_a_at	RP11-495K9.5	-1.064826191	0.0752868
242102_at	DIAPH3-AS1	-1.064835036	0.143266714
1560723_at	LOC283731	-1.064879884	0.155743852
1557235_at	RP11-495K9.3	-1.064885821	0.09743465
1562922_at	RP11-158I9.5	-1.064900042	0.107434046
1570234_at	RP11-799P8.1	-1.064907664	0.052047316
1559311_at	EHMT1-IT1	-1.06499169	0.107070621
1554318_at	LOC541473	-1.065058411	0.197227692
223768_at	SSR4P1	-1.065080358	0.265181642
1562935_at	RP11-264L1.1	-1.065203983	0.51000892
239440_at	LOC100506858	-1.065269192	0.178293535
1561103_at	RP11-378I13.1	-1.065416137	0.167564619
234141_s_at	LOC286059	-1.065474877	0.128814173

1564444_at	LOC100130264	-1.065699209	0.280134632
1553069_at	BRWD1-IT2	-1.065703184	0.131082446
1557608_a_at	LOC284080	-1.065855007	0.137481935
1566830_at	LOC440028	-1.065881006	0.133468671
1570301_at	RP11-6I2.3	-1.065881428	0.273218479
1565684_at	LOC400940	-1.06594708	0.116333647
234753_x_at	RP11-322E11.2	-1.065997496	0.531966562
240025_x_at	RP4-575N6.5	-1.066010748	0.385796262
240929_at	RBPMS-AS1	-1.066038296	0.185087905
1556743_at	RP11-159G9.5	-1.066087231	0.557726273
236929_at	LOC441242	-1.066138417	0.409926928
1562388_at	LOC285819	-1.06614169	0.099279321
240887_at	LOC100506470	-1.066220954	0.132988461
1570111_at	LINC00521	-1.066237422	0.460997484
237027_at	LSAMP-AS1	-1.066270379	0.191678591
232050_at	FLJ42627	-1.066344491	0.498868758
239569_at	FZD10-AS1	-1.066439277	0.268978145
242332_at	FENDRR	-1.06648821	0.156518616
1563781_at	LOC401320	-1.066516313	0.449501984
237313_at	C1QTNF1-AS1	-1.066720712	0.178048746
215946_x_at	IGLL3P	-1.066742994	0.603249073
1563224_at	RP11-25L3.1	-1.066746028	0.138291146
1556247_a_at	LOC100506271	-1.066774601	0.193211437
1569712_at	RP11-497K21.1	-1.067060184	0.02818168
209768_s_at	SEPT5-GP1BB	-1.067258857	0.377579441
214363_s_at	SNHG4	-1.067399925	0.472718175
208118_x_at	SLC7A5P1	-1.067415547	0.259802096
237642_at	CTBP1-AS2	-1.067469913	0.146624839
237425_at	SORCS3-AS1	-1.067517888	0.110050064
1559002_at	MORF4L2-AS1	-1.067552654	0.26604649
231497_at	ZBTB20-AS1	-1.067640147	0.193233069
1570255_s_at	ANKRD20A12P	-1.067698165	0.763886905
236886_at	LOC100049716	-1.067712524	0.207893387
1557286_at	RP11-517I3.2	-1.067784805	0.80162282
1556771_a_at	CNTFR-AS1	-1.067811937	0.191882969
239507_at	LINC00608	-1.06787807	0.204829846
231030_at	ZRANB2-AS1	-1.06803481	0.243115914
1557535_at	RP11-635L1.2	-1.068313024	0.272621058
1569996_at	ANKRD26P3	-1.068356891	0.13123056
1552564_at	NUDT9P1	-1.068370675	0.517125377
1570444_at	LOC643201	-1.068476811	0.398106809
230068_s_at	PEG3-AS1	-1.068647346	0.7320585
222304_x_at	OR7E47P	-1.068662141	0.375381518
1569554_at	RP11-544I20.2	-1.068731527	0.121277973
208662_s_at	TTC3P1	-1.068810219	0.474339764
1557177_at	LOC100506258	-1.069056799	0.202775512
1558640_a_at	GUSBP1	-1.069064471	0.38313068
224041_at	TTTY6B	-1.069110584	0.258010741
1559648_at	LINC00892	-1.06916411	0.210348769
215202_at	GUSBP11	-1.069180543	0.188464354

241047_at	LINC00340	-1.069185256	0.169469775
238308_at	RP11-343H5.6	-1.06923659	0.189878063
1560384_a_at	RP11-752D24.2	-1.069270647	0.143355565
244273_at	RP11-411B10.2	-1.069453718	0.157034833
236889_at	RP11-463O12.5	-1.06955839	0.652505121
1557491_at	LOC100240728	-1.069568946	0.28088323
236336_at	BOLA3-AS1	-1.069598944	0.561430253
1566480_x_at	RP11-1072C15.6	-1.069902231	0.509863636
234051_at	RP11-873E20.1	-1.069905657	0.377756621
1563221_at	LOC414300	-1.069952068	0.093888003
220824_at	RP11-203H19.2	-1.069987921	0.065953414
241563_at	RP11-384L8.1	-1.070070757	0.191959502
243095_at	RP11-109M19.1	-1.070159585	0.174887485
230177_at	GTF2H2B	-1.070225017	0.614196189
222080_s_at	RP1-223E5.4	-1.070241829	0.162414678
1554405_a_at	LINC00161	-1.070371537	0.056500469
240366_at	LHFPL3-AS1	-1.070407505	0.133861773
214340_at	ALOX12P2	-1.070563991	0.134377838
241972_at	ZNF674-AS1	-1.070568588	0.544028445
222081_at	RP1-223E5.4	-1.070574557	0.340632988
224040_at	TTTY5	-1.070586858	0.212719871
1557882_at	RP11-442G21.2	-1.070596456	0.308469701
236606_at	RP11-248J18.2	-1.070722355	0.420516083
207778_at	REG1P	-1.070749441	0.26675519
231540_at	LOC100130691	-1.070837841	0.365130311
222376_at	RP11-499E18.1	-1.070920904	0.518330425
242987_x_at	RP11-157P1.5	-1.071150035	0.195855682
235939_at	RP1-228H13.5	-1.071214679	0.493125447
241143_at	RP11-432J9.6	-1.071230841	0.209372867
234997_x_at	RP11-488L18.10	-1.071313248	0.569105204
221148_at	RP11-457I16.2	-1.071473499	0.074983406
1553849_at	CCDC26	-1.071596555	0.376608149
239781_at	LINC00545	-1.071709006	0.395210209
224258_at	DBIL5P2	-1.071714053	0.299426962
1568807_a_at	RBM26-AS1	-1.071940324	0.740499056
1569407_at	RP1-117O3.2	-1.071958072	0.056497836
230539_at	FAM182A	-1.072009903	0.691896913
206286_s_at	TDGF1P3	-1.072032135	0.3589342
1556958_at	RP1-168L15.6	-1.072082475	0.346960401
1556704_s_at	LOC286297	-1.072106283	0.494359384
1562219_at	LINC00951	-1.072129931	0.328233702
213775_x_at	ZNF638-IT1	-1.072484012	0.452570353
239239_at	BANCR	-1.072572031	0.328720419
1558830_at	RP3-341D10.4	-1.07268117	0.200618336
240945_at	RP1-155D22.1	-1.072798261	0.152523446
1564855_at	RP11-69H14.6	-1.072889196	0.081962871
1564244_a_at	LARGE-AS1	-1.073010695	0.035283039
237266_at	KCNIP2-AS1	-1.073146075	0.127055886
229857_s_at	KANSL1-AS1	-1.073228448	0.399082387
1563019_at	RP11-485G7.6	-1.073295745	0.14112278

224422_x_at	PMCHL2	-1.073412378	0.139276425
1556072_at	LINC00528	-1.07343108	0.076070778
231639_at	RP11-464O2.2	-1.073503566	0.187566756
205834_s_at	PART1	-1.073557413	0.229398436
239639_at	RP11-353N14.2	-1.073657431	0.546117588
1569597_at	RP11-360N9.3	-1.073701174	0.497030795
233077_at	NAV2-AS4	-1.073762737	0.166719287
1564729_at	RP11-794P6.6	-1.073775357	0.12147432
1564264_at	RP11-65J3.14	-1.073819611	0.052115567
1564027_a_at	LOC154761	-1.073997917	0.641455535
229319_at	RP3-425C14.4	-1.07400259	0.622172404
244800_x_at	RP11-167N24.3	-1.074111916	0.185524053
234822_at	RP11-319G9.4	-1.074180887	0.159846477
216479_at	RPL21P28	-1.074408954	0.185497335
239248_at	SDCBP2-AS1	-1.074423424	0.220681012
226365_at	RP11-846E15.2	-1.07453176	0.634223123
227885_at	FOXN3-AS1	-1.074536273	0.206510917
206824_at	CES1P1	-1.074602377	0.191910612
1562751_at	RP1-58B11.1	-1.074628245	0.293348109
1552856_at	LINC00311	-1.074667554	0.033648793
236753_at	LINC00689	-1.074706865	0.631065718
1557721_at	RP11-416I2.1	-1.074811767	0.403668723
230953_at	LOC100286922	-1.074822254	0.064006395
230952_at	RP11-426C22.5	-1.074922876	0.175919555
1568955_at	SRGAP2D	-1.074968533	0.513984421
1559595_at	LOC728084	-1.075048939	0.131568814
1560449_at	RP11-713M15.1	-1.075081318	0.057624491
233399_x_at	ZNF252P	-1.075255095	0.238495802
237614_at	LOC285740	-1.075307146	0.050668484
236311_at	LOH12CR2	-1.075629132	0.517869635
1552576_at	ALMS1P	-1.075659623	0.361495268
1556099_at	LINC00290	-1.075770495	0.107862892
1566204_at	RP11-392A14.8	-1.075806057	0.198999424
239656_at	LHFPL3-AS2	-1.075821275	0.172932825
230617_at	RP11-770G2.4	-1.075873657	0.144081812
242638_at	RP11-887P2.5	-1.076048718	0.327601754
1564836_at	RP11-18F14.4	-1.076082363	0.196547899
1570064_at	RP11-440I14.2	-1.076097706	0.097445197
1569585_at	RP11-254F7.1	-1.07618065	0.064214523
1561004_at	RP11-5N23.3	-1.076203312	0.331184802
1556244_s_at	LOC375196	-1.076240246	0.23755654
1561061_at	LOC729506	-1.076290116	0.05790654
220575_at	FAM106B	-1.076457052	0.595912837
1553586_at	FBXL19-AS1	-1.076592279	0.068552012
242140_at	ERVK3-1	-1.076651472	0.613174417
228267_at	RP11-245P10.8	-1.0768296	0.125118147
1553061_at	OR6W1P	-1.076839498	0.061379652
228412_at	LOC643072	-1.076890835	0.366813483
244786_at	SNHG10	-1.077008204	0.586078426
1559629_at	RP11-572C21.1	-1.077055613	0.089448365

235715_at	SPRNP1	-1.077057114	0.047351766
1560285_at	RP11-1069G10.2	-1.077100793	0.128354559
234017_at	LINC00923	-1.077143337	0.129159545
1556374_s_at	RP11-661G16.2	-1.077313795	0.162645423
1568904_at	RP11-218F4.1	-1.077330718	0.248872898
231469_at	NTRK3-AS1	-1.077725533	0.201921401
1560920_s_at	RP11-295G24.4	-1.077849062	0.081601099
235774_at	LOC100422737	-1.077872065	0.097761687
232616_at	LOC100129935	-1.078051552	0.107379223
1557131_at	SSSCA1-AS1	-1.078066619	0.377959626
243035_at	RP11-630C16.1	-1.078077381	0.115276624
1557518_a_at	RP11-715J22.6	-1.078138565	0.165415172
234627_at	FLJ21408	-1.078246077	0.235490059
237509_at	RP11-215H22.1	-1.078275729	0.630202189
1569032_at	LOC642852	-1.078406492	0.067067136
1564383_s_at	FLJ35934	-1.078437652	0.522625199
217609_at	RPL13P5	-1.078572112	0.422822785
207215_at	GSTTP1	-1.078576357	0.078003229
243762_at	CTD-2314B22.1	-1.078590903	0.079871746
236102_at	LOC100126784	-1.078690618	0.145465746
233475_at	MGC32805	-1.078749419	0.243444581
224003_at	TTTY14	-1.078817794	0.133444859
1556695_a_at	NR2F1-AS1	-1.078922346	0.493468007
1559920_a_at	CECR5-AS1	-1.078935881	0.31720778
1557874_at	GRID1-AS1	-1.07911963	0.065891085
1553130_at	LOC652276	-1.079305851	0.191689872
215861_at	RP4-724E16.2	-1.079473011	0.281473837
1556661_at	GTSE1-AS1	-1.079494605	0.119669364
1561896_at	RP11-111M22.3	-1.079542489	0.379070979
244843_x_at	RP11-495P10.6	-1.079683574	0.185068858
1556465_at	RP11-310J24.3	-1.079760815	0.135746249
1569188_s_at	XX-FW83563B9.5	-1.079880164	0.172513101
232933_at	KIAA1656	-1.080060819	0.084325434
211325_x_at	DSTNP2	-1.080208544	0.307872639
225584_at	HCG18	-1.080230599	0.285927054
1566760_at	LINC00884	-1.08037855	0.098780277
1561960_at	CSPG4P5	-1.080460887	0.112578471
236758_at	LOC100132304	-1.08047554	0.037312501
233835_at	LOC90246	-1.080528353	0.092021114
233959_at	ADCY10P1	-1.080604026	0.510338842
242914_at	RP11-307P5.2	-1.080797241	0.105280471
235921_at	RP11-554I8.2	-1.080825538	0.052792278
1569670_at	PP12613	-1.080925989	0.091391835
1563009_at	LOC284930	-1.081006168	0.133119754
236914_at	LOC100506790	-1.081186463	0.162745899
239126_at	CIRBP-AS1	-1.081318352	0.098491648
1569472_s_at	TTC3P1	-1.08137416	0.647641975
1566934_at	TYRO3P	-1.081374832	0.118763526
1556899_at	PRMT5-AS1	-1.081402065	0.121319784
215816_at	GUSBP2	-1.081417566	0.12696117

217436_x_at	HLA-J	-1.081900135	0.624119058
211718_at	MGC2889	-1.082015257	0.391346441
243873_at	RP11-383G10.5	-1.082098677	0.076969053
234445_at	ZNRD1-AS1	-1.082133225	0.362899666
1570266_x_at	ERVH-1	-1.082215044	0.100905183
1562157_at	RP5-991C6.3	-1.082235693	0.091105468
244701_at	LINC00595	-1.082245233	0.16527695
238557_at	CHKB-AS1	-1.082348119	0.265762605
1552604_at	LINC00308	-1.082377191	0.076979998
226146_at	HEIH	-1.082498317	0.110086099
234573_at	RP11-84C10.4	-1.082539566	0.155033596
241290_at	RP11-550H2.1	-1.082556776	0.072728565
1560527_at	NFE4	-1.082633283	0.053560589
240756_at	RP11-131M11.2	-1.082687629	0.1351467
215812_s_at	SLC6A10P	-1.08291685	0.49269197
1562841_at	LOC339666	-1.082974454	0.081952497
235373_at	LOC100506314	-1.083019229	0.088993822
208663_s_at	TTC3P1	-1.083179844	0.526640543
213810_s_at	AKIRIN2-AS1	-1.08319686	0.229868709
225860_at	MMP24-AS1	-1.083426862	0.383969792
228275_at	LINC00888	-1.083477289	0.456281365
1566505_at	ERVK13-1	-1.083615173	0.0822116
217115_at	MKRN7P	-1.083634073	0.068446308
243870_at	RP11-285E9.5	-1.083670242	0.066560202
1564204_at	LINC00869	-1.083729131	0.222923887
244411_at	LOC100507316	-1.08377504	0.576381591
1557037_a_at	UBAC2-AS1	-1.083937053	0.054893208
1556452_a_at	LINC00928	-1.083980317	0.043125316
243680_at	LOC100506476	-1.084096947	0.370733139
237447_at	ADORA2A-AS1	-1.084259641	0.132918946
244542_at	BCDIN3D-AS1	-1.084281269	0.162810772
1555568_at	GUSBP2	-1.084393091	0.123655685
1556453_at	LOC100506274	-1.084421135	0.178521885
1558844_at	LOC100506127	-1.084462497	0.478897388
230978_at	LINC00967	-1.084516527	0.190665426
227893_at	LINC00476	-1.084819549	0.294178984
1561436_at	RP11-453A12.1	-1.085031543	0.148293187
200624_s_at	SNHG4	-1.085080143	0.54299496
240793_at	RP11-171I2.2	-1.085124965	0.272197239
215298_at	RP11-714L20.1	-1.085154397	0.129997609
239444_at	LOC728024	-1.085177719	0.171932071
215603_x_at	GGT3P	-1.085316944	0.116835906
233679_at	LOC100506472	-1.085493704	0.144032408
1563854_s_at	LOC283045	-1.085712926	0.29639613
239882_at	RP11-557H15.4	-1.085784831	0.148881305
216935_at	LINC00302	-1.085792971	0.05298928
241090_at	RP11-304L19.4	-1.085947717	0.069217661
229870_at	LOC644656	-1.086072599	0.480176086
1564281_at	LINC00491	-1.086143893	0.226668418
243285_at	LOC283335	-1.086146573	0.109305158

210711_at	LINC00260	-1.08634041	0.647732842
216240_at	PVT1	-1.086386798	0.065506999
240069_at	RP11-10A14.4	-1.08640456	0.173750122
225332_at	OIP5-AS1	-1.08665174	0.569781661
209697_at	RP11-582J16.4	-1.086856231	0.127067151
226579_at	RP11-73M18.8	-1.087089816	0.43896831
1559817_at	LOC100288798	-1.087115165	0.122349635
243897_at	U91319.1	-1.087221177	0.193907494
239483_at	FLJ37035	-1.087334981	0.073532677
1570204_at	ZBED3-AS1	-1.08764213	0.100909434
1553642_at	C9orf163	-1.087656737	0.034164517
1559578_at	RP5-890E16.2	-1.087728421	0.24371914
1558787_a_at	RP11-797A18.4	-1.08784816	0.496549624
235186_at	LOC388692	-1.087965206	0.236434001
231447_at	LINC00851	-1.087996928	0.122232378
208346_at	PPBPP2	-1.088012647	0.059599751
217158_at	PTGER4P2-CDK2AP2P2	-1.088094725	0.036138464
1570503_at	RP11-680F20.10	-1.088126759	0.041056345
1562630_at	RP11-2O17.2	-1.088191978	0.093780508
238149_at	ZNF818P	-1.088233997	0.54878622
1559806_at	RP1-155D22.2	-1.088449733	0.050728023
1569006_at	LOC284379	-1.088641706	0.080269633
244307_s_at	RP11-38L15.3	-1.08877026	0.181645496
1564807_at	RP11-504A18.1	-1.088892287	0.547050281
230957_at	PCDHB19P	-1.088910192	0.028104241
1564277_a_at	LOC286297	-1.088930887	0.146815512
234943_at	LOC400927	-1.089216699	0.027560491
230115_at	DKFZp779M0652	-1.089305064	0.148637381
1559924_at	RP11-474P2.2	-1.089337278	0.069755212
1561446_at	RP11-626H12.3	-1.089457747	0.175626534
229157_at	PRKAG2-AS1	-1.089486332	0.577297992
236656_s_at	LOC100288911	-1.089518715	0.620964355
237591_at	LINC00173	-1.089539483	0.696830226
232420_x_at	MAN1B1-AS1	-1.089811496	0.280063957
229747_x_at	LOC146880	-1.089868768	0.147569008
228511_s_at	RP11-998D10.7	-1.089959568	0.058977438
1560856_at	ARHGAP26-AS1	-1.090064502	0.078301276
1557633_at	POM121L8P	-1.090385915	0.035621478
222244_s_at	TUG1	-1.090465215	0.366363073
1561278_at	RP11-360A18.2	-1.090518989	0.024250054
232246_at	TTC28-AS1	-1.090695528	0.069051817
233812_at	LINC00028	-1.09089702	0.075552597
235826_at	RP11-355O1.11	-1.090911691	0.244215138
235323_at	LOC100499489	-1.090989776	0.082568881
229569_at	RP1-193H18.2	-1.091100214	0.60270952
1558275_at	RP11-295D4.3	-1.09115743	0.161314726
1568785_a_at	RP11-461O7.2	-1.09126435	0.02764208
231071_at	YTHDF3-AS1	-1.091501246	0.117997182
239563_at	RP11-517B11.7	-1.091611283	0.259207149
231159_at	CXorf51B	-1.09169131	0.131319526

241354_at	ASMTL-AS1	-1.091912215	0.089692259
1561307_at	RP4-568F9.6	-1.091918735	0.125225649
243406_at	TMCO5B	-1.091941277	0.042736286
242557_at	ZNRD1-AS1	-1.092479202	0.373732459
235839_at	C22orf34	-1.092487927	0.117540882
229668_at	RP11-182J1.3	-1.092574747	0.167263242
1562739_at	LOC285593	-1.09262383	0.056666924
1557676_at	RP13-638C3.5	-1.092676866	0.091829218
1553494_at	TDH	-1.092693578	0.495865982
220660_at	LINC00474	-1.09285289	0.063657916
1560537_at	FGF13-AS1	-1.09301307	0.469027315
1556486_at	RP11-292F22.7	-1.093238279	0.034352021
1570395_a_at	FAM66C	-1.093289162	0.544872822
240648_at	RP4-613A2.1	-1.093309096	0.066156871
1552954_at	C5orf17	-1.093440426	0.337515328
241196_at	RP5-1031D4.2	-1.093536961	0.096681031
1561091_at	RP11-255G21.1	-1.093807953	0.07285875
221624_at	TCL6	-1.093863931	0.060613093
239799_at	LINC00476	-1.094005486	0.555758122
215872_at	RP6-91H8.1	-1.094173501	0.037656319
1558848_at	RP11-783K16.14	-1.094402392	0.045550559
1563680_at	LOC284950	-1.094424036	0.082293073
1561527_at	RP11-324L3.1	-1.094443846	0.043155272
220220_at	LRRC37A4P	-1.094623413	0.532230232
219840_s_at	TCL6	-1.094655247	0.049091998
239407_at	PAXBP1-AS1	-1.094868792	0.496148307
1564233_at	FLJ33534	-1.095090588	0.105930245
233525_s_at	LINC00475	-1.095251591	0.242087928
202091_at	RP11-407G23.3	-1.095540794	0.27690084
1559650_at	JAZF1-AS1	-1.095924137	0.286902105
239215_at	LOC100289495	-1.095929793	0.182073357
1563300_at	RP11-319E16.1	-1.095959333	0.078353303
222384_at	SMG7-AS1	-1.09598811	0.041091259
214381_at	LOC441601	-1.096021016	0.021087426
216188_at	MYCNOS	-1.09607207	0.144447591
1562065_at	RP11-794M8.1	-1.096079482	0.011152427
1558570_at	LOC145783	-1.096142906	0.129703384
1553420_at	SATB2-AS1	-1.096297001	0.225728968
236695_at	STK4-AS1	-1.096388605	0.100290748
233469_at	TPTEP1	-1.096522205	0.430121282
204673_at	MUC2	-1.096793089	0.018540102
228832_at	FLJ20021	-1.097011845	0.127254981
242262_x_at	RP4-598G3.1	-1.097354425	0.037220706
1560784_x_at	RP13-895J2.6	-1.097378898	0.125414217
230616_at	LAMB2P1	-1.097478634	0.373922572
1552698_at	TUBA3FP	-1.097553674	0.363298999
243344_at	RP11-109D9.4	-1.097619665	0.144169029
220703_at	IDI2-AS1	-1.097796195	0.579748979
1560181_at	LDLRAD4-AS1	-1.097848987	0.047049269
241759_at	RP11-445H22.4	-1.097869583	0.049052514

1560782_at	RP13-895J2.6	-1.098012794	0.120027211
1563082_at	LINC00486	-1.098025761	0.061593128
1552862_at	RUSC1-AS1	-1.098076764	0.375514392
221634_at	RPL23AP7	-1.098097509	0.367292942
1553449_at	LINC00304	-1.09809935	0.11766406
214850_at	SMA4	-1.098230448	0.570122189
237137_at	SCARNA2	-1.098715036	0.100483729
239602_at	BRWD1-IT2	-1.098717354	0.191864907
243426_at	LINC00667	-1.098797301	0.40010819
233174_at	LOC100287015	-1.09886912	0.22665122
210822_at	RPL13P5	-1.099002691	0.329653671
241804_at	RP11-846E15.3	-1.099005299	0.484276953
238708_at	ZNF582-AS1	-1.099043305	0.117891793
1558686_at	RP11-1021N1.2	-1.099054919	0.477203361
240015_at	PRKCQ-AS1	-1.099125078	0.289256279
1557761_s_at	LOC400794	-1.099130564	0.078089771
232828_at	NALCN-AS1	-1.099232115	0.074664969
1556404_a_at	ZNRF2P1	-1.09931357	0.22295965
223777_at	DDX11L2	-1.099430131	0.225920294
1556304_s_at	LINC00899	-1.099441252	0.282228357
1556154_a_at	SNAI3-AS1	-1.099547077	0.096661328
1554203_at	GRIK1-AS1	-1.099927161	0.031366695
206557_at	ZNF702P	-1.100308889	0.267815769
244274_at	RP1-179N16.6	-1.100769534	0.277165576
239344_at	RP11-491F9.1	-1.100781601	0.051293641
240305_at	RP11-809C18.3	-1.101592321	0.269908892
236310_at	ZNF37BP	-1.10162996	0.651649753
236164_at	FLJ10038	-1.101821705	0.498099541
242618_at	HCG18	-1.102233633	0.524375359
241625_at	LOC389834	-1.102511473	0.068030445
1570469_at	RP11-308N19.1	-1.102667647	0.313204189
1560412_at	LOC100507506	-1.102862543	0.048261411
1569378_at	LINC00942	-1.103008316	0.028676153
1558577_at	LOC148709	-1.103098154	0.088287877
222048_at	CRYBB2P1	-1.103503056	0.374019992
243101_x_at	RP1-310O13.12	-1.103632825	0.142968025
1557475_at	LINC00507	-1.103647189	0.544200762
235487_at	RP11-644F5.11	-1.103792817	0.100610317
237889_s_at	LOC100422737	-1.103934513	0.037603398
233278_at	RP11-45M22.2	-1.10406818	0.028246476
1557063_at	DICER1-AS1	-1.1045785	0.155329431
1556903_at	RP6-191P20.4	-1.104615638	0.131809291
1569235_a_at	RP11-209D14.2	-1.105085433	0.030370617
239753_at	ZNF252P	-1.105242576	0.096348991
1560589_a_at	RP11-231N3.1	-1.105974335	0.103517024
232731_x_at	RAMP2-AS1	-1.106035894	0.406856927
244074_at	RP11-629O1.2	-1.106082269	0.11612283
1557821_at	LINC00639	-1.106111145	0.310415304
1554940_a_at	LOC388882	-1.106131584	0.097508803
1561421_a_at	RP11-395B7.2	-1.106154377	0.081929426

215670_s_at	SCAND2P	-1.106249451	0.141275696
1555151_s_at	TDH	-1.106483098	0.265038231
1559194_a_at	CLEC4GP1	-1.106708018	0.091251314
220904_at	LINC00574	-1.107066096	0.201449622
239924_at	GUSBP11	-1.107189256	0.392321605
237116_at	LOC646903	-1.107254469	0.703990716
243712_at	XIST	-1.107514654	0.219576176
215725_at	DGCR11	-1.108046707	0.127830764
226458_at	RP1-39G22.7	-1.108368579	0.490558625
215542_at	RP11-298I3.3	-1.108411627	0.083245688
204132_s_at	FOXO3B	-1.108963641	0.301053629
37590_g_at	RP11-617F23.1	-1.109354063	0.522974377
1556971_a_at	RP11-85A1.3	-1.109477331	0.029541572
242491_at	RP11-974F13.6	-1.109740672	0.352914094
244543_s_at	BCDIN3D-AS1	-1.109950386	0.022417665
221979_at	TOPORS-AS1	-1.110332225	0.217687473
224489_at	RP11-798G7.6	-1.11051444	0.6284554
220719_at	RP11-197N18.8	-1.110741518	0.389223392
213378_s_at	DDX12P	-1.110904278	0.31968441
221172_at	C7orf69	-1.110978144	0.012726935
1569191_at	ZNF826P	-1.111021042	0.281520025
228612_at	RAB30-AS1	-1.111060908	0.238920386
231440_at	LINC00943	-1.111275791	0.135084079
1556334_s_at	RP11-408H20.3	-1.111481587	0.047289826
233962_at	FAM83C-AS1	-1.111509913	0.052735158
231233_at	PCAT6	-1.111652098	0.374640805
220747_at	LINC00652	-1.111869147	0.038443086
210589_s_at	GBAP1	-1.111918007	0.252870851
1557117_at	INTS6-AS1	-1.111925316	0.062372563
1567334_at	RP11-24J11.1	-1.111951203	0.022959354
1557124_at	TMEM198B	-1.112046336	0.056239261
1560390_s_at	LINC00663	-1.11216873	0.318364596
1563073_at	RP11-157D23.1	-1.112421562	0.059046687
225054_x_at	LINC00674	-1.11264136	0.051944484
225055_at	LINC00674	-1.112761766	0.20696084
205114_s_at	CCL3L1	-1.112881178	0.769959857
228601_at	HOXD-AS1	-1.113014021	0.72944306
228215_at	ADD3-AS1	-1.113168433	0.157764107
1570396_at	FAM66C	-1.113276922	0.277884167
1568615_a_at	SRD5A3-AS1	-1.113314268	0.143012994
1556505_at	LINC00605	-1.114140871	0.070568524
1558790_s_at	ZNF252P-AS1	-1.114290297	0.018847798
227332_at	PXN-AS1	-1.114524698	0.162928077
233512_at	SH3RF3-AS1	-1.11480782	0.080442723
1561309_x_at	RP4-568F9.6	-1.114928586	0.012867596
228799_at	RP11-85F14.5	-1.114940157	0.228866395
244429_at	RP11-796E2.4	-1.115069737	0.051877384
1552872_at	ASMTL-AS1	-1.115103511	0.061401814
237048_at	RP11-66N11.8	-1.115535385	0.166738225
220629_at	KCNQ1DN	-1.116277113	0.068895613

215470_at	GTF2H2B	-1.116306192	0.474664293
226210_s_at	MEG3	-1.116409015	0.764963973
230302_at	RP11-48B3.4	-1.11723297	0.48022564
1570212_a_at	GNN	-1.117334742	0.022125834
229338_at	LOC100289361	-1.11768148	0.195998385
244196_at	RP11-73M18.7	-1.117893263	0.243075455
1553357_at	LINC00889	-1.118215952	0.623727945
244741_s_at	ZNF667-AS1	-1.118692775	0.345708617
227925_at	ST3GAL4-AS1	-1.119237176	0.224365054
1561624_at	RP11-723G8.1	-1.119273753	0.027389638
217399_s_at	FOXO3B	-1.119413599	0.239946339
1558234_at	KCNJ2-AS1	-1.119494884	0.201970034
228172_at	RP11-244O19.1	-1.119928164	0.203174661
239168_at	LOC400940	-1.119963708	0.023997811
233095_at	RP11-389G6.3	-1.120123663	0.539145413
216404_at	ATXN8OS	-1.120148791	0.010040735
213657_s_at	RP11-617F23.1	-1.120331646	0.495402573
231333_at	LOC100505515	-1.12102709	0.021964289
241064_at	RP11-456O19.2	-1.1211544	0.016242804
1556797_at	RNF144A-AS1	-1.121205907	0.135708533
1561257_at	LOC286083	-1.121334082	0.022035347
222031_at	LOC389906	-1.121561197	0.101558648
243083_at	LOC100287834	-1.12166818	0.033553531
239584_at	RP11-767I20.1	-1.121679188	0.623102859
237385_at	LINC00867	-1.122194477	0.040533149
1568597_at	LOC646762	-1.122275667	0.215908978
1564426_x_at	LOC729732	-1.122536189	0.579450798
236323_at	PVRL3-AS1	-1.122584376	0.121216404
232294_at	TMEM254-AS1	-1.12296392	0.23615694
215590_x_at	ACVR2B-AS1	-1.123037319	0.010211542
1569960_at	BRD7P3	-1.123520726	0.037812464
231602_at	RP11-483H20.6	-1.1238762	0.034876437
206809_s_at	HNRNPA3P1	-1.124264736	0.212885854
231088_at	MORF4L2-AS1	-1.124277039	0.405747945
1569745_at	OSER1-AS1	-1.124738757	0.026285633
1568609_s_at	LINC00623	-1.125258758	0.289543883
1556401_a_at	RP11-547D24.1	-1.1255512	0.212333587
221944_at	FLJ42627	-1.125565044	0.233826847
1557118_a_at	INTS6-AS1	-1.125570217	0.397062741
230248_x_at	RP11-395I6.3	-1.125986464	0.423756531
1552422_at	C10orf25	-1.126234716	0.173524919
234592_at	RP11-375I20.6	-1.126606836	0.08234058
236111_at	RP11-341N2.1	-1.126964093	0.103987683
1559579_at	LINC00927	-1.127261904	0.014394217
233013_x_at	WAC-AS1	-1.127530124	0.053019658
1563853_at	LOC283045	-1.1289882	0.12140296
229640_x_at	LINC00621	-1.129339937	0.167224234
1561391_at	STAU2-AS1	-1.129553951	0.054584891
210655_s_at	FOXO3B	-1.129741873	0.356368405
1553426_at	C5orf64	-1.130728087	0.090993251

230236_at	TDRG1	-1.130781525	0.024731198
1556447_at	LINC00606	-1.130832068	0.319527254
1569805_at	RP11-1018N14.1	-1.13134095	0.022296686
1556448_a_at	LINC00606	-1.131734006	0.277344864
207582_at	PIN1P1	-1.132249393	0.028520981
211457_at	GABARAPL3	-1.132414174	0.064373095
225577_at	HCG18	-1.132730579	0.109493587
239174_at	LOC100505912	-1.132835341	0.58078596
237275_at	LINC00458	-1.132973126	0.007929768
237345_at	RP11-349E4.1	-1.133591796	0.007809134
228200_at	ZNF252P	-1.134098737	0.142515565
1559102_at	RP11-73K9.2	-1.13440401	0.27297472
1556183_at	ANKRD36BP2	-1.134482943	0.214124587
222205_x_at	FAM27B	-1.134514783	0.348268235
229187_at	LOC283788	-1.13477494	0.491398691
237111_at	LOC388942	-1.135329711	0.01721256
228593_at	MTMR9LP	-1.135333753	0.051693652
1557371_a_at	LINC00961	-1.135511002	0.055198916
230812_at	LINC00673	-1.135574633	0.117502672
206082_at	HCP5	-1.135751492	0.498542127
224444_s_at	LINC00467	-1.137397423	0.277446261
235203_at	RP11-498C9.15	-1.137404792	0.178339901
208661_s_at	TTC3P1	-1.137551232	0.230103387
1561480_a_at	RP11-632P5.1	-1.137710463	0.372262729
1561479_at	RP11-632P5.1	-1.137783411	0.496532793
237326_at	LINC00310	-1.137795305	0.169058722
1556414_at	LINC00515	-1.138561377	0.367774886
1568957_x_at	SRGAP2D	-1.138830784	0.306125385
240392_at	RP11-334C17.5	-1.139058396	0.313305054
230171_at	RP11-10L12.4	-1.139702479	0.08384724
1556846_at	RP11-1006G14.1	-1.139823387	0.016541684
242366_at	RP11-135F9.3	-1.139925327	0.1591565
237244_at	C10orf71-AS1	-1.140383376	0.002374267
1561369_at	RP1-63G5.5	-1.140421138	0.069147601
230858_at	LINC00673	-1.140531789	0.154434509
1561346_at	RP11-433M22.2	-1.141236783	0.302201128
1556789_a_at	PAXBP1-AS1	-1.141506501	0.215901451
1557215_at	LINC00648	-1.141628074	0.450090727
227664_at	FLJ37453	-1.141826049	0.09679848
219817_at	MAPKAPK5-AS1	-1.142459968	0.056917348
238529_at	RP11-196G18.23	-1.143074625	0.311865801
242692_at	GLYCTK-AS1	-1.143097405	0.391382014
213510_x_at	USP32P2	-1.143600734	0.365052926
243298_at	RP11-298I3.6	-1.144149023	0.085799338
1560010_a_at	SATB2-AS1	-1.144215164	0.007823583
49679_s_at	MMP24-AS1	-1.144226371	0.097239728
229698_at	SHANK3	-1.144352846	0.100339882
228117_at	NDUFA6-AS1	-1.144731039	0.018349347
1557038_s_at	UBAC2-AS1	-1.144889851	0.115519236
1559171_at	MGC57346	-1.145114641	0.246082767

224070_at	AKR1C6P	-1.145450193	0.002823494
225657_at	NCBP2-AS2	-1.145517639	0.132317781
238485_at	IQCH-AS1	-1.146440618	0.218238373
228925_at	ADAM1A	-1.147955798	0.355334322
232207_at	GUSBP4	-1.148043783	0.28317611
220710_at	ANP32A-IT1	-1.148385363	0.234140711
208247_at	ERC2-IT1	-1.149977731	0.243365032
229323_at	LINC00959	-1.150742273	0.288802497
1561262_at	RP11-80K21.3	-1.151479627	0.026542015
230325_at	LOC100133985	-1.152404219	0.09051324
217753_s_at	RPS26P11	-1.152592253	0.301359985
1557007_a_at	RP11-304C12.3	-1.152828066	0.007612748
1556301_at	LOC100287015	-1.152898617	0.231122122
232740_at	MCM3AP-AS1	-1.153605759	0.375512975
1554249_a_at	ZNF638-IT1	-1.154132606	0.356168196
233021_at	RBM26-AS1	-1.155142919	0.190600332
1568812_at	LOC100507140	-1.1554181	0.035078879
244740_at	ZNF667-AS1	-1.156166834	0.15484975
239242_at	SLC25A5-AS1	-1.156246148	0.186362537
243954_at	LINC00877	-1.156422742	0.158556588
230388_s_at	KANSL1-AS1	-1.15662923	0.494991878
209767_s_at	SEPT5-GP1BB	-1.157059055	0.282078224
215847_at	HERC2P3	-1.157630041	0.452689092
240409_at	TPTE2P5	-1.157725963	0.239069398
239015_at	THAP7-AS1	-1.158262765	0.090888497
1560006_a_at	LOC646762	-1.158374165	0.055406259
241624_at	LOC389834	-1.158436349	0.079407194
208664_s_at	TTC3P1	-1.158526927	0.35618568
220352_x_at	FLJ42627	-1.158614216	0.084717072
236076_at	LOC257396	-1.159144158	0.33988423
228650_at	RP5-935K16.1	-1.159256932	0.105710311
1558793_at	FRY-AS1	-1.160879243	0.123690494
1556195_a_at	RP4-798A10.7	-1.161340308	0.260537916
205510_s_at	FLJ10038	-1.161516017	0.197085329
233106_at	FRMD6-AS1	-1.161719082	0.188026475
240574_at	DNAJC3-AS1	-1.161792673	0.405531063
226457_at	RP1-39G22.7	-1.162925011	0.241343813
244308_at	RP11-38L15.3	-1.163900841	0.227592681
1559049_a_at	RP11-1055B8.6	-1.164239946	0.045116283
1557410_at	LOC100506606	-1.164319946	0.33290367
213971_s_at	SUZ12P1	-1.165734076	0.12934627
240352_at	RP11-7O11.3	-1.166318175	0.029147449
239064_at	RP11-378A13.1	-1.166867333	0.139810442
243428_at	KCNQ1OT1	-1.167046626	0.331583523
240577_at	TRAF3IP2-AS1	-1.167298061	0.078740139
1558046_x_at	LOC389906	-1.167928589	0.069250757
232632_at	RP11-797A18.5	-1.168033042	0.357078607
229829_at	LINC00526	-1.168258921	0.089204679
227715_at	MAFIP	-1.169467762	0.121295123
241671_x_at	LINC00340	-1.169518625	0.017199336

1558512_at	RP11-819C21.1	-1.169606544	0.263825938
1557443_s_at	LOC100505811	-1.16996558	0.049381517
237365_at	ELOVL2-AS1	-1.170352634	0.366735382
1561439_at	RP11-953B20.1	-1.1714254	0.014749321
221953_s_at	MMP24-AS1	-1.171813378	0.110270455
216476_at	OR7E91P	-1.171972857	0.014396822
1559163_at	INHBA-AS1	-1.172002416	0.373551648
235191_at	LINC00662	-1.173321383	0.257913228
1557107_at	SLC26A4-AS1	-1.173792143	0.445527469
228643_at	RAB30-AS1	-1.174855536	0.330703798
1563916_at	WDR11-AS1	-1.174862618	0.053167265
229689_s_at	RP13-39P12.3	-1.175323481	0.253566857
236798_at	LINC00888	-1.17543909	0.229587097
222177_s_at	SCAND2P	-1.178334246	0.014560683
1556216_s_at	RP11-45P15.4	-1.178361545	0.302372629
1556102_x_at	LOC389906	-1.17892921	0.159215571
213408_s_at	PI4KAP1	-1.179630631	0.270951202
229130_at	RP11-774O3.3	-1.179754997	0.278825862
229090_at	ZEB1-AS1	-1.18003433	0.341495972
231303_at	LINC00158	-1.180035441	0.113037097
215211_at	RRN3P1	-1.180211422	0.140900045
237450_at	LOC389332	-1.1824133	0.019063753
238727_at	RP11-384O8.1	-1.184335187	0.517219158
219731_at	ENTPD1-AS1	-1.184750939	0.021498847
1556742_at	GUSBP1	-1.184866561	0.202267366
1557133_at	LINC00632	-1.185619014	0.620053714
1555865_at	TOLLIP-AS1	-1.186899984	0.105631829
229227_at	DICER1-AS1	-1.187960171	0.133203794
1560482_at	RP11-966I7.2	-1.188039876	0.093725246
1570376_at	LINC00689	-1.188162891	0.50414486
235251_at	RP4-555D20.2	-1.188270251	0.569932155
235829_at	CAHM	-1.188481486	0.394901576
64432_at	MAPKAPK5-AS1	-1.188648025	0.055740088
239509_at	FLJ16779	-1.189889819	0.591228783
1553987_at	MAPKAPK5-AS1	-1.190079351	0.015215986
229328_at	ZNF571-AS1	-1.190221946	0.128393303
1558722_at	ZNF252P	-1.190305974	0.266934298
1557014_a_at	FAM201A	-1.190713707	0.217840541
236400_at	IDH1-AS1	-1.190891527	0.030626408
243726_at	RP11-203B7.1	-1.190980201	0.000676773
212337_at	TUG1	-1.191319005	0.085149364
208109_s_at	LINC00597	-1.191525486	0.304889785
226793_at	LINC00294	-1.192006514	0.146364325
227593_at	FLJ37453	-1.192253894	0.174205935
233310_at	RP11-143K11.1	-1.193167199	0.182485632
228972_at	PITPNA-AS1	-1.193573619	0.102946066
1565806_at	FLJ37035	-1.193906348	0.054946011
212725_s_at	TUG1	-1.193926587	0.047154392
238916_at	LINC00938	-1.195659843	0.033079465
1570281_at	RP11-604N13.1	-1.196762816	0.006251888

229480_at	MAGI2-AS3	-1.198537346	0.253818913
228889_at	ARHGAP5-AS1	-1.199179621	0.092663003
243435_at	KCNQ1OT1	-1.20013614	0.186112472
231592_at	TSIX	-1.201304877	0.480904359
238091_at	LOC100506388	-1.201688289	0.175758799
234594_at	ITPK1-AS1	-1.201889344	0.209577251
231337_at	TEX41	-1.203146467	0.273462254
233981_at	RP11-125K10.4	-1.203668507	0.063120538
232975_at	HCG18	-1.204212479	0.08487917
242364_x_at	TNRC6C-AS1	-1.204546903	0.109906249
232298_at	MBNL1-AS1	-1.205209129	0.112676563
1558167_a_at	MGC16275	-1.205698724	0.174031267
229318_at	RP11-119F19.2	-1.20856376	0.132955251
226211_at	MEG3	-1.209185853	0.583670831
232953_at	LINC00266-1	-1.211136382	0.187223807
1558166_at	MGC16275	-1.211329436	0.222531114
236716_at	RP11-731J8.2	-1.213916428	0.114325683
213013_at	LOC644172	-1.21400469	0.075641153
1557098_s_at	HAR1A	-1.214196531	0.160858058
222307_at	PDCD4-AS1	-1.216997218	0.128318393
230566_at	MORC2-AS1	-1.217384812	0.03092428
1554447_at	JPX	-1.219066495	0.254406333
1556043_a_at	TTN-AS1	-1.219994411	0.172245627
1564632_at	RP11-588G21.1	-1.221330856	0.050162539
1552665_at	JMJD1C-AS1	-1.22147876	0.015224288
228449_at	MORC2-AS1	-1.221556044	0.069522688
1556159_at	RP11-762L8.6	-1.222596892	0.205085054
240578_at	LINC00836	-1.223103987	0.15957721
214376_at	RP13-514E23.1	-1.223465273	0.409552097
1553448_at	FLJ34503	-1.224777491	0.066031998
1556147_at	RP11-16N11.2	-1.224796154	0.176753155
232001_at	PRKCQ-AS1	-1.225306677	0.149039257
1558474_at	RP11-1114A5.4	-1.226301983	0.141164721
1556992_at	LOC550113	-1.227626862	0.035106054
1560853_x_at	ZNF826P	-1.228487432	0.069518461
1556148_s_at	RP11-16N11.2	-1.228868755	0.089330399
59433_at	LOC389906	-1.228928799	0.049712126
215972_at	PART1	-1.22909355	0.284724202
235014_at	ILF3-AS1	-1.22961675	0.125555193
239325_at	DNAJC27-AS1	-1.22974495	0.018783375
1559045_at	LOC100128288	-1.231293158	0.041552205
214685_at	NOP14-AS1	-1.231519673	0.085374694
231186_at	LINC00643	-1.233904728	0.237338447
1557788_a_at	LINC00476	-1.234576396	0.015691208
228839_s_at	LOC642361	-1.235842452	0.083713271
1557542_at	RP11-384P7.7	-1.236234884	0.089318853
235207_at	RP11-1094M14.11	-1.236256027	0.03725158
238498_at	RP3-406A7.7	-1.239058608	0.266103167
230562_at	LOC100507530	-1.239491469	0.003968992
242873_at	RP11-277P12.20	-1.240382808	0.178497433

228160_at	LINC00667	-1.243861653	0.078666134
220324_at	LINC00472	-1.245797204	0.117054764
240246_at	LOC642236	-1.246163855	0.260964086
1557617_at	DCTN1-AS1	-1.247496682	0.098874226
231164_at	ABCA17P	-1.247887878	0.129211384
243261_at	LINC00689	-1.249306742	0.328426241
243553_x_at	TRAF3IP2-AS1	-1.249385951	0.002461737
1560751_at	AQP4-AS1	-1.249487122	0.119709364
243952_at	TPTEP1	-1.250213521	0.413536323
221621_at	SCARNA16	-1.256345283	0.018960461
238872_at	LOC100128239	-1.258097373	0.050708755
212182_at	NUDT4P1	-1.260543825	0.086497844
241745_at	LOC100507557	-1.261087348	0.174916693
230187_s_at	RP11-457M11.5	-1.261925736	0.193068849
239793_at	RP11-73M18.6	-1.262487904	0.038511044
240436_at	MIPEPP3	-1.26643699	0.085528545
230787_at	RP11-126K1.6	-1.270675743	0.070100628
1560425_s_at	RP11-75C9.1	-1.270981583	0.245583022
1560119_at	LINC00937	-1.271626871	0.072554286
1554143_a_at	SUGT1P3	-1.271803096	0.060297114
242208_at	ZNF37BP	-1.274070306	0.241442513
236555_at	TRAF3IP2-AS1	-1.274292833	0.015171572
239742_at	RP11-732M18.3	-1.274790299	0.140101283
205833_s_at	PART1	-1.27708381	0.00233199
239062_at	TNRC6C-AS1	-1.279286409	0.074660804
1569629_x_at	LOC389906	-1.280720854	0.146865554
228397_at	TUG1	-1.2813184	0.037393669
1557883_a_at	RP11-442G21.2	-1.286951601	0.007964217
235888_at	GUSBP1	-1.288016753	0.206501694
227330_x_at	MAFIP	-1.291100037	0.154744239
203463_s_at	EPN2-IT1	-1.291611849	0.044664171
244189_at	TTC28-AS1	-1.292582422	0.02892432
230526_at	TNRC6C-AS1	-1.305924207	0.042884537
1554741_s_at	KGFLP1	-1.316592873	0.129265043
1558045_a_at	LOC389906	-1.317925536	0.094534457
230738_at	RP11-196G18.23	-1.320185371	0.057085906
242577_at	LOC389834	-1.322611447	0.182266565
1553440_at	AQP4-AS1	-1.325791279	0.156312343
228012_at	SNHG4	-1.325941444	0.024677112
230449_x_at	RP11-410L14.2	-1.325994017	0.029364256
215358_x_at	ZNF37BP	-1.327515441	0.008188333
236902_at	LINC00643	-1.328087015	0.143007464
1556160_a_at	RP11-762L8.6	-1.329412457	0.069769104
226413_at	LINC00938	-1.331774088	0.011155509
215180_at	RP11-429B14.4	-1.332167122	0.083108157
227446_s_at	DHRS4-AS1	-1.33814901	0.021690862
227923_at	SHANK3	-1.340059733	0.125764189
241743_at	RP4-613B23.1	-1.342296714	0.012513884
1558906_a_at	OSER1-AS1	-1.343991052	0.009828273
222196_at	LOC389906	-1.344786627	0.061075154

1562953_s_at	WDFY3-AS2	-1.345239117	0.041825762
244631_at	LOC389834	-1.346911088	0.114058108
235936_at	LINC00925	-1.349791299	0.026639432
1569746_s_at	OSER1-AS1	-1.350344766	0.014021561
230356_at	RP13-238F13.5	-1.353374656	0.011193677
1562831_a_at	WDR11-AS1	-1.353656902	0.062519935
233562_at	LINC00839	-1.354338451	0.179728391
230589_at	TRAF3IP2-AS1	-1.356629931	0.173294627
1553796_at	LOC400940	-1.358249976	0.260838167
217637_at	RP4-791K14.2	-1.364024841	0.023599473
1564856_s_at	LOC727924	-1.366544402	0.022845603
1569348_at	TPTEP1	-1.37401833	0.280042844
1562389_at	RP11-9G1.3	-1.38233294	0.13727302
230307_at	SLC25A21-AS1	-1.384215652	0.096819734
1559826_a_at	LINC00960	-1.387543175	0.074303605
214623_at	FBXW4P1	-1.38988026	0.045920411
226947_at	GUSBP4	-1.390705461	0.018934744
203464_s_at	EPN2-IT1	-1.392519313	0.019124048
1569110_x_at	LOC728613	-1.393218645	0.121860593
1556314_a_at	RP11-389C8.2	-1.403195718	0.058093787
206565_x_at	SMA4	-1.404991193	0.006448666
227452_at	LINC00673	-1.407198996	0.038098736
1555892_s_at	PSMD5-AS1	-1.408995215	0.150816085
239863_at	RP11-305E6.4	-1.409556099	0.077585808
1557366_at	CCDC144B	-1.412573408	0.060666401
220459_at	MCM3AP-AS1	-1.416687252	0.061339592
1564175_at	LINC00960	-1.425844424	0.092464103
236575_at	ARHGEF26-AS1	-1.431731031	0.10066891
228376_at	GGTA1P	-1.432961515	0.124682828
206777_s_at	CRYBB2P1	-1.433672111	0.021535402
1558225_at	RP11-731J8.2	-1.435236682	0.048259489
229715_at	RP1-239B22.5	-1.43985123	0.070427422
231031_at	KGFLP2	-1.439864899	0.067249189
237885_at	SOX21-AS1	-1.444274831	0.111128963
244289_at	ZNF300P1	-1.446867053	0.184094171
224870_at	DANCR	-1.447355773	0.005376845
229748_x_at	MAFIP	-1.451160282	0.074167963
1564392_at	LINC00320	-1.455675026	0.068885999
229580_at	RP11-13L2.4	-1.456383827	0.219178877
228740_at	RP11-999E24.3	-1.468605771	0.05101143
237471_at	LINC00689	-1.484281246	0.335394919
215043_s_at	GUSBP9	-1.491478386	0.005887721
AFF.HUMRGE.M	#N/A	-1.498659105	0.034358107
1559827_at	LINC00960	-1.504694606	0.073919983
1564936_at	RP11-182J23.1	-1.51354934	0.096882069
237675_at	RP11-547I7.2	-1.52085007	0.090086049
237400_at	RP11-247L20.4	-1.524229292	0.007476715
231859_at	C14orf132	-1.529970841	0.028169139
229781_at	LOC100506725	-1.541509235	0.044298032
1570120_at	RP11-268G12.1	-1.563599004	0.211094262

230876_at	ZNF883	-1.5786939	0.031795046
217014_s_at	AZGP1P1	-1.58084155	0.041404326
218820_at	C14orf132	-1.583674113	0.025434952
229613_at	RP11-401P9.4	-1.585978821	0.076008236
230650_at	RP11-102F4.3	-1.6057136	0.246223154
1553797_a_at	LOC400940	-1.619737381	0.294398192
241401_at	WDFY3-AS2	-1.6503084	0.020713685
238081_at	WDFY3-AS2	-1.651983674	0.025694182
214120_at	RFPL1S	-1.703746869	0.145949619
1558226_a_at	RP11-731J8.2	-1.739679015	0.026783976
232833_at	RP6-201G10.2	-1.743300865	0.060694333
238360_s_at	LINC00672	-1.784330423	0.017339443
230741_at	RP11-340F14.5	-1.806502451	0.018193326
1557481_a_at	LINC00320	-1.807399882	0.035993792
226558_at	MAFIP	-1.83690181	0.052832816
239671_at	RP11-355I22.7	-1.847082512	0.027680631
1558388_a_at	UG0898H09	-1.888278803	0.051276553
238603_at	LINC00925	-1.968565962	0.007793102
1558387_at	UG0898H09	-1.982271974	0.019376898
1563367_at	MAPT-AS1	-1.985038306	0.00685486
240869_at	RP4-791K14.2	-2.002367141	0.011026718
1556904_at	RP11-731J8.2	-2.002488287	0.039334398
224589_at	XIST	-2.220479455	0.279076366
1559992_a_at	LINC00645	-2.419436201	0.003165824
214218_s_at	XIST	-2.45972015	0.252279633
221728_x_at	XIST	-2.514931544	0.253688694
227671_at	XIST	-2.742634897	0.264371864
224590_at	XIST	-2.761623574	0.224374338
237696_at	RP11-231C18.1	-2.798992159	0.004870465
230577_at	LINC00844	-2.940354956	0.010491933
224588_at	XIST	-3.302290561	0.215853516
