# Supplementary material for: The long non-coding RNA HOTAIRM1 promotes tumor aggressiveness and radiotherapy resistance in glioblastoma
Source: Cell Death Dis. 2021 Sep 28;12(10):885. doi: 10.1038/s41419-021-04146-0 (PMC8478910; doi:10.1038/s41419-021-04146-0)
Supplement: Supplementary file 13 — Supplementary Table 2 [file 41419_2021_4146_MOESM13_ESM.pdf]

Supplementary Table 2. RNA sequencing results of transient *HOTAIRM1* knockdown cells

Data supplied as Log2 Expresion (counts per million (CPM))

ID	LN-229_HOTAIRM1_KD	LN-229_control	T98G_HOTAIRM1_KD	T98G_control	U251_HOTAIRM1_KD	U251_control
7SK	0.258567	0.904172	0.292852	0.893036	0.162983	0.997336
A1BG	0.284893	0.292817	0.826729	1.00625	-0.537043	-0.0313164
A1BG-AS1	2.04126	1.82705	2.52612	2.54638	1.17327	0.49135
A2M	5.47746	4.16997	3.18932	3.29419	-0.659408	-0.963819
A4GALT	-0.323873	-0.508459	0.623914	1.25754	3.27632	3.16981
AAAS	4.27533	4.17105	3.48182	3.62249	3.55886	3.40443
AACS	4.06828	3.72203	4.35951	4.46954	3.90764	3.58791
AADAT	2.8459	3.25484	-0.723865	-0.899117	1.58765	1.33772
AAED1	2.7542	2.82948	2.39014	3.02046	2.67732	3.05151
AAGAB	4.26454	4.3605	4.45943	4.89469	4.92934	5.47629
AAK1	5.40682	5.27524	5.78694	5.78333	5.90312	5.49356
AAMDC	1.72345	1.7385	1.4137	1.7175	0.531539	0.463298
AAMP	5.26778	5.27904	5.1513	5.74448	5.86888	6.02501
AAR2	4.85072	4.80158	4.33314	4.71133	4.28071	4.44557
AARS	6.10666	6.1569	6.31457	6.87933	6.72716	6.79584
AARS2	3.87588	3.68548	3.23997	3.42563	3.55332	3.48241
AARSD1	3.28753	3.29996	2.92814	3.20899	3.24403	3.36168
AASDH	3.24783	3.18281	2.57471	2.81599	4.4945	4.97803
AASDHPPT	3.25368	3.5214	4.84706	5.21939	4.81462	5.39405
AASS	4.16356	3.80159	4.35949	4.29286	4.40105	3.97452
AATF	5.23719	5.23108	4.42288	4.80106	5.14139	5.07382
AB019441.29	4.52648	4.66211	4.54615	4.84165	5.2543	5.59255
ABALON	1.53313	1.69165	1.42058	1.70725	0.96691	1.43906
ABAT	2.60047	2.39103	-2.37543	-1.93836	-2.65941	-3.70484
ABCA1	5.02201	4.62063	3.39391	3.50036	7.2323	6.90338
ABCA11P	1.96504	2.00911	1.89689	2.00285	2.09747	2.07023
ABCA13	1.28396	1.06077	0.386425	1.03078	7.80722	7.54489
ABCA2	3.45194	3.32015	4.00011	3.82486	2.23755	2.02123
ABCA5	3.84288	4.04374	3.24484	3.27478	3.75045	4.03562
ABCA7	0.409538	-0.440295	2.41268	2.25314	2.76806	2.2933
ABCB1	1.60373	1.97427	-3.18222	-2.30078	-6.64581	?
ABCB10	4.49049	4.49342	4.17714	4.26612	3.88739	3.84943
ABCB4	1.62837	1.18401	-1.42826	-0.938639	1.1211	0.463298
ABCB6	4.06305	4.00865	5.02263	5.16561	5.36938	4.65047
ABCB7	3.54766	3.71083	3.29626	3.73509	4.4051	4.70797
ABCB8	3.5649	3.40825	4.05979	4.08386	3.21166	3.46439
ABCB9	4.03256	3.88858	1.47119	1.97443	3.47544	3.62946
ABCC1	5.26741	4.99143	7.86877	7.80319	6.15826	5.54475
ABCC10	3.02031	2.89251	3.04289	2.96532	3.77327	3.68989
ABCC2	0.289705	-0.177294	3.76338	3.56713	6.64659	6.91191
ABCC3	4.25947	4.52298	6.78242	6.66038	5.53074	5.62308

ABCC4	4.31352	4.60458	6.35996	6.47635	4.93213	5.47256
ABCC5	4.92753	4.86582	5.14739	5.04914	5.07143	4.57753
ABCD1	1.15272	1.17383	2.1019	2.3424	2.92278	2.75273
ABCD3	5.45539	5.44102	5.69414	6.0272	5.85858	6.11466
ABCD4	3.67541	3.44606	3.55507	3.39353	3.58525	2.98683
ABCE1	6.11531	6.3495	5.40179	6.02844	6.15258	6.52703
ABCF1	5.91999	5.91546	5.1795	5.46243	6.63907	6.30241
ABCF2	6.19472	6.09187	5.55835	5.97387	6.19439	6.10823
ABCF2P1	1.74485	1.97795	1.20884	1.71352	1.78078	0.96646
ABCF3	4.02287	3.8903	4.59619	4.75147	5.14743	4.9299
ABCG2	1.33988	1.8272	2.87989	3.72249	-0.470301	0.80115
ABHD10	3.5128	3.91327	3.60438	3.99051	3.30259	3.83051
ABHD11	1.91933	1.9035	2.53073	2.97864	2.41919	2.74862
ABHD12	5.27855	5.03798	4.50822	4.83807	4.59589	4.83727
ABHD13	3.99179	3.6945	3.90393	3.74136	3.96082	3.59934
ABHD14A	1.22463	1.35697	0.101166	-0.0253444	1.86129	2.34778
ABHD14B	4.10907	3.86582	3.6109	3.61227	4.41582	4.40348
ABHD15	2.66944	2.36689	2.59763	2.69843	2.71256	2.52692
ABHD16A	2.91552	2.69944	3.30626	3.65932	3.29233	3.57163
ABHD17A	2.87693	2.37906	2.38116	2.30991	3.50845	3.41618
ABHD17AP1	-0.135825	-0.314121	-0.291984	-0.525345	0.836789	0.971545
ABHD17B	3.57588	3.52583	3.12026	3.18119	3.26747	3.25618
ABHD17C	4.02883	3.88588	2.4052	2.71136	1.96907	2.11985
ABHD2	6.99244	6.95811	7.03769	7.6279	6.45999	6.3532
ABHD3	2.39105	2.65351	3.56299	4.08716	3.10124	2.77711
ABHD4	2.42764	2.63256	2.73833	3.28812	3.11448	3.2677
ABHD5	3.08638	3.32288	2.48654	3.29535	3.20232	3.98052
ABHD6	3.15423	3.12024	1.82773	2.41475	2.98715	3.50274
ABHD8	1.45513	1.35858	1.13784	1.60319	2.01028	2.17804
ABI1	3.96652	3.97886	3.56607	3.84112	4.38455	4.42514
ABI2	5.91288	6.02183	5.47171	5.54584	6.10383	5.99033
ABI3BP	0.567789	0.559609	2.6109	1.23995	4.87544	4.68774
ABL1	4.81758	4.73866	4.80799	5.08285	5.59264	4.94073
ABL2	7.24719	7.11369	5.814	6.25529	7.12917	7.01373
ABLIM1	3.78472	4.22277	-1.48269	-1.06415	6.32727	6.359
ABLIM3	2.07243	2.81793	4.50908	4.73744	2.68177	2.81293
ABR	5.95473	6.20275	4.24697	4.82512	5.2935	5.46002
ABRACL	2.60471	2.75391	2.35177	2.61228	3.5637	3.94792
ABT1	3.5429	3.16287	3.07858	3.23103	3.98354	3.95327
ABTB1	2.49569	2.48554	1.99641	1.92462	0.197777	0.293394
ABTB2	2.49322	2.50006	1.46757	1.6394	2.51712	2.33222
AC000089.3	3.25557	3.12663	2.72546	3.04609	3.48723	3.87779
AC000120.7	0.374055	0.247648	1.90956	1.61509	0.194591	-1.18844
AC000123.2	4.07075	3.88366	3.33218	3.21085	3.22539	2.71536
AC000123.3	0.672688	0.668419	-0.2205	-0.32184	-0.0822857	-0.0902315
AC002056.3	0.998087	0.860526	0.597584	1.28515	1.31205	1.28302
AC002543.2	0.965507	0.884332	1.13842	1.12005	0.597155	0.223336
AC002550.5	0.284964	0.590971	-0.171409	0.363283	0.410211	0.889567
AC004057.1	5.4584	5.4341	4.6911	5.25672	5.32964	6.0517
AC004076.5	0.388476	0.107125	0.259532	0.0612232	0.83161	0.0213376
AC004381.6	1.53356	1.72808	2.36267	2.63217	1.4321	1.77612
AC004453.8	5.95397	5.87201	5.04211	5.39558	6.79839	7.25129

AC004623.3	0.317964	-0.0108552	0.356662	-0.127017	0.907244	0.0256525
AC004893.11	2.23478	2.29978	2.18876	2.06088	1.49955	0.668981
AC004951.6	1.83533	1.87866	2.70333	2.28554	1.96302	0.798192
AC004980.7	1.93854	2.02005	2.7862	2.49516	2.39432	1.56553
AC004987.9	0.29935	0.0249295	1.56203	1.48841	0.978159	0.42678
AC005042.4	2.21435	1.94725	0.900807	0.933157	1.0969	-0.0112695
AC005062.2	4.54791	4.6111	4.31375	4.67479	4.87496	5.64562
AC005154.6	3.716	3.55778	5.03459	4.92092	4.03557	3.47735
AC005251.3	3.93478	3.99336	3.54326	3.96222	4.55193	5.61632
AC005253.2	0.955015	0.890898	0.445462	0.581583	1.62165	0.904758
AC005255.3	1.37228	1.45183	1.52722	2.05244	2.35046	3.01361
AC005336.4	-3.6888	-2.37465	2.11571	2.02048	-2.26731	-2.00571
AC005523.2	4.51731	4.27072	3.05477	3.26167	5.14415	4.70797
AC005562.1	0.812851	0.512894	0.458655	1.3378	1.25787	1.36151
AC005682.5	-0.744656	-1.14061	0.986462	0.845438	-0.615709	-0.619042
AC005786.7	3.79497	3.55926	0.305865	0.598184	2.05756	1.74789
AC005795.1	4.03858	4.21621	3.58327	4.02719	4.17306	4.29107
AC005822.1	2.63891	2.80274	2.29281	2.3618	2.43234	2.44777
AC006026.9	1.17511	1.85205	0.805705	1.55025	1.57106	1.5485
AC006116.22	0.613362	0.705031	0.344283	0.993711	1.16111	1.12812
AC006128.2	0.213988	-0.13796	0.91947	0.51663	1.09569	0.0991875
AC006262.5	-6.68418	?	1.41643	2.68234	-3.6585	-1.79933
AC006486.10	0.54893	0.559609	-0.598354	0.158078	0.185296	0.270672
AC006978.6	1.26675	1.19542	1.46037	1.39901	1.37315	1.2122
AC007038.7	1.30097	1.48178	0.956086	0.605504	1.76178	1.48315
AC007041.2	1.00522	1.08044	0.342361	1.11667	0.203477	0.378012
AC007099.1	0.108397	0.0291348	1.06446	1.06115	0.679613	0.863209
AC007191.4	1.67599	1.84446	1.44818	1.53507	1.85535	0.583452
AC007192.4	1.62039	1.27813	1.87462	1.72115	2.67039	1.23469
AC007229.3	-0.334318	-0.200066	0.369695	0.952102	0.388212	1.86218
AC007238.1	4.80201	4.85167	4.53266	4.75101	4.97699	4.96692
AC007246.3	-0.744656	-0.531913	0.401543	0.414828	0.732078	1.13883
AC007256.5	1.34676	1.54185	1.52533	1.71423	1.70133	1.75914
AC007318.5	4.84351	4.86264	4.28861	4.89436	5.49346	5.94882
AC007362.3	-0.549622	-0.0702529	-1.49199	-0.817364	2.47358	2.08857
AC007560.1	0.650149	0.289787	0.891061	0.85884	1.50759	1.67497
AC007620.3	0.982174	0.203289	0.626159	1.27443	1.27124	1.56813
AC007969.5	4.42722	4.42908	3.68956	4.39586	5.61537	5.9105
AC008079.10	0.40353	0.370321	1.15105	0.967715	0.553999	-0.807717
AC008427.2	0.187109	0.371503	0.787407	1.14245	1.24856	1.07848
AC008993.2	1.71458	1.59247	1.7234	1.6394	1.63434	1.25912
AC009120.6	1.75072	1.77889	2.26316	2.19647	2.03885	1.81073
AC009133.23	1.42691	1.36716	0.889232	0.80393	-0.263132	0.328951
AC009237.8	-6.68418	?	1.89332	1.75539	3.52425	3.32579
AC009245.3	2.82534	2.76839	1.86585	2.14556	2.90617	2.93924
AC009299.5	2.79893	2.75475	2.35672	2.34672	3.54047	3.26716
AC009302.2	2.0207	2.02591	0.551039	0.915834	2.16261	2.46173
AC009362.2	-0.509475	-0.311958	-0.224218	0.156086	0.640177	1.23101
AC009403.2	0.66732	0.676782	0.882635	1.02051	0.509706	0.126299
AC009404.2	0.470938	0.223769	-0.825729	-0.860651	1.91336	1.17598
AC009487.6	5.77746	5.82738	5.40059	5.61391	5.70992	5.58794
AC009501.4	0.742774	0.417142	0.107313	-0.189343	0.618568	0.00852992

AC009505.2	1.09796	1.27079	-0.0439716	0.266565	-3.97771	-4.70296
AC009506.2	1.9529	2.24464	1.74517	2.50498	2.72325	3.07671
AC009948.5	2.33711	2.47968	4.05846	4.19152	2.44561	2.39133
AC009948.7	1.21734	1.50884	0.855392	1.12433	1.74741	1.077
AC010226.4	0.0920856	0.0533477	0.534261	-0.183051	-0.0565422	-0.338265
AC010240.2	0.334442	0.251507	0.996447	0.736735	0.405873	-0.421131
AC010468.1	1.82106	1.88567	1.57878	2.10524	1.91977	3.60676
AC010642.1	2.98875	2.76009	2.58688	2.55118	3.35868	2.84846
AC010761.8	3.23689	2.98227	2.2049	2.3233	3.14562	2.64364
AC010878.3	0.651059	1.0412	-0.138184	0.587346	0.7689	0.759241
AC010976.2	0.941805	0.672335	0.63863	0.586078	0.718763	0.026885
AC011290.5	1.00971	1.23841	0.371169	0.563685	1.10129	0.879212
AC011330.5	1.58697	0.883347	1.01257	0.956808	1.08776	0.852319
AC011737.2	5.85648	5.82967	5.1596	5.54785	6.71574	7.45185
AC012065.1	1.04409	1.05943	1.82178	1.90482	2.11325	2.66667
AC012066.1	1.55896	1.60891	0.931017	0.815502	1.95674	0.74373
AC012146.7	1.60996	1.51975	0.171489	0.656698	1.25975	2.44842
AC012314.8	1.06758	1.02952	1.1593	1.37019	1.50213	0.968695
AC012354.8	1.11563	1.33161	0.772443	0.82198	1.36764	1.46719
AC012358.4	0.334442	0.248268	0.371169	0.521925	0.214809	-0.848487
AC012358.8	0.479624	0.441462	1.26168	1.13538	0.409798	-0.778435
AC012360.6	0.80878	0.794611	1.40897	1.61913	-0.00196773	-0.348991
AC012512.1	3.02884	3.34612	-3.37468	-2.29753	-0.0752099	0.293394
AC012531.25	0.973406	1.20254	0.816542	0.857026	0.222446	0.0483059
AC012613.2	-0.998365	0.370142	2.2131	1.94648	-0.393347	-1.24695
AC013470.6	0.731279	1.27893	0.23008	0.28948	1.33094	1.26809
AC015849.14	0.721994	0.86462	-0.309331	0.188831	0.646651	1.62916
AC015849.19	-0.026521	0.0450795	0.610974	0.399062	0.539452	-1.06257
AC015971.2	0.39157	0.517588	0.299514	0.224818	0.067501	0.0580255
AC016292.3	1.54299	1.42229	1.04089	1.36947	1.39473	1.96155
AC016549.1	1.67164	1.69706	2.09727	2.10551	1.98357	1.79316
AC016691.2	-0.530981	-0.466467	-0.148588	-0.296194	0.678405	0.573568
AC016722.4	1.11742	0.748684	1.17398	1.28558	0.923925	0.0352579
AC016732.2	0.0831385	0.357428	-0.191373	0.538647	0.821016	1.20023
AC016734.2	4.45702	4.44937	4.13285	4.58404	5.4432	6.02859
AC016738.4	2.00783	1.6033	-1.48166	-1.87061	-1.78745	-2.10446
AC016739.2	5.49633	5.46899	4.86424	5.14507	5.73994	6.14173
AC016747.3	2.42505	2.36372	2.32429	2.56714	2.27044	2.5787
AC016831.7	2.45322	2.23414	2.24276	2.45724	1.70604	0.832509
AC017002.1	0.52663	0.650562	1.55487	1.40544	0.323762	-0.706491
AC017002.2	1.85546	1.55462	2.50068	2.44476	1.62938	0.687079
AC018463.4	1.24396	1.02411	0.960215	1.13058	1.00101	0.934562
AC018804.7	2.49267	2.62564	1.55168	2.21205	3.09035	3.06378
AC019097.7	2.48384	2.64374	1.9248	2.32704	2.39314	2.34778
AC020951.1	1.15895	0.90354	0.488988	0.0612232	-2.20023	-3.38329
AC021016.7	1.03163	1.17255	0.341473	0.975014	1.45537	1.4384
AC021224.1	4.48747	4.42176	3.11916	3.27756	3.78889	3.89195
AC022007.5	0.503125	0.20969	1.05436	1.27736	0.624559	0.0202853
AC022182.2	1.839	1.93239	3.25448	3.5042	2.59553	2.10856
AC024560.3	2.49785	2.7545	3.44266	3.6092	3.29829	2.87488
AC025048.1	2.49566	2.4896	1.58869	1.52731	1.92835	2.20877
AC025165.8	-0.630575	-0.746277	0.998932	0.697489	-0.422	-1.09453

AC025171.1	0.918951	0.960601	2.32258	2.0561	1.17631	1.02127
AC025335.1	0.952035	1.05291	-0.0134653	0.414828	0.757612	0.33778
AC026271.5	4.30995	4.28116	4.3127	4.63265	4.89116	5.46318
AC027601.1	0.524655	-0.0938436	-0.0679464	-0.0517057	0.0296578	-0.322209
AC027612.1	1.07276	0.795202	0.65678	0.506622	0.760809	-0.117958
AC027612.6	1.69199	1.58009	2.05009	2.25216	-6.64581 ?	
AC058791.1	4.78775	4.6332	4.71017	4.92114	3.82954	3.9135
AC062029.1	-0.175369	-0.105152	0.770744	0.445854	0.448309	0.151378
AC063976.7	0.356302	0.645757	1.46757	1.52057	0.86332	-0.121607
AC064850.4	2.30822	2.82122	2.2995	2.74215	2.43874	2.47715
AC068491.3	0.803293	0.681152	1.10717	1.19997	1.10956	0.998152
AC068580.6	0.481785	0.377035	1.42227	1.73628	1.41076	1.47775
AC073052.1	0.255223	0.195474	0.623914	0.685662	0.258664	0.176042
AC073063.10	1.6049	1.79844	1.55781	1.781	1.70231	2.25646
AC073869.1	1.03407	0.943914	0.128861	0.891684	0.643348	0.134773
AC073869.20	1.87859	1.87091	0.681404	1.47929	2.27533	2.37665
AC074117.10	1.746	1.43659	1.30543	1.50616	1.59568	0.42163
AC074212.5	0.262084	-0.145419	0.118493	-0.0772952	0.18334	-0.372537
AC074286.1	0.215989	0.449197	2.10432	2.40318	2.144	1.93975
AC078899.1	2.50206	2.57402	2.64818	2.88706	3.27076	3.42172
AC079250.1	0.701939	0.399959	0.120896	0.623052	1.50737	1.64325
AC079922.3	1.03569	1.2237	-0.723865	-0.108685	-1.30241	-1.18284
AC083843.1	3.77867	3.64044	2.78803	2.79506	3.48052	2.89692
AC083843.4	0.980457	1.03706	-0.935332	-0.616766	1.04711	0.176042
AC083873.4	3.06543	2.74515	2.96874	3.09229	3.19136	3.0596
AC083884.8	1.43673	1.5189	2.29291	2.25223	1.80904	0.659578
AC083899.3	1.13194	1.38221	0.881234	0.507353	1.6854	1.59597
AC084082.3	-0.191134	-0.0876659	0.82777	1.07114	0.0122397	0.0747657
AC084219.2	1.94014	1.58112	1.02168	1.1007	1.89295	2.42204
AC084219.3	0.593947	0.491925	1.06507	1.13375	0.913332	0.216834
AC084219.4	1.13792	1.40795	-0.113992	0.127752	1.1765	1.11699
AC090945.1	0.599051	1.22152	-0.0132294	0.265929	-0.418253	-0.536593
AC091492.2	0.18716	0.151898	0.113767	0.66123	0.465118	0.377202
AC091729.9	0.529821	0.251507	1.41643	1.45345	0.0946955	-0.577229
AC092066.1	0.470938	0.635262	1.14744	1.77994	1.57813	1.62424
AC092168.2	1.76447	1.71211	-1.93506	-2.02079	-2.48959	-5.69922
AC092171.4	2.31774	2.11082	2.49948	2.45343	2.63891	2.25909
AC092641.2	-0.51033	-0.518536	-0.0370098	0.176693	0.181611	0.455649
AC092835.2	2.12112	2.3218	0.636738	0.549472	-0.777433	-0.31423
AC092881.1	2.96981	2.83408	2.61415	2.54224	1.9581	1.83247
AC093106.5	0.414828	0.625327	0.179269	0.406787	0.968946	1.23171
AC093323.3	2.8641	3.07459	2.58431	2.98093	2.9883	3.30464
AC093495.4	1.12091	1.00615	0.393526	0.785274	0.101274	0.295182
AC093616.4	1.31519	1.13994	2.12428	1.99252	2.15863	2.05839
AC093673.5	1.47089	1.33161	0.723445	1.0909	0.924716	0.247587
AC093690.1	0.700164	0.603701	0.65183	0.225361	0.673837	0.100762
AC093724.2	2.80325	2.90416	2.32535	2.80899	2.87349	2.81092
AC093838.4	2.84591	2.71492	2.84868	2.9903	2.40275	1.6142
AC096664.1	-0.356894	-0.0512706	-0.533002	0.32217	0.641934	1.16719
AC097711.1	0.144086	0.332175	0.107273	0.805581	1.0064	0.778276
AC098614.2	3.28229	3.48725	3.4922	3.80072	4.39914	4.28919
AC104297.1	1.34384	1.3653	0.468333	1.16049	1.77645	2.56203

AC104651.2	0.806794	0.881649	0.0948846	0.76623	0.989502	1.23311
AC107081.5	0.0368057	0.137399	-0.195072	-0.165577	-0.0659727	0.429343
AC108142.1	1.90294	1.62484	1.3166	1.14362	3.40179	3.10065
AC108479.2	0.109922	0.0353283	0.0446179	-0.0183724	0.450538	0.656808
AC108488.4	0.0742451	-0.114677	-0.185007	-0.436244	0.384072	0.188806
AC113404.1	4.97015	5.32068	4.34583	5.00968	5.15209	5.83788
AC114271.2	-0.0517924	-0.271252	-0.157105	0.548723	0.648438	0.372462
AC114776.1	0.244911	0.417088	0.14479	0.483241	1.05068	1.4979
AC115617.2	0.254461	0.456112	0.287283	0.758499	-0.114135	-0.0819817
AC116366.6	-0.592406	-0.559222	2.16183	2.18305	-0.124341	-0.888659
AC125232.1	2.07253	1.85539	1.05148	1.20804	1.96249	1.06919
AC125238.2	0.857973	1.1834	0.712842	1.17856	1.40814	1.04469
AC127904.2	0.0152977	-0.00328298	0.976408	0.924662	0.969108	-0.662104
AC133644.2	1.76736	1.64123	2.71721	2.80772	1.58667	0.669009
AC133644.3	-0.359536	-0.18573	0.389071	0.0312964	0.486992	0.0201857
AC137932.4	0.858275	0.929261	0.655828	0.799212	0.415164	-0.318668
AC138969.4	3.0515	2.56525	3.82365	3.56857	3.1025	2.38015
AC141586.5	1.70757	1.62468	1.99206	1.67288	1.68816	0.787449
AC142472.6	0.54893	0.237698	0.584741	0.505972	0.0122397	-0.421131
AC144530.1	1.57593	1.81336	0.839734	1.43007	1.53555	1.45684
AC144652.1	0.952035	0.514521	-0.428455	-0.0642959	-0.184426	0.176042
AC145676.2	1.62288	1.51447	1.25116	1.8512	-0.250761	-0.0627204
AC159540.1	0.874703	0.83779	0.780562	0.590636	2.7831	2.37849
AC241585.2	1.42347	0.948825	-0.247207	-0.36274	-2.85453	-1.34299
AC245100.1	1.36883	0.777123	-0.548134	-0.161558	-0.048824	-0.736312
AC253572.1	1.65502	1.54645	1.24331	1.17312	0.505149	0.257687
ACAA1	3.92314	3.6997	3.39454	3.71168	4.45429	4.40502
ACAA2	3.66073	3.41675	3.61302	3.85731	2.99175	3.14399
ACACA	6.75343	6.4931	6.8542	6.93639	7.21188	6.83871
ACACB	1.47089	1.53154	1.7707	1.77994	1.1471	0.937201
ACAD10	3.27677	3.17559	3.843	3.77991	3.21928	2.81293
ACAD11	3.01901	3.03937	2.74695	2.98933	3.20217	2.76708
ACAD8	3.20631	3.13335	3.65725	3.95591	4.11858	4.26913
ACAD9	3.98463	4.08226	4.23076	4.65299	3.66108	3.81337
ACADM	4.40813	4.72326	4.32625	4.84317	5.1157	5.84451
ACADS	0.840145	0.20969	-0.455418	-0.201784	0.197777	0.769095
ACADSB	3.18966	3.13335	3.59456	3.97065	4.23829	4.07629
ACADVL	6.13478	6.20425	6.71677	6.83019	6.67869	6.39394
ACAP1	0.207918	0.251507	1.39395	1.35065	0.539452	-0.183001
ACAP2	4.9609	5.42209	6.24667	6.62035	6.03883	6.3634
ACAP3	2.62022	2.08469	3.61899	3.47905	3.29315	3.07535
ACAT1	2.58633	2.37006	4.39096	4.85475	4.4657	4.87166
ACAT2	4.75739	4.86117	4.48957	5.12392	5.37651	5.94587
ACBD3	4.52688	4.6957	4.89123	5.36061	5.41061	5.46314
ACBD4	1.29532	0.868134	1.55122	1.53507	1.27048	0.97985
ACBD5	3.96002	3.98319	3.50298	3.9342	5.20078	5.18658
ACBD6	4.50486	4.42725	4.03634	4.19642	4.38187	4.27866
ACCS	1.97337	1.78511	2.24695	2.02048	0.0677351	-0.848487
ACD	2.30937	2.30866	2.27592	2.74916	3.07947	3.37011
ACER3	5.14247	5.23628	4.59372	4.8663	3.82048	3.87448
ACIN1	6.31797	6.28105	5.99174	6.14768	6.39359	5.75288
ACKR3	?	-6.45019	1.83887	1.50592	0.815493	0.483199

ACLY	7.2152	7.08188	7.14922	7.52064	7.96373	8.07732
ACO1	5.47049	5.38261	4.70443	5.39944	5.23146	5.25328
ACO2	4.24953	4.29271	5.2165	5.63857	5.01748	5.15175
ACOT11	0.807915	1.10522	-0.263444	0.370231	-0.682812	-0.421131
ACOT13	3.15933	3.24814	3.78518	4.11298	3.75934	4.10403
ACOT2	2.2877	2.31348	1.24069	1.77151	2.79912	3.06235
ACOT7	3.42367	3.3917	4.71411	5.23324	4.38871	4.87537
ACOT8	3.5735	3.62216	3.49965	3.78389	3.23354	3.42125
ACOT9	3.2287	3.14634	3.88793	4.34443	3.35092	3.34585
ACOX1	5.14093	4.83406	5.15166	5.11577	5.92896	5.61806
ACOX2	1.88966	1.53154	-0.568612	0.0612232	-0.777433	-0.214706
ACOX3	2.54411	2.39826	3.07858	3.377	3.20851	3.18811
ACP1	5.15308	5.20436	4.29424	4.87772	5.52944	6.03087
ACP2	3.82829	3.6018	3.92228	4.10154	4.46178	4.39521
ACP6	5.2014	5.1338	3.41453	3.56183	4.50208	4.86028
ACPP	0.800836	0.746683	0.882627	0.266325	?	-5.69922
ACRC	1.32887	1.2583	-1.48269	-0.979274	1.76178	1.53173
ACSBG1	0.0966802	-0.117457	-0.326881	-0.191134	0.610983	0.669036
ACSF2	1.72302	1.60328	4.11224	4.26076	1.43776	1.76092
ACSF3	3.72437	3.46492	3.54242	3.62897	2.92674	2.51134
ACSL1	5.23937	5.26594	4.07032	4.83147	2.34813	2.45821
ACSL3	7.98594	8.00208	7.45337	7.55443	7.65498	7.5024
ACSL4	5.11029	5.34047	6.73464	7.37633	7.25038	7.95372
ACSL5	-1.47325	-1.2146	3.81305	4.44817	-0.935975	-0.662104
ACSS1	5.63268	5.45862	2.25944	2.59509	2.21007	2.69844
ACSS2	4.47333	4.55466	3.93801	4.15796	2.64576	2.57407
ACSS3	-4.30714	-4.45968	-3.25397	?	3.11587	3.13147
ACTA2	2.3906	1.90917	0.507475	0.393405	-0.311021	-0.882653
ACTB	9.98583	9.96888	10.3495	10.5194	10.9942	10.893
ACTBL2	4.02201	4.34932	-3.59683	-3.52222	?	-5.69922
ACTBP11	-0.242144	0.0916254	0.132879	0.859477	0.994558	0.758721
ACTBP2	3.48567	3.63959	3.9828	4.06503	4.54668	4.45763
ACTBP7	1.38843	1.045	-3.85951	-2.52058	1.08037	-0.799586
ACTG1	9.46062	9.57086	9.24428	9.63883	10.805	11.0093
ACTG1P1	-0.489437	-0.122851	-0.568612	-0.327299	0.659934	0.863209
ACTG1P14	0.663099	0.862844	1.08608	1.46866	1.85109	1.33775
ACTG1P18	1.67599	1.35738	-6.17309	-3.78492	0.0402612	-0.247123
ACTG1P20	1.19257	1.44276	0.749328	1.25717	2.09019	2.06472
ACTG2	-5.69162	-5.45651	-3.01245	-2.30078	2.61582	2.48806
ACTL10	0.586404	0.357428	0.580888	0.640741	-0.320293	-1.18284
ACTL6A	4.59758	4.71184	4.46795	4.86591	5.16652	5.61439
ACTN1	7.36201	7.40875	6.25045	6.53524	7.02459	6.75757
ACTN4	7.59766	7.48221	6.65403	7.08826	8.61228	8.57701
ACTN4P1	2.22813	2.22052	1.41548	2.01598	3.4034	2.96667
ACTR10	3.80871	3.91219	3.07858	3.50588	3.87204	4.60729
ACTR1A	5.70764	5.54333	5.96304	6.20457	6.06649	5.98587
ACTR1B	3.80176	3.60458	3.53415	3.61227	3.26302	3.30451
ACTR2	6.60621	6.69943	6.66259	6.9835	7.42899	7.39548
ACTR3	6.8328	6.96804	6.83354	7.30499	7.5533	7.84361
ACTR3B	2.9506	2.84071	2.30669	2.03547	2.35384	2.07867
ACTR3P2	0.972274	1.32259	1.07737	1.38608	1.98545	2.04525
ACTR5	3.03733	3.25826	2.11571	2.47239	2.49199	2.60164

ACTR6	3.71138	3.79454	2.68377	2.88553	3.27632	4.31009
ACTR8	4.21456	4.2987	3.5697	3.99315	4.28367	4.42771
ACVR1	3.91654	3.82487	3.8595	3.94782	3.75858	4.03138
ACVR1B	3.60241	3.57882	3.72336	3.84246	2.59236	2.48312
ACVR2A	3.81348	3.85288	4.14695	3.79839	4.35771	4.30961
ACVR2B	5.40715	5.68308	3.70065	3.77382	4.28585	4.57057
ACVRL1	-6.68418 ?		4.63424	4.56005 ?		-5.69922
ACY1	3.32099	3.15738	2.27636	2.32191	2.53743	3.1165
ACYP1	1.43538	1.60865	1.47492	1.90801	2.51073	2.28766
ACYP2	-0.891472	-0.707737	0.27602	0.463758	0.871126	1.27877
ADA	1.76038	1.95081	0.759062	1.07114	1.17894	1.92993
ADAL	3.31355	3.04495	3.07151	3.1055	3.56851	3.65523
ADAM10	7.24477	7.16375	6.80303	7.0425	6.62635	6.80873
ADAM12	4.02543	4.08886	4.01246	4.83222	7.9408	8.35258
ADAM15	5.94681	5.75844	5.26973	5.49293	5.09581	5.38339
ADAM17	5.83795	5.90433	5.74501	6.02833	6.84832	6.84583
ADAM19	5.54945	5.73742	4.31831	4.47707	7.60747	7.44147
ADAM22	3.75829	4.11458	3.92891	4.01659	3.30692	3.78112
ADAM23	3.81957	4.04593	3.27591	3.4686	2.64804	2.76903
ADAM32	1.12752	0.775632	0.503064	0.724135	2.24057	1.92267
ADAM9	7.48866	7.37971	7.7829	8.23126	7.64009	7.51242
ADAMTS1	5.07445	4.32016	5.50253	4.65769	2.64119	1.76092
ADAMTS10	-2.9975	-4.13839	0.849967	0.414828	1.04018	0.443129
ADAMTS12	4.31006	4.71829	6.07268	6.12775	4.75595	5.33632
ADAMTS15	3.24637	3.40444	1.47473	1.54226	5.04315	4.39263
ADAMTS16	3.03054	2.94655	1.45314	1.98395	4.05823	3.55773
ADAMTS17	1.67305	1.27821	-3.37468	-3.94057 ?	?	
ADAMTS3	3.67377	3.30037	1.22592	1.15801	-1.80184	-0.95156
ADAMTS4	-0.768111	-1.55554	-1.86109	-2.10822	4.71472	3.81292
ADAMTS5	-3.8896	-4.45968	3.60274	1.42259	-0.302594	-1.70626
ADAMTS6	1.98744	2.49427	1.35568	1.59857	1.82755	2.18812
ADAMTS7	1.19161	1.04266	2.2498	1.62812	2.3236	1.44896
ADAMTS9	6.01529	5.88542	4.31323	3.46193	0.670133	-0.214706
ADAMTS9-AS1	2.02004	1.97018	1.16605	-0.074789	-5.04123	-4.70296
ADAMTSL1	-4.69535	-4.45968	-0.117291	0.879925	6.28514	6.39559
ADAMTSL4	0.670378	-0.021346	0.549502	0.744376	-0.181419	-1.85459
ADAMTSL4-AS1	1.30462	1.33872	0.754725	0.624466	0.256443	-0.400427
ADAR	7.77178	7.7248	7.57767	7.73774	7.89126	7.47101
ADARB1	5.49318	5.12211	4.18061	4.91976	4.56429	4.36206
ADAT1	4.20329	4.14077	4.10764	4.47142	3.89331	4.20017
ADAT2	2.29529	2.41061	2.03082	2.15798	3.19923	2.82467
ADCK1	1.03569	0.525929	-0.161008	-0.154483	1.04018	1.08775
ADCK2	4.04766	3.89879	2.78327	2.78058	3.47181	3.77174
ADCK3	4.3697	4.20071	2.93801	3.04093	3.62046	3.82271
ADCK4	3.09696	2.77557	3.22375	3.23547	3.01389	2.98678
ADCK5	0.520176	-0.036446	1.12031	1.06115	-0.267833	-0.38461
ADCY1	2.10184	1.64571	2.42382	2.18581	-1.80184	-2.12114
ADCY10P1	1.00142	1.38894	-1.18321	-2.93781	1.25263	0.719622
ADCY3	5.47395	5.33454	4.52864	4.58643	4.63664	4.58511
ADCY6	4.65955	4.63255	4.39202	4.33515	3.80831	3.70474
ADCY7	2.66713	2.6798	3.5666	3.68036	1.27969	0.989946
ADCY9	4.28889	4.13892	5.13501	5.18636	3.5769	3.0178

ADD1	5.78119	5.63683	6.03798	6.14085	6.53119	6.12574
ADD2	-2.69815	-3.29152	1.52387	1.46107	4.97767	4.5624
ADD3	4.83072	5.11127	6.13868	6.29254	3.4165	3.54594
ADGRA2	3.49888	3.14473	4.52594	3.9515	0.431912	-0.903665
ADGRA3	6.3705	6.43131	5.51514	5.72853	5.48173	5.34707
ADGRB2	2.95018	2.31525	2.67135	2.42646	0.939663	1.33772
ADGRE1	0.743937	0.90354	1.95093	2.36287	-5.07051	-5.69922
ADGRE5	4.74571	4.52449	4.53068	4.84722	5.0704	4.55027
ADGRF1	-3.11289	-2.46206	1.86635	1.57771	-6.64581	?
ADGRG1	3.36566	3.67927	1.48185	2.76155	6.24561	6.42867
ADGRG2	-2.69815	-1.87737	1.98141	2.50957	-2.33767	-1.24695
ADGRG6	4.67541	5.05482	-2.72315	-2.93781	1.46901	1.62424
ADGRL1	4.06464	3.68354	3.62095	3.60738	4.26719	3.86432
ADGRL2	5.86553	5.89715	3.6462	2.87132	-3.33694	-4.70296
ADGRL3	3.15111	2.53717	?	-5.10387	-2.41163	-2.8983
ADGRV1	-2.45038	-2.00285	4.49155	4.73822	6.78458	6.13956
ADH5	5.4731	5.48362	6.02277	6.27788	5.42836	6.20303
ADH5P4	2.41022	2.52962	2.97079	3.37251	2.52145	3.28507
ADI1	4.10662	4.66832	3.55625	4.71768	4.59526	5.82366
ADIPOR1	5.90662	5.99093	4.99948	5.5578	6.2749	6.57351
ADIPOR2	5.95555	6.03275	5.83883	6.22655	5.78107	6.14346
ADIRF-AS1	1.66814	1.83142	2.72224	2.93914	-0.403217	-1.0757
ADK	3.98132	4.02768	2.94183	3.40845	3.90706	4.47816
ADM	4.31912	4.02096	6.30203	6.20036	4.11858	3.04148
ADNP	6.32223	6.24688	6.22667	5.95154	6.30926	6.29114
ADNP-AS1	0.693311	0.251507	0.292311	0.266325	0.0812851	-0.848487
ADNP2	4.96669	4.91652	3.95218	4.1067	4.30979	3.97452
ADO	4.35742	4.37757	2.9166	3.26404	4.12899	4.61401
ADORA1	-1.74441	-1.105	2.62052	3.16264	-1.23372	-1.24695
ADORA2B	4.43469	4.48914	3.55288	4.16379	5.02443	4.92811
ADPGK	5.63376	5.4577	5.59223	5.958	5.32068	5.41907
ADPRHL2	3.50304	3.48844	2.8554	3.30269	4.3868	4.48063
ADPRM	0.586404	0.980157	1.20884	1.31748	1.70166	2.61522
ADRA1B	-0.306598	0.61405	-0.428455	-0.086319	2.52705	2.5925
ADRA1D	?	-6.45019	0.431281	0.300663	0.489522	0.359476
ADRB2	1.03569	2.01291	2.94576	3.75845	1.15352	-0.121607
ADRBK1	4.22572	3.76837	3.9839	4.05355	3.71689	3.51969
ADRBK2	-6.68418	?	2.65569	2.63601	4.37654	4.51607
ADRM1	5.62483	5.62239	4.74094	5.24852	5.489	5.84693
ADSL	5.99828	5.99313	4.66165	5.12658	5.62616	6.05192
ADSS	4.77336	4.90146	4.36484	4.80327	5.14871	5.56324
AEBP2	4.58977	4.61729	4.03806	4.07975	4.40308	3.85933
AEN	5.42341	5.4627	3.58466	4.03327	4.40308	4.54714
AES	4.85707	4.78949	4.54053	4.85764	5.65237	5.92665
AF127577.13	3.56418	3.037	3.23433	2.85696	1.40043	1.00759
AF127936.9	1.33871	1.48754	2.29424	2.25678	1.76436	2.23299
AF131215.2	1.71458	1.46201	1.89865	1.6394	1.7868	1.50278
AF131215.3	0.266817	0.305445	0.935482	0.563685	0.405873	-1.4584
AF131215.4	0.529821	0.344613	1.23438	0.833756	0.185296	-0.38461
AF131215.9	1.10833	1.07637	1.7707	1.27492	1.14064	0.832509
AF131216.6	1.48556	1.62524	1.86625	1.80307	0.843855	0.87611
AF196972.4	-0.394026	-0.101278	0.12446	0.947868	-0.087922	-0.151977

AFAP1	6.57899	6.43998	3.98637	4.14901	5.55029	5.18309
AFAP1-AS1	3.30583	3.27502	0.966512	0.793663	1.92904	1.09531
AFAP1L1	3.66396	4.0027	3.84576	4.65354	1.1471	0.50283
AFAP1L2	3.22277	3.39513	4.96175	5.01915	-6.64581	?
AFF1	4.35959	4.45004	4.84231	4.91146	4.67342	4.52928
AFF4	6.94691	6.95564	7.33423	7.44201	7.45333	7.13328
AFG3L1P	4.59266	4.61597	3.56634	3.60197	3.05909	2.43285
AFG3L2	4.81248	4.87914	4.78792	5.09714	5.33277	5.37945
AFG3L2P1	0.0305903	0.154894	0.228357	0.582874	0.911408	0.513572
AFMID	2.7726	2.87032	2.13842	2.68234	3.45473	3.54358
AFP	?	-6.45019	-3.18222	-2.40763	1.02625	1.493
AFTPH	5.17359	5.21158	4.05627	4.27178	4.97084	5.2392
AGA	4.03308	4.00475	2.49597	3.03838	2.90194	3.25907
AGAP1	5.26019	5.24624	4.2976	4.35265	5.14703	4.87542
AGAP2-AS1	1.06625	0.922868	1.42698	1.70515	1.57594	2.06693
AGAP3	4.70765	4.50797	4.58383	4.78371	4.85143	4.83146
AGAP4	2.56384	2.30004	1.23658	1.01422	2.73965	2.07872
AGAP5	-0.806927	-0.155989	0.412044	0.352883	1.14509	0.692784
AGAP6	2.58269	2.49315	2.22933	2.27996	3.32314	2.54027
AGAP7P	-0.927702	-0.592889	1.69948	1.75101	0.508885	-0.947531
AGAP9	1.37513	1.46354	2.0406	2.01347	1.90473	0.540474
AGBL5	4.69252	4.70278	3.76352	3.87379	4.33462	4.4479
AGFG1	5.69053	5.70675	5.81046	6.08315	6.58387	6.39394
AGFG2	2.98216	2.70709	3.28	4.35571	3.61698	3.76089
AGGF1	4.67974	4.82139	4.9776	5.10305	5.2906	5.64145
AGK	4.7741	4.82926	4.23311	4.48175	5.21615	5.48557
AGL	4.27746	4.25568	4.39673	4.54132	4.59472	4.69203
AGMO	1.52013	1.86361	-6.17309	-6.09892	?	?
AGO1	5.80051	6.08257	5.26665	5.67334	6.31161	6.00495
AGO2	6.89626	6.74202	5.90592	5.75884	6.58983	5.66777
AGO3	5.29067	5.42323	4.89127	4.97194	6.18477	5.91716
AGO4	3.50915	3.60593	2.94834	2.81302	3.33122	3.15748
AGPAT1	4.37962	4.19357	3.84856	3.98078	4.8564	4.81411
AGPAT2	2.37508	2.22017	2.31634	2.56361	1.59712	2.27059
AGPAT3	4.86935	4.91598	5.5554	5.95592	4.26598	4.44556
AGPAT4	3.15423	3.02499	3.91976	3.88694	5.53075	4.88948
AGPAT4-IT1	-1.1752	-0.214764	-0.117291	0.773887	1.82755	1.33772
AGPAT5	5.49935	5.4866	4.79161	5.01398	6.07037	5.88341
AGPAT6	5.74588	5.80972	4.95663	5.15496	5.29857	5.24017
AGPAT9	5.80523	5.62592	4.82421	5.28509	4.27042	4.40442
AGPS	5.33811	5.62891	6.2576	6.68598	6.97392	7.4888
AGRN	3.67595	3.17378	4.58629	4.30268	4.37724	4.06477
AGTPBP1	4.49507	4.69196	1.97637	2.62589	4.21544	4.80996
AGTRAP	3.04239	2.80395	3.53072	4.01141	2.03668	2.33222
AHCTF1	6.48315	6.46884	6.15627	6.35216	6.4841	6.31177
AHCY	7.01076	6.89542	5.97719	6.0928	6.69681	6.89903
AHCYL1	5.53632	5.92754	6.2301	6.6982	5.9681	6.52235
AHCYL2	3.35892	3.16286	2.77938	2.77076	3.52208	3.60163
AHDC1	2.24051	2.18822	2.79662	2.60543	3.05736	2.63759
AHI1	4.02202	4.13985	4.1652	4.48642	4.56669	4.8421
AHNAK	9.79411	10.2065	10.6791	11.207	8.01077	8.68122
AHNAK2	7.22041	8.29722	2.47446	3.57049	5.3938	6.47121

AHR	6.55258	6.50132	7.92495	8.16971	-2.33767	-2.38405
AHRR	4.1353	3.8947	4.52813	4.49849	-0.729347	-2.00571
AHSA1	5.69189	5.68498	4.1663	4.54491	6.05778	6.16942
AHSA2	5.0928	4.95335	5.56695	5.27427	5.07134	4.16194
AIDA	4.48083	4.49371	4.50118	4.85515	4.93751	5.22369
AIF1L	4.08635	3.63386	-1.09576	-0.682344	6.90587	6.87653
AIFM1	3.25803	3.08019	4.20018	4.58639	5.08116	4.76394
AIFM2	3.29952	3.32506	3.66823	3.98657	4.30619	3.98329
AIG1	2.46584	2.62992	3.61413	3.98657	3.73843	4.38207
AIM1	1.84398	2.10323	3.98765	4.70894	5.73977	6.12067
AIM2	0.54893	-0.177293	0.849967	1.76157	?	?
AIMP1	4.69192	4.72539	4.4226	4.76934	4.81324	5.22141
AIMP2	3.74234	3.65859	3.25475	3.62265	3.90476	3.97615
AIP	4.08555	3.83636	2.96623	3.23547	3.08621	3.51488
AJ011931.1	-1.99808	-3.00228	1.05494	1.04098	?	?
AJUBA	3.52973	3.54837	3.22162	3.7288	4.42516	4.1744
AK1	2.8738	2.73549	2.98087	3.30499	2.45264	2.98418
AK2	6.06276	6.12088	5.56022	6.06038	6.56174	6.66617
AK3	4.16634	3.93883	4.77832	5.06146	6.26212	6.33178
AK4	4.54736	4.39784	2.56214	2.80508	6.91106	5.84153
AK4P1	1.87269	1.61577	0.407625	0.398963	3.63212	2.63817
AK5	0.480917	0.90354	2.63347	3.54939	2.2254	2.81293
AK6	2.59781	2.6392	2.95261	3.39259	4.01495	4.86921
AKAP1	5.46392	5.74379	4.14832	4.55206	5.43009	5.3781
AKAP10	3.81069	3.68308	4.54098	4.70415	4.98218	4.96747
AKAP11	5.66258	6.0106	6.02134	6.37069	5.58494	6.22253
AKAP12	7.02751	7.19285	1.0162	1.71775	7.87731	6.96303
AKAP13	6.1659	6.20383	7.10848	7.15919	6.73811	6.32542
AKAP17A	3.2449	3.18102	4.32228	4.47236	2.8962	2.31011
AKAP2	5.6363	5.68057	4.91414	5.5826	6.42466	5.84417
AKAP5	2.11472	2.25485	1.30837	1.1104	2.71474	2.92629
AKAP6	1.10833	1.06859	4.34395	3.44577	3.26005	3.32396
AKAP7	1.22578	1.10707	0.770744	0.935633	1.99792	2.66398
AKAP8	3.73346	3.85344	3.26149	3.62926	4.37379	4.123
AKAP8L	3.61293	3.53397	3.80578	3.91641	4.50248	4.05807
AKAP9	7.28624	7.2518	7.66071	7.72363	7.66176	7.59435
AKIP1	2.08557	2.12589	2.63027	3.08839	2.94887	3.35938
AKIRIN1	5.2782	5.15554	4.4033	4.83515	5.86128	5.62978
AKIRIN2	3.07406	3.15921	3.0308	2.93557	4.13248	4.25906
AKNA	1.12115	1.12217	2.19587	2.47615	1.82756	1.65964
AKNAD1	1.37778	1.692	-1.01332	-1.06415	-2.01588	-1.70626
AKR1A1	3.34941	3.41675	4.30832	4.73508	4.12104	4.56241
AKR1B1	7.00333	6.55718	9.3901	10.4202	6.32861	6.75469
AKR1B10	-3.2383	-1.37616	7.59841	8.32719	1.10793	1.79309
AKR1B10P1	-6.68418	-4.45968	3.4078	4.29849	-2.75246	-2.00571
AKR1B15	-5.10914	-6.41219	2.11271	2.58337	-4.07293	-5.68708
AKR1B1P1	-0.3302	-0.457375	1.9192	2.98323	-0.562563	-0.662104
AKR1B1P2	0.582248	0.20969	2.9909	4.02931	-0.274152	0.200291
AKR1B1P7	0.0799481	0.0492125	2.85552	3.92398	-0.440514	-0.24095
AKR1C1	2.47623	2.25005	8.53036	9.18548	2.85136	3.32359
AKR1C2	-2.02373	-1.11652	8.05702	8.67295	-1.47704	-0.951362
AKR1C3	2.41287	2.28779	6.7699	7.33968	4.81476	5.38874

AKR7A2	3.39897	3.24452	3.5962	3.70495	4.09458	4.40181
AKT1	6.21833	5.9447	5.44678	5.75063	5.74126	5.91233
AKT1S1	3.62314	3.33523	3.7824	3.79856	4.62751	4.33837
AKT2	5.37672	5.21882	5.11797	5.19779	6.04143	5.97233
AKT3	6.56479	6.35715	5.28487	5.38417	6.00478	5.94941
AKTIP	2.44328	2.7069	2.86409	3.24425	3.89979	4.03929
AL023806.1	-0.0990262	-0.195907	1.87178	1.77994	1.28225	0.785207
AL133243.1	1.62288	1.9252	1.83887	1.42259	1.72342	0.878317
AL133243.2	2.27535	2.28872	2.12028	1.69856	2.35093	2.06149
AL133243.4	1.58636	1.74664	1.40148	1.195	1.7868	0.878317
AL133493.2	-3.2383	-3.46127	-2.27595	-2.40763	1.92468	1.12623
AL136419.6	-0.0156236	0.321362	-0.355512	0.112473	0.325651	0.683705
AL158801.1	2.44652	2.71143	2.17467	2.66553	2.28238	2.65384
AL162151.3	2.00034	1.71962	0.290861	0.770922	1.7571	2.21039
AL365181.2	1.70486	1.84521	0.581361	0.719954	1.61667	0.931607
AL589743.1	2.56303	2.73683	1.67449	1.71138	1.06767	0.993783
AL590762.6	-0.197398	0.000504855	0.198972	0.155477	0.00881672	-0.212101
ALAD	3.52129	3.50581	3.00383	3.4686	3.26599	3.35399
ALAS1	4.24049	4.3078	4.75751	5.32162	4.94702	4.96659
ALCAM	5.25872	5.04047	6.5749	6.66118	7.02109	7.54183
ALDH16A1	2.2313	2.01696	1.39934	1.60032	2.9225	3.46493
ALDH18A1	5.78978	5.96921	5.41774	5.81821	6.0268	6.07337
ALDH1A1	0.388476	0.756391	3.19369	3.80854	-2.75246	-2.70579
ALDH1A3	9.2924	9.47601	3.16129	3.45454	3.96039	3.95934
ALDH1B1	3.88588	3.88256	3.74277	4.24542	5.04012	5.15824
ALDH1L2	2.57169	2.68422	-0.757028	-1.06415	1.51964	1.08233
ALDH2	-4.37417	-3.65369	4.32821	4.48176	-5.65305	?
ALDH3A1	?	-4.87366	3.35564	4.29096	-4.65668	-4.70296
ALDH3A2	4.32744	4.56644	5.84299	6.27916	5.28439	5.77103
ALDH3B1	0.832372	0.603321	2.53073	2.95727	2.44823	3.3834
ALDH4A1	2.21197	1.48315	1.78805	1.77671	2.14547	1.99765
ALDH5A1	2.67161	2.57059	1.30034	0.724135	1.19772	0.597145
ALDH6A1	2.82749	2.48671	2.70973	3.14556	2.24632	2.30462
ALDH7A1	4.59581	4.50441	1.70439	1.92262	4.35474	4.38181
ALDH7A1P1	3.20861	3.14254	0.388586	0.340232	2.87983	2.74531
ALDH9A1	5.06787	4.60995	4.16962	4.09452	4.83853	4.76088
ALDOA	7.66749	7.53421	8.49734	8.85328	9.00159	8.95302
ALDOAP1	0.266817	0.237698	1.09265	1.72409	1.79504	1.00759
ALDOAP2	0.0839826	0.456112	1.43857	1.76259	1.90649	0.924206
ALDOC	2.1083	1.79457	-0.886753	-1.18987	-0.0903144	-1.18284
ALG1	3.67263	3.58938	3.79859	4.22176	3.42604	3.74578
ALG10	3.81073	3.92434	3.54987	3.51087	2.59638	2.05635
ALG10B	4.07902	4.05951	4.41711	4.306	3.21052	2.61421
ALG11	3.5936	3.5062	3.68111	3.8354	3.51683	3.42936
ALG12	3.41743	3.27001	4.10215	4.12578	2.90728	2.64384
ALG13	2.52493	2.70709	3.73236	3.63095	4.79141	4.54239
ALG14	2.24931	2.3413	2.30031	2.68234	2.56129	3.11984
ALG1L15P	0.121188	0.124196	-0.0361309	0.742894	-0.087485	-0.105554
ALG2	3.8284	3.82025	3.77647	4.15327	3.59709	3.28481
ALG3	3.51767	3.33319	3.66039	4.12718	4.10704	4.3045
ALG5	3.61946	3.58292	3.17403	3.55117	3.27633	3.87071
ALG6	4.87928	4.44854	3.42011	3.42256	3.2116	3.49786

ALG8	6.1165	6.10935	5.02653	5.34392	4.48051	4.69949
ALG9	2.34945	2.27519	3.85851	4.09758	4.05099	3.78154
ALK	1.24348	1.44408	-0.482896	-0.154483	0.258664	-0.31423
ALKBH1	2.63855	2.65611	1.74132	1.91357	2.52209	2.677
ALKBH2	4.01344	4.12305	1.75902	1.87419	3.16942	3.3781
ALKBH3	2.41467	2.30866	2.7413	3.11548	3.22081	3.33221
ALKBH4	2.73474	2.77691	1.9762	2.17936	2.57728	2.98766
ALKBH5	5.10816	5.01997	5.38866	5.76261	6.55962	6.11301
ALKBH6	1.867	1.614	2.65254	2.70175	2.46641	2.05486
ALKBH7	-0.00787984	-0.58	-0.223662	0.404816	0.904997	1.93717
ALKBH8	2.02886	2.28536	2.75018	3.00752	3.66499	4.16211
ALMS1	6.45522	6.37164	5.48578	5.43708	5.84597	5.86501
ALMS1-IT1	1.17748	1.10707	1.48894	1.2041	0.185296	-0.4586
ALOX12-AS1	0.636395	0.44211	1.7435	1.80287	1.05066	0.597145
ALOX12P2	0.278306	0.491443	2.80517	2.58118	1.37865	0.0213376
ALPK1	2.77095	2.75651	4.04409	4.13672	3.27361	3.42735
ALPK2	-0.740601	-1.14333	8.00204	8.1335	7.05739	6.82462
ALPK3	-2.23898	-1.93875	-1.93506	-2.10822	2.3227	1.80902
ALS2	4.80871	4.85343	4.84092	5.0062	5.1004	4.77204
ALX4	-2.9975	-3.13966	3.7128	4.0256	-1.61549	-2.12114
ALYREF	5.63938	5.55744	4.53243	4.86988	5.82803	5.96173
AMACR	2.99899	3.67905	2.38194	2.79491	2.69881	3.19451
AMBRA1	4.35281	4.21575	4.56383	4.55383	4.52207	3.94253
AMD1	6.33818	6.27748	5.5903	5.99285	7.53334	7.59038
AMD1P3	-0.501561	-0.207583	-0.568651	-0.342526	0.658554	0.834112
AMDHD2	2.52316	2.01545	2.55105	2.587	2.13057	2.13223
AMER1	3.21231	3.20428	4.06083	4.13672	3.08957	2.83632
AMFR	5.34886	5.51473	6.17923	6.50382	6.84103	6.7671
AMIGO1	2.24345	2.06466	-2.37543	-2.52305	2.23149	1.76092
AMIGO2	4.47329	4.75109	4.40508	4.98224	5.52277	5.62159
AMMECR1	4.25728	4.26167	3.93671	4.12599	5.82096	5.60219
AMMECR1L	4.95853	4.92549	3.91778	4.00151	4.47761	4.11027
AMN1	2.1248	2.08533	1.81671	2.09876	2.22065	2.71013
AMOT	3.69324	3.70079	4.50735	4.51596	5.10041	5.37141
AMOTL1	6.51094	6.81982	5.83745	5.86666	4.83606	4.93354
AMOTL2	4.51197	4.51144	5.02278	5.41051	5.90046	5.57436
AMPD1	-5.69162	-6.45019	?	?	3.75013	3.64201
AMPD2	4.45415	4.32017	5.33039	5.5233	5.36578	4.93119
AMPD3	0.207918	0.603321	2.88526	3.31744	-0.374735	0.615294
AMT	0.61388	0.537236	1.67449	1.54226	1.2586	0.483199
AMZ1	0.0692347	-0.177294	1.32432	1.04098	-0.338214	-1.79933
AMZ2	4.02485	4.02589	4.29688	4.70123	4.69604	5.32323
AMZ2P1	1.42883	1.26602	0.929693	0.833756	1.61119	1.89545
ANAPC1	6.24131	6.26695	5.48301	5.79973	6.0671	6.0803
ANAPC10	2.1768	2.00285	2.07245	2.19561	2.17064	2.69441
ANAPC10P1	-0.0370305	0.0806986	-0.0413934	0.0777215	-0.137246	0.563998
ANAPC11	3.86195	3.81481	3.0994	3.50743	4.46563	5.47495
ANAPC13	3.86975	3.74846	3.58464	4.05512	3.65678	4.29757
ANAPC15	3.49883	3.27138	2.60377	2.87613	3.14353	3.34133
ANAPC16	4.22764	4.2502	3.94285	4.18674	5.22391	5.56806
ANAPC2	3.09209	2.98631	3.29828	3.39698	2.85539	2.63759
ANAPC4	3.82349	3.95394	3.40519	3.65106	4.11365	4.4005

ANAPC5	6.18339	6.20081	5.72261	6.04534	5.97562	6.28004
ANAPC7	5.02794	5.26716	4.4872	4.91242	4.5591	4.87541
ANGEL1	3.43409	3.30122	2.71148	2.9938	2.95117	2.89816
ANGEL2	3.38174	3.6019	4.30832	4.4515	4.64974	4.86218
ANGPT1	2.63409	2.26509	6.74868	6.77323	3.42582	4.23286
ANGPT2	2.02204	2.08021	1.51	1.51326	3.20233	2.84019
ANGPTL1	1.78879	1.40138	-1.37581	-0.979274	0.282309	-0.0341529
ANGPTL2	3.26959	3.01289	2.4422	2.13437	-0.827177	-1.18284
ANGPTL3	0.676033	0.831845	0.793829	0.383121	0.134221	-1.006
ANK2	3.06414	3.62594	-2.01303	-1.58485	4.61465	5.11425
ANK3	4.10172	4.24076	3.89261	3.70615	5.12979	5.13374
ANKAR	0.792847	0.804012	-0.276471	-0.436219	0.983605	0.847941
ANKDD1A	-0.289348	-0.484239	1.58948	1.62928	-0.337166	-1.24696
ANKEF1	0.885949	0.929533	1.5913	1.92462	2.58288	2.52211
ANKFN1	0.133932	0.676782	0.558032	0.176693	-0.0752099	-0.00614162
ANKFY1	4.72412	5.02998	5.64986	6.18284	5.65989	5.76234
ANKH	4.81513	4.77077	4.00506	4.01659	4.56369	4.20318
ANKHD1	5.64386	5.61247	6.39657	6.27489	5.47311	5.2484
ANKHD1-EIF4EBP:	4.54154	4.34513	4.80547	4.84557	4.15548	4.08183
ANKIB1	6.13103	6.12164	7.25006	7.45423	6.25556	6.26556
ANKLE2	6.32049	6.44929	5.79304	6.11003	5.85414	5.62924
ANKMY1	2.98635	2.83424	2.79948	2.73353	2.44695	2.04819
ANKMY2	3.03733	3.11269	3.97635	4.27593	3.76699	4.14661
ANKRA2	1.82002	1.84552	2.56468	2.72373	3.17889	3.50556
ANKRD1	-1.45077	-1.76193	1.26773	1.51326	2.27044	1.80111
ANKRD10	5.21305	5.27651	4.72936	4.88269	5.52269	4.88387
ANKRD10-IT1	1.8934	2.06074	2.09727	1.97335	2.44823	0.100762
ANKRD11	6.07338	5.90154	6.21403	6.01088	5.69843	5.02871
ANKRD12	4.39234	4.26842	5.28527	5.17584	5.78091	5.73493
ANKRD13A	4.79069	4.88386	4.36576	4.73695	4.6901	4.8443
ANKRD13B	2.09535	2.03702	1.62387	1.59857	2.38955	2.10709
ANKRD13C	4.54409	4.1417	4.82699	4.80183	4.86274	4.55067
ANKRD13D	3.62172	3.46197	3.64143	3.80406	3.22691	2.74451
ANKRD16	0.398274	0.676782	-0.0381633	-0.285363	0.314058	-0.252367
ANKRD17	6.6841	6.57676	6.43691	6.52847	7.61521	7.37414
ANKRD18EP	1.57708	1.63522	1.16526	1.2921	1.99076	1.46325
ANKRD20A17P	-0.207091	-0.302468	1.79379	1.63108	-2.02517	-1.96079
ANKRD26	3.4895	3.44404	4.45491	4.31429	3.8255	3.51488
ANKRD27	4.36969	4.2219	4.61737	4.86558	4.96813	4.93443
ANKRD28	6.61648	6.59987	5.96621	6.45626	5.9017	6.12932
ANKRD29	-0.0695851	0.060836	0.711372	1.43037	-2.20023	-1.12145
ANKRD30A	1.49078	2.21313	-4.59508	?	?	-4.70296
ANKRD30B	-4.0473	-3.28251	-0.768941	-1.08276	1.08464	1.80155
ANKRD33B	3.39764	3.26337	4.32723	4.014	3.12105	2.39658
ANKRD36	4.88699	5.06175	4.19144	4.10333	4.47937	4.40115
ANKRD36B	4.07361	4.1044	3.46085	3.26415	3.4772	3.22373
ANKRD36BP1	0.639417	1.89637	0.184737	0.353414	0.944034	-7.21644
ANKRD36C	4.51519	4.6035	4.39652	4.30337	3.87911	3.87559
ANKRD37	-0.0426158	-0.13667	-0.49899	0.456323	1.96808	1.96459
ANKRD39	2.74063	2.72438	2.46424	2.34733	2.91965	2.7267
ANKRD40	5.64521	5.68657	4.97886	5.30479	5.49481	5.52929
ANKRD42	1.99586	1.885	2.91196	2.91828	2.25009	1.98475

ANKRD44	3.3804	3.47085	2.60439	2.12481	3.01565	2.52692
ANKRD46	2.52064	2.3632	2.70123	2.4475	2.82059	2.33844
ANKRD49	2.01774	1.95676	3.09422	3.22385	2.30299	2.8295
ANKRD50	5.1507	5.11175	5.71506	5.36233	6.21445	6.51697
ANKRD52	5.49905	5.53715	5.41312	5.44767	5.67898	5.62867
ANKRD54	3.92269	3.80229	3.70118	3.82327	3.64699	3.23155
ANKRD6	2.78135	2.60469	-2.21134	-2.24213	-0.544285	-0.72027
ANKRD61	0.278306	0.456112	0.64945	0.399062	0.0402612	-0.662104
ANKRD9	3.49003	3.36404	2.06442	2.13891	2.43916	1.99584
ANKS1A	3.83327	3.69576	3.01124	3.09819	4.36621	4.23139
ANKS3	2.79678	2.82487	2.73833	2.87417	2.89044	3.00754
ANKS6	4.1131	3.98629	3.56131	3.37297	2.5637	2.13251
ANKZF1	4.27246	4.16285	4.39579	4.61397	5.92346	4.63424
ANLN	7.75931	7.78677	7.36634	7.94617	7.48153	7.75249
ANO10	4.73214	4.69849	4.23817	4.67453	4.78936	5.37447
ANO5	2.36433	2.34452	-0.69145	-1.06415	-6.64581	?
ANO6	6.44063	6.63075	6.00193	6.40291	6.08387	5.80453
ANO7	0.367103	0.278722	0.292311	-0.52367	1.76598	0.33778
ANO8	1.06253	0.894767	1.43123	1.35064	2.35094	1.95865
ANP32A	5.99431	5.63945	5.83374	5.69943	6.04422	5.27064
ANP32AP1	1.92649	1.74358	1.2947	1.1781	1.7637	1.13196
ANP32B	4.78623	4.43163	3.76495	3.70664	3.84673	3.73718
ANP32BP1	1.06264	0.950439	0.38531	0.333344	0.118014	0.305515
ANP32E	6.6112	6.88672	5.5726	5.91664	6.41462	6.09174
ANTXR1	5.37272	5.38303	6.66723	6.58727	5.27668	5.47815
ANTXR2	3.24636	3.51586	3.70978	4.11034	3.02093	3.51487
ANXA1	7.22469	6.9009	6.93219	7.55429	7.28309	8.08547
ANXA11	5.00265	4.92677	5.55076	6.0111	5.11075	5.2569
ANXA2	8.42938	8.26113	7.95829	8.25389	9.68175	10.02
ANXA2P1	2.82683	2.86264	2.45819	2.76408	3.97929	4.07827
ANXA2P2	8.23173	8.1271	7.92146	8.15903	9.46123	9.88681
ANXA2P3	1.41022	1.57079	1.16558	1.57091	3.09279	2.85189
ANXA2R	0.178249	0.186057	1.72599	1.86848	1.02216	1.27061
ANXA3	-6.68418	-3.87578	-2.37543	-4.10635	0.901996	1.95152
ANXA4	2.73347	3.00272	4.28916	4.90169	3.3098	3.84018
ANXA5	6.56036	6.54892	6.92172	7.52075	6.92279	7.23874
ANXA6	4.75932	4.52796	7.05622	7.44982	-0.959604	-1.24695
ANXA7	5.12386	5.28159	4.97575	5.55492	5.62251	5.82252
ANXA9	-0.943927	-1.33321	0.544485	1.14862	0.0677351	-0.247123
AOC1	-4.69535	-4.87366	1.26773	2.42646	?	?
AOC2	0.586404	0.645757	-0.401986	-0.823182	0.405873	-0.0341529
AOX1	-1.48924	-1.2146	0.259532	1.62592	-0.729347	0.33778
AP000275.65	0.0915306	0.266337	-0.220818	0.253372	0.801779	-1.79157
AP000347.2	2.96318	2.85636	2.66739	2.72403	2.11392	0.698814
AP000350.5	0.727258	0.635262	2.96369	2.9383	2.8962	2.28766
AP000350.6	0.183671	0.514521	1.26048	1.40773	1.28225	0.9808
AP000357.4	-0.239069	-0.193003	-0.183372	0.248838	0.522297	0.293394
AP000462.1	1.21386	1.00482	-0.276471	-0.0426024	-0.0602604	-0.00614162
AP000472.2	1.84398	1.67674	-4.18092	-4.52056	-6.64581	?
AP000473.8	?	-6.45019	-5.1783	?	1.30552	1.90064
AP000487.5	1.08629	1.16749	0.680756	0.947561	-0.0543249	-0.123535
AP000560.3	1.14018	1.15192	2.05012	1.87988	-0.0602604	-0.899103

AP000577.2	0.54893	0.395227	0.838911	0.445854	-0.0602604	-1.18284
AP000648.5	3.1227	3.05286	4.23643	4.26403	3.33545	2.9515
AP000705.8	2.21383	1.93378	-5.1783	-6.09892	?	?
AP000892.6	1.53901	1.44831	1.66031	1.84545	2.17682	1.66884
AP001062.7	-0.200124	-0.280876	1.20587	1.03072	-0.0471521	-0.55378
AP001258.4	0.0568748	0.137189	0.675003	1.10068	0.0129405	0.270672
AP001372.2	0.768595	1.11463	0.431281	0.457563	-0.320295	-0.121607
AP001442.2	0.50068	0.656176	1.8772	1.39106	0.799187	-0.247125
AP001469.9	0.458655	0.794986	0.942428	1.2999	0.341758	0.0359335
AP003900.6	3.257	3.42551	1.90581	1.77322	-6.64581	?
AP006621.5	0.0152977	-0.105152	1.15638	1.2921	-1.20056	-2.8983
AP1AR	3.4302	3.58972	2.90394	3.27488	3.6571	3.97452
AP1B1	5.57034	5.3795	5.32328	5.54221	5.703	5.62363
AP1G1	5.65983	5.84691	6.30756	6.61782	6.77915	7.12913
AP1G2	2.9525	2.50874	3.00034	3.00602	2.80186	2.54044
AP1M1	4.36565	4.21927	3.74277	4.13196	5.41079	5.46321
AP1S1	4.94659	5.05481	3.80232	4.36993	4.6297	5.36406
AP1S2	4.60185	4.61169	3.66572	3.86998	4.0227	3.97025
AP1S3	0.510456	0.877069	-0.455418	-0.201784	2.19458	2.25331
AP2A1	4.18023	3.31448	4.85338	4.56277	4.97965	4.23641
AP2A2	4.80325	4.77555	4.81788	5.13196	4.1891	4.45693
AP2B1	6.51957	6.7867	6.7804	7.12642	7.39145	7.43245
AP2M1	6.0937	6.01489	6.92726	7.29694	8.15669	8.45367
AP2S1	4.9193	4.7356	4.65882	5.05167	5.86151	6.21029
AP3B1	5.0982	5.12783	5.74459	5.8957	5.71604	5.85514
AP3B2	2.71878	2.41061	3.22799	3.2288	-3.48878	-4.70296
AP3D1	5.37202	5.11172	5.39909	5.61696	6.22289	5.73984
AP3M1	4.99394	5.02749	4.83311	5.20724	5.77796	5.70518
AP3M2	3.86125	4.08599	3.12709	3.4515	3.41116	3.22992
AP3S1	4.25304	4.34464	4.46689	4.9216	4.61019	5.06422
AP3S2	4.49101	4.47739	4.70918	4.99156	4.25644	4.32204
AP4B1	3.96359	4.10033	3.4773	3.74918	3.52178	3.78554
AP4B1-AS1	0.500639	0.559746	0.301002	-0.000493486	-0.28093	-0.287003
AP4E1	4.41726	4.39357	3.81224	4.23989	4.62336	4.4519
AP4M1	2.82547	2.69694	2.17065	2.45536	2.44135	2.62755
AP4S1	1.85937	1.88148	1.47473	1.7241	1.87499	2.26485
AP5B1	3.85742	3.70081	3.07858	3.27055	2.81137	2.61523
AP5M1	4.57704	4.69386	3.67911	4.08223	4.99297	5.28692
AP5S1	2.98781	2.84913	3.65049	3.77043	3.06509	3.09027
AP5Z1	2.27248	1.97597	3.05727	3.12958	2.77433	2.74038
APAF1	4.12338	4.11703	4.07029	4.17883	3.6799	3.40437
APBA1	2.85167	2.41369	-3.59683	-4.10635	1.88274	1.55071
APBA2	-4.37417	-4.13839	3.9393	4.30477	2.02621	2.17595
APBA3	2.10845	1.89912	1.31926	1.57771	2.19518	2.25025
APBB1	3.84394	3.51729	1.14744	1.12963	2.39769	2.38077
APBB2	4.58862	4.72139	4.37202	4.51961	6.54944	6.07913
APBB3	2.3524	2.42598	2.4113	2.37407	2.84543	2.30261
APC	6.47065	6.52937	6.26588	6.50588	7.00823	7.17116
APC2	0.0483618	-0.312413	0.847243	0.39765	0.442142	0.434081
APCDD1L	5.56711	5.57263	-0.276471	0.139221	1.47928	2.21217
APCDD1L-AS1	4.50426	4.31687	1.33222	2.07607	1.71041	2.52692
APEH	5.46518	5.43044	4.63105	4.93761	5.81314	6.22881

APEX1	5.45192	5.25138	4.34394	4.59853	5.03838	4.99977
APEX2	3.57588	3.49571	2.42751	2.8337	3.25258	3.36206
APH1A	6.1474	6.06618	5.31624	5.71302	6.0206	6.66156
APH1B	3.46456	3.22366	3.53757	3.70014	2.77224	2.65085
API5	5.83256	5.91059	5.38341	5.84541	6.4053	6.55214
API5P1	2.8681	3.17856	2.6097	3.03545	3.47125	3.6164
APIP	3.27334	3.17811	3.12682	3.16327	1.23865	1.51767
APITD1	2.19151	2.21439	2.10905	2.40558	2.45213	3.18744
APLF	2.21682	2.37322	1.80519	2.01543	-3.33694	-1.89883
APLP1	3.1115	3.00068	3.12482	3.16264	0.351018	0.752791
APLP2	7.71593	7.70933	7.24558	7.60198	8.52511	8.22063
APMAP	7.71879	7.80287	6.27672	6.74878	6.71346	7.10212
APOA1BP	4.68407	4.84093	4.01737	4.33411	4.86471	5.5239
APOBEC3B	3.981	3.97273	4.47798	4.74985	-3.46323	-5.69922
APOBEC3C	3.09696	3.18785	4.35951	4.78068	3.0262	3.64643
APOBEC3D	0.290554	0.0622599	2.11513	2.10054	-1.94632	-2.71249
APOBEC3F	0.116325	0.0603656	2.40349	2.48714	0.258688	0.741601
APOBEC3G	0.86443	0.594147	4.69749	5.13155	-0.2406	0.57353
APOBEC4	0.586404	0.444137	0.73541	0.773887	-0.152369	0.0483059
APOD	2.87268	3.44705	-4.59508	-3.78492	-5.65305 ?	
APOL1	1.58172	1.53154	5.16354	5.50634	-0.184426	-0.577229
APOL2	3.29102	3.40752	4.7916	5.40191	4.1029	3.70479
APOL3	-3.2383	-1.65505	3.41266	4.23212	-0.393349	-0.214706
APOL6	3.66067	4.13054	5.87175	6.45769	4.5751	5.01523
APOLD1	1.86768	1.9762	3.36262	3.68005	2.58272	2.35429
APOO	1.67557	1.66131	1.94572	2.39897	2.30813	2.82355
APOOL	2.73746	2.9159	2.67758	3.00752	3.24788	3.62868
APOPT1	2.23519	2.27787	1.99853	2.19512	3.1494	3.65682
APP	8.15871	7.9539	8.65247	8.69868	8.43522	7.70478
APPBP2	4.97688	5.08982	5.01122	5.26946	5.76121	6.23431
APPL1	5.58972	5.779	4.90591	5.19941	6.41239	6.29608
APPL2	4.22795	4.30698	3.24484	3.377	3.63545	3.94074
APRT	4.36431	3.90238	3.80799	4.07728	4.26227	4.44809
APTR	1.52496	1.49139	1.75902	1.83372	1.57335	1.23585
APTX	3.67921	3.85117	3.3498	3.82486	4.56006	5.13169
AQP11	1.49572	1.67674	0.699196	0.632715	1.09463	1.25912
AQR	5.91148	5.99118	5.42149	5.57766	5.21619	5.33082
AR	-5.10914	-4.13839	5.31828	5.28133	5.24507	5.25908
ARAF	3.85263	3.69829	3.06678	3.34445	3.87884	4.00924
ARAP1	4.0098	3.51215	4.49085	4.56897	4.02734	3.88353
ARAP2	3.25075	3.57333	-6.17309 ?		-6.64581 ?	
ARAP3	4.11951	4.25654	3.87175	3.93008	3.48052	3.6398
ARCN1	5.95799	5.79338	6.35245	6.69349	6.28123	6.17443
AREL1	5.95402	5.90621	4.48624	4.67825	5.80701	5.50517
ARF1	6.31822	6.27083	6.3986	6.81834	6.82797	6.97926
ARF3	6.0491	5.9701	5.72822	6.143	5.91788	6.04899
ARF4	6.24183	6.00013	6.15027	6.46822	6.96947	6.65468
ARF4P1	1.26881	1.2314	1.19157	1.42459	1.97729	2.00067
ARF5	4.52238	4.43858	3.92522	4.13404	4.11345	4.71274
ARF6	5.6004	5.55221	4.75164	5.03773	5.18909	5.10224
ARFGAP1	5.35957	5.14354	4.97066	5.13314	5.05735	5.07628
ARFGAP2	4.71439	4.62293	4.81268	4.95072	5.20133	5.18029

ARFGAP3	4.31411	4.56016	4.09725	4.54581	4.47026	5.0058
ARFGEF1	5.6199	5.76745	6.10515	6.29755	5.66534	6.0869
ARFGEF2	6.72271	6.80203	6.21414	6.69469	6.11385	6.21363
ARFGEF3	2.91747	3.29037	0.503064	1.46107	5.28439	4.91075
ARFIP1	4.20555	4.20604	3.77936	4.22544	4.37929	4.60956
ARFIP2	5.42179	5.29202	4.4275	4.88481	5.32483	5.77759
ARFRP1	4.52613	4.45004	4.23853	4.49665	3.89043	4.00925
ARG2	3.38873	3.19515	2.47066	2.90422	3.95993	4.34876
ARGLU1	5.22126	5.18596	5.56004	5.43419	5.40711	4.90245
ARHGAP1	4.14719	4.13334	3.99887	4.37297	5.04748	4.99459
ARHGAP10	3.35348	3.29204	3.53587	3.45913	3.51461	3.52211
ARHGAP11A	6.37499	6.45564	5.67105	5.96854	5.55847	5.53791
ARHGAP11B	4.85701	4.89626	4.24451	4.36464	4.06414	3.3387
ARHGAP12	6.01017	5.98213	4.93541	5.23101	5.28841	5.74037
ARHGAP17	3.74903	3.67925	4.57885	4.56359	3.70382	3.37543
ARHGAP18	3.80574	4.04197	4.51431	5.03263	5.6483	6.1824
ARHGAP19	4.59897	4.60973	3.89195	3.95994	4.22613	4.41681
ARHGAP20	-1.61119	-1.55554	1.88259	1.13916	-5.07051	-4.11926
ARHGAP21	6.47277	6.33422	5.93325	6.19407	6.49896	6.20819
ARHGAP22	-2.1135	-1.93875	1.20023	1.94105	1.67957	1.43288
ARHGAP23	1.56656	0.849663	3.64201	3.42962	2.10789	1.53173
ARHGAP24	2.39801	1.98016	1.05741	0.834744	2.70745	3.17957
ARHGAP26	3.36836	3.60324	3.05727	3.26404	6.07525	5.39393
ARHGAP27	1.60474	1.10707	0.860939	1.07114	-0.0322677	0.315752
ARHGAP28	-0.24304	-0.177294	5.58353	5.63724	?	-5.69922
ARHGAP29	6.05602	6.07192	5.84576	6.1462	7.44881	7.39787
ARHGAP31	4.92479	5.20913	4.59701	4.83807	4.19065	4.51969
ARHGAP32	4.5512	4.59311	4.75164	4.77152	5.31518	5.2677
ARHGAP33	0.0954519	-0.354151	1.77651	1.55655	0.270529	-0.536593
ARHGAP35	6.78764	6.77662	6.70424	6.83896	6.98703	6.61182
ARHGAP39	0.146525	-0.418273	0.770744	0.28359	0.815493	0.33778
ARHGAP42	0.639232	0.675861	4.11238	4.09312	-1.70566	-2.53597
ARHGAP5	7.5745	7.62632	5.92013	5.93171	6.5555	6.96633
ARHGAP5-AS1	2.59185	2.78747	-0.110041	-0.131402	0.0181214	-0.706491
ARHGAP9	-2.79121	-2.87684	1.55165	1.57771	-4.65668	-3.12051
ARHGDIA	5.33879	5.03346	4.37005	4.41762	6.06436	5.96698
ARHGDIB	-1.27248	-0.604659	2.75017	3.37297	-3.85089	-2.53597
ARHGEF1	4.10988	3.93909	3.94189	4.11396	4.5171	4.66069
ARHGEF10	4.25293	3.93589	4.53243	4.54132	3.97177	3.17899
ARHGEF10L	-5.69162	-4.87366	2.58798	2.69854	3.23299	3.73
ARHGEF11	6.86131	6.68864	4.72335	4.7569	4.77328	4.60389
ARHGEF12	7.09118	7.1971	7.36151	7.67021	7.19227	7.29644
ARHGEF17	4.1735	3.70828	5.22309	5.02338	3.47401	2.92992
ARHGEF18	3.54657	3.44765	3.33272	3.72218	3.95503	4.11455
ARHGEF19	1.78476	1.33161	1.30034	1.79207	-1.65987	-1.38442
ARHGEF2	5.31003	5.1122	5.28407	5.66964	4.88706	4.74655
ARHGEF25	1.70991	1.22667	2.6549	2.53896	1.02984	1.08187
ARHGEF26	3.75719	3.59903	2.46035	2.02394	2.96523	2.61071
ARHGEF28	4.83764	5.49317	5.13049	5.69044	3.71255	4.07629
ARHGEF3	1.33988	1.00482	1.69303	2.03075	4.0676	4.42385
ARHGEF37	0.133932	0.122235	-0.973801	-1.10853	1.98717	1.0747
ARHGEF39	2.91893	2.63315	2.1561	2.28811	4.10267	3.54714

ARHGEF4	2.11472	2.3413	0.460428	0.317536	6.15446	5.89504
ARHGEF40	5.06704	5.21083	3.52816	3.65669	2.60577	3.29666
ARHGEF6	0.915697	0.980157	1.7995	2.35471	3.27338	3.25908
ARHGEF7	4.29527	4.21135	4.3498	4.33618	4.55764	4.06477
ARHGEF9	4.06579	3.88144	3.4531	3.38303	1.5829	1.83247
ARID1A	5.4497	5.17694	5.19314	4.96398	5.33755	4.96835
ARID1B	4.69951	4.49474	4.86747	4.77362	5.21192	4.86975
ARID2	5.87005	5.77014	5.35757	5.29098	5.18987	4.77305
ARID3A	2.46332	2.19539	2.44948	2.43808	2.31984	0.951558
ARID3B	1.38375	1.49047	1.37636	1.21092	1.87744	0.983583
ARID4A	4.15501	4.1032	3.63027	3.78295	3.53568	3.47071
ARID4B	5.48689	5.56101	5.76812	5.94734	5.63372	5.71098
ARID5A	1.8517	1.61401	1.19156	1.42259	1.79504	1.76905
ARID5B	4.57412	4.22976	6.25008	6.38152	6.16561	6.04189
ARIH1	5.55882	5.52856	5.95506	6.10624	5.87405	5.78695
ARIH2	6.24348	6.20589	5.24984	5.42118	6.42964	6.40171
ARIH2P1	1.05002	1.38023	0.0468263	0.621871	1.58604	1.60621
ARL1	4.88916	5.05234	4.48003	5.00685	4.6129	5.29117
ARL10	3.75933	4.2219	2.43488	2.67582	-1.37455	-2.24661
ARL13B	3.13223	3.15003	3.25942	3.47425	2.42981	2.74862
ARL14EP	3.83449	3.80536	3.53072	3.58166	3.83603	4.29328
ARL15	2.39369	2.63519	2.37873	2.26623	1.41119	1.86317
ARL16	3.45225	3.55873	3.39317	3.74379	4.37481	4.66178
ARL17A	4.71663	4.62445	4.77658	4.68226	3.42755	2.71782
ARL17B	0.590454	0.65976	1.67794	1.55435	0.0870826	-0.662243
ARL2	4.85832	4.49713	3.40309	3.54233	2.92169	3.37893
ARL2-SNX15	2.08967	2.2801	1.77045	1.6519	1.49819	1.83852
ARL2BP	3.83192	3.93806	4.04682	4.7012	5.4433	5.81241
ARL3	2.68247	2.67159	2.79948	3.04346	3.15827	3.70055
ARL4A	3.42801	3.64339	3.24226	4.06072	5.45924	6.10234
ARL4C	4.96538	5.05232	3.25111	3.84828	7.35708	7.23486
ARL4D	1.32887	0.929533	3.35564	3.78599	0.197777	-0.280287
ARL5A	5.84866	5.87911	4.88651	5.12051	5.97508	5.92433
ARL5B	5.26669	5.4052	4.29119	4.56359	5.35718	5.36809
ARL6	2.49569	2.79926	3.00096	3.17195	2.54916	2.6899
ARL6IP1	7.00966	6.27675	7.24238	6.89856	6.80359	6.79884
ARL6IP1P2	0.564583	-0.484487	0.274614	0.304359	0.220578	-0.00767967
ARL6IP4	4.97297	4.90469	4.5806	4.82533	4.81522	5.06617
ARL6IP5	5.71823	5.99991	5.40705	5.85623	5.89979	6.37309
ARL6IP6	3.76137	3.68436	3.52038	3.65272	3.69284	3.53169
ARL8A	2.77008	2.60231	2.58587	2.95653	3.26668	3.08432
ARL8B	5.05529	5.10792	4.44303	4.99248	5.27294	5.59876
ARMC1	4.36901	4.62793	4.44398	5.0939	4.46961	4.86218
ARMC10	3.86099	4.05402	3.38884	3.68711	4.32042	4.81169
ARMC10P1	1.67717	1.971	0.99324	1.49903	2.154	3.06559
ARMC2	-0.376978	-0.195907	0.488988	0.100749	0.448309	-0.151977
ARMC4	?	?	-4.18092	-2.64851	1.08794	1.37014
ARMC5	0.121267	0.0358491	0.123295	0.740479	0.490652	0.793954
ARMC6	3.73851	3.59489	2.60112	3.0717	4.55014	4.96835
ARMC7	1.73974	1.84552	2.09727	2.22658	3.01389	3.0178
ARMC8	4.40223	4.44404	4.10879	4.02688	4.47346	4.73829
ARMC9	3.61833	3.47967	4.9026	5.26838	3.08789	3.10386

ARMCX1	3.25948	2.92086	0.597917	1.02051	2.2008	2.2475
ARMCX2	1.88591	2.10703	1.70525	2.49481	-2.07475	-0.662104
ARMCX3	4.55652	4.51443	4.26562	4.55205	4.81036	4.88201
ARMCX4	3.24636	3.19893 ?		-6.09892	2.29681	1.9868
ARMCX5	3.42543	3.50289	3.45406	3.36076	3.50416	3.63431
ARMCX6	3.06305	2.9431	2.89211	3.22102	3.4035	3.66672
ARMCX7P	-0.576682	0.403017	-0.024631	0.717482	0.584828	0.217975
ARMT1	3.48704	3.60056	3.44402	3.91631	4.41314	5.07793
ARNT	4.43212	4.42969	4.12025	4.39896	4.54487	4.39788
ARNT2	4.35421	4.66238	3.9894	4.5074	-2.41163	-2.38405
ARNTL	3.10344	3.00273	3.30231	3.41082	3.21621	3.11347
ARNTL2	3.89056	4.40906	4.15744	4.86773	4.29679	4.90338
ARPC1A	5.91915	5.7658	5.4835	5.85931	6.12275	6.11423
ARPC1B	5.37287	5.36945	4.40158	5.02902	4.58715	5.40179
ARPC2	5.90798	5.89331	6.11882	6.52098	6.83738	7.11167
ARPC3	5.58595	5.64188	4.77321	5.3202	5.64725	6.55245
ARPC3P1	1.06794	1.23106	0.829452	1.16238	0.977302	2.07708
ARPC3P5	0.523241	0.806374	0.133616	0.406112	0.876338	1.44848
ARPC4	5.44691	5.47498	4.82099	5.27985	6.20858	6.63848
ARPC4-TTLL3	1.11464	0.633059	0.532706	1.04661	2.27928	2.52278
ARPC5	6.02903	6.29952	5.45537	5.99676	6.33441	6.95509
ARPC5L	3.94116	3.92193	3.21949	3.46481	4.10207	4.16672
ARPIN	4.23947	4.22048	4.58291	4.82773	0.619347	0.522809
ARPP19	6.87686	6.93917	6.09878	6.52996	7.40064	7.57014
ARRB1	1.33439	1.24456	4.93021	4.75612	-3.6585	-5.69922
ARRB2	3.26526	3.07826	1.90396	2.06112	3.76385	3.84018
ARRDC1	2.32607	2.07245	1.74725	1.98923	2.24359	1.94436
ARRDC1-AS1	2.11792	1.96344	2.40895	2.68885	2.3841	2.5925
ARRDC2	2.01175	2.08792	2.37491	2.8337	2.34252	2.22543
ARRDC3	4.52362	4.19298	5.97161	5.95258	6.36235	5.6275
ARRDC3-AS1	0.335998	-0.0589688	1.56441	1.37378	0.716253	-0.0314152
ARRDC4	1.30097	0.645757	3.87852	2.94103	-1.85245	-5.69922
ARSA	1.93757	1.41371	2.86633	3.0153	0.384182	0.293394
ARSB	4.12349	4.32261	4.19152	4.71135	4.37997	4.83921
ARSD	1.40425	1.41371	3.39579	3.94374	2.85538	3.27913
ARSE	1.95915	2.15188	1.13846	1.51326	0.723471	0.80115
ARSG	0.937359	1.19649	1.43758	1.93823	-0.704754	-0.192683
ARSJ	4.31284	4.11175	4.16298	4.64187	6.25293	6.39228
ARSK	2.82645	2.90567	3.25942	3.33412	3.00147	2.87072
ARV1	3.44431	3.38735	2.87987	3.34445	4.25332	4.66722
ARVCF	0.520176	0.503023	0.770744	0.383121	-0.905167	-0.899103
ASAH1	4.56477	4.37794	4.55878	4.88055	4.91666	5.18886
ASAH2	0.454123	0.723611	0.269787	-0.0962012	0.886659	1.20515
ASAH2B	1.55205	1.75319	0.939407	1.24387	2.94989	2.97484
ASAP1	5.27391	5.32668	6.16574	6.267	6.30797	6.26553
ASAP2	5.45528	5.66817	4.63038	4.74353	4.71977	4.6021
ASAP3	2.46332	2.00479	3.38444	3.55652	3.58643	3.4607
ASB1	5.15617	4.97073	4.39296	4.41375	6.47508	5.78863
ASB13	3.40552	3.49571	2.08328	2.30904	3.06591	3.65084
ASB14	1.02318	1.17661	0.805235	0.751361	1.84242	1.08851
ASB16-AS1	0.0387873	0.055973	1.66002	1.95989	1.75171	1.04823
ASB18	0.640852	0.756391	0.711372	-0.108685	-4.65668 ?	

ASB3	3.91521	3.80367	3.73432	3.94171	4.0063	4.17181
ASB5	?	-5.45651	3.09725	4.18118	-5.65305	?
ASB6	3.93598	3.96498	3.2385	3.71389	3.92416	4.19915
ASB7	3.87919	3.88589	3.53587	3.83807	3.83939	3.59249
ASB8	3.54646	3.54277	3.36723	3.5476	3.55886	3.72373
ASB9	2.84589	2.82025	2.54441	2.86846	1.05399	1.53173
ASCC1	3.32249	2.88675	3.03384	2.90008	4.03032	3.84649
ASCC2	3.70819	3.42285	4.02471	4.27595	4.43045	4.11665
ASCC3	6.09857	6.25074	5.25422	5.63432	6.43836	6.83316
ASF1A	3.647	3.80596	3.67257	3.93596	5.05839	5.54911
ASF1B	4.43148	4.39901	4.11224	4.5296	4.4278	4.54831
ASH1L	6.93023	6.82919	6.61061	6.54088	6.75328	6.51872
ASH1L-AS1	1.01603	1.05951	0.638343	0.810682	0.00751128	0.171053
ASH2L	3.89425	3.8484	3.37872	3.72846	3.24254	3.51969
ASIC1	2.54648	2.33158	1.80519	1.0511	0.688494	-0.0918619
ASL	2.51011	2.41638	2.9614	3.60958	3.39427	3.93098
ASMTL	-0.144345	-0.531913	2.78014	3.14359	0.293994	0.668401
ASNA1	4.61323	4.49932	3.94705	4.42255	5.25332	5.93803
ASNS	4.41205	5.08067	3.45853	4.28348	4.71582	4.85743
ASNSD1	4.76135	4.7008	3.69758	3.92183	5.29355	5.98849
ASPH	7.56291	7.25502	9.58566	9.73782	8.57429	8.07856
ASPHD1	2.63763	1.99135	1.1323	1.28536	-2.12536	-2.6451
ASPHD2	2.53804	2.74418	1.25378	1.68562	0.665284	0.878827
ASPM	8.01027	7.91122	7.53989	7.71829	7.39223	7.439
ASPN	0.255223	0.592522	0.882635	0.248826	-1.26766	-1.61882
ASPSCR1	2.72089	2.45305	1.0357	1.36695	3.04013	3.41741
ASRGL1	1.78475	1.67633	0.0833019	-0.0450533	0.197777	-0.157747
ASS1	?	?	0.94542	2.24667	-1.26766	-0.899103
ASTE1	1.5788	1.63409	2.38451	2.15263	2.32295	2.4673
ASTN2	1.46083	1.35098	1.73537	1.53507	1.11453	0.80115
ASUN	4.44938	4.6153	3.87311	4.34854	4.46638	4.62755
ASXL1	7.0464	6.91792	6.76304	6.77191	6.76216	6.24017
ASXL2	5.87974	5.91601	4.97319	5.08838	5.85563	5.74963
ATAD1	5.14039	5.39659	4.33217	4.70644	5.12017	5.69844
ATAD2	6.07402	6.2796	6.1193	6.53383	6.07079	6.39026
ATAD2B	4.63128	4.59985	4.17732	4.15326	4.46315	4.3472
ATAD3A	3.91943	3.84404	3.57162	3.82297	4.24308	4.27677
ATAD3B	4.50962	4.26012	4.04872	4.3539	4.15147	3.86248
ATAD5	5.12563	5.02504	5.39538	5.32928	4.91482	4.7781
ATAT1	1.61837	1.46201	1.48185	1.56364	2.79503	2.65962
ATE1	4.21815	4.42806	3.73009	4.18063	4.79244	5.24452
ATF1	3.54171	3.39358	3.07858	3.08839	3.43377	3.16057
ATF2	5.26956	5.36011	5.81679	5.91619	5.22575	5.2445
ATF3	2.16822	2.17017	0.711372	1.59165	2.3977	2.60618
ATF4	6.65958	6.62449	6.30855	6.92197	6.53256	6.84778
ATF4P3	0.0947766	0.382213	0.219847	0.377857	0.369494	0.16203
ATF4P4	0.830466	1.17342	0.296992	1.19766	0.850295	0.967014
ATF5	2.30719	2.02413	1.75442	1.76904	1.93174	1.7002
ATF6	6.4949	6.61912	5.61373	6.08715	6.13795	6.53377
ATF6B	4.14169	4.09653	4.12823	4.28562	3.96813	3.79114
ATF7	3.39395	3.19091	3.29692	3.19228	3.16734	3.17346
ATF7IP	5.49413	5.39861	6.09083	6.00783	5.62623	5.17056

ATG10	1.59181	1.82484	2.57163	2.89941	2.26958	2.63106
ATG101	5.08022	5.26169	3.56675	4.19265	3.9185	4.64843
ATG12	4.78406	4.80908	5.43527	5.72009	5.4367	5.56696
ATG13	4.49977	4.49121	4.00854	4.5159	5.11965	5.14476
ATG14	4.05162	4.11834	3.23644	3.19952	3.39362	3.55772
ATG16L1	4.99918	5.10463	4.72036	5.22711	5.2166	5.17669
ATG16L2	2.97347	2.91992	3.32403	3.63856	1.25182	1.05981
ATG2A	3.36969	3.025	3.21949	3.2575	2.65258	2.78515
ATG2B	5.70328	5.68937	4.65882	4.80031	5.24016	5.34991
ATG3	3.98654	4.12305	4.20233	4.72327	4.15267	4.44936
ATG4A	2.3316	2.34774	2.32824	2.58467	3.16942	3.5833
ATG4B	3.66833	3.51586	3.88256	4.12599	3.77744	3.84787
ATG4C	3.00482	2.9529	2.62384	2.7677	2.92466	3.38869
ATG4D	1.55114	1.311	1.43259	1.69803	3.02768	3.03488
ATG5	3.93028	3.90457	4.10072	4.4313	5.36412	5.85743
ATG7	4.94302	5.00306	4.19546	4.58216	4.40545	4.72049
ATG9A	5.23104	5.10613	5.55979	5.68105	6.13709	5.60691
ATHL1	2.41467	1.9295	3.42011	3.31953	0.976378	0.893261
ATIC	4.55003	4.58904	3.65095	3.74448	4.29824	4.47319
ATL1	2.39945	2.51475	1.60501	2.16758	0.877744	1.16937
ATL2	5.80474	5.91324	4.89728	5.1702	5.20774	5.53108
ATL3	7.12375	7.11025	6.12247	6.59462	6.49574	6.54681
ATM	5.06318	5.1995	6.81247	6.96701	6.42552	6.89456
ATMIN	5.77981	5.81872	5.38801	5.69807	5.52875	5.53641
ATN1	3.34941	2.85683	4.09724	3.86702	3.30692	2.89692
ATOH8	0.278306	-0.604659	1.52387	1.53507	-1.85245	-0.536593
ATOX1	4.1805	4.4236	3.93541	4.26294	3.96722	4.56357
ATP10B	2.5441	2.86359	-1.01332	-0.899117	-5.65305	?
ATP10D	3.34534	3.87905	5.27027	5.5515	4.91769	5.23999
ATP11A	?	-6.45019	5.00937	5.04472	0.427241	-0.00614162
ATP11B	5.98609	5.99513	5.536	5.62007	6.51424	6.39613
ATP11C	5.1786	5.18055	5.09201	5.15619	6.46249	6.74267
ATP13A1	4.61323	4.51943	4.07504	4.06983	4.97176	4.89133
ATP13A2	3.62182	3.37131	3.31154	3.25216	4.11091	4.25111
ATP13A3	7.25989	7.31849	6.90837	7.15496	7.48469	7.56631
ATP1A1	9.06113	8.92325	8.0809	8.45164	10.2405	10.1439
ATP1A1-AS1	3.19306	3.17016	2.45028	2.62839	4.29453	3.96503
ATP1B1	5.40519	5.63345	7.6225	7.73595	6.28105	6.42079
ATP1B1P1	0.00148521	0.166625	1.41643	1.22214	0.448309	0.247587
ATP1B3	6.30451	6.127	6.49771	6.80693	6.06177	6.15167
ATP1B3P1	1.72526	1.81366	2.19631	2.42505	1.48217	1.34728
ATP2A2	7.88271	7.84699	7.62158	7.60271	6.88152	6.73414
ATP2B1	6.33292	6.27672	5.37296	5.69568	6.62361	6.44111
ATP2B4	6.07956	6.53418	6.06708	6.64836	6.03096	6.75609
ATP2C1	5.71311	5.89393	5.9163	6.13646	5.98622	5.93811
ATP5A1	7.82649	7.73212	7.24136	7.65936	7.11511	7.45044
ATP5A1P1	1.66602	1.58678	-3.13615	-2.87089	-0.299285	-0.434574
ATP5A1P3	1.85582	1.96934	1.34276	1.82962	1.4339	1.14942
ATP5B	6.81587	6.75332	7.49189	8.06326	8.07295	8.42149
ATP5C1	5.30687	5.36496	4.87258	5.35418	5.63642	6.83731
ATP5C1P1	0.676656	0.724903	-0.330777	0.724187	1.11215	1.56832
ATP5D	2.23757	1.84096	1.496	1.61231	2.35331	2.89692

ATP5E	6.3265	6.31638	5.31771	5.75094	6.72668	7.86011
ATP5EP2	3.87175	3.92094	2.80197	3.36029	4.52447	5.35773
ATP5F1	5.83744	5.7776	5.59999	6.08329	6.31346	6.91707
ATP5F1P6	-0.144345	-0.233871	-0.568612	0.220441	0.246688	0.443129
ATP5F1P7	0.558395	0.251507	0.120378	0.781183	0.815493	1.37295
ATP5G1	4.93519	4.86599	4.10982	4.74127	4.28578	5.00494
ATP5G2	5.91959	5.84275	5.25256	5.5993	5.51263	6.01856
ATP5G2P4	0.477532	0.771767	-0.0319874	0.158505	0.21579	0.362823
ATP5G3	5.88254	5.8925	6.44169	6.90133	6.25128	6.87964
ATP5H	4.35918	4.4056	5.06068	5.4932	5.87039	6.7375
ATP5HP4	1.75053	1.96122	2.31331	2.35176	2.82278	3.39931
ATP5I	4.17318	4.10873	3.58374	4.04994	5.17025	5.91472
ATP5J	4.27786	4.21318	4.23873	4.54926	4.50927	5.22937
ATP5J2	5.50381	5.56298	5.22613	5.64495	5.73976	6.25696
ATP5J2-PTCD1	2.39996	2.10546	2.20117	2.18891	1.71603	1.78129
ATP5L	4.96983	4.93567	5.1506	5.54174	5.34095	6.06451
ATP5LP2	0.0938171	0.318716	0.74491	0.917875	0.847516	1.12457
ATP5O	5.00128	4.93803	5.81399	6.23325	5.47859	6.30831
ATP5S	3.63293	3.56643	2.65324	2.70501	3.5773	3.74029
ATP5SL	3.72928	3.50581	3.34004	3.65605	4.1341	4.22844
ATP6AP1	5.44523	5.29286	5.08558	5.19437	6.19686	6.07053
ATP6AP1L	-0.509064	-0.333394	1.37111	0.879925	0.529601	-0.247125
ATP6AP2	4.98285	4.94386	6.00653	6.30803	5.28693	5.43975
ATP6V0A1	4.78678	4.74904	4.97445	5.10733	4.83753	4.51728
ATP6V0A2	4.77746	4.71527	3.73384	4.09216	4.43984	4.2527
ATP6V0B	5.73865	5.84093	5.20826	6.03583	5.71176	6.67319
ATP6V0C	3.31106	3.07524	2.9618	3.06189	3.1943	3.0236
ATP6V0D1	4.56418	4.35413	4.90525	5.16845	5.89954	5.76444
ATP6V0E1	5.16897	4.95449	5.79944	6.21363	6.67491	7.12126
ATP6V0E2	3.97915	4.08056	3.48772	4.03247	4.97488	5.06475
ATP6V0E2-AS1	1.06527	0.800364	1.58249	1.50312	0.26052	0.401903
ATP6V1A	5.81539	5.81966	6.69218	7.14413	6.31033	6.22367
ATP6V1B2	5.84001	5.74267	4.89261	5.27972	5.64574	5.79014
ATP6V1C1	5.30091	5.21001	5.2651	5.75613	5.90119	6.22586
ATP6V1C2	3.54029	3.51586	2.81241	2.78491	3.10812	2.8156
ATP6V1D	4.38424	4.26355	3.42433	3.86343	4.60516	5.29719
ATP6V1E1	5.56494	5.46579	4.81486	5.37817	5.26464	5.92268
ATP6V1E1P1	2.04111	2.00249	1.43014	1.97422	1.73782	2.08652
ATP6V1E2	2.06547	2.10988	1.48894	1.28354	1.17845	1.28202
ATP6V1F	4.28462	4.07726	3.57467	3.98521	4.54975	5.14113
ATP6V1G1	4.57588	4.75086	3.75164	4.35674	5.09874	5.67374
ATP6V1G2-DDX39	3.25627	3.0536	2.39245	2.7812	2.21195	2.85766
ATP6V1H	4.08718	4.12587	4.1111	4.51505	3.79604	4.08445
ATP7A	3.85835	4.00163	5.48269	5.88298	4.90576	5.12215
ATP7B	2.51768	2.29206	2.02595	1.72409	-0.217212	-1.53638
ATP8A1	0.0692347	0.278722	2.50997	2.68885	-2.95876	-2.70579
ATP8A2	1.60016	2.08792	-0.825729	-0.786664	-3.33694	-2.24661
ATP8B1	1.55412	1.59663	2.99968	3.18227	1.51115	1.53373
ATP8B2	5.72561	5.33278	4.90128	5.10427	5.08282	4.54002
ATP8B3	0.334442	0.305445	2.02595	2.02562	0.0263185	0.0483059
ATP9A	6.74074	6.75433	5.54736	5.69368	5.20696	4.87448
ATP9B	3.82349	3.70833	3.28	3.13673	2.10458	1.65964

ATPAF1	3.97863	3.91327	4.64301	4.99118	4.80423	5.22402
ATPAF2	2.24175	2.08781	2.38735	2.29731	2.80268	2.84476
ATPIF1	4.14248	4.07922	3.9406	4.21647	4.5473	4.66941
ATR	5.16014	5.30574	6.06341	6.35415	5.44436	5.85274
ATRAID	5.40774	5.20431	4.23044	4.60117	4.25179	5.25111
ATRIP	3.25415	3.27719	2.33449	2.85991	2.92813	3.02946
ATRN	6.25529	6.3132	6.15934	6.42397	5.49889	5.42834
ATRNL1	3.24636	3.22541	-3.85951	-3.10759	-4.07293	-3.12051
ATRX	6.94158	6.89277	7.41639	7.34663	7.37541	7.20597
ATXN1	5.09411	5.18009	5.8785	5.68436	5.71092	5.69629
ATXN10	5.36193	5.26954	5.21849	5.43645	4.6938	5.38654
ATXN1L	4.86981	4.94934	5.24827	5.66834	5.90995	5.82795
ATXN2	4.92753	4.82139	4.8016	4.70815	4.49006	4.07628
ATXN2L	4.4307	4.19293	4.74147	4.73119	4.25767	4.30578
ATXN3	3.92869	4.02616	2.9792	3.00494	3.80049	3.96171
ATXN7	4.78724	4.74904	4.8256	4.88056	4.48625	3.84595
ATXN7L1	2.15269	2.26849	2.42751	2.26188	2.1145	1.85556
ATXN7L2	1.78073	1.81795	1.33222	1.37503	1.82756	1.91536
ATXN7L3	4.48642	4.38419	4.36143	4.65022	5.23906	5.33425
ATXN7L3B	5.62447	5.72741	4.99206	5.30564	5.194	5.10349
AUH	1.53458	1.49719	1.42385	1.71138	1.67511	1.65087
AUNIP	3.55897	3.56874	2.05117	2.51325	3.02609	3.24637
AUP1	6.22879	6.1837	5.51098	5.91141	6.10353	6.22117
AURKA	6.20713	6.13449	5.45702	6.0423	6.14176	6.33317
AURKAIP1	4.07982	4.05582	3.65568	4.02816	4.66387	5.6056
AURKAPS1	1.89251	1.92102	1.32342	1.67438	1.54192	1.33942
AURKB	4.65681	4.54418	4.02349	4.02174	4.6297	4.31704
AUTS2	-5.10914	-5.45651	3.60438	3.01012	4.28512	4.14503
AVEN	1.95558	2.041	2.02595	2.47239	2.45084	2.908
AVL9	5.62617	5.78514	5.93856	6.20112	6.39385	6.47082
AVPI1	3.52613	3.27525	1.65571	2.25313	0.121175	0.359476
AXIN1	1.83622	1.62994	2.92239	3.07108	3.06762	2.9515
AXIN2	0.852934	0.614908	2.23762	1.89984	-0.808639	-1.31405
AXL	7.79948	8.03509	7.09698	7.49	7.46898	7.72795
AZI2	4.54169	4.51364	3.94586	4.26483	4.76259	4.98263
AZIN1	6.41188	6.37341	6.59701	6.87413	6.53275	6.64478
AZIN2	-0.191134	-0.0876659	2.14293	2.11036	0.479334	0.736302
B2M	8.08041	8.11475	9.35168	9.5134	9.00866	9.80391
B3GALNT1	2.7726	3.459	-4.59508	?	0.258664	1.32676
B3GALNT2	4.16973	4.1792	3.77647	3.86558	4.22157	4.07792
B3GALT1	1.69693	1.99454	-6.17309	-5.10387	-0.11254	1.15132
B3GALT2	3.63407	3.54418	2.1019	1.87419	1.50965	1.20023
B3GALT4	0.345419	0.537236	-0.252626	-0.464785	-0.0903144	-0.151977
B3GALT6	4.17743	3.84321	3.30231	3.20859	3.64119	3.24457
B3GAT2	1.30209	1.33102	0.121572	0.191323	1.23967	0.970405
B3GAT3	4.21977	4.08337	3.3358	3.68038	4.69431	5.08677
B3GLCT	3.53334	3.80159	3.32625	3.41865	3.8769	4.36608
B3GNT2	4.20703	4.17647	4.29423	4.58292	4.44952	4.36207
B3GNT4	0.775346	1.41004	0.90829	0.84608	0.909174	-0.0765359
B3GNT5	4.6478	5.01639	1.84293	2.39073	4.15978	4.12092
B3GNT7	5.9416	5.68181	-2.27595	-1.64896	-3.33694	-3.12051
B3GNT9	1.00568	0.835124	3.47502	3.69596	4.02785	4.08465

B3GNTL1	2.48697	2.42835	2.93557	2.98987	2.83837	2.78916
B4GALNT1	4.11119	4.1481	5.62407	5.7025	4.2867	3.94124
B4GALNT3	1.9769	1.93378	-6.17309	-6.09892	0.997957	0.151378
B4GALNT4	3.06579	2.60325	2.39769	2.2086	-0.572647	-0.706491
B4GALT1	4.37573	4.37083	4.58051	4.89258	6.62667	6.62307
B4GALT2	4.57063	4.39201	4.35949	4.67335	4.49324	4.50638
B4GALT3	5.09498	5.20328	3.68521	4.18022	4.75077	4.89531
B4GALT4	3.69752	3.28534	3.50295	3.13186	2.96358	2.48312
B4GALT5	5.98478	6.06755	6.42795	7.01899	5.75489	5.52149
B4GALT6	5.45163	5.60323	3.95346	4.33307	-2.65941	-1.70626
B4GALT7	2.91564	2.55119	3.63025	3.77991	3.16624	3.24749
B4GAT1	4.59955	4.31523	4.16851	4.41277	3.47411	3.65523
B9D1	1.43895	0.950117	1.91271	2.15412	1.6678	2.34331
B9D2	-0.509064	-0.233871	-0.095919	-0.585061	0.427241	0.951558
BABAM1	4.73293	4.80686	4.24483	4.56448	5.68564	6.13562
BACE1	5.54113	5.30702	5.19907	5.12157	5.04328	4.97969
BACE1-AS	0.323578	0.169194	0.14659	0.237233	0.0752314	-0.414318
BACE2	6.33035	6.75146	5.0955	5.17542	3.87884	4.69629
BACH1	6.08696	5.95977	5.96969	6.08409	4.35301	4.40442
BACH1-IT2	0.50068	0.292146	0.805235	0.46112	-2.26731	-1.61882
BACH2	1.88591	1.86673	-0.69145	-0.40821	0.870676	0.171745
BAD	2.54987	2.27606	2.43257	2.79269	2.60887	3.51464
BAG1	3.18814	3.12212	2.69913	3.03584	4.54913	5.09499
BAG2	4.29369	4.40611	4.72844	4.95797	6.0196	6.25468
BAG3	3.71243	3.68817	4.54523	4.901	5.07776	5.00752
BAG4	4.79338	4.92177	4.16883	4.69538	4.48059	4.51966
BAG5	4.98653	5.04396	4.06914	4.34445	5.30979	5.21437
BAG6	5.39803	5.31836	5.25825	5.4286	6.121	6.28558
BAGE2	4.03053	3.90191	-2.07396	-2.28557	-2.72209	-2.78024
BAHCC1	2.49815	2.09942	3.2088	2.96799	-1.90489	-2.8983
BAHD1	3.7938	3.51565	2.79974	2.99312	3.2941	3.13843
BAIAP2	2.57863	2.27026	2.693	2.90201	2.2785	2.34315
BAIAP2-AS1	3.1243	2.68437	3.48182	3.55652	2.8134	2.10709
BAIAP2L1	4.40812	4.38211	2.98989	3.1218	0.736804	0.0747657
BAK1	3.33351	3.07152	3.04991	2.95364	3.80461	3.60522
BAK1P1	0.16651	-0.323864	0.121864	-0.123527	0.62174	0.43127
BAMBI	6.34287	6.36011	5.93633	5.7213	3.51959	3.61522
BANF1	4.80555	4.44128	4.27542	4.16668	4.07559	3.49595
BANP	3.18891	3.0604	2.90844	2.82619	2.3334	2.54094
BAP1	5.12706	5.10082	4.81788	5.13434	5.65511	5.79958
BARD1	4.20479	4.38811	5.33118	5.58421	4.62103	5.0015
BASP1	?	-5.45651	5.01798	5.48641	-6.64581	?
BATF3	0.96632	1.01295	-0.0541032	0.736735	0.448309	1.34861
BAX	4.33295	4.23412	3.62543	3.80257	4.36274	4.10385
BAZ1A	5.67927	5.62902	4.75283	4.96031	5.82216	5.69877
BAZ1B	7.25164	7.35051	7.06022	7.39805	7.31337	7.23455
BAZ2A	4.96516	4.83684	6.00533	5.9591	5.49849	5.01777
BAZ2B	5.12557	5.21725	4.59034	4.33495	5.81111	5.84753
BBC3	0.460879	0.0291348	0.138526	0.632724	0.0540566	0.33778
BBIP1	3.25263	3.22603	3.60142	3.70828	4.29175	4.27626
BBS1	2.49419	2.40831	2.82742	2.8983	2.14956	1.69097
BBS10	3.37104	3.27694	3.16521	3.17658	2.85933	2.81686

BBS12	1.75627	1.52587	1.32432	1.63267	1.46902	1.70271
BBS2	3.91592	3.96737	4.23853	4.18734	4.52781	4.69871
BBS4	3.30374	3.35094	3.31433	3.4284	3.52951	4.14971
BBS5	1.35938	1.13027	-0.406801	0.0430925	0.693516	1.01645
BBS7	2.84975	3.21664	3.06678	3.4515	2.94887	3.4582
BBS9	2.64966	2.57334	2.77068	2.85696	2.47669	3.06148
BBX	6.13479	6.18012	6.27923	6.67804	6.46182	6.53436
BCAM	0.66732	0.370142	2.61739	2.50589	1.26455	1.76092
BCAN	0.0961134	0.791231	-0.00291864	0.191727	0.269249	-0.171362
BCAP29	5.65673	5.60745	4.48285	4.79624	5.20251	5.48546
BCAP31	6.2735	6.23738	6.488	6.76575	7.2765	7.19189
BCAR1	3.76738	3.43679	4.62424	4.67644	3.88826	3.9315
BCAR3	4.18911	4.17514	4.62989	5.43302	4.48514	4.57721
BCAS2	4.21864	4.38769	3.32918	4.06023	5.83098	6.34878
BCAS2P2	0.38363	0.671973	0.0931274	-0.0710105	1.63472	2.26031
BCAS3	3.27523	3.00066	3.06176	3.0483	3.23451	3.07465
BCAS4	2.5303	2.6931	4.56379	4.6821	4.09865	4.69297
BCAT1	6.33316	6.22997	7.04651	7.60689	7.96152	7.78949
BCAT2	3.03224	2.66387	3.76338	3.75845	4.02356	4.15282
BCCIP	4.99911	5.01058	4.70936	5.18924	5.53128	5.9193
BCDIN3D	1.79365	1.70067	1.8627	1.97737	1.66897	2.13124
BCDIN3D-AS1	0.602837	0.815985	0.533484	0.552938	-0.0254193	-0.671095
BCHE	2.54885	2.72946	-6.17309	-6.09892	5.65623	6.16519
BCKDHA	1.50511	0.644429	2.10348	2.74935	2.35694	2.35401
BCKDHB	3.16975	3.24452	2.97383	3.0636	3.47026	3.44049
BCKDK	3.03394	2.83866	3.17403	3.26839	3.7981	3.62421
BCL10	3.35892	3.34612	3.46034	3.96933	3.98534	4.12776
BCL2	3.63184	3.46642	0.976408	1.40691	1.46385	1.23585
BCL2L1	4.54367	4.76962	4.46254	5.09429	4.84168	5.03256
BCL2L11	3.08065	3.04693	3.33415	3.17889	3.59942	3.60843
BCL2L12	2.20017	2.13852	1.73672	2.08552	2.82128	2.85456
BCL2L13	5.26417	5.26038	5.5533	5.80891	5.547	5.60786
BCL2L2	4.32044	4.3065	3.52263	3.78037	4.34859	4.08431
BCL2L2-PABPN1	0.51922	0.067625	0.217988	0.362318	0.695477	1.20474
BCL3	2.33986	2.26169	2.12483	2.3424	1.70604	1.72795
BCL6	4.93363	4.60839	4.50643	4.63566	4.11317	3.78431
BCL7B	3.94116	3.92086	3.60602	4.0994	4.38339	4.60503
BCL7C	2.29656	2.46486	2.36877	2.53308	2.93607	3.64835
BCL9	4.24343	4.09367	3.62705	3.35878	4.51959	4.42
BCL9L	3.10599	2.61345	4.46864	4.27784	3.51496	3.53395
BCLAF1	6.69908	6.71156	6.77272	6.94764	7.3656	7.20012
BCLAF1P2	4.68915	4.69278	4.57816	4.81388	5.30212	5.22526
BCOR	4.11149	4.14356	4.0063	4.01011	3.919	3.8421
BCORL1	2.35621	1.84096	3.28815	2.94646	2.57092	2.11348
BCR	4.22461	4.20797	4.66292	4.73679	3.53543	3.56853
BCS1L	3.56457	3.60029	3.4286	3.74434	4.01791	4.11406
BCYRN1	6.83861	6.45824	5.57956	5.33446	5.75986	5.08718
BDH1	2.01519	1.92519	2.80233	3.12	3.49197	3.51969
BDH2	2.63454	2.64674	2.65001	2.69047	1.53582	2.64745
BDH2P1	1.5854	1.67979	0.984444	1.09578	0.19439	1.34612
BDKRB2	3.69646	3.97727	0.860939	2.02567	-4.33548	-3.70484
BDNF	2.19647	2.17984	4.08251	4.62736	3.7426	4.38193

BDNF-AS	-0.102543	0.0815578	2.06276	2.14504	0.895101	1.15261
BDP1	5.91076	5.98862	6.21865	6.37017	6.88093	6.86838
BECN1	4.99076	4.99443	4.51935	4.88745	5.44713	5.46365
BEND3	2.14956	2.18104	1.99641	1.89682	2.17259	1.76905
BEND3P1	1.85588	2.24181	-4.59508	?	-1.00108	-0.189804
BEND3P3	-0.00370816	-0.133526	-0.130602	0.0324394	0.706756	0.385641
BEND6	2.47835	2.6587	1.66826	2.44961	0.597155	2.16367
BEND7	?	?	1.20023	1.02051	0.282309	0.0747657
BEST1	3.003	2.52565	3.50421	4.10059	3.75051	4.15317
BET1	2.81762	3.124	4.11394	4.67605	3.1276	3.69203
BET1L	3.80078	3.64699	3.94834	4.21421	3.41917	3.55068
BEX1	-5.69162	-3.87578	-5.1783	-4.52056	2.51213	3.6813
BFAR	5.12389	5.16559	5.1795	5.45007	5.21888	5.60446
BFSP1	3.30934	2.9801	2.11571	2.67582	1.2586	1.56011
BGN	2.59785	1.55956	-1.09576	-4.10635	-4.65668	-3.70484
BHLHB9	1.15272	0.785157	1.77651	1.8512	3.01566	3.14816
BHLHE40	3.45194	3.53574	4.3939	4.63263	6.10826	5.51607
BHLHE41	2.38173	3.2184	0.782333	1.28354	3.1673	3.1322
BICC1	3.94116	4.13985	4.60926	5.21815	4.83154	5.09336
BICD1	5.24375	5.33439	4.89929	4.91353	6.24806	5.82417
BICD2	4.75569	4.86244	4.45219	4.86344	4.44297	4.34584
BID	3.85742	3.57744	4.2912	4.66431	3.69504	4.25618
BIN1	3.91287	3.71707	4.60682	4.90029	3.15507	3.25329
BIN3	5.52146	5.49047	3.10415	3.43253	2.61454	2.6183
BIRC2	4.68192	4.70269	5.77863	6.22985	5.17691	5.39657
BIRC3	5.10102	5.63684	4.7685	6.08159	2.53446	2.96571
BIRC5	6.45902	6.34468	5.82316	6.25694	6.50547	6.66283
BIRC6	7.96027	7.97734	7.2875	7.4173	8.09013	8.02631
BIVM	3.49769	3.37	4.2357	4.17116	3.65059	3.36074
BIVM-ERCC5	1.38555	1.59078	2.25358	2.77081	2.21566	2.68332
BLACAT1	1.04917	1.60865	-2.01303	-2.02079	1.45867	1.15132
BLCAP	4.15268	4.08212	4.84022	5.26238	3.98354	3.99719
BLID	1.8934	1.39328	1.5714	1.51835	0.529601	0.832509
BLM	5.11869	5.18461	4.59126	4.7288	3.83054	4.01438
BLMH	4.81069	4.86302	4.85607	4.93898	5.37653	5.39394
BLOC1S1	3.75729	3.92171	2.83934	3.1912	3.51997	4.09945
BLOC1S2	3.48083	3.53855	3.15856	3.65273	4.48751	5.02967
BLOC1S3	0.816493	0.559609	1.17362	1.75527	0.661695	0.443129
BLOC1S4	2.78676	2.65351	1.59787	2.02562	2.54915	2.31567
BLOC1S5	2.60683	2.55814	2.74402	3.00588	3.30482	3.64241
BLOC1S5-TXNDC5	5.27215	5.26654	4.75465	4.89875	5.86033	5.67854
BLOC1S6	5.28104	5.34729	4.50556	4.8812	5.38575	5.72579
BLVRA	3.28818	3.18641	4.15633	4.56711	1.99435	1.99375
BLVRB	1.88215	1.79457	5.12651	5.51414	2.76176	3.59707
BLZF1	4.40158	4.56367	3.77212	4.23989	4.82046	5.65523
BMF	3.06413	1.89913	3.13841	2.41475	3.04533	2.19417
BMI1	4.00688	4.03227	3.56759	3.76244	4.56601	4.48003
BMP1	4.09126	3.81444	3.93411	3.88694	4.58404	4.25039
BMP2	2.14329	2.25827	-0.757028	-0.353769	-5.07051	-4.70296
BMP2K	4.74228	4.86919	6.11639	6.12682	5.04002	4.98047
BMP2KL	-2.16231	-1.59638	0.679262	0.770643	0.283104	0.432489
BMP4	0.219896	0.151976	2.95604	2.44961	-0.682812	-0.848487

BMP6	0.802814	1.18901	-1.78917	-2.64851	2.55159	2.67674
BMP7	?	-6.45019	2.759	3.02046	-6.64581	-5.69922
BMP8B	5.05499	5.50607	-0.310119	-0.254069	-0.533088	-0.583854
BMPR1A	4.91345	4.90806	4.71006	4.81441	5.59798	5.53812
BMPR1APS1	2.07256	2.18833	1.8026	1.97982	2.74193	3.09237
BMPR1APS2	2.1887	2.27712	1.89902	2.18989	2.93634	3.13487
BMPR1B	1.30659	1.09179	?	-5.10387	3.93402	4.17592
BMPR2	5.22717	5.28159	5.53498	5.54983	5.92378	5.63869
BMS1	5.89462	5.90678	5.27755	5.60777	5.94161	5.77603
BMS1P1	1.28806	1.21225	2.14794	1.6715	1.79459	0.926379
BMS1P2	1.42961	1.30921	1.8753	1.87758	1.41486	1.21383
BMS1P20	1.21233	1.17295	1.05185	1.13566	1.10052	0.170258
BMS1P22	1.41787	1.68159	0.582614	0.703685	0.104538	-1.4996
BMS1P4	1.16882	1.38401	1.56308	1.4822	1.69447	1.34217
BNC2	4.64244	4.73498	2.53073	2.50957	6.16721	6.17439
BNIP1	1.9303	2.02099	2.05967	2.36692	2.36207	2.90432
BNIP2	5.79026	6.06095	5.2359	5.71174	5.98556	6.37542
BNIP3	4.21953	4.16694	5.43626	5.77982	7.38707	6.52259
BNIP3L	5.27675	4.84889	6.05039	6.20829	6.92573	6.21373
BNIP3P1	2.57785	2.56926	3.86353	4.36205	5.76907	5.34468
BNIP3P4	2.63395	2.54555	-3.18222	-6.09892	-1.45047	-1.53638
BOC	-1.2962	-1.64566	2.65537	2.66561	0.6914	0.410721
BOD1	3.77665	3.83407	4.48269	4.93761	4.31769	4.78413
BOD1L1	6.92685	6.93736	6.31207	6.62482	6.83491	6.43347
BOK	2.39105	2.18463	0.674533	0.646135	-2.07475	-2.53597
BOLA1	1.80874	1.89912	0.904002	0.924662	0.134221	0.578775
BOLA2	1.00435	0.994565	0.665829	1.28301	1.76221	2.95979
BOLA2B	3.27672	3.30148	2.74677	3.34895	4.28073	5.19476
BOLA3	2.22871	2.20251	1.5847	2.17196	3.02093	3.85933
BOLA3-AS1	-0.0842313	-0.177293	1.0357	0.999755	-0.827177	-1.006
BOP1	3.98039	3.78626	3.48714	3.78901	4.45862	4.57057
BORA	3.13278	3.09066	3.04626	3.42031	2.81586	3.42838
BPGM	3.20027	3.3653	3.3653	3.90519	3.92276	4.42514
BPHL	2.22981	2.24795	2.84554	3.00157	2.42976	2.35379
BPNT1	4.09614	4.07436	3.40707	3.74915	3.91238	4.38075
BPTF	6.15552	5.98687	6.43742	6.23891	6.95984	6.6233
BRAF	5.09478	5.09709	4.86213	4.90494	5.18861	5.11188
BRAP	3.96892	4.0139	3.1541	3.35266	3.64004	3.57869
BRAT1	3.30935	3.09558	3.76046	4.02174	3.69943	3.76089
BRCA1	5.33537	5.38811	5.64738	5.84827	5.15106	5.38206
BRCA2	5.4462	5.58256	5.7435	5.98127	4.56249	4.89597
BRCC3	3.58756	3.68585	3.74091	4.15608	4.85434	4.75004
BRCC3P1	-0.342484	-0.0735344	0.750375	0.731557	0.680585	0.261567
BRD1	3.9909	3.85908	4.39107	4.35163	3.33687	3.03813
BRD2	6.58029	6.56197	6.08058	6.28618	6.68342	6.54538
BRD3	3.68138	3.37451	3.64981	3.55273	3.01858	2.87203
BRD4	4.64577	4.52157	4.18388	4.04092	4.3997	4.29046
BRD7	5.03994	5.00882	4.97859	5.06527	5.61609	5.3355
BRD7P2	3.86886	3.91257	3.73915	3.91959	4.6848	4.44458
BRD8	5.19156	5.14679	5.59085	5.73508	5.17139	4.89412
BRD9	5.074	5.00829	4.46393	4.57328	4.8174	4.29187
BRE	4.02292	4.00826	3.25527	3.5878	3.93491	4.39788

BRF1	3.72718	3.45154	2.58798	2.57066	2.98716	2.78915
BRF2	3.08456	3.11622	3.27708	3.41526	2.37467	2.57911
BRI3	3.54649	3.32292	3.97962	4.42085	3.64513	3.6398
BRI3BP	4.62006	4.24833	3.73928	3.79224	3.83312	3.4448
BRICD5	0.947576	0.915651	1.15851	0.90465	0.19672	-0.134056
BRIP1	4.86694	4.94121	4.84368	4.95253	4.83753	4.66069
BRIX1	4.90512	5.00279	4.99876	5.37939	5.31091	5.91453
BRK1	4.90547	4.61796	4.57136	4.86055	5.8571	5.8848
BRMS1	4.17282	4.10985	3.95986	4.49108	3.80423	4.16826
BRMS1L	1.94506	2.2934	1.56404	1.82247	2.16267	2.37767
BROX	5.31017	5.49083	4.6806	5.20634	5.498	5.76689
BRPF1	4.11791	4.02297	3.48003	3.6494	3.82349	3.44304
BRPF3	3.4895	3.34772	3.95091	3.97465	3.55036	3.13878
BRSK1	2.41207	1.9508	1.63029	1.83957	2.4351	2.2475
BRSK2	2.17746	1.54281	1.24279	0.95732	-1.90489	-3.70484
BRWD1	6.8187	6.76098	7.04364	7.0312	6.86068	6.7261
BRWD3	5.33572	5.19	5.67289	5.42789	5.86543	5.51183
BSCL2	3.25313	3.08566	3.55141	3.91595	3.28996	3.89413
BSDC1	4.8915	5.00372	4.89663	5.21536	5.64034	5.80155
BSG	6.58672	6.41795	6.46463	6.65036	7.2582	7.44868
BSN	1.7928	1.40138	0.259532	0.100749	-0.777433	-1.31405
BST2	1.60474	1.45605	5.29372	5.47378	3.01741	2.86694
BTAF1	6.11789	6.0121	5.41682	5.4662	6.40397	5.98025
BTBD1	5.19422	5.46378	4.87775	5.38446	6.21864	6.83505
BTBD10	3.75606	3.92402	3.5408	4.01082	3.87274	4.41168
BTBD11	?	?	1.24279	1.73669	-3.19956	-4.11926
BTBD18	0.388476	0.151976	0.488988	0.231137	0.373208	-0.577229
BTBD19	0.388476	0.278722	2.42382	2.52054	2.42981	1.76905
BTBD2	3.26535	2.69101	3.42546	3.1835	3.38722	2.74449
BTBD3	5.62601	6.01059	3.76553	4.26756	6.31465	6.85774
BTBD6	4.56301	4.72388	2.82741	3.28349	4.09375	4.46696
BTBD7	4.76189	4.63188	4.15854	4.10428	4.8445	4.3634
BTBD9	2.78272	3.06659	2.51346	2.56714	4.50144	4.58214
BTD	3.85352	3.74441	2.90198	3.22801	3.25656	3.63672
BTF3	6.66252	6.70432	6.60846	7.17398	7.32855	7.78011
BTF3L4	4.2095	4.44597	3.8171	4.60673	5.09715	5.43512
BTF3L4P2	2.64662	2.78578	2.31821	2.70271	3.0027	3.88822
BTF3P10	3.41405	3.45104	3.46331	3.8663	4.0111	4.48841
BTF3P5	0.253554	0.725602	0.354824	0.6862	1.03721	1.18626
BTF3P7	0.952088	1.10524	1.02476	1.67465	1.53923	1.75374
BTF3P9	-0.256668	-0.0721688	0.0439604	0.28193	0.533842	0.974397
BTG1	6.50843	6.43688	5.16465	5.05414	4.12268	4.37143
BTG2	1.68466	1.59788	0.00643448	0.231137	-0.374735	0.0213376
BTG3	4.15266	3.42742	3.49413	3.47237	3.7512	3.66615
BTN2A1	3.84837	3.89344	4.37003	4.8277	4.70095	4.50649
BTN2A2	3.93592	3.8502	3.85273	3.76793	4.32142	4.06956
BTN2A3P	1.37353	1.23069	2.40145	2.45724	1.89813	1.20023
BTN3A1	2.88908	3.07966	4.49394	4.62469	4.24689	4.36443
BTN3A2	3.4389	3.41311	4.88107	5.09899	4.80075	5.20323
BTN3A3	1.78058	1.90566	4.39678	4.60547	3.70469	3.74001
BTRC	3.59209	3.83178	3.66667	4.12118	4.20462	4.4607
BUB1	5.72876	5.76174	5.29827	5.85404	5.79529	5.88807

BUB1B	6.09615	6.09333	5.14518	5.38833	5.53109	5.42386
BUB3	6.07281	6.28048	5.76701	6.29363	6.60295	6.88759
BUB3P1	-0.191134	0.151976	-0.183372	0.231137	0.107996	0.315752
BUD13	3.35621	3.2289	2.80233	2.97333	2.99434	2.98329
BUD31	4.53483	4.60124	3.91968	4.40343	4.79838	5.4524
BVES	3.75006	3.45154	3.66039	4.04851	6.21791	6.26124
BX322557.10	1.78073	1.67674	3.44035	3.46293	2.15989	2.22994
BX470102.3	0.863225	1.06077	0.324373	0.711424	-0.121008	0.597145
BX842568.2	0.411882	0.570317	0.704553	1.18222	1.74968	1.47858
BYSL	3.28961	3.28367	2.72338	3.00753	3.97903	4.06478
BZW1	6.72567	6.92025	5.76077	6.30658	6.84452	7.39927
BZW1P1	1.58796	1.90109	1.55122	1.7204	1.81672	1.69771
BZW1P2	5.4212	5.56358	4.41927	4.99762	5.48038	6.30239
BZW2	5.88988	5.91515	5.22229	5.30194	5.7565	5.91273
C10orf10	4.22647	4.23325	1.63669	2.08102	3.88078	2.56942
C10orf12	3.71137	3.58836	4.04409	4.14147	4.89858	4.57173
C10orf2	4.2667	4.29619	2.71736	2.94375	4.63888	4.49296
C10orf32	2.23615	2.10529	0.286503	0.786069	2.93206	3.18118
C10orf35	-0.998365	-1.46246	0.805235	0.913608	0.670686	0.668401
C10orf76	3.04071	3.08212	3.56299	3.66101	3.5649	3.39131
C10orf88	2.36996	2.44472	2.41621	2.82478	3.3422	3.56696
C10orf90	4.43982	4.19357	-2.86056	-1.71605	0.448309	0.33778
C11orf1	0.516277	0.722134	2.01981	2.61389	1.32143	2.46418
C11orf24	4.94116	4.74965	3.5962	4.07852	3.82752	3.76699
C11orf30	4.95599	4.9257	4.78153	4.82559	3.67396	3.18203
C11orf31	3.42245	3.31852	2.54441	2.66597	3.91239	4.57289
C11orf45	-0.549135	-0.140771	0.503064	1.40691	-3.07415	-1.38442
C11orf49	2.73764	2.82948	3.35564	3.8145	4.07864	4.22402
C11orf54	2.13383	2.07723	3.24298	3.37036	3.17258	3.22911
C11orf57	3.10731	2.97102	4.26647	4.50288	4.66915	4.93278
C11orf58	5.83018	5.95396	6.04943	6.40645	5.80061	6.09718
C11orf63	-1.14419	-1.0363	0.816551	1.35882	1.91714	2.16367
C11orf68	4.21008	4.26846	3.18933	3.79505	2.78471	2.90432
C11orf71	0.456638	-0.00188106	2.48157	2.60996	1.84244	1.70955
C11orf73	2.17622	2.13791	3.10441	3.46195	2.98108	4.01461
C11orf74	1.2996	1.30169	1.2622	1.44654	-0.682814	-0.280287
C11orf80	1.72519	1.73731	1.67551	1.73247	1.41251	1.3167
C11orf84	3.83522	3.57469	3.37872	3.6191	3.58762	3.27343
C11orf95	3.50548	3.13148	3.35176	3.41473	2.54671	1.88577
C11orf98	3.97099	4.05594	3.02731	3.29194	3.97364	4.38702
C12orf10	3.62357	3.57427	2.92066	3.41974	3.34791	3.46904
C12orf29	3.68716	3.95785	2.86748	3.25036	3.58311	4.35714
C12orf4	3.95017	4.21046	3.60438	3.92597	3.88658	4.80997
C12orf43	3.74303	3.64174	2.76485	3.17195	2.78885	2.79314
C12orf45	2.73139	2.80629	1.10193	1.60545	2.08454	2.38077
C12orf49	4.69377	4.98526	3.50298	4.11758	4.16226	4.37676
C12orf57	4.04021	3.86622	3.2474	3.91744	3.38853	4.54042
C12orf60	0.50068	0.331671	0.782333	1.01017	-0.987612	-0.4586
C12orf65	4.76903	4.72202	3.7128	3.82042	4.06931	3.17593
C12orf66	2.46584	2.35095	1.77651	2.17659	1.38958	1.44308
C12orf73	2.29449	2.13303	1.64338	1.75264	1.6392	0.264729
C12orf75	4.18128	4.68562	2.48891	3.4948	3.02269	4.05815

C12orf76	2.73712	2.62721	2.20244	2.01473	2.15973	1.8381
C12orf79	1.20821	1.20163	-0.586833	-2.10822	-1.41201	-1.45835
C14orf1	3.32468	2.92948	2.18282	2.20407	2.86522	2.83633
C14orf119	4.0926	4.26535	3.37479	3.9273	4.44818	5.25304
C14orf132	2.15892	2.23761	4.52986	4.7973	0.0540566	0.270672
C14orf142	2.06581	2.26509	1.20023	1.69211	2.48945	3.19114
C14orf159	2.62286	2.75875	4.04425	4.19635	2.51246	2.5925
C14orf166	5.63828	5.67126	4.6882	5.05068	5.43455	6.06807
C14orf2	4.46395	4.49208	3.35952	3.87274	4.28073	5.2677
C14orf79	2.49076	2.53152	1.0357	1.50592	2.20079	1.92267
C14orf80	2.6467	2.16889	1.0353	0.923149	0.448309	1.20023
C14orf93	1.78476	1.6953	1.57744	1.63865	1.04018	1.34861
C15orf38-AP3S2	3.387	3.45707	3.23408	3.57331	2.73084	2.75418
C15orf39	3.51281	3.53997	3.44947	3.38503	2.65937	2.41742
C15orf40	3.52321	3.58524	3.35644	3.50349	3.31731	3.79853
C15orf41	2.86698	2.89031	0.893354	0.563685	2.37862	2.16982
C15orf52	5.75315	5.7821	4.04409	4.63684	4.27633	4.0465
C15orf57	2.9747	2.94152	3.00325	3.15402	2.97978	2.9551
C15orf61	1.399	1.23764	0.416483	0.736735	1.12053	1.65964
C15orf62	0.769916	0.552436	0.6317	0.74516	0.290955	-0.222966
C16orf13	2.6206	2.37006	2.28002	2.84828	2.72556	3.20318
C16orf45	3.06247	3.15187	2.39014	3.22209	-3.85089	-4.70296
C16orf52	5.78817	5.78255	3.48061	3.90521	3.46968	3.51004
C16orf58	4.22256	4.10771	4.97159	5.3236	3.99776	4.35038
C16orf59	2.54886	2.31196	2.17405	2.32998	2.44299	2.60163
C16orf62	3.3252	3.33063	2.86728	3.16612	2.39883	2.57017
C16orf70	3.17803	3.05018	3.58767	3.81686	3.95264	3.86668
C16orf72	6.70588	6.59729	6.9312	7.02536	7.32626	6.82803
C16orf74	?	?	-0.183372	0.0612232	-0.121008	0.443129
C16orf87	2.47086	2.49136	3.29424	3.66431	3.26005	3.4354
C16orf91	2.31496	2.1738	1.8772	2.17659	2.3593	2.76497
C16orf93	0.873451	0.305305	1.77923	1.81778	1.29077	0.685537
C17orf100	0.45076	0.151976	0.156448	0.300663	0.339787	0.401903
C17orf49	2.34793	2.41693	2.08211	2.29011	3.0439	3.16785
C17orf51	3.10518	3.21701	3.20607	3.82668	3.47415	3.0664
C17orf53	2.42247	2.20606	2.29627	2.65937	2.19458	1.88577
C17orf58	1.69756	1.87703	1.78805	1.99448	3.3411	3.35937
C17orf59	0.760425	0.456112	0.699196	1.25754	1.22238	1.88577
C17orf62	4.46771	4.19714	4.67368	4.73665	5.59353	5.37808
C17orf75	2.76853	3.00273	2.79376	2.9997	2.89428	3.27057
C17orf80	4.25073	4.26167	4.21308	4.48362	4.93541	5.30939
C17orf85	4.85551	4.8472	5.16962	5.18061	5.2112	4.67158
C17orf89	4.49507	4.38889	2.77068	3.01271	4.13572	4.64311
C17orf96	2.44559	2.0956	0.686917	0.761609	1.96907	0.878317
C18orf21	2.38382	2.35798	2.03587	2.2522	1.89692	2.3021
C18orf25	5.52519	5.59511	4.20879	4.42837	4.36067	4.26757
C18orf32	1.56349	1.71334	0.955491	1.50094	1.81005	2.03739
C18orf54	3.96803	3.94653	3.33218	3.18809	2.01214	1.88577
C18orf8	2.78006	2.79805	2.37213	3.03885	2.10949	2.33846
C19orf12	3.06081	3.04891	3.45311	3.69369	4.15667	4.31565
C19orf24	2.01175	1.73686	1.5714	1.77384	2.89044	3.48804
C19orf25	2.17746	2.12963	1.88797	2.30481	3.4298	4.19717

C19orf43	4.57413	4.56781	3.87987	4.34032	5.32981	5.31981
C19orf44	2.40475	2.40616	1.82588	1.78258	2.59802	2.44301
C19orf47	2.32053	2.29539	2.28002	2.58118	3.01565	2.68131
C19orf48	4.83327	4.65803	4.06083	4.46576	4.65878	4.5982
C19orf52	1.91933	1.81795	1.7234	1.93012	2.7363	2.84788
C19orf53	4.33121	4.25524	3.51765	3.86767	5.49883	5.71856
C19orf54	0.871631	0.503786	1.53104	1.18619	0.540454	0.338932
C19orf60	1.90086	1.8681	1.46757	1.59165	2.47925	2.74862
C19orf66	2.26799	2.27573	2.81884	3.23005	2.93947	2.78537
C19orf68	0.62629	0.4039	0.741954	0.679036	0.635792	-0.0439746
C19orf70	2.68031	2.57458	2.0405	2.44193	3.59708	4.73726
C1D	2.88606	3.01014	2.21265	2.38441	3.00855	3.0163
C1DP1	2.13185	2.00451	1.23172	1.66515	2.12531	3.09237
C1GALT1	4.5577	5.05581	3.85127	4.23657	4.50396	5.02885
C1GALT1C1	3.35349	3.20605	3.73535	4.08222	5.07434	5.72896
C1QBP	6.29942	6.35799	5.62591	6.08002	7.2783	7.71808
C1QBPP2	1.08178	1.40694	0.828989	0.957811	2.19012	1.95277
C1QL1	-0.289526	-1.2146	0.27602	0.399062	1.56854	0.993783
C1QTNF1	3.60356	3.4826	0.860939	1.38307	4.6088	3.83825
C1QTNF6	0.289705	0.265185	4.46294	3.9536	-1.41201	-1.70626
C1R	6.84158	6.06898	7.96913	8.38361	1.04711	0.893261
C1RL	4.581	3.99416	4.93899	5.04553	2.49062	2.26384
C1RL-AS1	3.3267	3.02995	4.17842	4.14007	-0.672635	-0.99638
C1S	5.86648	5.12258	7.3366	7.6333	-1.57243	-2.8983
C1orf106	2.8495	2.49136	0.82777	0.833756	-3.19956	-4.11926
C1orf109	4.04659	4.03101	3.31233	3.61569	3.73092	3.75476
C1orf112	4.43843	4.52801	3.5789	3.8777	3.57659	3.83981
C1orf122	2.83659	2.86562	1.96576	2.36514	3.45786	4.64634
C1orf123	3.21232	3.01492	3.71432	3.81894	3.58709	3.55773
C1orf131	2.60014	2.67416	2.39392	2.59162	2.6135	3.13878
C1orf132	2.83231	3.16652	4.94252	4.86772	2.59237	1.62424
C1orf147	-0.0124477	-0.433278	1.12224	1.19964	-0.878698	-1.06257
C1orf159	1.87838	1.58158	1.97637	1.83957	1.65714	1.34861
C1orf174	3.30233	3.38713	3.26149	3.636	4.24359	4.14971
C1orf198	4.66833	4.73314	3.0525	4.30269	5.60058	5.66669
C1orf21	5.64908	5.1324	3.76776	3.27272	2.71038	2.62422
C1orf216	4.31772	4.51156	3.78658	4.29525	4.48371	4.4187
C1orf220	1.28878	1.15925	-1.09576	-1.40802	-0.753191	-1.06257
C1orf226	2.59938	2.41871	-1.86109	-1.24444	2.27895	3.00822
C1orf27	4.44876	4.50438	3.99762	4.26622	4.4805	4.81685
C1orf35	2.25222	2.22717	1.98141	2.16265	2.65485	2.47321
C1orf43	6.27156	6.43161	5.31731	5.87344	6.51194	7.27469
C1orf50	0.760425	1.19542	1.98642	2.35062	1.58769	1.91536
C1orf52	3.40945	3.25311	3.44583	3.67906	4.2162	4.19565
C1orf56	1.17217	1.19799	1.02601	1.26657	1.34578	1.44944
C1orf74	3.10224	3.12824	2.85181	2.92751	2.47728	2.55195
C20orf144	0.356302	0.151976	1.48185	1.49112	0.395063	-1.12145
C20orf194	3.43717	3.29163	4.06893	4.23575	3.57408	3.33274
C20orf196	-0.129081	0.195474	0.914572	1.27492	0.587701	1.33772
C20orf24	5.32276	5.36148	5.61836	6.27532	5.35926	6.2348
C20orf27	3.04239	2.89031	3.47825	3.71773	3.2254	3.34585
C20orf96	-0.0990262	-0.036446	1.98141	1.72409	-0.753191	-0.799586

C21orf2	0.916614	0.597469	2.14434	2.27433	0.849647	1.01223
C21orf33	1.64486	1.48986	1.9492	2.0489	1.10067	1.77505
C21orf58	2.99441	2.60325	4.09492	3.94645	2.17576	1.50278
C21orf59	3.72327	3.86011	3.43787	3.80662	3.98686	4.28327
C21orf91	3.57588	3.65479	5.06617	4.58987	2.62972	1.86317
C22orf29	4.36763	4.47213	4.24279	4.59647	3.43205	3.22402
C22orf39	3.57227	3.65035	3.10505	3.31624	3.25761	3.37864
C22orf46	4.14405	3.84662	3.87175	3.69369	3.14059	2.23582
C2CD2	4.62227	4.74234	5.79553	5.94202	4.11448	4.00409
C2CD2L	3.08132	3.25901	3.14975	3.38576	2.85014	2.71863
C2CD3	4.62733	4.71954	4.78729	4.86054	4.23678	4.21364
C2CD5	4.46393	4.44854	3.81366	3.66431	3.69724	3.81096
C2orf16	1.6585	1.41984	1.0357	0.57776	0.679613	0.522196
C2orf27A	0.857925	1.142	-1.3263	-1.04039	0.681035	1.34024
C2orf42	2.11472	2.30535	1.89332	2.2086	2.49705	2.80899
C2orf44	3.80968	3.86246	3.05489	3.29844	4.27484	4.48188
C2orf47	2.34196	2.46215	1.86134	2.23969	2.67621	3.52475
C2orf49	3.99758	4.21311	3.59554	3.87885	4.2988	4.23579
C2orf68	3.97069	3.96446	3.52211	3.52416	4.39496	4.16826
C2orf69	3.87661	4.11187	3.51691	3.80416	4.9805	4.87448
C2orf74	2.8185	2.78818	2.32646	2.6117	3.37309	3.84154
C2orf76	1.21386	1.17383	0.416483	-0.108683	1.06084	1.44308
C2orf88	1.66289	1.95503	1.5847	1.52784	2.38683	2.22404
C3	7.2245	6.25931	7.0081	7.83685	-1.61549	-0.280287
C3AR1	-2.61075	-2.21426	-0.659745	0.535117	-0.374735	1.65964
C3orf14	1.7968	1.67161	-5.1783	-4.52056	-5.07051	?
C3orf17	4.65719	4.71256	4.68133	4.97662	4.48807	4.74134
C3orf18	2.00138	1.87257	1.66826	2.17196	1.14709	1.47324
C3orf33	0.631914	0.6247	-5.1783	-3.78492	0.479334	0.50283
C3orf38	3.9046	3.97073	3.52504	3.83443	3.90829	4.58122
C3orf52	1.43539	1.70209	1.75314	1.79207	?	?
C3orf58	4.0005	3.93484	3.90923	4.1638	3.41652	2.43285
C3orf62	2.01519	1.98012	1.85542	1.93559	3.05051	2.677
C3orf67	0.41996	0.840999	-3.59683	-5.10387	0.427241	0.702755
C3orf70	3.30092	3.57057	-6.17309	-5.10387	-5.65305	?
C4A	-1.90978	-2.11548	1.19629	0.679289	0.800869	0.173895
C4orf19	0.781863	1.34312	-2.22183	-3.78492	0.89096	1.48305
C4orf27	2.8087	2.81326	2.63027	3.12957	3.14865	3.84979
C4orf29	2.41187	2.61131	2.32032	2.50221	3.07061	2.91155
C4orf3	4.14875	3.96132	4.11224	4.08962	5.50678	5.07874
C4orf33	1.03183	1.28651	2.50066	2.43602	2.33356	2.6789
C4orf46	3.77082	3.72788	3.53415	3.63601	3.48481	3.02121
C5	2.36163	2.21665	2.83885	2.96532	0.714804	1.25912
C5orf15	6.26737	6.25017	6.34847	6.43022	6.00599	5.51055
C5orf22	3.87833	3.83522	3.80658	4.19951	4.76542	4.80996
C5orf24	5.27642	5.23773	5.25734	5.29842	5.83462	5.55877
C5orf28	1.78476	2.3218	1.85542	2.3424	2.13087	2.5787
C5orf30	3.57004	3.64698	3.54609	3.85407	3.83454	3.69843
C5orf34	2.26199	2.29116	3.01371	3.45727	2.54555	2.81984
C5orf42	4.65955	4.52227	5.71507	5.53052	5.141	5.03642
C5orf45	3.834	3.78837	5.23648	5.54619	4.67246	4.26842
C5orf46	-0.632781	-0.440295	?	?	3.26301	1.47324

C5orf51	4.33778	4.13147	4.11912	4.50496	5.01652	5.00236
C5orf56	0.568713	0.777005	3.23805	3.25005	0.849255	0.732929
C5orf66	1.38151	1.7153	4.07673	3.82523	2.35909	2.00216
C6orf1	2.14643	2.13709	1.33222	1.94105	2.27928	2.62869
C6orf106	5.0841	5.07384	5.7043	6.08306	6.15549	6.0625
C6orf120	4.18851	3.94039	3.68843	3.65086	5.29438	5.51301
C6orf136	1.10833	0.785149	0.73541	1.14862	1.99076	1.80111
C6orf141	2.39369	2.37637	-4.59508	-5.10387	2.67732	3.29047
C6orf163	0.937608	0.877069	-0.510905	-1.52346	0.587701	-0.183001
C6orf203	1.68466	1.85911	1.75314	2.03585	2.37862	2.65085
C6orf47	3.56184	3.72203	3.47647	3.9163	4.05993	4.41352
C6orf48	3.96537	4.08597	4.05846	4.84608	4.80525	5.1372
C6orf62	6.10716	6.24429	5.80869	6.31291	7.08924	7.12603
C6orf89	5.67811	5.78744	5.5929	5.83732	6.74933	6.87964
C7orf25	0.671003	1.1926	1.00487	0.811282	0.483375	-0.0511854
C7orf26	2.91564	2.92517	2.759	3.07109	2.82754	3.09419
C7orf31	-0.0550876	-0.354149	0.571444	1.13916	-0.878698	-0.899103
C7orf43	3.18172	2.90399	2.81171	2.69678	3.70991	3.27753
C7orf49	4.84139	4.66442	3.93345	4.10448	4.24536	4.54994
C7orf50	3.57005	3.08019	3.80374	3.76154	3.73736	3.88574
C7orf55	1.10901	0.646421	0.919843	0.958627	1.78123	2.23624
C7orf55-LUC7L2	5.56199	5.62558	5.3053	5.28381	5.86299	5.48026
C7orf60	3.32884	3.34612	3.0453	3.27919	3.91048	4.39788
C7orf73	4.4633	4.3795	4.00135	4.16146	4.71092	4.81684
C8orf31	-0.791952	-1.29258	1.43857	1.49854	-6.64581 ?	
C8orf33	4.47958	4.50438	4.01859	4.33307	5.07312	5.38604
C8orf37	0.301026	0.467978	0.805235	1.04098	1.33409	1.4532
C8orf4	1.49572	0.894767	-0.325375	-1.35358	2.92466	3.4607
C8orf44	-0.464329	-0.226388	1.26597	1.07541	0.498149	0.260712
C8orf46	-0.223192	0.479758	-2.48229	-1.93836	1.25263	2.03478
C8orf58	3.59573	3.6032	2.44562	2.4869	3.76275	3.46188
C8orf59	3.83639	3.91121	3.58144	3.76693	4.53173	5.40408
C8orf76	2.69945	2.84892	2.42324	2.81147	3.16685	4.14321
C8orf82	1.63187	1.68185	1.78229	2.0561	2.93774	2.96925
C8orf88	0.895783	0.933437	-2.96462	-2.52686	0.753459	1.20497
C9orf114	3.79176	3.66647	3.12474	3.36239	4.10654	3.94262
C9orf142	2.01175	1.85006	1.87178	2.00494	1.94334	2.03478
C9orf156	2.14329	1.9925	1.16526	1.49854	2.12107	2.30453
C9orf16	2.90641	3.05089	0.996447	1.27492	1.8195	2.59708
C9orf3	4.17541	4.24885	4.74942	4.84609	4.92136	5.10465
C9orf40	1.8401	1.67674	1.13846	1.42259	2.0879	2.13251
C9orf47	-1.33699	-0.907998	1.86894	2.01382	0.270529	-1.17987
C9orf69	3.43918	3.28535	2.7413	3.03839	3.85243	4.03138
C9orf72	2.99441	3.23412	2.39014	2.52781	2.76806	3.27913
C9orf78	4.35483	4.55814	3.35951	3.90936	4.70656	4.72687
C9orf84	-1.52875	-1.55554	1.43857	1.15801	-1.53062	-1.4584
C9orf85	2.13383	2.05287	1.48185	1.65939	2.1852	2.02802
C9orf89	2.19878	2.31525	3.12482	3.61056	2.00149	2.3377
C9orf91	3.31772	3.45899	2.64938	2.85118	3.41784	3.31288
CA11	0.676033	0.697098	0.340141	0.946514	-0.0903144	0.541297
CA12	1.14018	1.96344	4.81998	5.75302	5.59089	6.15824
CA13	1.45052	1.89912	-0.861345	-0.52367	2.59928	3.11347

CA5B	1.83678	2.06649	1.78407	2.1902	1.19533	1.36605
CA5BP1	1.45517	1.16787	1.59809	1.77303	1.28642	1.17992
CA8	0.539412	1.25145	-1.32519	-0.464785	1.15992	0.315752
CA9	-1.39121	-2.29018	-1.40575	-0.892955	6.511	4.8421
CAAP1	3.14013	3.30369	2.23012	2.39103	4.62566	4.50151
CAB39	5.79176	5.41941	5.35661	5.47611	5.22843	4.73519
CAB39L	1.58636	2.13709	1.71737	2.32164	2.3026	3.13249
CABIN1	4.76086	4.71706	5.10187	5.20971	4.52703	4.26912
CABLES1	2.30937	2.12094	2.90118	2.7993	2.88654	2.62153
CABLES2	2.76036	2.62197	2.96114	3.13435	2.43246	1.97279
CABP4	?	-6.45019	1.35568	1.3412	-6.64581	-5.69922
CABYR	1.48649	1.7195	-0.653237	-1.19498	-2.00481	-1.70626
CACFD1	0.278306	-0.681268	1.40148	1.37503	0.36215	-0.0341529
CACHD1	3.20631	3.26508	2.65254	2.34652	1.32842	1.08775
CACNA1C	-5.69162	-4.13839	4.02053	4.07628	-5.07051	-3.70484
CACNA1G	-3.69722	-4.43123	2.5512	1.83957	-4.97442	?
CACNA2D1	1.32333	1.86361	-3.18222	-3.52222	?	?
CACNA2D4	1.69756	1.35738	-0.206089	-0.616766	-0.302594	-1.38442
CACNB1	1.95558	1.86107	1.30838	1.4458	1.5059	1.11352
CACNB2	0.67123	1.22186	0.191626	0.231137	1.00351	0.270672
CACNB3	2.5006	2.43501	3.1111	2.93284	2.72556	2.70269
CACNG8	0.658554	0.292146	2.09727	2.1296	-3.6585	?
CACTIN	2.86259	2.43651	2.54028	2.45223	3.06862	2.58025
CACUL1	5.44013	5.58598	5.53243	5.9836	6.54729	6.5403
CACYBP	6.07236	6.14502	5.19394	5.64333	6.05052	6.42077
CACYBPP2	4.64207	4.79244	3.85189	4.24154	4.44226	5.3118
CAD	5.85793	5.75691	4.59782	4.6872	5.22653	4.96747
CADM1	7.16083	6.80801	5.26974	6.05009	6.2479	6.48555
CADM3	6.04593	5.71478	-1.87527	-1.12354	-6.34713	-4.46827
CADM3-AS1	2.23791	2.0849	-5.91681	-4.88337	-7.02289	-6.32604
CADM4	5.10706	5.77406	-0.117291	0.0612232	0.807363	0.597145
CADPS2	3.33573	3.5692	4.05249	4.11637	4.93073	5.23506
CALB2	2.3371	2.66902	2.19588	2.75536	-6.64581	?
CALCOCO1	4.1195	3.90675	4.42657	4.51047	3.6762	3.28197
CALCOCO2	5.14247	5.28785	5.63345	6.12328	4.97313	5.07791
CALCRL	-1.94365	-1.93875	-0.568612	-1.46458	3.80932	4.95684
CALD1	7.63825	7.63766	7.50737	7.66348	7.78583	7.37823
CALHM2	1.58272	1.77411	0.0620664	0.306414	0.0279042	-0.433286
CALM1	6.9194	7.23064	5.3203	5.99608	6.74999	7.22198
CALM2	7.8601	8.0046	7.33585	7.98977	8.36177	9.00809
CALM2P2	4.91285	5.00878	4.40252	4.99413	5.4133	6.06135
CALM2P3	3.02156	3.10456	2.35132	3.13685	3.64281	4.45399
CALM3	5.71397	5.31357	5.73794	5.89504	6.52919	6.28623
CALML4	1.878	1.64716	2.25476	2.22926	1.86665	0.872553
CALR	9.25715	9.06507	9.17064	9.38335	9.14782	9.1546
CALU	8.45483	8.12575	8.48681	8.7122	8.27834	7.97218
CAMK1	1.89683	1.67157	-0.500767	0.00222004	2.71049	2.85436
CAMK1D	?	-5.45651	1.89332	2.2086	1.47928	1.29333
CAMK2D	4.99047	4.89799	3.77647	4.01012	6.11197	6.09741
CAMK2G	3.94744	4.10482	4.24556	4.45197	4.58031	4.49651
CAMK2N1	1.31777	1.53719	1.53076	2.15329	-3.33694	-3.12051
CAMK4	5.5414	5.56987	2.49245	2.90241	4.441	4.71219

CAMKK1	1.37242	1.40138	2.52039	2.9383	1.72342	1.71117
CAMKK2	3.63854	3.43803	3.46573	3.54939	3.15988	2.65085
CAMKMT	1.40948	1.3764	0.53081	0.761609	1.41119	1.28202
CAMLG	3.84782	3.81792	4.11681	4.38002	4.08367	4.36206
CAMSAP1	5.0453	5.06997	4.96112	5.17135	5.0904	4.81389
CAMSAP2	6.29435	6.41883	6.00038	6.18146	6.43194	6.40994
CAMTA1	3.44175	3.19715	2.4164	2.78297	3.96173	3.77911
CAMTA2	2.4895	2.24685	2.27667	2.58571	2.68403	2.75273
CAND1	7.38314	7.50434	6.59516	6.95609	6.72822	7.07074
CAND2	1.17813	1.08399	-0.699972	-0.732879	0.0566806	-0.44397
CANT1	5.62764	5.59278	4.89262	5.07169	5.67089	5.64863
CANX	9.20863	9.18681	9.80519	10.233	9.51188	9.51292
CAP1	7.30979	7.54858	6.48503	7.15758	7.83758	8.40578
CAP1P2	1.91386	2.49111	1.05418	1.86014	2.50136	2.73096
CAP2	2.95018	2.8016	4.03926	4.29949	4.85685	4.51486
CAPN1	4.33296	4.25311	4.58631	5.02173	3.62162	4.11346
CAPN10	3.31765	3.00069	2.76362	2.8516	3.04011	2.78114
CAPN10-AS1	2.34533	2.41675	1.53076	1.69856	1.62974	1.40185
CAPN15	3.20781	2.96552	3.12936	3.16497	3.18361	3.19415
CAPN2	7.10313	7.29329	6.8928	7.55925	7.58443	7.7016
CAPN5	2.63408	2.62992	2.87177	3.081	1.18523	1.25912
CAPN7	4.81513	4.95342	4.19584	4.56358	4.59884	5.00495
CAPNS1	5.46079	5.59311	5.42057	5.94577	6.30236	6.5899
CAPRIN1	7.31348	7.37989	7.22599	7.61204	8.15328	8.33891
CAPRIN2	4.86458	5.07047	4.22692	4.33411	3.7277	4.1881
CAPS	5.2376	4.55681	1.32645	1.17792	7.36695	8.19879
CAPS2	-2.9975	-3.46127	2.77068	2.52781	1.73204	1.41227
CAPZA1	6.6661	6.76308	6.46099	7.01137	7.18319	7.73842
CAPZA2	5.59547	5.62202	5.49226	5.8824	6.16974	6.6768
CAPZB	4.93345	4.70519	5.2511	5.35624	5.8532	5.72999
CARD10	4.03223	4.48039	2.16079	2.39501	2.19769	2.18205
CARD16	-0.522356	-0.485378	2.70615	2.31619	-0.886822	0.620671
CARD6	1.66727	1.66131	3.37872	3.76922	3.15668	3.78313
CARD8	3.12469	3.43352	3.95052	4.10068	4.38154	4.25642
CARF	1.04916	1.39404	1.72244	1.47715	1.79093	1.87074
CARHSP1	4.95747	4.58946	5.23819	5.61493	4.21028	4.04215
CARKD	3.83718	3.69285	3.94394	4.25169	3.94146	3.87448
CARM1	3.4526	3.19156	3.02729	3.10201	4.34162	4.09551
CARNMT1	3.69001	3.81676	3.21308	3.68234	3.71037	4.0178
CARS	5.19177	5.15607	5.03732	5.53715	5.36519	5.34318
CARS2	3.95763	3.96702	3.88434	4.43273	3.68925	4.04062
CASC10	2.52734	2.50583	0.662041	0.350701	1.74911	1.51249
CASC15	-2.52833	-3.29152	-1.62185	-2.08438	3.10917	2.5535
CASC3	4.87123	4.94334	4.52468	4.7437	5.59296	5.37074
CASC4	5.2822	5.60596	5.49375	5.96419	6.34182	6.53534
CASC4P1	-0.228704	0.455828	-0.113776	0.876825	0.991427	1.08131
CASC5	6.53719	6.64695	5.95762	6.13105	5.34426	5.81734
CASD1	3.40026	3.49426	4.06322	4.15209	4.24206	4.54121
CASK	3.51888	3.38108	4.02715	3.75226	4.15587	3.81489
CASKIN2	1.78879	1.68694	2.15634	2.1904	2.09793	2.44305
CASP1	-1.85712	-2.55512	4.34854	4.07021	0.826062	1.82236
CASP2	4.09371	4.16194	3.91055	4.13672	4.30979	4.21958

CASP3	4.12189	4.36846	3.95218	4.62334	4.12267	5.1372
CASP4	2.39369	2.23066	5.48003	5.55517	4.11776	4.72478
CASP6	2.08884	2.17017	2.14741	2.3424	2.55402	2.95863
CASP7	2.66068	3.48844	3.51344	4.52193	3.46897	4.60615
CASP8	4.17205	4.25397	4.25838	4.64104	3.15187	3.97803
CASP8AP2	5.62908	5.57673	5.28156	5.331	5.65511	5.79162
CASP9	1.58636	1.73686	1.07392	1.59857	2.45084	2.86694
CAST	5.77786	5.77458	6.61085	6.93772	7.7926	7.61335
CAT	4.20254	4.13985	4.04529	4.2553	5.21158	5.66505
CATSPER2	1.69509	1.76334	0.907583	0.58426	1.4349	0.665011
CAV1	5.66642	5.88644	7.44757	8.03121	7.39439	7.6417
CAV2	3.0407	3.31688	3.14515	3.41669	4.98445	4.60729
CBFA2T2	4.08882	3.8925	4.49507	4.5187	3.85144	3.18507
CBFB	5.50793	5.67605	5.1305	5.379	6.43409	6.09094
CBL	5.21005	5.22933	5.58631	5.8003	5.02968	4.98328
CBLB	2.94838	2.77795	4.51691	4.62926	3.33828	3.40964
CBLL1	4.26664	4.33663	3.80626	4.16391	4.16483	3.94612
CBLN2	-5.69162	-5.45651	3.48535	1.38307	-2.07475	-3.38329
CBR1	2.1491	2.2096	3.50821	3.80987	5.21461	5.58788
CBR3	2.18749	2.15445	1.88256	3.00624	1.3569	2.11421
CBR3-AS1	0.313664	0.330398	0.759693	1.24292	-0.187967	-0.992021
CBR4	3.73438	3.82645	3.69621	3.64553	3.56621	3.44918
CBS	2.4918	1.21383	3.95813	4.07803	1.24335	0.222359
CBSL	-1.81208	-0.207105	1.74342	1.47916	2.68298	2.25952
CBWD1	4.17326	4.17512	4.04533	4.09662	4.79696	4.79024
CBWD2	3.73689	3.75981	3.51989	3.71275	4.52175	4.60622
CBWD3	3.60739	3.70306	3.16084	3.09431	4.13694	4.01086
CBWD5	4.61749	4.58372	3.9429	4.09531	5.1982	5.1449
CBWD7	3.62002	3.42498	3.26857	3.50415	4.11269	3.88868
CBX1	5.8121	5.86825	5.29518	5.63445	5.71535	5.74666
CBX2	4.70339	4.68308	4.74277	4.87202	2.82351	2.85935
CBX3	6.98596	6.97075	6.57278	6.93164	6.94178	7.33038
CBX3P9	4.35705	4.36745	3.90142	4.2591	4.20402	4.75863
CBX4	3.15423	3.09749	3.28	3.72089	2.83555	2.74862
CBX5	7.74502	7.85335	7.81657	8.06188	7.45413	7.3679
CBX6	5.94367	5.65217	5.70065	5.62291	5.38848	5.27984
CBX7	1.94052	2.23095	2.12145	2.3344	1.53456	1.57322
CBX8	3.14013	3.00069	2.07389	2.37903	2.20698	2.38076
CBY1	2.29262	2.10886	2.57484	2.51973	1.82932	1.37621
CC2D1A	2.56772	2.317	2.45311	2.69531	3.68706	3.61295
CC2D1B	4.81101	4.69897	5.20354	5.44598	5.2462	5.39633
CC2D2A	3.71454	3.74294	2.95347	3.05608	3.71147	3.95684
CCAR1	5.72247	5.56962	5.38085	5.49524	5.95348	5.63702
CCAR2	6.05246	6.04647	5.62969	5.75502	6.05611	6.00136
CCBL1	1.63634	1.41371	1.98141	2.03075	2.01567	2.08772
CCBL2	3.44599	3.34355	2.96796	3.2862	4.10434	4.90689
CCDC101	1.20436	1.35049	1.35017	1.80516	1.01342	1.31612
CCDC102A	0.377835	0.419895	0.584741	1.07114	0.86332	1.20023
CCDC102B	-0.744656	-0.655276	3.44947	3.09575	3.47668	4.18051
CCDC103	0.159474	-0.101954	-0.012717	0.454639	2.01164	2.4161
CCDC106	2.14956	1.72206	1.01621	0.95732	2.97631	3.11027
CCDC107	0.72396	0.874844	0.219686	0.517235	2.62746	2.38337

CCDC109B	2.88964	3.13335	1.13845	1.32585	3.42184	3.73623
CCDC112	2.22575	2.49427	1.75902	2.53504	2.0879	2.83245
CCDC113	-1.11382	-1.50825	1.67449	1.7698	2.5637	2.52691
CCDC115	2.73309	2.83408	2.24695	2.68885	2.77603	3.48064
CCDC117	4.80476	4.76116	4.74348	4.95928	4.53815	4.4005
CCDC12	3.14484	3.01896	2.32429	2.60199	3.25855	2.98329
CCDC121	1.11847	1.34106	0.631421	0.646135	1.50869	1.73501
CCDC122	1.00142	1.11463	0.259532	0.248838	-2.07475	-2.38405
CCDC124	2.49815	2.27864	2.26358	2.41082	3.13736	3.17593
CCDC125	2.97135	2.98501	1.96975	1.93553	3.46341	3.58437
CCDC126	2.04241	2.11082	1.98141	2.28351	2.41117	2.87072
CCDC127	3.79844	3.77675	4.37298	4.46061	3.72648	3.48558
CCDC129	-0.341358	-0.354149	?	-5.10387	2.44561	1.5971
CCDC130	2.28962	2.23761	2.4164	2.41866	3.14221	2.919
CCDC134	2.11151	1.88148	2.84991	3.00752	1.59711	1.23585
CCDC136	2.85743	2.99248	3.42011	3.44	2.51961	2.15129
CCDC137	4.62708	4.1582	3.57219	3.85983	4.89721	4.63413
CCDC138	3.37372	3.49571	2.39014	2.36692	3.17574	3.76699
CCDC14	6.15267	6.2069	6.64403	6.69227	5.88657	5.81585
CCDC142	3.01913	2.85458	2.65254	2.61911	2.77852	2.23109
CCDC144A	-4.69535	-7.41563	1.97872	1.82408	3.64474	3.46781
CCDC144B	?	-4.63312	5.15115	4.8764	3.03189	2.6401
CCDC144CP	-5.69162	-6.45019	3.03077	2.91153	4.90539	5.04952
CCDC146	0.309141	0.50228	-0.06726	0.0199296	1.37528	1.23384
CCDC149	2.63632	2.27526	1.20884	1.43811	2.07103	2.0415
CCDC15	3.14955	3.31687	3.0308	2.94103	2.30838	2.06149
CCDC150	3.65485	3.76616	2.92027	2.84538	3.08643	2.6198
CCDC150P1	1.34536	1.2583	0.793829	0.82198	0.499649	0.126299
CCDC157	1.27183	0.875473	0.95255	1.19118	0.539491	0.294147
CCDC159	-0.654465	-0.971108	0.528091	0.622171	0.24535	0.164516
CCDC163P	?	-4.13839	1.71737	1.94105	0.489522	-0.00614162
CCDC167	1.77262	1.76119	1.38638	1.69211	2.94518	3.40964
CCDC17	0.183671	-0.177294	0.904002	0.946514	0.282309	-1.24695
CCDC171	2.29812	2.65611	1.17408	1.24877	1.3621	1.70271
CCDC174	3.22425	3.29371	2.79662	2.83954	3.3909	3.44049
CCDC18	4.22913	4.33969	4.53942	4.78469	4.00709	4.20735
CCDC181	2.80475	3.0068	-2.01303	-2.64851	1.41654	2.15749
CCDC186	3.73867	3.68817	4.68142	4.69368	4.69668	4.72687
CCDC22	1.48086	1.66131	1.25116	1.76772	1.29393	1.90064
CCDC24	0.988768	0.932341	1.93392	2.24549	0.340015	0.541297
CCDC25	4.68246	4.69564	3.90363	4.30007	4.63523	5.21437
CCDC28A	2.10722	1.73686	1.61093	1.61231	3.25163	3.37224
CCDC28B	2.45828	2.5133	2.19587	2.42257	2.79297	3.59479
CCDC34	2.74801	2.72204	3.16078	3.2288	3.171	2.7609
CCDC39	1.00142	1.06077	0.25958	0.350701	1.7534	0.022034
CCDC40	-0.0407343	-0.23387	1.93025	1.93012	0.740643	0.200291
CCDC43	3.928	4.12956	3.99095	4.50508	4.71566	5.01705
CCDC47	6.57237	6.52033	6.45444	6.64959	7.72043	7.4096
CCDC50	5.99701	5.95711	6.07032	6.34752	6.79534	7.01775
CCDC51	3.39897	3.3169	2.28817	2.64941	3.1899	3.51488
CCDC53	2.7418	2.99447	2.25944	2.8337	2.16626	2.94434
CCDC57	4.88685	4.73954	3.95656	3.85294	4.49127	4.13741

CCDC58	2.62302	2.75745	2.1293	2.38951	2.86652	3.34381
CCDC58P3	0.314905	0.365189	-0.335055	-0.472383	0.0970309	0.731505
CCDC59	3.84185	3.88459	3.10597	3.41804	3.72659	4.06098
CCDC6	5.07075	5.17694	4.52985	4.96396	5.47282	5.86265
CCDC61	0.0954519	-0.177293	0.120378	0.476226	0.384182	0.359476
CCDC65	-0.489437	-0.418273	0.723445	0.646135	-0.250761	-0.752286
CCDC66	4.27213	4.3446	3.89101	4.00615	3.98997	4.01977
CCDC69	-0.509064	-0.790185	3.90924	4.15325	-5.65305	-4.70296
CCDC7	1.08701	1.60583	1.51514	1.66454	1.09188	2.0303
CCDC71	3.07076	2.82718	2.92501	2.91077	2.99254	2.96925
CCDC71L	3.44302	3.03301	3.2088	3.98392	1.11453	0.816919
CCDC73	0.995709	1.40502	0.627915	1.17375	2.26226	2.79373
CCDC77	4.39671	4.43216	3.87348	4.08565	2.821	3.25275
CCDC78	0.539412	0.432061	0.488988	0.383121	0.147163	-0.0627204
CCDC80	2.42764	1.94232	6.81788	6.82908	5.33896	5.8773
CCDC82	3.44302	3.50726	4.67833	4.83001	4.33192	4.22992
CCDC84	3.00656	2.93804	3.89556	4.10184	3.46381	2.68156
CCDC85B	3.05414	2.88589	1.69915	2.27489	1.57335	1.96574
CCDC85C	3.98144	3.94237	3.74546	3.73079	3.84402	3.28999
CCDC86	4.18186	3.97553	3.80216	4.28004	3.91667	3.91158
CCDC88A	6.74232	6.61686	7.24834	7.45593	6.61166	6.46275
CCDC88B	0.0824028	-0.214764	0.0645242	-0.108685	0.0540566	1.37015
CCDC88C	1.01255	1.09766	1.89348	1.71206	1.49547	1.42749
CCDC89	-5.69162	-4.45968	0.986462	1.22214	-0.121008	0.0213376
CCDC9	1.48086	1.59247	1.78805	1.90242	1.62049	1.2122
CCDC90B	3.52074	3.59753	3.80939	4.10305	3.88905	4.53784
CCDC91	3.59015	3.59925	2.75511	2.93081	2.61549	2.86623
CCDC92	2.77228	2.7333	2.4585	2.36048	1.93913	1.46325
CCDC93	4.92617	5.05034	4.54439	4.7677	5.33155	5.03474
CCDC94	1.75628	1.92519	0.956094	1.30061	2.04707	2.24167
CCDC97	2.60699	2.51445	2.65254	2.45724	3.00503	2.76903
CCHCR1	2.68463	2.64569	3.33808	3.65935	3.02969	2.97277
CCL2	9.20652	9.02374	5.24324	5.96829	3.5171	4.123
CCM2	3.74179	3.60054	3.78514	3.86415	4.112	3.7991
CCNA2	5.74195	5.90235	4.97564	5.39332	5.7972	6.08264
CCNB1	6.26954	6.32218	6.02561	6.50724	6.65358	7.12595
CCNB1IP1	3.90732	3.66772	2.68377	2.92185	3.91806	4.35127
CCNB2	4.99977	4.90214	4.21413	4.5085	4.95155	5.062
CCNB2P1	0.203978	0.4728	0.138526	0.19779	0.680981	0.0579424
CCNB3	1.42508	1.12217	-0.825729	-0.938639	-3.85089	-2.24661
CCNC	4.74208	4.76356	4.62717	4.86042	5.64062	6.12746
CCND1	7.79961	8.22408	6.02653	6.63514	5.92314	5.50999
CCND3	3.5429	3.51157	4.7743	5.2465	5.16465	5.23138
CCNDBP1	3.45574	3.27376	2.87837	3.38102	3.61987	3.99718
CCNE1	2.49323	2.40753	2.79663	2.85407	2.17576	2.19417
CCNE2	3.07316	3.05302	2.94012	3.12012	3.53648	4.00112
CCNF	4.952	5.02749	4.84132	4.87413	4.46865	4.15718
CCNG1	5.80646	5.54698	5.90491	6.03932	6.447	6.5251
CCNG2	3.10021	3.04693	3.00383	3.00231	3.13897	2.69844
CCNH	4.74381	4.90453	5.01101	5.22385	5.25497	5.83782
CCNI	5.96025	5.87199	5.58918	5.77228	5.92066	6.06848
CCNJ	2.91008	3.06654	2.80517	2.97864	3.44423	3.40964

CCNJL	3.48455	3.60459	4.20448	4.03838	3.86717	3.50518
CCNK	3.1566	3.05276	2.64612	2.74123	2.63792	2.83645
CCNL1	6.21866	6.00373	6.51561	6.49478	6.36031	6.01181
CCNL2	5.9731	5.78418	6.31231	6.18433	6.84872	6.16596
CCNT1	6.47348	6.45892	5.73424	5.93352	5.69283	5.7069
CCNT2	5.26522	5.28357	5.22783	5.28564	5.30907	5.36377
CCNY	4.60585	4.71894	4.03564	4.38702	5.12103	5.06972
CCNYL1	2.97563	2.81548	2.63344	2.83155	2.93912	2.98268
CCP110	4.60241	4.72699	4.89528	5.05293	4.70436	4.7331
CCPG1	5.2779	5.36812	5.70911	6.24599	6.07047	6.31662
CCS	2.43254	2.24798	2.89597	2.95187	2.06426	1.63317
CCSAP	4.68612	4.97425	4.11449	4.57937	4.79069	4.60939
CCSER2	5.2346	5.23324	4.9643	5.1067	5.78418	5.69256
CCT2	7.29704	7.35266	6.02226	6.43684	7.01233	7.78099
CCT3	7.82439	7.83295	6.69374	7.23159	7.55085	7.91255
CCT4	6.56561	6.76449	5.96639	6.45972	6.75276	7.40602
CCT5	8.16936	8.16392	7.55732	7.97027	8.41629	8.52689
CCT5P1	2.14851	2.2928	1.56445	2.17407	2.46429	2.21763
CCT5P2	2.62223	2.90362	2.07752	2.64469	3.09817	2.71352
CCT6A	7.69697	7.72578	6.77086	7.27569	7.67823	8.11379
CCT6P1	0.679523	0.792506	0.544634	0.888897	1.10568	0.806159
CCT6P2	3.20408	3.35814	2.40089	2.85598	3.25048	3.25175
CCT6P3	2.08124	2.2003	2.01611	2.20544	2.10913	1.57614
CCT7	7.13402	7.10626	6.47777	6.9466	7.2169	7.48562
CCT7P1	0.529821	0.920918	0.259532	0.82198	0.901996	0.597145
CCT8	6.36338	6.41994	6.53747	7.00474	6.47011	6.89483
CCT8P1	3.67624	3.96031	3.97419	4.46408	3.94405	4.31812
CCZ1	4.88907	5.01016	4.68242	5.04278	5.18292	5.75326
CCZ1B	3.4721	3.59018	3.77409	3.88752	4.72782	5.06432
CD109	8.11235	8.2718	5.35102	5.9251	5.99577	6.48762
CD151	6.9457	6.82631	6.59138	6.8315	6.16618	6.27013
CD160	0.907844	1.03007	1.33228	1.25169	1.49746	0.445102
CD163L1	2.25222	1.68694	2.83885	3.20632	?	?
CD164	6.62611	6.71703	6.13811	6.44884	7.33714	7.45013
CD1D	0.735617	0.407614	1.57806	1.96266	-5.65305	-5.69922
CD22	4.44047	4.43952	0.662041	1.64609	-5.65305	?
CD24	-5.10914	-4.87366	3.33583	3.99963	-2.65941	-2.70579
CD27-AS1	2.07419	2.3665	2.77242	3.01624	1.30581	1.21477
CD274	0.586404	1.04501	0.431281	1.30907	3.58166	3.85743
CD276	5.80796	5.27566	5.40613	5.22095	5.48655	4.90615
CD2AP	5.17359	5.21398	4.68297	4.85839	5.30149	4.91075
CD2BP2	5.26739	5.23714	5.05426	5.35521	5.53691	5.82759
CD302	4.31948	4.37413	4.20119	4.20394	3.34643	3.51924
CD320	4.77512	4.56987	3.40144	3.53684	4.79962	5.37343
CD33	0.388476	0.537236	2.50649	2.78296	?	?
CD34	-5.69162	-4.13839	0.355738	0.563685	3.26747	3.35127
CD37	-2.1135	-2.76144	1.49854	1.57059	-2.65941	-3.12051
CD3EAP	3.75198	3.853	1.90115	2.38352	4.1151	3.71696
CD44	8.24621	8.36291	9.27992	9.60582	9.15742	9.67677
CD46	6.45273	6.16171	7.01444	6.90082	6.69186	6.3024
CD47	5.37875	5.74566	6.32649	6.7175	4.66611	5.00064
CD55	4.37171	4.80977	3.1065	3.95322	0.799187	2.06809

CD58	3.49075	3.59108	1.99641	2.53864	4.90904	5.8055
CD59	8.91664	8.998	6.60808	7.08778	7.2596	7.48031
CD63	7.01388	6.96132	6.98655	7.40652	6.58374	7.27091
CD68	-0.2393	-0.260205	1.48438	2.90684	-1.59257	0.0442542
CD70	3.25803	3.42285	1.11115	1.85698	5.83355	6.49845
CD74	8.51607	8.03338	0.711372	0.798133	-1.75295	-3.12051
CD81	5.87859	5.85201	5.938	6.07604	5.26004	5.32325
CD82	-0.413499	-0.440295	3.74104	4.73944	1.50464	2.41713
CD83	4.03902	4.01388	-0.861345	-0.380732	2.49957	2.43796
CD9	3.70073	3.73805	4.72861	5.04027	4.19921	4.50639
CD96	4.74954	4.63517	0.138526	0.300663	-3.48878	-5.69922
CD99	4.75883	4.73128	5.88493	6.13076	3.90383	4.0041
CD99L2	4.62567	4.56575	5.25008	5.42835	4.63485	4.74657
CD99P1	-0.159773	-0.0876659	1.42385	1.27492	-0.0903144	-1.06257
CDADC1	1.00834	1.36375	1.25947	1.45345	1.05399	1.35942
CDAN1	3.27802	3.24697	3.03563	3.11724	3.36678	3.01781
CDC123	5.12668	5.16367	3.72384	4.20346	5.07136	5.53297
CDC14A	1.7732	2.11552	2.16056	2.27482	1.11161	1.01646
CDC14B	3.95128	3.4881	4.05672	4.14427	3.26369	2.52726
CDC14C	0.298541	0.181052	0.11422	0.406232	-0.316647	-1.0774
CDC16	3.81366	3.80978	4.10533	4.28026	4.16623	4.31288
CDC20	5.8705	5.80046	5.3937	5.96572	5.5046	5.65329
CDC20P1	2.693	2.83152	3.25193	3.73464	3.1518	3.19582
CDC23	5.16277	5.12821	5.09375	5.43323	5.1438	5.28338
CDC25A	5.18924	5.34492	3.99515	4.47047	4.78417	4.72582
CDC25B	5.05664	4.79808	6.03926	6.31666	4.69283	4.81879
CDC25C	3.17756	3.06869	3.17628	3.80858	2.55645	3.27057
CDC26	1.98187	1.86066	1.5909	1.91273	2.3678	3.54353
CDC27	6.687	6.776	6.19684	6.61509	6.57783	6.82526
CDC34	3.48207	3.21135	2.87447	3.1055	4.87155	4.35532
CDC37	5.95389	5.90536	4.89946	5.25717	6.55583	6.44536
CDC37L1	3.10344	3.18641	2.79662	2.9052	4.84351	5.56649
CDC40	4.26742	4.24279	4.04649	4.24652	5.28803	5.6915
CDC42	6.53503	6.61022	6.29563	6.67697	7.52597	7.73124
CDC42BPA	6.1432	6.15022	6.3218	6.45912	6.69584	6.79362
CDC42BPB	5.25018	5.22008	4.67124	4.72624	5.61717	5.48188
CDC42EP1	3.19877	2.92948	2.77938	3.10792	4.40577	4.5856
CDC42EP2	2.55124	2.85683	2.35952	2.57067	-0.168308	-0.214706
CDC42EP3	5.71243	6.12826	6.46371	6.90469	6.55207	6.47412
CDC42EP4	4.08964	4.18731	4.6198	5.08838	4.70487	5.05151
CDC42P6	4.18391	4.21436	3.86411	4.32212	4.97479	5.43681
CDC42SE1	6.64012	6.51857	5.65527	5.95957	6.2766	6.37198
CDC42SE2	4.61606	4.60927	4.76338	5.05419	5.11899	5.14894
CDC45	4.76341	4.85117	4.18715	4.43614	3.13248	3.36743
CDC5L	5.52732	5.66932	5.1513	5.39797	5.79808	6.22475
CDC6	5.66587	5.68403	5.42194	5.79165	5.47475	5.31356
CDC7	4.66502	4.75572	4.00876	4.12359	3.05394	3.3992
CDC73	5.27497	5.2219	4.83397	4.92802	5.23336	5.13642
CDCA2	5.44908	5.45488	4.95857	5.25638	4.90382	5.09821
CDCA3	4.49392	4.36412	4.2	4.3956	3.69714	3.62101
CDCA4	5.19573	5.24969	2.99141	3.21085	4.47605	4.69095
CDCA5	5.5237	5.54733	4.32823	4.68233	4.58761	4.48557

CDCA7	4.8807	4.87421	5.54737	5.44094	3.87204	3.73207
CDCA7L	4.90351	4.87395	5.06943	5.28751	5.34458	5.12981
CDCA8	4.76648	4.90675	4.01124	4.52416	4.74588	4.78315
CDH11	5.90572	5.40656	7.07914	7.12925	7.56674	7.29522
CDH13	6.59167	6.73348	4.96048	5.1632	4.65482	4.91075
CDH18	3.89056	3.96446	-6.17309	-6.09892	?	-4.70296
CDH19	6.52336	6.63717	-6.17309	-3.78492	-6.64581	-5.69922
CDH2	7.10849	7.2224	5.40877	5.69434	6.61614	6.64608
CDH24	1.77262	1.67674	1.74725	1.97335	2.19458	2.21217
CDH4	-3.37567	-6.45019	-0.117291	-0.52367	5.88081	5.38602
CDH6	-0.816197	-1.14061	7.16007	6.90293	6.39868	6.3191
CDHR3	1.60474	1.89912	-0.0541032	0.0410467	0.815493	0.359476
CDIP1	1.40425	1.2583	2.62384	2.9301	-0.551587	-1.53638
CDIPT	4.31126	4.11538	3.85265	4.09818	3.71037	3.96217
CDK1	5.64103	5.72851	5.67949	5.8663	6.19821	6.8402
CDK10	3.72509	3.46345	3.60438	3.60884	3.69614	2.99026
CDK11A	2.39338	2.06495	2.46269	2.70302	2.84755	2.45177
CDK11B	3.54626	3.45055	3.05159	3.36146	4.08462	3.86916
CDK12	5.94477	5.85059	6.25293	6.29683	6.30746	6.05441
CDK13	5.71582	5.72573	5.70026	5.86774	5.80396	5.74346
CDK14	4.76851	4.58494	-0.455418	-0.589346	4.75329	4.92446
CDK16	5.95486	5.72108	4.997	5.21984	4.76069	4.51969
CDK17	4.32398	4.39591	4.28203	4.35775	3.89139	4.03643
CDK18	1.31777	0.988427	2.24695	2.15798	-2.07475	-2.24661
CDK19	3.84782	3.83864	3.693	3.9052	4.09457	4.07792
CDK2	5.48329	5.46677	4.80657	5.17484	5.00634	5.07873
CDK20	0.987481	0.859151	1.496	1.31748	1.86328	1.93717
CDK2AP1	6.54761	6.44443	4.94291	5.31624	6.28389	6.03875
CDK2AP2	2.80674	2.57059	2.76777	3.081	2.54427	2.52692
CDK3	0.367483	0.0963023	0.332026	0.330708	0.734161	0.590233
CDK4	5.5538	5.52961	5.38001	5.73736	5.71872	6.12957
CDK5	3.40692	3.18735	2.78772	3.26759	3.67894	4.18945
CDK5R1	2.24345	2.15188	0.138526	0.266325	1.12765	0.785207
CDK5RAP1	4.6251	4.71706	4.21415	4.5296	4.02268	3.71324
CDK5RAP2	5.78119	5.88117	5.59125	5.70535	5.17021	4.74449
CDK5RAP3	5.17868	5.03231	4.89379	5.0795	5.42905	4.97917
CDK6	7.8283	7.9228	7.13389	7.5163	7.38127	7.19829
CDK7	3.00991	3.20072	2.70021	3.29153	3.64612	4.11665
CDK8	3.4603	3.45066	3.72154	3.91962	3.14182	3.11984
CDK9	3.58517	3.42437	3.85813	4.06485	3.67284	3.63313
CDKAL1	3.70712	3.87365	3.45854	3.69368	4.01121	4.29328
CDKL1	0.645766	1.16365	-0.635609	0.0877344	0.587307	1.02465
CDKL5	2.78877	2.69955	1.95093	1.90801	3.80423	3.27342
CDKN1A	3.92572	4.1998	2.97384	3.46293	2.59237	2.69418
CDKN1B	4.65462	4.6818	4.70446	4.89257	4.99521	5.21957
CDKN2AIP	3.2405	3.33319	3.13163	3.4948	3.46511	3.96039
CDKN2AIPNL	3.94206	3.86358	3.59783	4.08346	3.45863	3.57405
CDKN2B-AS1	1.73974	1.81394	0.614116	0.911063	?	?
CDKN2D	1.04917	1.10707	0.386425	1.15801	1.86719	1.85556
CDKN3	3.59668	3.49861	2.98641	3.69692	3.86521	4.5856
CDON	2.42292	2.79612	4.36607	4.83615	5.36473	5.7211
CDPF1	1.32887	1.44408	1.69915	1.85698	0.969108	1.55071

CDR2	3.41537	3.44751	3.64848	4.24058	3.80724	3.74455
CDR2L	1.89714	1.57061	3.27796	3.48176	3.43245	2.95507
CDRT1	-2.1617	-2.32862	2.08655	2.61931	0.109133	-0.417199
CDS1	1.76855	2.0007	1.51695	2.06112	-0.0163223	-0.0627204
CDS2	5.82325	6.00628	6.30882	6.67414	5.78574	6.11185
CDT1	2.76853	2.64307	2.12028	2.37097	1.86328	1.64205
CDV3	6.54696	6.50458	6.05965	6.45509	6.61181	6.53737
CDYL	4.36768	4.26336	4.29625	4.50863	5.16384	5.17822
CDYL2	4.45889	4.46048	3.21523	3.54222	-2.95876	-2.70579
CEBPA	-0.856614	-1.01204	-0.861492	-0.885268	1.27925	2.04911
CEBPB	3.0537	2.85411	3.49339	4.02748	2.92006	3.09411
CEBPD	4.00135	3.02098	3.41639	3.59334	1.76178	0.736302
CEBPG	4.17051	4.1491	4.07267	4.67825	5.82272	6.07343
CEBPZ	6.41335	6.60472	5.00827	5.37914	6.28855	7.14235
CEBPZOS	4.48768	4.4343	4.08374	4.31917	4.9629	5.0258
CECR5	4.51036	4.38498	3.42196	3.67253	4.3509	4.23286
CECR7	3.24997	2.93821	-3.24626	-4.06966	-4.0726 ?	
CELF1	6.37826	6.32239	6.2438	6.42378	6.93374	6.53138
CELSR1	0.388476	0.344613	5.53027	5.60111	-2.13612	-3.38329
CELSR2	4.97599	4.48333	3.02837	2.28779	5.24845	4.46946
CELSR3	3.07407	3.05482	4.55456	4.77458	2.96723	3.02461
CEMIP	1.52979	1.67161	3.32977	3.79148	-0.878698	-1.18284
CEND1	-2.05464	-0.818747	0.401532	1.65939	-0.152369	1.77713
CENPA	3.58152	3.6657	2.79018	2.81332	3.68074	4.05231
CENPB	5.3717	5.27016	5.61453	6.01948	5.00724	5.21883
CENPBD1	2.57002	2.46195	1.57806	1.6921	1.18521	1.13881
CENPBD1P1	3.69845	3.71147	3.29752	3.61964	4.476	4.77271
CENPC	4.17403	4.16523	3.93478	3.88404	4.44776	4.41056
CENPCP1	1.08511	1.16944	1.08786	1.02103	1.33236	1.26229
CENPE	6.53914	6.66072	6.22953	6.51738	5.71874	6.41483
CENPF	9.09303	9.18631	8.36741	8.64377	8.75454	8.89338
CENPH	3.30233	3.32832	3.14291	3.45532	3.34812	3.75066
CENPI	4.21679	4.2254	4.42749	4.76075	4.08537	4.34992
CENPJ	4.86125	4.87479	5.15462	5.41076	3.96083	3.75067
CENPK	3.48704	3.76957	3.90128	4.15209	3.89427	4.32533
CENPL	3.92571	3.91544	3.27796	3.67417	3.72554	4.06147
CENPM	2.66506	2.46198	2.8933	3.09819	2.2008	2.53647
CENPN	4.32988	4.3927	3.83952	4.46978	3.73418	4.3968
CENPO	5.02749	5.08164	4.31223	4.5573	4.66451	4.52746
CENPP	3.50358	3.44858	3.06571	3.22201	2.71686	2.75536
CENPQ	2.93755	3.17197	2.42012	3.04852	3.44821	4.42899
CENPT	3.3697	3.27015	4.0477	4.23547	4.21467	3.5856
CENPU	4.91227	5.10194	3.73813	3.84135	3.98041	4.26505
CENPW	2.06912	1.94657	1.74132	2.02048	2.61583	3.01439
CEP104	3.52371	3.43803	3.3498	3.72406	3.97811	3.71324
CEP112	1.74388	1.64571	2.99123	3.07344	0.529541	0.224139
CEP120	4.38838	4.44553	3.99265	4.43805	4.33687	4.69523
CEP126	-0.395122	-0.375208	2.85266	2.77076	1.41119	1.78517
CEP128	4.2153	4.42741	3.58301	3.83954	2.91712	2.74451
CEP131	2.99597	2.76561	2.65569	2.89961	2.34217	2.10626
CEP135	3.49936	3.84093	2.21737	2.38704	4.83103	4.81389
CEP152	5.31179	5.23946	5.27038	5.33847	4.6696	4.25132

CEP162	3.86886	3.55813	3.00383	2.74916	2.95624	3.2677
CEP164	4.00914	4.1417	4.44035	4.61653	3.80729	3.84787
CEP164P1	-0.13522	0.362666	0.310131	0.337174	0.14496	0.0480548
CEP170	7.10557	7.12986	6.31224	6.2946	6.43248	6.20921
CEP170B	3.61832	3.34129	2.53074	2.5494	2.58762	2.43285
CEP170P1	2.37222	2.59311	1.61806	1.52149	1.63704	1.20699
CEP19	0.604783	0.432061	2.45674	2.73038	1.40582	1.24753
CEP192	5.24964	5.19669	5.62463	5.70413	5.51617	5.83873
CEP250	6.40433	6.34	5.51768	5.71584	5.49684	4.60842
CEP290	4.60848	4.62481	5.0789	5.07868	4.39551	4.67984
CEP295	4.99759	4.93274	5.75115	5.82249	5.09348	4.8954
CEP295NL	0.598413	0.596954	0.93647	0.752877	0.441303	0.0242771
CEP350	7.48371	7.40188	6.66764	6.75419	6.47203	6.45755
CEP41	3.73032	3.52015	4.39955	4.46954	3.90478	3.98328
CEP44	3.4858	3.67032	3.63183	3.74378	3.2105	3.31129
CEP55	5.57788	5.70301	4.67834	5.24817	5.81691	6.18543
CEP57	4.53272	4.67029	5.25474	5.51456	4.66836	5.10385
CEP57L1	2.38811	2.70472	3.49979	3.62015	3.59592	3.8623
CEP57L1P1	-0.789334	-0.441546	0.486436	0.596859	0.909443	1.3534
CEP63	3.51896	3.35168	3.4513	3.71734	2.98766	2.90043
CEP68	3.86214	3.788	3.92097	3.95701	3.24264	2.98702
CEP70	3.73554	3.56506	3.4889	3.66924	3.18204	3.74656
CEP72	2.41894	2.35109	2.43559	2.29404	1.59248	1.0747
CEP76	2.24683	2.44725	1.97617	2.656	2.61286	2.96534
CEP78	4.97157	5.05087	4.35466	4.52325	4.59116	4.57984
CEP83	3.96933	4.04473	3.00793	2.99447	3.17384	3.6207
CEP85	5.1235	5.17423	4.74424	4.94373	5.05007	4.89876
CEP85L	3.93664	3.89691	3.77212	3.75381	4.72445	4.58675
CEP89	3.2048	3.33481	3.9301	4.1462	3.83954	3.52691
CEP95	4.45457	4.3492	4.39597	4.35472	4.6479	4.19852
CEP97	4.88352	5.2087	4.83328	5.22767	4.26449	4.42642
CEPT1	4.30484	4.38618	4.60436	4.67148	4.42217	4.88588
CERCAM	4.03647	3.97282	3.89328	4.0023	2.92841	2.7609
CERK	5.2667	4.60323	5.65921	5.52234	5.05607	4.28197
CERKL	4.20329	4.19625	1.5913	1.77384	3.16942	3.27628
CERS2	6.77064	6.82295	6.57118	6.96705	6.76776	6.87568
CERS4	0.631914	-0.140771	0.849967	0.978694	3.02794	2.87825
CERS5	4.09939	4.08982	4.21093	4.40391	3.77328	3.64864
CERS6	5.46337	5.85149	-1.031	-1.32478	5.09032	5.39938
CES1	-5.69162	-5.45651	2.34864	2.87086	?	?
CES2	3.66287	3.66772	4.54184	4.94644	4.21769	4.22253
CETN2	3.99004	3.91977	3.8263	4.15326	3.81946	4.3087
CETN3	2.15269	2.12963	3.14964	3.31744	3.02093	3.75476
CFAP20	3.67813	3.7466	3.21949	3.82338	4.56368	4.85553
CFAP36	3.01001	3.08212	3.37682	3.79204	3.7512	4.22254
CFAP44	3.23122	3.30006	2.40558	2.38748	3.41205	2.85009
CFAP69	0.710384	0.491443	2.35177	2.30482	-0.729347	-1.53638
CFAP97	5.4302	5.64243	4.86631	5.19836	5.47507	5.76036
CFB	-0.470075	-0.0533185	2.23349	3.01575	1.66615	1.90802
CFDP1	3.90825	3.92732	3.98765	4.07199	3.84228	3.80898
CFH	-3.33729	-3.73464	4.9817	4.72299	1.84493	3.0514
CFI	4.41921	3.98008	2.70218	2.36286	1.89813	1.70271

CFL1	8.57807	8.62657	7.84462	8.31163	8.48224	8.56535
CFL1P2	1.29013	1.69706	0.546699	1.48451	1.33513	1.33673
CFL2	4.30303	4.36845	3.80941	4.28563	5.18043	5.69363
CFLAR	4.93896	5.1265	3.99231	4.16119	4.7609	4.41803
CFLAR-AS1	0.460533	0.690542	-0.564595	-0.0494644	-0.0556498	-0.141288
CFTR	-5.10914	-4.87366	2.30433	2.16265 ?	?	
CGGBP1	5.89895	5.98346	5.69468	5.85208	5.66956	5.61056
CGN	2.1713	2.17017	0.138526	0.334214	2.61349	2.11986
CGNL1	-2.61075	-3.65369	-0.568612	-0.585061	5.61285	5.2862
CGREF1	1.53121	1.38173	-3.59683	-2.54535	-0.736141	-1.28049
CGRRF1	2.32053	2.37952	1.76487	2.07607	2.20389	2.908
CH17-189H20.1	1.71409	1.9184	1.47272	1.02565	0.722597	0.211349
CH17-258A22.4	0.461214	0.495009	1.80281	1.44149	1.61687	1.20646
CH17-260O16.1	-0.313689	-0.166375	0.819807	0.29813	0.234072	-0.115727
CH17-264L24.1	1.47076	1.26464	2.1471	1.95729	0.415586	0.246201
CH17-373J23.1	-5.69162	-4.45968	2.78515	2.64941	-2.26731	-1.79933
CH17-431G21.1	0.867533	0.653867	1.42922	1.40492	1.22753	0.695593
CH17-472G23.2	0.542129	0.503939	-0.337661	0.239117	0.489235	-0.147268
CH17-472G23.4	1.66629	2.21654	4.14469	4.2268	3.98721	4.03043
CH507-145C22.1	2.13389	2.05447	1.37813	1.98646	-0.0800391	-0.116184
CH507-145C22.3	2.02663	1.79487	1.4978	1.63778	0.577683	0.0426724
CH507-154B10.1	3.7083	3.80467	3.50542	3.54341	1.51987	0.633961
CH507-154B10.2	3.61772	3.79649	3.22723	3.10194	1.3223	1.15706
CH507-24F1.1	2.29262	2.42765	2.01396	1.68319	-0.352678	-0.0198986
CH507-24F1.2	2.60544	3.08733	2.45553	2.80775	1.43902	1.33907
CH507-254M2.1	0.992239	0.946259	1.03122	1.75458	0.377479	1.11984
CH507-254M2.2	2.04513	1.77167	0.777889	1.50972	1.26064	2.12338
CH507-254M2.3	2.45164	2.48649	2.60481	2.40871	0.818335	0.190615
CH507-338C24.1	4.82527	4.83048	4.78667	4.59197	2.63291	2.154
CH507-42P11.8	-0.550148	-1.38167	-0.897683	-0.540957	2.63423	1.81073
CH507-513H4.3	1.02713	3.41311	7.42801	1.43329	-1.80359	0.46069
CH507-513H4.4	7.75031	8.61713	12.6292	6.91864	5.10807	6.66148
CH507-513H4.5	-0.397672	1.75668	5.58746	-0.388716	-3.11286	-2.45144
CH507-513H4.6	1.20632	3.55025	7.89051	1.4753	-1.71001	0.933913
CH507-528H12.1	1.08246	0.464396	1.51245	1.24889	-0.946458	-1.84822
CH507-9B2.3	2.37422	2.07665	3.78822	4.07225	3.54844	4.1479
CH507-9B2.4	0.830961	0.236903	0.236082	0.172667	0.851719	0.0433725
CH507-9B2.5	3.0844	2.95934	3.43115	3.63406	4.25923	4.11761
CH507-9B2.9	1.48736	1.38065	2.35676	2.73026	2.54763	2.06188
CHAC1	-0.395122	-0.485378	-0.300715	0.491176	1.97271	1.1637
CHAC2	2.18663	2.58156	1.55122	1.70499	2.76176	3.08771
CHAF1A	4.84587	4.77973	4.51605	4.67907	5.00502	4.46195
CHAF1B	2.95735	3.02092	3.97129	4.48362	3.36067	3.16673
CHAMP1	4.04995	3.88919	3.56802	3.73979	3.33404	3.35668
CHCHD1	3.14641	3.12961	2.57804	3.05356	3.76386	4.69949
CHCHD10	1.17748	1.16657	2.4083	2.8827 ?	?	
CHCHD2	6.10553	6.05701	5.87103	6.45002	6.27746	7.15944
CHCHD2P6	2.88692	2.68393	2.66197	3.2352	3.01984	3.87924
CHCHD3	5.06153	4.98997	5.15478	5.54663	5.77805	5.94307
CHCHD3P3	2.3348	2.33628	2.42976	2.90791	3.15039	3.35111
CHCHD4	2.80871	2.93578	2.19588	2.96264	3.02962	3.56476
CHCHD5	1.52584	1.52019	0.977632	1.01017	1.53466	2.08121

CHCHD6	0.908313	0.938098	1.11115	1.25754	0.714804	0.993783
CHCHD7	3.41466	3.27356	3.15187	3.37297	3.06421	3.47071
CHD1	6.74829	6.71916	6.18711	6.23822	6.62723	6.60874
CHD1L	5.37578	5.40136	4.43441	4.62139	4.85025	5.03152
CHD2	6.13736	6.04018	6.32056	6.35323	6.68427	6.1661
CHD3	6.07158	5.83547	6.6878	6.71371	6.53793	5.90661
CHD4	8.21674	8.22048	7.67274	7.79398	8.37877	8.01372
CHD6	7.09414	7.11207	6.75549	6.80292	5.97472	5.53705
CHD7	6.34137	6.21001	5.12765	4.78598	3.76386	2.95863
CHD8	6.55696	6.58341	5.96526	6.14796	6.44244	6.04982
CHD9	6.21144	6.15045	6.30812	6.31948	7.10916	7.23102
CHEK1	4.40289	4.55743	4.20879	4.53592	4.79398	4.89691
CHEK2	3.69946	3.65089	3.15096	3.26628	2.17522	2.43546
CHERP	2.9407	2.60145	2.54893	2.44411	3.13834	3.02463
CHFR	3.21381	3.09175	2.34788	2.15331	3.51959	3.27354
CHI3L1	-5.69162	?	1.68069	3.30903	?	-4.70296
CHIC1	3.46331	3.6509	3.84438	4.29525	4.87301	5.32187
CHIC2	1.75628	1.84096	1.51	1.61231	4.50144	4.93443
CHID1	5.30478	5.181	5.04589	5.24707	4.81034	4.85933
CHKA	3.59701	3.3489	3.60984	3.67484	3.01509	2.44551
CHKB	3.31036	2.84557	3.48876	3.52813	2.70515	2.27344
CHKB-CPT1B	0.784194	0.720918	-0.129527	-0.000613276	-1.96728	-0.157439
CHM	4.09533	4.08018	4.17182	4.4342	4.83504	5.13405
CHML	5.66395	5.37556	5.84817	5.74642	6.3786	5.79561
CHMP1A	4.77157	4.70393	4.47735	4.85984	4.71471	4.85839
CHMP1B	3.35067	3.11997	4.40388	4.7253	4.56451	4.43945
CHMP2A	4.42761	4.19446	4.37489	4.42935	5.23943	5.37875
CHMP2B	4.64354	4.75146	3.54099	4.05607	4.04703	4.34041
CHMP3	5.24462	5.15511	4.40752	4.55755	5.63862	5.7107
CHMP4A	1.76367	1.81174	1.37558	1.52378	2.11812	2.16835
CHMP4B	4.4202	4.32171	4.36976	4.67342	4.30255	4.27317
CHMP4BP1	0.393405	0.167897	0.215045	0.0392507	0.0682168	-0.569922
CHMP5	4.38937	4.53003	4.08736	4.5343	6.04308	6.73325
CHMP6	2.45575	2.21313	2.54781	2.70817	2.88659	3.10386
CHMP7	4.77717	4.69892	4.60763	4.90027	4.38475	4.65741
CHN1	3.93391	3.91001	3.92107	4.26621	6.09538	6.77961
CHORDC1	4.8852	4.88969	6.03678	6.05529	5.29349	4.92573
CHP1	4.99309	4.7405	4.6876	4.97664	5.40405	5.32533
CHPF	4.52222	3.94941	4.42382	4.27056	4.59382	4.76189
CHPF2	5.92139	5.86697	6.11512	6.45987	6.48886	6.55039
CHPT1	4.98156	5.22275	4.0968	4.5353	3.99553	4.47386
CHRAC1	3.39895	3.42895	3.00877	3.42645	3.92276	3.60389
CHRDL1	5.27996	4.86415	?	-6.09892	2.95808	3.73623
CHRM3	4.10978	3.59187	0.386425	0.111031	1.95201	1.34861
CHRNA5	2.38906	2.59493	2.22532	2.59344	3.52263	4.01615
CHRNB1	0.539412	0.456112	1.27595	1.32585	2.15669	2.25909
CHRNB2	1.20786	1.23706	1.07761	0.615671	-0.233893	-0.981411
CHST1	2.42238	2.8016	1.20884	1.87419	2.00851	2.69418
CHST10	4.13143	4.22801	2.48892	2.65605	3.4983	3.44304
CHST11	7.01388	6.82329	3.1541	3.67417	2.45864	2.25909
CHST12	3.79176	3.56367	3.85814	3.65438	3.10456	2.84404
CHST14	4.65075	4.3581	3.80517	3.6494	3.05908	2.82077

CHST15	2.19576	2.20251	5.59536	5.59073	-5.07051 ?	
CHST2	2.24052	2.60056	0.82777	1.34243	2.22845	2.99719
CHST3	5.98982	5.78981	4.02227	4.03837	6.62595	6.07422
CHST6	4.21829	4.31193	-4.59508	-3.78492	-3.07415	-2.70579
CHST7	-0.026521	0.076436	1.12941	1.49112	1.28225	1.78517
CHSY1	6.61886	6.38537	5.44993	5.54983	6.06976	5.14189
CHSY3	2.07902	1.69706	-4.18092	-6.09892	0.210164	0.151378
CHTF18	2.76036	2.47895	3.13841	3.27217	2.08454	1.9868
CHTF8	4.66122	4.64502	4.91385	5.22153	5.73521	6.17095
CHTOP	5.19406	5.17367	4.73937	4.92763	5.4101	5.26833
CHUK	4.57353	4.76475	4.17072	4.66512	5.32126	5.7786
CHURC1	4.57641	4.68518	3.35564	3.58553	3.92458	4.76548
CIAO1	5.85478	5.94603	4.85676	5.02237	6.35631	6.57171
CIAPIN1	4.66296	4.75441	4.47503	5.0323	5.55201	5.95774
CIAPIN1P	1.04114	1.23175	1.05118	1.73307	2.01045	2.18189
CIART	0.710384	0.581631	0.460428	0.95732	1.71041	1.12623
CIB1	2.97688	2.89898	3.15849	3.73034	3.64004	4.04817
CIC	3.70179	2.97596	3.94962	3.70175	4.55885	4.4187
CICP14	2.69508	2.40656	2.71269	2.67967	2.81905	2.15232
CIDECP	1.69756	1.74664	1.59787	2.13437	2.19146	1.81688
CIITA	4.7782	4.12257	2.74648	3.1301	-0.706947	-1.14273
CINP	3.75603	3.63848	2.58397	2.95646	3.72968	3.9925
CIPC	4.11149	4.31162	2.59127	2.91208	3.68065	3.69392
CIR1	3.23687	3.1307	4.18156	4.33898	3.3605	3.66321
CIR1P1	-0.506845	-0.39658	0.431281	0.798133	-0.682812	-0.214706
CIRBP	5.10181	5.04444	5.20824	5.23823	6.15607	5.95727
CIRBP-AS1	0.278306	0.076436	-0.0541032	-0.0426024	0.293994	0.247587
CIRH1A	4.38173	4.26592	4.00506	4.26946	5.22003	5.19037
CISD1	3.30935	3.49717	3.53415	3.88269	4.11282	4.66941
CISD2	4.12974	4.10874	3.83465	3.91617	4.6559	4.25313
CISD3	2.95102	2.97673	3.04908	3.41335	3.62909	4.07735
CIT	7.18163	7.20819	5.53415	5.64063	4.61172	4.37942
CITED2	1.25516	1.11464	4.02592	3.81895	3.84549	3.54358
CITF22-1A6.3	1.21307	0.841893	1.81614	1.78543	0.249081	0.959941
CIZ1	4.19346	4.18641	3.85813	4.11396	4.38612	4.2975
CKAP2	5.54793	5.71144	5.06261	5.57328	5.24545	5.54061
CKAP2L	6.84304	7.03987	4.72994	5.02117	4.62605	4.89212
CKAP4	7.7031	7.82883	7.32624	7.91535	7.19659	7.78479
CKAP5	7.06094	7.04801	7.07844	7.37459	7.60959	7.36887
CKB	0.952035	0.305445	-0.973801	-0.860651	5.50366	5.55243
CKLF	3.10627	3.27739	3.18445	3.67866	4.25697	5.02681
CKMT2	0.0954519	0.237698	1.31637	1.13916	-0.338214	-0.619042
CKMT2-AS1	1.80477	1.79928	2.53416	2.64941	1.84751	1.50278
CKS1B	4.99717	4.97574	4.34997	4.97494	4.55948	5.01841
CKS1BP3	2.75368	2.70388	2.11083	2.65695	2.37983	2.76472
CKS2	4.47147	4.53644	3.84576	4.39001	5.04443	6.10706
CLASP1	6.99449	7.26379	6.51866	6.54236	5.68239	5.26055
CLASP2	5.42858	5.59312	4.45491	4.73822	5.33226	5.6152
CLASRP	3.0186	2.81328	3.0926	3.0611	3.59116	2.77307
CLCA2	-4.11163	-4.87366	2.22164	2.63602	-4.33548	-4.70296
CLCC1	4.86268	4.953	5.15034	5.24705	5.41165	5.10278
CLCF1	1.68033	1.42594	0.73541	1.2041	2.57332	2.83245

CLCN2	1.82842	1.75635	1.7707	1.90801	2.30549	2.48805
CLCN3	5.91423	5.93453	4.76557	5.0859	6.36759	6.50241
CLCN4	3.35621	3.57194	3.30231	3.72722	2.4767	2.908
CLCN5	5.95601	5.41786	3.47647	3.21536	2.99254	2.47817
CLCN6	3.58294	3.24205	3.56759	3.64948	3.67261	3.37466
CLCN7	4.00007	3.42033	4.23275	4.10428	3.95738	3.30029
CLDN1	0.0289651	0.237698	-0.428455	-0.000171691	1.96542	0.597145
CLDN11	0.399051	0.0918691	1.91979	1.97335	-2.48858	-3.38329
CLDN12	5.88888	5.73627	5.11603	5.1893	5.39092	5.1881
CLDN15	1.82451	1.38268	1.11115	1.21315	1.33409	0.422664
CLDN18	0.133932	0.395227	0.723445	0.414828	-0.932127	-1.12145
CLDN20	0.784805	0.581631	0.686917	0.535117	0.983605	0.463298
CLDN4	0.0559314	-0.195907	0.636738	0.810106	0.147163	-0.38461
CLDND1	5.56682	5.64957	5.30179	5.55916	4.41315	4.89968
CLEC16A	3.61267	3.70833	3.83745	3.95995	3.767	3.64643
CLEC18A	1.72631	1.44831	-5.59166	-2.45428	-3.21128	-3.15974
CLEC2B	?	?	3.23656	3.60091	3.63916	4.58685
CLEC2D	1.23238	1.22563	1.94503	2.29656	2.16367	2.22785
CLEC7A	?	?	1.5913	0.619178	1.59711	0.937201
CLGN	1.51041	1.45606	2.46395	2.71773	3.79811	4.4707
CLHC1	1.61837	1.91324	2.34397	2.24394	2.1594	2.22994
CLIC1	6.34972	6.41131	6.49052	7.12866	7.37099	8.07582
CLIC1P1	0.705412	0.959941	1.25524	1.73943	1.84826	2.28641
CLIC2	-2.61075	-2.6546	2.35177	3.01271	-0.374735	0.463298
CLIC4	7.08648	7.00733	6.97386	7.40342	7.41645	7.52905
CLIC4P1	2.48372	2.16764	2.24222	2.54937	2.40894	3.19799
CLIC4P3	1.64547	1.69043	1.52658	1.7854	1.96966	1.9602
CLINT1	5.70445	5.79128	6.14514	6.54848	6.45811	6.5527
CLIP1	6.01143	6.01236	5.58755	6.01513	6.18221	5.97676
CLIP2	3.02884	3.07632	4.45762	4.74525	4.38406	4.17287
CLIP3	1.60929	1.08411	1.5647	1.45345	2.02971	1.91536
CLIP4	5.88069	6.01313	5.11854	5.56536	5.95278	6.05566
CLK1	4.99397	4.90019	4.09956	4.24872	5.73119	5.75425
CLK2	4.50132	4.35845	3.99685	3.91839	4.42112	3.72965
CLK2P1	0.869319	0.742679	0.131774	0.407026	0.715401	0.0791016
CLK3	5.14078	5.01936	5.44199	5.55025	5.31558	4.79079
CLK4	4.26129	4.32389	4.68153	4.57543	4.02249	3.93726
CLMN	6.51874	6.47123	2.16964	2.81302	-2.20023	-1.79933
CLMP	5.09776	5.04097	5.49331	5.87843	5.0841	5.75066
CLN3	2.61608	2.68182	2.85738	3.18812	3.20388	3.13564
CLN5	3.89601	3.83322	3.66322	3.90273	4.41193	5.12121
CLN6	4.40158	4.0821	4.23535	4.17308	4.40909	4.0126
CLN8	3.43285	3.57196	3.9723	4.05592	4.52775	4.36838
CLNS1A	6.46675	6.45189	5.03684	5.35111	5.28731	5.61352
CLOCK	5.52248	5.66706	5.15465	5.28883	6.57895	6.9765
CLP1	3.30794	3.33805	2.58136	3.11999	3.40328	3.78923
CLPB	5.31459	5.21841	4.20987	4.44959	3.88078	3.80503
CLPP	2.69753	2.51445	2.17405	2.49851	3.74908	3.91533
CLPTM1	4.99484	5.0989	5.46077	5.76729	5.63344	5.77204
CLPTM1L	5.9163	6.00043	4.92498	5.34392	6.68221	6.87846
CLPX	4.68083	4.60188	4.31532	4.41669	4.70218	4.6073
CLSPN	5.287	5.38635	4.68203	4.85102	5.07594	4.64963

CLSTN1	6.53124	6.54331	6.05334	5.91733	6.70816	6.49261
CLSTN2	-6.68418	-2.00285	8.26595	7.56407 ?		-5.69922
CLSTN3	3.55595	3.19537	3.32823	3.25312	2.0086	1.74453
CLTA	5.25324	5.1546	4.92692	5.36233	6.92329	7.14832
CLTB	3.1589	2.90785	2.72637	3.31324	3.96265	4.24748
CLTC	9.00614	9.02403	8.86563	9.1689	9.70772	9.73784
CLTCL1	3.08883	2.9187	3.37107	3.28134	2.26748	1.76905
CLU	1.04244	1.49139	6.62282	7.32773	5.88078	5.35127
CLUAP1	2.91797	2.92363	3.27386	3.39302	2.45084	2.25331
CLUH	4.24686	4.00809	4.15521	4.33618	4.80014	4.51969
CLUHP3	2.17438	1.91656	2.23435	2.15329	0.740643	-0.121607
CMAS	4.30022	4.40364	3.65253	3.84681	3.23754	3.50274
CMB9-55A18.1	1.19245	1.46804	1.79797	1.8027	1.32695	0.914013
CMB9-55F22.1	0.840145	0.548466	0.986462	0.999755	-0.777435	-1.53638
CMBL	2.62286	2.60594	4.60192	4.81523	2.42451	2.82468
CMC1	3.16358	3.18301	3.03787	2.93357	3.30169	3.86455
CMC2	4.39489	4.55125	4.32843	4.40782	3.79193	4.38306
CMC4	0.590386	0.669762	1.23844	1.23698	2.04684	2.91844
CMIP	2.83036	2.64045	3.26356	3.09085	2.46124	2.00755
CMPK1	5.98104	5.64926	6.0146	6.0529	6.62347	6.33392
CMSS1	4.28961	4.36924	3.66197	4.07605	3.70163	4.23138
CMTM3	3.3219	3.48406	3.87582	3.95996	3.90955	4.57522
CMTM4	4.73658	4.89801	4.97065	5.12417	5.28987	5.26194
CMTM6	6.4986	6.67139	5.98387	6.50816	6.43587	6.88417
CMTM7	5.2194	5.12544	-3.18222	-2.78461	4.10136	3.56849
CMTM8	1.35081	1.03706	1.87179	2.03585	2.85145	2.91534
CMTR1	4.80623	4.76235	5.06736	5.30796	5.38715	5.42385
CMTR2	3.82056	4.12867	4.13953	4.42061	4.87739	5.55538
CMYA5	1.36166	1.02906	-0.375995	-0.55404	1.80324	1.42261
CNBP	6.45906	6.59515	5.62663	6.18952	6.64161	7.25656
CNDP2	5.02968	4.98989	5.00568	5.4719	3.74483	3.7991
CNEP1R1	2.64965	2.6587	3.24274	3.57942	3.82123	3.95826
CNIH1	5.59668	5.79747	4.75678	5.20801	5.45278	6.13877
CNIH2	0.850599	0.726849	-0.959641	-0.842149	0.185296	0.0483059
CNIH3	0.207918	0.318612	1.51	1.82784	-0.23389	0.483199
CNIH4	4.84587	4.76646	4.82198	5.1943	4.98744	5.57114
CNKSR2	2.68031	2.65351	-6.17309 ?		2.07442	1.75275
CNKSR3	3.37372	3.80842	4.16936	4.23781	5.07753	4.30988
CNN2	4.71979	4.25329	5.4401	5.26369	6.28373	5.58284
CNN3	6.95126	6.88076	6.23542	6.74949	8.72626	8.82142
CNNM2	3.67725	3.44852	4.73037	4.52618	4.24686	3.76825
CNNM3	3.79641	3.88702	3.14639	2.91638	3.32498	2.95949
CNNM4	3.73346	3.7405	3.26563	3.25093	3.4165	2.92629
CNOT1	6.73916	6.61723	7.55576	7.71587	8.01618	7.78004
CNOT10	4.43341	4.52442	3.4108	3.81747	4.46122	4.92719
CNOT11	4.33984	4.44704	3.85265	4.19495	4.69998	4.88853
CNOT2	5.67991	5.61936	5.07442	5.14617	4.98404	5.05435
CNOT3	3.32108	3.02307	3.43379	3.17553	3.23714	2.69129
CNOT4	3.77259	3.72944	3.87717	3.80909	3.54426	3.14504
CNOT6	4.73762	4.72203	5.58508	5.58814	5.24918	5.22254
CNOT6L	4.52285	4.59769	4.45529	4.37369	4.11945	4.11296
CNOT6LP1	0.514743	0.796797	0.811767	1.19757	0.246092	0.277794

CNOT7	5.69159	5.72093	5.55873	5.69265	5.99376	6.37656
CNOT7P1	0.207918	0.392878	0.150729	0.237662	0.154778	0.708028
CNOT8	3.97776	3.97594	4.74795	5.04978	4.7821	4.92899
CNP	6.30822	6.21604	5.55994	5.86017	6.71691	6.48685
CNPPD1	3.22822	3.13627	3.38221	3.65597	4.45907	4.5255
CNPY2	5.98792	5.79203	4.55879	5.04473	4.60564	5.42845
CNPY3	5.15773	4.98629	3.42935	3.6494	4.75595	4.84882
CNPY4	3.95814	3.57312	3.95344	3.86675	2.74012	2.56895
CNR1	1.22578	1.57061	-6.17309	-5.10387	7.67148	7.83872
CNRIP1	3.68678	3.71706	4.21842	4.73116	4.19689	4.74139
CNST	4.69	4.69322	4.35466	4.5485	4.9849	4.85933
CNTD1	0.617054	1.09329	0.604565	0.465222	1.06392	0.891917
CNTF	1.17663	1.34632	1.36867	1.55857	0.668927	0.305048
CNTLN	3.95465	3.94229	4.10649	3.84972	4.6812	4.93983
CNTN1	2.16202	1.74176	-5.1783	?	?	-5.69922
CNTNAP1	4.8548	4.66183	3.98667	4.03831	2.64403	2.58747
CNTNAP2	-1.61119	-1.81849	-3.85951	-3.52222	3.46381	3.78714
CNTNAP3	5.69257	5.816	4.32511	4.42546	5.19629	5.0462
CNTNAP3B	5.83251	5.82419	3.21309	3.04827	3.7895	3.44571
CNTNAP3P2	3.29862	3.4177	0.602818	0.468823	1.49035	0.792072
CNTRL	4.15735	4.33725	4.20772	4.28348	3.61697	3.54594
CNTROB	3.88682	3.83636	4.11224	4.18004	4.65426	4.21215
COA1	5.91585	5.93642	5.72598	5.86235	5.83403	5.99848
COA3	3.74374	3.56545	3.76287	4.13647	4.0308	4.39364
COA4	4.89757	4.88808	3.98514	4.42644	5.12267	5.5191
COA5	3.52853	3.44554	3.35758	3.44	3.88754	4.56708
COA6	2.36971	2.36343	2.84076	3.27757	3.51774	4.06455
COA7	4.94296	5.107	3.68066	4.34752	4.42315	4.57984
COASY	4.1155	3.76837	4.99139	5.13791	4.68675	4.31009
COBL	3.10182	3.05876	-6.17309	-5.10387	-5.65305	-4.70296
COBLL1	3.94116	3.88034	2.62384	2.83955	3.73522	3.47568
COG1	3.41697	3.51022	4.22437	4.31409	4.77957	4.63317
COG2	4.23754	4.15462	3.76338	4.03837	4.26228	4.5281
COG3	3.93685	4.12651	4.04696	4.53244	4.12582	4.62838
COG4	4.57122	4.54067	5.06499	5.39897	5.64459	5.78814
COG5	5.15721	5.38361	4.624	5.00722	4.71684	5.07723
COG6	3.18204	3.33158	2.89597	3.4342	3.48563	4.15126
COG7	2.57222	2.45671	2.7111	2.95875	2.59498	2.6097
COG8	4.20065	4.19105	4.18828	4.35464	5.4404	5.16361
COIL	3.90547	4.11646	3.51159	3.92169	4.43963	4.51363
COL10A1	0.878411	1.20963	2.37108	2.4911	0.509706	0.270672
COL11A1	9.24251	8.27531	-2.48229	-2.20128	8.33942	7.19378
COL11A2	4.08881	2.8903	-0.598354	-0.649181	-3.07415	-5.69922
COL12A1	7.87749	7.86717	8.66091	8.73024	7.52813	7.50778
COL13A1	0.301026	0.850103	-0.973801	0.020584	2.21315	1.87074
COL14A1	-2.17488	-2.6546	2.10652	1.99449	5.38031	5.36808
COL15A1	2.40946	2.05288	-4.59508	-6.09892	-5.07051	?
COL16A1	2.86126	2.59245	4.49419	4.72248	0.519683	0.90806
COL18A1	4.18953	3.59035	5.62021	5.51843	5.23764	5.62541
COL19A1	6.54646	6.45414	-1.42826	-1.93836	0.210164	0.832509
COL1A1	0.0425183	-0.604659	1.15638	0.563685	1.79093	1.77713
COL1A2	-1.1752	-1.105	8.99297	8.66843	9.27245	9.19574

COL21A1	-3.69722	-3.13966	-1.42826	-4.10635	5.31373	5.60447
COL22A1	4.29738	4.58496	-3.37468	-3.52222	-6.64581	-4.11926
COL24A1	-0.323873	-0.531913	-0.69145	-0.786664	2.26748	1.2122
COL27A1	4.87786	4.73067	4.32228	4.09941	2.15349	0.541297
COL3A1	-1.27248	-1.60444	4.73011	1.23107	-3.07415	-5.69922
COL4A1	6.20065	5.89634	5.45444	5.15326	5.63918	4.88667
COL4A2	6.65418	6.47967	6.61857	6.40514	6.02289	5.56882
COL4A3BP	4.97407	4.59513	4.50034	4.69126	4.555	4.52089
COL4A4	1.60474	2.20606	-3.01245	-2.20128	0.983605	0.816919
COL4A5	4.16588	4.53079	1.43857	0.57776	5.74191	5.77406
COL4A6	4.76698	5.25952	-1.65952	-1.93836	4.1029	4.48926
COL5A1	-2.79121	-1.93875	6.98646	6.64668	5.22042	4.82368
COL5A2	-0.0124477	0.292146	7.95649	7.96547	7.53678	7.58456
COL5A3	4.47581	3.64944	-5.1783	-6.09892	-6.64581	?
COL6A1	4.53335	4.22103	7.00007	7.10146	4.98939	5.37808
COL6A2	3.99091	3.58428	6.89147	6.88301	4.64517	4.91166
COL6A3	-1.94365	1.66647	11.8314	11.8295	1.79093	2.06809
COL7A1	2.51282	2.22717	6.52234	6.66453	2.35372	1.99375
COL8A1	7.95545	7.79373	6.94848	7.55006	3.4731	3.0041
COL9A1	1.21983	1.30538	?	?	-5.65305	-4.70296
COL9A2	1.90457	1.33161	-3.85951	-2.93781	0.0540566	-0.848487
COL9A3	5.40682	4.76956	-2.48229	-3.30006	3.21467	2.28766
COLEC12	-1.69862	-1.25306	6.3818	6.7095	-1.04532	0.0468961
COLGALT1	6.802	6.63088	6.16496	6.37723	7.31509	7.23873
COLGALT2	2.44814	2.6587	0.460428	-0.327299	4.17889	3.70903
COMMD1	2.92746	3.21771	2.7055	2.94135	3.40467	4.1775
COMMD10	3.26815	3.30159	3.44219	3.82553	3.52457	4.23259
COMMD2	3.76239	3.96969	3.96241	4.36792	4.68731	5.25617
COMMD3	2.28773	2.63522	1.79278	2.40979	2.47649	3.59844
COMMD3-BMI1	0.669789	-0.916472	-2.33217	-1.03513	0.918928	2.50219
COMMD4	4.76239	4.56298	4.35854	4.64939	4.22385	4.57868
COMMD5	3.09281	3.08042	3.04573	3.28152	4.02768	4.77096
COMMD6	2.94478	2.95712	2.91451	3.1858	3.82853	4.86219
COMMD7	3.38972	3.24451	3.33218	3.74136	3.85439	4.04315
COMMD8	2.75008	2.90348	2.08328	2.25313	3.88465	4.75373
COMMD9	3.83425	3.73559	3.63666	4.06485	4.27116	4.4954
COMT	4.07735	4.12023	4.20986	4.59161	4.5027	4.74655
COPA	7.05247	7.07647	6.84268	7.44243	6.97376	7.13072
COPB1	6.17724	6.33763	6.42527	7.01821	6.15133	6.66835
COPB2	6.27838	6.33865	6.58074	7.13759	6.18401	6.63314
COPE	4.00474	3.84321	3.58119	4.06735	4.84697	5.50942
COPG1	5.58399	5.58941	5.95093	6.48028	6.00526	6.05315
COPG2	4.01383	3.88353	3.49641	3.42354	3.80584	4.1291
COPRS	4.22347	4.23673	4.0465	4.44673	4.62622	4.79402
COPS2	5.77027	5.78742	5.10072	5.43324	5.94771	6.7214
COPS3	4.70977	4.79099	4.90128	5.28509	5.65027	6.10865
COPS4	3.69645	3.93163	3.606	4.14502	3.97177	5.19037
COPS5	5.02508	5.05665	4.97182	5.25265	4.88812	5.39131
COPS6	5.27567	5.35853	4.74277	5.11275	5.14099	5.40832
COPS7A	4.04742	4.01794	3.70978	4.08716	4.45474	4.90431
COPS7B	3.97812	4.02302	4.09801	4.29214	4.03412	3.95534
COPS8	4.77294	4.84198	5.33927	5.69134	5.3638	5.77122

COPS8P2	1.94417	2.04765	2.50101	2.87221	2.73087	3.26072
COPZ1	5.62791	5.48297	5.51689	5.91805	5.25854	5.47443
COPZ2	-1.05494	-0.508457	2.08328	2.52781	-0.0308197	0.315752
COQ10A	2.29883	2.33483	1.76733	1.98776	2.85539	2.84788
COQ10B	3.60356	3.57058	3.27591	3.75845	4.4773	4.79113
COQ2	2.55596	3.01289	2.05012	2.63938	2.86129	4.00753
COQ3	1.50063	1.59788	1.12031	1.46107	2.54182	3.16057
COQ4	3.48848	3.3499	3.47851	3.50811	3.58356	3.29784
COQ5	2.85744	2.89251	2.67135	2.84246	2.69505	3.48558
COQ6	2.63559	2.63131	2.08732	2.40727	2.61273	2.47915
COQ7	2.78071	2.60056	2.53073	2.4799	3.00681	2.62421
COQ9	3.55358	3.54559	4.15632	4.49571	5.57809	5.95192
CORO1A	0.594214	0.239752	0.924062	0.95732	-0.322491	-0.0948509
CORO1B	3.71877	3.29422	3.67134	4.07583	3.91144	4.48803
CORO1C	6.09876	6.45768	6.19044	6.86953	6.4632	6.87731
CORO2A	2.34806	2.54279	3.32229	3.52416	-0.682812	-0.0918635
CORO7	2.17433	1.92519	1.78624	1.8683	1.94332	1.83221
COTL1	7.27731	7.49269	5.33314	5.69571	6.00211	5.98893
COX10	3.01101	2.7577	3.39387	3.3013	3.99361	3.79612
COX10-AS1	2.28678	2.53998	3.27181	3.44768	3.15187	2.58331
COX11	4.93896	5.05415	4.62022	4.88988	4.83191	4.95155
COX11P1	0.274983	0.360499	-0.218143	0.107166	-0.109253	0.618079
COX14	3.22993	3.19695	2.34397	2.68825	3.12082	3.98504
COX15	4.66505	4.67222	5.04769	5.30849	5.25369	5.02883
COX16	3.73322	3.80507	2.93456	3.22916	3.87125	5.08729
COX17	2.60013	2.52288	2.42064	2.86245	3.05486	3.71437
COX18	2.75626	2.81561	2.79948	2.88836	3.82954	4.26914
COX19	2.39633	2.29206	2.89488	3.04915	2.86522	2.58588
COX20	3.22503	3.58817	3.39189	3.8332	4.84463	5.70414
COX20P1	0.20788	0.597612	0.304733	1.24624	1.84117	2.96291
COX4I1	6.66149	6.59107	6.49154	6.8777	6.3924	6.79535
COX5A	3.95196	3.92839	4.02715	4.50496	4.28585	4.76089
COX5B	4.21003	4.23413	3.6976	4.18117	4.91146	6.00194
COX6A1	5.1206	5.28472	4.65785	5.16061	5.33167	6.4023
COX6A1P2	3.8861	4.06112	3.59821	3.9871	4.18333	4.85134
COX6B1	4.64022	4.66061	4.60599	5.24487	5.85634	6.45689
COX6C	4.44365	4.44778	4.84713	5.14442	4.99028	6.09982
COX7A2	5.34086	5.43044	4.25319	4.70399	5.64859	6.95793
COX7A2L	5.0031	4.97596	4.75386	5.17021	5.23678	6.03981
COX7A2P2	-0.159773	0.0291348	-0.973801	-0.459493	0.270529	1.28352
COX7B	4.70978	4.73559	5.06142	5.59333	5.97654	7.13423
COX7C	5.43777	5.40904	5.55732	6.04475	6.29889	7.46814
COX7CP1	1.41066	1.50636	1.63087	2.08424	2.33539	3.28197
COX8A	6.34462	6.28952	5.2856	5.85839	5.96059	6.34346
CP	0.870132	0.142139	6.08954	5.90868	1.55084	0.752055
CPA4	3.27963	3.2184	2.70218	3.85407	4.68009	6.10825
CPA6	-3.8896	-2.87684	-1.53926	-0.716287	3.2645	3.9299
CPD	6.80558	6.58719	7.35307	7.43694	7.10356	7.03811
CPE	2.65408	2.02903	4.08794	3.89117	1.70604	1.67702
CPEB1	3.02795	3.02211	2.04532	2.40651	1.02625	1.1637
CPEB2	3.2449	3.26153	4.01974	4.27811	3.72007	3.78714
CPEB3	1.00142	0.766044	0.597917	0.491176	1.81545	1.55071

CPEB4	3.96091	4.02499	4.78658	5.09696	5.08745	4.72165
CPED1	3.97015	3.96652	4.15297	4.49015	0.848189	1.74453
CPM	2.4456	2.48261	-1.13882	-0.860651	-3.33694	-4.11926
CPNE1	5.99822	5.84681	5.81796	5.94162	5.42133	5.24427
CPNE2	2.89898	2.48261	3.58466	3.31744	3.94331	3.05816
CPNE3	5.63964	5.78299	5.42241	5.7875	6.47574	6.6514
CPNE8	2.59785	2.91654	3.01124	3.43614	-5.65305	?
CPOX	4.4895	4.36845	4.07032	4.18579	3.74271	3.33221
CPPED1	2.89339	2.60056	1.82213	1.76772	?	?
CPQ	1.71458	1.87703	2.26358	2.32998	3.57091	3.92627
CPS1	4.12985	4.14456	4.87918	5.09636	4.16148	4.22992
CPSF1	3.76546	3.50005	3.99763	4.07355	4.45926	4.05317
CPSF2	5.97643	6.06584	5.02043	5.33463	6.03295	6.26303
CPSF3	5.63712	5.53679	4.66667	5.02366	5.52336	5.86785
CPSF3L	4.41577	4.20268	4.53671	4.75225	5.01504	4.83164
CPSF4	4.05003	4.1774	3.92892	4.26403	3.89434	4.05981
CPSF6	6.41565	6.43874	5.79266	5.8922	5.95966	5.74497
CPSF7	5.3288	5.26847	5.15409	5.2176	4.88512	4.57868
CPT1A	5.62229	5.99864	5.39955	6.01044	4.71362	5.22327
CPT1B	3.18105	2.68077	2.27448	2.18043	0.859707	-0.396253
CPT1C	0.640852	0.980157	4.12253	4.3258	3.09958	3.15438
CPT2	3.61629	3.54571	3.77183	4.0456	3.76374	3.75733
CPTP	1.75628	1.60328	1.86635	2.15798	2.80934	2.91168
CRACR2A	2.95018	2.81793	-6.17309	-4.10635	?	-5.69922
CRADD	0.171399	-0.27286	0.597917	0.868521	1.89431	2.39133
CRAMP1L	3.66711	3.62728	3.77511	3.88806	3.23871	2.93839
CRAT	2.42506	2.22368	2.82771	3.12719	3.33687	3.64423
CRBN	4.44479	4.47357	4.1141	4.27381	4.44876	4.97793
CRCP	4.11561	4.20247	4.29151	4.77727	4.61789	4.80246
CREB1	5.05747	5.2267	4.65489	4.83734	5.63946	5.54298
CREB3	3.52863	3.75101	3.55928	4.26738	4.94745	5.59817
CREB3L1	-3.69722	-3.65369	2.46395	2.76155	-2.20023	-2.00571
CREB3L2	6.43026	6.45125	5.6795	5.69242	5.12148	4.91533
CREB3L4	2.22457	1.89096	1.57555	1.49095	0.816837	1.25912
CREB5	4.02968	4.09078	0.045429	-0.250687	4.10786	3.71534
CREBBP	4.65737	4.38029	5.1806	4.84245	4.397	4.07628
CREBL2	4.4114	4.21311	4.34199	4.59766	4.12186	4.68667
CREBRF	3.39236	3.13335	4.05965	4.25311	4.13085	4.31703
CREBZF	4.9957	4.98114	6.34662	6.29231	5.82676	5.06807
CREG1	4.70871	4.75328	4.0186	4.52688	2.07103	2.60618
CRELD1	3.92297	3.61129	2.8193	2.9301	2.84352	2.40704
CRELD2	4.53514	4.33075	4.20106	4.56118	2.45604	2.55069
CREM	2.73138	3.01087	1.39395	1.80409	2.2734	2.96925
CRHR1-IT1	2.78528	2.66787	2.32073	2.38119	3.08035	2.67675
CRIM1	6.27365	6.5483	6.46819	6.97895	7.7842	7.71214
CRIP2	2.21383	1.62465	-0.0748604	0.367002	2.2526	2.54596
CRIPAK	1.25516	1.06859	1.13846	0.786061	1.32842	1.0747
CRIPT	3.41859	3.48598	2.25502	2.8224	3.69793	4.30631
CRISPLD1	2.40684	2.26168	2.11112	0.924662	0.568587	0.541297
CRISPLD2	1.17133	1.94232	5.3972	5.51092	2.28229	2.36476
CRK	5.77404	5.88975	5.43071	5.76728	6.98032	6.95131
CRKL	6.19781	6.1454	5.71469	5.90449	6.13511	5.60446

CRLF1	1.39572	1.26761	1.42263	1.29788	2.23162	1.43022
CRLF3	3.59326	3.21947	3.54444	3.48005	4.23025	3.7359
CRLS1	4.00049	4.06852	4.07386	4.33307	4.26301	5.12932
CRMP1	1.78142	1.97779	4.14096	4.5834	1.26329	0.805945
CRNDE	1.68033	1.86361	2.27592	2.46104	1.39501	1.33772
CRNKL1	5.1969	5.30116	4.64381	4.92184	4.48372	4.85839
CROCC	1.65431	1.64688	1.89918	1.9267	1.58989	1.07503
CROCCP2	1.24007	1.04785	1.07452	1.06737	0.894341	0.347246
CROCCP3	2.23071	2.08075	2.69416	2.52177	1.87725	1.2068
CROT	2.96092	3.04297	5.7037	6.05009	3.38544	3.94971
CRTAP	7.28769	7.06325	6.88286	7.00161	7.22261	7.11066
CRTC1	0.577131	0.0450795	0.956086	0.445854	0.983605	0.443129
CRTC2	2.93392	2.65091	2.37874	2.8827	2.80934	2.76903
CRTC3	3.67905	3.53715	3.72611	3.68215	3.29068	2.60163
CRY1	3.55357	3.43651	3.02837	3.14384	2.78264	2.41742
CRY2	2.48084	2.282	3.39391	3.38503	2.83755	1.95865
CRYAB	-2.89067	-4.13839	-4.18092	-5.10387	5.55289	5.48995
CRYBB2P1	2.34806	2.39826	2.62335	2.78286	2.55159	2.00755
CRYBG3	5.48374	5.46167	6.77797	6.61995	5.5491	5.64709
CRYGS	-0.127589	0.017195	0.972862	1.01916	0.819513	0.625822
CRYL1	1.7188	1.15192	0.045429	-0.131403	2.1112	2.354
CRYZ	4.42591	4.80673	3.27627	3.86621	4.44624	5.11103
CRYZL1	2.68031	2.69198	3.84852	3.93008	2.85737	2.82077
CS	6.43112	6.21039	5.78085	5.92338	6.25566	5.91339
CSAD	1.34621	1.12152	2.24119	2.18501	1.95562	0.633217
CSAG1	3.66099	3.66047	-5.1783	-6.09892	4.41964	4.75356
CSAG3	1.54955	1.53364	?	?	1.64713	1.77276
CSDAP1	2.90945	3.12109	2.11616	2.35264	2.29388	2.27074
CSDE1	8.49143	8.15447	8.01147	8.03571	9.38305	9.2364
CSE1L	7.75197	7.81502	7.01576	7.48001	7.02127	7.48926
CSF1	4.53453	3.94972	7.30655	7.63082	6.26207	6.03936
CSF1R	-1.34118	-1.29258	-0.206089	0.231137	0.489522	1.24753
CSGALNACT1	3.20631	3.08791	0.904002	1.0511	-0.450672	-1.4584
CSGALNACT2	3.49075	3.669	3.00631	3.59161	4.50522	4.46195
CSK	3.45952	3.2219	3.22799	3.31534	3.30548	3.19113
CSMD2	-6.68418	-5.45651	-1.86109	-2.02079	1.32273	1.57872
CSNK1A1	6.33509	6.44982	6.7962	7.21623	7.49591	7.61623
CSNK1A1L	0.0165391	0.293712	0.699196	1.02677	1.25263	1.2124
CSNK1A1P1	-0.431686	0.0450795	-0.228674	0.213652	0.848982	0.615671
CSNK1D	4.90651	4.69433	5.27104	5.25706	6.29007	5.75165
CSNK1E	5.18531	5.04472	4.97413	5.21761	5.02794	4.54003
CSNK1G1	4.51857	4.63605	4.38992	4.63833	5.20326	5.21358
CSNK1G2	3.92113	3.64568	3.77503	3.75381	4.5146	4.3634
CSNK1G3	5.10343	5.04445	4.76337	4.76461	5.11117	5.28975
CSNK2A1	5.91227	5.90021	6.01765	6.31561	5.77658	5.81218
CSNK2A2	5.33261	5.37635	5.51561	5.66142	6.12452	5.93398
CSNK2A3	5.20232	5.14766	5.18004	5.46755	4.97054	4.89921
CSNK2B	5.07206	5.11381	4.95877	5.37195	5.93176	6.11457
CSPG4	8.14721	8.31283	-0.833889	-0.580897	-3.23558	-2.01974
CSPG4P10	2.66404	2.63303	2.66373	2.55966	2.20101	1.02112
CSPG4P11	0.626925	0.508652	2.9288	2.83183	2.02318	1.51252
CSPG4P12	2.91139	2.83304	3.53562	3.34023	2.79659	1.80826

CSPP1	3.68495	3.68477	4.61803	4.46415	3.44622	3.32667
CSRNP1	2.38839	2.61398	2.08796	2.52418	3.15348	3.00065
CSRNP2	3.89056	3.91869	4.33118	4.52688	4.00857	3.91716
CSRNP3	1.55835	1.87703	-5.1783	-4.52056	1.71041	2.29893
CSRP1	6.25587	6.72785	5.7256	6.29496	7.6104	7.69948
CSRP2	4.93571	4.74293	2.50649	2.5059	3.58524	3.23287
CSRP2BP	4.03447	3.93274	3.79362	3.92464	3.35849	3.56125
CST3	4.76032	4.78448	4.65568	5.10307	3.52828	3.90799
CSTB	3.13381	3.00068	3.69606	4.20745	3.40981	3.82076
CSTF1	5.12587	5.0821	4.66195	4.95659	5.31481	5.1782
CSTF2	4.36837	4.5421	3.16742	3.69529	4.31697	4.4519
CSTF2T	4.74023	4.89085	3.81366	4.16729	4.10869	4.08932
CSTF3	4.66668	4.6024	3.75458	3.96297	4.82749	4.75943
CTA-243E7.1	-1.37679	-1.60444	2.2131	1.69856	-0.00196773	-0.619042
CTA-276F8.1	0.676033	0.570657	1.69915	1.36695	-0.168309	-1.18284
CTA-276O3.4	0.983116	1.21132	-0.846877	-0.247406	0.970139	1.9049
CTA-29F11.1	1.54889	1.49139	1.68687	1.86848	0.782702	0.816919
CTA-363E19.2	-0.255978	0.223769	0.460428	0.367002	-0.136604	-0.0341529
CTA-384D8.36	0.639519	0.544901	0.0641517	0.414688	-0.396793	-0.971946
CTA-390C10.10	0.595618	0.71714	0.996447	1.23107	0.976371	0.560157
CTA-445C9.14	1.91933	2.12589	1.8772	2.12001	0.839605	1.08775
CTA-941F9.10	3.76038	3.68499	3.88377	3.66816	2.59534	2.11501
CTAGE5	3.22435	3.46426	2.54496	2.81713	3.04485	3.59877
CTB-113P19.1	-0.28777	-0.312083	0.191765	0.249057	0.741782	0.15182
CTB-119C2.1	1.06253	1.43806	0.386425	1.27492	0.437814	-0.38461
CTB-131B5.5	0.377835	-0.58	1.5913	0.563685	-0.393347	-3.38329
CTB-131K11.1	2.11472	2.45006	2.03082	2.29633	2.06081	2.44812
CTB-13F3.1	1.9402	2.05827	3.32189	2.46762	0.0259785	-0.865416
CTB-152G17.6	2.10829	2.1445	2.33613	2.12481	2.0332	1.65964
CTB-193M12.5	1.69756	1.68694	1.93025	2.08102	1.73203	1.55071
CTB-25B13.12	1.16865	0.912037	1.0573	0.892243	1.75161	1.13183
CTB-31O20.2	0.701877	0.71714	-0.183372	0.46112	1.62974	1.55071
CTB-33G10.1	1.35265	1.41051	0.566172	0.883644	1.85928	2.57216
CTB-47B8.1	0.453639	0.239789	-0.133342	0.28359	0.32848	-0.0341529
CTB-47B8.5	0.224213	0.445419	-1.0649	-0.598199	1.21673	0.351527
CTB-50E14.5	1.32195	1.50966	2.15849	2.43334	2.11372	2.10802
CTB-50L17.8	-0.255098	0.0425743	0.276866	0.24715	0.389181	-1.06148
CTB-52I2.4	3.56552	3.34474	2.8403	3.14748	3.31078	2.86413
CTB-55O6.12	0.983094	0.390767	0.659322	0.331877	-0.0699046	-1.67297
CTB-58E17.1	1.10059	0.994428	0.505363	0.692909	0.0746287	0.468917
CTB-63M22.1	5.02827	4.91613	4.00737	4.2684	5.00723	5.68061
CTB-79E8.3	3.67103	3.60132	2.28002	2.4588	4.05262	4.17686
CTB-89H12.4	5.09988	5.19898	5.98738	6.00609	6.15473	5.56741
CTBP1	4.6712	4.51532	4.66937	4.70471	5.58342	5.48136
CTBP1-AS	0.335049	-0.00931374	0.522658	0.325893	0.708064	-0.127297
CTBP1-AS2	2.62666	2.38308	2.46075	2.24787	2.83525	2.41742
CTBP2	5.31478	5.22327	5.34163	5.44605	5.53746	5.24853
CTBP2P4	-0.276553	0.133734	-0.286035	-0.0485211	0.202223	0.234943
CTBP2P8	1.27837	1.22355	-0.669052	0.325985	0.651353	0.587423
CTBS	3.08169	3.19509	4.51045	4.54274	3.30151	3.50653
CTC-205M6.5	0.399051	0.278722	1.12941	1.13916	-0.682812	-1.4584
CTC-231O11.1	5.67105	5.74263	2.20451	3.23103	-6.64581	-4.70296

CTC-260E6.2	3.37619	3.20568	2.69016	3.02345	4.34273	4.48259
CTC-260E6.4	0.93349	0.829492	0.723899	0.423331 ?		-5.02576
CTC-260E6.6	0.480917	0.237698	0.558032	0.476226	-3.65763 ?	
CTC-277H1.7	0.278306	0.137189	0.517004	0.445854	-0.753191	-1.70626
CTC-308K20.3	3.11634	3.31078	2.38029	3.11805	3.50519	4.15794
CTC-329H14.1	1.86397	2.01041	1.81651	2.36376	2.89724	2.58692
CTC-338M12.4	1.09729	1.58872	1.85368	2.30004	1.22423	1.64158
CTC-338M12.9	1.02208	1.06859	-6.17309	-6.09892 ?	?	
CTC-343N3.1	2.44048	2.64569	2.39392	2.18121	1.20393	0.315752
CTC-351M12.1	0.658554	0.955067	1.75315	1.32585	0.815493	0.668401
CTC-359D24.3	1.31071	1.05335	0.846553	0.537038	1.45128	0.429375
CTC-360J11.4	1.36705	1.40138	0.517004	0.892865	1.23456	0.668401
CTC-425O23.5	1.13386	1.31199	1.6494	1.83957	1.83157	0.80115
CTC-428H11.2	0.247745	0.570958	1.32385	1.38042	1.07005	0.749637
CTC-429P9.3	1.03569	0.859151	1.02599	0.89124	1.72774	1.03481
CTC-432M15.3	-1.69717	0.613626	-1.42715	0.22976	-0.673145	1.18134
CTC-435M10.3	1.93269	1.94662	2.40604	2.47928	2.8966	2.21173
CTC-444N24.11	4.02373	4.16285	3.46753	3.99183	4.94747	4.74346
CTC-444N24.7	2.82562	2.97743	2.46121	2.9091	3.5862	2.80879
CTC-444N24.8	0.61388	0.344613	0.371169	-0.108685	1.06767	0.126299
CTC-459F4.1	0.440559	0.996642	2.02107	2.06112	2.2496	1.60621
CTC-459F4.3	2.04322	2.17184	2.44538	2.72051	2.3739	2.70032
CTC-471F3.5	1.06253	0.894767	1.46757	1.12963	1.58943	0.224139
CTC-479C5.10	0.33867	0.343237	0.903069	0.745599	1.38672	0.812736
CTC-479C5.12	1.47055	1.21716	2.61569	2.68263	2.4865	2.39231
CTC-487M23.6	0.00148521	0.265185	0.386425	0.430424	-0.10558	-0.247123
CTC-487M23.8	1.12523	0.99974	0.0369885	0.108023	0.887611	0.657814
CTC-512J14.5	2.13945	2.01929	1.83938	2.40081	1.8548	2.21654
CTC-524C5.2	1.64535	1.74465	1.8877	1.98714	2.54913	1.89194
CTC-529P8.1	3.1417	4.14356	-0.628724	0.591699	-4.07293	-2.70579
CTC-534A2.2	1.20524	1.21485	0.783733	1.3614	1.72743	2.31955
CTC-534B23.1	0.151794	0.307265	-0.366833	-0.869325	0.523983	-0.154952
CTC-575D19.1	4.82614	4.74321	4.25938	4.75791	5.39468	5.86553
CTC1	3.22573	3.47232	2.693	3.10063	3.08621	2.85171
CTCF	5.18472	5.17922	4.65016	4.75535	5.37207	5.28125
CTD-2006C1.2	1.74604	1.50874	1.34214	1.39814	1.47209	1.23778
CTD-2006C1.6	0.702409	0.429675	0.874577	0.79511	-0.139374	-0.096138
CTD-2010I16.1	-0.0842313	0.318612	0.431281	0.317536	-0.356358	-1.006
CTD-2012J19.1	1.07725	1.13872	0.199462	0.161436	0.581997	1.02944
CTD-2013N24.2	1.52282	1.71436	1.71058	1.69239	1.52877	0.91731
CTD-2014D20.1	0.529821	0.137189	0.662041	0.605504	0.210164	-0.706491
CTD-2015B23.2	0.874836	1.26066	1.003	1.43313	2.15475	2.38679
CTD-2015H6.3	0.520176	0.444137	0.571444	0.605504	1.43776	1.37014
CTD-2017D11.1	1.2814	1.08788	2.52203	2.456	1.42898	0.942256
CTD-2017D11.2	0.266817	0.305445	1.5714	1.31748	1.22238	0.597145
CTD-2023N9.1	3.32744	3.67542	-3.85951 ?		-6.64581 ?	
CTD-2024I7.13	0.577131	0.697098	1.27595	0.968047	-0.637731	-0.0918635
CTD-2026D20.3	1.45396	1.51552	0.316818	0.317964	0.437888	0.0610158
CTD-2026K11.6	1.45578	1.5651	-1.59814	-1.52346	-3.85089	-5.69922
CTD-2033A16.2	4.62874	4.42969	4.44858	4.39062	5.10739	4.55618
CTD-2033D15.2	0.658554	0.988427	1.89865	1.90242	1.1211	0.315752
CTD-2033D15.3	-0.0582596	0.00502624	1.81471	1.72786	1.02244	-0.203638

CTD-2033D24.2	0.292216	0.353673	-0.370051	0.322344	0.234489	0.425867
CTD-2047H16.4	1.83531	1.68825	2.32497	2.0658	1.63344	0.99917
CTD-2083E4.4	0.494733	0.533583	0.221172	0.541654	-0.143645	-1.26908
CTD-2083E4.5	1.79134	2.02879	1.4101	1.78533	1.49998	1.94616
CTD-2090I13.2	1.02245	1.07681	0.893354	0.81069	1.46415	0.785207
CTD-2114J12.1	-2.65634	-2.06994	1.85562	1.99613	-0.664894	-0.475455
CTD-2116N17.1	0.733554	0.936078	1.12517	0.992667	1.02909	0.0136409
CTD-2135D7.2	0.119396	0.160301	0.433376	0.846489	-0.614682	-0.515272
CTD-2161E19.1	2.72312	2.75318	1.85303	2.31478	2.99705	3.43229
CTD-2179L22.1	-0.113985	-0.513162	1.17408	0.833756	-0.168804	-1.19468
CTD-2192J16.15	6.20767	6.0705	5.60073	6.04933	6.66546	7.33981
CTD-2192J16.20	1.61568	1.36038	1.23143	1.04063	2.22884	1.4447
CTD-2192J16.22	3.27275	3.19507	2.81067	3.23273	4.10616	3.94145
CTD-2201E18.3	0.887556	0.868134	1.42128	1.48244	0.278187	0.541297
CTD-2206G10.2	0.314151	0.459988	-0.510905	0.00201836	0.0277769	-0.0341529
CTD-2207A17.1	1.51528	1.62465	-6.17309 ?		-6.64581 ?	
CTD-2207O23.3	0.922388	0.864596	-0.01909	0.303775	1.38578	1.26279
CTD-2228K2.7	1.82253	1.44204	3.17321	2.80934	1.10064	0.0456666
CTD-2231E14.8	2.85453	2.81022	2.58907	3.0461	3.79417	3.81309
CTD-2233K9.1	1.52496	1.37009	2.0549	1.70499	0.886652	0.359476
CTD-2256P15.4	1.58835	1.63714	1.64639	1.88346	1.86558	2.23095
CTD-2260A17.1	-0.707105	-0.523255	1.48065	1.16027	0.921886	-0.303605
CTD-2270N23.1	4.27199	3.9975	3.45977	3.92092	4.37136	4.86694
CTD-2270P14.1	3.39306	3.14697	4.48812	4.46667	3.57483	3.13872
CTD-2270P14.5	0.973406	0.868134	0.82777	0.913608	-0.551587	-0.38461
CTD-2287O16.1	5.85673	5.79658	5.24951	5.64527	5.86255	7.1484
CTD-2303H24.2	-3.8896	-3.85615	3.17251	3.17482	1.08887	0.146942
CTD-2310F14.1	0.624279	0.265185	1.06807	0.61053	0.270529	0.102712
CTD-2336O2.1	0.586404	0.223769	0.904002	1.195	0.894341	1.38079
CTD-2336O2.3	-0.550231	-0.214764	1.44008	1.52878	-0.377667	0.230781
CTD-2349P21.3	1.40425	1.05291	2.33613	1.8912	1.45867	0.200291
CTD-2353F22.2	-1.38907	-0.884905	0.971045	0.605504	1.58335	0.752791
CTD-2366F13.1	1.20786	1.09945	1.35568	1.40691	0.549226	0.80115
CTD-2368P22.1	1.57972	1.66211	0.541921	0.491176	1.23337	-0.151977
CTD-2369P2.12	-0.0296396	1.02197	0.471292	0.622321	1.65364	1.29126
CTD-2369P2.8	2.13333	2.19798	-4.01161	-3.85519	-3.56111 ?	
CTD-2371O3.3	1.26655	1.54812	0.782333	1.15801	0.121175	0.270672
CTD-2373H9.3	0.0289651	0.237698	0.711372	0.659431	0.172692	-0.348991
CTD-2373N4.3	1.58636	1.03706	1.30837	0.902467	-2.07475	-2.38405
CTD-2410N18.4	0.344033	0.320519	0.638445	0.740427	0.968998	0.444063
CTD-2510F5.4	0.70496	0.295899	0.259532	0.80192	0.807363	1.15132
CTD-2517M22.14	0.739027	0.207393	1.52388	0.884801	0.915016	0.288689
CTD-2528L19.3	0.812211	1.21259	-0.310912	-0.347836	0.392691	0.921421
CTD-2541J13.2	2.46567	1.96395	2.08712	1.21348	2.65042	3.55379
CTD-2545G14.6	0.180224	0.0337507	0.706093	0.656661	1.54484	1.73892
CTD-2545G14.7	2.16173	1.68506	2.41439	2.93968	3.58037	4.22783
CTD-2545M3.2	0.0141839	0.290188	-3.40079	-2.9374	0.932409	1.11499
CTD-2547G23.4	1.18971	1.24456	1.42385	1.25754	0.384182	-0.214706
CTD-2553L13.9	0.480917	0.305445	0.770744	0.46112	-0.320293	-0.536593
CTD-2554C21.1	2.31795	2.44244	2.76139	2.98211	3.51667	3.41236
CTD-2555O16.4	1.2969	1.21107	0.700324	0.558023	0.976136	0.939272
CTD-2562J17.6	-1.02638	-1.29258	4.02104	3.74292	-5.07051 ?	

CTD-2574D22.3	1.36705	1.38894	0.893354	0.978694	0.437814	-0.4586
CTD-2574D22.4	1.61117	1.71274	0.889949	1.35709	0.586059	-0.0726769
CTD-2576F9.1	1.18389	1.02489	0.642166	0.789245	1.47213	1.35747
CTD-2583A14.11	0.564778	0.435437	0.137477	0.571395	0.995817	0.270134
CTD-2587H24.14	0.0664543	-0.252819	0.55155	0.460397	-0.0525657	-0.655794
CTD-2587M2.1	0.61388	0.479758	0.956086	0.491176	1.37315	0.359476
CTD-2600H12.2	0.701877	0.456112	1.48894	1.32585	1.67957	0.483199
CTD-2619J13.14	1.63678	1.54694	1.74815	1.59004	1.65312	1.02411
CTD-2619J13.17	-0.395122	-0.440295	0.849967	0.786061	1.2101	1.44307
CTD-2619J13.8	0.179269	0.0488918	0.843325	0.864541	-0.490053	-1.62075
CTD-2647E9.1	-0.215699	0.0526524	-0.280349	0.402449	0.726404	0.713379
CTD-2651C21.3	0.41996	0.371157	-0.227336	0.367002	1.07446	1.3814
CTD-3014M21.1	3.37238	3.2219	1.81086	1.76157	2.06422	2.58332
CTD-3035D6.1	2.86103	3.05038	2.21977	2.76123	4.11795	4.58582
CTD-3035K23.3	0.733111	0.144777	1.68475	1.40065	0.770119	0.659988
CTD-3074O7.5	0.265977	0.377213	0.307137	0.413128	-0.406989	-0.528471
CTD-3092A11.2	0.743937	0.938098	2.05967	2.32582	1.10129	-0.536593
CTD-3099C6.9	3.55713	3.6019	3.34396	3.22657	2.78885	2.27058
CTD-3113P16.11	0.662926	0.666811	0.568052	0.944169	1.02014	1.06999
CTD-3131K8.2	0.504651	0.527341	0.139417	0.537902	0.698405	-0.457255
CTD-3138B18.5	1.94278	1.9954	1.877	1.73268	2.83049	2.13733
CTD-3184A7.4	0.855575	0.804012	0.445928	0.520618	-0.0163223	0.578775
CTD-3185P2.1	0.798897	0.919065	0.392768	1.09628	0.987888	0.405154
CTD-3193O13.12	1.53084	1.3164	0.672724	0.713177	-0.368728	-0.198091
CTD-3222D19.8	0.587682	0.536033	0.441005	0.31919	0.944971	0.540672
CTD-3252C9.4	2.69753	3.037	1.89865	2.42257	2.88852	2.36476
CTD-3253I12.1	1.26097	1.554	-0.482896	-0.751045	-3.19956	-2.70579
CTDNEP1	3.41738	3.24463	3.746	3.65871	4.11801	4.17613
CTDP1	3.2287	3.26507	2.24275	2.82487	1.99792	1.6062
CTDSP1	4.06959	4.00578	4.27283	4.54132	4.08031	4.25618
CTDSP2	5.05037	4.82482	5.65486	5.79202	4.79962	4.37941
CTDSPL	5.33017	5.37516	3.72786	4.05858	4.32409	4.26051
CTDSPL2	5.44158	5.26949	4.98692	4.89042	5.45035	5.53606
CTGF	5.43469	5.33683	4.36914	4.61823	7.246	7.05649
CTH	0.409538	0.7564	1.19157	2.04094	0.723471	1.08775
CTHRC1	6.03791	6.40922	-0.428455	-0.086319	0.36215	0.315752
CTIF	3.79677	3.52868	3.74277	3.8584	1.85147	1.71117
CTNNA1	8.5399	8.39745	7.40377	7.81272	7.18551	7.09502
CTNNAL1	5.27138	5.24016	4.60519	5.452	5.2031	5.69577
CTNNAP1	2.63765	2.86288	1.60167	2.12072	1.61884	1.28064
CTNNB1	8.24789	8.12222	7.51015	7.65765	8.59091	8.68295
CTNNBIP1	3.76955	3.67286	2.68993	2.68234	1.55404	1.22407
CTNNBL1	4.77107	4.76224	4.41079	4.70491	4.45014	4.41482
CTNND1	6.02031	6.01267	6.13994	6.26869	6.10016	6.00494
CTNND2	-5.10914	-6.45019 ?		-6.09892	4.18596	3.0178
CTNS	3.23313	2.82025	3.01124	2.99447	3.39632	2.92265
CTPS1	5.54111	5.51084	5.43623	5.73744	5.66697	5.48804
CTPS2	2.49076	2.42591	2.82492	2.63265	2.56611	2.56008
CTR9	5.13516	5.21197	4.92909	5.25764	5.04459	5.18667
CTSA	6.86404	6.76832	6.11577	6.43792	5.72593	5.68735
CTSB	6.38024	6.39047	7.40942	7.80332	7.1088	7.24948
CTSC	6.93624	6.58466	4.26252	4.38001	6.57824	6.84403

CTSD	5.72428	5.6161	7.92761	8.35077	7.65114	8.001
CTSF	4.83522	4.6378	4.26871	4.31428	1.57813	1.42261
CTSH	0.0559314	-0.0703908	2.18718	2.31745	0.969108	1.08775
CTSK	3.28961	3.12587	0.292311	0.761609	0.969108	0.270672
CTSL	5.04576	5.15185	4.676	5.40145	4.9822	5.65138
CTSO	1.12752	1.33161	3.84852	4.00881	1.29393	1.80902
CTSS	5.08799	5.68626	5.22745	6.10882	-0.0163223	1.12623
CTSV	2.22575	1.93806	0.0645242	-0.493928	-1.80184	-1.18284
CTSZ	-2.9975	-2.87684	4.00754	4.29311	3.61232	3.63536
CTTN	7.34386	7.28868	6.66451	6.94832	6.84974	6.70424
CTTNBP2	-6.68418	-4.87366	3.67445	2.99709	?	?
CTTNBP2NL	4.71295	4.81791	4.16962	4.62249	5.34107	5.43219
CTU2	1.96608	1.77066	1.67688	2.29542	1.86225	2.27629
CUBN	2.89711	2.1408	-1.42826	-0.616766	2.92653	2.52212
CUEDC1	4.60983	4.49932	4.47736	4.57064	5.75014	5.65028
CUEDC2	2.94838	2.80395	3.6109	3.81301	3.39497	3.75066
CUL1	5.98106	5.92697	5.30259	5.6306	5.86215	5.92424
CUL1P1	0.0438764	0.468969	-0.158539	0.140255	0.186196	-0.0621585
CUL2	4.59726	4.65738	4.23538	4.68151	4.5424	4.86219
CUL3	5.43974	5.34339	5.67381	5.74544	6.03004	6.01379
CUL4A	5.17118	5.27607	5.14766	5.61199	5.56172	5.64545
CUL4B	4.33847	4.26847	5.51212	5.92906	6.78431	7.18085
CUL5	3.2191	3.34075	5.06904	5.21781	5.01275	5.39185
CUL7	3.8782	3.74313	4.13375	4.03747	4.08278	4.26947
CUL9	3.94638	3.90667	4.0124	4.09818	4.65483	4.51487
CUTA	4.99658	4.71082	4.87986	5.15443	5.84995	5.89968
CUTC	2.38586	2.32564	2.74144	2.96813	3.24388	3.16365
CUX1	5.67188	4.98681	4.87175	4.53322	4.5745	3.971
CWC15	3.31354	3.47527	4.0644	4.27811	4.24731	4.64753
CWC22	4.30652	4.37082	4.53501	4.65935	4.72877	5.19114
CWC25	3.83424	3.46493	3.80799	3.84391	4.07946	3.30172
CWC27	3.49814	3.48552	3.9713	4.13077	4.17417	4.31427
CWF19L1	3.9795	4.02124	3.53168	3.92039	3.8098	4.34138
CWF19L2	2.14329	2.25142	3.54099	3.70817	3.62626	4.02968
CX3CL1	1.399	1.74175	0.935482	1.17662	2.41652	2.54596
CXADR	2.39325	2.47954	2.60036	3.00013	4.45918	4.39397
CXCL1	6.94187	6.9476	-3.85951	-4.10635	-2.33767	-2.00571
CXCL12	?	-3.65369	6.0103	6.14826	-6.64581	-5.69922
CXCL16	0.0559314	0.548466	0.340141	1.02051	0.587701	0.100762
CXCL3	2.40422	2.79691	0.610974	2.17196	-0.510371	0.200291
CXCL8	8.45248	8.49613	3.59126	5.45769	1.22238	3.71113
CXCR6	1.43539	1.15192	1.46757	1.14862	0.740643	0.200291
CXXC1	4.39895	4.23412	2.85814	3.21985	2.67285	2.69417
CXXC5	2.98743	2.58429	4.15186	4.58378	5.06803	5.15979
CXorf23	1.91933	1.81331	2.85814	2.84828	1.90577	2.18205
CXorf38	3.53694	3.69703	4.20125	4.56976	3.3241	3.27057
CXorf40A	1.99325	1.89917	2.02365	2.34682	2.96451	3.39237
CXorf40B	2.93936	2.84826	2.86436	3.22686	3.57503	4.03525
CXorf56	3.67293	3.61591	3.51542	4.05362	4.30108	4.52122
CXorf57	2.72299	2.69451	-3.85951	-3.78492	-0.959604	-1.31405
CYB561	4.34532	4.1491	5.05068	5.28241	5.38202	5.49418
CYB561A3	3.84973	3.9719	4.38418	4.83381	3.91565	4.35389

CYB561D1	3.079	3.02299	2.56468	2.80706	3.3909	3.23287
CYB561D2	3.15387	3.07843	2.35891	2.83899	3.44802	3.89698
CYB5A	3.55122	3.56783	4.50384	4.88524	2.08454	2.20019
CYB5B	5.80695	5.71829	6.48889	6.68193	6.72203	6.75219
CYB5D1	3.18405	3.01956	3.32861	3.46621	3.74791	3.41391
CYB5D2	2.06581	1.64571	3.21736	3.30691	2.84749	2.76497
CYB5R1	3.8622	4.02599	4.56718	4.96666	4.29825	4.54596
CYB5R2	5.35245	5.63188	-3.59683	-2.64851	-0.302594	0.224139
CYB5R3	4.12029	4.13799	3.91976	4.28603	3.47798	3.54122
CYB5R4	4.17897	4.16468	3.34591	3.64439	4.69172	5.33629
CYB5RL	2.28026	2.10543	2.605	2.79832	2.57025	2.48079
CYBRD1	5.49259	5.34652	6.11973	6.56692	5.87231	6.66181
CYC1	4.87741	4.95077	4.93734	5.52689	5.53904	6.01694
CYCS	6.45306	6.62713	6.11187	6.87523	6.82297	7.14526
CYFIP1	6.03904	6.04829	5.70408	5.90249	6.20995	6.06848
CYFIP2	3.24116	3.49715	-3.01245	-2.10822	2.48945	2.42773
CYHR1	3.24324	3.00476	4.00872	4.03164	3.72654	3.6856
CYLD	4.5225	4.58018	5.36046	5.72129	5.22614	5.40767
CYP19A1	1.05586	1.35738	-0.935332	0.120113	-1.45047	-0.95156
CYP1A1	-0.306598	-0.58	1.08332	1.46107	?	?
CYP1B1	3.07817	3.4947	3.69795	4.14096	0.477947	0.688261
CYP1B1-AS1	2.36587	2.26563	-0.274798	-0.117232	-2.70408	-2.43731
CYP20A1	3.34395	3.33805	2.87176	3.18809	3.90097	4.02968
CYP26B1	2.55832	1.67161	0.82777	1.2041	-3.6585	-2.8983
CYP27A1	-0.590352	-0.629746	0.53081	1.04098	4.0803	3.95327
CYP27C1	0.768595	0.877069	1.37876	1.02051	-0.729347	-0.848487
CYP2J2	2.61835	3.27525	-3.59683	-2.20128	-4.65668	-2.70579
CYP2R1	2.76853	2.61665	2.49948	2.32582	-0.0752099	-0.0918635
CYP2S1	-4.69535	-4.45968	3.07151	3.66431	?	-5.69922
CYP2U1	3.35447	3.7021	2.88543	3.5178	2.20065	3.10054
CYP39A1	-2.17488	-1.60444	2.35952	3.07357	-6.64581	?
CYP3A5	0.908021	1.15925	1.15638	1.39082	0.878914	0.315752
CYP4F11	?	-4.87366	4.60193	4.92322	?	-5.69922
CYP4V2	2.44039	2.88146	3.74573	4.16147	1.83933	1.81678
CYP51A1	7.21746	7.31448	4.95833	5.25443	6.253	6.59164
CYP51A1P1	3.45435	3.57818	1.33909	1.61553	2.51567	2.75022
CYP51A1P2	4.41212	4.49035	2.11783	2.33268	3.28859	3.82494
CYP7B1	3.03733	2.57334	-3.85951	-5.10387	?	?
CYR61	4.74954	5.04397	6.76239	7.12808	6.44361	6.30279
CYSTM1	3.00829	3.01694	1.54443	1.81006	3.35789	3.91167
CYTH1	4.34257	4.09652	4.05488	3.97465	4.45213	3.63091
CYTH2	4.09126	4.06462	4.67444	4.73979	5.07269	4.7424
CYTH3	4.15965	4.32991	3.96369	4.25201	5.56554	5.09984
CYTL1	2.49322	2.85005	2.31634	2.96532	?	?
D2HGDH	2.01519	1.75633	2.50299	2.32357	1.56789	1.59704
DAAM1	3.78272	3.70331	3.1474	3.13197	3.64004	3.42771
DAAM2	4.33097	3.98566	2.58603	2.93384	-2.47153	-3.01119
DAB1	1.36705	0.736899	-6.14047	-6.09892	?	?
DAB2	3.13064	3.15921	4.31232	4.66596	3.88078	4.14347
DAB2IP	2.92846	2.92517	2.20881	2.30058	2.93215	2.91534
DACH1	-0.891472	-0.81875	?	-6.09892	3.74375	3.65523
DACT1	2.98567	2.69703	-3.85951	-3.10759	3.54304	2.79712

DAD1	4.92206	4.94282	4.59209	4.87558	4.97767	5.69361
DAG1	8.02704	8.09093	6.41311	6.50358	7.45489	6.92376
DAGLA	1.84784	1.64571	-0.628724	-0.301309	1.78489	1.65964
DAGLB	3.5225	3.4738	3.87311	4.09084	3.4125	3.06478
DALRD3	2.54594	2.73823	2.2442	2.63384	3.62769	3.99462
DANCR	4.49797	4.2304	2.22307	2.1661	5.39362	5.31605
DANT2	0.133932	0.265185	2.01617	2.04601	1.19149	1.37014
DAP	5.04069	4.90073	4.93281	5.20858	5.00812	5.36742
DAP3	5.86898	5.97692	5.21742	5.73929	5.58672	5.96987
DAP3P2	0.0268425	0.692998	-0.226966	0.495245	0.122381	-0.166598
DAPK1	-0.840854	-0.907998	0.242852	1.61913	-0.152369	-2.53597
DAPK2	0.377835	0.305445	1.68687	1.01017	3.4298	3.23873
DAPK3	3.30935	3.05088	2.541	2.98657	4.44821	4.39526
DARS	4.92408	4.88073	4.34075	4.64309	5.93629	6.16834
DARS-AS1	-0.262961	-0.434432	0.143184	0.0916931	1.25305	0.64667
DARS2	5.86789	5.92165	4.47556	4.70174	4.56368	4.79512
DAXX	4.98566	4.96185	4.15298	4.49201	5.46283	5.35059
DAZAP1	5.25	5.131	4.59865	4.68233	5.52735	5.40702
DAZAP2	4.64358	5.68101	4.31597	5.53191	4.31338	5.89175
DBF4	4.87577	4.98285	4.94958	5.37168	4.6786	5.17014
DBF4B	3.12906	3.35094	3.48536	3.80107	3.12596	2.63315
DBF4P1	2.66963	2.92388	2.77618	3.21796	2.62935	3.30828
DBI	6.12666	6.10313	5.6129	5.98756	5.85217	6.75685
DBIP1	1.15895	1.37122	0.723445	1.24877	0.89493	1.43288
DBN1	4.54231	4.10035	5.67872	5.66554	4.96586	4.82467
DBNDD1	4.75623	4.54418	1.31637	1.38307	3.79398	3.3073
DBNDD2	2.59661	2.61053	1.10815	1.20709	2.38496	2.60894
DBNL	5.89574	5.76639	6.30757	6.54146	6.14044	6.24162
DBP	1.3139	1.0965	2.62289	2.32275	2.26924	2.13915
DBR1	2.91011	2.9187	2.47827	2.9052	2.78471	3.10386
DBT	3.88023	3.80978	4.10072	4.2867	4.46121	4.5197
DCAF10	3.94747	3.93802	4.12253	4.23103	5.22309	5.08932
DCAF11	3.88494	3.72569	3.1828	3.52561	4.69211	4.84686
DCAF12	4.24269	4.41213	3.7113	4.13672	5.5882	6.08403
DCAF13	5.43751	5.61454	4.91201	5.51124	6.12688	7.01025
DCAF13P1	0.777275	1.10354	0.310852	1.03078	1.49151	2.26987
DCAF13P3	1.17857	1.23879	0.713801	1.05187	1.83211	2.13154
DCAF15	2.04481	1.77961	1.85652	2.10605	2.38098	2.14582
DCAF16	5.12507	5.04197	4.472	4.61397	5.53474	5.08851
DCAF17	3.70393	3.79219	3.32823	3.45341	3.554	3.73416
DCAF4	3.1417	2.69956	1.15638	1.03078	1.09463	0.33778
DCAF4L1	-0.129081	-0.105152	-1.59814	-1.46458	1.13416	0.401903
DCAF5	4.24562	4.05678	4.44856	4.41767	4.58166	4.16673
DCAF6	5.04826	5.06632	4.76705	5.03693	5.31245	5.57561
DCAF7	6.08738	6.031	5.99948	6.16496	6.92215	6.85099
DCAF8	5.59046	5.53934	5.50804	5.71959	6.03843	6.07004
DCAKD	2.25222	1.9035	3.31433	3.44385	3.7169	3.26195
DCBLD1	4.24036	4.10851	3.59852	3.87844	5.04153	5.03749
DCBLD2	8.0126	8.2269	7.8233	8.21034	10.1726	10.4402
DCC	2.52975	2.68182	-5.1783	-4.10635	-6.64581	-4.11926
DCHS1	3.14798	2.68437	1.54444	0.476226	-5.07051	-4.70296
DCHS2	1.72817	1.85096	-1.23379	-0.9805	-2.85191	-2.64707

DCK	3.64855	3.93804	3.12481	3.49851	4.26376	4.76496
DCLK1	-1.05494	-1.65505	-1.48269	-1.35358	4.44493	4.83437
DCLK2	0.520176	0.955067	2.60439	2.99184	1.99435	1.493
DCLRE1A	3.98391	4.03799	3.5697	3.80706	4.03578	4.55537
DCLRE1B	3.62734	3.72821	3.16742	3.52961	3.29244	3.17593
DCLRE1C	3.15009	3.2835	3.20768	3.27592	2.8752	2.83514
DCP1A	4.74333	4.63517	4.11797	4.32683	4.83254	4.61633
DCP1B	2.02204	2.13709	2.42751	2.62928	1.5246	1.66836
DCP2	6.71442	6.60188	6.64893	6.70074	6.88901	6.88405
DCPS	3.81958	3.92302	2.27182	2.64273	3.30259	3.38869
DCTD	4.52252	4.59174	4.1065	4.70655	4.61986	4.76088
DCTN1	6.37943	6.37569	6.25381	6.4491	6.20399	6.1612
DCTN2	4.23387	4.2574	4.938	5.40489	4.74375	5.05067
DCTN3	3.15579	3.16469	3.3302	3.69207	4.6588	5.06146
DCTN4	4.92981	4.88088	5.17182	5.44719	5.7424	5.72636
DCTN5	5.80249	5.95312	5.702	5.99113	5.97924	6.44934
DCTN6	3.21326	3.39254	2.96311	3.55058	3.4953	3.741
DCTPP1	3.66926	3.53096	3.00759	3.46956	4.06029	4.53901
DCUN1D1	4.98244	5.18447	5.12749	5.28876	5.81625	6.12803
DCUN1D2	2.74801	2.71243	3.29625	3.39897	2.97813	2.45317
DCUN1D3	3.35092	3.56675	3.75523	4.04219	3.3448	3.48096
DCUN1D4	4.89383	4.81501	4.95153	4.85478	5.51712	5.42256
DCUN1D5	2.2198	2.12589	3.84439	4.2097	3.85341	4.29328
DCXR	3.7345	3.64438	2.73236	3.09085	4.79706	5.32118
DDA1	3.59209	3.48669	2.87953	3.28778	4.33243	4.27169
DDAH1	4.2076	4.40894	3.4984	4.21128	6.03024	5.74639
DDAH2	1.70609	1.52019	2.13391	2.21312	2.77641	3.05484
DDB1	7.4171	7.47593	7.06145	7.55745	6.93282	7.0096
DDB2	5.86339	5.84177	2.98139	3.12	3.01035	2.56476
DDHD1	4.71243	4.62594	4.56633	4.47048	4.97402	4.60843
DDHD2	4.64204	4.92368	4.33062	4.67498	4.2593	4.36206
DDI1	-4.37417	-3.70359	2.28816	1.53506	-1.53468	-0.752294
DDI2	5.03313	5.2485	4.89131	5.14714	5.17717	5.09004
DDIAS	4.27962	4.32669	3.92368	4.23324	4.34951	4.56007
DDIT3	2.15442	1.96494	3.11564	4.00711	3.43395	3.62309
DDIT4	5.92575	5.5228	4.60517	4.89985	6.64462	5.68272
DDO	-4.37417	-3.29152	0.747284	0.810106	0.757612	0.863209
DDOST	7.4385	7.31122	6.85904	7.16794	7.49649	7.57179
DDR1	5.02926	5.09221	4.89261	5.07294	6.38183	6.53408
DDR2	6.5499	6.46604	6.48865	6.79127	7.10424	7.22897
DDRGK1	3.76954	3.57882	3.91844	4.17194	3.55643	3.028
DDT	3.77358	3.88312	3.22927	3.62601	3.39939	4.08181
DDTL	2.33859	2.30694	1.74364	1.97294	1.93867	1.55651
DDX1	6.05208	6.23908	4.69299	5.29151	5.59444	6.17475
DDX10	3.31764	3.27375	4.58576	4.85763	4.38612	4.19402
DDX11	5.16373	5.02739	4.91292	4.99304	4.37613	3.6776
DDX12P	2.32505	2.02756	1.72545	1.80257	0.512419	-0.120006
DDX17	8.34974	8.30892	8.55021	8.46283	8.00242	7.68626
DDX18	6.45666	6.56181	5.26396	5.67212	6.73038	7.17748
DDX18P1	0.4654	0.497117	0.503064	0.317536	0.243438	0.384061
DDX18P5	1.25482	1.23902	0.308431	0.31299	1.27182	1.30919
DDX19A	5.10805	5.12439	4.7522	4.9815	6.39769	6.33148

DDX19B	2.88912	2.70543	2.96396	3.48135	4.72913	4.84162
DDX20	4.63226	4.7749	4.11562	4.4029	4.2851	4.37409
DDX21	7.76588	7.90377	6.04018	6.53752	8.29178	8.4361
DDX23	6.21142	6.19436	5.62944	5.95822	5.66497	5.31629
DDX24	6.63475	6.63756	5.49145	5.80587	6.5826	6.36412
DDX26B	1.82059	1.81795	2.68685	2.6225	3.24508	3.31288
DDX27	5.86388	5.86398	5.06883	5.36944	5.81774	5.56332
DDX28	2.31772	2.3874	2.30378	2.67824	2.6673	3.26156
DDX31	3.74714	3.82993	2.90218	3.21917	3.47328	3.78669
DDX39A	5.32743	5.32505	4.23327	4.60283	5.17574	5.32118
DDX39B	6.62623	6.44452	6.41878	6.5108	7.04718	6.66653
DDX3P1	0.0954519	0.060836	0.045429	0.605504	0.282309	0.100762
DDX3P3	0.710384	0.997054	-0.723865	-0.0422696	0.863447	-0.0627204
DDX3X	8.15064	8.25987	6.84677	7.30539	8.11642	8.19904
DDX3Y	?	?	2.79948	3.17658	-6.64581	-5.69922
DDX41	4.41466	4.26252	4.80728	5.1632	5.47763	5.05649
DDX42	6.12428	6.09269	6.2285	6.39249	6.84909	6.33665
DDX46	6.71828	6.65602	6.44603	6.52751	6.58241	6.30175
DDX47	5.51245	5.52082	4.94663	5.35233	5.18127	5.39456
DDX49	3.884	3.69576	2.81084	3.24433	4.75331	5.17286
DDX5	8.34595	8.40604	8.31822	8.61494	9.00842	8.98468
DDX50	4.53477	4.59017	3.86132	4.18376	4.82862	5.42609
DDX50P1	1.51846	1.5108	1.25491	1.19029	1.55322	1.49402
DDX51	3.42892	3.33158	2.88795	3.11999	3.39903	2.79712
DDX52	4.59967	4.796	4.93003	5.32453	5.46373	5.76893
DDX54	4.39009	4.3845	3.38294	4.01302	3.97521	4.3107
DDX55	4.95807	4.8111	3.59887	3.84268	4.30983	4.04578
DDX56	5.46708	5.56883	4.86496	5.37699	5.50554	5.60161
DDX58	2.7418	3.31689	3.41266	3.96264	5.30657	5.76847
DDX59	3.24344	3.13149	2.7113	3.11518	3.08789	3.45316
DDX6	6.57047	6.60624	6.87203	7.06526	6.67186	6.88894
DDX60	2.33436	2.50006	3.83884	4.01788	3.53568	3.5833
DDX60L	3.16356	3.3637	3.90791	3.87131	4.46637	4.62195
DDX6P2	0.400079	0.225028	0.773186	1.13126	0.439347	0.407048
DEAF1	3.73137	3.43349	3.44035	3.5864	3.42317	3.4582
DECR1	2.1713	2.11082	3.75605	3.93693	3.29679	4.14191
DECR2	1.64525	1.30538	1.67769	1.7241	0.976371	1.00759
DEDD	4.12661	4.16122	3.32887	3.66346	4.20966	4.15543
DEDD2	2.91195	2.74905	2.87718	3.17657	3.57571	3.40962
DEF8	4.7235	4.46933	3.48352	3.70815	3.86912	4.46446
DEGS1	5.49627	5.42476	5.89861	6.18694	6.08594	6.05941
DEK	6.64309	6.86259	5.98607	6.55784	7.35031	7.77204
DENND1A	3.23313	3.39669	2.60765	2.98393	2.61582	2.73209
DENND1B	4.80106	4.56871	2.92762	2.75227	3.78284	3.47649
DENND2A	-3.22992	-3.13966	1.08332	0.549472	3.19767	2.48805
DENND2C	0.760425	0.467978	-3.18222	-2.52305	0.578176	0.247587
DENND3	3.16201	3.24968	3.60764	3.82928	-0.374735	-0.662104
DENND4A	5.02627	4.9976	4.41731	4.59247	4.72177	4.90706
DENND4B	4.22424	4.01692	3.61576	3.67253	4.29678	3.84402
DENND4C	4.93527	4.97749	5.21574	5.29787	5.99967	6.15514
DENND5A	5.78573	5.53326	5.97664	6.28964	5.98647	5.62755
DENND5B	5.89476	5.58632	4.33513	4.44767	5.17375	5.082

DENND6A	4.27533	4.34047	3.4108	3.50404	4.48625	4.78012
DENR	4.96152	5.20519	4.08059	4.58552	5.27124	5.59018
DEPDC1	5.60806	5.84313	5.36151	5.9803	5.86514	6.31643
DEPDC1B	4.19876	4.15094	3.86086	3.70815	4.30474	4.70479
DEPDC5	3.21381	3.09749	3.17403	3.3258	2.96175	2.919
DEPDC7	6.87815	6.6291	-1.09576	-1.46458	1.64351	1.38079
DERA	3.26526	3.09366	3.50472	3.85262	2.85736	3.38869
DERL1	4.68183	4.74123	4.9952	5.40081	5.13459	5.37552
DERL2	4.6603	4.60011	4.97814	5.26385	5.11927	5.44591
DESI1	4.18279	4.34853	4.29322	4.93214	4.43112	4.60843
DESI2	5.42526	5.52232	5.22253	5.65024	5.72474	5.94997
DET1	1.53634	1.20466	1.92739	1.86942	1.42761	1.28202
DEXI	1.56024	1.68263	2.49625	2.75486	2.50052	2.2016
DFFA	5.15474	5.36958	4.9669	5.37654	6.08349	5.86343
DFFB	2.37239	1.94657	1.32432	1.37503	2.10126	1.56011
DFNA5	4.04154	4.08018	4.02715	4.52143	3.40039	4.06643
DFNB31	0.480917	0.432061	1.15638	0.857026	1.65261	1.12623
DGAT1	2.31273	2.39165	2.62896	3.04098	2.42019	2.79676
DGAT2	3.88537	3.86525	1.18285	1.65939	-6.64581	-4.70296
DGCR11	1.78879	1.60328	1.496	1.25754	0.606556	0.668401
DGCR14	3.55818	3.4152	2.53416	2.88836	1.99435	2.32666
DGCR2	5.35042	5.12865	5.53372	5.45151	4.73895	4.4607
DGCR6L	2.90411	2.82982	3.19882	3.38495	2.68294	3.83073
DGCR8	5.06993	5.0355	4.72935	4.7875	4.76437	4.31843
DGKA	3.22167	3.08791	2.81084	3.1224	1.34536	1.31651
DGKD	4.20704	4.18101	5.3018	5.48687	4.8603	4.53169
DGKE	2.18968	2.05287	0.966283	1.22214	0.32848	-0.0918635
DGKG	2.62961	2.67671	3.17183	3.53863	3.43942	3.22107
DGKH	4.97766	5.05226	4.8141	4.87372	4.86016	4.76734
DGKI	1.88591	1.88591	-0.757028	-0.131402	1.24663	2.71115
DGKQ	1.18971	0.804012	2.25944	2.0561	2.31699	2.01441
DGKZ	2.40377	2.16396	2.81695	2.86232	3.12315	2.96025
DGKZP1	2.74216	2.6631	3.24659	3.32818	3.74133	3.50406
DGUOK	4.09208	4.10511	3.48714	3.80705	4.08621	4.48187
DHCR24	8.08763	7.99407	7.74232	7.92083	9.19358	9.0532
DHCR7	6.13202	6.14262	5.1151	5.49433	5.68923	5.67103
DHDDS	4.01517	4.037	3.75458	4.11759	4.5071	4.49051
DHFR	5.52432	5.54431	5.95607	6.23204	6.08548	6.24473
DHFRL1	2.779	2.66524	2.9415	2.95182	2.36894	2.09274
DHFRP1	3.4044	3.44263	3.66635	4.02793	3.6907	4.24829
DHODH	3.04408	2.98424	2.61739	2.87132	3.87496	3.581
DHPS	4.11601	4.27048	3.51065	3.87902	4.52686	4.60958
DHRS1	2.3316	2.17742	1.5714	1.79809	2.3026	2.15749
DHRS11	3.24637	2.98837	2.6109	2.23104	3.64689	2.96925
DHRS12	0.243547	0.331671	0.935482	0.773887	0.83161	1.13883
DHRS13	1.88706	1.82328	1.45219	2.05791	1.91214	1.63167
DHRS3	6.71944	6.41616	1.67449	2.04601	5.51554	5.27413
DHRS4	0.651013	0.556444	0.833344	1.3636	-0.17178	0.105236
DHRS4-AS1	0.718622	0.60826	0.359004	0.766358	0.817034	1.32339
DHRS4L2	0.365334	-0.152183	0.435543	0.604195	0.5397	1.15295
DHRS7	3.99947	4.31059	3.04985	3.36192	3.27487	3.98868
DHRS7B	2.67161	2.83408	3.21308	3.6208	3.02969	3.37011

DHRSX	1.02889	1.27191	1.89865	2.43808	1.72774	2.19417
DHTKD1	4.68299	4.72944	3.88391	4.31428	4.15747	4.05483
DHX15	7.23329	7.26485	6.4465	6.73369	7.20946	7.18524
DHX16	4.66669	4.63778	4.16631	4.66596	5.21775	4.94971
DHX29	4.58402	4.81732	5.06263	5.5323	4.7914	5.43539
DHX30	5.89499	5.77222	5.05189	5.24592	5.67171	5.61405
DHX32	4.27042	4.27233	4.90828	5.14145	4.9434	5.17845
DHX33	4.71137	4.50365	4.3302	4.45436	5.27961	4.80404
DHX34	3.54171	3.51872	3.15633	3.35674	3.72556	3.62196
DHX35	3.78574	3.72326	3.58796	3.7784	2.8215	2.63759
DHX36	5.23902	5.21794	5.39058	5.45388	5.76147	5.7971
DHX37	3.60698	3.54839	2.68376	3.18349	3.76909	3.65303
DHX38	4.9938	5.02487	4.95066	5.20781	5.19663	4.6818
DHX40	5.46721	5.68276	4.8249	5.42357	5.88441	6.45377
DHX57	4.63519	4.60323	4.35756	4.43709	4.75436	4.8421
DHX58	0.430296	0.151976	1.55122	1.82784	2.12107	1.96574
DHX8	5.45381	5.33236	5.68375	5.95521	6.01853	5.90659
DHX9	7.8175	7.84256	6.9388	7.279	7.51109	7.61979
DHX9P1	3.37508	3.67849	2.67409	3.0082	3.30064	3.0147
DIABLO	4.5324	4.41715	3.73955	4.14996	4.3249	4.66853
DIAPH1	6.06784	6.22957	6.5115	6.59141	5.60354	5.94926
DIAPH2	3.95026	4.0206	3.67279	3.86041	4.36125	4.80862
DIAPH3	5.15494	5.19972	4.57451	4.81362	4.67727	4.86787
DICER1	6.46252	6.19513	5.48092	5.21422	6.60354	5.5879
DIDO1	6.64147	6.56124	5.88827	5.97397	6.12165	5.69468
DIEXF	4.92296	5.10034	3.63665	4.06235	4.6773	4.89875
DIMT1	3.97334	4.04891	3.97886	4.25202	4.48434	4.67806
DIO2	2.19272	1.37009	3.88526	4.63851	3.97359	4.13562
DIP2A	4.12905	4.18371	5.13613	5.23046	4.20464	3.57405
DIP2B	5.84248	5.73898	5.29219	5.37296	5.75331	5.47195
DIP2C	3.33845	3.25997	3.57636	3.46293	3.66499	3.34314
DIRAS1	2.27248	1.98839	-1.53926	-2.10822	-2.41163	-2.70579
DIRAS3	-2.17488	-2.29223 ?		-4.10635	1.40581	2.02802
DIRC2	3.36297	3.43197	3.28	3.46483	2.90003	3.11984
DIS3	5.90887	5.93497	5.16608	5.40249	4.58749	4.80871
DIS3L	3.40552	3.35253	3.8249	4.17194	3.75755	3.85552
DIS3L2	3.83325	3.80653	3.83776	3.79417	3.61046	3.49725
DISC1	3.30743	2.93566	3.24555	2.94871	1.34288	0.826079
DISP1	2.48581	2.56507	2.49597	2.68885	5.07121	4.78371
DISP2	0.845109	0.0481804	1.17408	0.884113	-0.587897	-1.14178
DIXDC1	2.61381	3.02299	3.92761	4.36994	4.4754	4.76901
DKC1	6.02709	6.07485	4.75905	5.27917	6.44269	6.36002
DKFZP434I0714	1.32887	1.52019	1.662	1.59857	0.939663	0.922708
DKFZp434P228	2.73323	3.29114	1.48856	1.33087	3.56942	3.19585
DKK1	3.53453	3.47673	2.46754	3.55117	5.48274	6.57868
DKK3	6.13713	6.00352	6.26433	6.9638	5.88319	6.40049
DLAT	3.3302	3.35414	4.54525	5.05293	5.19689	5.30307
DLC1	5.82891	5.67222	5.41497	5.27218	0.519683	0.0483059
DLD	6.02241	6.01442	5.59904	5.71691	6.00079	6.39852
DLEU1	2.71878	2.69956	2.56803	2.67582	3.12105	3.34314
DLEU2	3.40724	3.38807	3.68844	3.52937	3.57079	2.87153
DLEU2L	2.1139	1.99506	2.59303	2.32882	1.23057	0.982904

DLG1	5.05784	5.02143	6.12534	6.36756	7.05431	6.87536
DLG3	2.20783	2.47086	1.77651	2.18581	0.652647	1.75275
DLG4	2.13699	1.51447	3.19152	3.17426	2.66837	2.28199
DLG5	4.20083	4.20109	5.31421	5.42296	5.68196	5.49408
DLGAP1-AS1	2.1713	2.19181	0.517004	0.248838	1.39501	1.41227
DLGAP1-AS2	2.49076	2.37952	1.60441	1.95729	2.50712	2.37012
DLGAP4	4.28945	4.14274	3.96458	3.85433	3.07257	3.03458
DLGAP5	5.668	5.69767	4.61899	5.04788	5.16545	5.71377
DLST	4.8795	4.8491	4.05287	4.36433	4.87247	4.96784
DLX1	3.39368	3.23933	1.27595	1.64609	1.60182	1.76905
DLX6	1.72721	1.61934	?	-5.10387	1.01923	1.18815
DLX6-AS1	3.18509	3.06464	-4.59508	-6.09892	3.30548	3.53646
DMAP1	3.86125	3.8489	4.80728	4.86558	4.14784	3.63758
DMD	-0.917461	-0.105152	0.925065	0.476226	2.2526	1.77713
DMPK	2.45518	2.01652	2.79863	2.78766	4.23831	3.93226
DMRT2	2.12431	1.91656	?	-6.09892	-6.64581	-5.69922
DMRTA1	2.72929	2.57608	-1.13882	-0.979274	3.16624	3.18811
DMTF1	5.63293	5.57561	6.34363	6.16526	5.87076	5.40043
DMWD	3.28667	3.06055	3.28842	3.46672	4.02077	3.63266
DMXL1	5.73635	5.80686	5.49772	5.81706	5.91967	6.11784
DMXL2	5.91618	5.79744	6.61746	6.8348	5.54931	5.52904
DNA2	4.07241	4.15738	3.58632	3.68071	3.94979	4.08931
DNAAF2	3.08884	3.17377	1.35568	1.60545	2.61117	3.24458
DNAAF5	3.85263	3.70446	3.9776	4.18004	4.23826	4.30591
DNAH1	-0.0550876	-0.053317	1.85542	2.00494	0.807363	0.483199
DNAH17	0.649726	0.53095	0.386425	0.505972	0.269392	-0.214706
DNAH5	0.334442	0.697098	4.0063	4.30374	2.05396	2.22994
DNAH7	-0.159773	-0.27286	-2.18288	-2.93781	1.37865	1.37014
DNAH9	2.67999	2.68182	-3.85951	-3.52222	-2.95876	-3.70484
DNAJA1	6.56254	6.52546	6.36071	6.70435	7.89616	7.93047
DNAJA1P3	1.53032	1.80397	1.496	2.1152	3.16805	2.80505
DNAJA2	5.33639	5.44366	5.33954	5.80256	6.33119	6.75245
DNAJA3	4.83473	4.86526	4.71356	5.17076	5.02486	5.31357
DNAJA4	0.0425183	-0.177294	-0.790968	-0.436219	0.983605	0.380851
DNAJB1	5.66682	5.62453	5.40315	5.81899	7.17373	7.29029
DNAJB11	5.82545	5.54929	5.93563	6.13098	6.21197	6.07434
DNAJB12	4.48393	4.52582	4.0984	4.56712	5.0509	5.15747
DNAJB14	5.37974	5.43855	5.7378	5.85885	5.70491	5.72733
DNAJB2	3.69323	3.51013	3.59291	3.7193	4.42317	4.20916
DNAJB4	3.22425	3.38891	4.59947	5.17598	5.43013	6.12972
DNAJB5	1.77263	1.13712	2.13842	2.17659	3.21005	2.74038
DNAJB6	5.57585	5.46063	6.12113	6.20033	5.46681	4.9857
DNAJB9	3.75558	3.81713	3.11569	3.5565	4.11858	4.50874
DNAJC1	3.82839	3.58018	2.90395	3.19496	3.75754	3.76902
DNAJC10	6.25258	6.5543	5.6121	6.00196	6.47606	7.05171
DNAJC11	4.57575	4.56124	4.24585	4.53455	4.90827	4.82712
DNAJC12	0.586404	0.822624	-2.48229	-1.25052	2.2734	3.53407
DNAJC13	5.74256	5.81327	5.8161	6.09451	5.41649	5.60446
DNAJC14	5.47136	5.44145	4.63281	4.87584	4.91148	4.67635
DNAJC15	2.19576	2.24798	2.23435	2.47239	-0.551587	-0.348991
DNAJC16	4.06828	4.15186	3.97635	4.3547	4.72284	5.01437
DNAJC17	2.48537	2.38786	1.92417	2.03855	1.91086	1.77423

DNAJC18	3.09372	3.01289	3.59291	3.81003	3.49451	3.48311
DNAJC19	2.18518	2.09365	2.66241	2.71955	3.13576	3.25914
DNAJC19P9	1.01162	1.17751	1.11906	1.29574	2.07436	2.62411
DNAJC2	5.07683	5.15195	4.54645	4.93185	5.24529	5.17432
DNAJC21	4.77107	4.8237	5.36337	5.50034	5.40845	5.24601
DNAJC22	2.76241	2.36689	4.00876	4.24872	3.39226	3.73
DNAJC24	2.85167	3.11081	2.77068	3.1248	3.4651	3.83244
DNAJC25	2.17026	2.24078	2.68635	3.13379	2.57952	2.3212
DNAJC27	1.88116	1.78781	1.73956	1.6855	2.26748	2.18446
DNAJC3	6.22743	6.16009	6.15354	6.60173	5.59795	5.61633
DNAJC3-AS1	1.09539	0.813344	1.07392	0.946514	-0.0454657	-0.577229
DNAJC30	2.15634	1.94976	2.20593	2.36317	1.92956	1.65347
DNAJC4	1.79866	1.46663	1.82772	1.93808	2.00567	2.17595
DNAJC5	6.30155	6.23086	5.8751	6.1067	6.63164	6.44582
DNAJC6	3.87739	4.29453	-1.01332	0.0410467	3.96448	4.20167
DNAJC7	5.10468	5.18104	5.33406	5.6944	5.881	5.92775
DNAJC8	5.60798	5.53137	4.97165	5.31276	5.72316	6.23209
DNAJC9	4.352	4.54636	3.44906	3.81972	5.13282	5.29695
DNAL1	3.29386	3.54405	2.693	3.3605	3.14728	3.56474
DNAL4	1.35081	0.831845	1.98141	2.0711	1.19772	1.23585
DNASE1	4.05517	3.97227	4.53141	4.45973	3.35654	2.77992
DNASE1L1	3.32787	3.42225	2.62403	3.13484	3.48064	4.03873
DNASE2	4.2245	4.05552	3.36095	3.58799	4.98835	5.27803
DNER	0.480917	0.746683	1.29226	2.26188	2.71474	2.91534
DNHD1	4.02997	4.1882	3.74953	3.90686	3.53569	2.82467
DNLZ	-0.154805	0.222285	-0.18935	-0.242198	-0.0706438	0.696216
DNM1	3.91287	3.65089	3.39202	3.14147	3.91712	2.97629
DNM1L	6.60446	6.63437	5.58274	5.89457	5.56247	5.59043
DNM1P51	1.42812	1.09706	1.63716	1.35191	0.731383	-0.234184
DNM2	5.23459	5.07771	5.55686	5.7223	5.83674	5.66521
DNM3	3.97069	3.98527	1.35568	0.773887	4.91332	4.35397
DNM3OS	1.37242	1.58158	-1.72363	-1.93836	-0.217212	-1.006
DNMBP	4.74174	4.95252	4.71556	4.91473	4.62434	4.33448
DNMT1	6.76927	6.75801	6.00752	6.22403	6.4118	6.12538
DNMT3A	4.15578	3.84321	3.79519	3.4496	4.97132	4.15436
DNMT3B	2.91931	2.82718	2.48182	2.50589	1.85147	1.52214
DNPEP	3.21978	3.18282	4.09608	4.62249	4.08284	4.45693
DNPH1	1.00834	1.20963	-0.598354	-0.52367	2.47669	2.87449
DNTTIP1	4.59207	4.5863	3.0477	3.47614	2.88273	3.00065
DNTTIP2	6.20231	6.29434	5.70978	6.16377	6.00616	6.50456
DOCK1	6.13697	6.08634	5.98618	5.95273	5.86792	5.90891
DOCK10	3.6727	3.93483	5.91713	6.0942	5.9284	6.14933
DOCK11	2.58116	2.4614	4.25123	5.01063	1.72342	2.18701
DOCK11P1	-1.55974	-0.871741	0.70967	1.59309	-2.13612	-1.88008
DOCK3	0.693311	0.635262	0.662041	0.935626	-0.320293	-0.848487
DOCK4	4.59093	4.90674	1.93025	2.27489	4.3544	4.929
DOCK4-AS1	0.752201	0.988427	-1.37581	-2.52305	-0.0308197	-0.121607
DOCK5	6.46269	6.33639	4.71657	4.93214	5.68231	5.50213
DOCK6	0.735617	0.419895	1.30034	1.195	2.45604	2.22994
DOCK7	6.13421	6.15324	5.61332	5.74839	5.96951	5.58445
DOCK9	4.162	4.25139	3.62383	3.74136	4.53628	4.26912
DOHH	0.784805	0.370142	0.308431	0.399062	0.91718	0.80115

DOK1	4.32536	4.33318	2.19587	2.49852	2.10458	2.56942
DOK4	2.51525	2.30535	3.87068	4.15462	0.969197	1.04823
DOK5	?	?	-1.65952	-2.02079	0.246688	1.35942
DOK6	0.752201	0.382745	-2.48229	-2.64851	2.85332	2.11349
DOLK	3.66505	3.54837	3.24484	3.53863	3.39362	3.49296
DOLPP1	3.31913	3.29539	3.07151	3.3048	3.43508	3.12934
DONSON	3.37546	3.28177	4.02043	4.05929	3.68175	3.60724
DOPEY1	4.97112	4.97902	3.90756	3.99148	4.72819	4.75379
DOPEY2	1.20786	1.14454	2.39392	3.05357	2.65485	2.85554
DOT1L	3.49358	3.14052	3.05285	2.5462	4.17425	3.52958
DPAGT1	4.28646	4.48227	3.97755	4.342	4.23954	4.23319
DPCD	1.89612	2.0738	2.44007	2.48676	3.7529	3.6153
DPF1	0.577131	0.537236	0.416483	0.350701	0.807363	0.769095
DPF2	4.41514	4.43343	3.82483	4.03837	3.46761	3.2181
DPH1	2.93071	3.07012	3.63209	4.04039	3.30214	3.51243
DPH2	4.86268	4.84605	3.77936	4.32684	5.31981	5.79513
DPH3	4.05664	4.00986	3.27796	3.78447	4.9553	5.42061
DPH5	3.11693	3.16582	3.08327	3.47685	3.53613	4.09455
DPH6	3.07736	2.88146	1.66826	1.94648	2.18833	2.34315
DPH7	3.29669	3.24279	3.07858	3.17426	3.66612	3.58791
DPM1	5.06661	5.097	4.38348	4.7288	4.57029	5.39787
DPM2	4.18356	4.15067	3.25919	3.41669	4.58282	4.25906
DPM3	2.1083	1.52018	1.28413	1.41477	2.22845	2.99026
DPP3	4.38858	4.23439	3.95855	4.14066	4.14023	4.30114
DPP7	3.86602	3.435	3.10188	3.24653	3.37998	3.47568
DPP8	5.04827	5.19937	5.02897	5.34752	5.34143	5.52451
DPP9	3.98931	3.85851	3.744	3.92738	4.82247	4.58377
DPY19L1	6.25756	6.22004	5.6565	5.67262	5.92639	5.32771
DPY19L1P1	0.219203	0.0709938	0.405862	0.364909	-0.00219312	-0.2613
DPY19L2	0.730444	0.736518	0.279923	-0.0703181	1.80982	1.76586
DPY19L2P1	0.00842952	-0.373983	-0.418049	-0.341426	2.73534	2.84924
DPY19L2P2	3.07244	3.10558	0.957959	1.28354	2.5078	2.2044
DPY19L3	5.33123	5.12822	4.95601	4.917	5.44199	5.24457
DPY19L4	4.53453	4.71145	4.72412	4.88763	4.67842	4.94433
DPY30	3.65914	3.91715	2.69459	3.01188	3.80634	4.2091
DPYD	5.06827	5.11076	5.35898	5.65724	6.06755	6.2684
DPYSL2	7.15533	6.94082	6.47826	6.74444	5.27929	5.16133
DPYSL3	5.68464	6.18871	7.73333	8.05343	5.74351	5.92808
DPYSL4	-2.05464	-2.46206	3.41266	2.97599	3.72662	3.06808
DR1	5.84444	6.00093	5.14963	5.51824	6.14823	6.05231
DRAM1	4.69591	4.82659	4.997	5.40193	4.01829	5.05649
DRAM2	2.95988	3.41737	2.87718	3.55255	3.25586	4.41147
DRAP1	5.35586	5.33115	4.52211	5.05544	4.30331	4.51487
DRD2	-2.69815	-2.1403	-0.69145	0.833756	0.529601	1.38079
DRG1	4.98193	5.00958	4.32907	4.94262	4.13293	4.58919
DRG2	3.90695	3.77006	4.03911	4.18156	4.67843	4.76938
DROSHA	5.00352	4.90129	4.81294	4.68882	5.0479	4.75578
DSCC1	3.02714	3.34934	2.62706	2.91355	3.23451	4.01438
DSCR3	4.33657	4.45514	4.70657	4.97266	5.27289	5.41676
DSE	3.62416	3.65869	6.05834	6.27855	3.45858	3.53058
DSEL	4.70022	4.02888	4.26787	3.62074	5.1875	5.71045
DSG2	6.26168	6.73114	1.41643	1.9191	1.72306	2.33222

DSN1	5.31736	5.29536	4.74943	5.13255	4.01212	4.33082
DST	9.23339	9.22177	9.63793	9.9021	10.5922	10.6456
DSTN	7.68071	7.52107	6.85864	7.34129	7.55361	7.83387
DSTNP2	2.12112	2.27188	1.97637	2.32164	2.18206	1.95152
DSTYK	4.50977	4.59039	4.26974	4.61312	4.77171	4.7761
DTD1	4.86521	5.01485	3.91388	4.45637	4.12616	4.45899
DTD2	2.33607	2.63584	2.10325	2.61843	2.64791	3.49197
DTL	5.28889	5.39782	5.15073	5.60842	4.83949	4.9034
DTNA	0.183671	0.060836	-1.01332	-0.301307	3.73522	3.87824
DTNB	2.35622	2.43803	1.70458	1.8454	2.69505	2.82857
DTNBP1	1.91933	2.06074	1.5714	2.02562	1.92843	2.00755
DTWD1	3.61241	3.53639	2.98348	3.03743	3.32998	3.83715
DTWD2	2.85167	2.57883	0.544485	0.505972	3.67954	3.66397
DTX1	1.24934	1.27867	-0.825729	-1.15432	?	?
DTX2	1.60245	1.12784	2.17022	2.49463	2.8757	2.87185
DTX2P1-UPK3BP1-	0.124566	-0.138543	0.898959	0.797743	0.936123	0.957439
DTX3	2.2696	2.09559	3.77213	3.85551	3.68952	3.29047
DTX3L	3.91388	4.04043	5.09493	5.3765	6.36791	6.23305
DTX4	-1.08408	-1.25306	2.39769	2.35062	-0.267833	-0.536593
DTYMK	4.46331	4.54065	4.14064	4.378	4.78157	5.48988
DUS1L	5.05663	4.83578	3.99638	4.10791	5.25332	5.01352
DUS2	2.12751	1.94013	2.76233	2.93354	3.45795	3.2006
DUS3L	2.84191	2.22249	1.5847	1.40691	3.03618	3.00065
DUS4L	2.81491	3.02464	2.2881	2.4659	1.97456	2.18595
DUSP1	1.4199	1.18826	2.17844	2.79205	4.59235	4.29328
DUSP10	3.12589	3.95078	4.32229	4.76152	4.02181	4.48311
DUSP11	3.13855	3.20781	2.44585	2.73039	3.14382	3.63536
DUSP12	3.86982	3.86695	3.04048	3.43614	3.51586	3.84595
DUSP14	4.03902	3.88587	3.43303	3.97331	4.3786	4.39
DUSP16	4.60126	4.37713	3.42196	3.74136	4.37033	4.35667
DUSP18	1.29184	1.30521	1.43797	1.93086	0.342452	0.337745
DUSP19	0.783515	0.656176	0.782333	1.2041	1.5246	1.70271
DUSP22	2.22871	2.02501	2.23435	2.33413	2.40579	2.01441
DUSP28	1.35453	1.5651	1.60441	1.65939	1.47172	1.78517
DUSP3	3.68461	3.8214	4.62864	5.14915	5.98421	5.99411
DUSP4	5.57792	5.43612	5.32029	5.84501	3.2997	3.31565
DUSP5	3.14013	3.63782	3.30833	4.20632	2.53937	2.22404
DUSP6	5.64891	5.7584	2.37924	2.29585	2.58265	2.38385
DUSP7	3.58284	3.25312	2.04532	2.15798	3.65144	3.40181
DUSP8	0.595618	0.151976	-3.59683	-3.30006	1.54918	1.18815
DUT	5.04913	5.25755	4.29867	4.49594	4.46612	4.97715
DUXAP8	4.40979	4.51764	3.45473	3.62238	3.21894	3.2539
DUXAP9	3.63276	3.92654	2.52493	2.64722	3.70907	4.00967
DVL1	3.17743	2.88589	2.96877	3.26186	3.632	3.95327
DVL2	4.18432	4.15462	4.21842	4.49665	4.82601	4.87636
DVL3	3.76035	3.60996	4.7014	4.66842	5.19882	4.79212
DXO	3.12076	2.90046	2.67107	2.8853	3.86099	3.54096
DYM	4.6234	4.46418	3.93021	3.99051	3.50711	3.62868
DYNC1H1	9.21548	9.25533	9.01359	9.21728	9.06907	8.67256
DYNC1I1	1.14646	1.60865	1.09265	1.30061	3.53691	3.86504
DYNC1I2	5.45288	5.4458	4.87808	5.16136	5.29376	5.38452
DYNC1I2P1	4.49382	4.52961	3.9453	4.23009	4.12458	4.56625

DYNC1LI1	4.61324	4.84714	4.30532	4.87343	4.93472	5.42082
DYNC1LI2	6.38084	6.39063	7.09347	7.32458	7.48997	7.18278
DYNC2H1	4.10988	4.07437	5.8357	5.89855	4.31982	4.71324
DYNC2LI1	2.26383	2.33483	2.42751	2.46861	2.64576	3.20618
DYNLL1	5.90633	5.87311	5.41916	5.93589	6.04444	6.42353
DYNLL2	5.64881	5.52831	5.38252	5.43032	5.94585	5.52869
DYNLRB1	4.60296	4.45005	4.44037	4.7692	4.82551	4.68022
DYNLT1	3.31354	3.63122	3.93412	4.4619	4.67226	5.71849
DYNLT3	3.76239	3.85116	3.16078	3.5076	4.41112	4.65738
DYRK1A	5.17635	5.22323	5.66497	5.67059	5.33979	5.15047
DYRK1B	-0.865939	-1.105	0.674533	0.857026	0.878914	1.02127
DYRK2	6.10498	6.08862	3.88874	3.64002	5.4367	4.90212
DYRK3	2.53695	2.73928	2.48891	3.04347	2.69064	3.05816
DYRK4	2.15398	2.15966	1.85604	2.39996	2.04253	2.70435
DYX1C1-CCPG1	0.262	0.224522	-1.70353	-0.988808	1.94162	3.08195
DZANK1	1.6051	1.53154	1.12941	1.57771	1.2101	0.951558
DZIP1	4.6475	4.58434	4.66393	4.6545	3.53691	3.25039
DZIP1L	2.34806	2.43199	2.70523	2.7398	0.947083	0.633217
DZIP3	3.85262	3.83751	5.07797	5.07851	3.51086	3.44049
E2F1	5.10544	4.89305	4.73682	5.01593	4.48051	4.22106
E2F2	3.78876	3.84777	2.5273	2.70496	3.37723	3.31843
E2F3	4.4633	4.2574	4.20017	4.03454	4.64059	4.0565
E2F4	4.88774	4.7411	4.91778	5.29258	6.18475	6.09217
E2F5	3.65823	3.68523	2.49579	2.4629	4.07195	4.28397
E2F6	4.02112	3.95814	3.28841	3.23829	3.91577	4.08438
E2F6P1	2.05527	2.017	1.10031	0.998607	2.08028	2.2937
E2F7	4.78372	5.02799	5.11966	5.67086	4.49892	4.28339
E2F8	3.37907	3.16286	2.46754	2.36286	2.77433	2.30453
E4F1	2.72719	2.5456	2.78803	3.0153	2.34533	2.5925
EAF1	4.50541	4.80012	3.31457	4.14617	4.70835	5.13978
EAPP	3.03732	3.19537	2.21737	2.79506	3.11118	3.74244
EARS2	4.40224	4.19178	4.14963	4.37094	4.68286	4.54831
EBAG9	2.73138	2.81561	2.40521	2.77076	2.90955	3.27057
EBF1	1.81665	2.31196	3.12936	2.86273	1.89431	1.54125
EBF3	2.93755	3.17892	-6.17309	-3.78492	?	?
EBF4	1.17748	0.859151	2.0405	1.58469	-2.85193	-2.8983
EBLN2	1.96983	1.95503	2.36339	1.98923	1.43249	0.937201
EBLN3	4.55003	4.61863	4.47735	4.66841	5.8192	5.78162
EBNA1BP2	5.88299	5.89191	5.12409	5.65599	6.05142	6.21923
EBP	5.24266	5.23064	3.7398	4.01787	4.29242	4.47071
EBPL	3.1211	3.44923	3.78013	4.22991	3.28367	3.52691
ECD	4.12826	4.23412	3.65883	4.00752	4.24733	4.5197
ECE1	6.48702	6.67429	6.00846	6.16758	5.91451	5.48001
ECE2	2.21842	1.9633	1.78828	2.18909	1.98554	2.72442
ECH1	4.29952	4.07173	4.05923	4.28553	4.191	4.29077
ECHDC1	4.63742	4.59175	4.30531	4.61823	5.72148	6.10825
ECHDC2	1.61384	1.74664	-1.79072	-2.52305	2.1145	1.1007
ECHS1	4.27533	4.29287	4.54779	5.02173	5.61202	6.11385
ECI1	2.52493	2.23761	2.77938	3.10306	2.9654	3.26195
ECI2	3.58179	3.54231	3.89567	4.22142	4.63615	5.19367
ECM1	3.87267	4.12211	4.44126	4.75692	1.36764	1.93717
ECM2	1.20183	1.06859	1.42385	0.879925	0.107996	-0.4586

ECSIT	2.2198	2.12214	1.94578	2.15329	2.86717	3.27913
ECT2	5.96293	6.14561	5.67096	6.05891	6.29984	6.52748
EDA2R	-5.69162 ?		-4.59508	-4.10635	1.5394	1.70271
EDARADD	3.23323	3.29905	3.29352	3.87806	4.36261	4.02303
EDC3	4.30049	4.30048	4.08519	4.26167	4.1281	4.25087
EDC4	4.55389	4.51665	4.74273	4.93623	5.57913	5.53958
EDEM1	6.22775	6.06633	5.75973	6.09268	5.99942	5.745
EDEM2	4.80972	4.75872	4.14291	4.4515	3.69724	4.18657
EDEM3	6.31927	6.61658	5.07682	5.53591	6.12514	6.61012
EDF1	5.26886	5.11836	4.89261	5.22264	5.4115	5.7206
EDIL3	4.40932	3.80157	6.20281	6.20534	6.93101	6.46007
EDN1	-2.61075	-1.14061	0.558032	1.43037	1.44302	0.893261
EDNRB	4.42891	4.36052	-0.757028	-1.02108	3.58287	3.16674
EDRF1	4.88	4.88803	4.48819	4.55428	5.23585	5.2542
EDRF1-AS1	0.21364	-0.290692	0.272752	0.0711723	0.240119	0.0138981
EEA1	5.83221	6.01326	5.63078	5.72266	5.10901	5.42832
EED	3.1115	3.10321	4.1214	4.31743	3.54059	3.75681
EEF1A1	11.0417	10.9707	9.85917	10.4018	11.2501	11.7675
EEF1A1P11	2.75944	2.82868	1.54392	2.25602	2.60417	3.79776
EEF1A1P12	1.66933	2.23496	1.05584	1.49188	2.15923	2.0542
EEF1A1P13	3.80029	4.07144	2.80353	3.52191	4.27243	4.54685
EEF1A1P16	0.748195	1.0027	-0.278948	0.44579	1.08923	1.10988
EEF1A1P19	2.86986	2.92106	2.35046	2.66208	3.22055	3.53481
EEF1A1P22	0.230301	0.893393	0.242962	-0.0273647	0.60826	0.670967
EEF1A1P3	0.194264	0.165687	0.747284	0.833756	-0.216342	-0.0660754
EEF1A1P4	1.58884	1.92036	1.22206	1.29747	1.60303	1.27747
EEF1A1P5	11.298	11.1864	10.1272	10.6192	11.5588	11.9986
EEF1A1P6	7.47466	7.61631	6.45598	7.06875	7.78012	8.137
EEF1A1P9	0.0871233	1.20877	-0.00587506	0.53438	0.774865	0.990178
EEF1A2	-4.37417	-4.13839	2.74129	2.78296	3.88079	4.00926
EEF1B2	5.79948	5.65948	4.55533	4.99317	6.19829	7.0001
EEF1B2P3	6.19936	6.04487	5.06336	5.46192	6.65508	7.31514
EEF1B2P6	3.74475	3.62671	2.51613	3.0256	4.04066	5.03992
EEF1D	6.3881	6.17807	6.3028	6.54976	7.02045	7.06113
EEF1DP1	2.44599	2.42099	2.39934	2.66153	3.0663	3.07495
EEF1E1	4.14011	4.15641	3.26563	3.77665	4.68508	5.3951
EEF1G	7.82914	7.79193	7.50658	7.90801	7.86489	7.99322
EEF1GP1	1.38843	1.46201	-0.0335267	0.591967	1.33409	1.68564
EEF2	8.94811	8.76404	8.18969	8.47496	9.929	10.0852
EEF2K	4.3984	4.42068	4.60763	4.60541	4.71483	4.39657
EEF2KMT	2.39351	2.4233	2.55249	2.84473	2.67398	3.17766
EEFSEC	1.01522	0.963482	1.34765	1.72409	1.42582	1.56944
EEPD1	0.289705	0.265185	2.94576	2.74293	0.185296	0.293394
EFCAB11	2.14956	2.3218	1.28413	1.35064	1.2586	2.10053
EFCAB13	2.51675	2.59748	1.00557	0.883691	1.32189	1.06809
EFCAB14	5.11353	5.81509	5.81761	6.83013	4.9	6.00732
EFCAB2	1.31235	1.0353	1.64837	1.30552	1.70952	1.81687
EFCAB7	3.01	2.82775	3.18353	3.20783	2.02621	2.62869
EFEMP1	7.15244	6.56159	2.693	1.8512	6.44707	6.52659
EFEMP2	3.80077	3.24279	5.15687	5.226	0.932212	0.463298
EFHC1	3.76444	3.93161	4.18825	4.22544	4.22158	4.26051
EFHD2	3.4029	2.96552	3.89729	4.02816	5.08745	4.85647

EFNA1	3.85839	3.57471	2.34788	2.67909	2.81137	3.11027
EFNA3	1.66727	1.74176	-0.736607	0.248838	0.293994	0.151378
EFNA4	1.867	1.7171	1.93007	1.81601	1.20392	1.57873
EFNA5	4.20478	4.16559	3.41824	3.15327	1.85935	2.15749
EFNB1	1.66289	1.74664	3.65095	4.03072	0.578176	0.816919
EFNB2	4.11068	4.35253	-3.59683	-1.93836	5.85956	5.92582
EFR3A	4.68247	4.54907	5.37012	5.46289	5.75671	5.70953
EFR3B	2.3426	2.24453	-0.790968	-0.52367	0.82357	0.863209
EFTUD1	3.9884	4.17274	3.25735	3.71119	4.51859	4.6312
EFTUD2	6.38217	6.4022	6.05373	6.53832	7.20147	7.16781
EGF	0.915689	1.27191	4.23853	4.44	3.43377	3.27343
EGFL7	1.12752	0.868134	1.37111	1.22214	-0.878698	-0.497069
EGFL8	0.756588	0.209316	1.01656	1.02064	1.3822	-0.115713
EGFR	5.65389	5.71672	7.12314	7.41107	5.64285	5.46592
EGFR-AS1	0.167268	-0.41703	1.58427	1.4674	-0.149775	-0.917355
EGLN1	6.01085	6.00551	4.27283	4.41668	6.24072	5.36407
EGLN2	4.4949	4.37393	4.47199	4.62076	5.03096	5.13268
EGLN3	-0.721575	-1.105	3.58796	3.42644	4.43442	4.31009
EGR1	0.480917	0.013012	2.73535	2.30058	-0.338214	-0.214706
EGR3	2.07902	2.37006	-4.59508	-3.30006	-5.65305	?
EHBP1	5.99515	5.8143	5.44782	5.37453	5.94766	5.90765
EHBP1L1	5.35485	5.27861	4.99388	4.99446	-1.41201	-2.8983
EHD1	4.69161	4.52157	4.00629	4.26839	4.29606	4.06147
EHD2	2.06581	1.40138	3.86768	3.35471	2.66837	1.70271
EHD3	4.00481	4.52798	2.23434	2.88836	0.197777	1.23585
EHD4	5.30163	5.40944	3.38063	4.10791	5.39598	5.08443
EHF	0.944836	-0.27286	-3.18222	-2.30078	4.20773	3.89691
EHHADH	1.66289	1.766	1.0357	1.01017	-0.412205	-0.662104
EHMT1	4.36801	4.22341	4.33827	4.29039	4.34191	3.92507
EHMT2	4.35348	4.41749	4.1429	4.36081	4.48178	4.57057
EI24	5.48393	5.8234	4.95082	5.69971	5.3495	5.96608
EI24P2	0.710102	0.963733	-0.322682	0.564515	0.373208	0.227026
EID1	6.94613	6.94199	6.49679	6.83715	7.06997	7.06479
EID2	2.55832	1.9295	2.34397	1.89682	2.67062	1.68564
EID2B	1.54413	1.52587	1.24279	1.4458	1.22848	1.08775
EID3	1.867	1.68694	4.50909	4.20405	1.88275	0.965773
EIF1	5.79288	5.80478	5.92338	6.30985	6.43965	6.81558
EIF1AD	4.46757	4.33026	3.74869	3.95592	3.70272	3.27628
EIF1AX	5.60351	5.64156	4.77039	5.19486	5.09868	5.00381
EIF1AXP1	3.91391	3.88538	3.04622	3.60738	3.44577	3.74926
EIF1B	2.84589	3.00272	2.44948	2.69531	2.77224	3.19416
EIF2A	5.25979	5.32086	5.23528	5.68342	6.14359	6.62221
EIF2AK1	5.54832	5.49246	5.54017	5.76599	6.09956	6.15375
EIF2AK2	5.7269	5.78367	6.21422	6.33898	6.94948	6.59127
EIF2AK3	4.31671	4.39487	4.13471	4.53013	4.91796	5.02503
EIF2AK4	6.45685	6.39104	5.29018	5.68558	5.48207	5.49297
EIF2B1	5.31575	5.35243	4.10692	4.48068	5.47375	5.54205
EIF2B2	4.58115	4.48019	3.40572	3.71541	4.32618	4.46558
EIF2B3	3.58325	3.61407	4.16278	4.53503	3.78447	4.30333
EIF2B4	4.35688	4.33235	3.33415	3.68071	4.23116	4.24893
EIF2B5	4.74593	4.801	4.90194	5.27864	5.48562	5.52749
EIF2D	4.26525	4.07728	4.19368	4.38402	4.4888	4.56475

EIF2S1	6.25931	6.49983	5.05111	5.64668	6.32735	6.83977
EIF2S2	6.2179	6.25025	5.82562	6.26951	5.97906	6.45144
EIF2S2P3	2.07643	2.00033	1.67229	2.15859	1.96263	2.16285
EIF2S2P4	5.62062	5.62124	5.20833	5.77698	5.47266	5.91394
EIF2S3	6.3698	6.49025	5.94785	6.27468	6.33552	6.11101
EIF2S3L	5.36682	5.4369	4.88285	5.15365	5.36329	5.34729
EIF3A	7.88921	7.85318	7.4972	7.79662	8.38995	7.96467
EIF3B	6.99798	6.96771	6.84697	7.24923	7.32213	7.21438
EIF3C	7.31396	7.24764	6.33847	6.67118	6.70354	6.53911
EIF3CL	6.26765	6.39392	5.27473	5.61474	5.63333	5.53587
EIF3D	6.29012	6.40751	5.7226	6.28052	6.40088	6.5665
EIF3E	6.5723	6.52604	6.04574	6.43337	6.7362	7.81634
EIF3EP1	3.83498	3.98621	3.13959	3.48218	3.90401	4.76659
EIF3F	5.2626	5.3569	4.84839	5.20495	4.791	5.13096
EIF3FP1	3.36983	3.41895	1.92702	2.00677	-3.65898	-3.63411
EIF3FP3	4.49226	4.63254	3.98986	4.62916	4.27606	4.6417
EIF3G	5.36437	5.32698	4.48514	4.8688	6.14168	6.44558
EIF3H	6.1456	6.16673	5.74388	6.24848	6.53612	7.09559
EIF3I	6.85876	6.92612	5.89822	6.54827	7.74442	8.06068
EIF3IP1	-0.0695851	0.265185	-1.26853	-0.585061	0.919081	0.668401
EIF3J	5.35646	5.34762	4.63389	4.89297	5.60206	5.70571
EIF3J-AS1	1.68217	1.66382	1.24378	1.31796	1.44828	1.45967
EIF3K	5.38629	5.40678	5.05428	5.59679	6.41322	6.87117
EIF3L	7.30752	7.28225	6.30038	6.72554	6.98159	7.33956
EIF3LP1	1.62311	1.53842	0.72472	1.1942	1.55202	1.53306
EIF3LP2	1.47552	1.52731	0.458224	0.907214	0.629557	0.877744
EIF3M	6.19683	6.23637	5.89585	6.29558	7.18533	7.89062
EIF4A1	8.61759	8.64799	7.90411	8.40213	9.27198	9.51394
EIF4A1P10	6.77471	6.83292	5.95041	6.59915	7.4359	7.60982
EIF4A1P2	5.06205	5.21585	4.34503	4.96355	5.74915	5.95314
EIF4A1P4	1.77819	2.14002	0.956324	1.83546	2.40671	2.70701
EIF4A2	7.18329	7.21457	7.63206	7.84293	8.32064	8.56582
EIF4A2P4	1.47227	1.84932	2.09193	2.4069	2.99656	3.01364
EIF4A3	5.52584	5.63978	4.59619	5.16204	5.50489	5.71376
EIF4B	8.17032	8.2425	7.22692	7.6191	7.62501	7.90762
EIF4BP3	4.3277	4.42024	3.28233	3.75278	3.69043	3.82384
EIF4BP6	5.79947	5.81585	4.82737	5.15348	5.15225	5.33977
EIF4BP7	5.39008	5.41101	4.37891	4.72793	4.71767	4.98176
EIF4E	4.90481	5.05063	4.83685	4.96939	5.25744	4.97933
EIF4E2	5.18886	5.13337	4.82103	5.02788	5.93994	6.0546
EIF4E3	-2.30606	-2.76144	3.9889	4.19153	-2.26731	-3.12051
EIF4EBP1	4.96932	5.0553	3.50298	4.22769	4.66888	4.81488
EIF4EBP2	5.61464	5.25525	5.45354	5.49942	5.76804	5.46945
EIF4ENIF1	3.82988	3.67712	3.59729	3.66686	3.35551	3.06333
EIF4EP2	4.61102	4.63249	4.13474	4.50141	4.83263	5.22483
EIF4G1	7.86065	7.81342	8.2216	8.39811	8.86701	8.56729
EIF4G2	8.52579	8.2984	8.04752	8.23758	8.37882	8.48394
EIF4G3	5.70816	5.73895	5.8926	6.06547	6.17376	6.12102
EIF4H	7.07372	6.9949	7.16564	7.56503	7.77107	7.65835
EIF4HP1	4.50417	4.49789	4.71568	5.063	5.22075	4.99514
EIF5	7.16549	7.17183	6.15468	6.48476	7.21243	7.52615
EIF5A	6.8284	6.70623	6.42651	6.82831	7.45855	7.63116

EIF5A2	2.43536	3.037	-3.01245	-3.78492	3.89235	4.41998
EIF5AL1	5.7638	5.65934	5.4131	5.78213	6.43386	6.56161
EIF5AP4	1.11742	1.23089	0.880137	1.31063	1.9181	1.58531
EIF5B	6.13851	6.07604	5.43617	5.60946	6.11726	5.86669
EIF5P1	1.88009	2.03699	0.722553	1.07141	1.9885	2.09972
EIF6	6.23865	6.29944	5.16314	5.94397	5.94284	6.54489
ELAC1	2.01	2.29872	1.55122	1.86971	-0.0602619	0.270565
ELAC2	4.98171	4.93482	5.2285	5.53502	6.05263	5.93307
ELAVL1	5.05204	5.00579	4.68683	4.82339	5.59971	5.34584
ELAVL2	1.12115	1.02102	-0.510905	-0.823182	-0.637731	-0.183001
ELF1	3.55789	3.47257	3.30246	3.82271	3.47713	3.69417
ELF2	4.2867	4.23051	4.56716	4.52587	4.29086	4.53151
ELF4	2.94478	2.45901	4.08091	4.38603	4.89761	4.80305
ELFN2	-3.22536	-3.13966	2.88795	2.9052	-0.121008	-0.495888
ELK1	3.83716	3.71082	2.56468	2.9301	3.24958	3.26771
ELK3	5.91992	5.96557	4.71875	4.84736	4.79227	4.89613
ELK4	5.73955	5.82454	5.62711	5.75519	6.03059	6.14291
ELL	2.09535	1.71211	2.09727	2.11036	2.15028	2.11348
ELL2	4.42285	4.5549	7.63541	7.73395	6.58026	6.66484
ELL2P1	0.933089	1.35511	3.71352	4.06174	3.10681	3.396
ELMO1	2.08551	1.7708	-1.09576	-0.938639	2.54915	2.354
ELMO2	4.52659	4.61061	4.82625	5.19837	3.89119	4.01096
ELMOD1	-2.37642	-2.00285	-4.59508	-5.10387	1.83956	2.53647
ELMOD2	4.25584	4.035	4.34199	4.24321	4.96402	4.84979
ELMOD3	3.079	3.04891	3.3923	3.49851	3.22539	2.83273
ELMSAN1	4.7807	4.59852	4.55625	4.5123	4.13977	3.24748
ELOF1	4.05662	3.82833	2.92239	3.31113	4.76385	4.49541
ELOVL1	5.00567	5.08596	5.1457	5.64188	5.36724	5.74962
ELOVL2	3.72298	3.72574	-3.59683	-3.78492	-0.0163223	-0.577229
ELOVL4	3.56887	3.91327	0.156448	0.491176	1.70166	2.35939
ELOVL5	6.85112	6.70246	7.33259	7.5433	8.8632	8.77443
ELOVL6	4.15423	4.18641	5.1134	5.52959	6.14903	6.50883
ELOVL7	1.63634	1.45605	?	-4.10635	-0.0163208	0.270672
ELP2	5.22015	5.33826	4.62008	4.85397	4.55433	4.71689
ELP3	4.12747	4.21311	3.75752	4.29418	3.93215	4.04984
ELP4	1.71034	1.94232	2.41268	2.81302	2.79912	3.42
ELP5	3.84394	3.8969	3.58796	4.05482	4.31267	4.54239
ELP6	4.08963	4.05481	3.12026	3.35878	4.05135	4.63979
EMB	3.64112	3.99192	?	-6.09892	-6.64581	?
EMC1	6.62166	6.58055	6.42995	6.6325	6.76816	6.4751
EMC10	4.80405	4.64708	2.09785	2.23548	4.88731	5.23276
EMC2	3.74179	3.74295	3.33415	3.5968	3.40712	3.83631
EMC3	4.98495	4.98372	4.94151	5.42701	5.51706	6.01552
EMC3-AS1	2.48939	2.31523	2.67644	2.71206	2.51016	1.50637
EMC4	5.21333	5.12757	4.63932	4.9909	5.00151	5.33932
EMC6	2.35883	2.35013	2.54451	2.69109	3.26837	3.75699
EMC7	4.99656	5.02197	4.52123	5.04282	4.84452	5.31426
EMC8	3.95465	3.96656	3.70521	3.99052	3.77118	4.01268
EMC9	1.26928	1.27439	1.16681	1.33305	2.33386	2.43521
EMD	3.1211	2.89251	2.87988	3.19267	3.51086	3.5293
EME1	4.07811	4.05085	4.20172	4.46657	3.30507	2.92274
EME2	2.89064	2.9613	2.96549	2.99618	2.7815	2.40132

EMG1	5.472	5.44535	4.65013	5.02154	4.60811	4.87577
EMILIN1	5.58313	4.87811	-1.72363	-2.40763	-3.6585	-3.12051
EMILIN2	-1.8912	-1.81849	1.77651	1.61913	1.22848	1.06153
EML1	3.78976	3.96236	2.76485	3.30692	4.69503	5.0101
EML2	2.57613	2.54074	-2.48229	-2.10822	-0.551587	-0.464246
EML3	3.87301	3.45274	3.11314	3.0492	3.31899	3.35267
EML4	5.57274	5.64384	5.73867	5.79561	6.26383	6.05791
EML5	5.07664	4.88121	1.92135	1.9487	3.00003	3.41103
EML6	2.76853	2.89911	2.01126	1.95189	2.27929	2.35939
EMP1	4.28463	5.02699	3.74573	5.78447	4.00945	4.72164
EMP2	6.29555	6.10118	5.79085	5.92801	3.89411	3.86124
EMP3	4.53513	4.51585	5.05068	5.42935	4.42582	4.91808
EN1	0.727258	0.318612	-0.138983	-0.108685	1.31128	1.04823
ENAH	6.77321	6.78505	6.42561	6.88885	8.24288	8.09173
ENC1	5.15777	5.25059	4.56035	5.12512	6.83329	6.74725
ENDOD1	4.60754	5.12867	3.34396	3.83954	4.9484	5.47132
ENDOV	2.01175	1.80397	2.50997	2.58467	1.68845	1.34861
ENG	3.65049	3.4357	5.74722	5.52381	0.0122397	-0.214707
ENGASE	3.89337	3.53856	3.69912	3.58292	3.11283	2.56942
ENKD1	1.77668	1.5651	1.99641	1.99448	2.59473	2.83445
ENO1	9.64694	9.65119	9.72085	10.2207	10.9418	10.898
ENO1-IT1	-0.611409	-0.734698	-0.0541032	-0.000171691	1.04711	0.359476
ENO1P1	5.15081	5.37776	5.47673	5.93471	6.62789	6.18257
ENO2	3.99265	3.86021	3.62222	4.06484	5.37515	4.13249
ENO3	1.43178	1.17331	0.901788	0.649303	0.265761	0.166741
ENOPH1	4.19875	4.20159	3.583	4.03327	4.65198	4.86407
ENOSF1	4.16047	4.06011	2.923	2.94882	2.47488	2.17878
ENOX1	1.43538	1.16656	0.474784	0.736735	0.339787	1.17598
ENOX2	2.74594	2.72945	1.89865	2.30481	3.64689	3.89691
ENPP1	3.92389	4.05973	2.18282	2.44961	3.45473	4.32808
ENPP2	2.41207	1.68694	2.03082	1.96801	-2.75246	-2.53597
ENPP4	3.55476	3.66772	-4.59508	-4.10635	-4.07293	-4.11926
ENSA	6.20914	6.24479	5.34331	5.72395	6.11808	6.43448
ENSAP2	2.92446	2.95004	2.09859	2.5575	3.3061	3.72748
ENTHD2	3.12832	2.88889	2.73734	3.03945	2.34466	2.03633
ENTPD1	4.52566	4.5714	3.44459	3.49357	3.75357	3.66015
ENTPD1-AS1	1.6352	1.66555	1.04909	0.794087	1.665	1.36753
ENTPD4	5.98086	6.0275	5.87386	5.88483	5.67427	5.6398
ENTPD5	3.5062	3.48633	2.73296	2.98692	3.19275	3.22503
ENTPD6	7.32027	6.99435	4.5929	4.64271	5.65868	5.53882
ENTPD7	4.20326	4.64425	3.84846	4.18688	4.14607	4.48804
ENY2	4.94476	4.7963	4.18167	4.41669	4.74429	5.22544
EOGT	4.02286	4.16377	3.07387	3.23769	3.07441	2.90062
EP300	5.04448	4.79217	5.45671	5.38252	5.09957	4.91442
EP400	5.2191	5.06953	5.08384	4.86615	4.51211	4.35925
EP400NL	3.0165	2.97587	3.62541	3.39936	2.2851	1.66045
EPAS1	2.68031	1.85459	6.01766	6.34134	6.06374	5.63313
EPB41	5.2605	5.13067	4.55614	4.69005	5.34385	4.97216
EPB41L1	3.15891	2.99454	4.07622	4.25092	1.7406	1.79316
EPB41L2	6.47427	6.55047	5.29068	5.33825	7.01465	6.65276
EPB41L4A	1.58544	1.61282	-1.33115	-0.919771	1.86382	1.57805
EPB41L4A-AS1	3.61062	3.59409	2.70255	2.93974	3.25387	3.22424

EPB41L5	4.0202	3.67144	3.80361	3.53498	3.53658	3.19717
EPC1	3.37723	3.33915	3.59138	3.33797	3.9972	3.9051
EPC2	4.08228	4.00781	3.36915	3.20179	3.91238	3.81488
EPDR1	4.748	5.01135	5.135	5.37651	5.42479	5.51306
EPG5	5.56243	5.44065	4.86429	5.17079	3.74802	3.71323
EPHA2	5.88585	5.88641	5.67948	6.19838	6.50758	6.59733
EPHA3	2.62286	2.60594	-4.59508 ?		7.7726	8.30936
EPHA4	4.30342	4.56773	6.31989	5.82034	3.78526	3.51682
EPHA5	-0.52896	-0.681268	-3.12632	-2.64879	5.68306	6.01142
EPHB2	3.1131	2.9423	5.52985	6.00262	4.37381	4.38999
EPHB3	2.27248	1.95924	1.84441	1.30061	1.79504	1.23585
EPHB4	4.66941	4.47084	3.54609	3.56005	4.37586	4.32395
EPHX1	3.08392	2.82257	7.3609	7.57011	5.7678	5.5424
EPM2A	0.696492	1.2616	1.21549	1.5363	1.49319	1.9654
EPM2AIP1	4.85933	4.87644	4.87378	4.9773	5.2085	5.17439
EPN1	3.94657	3.67671	4.24898	4.27163	4.927	4.71324
EPN2	3.87606	3.90873	3.6585	3.76968	4.23557	4.33626
EPOR	1.51041	1.52019	1.44587	1.81601	0.807363	1.18815
EPRS	7.02887	7.09798	6.61712	7.07538	7.26185	7.59347
EPS15	5.27566	5.42549	5.37869	5.726	5.38438	5.73571
EPS15L1	3.13381	3.12774	3.6541	3.71454	4.08955	3.53646
EPS8	6.17225	5.8089	4.29018	4.93487	5.17652	5.68666
EPSTI1	-5.69162	-4.13839	1.89332	1.93559	3.39632	3.54358
EPT1	6.50357	6.62912	5.7758	6.12091	5.9981	6.1117
ERAL1	3.72298	3.63255	3.79661	4.11397	4.20849	4.07955
ERAP1	5.60112	5.71419	6.82795	6.9972	5.36763	5.2796
ERAP2	3.93209	4.28114	7.33693	7.6451	4.62625	4.94343
ERBB2	6.3706	7.20902	4.54385	4.62349	-0.249757	-0.746255
ERBB2IP	6.62564	6.6215	6.77741	6.91109	7.46863	7.79497
ERBB3	7.98277	7.68628	-0.825729	-1.46458	-1.48999	-1.61882
ERC1	6.10443	6.04494	5.96875	5.91976	5.43441	5.04565
ERCC1	5.2486	5.23646	4.33466	4.70805	5.29395	5.56546
ERCC2	3.81059	3.34129	3.6206	3.57416	5.50451	4.94057
ERCC3	5.42537	5.50003	4.62865	5.02431	5.44786	5.49293
ERCC4	3.59225	3.66313	3.6897	3.97852	3.2836	3.12765
ERCC5	3.82143	3.7543	4.71289	4.80155	4.32831	4.1341
ERCC6	3.83627	4.17096	3.05149	2.99604	3.83811	3.69273
ERCC6-PGBD3	2.97323	3.00682	1.80757	1.97321	1.76693	1.78241
ERCC6L	4.3777	4.64373	4.20449	4.53592	4.56548	4.81879
ERCC6L2	5.24372	5.25988	4.6342	4.57399	4.98829	5.12198
ERCC8	2.43303	2.68075	3.14273	3.32498	3.4452	4.2103
EREG	3.28106	3.90783	-0.723865	0.491176	-1.26766	-1.31405
ERF	3.97068	3.65479	2.78803	2.91632	4.42315	4.45315
ERGIC1	6.13085	6.22775	6.29273	6.86272	6.88648	7.03793
ERGIC2	5.58872	5.51419	5.21947	5.33203	5.15745	5.71903
ERGIC3	7.61482	7.55689	6.7243	7.0447	6.77295	7.07174
ERH	5.68691	5.83547	4.53964	5.04211	5.6775	6.13127
ERHP1	0.826437	0.921261	-0.239995	0.384956	1.06101	1.1015
ERI1	3.92205	4.04197	3.91977	4.20178	4.37381	4.4607
ERI2	3.12276	3.13504	3.52787	3.73161	3.5457	3.73359
ERI3	4.20704	4.19626	4.56633	4.87913	4.62565	5.10706
ERICH1	1.52013	1.06077	2.18718	2.19497	2.24057	2.2002

ERLEC1	5.04738	5.26253	4.14852	4.84027	5.167	5.92581
ERLIN1	5.08759	5.14632	5.42287	5.75729	5.37276	5.48249
ERLIN2	5.22607	4.94547	5.16631	5.11938	5.24206	4.80503
ERMAP	2.15638	2.18556	2.58162	2.77269	2.16507	1.89403
ERMARD	2.23758	2.1408	2.58467	2.47615	2.66613	3.09742
ERMP1	4.04482	4.28125	5.17416	5.15002	4.59563	4.80577
ERN1	2.40946	2.3413	2.42382	2.74605	2.73202	2.36476
ERO1A	5.68569	5.98267	4.46663	5.17888	5.41716	5.59706
ERO1B	4.65241	4.38968	2.5512	2.66597	1.57335	1.77713
ERP29	6.59048	6.38952	5.20949	5.40506	5.79277	5.59884
ERP29P1	3.13764	3.01263	1.86846	1.89927	2.38575	1.91932
ERP44	4.66762	4.544	4.77491	4.95512	4.43872	4.55133
ERRFI1	4.78724	4.94919	7.06183	6.84881	5.99029	5.7522
ERV3-1	1.57267	1.48737	3.72515	3.22758	1.4936	0.416332
ERVFRD-1	1.57708	1.84552	1.80519	1.37503	1.89046	0.993783
ERVK3-1	4.71017	4.6992	4.17879	4.42322	5.25392	5.36155
ERVW-1	-0.816197	-0.0703908	1.34008	1.33416	0.766027	-0.214706
ESCO1	4.5828	4.70336	3.88122	4.04347	4.66782	5.33769
ESCO2	4.73345	4.99709	4.03806	4.4371	3.79501	4.04315
ESD	4.80524	4.81443	4.50297	4.86416	4.79192	5.47443
ESF1	5.40712	5.43841	4.61493	4.9246	4.83903	5.1567
ESPL1	5.69805	5.65187	4.61819	4.70975	4.20851	3.85552
ESRRA	3.27277	3.03995	2.35714	2.79952	3.35403	4.24959
ESRRG	1.13386	0.785149	-4.59508	-5.10387	1.11453	1.1637
ESYT1	7.04553	6.99294	5.74984	5.95391	5.844	5.64683
ESYT2	6.50558	6.75414	6.23509	6.75051	5.50959	5.66398
ESYT3	1.73974	1.44408	-0.161008	-0.154483	-0.285108	-0.619042
ETAA1	3.99179	3.8969	4.10765	4.19837	4.27042	4.28622
ETF1	7.24476	7.33467	6.48717	7.05646	7.49525	8.01393
ETF1P2	3.01416	3.14373	2.66432	2.82332	3.16909	3.10113
ETFA	5.19155	5.18819	4.86495	5.11938	5.04834	5.4682
ETFB	4.11418	4.14057	4.3115	4.6503	3.78934	4.24311
ETFDH	2.74866	2.78088	3.13541	3.28483	3.02526	3.18852
ETHE1	2.30775	2.18694	1.8702	2.48713	1.8334	2.59184
ETNK1	5.78825	5.87169	5.27999	5.3557	5.573	5.2641
ETNK2	2.87584	2.53569	3.17215	3.38493	3.35873	3.65197
ETS1	6.17088	6.38714	7.27526	7.54381	6.01365	6.08565
ETS2	3.1805	3.27693	3.3498	4.26295	3.83053	3.73829
ETV1	5.45161	5.49279	0.225978	0.520618	3.91617	3.96217
ETV3	5.58456	5.68495	4.2436	4.56969	4.54242	4.35127
ETV4	4.26958	4.03001	1.94578	2.43422	0.159978	-0.247125
ETV5	5.49197	5.52903	4.90791	5.26891	5.42007	5.00235
ETV6	4.77005	4.55117	4.49243	4.50219	4.67619	4.44683
EVA1A	2.44048	2.52016	3.25319	4.11759	4.28146	5.19339
EVA1B	0.704615	0.370767	0.614031	0.965197	1.15625	1.74748
EVA1C	2.84009	2.81537	-0.252626	0.46112	1.29393	1.70271
EVC	4.07474	4.25617	4.37751	4.67616	3.90974	3.61227
EVI2A	0.171399	0.395227	3.01654	3.83555	1.55968	2.57062
EVI2B	0.80878	0.885949	2.68877	2.96511	1.43863	0.909466
EVI5	5.56796	5.44139	4.84298	4.86703	4.83456	4.70584
EVI5L	0.0954519	-0.195907	2.4312	2.45343	1.5394	1.20023
EVL	2.90438	2.30455	1.96568	1.23037	2.14952	1.23482

EWSR1	7.30462	7.27655	7.3508	7.42647	7.0852	6.68703
EXD2	3.42245	3.78627	2.45311	2.894	3.70053	3.97628
EXO1	5.0699	5.19848	4.75015	5.00296	4.65767	4.99891
EXO5	1.87907	2.22496	0.855208	1.13603	1.21062	1.03149
EXOC1	4.15501	4.24536	4.41451	4.59073	6.01696	6.55831
EXOC2	4.73867	4.88588	5.03079	5.29577	4.73896	4.78313
EXOC3	4.71206	4.61251	4.39418	4.63659	4.95122	4.88922
EXOC3-AS1	1.52978	1.14454	1.10193	1.21315	1.36764	0.847941
EXOC4	5.75188	5.77706	5.45399	5.59594	5.58345	5.82954
EXOC5	5.66353	5.81332	4.69257	4.99479	5.8503	5.98256
EXOC5P1	2.84308	3.05655	1.79115	2.23321	3.08038	3.43196
EXOC6	4.42565	4.65152	2.84715	3.14384	3.99074	4.60956
EXOC6B	4.39696	4.33399	4.83258	4.76922	4.40443	4.36071
EXOC7	6.1238	6.16409	5.90855	6.13814	6.77855	6.80971
EXOC8	4.15175	4.26199	4.05612	4.48748	4.49598	4.39493
EXOG	3.45826	3.57333	2.70218	2.81004	3.35232	3.45063
EXOSC1	3.0658	2.98837	3.21308	3.63264	3.44952	3.90615
EXOSC10	5.57537	5.4997	4.99378	5.31736	5.30102	5.12117
EXOSC2	4.976	4.96966	4.38633	4.6864	4.3523	4.26339
EXOSC3	4.09006	4.10701	3.17842	3.45532	5.14214	5.25752
EXOSC4	2.72751	2.5945	1.57693	2.20852	3.41727	4.10707
EXOSC5	2.65408	2.55397	1.57806	2.03075	3.31553	3.9281
EXOSC6	4.52913	4.54768	4.28408	4.58552	5.74588	5.37143
EXOSC7	3.88211	3.93054	3.24902	3.61147	4.19999	3.99198
EXOSC8	3.653	3.68128	3.77718	4.04983	3.67596	3.8696
EXOSC9	4.9606	4.90024	4.39589	4.62194	4.49353	4.72415
EXPH5	-0.359059	-0.0876659	0.849967	1.15801	1.54918	1.23585
EXT1	5.43724	5.37162	7.04226	7.03154	4.29097	4.18355
EXT2	6.89295	6.83072	6.22825	6.54246	6.09017	6.21434
EXTL1	5.31458	4.9749	-2.86056	-3.78492	0.0263185	-0.4586
EXTL2	4.51765	4.88364	4.14514	4.42058	5.21122	5.95236
EXTL3	6.19505	6.03351	5.50863	5.3785	6.39127	6.18808
EYA1	-1.37679	-0.655276	3.78659	3.34605	-4.33548	-5.69922
EYA2	?	?	-1.72363	-0.716287	1.13416	1.20023
EYA3	4.4589	4.49498	3.90128	3.98657	4.90002	4.71534
EYA4	3.91924	3.82371	5.03261	5.33558	5.86619	6.06766
EYS	2.27106	2.4428	0.662051	0.491176	1.00508	0.522196
EZH1	4.36566	4.2625	3.92107	3.90798	4.4469	3.80108
EZH2	5.12637	5.07611	5.15788	5.14783	5.09566	4.87709
EZR	5.71505	5.70865	4.87037	5.54715	7.08174	7.14617
F11R	1.8792	1.6959	2.83637	2.82649	4.71222	4.45859
F2R	6.46377	6.38831	5.95121	5.95523	5.79423	6.07873
F2RL1	-4.37417	-4.87366	2.17844	2.58118	-5.65305	-4.11926
F2RL2	3.06248	3.00883	5.53969	5.55471	1.9581	2.68131
F3	1.93394	2.51731	6.10015	5.94542	2.63662	2.2533
F8	1.44058	1.99275	0.723445	1.13959	1.00538	2.00755
F8A1	2.42553	2.19181	2.09069	2.07687	1.79252	1.64506
F8A3	-3.73066	?	-0.196632	0.297931	1.65085	1.31192
FAAP100	3.41075	3.3637	3.23433	3.28349	4.08535	4.31009
FAAP20	2.59053	2.45058	2.83182	2.80104	3.01074	2.8576
FAAP24	1.05586	0.920918	1.15638	1.34243	1.22848	1.2122
FABP7	1.06916	1.53154	-6.17309	-6.09892	5.15504	5.8622

FADD	3.65968	3.84323	2.85413	3.58596	2.97127	3.16951
FADS1	7.91214	7.81748	7.16	7.63835	6.48171	6.93706
FADS2	7.5125	7.18825	7.93879	8.26217	4.98372	4.97301
FADS3	3.65847	3.60593	2.86361	3.43226	4.04359	3.8305
FAF2	5.89664	5.73252	6.66958	6.82688	6.56609	6.19678
FAH	3.20179	3.19715	3.75311	4.42936	3.59708	4.09094
FAHD1	2.7542	2.83866	2.8403	3.4775	2.91044	3.3992
FAHD2A	3.32069	3.23834	3.17889	3.41148	4.10335	4.2291
FAHD2B	1.21257	1.17486	-0.515858	-0.478047	1.19335	1.3126
FAIM	1.90086	1.7708	1.60441	1.58469	0.0540566	0.522196
FAIM2	-1.52875	-0.354151	0.355738	1.59857	-1.13645	0.993783
FAM101B	1.15895	0.013012	4.78873	4.81968	-6.64581	?
FAM102A	2.5802	2.5528	2.60982	3.06165	2.52866	2.3963
FAM102B	4.09289	4.42287	3.56969	3.85407	1.72343	2.00756
FAM103A1	3.43204	3.49096	2.49935	2.9332	3.84792	4.13933
FAM103A2P	2.57238	2.45146	1.70903	1.99534	3.11008	3.00728
FAM104A	4.03714	4.05134	4.57619	5.01787	5.36812	5.61928
FAM104B	1.27825	1.12217	0.571444	0.685662	1.81139	2.11985
FAM105A	0.183671	-0.00328298	2.81084	2.87702	-1.45047	-1.70626
FAM106A	?	?	1.5375	1.17718	0.833441	-0.104544
FAM107B	3.98391	4.16376	3.52728	4.48175	3.05222	3.10707
FAM109A	1.98394	1.94657	1.39394	1.41477	0.939663	0.359476
FAM109B	2.6911	2.36689	1.88259	1.97335	-4.07293	-4.11926
FAM110A	-0.223172	0.060836	0.871828	1.03078	-1.44343	-0.848487
FAM110B	-5.69162	-6.45019	1.14744	1.0511	-0.0752099	-0.214706
FAM111A	5.27028	5.29743	5.79977	5.94984	5.33292	5.38605
FAM111B	4.59612	4.70394	4.63426	4.70012	4.53075	4.94254
FAM114A1	4.82741	4.6193	5.10186	5.60111	5.10621	5.13247
FAM114A2	2.5055	2.77796	3.49067	3.78447	4.02794	4.47568
FAM117A	1.12061	0.582527	1.81567	2.0766	-0.664434	-0.421131
FAM117B	2.80674	2.82025	4.7196	4.91561	2.27928	1.89323
FAM118A	2.69325	2.8991	2.74426	3.03838	2.68843	2.54122
FAM118B	2.93918	2.92781	3.17564	3.54026	3.05175	3.39035
FAM120A	5.84091	5.91734	6.31757	6.64049	6.76487	6.49102
FAM120AOS	3.27613	3.35088	3.11472	3.53923	3.91787	4.14973
FAM120B	4.3804	4.32014	4.61981	4.63766	5.04749	5.0101
FAM120C	4.43726	4.6411	3.06204	3.27488	2.30549	2.97629
FAM122A	2.37508	2.29872	2.24276	2.44961	2.58049	2.14505
FAM122B	3.71243	3.50294	4.42565	4.31112	4.38613	4.01096
FAM122C	0.00148521	-0.105152	1.25947	1.30907	0.961801	0.893261
FAM126A	6.68758	6.53293	6.0768	6.34542	5.90476	6.07341
FAM126B	4.4865	4.54821	4.22565	4.36974	4.97659	4.59935
FAM127A	2.49569	1.93378	4.03323	4.18455	5.34459	5.39392
FAM127B	1.17748	0.75605	2.13391	2.24997	3.4675	3.28214
FAM127C	-5.69162	-5.43143	3.13615	3.2328	3.18383	3.07446
FAM129A	6.70158	6.68738	4.74868	5.57505	7.2822	7.53107
FAM129B	5.98888	5.84007	5.87712	6.22676	6.47608	6.71039
FAM131A	-1.61119	-2.06994	2.72937	2.96799	4.14543	4.27056
FAM132B	1.85554	1.03706	0.686917	0.605504	0.405873	0.68568
FAM133A	1.37777	1.25145	-1.72363	-1.15432	-2.41163	-2.53597
FAM133B	4.16861	4.19798	4.7899	4.75803	4.09381	4.17617
FAM133CP	0.0463933	0.105383	1.01082	0.832793	0.24687	-0.171404

FAM133DP	2.49649	2.69913	3.08243	3.02407	2.51279	3.14023
FAM134A	5.09545	5.53907	5.07748	5.73155	5.81193	6.39758
FAM134B	3.35392	3.4044	-0.510905	-0.201784	-0.876885	-0.899103
FAM134C	5.04867	5.31235	5.43072	5.85695	5.72607	5.9208
FAM135A	4.76954	4.70116	2.70762	2.49851	4.29968	4.15592
FAM136A	4.64577	4.7477	3.77199	4.25844	5.50941	5.86781
FAM13A	3.9706	3.8976	4.58708	4.62518	3.56979	3.38031
FAM13A-AS1	0.988179	0.659943	2.14773	2.08998	1.35054	0.865729
FAM13B	4.34988	4.32906	4.7279	4.85636	4.94803	5.08044
FAM13C	-2.17488	-2.6546	-1.86109	-2.52305	4.9892	4.76436
FAM149B1	4.11136	4.17669	3.9466	4.18641	4.83596	5.06676
FAM155A	1.18971	1.05291	-4.18092	-5.10387	?	?
FAM156A	3.78838	3.81033	3.47787	3.46514	3.51048	2.82705
FAM156B	3.1626	3.08508	2.95403	2.89634	2.51788	1.78829
FAM160A1	2.71667	2.35346	0.699196	1.42259	3.21587	2.40704
FAM160A2	3.53932	3.45005	3.15187	3.21985	2.90194	2.55539
FAM160B1	4.48207	4.23931	4.22799	4.16962	4.83503	4.06147
FAM160B2	3.15423	2.94443	3.62222	3.74603	3.03492	2.85935
FAM161A	2.46835	2.48261	2.01617	1.95189	2.61815	2.34315
FAM161B	1.57073	1.58893	0.803095	0.961179	1.26991	1.0163
FAM162A	2.89339	3.13335	2.34787	2.72723	5.07651	4.88292
FAM168A	5.54408	5.53326	5.27179	5.39645	6.08974	6.00709
FAM168B	6.39434	6.38303	5.66235	5.91631	6.69159	6.45409
FAM169A	3.13556	3.22339	2.41332	2.3989	3.41614	3.56344
FAM171A1	4.30302	4.06853	3.51691	3.30692	3.43376	2.49787
FAM171A2	0.915697	0.181128	1.63029	0.798133	-1.37455	-2.53597
FAM171B	4.1651	4.24365	3.06915	3.29844	4.49197	5.1964
FAM172A	3.67839	3.56506	3.56802	3.53142	3.97636	4.23873
FAM173A	0.847877	0.537236	0.838911	0.724135	1.17263	1.68564
FAM173B	3.50793	3.60996	3.76922	3.80107	3.62856	4.00581
FAM174A	1.26097	1.2583	1.18285	1.24877	2.86522	2.54596
FAM175A	2.79251	3.05672	2.73732	2.82207	2.45994	2.73675
FAM175B	3.75933	3.877	3.97003	4.31007	4.31696	4.39788
FAM177A1	3.28819	3.68436	2.80233	3.53142	3.86128	4.00925
FAM179B	4.07643	4.15457	3.57137	3.60197	4.44184	4.61859
FAM184A	2.85024	2.64307	-2.5977	-4.10635	2.95321	2.51148
FAM185A	1.7247	1.77985	1.74944	1.78083	1.27424	1.42799
FAM186B	0.643025	0.887744	0.725279	0.928511	-0.199739	-1.77
FAM187A	0.579306	0.305083	1.37144	1.66978	2.56867	3.3277
FAM188A	3.25657	3.30203	2.89863	3.07357	3.20387	3.78913
FAM188B	2.42308	2.13328	0.817713	0.666666	1.9688	1.9121
FAM189B	3.69216	3.59243	2.49597	2.74916	3.24508	3.42771
FAM192A	4.32054	4.12075	5.00505	5.15119	5.48828	5.36581
FAM193A	3.54409	3.3998	3.54269	3.44959	4.16464	3.56708
FAM193B	3.07736	2.81793	4.25006	4.06734	3.40039	2.45821
FAM195A	1.88966	1.71211	1.84993	2.04094	2.56129	3.19717
FAM195B	3.32744	2.79691	2.05012	1.87419	4.72014	5.08607
FAM196A	2.32885	2.282	2.60113	2.30058	1.17263	1.00759
FAM196B	0.00148521	0.570657	-1.2763	0.139221	2.37313	3.74861
FAM198B	4.7146	4.78503	-2.72315	-3.10759	5.97145	5.89506
FAM199X	5.44937	5.68692	5.64855	6.12148	6.03531	6.21288
FAM19A2	3.3764	4.21486	-0.935332	-1.46458	-2.01588	-2.24661

FAM19A3	2.61834	3.04494	-6.17309	-4.52056	?	?
FAM200A	2.58888	2.53327	2.41948	2.43225	2.64377	2.76094
FAM200B	4.83667	4.75025	2.83608	3.02046	3.96357	3.87071
FAM204A	4.25438	4.22837	4.45219	4.68713	4.77117	4.85362
FAM206A	2.53455	2.60056	1.83331	2.21762	3.02794	3.4582
FAM207A	0.636228	0.74123	0.571444	1.14437	1.33222	1.38254
FAM207BP	-0.107469	0.271724	-0.377315	0.610908	0.716438	0.905197
FAM208A	6.53624	6.69448	5.57082	5.72802	6.31173	6.29321
FAM208B	7.00454	7.06443	5.62576	5.83746	6.92181	6.79927
FAM20A	2.11151	1.54281	1.16526	1.14861	1.56373	1.1637
FAM20B	6.61181	6.4333	5.4481	5.73939	6.20696	6.01523
FAM20C	6.37584	5.9909	5.76702	6.25995	5.14906	5.26914
FAM210A	3.2287	3.04297	3.01124	3.28134	4.37791	3.92082
FAM210B	5.6612	5.28785	3.99514	4.03835	5.01696	4.64973
FAM212B	2.6233	2.44571	1.95803	2.13236	3.88028	3.42515
FAM213A	3.59554	3.51725	1.24279	0.120113	-4.33548	-3.70484
FAM213B	2.31496	2.18823	2.65883	3.08839	2.45344	2.82857
FAM214A	3.19165	3.02614	3.95724	3.72683	3.85632	3.66123
FAM214B	2.62286	2.08021	3.20665	3.42256	4.6446	4.51003
FAM215B	2.04575	1.96344	1.64306	1.50592	0.5192	-0.348991
FAM216A	2.96937	2.99334	1.94279	2.25171	2.44426	2.7689
FAM217B	4.06097	4.26206	3.01706	3.27123	3.61293	3.95583
FAM219A	3.29387	3.24452	3.8263	4.06235	5.82148	4.99458
FAM219B	3.95657	4.07742	4.12356	4.33842	4.25949	4.29798
FAM21A	4.77041	4.72889	4.35416	4.64377	4.73488	4.81479
FAM21C	4.53158	4.63368	4.06998	4.40824	4.50213	4.61064
FAM220A	3.0186	3.16104	3.14963	3.59507	3.18204	3.5293
FAM221A	-4.11163	-4.87366	-2.37543	-2.78591	2.31699	1.44308
FAM222A	1.27825	1.08411	-1.72363	-1.30113	-0.105579	-0.619042
FAM222B	2.88589	2.43199	2.59785	2.50957	3.35372	2.34315
FAM225A	3.08969	2.63584	2.18317	2.55871	-1.38691	-1.97994
FAM225B	2.33014	2.31115	1.90884	2.26789	0.151443	0.354418
FAM227A	1.04917	0.877069	2.16964	1.93559	-0.777433	-2.24661
FAM227B	0.499762	0.508429	1.17739	0.825729	0.503685	0.327952
FAM228B	0.62293	0.346146	0.569122	0.248656	1.14002	0.37759
FAM229A	1.70957	1.66014	1.67982	1.60937	1.93748	0.76427
FAM229B	0.784805	0.97184	1.47473	1.66599	2.54427	3.69203
FAM26E	-4.69535	-2.87684	2.88795	3.27704	1.34536	1.4532
FAM27C	1.03912	0.94694	-0.715936	-0.221497	0.46335	0.618765
FAM27E3	1.58173	1.68185	0.445928	0.588603	0.898146	0.832509
FAM32A	4.92752	4.86918	4.33807	4.74058	6.16237	6.42805
FAM35A	4.72757	4.62699	4.29424	4.54233	5.44262	6.14934
FAM3A	2.98392	2.70458	2.91451	2.84828	3.29098	3.25329
FAM3C	5.86329	5.89644	3.96151	4.35292	6.51801	6.25248
FAM3C2	4.79751	4.83389	2.88715	3.33563	5.45989	5.50527
FAM43B	-0.0407343	-0.629746	0.759062	0.773887	?	?
FAM45A	3.0549	2.88848	3.30209	3.67065	3.32557	3.73351
FAM45B	2.82752	2.68901	3.05275	3.42673	3.20384	3.31653
FAM46A	5.31669	5.14307	3.97507	4.73899	5.66359	5.6952
FAM46B	1.94119	1.81331	-0.0541032	0.736735	-2.33767	-3.12051
FAM46C	1.14018	1.61934	1.02599	2.07607	0.147163	1.03481
FAM47E-STBD1	2.00098	1.90775	2.38944	2.94894	2.92999	2.70692

FAM49A	-3.2383	-2.6546	2.33219	1.86274	-5.07051	-3.70484
FAM49B	5.1206	5.2873	4.79951	5.12418	5.50145	5.78913
FAM50A	3.95825	3.81444	3.89462	4.23102	4.88272	4.74139
FAM53B	3.48144	3.25744	3.81638	3.90985	4.3095	4.08177
FAM53C	5.28139	5.04192	4.95665	5.26347	5.09457	5.0058
FAM57A	3.74074	3.67414	3.30833	3.52598	4.83453	4.47568
FAM58A	2.00485	1.97181	2.25062	2.69179	2.55644	3.1599
FAM60A	5.19859	5.50812	4.02988	4.42552	4.68865	5.08791
FAM60CP	2.05118	2.39728	0.545662	1.21205	1.43832	1.85237
FAM63A	2.56537	2.63256	3.95857	4.60455	3.61697	3.28197
FAM63B	4.38373	4.55325	3.96623	4.44862	4.41314	4.16365
FAM64A	3.83812	3.74039	3.98616	4.51731	4.30026	4.80074
FAM65A	2.51264	2.12555	3.54092	3.44479	3.2704	3.23287
FAM65B	-3.8896	-5.45651	-2.48229	-1.58485	3.5947	3.29551
FAM65C	0.0425183	0.137189	5.80303	5.94747	0.766027	-0.348991
FAM69A	3.22207	3.03525	4.55764	4.60421	5.14701	5.39609
FAM72A	2.50818	2.67314	2.26837	2.62343	2.64789	2.7925
FAM72B	2.93141	2.74175	2.15136	2.11064	3.78391	3.69257
FAM72C	2.3658	2.23864	1.59938	1.64278	2.19176	1.34637
FAM72D	2.43086	2.64661	1.45878	2.0837	2.06374	2.26032
FAM73A	4.61436	5.01339	4.54863	5.19551	5.07269	5.48557
FAM73B	3.17897	2.80862	3.09027	3.26404	3.06761	3.12301
FAM76A	1.85554	1.97488	1.98141	2.28553	2.2254	2.22994
FAM76B	2.79878	2.91654	3.48182	3.50772	3.15508	2.61523
FAM78B	4.67759	4.55048	-3.18222	-3.78492	-4.33548	?
FAM81A	1.6541	1.79457	-1.13882	-1.30113	1.94705	2.01441
FAM83D	5.39627	5.42967	5.05306	5.58856	5.25254	5.073
FAM83G	3.45067	3.53284	1.18978	1.48311	4.39427	4.24453
FAM84A	-4.11163	-6.45019	-0.252626	-0.226029	1.61119	1.86317
FAM84B	5.24071	5.87424	1.3479	2.14386	3.70545	4.56474
FAM86B3P	0.614795	0.714127	1.40266	1.43781	0.0061041	-0.364177
FAM86C1	1.97184	1.80538	1.65423	2.18376	1.01761	1.13117
FAM86C2P	-0.337702	-0.838711	1.32602	1.22879	-0.617514	0.0484734
FAM86DP	4.66551	4.64508	2.59285	3.08479	2.02989	2.14682
FAM89A	3.14328	3.17378	2.91977	3.12958	2.35094	2.77711
FAM89B	3.11498	2.95174	2.38103	2.50286	2.56085	2.87527
FAM8A1	4.19042	4.09554	3.90526	3.92731	5.12715	5.15204
FAM91A1	5.556	5.55375	5.38434	5.82952	6.48218	6.3516
FAM91A3P	2.13125	2.02411	1.71224	2.26166	2.85424	3.1607
FAM92A1	3.73065	3.61526	4.29004	4.23518	3.85335	3.57323
FAM92A1P1	1.06879	0.626084	1.63559	1.64131	1.59977	1.59788
FAM95B1	1.12667	0.964147	-2.30381	-2.40763	1.47616	0.670396
FAM96A	4.46202	4.43801	3.71884	4.03455	4.46445	5.35127
FAM96B	3.40421	3.44705	3.44583	4.03965	4.825	5.28905
FAM98A	4.96179	5.00169	3.9889	4.57855	5.37415	5.48311
FAM98B	4.09452	4.037	3.12709	3.19496	3.01917	3.2358
FAM98C	1.50553	1.52587	1.00636	1.4458	1.77017	2.0482
FAN1	4.81135	4.69411	4.42902	4.4676	3.91296	3.93706
FANCA	5.09673	4.99284	4.54352	4.57088	3.79848	3.17753
FANCB	3.46583	3.5609	3.10419	3.24873	2.99791	3.36206
FANCC	3.25047	3.18886	3.57978	3.51402	3.46798	3.20293
FANCD2	5.62761	5.67908	5.01021	5.13163	5.15862	5.01218

FANCE	2.32053	2.47968	1.78805	2.41082	3.08621	3.13878
FANCF	2.73138	2.82487	2.56468	2.65937	2.0086	2.10066
FANCG	4.01687	4.00169	3.46214	3.79656	4.21158	4.40703
FANCI	6.61634	6.74533	6.16736	6.35976	5.88125	5.83307
FANCL	4.02781	4.02379	3.83725	3.78683	3.77848	3.9429
FANCM	4.3412	4.44328	3.72186	3.88835	4.05479	4.0958
FAP	3.26847	2.5487	0.272059	0.348153	-3.5256	-3.70484
FAR1	5.03688	5.09074	5.36335	5.39897	5.04052	4.92808
FAR2	3.11983	3.29037	1.65571	1.63902	2.11628	2.92566
FARP1	5.581	5.40999	5.69454	5.3512	3.35879	2.53745
FARP2	5.71162	5.71286	4.79854	4.99937	4.97849	4.83274
FARS2	2.5055	2.41981	2.21738	2.49852	2.84946	3.20318
FARSA	5.1999	5.13286	3.66979	4.16729	5.88997	5.89643
FARSB	4.61549	4.63847	4.62945	5.10123	4.96036	5.23945
FAS	3.60708	3.48456	2.66628	2.8656	2.42717	2.52212
FASN	7.03349	6.76143	5.91352	5.89398	6.96011	7.66341
FASTK	4.17973	3.96027	4.51257	4.85622	4.72824	4.98938
FASTKD1	3.57472	3.435	3.22587	3.44385	3.9284	4.71611
FASTKD2	4.90499	5.05875	4.01124	4.37498	4.9763	5.61463
FASTKD3	3.32328	3.32179	2.93801	3.10549	3.21006	3.581
FASTKD5	4.54104	4.38107	3.92859	4.22321	4.037	4.12257
FAT1	9.40009	9.31844	8.41311	8.24657	8.49709	8.16979
FAT3	-2.79121	-3.29152	-1.37581	-1.78641	4.93724	5.04734
FAT4	2.3535	2.40445	5.45807	5.08779	5.37483	5.61463
FAU	6.23062	6.1754	5.18199	5.74947	5.57821	6.406
FAUP1	4.44904	4.47543	3.48952	3.90339	3.90229	4.38486
FAXC	0.399051	0.432061	-0.0748604	0.549472	4.36896	4.42899
FAXDC2	2.59785	2.42895	3.23644	2.96264	2.3397	1.75275
FBF1	2.80011	2.5525	2.92857	2.89473	1.9215	1.84977
FBL	4.65296	4.42208	4.43118	4.72167	5.30691	5.08688
FBLIM1	-3.2383	-3.46127	3.23222	3.39101	4.55098	4.92537
FBLN1	-6.68418	-6.45019	1.662	1.41477	2.77849	2.70692
FBLN7	1.9484	1.59247	2.11571	1.71775	-1.80184	-2.38405
FBN1	6.90718	7.0695	6.67402	6.80999	6.49337	6.60245
FBN2	5.20176	5.06509	4.72786	4.53141	4.10124	3.9299
FBRS	3.1179	2.85909	3.57636	3.58465	3.02444	2.71956
FBRSL1	1.15272	0.877069	1.5714	1.30061	1.22848	0.597145
FBXL12	2.06249	1.72206	2.53417	2.47615	3.24958	2.49297
FBXL14	1.36166	1.12967	1.78805	2.15329	1.70166	1.92267
FBXL15	0.311956	-0.130408	-0.0336419	0.185093	0.166458	0.265557
FBXL16	0.121214	-0.418273	1.95605	1.99972	-3.6585	-4.11926
FBXL17	2.92846	2.85684	2.83608	2.84828	3.23148	3.59249
FBXL18	2.23168	2.02903	2.30031	2.4911	2.50962	1.97981
FBXL19	3.30794	3.1681	3.05237	3.26186	3.18204	2.97277
FBXL19-AS1	1.10187	0.90354	1.01621	1.02051	0.947083	-0.38461
FBXL2	2.94298	2.84094	2.68993	2.72723	3.64575	3.48558
FBXL20	4.39434	4.26592	4.30331	4.25092	4.69668	4.40571
FBXL3	4.27403	4.50707	4.30552	4.60692	4.56238	4.87755
FBXL4	3.40552	3.48259	3.55456	3.5636	4.40444	4.39919
FBXL5	5.28603	5.26378	4.81365	5.10062	5.58585	5.98021
FBXL6	2.02244	1.89209	2.03788	2.33701	2.39882	2.09021
FBXL7	1.399	1.31199	2.16964	2.20408	?	?

FBXL8	-0.730562	-0.655276	0.975755	0.769433	0.427241	0.0163822
FBXO10	2.2299	2.11361	2.28681	2.38847	3.89302	3.62077
FBXO11	4.84789	4.7412	4.49816	4.37617	4.62366	4.75806
FBXO16	0.374043	0.310445	0.518384	0.677359	-0.309408	0.151002
FBXO17	2.02544	2.18103	3.54778	3.70495	2.83155	2.61867
FBXO18	4.6847	4.54726	4.49047	4.62315	5.13637	5.08029
FBXO21	4.79578	4.80041	4.15632	4.55651	4.61637	4.6813
FBXO22	6.23976	6.41089	4.59587	4.78801	4.73882	4.77769
FBXO25	2.94373	2.94679	2.27592	2.89537	3.88651	4.10667
FBXO27	-2.17488	-1.7075	1.68687	2.34114	-1.90489	-1.81523
FBXO28	4.93117	5.02247	4.48447	4.74603	5.32943	5.46446
FBXO3	4.1828	4.21575	4.20341	4.35469	4.54181	4.61746
FBXO30	4.01773	4.06853	5.37911	5.37397	5.55913	5.43281
FBXO31	3.8555	3.73068	3.83883	4.00231	3.23731	3.06148
FBXO32	3.75727	3.66127	4.13275	5.04345	8.04693	7.85906
FBXO33	3.49321	3.58154	2.81366	3.20859	3.58762	4.1881
FBXO34	4.55358	4.57813	3.26562	3.69208	3.98263	3.99199
FBXO36	0.604783	0.292146	1.94578	2.18581	0.886652	0.702755
FBXO38	4.51645	4.53503	5.23906	5.50863	5.25107	5.46508
FBXO4	1.53936	1.43806	0.759062	1.08105	1.5443	2.21217
FBXO41	3.39103	3.05286	3.43853	3.20406	1.52955	0.878317
FBXO42	3.63965	3.50581	3.99514	4.09206	4.57091	4.22697
FBXO44	1.66727	1.59788	3.12464	3.44146	1.95392	1.89323
FBXO45	4.25583	4.26846	4.40893	4.56007	5.82673	5.71639
FBXO46	1.96272	1.78622	2.0486	1.75539	2.67736	2.09257
FBXO48	1.16516	1.24456	1.30837	1.55655	0.134221	-0.799586
FBXO5	4.09127	4.1051	3.9393	4.15444	4.49828	4.6607
FBXO7	6.45875	6.37614	5.54098	5.89574	5.13411	5.21808
FBXO8	2.15579	2.14443	2.09271	2.49646	2.47596	3.28643
FBXO9	4.02287	3.79807	3.87582	4.10791	4.87155	4.55302
FBXW11	5.10099	5.33763	5.43025	6.03611	6.01895	6.18128
FBXW11P1	-0.611409	-0.292758	0.102	0.687195	0.459264	0.151378
FBXW2	5.54765	5.6437	5.12935	5.41179	6.05929	5.92628
FBXW4	1.37928	1.26379	1.95093	2.24354	1.82245	1.90744
FBXW5	3.69431	3.19715	3.32823	3.46483	3.5769	3.56241
FBXW7	4.00445	3.97679	4.36508	4.40048	4.4136	4.31121
FBXW8	3.44324	3.35678	2.70294	3.05206	2.48728	2.28446
FBXW9	1.2317	0.756391	0.966283	0.749225	2.35093	2.61071
FCF1	4.13595	3.99125	3.27158	3.64773	4.55634	4.45528
FCGR2A	1.98713	1.77171	1.49588	1.42763	-1.53062	-1.18284
FCGRT	1.05586	0.432061	3.97761	3.93556	-0.43131	-1.006
FCHO2	3.19725	3.29038	3.88256	3.90937	3.37174	3.25329
FCHSD1	3.17766	3.11623	3.58706	3.86278	3.79144	3.86618
FCHSD2	3.91003	4.01636	3.2576	3.45051	2.2197	2.36546
FCRLA	5.25691	4.20957	-6.17309	-3.52222	?	-5.69922
FDCSP	8.50689	7.79136	-3.18222	-2.93781	-3.48878	-3.38329
FDFT1	6.23774	6.15554	5.83753	6.36614	5.88633	6.3946
FDPS	6.64927	6.56561	5.65019	6.0712	6.01821	6.39273
FDPSP1	1.40085	1.82799	0.929185	1.56764	0.952572	1.26395
FDPSP7	0.111005	0.13184	-0.349265	0.000201963	0.130351	0.267032
FDX1	1.23489	1.19305	2.73688	3.15984	2.8206	3.23324
FDX1L	1.78747	1.65646	1.38465	1.59317	2.65224	2.92352

FDXACB1	0.09815	0.497117	1.17363	1.68141	0.156927	1.07138
FDXR	1.51041	1.40138	1.20023	1.31748	1.67957	2.00067
FECH	3.70073	3.68563	4.16409	4.15209	3.34673	3.53169
FEM1A	1.66551	1.32714	0.637323	0.60666	1.81173	0.719262
FEM1B	5.4289	5.62426	5.33512	5.59421	5.83828	6.13444
FEM1C	4.62002	4.39357	4.48092	4.76768	4.73789	4.50639
FEN1	6.4699	6.6478	5.43163	5.97331	6.06356	6.13954
FENDRR	-4.37417	-2.6546	6.35491	6.48082	-6.64581	-3.70484
FER	5.48205	5.54032	5.16242	5.2624	5.15346	5.41027
FER1L4	-0.744656	-1.0363	3.63825	3.18119	0.373208	-1.89883
FERMT1	3.09209	3.46048	-1.05395	-0.380732	2.3026	2.47817
FERMT2	5.17282	5.21622	3.94833	4.23879	5.60912	5.66668
FERP1	0.0753683	0.356865	0.387704	1.0701	0.600755	0.82158
FEZ1	3.40421	3.55117	-2.18288	-1.15432	3.22995	3.68774
FEZ2	4.58689	4.82486	4.52986	4.90728	5.86226	6.45818
FGD1	2.28393	2.10323	2.25529	2.15798	1.43249	1.46325
FGD4	4.01945	3.94442	-0.138983	-0.353769	0.740643	0.847941
FGD5-AS1	6.16005	6.26333	5.3744	5.73705	5.55431	5.8271
FGD6	1.98042	2.08792	3.9573	3.91214	2.07103	1.95865
FGF1	-1.74441	-1.93875	1.14744	1.97866	0.293994	0.769095
FGF12	2.73138	2.88146	-3.59683	-3.78492	3.554	3.71954
FGF13	3.12857	2.86359	-0.401986	0.231137	2.86717	3.52691
FGF14	-2.97375	-2.43591	2.03133	2.74873	0.269464	-0.0383811
FGF2	5.24896	5.52657	5.10372	5.85135	6.10616	6.13858
FGF5	-0.289526	0.707154	-4.18092	-2.20128	1.17894	1.69389
FGFR1	2.68463	3.039	4.43422	4.77373	3.30547	3.47071
FGFR1OP	3.79608	4.04839	3.93496	4.11937	4.29593	3.98133
FGFR1OP2	4.39678	4.51371	4.54948	4.6676	4.15746	4.37143
FGFR3	-0.306596	-0.93902	-0.206089	-0.899119	2.04015	1.56011
FGFR4	2.56772	1.78985	-1.37581	-1.10853	-0.510371	-1.006
FGFRL1	5.70841	5.17735	3.7113	3.79657	6.68591	6.18275
FGG	-3.8896	-5.45651	4.03322	2.61228	?	-4.70296
FGGY	0.727258	0.603321	1.60441	1.83957	1.47416	1.80111
FH	5.15712	5.08539	5.04147	5.41116	5.58236	5.89775
FHDC1	4.14876	3.42285	1.68069	2.3588	2.44299	2.66398
FHL1	1.49572	1.49719	5.23801	5.55649	4.83104	5.14502
FHL2	4.10424	4.12117	4.3361	4.92253	4.21696	4.72687
FHL3	1.87459	1.64047	1.02599	1.01017	2.49705	2.07467
FHOD1	3.75404	3.81013	4.14749	4.31396	4.52027	4.59802
FHOD3	2.61976	2.68771	0.217851	0.372451	3.04238	2.75202
FHP1	0.275341	0.484035	0.220986	0.697213	1.01895	0.556964
FIBCD1	?	?	-1.53926	-1.25052	1.20393	2.01441
FIBP	4.48765	4.56919	3.79662	4.34547	3.77744	4.42642
FICD	2.89711	2.80395	2.84439	3.15562	3.09458	2.98329
FIG4	1.76038	1.74176	0.308431	0.857026	3.51211	3.8876
FIGN	4.09126	4.14263	3.39391	3.32163	6.16225	5.72268
FIGNL1	5.36126	5.55048	4.54013	4.77762	4.80064	4.77206
FILIP1	-0.632781	-0.158917	0.793829	0.570414	0.878914	0.878309
FILIP1L	3.47709	3.35414	4.4942	4.43421	5.99522	5.78863
FIP1L1	4.50597	4.56793	4.45973	4.60707	4.78516	4.57767
FIRRE	0.699116	0.980157	1.83852	1.96706	1.56281	1.794
FIS1	4.31981	4.18731	3.56467	3.94645	4.49766	5.60047

FITM2	3.90362	4.30698	3.81788	4.53412	2.8215	3.40964
FIZ1	1.55834	1.55956	1.69915	1.22214	1.93698	1.60621
FJX1	3.62622	3.59515	2.60765	2.87987	3.6537	3.40181
FKBP10	6.32847	6.07168	6.55054	6.70313	3.48944	3.38869
FKBP11	4.05079	3.96342	3.57802	3.57767	3.04878	2.79314
FKBP14	4.88635	5.02146	5.45219	5.75843	4.93168	4.99458
FKBP15	4.52924	4.53292	4.68243	5.02506	4.65261	4.45691
FKBP1A	5.94377	5.91955	6.33239	6.60753	5.46066	5.5213
FKBP1C	3.18653	3.00592	3.51978	3.96919	3.00935	3.17689
FKBP2	4.25947	4.12585	3.60438	3.62588	3.5745	3.34041
FKBP3	4.29737	4.4168	3.55909	3.82331	4.34174	4.76114
FKBP4	5.75895	5.78427	5.5876	6.0772	5.72801	5.79045
FKBP5	2.63184	2.77318	3.29423	3.58292	3.01389	2.76903
FKBP7	1.99094	1.84552	4.69145	5.01981	1.66165	2.34315
FKBP8	4.33287	3.9965	4.10187	4.35054	5.24279	5.7169
FKBP9	6.13654	6.07996	6.63254	6.86904	4.96856	4.55875
FKBP9P1	-0.0255089	0.165687	1.0314	1.13528	0.887018	-1.05512
FKBPL	2.01862	2.02903	1.53761	2.07607	1.9359	2.53647
FKRP	2.8186	2.45901	2.93282	3.03073	3.27486	3.19114
FKTN	4.27388	4.33967	4.48712	4.80704	4.92653	5.19188
FLAD1	4.2995	4.32913	3.61252	4.02431	4.28147	4.59363
FLCN	3.20471	3.15737	4.26871	4.38702	4.45986	4.10385
FLG	0.0152977	1.46106	0.0417196	0.387098	3.45702	2.81789
FLI1	-3.8896	-4.87366	4.2208	4.6818	1.48439	1.63317
FLII	4.95823	4.88033	5.59495	6.05324	6.14623	6.15087
FLJ22447	-2.22312	-3.46128	-0.935379	-0.504403	1.79061	1.51601
FLJ37453	0.9009	0.912259	0.558032	0.445854	0.384182	0.422664
FLJ42393	1.6585	1.47383	1.5847	1.14862	1.56854	0.522196
FLNA	9.02475	9.17677	9.78101	10.11	9.62971	9.79818
FLNB	7.43202	7.34553	7.72843	7.95328	9.28395	8.52222
FLNB-AS1	1.53098	1.35368	1.86375	2.09678	3.31667	2.00468
FLNC	-6.68418	-4.13839	5.49816	5.55604	-2.98535	-2.12114
FLOT1	3.98741	3.75511	5.93607	6.35211	6.39275	6.47937
FLOT2	4.06329	3.90347	3.99389	4.31533	5.25183	5.13483
FLRT1	0.855878	0.45058	0.428389	0.5635	-0.878443	-1.67189
FLRT2	-4.69535	-4.13839	5.45173	4.68477	3.16783	3.44811
FLRT3	5.13932	5.23888	1.35568	0.845438	3.6762	4.08769
FLT1	7.23652	7.12492	2.00633	2.3424	-2.41163	-3.12051
FLT3LG	-0.166036	-0.68484	0.786178	0.823122	-0.811234	-0.623932
FLVCR1	4.47397	4.51586	3.51518	3.85407	4.09624	3.71745
FLVCR1-AS1	0.50068	0.357428	-0.095919	0.248838	1.5443	1.87074
FLYWCH1	2.56772	2.49427	3.57862	3.70815	2.77224	3.15127
FLYWCH2	1.75628	1.21668	2.42751	2.49851	1.53448	2.15749
FMN1	4.14562	3.89801	4.57718	4.74681	1.24061	1.06153
FMN2	2.54638	2.33767	-1.09576	-1.20162	3.51614	3.25907
FMNL1	-2.05464	-2.46206	2.0217	2.53724	1.3899	1.14712
FMNL2	6.13424	6.10795	5.09608	5.03453	6.67237	6.2207
FMNL3	5.94371	6.14144	4.73461	4.83293	3.31742	3.20019
FMO5	0.312247	0.537236	0.610974	0.266325	0.549226	0.247587
FMR1	4.69859	4.65089	4.88188	4.91076	5.43243	5.16133
FN1	9.82102	9.33346	10.1305	10.1156	8.28505	7.63575
FN3KRP	3.85797	3.75331	3.27682	3.57872	4.56823	4.34857

FNBP1	3.28818	3.06854	3.47468	3.393	3.58048	3.18811
FNBP1L	5.468	5.66191	4.5613	4.73744	4.82903	4.65741
FNBP4	5.52159	5.43537	5.67716	5.70455	5.99656	5.27342
FNDC3A	5.93478	5.87867	5.09318	5.26946	6.4758	6.26482
FNDC3B	6.79309	6.52968	6.88526	7.06937	6.74861	6.16556
FNDC4	1.02208	0.912259	-0.757028	-0.464785	1.93217	2.71536
FNIP1	5.5186	5.23599	5.51792	5.66208	5.66725	5.59426
FNIP2	3.95555	4.03001	3.65883	3.74759	4.1773	3.7991
FNTA	5.00895	5.03017	4.24806	4.48058	4.65261	5.2456
FNTAL1	-0.0348312	0.415683	-1.05756	-0.103155	-0.0614522	0.661075
FNTB	3.92489	3.84317	1.9964	2.18437	3.17325	3.00349
FO538757.2	2.59926	2.18359	2.51702	2.84673	2.81279	2.09875
FOCAD	4.983	5.17921	4.15966	4.45912	5.70135	5.95236
FOPNL	4.02542	4.1537	4.64459	5.33307	4.49386	5.01353
FOS	1.27251	1.09945	1.05494	1.27492	-1.57243	-1.89883
FOSL1	5.0757	4.99863	3.46213	4.41376	3.23754	2.94075
FOSL2	4.86839	4.86076	5.29827	5.78636	7.49654	7.11843
FOXA1	0.207918	0.407614	2.01617	2.04094	2.02271	1.43288
FOXC1	2.86888	2.78983	1.12941	1.30061	3.0761	2.61973
FOXC2	1.07442	1.0276	-0.628724	-0.716287	-6.64581 ?	
FOXD1	1.59756	1.49341	1.35784	1.97012 ?	?	
FOXD2	3.26526	3.39824	0.945825	0.535117	-1.04532	-1.06257
FOXD2-AS1	2.19878	2.15556	1.01621	0.798133	-0.660096	-0.899103
FOXF1	-0.611409	-0.375208	5.07385	5.20518	-1.57243	-1.38442
FOXF2	-1.14419	-1.81849	3.59455	3.68558 ?	?	
FOXG1	1.6541	1.614	2.39014	2.63938	3.87399	3.69417
FOXJ2	2.88777	3.00273	3.55118	3.75536	2.41384	2.43796
FOXJ3	5.45158	5.54242	5.51865	5.77379	5.31768	5.45881
FOXK1	5.19951	4.98784	5.69298	5.53321	5.42216	4.96571
FOXK2	5.61574	5.47086	4.90902	5.04296	5.97171	5.73492
FOXL2NB	0.334442	0.419895	2.51693	2.65605 ?		-4.70296
FOXM1	6.10446	6.04958	5.98981	6.25926	5.72178	5.52604
FOXN2	5.23017	5.43044	4.50996	4.75535	4.48435	4.62195
FOXN3	5.23843	5.0712	4.65519	4.6845	6.06758	5.58149
FOXO1	2.68247	2.87255	2.35952	2.38704	3.61348	3.50274
FOXO3	5.3409	5.2352	4.61264	4.77154	6.64001	6.31836
FOXO3B	1.89955	1.74028	1.92239	2.04966	2.30162	1.53846
FOXO4	1.69755	1.34455	0.723445	0.913608	1.73632	1.68564
FOXP1	-1.02638	-1.7075	5.55246	5.74487	4.9813	4.40441
FOXP1-IT1	-1.65424	-1.41808	2.12028	1.8912	-1.20056	-2.12114
FOXP2	2.68895	2.33607 ?		-5.10387 ?		-5.69922
FOXP4	1.923	1.60865	2.39392	2.12481	1.62513	1.39136
FOXR2	1.7968	1.80397 ?	?		2.01567	1.70271
FOXRED1	4.05997	3.86245	3.97886	4.1067	3.29969	3.35399
FOXRED2	5.17318	5.12446	4.40143	4.28457	3.19301	2.96218
FPGS	2.89711	2.5623	2.9939	3.44767	3.51086	3.83439
FPGT	2.9548	3.10103	3.0102	3.18643	2.92931	3.55346
FRA10AC1	3.5583	3.46345	3.72937	3.5476	3.25706	2.72375
FRAS1	0.840145	1.3764	3.3749	2.77382	-2.41163	-1.53638
FRAT2	2.51282	2.46198	1.93025	2.03585	3.12433	3.24166
FREM1	4.45258	4.16378 ?		-6.09892	-5.65305 ?	
FREM2	6.74217	6.49841	-3.59683	-3.10759	4.97222	4.40311

FRG1	3.65709	3.64632	2.59521	2.8926	3.1239	3.54432
FRG1BP	0.379776	0.382225	-2.93107	-3.41363	1.22097	1.08553
FRG1HP	2.20541	1.90959	1.60137	1.97397	2.34393	2.32666
FRMD3	3.884	4.30698	3.12936	3.13673	4.19844	4.06642
FRMD4A	5.98687	5.93047	2.96846	2.97369	4.24632	3.52354
FRMD4B	3.53574	3.81676	3.07858	2.97599	-6.64581	-4.70296
FRMD5	5.63353	5.40827	1.98643	2.08102	5.8679	5.49234
FRMD6	5.87926	6.06193	5.0709	5.29577	5.51271	5.71639
FRMD6-AS1	1.17133	1.26512	0.045429	-0.275776	-0.184426	-0.662104
FRMD8	5.32499	5.53222	3.23222	3.15091	3.46866	3.19717
FRMPD4	2.28108	2.10703 ?		-6.09892	0.688494	0.463309
FRRS1	1.46586	1.36374	1.82773	2.01014	2.12107	2.32121
FRS2	4.26958	4.5094	3.64143	3.83954	3.96996	4.37676
FRY	-1.8406	-1.14061	5.53094	5.33701	-2.07475	-1.24695
FRYL	6.1177	6.17806	5.99511	6.10851	6.70873	6.65164
FSBP	1.58568	1.73174	1.23569	1.66815	2.38867	3.26821
FSCN1	5.0757	5.07932	3.9481	4.03793	4.2059	4.73317
FSCN1P1	1.01528	1.41843	0.229034	0.068203	0.431174	0.50174
FSD1	2.41728	2.40753	2.11571	2.27489	2.49705	2.82467
FSD1L	2.91931	2.97387	3.20019	3.19724	2.7234	2.07467
FSD2	1.2901	1.46254	-0.495514	-0.00403364	0.849983	0.1145
FSIP1	-0.0596017	0.114234	-0.598354	-0.436219	0.637833	1.4532
FSIP2	2.01569	2.17755	-0.0281751	0.237833	1.88222	1.99467
FST	2.37775	1.41371	3.22587	3.9342	-0.490197	-0.799586
FSTL1	6.15459	5.84804	7.45919	7.95066	7.37989	8.01926
FSTL3	1.7439	1.8546	1.09265	1.59165	4.15348	4.43922
FSTL5	5.00685	4.29166	-3.06858	-4.10635	-2.45576	-1.6839
FTH1	7.16674	6.8485	7.96469	8.45896	8.4477	8.63595
FTH1P10	4.07873	3.7043	4.83255	5.27365	5.28366	5.67727
FTH1P11	3.56122	3.32213	4.4362	4.8011	4.73995	5.12336
FTH1P16	0.839613	0.642443	1.42865	1.87725	1.67889	2.10406
FTH1P2	4.59609	4.1791	5.30965	5.84011	5.72183	6.09774
FTH1P20	3.96211	3.57881	4.84552	5.19941	5.25898	5.43206
FTH1P23	-0.0642099	-0.0577265	0.434423	0.55612	0.477874	0.432799
FTH1P7	4.25825	3.69022	4.82583	5.38003	5.32698	5.70057
FTH1P8	3.79743	3.47838	4.4719	5.05258	4.89864	5.51764
FTL	8.36037	8.16737	10.9831	11.7282	11.1826	11.4108
FTLP2	-1.37652	-1.41612	1.5048	2.20071	1.49555	1.7865
FTLP3	4.96673	4.69492	7.6854	8.36562	7.67115	8.26735
FTO	4.53573	4.53009	5.3188	5.55916	6.15345	6.08199
FTO-IT1	0.108397	-0.0197685	0.945825	0.902467	0.469063	0.200291
FTSJ1	4.47772	4.63189	3.20449	3.75381	3.87302	4.31148
FTSJ2	4.5134	4.53644	3.91844	4.32683	5.12474	5.28693
FTSJ3	6.08537	6.16465	5.0213	5.45747	6.99622	6.73239
FTX	3.63004	3.70085	4.82747	4.64073	4.51412	4.00986
FUBP1	6.59782	6.65704	6.67948	6.80929	6.69984	6.27376
FUBP3	4.86982	5.02448	4.31033	4.66843	5.24506	4.98676
FUCA1	3.59209	3.3637	3.61089	3.49294	3.04878	3.17593
FUCA2	-2.9975	-2.46206	4.82851	5.21876	6.51782	6.94216
FUK	2.39633	2.33483	2.2841	2.45343	2.53692	2.354
FUNDC1	2.06581	1.94657	0.431281	0.902467	1.72773	2.42258
FUNDC2	4.22554	4.08211	3.5191	3.78622	5.04278	5.6254

FURIN	5.60184	5.70112	4.669	4.8954	5.1029	5.08281
FUS	6.36698	6.26152	6.80341	6.64022	5.77688	5.50405
FUT10	3.33021	3.26167	2.7113	2.58118	3.45863	3.38074
FUT11	3.9282	4.14142	3.26269	3.57375	5.70594	5.39306
FUT4	1.80345	1.45273	-0.164687	-0.494056	1.19149	0.576348
FUT8	4.24781	4.52646	5.19136	5.07553	4.53789	5.27617
FUZ	2.42069	2.32982	1.94529	1.83331	0.423062	0.695691
FXN	1.87838	2.0007	1.35568	1.4905	1.64771	1.44307
FXR1	6.17116	6.06589	6.73075	6.84614	7.06358	6.94312
FXR2	4.74631	4.59983	4.33016	4.62066	5.01118	4.87255
FXYD3	3.85359	3.67798	-3.37468	-2.93781	-1.95932	-2.70579
FXYD5	4.86886	4.79571	4.76411	5.10791	4.25032	4.28905
FYCO1	4.60467	4.80393	4.49418	4.76767	5.07228	5.20468
FYN	3.57936	3.57607	0.517004	1.16735	3.69174	3.36475
FYTTD1	5.19459	5.44026	5.63705	6.05575	6.65425	7.19166
FZD1	5.62425	5.87337	3.35758	3.22209	2.97994	2.52212
FZD2	1.90086	1.24456	3.3653	3.55295	1.50464	1.56945
FZD3	4.96046	5.00425	0.571444	0.158078	4.79141	4.44809
FZD4	3.25441	2.86015	3.75695	3.81964	3.9539	3.97824
FZD5	2.6206	2.05681	2.02107	1.69211	3.2866	2.64644
FZD6	5.0553	5.77519	2.57804	2.74605	5.72824	6.34855
FZD7	2.88024	2.93162	5.45716	5.71254	3.21774	2.74862
FZD8	3.31773	2.96342	0.27602	-0.436219	1.74486	1.80111
FZR1	3.6138	3.49715	3.39579	3.47614	4.66386	4.71849
G0S2	?	-5.45651	2.93542	4.04725	-6.64581	?
G2E3	4.94174	4.99207	4.16066	4.27888	4.63698	4.87986
G3BP1	7.43955	7.96852	7.48227	8.08916	7.61875	8.07647
G3BP2	6.95064	6.98697	6.36267	6.69287	8.18055	8.40855
G6PC3	3.78474	3.89581	4.14403	4.2995	3.78676	4.24311
G6PD	4.77352	4.54087	6.68173	6.85655	6.43092	6.60119
GAA	3.26959	2.67159	4.28103	4.03072	1.91715	1.55071
GAB1	3.19725	3.03301	3.29828	2.96532	3.70053	3.0041
GAB2	4.64688	4.56367	1.98643	2.1904	2.31984	1.97981
GAB3	0.41996	0.859151	-1.48269	-0.860651	0.661695	1.06153
GABARAP	4.21586	4.32499	4.26903	4.76049	5.56845	6.34414
GABARAPL1	1.88591	1.82752	5.2723	5.54805	4.37519	4.3992
GABARAPL2	4.59669	4.54067	4.51518	4.79051	4.22615	4.70798
GABBR1	3.08344	2.90238	2.86067	2.72725	1.8276	1.1012
GABPA	3.76735	3.73113	4.06574	4.0138	4.31474	3.95754
GABPB1	4.68861	4.6859	4.15746	4.38507	4.19286	4.11887
GABPB1-AS1	5.63647	5.69499	4.82628	4.73741	5.54216	4.85419
GABPB2	-2.9975	-2.76144	2.38635	1.93559	2.51463	1.95865
GABRA2	-5.10914	-1.55554	7.45875	7.44035	?	?
GABRA3	2.53455	2.30204	0.0833836	-0.201784	3.37861	3.15438
GABRB3	-5.10914	-3.87578	5.0715	5.20177	?	?
GABRE	2.32053	2.33158	5.25786	5.34494	4.48051	4.50881
GABRQ	2.39105	2.28872	-2.48229	-1.86039	2.36484	2.08121
GAD1	3.05686	3.01838	-1.42826	-1.35358	1.84146	1.24753
GADD45A	3.01517	3.14079	3.06441	3.4948	2.1852	2.18812
GADD45B	1.84784	1.71211	2.52385	2.85984	3.69504	3.82661
GADD45GIP1	3.64229	3.44835	3.46483	3.79047	4.58422	4.97419
GAGE1	3.5591	3.47234	-6.17309	?	-11.1751	?

GAGE12C	2.48514	2.24436	-8.43308	-8.04317	-7.60486	?
GAGE12D	3.14461	3.08155	-8.43308	-8.04317	-7.60486	?
GAGE12E	2.60495	2.26431	-7.51197	-7.1163	-6.67315	?
GAGE12J	3.85685	3.72602	?	-6.09892	-8.5559	?
GAGE2A	5.1472	5.04941	-4.18092	-6.09892	-6.64581	?
GAK	5.41856	5.18909	3.63186	3.87985	4.73092	4.65742
GALE	4.09858	4.05383	3.56467	4.21422	4.41315	5.08444
GALK1	2.0986	2.02501	1.8609	2.22211	1.90957	2.84019
GALK2	3.18218	3.40149	3.50757	3.72039	3.11547	3.65841
GALNS	3.76444	3.53715	4.28713	4.42158	3.31697	3.13249
GALNT1	5.97752	6.03073	5.32179	5.55917	5.29099	5.63645
GALNT10	4.64077	4.77615	5.433	5.76423	6.41346	6.77
GALNT11	4.89291	4.95691	3.94664	4.2072	4.90824	5.06434
GALNT13	3.21531	3.3103	-6.17309	-6.09892	2.09793	2.45317
GALNT18	3.82447	3.77077	1.68069	1.90801	-1.53062	-1.70626
GALNT2	7.74876	7.30776	6.54955	6.46125	6.83693	5.90751
GALNT4	3.28384	3.10576	2.75713	2.86875	2.7677	2.46543
GALNT5	5.11229	4.7245	-1.93506	-1.30113	3.55158	4.03475
GALNT6	-6.68418	-5.45651	-4.18092	-3.52222	1.90577	2.33222
GALNT7	5.38204	5.51227	6.04289	6.36458	5.9606	6.3475
GALNT9	?	-4.45968	1.78836	2.64017	-5.07051	?
GALT	2.69347	2.62381	3.45892	3.6232	0.631225	0.633217
GAMT	2.16203	2.18463	2.19153	2.33827	1.77435	2.38606
GAN	5.55239	5.34731	4.63265	4.65519	5.2081	4.36875
GANAB	8.11859	8.01409	8.07018	8.20347	7.37165	7.10945
GANC	2.74368	2.47485	2.78751	3.0273	3.3748	3.68672
GAP43	-1.14419	-0.58	-6.17309	-4.52056	1.97271	3.48311
GAPDH	10.3264	10.6092	10.2873	11.003	11.1546	11.1026
GAPDHP1	6.0851	6.53873	6.30965	7.00619	7.04016	6.94583
GAPDHP33	1.45071	1.9252	-3.01245	-3.78492	-2.95876	-4.11926
GAPDHP38	0.395633	1.35118	1.07702	1.77014	1.22071	0.957699
GAPDHP40	1.39366	1.98105	1.84559	2.48402	2.62884	2.50251
GAPDHP55	1.80477	1.54842	-3.18222	-3.30006	1.24663	0.33778
GAPDHP60	1.93847	2.50841	2.34125	2.88388	2.70281	2.64525
GAPDHP61	0.799941	1.12262	1.47598	1.50702	1.29582	0.703367
GAPDHP63	0.680621	1.62667	1.29377	1.95933	1.93529	1.77263
GAPDHP65	4.51774	4.99191	4.85845	5.53256	5.76374	5.4381
GAPDHP69	-2.93383	-2.55512	0.23271	1.14516	1.4382	1.97372
GAPDHP70	-0.541259	0.0526385	-0.00109109	0.420391	0.558944	-0.560251
GAPDHP71	0.833651	1.27274	0.831788	1.19121	1.30219	0.55715
GAPDHP72	0.135929	0.776651	0.903555	1.38977	1.20722	0.853676
GAPVD1	6.34922	6.23302	5.97381	6.09053	6.17633	5.97495
GAR1	2.72299	2.62992	1.71738	1.85699	2.71908	2.677
GAREM	4.67052	4.12438	3.0517	3.18527	0.913562	0.21303
GARNL3	1.61837	1.28539	1.20884	1.22214	0.997957	0.401903
GARS	5.85359	5.9868	5.64143	6.29019	6.46844	6.587
GART	5.80666	5.90521	5.56768	6.01383	5.85948	6.25029
GAS1	2.43793	2.52016	-2.27595	-2.52305	1.59711	2.03478
GAS2L1	1.87838	1.97181	2.22164	2.6157	1.64806	1.92993
GAS2L3	5.02499	5.08355	3.55963	4.04978	4.47476	4.49295
GAS5	6.48191	6.67879	5.18988	5.55027	6.75395	6.97979
GAS5-AS1	1.2317	1.61934	0.945825	0.935626	1.26455	0.937201

GAS6	-2.05464	-1.81849	2.53346	2.83294	3.75892	4.16186
GAS7	8.45451	8.14802	0.225978	0.19507	-1.37455	-1.53638
GAS8	4.30894	4.2025	3.49142	3.73558	3.43843	3.17162
GATA2	0.108397	0.107125	0.0645242	0.367002	0.990802	0.816919
GATA3	0.567789	-0.0364475	1.31637	0.845438	0.210164	-0.31423
GATA4	-1.74441	-1.87737	1.18285	1.49854 ?	?	
GATA6	1.16711	1.45897	-0.209944	0.367002	0.477739	-0.0627204
GATAD1	4.49013	4.51799	5.50078	5.46907	4.45278	4.35127
GATAD2A	4.83668	4.79218	4.60356	4.71454	5.73147	5.36005
GATAD2B	4.73502	4.66321	4.55962	4.42935	4.41516	3.98851
GATB	3.35614	3.3177	2.91763	3.19462	3.09965	3.39453
GATC	4.69772	4.80553	4.3722	4.66724	4.56253	4.90566
GATM	-2.89067	-1.93875	-3.59683	-5.10387	3.42183	3.48311
GATS	2.24625	1.48516	2.86871	2.63597	1.65205	1.02246
GATSL2	0.866655	0.411817	1.18659	0.910088	1.0689	0.556228
GATSL3	0.628736	0.0493938	0.961697	1.1316	0.614041	1.15234
GBA	6.27168	6.09457	5.31756	5.73751	5.47319	5.76067
GBA2	4.51214	4.35044	4.12049	4.27155	6.53474	6.29892
GBAP1	1.97114	1.95592	2.13616	2.26987	1.35577	1.01381
GBAS	5.03818	4.77912	4.42657	4.4515	5.72878	5.75986
GBE1	4.55889	4.50725	6.25057	6.08653	6.55568	6.23653
GBF1	5.09085	5.14031	5.77102	5.95253	5.51273	5.45567
GBP1	2.59773	2.91641	4.90759	5.20453	4.257	4.31147
GBP2	4.1828	3.84778	5.30179	5.84019	4.5467	4.51244
GBP3	2.25804	2.47968	4.12709	4.54527	3.54304	4.04483
GBP4	-0.632781	0.137189	5.03561	5.61778	-2.85193	-4.70296
GBP5	-4.37417 ?		0.460428	1.06115	2.25857	1.38079
GCA	0.973215	1.11463	1.82213	1.48367	1.70941	1.76905
GCAT	2.3884	2.28685	2.12484	2.6225	2.89428	3.12301
GCC1	4.58153	4.666	3.82369	4.38207	4.67137	4.78613
GCC2	5.65966	5.72493	5.24962	5.45611	5.44819	5.61868
GCDH	2.16944	1.97098	2.02778	2.36765	2.74802	2.70015
GCFC2	3.67614	3.74306	2.81084	2.90654	3.86747	4.02101
GCH1	1.24348	1.20963	0.259532	0.491176	1.29974	1.20023
GCLC	3.97594	4.10985	5.86699	6.18692	4.76122	4.72373
GCLM	4.78323	5.21176	6.11712	6.75656	5.97829	6.18809
GCN1	6.96724	7.04109	6.26793	6.51413	6.93401	6.75757
GCNT1	3.2782	3.025	1.48185	2.03075	4.49704	5.01096
GCNT2	1.73974	1.88148	5.16795	5.32059	5.88367	5.62196
GCOM2	0.403475	0.585866	0.181026	1.06678	0.878576	1.66429
GCSH	3.5248	3.04127	2.71679	2.31094	3.22064	3.09475
GCSHP5	3.44534	2.86954	2.67193	2.54733	3.00388	3.62993
GDAP1	4.26237	4.51658	3.583	3.48175	3.65143	4.22106
GDAP2	5.27174	5.42398	5.01675	5.23101	6.30527	6.44017
GDE1	4.85693	4.91273	5.1333	5.58378	5.18045	5.37276
GDF11	4.69864	4.43077	4.25362	4.03068	4.10095	3.68274
GDF15	0.61388	-0.00328298	1.86635	2.32582	2.57332	2.32121
GDI1	5.1801	5.08356	5.04289	5.35928	6.37959	6.09012
GDI2	6.53593	6.5376	5.77888	6.21409	6.92197	7.48498
GDI2P2	2.04152	2.13607	0.948616	1.79477	2.16584	2.51208
GDNF	5.09368	4.77794	0.27602	-0.327299	-2.95876	-2.8983
GDNF-AS1	1.17748	1.8272	-0.790968	-0.649181	-5.65305 ?	

GDPD1	1.27251	1.15192	-0.428455	-0.201784	0.539452	0.752791
GDPD5	1.61384	1.36374	2.03082	2.36692	-4.07293 ?	
GDPGP1	1.36166	1.48589	0.861241	1.05132	1.48948	1.55071
GEM	2.26384	2.43803	2.25528	3.4206	4.23374	4.56823
GEMIN2	2.43022	2.40753	1.3479	1.71775	2.01919	3.028
GEMIN4	4.88888	4.89911	4.21948	4.43612	5.32269	5.23066
GEMIN5	5.16122	5.24019	5.1485	5.53637	5.16504	5.0515
GEMIN6	3.81761	3.72943	2.93022	3.16264	4.19689	4.75986
GEMIN7	2.67913	2.49525	2.23117	2.41547	3.15412	3.45133
GEMIN8	1.399	1.54842	0.925065	0.978694	1.48439	1.65964
GEN1	5.09738	5.08886	4.50909	4.50312	5.14019	5.09256
GET4	2.98618	2.53852	3.2735	3.30302	3.03037	2.92688
GFAP	-0.113974	-0.177293	-1.42826	-0.938639	4.49779	5.46703
GFER	2.91336	2.74083	2.77715	2.96764	2.74183	3.02866
GFM1	5.15928	5.26848	5.37297	5.76346	5.82197	5.99068
GFM2	4.38267	4.28952	4.83689	5.39431	4.55072	5.08568
GFOD1	1.64968	1.8926	2.56543	3.0661	3.13735	2.7725
GFOD2	3.51523	3.63519	3.96368	4.07729	4.87057	4.54121
GFPT1	5.90911	6.02424	5.78657	6.40392	6.13591	6.22657
GFPT2	4.20254	4.50654	2.57138	3.38903	4.72501	4.8848
GFRA1	4.97686	5.17149	5.51645	5.28082	7.43917	7.2148
GGA1	4.3002	4.1491	4.13501	4.19267	3.8235	3.38074
GGA2	5.23533	5.15829	5.03745	5.19036	5.56035	4.96747
GGA3	3.41607	3.27529	4.09959	4.18468	4.37597	4.2285
GGCT	4.74741	4.68988	4.06193	4.2939	4.82541	5.28609
GGCX	5.2906	5.4261	5.32982	5.65271	4.73179	4.91042
GGH	4.78825	4.94813	4.56467	5.0896	4.05308	4.81095
GGNBP2	5.20404	5.13613	5.5221	5.57416	5.41315	5.04816
GGPS1	3.08882	3.08019	3.42381	3.65439	3.58286	4.01096
GGT1	-5.10914	-3.65369	2.07154	2.10071	-0.43131	-0.899103
GGT7	3.88306	3.79807	3.33808	3.47048	2.59237	2.65524
GHDC	2.28678	1.93378	2.37491	2.39501	1.61585	1.8479
GHITM	6.00035	5.98269	5.63664	6.26676	6.6774	7.26181
GHR	1.34536	1.96763	-1.79072	-2.40763	-6.64581 ?	
GHRLOS	0.468531	0.479758	0.633803	0.383121	-0.415944	-1.38442
GID4	2.59555	2.77557	2.50649	2.83662	2.21007	2.55539
GID8	5.51675	5.33885	4.56382	4.52325	5.71173	5.22697
GIGYF1	4.87834	4.41903	4.30632	4.29205	4.32268	3.57869
GIGYF2	6.05934	6.06978	6.19602	6.42301	6.21326	5.81922
GIMAP2	-0.191134	0.013012	2.12484	2.48738	-2.20023	-0.848487
GIN1	1.64525	1.79457	2.49948	2.4799	2.35651	3.0178
GINM1	3.7228	3.7392	4.24054	4.47512	4.94414	5.14267
GINS1	5.45604	5.76235	4.4108	4.98128	4.14382	4.28055
GINS2	4.45108	4.46014	4.1028	4.43102	4.06742	4.2255
GINS3	2.90147	3.01264	2.68001	3.17544	3.53364	3.74226
GINS4	5.1573	5.23621	4.07045	4.44353	3.72517	3.65184
GIPC1	4.94521	4.38656	3.76776	3.88411	5.39803	5.22697
GIPC3	1.38311	1.045 ?		-5.10387	-4.33548 ?	
GIPR	-1.30642	-2.00285	0.73541	0.902467	0.643551	0.126299
GIT1	3.88305	3.67925	4.0926	4.34752	4.5947	4.35263
GIT2	4.85348	4.85856	4.51124	4.62905	4.45195	4.1291
GJA1	3.63901	4.12852	6.64783	6.40534	6.72657	6.56691

GJC1	6.24254	6.19558	5.87074	5.86377	5.44624	5.00838
GK	0.381339	0.17104	2.20868	2.48336	0.739407	0.709326
GK5	3.79377	4.03599	3.99513	4.17888	3.80728	3.48558
GKAP1	0.595628	0.6247	-0.0541032	-0.131402	-0.0308197	-0.421131
GLA	4.37171	4.58631	5.28511	5.78788	4.84352	5.2482
GLB1	5.39895	5.22283	5.09274	5.40939	5.36855	5.53589
GLB1L	1.82451	1.52019	2.37491	2.23992	1.42719	1.47324
GLCCI1	2.34315	2.18435	2.26971	1.94041	1.40201	0.594214
GLCE	5.33913	5.76054	6.02194	6.54376	3.71799	4.30311
GLDN	-0.175367	0.0918691	3.77936	4.97066	2.25559	1.64205
GLE1	4.18121	4.2358	4.22257	4.58101	4.37233	4.32937
GLG1	7.16675	7.20962	7.67421	7.93468	6.84835	6.5012
GLI1	2.62511	2.43803	0.292311	0.520618	-3.6585	-4.11926
GLI2	4.47147	4.66256	0.517004	0.430424 ?		-5.69922
GLI3	4.75523	4.42285	4.51779	4.22208	5.36067	4.84402
GLI4	0.629212	0.382745	1.87665	1.82137	1.49434	1.18601
GLIDR	0.182184	0.51925	-1.24156	-1.70413	0.838871	1.20341
GLIPR1	4.452	4.72716	5.1019	5.59849	6.62645	6.85132
GLIPR2	-0.272655	-0.734698	2.13391	2.34651	4.56971	5.02034
GLIS2	0.129942	0.102389	1.02577	1.18328	1.11038	0.0705267
GLIS3	4.05756	3.77606	4.18877	4.51653	6.28619	5.91511
GLMN	2.77868	2.9801	2.34397	2.51324	2.41652	3.30171
GLMP	5.14093	4.9513	2.937	3.04613	4.19533	4.27912
GLO1	6.19999	6.33397	5.63263	6.08687	6.73873	7.40541
GLOD4	3.80149	3.84589	4.08585	4.44355	4.48472	4.72857
GLP2R	-4.37417	-4.13839	3.35176	3.33825	-6.64581 ?	
GLRB	2.14329	2.47086	0.838911	1.14862	3.92183	3.7383
GLRX	1.46083	1.60865	3.92107	4.5202	2.50319	3.14504
GLRX2	2.60926	2.62727	1.90396	2.63265	2.39499	2.87449
GLRX3	4.43101	4.54159	4.5021	5.09019	4.89695	5.53642
GLRX3P2	2.22791	2.4431	2.4854	2.88849	2.81826	3.46319
GLRX5	4.18885	4.33605	2.74244	3.23842	4.4244	4.47538
GLS	5.94067	6.0968	6.62096	6.85065	7.41006	7.17528
GLT8D1	5.11149	5.19189	4.4749	4.66707	4.81021	5.07034
GLT8D2	0.480917	0.166625	0.945817	1.82784	1.16629	2.02123
GLTP	4.34246	4.40452	2.96336	3.30222	4.36293	4.35645
GLTPP1	0.123415	0.665247	-1.65146	-0.487498	0.336855	0.10494
GLTSCR1	0.231764	0.107125	0.925065	0.833756	0.539452	0.126299
GLTSCR1L	2.69753	2.88145	2.24695	2.49851	3.55035	3.5624
GLTSCR2	4.01223	3.73176	3.42313	3.57503	4.11232	4.34882
GLUD1	4.88288	4.95059	5.00612	5.6276	5.81223	6.31749
GLUD2	3.11437	3.09622	3.20439	3.75482	3.99483	4.50719
GLUL	4.95687	4.8173	5.76227	6.32421	2.96905	3.56241
GLYCTK	2.1713	2.02903	1.62879	1.54942	1.91336	1.71117
GLYR1	4.77564	4.80798	4.95851	5.12837	4.98397	4.70899
GM2A	4.2988	4.36529	5.25734	5.60068	3.7277	4.0212
GMCL1	3.8016	3.93725	3.12909	3.49025	3.68322	3.70478
GMDS	2.57938	2.48845	2.20881	2.37097	3.2645	3.09742
GMDS-AS1	1.17133	1.28539	2.18718	1.76772	2.07442	1.80902
GMEB1	3.20781	3.23064	2.51693	2.43035	3.83054	3.40181
GMEB2	2.94478	2.74174	2.85266	3.09085	2.8073	2.84019
GMFB	5.94289	5.87581	4.82806	5.13062	6.03798	6.56521

GMFG	-5.69162	-5.45651	1.5647	1.76157	-5.65305	-5.69922
GMIP	0.207918	0.137189	1.06446	0.924662	1.5394	1.80902
GMNN	3.96359	3.95288	3.45673	3.67253	4.56909	5.17745
GMPPA	3.36835	3.27525	3.79375	4.25201	3.84152	3.93534
GMPPB	4.80534	4.52576	4.06796	4.35526	4.5043	4.19069
GMPR	-3.2383	-2.87684	1.24279	1.81006	2.02271	1.87074
GMPR2	4.26454	4.37004	3.26356	3.66431	4.54548	5.01694
GMPS	5.70613	5.7331	6.12566	6.38216	5.97172	6.15187
GMPSP1	2.22779	2.30873	2.67422	3.18676	2.72169	2.95655
GNA11	3.96536	3.50079	4.00876	4.33618	4.8708	4.62083
GNA12	4.85407	4.4312	5.45173	5.39101	5.09082	4.43538
GNA13	5.79051	5.73221	6.02867	6.23961	6.58714	6.39396
GNAI1	3.33021	3.07243	4.39296	4.51413	3.12433	3.12934
GNAI2	6.57094	6.67428	6.06143	6.45269	7.27477	7.37463
GNAI3	6.30822	6.4893	6.13128	6.58158	7.2528	7.43533
GNAL	-0.607786	-0.0346272	2.75216	3.12076	0.26883	0.202186
GNAO1	2.57705	2.32508	1.57806	1.42259	-1.80184	-2.00571
GNAQ	4.62091	4.41462	4.47577	4.64019	4.19864	3.77663
GNAS	7.87842	7.53632	7.76367	7.73596	8.14917	7.63582
GNAZ	1.20183	1.34455	2.27182	2.64273	-1.16815	-0.497071
GNB1	6.48603	6.23342	6.49572	6.71868	7.58578	7.25781
GNB1L	0.999675	0.729183	-0.0555613	0.323981	-0.228958	0.443225
GNB2	4.77461	4.58427	4.55203	4.8063	4.86324	5.28551
GNB2L1	8.51603	8.48279	8.38678	8.89404	9.48005	9.75684
GNB3	1.18877	0.88121	0.462722	0.511569	0.0275929	-0.687574
GNB4	5.98824	6.10306	5.65401	5.9529	5.99944	5.82666
GNB5	3.81365	3.86211	3.19514	3.45927	3.55336	3.6398
GNE	4.90361	5.27015	5.17732	5.68232	5.16265	5.10947
GNG10	3.92886	4.19983	4.33024	4.82212	4.0119	4.3362
GNG11	3.68893	3.70707	5.59824	5.80555	3.5888	4.48927
GNG12	7.16131	7.39445	5.69375	6.40168	6.98534	7.3736
GNG2	4.69858	4.76295	4.69986	4.98985	2.93029	3.5003
GNG4	2.32053	2.34774	0.723445	0.845438	-2.85193	-2.8983
GNG5	4.02594	3.61255	3.96228	4.15124	4.77641	4.86494
GNL1	4.91622	4.85099	4.63646	4.88636	5.70512	5.97974
GNL2	6.28157	6.20314	4.90658	5.2808	6.13935	6.23836
GNL3	6.26599	6.21463	4.83676	5.42255	6.27541	6.58589
GNL3L	4.67867	4.70331	3.15188	3.5278	4.07185	3.96217
GNL3LP1	-0.0426708	-0.284704	0.828071	0.961534	-0.280315	-0.144817
GNPAT	4.35007	4.27777	4.36625	4.67498	4.74749	5.36875
GNPDA1	4.97644	4.93429	4.01982	4.28027	4.68066	4.95683
GNPDA2	3.82741	3.90128	3.60438	3.86306	4.23222	4.4005
GNPNAT1	4.75108	4.9678	3.44911	3.86269	5.13335	5.4966
GNPTAB	5.81218	5.95077	4.81788	4.85911	5.43046	6.00838
GNPTG	3.07194	2.9326	3.43102	3.60387	3.09117	3.18785
GNRH1	1.95558	2.1482	0.976408	0.845438	1.36764	0.615294
GNRHR	-1.52875	-0.604659	-0.455418	-0.177939	1.50965	1.54125
GNS	7.65534	7.59488	7.06463	7.37638	7.14499	7.18539
GOLGA1	3.33029	3.25623	3.24384	3.38406	4.05068	4.11147
GOLGA2	4.60354	4.51394	4.50472	4.90309	4.92088	4.54831
GOLGA2P10	2.17873	1.78578	2.24326	1.98306	1.32889	-0.00923969
GOLGA2P5	0.569909	0.552702	0.425685	0.576783	2.02171	1.02136

GOLGA2P7	4.78036	4.73282	5.49342	5.30759	4.705	3.72595
GOLGA3	6.2622	6.25227	5.89364	6.24544	6.71177	6.39719
GOLGA4	7.88796	7.86885	6.87557	7.24348	7.75059	7.60868
GOLGA5	4.17513	4.33724	3.55288	4.001	4.263	4.77407
GOLGA6L10	1.03005	1.07469	0.265557	0.462199	-3.07319	-3.74558
GOLGA6L4	0.0548901	0.422621	2.83206	2.46858	0.654436	-0.0964634
GOLGA6L5P	-2.26866	-1.99954	2.92227	2.93789	1.13206	-0.444614
GOLGA6L9	3.52549	3.29662	4.19838	3.77485	3.32217	2.02871
GOLGA7	4.52492	4.52583	3.89062	4.34752	4.45409	4.75374
GOLGA7B	2.53694	3.32662	-4.59508	-2.30078	-4.07293	-4.11926
GOLGA8A	4.79145	4.51046	1.59519	1.08828	3.56256	2.15611
GOLGA8B	5.38249	5.23515	5.33644	5.08823	5.39615	4.20722
GOLGA8N	-0.597516	-0.74946	1.98925	1.7721	-0.761695	-1.42354
GOLGB1	6.66394	6.70699	7.2318	7.53292	6.80797	6.96305
GOLIM4	5.16897	5.35733	5.38443	5.61951	5.65848	5.52389
GOLM1	6.61757	6.59568	4.82349	5.23768	5.09748	5.15047
GOLPH3	4.64631	4.54628	4.4431	4.8256	5.67201	5.58618
GOLPH3L	3.52492	3.51301	3.22375	3.46672	3.86521	4.13877
GOLT1B	5.0335	5.24924	4.8228	5.44142	4.69448	5.18961
GON4L	6.05246	5.99681	5.59719	5.71557	5.95857	5.57657
GOPC	4.51232	4.52826	4.87888	5.14744	5.49569	5.63378
GORAB	4.22572	4.20516	2.83329	3.15796	3.47155	3.66615
GORASP1	4.14945	4.02699	3.8499	4.22209	3.77847	3.882
GORASP2	4.78019	4.66321	4.71959	5.0371	4.80625	4.84979
GOSR1	4.16913	4.31805	4.47557	4.80779	4.94664	5.01932
GOSR2	4.90351	4.81703	4.42176	4.86667	5.17129	5.2986
GOT1	3.81266	3.67414	4.34199	4.95118	4.4926	4.6596
GOT2	5.48515	5.50004	5.65212	6.05041	6.67115	6.96239
GOT2P2	0.0559314	-0.440295	-0.375995	0.139221	0.886652	1.02127
GOT2P3	0.171937	0.013012	-0.568612	-0.086319	0.427241	0.443129
GPAA1	4.37973	4.1891	4.36625	4.5287	4.46639	4.86218
GPALPP1	3.6138	3.67414	4.28815	4.45437	3.73736	3.90246
GPAM	4.39763	4.22277	3.98765	4.17657	4.25107	4.44557
GPANK1	3.34503	3.37012	2.6854	3.04759	2.96722	3.50703
GPATCH1	3.01001	2.82948	3.06678	3.34445	3.07101	2.65962
GPATCH11	4.31139	4.3011	3.47649	3.83791	4.58337	4.78104
GPATCH2	4.38175	4.60459	4.23432	4.50219	4.86618	4.83147
GPATCH2L	6.04112	6.02974	5.30832	5.43999	6.03728	5.75014
GPATCH3	2.80257	2.76598	1.68022	2.01471	2.41117	2.41742
GPATCH4	5.44374	5.50259	4.00908	4.43451	5.64711	5.26007
GPATCH8	5.00569	4.94123	5.39531	5.25585	5.19027	4.91625
GPBP1	5.36127	5.42133	5.78585	6.01529	5.77327	5.97275
GPBP1L1	5.95848	5.94656	6.26565	6.43858	6.32392	6.13411
GPC1	5.21492	5.16285	5.71695	5.74682	4.91002	5.02459
GPC4	-4.69535	-6.45019	2.58467	1.81601	6.51647	5.70743
GPC6	5.5881	5.26326	-1.42826	-1.64896	5.45922	5.90294
GPCPD1	4.12772	4.00319	5.76105	5.53562	4.59076	4.54155
GPD1L	4.12429	4.20068	2.87987	3.16963	5.04226	5.27552
GPD2	6.47582	6.32893	6.01389	6.37311	6.68462	6.87487
GPER1	0.0559314	-0.46266	-1.13883	-0.899117	1.46385	1.0747
GPHN	3.44302	3.45453	0.225978	-0.108685	2.3292	2.26485
GPI	6.69294	6.77525	7.79471	8.11169	8.13413	7.64547

GPKOW	3.70179	3.5692	3.13841	3.5689	2.66388	2.61523
GPM6A	-1.30642	-1.105	-3.37468	-2.93781	7.14472	7.36171
GPM6B	5.32292	4.77792	0.102	-0.131402	6.17338	6.01096
GPN1	4.36833	4.4458	3.34251	3.82589	5.5052	6.35415
GPN2	4.44493	4.42056	3.56635	3.92871	4.83153	4.64311
GPN3	3.55623	3.55198	2.74893	3.13274	3.12353	3.75687
GPNMB	1.12752	0.491443	4.18716	4.98193	4.25183	4.7619
GPR1	-4.69535	-6.45019	-1.53926	-0.751045	1.12765	1.51249
GPR107	5.48299	5.56469	5.53327	5.88622	5.56277	5.80848
GPR108	3.53334	3.30037	3.32428	3.72722	3.84946	4.06807
GPR132	5.54406	5.45375	-5.1783	-4.52056	-2.75246	-4.70296
GPR135	2.65216	2.84882	2.84369	2.68368	2.61901	2.38986
GPR137	3.29891	3.24583	2.42245	2.75471	2.41111	2.1394
GPR137B	2.44048	2.55119	1.5714	1.92462	1.82353	2.42773
GPR137C	2.46584	2.67159	1.78805	1.76157	1.41655	1.53173
GPR153	2.04241	1.52587	1.68069	1.6394	0.568596	-0.121607
GPR155	3.91746	3.78862	2.21737	2.03585	3.36482	2.46822
GPR156	0.973406	1.045	-0.252626	0.158078	0.932205	0.315752
GPR157	2.24345	2.17742	2.40145	2.67254	2.95624	2.66181
GPR158	3.53557	3.4524	-1.37581	-1.15432	0.0263185	-0.151977
GPR160	0.375735	0.775632	0.102	0.120113	1.19772	0.380851
GPR161	5.0537	5.06611	4.62463	4.89188	4.35928	4.33357
GPR162	0.800836	0.645757	1.3479	1.40691	2.18833	2.16989
GPR173	1.7314	2.01291	2.27592	1.94648	1.04018	1.12623
GPR176	5.7232	5.82543	3.98639	4.93076	3.84551	4.2975
GPR18	1.18361	1.08411	1.13846	0.935633	0.351018	-0.38461
GPR180	5.24708	5.27059	4.40237	4.5724	5.09749	5.17592
GPR183	0.701877	0.479758	1.23438	1.12005	0.234612	-0.421131
GPR22	2.13699	2.20606	1.8772	1.81601	1.07446	0.816919
GPR3	2.18663	2.31525	-0.482896	-0.301307	0.679613	0.965773
GPR37	3.58747	2.96761	1.81651	1.96801	?	?
GPR39	3.25017	3.31333	2.78258	3.56643	3.5211	4.00718
GPR52	0.54893	0.756391	0.225978	-0.0212303	-0.572647	-1.12145
GPR55	-0.13626	0.414038	0.445928	1.06287	-6.64581	?
GPR63	1.91933	1.84552	1.30034	1.24877	-4.33548	-5.69922
GPR75	1.68033	1.46793	0.966283	0.685662	1.34536	0.0483059
GPR89A	3.74346	3.65228	4.04681	4.15458	3.59695	3.96797
GPR89B	3.44097	3.49415	3.46347	3.49829	3.22403	3.38794
GPRASP1	1.87838	2.0802	2.92501	2.49587	2.25857	1.67702
GPRASP2	1.01876	1.15181	2.99802	3.10342	3.26133	3.41998
GPRC5A	7.07187	7.19027	4.38727	4.92665	2.21315	1.62424
GPRC5B	4.59617	4.63305	3.43863	4.00168	3.7743	3.82613
GPRIN1	3.14141	3.13284	3.5675	3.78729	3.5006	3.54122
GPRIN3	1.70183	1.72701	2.2002	2.7398	-4.33548	-2.8983
GPS1	5.7244	5.50691	4.85245	5.01951	6.13588	6.071
GPS2	3.77062	3.83145	4.18406	4.50338	5.02353	4.94428
GPS2P1	0.34334	0.545781	0.471187	1.14912	1.52997	1.87123
GPSM1	2.80394	2.39986	3.24419	3.15505	1.85498	1.65831
GPSM2	6.24062	6.46323	4.75798	4.74915	5.35873	5.15524
GPSM3	-1.41331	-1.87737	1.90397	2.03585	-1.30241	-1.12145
GPT2	3.6047	3.48112	4.02836	4.3465	5.00681	4.58904
GPX1	4.48956	4.50366	2.74808	3.38261	5.0179	6.30627

GPX1P1	1.56267	1.60845	-0.0600558	0.269895	1.92424	3.29627
GPX3	0.121214	-0.0533185	3.89328	4.04977	-0.615709	-1.31405
GPX4	4.44367	4.09652	3.98138	4.3258	5.32089	5.7069
GPX7	3.07406	2.64045	1.30838	0.946514	?	?
GPX8	3.77055	3.76836	5.52468	5.99149	4.88513	5.62923
GRAMD1A	3.06081	2.68945	3.60926	3.69853	3.98083	3.64423
GRAMD1C	0.459064	1.32336	1.42907	1.85456	-0.285108	0.752791
GRAMD3	3.91311	3.51168	2.74154	3.14359	4.43554	4.73475
GRAMD4	3.42763	3.26848	2.83608	2.85407	1.72343	1.78517
GRB10	6.10283	6.33986	3.46933	4.05858	7.23257	7.19772
GRB2	5.07613	5.19581	5.14905	5.6473	6.50939	6.83851
GREB1	2.78676	2.76598	1.99641	1.92462	-3.19956	-3.38329
GREB1L	0.62293	0.746683	1.92503	1.47617	0.405873	0.315752
GREM2	1.03569	0.920918	0.871828	-0.938639	-0.959604	-1.38442
GRHL1	2.37508	1.90787	-0.138983	-0.493926	1.14064	-0.280287
GRHPR	4.46648	4.39122	4.73123	5.02236	5.7098	5.85679
GRIK2	5.13733	4.94707	-2.18288	-1.52346	4.56428	4.48927
GRIK4	?	-6.45019	2.27592	2.42257	-0.572647	-0.247123
GRIN2A	0.752201	0.076436	1.59787	0.711424	-1.26766	-1.38442
GRIN3A	0.41996	0.479758	0.488988	0.605504	0.293994	-0.0627204
GRINA	4.72665	4.49569	5.27948	5.51276	5.28403	5.03812
GRIP1	1.77482	1.59526	-4.16641	-3.10759	0.0211243	-0.0887103
GRIP2	-0.94121	-1.13776	2.13935	2.37633	0.213378	-1.18215
GRIPAP1	4.44811	4.36608	3.98264	4.04345	3.33969	3.08771
GRK4	1.07577	1.02102	0.208904	0.334214	1.28225	1.1637
GRK5	2.26672	2.62992	2.93542	3.03838	1.13416	1.0747
GRK6	3.36121	3.20204	3.8859	4.08506	4.55037	4.4816
GRK6P1	-0.359059	-0.43467	0.0930733	0.261134	0.628186	0.200291
GRM8	3.89329	3.71825	-4.18092	-3.78492	-3.6585	-5.69922
GRN	5.07735	4.82717	5.93477	6.04757	4.82852	4.79909
GRPEL1	4.38306	4.26421	4.13388	4.53682	5.21234	4.97187
GRPEL2	4.63233	4.47195	4.18747	4.29228	5.36282	5.05641
GRSF1	5.83813	5.98397	4.70521	5.13671	6.29806	6.55948
GRWD1	4.69102	4.55743	3.93929	4.20516	5.18791	5.56878
GS1-124K5.11	1.52013	1.64571	2.32429	2.32164	2.0332	1.04823
GS1-124K5.12	0.278306	0.338955	0.473506	0.491176	0.529601	0.363799
GS1-257G1.1	1.04207	0.782467	1.49592	1.38027	1.87216	1.69998
GS1-309P15.4	1.15061	1.23949	1.38307	1.35826	2.11114	1.39602
GS1-358P8.4	2.39633	2.35095	4.0477	4.15091	3.5528	3.00754
GS1-44D20.1	3.2123	3.27496	3.49308	3.88538	4.4649	4.46157
GSAP	2.47585	2.79455	2.41268	2.8827	2.80934	3.44049
GSDMB	0.480917	0.0291348	2.09262	2.16732	-0.217212	-1.006
GSDMD	1.28965	1.15925	4.0247	4.25859	3.19144	3.60617
GSE1	3.93119	3.85344	4.85538	4.85838	4.63832	4.33904
GSG2	3.12747	3.14449	2.96623	3.40885	3.15508	3.03476
GSK3A	3.53217	3.38518	2.38501	2.77939	3.82347	3.9311
GSK3B	5.01515	5.07437	5.07975	5.20292	4.94097	5.17973
GSKIP	3.32382	3.38134	2.44584	2.74917	3.46897	4.28622
GSN	5.82387	5.89939	4.81143	5.08344	4.19939	4.50752
GSN-AS1	2.64912	2.83109	2.26639	2.28436	1.21231	0.789971
GSPT1	6.23072	6.08645	6.38015	6.6639	6.75146	6.41804
GSPT2	3.10182	3.18821	2.4164	2.70175	2.79091	2.73209

GSR	5.33263	5.46161	6.32376	6.73311	5.76513	5.86122
GSS	4.80424	4.63976	4.38822	4.68395	4.33757	4.70479
GSTA4	0.930336	0.727049	-2.37543	-1.71605	2.52953	3.55303
GSTCD	3.98281	4.26834	3.33891	3.70403	3.49424	3.94134
GSTK1	4.33709	4.42817	4.74498	5.03582	5.36343	5.7274
GSTM2	-0.151452	-0.493896	1.90783	1.91503	2.04766	1.80543
GSTM3	3.5429	3.34934	4.75678	5.23577	5.30502	5.57724
GSTM4	2.20716	2.08267	1.88491	2.43664	2.8188	3.01212
GSTO1	3.65957	3.68436	4.27592	4.80031	4.50018	5.78712
GSTP1	6.68485	6.57949	6.61411	6.81747	5.22462	5.61801
GSTT2B	2.37121	2.48158	-1.44476	-1.54198	1.48802	2.07667
GSTZ1	2.38042	1.81795	2.19587	1.8454	2.42451	1.81687
GTDC1	3.87991	3.93696	1.7234	1.70499	4.4469	4.62978
GTF2A1	5.06172	5.21442	4.3939	4.67692	5.22919	4.98357
GTF2A2	4.03731	3.95289	3.37298	3.63095	4.48816	4.73829
GTF2B	3.93027	3.87477	3.37298	3.75071	3.79604	4.62419
GTF2E1	3.19574	3.18821	3.43853	3.86013	3.65597	3.54358
GTF2E2	3.57004	3.60054	3.42935	3.75999	3.6389	3.8932
GTF2F1	4.96492	4.93964	4.60926	4.98525	5.78705	5.66668
GTF2F2	3.84492	3.94123	3.44766	4.02174	4.91947	5.51002
GTF2H1	5.03726	5.11082	4.20017	4.59594	5.28722	5.74448
GTF2H2	3.73593	3.79013	4.11228	4.18501	4.22836	4.04536
GTF2H2B	2.68065	2.69981	2.78619	3.22233	2.92908	3.14889
GTF2H2C	4.27644	4.36505	4.42902	4.50809	5.22899	5.57703
GTF2H3	4.50853	4.52937	3.8221	4.24872	4.50143	4.67483
GTF2H4	2.37064	2.11689	2.32339	2.2865	2.6895	2.52341
GTF2H5	3.21381	3.22541	1.90396	2.16732	3.98894	5.02799
GTF2I	6.60077	6.53277	7.02126	7.00119	7.00002	6.89473
GTF2IP1	5.56918	5.28367	5.36648	5.44034	5.31395	4.89943
GTF2IP4	5.95973	5.39154	5.1693	5.17852	5.30313	4.86616
GTF2IRD1	2.79678	2.55397	3.89996	3.97198	3.52332	3.39657
GTF2IRD2	1.28437	1.10298	1.84014	1.43359	2.74924	2.07758
GTF2IRD2B	1.32335	0.936938	1.9711	1.92856	2.73352	2.37376
GTF3A	4.98462	4.9422	4.46297	4.79735	5.17924	5.51229
GTF3C1	6.50058	6.40983	6.73738	6.82153	6.08976	5.65741
GTF3C2	5.21994	5.09554	4.1247	4.3996	5.35108	5.27605
GTF3C2-AS1	0.558875	0.679919	-0.00412623	0.00626211	0.824857	0.301236
GTF3C3	4.47851	4.47954	4.30311	4.40786	4.72278	5.18656
GTF3C4	5.59174	5.66662	4.92869	5.19569	5.44187	5.47581
GTF3C5	4.52431	4.40056	4.07386	4.33307	4.33615	4.19867
GTF3C6	3.71404	3.87838	2.2841	3.01207	4.87567	5.50658
GTPBP1	4.46541	4.35475	4.42747	4.5796	4.00903	3.49694
GTPBP10	3.68348	3.9054	4.0252	4.33928	4.03916	4.38964
GTPBP2	4.75676	4.63519	4.17073	4.52598	5.28658	5.082
GTPBP3	2.33986	2.08407	2.16964	2.17196	2.26452	1.97279
GTPBP4	5.94447	6.12931	4.20303	4.73753	5.86366	5.91115
GTPBP6	1.93757	1.71211	2.60439	2.9052	1.27048	1.81687
GTPBP8	2.38009	2.58599	2.11526	2.4021	2.70329	3.2531
GTSE1	4.8555	4.79748	4.75605	5.00034	3.94886	3.86693
GUCD1	5.86266	5.69997	5.56179	6.07071	5.22676	5.27139
GUCY1A2	-2.79121	-3.13966	-0.428455	0.213217	4.15346	4.63646
GUCY1B3	-6.68418	-6.45019	-3.59683	-4.10635	1.58765	1.60621

GUF1	4.15188	4.05481	3.60438	3.77381	4.66948	4.51366
GUK1	4.32882	4.24709	4.76264	5.20178	5.56127	6.38901
GULP1	5.18775	5.32953	2.57138	2.22658	6.20598	6.35296
GUSB	4.96355	4.85562	4.92956	4.93758	4.33191	3.71534
GUSBP1	2.06307	2.02539	2.20224	2.11803	1.93073	1.75588
GUSBP11	2.20418	1.99001	2.88692	2.41521	1.12228	1.08031
GUSBP3	0.17218	0.414146	1.35838	0.973435	1.16964	0.278603
GUSBP9	1.6526	1.29415	1.98684	1.59878	1.28202	1.35997
GVINP1	0.595618	0.697098	1.18285	1.12005	?	?
GVQW1	0.647075	0.494538	0.863114	0.693186	1.05892	-0.543078
GXYLT1	5.82912	5.91949	5.05546	5.15091	4.76752	4.96747
GXYLT2	4.43341	4.30616	4.22374	4.03965	4.63831	4.16519
GYG1	3.29244	3.53008	3.59784	4.26837	3.93214	4.5624
GYG2	?	?	2.23435	1.81006	-2.26731	-4.11926
GYPC	1.75628	1.50874	3.52038	3.75536	-6.64581	-4.70296
GYS1	3.63297	3.43197	4.1248	4.21197	4.88804	4.10867
GZF1	4.82152	4.80627	4.76775	4.86844	4.15427	4.0515
H1F0	5.01043	4.66256	6.08091	6.14294	3.62624	2.83633
H1FX	2.96813	2.50415	3.46634	3.56506	2.02892	2.34214
H2AFJ	1.14646	0.357428	?	-6.09892	0.839605	0.769095
H2AFV	5.85969	5.94028	5.93043	6.21057	5.87359	5.91273
H2AFX	4.23606	3.99246	4.0656	4.10305	4.29023	4.28621
H2AFY	6.97113	7.08924	7.30515	7.48288	6.84831	6.678
H2AFZ	7.38734	7.43903	6.9374	7.45031	7.57473	8.28434
H2AFZP3	1.88804	2.04198	1.41445	1.87719	2.08691	2.92444
H2BFS	1.05846	0.924624	-1.91141	-1.49178	0.154531	0.982269
H3F3A	5.83579	5.67948	5.58206	5.5487	6.44838	6.36412
H3F3AP4	5.20546	4.89807	4.65898	4.77615	5.74458	6.17661
H3F3B	8.21181	8.10461	7.44015	7.64758	8.65413	8.72191
H3F3C	2.67512	2.59687	1.51123	1.87731	3.06088	2.89116
H6PD	4.47521	4.05185	5.5182	5.38301	3.66161	3.42257
HABP4	2.86239	2.93482	2.27017	2.69352	2.72504	2.94437
HACD1	2.5441	2.31525	3.04289	3.48364	-0.10558	0.422664
HACD2	4.71401	4.75752	4.38348	4.94916	6.01497	6.50789
HACD3	6.6328	6.56039	6.87427	7.01057	6.78081	6.73361
HACD4	1.82842	2.08021	1.60441	1.36695	2.20389	1.84021
HACE1	3.12588	3.18101	3.63666	3.5968	3.40039	3.21215
HACL1	3.99186	4.01874	2.50907	2.70289	3.55685	4.21569
HADH	3.43918	3.34932	3.39955	3.62926	2.27928	2.90062
HADHA	6.4628	6.45957	5.85005	6.27163	5.99335	6.27532
HADHAP1	1.26884	1.48209	1.25302	1.50243	1.04321	1.06652
HADHB	5.22757	5.3364	4.73978	5.11275	5.2135	5.58502
HAGH	3.05079	2.97387	2.66511	3.0933	2.80947	2.74862
HAGHL	1.52275	1.11725	0.948534	1.04437	1.06223	1.99059
HAGLR	3.05329	2.83754	1.76404	1.8028	2.34806	2.43204
HAND2	2.81821	2.75805	-3.85951	-13.2477	-4.65668	-5.69922
HAPLN1	5.91952	5.27819	-4.18092	-3.52222	-5.65305	-4.11926
HAPLN3	2.82252	3.00068	0.371169	0.857026	?	-3.38329
HARBI1	1.09102	1.0306	1.05664	1.23164	1.52158	1.66267
HARS	5.85401	5.85274	5.67902	5.94085	6.40654	6.3708
HARS2	4.69043	4.80573	4.79821	5.08706	4.92673	5.33166
HAS2	2.95222	3.21541	0.636673	-0.630687	2.52286	1.99746

HAS3	2.24052	1.85459	0.996447	0.82198	3.18047	2.81686
HAT1	5.57502	5.5747	4.93607	5.33153	5.75198	6.47136
HAUS1	3.47584	3.56203	2.80775	3.22433	2.31412	3.42741
HAUS2	3.89989	4.0139	3.26563	3.88411	4.21466	4.42256
HAUS3	4.04996	4.1633	3.6798	3.8809	3.92066	4.00327
HAUS4	3.24927	2.99929	2.14942	2.32223	2.54013	3.04619
HAUS5	2.69754	2.70207	3.16742	3.29844	2.73202	2.55069
HAUS6	6.03293	5.93961	5.02726	5.10606	6.48291	6.46435
HAUS6P1	3.20003	3.17718	2.18294	2.44296	3.67747	3.98781
HAUS6P3	-0.161889	0.0155689	-0.63627	-0.414047	0.73914	0.496708
HAUS7	2.41467	2.12586	2.55793	2.64948	2.73625	2.86315
HAUS8	2.66287	2.6587	2.38255	2.74916	2.92278	2.65085
HAX1	5.24281	5.25484	4.56613	5.06395	5.24534	5.92242
HBEGF	2.69539	2.8881	2.68067	2.98129	3.52457	2.25909
HBP1	4.94304	4.99594	4.70276	5.01022	4.5081	4.94809
HBS1L	4.93481	5.04692	4.98451	5.15208	5.99856	6.44207
HCCS	2.47835	1.67161	2.66198	2.24434	3.55523	2.84404
HCFC1	5.11791	4.77552	4.96049	4.69771	5.15868	5.20541
HCFC1R1	2.88472	2.80037	3.13443	3.5835	3.72604	4.0644
HCFC2	3.27677	3.35094	3.33021	3.47425	3.58286	3.93714
HCG18	4.79752	4.8658	4.957	5.04461	5.52252	5.43224
HCG4P5	0.514723	0.642443	2.48338	2.9417	2.2814	2.936
HCN2	-1.14812	-2.22414	1.78471	0.987517	1.75907	0.307953
HCN3	1.37778	1.18826	1.62387	1.46864	1.08794	0.315752
HCP5	-0.132739	-0.970721	-2.5977	-1.86039	1.2881	1.54125
HDAC1	5.80076	5.57332	5.62664	5.9111	6.28333	6.23873
HDAC10	1.30441	0.962808	2.16697	2.26317	1.20807	1.27979
HDAC11	-1.17328	-1.7075	1.72383	1.70499	0.558944	0.633217
HDAC1P2	-0.129081	-0.0876659	-0.300715	-0.436219	0.82357	0.100762
HDAC2	5.58023	5.59987	5.408	5.52326	6.31427	6.65165
HDAC3	4.54714	4.46454	4.94704	5.13195	5.43699	5.42706
HDAC4	4.08963	3.97282	3.44766	3.48364	3.26004	3.12618
HDAC5	3.1211	2.78746	3.07858	3.4342	3.46768	3.48804
HDAC6	3.67378	3.38733	3.8221	3.88127	3.29389	2.93354
HDAC7	3.48316	3.24398	4.46802	4.47121	4.41951	4.16961
HDAC8	3.59323	3.67287	3.66353	3.67744	3.65822	3.62421
HDAC9	5.18927	5.29119	4.21414	4.34444	6.18516	6.31391
HDDC2	3.53932	3.77316	3.03565	3.46671	4.30331	5.32601
HDDC3	2.52264	2.57949	1.46354	1.73689	1.54823	1.835
HDGF	8.09032	8.24492	7.03031	7.58226	7.91528	8.05299
HDGFRP2	4.16974	3.83292	4.13276	4.24211	4.42715	4.01609
HDGFRP3	5.42115	5.15605	5.55161	5.51341	5.11145	4.77508
HDHD1	1.68033	1.554	2.13391	2.27489	2.65711	2.84788
HDHD2	4.21364	4.26947	3.36091	3.40558	3.34354	3.39775
HDHD3	1.6585	1.61934	0.945825	1.28354	-0.852709	-0.121607
HDLBP	8.52296	8.39787	8.38507	8.76352	8.62258	8.505
HDX	2.12749	2.25142	2.64621	2.69208	3.01388	3.35938
HEATR1	6.78385	6.83864	5.92449	6.29217	7.13503	7.1052
HEATR3	3.57588	3.6509	3.44766	3.68883	4.76385	5.18506
HEATR5A	4.48715	4.816	3.23674	3.73531	4.22692	4.69693
HEATR5B	5.07281	5.05038	4.3459	4.48456	5.23033	5.08851
HEATR6	4.13776	4.34127	3.60111	3.99707	4.36482	4.40571

HEBP1	1.50553	1.06077	3.50073	3.57065	1.18523	0.847941
HEBP2	3.29103	3.3429	3.46394	3.7507	4.47988	4.75168
HECA	3.34314	3.33467	2.90313	3.10522	4.48275	4.21201
HECTD1	7.09006	7.10625	6.24327	6.44119	6.96495	6.99481
HECTD2	3.44558	3.459	2.8305	2.86846	4.20927	4.27627
HECTD3	3.31354	3.10131	4.27999	4.38202	4.12103	3.87072
HECTD4	5.40286	5.3147	5.57805	5.45636	5.10035	4.49388
HECW1	-0.970891	-1.00314	4.12761	4.46641	-1.95932	-0.979598
HECW2	1.49571	1.67674	2.0549	2.37903	-2.48959	-1.18284
HEG1	5.84543	6.05925	8.15875	8.23864	6.7375	6.79933
HEIH	1.24787	1.21186	0.925149	1.21883	2.12004	2.56107
HELB	4.49411	4.63089	3.19793	3.28349	4.05768	3.90041
HELLPAR	7.20605	7.49078	3.70484	3.77524	5.80014	5.43991
HELLS	6.11578	6.1793	6.39504	6.33381	5.84004	5.75168
HELQ	2.68247	2.71956	2.39014	2.58815	2.56612	2.78514
HELZ	5.85936	5.68595	6.01521	5.82853	6.51362	6.01182
HELZ2	3.07736	2.96342	3.69146	3.62757	4.75807	4.99458
HEMK1	3.74697	3.71458	3.91714	3.99314	3.44298	3.38074
HERC1	6.56196	6.57461	6.7236	6.8383	6.76908	6.51999
HERC2	6.33535	6.26261	6.34416	6.41825	6.15977	5.75208
HERC2P2	5.78513	5.63698	6.21703	6.27276	5.50856	4.49315
HERC2P3	4.19449	4.03995	2.97984	3.07768	2.22247	1.31188
HERC2P9	4.50287	4.36196	4.63251	4.58077	3.95142	3.29819
HERC3	3.70321	3.6857	4.16851	4.48642	4.11202	4.68587
HERC4	5.23312	5.25247	4.74114	4.89916	5.48274	5.53107
HERC5	3.32052	3.45154	1.5913	1.83957	3.42582	3.73207
HERC6	1.52496	1.65613	1.31637	1.46864	2.89428	2.89692
HERPUD1	3.89803	3.82602	4.90989	5.40489	5.67758	5.86171
HERPUD2	3.71031	3.34774	3.76485	3.57066	3.5959	3.15748
HES1	2.84589	2.96343	1.49599	1.48367	3.25707	3.35127
HES6	1.91933	1.47971	0.120378	0.176693	1.38413	1.92267
HEXA	5.65552	5.44728	5.59455	5.79483	5.38987	5.3252
HEXB	6.04307	5.9065	6.27538	6.85397	6.27381	6.70284
HEXDC	1.03569	0.707154	1.79378	1.77384	1.65713	1.60621
HEXIM1	4.28875	4.33134	4.48606	4.72219	5.83144	5.77231
HEXIM2	1.47882	1.14107	1.66355	1.69073	0.743808	1.5296
HEY1	0.640852	0.467989	2.56803	2.81302	4.63431	3.98503
HEY2	1.9303	2.03303	-4.59508	-4.52056	?	?
HFE	2.47835	2.98217	3.86496	4.38802	3.81339	4.33904
HGF	3.2168	3.51157	-1.48269	-3.78492	-5.65305	?
HGH1	2.87078	2.84323	2.93283	3.29844	3.52705	3.68988
HGS	5.42641	5.12174	4.52148	4.94847	5.45843	5.27208
HGSNAT	4.30671	4.33499	3.9902	3.90816	2.87451	2.82183
HHAT	2.29811	2.3413	0.558032	0.46112	1.51465	1.97279
HHIP	2.70173	4.05875	-1.09576	-1.10853	-1.17713	-1.06257
HHIPL2	-2.89067	-2.55512	3.28	3.7351	-0.510371	-0.183001
HIAT1	5.08677	5.29869	4.54269	5.00165	5.2682	5.48125
HIATL1	5.07697	5.18119	4.64185	5.23532	4.99919	5.63631
HIBADH	4.85405	4.66321	4.79518	5.26999	3.59472	4.11505
HIBCH	3.04744	3.29196	3.42566	3.43614	3.72811	4.0038
HIC2	2.61843	2.31853	2.72196	2.55652	1.7191	0.541297
HIF1A	8.8209	8.63075	7.42915	7.75552	9.36972	9.09028

HIF1AN	5.1292	5.23422	5.36911	5.67068	5.78098	5.83679
HIGD1A	4.62614	4.59784	4.48157	4.88759	5.34582	6.04764
HIGD1AP1	-0.509374	-0.476579	-0.370639	-0.19731	0.506724	0.886449
HIGD2A	2.95836	2.78598	3.44569	3.75119	4.34848	4.50853
HILPDA	3.14404	3.28016	2.44893	2.77973	2.84979	2.38506
HINFP	3.01688	3.05088	3.19152	3.28564	3.05394	2.85935
HINT1	6.50455	6.46154	6.48065	6.94537	6.56392	7.42819
HINT1P1	1.13487	1.44505	1.37231	1.76494	1.75928	1.80111
HINT2	2.80077	2.59786	2.76777	3.11035	3.16942	3.71745
HINT3	2.80276	2.85004	2.09727	2.6225	4.00768	4.11026
HIP1	5.17245	5.19847	5.53196	5.59681	4.22462	3.79511
HIP1R	2.57706	2.49136	2.5273	2.60199	2.51213	1.99375
HIPK1	5.82364	6.0209	6.02408	6.44626	6.0141	5.8832
HIPK2	7.4072	6.30325	8.51538	7.74489	7.20518	5.9299
HIPK3	6.03881	5.53501	5.23327	5.12897	6.14442	5.77759
HIRA	4.37536	4.25126	3.81889	3.88889	3.4808	3.22203
HIRIP3	3.05839	2.80218	2.35183	2.39441	2.77949	1.93271
HIST1H1B	7.16412	7.15105	7.26987	7.56159	6.79898	7.84426
HIST1H1C	7.1011	7.3787	7.58227	8.14818	7.27916	7.98458
HIST1H1D	5.50609	5.6424	6.5277	6.92441	6.17138	6.97166
HIST1H1E	6.21043	6.20691	6.78944	7.00409	6.22842	7.07709
HIST1H2AB	3.89803	3.82833	3.96749	4.24762	3.5888	5.33904
HIST1H2AC	5.6192	5.56748	5.82769	6.1783	5.87908	6.61937
HIST1H2AD	5.24306	5.15278	5.25993	5.63726	4.94166	6.04063
HIST1H2AE	5.65791	5.67029	5.22639	5.52779	4.87447	5.46445
HIST1H2AG	6.16316	6.22889	5.84782	6.23379	5.79449	6.72503
HIST1H2AH	5.51548	5.49834	2.04532	2.2562	5.00699	6.0205
HIST1H2AI	5.94074	5.90888	6.13247	6.44248	5.71333	6.53166
HIST1H2AJ	3.64051	3.61811	3.99268	4.11483	2.05436	2.77715
HIST1H2AK	1.9633	1.68045	1.23194	0.676755	1.61928	2.23353
HIST1H2AL	2.73347	2.41981	2.88795	3.1835	3.3537	4.13091
HIST1H2AM	4.43646	4.32489	4.70002	5.15675	4.02699	4.79887
HIST1H2BB	5.33447	5.40307	3.85138	4.29457	4.64115	5.49815
HIST1H2BC	5.61895	5.58179	5.66659	6.26592	5.60175	5.95217
HIST1H2BD	6.33153	6.31915	6.15742	6.47595	6.24959	6.62472
HIST1H2BE	4.2364	4.07128	2.30731	2.63551	3.17761	3.21527
HIST1H2BF	4.48082	4.27045	4.54591	4.90404	4.53215	4.57388
HIST1H2BG	6.4281	6.59547	6.29372	6.76787	5.06717	5.57231
HIST1H2BH	5.08063	4.91598	5.48933	5.8555	5.37035	5.70373
HIST1H2BI	0.908313	0.912259	5.57136	6.09504	5.23629	5.89644
HIST1H2BJ	5.322	5.49102	4.65097	5.46575	5.04851	5.58558
HIST1H2BK	5.80032	5.88108	2.3598	2.74148	5.37973	5.85912
HIST1H2BL	4.58022	4.60318	4.74573	5.07845	4.21798	4.72731
HIST1H2BM	2.8284	2.6352	2.0785	2.23549	-0.267833	0.100762
HIST1H2BN	4.89523	4.76375	4.82247	5.16967	4.95012	5.35302
HIST1H2BO	4.91681	5.00925	5.13668	5.65111	4.77918	5.11644
HIST1H3A	3.33984	3.12961	2.65569	3.14856	2.96175	3.97978
HIST1H3B	5.80399	5.76355	5.66509	6.14471	5.73361	6.88854
HIST1H3C	5.75546	5.69006	5.55202	6.01787	5.9312	6.9135
HIST1H3D	4.68731	4.41366	4.46663	4.59074	4.45473	5.70055
HIST1H3E	0.159022	-0.214764	0.416483	0.266325	0.416602	0.922708
HIST1H3F	4.87456	4.58768	4.80018	5.20858	5.0803	6.56065

HIST1H3G	-3.69722	-3.65369	5.97223	6.49641	5.68064	7.10926
HIST1H3H	5.15967	5.00373	5.29675	5.55694	5.21236	6.28904
HIST1H3I	4.18356	4.03301	4.16962	4.39101	3.27927	3.93353
HIST1H3J	-2.99749	-4.13838	4.37472	4.77693	2.30853	2.77804
HIST1H4A	3.44431	3.03501	3.41267	3.52598	3.40712	4.20467
HIST1H4B	5.38605	5.44554	5.23221	5.90064	5.96013	7.18638
HIST1H4C	6.32362	6.40905	5.85641	6.40982	6.73025	7.93499
HIST1H4D	5.2562	5.25096	5.24483	5.5305	5.69145	6.64724
HIST1H4E	5.58487	5.60256	5.68683	6.06765	5.6931	6.4373
HIST1H4H	5.95375	6.05284	5.60763	6.0664	6.02968	6.56211
HIST1H4I	3.07736	2.49136	-1.13882	-1.25052	2.74058	3.67266
HIST1H4J	5.69039	5.48717	5.11795	5.58626	?	?
HIST1H4K	2.04733	1.96574	1.71157	2.51791	?	?
HIST1H4L	4.6279	4.44027	4.27077	4.6301	-6.64581	?
HIST2H2AA3	6.73333	6.58133	6.29159	6.64358	5.79964	6.54364
HIST2H2AA4	6.00753	5.98946	5.485	5.95491	4.90008	5.47077
HIST2H2AB	4.20178	4.00578	3.36915	3.52416	2.59473	3.61295
HIST2H2AC	4.64363	4.73826	4.60167	5.01765	4.29296	5.23479
HIST2H2BB	1.93145	1.6529	-2.09954	-2.38244	1.33705	0.772418
HIST2H2BE	6.43669	6.45048	6.1954	6.60794	5.91206	6.00617
HIST2H2BF	6.84009	6.66558	6.05102	6.26949	5.60023	5.65632
HIST2H3A	5.43015	5.27555	4.7618	5.39981	4.39529	5.42441
HIST2H3C	5.32939	5.04935	4.90318	5.20223	4.26105	5.6785
HIST2H3D	3.54726	2.81173	3.63742	3.78048	2.76992	4.42451
HIST2H4A	6.96337	6.55565	6.59319	7.10679	6.33729	7.47491
HIST2H4B	5.58836	5.67692	5.41837	5.98704	5.29242	5.9715
HIST3H2A	1.33439	0.592522	-6.17309	-5.85885	1.40582	2.09421
HIST3H2BB	3.30595	2.7042	-4.18092	-3.78492	2.62384	2.56545
HIST4H4	2.30936	1.78985	3.02349	3.28349	3.32695	3.99373
HIVEP1	4.57528	4.57126	4.95856	5.06982	4.37587	4.09903
HIVEP2	5.06002	5.05778	4.84717	4.92182	5.01479	4.5959
HIVEP3	4.39631	4.35092	2.61739	2.54582	3.93959	4.06807
HJURP	6.40774	6.36745	6.56314	6.83407	5.74191	5.80555
HK1	5.90567	5.80836	6.42754	6.91734	7.66665	7.21439
HK2	5.02811	4.3725	4.05428	4.38334	5.24006	3.73113
HK2P1	1.45458	0.840105	0.717491	0.655104	1.50085	0.211249
HKR1	4.25146	4.13335	3.33218	3.29632	4.0548	3.27628
HLA-A	7.55668	7.50164	8.6987	8.86319	8.1722	8.44009
HLA-B	5.03664	4.92177	8.23718	8.378	6.53937	6.6631
HLA-C	4.13413	3.97787	7.9851	8.15479	5.93724	5.91259
HLA-DMA	4.98609	4.15828	1.37111	1.93559	-3.07415	-1.70626
HLA-DMB	3.65517	3.11081	1.26773	2.11519	?	?
HLA-DOA	3.57122	2.3413	-3.85951	-2.78591	-6.64581	-5.69922
HLA-DPA1	6.85929	6.44384	2.6109	3.05859	-6.64581	?
HLA-DPB1	5.26004	4.63585	0.770744	1.48367	-5.07051	?
HLA-DQA1	5.08299	3.95064	-6.17309	-6.09892	?	?
HLA-DQB1	4.99348	4.32456	-6.17309	-6.09892	?	?
HLA-DRA	8.10682	7.36908	-0.861345	-0.380732	-3.85089	-5.69922
HLA-DRB1	7.35274	6.86844	-1.42826	-1.48579	-5.65305	-4.11926
HLA-DRB5	4.32077	3.78091	-4.59508	-3.43715	?	?
HLA-E	5.44338	5.3343	6.61275	6.87191	6.43396	6.71827
HLA-F	2.30198	1.67991	3.4489	3.4779	2.2372	2.32992

HLA-F-AS1	0.629399	0.640427	1.13749	1.49703	0.696118	0.471583
HLA-H	-1.79002	-0.935252	0.854307	1.47639	-0.00209776	0.301108
HLA-L	1.77839	1.67203	1.46358	1.37688	2.00134	1.41481
HLCS	3.7675	3.78863	4.30331	4.28562	3.767	3.64202
HLF	0.0289651	0.0918691	-1.229	-1.10853	0.871134	1.12623
HLTF	5.41334	5.57197	6.42448	6.59725	6.27024	6.68614
HM13	6.40465	6.27149	6.19936	6.54658	6.07083	6.0486
HMBOX1	3.91102	3.76837	4.00259	3.86845	3.39226	3.03138
HMBS	3.40026	3.15003	3.66197	3.90379	3.00147	3.3101
HMCES	3.84395	3.97698	3.40332	3.82928	3.10788	3.27913
HMCN1	8.2533	8.36873	5.99077	4.84536	6.85746	6.88643
HMG20A	5.00424	4.89632	4.86493	4.84449	4.9576	4.76495
HMG20B	5.77713	5.11552	3.42895	3.62012	4.19348	4.0212
HMGA1	7.67885	7.45396	5.19058	5.73332	6.79691	6.57181
HMGA1P2	3.35043	3.16344	1.30444	1.44717	2.39999	2.06306
HMGA1P8	1.67708	1.67224	-0.0256087	0.164181	1.15134	0.445441
HMGA2	6.50711	6.29357	3.30246	3.72233	4.53476	4.71324
HMGB1	7.58471	7.5242	6.84001	7.01321	6.83312	7.06878
HMGB1P5	6.35899	6.36206	5.56367	5.71309	5.57388	6.0378
HMGB2	6.68728	6.43837	5.93151	6.04945	6.60428	6.38006
HMGB3	4.73431	4.5455	4.14341	4.20402	4.93547	4.93724
HMGB3P6	0.00396197	0.223435	0.0163965	0.0980826	0.63671	0.440357
HMGCL	1.91933	1.76119	3.22587	3.44577	2.92841	3.33221
HMGCR	7.37451	7.18059	6.18033	6.46246	6.01849	6.43887
HMGCS1	5.17756	5.18843	5.34678	5.80984	5.3221	6.23959
HMGN1	5.41878	5.45009	6.45033	6.57505	5.98687	6.27729
HMGN1P38	2.28743	2.29149	3.32448	3.55168	3.19182	3.32272
HMGN1P4	-0.0114149	0.180021	1.17646	1.09432	1.25525	0.710957
HMGN2	7.04192	6.68336	6.33727	6.41115	7.18269	7.45427
HMGN2P4	1.79561	1.48578	1.35653	1.26116	2.06681	1.82836
HMGN2P5	5.53484	5.16388	4.96125	4.90527	5.72676	5.52309
HMGN3	4.77451	4.78636	3.23983	3.41003	4.6508	4.67017
HMGN3-AS1	0.988856	0.600184	0.109106	0.202299	0.749903	0.0788147
HMGN4	4.09208	4.24967	3.79947	4.17888	4.9754	5.53466
HMGXB3	5.27904	5.25465	5.6659	5.82929	6.00463	5.8811
HMGXB4	4.99745	4.95131	4.39579	4.43226	3.90955	3.73
HMHA1	1.62738	0.929533	1.23438	1.51326	2.27928	2.28766
HMMR	4.47004	4.58275	5.16559	5.82479	5.18971	6.01177
HMOX1	2.08557	1.40138	3.61737	4.09818	5.33403	5.33219
HMOX2	3.4505	3.65933	3.48536	3.85407	3.3565	3.71113
HN1	4.86316	4.79277	5.03866	5.54937	6.46992	6.66886
HN1L	6.38791	6.6079	5.5091	5.90537	6.02178	5.92255
HNMT	0.71885	0.265185	3.07151	3.43614	4.48434	5.88807
HNRNPA0	5.64742	5.57983	4.96749	5.07418	5.30474	4.9199
HNRNPA1	7.54546	7.44018	6.45265	6.72188	7.22075	6.93512
HNRNPA1L2	4.28753	4.20438	3.24445	3.56954	3.90959	3.82822
HNRNPA1P10	4.47841	4.33324	3.00886	3.65313	4.22421	3.80595
HNRNPA1P16	0.613598	0.342589	0.784537	0.685662	0.172692	-1.31405
HNRNPA1P4	0.675617	0.741825	-0.405853	-0.361478	0.0816669	0.0812851
HNRNPA1P48	7.68722	7.52595	6.31546	6.61275	7.36469	6.90209
HNRNPA1P49	-0.0550876	-0.00328298	0.259532	0.0410467	0.939663	-0.577229
HNRNPA1P7	7.3236	7.21154	6.28389	6.42321	6.89211	6.91624

HNRNPA2B1	8.75404	8.64058	8.52954	8.53674	8.63243	7.9558
HNRNPA3	6.26841	6.1913	7.21907	7.04364	6.38803	6.20341
HNRNPA3P10	0.931955	0.815477	0.827949	1.04801	1.34025	0.0803575
HNRNPA3P5	1.08519	1.017	1.4774	1.77276	1.59198	0.486405
HNRNPA3P6	4.86247	4.6569	5.3476	5.26023	5.0886	4.8999
HNRNPAB	5.83454	5.83569	5.71278	6.11074	6.30202	6.28368
HNRNPABP1	0.490837	0.737496	0.782333	1.12963	1.10205	0.686433
HNRNPC	7.94643	7.97183	7.16551	7.3438	8.02244	8.36421
HNRNPCL1	0.346395	0.460952	-0.492026	-0.512164	0.178594	0.82881
HNRNPCP2	5.7959	5.84679	4.92284	5.1716	5.91489	6.37002
HNRNPCP3	0.537604	0.908037	0.115313	-0.00298658	1.04967	0.76562
HNRNPCP4	0.614173	0.888235	-0.145273	0.169104	0.674171	1.34948
HNRNPD	5.71902	5.59269	5.60823	5.41111	5.45138	5.22623
HNRNPDL	6.92558	7.00425	6.56319	6.72844	7.05041	7.01695
HNRNPF	6.98484	7.05605	5.976	6.31007	7.13409	7.29435
HNRNPH1	8.39421	8.36622	8.38202	8.51789	8.75393	8.06734
HNRNPH1P3	1.86513	2.21255	1.70682	2.14961	2.1154	1.52739
HNRNPH2	5.60155	5.44928	5.21209	5.40072	6.17151	5.98033
HNRNPH3	5.27342	5.24083	5.17114	5.25995	5.11518	4.44551
HNRNPK	7.88671	7.81835	7.30585	7.60135	8.05641	8.20288
HNRNPKP1	1.55898	1.54133	1.1488	1.28706	1.79942	1.84267
HNRNPKP2	3.01486	3.17071	2.46167	2.92545	3.21228	3.14535
HNRNPKP4	5.41129	5.38333	4.77985	5.16352	5.55516	5.69194
HNRNPL	6.5049	6.49043	6.19309	6.36119	6.7199	6.52515
HNRNPLL	4.59495	4.61996	4.40331	4.73194	4.71852	5.03643
HNRNPLP1	0.0806304	0.427145	-0.467071	-0.045293	0.278794	-0.178865
HNRNPLP2	4.88489	4.97656	4.40201	4.76342	5.02948	4.96559
HNRNPM	6.35442	6.29596	5.88624	6.04027	6.39682	5.81241
HNRNPR	7.43047	7.46437	6.80596	6.92609	7.31501	6.97992
HNRNPRP1	3.24977	3.21431	2.52169	2.81064	3.30786	2.77042
HNRNPU	8.45677	8.44056	8.07841	8.2784	8.619	8.34346
HNRNPU-AS1	4.93901	4.99687	5.3741	5.3132	5.05562	4.43595
HNRNPUL1	6.55559	6.44675	6.14064	6.2509	6.49465	6.30534
HNRNPUL2	5.21153	5.04113	5.0066	5.06806	4.85983	4.50736
HNRNPUL2-BSCL2	4.34966	4.2129	3.90209	4.02319	4.11333	3.30367
HNRNPUP1	3.19632	3.19923	2.55458	2.88984	3.4773	2.56556
HOMER1	3.69538	3.87254	4.02958	3.70175	3.89619	3.74244
HOMER2	3.71031	3.62196	2.79087	2.75537	0.961801	0.650921
HOMER3	2.83841	2.69931	2.6725	2.93731	4.56283	4.95875
HOMEZ	0.863225	0.920918	2.35565	2.20407	0.714804	0.483199
HOOK2	1.61384	1.41984	0.531958	0.913608	1.59711	1.02127
HOOK3	5.8495	5.96941	5.47333	5.47848	5.21313	5.20541
HOTAIR	-0.816197	-0.707737	1.74132	1.85699	-2.33767	-2.53597
HOTAIRM1	-0.00686333	1.02594	1.15638	1.85677	-0.660096	0.35026
HOXA-AS2	1.13386	1.21668	0.959206	0.563685	-2.74516	-4.70296
HOXA1	0.684702	0.559609	0.120378	0.605504	-0.551587	-0.458602
HOXA10	2.73175	2.25991	-0.69145	-0.823182	?	?
HOXA2	0.345419	0.166625	0.816542	0.786061	-2.07475	-2.24661
HOXA3	1.05586	0.395227	0.723445	0.0410467	-3.85089	?
HOXB-AS3	1.01774	0.824597	-1.9199	-2.62764	?	?
HOXB3	1.97223	1.60754	1.25445	0.896861	3.11118	2.76497
HOXB4	-0.0381159	0.120962	0.651224	0.86078	-1.25135	-1.1782

HOXB6	2.47575	2.3732	-0.499551	-0.60591	-0.530833	-0.662104
HOXB7	3.54528	3.50581	-5.1783	-5.10387	3.09958	3.38869
HOXB9	3.03054	2.39515	-1.42826	-1.15432	-0.121008	-0.38461
HOXC10	2.43022	2.38266	2.04532	2.12001	2.5221	2.78756
HOXC11	1.54413	1.66647	0.544485	0.902467	-6.64581	-5.69922
HOXC13	1.68444	1.24877	1.54923	1.27766	1.91279	1.40019
HOXC4	0.917019	1.0518	0.655416	0.660062	0.790972	1.11596
HOXC6	1.68423	1.8609	0.943284	0.879729	1.22319	1.31548
HOXC8	1.4199	1.33161	-0.0134653	0.020584	-1.23372	-2.24661
HOXC9	1.5292	1.58158	-0.117291	-0.716287	0.420703	-0.0404257
HOXD10	1.26628	0.888734	3.25599	2.71723	3.63975	2.82008
HOXD11	0.744618	0.428239	2.07698	1.81101	2.78107	2.50847
HOXD13	2.29246	2.35415	0.431281	0.619178	-0.285108	0.293394
HOXD3	-0.244316	-0.0905587	-0.649141	-0.669003	0.661786	0.758619
HOXD4	0.433483	0.408842	-0.00867934	0.289315	0.570142	0.334809
HOXD8	2.18664	2.16653	1.94578	2.03075	2.25857	3.12934
HOXD9	1.58636	1.47971	1.46757	0.924662	1.80732	1.40185
HP1BP3	6.40778	6.64683	6.82102	7.10411	6.74135	6.72817
HPCAL1	4.88399	4.80277	5.08383	5.52823	4.68951	4.4647
HPRT1	2.01519	2.22717	3.08093	3.36691	4.09957	4.40181
HPS1	-6.68418	-6.45019	3.10403	3.50956	3.33969	3.33768
HPS3	3.68853	3.70666	5.88339	6.20761	4.60111	4.7946
HPS4	4.52344	4.5609	4.51577	4.68314	3.91957	3.64194
HPS5	5.59065	6.06021	4.00011	4.49849	4.62798	5.02205
HPS6	3.24637	3.14634	3.00136	3.36894	3.15987	3.74037
HPSE	1.68033	2.21313	2.05967	3.1835	3.01388	3.48064
HR	3.68355	4.30698	4.10534	4.13553	2.55887	2.33222
HRAS	3.64522	3.58018	3.13163	3.58814	2.73844	2.66398
HRCT1	-0.865939	-1.7075	-0.325375	-0.464785	4.46057	4.27627
HRH1	3.4895	3.71083	3.26768	4.13554	3.31267	3.94074
HRNR	-6.68418	-5.45651	0.544485	0.833756	2.22845	1.1637
HRSP12	1.90457	1.97181	2.52039	3.10063	2.35093	3.07465
HS1BP3	3.24783	3.08019	2.53073	2.94917	3.43376	3.39657
HS2ST1	5.84107	5.77622	5.93541	6.08474	5.70417	5.71938
HS3ST3A1	-4.45013	-4.45968	-1.36986	-1.39086	4.59417	4.49159
HS3ST3B1	-5.51808	?	3.30809	3.31259	5.42677	5.21373
HS6ST1	3.90537	3.69716	3.8475	3.70149	3.2603	2.70269
HS6ST2	?	?	-2.72315	-2.40763	4.28659	4.58446
HS6ST3	-5.10914	-5.45651	2.70218	3.0153	?	?
HSBP1	6.47148	6.42814	5.53875	5.87524	6.16618	6.65681
HSBP1L1	1.30097	1.30538	2.39392	2.94375	1.67511	1.58794
HSBP1P2	0.288831	0.636061	-0.0491196	-0.0657432	0.429803	0.61884
HSCB	1.14135	1.045	1.21235	1.23082	1.52049	1.83627
HSD11B1	-5.10914	-5.45651	1.54444	2.46483	?	?
HSD11B1L	0.205668	0.137189	0.401532	0.47387	1.14422	1.94304
HSD17B1	1.59686	1.15292	1.60897	1.6719	2.17647	1.53742
HSD17B10	4.56946	4.56919	3.48182	3.91353	4.14139	4.53406
HSD17B11	4.34395	4.29616	2.93543	3.19724	1.97271	2.91168
HSD17B12	4.59082	4.53105	3.49421	3.72569	6.03638	6.08506
HSD17B4	5.63813	5.71043	5.50337	5.77402	5.45101	5.61516
HSD17B7	3.63615	3.64223	2.79802	3.2073	2.84486	3.25387
HSD3BP5	2.33353	2.46728	-2.27595	-1.02108	-1.10542	-0.662104

HSDL1	4.29385	4.2514	4.62866	4.84754	4.08956	3.72791
HSDL2	4.14718	4.22802	4.2152	4.4883	4.27705	4.49784
HSF1	3.04877	3.01315	3.80169	3.98021	3.83423	4.06657
HSF2	3.27963	3.09926	3.16943	3.27056	3.63993	3.83438
HSF4	-0.0789568	-0.448296	1.90887	1.6043	0.0450515	-1.24187
HSP90AA1	9.48092	9.3091	8.39713	8.57225	9.71332	9.9952
HSP90AA2P	5.74039	5.6188	4.62769	4.87823	5.97651	6.19503
HSP90AB1	9.43429	9.38321	9.05271	9.33374	10.1117	9.88336
HSP90AB2P	1.71902	2.01803	1.31311	1.75322	2.54127	1.97702
HSP90AB3P	5.96219	6.00456	5.6651	5.91415	6.67535	6.31993
HSP90B1	10.0964	10.0032	9.01036	9.35154	8.84314	9.07211
HSP90B2P	5.28987	5.44307	4.3864	4.84377	4.10066	3.93263
HSP90B3P	4.53498	4.78614	3.60688	4.11348	3.47823	3.28405
HSPA12A	3.87929	3.88809	2.75312	2.93557	-1.65987	-2.24661
HSPA13	5.91447	6.02423	6.13275	6.55227	5.54822	6.08688
HSPA14	4.37237	4.44779	3.78802	4.20178	4.40106	4.90245
HSPA1A	5.19513	5.00578	5.89435	6.06121	2.91607	2.85005
HSPA1B	5.60423	5.38422	6.89508	7.15056	6.804	6.59064
HSPA4	7.31863	7.32756	7.01995	7.37104	7.39558	7.72718
HSPA4L	5.18875	5.49038	1.68422	1.55906	4.88914	4.75095
HSPA5	8.82057	8.57582	8.48513	8.74424	8.07889	8.06319
HSPA8	10.2571	10.1979	9.96671	10.3808	10.2615	10.5841
HSPA8P1	4.17201	4.22676	2.39186	2.84985	4.21671	4.55562
HSPA8P5	3.0637	3.31924	2.67343	3.22613	2.88561	3.12145
HSPA8P7	3.17562	3.15824	2.84525	3.50887	3.10445	3.69971
HSPA8P8	3.37142	3.3654	3.11931	3.49453	3.39025	3.61988
HSPA8P9	1.71181	1.95667	2.10797	2.53488	1.50665	1.79837
HSPA9	7.72441	7.70407	7.6139	8.10159	8.09963	8.15483
HSPA9P1	3.97272	3.98864	3.78955	4.39329	4.40366	4.18667
HSPB1	4.55815	4.2744	4.99874	5.3868	5.45591	5.62427
HSPB11	3.32744	3.3429	3.90261	4.30162	3.52332	4.33357
HSPB1P2	-0.590352	-0.56876	0.294194	0.563685	0.804054	0.463298
HSPB8	2.41468	2.66387	3.08561	3.66101	3.23451	3.34857
HSPBAP1	2.48084	2.47675	1.69303	1.73669	1.63434	2.14505
HSPBP1	3.00828	2.95079	2.02107	2.43035	3.5745	3.60391
HSPD1	8.14999	7.97408	7.29968	7.57406	8.82138	8.88429
HSPD1P1	5.49293	5.42358	4.68816	4.95013	6.22752	6.14649
HSPD1P11	0.586404	0.395227	2.7909	2.57066	0.790972	-0.31423
HSPD1P5	0.103545	0.135338	-0.968483	0.000461589	0.719587	0.413832
HSPD1P6	0.672471	0.81004	-0.152059	0.139902	1.44356	1.4025
HSPE1	5.46105	5.20397	4.66358	4.84488	6.21593	6.5649
HSPE1-MOB4	0.628363	0.27066	0.295123	-0.794918	-0.468433	2.33189
HSPE1P2	3.24746	2.8816	2.46455	2.54566	3.88552	4.22735
HSPE1P3	2.14171	1.8573	1.29124	1.54711	2.63318	3.24155
HSPG2	6.54632	6.19119	5.12652	4.65843	0.159978	0.633217
HSPH1	6.41257	6.42876	6.68856	7.10423	7.03271	7.13585
HTATSF1	4.22055	4.31195	4.97256	5.25309	5.81814	5.80652
HTN1	1.53936	1.89473	?	-6.09892	-6.64581	?
HTR2B	1.11453	1.15798	2.115	1.86268	0.498608	0.863209
HTR7	3.18792	3.37005	0.0645242	1.27492	4.33683	4.35533
HTR7P1	1.17838	0.727049	1.93544	1.71138	-0.373808	-1.006
HTRA1	5.74022	5.39396	2.98891	3.1224	3.87593	4.41741

HTRA2	3.95337	3.79596	3.54521	3.77964	3.55429	3.88006
HTRA3	?	?	1.46757	1.34243	-1.41201	-2.12114
HTT	5.69564	5.62325	5.80838	5.85656	6.49445	6.2471
HUS1	3.43918	3.54837	3.12026	3.26621	3.72662	3.84018
HUWE1	7.86083	7.82842	7.29062	7.36055	7.34627	7.0736
HYAL2	5.42275	5.34572	3.80232	3.83076	4.80982	5.16519
HYAL3	2.4223	2.67351	0.25753	1.09899	0.778267	0.805994
HYI	4.90141	4.85161	3.45635	3.81994	1.56815	1.56467
HYKK	0.863225	0.318612	0.584741	0.761609	-0.374735	-0.31423
HYLS1	2.15581	2.16653	0.371169	0.810106	0.69732	1.11352
HYOU1	7.84626	7.62969	7.51276	7.75314	7.02131	6.67804
HYPK	2.43031	2.66272	2.76156	2.71438	3.07474	2.54474
IAH1	3.83689	3.76339	3.12923	3.35356	3.60991	4.02545
IARS	7.06476	7.16228	6.43781	6.98391	7.0535	7.19319
IARS2	6.3643	6.45189	5.58092	6.01916	6.53857	6.58099
IBA57	1.85169	1.554	1.61093	1.43037	2.13412	1.80111
IBTK	6.02929	5.90402	5.20341	5.41129	6.11879	6.29257
ICA1	1.98744	2.27188	-6.17309	?	-3.48878	-4.11926
ICA1L	1.52526	1.62764	2.16444	2.04344	1.07686	0.626084
ICAM1	8.10741	7.94011	2.73282	2.87433	2.1517	1.50278
ICAM3	0.699925	0.940708	0.628522	1.08157	0.620202	1.67178
ICE1	6.58255	6.57675	6.22214	6.39399	6.66146	6.6183
ICE2	4.38369	4.47967	4.57795	4.70002	5.16296	5.42126
ICK	3.68029	3.6365	3.12254	3.7193	5.9553	6.0962
ICMT	6.12707	6.20715	5.80231	6.34673	6.38119	6.61129
ICOSLG	0.61388	1.19542	2.84439	2.82782	-3.07415	-2.24661
ICT1	2.59785	2.68182	3.12254	3.74292	3.88851	4.79213
ID1	3.67487	3.8086	4.5722	5.23546	5.13657	6.18734
ID2	-0.395122	-0.0876674	1.5913	2.20407	4.11448	5.7971
ID3	2.56538	2.23761	3.56802	3.93146	5.21465	6.31632
ID4	2.43536	2.55397	-1.86109	-2.30078	2.97269	3.72165
IDE	5.41567	5.48734	5.27743	5.66145	5.62278	5.88945
IDH1	5.49167	5.37515	6.4751	6.95675	5.88995	6.39332
IDH2	3.86221	3.16103	5.36192	5.72881	2.34813	1.55071
IDH3A	4.84631	4.79613	4.49601	4.82968	5.60955	5.709
IDH3B	5.0608	5.08742	5.20178	5.70373	5.07312	5.21064
IDH3G	3.51973	3.2882	3.60784	3.9551	4.41035	4.48167
IDI1	5.31979	5.46847	4.25437	4.86337	5.18647	6.20083
IDI2-AS1	0.14702	0.0802074	-0.903255	-0.37916	-0.0953903	1.09099
IDS	4.65682	5.45263	5.06405	6.15629	6.63526	7.18163
IER2	2.88777	3.10891	2.82211	3.08593	3.43244	3.59249
IER3	7.1279	6.56825	2.67755	2.93466	2.81816	1.05645
IER3IP1	5.15188	5.26888	4.10172	4.54177	3.69968	4.25768
IER5	4.02714	4.13706	4.87446	4.91769	2.95808	2.98329
IER5L	1.02208	0.635262	1.44014	1.89682	3.29077	3.83972
IFFO1	3.58517	3.23412	3.46394	3.63769	2.72772	2.41224
IFFO2	3.97553	4.3468	3.73086	4.16557	7.21016	6.75617
IFI16	6.38174	6.28933	5.47021	5.81746	5.44134	5.75629
IFI27	-1.94365	-2.00285	-1.229	-2.10822	3.6158	3.25618
IFI27L1	2.07902	1.9925	1.39395	1.83372	1.96176	2.44812
IFI27L2	2.41207	2.33483	1.27595	1.87988	1.5394	2.87073
IFI30	2.22414	2.09268	4.49466	5.04609	0.402231	1.38276

IFI35	1.68897	1.85006	3.30833	3.77382	3.18047	3.59249
IFI44	2.30937	2.33158	4.55033	4.4686	6.15762	6.37743
IFI44L	-0.69886	-0.847889	4.16298	3.42645	7.10838	6.89596
IFI6	1.92665	1.80864	3.63985	3.77381	5.65059	4.59707
IFIH1	2.59325	2.93162	3.41266	3.56183	3.95807	3.95862
IFIT1	-0.223192	0.237698	3.27591	3.82926	5.44882	6.36748
IFIT2	0.41996	0.223769	2.42012	2.91908	3.4298	4.59021
IFIT3	1.48583	1.89912	3.43303	3.82928	4.09874	4.64863
IFIT5	3.15423	3.3103	3.74425	4.20405	4.89331	5.40897
IFITM1	0.278853	-0.783519	3.43018	3.42473	4.76852	4.08496
IFITM10	-1.99808	-2.06994	3.06371	3.74306	3.23442	2.72115
IFITM2	-0.323933	-2.03057	3.24449	3.44026	1.89951	1.97405
IFITM3	5.87544	5.42399	6.43791	6.73333	4.95881	4.7573
IFNAR1	5.05078	5.25139	5.32871	5.47001	4.93214	5.10946
IFNAR2	3.21597	2.84147	4.25675	3.62955	1.92811	1.49015
IFNE	0.529821	0.831845	0.945825	0.535117	-0.356358	0.247587
IFNGR1	3.71453	3.36687	4.93345	4.94715	4.24508	4.15436
IFNGR2	5.23231	5.748	4.37721	4.62526	3.51826	3.8726
IFRD1	4.83034	4.86806	5.68567	6.12628	5.86103	5.78513
IFRD2	4.88401	4.82241	3.8349	4.30027	4.90538	4.93029
IFT122	2.6575	2.57789	3.41936	3.70499	3.72623	3.47147
IFT140	2.11286	1.91656	2.76315	2.53364	2.13417	1.60621
IFT172	3.85359	3.92193	3.08327	3.21985	4.16384	3.83825
IFT20	3.02427	2.98406	2.93702	3.15625	3.83246	4.50277
IFT22	3.23756	3.05876	3.18717	3.43614	3.23905	3.64201
IFT27	1.43024	0.912259	4.13162	3.98657	-0.551587	-1.006
IFT43	1.96634	1.95115	1.33222	1.6394	2.07796	2.74451
IFT46	2.99267	3.09366	2.57138	2.89399	1.73203	2.354
IFT52	3.39104	3.56229	3.65883	4.11154	3.14544	3.8932
IFT57	3.26237	3.33644	3.92369	4.27271	3.17889	3.88387
IFT74	2.54426	2.47406	2.8637	3.22228	3.66102	4.68576
IFT80	3.75953	4.11525	4.98666	5.27549	4.26995	4.61662
IFT81	3.65957	3.36529	3.40144	3.33618	3.23299	3.16673
IFT88	2.98918	2.96761	3.11569	3.09819	2.63432	3.0041
IGBP1	3.77462	3.92408	3.42195	3.97064	4.6065	5.08283
IGBP1P1	-0.0695851	-0.177293	-0.404514	-0.201784	0.606556	0.832509
IGF1R	7.16653	6.86075	5.12935	5.02559	6.15005	5.73154
IGF2BP2	5.66067	5.56615	4.59175	4.79204	5.6708	5.29541
IGF2BP2-AS1	1.43024	1.25145	0.503064	0.350701	0.405873	-0.577229
IGF2BP3	3.19627	2.90472	5.96092	6.4293	4.5401	4.5204
IGF2R	7.30338	7.33631	7.68327	7.86558	7.91494	7.6489
IGFBP2	1.46083	1.23764	4.8023	4.43806	2.50712	2.54596
IGFBP3	2.20482	4.23499	5.41034	6.37019	1.90957	1.64205
IGFBP4	-4.11163	-2.37465	6.9984	7.03263	2.20079	2.39659
IGFBP5	7.15501	6.51782	12.2702	12.0428	9.11489	8.11933
IGFBP6	2.15268	2.33807	3.96369	4.87272	0.172692	1.74453
IGFBP7	5.92848	6.03696	3.8352	3.88738	7.80749	8.15508
IGFL4	0.0152977	-0.333394	-3.18222	-2.64851	1.89813	0.97985
IGFLR1	0.0703756	-0.375208	0.464438	0.672335	0.46134	0.593229
IGHMBP2	3.49444	3.43046	2.87177	3.09575	2.82552	2.61973
IGIP	2.42247	2.68437	2.6109	2.70175	2.31125	2.77307
IGSF1	1.16516	1.01295	0.371169	0.632724	?	?

IGSF10	-0.329616	-0.538689	2.72468	2.72284	1.08847	0.657219
IGSF3	5.923	5.54004	4.91736	4.41545	6.17323	5.89996
IGSF6	-0.144345	0.257831	0.827461	0.591699	-0.43131	-0.577229
IGSF8	4.1927	3.76837	3.06204	3.35878	2.87303	3.15127
IGSF9B	1.48086	1.60328	0.986462	0.685662	-2.85193	-5.69922
IK	5.57599	5.54525	5.14612	5.46793	5.74203	5.71356
IKBIP	5.1334	5.24451	4.40425	4.75148	4.62566	4.71745
IKBKAP	5.12544	5.32667	4.38824	4.78825	4.79501	4.97978
IKBKB	4.63909	4.73436	3.91845	3.98128	4.69062	4.0465
IKBKE	2.81663	2.83564	3.88093	4.28509	2.38136	3.07792
IKBKG	2.90274	2.6091	3.26632	3.55465	3.78049	3.98175
IKBKGP1	-0.392429	-0.262337	0.211261	0.385983	0.588929	0.868387
IKZF2	1.80079	1.74176	-1.229	-1.35358	2.61116	2.16982
IKZF4	0.710384	0.559609	1.17408	0.786061	0.36215	0.0213376
IKZF5	2.95555	2.73438	3.16742	3.37701	3.64575	3.27057
IL10RB	3.14878	2.77604	5.46352	5.72787	4.0184	4.4019
IL11	2.61154	2.1408	-2.37543	-1.06415	4.193	5.06725
IL11RA	1.30601	1.16152	1.80397	1.99231	-2.05422	-2.38405
IL12A	-1.08408	-1.29258	1.0357	1.35065	-0.200727	-0.799586
IL13RA1	4.85887	4.95552	5.72148	6.06422	4.98623	5.00322
IL13RA2	-3.2383	-4.13839	-6.17309	?	5.11823	6.08853
IL15	3.20781	2.92948	2.18282	2.18121	0.661695	1.02127
IL15RA	-2.1135	-2.55512	2.42012	2.51324	-1.95932	-3.12051
IL16	3.53969	3.20881	-0.987408	-0.20485	-3.23744	-2.90575
IL17D	0.485283	0.613211	-0.65032	-1.2711	1.67702	1.5109
IL17RA	4.31144	4.23758	3.94446	4.04473	3.44821	3.19717
IL17RB	0.345419	0.491443	0.0645242	-0.108685	0.427241	0.0213376
IL17RC	2.78273	2.56231	3.32823	3.54939	3.01917	3.22993
IL17RD	1.98042	2.58429	1.05494	0.857026	4.35301	4.72582
IL18	-2.89067	-3.65369	-0.69145	0.300663	4.96082	5.26841
IL18BP	2.88962	2.81421	2.97146	2.88885	2.16848	1.20314
IL18R1	1.63187	2.66902	-0.69145	-0.131402	-5.65305	-4.70296
IL1A	5.88611	6.58188	-0.598354	-0.327299	-1.57243	-1.18284
IL1B	4.03985	4.73375	-0.0541032	0.28359	-2.13612	-2.12114
IL1R1	3.16821	2.95502	6.58545	6.33017	1.80732	2.33769
IL1RAP	5.5264	5.75054	3.42935	4.06734	6.00634	5.43602
IL1RAPL1	2.5201	2.05681	1.34008	0.786061	-4.65668	-5.69922
IL20RB	-0.744656	-0.818747	2.64304	2.61911	-0.136604	-1.18284
IL22RA1	-0.159773	0.20969	1.48185	1.61913	-4.07293	-4.70296
IL27RA	3.49568	3.48113	3.16963	3.36691	1.49454	1.27061
IL31RA	2.4608	2.62992	2.00137	2.88836	3.39768	3.71536
IL32	1.08887	1.43201	-1.229	-0.795121	-1.41201	-0.247123
IL4R	0.800836	1.16656	4.0063	4.67006	-6.64581	?
IL6	3.51379	3.11257	1.8934	3.62982	0.775674	0.785944
IL6R	3.18662	2.28201	1.70525	1.51326	3.14383	2.54596
IL6ST	7.81442	7.96906	8.92801	9.60677	7.79479	8.30765
IL6STP1	1.93115	2.29859	3.13645	3.82479	1.88958	2.21096
IL7	1.37339	1.71244	2.02107	2.33413	0.458718	1.29424
IL7R	3.60127	3.25312	5.17677	5.30267	5.85834	5.50151
ILDR2	-3.2383	-4.13839	2.08328	2.36286	0.615896	1.35942
ILF2	7.00768	7.10626	6.23797	6.75477	6.85798	7.11106
ILF2P1	1.04945	1.31871	0.243182	0.868521	1.10129	0.422922

ILF3	7.20603	7.04344	6.80959	6.79299	7.51961	6.84474
ILF3-AS1	2.55123	2.44105	1.7995	2.01014	2.68621	2.05486
ILK	5.46244	5.59029	4.83234	5.39894	4.87849	5.24017
ILKAP	3.65957	3.59243	3.07622	3.14384	3.37036	3.37011
ILVBL	3.75213	3.65089	2.97887	3.35878	3.88851	4.54476
IMMP1L	1.28965	1.3251	1.69303	1.67257	2.02971	2.40182
IMMP2L	3.78876	3.44554	-0.568612	-0.823182	0.815493	1.30456
IMMT	6.19293	6.22491	5.61708	5.97941	6.32997	6.39869
IMMTP1	1.3414	1.37335	0.904496	1.20451	1.52038	1.26033
IMP3	4.36432	4.37473	3.36994	3.77022	4.5634	4.80303
IMP4	5.27753	5.19223	3.87446	4.35674	5.26863	5.50394
IMPA1	3.35892	3.64306	2.8193	3.49851	4.63315	5.14815
IMPA2	2.93392	2.55119	2.4422	2.44961	2.76806	2.5925
IMPACT	3.9147	3.91435	3.19369	3.68874	2.50461	2.90432
IMPAD1	6.82029	6.86021	6.709	6.98409	6.51367	6.22771
IMPDH1	4.5305	4.50615	3.85714	4.22595	3.70604	3.82652
IMPDH2	6.60367	6.75342	5.26045	5.82779	7.41491	7.78354
INADL	3.61946	3.62859	3.91319	3.99314	4.09957	4.07628
INAFM2	1.28396	1.31856	0.156448	0.591699	1.51964	0.878317
INCENP	5.5408	5.53749	4.79947	5.04091	4.63316	4.07629
INE1	0.567789	0.635262	0.416483	0.0811214	-1.20056	-1.79933
INF2	4.41206	4.08018	3.68375	3.70013	2.90385	2.93715
ING1	2.82778	2.62874	2.74627	2.66438	2.67786	2.26202
ING2	1.61837	1.53719	1.86635	2.09087	2.17576	2.03478
ING3	3.34258	3.43046	2.66511	2.97333	2.78264	3.12618
ING4	3.00828	2.68691	3.07387	3.44192	3.43904	3.97628
ING5	3.40526	2.97712	2.93277	2.58567	4.03923	3.1305
INHBA	5.41042	6.43726	2.2677	3.1055	1.8711	2.18812
INIP	3.85454	3.8969	3.28815	3.377	4.2593	4.12933
INO80	4.92649	4.93869	4.28377	4.42032	5.1339	4.86125
INO80B	2.50015	2.67047	1.51383	1.95526	2.13612	2.39595
INO80B-WBP1	2.12485	1.53099	1.50088	1.95305	1.95576	2.22132
INO80C	1.14018	1.60774	0.386425	0.821824	0.909612	0.719622
INO80D	4.83508	4.90101	4.27865	4.19159	5.02077	4.96055
INO80E	4.22295	4.08321	3.91956	4.15568	3.95981	4.03514
INPP1	1.952	2.02075	2.17844	2.67582	2.65711	2.89321
INPP4A	4.33846	4.36608	4.79446	4.8989	4.3446	3.96216
INPP4B	5.00785	4.93268	2.91451	2.89961	-4.65668 ?	
INPP5A	3.03437	2.81398	2.39298	2.8151	3.87158	3.78313
INPP5B	3.91747	3.80627	3.6729	3.95321	4.19221	3.81488
INPP5E	1.48086	1.46201	1.71133	1.79207	1.9359	1.75275
INPP5F	6.38918	6.44399	3.94319	4.26186	5.82551	5.64905
INPP5K	3.37104	3.34934	2.80233	3.11276	4.01828	4.26338
INPPL1	5.87219	5.62062	5.45128	5.57547	5.26002	4.96128
INSIG1	5.85025	5.94924	6.66172	7.14256	6.62874	7.67816
INSIG2	3.10506	3.43803	2.89863	3.1603	3.99342	4.05981
INSR	4.85214	4.76536	2.74129	2.83076	2.1112	1.54125
INTS1	5.04743	4.83463	5.34492	5.40045	5.07354	5.30868
INTS10	4.98127	5.11031	4.2759	4.66348	4.11776	4.65851
INTS12	2.59469	2.5239	2.61386	3.06579	2.91122	3.31701
INTS2	4.84587	4.74719	4.53415	4.53321	4.84846	4.39
INTS3	5.36085	5.21228	5.16637	5.01979	5.11624	4.72668

INTS4	4.89522	5.04696	4.15716	4.3418	3.63143	3.90125
INTS5	3.71243	3.61664	3.20665	3.54067	3.22386	3.25039
INTS6	5.70108	5.64302	6.03678	5.98984	5.9324	5.65641
INTS6P1	1.36685	1.45532	1.4758	1.51517	1.66552	1.66102
INTS7	4.32258	4.36845	4.13953	4.39896	4.21005	4.53884
INTS8	4.30482	4.23492	4.91067	5.11694	4.75614	5.03283
INTS9	2.98041	2.79926	2.98641	3.19724	2.88079	2.66833
INTU	2.85359	2.83178	-6.17309	-6.09892	3.33262	2.80505
INVS	3.81661	3.75512	3.93151	4.02045	2.96175	2.84788
IP6K1	4.9839	4.8859	4.33118	4.56446	5.52948	5.17438
IP6K2	4.05329	3.89581	4.82979	4.95388	4.70873	4.53049
IPMK	2.95727	2.9423	3.62543	3.80107	3.82849	3.1819
IPO11	4.56763	4.65442	4.72394	5.07555	4.7391	5.0625
IPO13	4.60527	4.4986	4.61333	4.9239	4.64859	4.36072
IPO4	4.7904	4.69869	2.97725	3.13832	4.91374	4.79548
IPO5	7.20703	7.29057	6.37129	6.7365	6.7211	7.00732
IPO5P1	1.24348	1.10707	2.05967	1.97335	1.5246	1.18815
IPO7	6.21154	6.23148	6.79645	7.13339	7.72953	7.76998
IPO7P1	0.75867	1.09043	1.30986	1.89919	2.57703	2.27497
IPO7P2	2.78642	2.87671	3.24584	3.62612	4.17161	4.4145
IPO8	5.41565	5.43912	4.55795	4.77073	4.87351	5.12775
IPO8P1	2.02357	2.04642	2.54838	2.9239	3.37062	3.37473
IPO9	6.79049	6.9619	6.4474	6.73854	6.45108	6.50396
IPP	2.43792	2.36371	3.41453	3.51323	2.6774	2.92629
IPPK	4.07851	3.97487	2.9288	3.1713	4.05548	3.6582
IQCB1	3.28775	3.16788	2.55434	2.71438	3.04687	3.22993
IQCC	2.19625	2.23637	2.13866	2.17403	2.23	1.78962
IQCE	2.48084	2.46791	3.97382	4.16845	3.07101	2.64644
IQCG	2.52734	2.93162	2.8305	3.10306	5.85612	6.22214
IQCH	0.51789	0.616424	0.99492	1.03701	0.162867	0.87998
IQCH-AS1	1.16662	1.40619	0.851655	0.555031	0.508307	1.06006
IQCJ-SCHIP1	2.80958	2.90055	2.36328	2.77555	4.49308	4.47078
IQCK	0.580136	0.865705	0.931502	1.24351	0.470126	0.56699
IQGAP1	7.90315	7.96167	7.6694	8.0958	8.57924	8.61394
IQGAP2	1.23761	1.3764	6.32598	6.45793	0.258664	-0.280287
IQGAP3	6.26637	6.22164	4.73978	5.07169	5.84345	5.25908
IQSEC1	-2.17488	-2.6546	2.78516	2.47615	1.40582	1.22407
IQSEC2	0.367103	-0.177293	2.38635	2.81004	0.458718	0.878317
IRAK1	6.00304	5.94337	5.59904	6.07232	7.51272	7.87863
IRAK1BP1	3.08309	3.28535	-0.571714	-1.3793	2.48171	2.56556
IRAK2	3.82349	4.04693	2.60112	3.15327	2.29681	2.41742
IRAK4	2.50551	2.56783	3.13841	3.5187	2.87886	3.30451
IREB2	5.95868	5.90263	5.88288	5.90414	6.29784	6.17782
IRF1	3.53932	3.84322	5.76337	6.10488	3.47411	3.70268
IRF2	3.3412	3.45303	3.59455	3.97465	3.21928	3.35668
IRF2BP1	3.13697	3.03101	1.71737	2.02562	3.08789	3.32119
IRF2BP2	5.17489	5.28614	5.32865	5.45119	5.51028	5.43278
IRF2BPL	3.74424	3.38813	3.58668	3.34026	3.70113	3.35303
IRF3	3.1623	2.90324	3.67075	4.07917	3.50369	3.62016
IRF7	0.255223	-0.0703893	1.26773	1.33416	-1.65987	-1.70626
IRF9	2.05603	2.035	2.61536	2.70794	4.14815	4.19557
IRGQ	5.57731	5.51516	4.67523	4.88052	5.72985	4.97189

IRS1	3.32606	2.98837	4.13727	4.36993	4.99789	5.06724
IRS2	3.51646	3.40493	1.77207	2.05107	3.25335	3.12618
IRX1	-2.23898	-2.21426	0.849967	0.749225	3.78055	3.52211
IRX2	1.16516	0.746683	1.11115	0.924662	?	?
IRX3	1.17375	1.10269	1.24216	1.12275	1.52888	0.977185
ISCA1	3.83759	3.72889	2.83409	3.06961	4.21597	4.37844
ISCA1P1	1.6764	1.52412	0.4804	0.628288	1.82731	2.43331
ISCA2	2.61143	2.45305	2.03567	2.40294	2.51712	3.31558
ISCU	4.3494	4.46195	3.78514	4.39001	4.3425	4.8773
ISG15	-0.255978	-0.0197685	2.34397	2.46104	3.81946	4.47194
ISG20	0.768595	0.988427	0.371169	1.1104	-0.660096	-1.31405
ISG20L2	5.16804	5.27982	3.70082	3.85752	5.22413	5.33705
ISL2	0.621131	0.488895	0.0427984	0.306437	0.285071	-0.253125
ISLR	0.323393	1.43201	0.156448	0.491176	-0.637731	0.50283
ISOC1	3.91747	4.03501	3.00631	3.16963	2.84749	3.05484
ISOC2	2.20783	2.04497	3.30432	3.83516	3.40039	4.21512
IST1	5.35796	5.33376	5.76994	5.99136	6.49792	6.40803
ISY1	4.02966	4.0967	3.21684	3.56019	3.97103	4.21377
ISY1-RAB43	3.04287	2.70878	4.55145	4.71488	4.02404	3.72066
ISYNA1	2.5574	2.03245	0.940174	0.773076	-1.1236	-2.40762
ITCH	6.35771	6.38828	6.18605	6.47073	5.83352	6.05692
ITFG1	4.56221	4.53491	4.89254	5.20212	5.79132	6.32598
ITFG2	3.1609	2.96086	2.7812	2.7418	2.83089	2.342
ITFG3	3.15902	2.54882	4.91193	4.76124	4.3002	4.24534
ITGA1	4.70382	4.81735	4.9509	5.32248	-0.60452	-0.0622413
ITGA10	-0.0842313	-0.214764	1.29226	1.53313	-2.48959	-2.24661
ITGA11	0.567789	0.727049	5.23379	5.22433	-0.200727	-0.619042
ITGA2	6.27895	6.72811	6.73537	7.22305	5.68259	5.77457
ITGA3	7.84253	8.44325	7.16743	7.71659	7.88129	8.20868
ITGA4	6.62084	6.5189	3.70978	3.85407	5.19027	5.40702
ITGA5	7.52963	7.83233	6.59286	7.04044	5.29168	5.55948
ITGA6	8.15601	8.09784	6.48032	7.16033	6.37989	6.46217
ITGA7	1.55835	1.41984	-1.2763	-1.06415	1.15352	1.68564
ITGA9	4.046	3.48325	-0.748505	-0.52367	5.78928	5.93575
ITGA9-AS1	0.766103	0.722641	-0.945035	-0.716287	0.522267	-0.493861
ITGAE	2.60927	2.49427	2.80517	3.10307	2.91712	3.89505
ITGAV	8.13912	8.25835	7.46553	7.59236	7.91048	7.80109
ITGAX	-5.69162	-4.87366	3.63985	3.93008	-6.64581	?
ITGB1	9.09507	9.00168	8.45361	8.39053	8.83447	8.79666
ITGB1BP1	4.31938	4.29162	3.913	4.33743	5.29521	5.61947
ITGB1P1	6.40161	6.24027	5.80501	5.6131	6.05854	6.22543
ITGB3	8.81823	9.01861	4.41	5.10003	5.57938	5.31897
ITGB3BP	3.79986	3.78241	4.12456	4.31043	3.3565	4.40572
ITGB4	0.61388	0.382745	-0.138983	-0.464785	7.241	7.5916
ITGB5	6.01019	5.71673	5.28562	5.41031	5.49419	5.42771
ITGB8	9.28403	8.9763	5.40892	5.0088	8.46247	8.34024
ITGBL1	-3.85083	-3.33921	4.22893	5.10739	2.51427	2.96624
ITIH6	2.09536	1.53154	?	?	-6.64581	-5.69922
ITM2B	5.82912	5.84776	6.08033	6.30241	7.70361	7.60188
ITM2C	6.6521	6.35332	6.33925	6.34314	5.3446	5.32463
ITPA	3.53574	3.39513	3.25735	3.55652	3.88465	4.44937
ITPK1	5.21342	5.38228	1.80519	2.72407	4.00146	4.45063

ITPK1-AS1	1.33988	1.23764	-2.18288	-0.979274	-1.57243	-2.00571
ITPKB	3.16511	3.29705	3.08795	3.0611	4.06591	4.03811
ITPKC	2.60927	2.57608	3.01124	3.16963	3.22691	3.31566
ITPR1	4.12508	4.53361	4.63744	5.04273	5.44819	5.07464
ITPR2	5.82104	5.72541	6.57491	6.48082	4.24131	3.92627
ITPR3	6.01819	6.01001	5.24581	5.55783	4.62341	4.79837
ITPRIP	4.02459	4.20249	4.21735	4.66017	3.40443	3.12618
ITPRIPL1	2.29812	2.56507	0.699196	1.04098	-4.07293	-4.70296
ITPRIPL2	5.59016	5.58608	5.01915	5.03121	4.86673	4.59359
ITSN1	4.2768	4.22439	5.35384	5.54275	4.22972	4.01267
ITSN2	5.1877	5.23016	4.61163	4.71906	5.30326	5.41157
IVD	4.26309	4.07437	4.10072	4.33307	3.78676	3.78313
IVNS1ABP	6.14227	6.13357	5.3811	5.50725	6.09038	6.29187
IWS1	5.36984	5.21594	4.91966	4.96624	5.56174	5.00132
JADE1	4.83474	4.99907	4.57867	4.62164	5.21542	5.21809
JADE2	5.30161	5.42169	5.12082	5.44432	5.60881	5.79163
JADE3	3.6363	3.80861	3.17842	3.1858	2.99791	2.5925
JAG1	5.1014	5.62164	3.80516	4.20292	7.23742	6.50822
JAGN1	4.46896	4.29037	3.48536	3.77991	4.08368	4.30451
JAK1	6.58414	6.57674	6.69623	7.05418	6.46524	6.78837
JAK2	2.58864	2.82487	2.96114	3.43033	3.55643	3.59707
JAKMIP2	2.93574	3.05288	-2.58563	-1.95998	2.06182	3.11843
JAKMIP3	-3.8896	-3.65369	3.02846	2.73864	3.44792	1.84021
JAM2	0.567789	0.813344	2.0405	1.99449	-2.65941	-3.12051
JAM3	6.08051	6.025	4.61522	4.96104	5.71205	5.71906
JARID2	5.10905	4.93105	4.25527	4.25422	5.23068	4.59477
JAZF1	1.63485	1.83344	3.02133	3.14125	2.72673	3.24399
JDP2	-2.37642	-2.46206	1.53076	2.01532	0.0122397	0.126299
JKAMP	4.73502	4.55117	4.3302	4.64268	5.2492	5.64697
JMJD1C	5.90306	5.74361	5.79399	5.68637	6.12402	5.90691
JMJD4	3.60837	3.59211	3.39208	3.6365	3.14037	3.29007
JMJD6	4.64532	4.56368	3.94189	4.20065	4.8655	4.41351
JMJD7	0.906106	0.708081	0.810986	0.899354	0.391999	1.3453
JMJD7-PLA2G4B	2.13154	2.09551	2.88935	2.75073	2.62034	1.90972
JMJD8	4.8516	4.63899	4.71484	4.90755	4.51452	4.29645
JMY	3.93209	4.0139	3.5478	3.5864	4.58702	4.53288
JOSD1	5.09045	5.26251	4.82909	5.26892	5.14784	5.13563
JOSD2	0.146525	-0.292758	-0.0541032	-0.0642959	0.706084	1.73626
JPH1	2.93937	3.06659	-0.138983	-0.493926	0.293994	-0.280286
JPH2	-4.11163	-3.87578	1.09265	1.77384	1.99792	1.56945
JPX	3.16351	3.1032	3.80588	3.91946	3.84684	3.65137
JRK	4.42737	4.3413	4.47828	4.60033	3.86092	3.19088
JRKL	2.45575	2.64569	3.49243	3.65273	2.74484	2.62869
JTB	5.39102	5.49746	4.27078	4.86987	5.53501	6.21849
JUN	4.40092	4.43045	4.25734	4.85551	5.76909	5.60048
JUNB	2.95556	2.68182	1.74725	2.32164	2.58763	2.78114
JUND	2.72719	2.1445	1.71133	1.76772	4.40038	3.70055
JUP	?	?	1.27595	1.55654	2.16625	2.22994
KALRN	2.12122	1.45008	3.02629	2.81302	-1.37455	-1.79933
KANK1	4.19648	3.93268	5.95025	6.06171	5.86887	5.77715
KANK2	3.63295	3.46048	4.99327	4.97929	4.92229	4.65742
KANSL1	5.49336	5.32356	5.87291	5.72206	4.9821	4.64844

KANSL1L	3.88093	3.93148	4.2933	4.39399	3.95621	3.92882
KANSL2	3.6986	3.6522	3.88525	3.98128	3.34391	3.43795
KANSL3	5.22311	5.17378	4.91713	5.05607	5.47761	5.59648
KANTR	1.44068	1.29657	0.891886	1.02402	0.645324	-0.881925
KARS	6.50607	6.53961	5.95384	6.5251	6.18556	6.70234
KARSP1	0.255223	0.432061	-0.183372	0.430424	0.0402612	-0.497071
KAT2A	3.83095	3.56075	4.19691	4.00697	4.19919	3.6122
KAT2B	5.11468	4.36211	3.82771	3.71771	4.96173	4.97452
KAT5	4.06734	4.00382	2.77159	3.02049	3.49229	3.74757
KAT6A	5.85454	5.92112	5.66314	5.76114	5.11693	4.82952
KAT6B	3.1179	2.72698	4.08911	3.87558	4.86961	4.1698
KAT7	5.33017	5.42717	4.93612	5.25907	4.82213	4.77577
KAT8	3.21978	3.13707	3.04289	3.2399	3.70492	3.22403
KATNA1	2.6206	2.54839	2.30433	2.43808	3.21006	3.34857
KATNAL1	4.56183	4.6066	4.62383	5.02109	4.8856	4.7185
KATNAL2	2.51282	2.51731	1.75315	1.73669	-0.43131	-1.18284
KATNB1	3.31494	2.99249	3.20018	3.65273	4.441	4.52571
KATNBL1	3.63053	3.84277	3.45109	3.56487	3.33587	3.70636
KATNBL1P6	1.09644	1.3914	0.532297	0.837055	0.805012	1.88771
KAZALD1	2.0083	1.74176	1.0357	0.761609	-0.200727	-0.38461
KAZN	-0.129199	-0.0185039	0.723445	1.32383	1.69109	1.28202
KB-1208A12.3	3.75985	3.83977	3.46447	4.01729	4.26694	5.41535
KB-1460A1.5	-0.323873	-0.036446	-0.161006	0.563685	0.679613	0.0213376
KB-1572G7.2	2.46262	2.38338	2.22988	2.19468	0.903324	-0.17222
KB-431C1.5	0.278306	-0.120595	1.01783	0.903293	-0.0163223	-0.4586
KBTBD2	5.33018	5.42932	5.17127	5.33567	5.29897	5.42835
KBTBD3	-0.547721	-0.25044	1.2174	1.24116	0.816395	1.76092
KBTBD4	3.30122	3.14991	3.05889	3.29882	3.82909	4.05096
KBTBD6	4.25721	4.55493	3.56716	3.91493	4.21223	4.25191
KBTBD7	3.48966	3.38984	3.37397	3.46292	3.44188	3.36461
KBTBD8	2.69967	2.53152	1.45314	1.43811	1.91714	2.42258
KC6	2.37775	2.49136	-5.1783	-6.09892	-6.64581	-5.69922
KCMF1	5.05246	5.02699	4.8249	5.22656	5.361	5.3533
KCNA2	1.76447	1.65093	-4.18092	-3.52222	?	-5.69922
KCNAB1	1.49565	1.95118	1.79388	1.99972	0.93937	0.474306
KCNAB2	0.930336	0.894767	3.0501	3.51323	2.32554	1.89323
KCNAB3	1.74389	1.43806	0.27602	0.158078	-0.338214	-1.61882
KCNC4	2.92298	3.43953	0.340141	0.798133	-2.85193	-3.70484
KCND1	1.61384	0.912259	1.83887	1.38307	-1.95932	-2.70579
KCNE4	0.440559	0.181128	5.66978	5.68233	4.18361	3.61522
KCNG1	3.49838	3.56085	0.686917	0.798133	-5.65305	?
KCNH1	1.5991	1.71068	-0.962089	-0.792822	-3.78453	-2.24816
KCNH2	?	-5.45651	0.711372	1.24877	-6.64581	?
KCNH5	2.93574	2.93804	-6.17309	?	-1.80184	-0.183001
KCNIP3	0.768595	0.840999	0.759062	0.902467	0.519683	1.12623
KCNIP4	1.65027	1.64433	0.610974	0.28359	1.64653	1.57817
KCNIP4-IT1	4.10424	4.17286	?	-3.78492	2.89812	2.64644
KCNJ13	1.84508	1.86831	2.46193	2.17536	1.76874	1.2539
KCNJ14	1.08953	0.936259	-0.471995	-0.380164	0.551344	0.127792
KCNJ2	-0.676496	-1.14061	3.19123	3.11035	4.05048	3.24748
KCNK1	?	?	0.699196	1.10068	-1.85245	-2.00571
KCNK5	2.96804	2.94655	-5.1783	-6.09892	-6.64581	?

KCNMA1	4.74049	5.46928	4.50838	5.10132	5.40004	5.80307
KCNMA1-AS1	1.09207	1.77653	1.08146	1.32456	0.831974	0.401084
KCNMB3	0.211859	0.153676	1.19404	0.842609	1.44068	0.59343
KCNMB4	1.74389	1.41371	-1.05395	-1.78641	0.597155	-0.348991
KCNN3	5.04787	5.30577	-0.69145	0.317536	-5.07051	-3.12051
KCNN4	5.19649	5.4946	2.96114	3.27272	4.40845	4.25762
KCNQ1OT1	9.93594	9.734	7.34823	7.05402	6.03312	5.67345
KCNQ5	3.70073	3.62063	-6.17309	-6.09892	3.40174	3.2677
KCNRG	1.2357	1.56891	1.27715	1.13162	1.82346	1.69141
KCNS2	0.367103	-0.214763	0.488988	0.100749	0.197777	0.0213376
KCNS3	4.31634	4.9013	0.208904	1.55655	0.597164	1.50278
KCNT2	1.52013	1.06859	-0.428455	-0.823182	0.578176	1.25912
KCNV2	-0.549135	-0.27286	-1.42826	-1.71605	1.26455	0.965773
KCTD1	2.57706	2.59192	2.35177	2.4799	1.85891	1.75275
KCTD10	5.05432	4.99858	5.06309	5.23185	4.95016	4.7619
KCTD11	1.7968	1.73686	2.36724	2.72723	2.15669	1.92993
KCTD12	1.88966	2.08021	4.76411	4.35162	5.9326	5.16749
KCTD13	2.24231	2.4329	1.77418	1.81168	1.2071	1.0747
KCTD14	-3.91265	-3.03182	1.58472	1.9097	-3.77088	-3.38849
KCTD15	3.84298	3.55952	3.52211	3.54402	3.97268	3.75272
KCTD17	2.03903	2.24798	2.36339	2.9301	1.72774	2.31011
KCTD18	2.48581	2.6587	2.46754	2.69531	2.71908	2.96925
KCTD2	3.38174	3.52015	3.99264	4.48828	4.83053	5.20915
KCTD20	6.03479	6.24668	5.93185	6.45842	6.96351	7.30403
KCTD21	2.6996	3.01135	2.43326	2.91362	2.43226	2.59344
KCTD21-AS1	2.85941	3.04051	1.02051	1.16711	-0.571088	-0.585622
KCTD3	5.72035	5.74633	4.64302	5.02109	5.43246	5.60504
KCTD4	-0.0550876	-0.604659	0.914572	1.43811	0.679613	0.816919
KCTD5	3.61143	3.47725	3.65634	4.00758	3.87658	3.99342
KCTD6	2.80077	2.73928	1.78229	2.03586	2.65259	2.55539
KCTD7	2.8584	2.92462	2.93891	2.99571	3.36859	3.38598
KCTD9	4.69672	4.72892	4.19488	4.66142	4.88623	4.75307
KCTD9P1	-0.0824705	-0.411708	1.12269	1.52156	2.41459	2.34071
KCTD9P2	0.675708	0.788945	-0.13983	0.658975	0.81487	0.913286
KCTD9P4	0.917554	0.450728	0.385508	0.865966	0.915919	0.892445
KDELC1	3.71337	3.40133	3.58796	3.60713	3.1789	3.49027
KDELC1P1	0.221209	0.603321	0.558032	0.317536	-0.0752099	-0.211608
KDELC2	4.5435	4.2803	6.52775	6.63008	5.05221	4.9043
KDELR1	5.21228	5.10318	5.80761	5.93355	5.73545	5.82075
KDELR2	6.84618	6.81272	7.42315	7.61938	6.999	6.76522
KDELR3	4.23976	4.28114	3.69606	4.2432	2.77015	3.59249
KDM1A	6.43391	6.45749	5.53201	5.84972	5.80089	5.93397
KDM1B	3.23461	3.435	2.12484	2.55296	3.61348	4.01438
KDM2A	5.67976	5.64598	5.83675	5.96863	5.35684	4.84595
KDM2B	4.16735	4.03967	3.67655	3.61308	3.83747	3.02722
KDM3A	4.98301	4.9062	4.97823	4.76154	5.96515	5.58157
KDM3B	5.81566	5.77708	5.9589	6.04535	5.97177	5.75322
KDM4A	4.94052	4.80768	5.26262	5.43713	4.96154	4.61108
KDM4A-AS1	0.843132	0.609689	0.994601	1.20345	0.251483	-0.816433
KDM4B	2.69753	2.48845	3.25942	3.26839	4.18518	3.64643
KDM4C	4.017	4.07587	3.64313	3.72203	5.27204	4.74187
KDM5A	6.76026	6.69285	6.53858	6.69755	6.23718	6.08493

KDM5B	5.99699	6.21467	5.97477	6.19725	5.21429	5.16826
KDM5C	5.90453	5.88864	5.33561	5.61354	5.57602	5.17516
KDM5D	-6.68418	-6.45019	4.63905	4.62163	-5.65305	?
KDM6A	4.29527	4.15553	2.77358	2.77077	4.04532	3.31565
KDM6B	1.51527	1.10707	2.74427	2.35471	1.91714	1.17598
KDM7A	4.49074	4.72575	2.759	3.02304	3.83054	4.14503
KDR	-6.68418	-4.13839	?	-6.09892	2.24359	3.06478
KDSR	5.57267	5.68752	3.95072	4.34752	3.47924	3.56708
KEAP1	3.93664	3.92624	4.17513	4.70174	4.36414	4.77809
KHDC1	1.09539	0.946604	-1.37581	-1.40802	2.78057	2.48806
KHDRBS1	5.98936	5.93887	5.78187	5.77801	5.95761	5.81489
KHDRBS3	4.07406	4.25996	-2.86056	-2.64851	4.72122	4.50395
KHK	1.84669	1.79528	-1.32519	-0.709972	1.1995	1.70699
KHNYN	5.20497	5.06284	4.40711	4.45412	5.03773	4.85088
KHSRP	5.37486	5.12013	4.76621	5.01335	5.477	5.25038
KHSRPP1	2.08411	1.9174	1.56548	1.69856	2.29379	2.02123
KIAA0020	5.53992	5.46935	4.29726	4.5278	6.51942	6.66505
KIAA0040	4.28603	4.81734	-1.01332	-0.52367	-4.65668	-5.69922
KIAA0100	7.48234	7.52193	7.19917	7.43469	8.45151	8.35801
KIAA0101	4.82945	4.73155	4.45343	4.6043	4.83911	4.8103
KIAA0141	4.71508	4.63649	4.51778	4.64438	4.58939	4.47195
KIAA0195	3.6352	3.24624	4.60844	4.4313	4.6053	4.38999
KIAA0196	4.85796	4.86204	4.66781	5.03589	4.59154	5.03347
KIAA0226	3.33159	3.22366	3.76776	3.93966	5.17494	5.00236
KIAA0226L	-0.191134	-0.00302562	?	?	0.549226	0.0483059
KIAA0232	5.98736	5.99216	5.77066	5.74756	5.77925	5.39461
KIAA0319	-1.20691	-0.292758	0.711372	0.857026	0.782702	0.200291
KIAA0319L	6.05582	5.90508	5.80728	5.84602	6.36031	6.34345
KIAA0355	3.82546	3.8523	3.95218	4.16962	4.76909	4.65193
KIAA0368	6.29669	6.38712	5.92793	6.18984	5.99901	5.97099
KIAA0391	4.46882	4.44753	3.61128	3.93945	4.54027	4.74531
KIAA0408	0.925483	1.67661	-0.12203	0.110697	3.20615	3.42124
KIAA0430	4.63072	4.66965	4.50821	4.68313	4.58166	4.5209
KIAA0513	2.98217	2.82718	3.37107	3.80555	3.12759	3.11983
KIAA0556	3.09156	3.19292	3.63441	3.73571	3.56546	3.40406
KIAA0586	4.39053	4.42478	3.5769	4.00859	4.23215	4.53763
KIAA0753	3.66112	3.63222	4.44557	4.40148	4.3549	4.11843
KIAA0825	0.71885	0.822624	0.0671844	0.0811214	-0.10558	1.17598
KIAA0895	3.16665	3.19192	3.08097	3.42852	3.33548	3.35945
KIAA0895L	2.8136	2.46584	3.56533	3.26446	3.6152	2.81747
KIAA0907	5.77439	5.92893	5.1231	5.54714	5.66444	5.46633
KIAA0922	3.69859	3.82139	3.65726	3.53684	3.2866	3.27057
KIAA0930	4.91516	4.82486	5.82104	6.14707	4.60763	4.41611
KIAA1024	1.59098	1.54281	1.13845	1.32585	2.84153	2.75273
KIAA1033	6.019	6.09197	5.43071	5.75882	5.90315	6.24854
KIAA1107	1.02208	0.831845	1.98643	1.8912	-0.412205	-0.0627204
KIAA1109	6.57002	6.56521	7.37191	7.43782	6.60922	6.737
KIAA1143	4.84109	4.83861	4.38417	4.70042	5.17347	5.28885
KIAA1147	5.76112	6.00373	4.89061	5.13731	6.03556	6.24785
KIAA1161	2.17438	1.92088	1.57806	1.39901	3.16147	2.2475
KIAA1191	5.01853	5.08236	5.4064	5.8993	5.36841	5.57587
KIAA1211	1.72302	1.91656	-1.53926	-1.58485	-1.41201	-1.89883

KIAA1217	4.57704	4.8156	4.18825	4.29417	3.46639	3.13564
KIAA1324L	5.05706	5.52085	-2.09546	-3.10759	?	-5.69922
KIAA1328	2.01677	1.88591	2.48183	2.37815	1.49671	1.04823
KIAA1407	0.171373	0.503023	1.57806	1.24877	0.384182	-0.706491
KIAA1429	5.87197	5.9054	5.91121	6.15034	6.06312	6.34722
KIAA1462	0.45076	0.407614	5.48402	5.37094	0.939663	0.80115
KIAA1467	3.86698	3.71707	3.52901	3.64439	2.84551	2.25331
KIAA1468	4.85214	4.88365	4.06796	4.37296	3.41784	3.5003
KIAA1522	2.95555	2.8881	2.78803	2.87702	2.92654	2.89692
KIAA1524	4.87928	5.10794	4.75531	5.21534	4.49639	4.9789
KIAA1549	4.94882	5.36011	4.99576	4.91215	4.90526	5.01523
KIAA1549L	6.58541	6.62746	1.68687	1.8912	1.12703	0.100762
KIAA1551	5.53753	5.46712	6.63605	6.60973	4.88125	5.14736
KIAA1586	3.81493	3.82673	3.54269	3.64487	3.82901	4.08161
KIAA1614	2.33309	2.02773	2.03313	1.7288	1.31821	0.42663
KIAA1644	4.20555	4.59919	2.99141	3.68558	-4.33548	-1.18284
KIAA1671	0.566464	0.206981	4.42897	3.85987	3.51218	2.41742
KIAA1715	4.84973	5.15507	5.97348	6.27892	5.92869	5.49724
KIAA1755	4.05412	3.96026	-2.37543	-2.40763	-4.65668	-5.69922
KIAA1841	2.22674	2.11303	2.84352	2.72436	3.20217	3.37246
KIAA1919	3.44047	3.50149	3.49948	3.49851	4.14623	3.87637
KIAA1958	3.2449	3.02901	2.97635	2.53504	4.23146	3.73416
KIAA2013	4.80377	4.7051	4.30439	4.7169	5.94529	5.77228
KIAA2018	4.20404	4.18998	4.16188	4.30796	3.91334	4.10546
KIAA2026	3.97688	3.92301	4.26666	4.1391	5.30619	5.073
KIDINS220	5.81685	5.72603	5.83502	5.93387	6.34704	6.25401
KIF11	6.23405	6.36389	5.7472	6.03964	6.02552	6.19527
KIF13A	5.43756	5.52087	4.80089	5.42353	6.31925	6.64144
KIF13B	4.26828	4.2402	3.67463	3.852	4.60937	4.21325
KIF14	6.33105	6.46787	5.68066	5.98689	5.72339	5.83535
KIF15	5.24598	5.32587	4.77576	4.97796	4.46767	4.74963
KIF16B	4.84393	4.89029	3.93801	4.11758	2.94517	2.98329
KIF18A	4.58342	4.67797	4.69452	5.145	4.65029	5.33219
KIF18B	4.86746	4.76778	4.46931	4.59421	4.55581	3.99026
KIF1B	6.00373	5.94944	6.36239	6.38949	6.90465	6.43091
KIF1BP	4.04743	3.9976	3.64937	4.00361	4.17019	4.32395
KIF1C	5.47832	5.28912	5.71921	5.901	6.62043	6.1644
KIF20A	6.23403	6.28197	5.89162	6.43275	5.88877	5.88526
KIF20B	6.10564	6.1816	5.81751	6.17337	5.93421	6.32327
KIF21A	5.10342	4.98939	4.70977	4.60541	4.42516	4.15747
KIF21B	-1.65424	-1.65505	2.42752	2.61228	-5.65305	-5.69922
KIF22	5.38035	5.42851	5.01156	5.25155	4.78956	4.85738
KIF23	6.38666	6.33924	5.85394	6.1768	6.27961	6.14771
KIF24	3.58204	3.69069	3.61108	3.80254	4.21014	3.81901
KIF26B	-0.272655	-0.707737	2.19153	1.9191	3.01565	3.64423
KIF27	1.89343	1.923	2.05281	2.33817	0.0396157	0.50283
KIF2A	4.85598	4.85569	5.11681	5.39398	5.19845	5.55949
KIF2C	5.74679	5.95215	5.76676	6.30974	5.65032	5.92849
KIF3A	4.65019	4.58222	4.36144	4.32475	4.24957	3.6813
KIF3B	6.24196	6.17309	6.12708	6.31585	6.71254	6.4018
KIF3C	3.70179	3.77913	3.13841	3.21085	3.91428	3.55068
KIF4A	5.32067	5.4406	5.1506	5.5061	5.77761	5.92256

KIF4B	2.36567	2.51196	2.12143	2.55834	2.76874	2.67331
KIF5A	1.60016	2.00478	1.78229	2.50221	1.99076	1.90064
KIF5B	7.37866	7.33267	6.70426	6.98672	7.6211	7.46633
KIF5C	5.52549	6.00246	2.59785	3.63094	2.52706	3.47568
KIF7	2.55773	2.23311	3.72557	3.95527	2.16798	1.64122
KIF9	1.15677	1.12027	0.281265	0.676592	1.48651	2.33464
KIFAP3	4.45887	4.43422	3.09494	3.28349	3.79706	4.30591
KIFC1	4.97575	4.91584	4.52996	4.85329	4.87298	4.66
KIFC2	2.40927	2.08694	3.12831	3.17111	2.66392	2.19342
KIFC3	4.00362	3.88361	4.35467	4.88003	4.97811	5.07135
KIN	2.7542	2.71707	2.44585	2.36692	2.84153	2.85554
KIRREL	7.4628	7.2325	7.24439	7.34731	7.58538	7.40955
KIRREL3	2.07243	2.53435	-0.568612	-0.226029	1.90957	2.69844
KITLG	3.98567	4.36131	4.55033	4.07481	3.38272	4.23139
KIZ	3.33984	3.39668	2.76485	2.97598	3.75013	3.92628
KLB	1.03569	1.06077	-0.69145	-0.301307	1.26455	0.422664
KLC1	6.22492	6.14081	5.31512	5.38493	6.09135	5.64878
KLC2	3.55934	3.52958	3.0581	3.33565	3.37337	3.02364
KLC4	2.46174	2.43012	2.40519	2.56483	2.99289	2.88929
KLF10	3.97864	4.15002	4.88186	4.83808	4.24505	3.95327
KLF11	3.79377	3.75389	1.7294	1.65939	2.22845	1.99375
KLF12	3.92022	3.85569	3.7663	3.76308	4.40509	4.25329
KLF13	2.91564	3.28031	4.74054	5.0994	3.69393	3.69203
KLF16	1.63634	1.39518	1.0357	1.51326	2.38955	2.354
KLF3	4.10744	3.83178	3.90362	3.75381	5.17453	4.61745
KLF4	1.62738	0.996642	0.371169	0.773887	-1.75295	-1.31405
KLF5	3.44685	3.64175	2.0786	2.52781	2.0332	1.40185
KLF6	4.53156	4.4701	4.78296	5.15268	4.47541	4.24894
KLF7	3.62171	3.90566	3.45129	3.71295	4.81439	4.4174
KLF9	6.56799	6.0558	4.83049	4.84026	4.17258	3.581
KLHDC10	5.41205	5.42893	5.08326	5.41423	5.46864	5.49968
KLHDC2	3.84735	3.97577	2.87194	3.04753	3.7853	4.27951
KLHDC3	5.06083	4.76657	4.4052	4.44767	6.24001	6.23467
KLHDC4	3.89897	3.86021	3.52728	3.56005	3.58524	3.47568
KLHDC8B	2.42764	2.19538	2.46754	2.51324	1.78266	2.06149
KLHL11	3.27103	3.4198	3.39579	3.65106	4.05393	4.10546
KLHL12	4.61284	4.55067	4.07308	4.16263	4.25445	4.46537
KLHL13	1.07353	1.10179	-4.18092	-6.09892	5.41693	5.37436
KLHL15	2.84782	3.03101	3.27796	3.49851	3.26747	3.49786
KLHL17	1.84784	1.53719	1.70525	1.91357	2.51712	2.09421
KLHL18	4.39851	4.62115	3.40148	3.87399	4.53674	4.64186
KLHL2	2.6208	2.994	2.73701	3.24389	3.52219	3.56441
KLHL20	4.088	4.19714	3.56802	3.70014	4.07016	4.16057
KLHL21	4.81365	4.76175	3.9393	4.14856	5.15826	4.67158
KLHL22	3.39756	3.4826	3.60438	3.61806	2.14706	2.02123
KLHL23	4.57934	4.84315	2.81392	2.63407	3.8767	3.81223
KLHL24	4.22572	4.09558	4.30431	4.39698	5.17059	5.5263
KLHL25	2.59095	2.52869	2.66198	2.74293	1.57334	0.922708
KLHL26	0.693311	0.548466	-1.53926	-0.899117	1.01923	0.578775
KLHL28	3.68041	3.72828	2.24695	2.18123	4.37841	4.00236
KLHL29	3.67705	3.67927	1.68687	1.9191	1.7191	1.80902
KLHL36	5.07446	4.93589	4.59043	4.57679	4.20309	3.73623

KLHL4	-6.68418	-5.45651	1.26773	2.01532	1.38413	2.57407
KLHL42	4.8901	5.11646	4.54948	4.75535	4.70601	4.94613
KLHL5	4.68849	4.77741	4.93389	5.32219	5.40742	5.52997
KLHL7	4.42373	4.50798	4.454	4.69287	4.09625	4.5257
KLHL7-AS1	0.0289651	0.0918691	1.63029	1.65939	-1.61549	-1.61882
KLHL8	3.79477	3.86245	3.31832	3.57066	3.58524	3.46571
KLHL9	4.96002	5.02749	4.71507	4.95321	5.80983	6.23028
KLRAP1	0.930336	0.444137	1.52387	1.59165	0.86332	-0.0341544
KMO	-0.402845	-0.137938	0.61421	0.93876	-0.00296634	0.312433
KMT2A	6.96103	6.88812	7.33979	7.29206	6.4559	6.14269
KMT2B	3.47585	3.2219	4.21628	4.0127	3.67954	3.27913
KMT2C	7.03112	6.90047	7.13693	6.89954	7.41805	6.94348
KMT2D	5.76227	5.4504	5.77691	5.53114	5.13953	4.95615
KMT2E	5.40618	5.35412	5.26272	5.22313	5.59457	5.46156
KNOP1	5.21551	5.15427	4.4937	4.57839	4.9489	4.68556
KNSTRN	5.5662	5.65837	4.27693	4.9163	4.80013	5.22401
KNTC1	6.02434	6.03399	6.33411	6.4641	5.50772	5.49234
KPNA1	5.09098	5.05696	5.21177	5.40839	5.45008	5.36634
KPNA2	7.35664	7.27449	6.99067	7.43194	7.99241	8.00636
KPNA3	5.03096	5.16968	5.0984	5.4596	5.30221	5.41481
KPNA4	5.47374	5.37617	5.87428	6.16655	6.45021	6.30318
KPNA5	2.61381	2.82488	2.37491	2.69854	3.65597	4.53168
KPNA6	6.17465	6.75483	6.03443	6.89025	6.24477	6.9226
KPNB1	8.70795	8.52284	7.71597	7.98389	8.55433	8.5702
KPTN	1.2317	0.920926	-0.183372	-0.327299	1.06767	0.769095
KRAS	5.0215	4.96737	4.7535	4.92896	4.56158	4.17738
KRBA1	2.85743	2.74173	2.60439	2.57769	2.41117	2.0415
KRBA2	1.19395	1.28127	2.26109	2.27523	2.47902	2.27508
KRBOX4	1.70667	1.85427	3.59105	3.67858	2.94001	2.89843
KRCC1	3.2287	3.30037	3.3768	3.58292	2.8554	3.51004
KREMEN1	3.83132	3.52867	4.60356	4.69932	0.568596	0.0747657
KRI1	4.14447	4.09667	3.12212	3.34667	4.22627	3.77904
KRIT1	4.96996	4.86542	6.16629	6.13422	5.09426	4.72696
KRR1	5.28422	5.26024	5.01794	5.16674	5.11422	5.52545
KRT10	1.89714	1.88591	1.40148	1.8512	3.23299	3.39657
KRT15	-1.20691	0.265185	-2.48229	-0.751045	1.21626	3.19114
KRT18	6.25064	6.54808	5.7897	6.20864	1.78625	2.23709
KRT18P11	1.07572	1.66184	0.680972	1.26844	-3.01585	-5.53632
KRT18P15	0.744299	0.675753	1.84772	1.5274	0.233459	-0.500516
KRT18P31	1.25538	1.12963	2.00137	1.55655	0.947083	0.463298
KRT18P57	0.62293	0.491443	0.571444	0.317536	-0.530833	-1.53638
KRT7	0.258193	0.533025	-0.275275	0.637694	-0.941116	-0.103928
KRT8	1.63354	1.8143	1.39394	1.15211	-1.98063	-1.34115
KRT80	2.45068	2.93591	2.39769	3.76307	4.34039	4.8305
KRT81	-1.53421	-2.03355	3.37115	4.70695	7.42605	8.52866
KRT86	-4.07937	-3.56131	0.317455	0.691749	0.69215	1.87929
KRT87P	-6.68418	-4.87366	-4.94564	-2.97969	0.894721	2.46933
KRT8P12	0.939512	0.831845	1.34853	1.40736	0.824589	0.294982
KRT8P3	-0.471271	0.0375931	1.08829	1.1388	-0.522462	-1.39435
KRT8P33	0.584857	-0.0174317	0.466162	0.32277	-0.00779861	-1.12145
KRT8P39	1.02208	0.938098	0.156448	0.020584	-0.285108	-1.31405
KRTCAP2	4.52543	4.57332	3.98747	4.241	4.54792	4.74717

KSR1	3.14588	3.37859	1.52837	1.63266	3.28223	2.91309
KSR2	3.95911	3.54977	?	?	-0.530833	0.0213376
KTI12	2.44871	2.56045	2.21308	2.55984	2.82543	2.98173
KTN1	7.98958	8.13289	7.54707	7.69762	8.47754	8.56413
KTN1-AS1	0.878411	0.794611	1.04535	0.845438	0.568596	1.0747
KXD1	4.17788	4.12159	4.0125	4.39074	5.20493	5.34531
KYNU	5.70481	5.33845	5.46585	6.64828	-2.95876	-2.70579
L1CAM	5.94622	5.95541	4.30109	5.01108	-2.13612	0.151378
L2HGDH	3.1824	3.07323	2.19581	2.10867	2.80291	2.8355
L3HYPDH	3.21062	3.37564	2.93609	2.86155	3.43196	3.91767
L3MBTL1	1.13383	1.20254	2.35177	2.20407	1.40582	0.97985
L3MBTL2	4.06281	4.13457	4.30625	4.72276	4.25867	4.15496
L3MBTL3	2.54106	2.59875	2.36874	2.63506	2.21819	2.4726
LA16c-358B7.3	1.82335	1.66669	2.23205	2.20637	1.32949	0.720716
LA16c-3G11.7	0.34975	0.12071	0.255755	1.04232	1.01212	1.4099
LA16c-431H6.6	1.08699	1.06713	0.559854	-0.0568123	0.0679003	-0.547805
LA16c-431H6.7	1.36705	1.3764	0.517004	0.383121	0.0812851	-0.95156
LA16c-60H5.7	1.8132	1.75161	1.60636	1.95164	2.45591	2.16543
LACC1	2.50841	2.45401	2.27788	2.64883	-0.0602604	0.406526
LACE1	0.792847	1.06859	1.76487	1.98923	2.08454	2.34315
LACTB	3.26959	3.44404	4.01737	4.48736	4.56369	4.75373
LACTB2	1.36689	1.66935	-3.37468	-2.97055	1.79986	2.45603
LAGE3	2.21682	2.31196	1.14745	1.23107	3.24357	4.03305
LAMA2	-1.94365	-1.80443	-1.53926	-1.20162	3.27938	3.81046
LAMA3	-3.2383	-3.87578	1.65571	1.90242	0.305574	0.100762
LAMA4	8.19867	8.67074	6.18845	6.08137	5.73703	6.27403
LAMA5	1.86601	1.40129	1.18136	1.0629	5.12207	5.28687
LAMB1	8.80809	9.07487	9.21147	8.98382	7.30131	7.32763
LAMB2	5.96313	5.62063	5.15017	5.32891	6.30058	5.66832
LAMB3	2.26672	2.68182	4.44126	5.50957	3.31698	4.00925
LAMC1	9.59586	9.41388	8.39347	8.73227	8.26988	8.21334
LAMP1	5.40556	6.47276	5.4942	6.58779	5.12431	5.83535
LAMP2	6.73421	6.85996	6.54376	7.16877	8.74241	8.77177
LAMTOR1	4.85454	4.56642	4.10534	4.3048	4.59472	4.60843
LAMTOR2	3.55121	3.61264	2.86905	3.19038	3.10622	3.60389
LAMTOR3	3.53932	3.68308	3.05965	3.57066	4.08704	4.79809
LAMTOR4	4.14028	4.16902	3.74379	4.16093	4.31298	5.24716
LAMTOR5	3.98478	4.1296	3.74826	4.17826	4.38818	4.93623
LAMTOR5-AS1	2.9168	2.96903	2.68135	2.35337	2.25552	1.82366
LANCL1	5.03306	5.27354	6.05875	6.30484	5.47924	5.74911
LANCL2	3.49936	3.43349	2.67757	2.87702	3.65143	3.99892
LANCL3	0.399051	0.868134	-4.18092	-6.09892	1.20392	1.77713
LAP3	4.18105	4.32045	4.54018	5.17337	5.51921	5.61717
LAPTM4A	5.50824	5.27017	5.59619	5.86271	5.74748	6.15514
LAPTM4B	7.0926	7.11501	5.78945	5.66508	6.47548	6.53196
LAPTM5	-2.52833	-3.00228	5.93961	6.3271	-0.987612	-1.61882
LARGE	4.12747	4.41803	2.15635	1.92688	-1.80184	-2.38405
LARP1	6.70578	6.79629	7.58537	7.85704	7.43252	7.0471
LARP1B	3.81558	3.94009	3.06191	3.4534	3.60395	3.57405
LARP4	6.64987	6.70029	6.08653	6.40219	6.14504	6.04519
LARP4B	5.43952	5.39864	4.74794	4.82633	5.52267	5.21586
LARP4P	1.50673	1.58941	0.609112	0.820306	0.870061	0.383132

LARP6	3.19261	3.21625	3.42187	4.24199	4.40037	4.90061
LARP7	4.16051	4.10195	3.74269	3.8855	4.23073	4.73441
LARS	7.4072	7.43657	6.32944	6.69045	7.2434	7.43889
LARS2	5.00352	5.08067	4.18716	4.47988	4.72178	4.77003
LAS1L	4.46265	4.46938	4.86971	5.11213	5.1828	5.00753
LASP1	5.39432	5.37479	6.21035	6.85279	6.0563	6.3523
LAT2	0.345419	0.456112	-0.300715	0.139221	-0.0308197	-0.38461
LATS1	4.75619	4.75868	4.54758	4.57669	5.44224	5.16896
LATS2	3.67378	3.66514	3.62543	3.83515	3.6251	3.21513
LAYN	-5.69162	-5.45651	3.57136	4.14621	6.29907	6.80971
LBH	2.0921	2.1482	-1.229	-1.58485	-4.33548	-3.12051
LBHD1	2.46685	2.70226	1.98072	2.3063	1.57615	1.17593
LBR	6.4001	6.65071	6.42729	6.8145	6.08496	6.20165
LCA5	2.59326	2.71708	2.07859	2.20407	3.38135	4.05815
LCAT	0.989372	0.693614	1.51822	1.43042	1.16187	0.3684
LCLAT1	5.41303	5.38067	4.34394	4.33308	5.06419	4.95772
LCMT1	2.55596	2.51445	2.50299	3.15327	2.66388	3.01439
LCMT1-AS2	1.70609	2.02099	2.64938	2.52781	1.48948	0.736302
LCMT2	3.63184	3.62063	2.64304	3.1603	3.13248	3.42257
LCN2	5.74954	6.4244	-3.59683	-3.78492	-6.64581	-5.69922
LCOR	4.56007	4.67158	4.64699	4.84464	5.13856	5.1782
LCORL	4.50087	4.48196	4.32853	4.33474	4.84343	4.86186
LCP1	0.312247	2.01696	-3.18222	-1.15432	-0.136602	0.50283
LCTL	1.82812	1.68092	-0.3852	-0.174346	3.31199	3.5482
LDAH	4.155	4.05481	3.63025	3.67744	4.0642	4.3087
LDB1	3.73973	3.57744	4.61495	4.67252	4.97952	4.66833
LDB2	-0.144345	0.20969	3.44746	3.32051 ?		-5.69922
LDHA	8.79795	8.88337	9.1006	9.42938	3.49357	3.84492
LDHAP2	1.62619	1.49578	1.98751	2.28802	0.232071	0.229785
LDHAP3	0.140792	0.364079	0.842858	1.40813	-4.44942	-4.06289
LDHAP4	4.48065	4.67163	5.95315	6.19537	-0.162623	0.42562
LDHAP5	1.71865	1.64662	2.23792	2.50709	-4.07293	-3.70484
LDHAP7	2.31884	2.73276	3.35338	3.54364	-2.69678	-2.8355
LDHB	8.42579	8.31844	7.62078	8.03524	7.9161	8.58726
LDHBP2	3.80296	3.80297	3.09067	3.54432	3.31769	3.52229
LDLR	6.97267	6.90399	6.22054	6.59333	6.65515	6.55212
LDLRAD3	4.60641	4.61063	2.03082	2.39898	4.02618	4.08281
LDLRAD4	-1.61119	-2.46206	1.92897	1.13916	3.57323	2.87407
LDLRAP1	2.63855	2.9529	2.72338	3.26621	3.08116	3.18203
LDOC1	-1.20691	-1.87737	1.82213	1.7367	3.98805	3.85362
LDOC1L	5.14405	5.23281	4.13275	4.58465	4.56368	4.49663
LEAP2	1.83233	1.61934	2.48892	2.02562	1.60651	0.752791
LEF1	4.79695	4.61324	4.03102	3.61695	2.2215	2.36488
LEMD2	4.37972	4.33886	4.86836	5.11215	4.81186	4.61633
LEMD3	4.96091	4.93482	3.99514	3.9826	4.39429	4.28905
LENG1	0.998578	0.81295	0.94152	1.11479	1.28962	1.63917
LENG8	4.49292	4.28085	5.54355	5.49218	4.97621	4.31212
LEO1	4.52067	4.55816	3.93412	4.19153	4.64462	4.48557
LEPR	1.68866	1.98628	2.36167	2.50089	3.72564	4.72278
LEPROT	5.29071	5.13587	5.47622	5.78725	5.22766	5.72527
LEPROTL1	4.12091	4.54559	4.00639	4.76076	4.35236	5.03709
LETM1	5.40746	5.37242	4.67596	4.97597	6.02945	5.74036

LETM2	1.11013	1.25593	0.913715	1.31855	-0.987612	-0.433764
LETMD1	4.35958	4.25568	4.52642	4.6097	3.78159	3.59934
LFNG	-0.768111	-0.847889	1.20884	1.78602	0.954457	1.97981
LGALS1	7.88754	7.76869	7.16567	7.82774	7.13126	7.66204
LGALS3	3.21382	3.3429	3.81788	4.5169	2.86912	3.79114
LGALS3BP	7.37718	7.26038	7.88814	8.00777	8.3849	8.33491
LGALS8	4.83236	4.8856	4.99586	5.36685	5.3362	5.41856
LGALSL	3.18966	3.3103	1.65571	1.92462	0.855456	0.951558
LGMN	4.21573	4.05434	2.79263	3.20632	3.29764	3.36206
LGMNP1	1.05213	0.933754	-0.15225	-0.275776	0.493709	-0.497071
LGR4	4.33916	4.14725	4.80586	4.56271	5.43447	5.81735
LHFP	4.1782	4.30119	1.93025	2.27921	4.6158	4.1805
LHFPL2	5.90574	5.69926	5.10358	5.46619	6.34572	6.19568
LHX4	3.18055	3.07478	2.68201	2.66172	1.89131	1.22788
LHX8	-2.43244	-1.87737	1.12941	0.957328	?	?
LIAS	1.27825	1.40756	1.33222	1.69856	2.44036	2.41742
LIF	8.80317	9.01847	3.45375	4.09304	4.06831	3.97431
LIFR	4.60886	4.62993	8.0625	7.64038	5.30291	5.36428
LIFR-AS1	-0.925423	-1.03746	1.86471	1.19182	-1.53062	-1.26753
LIG1	4.31633	4.2253	4.30673	4.29507	4.3111	4.27484
LIG3	5.55313	5.53747	4.77549	4.83392	5.02778	4.77785
LIG4	3.74904	3.6907	3.75458	3.85408	4.73576	4.74862
LIMA1	6.68787	6.66223	6.4736	6.88888	6.00904	6.16286
LIMCH1	5.48641	5.75479	7.0055	6.45627	8.37542	7.98011
LIMD1	4.57521	4.45358	4.49757	4.68169	5.24498	4.99696
LIMD1-AS1	0.736925	0.998802	0.342714	1.06904	1.45959	1.7384
LIMD2	3.50298	2.78892	3.39834	3.21513	0.83825	0.453607
LIME1	1.38363	1.23533	1.04705	1.06003	1.18666	0.869232
LIMK1	3.23018	3.03502	5.05667	5.41815	5.23678	5.4161
LIMK2	3.43292	3.35896	3.16303	3.0933	2.13097	2.11361
LIMS1	4.89171	5.43043	4.72741	5.32424	5.59971	5.6758
LIMS3	-0.307737	0.415001	-0.821327	-0.0448256	0.196985	0.295323
LIN28B	2.17746	2.49717	-2.48229	-4.52056	-5.65305	?
LIN37	1.38903	0.865167	1.99445	2.26685	2.1785	1.9963
LIN52	2.90825	2.82256	1.78229	2.37096	2.75121	3.11984
LIN54	4.20027	4.29037	4.21949	4.57153	4.22538	4.0212
LIN7C	5.16428	5.55635	4.76188	5.39549	5.97896	6.231
LIN9	3.23903	3.37004	3.15855	3.4342	3.10953	3.98503
LINC-PINT	4.46268	4.23932	4.97697	5.1938	4.03056	3.93714
LINC00052	-2.69815	-2.87684	-3.18222	?	2.45605	2.78915
LINC00094	2.93756	2.84363	3.0966	3.30928	3.40758	3.29794
LINC00116	1.11475	0.666511	0.623914	1.48367	1.71041	3.30731
LINC00152	4.28106	4.0501	4.12631	4.35325	4.53562	4.28291
LINC00174	0.840145	0.727049	1.95605	1.82784	0.670686	-0.536593
LINC00176	0.409538	0.491443	0.431281	-0.000171691	-0.338214	-0.280287
LINC00205	0.345419	0.525929	2.00137	2.10065	0.901996	0.33778
LINC00216	1.04203	0.954591	0.460428	0.300663	0.458718	-0.0918635
LINC00235	1.68033	1.76119	1.09265	1.48367	0.305574	0.422664
LINC00265	0.395392	0.578948	0.639788	0.336478	-0.160869	0.0229714
LINC00294	3.20868	3.44761	1.56295	2.23367	2.70669	3.01039
LINC00310	2.06249	1.77559	3.08794	2.64941	0.625168	-0.421131
LINC00324	0.460879	0.432061	0.0260635	0.476226	0.0263185	0.97985

LINC00326	1.85937	1.17383	-2.72315	-0.938639	-2.41163	-2.70579
LINC00327	1.61384	1.39518	-2.72315	-2.64851	-1.70566	-1.18284
LINC00339	2.57705	2.32507	2.62706	2.70817	1.86719	2.10066
LINC00342	0.753664	0.968238	1.508	1.42215	1.77069	0.852039
LINC00346	3.46959	3.19	2.23435	2.38704	3.70492	3.23287
LINC00355	1.00834	1.06859	-0.117291	0.020584	?	?
LINC00460	1.82451	2.10703	-5.1783	-6.09892	2.2008	2.75273
LINC00461	2.12112	2.29873	2.8193	3.07853	7.36643	7.40986
LINC00467	1.05586	0.97184	2.17844	2.47615	1.64806	2.16982
LINC00470	2.60471	2.48554	-3.37468	-3.78492	?	?
LINC00472	3.97467	3.464	2.88257	3.05755	3.5537	3.31367
LINC00473	4.53358	4.37452	-5.1783	-6.09892	-0.466855	-0.0133459
LINC00476	1.90527	1.72789	1.34056	1.19681	1.27304	1.60882
LINC00493	4.07546	4.1316	3.23955	3.72072	4.06398	5.0543
LINC00511	5.04869	5.17739	2.9066	3.25312	5.26226	5.12141
LINC00536	-4.11163	-3.46127	1.81651	2.37903	-0.470299	-0.38461
LINC00601	-0.191134	-0.681268	1.13846	1.82784	1.45867	1.97279
LINC00622	2.88213	2.9529	-1.65952	-0.786664	-0.878698	-0.577229
LINC00623	1.47181	1.13697	0.271115	0.573045	0.248134	0.409527
LINC00624	2.26606	2.33463	1.56118	1.31141	1.12369	0.377146
LINC00630	1.71458	1.64021	2.37108	2.3602	2.05932	1.56011
LINC00639	-3.8896	-4.45968	-6.17309	?	3.18269	2.97858
LINC00641	3.79777	3.71082	3.39955	3.24653	3.81339	3.11027
LINC00657	8.14149	8.08933	7.65094	7.91006	7.72	7.42449
LINC00662	2.13164	2.34051	3.4081	3.5252	3.52677	3.66146
LINC00665	3.28361	3.19238	1.72026	1.67807	0.0116674	-0.4586
LINC00667	-6.68418	-6.45019	4.20105	4.30802	4.91762	4.71235
LINC00680	2.26897	2.27013	2.22306	2.53112	2.53319	2.34839
LINC00704	0.880497	0.988427	-2.18288	-0.716284	0.799187	1.03481
LINC00707	2.06581	2.87032	-3.01245	-1.78641	1.31701	2.00067
LINC00839	1.85553	1.5651	1.55798	1.93559	0.270529	0.0483059
LINC00847	0.463403	0.522337	0.81646	0.939655	1.84875	2.26364
LINC00852	0.195852	0.491443	0.474784	0.176693	-0.470299	-1.4584
LINC00857	1.17133	1.01295	-0.825729	0.0410467	-0.0454657	0.463298
LINC00863	0.966128	0.936493	1.57227	1.42996	1.29497	1.04558
LINC00869	2.80447	2.73749	1.89024	1.92969	2.46648	1.92431
LINC00882	-2.17488	-3.87578	-0.790968	-0.979274	2.35651	2.677
LINC00883	2.38042	2.39204	2.85814	3.38703	2.50712	2.78114
LINC00886	2.02204	1.84096	0.860939	1.07114	-5.07051	-5.69922
LINC00888	1.30659	1.47941	1.75314	2.24434	1.66165	2.32672
LINC00893	-0.318925	-0.715273	0.680909	0.710781	0.522257	0.295147
LINC00894	0.217268	-0.030547	0.984903	0.765212	1.13756	0.318369
LINC00899	-0.611411	-0.847889	1.14744	1.1104	0.246688	0.247587
LINC00909	2.59095	2.36372	1.99143	2.26623	1.18523	1.33772
LINC00920	0.930336	1.07637	0.308431	0.367002	1.02625	1.86317
LINC00941	0.243547	0.766044	-0.325375	0.020584	-0.802092	-0.31423
LINC00942	-2.37642	-2.6546	4.4005	4.71851	-1.65987	-2.38405
LINC00960	0.987481	0.938098	-1.93506	-2.30078	?	?
LINC00961	-1.94365	-3.46127	-0.723865	-1.02108	3.23602	3.29891
LINC00963	3.83303	3.49346	3.25527	3.47989	5.23126	4.39525
LINC00969	3.5527	3.45302	5.39308	5.40522	5.46767	5.02471
LINC00973	-0.721575	-1.55554	-0.723865	-0.0426024	2.64804	3.2358

LINC00998	4.22426	4.11741	2.96114	3.2131	3.37311	4.23286
LINC01001	1.67629	1.37807	0.543417	0.397912	-0.0756051	-0.653051
LINC01002	1.72579	1.70632	1.39116	1.38776	0.27664	-0.618965
LINC01004	0.108397	0.382745	0.488988	0.231137	-0.932127	-0.706491
LINC01089	2.01522	1.88696	2.10326	2.11716	1.31367	1.37306
LINC01116	2.28667	2.63732	2.04493	2.47223	2.79697	3.65726
LINC01119	0.255223	0.727058	0.208904	0.176693	0.499649	0.615294
LINC01128	2.26773	2.14974	1.72016	2.11431	3.04446	2.6853
LINC01137	0.0824028	0.603321	0.793829	1.4458	-0.490197	-0.0918635
LINC01138	2.36947	2.20897	2.07423	2.19205	1.75155	0.994587
LINC01184	3.16201	3.31851	3.39579	3.50956	3.54304	3.84979
LINC01224	-1.7917	-1.81849	1.95605	2.40294	-0.393347	-0.799586
LINC01232	0.999011	0.871449	2.65782	2.50589	-0.173838	-0.577229
LINC01268	-1.8406	-2.1403	-1.93506	-1.40802	3.66048	3.34857
LINC01278	1.21386	0.859151	1.76487	1.43037	1.56854	1.17598
LINC01291	1.8934	1.87257	0.584741	1.02051	1.60651	1.50278
LINC01296	3.74485	3.78221	2.19339	1.64607	3.79895	4.06202
LINC01311	1.09539	0.90354	-0.206089	-0.108685	-0.682812	-0.706491
LINC01347	0.256141	-0.257609	0.481526	0.280196	0.311898	-0.843966
LINC01355	4.3824	4.51443	3.24274	3.30269	3.97993	3.73207
LINC01410	1.53341	1.58221	0.499149	0.486272	1.34621	1.14509
LINC01419	2.56924	2.96076	-6.17309	-3.10759	-3.85089	-4.70296
LINC01420	1.867	1.9295	1.92503	2.42646	2.88273	3.46322
LINC01426	2.0921	2.27188	-0.897866	-0.786664	-3.33694	-2.8983
LINC01444	?	?	0.558032	1.64609	-0.959604	1.00759
LINC01447	2.32607	2.64803	-1.42826	-1.78641	-5.65305	-3.70484
LINC01465	1.72721	1.64571	0.759062	1.01017	-0.470299	0.151378
LINC01468	-2.52833	-3.65369	?	?	1.67957	1.9868
LINC01481	1.06521	0.633087	-0.0126762	-0.177162	-0.186485	-0.63717
LINC01515	2.44048	2.35735	-6.17309	?	1.99076	1.47324
LINC01521	1.27825	0.996649	0.996447	0.414828	-0.23389	-0.848487
LINC01551	-2.52833	-3.65369	1.25947	1.78602	3.0625	3.25039
LINC01572	0.317431	0.0273664	0.993312	0.901417	0.615934	-0.0428625
LINC01578	4.6648	4.67065	5.05145	4.83703	5.3221	5.18249
LINC01579	0.727258	0.938098	?	-6.09892	2.93775	2.98329
LINC01604	0.752201	0.278722	0.558032	0.605504	0.947083	0.633217
LINGO2	-0.359059	-0.333394	?	?	1.78266	2.14505
LINS	3.54066	3.45154	3.02837	3.31324	2.89266	3.0178
LIPA	5.57531	5.476	5.54272	5.76574	5.82528	6.36642
LIPG	-1.56938	-1.37502	?	-6.09892	1.40043	2.5925
LIPH	-2.37642	-1.33321	0.120378	0.367002	1.98357	1.92993
LIPT1	0.906567	0.939603	0.842963	0.869808	0.815789	1.29519
LITAF	5.19459	4.77077	5.00506	5.09145	4.52457	4.01609
LIX1L	4.42317	4.56636	4.16091	4.36609	4.71903	4.59038
LL0XNC01-237H1.2	0.0692347	0.0299405	1.11115	1.12634	1.26713	0.878317
LL0XNC01-7P3.1	1.30659	1.03706	1.48185	1.38307	-0.136604	-1.006
LL22NC03-80A10.6	2.31467	2.17973	2.08193	2.31929	1.76576	1.64809
LL22NC03-86G7.1	4.2398	4.19902	3.69901	3.82296	3.07615	2.58653
LL22NC03-N14H11	1.80874	1.98426	0.871828	0.505972	1.02625	0.560157
LL22NC03-N64E9.1	2.03794	2.34647	1.076	1.10816	1.21475	0.532407
LLGL1	3.26526	3.09749	3.74277	3.99183	3.90098	4.11982
LLNLR-222A1.1	2.33898	2.16368	2.357	2.28672	2.11858	1.92916

LLPH	4.17926	4.27926	2.98018	3.50551	4.62632	4.7417
LMAN1	7.33331	7.43261	7.17043	7.67638	5.65284	5.98197
LMAN2	5.71321	5.53926	6.05368	6.38099	6.34337	6.52808
LMAN2L	3.71771	3.66386	4.32327	4.68881	4.27116	4.45694
LMBR1	5.73081	5.78426	5.90738	5.88918	4.84842	4.71799
LMBR1L	3.38041	3.2376	3.65725	3.73038	2.78885	2.677
LMBRD1	3.42763	3.55396	2.93022	3.28777	3.49071	4.25329
LMBRD2	3.39499	3.53151	4.79446	4.89398	4.90097	4.88853
LMCD1	4.74645	4.84092	1.66826	1.70499	4.77951	3.62421
LMF1	0.9596	0.645757	0.796083	-0.086319	-2.01588	-3.38329
LMF2	4.24342	3.95817	5.13782	5.25692	1.69286	1.87827
LMLN	1.7928	2.02502	3.43113	3.79651	3.46639	4.08605
LMNA	7.59802	7.47259	6.78712	7.27609	6.65907	6.83342
LMNB1	5.9551	6.06119	5.94897	6.13287	6.0068	6.05316
LMNB2	6.20405	5.88973	5.65568	5.89327	6.55856	6.08118
LMO3	-0.840854	-0.847889	2.09727	2.36286	5.11447	5.10626
LMO4	4.58458	4.24537	4.15744	4.39499	4.39564	5.32326
LMO7	6.03239	6.22796	4.8965	5.07634	5.05854	4.83697
LMTK2	5.48888	5.28282	5.53329	5.3935	4.96036	4.44557
LNP1	0.414449	-0.113543	0.642443	0.595991	0.788126	0.85366
LNPEP	5.96901	5.91	6.79211	6.85803	6.47711	6.46504
LNX1	0.925331	0.984713	0.679433	0.997784	2.63422	2.92497
LNX2	2.61608	2.53998	3.06915	3.36488	2.78264	2.71956
LOH12CR1	2.92481	2.91002	2.29223	2.02048	2.16943	2.5925
LONP1	5.13853	4.81385	4.63824	4.85984	5.60618	5.54417
LONP2	5.7215	5.829	6.08493	6.39964	6.89129	6.99185
LONRF1	2.00485	2.00478	2.68993	2.93284	1.83956	2.2002
LONRF2	5.43211	6.10199	0.711372	1.32585	4.24432	3.85933
LONRF3	0.195852	-0.508457	0.914572	1.0511	1.32273	1.1637
LOX	1.52511	1.58948	5.61261	6.16483	3.56318	2.06187
LOXL1	-5.69162	-5.45651	4.24797	3.84246	1.99433	2.39465
LOXL1-AS1	-6.68418 ?		2.26358	2.51324	1.14712	2.08361
LOXL2	8.14027	7.91334	8.23795	8.16006	7.40975	7.04232
LOXL3	7.20169	6.72898	2.49297	2.5031	3.23864	3.0604
LOXL4	-0.578973	-1.105	1.00237	0.773887	2.09125	0.749826
LPAR1	-4.11163	-4.13839	4.40801	4.97132	-6.64581 ?	
LPAR6	2.02204	2.02099	2.4052	2.32164	2.83155	3.01438
LPCAT1	5.26634	5.08979	4.45851	4.84754	4.92604	4.89506
LPCAT2	-2.45038	-1.93875	1.85174	2.96594	4.97618	5.3469
LPCAT3	4.00972	3.75879	4.01369	4.14696	3.05684	2.99882
LPCAT4	2.90641	2.99043	3.09493	3.20632	3.11448	2.63759
LPGAT1	6.64346	6.53448	6.47859	6.61732	6.13328	5.86028
LPIN1	5.2209	5.23715	5.23536	5.55206	6.03993	6.36306
LPIN2	3.01663	3.00853	6.08054	6.20501	5.81056	5.42318
LPL	2.57705	3.33158	-3.18222	-3.78492	-4.65668	-4.11926
LPP	7.04287	6.92867	6.78674	6.78953	7.5204	7.31922
LPP-AS2	2.19586	2.13906	1.93561	2.09183	1.42052	1.03898
LPPR2	2.29245	1.94232	2.81649	2.93557	3.08788	3.27628
LPXN	0.399051	0.6247	2.90394	3.94645	1.15352	1.92267
LRAT	2.78071	2.90567	-3.59683 ?		0.427241	0.878317
LRBA	6.05791	6.01446	6.09181	6.0754	6.04142	6.03207
LRCH1	4.10019	4.18281	3.98263	4.03072	3.6274	3.49786

LRCH2	-0.272655	-0.39658	-6.17309	-3.10759	3.39768	3.2358
LRCH3	4.43593	4.37714	4.88113	4.8107	4.98292	4.77396
LRCH4	2.32639	2.086	2.62028	2.61161	2.21257	2.17953
LRFN3	1.25516	1.15925	1.05494	0.946521	0.362161	0.422664
LRFN4	2.31173	1.99202	1.60838	2.04579	1.4128	1.86698
LRIF1	4.01511	3.94603	3.57756	3.79495	3.10257	2.89324
LRIG1	4.49554	4.68289	2.83556	2.80736	5.0785	4.56669
LRIG2	4.71398	4.66549	4.66274	4.6552	4.83051	4.48547
LRIG3	4.88351	4.98732	4.28203	4.30162	5.11281	5.10705
LRP1	4.47832	4.25397	7.03862	6.66406	2.27634	2.37545
LRP10	4.92251	4.81094	4.97318	5.12058	4.93167	5.07545
LRP11	4.15771	4.19752	5.25619	5.5426	2.92229	2.9515
LRP12	4.7256	4.90458	5.22212	5.39398	5.6745	5.74603
LRP1B	3.65296	3.24452	-1.34596	-1.78641	5.76645	5.33288
LRP2BP	2.18968	2.06856	2.01617	1.68562	1.99792	1.17598
LRP4	3.66892	3.31985	3.93665	3.41446	3.82266	3.48571
LRP5	4.21078	3.47379	4.32428	4.44191	3.05566	2.79314
LRP5L	2.3724	2.19181	2.35177	2.375	1.53448	0.863209
LRP6	7.11888	7.01989	6.27437	6.18525	6.25746	6.05607
LRP8	6.45286	6.62409	6.30424	6.67073	6.22115	6.39516
LRPAP1	5.89149	5.62062	5.15464	5.24651	5.78676	5.72006
LRPPRC	7.09886	6.95011	6.37009	6.51801	6.69502	6.85957
LRR1	3.63629	3.70875	2.61483	2.92118	3.06761	3.88689
LRRC1	2.76605	2.98978	1.66449	1.83212	3.68222	3.79917
LRRC14	3.60013	3.45303	3.85613	4.01858	3.87751	3.68889
LRRC15	4.32746	4.51586	-4.59508	-4.52056	-4.33548	-5.69922
LRRC16A	3.9618	3.96236	3.36723	3.55295	3.59708	3.83244
LRRC17	2.52202	2.0802	4.64457	4.14789	2.08339	2.06809
LRRC2	-2.30606	-2.6546	-6.17309	-5.10387	2.72772	3.29047
LRRC20	2.26383	2.03702	2.52729	2.75846	1.94334	2.48805
LRRC23	0.572114	0.151924	1.7024	1.70987	0.398263	0.127118
LRRC27	0.768595	0.666511	1.7707	1.69856	1.60182	1.71117
LRRC28	2.74801	2.81329	2.58354	2.95457	2.59944	3.19113
LRRC37A	1.51168	1.51666	1.96663	1.94148	0.536073	0.297884
LRRC37A16P	0.0266726	-0.202957	1.51514	1.62655	1.61197	1.567
LRRC37A17P	2.42249	2.42269	3.15617	3.16433	2.44403	2.4217
LRRC37A2	4.58601	4.47094	4.53635	4.32154	3.2306	2.32814
LRRC37A3	2.00988	2.11978	2.66136	2.25878	0.934705	0.514117
LRRC37A4P	4.10842	3.95094	4.61925	4.53571	4.42981	3.85731
LRRC37B	2.43035	2.2461	2.23165	2.23441	2.54392	1.98991
LRRC37BP1	3.31272	3.17852	3.59069	3.57085	4.01994	3.6506
LRRC4	1.60016	1.45605	1.51695	1.49854	0.625168	-0.00614162
LRRC40	4.03393	4.27355	4.15743	4.57065	3.75226	4.58445
LRRC41	5.34715	5.39815	6.13747	6.44235	5.47732	5.4962
LRRC42	3.73241	3.68689	3.95731	4.36892	3.44298	3.73623
LRRC45	3.18509	2.66387	2.4422	2.49481	2.81543	2.5317
LRRC47	4.28084	4.10052	3.75035	4.25529	5.15601	4.93112
LRRC48	0.749277	0.788686	0.924594	1.15625	0.718956	-0.176719
LRRC49	2.43268	2.75887	2.17271	2.65653	1.85575	3.02567
LRRC57	3.51613	3.8144	2.40141	3.11757	3.41543	3.79558
LRRC58	6.00394	6.23736	5.65331	6.14559	6.27649	6.29855
LRRC59	7.04964	7.07993	6.4582	7.06376	7.35904	7.27475

LRRC7	-0.359059	-0.604659	0.156448	-0.275776	1.96907	0.937201
LRRC70	-0.254317	0.377213	0.978701	0.708929	-0.859826	-0.546745
LRRC75A	3.05408	2.94966	2.46801	2.56615	3.53005	3.26634
LRRC75A-AS1	6.81204	6.7698	6.6629	7.06206	7.66443	8.168
LRRC8A	5.11027	5.00731	4.31732	4.51321	5.52119	5.70691
LRRC8B	4.58997	4.72855	2.08796	2.04071	4.73474	4.86028
LRRC8C	4.15967	3.7356	4.40143	4.17657	4.11283	3.54831
LRRC8D	5.40318	5.43464	5.59	6.00918	6.31306	6.35392
LRRC8E	2.25449	1.90056	2.09794	2.11936	2.01804	1.77292
LRRCC1	3.21232	3.39203	2.20451	2.45343	3.89906	4.74449
LRRFIP1	5.5383	5.75367	6.70802	6.9875	6.37738	5.97661
LRRFIP1P1	0.763352	0.774292	1.6688	1.80903	2.42945	1.61684
LRRFIP2	5.00611	5.14169	4.21628	4.61055	5.5049	5.89366
LRRIQ3	1.20786	1.5651	0.636738	0.95732	-0.959604	0.0747657
LRRK1	0.938286	1.78454	4.44442	4.3115	-0.597952	-1.53638
LRRK2	2.39897	2.58973	-5.1783	-5.10387	-1.26766	-0.497071
LRRN2	-4.69535	-6.45019	2.23435	2.40294	-3.33694	-3.70484
LRRN3	4.34259	4.08886	-0.757028	-1.15432	-0.0308211	-0.348991
LRRTM2	3.48092	3.41901	3.23084	3.08507	2.36761	2.24673
LRRTM4	2.26094	1.53154	0.649441	-0.154483	-6.64581 ?	
LRSAM1	1.85937	1.83639	1.98141	2.13437	2.24359	1.9868
LRTOMT	2.24812	2.30726	2.3173	2.26762	1.80094	1.70851
LRWD1	3.64299	3.61531	2.54441	2.94917	3.34391	3.49296
LSAMP	4.49011	4.65218	1.60441	1.61913	3.68951	4.09902
LSG1	5.13854	5.25525	5.3648	5.73193	5.92086	6.03936
LSM1	3.67971	3.69715	2.9627	3.35029	3.8843	4.57058
LSM10	2.71244	2.6483	2.29628	2.7209	3.19612	3.44302
LSM11	3.76852	3.69954	3.0501	2.98921	4.2578	3.90246
LSM12	3.66898	3.44983	3.51576	3.86126	4.88706	4.9224
LSM12P1	2.52351	2.51774	2.41517	2.71461	3.8011	3.90478
LSM14A	5.47583	5.37032	5.3667	5.59413	6.13614	5.73983
LSM14B	4.66395	4.38889	3.8839	3.81003	4.74002	4.17746
LSM2	3.06248	3.1756	2.55703	2.95996	3.65868	4.31426
LSM3	4.55195	4.5409	3.81534	4.242	4.61729	5.66164
LSM4	4.71664	4.4986	4.30531	4.60284	5.77089	6.09616
LSM5	4.94566	4.92086	4.38443	4.55561	4.95623	5.60899
LSM6	2.8107	2.83866	2.06917	2.10065	2.3593	2.29893
LSM7	2.98743	2.85457	2.02595	2.5169	3.39361	3.93353
LSM8	4.36431	4.30119	4.48181	4.55561	4.3033	4.63979
LSR	1.07576	1.05291	-0.401986	0.0410467	-0.551587	0.0213376
LSS	4.50936	4.21104	5.59698	5.8793	5.60566	5.71869
LTA4H	5.13234	5.00949	4.85145	5.02918	4.29865	4.60688
LTB4R	1.37778	1.3251	1.61741	1.49854	0.469063	-1.24695
LTB4R2	1.53634	1.24253	0.555384	1.29588	0.864572	-0.23555
LTBP1	2.58402	2.41675	2.13843	2.85985	5.73525	5.94524
LTBP2	2.96502	2.91767	0.816542	1.07114	2.14297	2.21217
LTBP3	4.44994	4.33836	3.99129	3.92399	3.59721	3.34233
LTBP4	2.13383	1.62465	2.44585	2.42257	1.83557	2.00755
LTBR	5.21902	5.17678	5.68577	5.86009	4.3453	4.41485
LTF	-5.69162	-5.45651	-5.1783	-4.10635	2.56129	3.26771
LTN1	5.5435	5.69324	6.00442	6.29602	5.9272	6.59678
LTV1	4.45763	4.37714	3.66197	4.0127	5.08283	5.27695

LUC7L	3.07736	3.03701	4.6454	4.52143	4.48689	4.19565
LUC7L2	3.97378	3.55116	3.27083	3.62636	4.44324	4.63923
LUC7L3	6.71043	6.65705	7.03275	6.9695	6.76895	6.1462
LUCAT1	2.90597	2.72366	5.60513	5.724	1.43523	0.50283
LUM	1.52013	1.54842	-0.973801	-0.716284	-1.57243	-0.38461
LURAP1L	1.76855	1.37009	4.46663	4.54132	3.40981	3.67482
LUZP1	6.08232	5.99261	5.95542	6.13627	5.95557	5.68962
LY6E	4.85357	4.87866	5.60929	5.85117	-1.65987	-2.00571
LY6G5B	2.07926	1.86618	2.30905	2.2328	2.12935	1.29783
LY6K	4.7476	4.4339	-0.161243	0.292252	-0.675012	-0.756414
LY96	?	-5.45651	1.24279	1.74295	2.14707	3.69629
LYAR	4.0994	4.13891	2.47827	3.0586	4.70654	4.70373
LYN	?	-5.45651	3.7037	4.11396	3.09124	3.15438
LYPD1	5.66406	5.51057	2.22118	2.67383	6.52095	6.90893
LYPD6	2.92846	3.29538	2.40521	2.54224	1.44301	1.97981
LYPD6B	?	-6.45019	3.55963	3.90938	?	-5.69922
LYPLA1	5.29629	5.41643	4.98539	5.21255	5.19482	4.95538
LYPLA1P3	2.26967	2.37592	1.61483	2.18556	2.03071	2.46135
LYPLA2	4.01812	3.94942	3.00832	3.40419	4.77928	5.19125
LYPLA2P1	2.18996	1.94998	1.02622	1.21881	2.76542	2.91359
LYPLA2P2	0.328503	0.173537	-0.623825	-0.137643	0.988739	1.53458
LYPLAL1	2.69325	2.59516	2.96877	3.23547	2.70602	3.2677
LYRM1	2.33505	2.12765	2.48679	2.97599	2.22702	2.96956
LYRM2	5.1863	5.16753	3.66403	4.06287	5.50635	5.97465
LYRM4	3.18814	3.26677	2.4164	2.68885	3.78677	3.93353
LYRM5	1.82125	1.82916	1.99375	2.29054	1.94883	2.57431
LYRM7	3.80375	3.83865	4.26149	4.57065	5.01916	4.98589
LYSMD1	2.80078	2.79926	2.18282	2.19497	2.62279	2.82857
LYSMD2	1.82842	2.06856	1.25115	1.76772	1.43249	1.97981
LYSMD3	3.81463	3.77316	4.25422	4.77306	5.1781	5.51786
LYSMD4	2.64401	2.35995	-1.54864	-1.64961	1.67948	1.46623
LYST	6.05911	5.62563	6.18037	6.28985	6.03827	6.27688
LZIC	3.5429	3.53149	2.61415	2.84247	3.8544	4.17133
LZTFL1	2.9627	2.61665	2.73832	2.67254	3.23754	3.36474
LZTR1	4.71083	4.60189	4.1991	4.5278	3.98804	3.80306
LZTS1	4.30374	4.29786	2.49948	2.65605	1.49454	1.70271
LZTS2	4.48563	4.27583	4.1257	4.33788	4.19207	3.88195
LZTS3	1.80478	2.32833	1.51695	2.33414	2.52458	3.03476
M6PR	7.17893	7.17949	6.35657	6.68976	7.0065	7.42668
MAB21L2	0.367103	-0.0876659	0.401532	0.685662	-0.121008	-0.95156
MACF1	8.86519	8.76081	8.78737	8.83069	10.0017	9.4865
MACROD1	1.83622	1.67161	0.460428	0.248838	0.732078	1.06153
MAD1L1	3.48455	3.3637	3.48536	3.81746	3.32553	3.22403
MAD2L1	5.66346	5.8055	4.81028	5.20562	6.14042	6.76206
MAD2L1BP	3.36702	3.38578	2.74722	3.31534	3.90479	4.52452
MAD2L2	2.94117	2.78509	2.77649	3.28349	3.03492	3.4632
MADD	4.63127	4.46345	4.94059	5.07355	4.43837	3.92082
MAEA	4.07323	4.09079	3.70978	4.15911	4.5947	4.9824
MAF	2.03226	1.8272	2.05967	2.39103	4.18675	4.12142
MAF1	4.23387	4.22628	4.24378	4.69691	4.97948	5.50637
MAFB	?	?	2.00137	2.16265	-1.20056	-1.38442
MAFF	3.53813	3.61797	1.70525	2.52055	0.749148	0.176042

MAFG	5.04666	4.91254	5.31517	5.59879	5.37388	4.78882
MAFG-AS1	1.39372	1.23069	0.759062	1.04098	-0.615709	-0.848487
MAFK	2.44724	2.44431	2.70638	3.16682	3.36295	3.04783
MAG	3.5006	3.3429	-4.59508	-4.10635	?	?
MAGEA1	4.61889	4.55117	-1.65952	-1.02108	-4.65668	-2.8983
MAGEA10	5.45382	5.54194	-5.1783	-5.10387	-4.65668	-4.70296
MAGEA12	5.64497	5.60055	-6.17309	-4.10635	6.05846	6.38392
MAGEA2	4.58565	4.34025	-5.1783	-4.77239	4.01782	3.97268
MAGEA2B	3.91868	4.31351	?	-7.14238	3.7443	3.8744
MAGEA3	6.24054	6.28502	0.210164	0.757074	2.40744	2.50269
MAGEA6	6.19017	6.18466	-1.54351	-0.370764	6.27946	6.54408
MAGEB17	2.12563	2.36386	-4.59508	-3.80198	-4.65668	?
MAGEC1	1.8934	1.38268	-2.5977	-5.10387	-6.64581	?
MAGED1	6.01127	5.89359	6.0958	6.37318	5.62479	5.65468
MAGED2	5.93957	5.84775	5.11282	5.63894	4.80882	5.28622
MAGED4	1.01716	0.622659	1.96441	2.32906	0.11274	0.560676
MAGED4B	1.15719	0.998217	1.79055	2.11477	0.180784	0.596649
MAGEF1	4.03647	3.99556	3.79804	4.04598	5.20309	5.08363
MAGEH1	2.14014	1.89033	2.86088	3.04852	-5.07051	-4.11926
MAGI1	2.77724	2.93652	2.23513	2.03172	1.41201	1.2732
MAGI2	1.94375	1.89822	-1.229	-0.938639	2.19176	2.05296
MAGI2-AS3	4.52026	4.55549	-0.482896	-0.464785	3.69162	3.95912
MAGI3	3.82056	3.70206	4.41359	4.38702	5.29896	5.38867
MAGOH	3.25074	3.32179	2.759	3.1391	3.24658	3.52211
MAGOHB	3.77766	3.74173	3.60602	3.84827	3.29232	3.32671
MAGT1	5.76034	5.97071	6.35682	6.86918	6.05881	6.33969
MAK	0.146525	0.344613	0.636738	0.350701	0.480989	-0.49244
MAK16	5.58502	5.55162	4.29798	4.59543	5.82494	5.92911
MALAT1	12.1966	11.7236	13.4103	13.0198	12.9598	12.262
MALSU1	3.43404	3.34476	3.95593	4.30477	3.90914	4.24091
MALT1	5.52967	5.61728	6.51669	6.84177	7.04885	7.21355
MAMDC2	-2.50222	-1.45032	-1.2763	-2.07775	3.73764	3.59912
MAMDC4	0.959318	0.614144	0.871828	0.491176	-0.551587	-1.38442
MAML1	4.0798	4.036	4.85196	4.75767	4.82045	4.50152
MAML2	4.31702	4.2315	5.0891	5.13791	5.13591	4.93714
MAML3	4.53429	4.57173	0.919516	1.05962	2.26712	2.20619
MAMLD1	2.02204	1.93806	1.52387	1.49112	1.85147	1.2122
MAN1A1	3.52492	3.6535	2.19587	2.47239	4.40577	4.75885
MAN1A2	6.42227	6.49152	5.97254	6.14028	7.55417	7.58762
MAN1B1	5.48018	5.42931	5.17292	5.44142	5.14865	5.26121
MAN2A1	6.0145	5.90265	5.47914	5.55294	6.72607	6.92786
MAN2A2	5.23638	5.08083	4.23311	4.30705	4.03952	3.35287
MAN2B1	4.25743	3.97097	4.16851	4.03997	4.00284	3.67138
MAN2B2	3.03563	3.15738	4.12254	4.3434	4.49261	4.37543
MAN2C1	4.26335	4.05544	4.44686	4.55743	4.23225	3.68438
MANBA	4.38904	4.53008	3.60275	3.91076	3.21927	3.17899
MANBAL	4.51281	4.32585	3.51345	3.66595	3.80424	3.69843
MANEA	3.52853	3.63256	3.39202	3.51689	4.2269	4.26194
MANEAL	3.45235	3.45021	3.08171	3.32987	3.29335	3.63741
MANF	5.96178	5.80334	5.06499	5.52416	5.35089	5.34313
MANSC1	2.89897	2.99454	-0.095919	0.46112	3.34531	3.98503
MAOA	2.52252	2.38891	-1.65952	-0.751045	-4.33548	-4.70296

MAP1A	3.26958	3.13893	2.54781	3.30269	5.16424	5.0974
MAP1B	7.94612	8.42287	8.81607	9.32945	9.86495	10.0325
MAP1LC3A	1.78476	1.31199	1.12941	1.30061	1.73203	1.75275
MAP1LC3B	4.36675	4.23916	4.61822	5.01336	4.30587	4.47011
MAP1LC3B2	1.93155	1.76687	2.11558	2.56694	1.89993	2.06467
MAP1S	1.08887	0.859151	1.2174	1.34243	1.3621	1.91536
MAP2	0.994464	1.31856	4.90326	5.13731	6.26544	5.69256
MAP2K1	4.16771	4.31639	4.20669	4.62972	5.40915	5.171
MAP2K1P1	0.0283428	0.600374	0.282143	0.69562	1.5194	1.26448
MAP2K2	4.54613	4.2222	3.83642	4.16416	5.61469	5.27689
MAP2K3	3.83327	3.69071	3.1111	3.48362	3.41917	3.02121
MAP2K4	4.27276	4.27978	4.50882	4.85177	5.24176	5.45518
MAP2K4P1	-0.262824	-0.166064	0.0701145	0.713669	0.937744	1.11078
MAP2K5	1.75628	1.62465	1.46757	1.49112	1.50965	1.97981
MAP2K6	1.53936	1.73194	2.32429	2.32998	3.05222	3.45568
MAP2K7	2.54649	2.13336	2.29627	2.11036	3.24508	2.84404
MAP3K1	5.4269	5.62177	5.93341	6.0414	6.0715	5.67308
MAP3K10	2.03565	1.65613	1.48894	1.47617	1.85542	1.33772
MAP3K11	5.32502	5.08259	4.07504	4.241	4.04012	3.90983
MAP3K12	4.15582	4.14943	4.6474	4.83117	3.46762	3.60356
MAP3K13	3.2782	3.28714	4.01105	4.06803	5.1478	4.82794
MAP3K14	2.65679	2.40516	4.5959	4.62915	4.6869	4.05008
MAP3K14-AS1	0.0793884	0.057166	1.96296	2.07175	1.24489	0.830385
MAP3K2	6.002	6.16355	5.56257	5.85371	6.75753	6.87895
MAP3K3	4.10267	3.78554	4.34264	4.52152	3.87026	3.46942
MAP3K4	5.24595	5.14863	4.3459	4.53409	6.50682	6.5867
MAP3K5	2.75625	2.49427	2.81366	3.46672	3.27927	3.55773
MAP3K6	1.82842	1.7708	1.48185	1.67257	2.06763	1.95152
MAP3K7	5.80323	5.98785	4.867	5.14619	5.64856	5.81978
MAP3K8	-0.395124	-0.39658	2.40521	2.56361	-0.217212	0.247587
MAP3K9	3.71031	3.51729	3.17404	3.17889	3.71256	2.93354
MAP4	7.09254	6.97322	6.64936	6.69512	7.32753	7.26197
MAP4K2	3.56654	3.48989	2.47113	2.62928	2.9209	2.64644
MAP4K3	3.81958	3.81094	4.06677	4.26946	4.70217	4.61069
MAP4K4	7.51772	7.55318	6.95921	7.23408	7.35071	6.8714
MAP4K5	5.382	5.38648	3.88511	4.18004	4.90241	4.91716
MAP6D1	0.243547	0.223769	0.340141	0.213217	2.01567	1.48315
MAP7D1	4.52008	4.32831	4.71809	4.93555	5.5775	5.47815
MAP7D3	0.595618	0.71714	3.84439	4.2867	4.43178	4.56591
MAP9	-5.69162 ?		2.25683	2.3864	3.39292	4.15936
MAPK1	6.40731	6.83891	5.64896	6.29871	5.70025	6.1305
MAPK10	-2.05464	-2.29223	-0.276471	-0.899117	1.96542	2.70269
MAPK11	2.52975	2.40135	3.50299	3.50956	-0.753191	-0.619042
MAPK12	3.91045	3.57618	4.85648	4.76822	1.88788	1.77871
MAPK14	5.27104	5.15277	5.37008	5.61354	5.32764	5.19943
MAPK1IP1L	5.74489	5.76716	4.6068	4.83515	5.72203	5.46697
MAPK3	3.17129	3.0068	3.45491	3.73353	3.06421	3.14504
MAPK6	6.12376	6.34495	5.43551	6.04457	6.76883	7.20085
MAPK6PS3	0.714628	0.825965	0.602153	0.595446	0.00219123	0.383596
MAPK7	2.10283	1.80203	2.67244	2.73297	2.65132	2.20002
MAPK8	3.88023	4.27609	3.85813	4.10791	4.01652	4.89412
MAPK8IP1	1.82396	1.36375	1.84441	1.94621	3.76685	3.2181

MAPK8IP3	4.25438	3.92409	4.58878	4.36487	4.18203	3.36475
MAPK9	4.27174	4.01692	5.15799	5.12298	4.92136	4.84114
MAPKAP1	5.01731	4.98216	4.59701	4.81375	5.51928	5.83389
MAPKAPK2	4.88776	4.5692	4.81152	4.90659	5.10908	4.4443
MAPKAPK3	3.79177	3.80861	1.88259	2.73667	3.76806	4.03474
MAPKAPK5	4.73669	4.71091	4.50979	4.48894	4.08855	3.72899
MAPKAPK5-AS1	2.46962	2.44253	2.32201	2.73927	2.21281	2.50477
MAPKBP1	4.22394	4.28574	3.64936	3.88949	3.80573	3.27057
MAPRE1	6.70747	6.69538	6.3703	6.71052	6.53237	6.67827
MAPRE1P1	0.721346	0.9152	0.137608	0.178594	0.619751	0.706128
MAPRE2	4.22127	4.19893	4.53328	4.72247	4.51398	4.26195
MAPRE3	2.07243	1.96344	2.45311	2.38704	2.63662	2.48805
MAPT	3.31773	3.24969	1.71738	1.42259	-0.00196773	-1.006
MARCH1	2.94298	3.07632	2.66823	2.67255	4.18752	4.11347
MARCH2	1.44051	1.2583	0.759062	1.02051	2.2734	2.56476
MARCH3	2.16822	2.12589	0.292311	0.82198	2.11778	2.49787
MARCH5	3.40159	3.34772	3.11569	3.19724	4.10704	4.22992
MARCH6	6.33917	6.44797	7.0152	7.07973	6.7869	6.74321
MARCH7	6.09784	6.20444	6.19232	6.28324	6.11677	6.29074
MARCH8	3.22721	3.55256	1.63029	2.14386	3.25108	3.57636
MARCH9	2.51282	2.51445	2.69913	2.8996	2.57811	2.70269
MARCKS	5.53393	5.55674	6.00385	6.17831	8.29412	8.1503
MARCKSL1	5.05246	4.7466	3.51865	3.36488	4.8539	4.19264
MARK1	0.710384	0.676782	?	?	2.68621	2.54122
MARK2	3.84684	3.75633	3.29828	3.27704	3.31123	3.10386
MARK3	5.90824	5.92462	4.66509	4.73665	5.70409	5.69309
MARK4	1.94119	1.50873	2.83608	2.85696	2.67955	2.29893
MARS	5.93764	5.99263	5.83605	6.40275	6.56321	6.39378
MARS2	3.5677	3.71954	1.38638	1.97335	3.10622	3.51245
MARVELD1	4.80919	4.7183	3.91055	3.93008	3.53321	3.66397
MASP1	?	-6.45019	2.64938	3.55652	-5.65305	-4.70296
MASP2	0.312247	-0.105152	0.636738	0.563685	0.947083	-0.0341529
MAST2	4.65736	4.48552	5.16354	5.25638	4.99207	4.5982
MAST3	1.45575	0.843622	2.42563	1.97542	1.98628	0.893261
MAST4	0.915689	0.988427	-1.2763	-0.979274	2.5221	2.74862
MASTL	4.88082	4.87626	4.22749	4.40654	4.38563	4.56916
MAT2A	7.06483	6.98119	7.10446	7.23413	6.80268	6.76756
MAT2B	3.52733	4.36686	4.20126	5.42498	3.91333	5.64311
MATN1-AS1	0.945188	1.08186	0.0533199	0.0992548	-0.379337	-0.44602
MATN2	5.20705	5.87366	3.32823	3.62249	3.46768	4.69416
MATN3	1.34536	1.95081	0.340141	1.08105	-5.07051	-3.70484
MATR3	8.11503	8.21685	7.90523	8.04913	8.19994	8.3805
MAU2	4.22053	4.22803	4.17402	4.39101	5.0014	4.90431
MAVS	5.78571	5.53538	6.83257	7.22586	6.18182	6.11466
MAX	3.97355	3.92812	3.06911	3.34755	3.46305	3.35524
MAZ	5.85382	5.78863	4.90329	5.0102	6.22349	6.05119
MB21D2	2.62286	2.78034	1.7234	1.86848	3.95348	3.59249
MBD1	4.79026	4.73005	3.94318	4.19038	3.89906	3.73416
MBD2	4.98653	5.03251	4.34297	4.88552	4.07863	4.1881
MBD3	4.45574	3.86359	3.98766	3.85552	5.10497	5.18125
MBD4	3.87361	3.90238	4.25734	4.5724	4.50585	4.51848
MBD5	3.86249	3.74826	4.02668	3.8172	4.28242	3.95408

MBD6	1.47588	1.16656	2.48183	2.17196	1.9581	1.54125
MBIP	2.20181	2.16653	2.25944	2.35062	2.57811	2.85935
MBLAC2	2.36433	2.456	2.55027	2.5169	2.99969	2.95507
MBNL1	6.43707	6.49903	7.04927	7.35109	7.21998	7.16663
MBNL2	5.9909	6.02121	5.22374	5.41619	5.48304	5.70955
MBNL3	3.17437	3.35253	3.58135	3.53142	3.36205	3.9043
MBOAT1	1.93394	1.99661	2.00632	2.06112	1.59711	1.88577
MBOAT2	4.55712	4.98113	5.52512	6.01399	4.30907	5.18885
MBOAT7	4.62679	4.57676	4.89194	5.1073	4.89234	5.00235
MBP	0.16579	-0.118594	0.138526	-0.0869685	2.62279	2.35398
MBTD1	4.38772	4.24623	4.30229	4.09696	4.2704	3.74037
MBTPS1	6.37857	6.39183	6.12396	6.4479	5.69831	5.70532
MBTPS2	3.444	3.37337	4.64944	4.7463	3.35942	3.65444
MC1R	3.01157	2.94623	1.41191	1.83045	0.52295	0.80719
MCAM	7.83761	6.88644	4.56885	4.61907	5.0539	4.80107
MCAT	2.6803	2.70458	2.30433	2.65937	2.31699	2.32121
MCC	5.28958	5.39513	3.25111	3.1835	7.20633	7.10868
MCCC1	2.99441	3.10701	3.80941	4.26403	4.04272	4.46696
MCCC2	4.18126	4.39435	4.62222	4.94712	5.0323	5.18507
MCEE	0.710384	0.537236	1.00636	1.46107	1.06767	1.48315
MCF2L	-3.8896	-2.37465	2.60765	3.0933	?	?
MCFD2	7.42605	7.18592	7.43444	7.82416	7.56724	7.9583
MCL1	8.2	8.09869	6.60074	6.83254	7.41707	7.13465
MCM10	5.47961	5.61498	4.05368	4.22096	4.45798	4.40311
MCM2	6.17285	5.96942	5.50865	5.72484	5.57629	5.5082
MCM3	6.71563	6.74364	5.9917	6.38226	6.84833	6.69121
MCM3AP	5.46823	5.4988	6.23968	6.34542	5.75751	5.52787
MCM3AP-AS1	2.99349	3.15996	3.67427	3.65618	2.95051	2.54987
MCM4	7.08108	7.11813	6.45196	6.74837	6.43341	6.41319
MCM5	6.14008	6.05285	5.40846	5.72603	4.95758	5.03727
MCM6	6.10504	6.03498	4.6822	4.75922	5.24243	5.34244
MCM7	7.89327	7.91377	6.55435	6.99959	6.91015	7.09195
MCM8	5.93003	6.05308	5.94024	6.06797	5.91024	5.48246
MCM9	3.26987	3.5161	3.02157	3.40212	3.9928	3.93649
MCMBP	5.77512	5.91756	5.29424	5.78861	5.67895	6.07258
MCMDC2	0.600365	0.65817	-0.243151	-0.742207	0.084037	-0.111114
MCOLN1	2.35358	2.06894	1.79779	2.14437	2.35033	2.04436
MCOLN2	2.73764	2.27526	4.21735	4.48828	4.48244	5.10866
MCOLN3	1.39372	1.30539	2.68067	2.894	-0.152369	-0.247125
MCPH1	4.26944	4.34235	3.88063	4.05592	4.18117	4.19207
MCRS1	4.7256	4.61463	3.73831	3.96397	4.23904	4.5221
MCTP1	3.96714	4.07145	2.28002	2.04601	2.70383	2.96218
MCTP2	0.959191	0.794611	4.32129	4.75844	-2.95876	-2.70579
MCTS1	2.57006	2.61047	3.52436	4.16697	4.57367	5.46939
MCTS2P	0.795027	0.513905	1.31386	1.3644	1.34276	1.14893
MCU	3.64965	3.60056	2.90924	3.0686	4.14301	4.59248
MCUR1	3.55595	3.80604	3.13195	3.54939	4.07288	4.6309
MDC1	4.73622	4.52512	4.63018	4.46007	4.732	4.24893
MDFIC	4.60297	4.71397	3.97634	4.0933	3.84253	3.70692
MDGA1	0.595618	0.344613	3.11798	3.47614	2.18833	3.49541
MDH1	6.24745	6.37339	5.56715	6.04976	6.44395	7.28265
MDH2	6.06786	6.02424	5.60395	5.95184	6.53631	6.61662

MDK	3.57587	2.58429	5.54611	5.32214	2.91524	2.2062
MDM1	3.21978	3.29871	3.46213	3.38102	3.23754	3.15127
MDM2	7.77514	7.54127	5.13022	5.3456	6.33742	5.7994
MDM4	6.3262	6.22473	5.57052	5.26023	6.11796	5.29046
MDN1	7.58528	7.55821	6.50081	6.61731	6.95311	6.51419
MDP1	0.605893	0.914733	0.1424	0.940159	0.700005	2.35291
ME1	4.55003	4.38107	-2.72315	-2.93781	-1.30241	-0.752286
ME2	5.21077	5.39304	3.75718	4.04599	3.84351	4.35263
ME2P1	0.930949	1.13241	-0.396005	-0.301307	-0.572647	-0.619042
ME3	?	-5.45651	-4.59508	-4.52056	1.63893	1.50278
MEA1	4.51798	4.67377	4.2116	4.82084	5.92719	6.10658
MEAF6	4.09614	4.20514	3.30432	3.64104	4.02531	4.10385
MECOM	2.68031	2.88146	4.07739	4.48922	2.75332	3.42257
MECP2	4.92708	4.92031	5.04288	5.23268	5.4625	5.56416
MECR	2.71032	2.66128	2.08328	2.61228	2.7234	3.0178
MED1	5.58166	5.92648	5.37394	5.76884	5.60298	5.77456
MED10	2.54648	2.47381	2.52385	2.76155	2.79913	2.93354
MED11	2.02884	1.77257	2.16964	2.11922	2.67071	2.99466
MED12	4.09125	4.03799	4.25838	4.24872	4.11447	3.92446
MED12L	2.7404	2.54359	-1.62436	-1.8311	3.55754	3.43703
MED13	7.18278	7.0995	6.91007	7.023	7.63498	7.24348
MED13L	6.39464	6.14964	5.79022	5.68455	6.39027	5.81407
MED14	5.17397	5.08837	5.24116	5.14796	4.79706	4.57868
MED15	5.00925	4.85617	4.49943	4.72247	4.34154	4.1754
MED16	1.9769	1.62994	2.31235	2.29207	2.77641	3.16365
MED17	3.45952	3.57469	4.23853	4.33512	4.26581	4.33083
MED18	2.66725	2.61398	2.03567	2.2792	2.9209	3.11983
MED19	2.26094	2.32507	2.25112	2.38304	2.70384	2.90432
MED20	3.55121	3.53574	3.57304	3.89679	4.4225	4.70479
MED21	3.76035	3.83061	3.65725	4.05229	3.99341	4.41351
MED22	4.31702	4.23063	3.91316	4.05355	3.84847	3.56474
MED23	4.07324	4.2315	4.22692	4.44192	4.84001	4.81587
MED24	3.71877	3.71831	3.77792	3.94509	4.21236	4.0958
MED25	1.78964	1.65406	2.38593	2.49955	2.23354	1.89856
MED26	1.77476	1.70478	1.77403	1.64488	2.36093	1.86342
MED27	2.48139	2.51279	2.31198	2.89831	2.30882	2.6564
MED28	4.97388	4.74615	4.80013	4.70331	5.4385	4.80571
MED29	3.93664	3.90785	4.03322	4.36792	4.84747	4.84209
MED30	1.98042	1.86361	1.37876	1.70499	1.2101	1.62424
MED31	2.7889	2.93343	2.44194	3.05008	3.39302	4.67198
MED4	3.6031	3.80965	3.24659	3.54513	3.40428	3.78262
MED6	3.74283	3.72574	2.87448	3.081	3.55036	3.90799
MED7	2.51525	2.59244	3.19152	3.1603	3.17416	3.83826
MED8	4.47656	4.21034	4.58305	4.78282	4.77058	4.89933
MED9	2.7035	2.62728	3.136	3.41266	3.26553	3.18352
MEF2A	5.27946	5.41296	4.07523	4.26513	4.22616	4.61825
MEF2BNB	2.66276	2.51496	2.42651	2.50621	3.23432	3.20831
MEF2C	3.60013	3.29654	-1.43719	-1.41436	4.08985	4.33877
MEF2D	3.25367	2.80629	3.35758	3.25531	2.99433	2.52692
MEG3	?	?	2.18718	2.97599	1.34536	0.863209
MEGF10	2.96981	3.05088	?	?	-0.959604	-1.79933
MEGF11	1.76856	1.71211	1.58469	1.25754	1.29393	0.97985

MEGF8	5.07035	4.51587	4.54524	4.30374	4.48432	4.29188
MEGF9	3.74179	3.6299	4.17951	4.19723	3.92277	3.59021
MEIS1	3.82833	3.39289	4.85569	4.56258	3.44831	3.1378
MEIS2	3.99183	3.80204	4.58773	4.04212	3.87297	3.9022
MEIS3	1.63024	0.807874	2.56586	2.4105	1.54304	2.01507
MELK	4.89756	4.84719	4.57469	4.93624	5.74694	5.87682
MEMO1	3.5335	3.41366	2.47575	2.91418	4.02753	4.3269
MEMO1P1	2.37462	2.66344	1.55846	1.83076	3.09679	3.4026
MEN1	4.21755	4.07147	3.41824	3.5968	2.95256	2.82857
MEPCE	4.60384	4.55729	3.31745	3.85209	4.39407	4.53278
MERTK	-3.37567	-4.45968	0.416483	0.213217	1.74486	1.25912
MESDC1	2.66944	2.97387	1.83331	1.71775	1.63434	1.71117
MESDC2	5.63939	5.77493	5.6347	6.05867	5.48402	5.59764
MEST	-1.8123	-2.37465	3.84081	4.14124	4.6988	5.65129
MESTP1	?	?	0.0166532	0.103383	0.958033	1.82306
MET	6.16664	6.69496	6.58091	7.23226	6.73143	6.88149
METAP1	4.73936	4.85947	4.3055	4.64208	4.49065	4.88015
METAP1D	2.64966	2.62728	2.08328	2.13437	2.54427	2.47321
METAP2	6.05829	6.21419	5.15465	5.65271	5.91405	6.1427
METRN	3.08228	2.42895	2.86633	2.97066	3.39497	3.92082
METRNL	1.69756	1.38894	2.15189	2.66267	0.258664	-0.280287
METTL1	1.74106	1.8296	1.56462	2.0972	1.95069	2.06955
METTL10	3.08893	3.1658	3.28513	3.39943	3.46952	3.68026
METTL12	3.29146	3.38395	2.17225	2.21705	2.39477	2.41234
METTL13	5.18126	5.09651	3.60111	3.88976	4.47282	4.45693
METTL14	3.76955	3.82486	3.65568	3.77535	3.81034	4.20168
METTL15	2.84752	2.70884	3.22413	3.34805	3.72285	3.59178
METTL15P1	1.43106	1.66288	1.72232	1.66104	0.859493	1.22777
METTL16	4.06579	4.16102	4.23896	4.52325	4.56849	4.3634
METTL17	4.45305	4.4144	3.71376	3.9588	4.20756	4.18296
METTL18	2.48551	2.51695	1.74725	2.04601	1.56854	2.30453
METTL20	0.936485	0.856189	1.25917	1.34562	0.554638	0.507536
METTL21A	2.94722	3.02161	2.1517	2.4201	3.19959	3.37735
METTL21B	0.829898	1.21244	1.72868	2.29446	2.10442	2.11138
METTL22	2.74179	2.47086	3.15187	3.081	2.0332	1.56945
METTL23	3.97423	3.94654	3.53072	3.66596	4.13166	4.60956
METTL25	0.93073	0.808171	-0.0865395	-0.397277	0.695655	1.27395
METTL2A	4.17052	4.26078	3.2805	3.56327	4.57615	4.86372
METTL2B	4.96581	5.05262	4.321	4.59995	5.30959	5.32036
METTL3	4.98599	5.00194	3.66968	3.89265	4.92824	4.78817
METTL4	2.32884	2.38892	2.59785	2.77688	3.34391	3.76496
METTL5	3.519	3.63536	3.04059	3.40887	3.73576	4.6909
METTL6	3.04161	3.12024	2.40521	2.64607	3.35667	3.78631
METTL7A	-0.289526	-0.418271	4.24902	4.3237	4.33048	4.47692
METTL8	4.20614	4.44777	3.68838	3.93928	4.01475	4.35743
METTL9	4.94521	5.05312	5.06264	5.35264	4.96448	5.39788
MEX3A	4.39895	4.02499	3.32229	2.76154	3.13573	2.27058
MEX3B	0.377835	0.20969	0.966283	0.95732	0.246688	0.270672
MEX3C	6.54222	6.92732	3.95602	4.18808	3.65258	3.99372
MEX3D	1.88215	1.73686	1.95605	2.04094	3.03318	2.75273
MFAP1	4.55594	4.60793	4.13388	4.26186	5.16545	5.03642
MFAP3	4.67703	4.67541	4.8326	5.02366	5.24805	5.4005

MFAP3L	3.93021	3.82012	-3.85951	-4.52056	4.40771	4.29457
MFF	5.02445	5.00978	4.88764	5.17115	5.86536	6.17657
MFGE8	4.84636	4.60928	5.20448	5.21984	1.25263	0.847941
MFHAS1	3.99004	3.8375	3.42935	3.3258	4.09791	3.80503
MFI2	4.04154	3.95606	1.61741	1.60545	1.18523	1.24753
MFN1	5.01377	5.06694	5.40501	5.67651	5.95521	6.35021
MFN2	5.74536	5.57984	5.63503	6.02855	6.76814	6.48096
MFSD1	4.37014	4.34443	4.56444	5.10183	4.83945	5.39587
MFSD10	2.97158	2.85457	2.68067	2.83663	3.59118	3.52691
MFSD11	3.73931	3.69568	3.67597	3.94888	3.73606	4.1473
MFSD12	7.4017	7.02854	3.60307	3.58598	5.41395	5.03278
MFSD1P1	0.40317	0.0931274	0.979089	1.0909	0.57974	0.15243
MFSD2A	3.08064	2.82718	0.64945	1.36695	4.2917	3.59935
MFSD3	1.51528	0.794611	1.43857	1.86274	1.61119	2.0482
MFSD4	0.436204	0.332943	0.514461	0.238506	-0.219472	-0.519441
MFSD5	3.76649	3.81676	3.14291	3.70175	3.55401	3.97277
MFSD6	3.08064	3.54837	3.64461	4.48269	5.15386	5.6563
MFSD8	2.9823	3.3732	3.14515	3.52416	3.08661	3.50526
MFSD9	3.19574	3.31851	2.99639	3.24432	2.96905	2.66834
MGA	6.29968	6.131	5.16077	5.12347	6.00035	5.71586
MGAM2	3.71561	3.82486	-5.1783	-6.09892	-5.65305	-4.70296
MGAT1	4.68516	4.46567	5.49859	5.7909	5.532	5.7972
MGAT2	2.89799	2.53042	2.19498	2.13923	2.47094	3.37255
MGAT4A	-2.05464	-2.21426	2.87988	3.27272	1.30552	1.37014
MGAT4B	5.65008	5.21406	5.51726	5.50886	5.66117	5.49249
MGAT5	6.88583	6.82968	5.75639	5.67336	5.01343	4.68668
MGAT5B	2.56537	2.47381	1.99641	2.02048	2.88852	2.919
MGEA5	5.24232	5.33073	6.17924	6.32242	6.63218	6.4729
MGLL	5.41821	5.56745	2.88795	3.42256	0.405873	0.736302
MGME1	4.51948	4.3144	4.19586	4.26185	4.16225	4.29749
MGP	0.727258	-0.93902	2.38255	1.46107	-0.136604	-2.24661
MGRN1	2.99789	2.78034	3.20234	3.21985	2.83355	2.6899
MGST1	3.81662	4.04791	5.94059	6.39299	6.41899	6.76369
MGST2	-1.43547	-1.91931	1.60787	1.99529	2.12475	2.83245
MGST3	4.36969	4.46714	3.34004	3.80107	3.85539	4.5209
MIA	5.58653	5.50072	-3.37468	-5.88892	-5.11342	-4.70296
MIA3	6.20972	6.31382	6.48535	6.7713	6.30046	6.32815
MIAT	1.15442	1.57905	2.09603	2.1139	2.76725	2.59489
MIATNB	0.536123	0.181052	0.800231	0.865515	0.129283	0.290247
MIB1	5.53063	5.84064	5.8041	6.25639	5.86592	6.27769
MIB2	1.73558	1.12967	2.31634	2.25313	1.92333	1.74585
MICA	3.15552	2.98779	2.92838	3.08534	5.41582	5.66368
MICAL1	3.13189	3.05195	3.57749	3.70443	3.72246	3.8191
MICAL2	2.13699	2.72945	4.31631	5.36334	3.6862	4.28338
MICAL3	7.25364	7.0103	4.98071	4.85407	6.248	5.64435
MICALL1	6.06534	5.90731	3.66979	3.9024	4.98355	4.65851
MICALL2	2.72929	2.18822	3.19152	3.1248	2.92466	2.21812
MICB	3.76259	3.48447	3.31574	3.74642	2.81567	2.48584
MICU1	4.10021	4.16011	4.23854	4.61653	4.73038	4.95238
MICU2	3.67487	3.95501	3.17843	3.58988	3.62508	4.14971
MICU3	?	?	3.00878	2.58118	0.909612	0.878317
MID1	4.20178	4.12023	4.86631	5.25146	6.06758	6.10905

MID1IP1	2.7251	2.50902	3.30466	3.5864	3.08045	3.10386
MIDN	3.05079	2.44406	3.24275	3.05356	3.60766	3.54831
MIEF1	5.35587	5.31358	4.62944	5.02944	5.1844	5.25833
MIEF2	1.64081	1.37009	2.30433	2.54224	2.4351	2.48805
MIEN1	2.92088	2.86159	2.40762	2.77935	3.42972	3.85909
MIER1	4.85166	4.98268	4.98075	5.23658	5.50301	5.55478
MIER2	2.62736	2.31853	2.12028	2.22658	3.00147	2.68561
MIER3	4.65783	4.81269	4.88095	5.0621	4.95577	4.96203
MIF	5.12256	4.69723	4.45798	4.82549	-2.00149	-3.42671
MIF-AS1	0.319445	0.342896	0.617214	0.718675	-0.272146	-0.491296
MIF4GD	1.59326	1.67611	1.6133	1.48647	2.15689	2.37069
MIIP	1.84398	1.58704	2.05967	2.23992	2.13737	2.36477
MILR1	1.7314	1.54281	2.35177	2.84828	1.69287	1.76092
MINA	4.33331	4.50852	4.88212	5.14725	3.93225	4.39103
MINK1	4.00894	3.76234	4.54919	4.55407	4.74481	4.65303
MINOS1	3.69712	3.69252	3.7771	4.01213	4.32188	4.79981
MINPP1	4.58399	4.52867	4.06914	4.20519	4.86423	5.06806
MIOS	4.31982	4.42969	3.81506	3.93556	4.18518	4.5752
MIPEP	1.97496	1.87547	2.14741	2.35471	2.07442	2.78515
MIPOL1	3.47085	3.4928	2.73833	2.56361	2.99075	3.22403
MIR133A1HG	0.80878	0.831845	1.82773	1.50592	0.939663	0.270672
MIR17HG	3.5055	3.70832	1.5647	1.75539	1.5246	1.70271
MIR181A1HG	3.79277	3.67159	3.43118	3.09819	3.54304	3.22107
MIR210HG	-0.159773	-0.629746	1.91453	1.73041	2.38682	1.17598
MIR222HG	3.97688	3.69323	3.43669	3.56183	3.67731	3.16057
MIR22HG	2.89898	2.89691	4.01246	4.18694	4.29025	3.76699
MIR4435-2HG	4.62939	4.5152	5.024	5.13412	5.08695	4.85477
MIR4458HG	-2.30606	-3.65369	2.42382	2.69208	1.02625	0.80115
MIR4697HG	2.29246	2.42895	1.20023	0.857026	?	?
MIR503HG	-0.341358	-1.07024	0.782333	0.591699	0.172692	-0.38461
MIR600HG	2.7053	2.3336	1.84782	1.43347	3.08861	2.28107
MIR646HG	1.45071	1.43806	-2.5977	-3.10759	?	?
MIR99AHG	-1.69862	-1.93875	-0.428455	-0.716287	4.26376	4.88293
MIRLET7BHG	3.65846	3.45453	4.29726	4.24542	3.86814	3.25329
MIRLET7DHG	0.568392	0.895605	0.452543	1.26434	-0.0365333	0.752243
MIS12	4.18257	4.22422	4.24916	4.41407	4.10565	4.43979
MIS18A	2.44814	2.66902	2.95603	3.21536	2.15669	1.97279
MIS18BP1	4.22349	4.38499	3.40519	3.51323	4.57628	5.04732
MITD1	3.42017	3.4546	3.17821	3.27488	3.30908	3.09879
MITF	3.31773	3.29538	3.3749	3.70334	2.52953	2.21811
MKI67	8.61695	8.43816	8.73848	8.63889	8.75554	7.99205
MKKS	4.19875	4.0994	4.29826	4.58814	4.81238	5.18201
MKL1	3.22425	3.03101	3.44219	3.37095	2.80526	2.54122
MKL2	4.89605	4.80409	5.34085	5.26579	4.1843	3.9713
MKLN1	6.26026	6.22574	6.8745	7.10657	6.47669	6.37544
MKLN1-AS	0.133932	0.107125	1.04535	1.0909	0.234612	0.541297
MKNK1	3.66176	3.75025	4.04155	4.36386	4.02096	3.94433
MKNK2	4.1179	3.50149	4.45219	4.49015	5.41047	4.90707
MKRN1	5.72496	5.77516	5.25675	5.63745	6.20991	6.34811
MKRN2	4.45387	4.55179	3.87697	4.18854	4.45536	4.91295
MKRN3	2.81313	2.8401	3.23121	3.21609	2.52466	2.18067
MKRN5P	0.870156	1.00482	1.05281	1.01017	0.715208	0.86236

MKS1	2.19271	2.06856	1.82198	2.02562	2.15663	2.20016
MLEC	8.35369	8.5712	7.57636	7.89827	7.43358	7.67899
MLF1	2.91195	3.0068	2.99888	3.44577	2.68621	3.5245
MLF2	5.2843	5.21311	4.90526	5.22321	5.61641	5.66286
MLH1	4.72875	4.70017	4.10304	4.44575	4.87252	4.91075
MLH3	-0.632781	-0.508457	4.34785	4.31638	3.66612	3.47568
MLK4	2.02886	2.13336	2.39769	2.53143	2.42717	1.97279
MLK7-AS1	0.0977591	-0.0582957	1.34333	1.3372	-0.609971	-1.08729
MLKL	2.14014	2.29872	2.90129	3.34238	1.90577	2.42773
MLLT1	4.02713	3.54698	4.12708	4.20858	4.68619	4.12775
MLLT10	4.44558	4.44328	3.56802	3.6037	4.38613	4.34585
MLLT11	3.82362	3.85024	5.70675	6.06016	5.79354	5.90925
MLLT3	4.15656	4.2289	2.45311	2.55296	4.8144	4.70479
MLLT4	5.3494	5.26549	5.27743	5.34598	5.78908	5.38868
MLLT6	5.09305	5.14434	4.99849	5.04172	5.20909	5.38262
MLPH	2.99267	3.37004	3.34004	4.03583	2.93589	3.77508
MLST8	2.86816	2.35929	2.99828	3.11471	2.9891	2.94223
MLX	4.88183	4.64948	4.65562	4.81208	5.71725	5.54887
MLXIP	4.45447	4.19893	4.32229	4.18694	5.06931	4.43155
MLYCD	2.90847	2.72962	3.59543	3.67235	2.11678	1.94159
MMAA	1.42475	1.2583	1.06446	1.1104	0.447209	1.24753
MMAB	3.51118	3.3086	2.44208	2.81599	2.57092	3.08046
MMACHC	2.95803	3.01751	4.97178	5.26346	4.86077	4.88496
MMADHC	5.86549	5.88805	4.78394	5.31259	6.12401	7.09121
MMD	3.76546	4.31933	5.66744	5.87237	2.51712	3.20618
MME	?	-4.87366	4.11302	4.79084	-3.85089	-3.70484
MMGT1	3.78373	3.83406	4.5655	4.88764	5.01652	5.2099
MMP1	-2.52833	-2.29223	4.25527	6.45816	?	-3.12051
MMP13	-5.69162	-6.45019	3.63983	4.79579	-5.65305	?
MMP14	5.19386	4.78921	5.60887	5.84351	4.87056	4.58559
MMP15	4.32813	3.99453	1.09265	1.23995	1.59239	1.62424
MMP16	7.1303	6.92101	2.32824	2.50221	6.00255	6.27654
MMP17	2.87646	2.31525	0.138526	0.19507	-1.23372	-1.70626
MMP19	-1.13684	-2.12563	1.99441	1.99952	-3.52019	-2.43702
MMP2	8.23223	8.5668	8.76681	8.95299	6.5498	6.05295
MMP24	1.47523	1.05428	1.12879	1.19269	1.12858	0.788702
MMP24-AS1	3.37127	3.11672	2.6336	2.65986	4.18324	4.18563
MMP3	-5.10914	-4.87366	3.24484	5.27	-2.572	-1.12145
MMS19	4.70977	4.65933	4.79303	4.99839	5.26077	5.14112
MMS22L	5.02926	5.08018	5.21147	5.21703	5.40342	5.10546
MNAT1	3.93845	3.96864	3.45673	3.52416	3.41784	3.98666
MND1	2.06913	2.1738	1.30837	1.39106	1.59239	1.85556
MNS1	2.61342	2.63677	1.45185	1.68484	1.91515	1.93443
MNT	1.90457	1.39517	2.39392	2.32312	2.39155	1.76137
MOAP1	3.3844	3.3637	2.13842	2.09576	3.86031	3.7609
MOB1A	6.0935	6.49242	6.22586	6.80086	6.76658	7.3814
MOB1B	4.82985	4.81618	4.58714	4.56536	5.06035	4.46945
MOB2	2.95556	2.81793	1.82213	2.46104	2.63202	2.74862
MOB3A	2.0921	1.59788	3.38444	3.39302	2.74058	2.22994
MOB3C	3.37106	3.45004	3.6604	4.10185	3.15181	3.07793
MOB4	4.13904	4.37848	3.80875	4.36189	4.7344	5.11626
MOCOS	3.56654	3.53151	-0.138983	0.46112	1.44826	1.56011

MOCS1	3.0269	2.91831	2.51137	3.20101	2.13339	2.15456
MOCS2	3.94658	4.04791	4.24169	4.52779	4.40712	5.01352
MOCS3	4.11389	4.19089	2.66198	2.97333	3.1276	2.99373
MOGS	5.86479	5.75843	4.96304	5.04978	5.92324	5.91761
MOK	5.55239	5.63971	2.23572	2.10065	2.0778	1.84021
MON1A	2.79678	2.81793	1.32432	1.74918	2.86325	3.19717
MON1B	4.09776	4.032	3.9026	4.14737	3.98354	3.87824
MON2	5.81315	5.75359	5.55329	5.72168	5.26931	5.04983
MORC2	3.99416	3.74731	3.98087	4.05028	4.15788	3.83024
MORC3	4.06634	4.06698	4.59212	4.7356	4.60406	4.90341
MORC4	4.60523	4.52653	3.03807	3.41669	2.33406	2.43285
MORF4L1	5.34364	5.66045	5.48135	5.97329	5.98511	6.60593
MORF4L1P1	5.60402	5.85068	5.5323	6.1517	6.21325	6.9934
MORF4L2	7.22554	7.3321	7.00981	7.50861	7.79803	8.36029
MORN1	1.85554	1.95503	2.72338	2.86272	1.82353	1.46325
MORN2	2.14329	2.11344	1.50301	1.57037	2.4351	3.03813
MORN4	0.71885	0.318612	1.91453	2.15798	0.901996	1.25912
MOSPD1	2.48333	2.72204	3.12709	3.55117	2.80322	3.89876
MOSPD2	3.05414	2.82025	2.93022	3.20859	3.71255	3.79512
MOSPD3	0.50068	0.407614	0.610974	0.591699	0.723471	0.785207
MOV10	4.78156	4.60117	5.67193	5.72435	5.65157	4.9876
MOXD1	3.65074	3.47967	-1.93506	-1.64896	5.80752	6.59878
MPC1	1.72302	1.82258	2.43854	3.02046	2.74058	3.27914
MPC2	3.15132	3.27322	3.3302	3.73641	3.71488	4.2751
MPDU1	4.74786	4.8001	4.74802	5.04563	5.38193	5.98591
MPDZ	5.54587	5.55708	5.22798	5.29896	6.43079	6.21919
MPG	1.98998	1.65277	3.00469	3.22073	2.61501	3.62131
MPHOSPH10	4.67104	4.70456	3.9573	4.2727	4.64005	4.83341
MPHOSPH6	3.11149	3.22191	3.529	3.86127	2.85737	3.24165
MPHOSPH8	4.45213	4.37726	4.35207	4.4046	3.9457	3.72751
MPHOSPH9	6.10799	6.07543	5.38964	5.42499	5.41788	5.3605
MPI	4.57113	4.45516	4.45137	4.71758	5.00713	4.5725
MPLKIP	3.99353	3.98836	3.95858	3.83368	3.47668	3.34857
MPND	0.310864	-0.101508	0.538041	0.704969	1.25008	1.36963
MPP1	2.64078	2.51158	2.73535	3.69692	2.85933	2.92265
MPP2	1.91565	1.59247	2.65884	2.18121	0.172692	-0.536593
MPP3	1.28396	1.14454	1.91979	2.28351	1.56372	1.25912
MPP5	5.05373	5.02849	4.676	4.87771	4.67115	4.7143
MPP6	4.6335	4.59379	5.19097	4.92391	4.68342	5.14347
MPPE1	1.64513	1.63199	2.04654	2.22222	2.52728	2.86586
MPRIP	6.5145	6.722	6.88338	6.83495	6.44831	5.85107
MPRIPP1	2.57485	2.79617	3.17215	3.04854	2.59924	1.91037
MPST	3.10344	2.75633	3.51865	3.71135	2.65937	2.30453
MPV17	4.27963	4.04791	3.44402	3.62588	4.93587	5.05067
MPV17L2	2.19576	1.95924	1.74725	2.16731	2.26748	2.43431
MPZ	6.54842	5.52762	0.782333	0.100749	-2.65941	-2.8983
MPZL1	6.03982	6.043	6.5744	6.94187	5.95507	6.10625
MR1	1.74803	1.51447	4.51777	4.60541	2.67732	3.29891
MRAS	2.7418	3.19179	2.40895	2.7209	-3.07415	-3.12051
MRC2	5.55948	5.17938	7.09458	6.84802	5.89006	5.21251
MRE11A	3.65104	3.77745	4.8556	4.95321	3.68827	3.96336
MREG	2.12858	2.61461	1.19244	1.15801	2.55282	2.40668

MRFAP1	6.37966	6.35479	6.02406	6.35722	7.10247	7.24668
MRFAP1L1	4.74593	4.80041	4.52556	4.79278	5.26152	5.39981
MRGBP	4.3072	4.3716	3.73665	4.21197	4.70381	4.59134
MRI1	3.49393	3.50251	2.70135	3.11534	4.2695	4.15944
MRM1	1.52496	1.554	0.935482	1.16735	1.10792	1.12623
MROH1	2.23758	1.82258	2.86361	2.8827	2.42185	2.08772
MRPL1	2.78273	2.95078	2.44949	2.81302	2.7363	3.64864
MRPL10	4.18357	4.19893	3.62383	3.95456	4.09122	4.18202
MRPL11	4.25293	4.15185	3.77067	4.17077	3.50454	4.02291
MRPL12	3.48912	3.41147	2.84509	3.23177	4.24128	4.97806
MRPL13	3.99615	4.0879	3.94704	4.32788	4.39158	5.63535
MRPL14	3.56067	3.53434	3.45129	3.99445	4.45408	5.0212
MRPL15	3.85358	3.75511	3.34591	3.86128	3.28952	4.30312
MRPL16	3.85454	3.86246	3.5962	3.74448	3.69834	3.7383
MRPL17	4.99004	5.01339	4.10534	4.72168	4.87788	5.70532
MRPL18	3.78976	3.81095	3.61252	4.1067	5.04401	5.50396
MRPL19	5.11152	5.16605	4.40425	4.70013	5.25062	5.63099
MRPL2	2.41537	2.43579	2.40895	2.7398	3.3448	3.31793
MRPL20	4.56859	4.56538	3.89186	4.3025	5.3849	5.94852
MRPL21	4.07158	4.15278	3.47821	3.97863	3.92088	4.93623
MRPL22	2.72299	2.77795	3.53758	3.73509	3.45993	3.86693
MRPL23	3.83718	3.75511	2.84162	3.35879	2.77433	3.31843
MRPL24	4.59267	4.48916	3.61089	3.97465	4.37792	5.12143
MRPL27	4.58343	4.51156	3.75899	3.75381	4.81441	5.10384
MRPL28	2.68895	2.58156	4.34883	4.78826	4.20618	4.72791
MRPL3	5.12727	5.23399	4.43285	4.95091	5.48151	6.29615
MRPL30	4.87689	4.92561	4.04316	4.33621	4.87974	5.563
MRPL32	4.74332	4.8636	5.18007	5.42204	4.72068	5.28124
MRPL33	4.59607	4.44442	3.16078	3.48008	6.16961	6.7357
MRPL34	2.66753	2.55623	1.64983	2.32719	3.62916	4.2928
MRPL35	5.02478	5.07301	4.22754	4.51474	5.12544	5.42877
MRPL36	3.95465	4.02699	3.42751	3.95726	4.86471	5.17515
MRPL37	5.12199	5.13892	5.20448	5.66936	5.53084	5.78362
MRPL37P1	-0.676496	-0.508457	0.793829	1.07114	0.990802	0.151378
MRPL38	4.02692	4.03043	3.73016	4.11694	4.41466	4.57779
MRPL39	3.22126	3.12212	3.16742	3.82042	2.93775	3.60391
MRPL3P1	1.84568	2.07353	0.978166	1.95946	2.22175	2.81233
MRPL4	3.09467	2.72885	2.42451	2.78296	3.74469	3.99661
MRPL40	4.23238	4.09461	3.72488	4.09451	3.67172	4.22844
MRPL41	2.40946	2.33483	2.25112	2.65273	3.32268	4.07792
MRPL42	5.63894	5.5992	4.52377	4.85968	5.61655	5.83489
MRPL43	3.69944	3.69592	3.4348	3.87498	4.6377	5.2268
MRPL44	3.27331	3.3347	3.16078	3.43033	4.00478	4.26482
MRPL45	3.9297	3.8521	3.47733	3.95049	4.02269	4.43222
MRPL46	3.70074	3.69219	3.42378	3.75845	3.33687	3.94592
MRPL47	3.53152	3.73251	3.64881	4.03444	4.42977	5.43723
MRPL48	3.55713	3.64044	2.46035	2.73039	2.81543	3.20318
MRPL49	5.41713	5.45062	4.3592	4.82263	4.56003	4.97188
MRPL50	3.75213	3.76231	3.15846	3.50588	3.76172	4.52448
MRPL51	5.55724	5.54673	5.04838	5.46596	5.43683	6.04778
MRPL52	3.09209	3.10701	2.64938	2.93284	3.69504	4.36876
MRPL53	3.33674	3.19715	2.67757	2.85984	3.22841	3.61252

MRPL54	2.47336	2.29872	1.25116	1.53507	3.35232	4.4868
MRPL55	2.71667	2.47674	2.62706	3.1224	3.21621	3.35668
MRPL57	3.64188	3.68943	3.36145	3.72089	3.78159	4.32533
MRPL9	4.34524	4.40528	3.50201	3.88433	3.9041	4.28154
MRPS10	3.87468	3.79004	3.98302	4.48187	5.15287	5.51448
MRPS11	3.88527	3.76896	4.12691	4.34371	3.9733	4.327
MRPS12	3.20532	3.03791	2.80135	3.1908	3.84064	4.32647
MRPS14	3.81957	3.6561	3.60772	3.82494	3.632	4.00237
MRPS15	4.65625	4.62923	4.27898	4.5968	5.005	5.36609
MRPS16	5.24963	5.2845	4.83469	5.24154	6.03577	6.42541
MRPS17	3.56589	3.50005	3.13446	3.51874	3.73447	4.17967
MRPS18A	3.86239	3.72458	3.26821	3.63649	4.15323	4.31462
MRPS18B	4.4652	4.43727	4.3302	4.7784	4.90145	4.82953
MRPS18C	3.3838	3.55515	3.09829	3.50599	3.62873	4.44657
MRPS2	3.59494	3.47914	3.25509	3.66639	4.04856	4.05719
MRPS21	5.33539	5.1998	4.18061	4.40192	5.44202	5.93983
MRPS22	4.11145	4.20857	3.91831	4.36773	4.1677	4.75575
MRPS23	5.19383	5.29203	4.18934	4.63348	5.90979	6.73285
MRPS24	4.49721	4.21811	4.04418	4.54896	4.29652	4.48434
MRPS25	5.09769	5.01382	4.53236	4.55404	5.08302	4.72969
MRPS26	3.94567	3.96236	3.25111	3.77229	3.63775	4.14659
MRPS27	5.13379	5.21178	5.50163	5.8465	5.83713	6.05004
MRPS28	2.70201	2.69686	2.77195	3.0686	3.75251	4.45753
MRPS30	4.39103	4.60727	4.29322	4.56713	5.13328	5.13091
MRPS31	2.88589	2.77795	2.83608	2.95996	2.8073	2.87072
MRPS31P4	0.0961134	0.604185	0.453122	0.339263	0.219128	0.883629
MRPS31P5	1.64438	1.74978	1.61365	1.64532	1.13809	0.534928
MRPS33	3.22425	3.10511	3.12482	3.57416	4.23449	5.04315
MRPS34	4.27821	4.08116	4.03563	4.35571	4.46572	4.70903
MRPS35	4.58342	4.75268	4.13503	4.63349	4.05134	4.7751
MRPS36	1.9588	2.20503	2.31739	2.81599	2.96643	3.91209
MRPS36P1	0.769399	0.729896	0.963592	1.4148	1.70797	2.55902
MRPS5	4.81068	4.79218	4.4818	4.77228	5.29243	5.11506
MRPS6	6.08993	6.2117	6.63893	6.78334	5.33445	5.24618
MRPS7	4.1915	4.15562	4.60323	5.03556	5.57203	6.15857
MRPS9	3.7295	3.83068	3.21057	3.64931	3.67584	3.92707
MRRF	3.98575	3.93412	4.04669	4.13127	4.52811	4.73715
MRS2	4.27246	4.00405	3.59291	3.73269	4.93399	5.05567
MRTO4	4.99082	5.07184	4.44544	4.96438	5.46759	5.59248
MSANTD2	3.48333	3.55368	3.80638	3.7988	3.12923	2.66834
MSANTD3	4.69589	4.85117	4.08326	4.44072	4.95392	4.66287
MSANTD3-TMEFF	1.2257	1.21359	1.54476	1.17236	1.70682	0.968201
MSANTD4	2.74594	2.68945	3.63506	3.841	4.80983	4.85361
MSC	-3.40093	-4.87366	3.90916	4.44288	?	?
MSC-AS1	-3.42216	-2.64014	3.52261	3.80149	?	?
MSH2	5.52823	5.61361	4.91779	5.03135	5.19572	5.53167
MSH3	3.73948	3.68886	4.04411	4.10242	4.26398	4.60722
MSH5	3.31703	2.96485	3.47368	3.40474	2.68884	1.68701
MSH5-SAPCD1	1.82299	1.38724	2.03872	1.66184	0.829005	0.894426
MSH6	6.70251	6.76393	6.42221	6.69026	6.42338	6.369
MSI2	5.81019	5.80831	4.69299	4.67989	7.24571	6.93329
MSL1	5.02158	4.90402	5.60111	5.60411	5.87641	5.61689

MSL2	3.94566	3.85907	3.98389	4.03328	3.77952	3.57174
MSL3	3.06213	3.17378	3.1111	3.32573	3.361	3.53568
MSL3P1	-2.1653	-2.46208	-2.77279	-2.29725	1.43853	1.66391
MSMO1	5.44936	5.45077	5.82457	6.28695	5.38478	6.12777
MSN	7.23635	7.45812	8.26055	8.70142	8.45484	8.34054
MSNP1	2.84402	3.2083	4.07899	4.47843	4.20087	3.75536
MSRA	1.62738	1.92088	1.7294	2.03075	3.14705	3.42771
MSRB1	1.78476	1.58704	2.00633	2.52781	2.2997	3.07136
MSRB2	-0.489437	-0.818747	1.31637	1.6394	0.405873	1.03481
MSRB3	4.40812	4.95555	1.85542	2.96532	3.34672	4.00753
MSS51	0.0954519	-0.0197685	-0.935332	-1.30113	1.09463	-0.121607
MST1	2.43076	1.88282	1.71509	1.53316	1.22414	0.848598
MSTO1	3.92834	3.76157	3.49374	3.92149	3.59294	3.40548
MSTO2P	0.732087	0.37109	0.155555	0.569958	0.855982	-0.942889
MSX1	0.388476	0.166625	2.45311	2.97864	0.0946955	0.176042
MSX2	-1.37679	-1.65505	1.05494	1.82784	-1.48999	-1.61882
MT1E	0.776727	1.64571	0.225978	1.8512	5.2023	6.70998
MT1F	?	?	-1.79072	-0.40821	0.939663	2.24754
MT1P3	-3.8896	-3.13966	-3.59683	-2.10822	0.479334	2.42894
MT1X	1.58589	1.99758	1.55707	2.82102	3.7981	4.951
MT2A	5.21342	5.28728	3.53245	4.16997	5.68481	6.29713
MT2P1	0.323774	0.903409	-1.37621	-0.398186	0.798349	1.58757
MTA1	4.44296	4.13509	3.21175	3.22749	3.93557	3.43427
MTA2	6.01369	6.21885	5.30984	5.84971	6.09475	6.42802
MTA3	3.81957	3.61664	3.73086	3.59161	3.79399	3.60163
MTAP	5.78865	5.90692	4.64206	4.98587	6.67094	6.73962
MTAPP2	0.273349	0.759233	1.07934	0.868521	1.18934	1.25359
MTATP6P1	5.37605	4.99862	5.97286	5.40143	5.87617	5.10224
MTBP	3.90491	4.01039	3.64936	3.79242	3.58209	3.79742
MTCH1	5.24635	5.12633	5.71092	6.18751	6.34304	6.39
MTCH2	4.68516	4.66449	4.60355	5.04408	5.57239	5.8862
MTCL1	2.64468	2.6352	4.1214	4.18119	5.69593	5.21442
MTCP1	1.18098	1.18599	1.49963	1.88745	1.60214	1.88691
MTDH	6.91796	7.08991	7.08266	7.53976	7.17902	7.41497
MTERF1	4.40617	4.42893	4.28815	4.40094	3.89523	4.19112
MTERF2	3.29632	3.14213	3.11566	3.13492	2.81167	2.64118
MTERF3	2.91011	2.93162	2.78803	3.10063	3.00148	3.52691
MTERF4	3.82607	3.94535	3.90088	3.96045	4.54063	4.25219
MTF1	4.18813	4.43853	3.44541	3.89117	4.77561	5.04315
MTF2	4.93391	4.70644	5.30631	5.14027	4.9672	4.43666
MTFMT	2.35887	2.24107	2.46754	2.64941	2.35093	2.5317
MTFP1	3.60712	3.40166	2.88092	2.75115	3.45762	3.42334
MTFR1	4.19341	4.30308	3.9523	4.2412	3.50857	3.91171
MTFR1L	4.75116	4.63103	4.1325	4.36845	5.34685	5.46479
MTFR2	1.58636	1.59788	1.12941	1.41477	2.18206	2.47818
MTG1	3.58957	3.31702	3.4359	3.53382	3.81037	3.6223
MTG2	4.40945	4.32669	3.84853	4.10913	4.85487	4.63758
MTHFD1	6.00283	6.059	4.72056	5.1821	5.60159	5.87117
MTHFD1L	4.94385	5.0149	3.1111	3.3048	4.90049	4.56823
MTHFD1P1	2.66974	2.91694	1.57731	1.92657	2.47313	2.39229
MTHFD2	5.22652	5.93101	4.67227	5.66888	5.68099	6.38516
MTHFD2L	3.47696	3.63247	2.84654	3.19258	5.01362	5.18553

MTHFD2P7	0.323393	1.30865	-0.0973012	1.21373	0.817083	1.64815
MTHFR	2.87901	2.6075	3.48326	3.54244	3.43435	3.02829
MTHFS	1.47451	1.57637	1.64185	2.04645	0.630722	1.33407
MTHFSD	3.05247	3.05483	3.23854	3.30903	2.17891	2.03478
MTIF2	4.64212	4.58942	4.18817	4.42045	4.85365	5.17791
MTIF3	2.32425	2.43554	2.48915	2.81288	3.19622	3.85416
MTM1	2.47586	2.55676	2.47827	2.61229	3.17417	3.28197
MTMR1	4.19194	4.50293	3.8235	4.21872	5.03752	5.11505
MTMR10	3.86758	3.85527	3.27458	3.39311	3.34305	3.46582
MTMR11	1.99443	2.15188	2.89062	3.12958	2.83555	3.22107
MTMR12	4.60925	4.35811	4.34786	4.44288	5.32589	5.02883
MTMR14	3.91747	3.8936	3.31832	3.41277	4.49956	4.60503
MTMR2	4.46582	4.62858	5.14008	5.5864	5.71824	6.12973
MTMR3	4.64468	4.64599	4.95562	5.02858	5.00322	4.74968
MTMR4	5.46392	5.83381	5.02591	5.68395	5.65312	6.3952
MTMR6	4.65956	4.90238	4.42934	4.96731	4.35161	4.89598
MTMR7	1.30193	1.11343	1.92932	1.90786	1.12764	0.607787
MTMR9	4.14284	4.40502	4.02304	4.37905	4.27653	4.63935
MTMR9LP	-2.05464	-1.60444	0.747284	1.18585	0.578176	-0.0341529
MTND1P23	2.95018	2.76598	3.1088	2.65273	3.56489	2.68561
MTND2P28	5.75444	5.56229	5.74462	5.15032	6.19143	6.09821
MTND4P12	-1.05494	-0.93902	-1.229	-0.649181	2.89044	2.2933
MTO1	4.22635	4.16817	3.36131	3.77357	3.90949	3.92428
MTOR	6.27448	6.27686	6.05254	6.45315	6.58731	6.53542
MTPAP	3.9883	4.06755	3.43303	3.62926	3.83053	3.89505
MTPN	7.41022	7.53727	6.67834	7.14116	7.49973	7.81008
MTR	6.13437	6.13191	5.96017	6.08314	6.00924	5.62476
MTRF1	2.2493	2.26509	2.16964	2.27057	2.31412	2.83245
MTRF1L	4.00148	4.05019	3.74196	3.93496	4.60367	4.54225
MTRNR2L12	1.02146	0.866963	2.71132	0.901216	0.878914	0.633217
MTRR	4.91608	4.84435	4.38538	4.81152	4.69833	4.7414
MTSS1	1.00142	0.980157	1.37111	1.17662	0.97892	0.422664
MTSS1L	3.5349	2.92848	5.18777	5.09461	5.65503	5.1926
MTTP	-5.10914	-4.13839	-2.18288	-3.10759	1.90577	2.16982
MTURN	3.75421	3.7739	2.56803	2.87702	3.08915	3.40601
MTUS1	2.98532	3.30885	4.47928	4.92097	0.91718	0.151378
MTX1	4.39828	4.23054	3.69599	4.12241	3.5398	3.52428
MTX2	3.5213	3.53151	3.3653	3.6953	3.98625	4.5003
MTX3	4.67976	4.81153	5.0815	5.35059	5.398	5.46883
MUC5B	1.76445	1.69706	-3.18222	-3.52222	-4.65668	-5.69922
MUL1	4.18966	4.16011	4.39484	4.80705	4.55157	4.81586
MUM1	4.26064	4.03779	4.14053	4.15185	4.63805	4.42971
MUS81	4.02018	4.09201	3.58798	3.84391	3.34391	3.39395
MUT	3.46331	3.60862	3.89862	4.10427	5.32481	5.53109
MUTYH	2.49479	2.54022	2.95232	3.09235	2.55611	2.79995
MVB12A	2.68419	2.72245	2.05959	2.27402	3.26436	3.96217
MVB12B	2.85167	2.64045	0.711372	0.383121	-0.0308197	-0.848487
MVD	2.87078	2.66645	3.04048	3.27704	1.95074	2.57407
MVK	3.68066	3.58976	2.69311	3.12	2.95624	3.44614
MVP	2.57937	2.66642	5.63984	6.34521	5.15707	5.97891
MX1	-1.65424	-1.76193	3.33312	3.26152	5.46566	4.75787
MX2	-3.8896	-3.46127	3.31433	3.87843	-0.136604	0.100762

MXD1	1.94482	2.09942	1.6494	2.05364	2.3227	2.44966
MXD3	2.6837	2.41008	3.09365	3.23299	2.73508	3.06409
MXD4	2.6206	2.05287	3.13389	2.96532	3.24658	3.028
MXI1	3.72718	3.82023	2.84439	3.04092	3.72446	3.24165
MXRA7	6.26122	6.32965	5.78499	6.00665	6.84944	7.07595
MYADM	3.18356	2.99043	3.78946	3.96933	2.98355	2.81686
MYBBP1A	4.96705	4.75603	4.27027	4.45958	5.72032	5.67601
MYBL1	3.96181	4.19982	4.29524	4.76691	4.2269	4.0178
MYBL2	6.18996	6.02925	4.41359	4.38802	5.09915	4.80307
MYC	4.2033	4.34934	3.94317	4.30691	3.18674	2.919
MYCBP	4.09207	4.1009	3.26021	3.64269	4.62727	5.20695
MYCBP2	6.29826	6.34656	6.52556	6.60611	6.4821	6.29067
MYCBP2-AS1	-0.0550816	0.249555	0.544782	0.261603	-0.122562	-0.235222
MYCN	3.65847	2.76116	-1.65952	-0.751045	-6.64581	-5.69922
MYD88	2.76241	2.36371	2.95603	2.97067	3.8245	3.61295
MYDGF	5.25329	5.14263	4.49682	5.01594	5.42385	5.8085
MYEF2	3.40155	3.27694	-0.095919	0.300651	5.28318	4.49418
MYEOV	5.49784	5.64011	-0.0336419	1.07114	0.0946955	0.878317
MYEOV2	3.74283	3.67927	3.12481	3.33825	4.40307	5.32946
MYH10	7.30927	7.08928	6.52751	6.22516	2.98895	1.9868
MYH11	1.43744	1.33336	1.25673	1.24742	0.74223	-1.0508
MYH14	2.84975	2.41352	-4.59508	-6.09892	?	-5.69922
MYH15	0.430296	1.045	2.56468	4.01141	-1.95932	-0.799586
MYH9	8.77433	8.82983	8.41869	8.56098	9.19219	8.66162
MYL12A	4.67342	4.90065	5.3852	5.98856	6.53523	7.16067
MYL12B	5.49499	4.78569	5.78736	5.66531	7.26356	6.9387
MYL5	0.165597	0.224658	1.38681	1.40443	0.26438	0.601078
MYL6	7.20924	7.57419	6.77747	7.52276	7.90703	8.90772
MYL6B	4.35192	4.30863	4.29258	4.42498	4.82069	5.00142
MYL6P3	0.658554	1.40138	0.225978	1.12005	1.68845	1.93056
MYL6P5	-0.0477872	0.51788	-0.648329	0.357946	0.898874	1.51759
MYLIP	2.70394	2.69451	3.6853	3.73979	3.09458	3.27628
MYLK	2.99847	3.05286	6.0664	6.83637	5.40682	6.87596
MYNN	3.46861	3.48169	3.55528	3.77279	3.77827	4.27264
MYO10	5.37304	5.17196	6.62941	6.55405	6.1302	5.45377
MYO18A	5.42796	5.20335	6.55489	6.59325	6.35883	5.84029
MYO19	5.93613	5.89973	5.15117	5.416	6.14514	5.91303
MYO1B	6.14482	5.96683	-1.37581	-1.86039	5.84675	5.78012
MYO1C	5.94138	5.92813	5.65882	6.02012	6.61076	6.71143
MYO1D	3.63184	3.73794	-3.59683	-2.93781	-4.07293	-2.24661
MYO1E	6.00438	6.17306	3.70825	3.96263	6.13184	5.95817
MYO5A	6.42099	6.57763	4.73384	5.04345	5.50081	5.70001
MYO5B	0.96598	1.31856	1.43687	1.41272	1.41031	2.01383
MYO6	4.37638	4.64043	4.39107	4.39598	4.30402	4.33904
MYO9A	5.41767	5.43924	5.6304	5.56238	5.3982	5.31903
MYO9B	4.96868	4.79677	5.25986	5.28171	5.77238	5.31564
MYOF	6.78581	6.6443	6.56968	7.17755	7.97629	7.71054
MYPN	3.26382	3.32832	-1.48269	-0.493926	-4.65668	?
MYPOP	0.752201	0.68698	0.558032	0.659431	0.901996	1.20023
MYRF	1.04254	0.822624	2.28149	2.27916	2.0177	2.04251
MYRIP	2.1982	2.12571	-0.742746	-1.78641	-4.33548	-4.11926
MYSM1	5.7759	5.80541	5.36623	5.24319	5.83282	5.1805

MZF1	3.13144	2.89939	3.02813	2.84083	3.70041	2.78034
MZF1-AS1	1.23465	0.715841	0.840008	1.01597	0.47024	-0.610724
MZT1	4.20254	4.50653	3.4942	3.9586	4.98579	4.86976
MZT2A	3.12343	2.92604	2.09845	2.39197	2.86345	3.22687
MZT2B	3.1732	2.85982	2.59318	2.92987	3.27883	4.27548
Metazoa_SRP	14.248	14.0391	13.5042	13.9186	14.6805	15.1661
N4BP1	3.92571	3.96027	4.49507	4.57591	4.88321	5.16904
N4BP2	4.52189	4.60323	3.64462	3.74136	4.13896	4.5257
N4BP2L1	0.264344	-0.0877349	1.33218	1.38232	-0.217227	-0.585715
N4BP2L2	5.88525	5.96494	6.13697	6.10251	5.68619	5.54387
N4BP2L2-IT2	3.10182	3.04495	2.62384	2.81599	2.76596	2.43285
N6AMT1	2.67774	2.33807	3.35949	3.10792	2.06763	2.04819
N6AMT2	-0.432112	-0.629746	0.544485	0.619169	0.107996	0.422664
NAA10	3.80176	3.72326	3.95984	4.28347	4.91285	5.28975
NAA11	4.23313	4.31604	-5.1783	-3.78492	-4.07293	-5.69922
NAA15	6.27391	6.36803	5.79842	6.11813	6.22591	6.54065
NAA16	3.90269	3.86695	3.74722	3.72406	3.52705	3.3101
NAA20	4.92068	4.89249	4.35077	4.5467	4.55824	4.87917
NAA25	5.6427	5.71175	5.31731	5.53411	5.15786	5.24237
NAA30	3.95734	4.18372	3.00631	3.40096	3.85242	3.88387
NAA35	3.66287	3.6153	3.34786	3.52598	3.71906	3.84018
NAA38	3.22006	3.19235	3.45037	3.91613	3.8515	5.22523
NAA40	4.928	4.85286	4.68914	4.86916	4.11776	3.88574
NAA50	6.30198	6.37635	6.21763	6.75652	6.57536	6.79288
NAA60	3.13533	2.74662	3.24065	3.046	3.35789	2.78113
NAAA	1.22578	0.946604	2.82771	2.96264	-0.217212	-0.247125
NAALAD2	0.539412	0.537236	1.20023	1.25754	0.625168	-0.619042
NAB1	5.74905	5.43855	4.12713	4.03327	4.73259	4.40939
NAB2	2.5055	2.50296	2.4312	2.83076	1.83157	2.02123
NABP1	4.90761	4.98456	5.83619	6.11262	6.13026	6.02819
NABP2	4.35619	4.34208	3.48536	3.90659	4.2428	4.5483
NACA	6.29251	6.25571	6.76146	7.21601	7.11242	7.70265
NACA2	2.41103	2.4622	2.75109	3.35945	3.3806	3.78103
NACA3P	2.50317	2.5547	2.81374	3.30286	4.27565	4.69161
NACAD	0.784805	0.804012	-0.375995	-0.000171691	-1.01618	-1.06257
NACAP1	1.13285	1.11818	1.81733	2.25199	2.15778	2.39897
NACC1	4.70891	4.34789	4.4413	4.66662	5.61285	5.32876
NACC2	4.95823	3.76839	3.38633	2.95726	5.03011	4.09742
NADK	4.06827	3.87701	4.27077	4.5565	5.11939	5.15592
NADK2	2.87268	3.06659	4.95729	5.25145	3.72662	3.75066
NADSYN1	3.69323	3.44255	4.26562	4.44384	3.78367	3.5293
NAE1	4.91655	4.91923	4.91186	5.16962	5.22919	5.71587
NAF1	2.18968	2.17017	1.84441	1.98395	1.37865	1.493
NAGA	4.31214	4.24191	4.33414	4.53772	3.83354	3.72999
NAGK	3.31773	3.32832	3.80747	3.85696	3.7427	3.70903
NAGLU	3.86697	3.61398	4.12708	4.27271	3.22996	3.63313
NAGPA	2.99431	2.64232	2.68701	2.76589	2.26884	1.90802
NAGS	-0.768111	-0.707734	0.355738	0.563685	-0.217214	0.597145
NAIF1	2.70607	2.62463	1.71737	2.02048	2.88659	2.85554
NAIP	3.04992	2.90874	3.89392	3.72255	3.36874	2.95965
NALCN	0.920621	1.17021	-0.350462	0.646144	0.457836	0.613136
NAMPT	7.16809	6.48221	6.90522	7.43151	5.31198	5.34673

NAMPTP1	6.66145	5.9711	6.25813	6.86946	4.68225	5.00816
NANOS1	0.377835	0.537236	1.42385	1.07114	2.44823	1.97279
NANP	4.4918	4.55813	3.58631	3.73194	3.79193	3.77307
NANS	3.35892	3.40384	3.44219	3.98525	3.64193	3.70981
NAP1L1	7.56362	7.64956	7.31453	7.49301	7.85472	7.96698
NAP1L1P1	2.18016	2.30046	2.02662	2.21216	2.45727	2.47753
NAP1L1P3	2.00156	2.09051	1.7508	2.01577	2.10197	2.15149
NAP1L4	6.06546	6.05767	5.67797	5.99643	5.22108	5.56114
NAP1L4P1	1.4483	1.75317	1.18199	1.39115	2.26831	1.78645
NAP1L4P3	1.87109	2.05359	1.53007	1.98457	2.1591	2.10671
NAP1L5	-4.11163	-3.46127	-1.48269	-0.938639	2.13737	2.49297
NAPA	4.06361	3.92782	4.11477	4.59283	4.85568	4.90201
NAPB	3.97775	3.97802	4.32723	4.47988	3.52331	3.32395
NAPEPLD	4.28747	4.44027	3.19802	3.36488	4.62045	4.68881
NAPG	3.81958	3.93803	4.31332	4.84318	4.95531	5.06477
NAPRT	0.649238	0.281977	0.470407	0.147789	-0.350791	-0.497073
NARF	4.4633	3.97906	3.99514	3.72879	5.83475	5.08036
NARFL	2.5594	2.40955	2.5838	2.85888	2.7208	2.95669
NARS	7.18298	7.27639	5.81998	6.36331	6.21925	6.39755
NARS2	4.21659	4.28141	2.76561	2.8857	2.75235	3.20979
NASP	6.59313	6.53119	7.29439	7.38075	6.6817	6.11416
NAT1	0.345419	0.357428	0.860939	1.32585	0.282309	1.06153
NAT10	4.66121	4.47525	4.56215	4.86269	5.66272	5.32877
NAT14	3.03733	2.86134	2.59127	2.97067	4.24884	4.70901
NAT6	1.59587	1.57201	0.625467	1.00157	1.15053	1.22045
NAT9	3.4302	3.36846	3.86632	4.04978	4.58997	4.60162
NATD1	0.701877	0.318612	1.96116	1.87419	1.44302	0.769095
NAV1	5.91377	5.68517	5.63465	5.56492	4.07101	4.00064
NAV2	3.2623	3.55209	2.56093	3.42283	4.72733	4.52993
NAV3	3.94477	4.23759	4.63025	5.6208	2.47669	2.54122
NBAS	5.7688	5.85596	5.24955	5.53772	5.30581	5.43154
NBEA	5.13012	5.04824	3.76951	3.59239	4.57113	4.56438
NBEAL1	6.07713	6.03342	5.60808	5.93877	6.89203	7.35817
NBEAL2	2.79877	2.44105	2.44948	2.1904	3.44952	3.06147
NBL1	3.28241	2.92042	3.35592	3.07003	-0.000170248	-0.175494
NBN	4.96403	5.23588	4.60764	4.80031	5.66696	6.44014
NBPF1	5.10423	5.25254	4.95817	5.08078	5.13981	4.4356
NBPF10	5.14692	5.0418	4.65732	4.66852	4.00102	3.19583
NBPF11	4.20243	4.2805	3.55813	3.66805	3.50429	2.82601
NBPF12	3.91242	4.00822	3.59363	3.76113	3.95276	3.14059
NBPF14	5.41689	5.34605	5.377	5.48359	4.82612	4.26843
NBPF15	5.03251	5.03072	4.74502	4.77914	4.42624	4.16835
NBPF19	5.66287	5.60833	5.69248	5.60198	5.07354	4.19568
NBPF20	4.95031	4.89766	4.46548	4.60231	3.6251	2.72987
NBPF25P	2.58576	2.64545	1.78227	2.14504	2.23856	1.33137
NBPF26	3.9974	3.98851	3.4353	3.54575	3.2153	2.54143
NBPF3	3.06519	3.27435	2.71455	2.98942	1.0274	1.27277
NBPF8	5.45555	5.4902	4.83517	4.94304	5.03183	4.49744
NBPF9	5.60885	5.69801	5.15255	5.22336	5.0193	4.64901
NBR1	5.64766	5.79808	5.87041	6.26243	5.95625	6.50001
NCALD	3.09859	2.79926	-0.790968	-1.52346	0.395063	0.615294
NCAM1	4.22208	4.65071	2.05481	1.91892	3.38796	3.32226

NCAM2	4.59955	4.47672	-2.5977	-2.64851	?	?
NCAPD2	7.29309	7.04971	6.82157	6.9382	6.66842	6.1038
NCAPD2P1	0.60502	0.86082	0.460889	0.738111	0.340915	-0.0616585
NCAPD3	5.98337	6.0091	5.80861	6.08971	6.0446	5.79801
NCAPG	5.9721	5.95361	5.33252	5.67064	6.04141	6.37519
NCAPG2	6.1507	6.10128	6.22745	6.38677	5.35858	5.03052
NCAPH	5.20254	5.37908	4.05009	4.47707	4.91047	4.86219
NCAPH2	4.37773	4.15462	4.3203	4.59679	3.49324	3.74243
NCBP1	5.14679	5.23715	4.81788	5.14146	5.20154	5.3533
NCBP2	5.54457	5.53595	5.22081	5.43563	6.71658	6.74386
NCBP2-AS2	2.59555	2.44105	2.40145	2.6191	3.8981	3.95506
NCDN	3.91741	3.76972	3.0186	3.26234	4.51398	4.7185
NCEH1	3.94116	4.32913	4.48978	5.38904	5.12024	5.68986
NCK1	3.43663	3.48113	4.45491	4.61312	3.56971	3.57638
NCK1-AS1	-0.58107	-0.626072	1.49575	1.55573	-0.433896	-0.27296
NCK2	4.54919	4.58847	3.37586	3.56711	-1.38979	-3.70484
NCKAP1	6.77704	6.31913	6.39723	6.29395	7.27152	7.15241
NCKAP5	1.89714	1.70711	0.723445	0.833756	-4.65668	-3.12051
NCKAP5L	3.87267	3.65869	3.98824	3.92459	3.25856	3.21931
NCKIPSD	3.18204	3.06268	3.30633	3.54402	4.63373	4.3059
NCL	8.76525	8.79146	7.98339	8.32508	8.80031	8.5086
NCLN	4.78171	4.58904	4.34003	4.5187	5.25332	5.18202
NCLP1	1.49572	1.61934	0.82777	1.22245	1.41661	0.702755
NCOA1	4.14953	4.14448	4.28205	4.46102	4.86226	4.88854
NCOA2	4.59896	4.55327	5.36672	5.50542	4.53754	4.44556
NCOA3	6.05476	6.30948	6.39904	6.70088	5.57331	5.69307
NCOA4	5.80353	5.91455	5.85876	6.58244	6.39243	7.00492
NCOA5	4.63798	4.56713	3.83745	3.95726	4.07946	3.96039
NCOA6	5.2123	5.1141	5.08091	5.10183	4.48308	4.26626
NCOA7	5.07613	4.62129	4.56635	4.88482	4.96903	4.53049
NCOR1	6.22163	6.20584	6.88311	6.9333	6.95229	6.62922
NCOR2	4.04154	3.70833	4.02958	3.47989	4.72176	4.37809
NCR3LG1	5.13342	4.84377	1.71133	1.96266	3.9979	3.27913
NCS1	4.60639	4.15737	4.18498	4.29843	4.353	3.7991
NCSTN	5.47424	5.46657	4.75513	4.90798	5.39578	5.55595
NDC1	6.5902	6.80011	5.75789	6.28696	6.22078	6.71718
NDC80	3.81366	3.91978	3.94704	4.30796	4.63315	5.28692
NDE1	4.29462	4.15988	3.90888	4.18712	3.94334	3.73995
NDEL1	4.13064	3.7356	4.38157	4.33722	4.41449	3.73829
NDFIP1	4.80869	5.05233	4.91715	5.25641	5.18123	5.55303
NDFIP2	4.72562	4.84378	3.925	3.9342	4.49514	4.9824
NDNF	1.20786	1.35098	?	-6.09892	4.04272	3.44304
NDNL2	2.86317	3.33158	2.77359	3.52234	2.65258	3.31566
NDOR1	1.66727	1.50874	1.65571	1.8512	1.28225	1.23585
NDP	-4.37417	-2.6546	-1.79072	-0.916649	1.69157	2.80064
NDRG1	5.33949	5.44027	6.63745	6.63492	6.30129	4.81291
NDRG2	1.19579	0.996642	0.871828	0.761609	-4.65668	-3.70484
NDRG3	4.60925	4.66027	4.65568	4.97398	4.88366	4.9299
NDRG4	2.74801	2.70709	1.82213	2.03075	3.86911	4.24311
NDST1	5.32811	5.0558	6.26507	6.36206	5.94398	5.64917
NDST2	2.69507	2.62381	2.45926	2.67397	2.75616	3.06865
NDUFA1	3.5491	3.51129	4.32183	4.71273	5.42922	6.26794

NDUFA10	5.24233	5.18236	4.84438	5.12897	5.48402	5.31147
NDUFA11	4.07507	3.73573	3.12918	3.24365	4.76457	5.46684
NDUFA12	3.6047	3.74904	3.15187	3.71294	3.47668	4.27199
NDUFA13	4.70747	4.71028	4.1694	4.39298	5.81923	6.78981
NDUFA2	3.72718	3.67158	3.79375	4.08715	4.013	5.1843
NDUFA3	2.8735	2.7675	2.7251	3.20511	4.13604	4.50641
NDUFA4	5.76459	5.83691	5.62027	6.09259	5.73177	6.86266
NDUFA5	4.37	4.59312	4.07223	4.53267	4.36138	5.13013
NDUFA5P11	1.59351	1.61923	1.10544	1.50303	1.42176	2.43273
NDUFA6	3.73554	3.6113	4.74573	5.14855	4.12512	4.72374
NDUFA6-AS1	0.646227	0.569608	0.138526	-0.0212303	-0.230024	0.374155
NDUFA7	2.78067	2.558	2.29024	2.57251	3.20548	3.83251
NDUFA8	2.91379	2.87478	3.0308	3.50588	3.15668	3.64643
NDUFA9	4.78337	4.87646	4.74056	5.14593	4.34674	4.78378
NDUFA9P1	1.62351	1.73686	1.82213	1.8912	0.75262	0.832509
NDUFAB1	3.93481	3.85795	3.5427	3.99052	4.09706	4.79512
NDUFAF1	2.41207	2.42895	2.13391	2.25751	3.04878	3.53407
NDUFAF2	2.03143	2.11902	2.54121	3.0012	3.14282	3.96285
NDUFAF3	3.36919	3.59634	2.65726	3.31311	2.88238	3.96724
NDUFAF4	2.2198	2.40753	1.37111	1.90242	3.22233	3.86883
NDUFAF5	3.6352	3.64306	3.38633	3.44768	3.02794	3.40443
NDUFAF6	1.867	2.04497	1.496	1.81601	2.2496	2.91168
NDUFAF7	3.98487	3.99989	3.47569	3.56357	4.34158	4.29835
NDUFB1	3.34805	3.34932	2.66511	2.86272	3.62625	4.55656
NDUFB10	3.97862	4.21311	3.84852	4.45627	4.37654	4.87165
NDUFB11	4.4876	4.38213	4.4318	4.7862	3.94721	4.52237
NDUFB2	4.5021	4.36138	3.63597	4.06957	5.078	6.19588
NDUFB3	3.46701	3.51719	3.08084	3.63062	4.29699	5.46959
NDUFB4	4.02701	4.06693	4.48677	4.88807	4.96063	5.50694
NDUFB5	3.84395	3.96027	4.54609	4.92802	5.3141	6.18202
NDUFB6	3.82645	3.94548	3.20018	3.78447	4.7852	6.04982
NDUFB7	3.01346	2.64044	2.82772	3.16498	4.00148	5.00839
NDUFB8	5.26563	5.54773	4.18346	4.68449	4.7619	5.18439
NDUFB9	5.24496	5.42397	5.06384	5.64706	5.90193	6.49344
NDUFC1	3.32363	3.30203	2.64039	3.03584	3.31421	3.7677
NDUFC2	5.76911	5.62831	4.84882	5.21702	4.57306	4.92264
NDUFS1	6.06449	6.09147	5.66508	6.00163	6.89651	7.11224
NDUFS2	5.66231	5.44102	4.68914	4.89609	5.79423	5.68989
NDUFS3	4.24396	4.33768	3.99095	4.52855	5.13878	5.84538
NDUFS4	3.29386	3.42742	3.74277	4.20632	4.38817	5.43091
NDUFS5	5.6854	5.57398	4.43393	4.83368	6.19118	6.9082
NDUFS6	3.73763	3.76477	3.1134	3.68071	4.53506	4.65193
NDUFS7	2.23687	1.87383	2.0493	2.49033	2.78718	3.40801
NDUFS8	3.99004	3.74172	4.22374	4.65686	4.18125	4.62867
NDUFV1	5.45667	5.34611	5.10418	5.46576	4.95623	5.01523
NDUFV2	3.48675	3.59113	4.33636	4.69908	4.87275	5.60999
NDUFV2P1	2.72316	2.74008	3.46392	3.9457	4.22769	4.90511
NDUFV3	4.2123	4.1592	3.59455	3.66596	4.14865	3.92446
NEAT1	10.7483	10.3549	12.3194	12.1531	10.7675	9.75982
NEB	2.3316	2.41061	1.25116	1.30061	2.35372	1.54125
NEBL	-0.489437	-0.0703908	-2.48229	-2.02079	5.01034	4.16364
NECAB1	1.27071	1.3672	-1.74367	-1.2995	1.13735	0.857193

NECAB3	3.09046	2.6483	4.24932	4.38141	3.94517	3.52931
NECAP1	4.53036	4.685	4.11681	4.73353	4.71239	5.073
NECAP2	4.34051	4.37241	4.54269	4.77304	5.19842	5.44555
NEDD1	4.90361	5.0489	3.85676	4.18233	4.65086	4.97977
NEDD4	5.8882	5.95234	6.46593	6.96655	6.8674	7.28497
NEDD4L	6.25363	6.48641	4.05129	4.78218	3.36757	3.40703
NEDD8	5.42723	5.4559	4.3874	4.82964	5.95254	6.2967
NEDD9	4.41334	4.53432	3.606	3.35061	6.48005	5.84161
NEGR1	2.96449	3.50869	-2.37543	-3.78492	2.12107	3.05151
NEIL1	1.79773	1.48217	1.46519	1.41662	1.44676	0.421781
NEIL2	2.77869	2.82256	2.02595	2.41082	3.18361	2.98329
NEIL3	3.12747	3.27525	4.3302	4.7761	3.24658	3.49786
NEK1	3.11471	3.24279	3.342	3.375	3.68065	3.78313
NEK10	2.67162	2.84322	2.23012	2.02048	2.75755	3.028
NEK11	-1.37679	-0.970724	0.558032	0.810106	0.983605	1.41227
NEK2	4.41965	4.6604	4.14173	4.77376	4.21997	4.6117
NEK3	0.409538	0.444137	1.20023	1.49854	1.93963	2.23582
NEK4	4.81055	4.81959	4.32823	4.52353	4.8951	4.82024
NEK6	4.56887	4.10225	4.38822	4.56624	4.88803	4.3781
NEK7	5.10018	5.541	4.86767	5.32943	5.16703	5.84451
NEK8	0.35629	-0.292758	0.814903	0.367002	0.258664	0.151378
NEK9	3.29535	3.03456	4.63366	4.68035	4.37656	4.18888
NELFA	2.41988	2.29872	2.0405	2.07607	2.64804	2.5317
NELFB	3.43407	3.23064	3.0926	3.27919	3.81441	3.85933
NELFCD	6.10766	6.04618	4.92238	5.15444	6.04574	5.85885
NELFE	4.5811	4.65414	4.01982	4.66018	5.0535	5.56357
NELL2	2.58864	2.57883	-6.17309	-4.52056	-2.65941	-4.11926
NEMF	5.3116	5.23895	4.41539	4.57976	4.74982	4.86573
NEMP1	4.93207	4.98421	5.97892	6.24043	4.91715	5.04147
NEMP2	3.73554	3.79218	2.94319	3.3258	2.79913	2.92992
NENF	4.3002	4.34689	3.55963	3.86845	4.03751	4.70373
NEO1	-1.1752	-1.50825	6.16021	6.26699	-1.37455	-2.53597
NES	8.49227	8.52698	-0.206089	-0.0426024	8.98728	8.36398
NET1	4.54765	4.85964	4.0429	4.40688	4.59294	4.67373
NETO2	5.03773	5.12024	-3.59683	-6.09892	-3.6585	-2.8983
NEU1	5.1801	5.17559	4.92828	5.70135	6.06329	6.42774
NEU3	4.42762	4.38108	3.76192	3.93146	3.68288	3.51729
NEURL1B	0.05414	0.356426	2.63026	2.86842	-0.235834	-0.0939684
NEURL4	3.46205	3.33805	3.87487	4.03321	4.06936	3.51709
NEXN	2.44962	2.30204	3.17505	3.49322	3.46869	3.10548
NF1	6.04741	5.96288	6.73526	6.62873	5.73289	5.35352
NF2	7.17924	6.88662	5.78475	5.95994	7.9107	7.56419
NFASC	3.96803	4.04494	-0.973801	-0.327299	-1.85245	-2.53597
NFAT5	5.92976	5.80215	7.07602	6.79164	6.53611	6.08159
NFATC1	2.66287	2.35415	0.793829	0.913608	-1.53062	-1.12145
NFATC2	5.25001	4.64502	-0.598354	-0.52367	-0.217212	-1.70626
NFATC2IP	4.60869	4.56988	4.57219	4.68721	4.62912	4.20167
NFATC3	4.9178	4.86259	4.86388	4.95862	5.49359	5.44052
NFATC4	3.2033	2.97803	2.17844	2.1152	-6.64581	?
NFE2L1	7.79785	7.77864	6.95729	7.26321	7.50468	7.23068
NFE2L2	5.96753	5.93199	7.37229	7.28381	5.2762	5.31053
NFE2L3	7.35115	7.49635	5.90125	6.33332	5.05992	5.42699

NFE2L3P1	2.16317	2.40918	0.157044	0.845903	-0.267833	-0.00308489
NFIA	3.82056	4.18101	3.0525	2.786	2.30549	2.92265
NFIB	5.87526	6.08015	4.73758	5.01916	6.17441	6.45342
NFIC	3.87267	3.46937	4.17841	3.98392	4.67842	4.31148
NFIL3	2.8284	1.80397	3.51691	3.74915	2.31699	2.09421
NFIX	3.26382	2.57608	3.80658	3.6208	5.19142	4.71639
NFKB1	5.82744	5.88034	3.83746	4.20064	4.02532	4.04315
NFKB2	4.63631	4.68878	2.77649	3.11518	2.58763	1.94436
NFKBIA	5.25293	5.21134	4.73458	5.30585	4.01741	4.31009
NFKBIB	2.27248	2.23414	1.62387	2.21312	2.4767	2.10709
NFKBID	1.00834	1.3381	0.308431	0.798133	-0.0163223	-0.706491
NFKBIE	3.78776	3.9497	2.53758	2.81599	2.04362	2.08772
NFKBIL1	1.14646	1.20963	1.07392	1.2041	2.12107	2.37012
NFKBIZ	4.29562	4.05344	4.52422	5.2429	2.83369	2.19397
NFRKB	4.19421	4.15921	4.30129	4.50127	4.77433	4.34856
NFS1	3.93027	3.97072	3.55794	3.80107	4.36066	4.24311
NFU1	2.23758	2.26168	2.34397	3.05357	2.26748	3.24748
NFX1	4.86075	4.90072	4.3459	4.60111	5.86521	5.71429
NFXL1	3.56504	3.62001	2.65007	2.90521	3.77428	3.71375
NFYA	4.4334	4.19358	4.43025	4.4313	4.73577	4.65083
NFYB	3.89056	3.82486	3.43669	3.60713	3.03839	3.46286
NFYC	3.49931	3.63824	3.79784	4.0985	3.87058	3.78001
NFYC-AS1	1.52519	1.49532	2.55505	2.57325	0.972729	0.64308
NGDN	4.12029	4.16742	2.63347	2.94917	3.5661	4.07628
NGFR	4.3627	2.28126	-4.59508	-3.10759	-4.33548	-1.18284
NGFRAP1	5.08757	4.93909	4.82977	5.11878	5.24433	5.69842
NGLY1	3.93231	4.11945	3.91179	4.11999	4.64164	4.7981
NGRN	5.63208	5.64495	5.33488	5.67623	6.05905	6.27
NHEJ1	2.42654	2.3435	2.15453	2.33493	2.82058	3.17021
NHLRC2	5.17666	5.23628	5.1806	5.08961	5.51086	5.14893
NHLRC3	3.34805	3.57881	3.74128	3.94781	3.65484	4.21958
NHP2	4.71727	4.81924	3.99978	4.57558	5.41941	6.20954
NHP2P1	2.7025	2.81036	2.08362	2.5803	3.70644	3.89134
NHS	3.3453	2.98631	3.21523	3.60714	-1.01618	-1.70626
NHSL1	4.39897	4.45602	2.19587	1.87419	4.88657	4.70478
NHSL2	1.23776	1.68721	1.39422	1.97366	2.02657	2.10079
NICN1	2.70394	2.7269	2.97121	3.10792	2.62719	2.46765
NID1	7.67027	7.73243	6.09106	5.99055	5.85241	5.19714
NID2	5.49862	5.66801	4.45229	4.29644	-2.81814	-3.34576
NIF3L1	3.74895	3.78508	3.00877	3.35674	3.8134	4.35398
NIFK	5.01087	4.99562	3.52145	3.87655	5.26716	6.11725
NIFK-AS1	2.58841	2.52514	2.10345	2.15952	1.89335	1.71476
NIM1K	-0.395122	-1.07024	-1.32519	-0.616766	1.90196	1.2122
NIN	6.13972	6.63519	5.10186	5.66801	5.52456	5.74654
NINJ1	2.66507	2.41981	4.11224	4.72167	3.04706	2.85553
NINL	3.78171	3.57471	2.28002	2.15329	-2.75246	-4.70296
NIP7	4.69952	4.84049	4.07998	4.44207	6.05711	6.4517
NIPA1	4.67705	4.73436	4.73608	5.01398	5.1965	4.7971
NIPA2	5.47445	5.5527	5.18552	5.60055	6.00796	6.17669
NIPAL1	3.21423	3.80448	-3.92409	-2.10853	-1.61414	-1.13601
NIPAL3	5.16543	5.25179	3.60432	4.56445	5.52386	5.7593
NIPBL	5.97577	5.82745	6.42008	6.21083	6.25273	6.07259

NIPSNAP1	4.51341	4.64567	1.71133	2.01014	4.97541	5.43347
NIPSNAP3A	1.45578	1.70209	2.03567	2.27489	2.36207	3.5076
NISCH	4.79025	4.52867	5.09201	5.05606	5.05092	4.46945
NIT1	3.68902	3.73042	3.47232	3.72646	3.39429	3.54671
NIT2	4.20931	4.19089	4.68914	4.68964	4.15906	4.43411
NKAP	2.93755	2.73438	2.96877	3.21985	3.27927	3.51969
NKILA	5.26483	5.57901	0.629333	0.654481	1.01434	0.93191
NKIRAS1	3.21317	3.69844	2.40932	2.88806	3.72452	4.76028
NKIRAS2	3.87745	3.80688	4.13886	4.44219	5.19623	5.22961
NKRF	2.91011	3.0743	3.47634	3.74752	4.50657	4.54473
NKTR	6.93731	6.85993	7.05858	6.85204	7.14491	6.37141
NKX2-2	1.50144	1.35737	-0.453959	-0.725802	?	?
NKX3-1	0.727258	1.20963	2.42012	3.08347	0.670686	0.863209
NLE1	4.19422	4.12305	3.23019	3.66596	4.0199	4.09158
NLGN1	3.6857	3.69954	-5.1783	-4.52056	3.0436	3.60389
NLGN2	3.41857	3.27187	3.99763	3.59161	3.26004	2.78915
NLGN4X	4.7759	4.59968	-4.9135	?	3.3411	3.46059
NLGN4Y	-4.00826	-4.36068	1.75076	1.59165	?	-5.63497
NLK	4.25148	4.47893	3.66667	3.79354	4.62855	4.65413
NLN	4.49075	4.54977	4.72034	4.79128	5.36688	5.5424
NLRC5	1.27251	1.74176	4.12595	4.28348	5.16663	4.96216
NLRP1	3.58749	3.43801	3.44947	3.66596	1.43776	0.68568
NLRX1	1.57708	0.988427	2.23435	1.92462	1.72342	1.06153
NMB	2.8107	3.05286	0.00643448	0.619178	2.2008	3.10064
NMD3	4.82777	4.98622	4.89009	5.36686	4.98038	5.59189
NMD3P1	-0.0513199	0.0310987	-0.165243	0.506947	-0.10558	0.522849
NME1	6.71517	6.70205	5.34022	5.86365	6.6498	7.17946
NME1-NME2	6.88957	6.76323	5.7681	6.25748	6.8574	7.645
NME2	3.9371	3.6776	2.14715	3.0923	3.77317	4.51264
NME2P1	3.79772	3.83827	2.83535	3.2914	3.81182	4.24739
NME3	1.31777	1.20963	1.61741	1.78602	1.61584	2.99026
NME4	3.46834	3.24796	4.31978	4.42645	4.55946	4.5412
NME6	2.93936	3.12587	2.48538	2.87417	3.6053	3.51729
NME7	3.85638	3.92549	3.32586	3.7601	4.19473	5.16247
NME9	0.480917	0.570667	0.371169	-0.0212318	-0.217212	-0.706493
NMI	2.45575	2.62728	1.77651	2.01532	2.55645	3.32671
NMNAT1	1.35625	1.37009	2.5273	2.80706	2.67509	2.63315
NMNAT2	3.38705	3.5623	1.02599	2.09087	2.33406	2.90432
NMRAL1	1.84835	1.89254	2.22164	2.45724	-3.6585	-2.70579
NMRK1	0.923035	0.756391	-0.325375	-0.716287	0.679613	1.27061
NMT1	6.23368	6.1704	6.10419	6.47635	6.42617	6.39326
NMT2	2.99617	2.78156	4.70343	5.02859	3.25663	3.34278
NNMT	4.20329	3.05286	4.8291	5.65062	4.0323	4.38604
NNT	5.52917	5.58529	5.96281	6.26656	5.9721	6.18286
NNT-AS1	2.86851	2.94867	2.89679	2.98292	3.69232	3.54778
NOA1	3.01179	3.04479	2.62711	3.0437	4.74492	5.06505
NOB1	3.89108	3.90362	3.8349	4.20467	4.85394	5.06348
NOC2L	5.78297	5.76056	5.05536	5.47062	6.46441	6.57386
NOC3L	4.95107	5.04691	4.16188	4.45436	5.5664	6.22325
NOC4L	1.53936	1.3381	1.30838	1.67257	1.09463	1.51249
NOCT	3.49936	3.57333	2.48183	2.82192	3.34812	2.79712
NOD1	3.16343	3.13864	2.85155	2.77809	2.31894	1.96541

NOL10	4.67566	4.7906	3.67081	3.92579	5.02948	5.42877
NOL11	4.85335	4.99053	4.63959	5.04144	5.56492	6.23032
NOL12	3.23018	3.05286	2.62062	2.76155	2.47669	2.41742
NOL3	1.99629	2.10208	2.84605	3.17382	3.519	3.73175
NOL4L	4.6275	4.61516	4.99325	4.92419	4.07212	3.69594
NOL6	5.61041	5.56125	4.22374	4.66431	6.70453	6.53586
NOL7	3.73559	4.00268	3.34639	3.95502	4.72306	5.27954
NOL8	4.95286	4.93264	4.1817	4.44605	4.65771	4.64971
NOL9	5.14012	5.34087	4.59783	5.12777	5.27742	5.00838
NOLC1	6.76711	6.7726	5.57426	5.97662	7.05406	6.87963
NOM1	5.06785	5.12305	4.4275	4.67089	3.81644	3.38074
NOMO1	6.89233	6.73354	6.68198	6.92089	6.23338	6.09572
NOMO2	6.40278	6.31293	6.69123	7.08224	6.36714	6.23209
NOMO3	5.67737	5.61906	6.17192	6.4477	5.87775	5.71754
NONO	7.77344	7.51143	7.5836	7.64235	8.30649	7.66209
NOP10	5.14561	5.01136	3.80516	4.19609	5.26559	5.92851
NOP14	4.98283	4.95612	4.11113	4.47071	5.4376	5.25545
NOP14-AS1	3.13926	3.04086	2.58782	2.5733	3.25773	2.93704
NOP16	4.35717	4.29419	3.32223	3.75177	5.05968	5.45832
NOP2	5.54765	5.59344	4.52814	4.94712	4.98849	5.00752
NOP56	7.02831	6.99687	6.89953	7.10062	6.39738	6.1846
NOP58	6.0908	6.1008	4.68293	5.09451	5.85609	6.1636
NOP9	4.91431	4.9376	4.10831	4.37453	5.03968	4.95741
NOS1AP	2.05861	1.91161	-2.5977	-1.86039	1.67188	1.67185
NOSIP	3.23903	3.0568	3.21736	3.78599	3.90003	4.68774
NOSTRIN	1.85937	1.97597	1.12941	1.01017	1.1471	0.483199
NOTCH1	3.60127	3.12775	2.48183	2.24874	2.41117	2.1262
NOTCH2	7.68703	7.79376	7.78485	8.13992	8.38486	8.08125
NOTCH2NL	5.50243	5.42534	5.41067	5.37673	4.41002	4.10832
NOTUM	-1.27248	-3.00228	-6.17309	-4.10635	1.88661	2.48805
NOV	3.58401	3.025	2.43854	3.19496	-4.07293	-1.79933
NOVA1	1.71458	1.60328	?	-6.09892	1.68845	1.72795
NPAS2	5.30673	4.98397	1.63043	1.7255	0.814206	0.173882
NPAS3	-0.255978	-0.46266	3.83189	3.53864	3.88078	3.01439
NPAT	3.54559	3.58109	5.25546	5.40714	4.54519	4.76616
NPC1	4.83828	4.83207	5.97659	6.67597	4.26117	4.06123
NPC2	4.86175	4.61462	4.21415	4.50863	4.59294	4.98591
NPDC1	-0.549135	-0.629746	2.61091	2.66926	1.15352	1.56945
NPEPL1	2.75697	2.20521	2.0389	1.99019	2.89379	2.83388
NPEPPS	5.95219	5.86756	6.3014	6.23512	6.12355	5.90494
NPHP1	1.82058	1.85459	1.43123	1.64609	1.77851	2.03478
NPHP3	3.82252	3.7716	3.99699	3.94831	3.5245	2.9676
NPHP3-ACAD11	0.804483	0.731479	0.231654	-0.201254	-0.103888	0.2931
NPHP4	2.75831	2.51158	3.03565	3.0586	2.61583	2.3377
NPIPA1	3.27332	3.02163	3.62535	3.6505	3.02525	2.5586
NPIPA2	0.607872	0.464836	0.974948	1.16467	0.451509	-0.836192
NPIPA5	-0.509136	-0.80933	1.02462	1.17349	-0.569486	-1.73922
NPIPB11	0.250113	0.197941	0.433387	0.282487	-0.425174	-0.604174
NPIPB3	2.79068	2.50988	3.04208	3.04822	1.90799	1.72346
NPIPB4	3.29593	3.3364	3.96712	3.97097	3.14202	2.36238
NPIPB5	4.65151	4.55133	5.28624	5.13504	4.27416	3.60747
NPIPB9	-0.29928	-0.053925	0.981823	0.72534	0.183353	0.148374

NPIPP1	0.301599	0.505921	1.65582	1.49793	0.823912	-0.151612
NPL	2.27248	2.40136	-0.598354	0.445854	-1.61549	-0.752286
NPLOC4	6.69378	6.67446	6.47066	6.77591	7.35392	7.11245
NPM1	7.95482	7.96158	7.98672	8.41988	8.73297	9.24865
NPM1P12	-0.367843	-0.0946522	-0.430046	0.248365	0.256685	1.14266
NPM1P18	0.216697	-0.27666	0.0321714	0.976144	0.566533	1.74317
NPM1P19	1.40964	1.12925	1.10487	1.96302	1.59708	2.38977
NPM1P24	3.83641	3.94536	4.12226	4.36839	4.6861	4.99087
NPM1P26	1.10242	1.03757	1.5056	1.5474	1.42602	1.57935
NPM1P27	7.2786	7.26081	7.43683	7.79054	8.1195	8.60034
NPM1P29	-0.413499	-0.333394	1.73537	1.195	1.51964	0.769095
NPM1P39	4.23438	4.27906	4.37902	4.82134	5.03175	5.57385
NPM1P46	0.780176	1.04516	0.732877	1.22222	1.61065	2.01832
NPM1P5	-0.0400162	-0.0479677	0.242816	0.740392	1.04004	1.50186
NPM1P6	3.4832	3.44057	3.4564	4.12945	4.30775	4.78362
NPM3	3.1131	3.26508	2.46395	2.91077	3.17731	3.66832
NPR2	2.49774	2.07616	3.22074	3.18103	1.83353	1.5077
NPR3	2.50551	1.72701	0.0833836	0.46112	3.01034	2.42259
NPRL2	3.62895	3.67523	3.03527	3.48374	3.84556	4.05162
NPRL3	1.38458	1.10427	2.89238	2.67214	2.16735	1.92229
NPTN	5.74979	5.59243	5.578	5.88055	5.56066	5.29258
NPTN-IT1	1.80477	1.65093	1.92503	1.77384	1.37315	0.615294
NPTX1	?	-6.45019	3.50821	3.81599	-2.65941	-3.12051
NPTXR	0.863225	0.61405	3.67756	3.54042	-3.48878	-2.8983
NQO1	6.93109	6.98809	9.57494	9.89164	8.65995	9.46313
NQO2	2.15518	2.43669	4.65644	5.04984	3.36832	3.19374
NR1D1	0.0021048	-0.394367	1.23829	1.39675	1.34822	1.21972
NR1D2	5.04282	5.24882	4.18389	4.83661	5.35022	5.77861
NR1H2	3.25075	2.99659	3.0926	3.54402	4.29388	4.71849
NR1H3	0.685662	0.604081	2.34037	2.69611	0.724511	0.769602
NR2C1	3.64522	3.513	3.7663	3.67744	3.39226	3.14816
NR2C2	6.0169	6.0086	5.61335	5.66668	6.14894	5.77724
NR2C2AP	3.00571	2.78899	2.02467	2.38121	3.03712	3.31274
NR2E1	?	?	-6.17309	-6.09892	2.85342	3.02801
NR2F1	1.0603	0.581631	6.5491	6.05793	4.2122	3.86275
NR2F1-AS1	1.82582	1.73194	4.79926	4.49302	2.99651	2.87527
NR2F2	4.2168	4.52087	2.81366	3.03584	3.16624	3.47319
NR2F2-AS1	1.78553	2.10695	0.30688	0.382922	-0.895194	-1.36737
NR2F6	3.25221	3.11836	2.93283	3.07109	4.12104	4.00064
NR3C1	5.49967	5.48149	6.76569	6.83001	6.78374	6.85145
NR3C2	1.18361	1.15192	-0.0748604	-0.301307	0.606556	0.200291
NR4A1	5.0562	5.28426	0.138369	0.139221	-0.511857	-0.421133
NR4A2	3.41336	2.6483	-2.37543	-1.64896	-0.250761	-1.12145
NR6A1	2.69539	2.33159	2.37108	1.96266	1.94334	1.15132
NRAS	6.38804	6.45059	5.54141	6.06015	7.6597	7.84726
NRAV	2.2696	2.27188	1.88797	2.24434	2.18206	2.42258
NRBF2	3.18637	3.23863	2.48073	2.90435	4.04671	4.19414
NRBP1	5.43338	5.34009	4.92106	5.1725	5.91943	6.4735
NRBP2	2.10184	2.12214	3.1603	3.29844	3.41233	3.10048
NRCAM	0.520176	0.370142	5.70938	5.77495	6.78943	7.04419
NRD1	6.55715	6.5865	6.59476	6.89268	6.73083	6.86746
NRDE2	4.49185	4.29312	3.22559	3.20021	3.67832	3.28679

NREP	4.55972	4.51821	4.39844	4.21839	4.93685	4.78423
NRF1	3.48579	3.39358	2.95091	3.23547	3.05394	3.1325
NRG1	3.02202	3.06072	5.97381	6.38201	3.93214	3.55773
NRG2	0.243547	0.318612	1.05494	1.31748	2.0778	2.02123
NRIP1	7.0684	6.79418	6.49223	6.49906	5.57384	5.89459
NRIP3	1.29532	1.3764	-4.18092	-4.10635	2.42717	2.82468
NRM	3.07901	3.07244	3.53758	3.73038	2.93775	2.80109
NRN1	2.5055	2.43198	3.16077	3.61227	2.55159	2.32121
NRP1	6.34644	6.52478	6.14825	6.63483	7.60001	7.63826
NRP2	6.58963	6.86023	4.9238	5.80922	9.46036	9.59959
NRSN2	3.56301	3.39202	4.5117	4.70495	3.84151	4.06311
NRXN3	4.32675	4.65739	2.12028	2.32582	1.91336	1.90802
NSA2	4.65489	4.8239	4.61561	5.33835	5.66014	6.45399
NSD1	6.33182	6.3284	6.81839	6.82633	6.86827	6.64491
NSDHL	4.4009	4.35414	3.62383	4.07233	3.69944	3.87071
NSF	5.14603	5.31712	4.93587	5.37128	5.60235	5.91422
NSFL1C	4.82203	4.86695	5.49594	5.80106	4.44428	4.40832
NSFP1	2.26368	2.08535	1.07675	0.272107	-0.15233	0.985464
NSL1	4.11789	4.18281	4.17951	4.43614	4.23828	4.83534
NSMAF	4.16045	4.19982	4.19151	4.63768	4.20773	4.27343
NSMCE1	2.84782	2.80862	3.23644	3.62926	3.21467	3.80504
NSMCE2	3.11631	3.18104	2.97635	3.2878	3.24808	4.25039
NSMCE4A	3.71962	3.71443	2.83393	3.10151	3.68333	4.25159
NSMF	3.29527	2.78034	3.77213	3.92322	3.62624	3.38604
NSRP1	3.40084	3.33985	4.21972	4.50027	4.39208	4.06429
NSRP1P1	0.307382	0.393866	0.684253	0.492294	0.541782	-0.3042
NSUN2	5.96093	6.00654	5.41637	5.7757	6.13084	5.87779
NSUN3	2.52214	2.52162	3.06832	3.31328	2.67848	2.71211
NSUN4	4.47272	4.54277	4.02227	4.31638	4.29824	4.24018
NSUN5	0.631384	0.288595	1.51082	1.83826	1.44912	1.29906
NSUN5P1	4.20771	4.19515	4.51772	4.53119	4.63444	4.08892
NSUN5P2	1.536	1.60718	1.9645	2.19911	2.06239	1.81902
NSUN6	2.42247	2.57059	1.69303	1.71138	2.81137	2.87072
NT5C	2.99854	2.92876	2.90773	2.80953	3.22432	3.81426
NT5C2	3.91361	4.01088	5.5421	5.79871	5.68136	5.57427
NT5C3A	3.80169	4.03352	3.56995	3.97187	3.69826	4.00621
NT5C3AP1	1.16561	1.52292	1.14603	1.4076	1.31169	1.43048
NT5C3B	3.56301	3.41673	3.75605	4.08716	4.12841	4.41611
NT5DC1	2.84129	3.01108	3.86731	4.27191	3.30431	4.03465
NT5DC2	5.20768	4.86745	4.25111	4.33095	5.0836	4.70267
NT5DC3	4.69859	4.7405	3.37872	3.77839	5.60089	4.81782
NT5E	6.9905	7.60592	4.15074	5.79728	4.16386	5.64311
NTAN1	3.22719	3.13056	2.88883	3.23898	2.60271	2.80962
NTAN1P2	0.00124018	0.212021	0.517004	0.659431	0.0812851	-0.309336
NTHL1	2.77055	2.37635	1.67924	1.89682	1.97989	2.68122
NTM	4.87449	5.10837	-5.1783	-3.78492	1.83949	2.46823
NTMT1	3.10977	2.95184	2.71592	3.24671	3.86589	4.12425
NTN1	2.01175	2.03702	1.02599	2.18581	-1.23372	-1.38442
NTN4	4.1179	4.50152	1.83331	2.45343	4.4508	4.29609
NTNG1	2.64966	2.34452	-5.1783	-4.10635	2.77433	2.99373
NTNG2	-3.37567	-2.87684	1.51	1.60545	-3.85089	-3.12051
NTPCR	4.33865	4.37576	5.37034	5.48523	4.29009	4.56143

NTRK3	-5.69162	-6.45019	-2.5977	-2.40763	2.8962	3.79711
NUAK1	3.51767	3.47085	2.84715	3.33825	3.55158	3.60163
NUAK2	1.60474	2.01586	2.1019	2.44577	3.43373	3.67482
NUB1	4.75686	4.90536	4.95163	5.54991	5.15503	5.56649
NUBP1	2.78714	2.72621	2.09253	2.38393	2.48217	2.79665
NUBP2	3.08064	2.84777	2.74722	2.86272	2.90194	3.49296
NUBPL	2.50794	2.55397	2.08796	2.05107	2.32554	2.44812
NUCB1	5.80175	5.60388	5.87305	5.95812	5.86054	5.7486
NUCB2	4.5213	4.50797	4.91713	5.56536	3.73736	3.99546
NUCKS1	8.20242	8.15214	8.05622	8.13813	8.5465	8.53987
NUDC	5.46688	5.3589	4.61899	5.05809	5.72656	5.56904
NUDCD1	5.26743	5.39474	4.42749	4.81078	4.79346	5.64311
NUDCD2	3.55476	3.58154	4.12822	4.16029	4.43442	4.4174
NUDCD3	5.50364	5.52548	5.68608	5.87734	5.49291	5.56005
NUDT1	3.1805	3.20427	3.31234	3.64606	3.38818	3.98851
NUDT11	1.16882	1.12217	2.52827	2.78941	1.01848	0.893261
NUDT12	3.56301	3.58156	3.42381	3.50034	3.85931	4.29891
NUDT13	1.0472	1.18944	-0.111639	0.179893	0.840499	0.133682
NUDT15	4.04154	4.11269	2.96114	3.38703	4.16146	4.32808
NUDT16	2.64966	2.37322	3.92368	4.11153	4.03754	3.41998
NUDT16L1	2.35893	2.03304	2.1019	2.37097	3.05051	3.45568
NUDT18	0.727258	0.581631	1.18285	1.77994	0.823578	1.27061
NUDT19	2.6296	2.6587	2.58467	3.00231	3.31697	2.82467
NUDT2	1.60474	1.3764	2.63027	2.9892	3.19766	4.12617
NUDT21	6.46691	6.46177	6.61555	6.80197	7.5035	7.5269
NUDT22	2.94331	2.9309	2.56545	2.95467	2.78734	3.12342
NUDT3	5.32892	5.43825	5.68047	5.88603	6.63501	6.58112
NUDT4	5.73529	5.86106	4.65269	5.09919	5.68957	5.93395
NUDT4P1	2.52567	2.30411	0.312514	1.41699	2.53119	2.76398
NUDT4P2	1.96423	2.55241	1.21032	1.55132	1.52579	2.21808
NUDT5	5.09666	5.02154	4.27434	4.52908	5.38818	5.61904
NUDT6	2.31455	2.48245	3.10138	3.39412	1.71147	1.7239
NUDT9	3.29668	3.29205	2.54781	2.72723	2.86913	2.73624
NUF2	4.9389	5.04443	4.10649	4.28026	4.79707	5.4831
NUFIP1	2.93454	2.88185	2.06357	2.35925	2.55731	2.86889
NUFIP1P	0.536162	0.754682	0.311329	0.078883	0.584299	0.776929
NUFIP2	6.87609	6.97438	7.49715	7.79033	7.89948	7.89434
NUMA1	7.16624	7.18954	6.98019	7.07092	6.24772	6.04157
NUMB	4.52008	4.35573	4.70781	4.73772	4.4185	3.94613
NUMBL	1.74389	1.77559	2.68685	2.80107	3.11118	2.919
NUP107	5.40347	5.51386	4.5895	4.97661	4.81223	5.24227
NUP133	5.13696	5.26846	4.66117	5.13672	4.94052	5.20242
NUP153	6.41456	6.41365	5.98021	6.03761	6.41821	6.30559
NUP155	6.46626	6.31235	6.20451	6.28907	6.52052	6.31772
NUP160	5.8025	5.79511	5.77862	6.05577	6.01235	5.96834
NUP188	5.68569	5.64075	4.87446	5.09634	5.58195	5.3721
NUP205	7.18306	7.26348	6.49283	6.82944	6.50601	6.45474
NUP210	?	?	-5.1783	-3.78492	5.75466	5.16287
NUP214	4.9668	4.91656	4.73072	4.83506	4.9716	4.61427
NUP35	3.59554	3.65733	2.15188	2.63938	3.54425	4.13249
NUP37	4.03308	3.86695	3.20665	3.58814	3.43245	4.19565
NUP43	5.18584	5.26421	4.83536	5.18923	5.8861	6.03167

NUP50	6.52832	6.42213	5.81224	5.83117	5.82726	5.50329
NUP50-AS1	1.40949	1.79457	1.66826	1.94105	0.529601	0.937209
NUP54	4.59266	4.79982	3.93022	4.50311	5.26376	5.89133
NUP62	5.00896	4.88811	4.77305	5.04124	5.56957	5.39462
NUP85	4.67052	4.68943	4.77285	5.06796	4.88223	5.0263
NUP88	4.8888	5.01508	4.87219	5.3651	5.12358	5.56543
NUP93	5.23901	5.23193	5.24011	5.56269	6.08995	5.87872
NUP98	7.10515	7.0074	6.41312	6.73101	6.35348	6.09941
NUPL1	6.27943	6.23325	5.86461	6.06014	6.22917	6.08891
NUPL2	4.01184	4.01693	3.47647	3.72406	3.85834	3.97452
NUPR1	-6.68418	-5.45651	2.541	3.00752	1.53448	1.93717
NUS1	4.90835	5.04059	4.66683	4.8941	5.22208	5.38537
NUS1P1	2.21013	2.42217	1.87057	2.11439	2.51373	2.82081
NUSAP1	6.40108	6.37083	5.59495	5.84828	6.0255	6.2125
NUTF2	5.06946	5.11335	5.03745	5.40417	6.25247	6.35076
NUTF2P2	0.276461	0.547914	0.391009	0.621918	1.82478	1.729
NUTM2A	-0.483037	-0.69698	1.17068	0.265149	0.444784	-0.88417
NUTM2A-AS1	3.2939	3.23624	2.93998	2.99204	3.13258	3.1293
NUTM2B	0.374589	0.348176	-0.0969247	-0.359492	1.49119	0.662297
NUTM2B-AS1	4.16359	4.20503	4.07184	3.89403	4.28699	3.93746
NUTM2D	1.05166	1.15463	1.18641	1.28423	0.648456	0.682483
NUTM2G	0.557248	0.331155	0.442195	0.367002	-0.165176	-0.848487
NVL	3.84541	3.86721	4.00187	4.02671	3.75422	3.67798
NXF1	4.74644	4.62328	4.35854	4.5296	4.632	4.45819
NXN	-4.11163	-5.45651	3.68375	3.98128	3.78884	3.14191
NXPE3	5.15558	5.2168	5.81561	5.98907	6.0303	5.943
NXT1	3.50182	3.71083	2.80517	3.55295	3.20543	3.6398
NXT2	1.44051	1.64048	2.41268	2.77992	2.70383	3.40443
NYAP2	?	-4.87366	2.57804	2.57769	?	-5.69922
OAF	4.42114	4.19357	3.39013	3.73979	0.479334	0.832509
OARD1	2.84782	2.69703	3.1153	3.18119	3.50135	3.46433
OAS1	1.6629	1.68185	2.58793	2.87702	3.67284	3.38604
OAS2	-6.68418	-6.45019	2.94834	2.89118	3.37998	2.83245
OAS3	4.37573	4.61195	4.31931	4.59852	4.71309	4.09903
OASL	-0.816197	-1.07024	0.00643448	0.231137	1.23456	2.21812
OAT	5.3086	5.28652	5.51553	6.182	5.19491	5.57636
OAZ1	6.83681	6.64214	6.81751	7.29585	7.77441	8.24055
OAZ2	4.33914	4.33319	4.60596	4.68558	4.69117	4.87823
OBFC1	1.76038	1.74176	2.15188	2.53143	2.78678	3.44049
OBSCN	2.23738	1.82102	4.21113	4.09293	-2.05768	-2.38405
OBSL1	4.34631	3.83691	4.99534	4.80004	4.31221	4.16911
OCEL1	1.14646	0.831845	0.445928	0.736735	2.09793	2.16367
OCIAD1	5.49444	5.6086	4.91516	5.23323	6.08997	6.42029
OCIAD2	-3.37567	-4.13839	0.871828	1.30061	5.63287	6.65247
OCLN	0.381837	0.350158	1.08215	1.30377	3.18363	2.99683
OCRL	3.7807	3.66257	4.35175	4.45435	4.87692	4.58906
ODC1	5.57003	5.52831	3.72036	3.83661	6.722	6.91096
ODCP	0.567789	0.603321	0.73541	0.266325	0.305574	0.0483059
ODF2	4.7525	4.72685	4.43385	4.75141	4.60457	4.36459
ODF2L	4.39235	4.42436	4.90393	4.91907	4.21159	4.0515
OFD1	2.83527	2.60136	3.13722	3.04324	3.46727	3.23514
OFD1P17	-0.167846	0.247405	1.08824	0.750315	0.522879	-0.11478

OGDH	5.28673	5.16879	5.6694	5.87451	5.59294	5.41156
OGFOD1	4.53674	4.70218	4.4986	5.04196	5.81155	6.16624
OGFOD2	1.58152	1.35717	1.84801	1.68916	1.51053	1.55048
OGFOD3	3.61153	3.52726	3.24065	3.20179	4.09541	4.05483
OGFR	4.5677	4.40134	4.40519	4.66513	4.63488	4.55773
OGFRL1	5.19503	5.44637	4.59235	5.14082	6.22307	5.94817
OGG1	3.36895	3.23575	3.30077	3.38725	3.866	3.69925
OGN	0.980464	1.33162	1.27595	0.968047	-0.0602619	-0.0627204
OGT	6.49016	6.58171	6.98733	7.13297	7.61642	7.41625
OIP5	2.2386	2.31285	1.64565	1.9684	2.0225	2.80913
OIP5-AS1	5.86212	6.00367	5.39701	5.74171	6.13755	6.66857
OLA1	5.64019	5.76535	6.12911	6.59644	6.17289	6.63216
OLA1P1	3.43412	3.68181	3.93899	4.6136	3.94453	4.40511
OLFM1	?	-5.45651	2.98139	3.01788	?	-5.69922
OLFM2	1.69756	1.65093	-2.37543	-2.30078	-1.80184	-2.24661
OLFML2A	4.4462	4.42967	3.20019	3.26838	2.50963	2.41224
OLFML2B	-1.61119	-1.37502	2.93542	3.37498	5.00682	4.69841
OLFML3	1.17133	0.988419	1.99142	1.61913	-2.85193	-3.38329
OLMALINC	2.17438	1.89913	1.19157	0.999755	2.53446	2.02802
OLR1	?	-4.87366	3.48714	3.74603	?	-5.69922
OMA1	3.2287	3.00068	3.23006	3.17658	3.02794	3.45063
OMD	0.693311	0.90354	1.27595	1.02051	-0.136604	-0.619042
OMG	1.95915	1.8318	2.86633	2.40688	2.34533	2.04819
ONECUT2	3.95017	3.52726	3.84438	3.3258	-1.20056	-2.53597
OPA1	5.8495	5.94997	5.87345	6.27838	6.92854	7.20113
OPA3	4.20178	4.30947	3.80089	4.11517	5.28146	5.05981
OPHN1	3.89431	4.16832	2.91451	3.11276	5.48179	5.15203
OPN3	3.62445	3.07018	3.99234	3.78252	3.48443	2.94129
OPRD1	4.7423	5.37163	-2.86058	-2.92969	0.147163	0.0747657
OPTN	3.97157	4.11363	4.35369	4.94102	3.92464	4.42256
OR7E126P	0.460879	0.272489	0.987183	0.383121	-0.705893	-2.15312
OR7E38P	1.92658	1.85193	2.04476	2.23097	2.80115	2.96898
ORAI1	2.88589	2.60056	2.23435	2.3588	2.26452	2.58332
ORAI2	4.83247	4.60017	4.81085	5.04688	5.19428	5.02267
ORAI3	1.64327	1.19057	3.48027	3.41938	2.74267	3.20879
ORAOV1	3.22114	3.232	3.01123	3.28808	2.84827	2.36508
ORC1	4.76446	4.82024	4.4117	4.75769	4.37244	4.09095
ORC2	4.37552	4.48857	3.87025	4.04074	4.9839	5.10207
ORC3	4.54051	4.78564	3.86632	4.18121	4.41248	5.25543
ORC4	4.48429	4.45237	4.11286	4.18082	4.50628	5.13681
ORC5	3.7624	3.7319	3.23854	3.50772	3.62278	4.0178
ORC6	3.68893	3.60593	4.02227	4.23324	4.58048	4.53526
ORMDL1	4.58343	4.52867	4.29625	4.48922	5.7188	5.90475
ORMDL2	3.84037	3.86419	3.64271	4.0007	3.51268	3.97223
ORMDL3	3.79578	3.73805	4.38538	4.53412	4.29679	4.35262
OS9	5.92594	5.93989	6.62262	6.8966	6.15884	6.05358
OSBP	4.52973	4.58496	4.99015	5.30954	4.7607	4.83824
OSBP2	1.6585	2.08407	-2.09546	-1.58485	-1.80184	-3.12051
OSBPL10	6.35568	6.23758	3.0308	3.16963	6.57419	6.19679
OSBPL11	3.49075	3.62727	3.53757	3.77839	3.80729	3.95149
OSBPL1A	3.67135	3.77782	3.59192	3.72872	2.75292	2.52212
OSBPL2	4.86602	4.69071	4.71023	4.94915	4.88897	4.47816

OSBPL3	5.70632	6.02097	4.01246	4.2399	4.78574	4.64533
OSBPL5	2.99615	2.50871	3.25111	3.35674	-1.10542	-1.38442
OSBPL6	3.11951	3.28367	1.5714	1.68562	4.2968	4.23286
OSBPL7	2.28393	2.33483	2.75018	2.84246	2.67732	2.50762
OSBPL8	5.73866	5.79921	5.5896	5.80068	5.63978	5.65795
OSBPL9	5.29626	5.23103	6.15222	6.32197	5.74585	5.70464
OSCP1	0.693311	0.0450795	0.849967	1.32585	0.939663	1.41227
OSER1	4.58978	4.61994	4.45217	4.9773	4.48498	5.10221
OSER1-AS1	0.631914	0.467978	0.893354	1.39106	0.246688	0.33778
OSGEP	3.22143	3.33563	2.35953	2.80011	3.16361	3.10281
OSGEPL1	2.18151	2.21532	1.88213	2.17999	2.64119	3.14321
OSGIN1	0.586404	0.419895	3.73236	4.03963	1.06084	1.1637
OSGIN2	3.92662	4.10321	4.10648	4.69529	4.54365	4.69521
OSMR	5.92235	5.59941	7.61951	7.74073	6.4785	6.16599
OSR1	1.06916	0.635262	1.69303	1.64609	0.282309	-0.95156
OSR2	-1.45077	-0.907998	-0.0748589	0.158078	1.04018	0.847941
OST4	4.57117	4.6639	3.61173	3.99808	5.42184	6.02132
OSTC	4.98869	4.95021	5.43623	5.68112	4.85041	5.72319
OSTF1	1.70183	1.74664	1.86635	2.46104	2.11778	2.78915
OSTM1	4.93893	4.93373	5.13895	5.42253	7.53161	7.99435
OTOGL	-3.11289	-2.76144	-5.1783	-2.78591	2.88273	3.0877
OTUB1	5.0087	4.98681	4.29928	4.74057	4.61696	5.03895
OTUB2	2.50766	2.63108	0.874396	0.969668	1.059	0.767273
OTUD1	1.64525	1.50873	1.08332	1.57069	-2.65941	-2.70579
OTUD3	3.95197	3.85456	3.9889	3.96531	3.88658	3.00065
OTUD4	5.56181	5.6142	5.08375	5.28071	5.41122	5.06622
OTUD4P1	2.08384	2.20765	1.94319	2.12223	1.84426	1.60322
OTUD5	4.16588	4.14263	4.04047	4.48923	3.8798	3.91166
OTUD6B	3.65847	3.73682	3.51344	3.75536	4.38543	4.21958
OTUD6B-AS1	3.16201	3.05286	3.69146	3.8145	3.86521	4.11505
OTUD7B	5.53811	5.44442	4.69605	4.80779	5.16464	4.81095
OTULIN	5.22979	5.21487	5.21253	5.35775	5.35091	5.11106
OVCA2	3.08444	2.92366	3.25655	3.76051	3.856	4.53917
OXA1L	4.67159	4.55673	4.0477	4.26403	5.04098	5.07382
OXCT1	4.27243	4.39901	3.14291	3.57592	4.42448	4.7568
OXLD1	3.55177	3.31044	2.61255	3.00492	3.43888	3.89333
OXNAD1	3.9193	3.96027	3.48003	3.69207	3.82954	4.11506
OXR1	4.53093	4.77276	3.4531	3.79398	4.10206	4.75818
OXSM	2.54115	2.61621	1.61135	2.08102	2.75795	3.60843
OXSR1	5.83205	5.71425	4.64619	5.07852	6.29388	6.17363
OXTR	0.743937	0.90354	-0.325375	-0.0642959	0.847548	0.68568
P2RX4	1.69756	1.692	2.51346	2.81896	1.90577	2.14505
P2RX5-TAX1BP3	0.430039	0.367427	0.483075	0.155827	-0.5694	-0.49705
P2RX6	-1.41331	-1.2146	2.18282	2.48628	-2.572	-2.38405
P2RX7	0.768595	0.736899	1.81086	3.0611	-0.43131	-0.536593
P2RY1	3.01173	3.33481	?	?	1.77017	1.63317
P2RY11	1.48976	1.13154	-0.223722	-0.0972858	1.97841	1.29276
P3H1	5.74359	5.33845	6.58922	6.73526	5.01786	4.50517
P3H2	4.88728	4.77376	6.60068	6.81411	5.43274	4.66723
P3H3	4.78021	4.78387	4.17182	4.45055	4.21236	4.63868
P3H4	4.78273	4.59307	4.35175	4.46954	3.4298	3.59224
P4HA1	5.49137	5.33968	5.66706	5.96599	6.80898	6.49661

P4HA2	5.22387	5.26845	5.86427	6.11912	6.07573	5.38794
P4HB	9.04242	8.89895	8.64582	8.94931	8.83336	8.72403
P4HTM	3.62036	3.36345	3.07622	3.32126	3.64689	3.36681
PA2G4	6.79852	6.81936	5.27919	5.69875	6.24642	6.32577
PA2G4P1	2.00587	1.86289	0.76612	0.633682	1.28125	1.17556
PA2G4P2	2.24182	2.38315	0.755409	1.23445	1.72246	1.57492
PA2G4P4	4.46842	4.61054	2.98361	3.26232	3.69072	3.81041
PAAF1	3.15423	3.15003	3.17623	3.47237	2.77433	2.68561
PABPC1	7.2029	6.92348	7.46325	7.31049	8.04698	7.87008
PABPC1L	3.457	3.51872	3.42565	3.37298	3.38272	2.47321
PABPC1P3	-0.419098	-0.474893	0.346543	0.132208	0.502351	0.0136695
PABPC1P4	1.94995	1.65755	2.19254	1.9712	2.76623	2.37091
PABPC3	3.42394	3.20796	3.75993	3.62563	4.17913	3.99164
PABPC4	5.95025	5.80152	4.973	5.0314	5.92614	5.61256
PABPN1	5.06402	4.78989	4.1398	4.22811	5.49515	5.13752
PACRGL	2.35622	2.53998	1.83887	2.02562	2.77849	3.14816
PACS1	3.74696	3.62594	3.88525	3.83368	3.19612	3.25619
PACS2	3.75213	3.50581	2.5995	2.49481	3.22843	3.08771
PACSIN2	5.09822	5.00987	5.26666	5.62798	4.99747	4.66614
PACSIN3	3.92846	3.70582	3.03807	3.4049	3.52952	3.35668
PADI3	-2.9975	-3.29152	1.05494	1.74918	0.983605	3.55068
PAF1	4.50609	4.51586	4.21308	4.54491	5.21312	4.80897
PAFAH1B1	5.98678	6.07068	6.29763	6.56195	6.93643	7.01862
PAFAH1B2	5.99634	6.53535	5.803	6.66369	6.30601	6.84089
PAFAH1B3	2.71002	2.67867	2.28086	2.62178	3.25885	3.83555
PAFAH2	3.15268	3.12962	2.72338	3.17426	3.50334	3.8188
PAG1	3.34531	3.55675	4.29221	4.60111	4.1694	3.76089
PAGE2	3.92296	3.80641	-6.17309	-6.09892	?	?
PAGE5	4.04639	3.98792	-3.59683	-2.40763	-6.64581	-5.69922
PAGR1	4.00467	3.92399	3.41263	3.5496	3.46239	3.42911
PAICS	6.95471	7.00175	6.37316	6.76832	9.03045	9.23098
PAICSP4	2.80367	3.12075	2.40598	2.80646	4.84032	4.93585
PAIP1	3.77374	3.78624	3.76866	4.05731	4.39462	4.76701
PAIP2	5.23647	5.1426	5.27585	5.53862	5.82683	6.21549
PAK1	4.84857	4.96547	3.9367	4.22656	3.97509	4.1969
PAK1IP1	3.84588	3.86919	2.80517	3.25531	4.82802	5.31495
PAK2	5.32479	5.20944	5.53585	5.73229	6.10417	6.06265
PAK3	-0.223192	0.076436	-0.825729	-0.353769	3.62971	4.50273
PAK4	2.0823	2.041	2.693	2.97599	3.06931	2.96572
PALB2	3.89056	4.01086	3.9914	4.45531	3.53568	3.6856
PALLD	4.62236	4.58334	5.89094	6.05647	6.44525	6.59549
PALM2	2.06204	2.49567	1.05938	0.385674	0.746356	-0.0369639
PALM2-AKAP2	4.23388	3.94279	1.83921	2.86413	3.24786	2.91817
PALM3	0.399051	0.592522	0.849967	0.935626	-2.75246	-2.70579
PAM	6.97478	7.10832	6.34028	6.35853	6.92828	6.72765
PAM16	1.28963	1.38268	1.14718	1.63263	1.37315	2.43506
PAMR1	-5.69162	-6.45019	4.48713	4.53952	-3.19956	-4.70296
PAN2	3.7114	3.17921	4.36722	4.06735	3.24207	2.53647
PAN3	4.2988	4.15278	4.38252	4.25858	4.04358	3.48064
PANDAR	0.449862	0.693712	0.762221	1.39889	1.67671	1.75313
PANK1	2.46332	2.39204	1.95937	2.06609	2.57572	2.5173
PANK2	4.73764	4.65305	5.20233	5.40341	4.43311	4.42315

PANK3	6.5242	6.79669	6.43389	6.8864	7.41718	7.56818
PANK4	2.58864	2.40753	2.77068	2.94375	3.16465	2.92629
PANX1	4.26956	4.3453	4.63506	4.96733	4.54788	4.80996
PANX2	-1.45077	-1.46246	1.06446	0.845438	0.86332	0.878317
PAPD4	4.35144	4.53574	5.0926	5.20234	5.30185	5.38405
PAPD5	3.58747	3.82718	3.88121	4.01658	4.76226	4.7164
PAPD7	4.78322	4.63912	4.76776	4.661	4.825	4.11506
PAPOLA	6.53871	6.4758	5.91549	6.04567	6.66622	6.51094
PAPOLG	3.66176	3.77437	3.48358	3.51689	3.51086	3.6107
PAPPA	5.79913	5.84633	2.96999	2.78761	5.81177	5.55421
PAPSS1	4.97909	5.06071	5.00196	5.27541	4.78418	4.74552
PAPSS2	4.38242	3.71333	4.56719	5.1444	3.15668	3.27056
PAQR3	4.03802	4.68742	3.9053	4.20901	3.85846	4.59104
PAQR4	4.00287	3.88132	3.23576	3.44709	2.76035	2.60516
PAQR5	1.59558	1.58665	-0.240653	-0.381793	-1.15445	-1.18284
PAQR7	3.91053	3.91291	2.97926	3.16734	3.4092	3.18194
PAQR8	4.18125	4.36606	4.04288	4.42935	4.01564	4.24456
PARD3	5.42503	5.37792	2.01126	1.98923	4.88415	4.54121
PARD3B	2.35078	2.45603	2.49949	2.28779	2.95991	2.77711
PARD6A	-0.159773	-0.27286	-3.37468	-2.10822	2.1726	2.49297
PARD6B	4.14326	3.79454	4.82349	4.94033	3.75332	3.10386
PARD6G	3.30515	3.23085	1.1304	0.893937	0.282309	-0.283387
PARD6G-AS1	1.33439	1.614	0.371169	0.19507	-0.530833	-0.619042
PARG	4.60194	4.58561	4.08801	4.20559	4.80327	4.7384
PARGP1	2.79246	2.71424	2.55945	2.22999	2.68442	2.75611
PARK7	5.60869	5.57435	5.19369	5.54938	6.0906	6.61548
PARL	3.55893	3.56641	3.84852	4.25082	4.44634	4.9342
PARM1	4.05581	3.48262	1.93024	0.761609	-5.07051	-5.69922
PARN	4.18049	4.04097	3.97002	4.15209	3.84847	3.92081
PARP1	6.98231	6.90914	6.53232	6.77741	6.77624	6.68332
PARP10	1.50553	1.35098	3.55118	3.65604	3.30403	3.38605
PARP11	1.08233	0.746683	1.73537	1.69856	-1.75295	-2.70579
PARP12	3.6943	3.88809	3.84714	4.09696	4.30978	4.31287
PARP14	5.15889	5.37278	7.10757	7.09527	8.14812	8.21465
PARP16	1.47089	1.23069	2.02107	2.17196	2.26155	2.16367
PARP1P1	2.38381	2.47869	1.64004	1.89916	2.07271	1.46813
PARP2	3.85647	3.87254	2.75312	3.01788	3.71146	3.42514
PARP3	2.51282	2.38892	2.84162	3.21536	1.60651	2.08121
PARP4	5.64939	5.94446	5.8357	6.22155	5.41783	5.80601
PARP6	4.25438	4.20869	4.53928	4.59593	4.54603	4.08607
PARP8	3.69674	3.73437	3.28612	3.19724	2.47208	2.20019
PARP9	2.79459	2.97294	3.93927	4.31531	5.96178	6.3092
PARPBP	4.7811	4.87641	3.57396	3.78778	4.01568	4.49174
PARS2	1.80874	2.02903	2.11571	2.59509	2.25259	2.33222
PART1	-0.228209	-0.0918512	0.780612	1.08275	-1.59203	-1.4238
PARVA	4.1211	3.96969	5.13162	5.32057	4.12922	4.19113
PARVB	1.68897	1.63505	2.96114	3.45913	-2.95876	-2.38405
PASK	4.09259	3.79296	3.63349	3.58148	3.14621	2.59737
PATL1	3.53334	3.51586	3.76776	3.9586	3.33917	3.21215
PATZ1	4.1589	4.05875	3.00383	3.16264	3.26895	3.46822
PAWR	4.81377	5.03749	4.59125	4.78977	5.56239	5.36541
PAX3	-0.917461	-1.37502	2.27592	2.40294	-3.6585	-2.70579

PAX6	-0.69886	-0.629746	1.45314	1.39901	2.47925	2.18812
PAX8	3.37608	3.77562	0.163035	1.10341	1.33768	2.13214
PAX8-AS1	5.45369	6.06234	0.42219	1.60983	0.664201	1.43766
PAX9	-0.97084	-1.20847	0.888204	0.273098	0.979616	0.483674
PAXBP1	5.14326	5.028	5.77791	5.69489	4.60647	4.30031
PAXBP1-AS1	1.20183	1.00482	2.1019	1.95189	0.479334	0.100762
PAXIP1	4.81979	4.79368	4.25994	4.29196	3.85218	3.23536
PAXIP1-AS1	1.76038	1.79928	1.5847	1.77384	0.990802	0.863209
PAXIP1-AS2	1.6956	1.71937	2.18059	2.2225	1.39101	1.12815
PBDC1	2.90083	2.93804	1.99142	2.53864	3.41382	3.54122
PBK	5.33846	5.3994	4.56801	4.849	4.5673	4.93353
PBLD	0.195852	-0.0533185	1.88259	1.59857	1.8711	1.38079
PBRM1	6.20535	6.26519	5.64634	5.78933	6.27805	6.18079
PBX1	2.9138	2.65091	3.76485	3.33412	6.14198	5.90337
PBX2	4.34237	3.94063	3.99112	3.85707	4.3407	3.71441
PBX2P1	1.62867	1.57919	1.46197	1.2304	1.64145	1.05415
PBX3	3.25802	3.3998	1.98141	2.13437	3.38818	3.60163
PBXIP1	5.17092	4.66385	5.34538	5.46768	5.5215	5.63254
PC	4.33168	4.32759	4.30268	4.78223	3.0344	3.20168
PCBD1	1.92665	2.23066	1.77651	2.39898	3.32268	3.45568
PCBD2	2.36879	2.6728	2.76067	2.86795	2.3319	2.22285
PCBP1	5.24122	5.11919	4.46127	4.5359	5.25529	5.1964
PCBP1-AS1	2.97294	2.75448	2.69896	2.41871	2.55568	2.0483
PCBP2	6.394	6.23736	6.29063	6.2137	5.95272	5.83624
PCBP3	-1.52875	-1.93875	1.98643	2.33827	?	?
PCBP3-OT1	-1.8406	-2.76144	1.61741	1.65276	-6.64581	?
PCBP4	4.64576	4.2254	3.0308	3.21085	3.60881	3.60389
PCCA	2.30153	2.24672	3.5089	3.48178	2.42678	2.57321
PCCB	3.73049	3.69185	3.0917	3.53043	3.54472	3.83326
PCDH1	5.72247	4.83865	0.225978	1.12005	-3.33694	-2.53597
PCDH10	3.16201	3.04495	-0.161006	-0.585061	4.5295	4.00409
PCDH17	3.6172	3.43349	?	?	2.60179	2.68131
PCDH18	1.67164	1.70711	7.04943	6.36465	3.59235	3.84788
PCDH20	-1.41331	-2.37465	0.120378	0.902467	5.44133	6.60417
PCDH7	2.39633	2.59245	-6.17309	-3.78492	-0.660096	-0.247123
PCDH9	5.68566	5.37239	-1.37581	-1.64896	5.5793	5.80007
PCDHA10	3.33907	3.2967	-4.29892	-3.52234	-3.63244	?
PCDHA11	2.17753	2.35188	-5.1782	-6.08452	-5.65295	-4.39837
PCDHA2	2.12548	1.86501	-4.05396	-3.51044	?	-13.2868
PCDHA3	3.87141	3.73113	-2.91128	-4.52056	-4.33541	-4.69257
PCDHA4	3.30685	3.24344	-3.37468	-3.73046	-3.85089	-4.68098
PCDHA5	2.52977	2.40842	-5.1783	-3.88131	-6.1458	?
PCDHA6	3.24508	3.15347	-3.85951	-6.09883	-5.07051	-4.69757
PCDHA7	3.14545	2.87718	-5.84661	-5.59246	-3.51959	-4.69678
PCDHAC1	2.56396	2.41648	-5.58868	-4.10635	-5.08692	?
PCDHAC2	2.33799	2.43409	-3.47952	-2.93671	?	?
PCDHB10	1.91381	1.91447	0.722536	0.288359	0.248814	-0.0933326
PCDHB11	1.84535	1.70315	0.597917	0.335758	-2.07475	-2.70579
PCDHB12	2.47982	2.36589	0.893354	0.430424	-3.15591	-4.11926
PCDHB13	2.09323	1.86743	1.29534	0.799145	-3.20887	-3.86617
PCDHB14	1.44953	1.4024	2.55303	2.14901	1.56854	0.978518
PCDHB15	1.80505	2.07603	0.238946	-0.383375	-2.41163	-2.74991

PCDHB16	4.07403	3.81103	2.98269	2.25817	-0.0637725	-0.982845
PCDHB17P	2.21002	1.98911	-1.91719	-2.93571 ?	?	
PCDHB2	3.96062	3.82964	1.84539	1.01662	-0.34409	-1.16796
PCDHB5	4.35565	3.96456	-3.85951	-2.64851	-5.61326	-4.64778
PCDHB6	3.25334	3.34251	-4.17617	-4.10635	-5.64523	-5.69922
PCDHB7	2.97426	2.64596	-0.757028	-1.09739	-0.905167	-0.899103
PCDHB8	4.08672	3.95603	-0.653591	-1.58116	-4.53431	-5.19176
PCDHB9	2.05898	2.03798	1.15374	0.73508	-0.273545	-0.832306
PCDHGA1	0.726212	0.159048	0.232771	-0.336116	0.636924	-0.0482019
PCDHGA10	2.59249	2.48314	2.64653	2.21425	1.12611	0.27978
PCDHGA11	0.836215	0.81619	0.817394	0.578533	0.387914	-0.725172
PCDHGA4	1.26354	0.982284	0.983649	0.929632	1.38286	0.34276
PCDHGA5	1.2093	0.436897	1.10883	0.585174	1.55757	0.545148
PCDHGA6	1.51095	1.29602	1.98902	1.51905	2.20774	1.15867
PCDHGA8	0.452627	0.72396	0.446584	-0.0945798	0.894457	-0.0101634
PCDHGA9	0.667765	0.493145	2.68674	2.49812	1.87896	1.09828
PCDHGB1	0.844611	0.525748	0.731661	-0.0818581	1.8729	0.672652
PCDHGB2	2.76618	2.829	2.19585	2.04029	2.85066	2.74209
PCDHGB3	1.37151	0.987066	1.76438	1.0127	1.92409	1.00398
PCDHGB4	0.593354	0.108625	0.239105	-0.0954319	0.849991	-0.019824
PCDHGB6	0.75628	0.715507	0.713678	0.417607	0.749208	-0.206424
PCDHGB7	2.65743	2.59661	1.6592	1.28751	0.836457	0.235703
PCDHGC3	6.9489	6.59184	6.40114	6.28523	6.25214	5.94788
PCDHGC4	2.46364	1.81024	1.62681	1.16356	1.2387	0.362734
PCDHGC5	2.38316	1.57768	1.64771	1.13205	1.69439	0.37709
PCED1A	2.85935	2.50583	3.75017	3.76615	1.79093	1.46325
PCED1B	-0.591601	-0.622086	-0.254699	0.0321291	0.664192	0.662515
PCF11	5.27498	5.18394	5.49287	5.57498	5.28887	5.18462
PCGF1	2.36163	2.30535	1.83331	2.29584	2.26748	2.45317
PCGF2	2.51118	2.37236	2.28502	2.02601	2.01167	1.84661
PCGF3	5.00091	4.78024	5.23063	5.2673	5.85042	5.17056
PCGF5	4.77715	4.72265	4.09143	4.63179	5.37004	5.23283
PCGF6	2.5636	2.51482	2.28305	2.51108	2.79131	2.82456
PCGF7P	0.396466	-0.0386523	-0.270293	0.310631	0.244351	0.294018
PCID2	4.52203	4.41673	4.66234	4.95834	4.52918	4.32636
PCIF1	3.26815	3.11647	2.89063	2.98921	2.88273	3.09742
PCK2	2.19878	2.30535	2.5273	3.01271	2.04015	2.18205
PCLO	4.68514	4.75329	-0.300715	-0.154483	-5.65305	-5.69922
PCM1	6.51642	6.60003	6.42242	6.61781	6.09895	6.25435
PCMT1	4.6462	4.87127	4.90433	5.32191	5.53147	6.01601
PCMTD1	4.61491	4.80745	4.58958	4.73271	3.96173	4.49784
PCMTD2	5.43534	5.49532	4.65173	4.52143	4.97357	4.97803
PCNA	6.61979	6.7247	6.48225	6.8674	6.42916	6.97003
PCNP	5.80111	5.9012	5.52493	5.76559	5.61999	5.51151
PCNPP5	2.03462	2.17158	1.61434	2.01272	1.73886	1.94095
PCNT	5.16203	5.0484	6.28764	6.36581	4.85737	4.50274
PCNX	6.58182	6.43122	6.07945	6.08716	6.38234	6.26911
PCNXL2	4.91069	5.16904	4.88456	5.13916	4.33971	4.20396
PCNXL3	5.1805	4.85342	4.76774	4.64522	4.54059	4.58905
PCNXL4	6.03142	6.10597	5.23542	5.46314	5.4446	5.67584
PCOLCE	6.089	5.74545	5.76889	5.61883	-1.37455	-1.38442
PCOLCE2	1.12115	1.08411	0.416483	0.491176	1.77851	2.56008

PCSK1	0.0425183	-0.681268	1.81086	0.833756	1.17894	2.0681
PCSK5	4.53032	4.98784	0.416483	1.23107	-2.41163	-2.12114
PCSK7	4.99192	5.0131	5.32085	5.45442	5.36389	4.97875
PCTP	3.4532	3.4928	3.6206	3.70174	2.90765	3.2677
PCYOX1	6.10627	6.05085	6.01184	6.19891	6.07271	5.78514
PCYOX1L	3.21081	3.17921	4.02104	4.24872	2.65485	3.04483
PCYT1A	5.21921	5.39782	5.93561	6.27653	6.85967	7.02626
PCYT1B	0.44057	0.71714	0.945825	0.999755	1.55404	0.951558
PCYT2	3.98736	3.64534	4.03902	4.65136	4.79441	5.35772
PDAP1	5.85751	5.76786	4.99147	5.35877	6.34048	6.27473
PDCD10	3.94206	3.93589	4.0926	4.46102	4.59825	5.11426
PDCD11	6.34564	6.37279	5.44081	5.64522	6.65315	6.36507
PDCD1LG2	-0.207073	-0.00328298	0.747284	1.45345	2.32839	3.22107
PDCD2	4.52488	4.64485	3.81635	4.12469	5.74339	6.38991
PDCD2L	1.3937	1.58158	0.623811	1.43037	2.00853	2.72792
PDCD4	3.82568	4.24911	3.87165	4.11288	3.55915	4.17808
PDCD5	4.44706	4.37207	4.03585	4.51479	4.62716	5.06371
PDCD5P1	0.653234	0.824222	0.201069	0.949565	1.05669	1.19953
PDCD6	5.56356	5.53941	5.06039	5.36595	5.96984	5.97874
PDCD6IP	5.99569	6.10278	5.73937	6.02929	6.31119	6.30391
PDCD7	3.42892	3.37479	3.12936	3.36488	3.32553	3.24749
PDCL	3.66176	3.81676	3.36915	3.68071	3.88272	4.18354
PDCL3	2.45726	2.67435	1.56468	1.85095	3.04491	3.59445
PDCL3P4	0.33538	0.556238	0.0892541	0.161797	0.681773	0.847235
PDCL3P5	1.35768	1.27344	-1.61683	-0.35792	2.10789	2.0291
PDDC1	4.32881	4.42743	4.5478	4.65273	4.36619	4.0195
PDE10A	4.00914	4.01996	2.95859	3.06859	-1.90489	-2.53597
PDE12	5.28745	5.48076	4.31033	4.53772	5.87572	5.57346
PDE1C	7.00038	7.43253	-3.37468	-3.52222	8.39538	8.57555
PDE3A	0.0954519	-0.399458	?	?	2.47974	2.55069
PDE4A	1.10833	0.946604	1.662	1.80409	0.732078	0.50283
PDE4B	5.35958	5.51154	-0.375995	-0.899117	4.32552	4.20317
PDE4D	3.49852	3.5765	4.06576	4.10549	4.29968	4.4443
PDE4DIP	4.39446	4.52285	5.51706	5.75488	5.26721	5.9055
PDE5A	2.82767	2.86771	8.8469	8.32153	2.37268	2.30057
PDE6D	2.96448	3.08791	2.86361	3.0686	2.80526	3.48558
PDE7A	3.99446	4.00244	4.76769	4.55367	3.30379	2.70684
PDE7B	0.628074	0.261747	2.14766	2.31971	-3.6585	-4.11926
PDE8A	4.32051	4.3732	4.78729	4.90309	5.63746	5.5542
PDE8B	1.93076	2.07887	2.25451	2.25564	1.2743	1.513
PDF	0.526399	0.218781	0.227445	0.337665	2.27983	2.75153
PDGFA	4.35892	4.34691	3.75017	3.77534	0.847548	1.5971
PDGFC	3.72298	3.98112	4.78081	4.72167	5.334	5.06972
PDGFD	0.510456	1.17556	6.35364	6.21668	3.92663	4.85074
PDGFRA	3.27072	2.87401	5.57584	5.2638	7.05697	6.96184
PDGFRB	1.48087	1.67161	7.55616	7.53303	3.27485	3.06478
PDHA1	3.91448	3.89432	5.00235	5.37741	4.83303	4.81684
PDHB	5.10105	5.14999	4.42382	4.8707	5.32782	5.99632
PDHX	4.39726	4.39235	4.57696	4.9834	5.36199	5.606
PDIA3	7.81446	7.68654	7.52932	7.8234	7.06191	7.18818
PDIA3P1	6.81464	6.81739	6.61978	6.94324	6.13159	6.24266
PDIA4	8.36336	8.34887	8.03411	8.4309	7.69209	7.96945

PDIA5	3.09866	3.12964	2.80233	3.2288	4.22311	4.49418
PDIA6	8.50695	8.33102	7.44158	7.59284	7.96678	7.87917
PDIK1L	3.38838	3.64175	2.57471	2.96264	3.60529	3.80503
PDK1	4.99606	4.77316	5.04649	4.94915	6.59395	5.48182
PDK2	2.33943	2.18922	2.8026	3.0068	2.17757	2.31015
PDK3	1.49571	1.33161	2.9939	3.1391	2.71691	2.65085
PDK4	1.73558	1.69706	-1.13882	-2.20128	?	?
PDLIM1	3.23903	3.06463	-0.973801	-0.275776	3.65919	3.78513
PDLIM1P4	1.83957	1.89895	0.0850983	-0.40821	-2.65941	-2.53597
PDLIM2	2.7738	2.62237	2.97532	3.08897	3.08826	3.62585
PDLIM3	2.96448	2.72945	1.22592	1.66599	2.58049	2.56942
PDLIM4	4.76751	4.82312	-2.48229	-2.52305	3.73629	3.70268
PDLIM5	5.27498	5.40867	4.48536	4.77687	5.94977	5.78512
PDLIM7	2.72929	2.53152	3.02837	3.24211	3.64348	3.87071
PDP1	6.52434	6.03249	5.34297	5.84936	6.24767	5.99762
PDP2	3.75624	3.86246	4.44492	4.68477	4.67953	4.78413
PDPK1	4.56654	4.26094	4.72473	4.78499	5.02523	4.36287
PDPK2P	0.728826	0.86637	1.27043	1.48704	1.16973	0.204078
PDPR	5.88622	5.87821	6.03466	6.23235	7.01728	6.5901
PDRG1	3.41955	3.29662	2.73165	3.06842	2.83351	3.00063
PDS5A	6.75868	6.75961	6.05724	6.25748	7.14673	7.05772
PDS5B	5.54913	5.43612	6.01706	5.89116	5.02048	4.94791
PDSS1	2.48948	2.44366	1.26904	1.6443	2.44604	2.86315
PDSS2	2.23757	2.17742	2.40521	2.63265	3.34672	2.98329
PDXDC1	5.53722	5.40816	5.78504	6.07057	4.49262	4.56666
PDXDC2P	2.94748	2.72125	3.60896	3.63151	3.08582	2.59129
PDXK	4.57472	4.38578	5.472	5.5877	5.05434	4.90707
PDXP	4.1632	4.01949	2.80843	2.85333	4.00191	3.44734
PDZD11	3.51469	3.60593	3.62137	4.1682	4.64324	5.44093
PDZD2	1.53936	1.01295	3.60602	3.73195	4.22615	3.64201
PDZD4	-0.207075	-1.07024	1.76487	1.91357	-3.19956	-3.38329
PDZD8	5.92652	5.95067	5.69945	5.90126	6.37702	6.28272
PDZRN3	2.35335	2.36048	2.61709	3.24337	4.33812	4.4974
PEA15	5.75132	5.71925	4.97948	5.73698	7.53948	7.84796
PEAK1	5.59841	5.47195	5.59126	5.29788	5.96975	5.76395
PEBP1	6.38105	6.25838	5.46694	5.74401	6.11194	6.00796
PEBP1P2	1.50728	1.29339	0.492335	0.904288	1.1364	1.10092
PECR	1.96506	1.92419	2.02546	2.17197	2.42317	2.78543
PEF1	4.81189	4.8054	3.81287	4.31113	4.96631	5.27539
PEG10	7.42028	6.79909	6.7281	6.68623	9.01984	9.09136
PEG3	4.48586	4.47919	-5.1783	-4.52056	?	-3.38329
PELI1	3.67596	3.97073	2.11571	2.15798	3.43113	3.01439
PELI2	4.05213	3.82761	3.9426	3.6208	4.65426	3.8988
PELI3	2.957	2.89965	2.61009	2.69846	1.62449	1.48168
PELO	4.24426	4.38339	4.45457	5.00968	4.32976	4.59017
PELP1	3.75933	3.82717	4.08676	4.23213	3.82048	3.71114
PEMT	1.53259	1.42969	1.07876	0.980545	1.65138	1.86456
PEPD	3.87624	4.04227	3.15582	3.38445	4.18125	4.52551
PER1	2.33711	1.80397	2.02595	1.76772	2.2254	1.80902
PER2	1.8738	1.92463	2.49772	2.5062	3.04016	2.68626
PER3	3.00212	2.84774	2.91732	2.9316	4.28388	3.9085
PERP	5.39723	5.64398	6.3505	6.8399	-5.65305	-5.69922

PES1	5.65461	5.57915	4.68144	5.28829	5.25671	5.10065
PET100	1.63229	1.69412	1.60334	1.85754	2.23854	2.53671
PET117	3.65005	3.47518	2.53106	2.69521	3.00781	3.08605
PEX1	4.0621	4.16791	4.9532	5.07188	3.81592	4.2783
PEX10	2.30731	2.34674	2.25443	2.52928	2.93633	3.56263
PEX11A	2.06912	2.21313	2.75018	2.85984	1.67511	1.66836
PEX11B	3.05079	2.83147	3.06417	3.35616	3.06725	3.20618
PEX12	2.2696	2.06074	1.99641	1.88555	2.60883	2.61523
PEX13	4.24524	4.20672	4.2368	4.24202	4.95909	5.07313
PEX14	2.06139	1.95562	2.04973	2.30862	2.59673	2.55724
PEX16	1.4199	1.46793	1.69915	2.22211	2.07442	2.62421
PEX19	5.00993	5.33914	4.60785	5.35955	4.61344	5.28414
PEX2	3.81267	3.9801	3.99639	4.21872	4.74323	5.65907
PEX26	5.86983	5.82019	4.96803	5.35325	5.65006	5.31142
PEX3	2.63766	2.68123	3.01175	3.45549	3.4234	3.43908
PEX5	4.02628	3.95184	3.67601	4.01141	3.20542	3.26483
PEX5L	0.832372	0.592522	0.474784	0.605504	1.29974	0.597155
PEX6	1.93394	1.65613	3.00135	3.07605	2.2734	2.55539
PEX7	0.45076	0.344613	0.401532	1.0511	1.2881	1.88577
PFAS	5.22867	5.11269	4.69529	4.87629	4.88125	4.5982
PFDN1	4.46565	4.46567	4.46963	4.93272	5.00162	5.2835
PFDN2	4.51886	4.56159	2.77649	3.25749	4.09624	4.18962
PFDN4	4.49499	4.54485	3.56131	3.92321	3.39902	4.65742
PFDN5	5.78676	5.79689	5.43666	5.86772	5.71255	6.76849
PFDN6	4.03788	3.94199	3.21983	3.5089	4.84696	5.09118
PFKFB2	3.65718	3.57817	2.65608	3.00824	3.25881	3.40224
PFKFB3	4.08964	4.14355	4.77864	4.9003	7.52289	6.80948
PFKFB4	4.85666	5.01836	3.14401	3.54254	6.8575	6.14554
PFKL	3.48827	3.29871	5.21147	5.47236	4.56367	4.47071
PFKM	5.2014	5.37322	4.92765	5.52282	5.0397	5.26433
PFKP	5.60983	5.79751	5.70139	6.17831	6.87497	6.35127
PFN1	7.56194	7.47583	7.0945	7.51769	8.48206	8.63368
PFN1P1	3.16335	3.23706	2.76917	3.13228	4.12358	4.24509
PFN1P2	2.44147	2.6904	2.1773	2.24284	2.61511	2.19391
PFN1P9	0.323393	0.060836	-0.325375	0.100749	1.04018	0.702755
PFN2	5.75601	5.86221	6.76176	7.02638	7.36352	7.87475
PGAM1	6.7404	6.59506	7.23649	7.58064	8.49173	8.41232
PGAM4	0.249482	0.0253974	0.8767	1.4248	1.86345	2.06402
PGAM5	5.46301	5.46528	4.11109	4.52416	5.665	5.8193
PGAP1	4.7303	4.76294	4.08326	3.80854	4.71309	4.44049
PGAP2	2.33436	2.22367	1.81086	1.7241	2.30837	2.06149
PGAP3	1.33988	1.2854	2.14741	2.19497	1.98357	2.17595
PGBD1	2.26383	2.35095	2.53759	2.99184	3.0761	3.28481
PGBD2	2.36971	2.35095	1.42385	1.21315	1.18523	1.55071
PGBD4	2.52252	2.50871	1.90396	2.03585	1.99792	1.78517
PGD	5.86957	5.84952	7.70741	8.19873	6.92232	7.00204
PGDP1	1.42009	1.75558	3.40931	3.97141	2.67431	2.32959
PGF	1.399	1.33161 ?		-3.30006	3.33687	3.48558
PGGT1B	4.50901	4.64066	4.69575	4.85296	4.95204	5.16426
PGK1	7.80055	7.6937	8.28993	8.67402	10.0298	9.40979
PGK1P2	2.34544	2.30189	2.93505	3.44027	4.6983	3.86745
PGLS	2.50983	2.14774	2.64938	2.70797	2.72124	3.12607

PGM1	5.04533	4.91599	5.40139	5.88724	5.43744	5.16516
PGM2	3.3562	3.70456	3.53757	4.21084	5.52363	5.75578
PGM2L1	3.47335	3.33158	2.68377	2.94103	7.75909	7.64435
PGM3	5.53091	5.77498	4.49266	4.97348	5.39737	5.81729
PGM5P2	2.167	1.99781	-0.236079	-0.290822	5.20843	4.49679
PGP	4.00135	4.036	3.49771	3.87985	3.54549	3.59935
PGPEP1	3.00483	2.82948	4.26149	4.66595	5.33685	5.08688
PGRMC1	3.4392	3.74294	5.87446	6.26192	6.97783	7.32674
PGRMC2	4.03393	4.02298	4.51951	4.53772	4.83902	4.78011
PGS1	4.31074	4.20692	4.32624	4.4515	4.441	4.13877
PHACTR1	3.16639	2.66939	0.961623	1.17071	0.173972	-0.151434
PHACTR2	4.31144	4.19715	5.11339	5.35008	4.73789	4.4811
PHACTR2P1	1.80874	1.72206	-0.69145	-0.682344	0.670686	-1.006
PHACTR4	4.19042	4.38185	3.72185	4.07232	4.69447	4.69416
PHAX	4.8957	5.15048	4.58877	5.20064	5.51774	5.91579
PHB	6.46563	6.37768	5.51987	6.03475	6.11697	6.61032
PHB2	6.70863	6.56994	5.66035	5.98684	6.50073	6.77704
PHBP11	0.0387312	0.316644	-0.392857	-0.0509911	0.15056	0.148205
PHBP21	1.13567	1.13149	0.631328	0.935075	1.09807	1.25511
PHBP9	0.739088	0.598746	0.189034	0.53827	0.367785	0.272716
PHC1	3.09667	2.85578	2.90495	2.9118	2.91952	2.80908
PHC1P1	2.77649	2.59737	2.95794	2.82772	2.87007	2.54244
PHC2	5.29522	5.21009	4.7786	4.9264	5.49756	5.45302
PHC3	5.45256	5.38262	6.6828	6.68089	6.13427	5.66886
PHEX	1.80477	2.51445	-0.482896	1.24877	2.12761	2.5925
PHF1	3.17437	2.94655	3.5444	3.68559	4.071	3.74655
PHF10	4.42442	4.96993	3.93087	4.20041	4.76691	4.60119
PHF11	1.56305	1.78985	2.03567	2.04601	2.49705	2.58792
PHF12	3.9694	3.80793	4.59135	4.60026	4.42504	4.0805
PHF13	3.91378	3.79807	4.13502	4.02688	4.12922	3.80108
PHF14	5.30303	5.26208	5.49286	5.34083	5.1832	5.19792
PHF19	5.44205	5.40133	4.15186	4.64103	5.30978	5.23065
PHF2	3.46205	3.29704	3.44948	3.50404	3.44035	2.95151
PHF20	5.60154	5.48843	5.26252	5.35418	4.96539	4.67806
PHF20L1	5.29731	5.25649	5.94821	6.0223	6.10238	5.90078
PHF21A	3.52613	3.24106	3.9889	3.88269	4.31983	3.8726
PHF23	4.79676	4.79513	4.39579	4.77687	5.48847	5.81879
PHF3	6.91108	7.04663	6.03986	6.2654	6.37428	6.63256
PHF5A	3.98829	3.95606	3.65252	4.24872	4.13489	5.02372
PHF6	4.45763	4.39358	5.26976	5.43952	5.76362	5.53049
PHF7	0.323393	-0.122853	0.208904	0.0811214	0.643551	0.0213376
PHF8	3.8971	3.71707	2.61415	2.74916	3.15987	2.85554
PHGDH	5.00265	5.33886	4.925	5.71375	5.85783	6.03387
PHIP	7.15541	7.16008	6.05638	6.05339	6.81008	6.59984
PHKA1	3.84692	3.90306	2.05588	2.07663	3.83861	3.50386
PHKA1P1	1.53892	1.4037	0.116644	0.117934	1.59204	0.987408
PHKA2	2.82676	2.50465	3.64775	3.60103	2.91017	2.44812
PHKB	3.71156	3.80159	5.46526	5.52786	5.91592	5.93578
PHKG1	0.35156	0.351413	0.46925	0.825061	0.182985	-0.921907
PHKG2	3.55714	3.44856	3.93957	4.24054	3.64777	3.3282
PHLDA1	7.58949	7.41869	7.26234	7.50696	6.2367	6.0104
PHLDA2	1.42508	1.15925	0.64945	0.946514	0.469063	0.847941

PHLDA3	4.08392	4.19357	2.30433	2.52418	2.55644	2.95863
PHLDB1	5.21693	5.22549	4.90472	5.02676	5.20505	4.79758
PHLDB2	4.56359	4.946	5.57218	5.5877	4.00679	3.9135
PHLDB3	0.207918	0.195474	0.208904	0.0410467	1.05399	0.68568
PHLPP1	4.12029	3.82371	1.97132	1.61913	3.6862	3.48311
PHLPP2	4.67347	4.29238	5.13379	5.10371	6.32891	5.30144
PHOSPHO2	0.760783	0.480917	-0.163022	0.392372	0.249178	0.970684
PHPT1	4.19875	3.90237	2.81649	3.01788	3.53321	4.20467
PHRF1	4.84974	4.60054	4.76629	4.88126	4.26228	4.05484
PHTF1	4.75316	4.7821	5.24953	5.3106	4.96219	4.97011
PHTF2	4.94206	4.7362	5.85023	5.71333	5.38885	5.08118
PHYH	1.56774	1.54842	1.20023	1.71775	0.807363	1.24753
PHYKPL	3.30463	3.2657	4.43853	4.46077	4.10385	3.78359
PI15	0.885949	-0.354151	5.81612	3.69045	6.70696	6.86787
PI4K2A	4.34103	4.00666	4.02979	4.2536	4.1764	3.47046
PI4K2B	3.95734	4.10035	3.09708	3.58117	4.39565	4.86882
PI4KA	5.57828	5.53664	5.23753	5.54278	4.79637	4.80129
PI4KAP1	3.12356	2.8243	2.41342	2.16891	1.42838	0.227692
PI4KAP2	3.2941	3.14018	3.32872	3.27366	2.35686	1.44
PI4KB	5.62499	5.68998	5.0635	5.51416	5.98899	6.10603
PIAS1	4.63301	4.55879	5.14332	5.23137	4.66043	4.43577
PIAS2	5.9339	5.99657	4.59125	4.56976	4.23223	3.58791
PIAS3	3.52813	3.45175	3.70386	3.8426	3.57662	3.52012
PIAS4	2.49569	2.37637	2.01126	2.27489	3.52332	3.20318
PIBF1	3.70286	3.87143	3.60927	3.66761	2.68621	3.48804
PICALM	5.51646	5.52833	6.34098	6.54065	6.19085	6.30205
PICK1	1.15272	0.868134	1.12031	1.17663	2.98535	2.84396
PID1	0.409538	0.804012	-1.72363	-0.493928	1.41119	2.11348
PIDD1	2.30375	1.59788	2.40895	2.2041	1.46387	1.33774
PIEZO1	5.82258	5.57356	5.57272	5.59473	4.88348	4.80086
PIEZO2	-5.10914	-4.87366	1.5643	1.76772	3.57844	3.29011
PIF1	2.98216	2.9423	3.02837	3.23547	2.90194	2.34315
PIGA	2.72929	2.67415	3.01369	3.37701	3.3516	3.34041
PIGB	2.40297	2.42283	2.4326	2.6784	3.42848	4.15497
PIGBOS1	1.51694	1.60037	0.942601	1.20142	1.5356	1.75777
PIGC	4.51097	4.46713	3.32229	3.60884	4.23904	4.03811
PIGCP1	1.77435	2.05927	0.478288	0.850191	1.30953	1.32861
PIGF	3.52638	3.49326	3.19089	3.27934	3.4066	4.0197
PIGG	4.83229	4.95553	4.67056	4.89749	5.50961	5.63701
PIGH	2.99076	3.08235	1.82053	2.42897	2.04196	2.78044
PIGK	5.27894	5.43159	5.34442	5.84173	5.34071	5.97931
PIGL	1.9939	1.9883	3.03003	3.00574	2.82819	2.5173
PIGM	3.88869	3.81909	3.48182	3.27488	3.58286	3.15748
PIGN	4.81465	4.85852	4.23959	4.72325	3.25707	3.69629
PIGO	4.98347	4.79512	4.01983	4.19609	5.27447	5.16594
PIGP	1.82451	1.72206	2.77649	2.8968	1.45867	2.00067
PIGQ	2.94658	2.60863	3.59455	3.68827	2.56667	2.80505
PIGS	4.7371	4.71393	5.05964	5.37245	5.24881	5.17974
PIGT	6.33394	6.32035	5.62301	5.9532	5.76227	5.7414
PIGU	4.41689	4.28211	3.93301	3.95383	3.89253	3.78269
PIGUP1	1.16849	1.16529	0.881007	0.816649	0.772823	0.275722
PIGV	3.29244	3.20249	3.10881	3.28564	2.67509	2.91168

PIGW	3.8107	3.84129	3.12842	3.41035	4.14915	4.11322
PIGX	3.0534	3.18668	4.08027	4.39895	3.49011	3.72022
PIGZ	0.577131	0.581631	1.16526	1.17663	-0.878698	-1.006
PIH1D1	4.68413	4.55951	3.74277	3.98392	5.64772	5.52989
PIK3C2A	6.10545	6.0862	6.08968	6.18119	6.06865	6.0398
PIK3C2B	1.51528	1.46201	3.26769	3.28992	0.878914	0.769095
PIK3C3	5.75186	5.75662	4.22904	4.24762	4.73576	4.77205
PIK3CA	4.50139	4.68268	5.21104	5.58034	5.54754	6.10368
PIK3CB	3.92388	3.79572	3.63665	3.79956	3.74483	3.99719
PIK3CD	3.60698	3.52015	2.29306	2.75537	3.74424	3.37009
PIK3CG	1.35081	1.72701	-2.48229	-4.10635	0.147163	0.50283
PIK3R1	4.71823	4.72078	4.65646	4.6758	6.29605	6.10383
PIK3R2	2.74199	2.11777	2.4709	1.91492	4.21501	3.5074
PIK3R3	4.11069	3.62595	5.15019	5.3169	4.75805	4.5982
PIK3R4	5.03223	5.08886	4.90592	5.13909	4.87835	4.89319
PIKFYVE	5.76391	5.72914	5.58796	5.74485	5.88585	5.66832
PILRB	4.78305	4.52621	4.06354	4.16086	4.15679	3.25713
PIM1	2.56303	2.66387	3.18498	3.28349	3.13736	3.05816
PIM2	2.87457	3.23586	1.74132	2.61228	2.46641	2.80505
PIM3	4.00135	3.77913	4.1384	4.36182	2.82955	3.30452
PIN1	3.47584	3.31359	3.19586	3.57942	4.35858	4.60047
PIN4	3.08559	3.07573	3.32665	3.54225	3.28679	3.60628
PIN4P1	0.839935	0.816166	0.943756	1.51317	1.12681	0.877634
PINK1	3.17575	3.10401	4.47086	5.02827	3.3044	3.34392
PINK1-AS	2.47504	2.33648	3.66084	3.6066	2.56144	1.74798
PINX1	2.45268	2.40807	1.89332	2.34652	1.60651	2.10066
PIP4K2A	3.36969	3.57333	3.30231	3.76769	4.88078	4.98416
PIP4K2B	5.80549	5.62261	5.5863	5.65603	5.86447	5.41675
PIP4K2C	2.77868	2.91654	3.26975	3.65272	4.24809	4.3363
PIP5K1A	6.11007	6.13225	5.40108	5.72753	5.93594	5.83762
PIP5K1C	2.68463	2.23761	2.67136	2.58118	3.04013	2.908
PIPOX	-2.69815	-2.76144	-0.787939	-0.463167	3.5762	4.27199
PIPSL	4.32747	4.55099	3.98777	4.4013	4.44891	4.62564
PIR	2.82448	3.07438	4.99949	5.21701	3.49451	3.98503
PISD	4.31841	4.15094	4.24901	4.33098	3.96448	3.89876
PITHD1	3.95465	4.11269	3.28001	3.96397	5.0059	5.16287
PITPNA	4.91604	4.86288	4.91749	5.0649	5.58434	5.40958
PITPNA-AS1	0.441441	0.184623	-0.265573	-0.652282	1.53938	1.91593
PITPNB	5.11804	5.2192	4.76213	4.97714	4.60676	4.75499
PITPNC1	3.91654	3.48698	1.15638	0.82198	1.79915	1.06153
PITPNM1	2.93028	2.52016	3.47468	3.85696	3.28221	3.56474
PITPNM2	3.94747	3.63782	2.81648	3.00752	2.99254	2.78915
PITPNM3	-2.23844	-2.2135	0.358711	0.370611	2.43035	2.93115
PITRM1	4.82925	4.76112	4.64947	5.18203	4.87152	4.81186
PITRM1-AS1	0.88027	0.84145	1.199	1.1056	1.20178	0.614729
PITX1	0.345419	-0.292756	3.36578	3.22474	1.67064	1.71117
PITX2	-1.74441	-2.06994	1.48894	1.59165	2.07442	2.06149
PJA1	3.09046	3.2887	3.89329	4.31428	3.17889	3.23581
PJA2	6.07963	6.09607	6.0853	6.29713	7.12616	7.14287
PKD1	3.28244	2.62864	4.60364	4.51037	4.53143	4.11084
PKD1P1	-0.579698	-0.180649	1.35014	1.97176	0.673068	1.10064
PKD1P6	1.10593	0.845727	2.39355	2.43316	1.19974	0.738353

PKD2	4.49996	4.518	5.29523	5.42546	4.73629	4.74654
PKDCC	2.76241	1.68185	1.79379	1.46107	-3.85089	-3.12051
PKI55	3.5429	3.79572	2.89597	3.17195	3.15347	3.46322
PKIA	1.77668	2.09942	-3.18222	-2.93781	2.93775	3.57638
PKIG	3.18966	3.02299	3.2045	3.44	2.61815	2.92991
PKM	9.77904	9.76454	10.2896	10.9265	10.9734	11.0464
PKMP1	3.9439	4.1848	4.61312	5.25804	5.34451	5.07415
PKMP4	0.985945	1.49719	1.98643	2.65273	2.41117	2.31023
PKMP5	2.08566	2.33827	2.75024	3.46493	3.52462	3.09107
PKMYT1	3.31642	3.08041	2.98245	2.97761	1.96474	1.87817
PKN1	3.70393	3.51014	4.21521	4.6301	4.53381	4.69096
PKN2	5.80996	5.85202	5.82385	5.94306	5.88009	5.90477
PKN3	2.45828	1.93806	1.34008	1.42259	1.04711	0.633217
PKNOX1	2.79878	2.77795	3.13615	3.51139	2.60414	2.88575
PKNOX2	2.51038	1.88591	-4.59508	-3.10759	-5.65305	-5.69922
PKP2	-5.69162 ?		0.045429	0.280659	2.71066	2.50275
PKP3	1.73558	1.38894	-0.659745	0.020584	-1.95932	-1.89883
PKP4	5.86044	5.70449	4.85694	4.94593	5.95406	5.55484
PLA2G12A	3.5752	3.8654	3.67579	4.00516	3.77493	4.35562
PLA2G12AP1	-0.182033	0.200755	0.373286	0.31436	0.483457	1.00453
PLA2G15	4.09452	3.48698	4.27487	4.01141	4.9997	4.52809
PLA2G16	3.17436	3.07049	1.43123	2.19497	3.14221	3.48064
PLA2G4A	3.5967	3.71332	3.8595	4.40489	2.88079	3.75066
PLA2G6	1.56189	1.58519	1.16171	0.789988	0.825354	0.456028
PLAA	4.51338	4.77371	4.47465	4.87339	5.91156	6.15392
PLAC4	4.26525	4.52937	3.94833	3.53142	1.94705	1.55071
PLAG1	3.52973	3.37004	1.94578	1.80409	1.56854	0.97985
PLAGL1	-4.11163	-5.45651	-6.17309	-5.10387	2.83155	1.20023
PLAGL2	3.92141	3.9755	3.77327	3.89095	3.25536	3.19416
PLAT	7.97865	8.22657	2.93802	2.77687	6.677	6.02077
PLAU	-2.46283	-2.00285	6.28794	7.29456	7.00792	7.47526
PLAUR	4.03902	4.16651	2.65569	3.90798	4.02619	4.0058
PLBD1	4.99951	4.92824	-3.18222	-3.52222	-1.33803	-1.61882
PLBD2	3.85454	3.51729	4.26665	4.22544	3.07101	3.14816
PLCB1	3.81219	4.08886	-0.229171	-0.108685	6.18085	5.81537
PLCB3	4.36564	4.25655	3.90393	4.08099	3.77537	3.55773
PLCB4	4.17128	4.04593	4.27384	3.59507	-0.267833	-1.31405
PLCD1	1.27825	0.920918	1.88259	1.85699	1.52955	1.52214
PLCD3	2.82645	2.56784	3.01124	3.27488	3.42184	3.46822
PLCD4	1.2353	1.06379	2.14936	2.42547	3.44245	3.40013
PLCE1	5.18861	5.29588	2.89863	2.9997	5.30416	5.34975
PLCG1	6.63373	6.46632	6.66148	6.58623	5.71381	4.94786
PLCG2	-0.489066	-0.598389	0.121519	0.8424 ?	?	
PLCL2	3.33295	3.44854	1.14744	1.24877	4.08621	4.67049
PLCXD1	3.22276	2.84322	2.32428	2.28779	1.7868	0.597145
PLD1	4.85215	4.4928	3.6822	3.97198	3.87009	3.90246
PLD2	3.1682	2.87032	3.08794	3.22433	3.30836	3.08445
PLD3	5.00481	4.76596	4.73534	5.03072	4.39428	4.44176
PLD6	1.91846	1.82432	-2.86056	-3.30006	1.88025	1.16369
PLEC	6.61819	6.5905	7.62314	7.62666	6.34201	6.65548
PLEKHA1	4.93432	4.66432	3.62668	3.2903	5.07376	4.09419
PLEKHA2	1.48085	1.338	4.77502	5.06671	5.68149	5.61577

PLEKHA3	4.23351	4.43679	5.30109	5.67456	4.97211	5.10007
PLEKHA3P1	-0.984948	-0.639539	0.265941	0.989684	0.699401	0.903686
PLEKHA4	3.60355	3.59107	2.759	2.98658	-0.121008	-0.348991
PLEKHA5	4.54411	4.59987	4.75311	4.83147	0.99089	1.29333
PLEKHA6	-6.68418	-4.45968	4.08795	4.90241	-4.65668	-4.70296
PLEKHA7	-5.10914	-4.13839	-4.59508	-2.64851	4.29968	3.84595
PLEKHA8	4.86764	4.64005	5.0789	5.02124	4.81722	4.1565
PLEKHA8P1	2.4571	2.3846	2.37287	2.49414	2.23579	1.92721
PLEKHB1	3.46959	3.40905	-1.72363	-1.78641	3.61347	3.3834
PLEKHB2	6.15942	6.22077	5.55126	6.0749	6.74412	6.93916
PLEKHF1	-0.175369	-0.354149	1.71737	2.16265	2.24359	2.78114
PLEKHF2	2.54172	2.72451	2.48182	2.90799	2.99612	3.2181
PLEKHG1	3.60355	3.55117	-1.13882	-1.86039	6.89927	6.8598
PLEKHG2	3.40421	3.0568	4.01615	3.62589	3.89619	3.07137
PLEKHG3	3.43681	3.34621	2.54313	2.47199	0.790972	-0.00615756
PLEKHG5	1.01523	1.15926	3.1088	2.77688	3.14705	2.908
PLEKHG6	-0.45097	-0.681268	1.29225	1.04098	-0.753191	-1.61882
PLEKHH1	3.42274	3.08613	-0.368676	-0.430946	0.885949	-0.255853
PLEKHH2	2.7082	2.44105	2.31235	1.61231	3.71364	2.91167
PLEKHH3	1.93757	1.45008	2.16522	2.25751	2.3397	1.95152
PLEKHJ1	3.30354	3.13272	2.97308	2.9522	4.45238	4.44585
PLEKHM1	3.24967	3.08446	3.66	3.88547	3.92889	3.9308
PLEKHM1P	2.68869	2.51548	3.59292	3.57354	3.6541	3.10887
PLEKHM2	4.00575	3.74209	4.20621	4.43303	4.94364	4.95317
PLEKHM3	2.22871	2.1482	2.03567	1.76157	2.31699	2.19417
PLEKHO1	3.76035	3.39358	3.3498	3.51871	3.3298	2.76497
PLEKHO2	3.4068	3.20137	3.91185	4.2638	2.30332	2.00067
PLEKHS1	-1.52875	-2.37465	0.996447	1.0511	0.886652	-0.577229
PLGRKT	1.16516	1.14454	1.57806	2.03075	3.22234	4.19264
PLIN2	-3.2383	-3.00228	3.04289	3.65438	-4.65668	-5.69922
PLIN3	4.30093	4.1882	4.54099	5.07107	5.08869	5.38271
PLK1	4.8224	4.84617	4.39202	4.79506	4.46002	4.39947
PLK2	5.15032	4.84264	7.60082	7.98547	5.95943	6.33253
PLK3	0.878411	1.18826	1.36281	2.06612	1.63434	2.0681
PLK4	4.75213	4.77674	4.0891	4.2176	4.41315	4.49172
PLLP	1.03142	1.41318	1.16273	1.92826	2.23755	2.24759
PLOD1	6.5412	6.35035	6.8244	6.87946	8.1477	7.17766
PLOD2	6.78522	6.81632	8.97505	9.42493	8.62684	8.03934
PLOD3	7.04414	6.69891	5.54219	5.58679	5.86444	5.43258
PLP1	6.21976	6.12732	-0.300715	-0.464785	-1.57243	-3.38329
PLP2	4.82693	4.88531	4.80161	5.52644	4.34249	4.69201
PLRG1	5.08145	5.22627	4.84576	5.27971	4.91428	5.51788
PLS1	2.60471	3.11648	1.75902	2.44577	3.62739	4.33768
PLS3	4.79125	4.97282	6.23826	6.75463	7.63453	8.50785
PLSCR1	2.9321	3.08019	3.98389	4.13553	4.97177	5.15436
PLSCR4	1.05586	1.43806	4.47735	5.03581	1.46385	2.02123
PLXNA1	5.22051	4.9832	5.87412	5.69488	4.9599	4.54712
PLXNA2	1.12115	0.877069	3.9509	3.80854	-0.594016	-0.706491
PLXNA3	3.17897	2.94443	3.18499	3.4206	3.55053	2.94075
PLXNB1	4.80026	4.25739	3.52901	3.4686	3.94238	3.6618
PLXNB2	5.92183	5.67287	6.92154	6.74893	5.31445	4.97977
PLXNB3	3.28252	2.69264	2.31824	2.32464	3.05642	2.76088

PLXNC1	2.28188	2.42354	2.67243	3.55304	0.818106	0.886722
PLXND1	4.05497	3.8375	6.19556	6.00198	6.08682	5.90936
PM20D2	4.0691	4.51514	1.61093	2.14386	3.02269	2.9515
PMAIP1	4.21456	4.34127	3.54781	4.001	4.47731	4.71007
PMEPA1	8.35444	8.48442	3.31149	3.31192	6.3146	6.12273
PMF1	4.27981	4.21411	2.76234	3.17073	4.02306	4.31087
PML	3.5583	3.58972	4.79589	4.877	4.27264	4.10063
PMM1	2.23727	1.84839	3.21998	3.49342	2.96975	3.34179
PMM2	4.43031	4.21596	4.15514	4.38983	4.67586	4.38055
PMP22	7.53015	7.14837	4.27524	4.41955	6.53866	6.11609
PMPCA	4.92066	4.74186	4.31304	4.51779	5.01341	4.74305
PMPCB	5.20601	5.19705	4.5909	5.0297	5.53055	5.70167
PMS1	4.01173	4.07145	3.41639	3.50588	4.37655	4.71849
PMS2	3.86836	3.78246	3.95845	4.06868	3.60798	3.56207
PMS2CL	2.69913	2.55384	3.24196	3.13877	3.03894	2.69331
PMS2P1	1.10761	1.04298	0.920194	1.18316	1.13529	1.74959
PMS2P3	1.76246	1.75893	2.02295	2.17799	1.71949	1.2884
PMS2P4	0.290436	0.0331587	0.219339	0.474369	-0.142693	0.00947617
PMVK	2.65628	2.40135	1.89865	1.99449	2.45344	2.61523
PNISR	5.92202	5.89825	6.569	6.39865	7.39639	6.62136
PNKD	3.59325	3.65998	3.89862	4.378	3.67731	4.3031
PNKP	2.88213	2.8455	2.96369	3.08839	3.55521	3.44811
PNMA1	2.60699	2.19895	2.83885	2.82192	3.59235	2.85554
PNMA2	?	-6.45019	3.19369	3.53684	-2.02922	-2.28115
PNMAL1	-6.68418	?	-4.18092	-3.30006	2.49706	2.56008
PNN	6.57377	6.54661	5.71808	5.77495	6.57489	5.74397
PNO1	4.02975	4.05475	3.26765	3.83665	4.25549	4.62994
PNP	2.23463	2.41675	2.93283	3.58466	4.10868	4.9506
PNPLA2	2.94298	2.67159	3.48536	3.63923	2.94518	2.77307
PNPLA3	3.21246	3.13578	3.26173	3.59186	-6.64581	-5.6744
PNPLA6	4.13578	4.06851	3.92467	4.0978	4.71244	5.06704
PNPLA8	4.56094	4.73259	4.12513	4.53759	4.88918	5.83501
PNPO	3.92113	3.8156	3.22587	3.76461	4.36537	4.65194
PNPT1	5.0591	5.10252	4.10746	4.50504	5.16561	5.30705
PNPT1P1	1.84034	1.9654	0.937103	1.25669	2.13575	1.71412
PNRC1	4.35958	3.88809	3.95218	3.9892	4.39294	4.08607
PNRC2	6.12232	6.14284	5.12172	5.31163	6.06739	6.38177
POC1A	3.50916	3.53152	3.13615	3.63431	3.36482	3.51487
POC1B	3.27041	3.60692	3.1405	3.36669	2.69255	3.29384
POC1B-GALNT4	0.983773	1.29821	0.594147	1.34253	-0.262654	0.509858
POC5	2.77868	2.85231	3.17623	3.48364	2.91145	3.51003
PODXL	9.92316	10.0103	3.1541	3.47237	5.42615	5.17821
PODXL2	3.94296	3.89581	-6.17309	-6.09892	-2.13612	-4.70296
POFUT1	7.15215	7.19578	6.73848	7.07803	6.43344	6.39295
POFUT2	3.66833	3.56645	5.33757	5.46198	4.75332	4.3634
POGK	6.26375	6.38262	4.68298	5.09389	5.43768	5.64312
POGLUT1	3.76444	3.95606	4.18934	4.66018	3.97085	4.39788
POGZ	5.59928	5.37319	5.02836	4.75922	5.36965	4.70901
POLA1	4.6535	4.62988	5.05488	5.19437	4.67509	4.72998
POLA2	5.04112	5.04841	4.21735	4.43999	3.16306	2.95507
POLB	2.60927	2.82718	2.35177	2.65605	2.50963	3.20318
POLD1	3.39631	2.9423	2.92762	3.19724	3.28221	3.27913

POLD2	5.17204	5.04939	5.35804	5.71014	5.5724	5.76849
POLD3	4.63965	4.60122	5.00321	5.06797	3.86128	3.60843
POLD4	2.96795	2.87417	2.97141	3.27006	3.02553	3.42417
POLDIP2	4.88537	4.81675	4.88794	5.35263	6.17662	6.26445
POLDIP3	5.32188	5.392	4.81294	4.99837	5.08071	5.13563
POLE	6.21248	5.99351	5.63423	5.56137	4.97993	4.38472
POLE2	2.34259	2.29539	1.34008	1.55654	2.0086	2.26485
POLE3	5.36176	5.33117	4.52273	5.05357	5.39784	5.70741
POLE4	2.78676	2.69451	0.674533	1.39106	3.97359	4.94612
POLG	4.77049	4.59868	4.54366	4.56804	4.40381	4.06754
POLG2	3.07736	3.14264	2.76193	2.73353	3.54671	3.24749
POLH	3.24637	3.53151	3.48358	3.80258	4.08366	4.05981
POLI	3.852	3.73751	3.81228	3.68939	2.80322	2.45158
POLK	4.24442	4.32668	5.15465	5.28294	4.78075	5.11026
POLL	2.87887	2.89175	3.01021	3.25567	3.54473	3.25607
POLM	3.1759	2.8881	2.9839	2.87417	2.80526	2.29893
POLQ	5.43209	5.53434	5.8636	5.94164	4.98574	4.86976
POLR1A	6.04365	6.04197	5.38013	5.59462	5.88924	5.41158
POLR1B	6.02499	6.09726	4.03927	4.3506	6.12615	6.13995
POLR1C	3.54691	3.67315	2.70073	3.09304	4.00989	4.34123
POLR1D	4.53633	4.38577	4.01614	4.14383	4.72877	5.01438
POLR1E	4.1843	4.34932	3.48713	3.91769	5.64406	5.68452
POLR2A	5.42856	5.6548	5.80018	6.21365	5.76544	6.10667
POLR2B	6.41214	6.34634	5.77232	6.20051	7.9845	8.26363
POLR2C	4.70818	4.877	4.72996	5.35563	6.28148	6.83146
POLR2D	5.32606	5.47046	4.49331	4.80181	5.84806	6.04482
POLR2E	4.59392	4.32178	4.36722	4.65928	5.31981	5.41286
POLR2F	4.53974	4.45325	2.96685	3.37363	3.66242	3.89701
POLR2G	4.20254	4.21134	3.66667	4.01011	4.19689	4.88853
POLR2H	4.41205	4.40134	4.1214	4.46197	5.63574	6.16748
POLR2I	2.16427	2.21048	1.97205	2.411	2.83953	3.11489
POLR2J	4.20634	4.04089	3.33544	3.76434	4.17637	4.7159
POLR2J2	2.20409	2.12771	3.26752	3.19261	2.04421	1.48344
POLR2J3	5.07268	4.91711	5.45824	5.35198	5.37352	4.57628
POLR2J4	2.56759	2.59611	2.94848	2.771	2.65656	2.24028
POLR2K	4.39368	4.47525	3.606	4.15092	5.0891	6.09337
POLR2L	4.61827	4.32817	4.44698	4.70963	4.29265	4.6965
POLR2M	4.12711	4.37288	3.92226	4.55509	4.75578	5.25773
POLR3A	4.9883	4.82774	4.27385	4.3669	5.5748	5.32463
POLR3B	3.78168	3.88469	2.82771	3.27048	3.18674	3.33209
POLR3C	3.78776	3.88033	3.15187	3.66595	3.97633	4.34721
POLR3D	4.80822	4.78863	3.92238	4.27054	4.51211	4.22697
POLR3E	4.51097	4.57194	3.89995	4.29098	4.14623	4.16211
POLR3F	3.82831	4.03898	2.82772	3.3258	2.95441	3.28481
POLR3G	3.52716	4.13948	2.92274	3.49851	4.21235	4.55518
POLR3GL	1.88966	1.91222	2.71433	2.97066	2.49198	2.75682
POLR3H	5.10407	5.02	4.39861	4.60182	4.80974	4.77944
POLR3K	1.73035	1.58126	1.87079	2.47152	2.58832	2.38059
POLRMT	3.27212	3.31424	2.64823	2.8705	3.79651	3.86598
POLRMTP1	2.00913	2.05091	1.25239	1.59935	2.34651	2.08341
POM121	4.64962	4.35563	4.9418	4.69	4.88072	4.50978
POM121C	4.78124	4.55617	4.67958	4.38695	4.62736	4.3479

POM121L9P	-3.50674	-3.42223	1.58485	1.93174	-5.65305 ?	
POMGNT1	5.56077	5.69389	5.60724	5.96232	5.62922	6.32089
POMGNT2	4.24708	4.22976	2.72338	3.0611	3.82553	3.84017
POMK	4.8976	5.21925	3.32229	3.6494	3.88658	3.72583
POMP	5.34184	5.55816	4.96746	5.62165	5.24464	6.32976
POMT1	3.57704	3.37793	3.26769	3.27272	3.9086	3.2677
POMT2	4.03478	3.78981	3.35176	3.21761	4.01476	3.66832
POMZP3	2.10251	1.90266	1.77122	1.998	1.35413	1.30634
PON2	5.38447	5.51651	5.47401	5.7525	5.35553	6.12674
POP1	4.33638	4.4175	4.31332	4.70895	4.57749	4.91716
POP4	3.24783	3.30203	3.69606	4.18694	4.27778	4.89783
POP5	3.33159	3.41213	2.72638	3.17195	3.29824	3.93353
POP7	3.94206	3.79454	2.76777	3.1248	3.81542	4.07955
POPDC3	2.97688	2.66387	2.04532	2.56714	4.9599	5.56005
POR	4.85886	4.75086	4.79304	5.18866	5.01651	5.06806
PORCN	1.95199	2.55119	1.0162	1.96801	1.04018	1.54125
POSTN	1.82451	1.38894	2.93023	0.935626	-0.490197	-2.38405
POT1	3.93482	3.91869	3.85539	4.11517	3.7847	4.30171
POTEE	2.41797	2.42939	2.85254	3.03912	3.39269	3.24828
POU2F1	5.94068	5.83894	4.95473	4.85477	4.96492	4.14348
POU3F2	4.30934	4.26336	-3.59683	-5.10387	5.68425	5.3363
POU3F3	-0.144345	-0.105152	-6.17309 ?		3.07271	2.66398
POU5F2	2.12991	2.24453	2.15189	2.02562	2.06403	1.39136
PP7080	2.52905	2.5821	2.28693	2.48849	2.24635	2.07646
PPA1	5.0667	5.18616	4.2667	4.93798	5.55157	6.00935
PPA2	4.0143	4.1656	3.50823	3.78751	3.8914	5.22253
PPAN	3.56177	3.37951	2.12483	2.5494	4.11612	3.84787
PPAP2A	1.89156	2.12294	5.64849	5.59109	3.68032	3.92816
PPAP2B	3.88681	3.64568	2.9406	3.28134	0.82357	1.87074
PPAP2C	4.58978	4.7405	4.04409	4.46197	5.22576	5.4341
PPAPDC1A	2.643	2.69703	0.156448	1.06115	2.04362	3.03812
PPAPDC1B	2.84396	3.00068	3.44766	3.87273	2.79708	2.86315
PPAPDC2	2.14324	2.26509	0.956168	1.01017	3.5637	3.71745
PPARA	3.78775	3.52726	4.89128	5.0075	4.02968	3.63536
PPARD	2.30937	2.041	3.11798	3.393	4.4145	4.13563
PPARG	3.98741	4.18551	-2.18288	-2.10822 ?		-4.11926
PPARGC1A	-4.37417	-5.45651	2.77359	3.23325	3.10622	3.42257
PPARGC1B	2.30375	2.12589	2.84991	3.1603	2.08454	1.87074
PPAT	4.75821	4.83944	3.96403	4.32421	6.46882	6.48553
PPATP1	1.41384	1.36007	0.0992952	0.898433	3.09058	3.1644
PPCDC	1.96272	1.95503	2.36724	2.46861	2.03668	2.00067
PPCS	3.27937	3.2824	3.29053	3.54813	3.16688	4.0011
PPDPF	1.7314	1.55956	1.41643	1.37503	1.4996	1.72795
PPFIA1	5.5168	5.65275	5.07288	5.34422	5.05413	5.03952
PPFIA2	-2.1899	-1.81849	-7.52086	-6.09892	0.654023	2.08334
PPFIA3	1.43024	0.877069	1.12031	1.13916	-0.217212	-0.280287
PPFIA4	1.06916	1.13712	1.11115	0.659441	3.05394	0.0747657
PPFIBP1	6.69802	6.61272	5.20495	5.41277	5.80528	5.49949
PPFIBP2	3.08229	3.06854	-1.37581	-0.823182	-0.932127	-0.706491
PPHLN1	5.34258	5.3733	4.75762	4.91009	4.73629	4.75991
PPIA	8.41128	8.40467	8.347	8.70662	8.75021	9.11559
PPIAP22	7.83386	7.78387	7.80742	8.12728	8.02888	8.48836

PPIAP29	3.8631	4.04369	3.94863	4.38602	4.49494	4.59901
PPIAP31	4.83951	4.72068	4.83765	5.01976	4.95775	5.67712
PPIB	8.24087	7.91075	7.46759	7.7445	7.53174	7.88045
PPIC	4.21666	4.18055	4.12534	4.35974	3.87099	4.07942
PPID	3.81684	3.86012	3.38885	3.57483	4.13424	4.10363
PPIE	4.17298	4.25276	3.4942	3.87274	4.29896	4.74861
PPIF	5.40092	5.08789	5.24273	5.70455	6.18499	6.06971
PPIG	5.75518	5.83033	5.25733	5.45246	5.91498	5.99283
PPIH	2.94658	3.07438	2.63027	3.09085	3.08284	3.61295
PPIHP1	0.77894	0.83556	1.08969	1.25414	1.21013	1.54635
PPIL1	4.35753	4.18729	3.73384	4.12838	4.88273	5.33903
PPIL2	4.55424	4.53662	4.15181	4.07144	4.14867	3.95705
PPIL3	2.27822	2.36372	2.30031	2.42257	3.08956	3.77104
PPIL4	3.93754	3.91978	3.86359	3.9586	4.4696	4.7016
PPIP5K1	3.95721	4.04359	3.45845	3.65738	4.01173	3.91279
PPIP5K2	5.31632	5.76798	5.39254	5.90209	5.37582	6.35001
PPL	0.312247	-0.0197685	1.5847	2.40294	-1.16815	-1.4584
PPM1A	5.09776	5.28491	4.0891	4.21084	4.65596	4.7951
PPM1B	4.31109	4.31283	4.30231	4.39995	4.67471	4.39657
PPM1D	3.81564	3.90675	3.05727	3.24432	3.68731	3.82855
PPM1E	3.04659	2.77591	0.285923	0.682969	0.699738	0.772156
PPM1F	5.71272	5.5795	4.53291	4.91656	4.73885	4.85226
PPM1G	6.72719	6.67582	5.39907	5.59762	7.09377	6.65635
PPM1H	1.48583	1.41371	1.40148	1.85699	-2.26731	-2.53597
PPM1K	3.36701	3.31523	3.75605	3.70975	3.77536	3.5003
PPM1L	2.42506	2.10703	3.20018	2.97599	-0.615709	-1.24695
PPM1M	2.70607	2.67415	2.64938	2.74605	2.69505	2.95863
PPME1	5.20187	4.98864	5.29262	5.31756	5.07794	4.57942
PPOX	2.63823	2.56844	2.19178	2.35405	2.36419	2.34701
PPP1CA	5.98207	5.94461	5.24973	5.4993	5.78178	6.01481
PPP1CB	6.79397	6.93529	6.08561	6.53924	8.36035	8.68214
PPP1CC	6.61521	6.51279	5.71506	6.08191	6.54836	6.65578
PPP1R10	4.98828	4.92301	4.98952	5.19209	5.33475	5.24965
PPP1R11	3.6251	3.60718	3.20439	3.71747	4.60808	5.12368
PPP1R12A	5.71763	5.86285	5.70677	5.85517	6.39865	6.24935
PPP1R12B	4.68145	4.65391	4.70661	4.61869	3.91196	3.38435
PPP1R12C	3.51888	3.20249	3.78514	4.09696	4.14462	4.38471
PPP1R13B	2.28678	1.99661	1.34008	1.30061	1.27048	1.1637
PPP1R13L	0.409538	0.195474	0.503064	0.248838	2.42716	1.63317
PPP1R14B	5.27544	5.13669	3.68715	3.84485	5.00043	5.11908
PPP1R14BP3	5.12011	5.13788	3.5508	3.87324	4.99578	4.78306
PPP1R14C	2.643	2.75633	-5.1783	-6.09892	3.10622	2.68131
PPP1R15A	4.03901	3.90128	4.0644	4.64939	3.54914	3.22403
PPP1R15B	6.66586	6.69638	6.08881	6.31533	6.78275	6.90844
PPP1R16A	1.8042	1.65697	2.05036	2.4556	2.77284	2.80191
PPP1R17	-0.569597	-0.375208	?	?	1.80732	1.12623
PPP1R18	4.95598	4.8602	5.1652	5.3169	5.39496	5.49418
PPP1R2	4.11338	4.24729	4.87215	5.2155	5.29491	5.35851
PPP1R21	2.51525	2.58429	2.6109	2.86846	3.17889	3.77104
PPP1R26	1.77668	1.74664	3.64302	3.92183	2.59944	2.64644
PPP1R2P3	0.426447	0.927517	1.35864	1.81427	1.89861	2.69951
PPP1R35	2.30994	2.37637	2.02836	2.18086	2.50996	2.69633

PPP1R37	2.71878	2.56231	3.0235	3.26404	3.20543	3.30172
PPP1R3B	2.98772	3.16443	2.10445	2.52566	2.83559	2.67995
PPP1R3C	1.49572	1.27191	1.24279	1.36695	3.94701	3.33221
PPP1R3D	2.91161	2.84899	1.96438	2.16587	2.45468	2.42035
PPP1R3E	1.43438	1.61209	1.44587	1.20285	1.20393	0.815379
PPP1R3F	2.18052	2.26168	1.29226	1.57069	-1.95932	-1.79933
PPP1R3G	-2.52833	-2.87684	-1.93506	-1.86039	1.76178	1.58794
PPP1R7	3.58097	3.65782	3.5324	3.99423	4.31948	4.92075
PPP1R8	4.94342	5.0022	3.76191	4.23989	4.97492	5.20017
PPP1R8P1	0.944836	0.955067	-0.935332	-1.30113	0.186095	-0.280287
PPP1R9A	4.74799	4.48186	-3.85951	-2.64851	5.88803	5.85647
PPP1R9B	5.25472	5.15553	4.46301	4.72326	5.11612	4.81782
PPP2CA	6.35574	6.39223	6.19346	6.63337	6.87865	7.03126
PPP2CB	4.25581	4.40133	3.61252	4.11034	4.77223	4.94782
PPP2R1A	5.83567	5.65118	5.53746	5.79241	6.5917	6.46256
PPP2R1B	3.94597	4.09682	4.75466	4.99974	5.10009	4.99381
PPP2R2A	4.54765	4.62858	4.32427	4.44575	4.53567	4.58905
PPP2R2B	1.02208	1.06077	3.35951	3.57942	4.23374	4.74963
PPP2R2D	3.6251	3.54138	3.89195	4.13909	4.20154	4.1698
PPP2R3A	3.43277	3.46937	3.32823	3.2399	4.54425	4.57984
PPP2R3B	2.2198	2.1408	2.50299	2.52418	0.448309	0.463298
PPP2R3C	2.85153	2.91581	2.49123	2.76308	3.22205	4.10679
PPP2R4	5.20027	5.12257	4.81011	5.08469	6.11571	6.44238
PPP2R5A	3.40945	3.35574	3.21308	3.30057	3.3909	3.46571
PPP2R5B	2.52735	2.51158	2.18282	2.52418	2.54672	1.99375
PPP2R5C	5.39854	5.28694	4.67862	4.76609	5.69897	5.77792
PPP2R5D	4.64546	4.62765	4.3067	4.51727	5.11946	4.849
PPP2R5E	4.78756	4.78544	4.0061	4.1379	4.45829	4.51221
PPP3CA	4.12905	3.91978	5.4693	5.63138	4.49196	4.14659
PPP3CB	4.04575	4.10415	3.17183	3.39698	4.95025	4.98415
PPP3CB-AS1	0.728748	0.804211	1.38361	1.36213	1.32843	1.28379
PPP3CC	2.54784	2.67923	1.82299	2.08115	2.48019	2.68896
PPP3R1	4.9209	4.95361	4.4828	4.67548	5.0487	5.2566
PPP4C	4.8755	4.73988	4.54439	4.77228	5.1062	5.33219
PPP4R1	4.63631	4.60793	5.72335	5.98326	6.61667	6.65247
PPP4R1L	3.10505	3.27017	0.474784	0.19507	1.17894	0.893261
PPP4R2	6.09269	6.24792	5.68771	5.83984	5.81114	5.87092
PPP4R3A	5.20254	5.2315	4.3624	4.50034	5.48466	5.36071
PPP4R3B	5.75081	5.84812	5.47717	5.7591	6.20009	6.53778
PPP4R3CP	2.42247	2.28872	?	-6.09892	?	-4.70296
PPP4R4	2.5441	2.95922	-1.37581	-1.25052	-5.65305	-5.69922
PPP5C	4.87504	4.81734	4.39296	4.52598	5.0196	4.89598
PPP6C	4.74129	4.7809	4.47913	5.03068	5.32048	5.49847
PPP6R1	4.74814	4.48006	4.59711	4.76509	5.09772	5.08621
PPP6R2	4.24416	4.0602	5.16273	5.22965	4.2738	4.40442
PPP6R3	6.9796	6.89986	6.77967	6.92381	6.39949	6.1012
PPRC1	4.78069	4.75087	4.51692	4.70974	4.85883	4.5293
PPT1	6.78169	6.93128	6.44197	6.83457	6.90105	7.45517
PPT2	4.4997	4.36739	3.13204	3.36905	3.89961	3.87948
PPT2-EGFL8	1.67559	1.5514	1.34369	1.39877	1.87554	1.21079
PPTC7	4.12429	4.14541	3.06915	3.53142	3.84052	3.73
PPWD1	3.96625	3.99453	4.48536	4.60111	4.28951	4.53049

PQBP1	3.95286	4.06267	3.42566	4.00361	3.5637	3.87824
PQLC1	3.18357	2.87701	2.63027	2.786	1.66165	2.16367
PQLC2	2.80276	2.44706	3.08794	3.31113	3.20543	2.89321
PQLC3	0.595628	0.988427	1.46038	1.79207	1.02625	2.16983
PRADC1	2.41689	2.38221	2.48892	2.90799	2.98503	3.64201
PRAF2	1.82451	1.87703	2.43488	2.66117	1.83157	2.07467
PRC1	7.11023	7.15498	6.80797	7.28353	6.87855	6.88998
PRC1-AS1	0.74719	0.442068	0.783423	0.719674	-0.366868	-2.74829
PRCC	4.33709	4.19625	3.68684	3.83954	4.22461	4.17898
PRCP	5.05538	4.90564	5.7181	5.89117	4.62968	4.69415
PRDM1	0.0559314	0.635262	2.96369	2.92185	2.97087	2.15129
PRDM10	3.58864	3.71582	3.14516	3.19267	3.50585	3.20018
PRDM11	2.15892	1.93806	1.5913	1.71138	0.405873	0.483199
PRDM15	3.61267	3.44404	3.4275	3.41865	3.38681	2.82468
PRDM2	3.8284	3.69955	4.4033	4.10792	4.39499	4.05815
PRDM4	4.33402	4.26838	3.62563	3.83794	4.47068	4.53455
PRDM5	3.24344	2.9122	1.31637	1.31748	2.87303	2.6899
PRDM6	-0.998365	-1.93875	1.42385	1.91042	-1.07506	-0.348991
PRDX1	7.15697	7.07415	8.57808	8.9507	9.00624	9.44356
PRDX1P1	2.22938	2.34814	3.77961	4.23332	4.3605	4.12668
PRDX2	5.30999	5.19758	4.36914	4.53772	6.09682	6.12577
PRDX3	5.97775	6.15026	5.64739	6.2648	6.54635	7.37725
PRDX3P1	0.323393	0.656176	0.371169	0.833756	0.395063	0.817263
PRDX4	4.91059	4.81211	4.97257	5.35824	5.10459	5.85028
PRDX5	5.4494	5.37199	4.13614	4.66265	4.78626	5.62644
PRDX6	6.68548	6.92914	6.10789	6.75805	6.32246	6.89688
PREB	4.81168	4.63254	3.92238	4.17541	4.79398	4.85647
PRELID1	5.44238	5.39335	4.99825	5.50973	6.52537	6.8552
PRELID1P1	1.00018	1.23484	0.635987	1.35604	2.14072	2.45998
PRELID2	1.39899	1.58158	2.74426	3.0686	2.94147	2.93354
PREP	4.38506	4.19804	3.33415	3.39897	5.54364	5.41546
PREPL	5.17343	5.26658	4.9345	5.26122	5.44485	5.61367
PREX1	5.5426	5.55636	3.70521	4.58205	3.85736	4.50394
PRICKLE1	0.821457	1.16613	0.978393	0.497556	5.49731	4.83921
PRICKLE2	3.78857	3.67924	4.66552	4.57463	4.96386	4.84612
PRICKLE2-AS1	-0.212203	0.117083	1.13738	0.986695	0.265605	0.213553
PRICKLE3	0.440559	0.382745	0.686917	1.1104	0.172692	-0.31423
PRICKLE4	1.1901	1.37035	1.72162	1.82296	0.985799	0.72772
PRIM1	2.90704	3.05483	3.38667	3.61144	2.93814	3.58739
PRIM2	4.1651	4.44555	4.25005	4.53682	4.35021	4.96923
PRIMA1	1.98043	1.96763	-5.1783	-6.09892	?	?
PRIMPOL	2.50374	2.44294	2.89096	3.0976	1.86511	2.46743
PRKAA1	5.15888	5.54907	5.50341	5.97531	5.76987	5.99978
PRKAA2	4.24855	4.38185	0.64945	1.1104	5.24808	5.08524
PRKAB1	3.51646	3.39358	2.6109	2.92735	3.29969	3.35668
PRKAB2	4.84925	4.7362	4.10533	4.25201	5.59971	5.63813
PRKACA	2.63408	2.15556	2.94319	2.786	3.37174	3.02452
PRKACB	4.64519	4.69195	4.52556	4.95052	5.01519	5.36607
PRKAG1	4.73818	4.79584	3.7005	4.14668	4.71762	5.41911
PRKAG2	2.97511	3.0068	2.73236	3.26186	4.91428	5.13798
PRKAR1A	7.14355	6.96419	6.46002	6.86856	7.18208	7.11405
PRKAR1B	1.46083	1.78551	1.22592	1.48367	2.31993	2.54122

PRKAR2A	5.59467	5.55083	5.1729	5.40834	6.81418	6.76852
PRKAR2B	3.86793	4.25226	3.59947	4.14855	2.18206	2.15129
PRKCA	2.5536	2.46198	5.45446	5.48268	6.13795	5.23799
PRKCA-AS1	-0.026521	0.344613	1.91453	1.71138	0.894341	0.0483059
PRKCD	4.18816	3.95922	2.28002	2.4799	0.185296	0.422664
PRKCDBP	4.16741	4.31524	-0.539469	0.591699	0.210164	1.3157
PRKCE	3.24197	3.27017	3.5444	3.86845	3.68176	3.67483
PRKCH	1.14018	0.766044	-2.72315	-0.836981	2.85143	3.17593
PRKCI	4.43049	4.74137	5.13516	5.51059	4.76297	4.84022
PRKCSH	6.13874	6.03575	6.1685	6.26865	6.79822	6.43794
PRKD1	3.15735	2.85004	1.88887	1.96801	3.65029	3.72583
PRKD2	2.1083	1.71211	2.92239	3.05357	3.05394	2.64644
PRKD3	6.28533	6.39395	5.66409	5.78827	5.40723	5.39682
PRKDC	9.48477	9.51379	8.94369	9.13817	8.54766	8.2738
PRKRA	3.3562	3.35733	4.74054	4.92665	4.07609	4.55184
PRKRIP1	3.33915	3.29288	3.60445	3.57999	3.44196	3.04297
PRKRIR	5.72768	5.99177	4.75141	5.16108	4.73643	5.56927
PRKRIRP7	2.66669	2.83361	2.3797	2.43657	1.85426	2.57023
PRKX	2.83398	2.95051	2.8595	2.9888	2.76685	2.29832
PRKY	-6.66478	-5.44505	3.52641	3.32813	?	-5.66892
PRLR	-1.27248	-1.50825	-2.09546	-0.380732	3.35789	3.94432
PRMT1	5.70763	5.57525	5.26745	5.76701	6.20323	6.46496
PRMT1P1	0.454292	0.598156	0.963252	0.988187	1.21239	0.649036
PRMT2	4.42697	4.35573	4.63186	4.82928	4.99431	5.0058
PRMT3	4.05413	4.25054	3.3498	3.62926	3.88658	4.24165
PRMT5	5.04057	5.11338	3.80718	4.13734	4.95733	5.23058
PRMT5-AS1	0.801531	0.825419	-0.177591	0.106844	0.465306	0.0640827
PRMT6	3.93754	3.99658	2.63985	3.00752	4.33615	4.56474
PRMT7	3.4113	3.3113	3.64498	3.96297	3.99672	3.8534
PRMT9	2.94658	2.9187	2.86905	2.97599	2.69505	3.36475
PRNP	7.46681	7.55615	8.00558	8.68018	7.2808	7.441
PROCA1	0.255223	-0.00328298	1.38638	1.0909	0.134221	-0.95156
PROCR	4.52831	4.76803	4.4337	4.64436	3.54824	4.87241
PRORSD1P	0.377835	0.432061	0.871828	0.761609	0.147163	-0.752286
PROS1	2.02199	1.95081	2.33612	1.80409	0.107996	0.816919
PROSC	3.36835	3.35253	3.36145	3.71612	3.76175	4.07792
PROSER1	3.28106	3.14634	3.1541	3.05357	2.79913	2.70692
PROSER3	1.7169	1.60744	3.25635	3.35208	2.4849	2.40472
PROX1	2.52207	2.44406	-6.17309	-4.10635	3.1896	3.23873
PRPF18	2.93241	3.07028	2.55073	2.85104	3.26017	3.6638
PRPF19	5.46864	5.35933	4.41452	4.81152	4.67619	4.81683
PRPF3	5.30526	5.35605	4.72839	4.87886	5.32157	4.96933
PRPF31	4.37576	4.25061	4.18794	4.48214	4.96309	4.78968
PRPF38A	5.20473	5.10262	4.95401	5.01885	5.47356	5.22843
PRPF38B	5.6598	5.62924	5.2045	5.14501	5.59911	5.00235
PRPF39	4.76651	4.65603	4.21128	4.12703	4.5246	4.23983
PRPF4	5.0542	5.14655	3.99186	4.37795	5.30747	5.27913
PRPF40A	6.58762	6.62875	6.36961	6.57985	6.72177	7.11805
PRPF40B	2.7956	2.77047	2.58407	2.71352	1.40381	1.32331
PRPF4B	6.37128	6.35877	6.22897	6.16813	6.90814	6.71194
PRPF6	6.14326	6.18662	5.16187	5.56756	6.21101	6.25074
PRPF8	7.81842	7.78001	7.7962	7.99842	8.16394	7.83835

PRPS1	4.69614	4.72743	4.40037	4.9306	4.8513	5.11816
PRPS1P2	0.553469	0.30793	0.805235	0.750589	0.237613	-0.312577
PRPS2	4.21679	4.1537	4.10534	4.44576	4.94564	4.91625
PRPSAP1	3.89617	4.09939	3.59456	3.88269	4.1961	4.31981
PRPSAP2	2.83035	3.08983	3.0137	3.4952	3.65839	4.04315
PRR11	5.26513	5.34741	5.0762	5.54646	5.61553	5.57753
PRR12	1.83622	1.45008	2.39769	2.07607	2.01214	1.97279
PRR13	4.09902	4.09693	4.0896	4.74902	4.23878	4.52131
PRR13P5	0.823749	0.876519	1.0154	1.64047	1.08045	1.61592
PRR14	1.73558	1.554	1.48185	1.71138	1.62974	1.70271
PRR14L	6.16299	6.1221	6.02835	6.1958	5.88126	5.70161
PRR16	0.840145	0.060836	0.00643448	0.383121	2.08118	1.493
PRR26	2.37775	2.18822	2.38255	1.97335	1.17263	0.401903
PRR3	3.54725	3.41262	3.30882	3.34408	4.12855	3.64665
PRR34-AS1	0.577131	0.929533	0.893354	1.49112	1.35654	1.84021
PRR4	2.31483	2.44671	2.67859	2.47464	1.96114	1.60141
PRR5L	-1.34118	-1.46246	2.24276	2.61228	2.76176	2.99026
PRR7-AS1	1.23351	0.6247	-0.117291	0.213217	0.485993	0.156837
PRRC1	5.02926	5.13333	5.31033	5.58247	5.02179	5.1458
PRRC2A	5.71219	5.59684	5.96747	5.89187	6.50941	6.54863
PRRC2B	6.42491	6.20022	6.44151	6.26187	6.49748	6.08624
PRRC2C	8.16697	8.20307	7.95274	8.05634	7.66458	7.41849
PRRG1	2.55515	2.43803	3.72186	3.96932	2.16462	2.2475
PRRG4	1.57708	1.93378	1.7995	2.37097	2.18833	2.31011
PRRT2	1.40759	1.31462	0.731696	0.631933	0.645121	-0.173396
PRRT3	1.08111	1.02103	1.64306	1.66599	1.29902	1.32675
PRRX1	5.33123	5.13194	-1.86109	-1.15432	6.04898	6.44398
PRSS12	3.03054	3.13521	-0.628724	-0.380732	5.5543	5.45818
PRSS23	6.33973	6.25279	8.04946	8.45552	7.64057	7.69147
PRSS35	-0.943927	-2.37465	2.29627	3.4948	6.62117	6.87895
PRSS53	1.15427	0.86232	1.65008	1.72268	0.887853	-0.167275
PRTFDC1	3.75145	3.6594	3.12239	3.34153	-0.0903144	0.702755
PRTG	4.47829	4.09748	4.13049	3.71503	3.21915	2.5173
PRUNE	3.75213	3.76116	3.35176	3.69852	3.56971	3.79113
PRUNE2	4.83474	4.25911	0.82777	1.06115	3.64233	4.00581
PRX	-0.94393	-0.790185	1.51	1.6394	-0.393347	-0.0918635
PSAP	8.84157	8.7144	8.2218	8.41143	9.87939	9.84989
PSAT1	4.03429	3.38375	4.07482	4.08434	5.06567	4.15583
PSD3	5.90593	5.81093	6.80994	6.56641	6.68709	6.80109
PSD4	0.529821	1.23764	0.699196	2.40689	5.93026	6.63784
PSEN1	5.35076	5.38107	4.5863	4.8033	5.47698	5.46445
PSEN2	3.44558	3.66644	2.80517	3.31954	3.25855	3.76699
PSENEN	3.46792	3.60145	4.08507	4.49215	4.15248	4.83423
PSG4	-1.94365	-2.76144	2.2131	2.93778	-2.14756	-2.25798
PSIP1	5.8437	5.82849	5.32096	5.38463	6.69365	6.3537
PSKH1	3.31355	2.88589	3.97635	4.07471	4.29239	3.65083
PSMA1	5.96578	6.04678	6.41394	6.78617	6.03224	6.36407
PSMA2	6.04746	6.13849	6.26898	6.79108	6.07543	6.83255
PSMA2P3	1.39468	1.73596	1.75392	2.40034	1.7059	1.8084
PSMA3	5.14446	5.30615	4.54778	5.09879	4.94192	5.89318
PSMA3-AS1	4.44526	4.43504	4.04889	3.81747	4.53321	4.07743
PSMA4	5.97862	6.13123	5.93151	6.50149	6.33913	7.30502

PSMA5	5.54555	5.67639	5.51605	6.10911	5.7147	6.4544
PSMA6	5.25364	5.35422	4.8177	5.32498	5.62586	6.66529
PSMA6P1	2.9929	3.23514	2.84476	3.34772	3.31586	4.39552
PSMA7	6.96974	7.01016	6.22666	6.69773	6.84462	7.44562
PSMB1	5.5473	5.58052	5.83812	6.26267	6.64179	7.14896
PSMB10	1.62016	1.73726	3.65223	4.19986	3.50755	4.61685
PSMB2	5.90965	5.94596	5.78297	6.23794	6.13409	6.13485
PSMB3	5.73451	5.82859	5.46392	6.05104	6.10394	6.66235
PSMB4	7.36205	7.43019	6.61735	6.99278	6.92297	7.29645
PSMB5	5.99222	6.03073	4.98515	5.48549	6.2334	6.53226
PSMB6	4.77108	4.98423	5.43942	5.95958	5.72795	6.04859
PSMB7	5.61833	5.68022	5.1773	5.82634	5.73462	6.19677
PSMB8	3.48326	3.64338	4.69806	5.21704	4.52683	5.3627
PSMB8-AS1	0.742756	0.816338	2.47024	2.71496	1.89626	1.79552
PSMB9	2.09536	2.40135	3.70527	4.1224	1.14064	2.37012
PSMC1	3.73528	3.79451	2.96127	3.35222	3.63817	3.9426
PSMC1P1	6.1844	6.22275	5.34137	5.75259	6.05188	6.23526
PSMC2	6.01391	6.15175	5.76823	6.3428	6.21338	6.79948
PSMC3	5.998	5.9489	5.90887	6.30187	6.45892	6.63174
PSMC3IP	2.52881	2.6565	2.5241	2.98449	3.4255	3.34055
PSMC4	6.10382	6.24364	5.23274	5.85516	6.50522	6.63198
PSMC5	5.54075	5.63656	5.33737	5.83238	6.53085	6.61653
PSMC6	4.80146	4.95917	4.26982	4.61307	4.84748	5.29346
PSMD1	6.71474	6.87342	7.14532	7.52129	6.98263	7.15338
PSMD10	4.21529	4.26166	4.00259	4.39895	4.65199	4.94971
PSMD10P1	1.04244	0.813344	1.19157	0.810106	0.670686	0.151378
PSMD11	6.47512	6.54763	6.33515	6.61126	6.76748	6.66896
PSMD12	5.65784	5.83236	5.85822	6.20261	6.8558	6.99598
PSMD12P	0.762077	1.16571	1.17137	1.21386	2.05339	1.9488
PSMD13	6.28218	6.35288	6.19173	6.56096	6.02487	6.50243
PSMD14	6.14052	6.18977	5.94317	6.30504	6.57747	6.97031
PSMD2	6.84477	6.98393	7.35374	7.80529	7.78142	7.76127
PSMD3	5.66453	5.74386	6.27539	6.62292	6.42002	6.33081
PSMD4	6.12278	6.19176	5.77586	6.2014	6.13067	6.40534
PSMD5	4.24269	4.26421	2.7909	3.17889	4.70546	5.01864
PSMD5-AS1	3.66614	3.587	2.02595	1.94648	3.41382	2.88575
PSMD6	6.0669	6.14938	5.62267	5.8691	5.76561	5.97643
PSMD6-AS2	2.05464	1.94541	2.53714	2.16334	1.3909	0.435746
PSMD7	5.9294	5.96852	5.92957	6.3183	5.80619	6.02578
PSMD8	5.68929	5.73079	5.46798	5.96869	6.5398	7.02951
PSMD8P1	0.116631	0.365995	0.045429	0.646135	1.32661	1.35679
PSMD9	4.18509	4.06905	3.1661	3.62165	4.02661	4.31536
PSME1	4.63127	4.79747	4.19043	4.62418	4.88754	5.27769
PSME2	4.33201	4.58151	4.5146	5.10866	4.83804	5.37815
PSME2P2	2.40891	2.72786	2.88988	3.31163	2.59035	3.09161
PSME3	5.45321	5.3344	4.92412	5.16961	6.03183	6.14597
PSME4	6.6128	6.55953	6.24013	6.25475	6.79226	6.88382
PSMF1	4.64688	4.84379	5.34248	5.77587	4.712	4.87166
PSMG1	3.36566	3.55396	3.6462	4.29205	3.92183	4.49172
PSMG2	3.23144	3.44694	3.27189	3.75848	3.78911	4.33098
PSMG3	4.18965	4.16924	3.5478	3.97465	3.98534	4.40573
PSMG3-AS1	-1.27248	-2.1403	1.96116	2.08595	-0.987612	-1.89883

PSMG4	3.46982	3.43226	3.48814	3.46155	3.90008	3.84781
PSPC1	5.40156	5.4817	4.61414	4.71055	4.74749	4.80306
PSPH	3.40158	3.31688	3.58299	3.84676	4.34596	4.23286
PSPN	0.416559	0.151976	-0.0798272	0.259977	0.720068	0.463298
PSRC1	4.24563	4.20248	3.85403	4.14502	4.38953	4.37544
PSTK	0.952035	0.894767	0.64945	0.430424	2.28515	2.68131
PTAR1	6.84587	6.86328	5.45309	5.77113	7.06449	7.19772
PTBP1	6.27083	6.18999	5.67017	5.9928	6.37683	6.29738
PTBP2	4.99744	4.9863	5.08793	4.70735	4.76542	4.41352
PTBP3	5.77891	6.00605	5.44123	5.56797	6.23442	5.91031
PTCD1	3.42148	3.55205	2.82274	3.19659	3.18403	3.10768
PTCD2	3.56537	3.6019	4.19585	4.45341	3.82701	3.96833
PTCD3	6.00135	5.87699	5.55541	5.64974	5.90454	5.71797
PTCH1	6.16938	6.46743	3.47206	3.03921	1.2102	0.958026
PTCHD4	2.69753	2.87701	-0.69145	-0.979274	-1.13645	-1.12145
PTDSS1	5.85856	5.94683	5.88454	6.41352	6.46866	6.57257
PTDSS2	4.05414	3.86358	4.20556	4.19038	3.44166	3.25907
PTEN	5.7244	5.39362	5.89827	6.00951	5.5348	5.22632
PTENP1	3.40339	3.15157	3.31626	3.46813	2.7505	2.96861
PTER	2.44304	2.19181	?	-6.09892	-4.65668	-4.70296
PTGER4	-1.05494	-0.508457	-0.138983	0.139221	1.77435	1.28202
PTGES	2.09859	2.25142	-2.37543	-1.30113	-3.6585	-1.89883
PTGES2	4.12905	4.06658	3.21522	3.5278	3.95715	4.34041
PTGES3	5.82824	5.88663	6.35232	6.65424	6.89996	7.16271
PTGES3P1	3.43409	3.46674	3.91564	4.18984	4.437	4.28079
PTGES3P3	1.05965	0.766476	1.54926	1.57865	1.77405	2.06527
PTGFR	-0.569597	-0.58	3.07386	4.02944	3.73523	3.971
PTGFRN	6.76583	7.53325	5.45037	5.96028	5.62941	5.95639
PTGR1	2.18038	2.26849	6.56065	7.31257	2.74058	3.15739
PTGR2	2.77694	2.73507	2.55145	2.68052	2.31964	2.26433
PTGS2	1.59558	1.17383	3.01124	3.42838	0.489522	0.0213376
PTHLH	1.18307	1.81683	-0.0748589	0.383121	5.48823	5.42838
PTK2	5.83886	5.94441	6.02104	6.30267	5.95599	6.471
PTK7	5.29562	4.97698	6.13501	6.11848	5.89258	5.87683
PTMA	8.38857	8.2827	7.89174	8.13303	8.37778	8.19053
PTMAP2	5.70875	5.66942	5.29403	5.39853	5.73199	5.32072
PTMAP5	6.03702	5.91393	5.535	5.79157	6.16921	5.8077
PTMS	4.17359	4.03301	4.08326	4.14973	4.85342	4.31009
PTN	5.39231	5.32873	2.40521	2.48738	6.32122	6.46104
PTOV1	4.09392	3.76687	4.45893	4.51943	4.8643	4.67772
PTOV1-AS1	1.8673	1.68041	2.76474	2.35936	2.51554	2.31717
PTOV1-AS2	-0.0703423	-0.122741	0.307219	0.336432	0.4027	-0.0282633
PTP4A1	6.08818	6.12421	4.89716	4.86474	5.19688	4.83434
PTP4A2	6.62011	6.49204	6.02634	6.24646	7.0378	6.97873
PTP4A2P1	1.77869	1.68891	1.20146	1.32676	2.23474	2.2075
PTPDC1	3.72193	3.72327	2.83607	2.73981	2.78884	2.32673
PTPMT1	3.76545	3.69879	3.89027	4.01742	4.24017	4.28313
PTPN1	4.85874	5.17388	5.2188	5.79329	5.70767	5.99894
PTPN11	7.45974	7.51794	6.39708	6.79477	7.07855	7.12172
PTPN12	5.63627	5.6213	5.13049	5.04282	5.56281	5.74139
PTPN13	5.00091	4.80861	5.42196	5.65562	4.48052	4.47194
PTPN14	6.51971	6.58569	6.85788	7.19245	6.36989	6.2898

PTPN18	3.16356	3.22541	3.02349	3.31534	3.00503	2.8932
PTPN2	3.2381	3.39808	3.24911	3.49175	3.95089	4.1814
PTPN21	2.72879	3.08791	1.69105	1.79748	2.62978	2.91883
PTPN23	2.84975	2.60595	3.04048	3.05609	2.95624	3.17899
PTPN2P1	0.403977	0.296581	-0.736033	-0.284892	0.497382	0.403257
PTPN3	4.78372	5.03949	-6.17309	-5.10387	-6.64581 ?	
PTPN4	4.48393	4.77196	4.02471	4.25859	4.4651	4.61746
PTPN9	4.46646	4.1296	4.18498	4.35161	4.15024	3.72374
PTPRA	5.0248	5.05095	4.91362	5.09777	4.64098	4.78203
PTPRB	-0.569597	0.256552	1.19454	0.924685	5.67239	4.7131
PTPRD	3.52613	3.40444	-6.17309	-6.09892 ?		-5.69922
PTPRE	1.68466	1.69706	3.19369	3.48176	-5.65305	-3.12051
PTPRF	8.23959	7.89856	6.12735	5.87986	5.25442	4.33357
PTPRG	-0.16608	-0.228138	6.59328	6.72935	5.76591	5.54788
PTPRG-AS1	0.55278	0.556287	0.447801	0.352984	-0.875309	-1.19952
PTPRH	2.68463	2.21665	1.59787	1.90242	-1.37455	-1.53638
PTPRJ	5.89621	5.93458	7.01219	7.34208	7.3973	7.12776
PTPRK	5.05578	5.07824	6.44444	6.61733	7.2356	7.31889
PTPRM	2.83426	2.66902	5.44628	5.51184	-6.64581 ?	
PTPRN	-3.69722	-4.45968	3.14066	3.48922	-3.62454	-5.69922
PTPRN2	-2.23898	-1.25306	2.12483	2.8968	-5.65305	-2.53597
PTPRO	-3.5275	-5.45651	-2.5977	-3.30006	1.3621	1.84021
PTPRR	-2.78042	-1.25306	-4.18092	-2.10822	2.76651	1.84021
PTPRS	6.34432	6.06243	5.66509	5.52097	6.25906	5.8941
PTPRU	3.93209	3.58973	2.84991	3.50405	0.969108	1.493
PTPRZ1	5.09167	4.39512	-4.59508	-4.52056	6.50913	6.16941
PTRF	6.27173	6.26995	6.56046	7.11108	7.38689	7.17439
PTRH2	4.83512	4.83551	4.53549	4.75562	5.56811	5.62211
PTRHD1	2.13184	1.76062	0.564846	0.822689	2.43338	2.80884
PTS	2.26275	2.0377	2.76358	3.08462	3.32298	4.26626
PTTG1	5.06408	5.20936	5.50026	6.20439	5.42969	6.23432
PTTG1IP	6.88459	6.70764	7.50422	7.85046	6.40942	6.31426
PTTG2	-0.52896	0.0613753	0.276497	0.89124	0.32848	0.299092
PTTG3P	0.378933	0.880999	1.13108	1.98252	1.12972	1.66661
PTX3	4.70977	4.69954	4.67056	5.5305	0.172692	0.50283
PUF60	5.47085	5.34854	5.14289	5.44239	6.09914	6.21475
PUM1	6.10909	6.00195	5.96969	6.04058	6.25107	5.91715
PUM2	5.91811	5.86354	5.24065	5.35417	6.18264	6.18354
PURA	5.24562	5.47047	5.33463	5.3032	5.0929	5.3198
PURB	6.3043	6.71041	5.89815	6.30599	5.91553	6.32482
PUS1	3.18966	2.97595	1.93544	2.27921	2.57332	2.5317
PUS10	1.6696	1.87357	2.25798	2.29238	2.39163	2.22899
PUS3	3.1759	3.19	2.9713	3.21311	3.32553	3.51487
PUS7	4.78925	4.82948	3.59947	3.96396	4.80526	4.69842
PUS7L	5.09452	5.13985	4.91648	5.04346	4.84201	5.13719
PUSL1	1.52713	1.62789	1.34008	1.87106	2.4967	2.63139
PVR	5.19267	5.23843	4.77064	5.32521	6.04446	6.0101
PVRL1	3.6251	3.43197	3.04048	3.06361	1.31701	1.71958
PVRL2	4.4184	4.06532	5.59154	5.56968	5.11816	4.70791
PVRL3	5.32744	5.48989	5.80443	5.95119	5.70161	5.98981
PVT1	4.13811	4.09288	3.30479	3.50562	3.35102	3.15944
PWAR5	2.87268	2.89251	-6.17309 ?		2.51637	1.51249

PWAR6	1.98622	2.09487	?	?	1.79341	1.06153
PWP1	4.94656	4.99965	4.14178	4.69206	4.56851	4.9043
PWP2	3.19467	3.28733	1.7271	2.07039	1.3635	1.45309
PWWP2A	3.69538	3.6299	4.39012	4.34445	3.9979	3.74037
PXDC1	2.8173	2.81388	2.31619	2.77693	3.71429	4.05702
PXDN	-1.37679	-2.06994	7.42005	7.19178	7.9801	7.18087
PXK	3.56654	3.51586	3.48182	4.00622	3.58287	3.3834
PXMP2	3.00136	2.53999	2.28002	2.12481	2.50209	2.677
PXMP4	1.54889	1.49139	3.3749	3.65935	3.09458	3.06478
PXN	5.78196	5.93	5.02039	5.25598	5.26112	5.31099
PXN-AS1	0.759761	0.383652	0.227224	0.0150408	0.427982	0.37421
PXYLP1	6.63633	6.99263	0.699196	0.924655	-1.65987	-1.24695
PYCARD	-8.51779	-5.70452	2.75441	2.88165	-5.90088	-8.52515
PYCR1	4.64779	4.44172	3.84017	4.18724	4.25251	4.74746
PYCR2	4.12189	3.86806	4.10649	4.23324	4.65879	4.7486
PYCRL	2.05583	1.80865	2.17844	2.70817	1.49454	2.06149
PYGB	9.49228	9.45522	5.39011	6.0036	7.85277	8.2667
PYGL	5.2401	5.38179	2.79662	3.44577	5.43506	5.63868
PYGO1	4.46895	4.29205	4.22161	4.03965	3.89523	3.24166
PYGO2	4.59898	4.56772	3.75899	3.92321	4.65787	4.68022
PYROXD1	3.1969	3.35636	2.71191	2.95681	2.75117	3.28141
PYROXD2	0.885949	0.419895	2.73535	2.92185	2.29681	2.21217
PYURF	3.69824	4.06164	3.04772	3.58117	3.86907	4.7151
QARS	6.23976	6.10439	5.76154	6.10062	6.4768	6.3363
QDPR	2.18968	2.0956	1.84441	2.11519	2.6135	2.86694
QKI	7.01202	7.02638	6.0771	6.21534	8.04449	7.646
QPCTL	3.37775	3.19716	2.94834	3.24432	3.52331	3.62868
QPRT	-4.69535	-4.13839	3.53928	3.35878	-1.41202	-2.53597
QRICH1	5.62959	5.59751	5.18115	5.38602	5.64573	5.52389
QRSL1	2.8284	2.94655	2.82491	3.37298	3.73736	4.03811
QSER1	6.41253	6.09965	5.97541	5.74681	6.15343	5.87588
QSOX1	6.16044	6.19937	5.41406	5.68313	6.2164	6.35903
QSOX2	5.0365	5.08114	4.67834	5.0994	4.35928	4.24019
QTRT1	2.55123	2.37637	1.84441	2.1296	2.60649	2.70269
QTRTD1	4.50915	4.6907	4.26871	4.61824	4.66723	4.73622
R3HCC1	2.99963	3.11081	2.93283	3.48176	3.14382	3.60843
R3HCC1L	3.78474	3.61264	3.96624	4.00491	4.05563	4.03307
R3HDM1	4.4462	4.41597	4.25006	4.22433	4.81794	4.79512
R3HDM2	2.17438	2.1738	3.1111	2.97864	3.07441	2.76903
R3HDM4	2.86126	2.87701	2.57804	2.85408	3.51336	3.80701
RAB10	5.77259	5.7848	5.51386	5.94	6.51288	6.88549
RAB11A	5.40551	5.37398	5.39998	5.69043	5.79501	6.13639
RAB11B	1.7001	1.21668	1.91253	1.56658	1.69932	1.64956
RAB11FIP1P1	1.84476	1.66955	2.86007	2.78671	2.77695	2.47346
RAB11FIP2	3.92431	3.95713	3.85495	4.10649	4.79493	4.51168
RAB11FIP3	3.23903	2.89691	3.30833	3.25531	2.9209	2.42258
RAB11FIP4	3.37907	3.16834	2.13391	2.13437	2.87691	2.5317
RAB11FIP5	3.73138	3.73067	4.15187	4.75146	4.56607	4.64641
RAB12	3.35267	3.37404	4.02796	4.34589	5.18628	4.94409
RAB13	6.08081	5.86202	4.70997	5.06124	4.57241	4.48047
RAB14	5.13301	5.19892	4.72711	5.00556	5.17376	5.29257
RAB15	4.21005	3.98423	2.51693	2.43808	2.08454	1.58794

RAB17	-0.191134	-0.418273	3.55795	3.50036	-1.45047	-2.12114
RAB18	4.58284	4.60392	4.57721	5.07666	5.08911	5.13878
RAB1A	5.32126	5.34801	5.27833	5.68204	5.9142	6.04301
RAB1B	5.52043	5.26527	5.17342	5.49888	5.25102	5.42876
RAB1C	1.10421	1.12237	1.04648	1.15102	0.572511	0.847043
RAB20	0.824548	0.331671	2.18718	2.4799	2.72987	2.18812
RAB21	5.03604	5.11363	4.11339	4.44191	5.11323	4.74758
RAB22A	6.12984	6.2445	5.23221	5.40785	5.72689	5.61746
RAB23	3.0777	2.96098	3.0768	3.04326	3.89803	4.14363
RAB24	3.04724	2.87165	3.46364	3.69018	3.37308	3.11516
RAB26	1.67164	1.67161	1.37111	1.62592	2.60414	2.55539
RAB27A	2.58823	2.28511	4.11756	4.43152	2.74492	2.76903
RAB27B	3.48827	3.7827	3.33808	3.79594	2.52705	3.85362
RAB28	2.94298	2.78983	2.49518	2.60477	3.30546	3.09389
RAB29	4.00309	4.20427	3.51344	4.14974	4.28366	4.62083
RAB2A	4.31842	4.33075	5.09956	5.5305	4.88609	5.03306
RAB2B	2.64744	2.6509	1.80519	2.17659	3.03493	3.29611
RAB30	2.98742	3.09175	4.53843	5.00685	3.61115	3.77307
RAB30-AS1	2.82645	2.81793	3.40332	3.4515	2.53692	1.9868
RAB31	3.81662	3.81211	5.38252	5.99675	6.19551	6.65193
RAB32	1.26675	1.73686	1.69915	3.2176	3.82853	5.57521
RAB33B	1.91197	1.95924	1.98141	2.48364	2.50778	2.41647
RAB34	4.26019	4.1324	4.70445	5.10427	5.30113	5.89551
RAB35	4.30948	4.30946	3.82831	4.1852	4.5534	4.55067
RAB36	1.18361	0.894767	2.32429	2.44193	0.969108	0.633217
RAB38	2.22278	2.52585 ?		-6.09892	-1.80184	-1.24695
RAB39B	0.676033	1.14454	-3.59683	-1.15432	-0.0308197	2.25331
RAB3B	2.30375	2.03303	3.11798	3.81152	4.02793	3.64864
RAB3D	2.99905	2.67472	-0.752046	-0.000171691	-1.61566	-1.66208
RAB3GAP1	4.97687	5.1431	4.81365	5.17714	5.55036	5.7169
RAB3GAP2	5.7365	5.70455	5.01518	5.21496	5.62497	5.6775
RAB3IP	4.07405	4.1882	-0.598354	-0.682344	4.11931	4.08607
RAB40B	1.52496	1.01295	0.64945	0.46112	1.5829	1.18815
RAB40C	1.92665	1.67161	1.5913	1.65276	2.10126	1.84021
RAB42	-3.11289	-5.45651	1.09265	1.8565	2.1464	2.34859
RAB43P1	-0.538627	-0.666455	1.06966	1.14536	0.290967	0.217516
RAB4A	1.99443	2.17017	1.496	1.80409	3.97268	4.71324
RAB4B	2.56906	2.15224	1.38262	1.37587	1.69466	1.34496
RAB5A	4.6579	4.76291	4.05366	4.46291	4.9419	5.15514
RAB5B	4.87123	4.85739	4.77574	5.2727	4.23072	4.65632
RAB5C	5.05501	4.84928	4.98824	5.46136	5.77193	5.93582
RAB6A	5.48631	5.58359	5.02106	5.46307	5.00316	5.03937
RAB6B	2.08496	1.9482	1.49246	1.57002	-1.30419	-1.71477
RAB6C	0.468844	0.399116	0.290165	0.592914	-0.0755306	-0.503988
RAB7A	5.391	5.23411	4.94057	5.27324	5.69859	5.46818
RAB7B	2.14643	2.55676	0.793829	1.49112	-4.33548	-2.8983
RAB8A	4.38208	4.48868	4.27966	4.54846	5.33395	5.21803
RAB8B	5.84756	5.92516	5.5929	5.85695	5.43342	5.51969
RAB9A	1.75628	1.9035	2.50649	2.98394	2.67955	3.03476
RAB9B	1.9303	2.23761	1.34008	0.978694	-0.705893	-2.12114
RABAC1	2.52975	2.07245	2.78227	3.55117	1.83956	2.92265
RABEP1	5.67386	5.62501	5.69454	5.86538	6.10073	6.20877

RABEP2	1.27825	1.00482	0.956086	1.05027	1.71476	1.92267
RABEPK	2.78474	2.94443	2.18718	2.68885	3.15508	3.68345
RABGAP1	4.75066	4.62384	4.97856	5.17659	4.80398	4.93556
RABGAP1L	4.67867	4.69513	3.83607	3.98392	4.32054	4.60503
RABGEF1	4.37008	4.46614	4.17524	4.61371	4.60478	4.89468
RABGGTA	2.53455	2.53717	0.871828	1.16735	2.45864	2.55539
RABGGTB	6.53196	6.53695	6.13417	6.2327	6.8027	6.8305
RABIF	2.69753	2.66387	1.86635	2.24434	2.74909	3.12934
RABL2A	1.73379	1.62473	2.0581	2.2252	2.01528	1.58095
RABL2B	2.07923	1.99443	2.84072	2.99358	2.48262	2.35186
RABL3	2.40946	2.38266	3.21522	3.33577	3.03666	3.12618
RABL6	4.59487	4.34768	4.51284	4.61091	4.92357	4.74419
RAC1	6.10478	6.19178	6.20628	6.59666	6.63898	6.52946
RAC1P2	4.62691	4.62265	4.5948	4.99292	5.0749	5.0105
RAC2	?	-4.87366	3.6462	4.36893	-2.85193	-1.06257
RAC3	2.41728	1.97597	1.48185	1.52057	2.98715	3.40181
RACGAP1	6.17518	6.38846	5.45221	6.05573	5.42177	5.88153
RAD1	4.06637	4.2581	4.60282	5.14514	4.5732	4.94317
RAD17	3.7756	3.59011	4.29265	4.38793	4.52233	4.82698
RAD18	5.21455	5.2153	4.36529	4.61824	4.64802	4.67805
RAD21	7.03839	7.00366	6.79718	6.9922	6.9687	7.11345
RAD23A	4.31956	4.13718	4.17458	4.41868	5.66828	5.44897
RAD23B	6.61556	6.68517	6.01664	6.4147	6.67175	6.50684
RAD23BP1	1.84056	2.07604	1.03831	1.74988	2.02586	1.77166
RAD50	4.97771	4.86836	5.20658	5.3407	5.37608	5.49609
RAD51	2.93936	2.96552	2.13391	2.41866	2.19146	2.09421
RAD51-AS1	0.959191	0.752809	0.179855	0.317536	0.0402612	-0.4586
RAD51AP1	4.0558	3.45154	3.8595	3.70655	3.20233	3.04148
RAD51B	1.68033	1.89033	2.40895	2.35879	1.19772	1.71958
RAD51C	3.77056	3.85344	3.06441	3.40885	4.03578	4.56007
RAD51D	3.49321	3.49135	3.47826	3.70334	3.85342	3.81095
RAD52	2.83122	2.89966	3.08944	3.02362	2.24029	2.00733
RAD54B	3.84699	3.87482	3.92609	4.01745	4.11227	4.39503
RAD54L	3.81619	3.62359	4.11485	4.16392	3.14371	2.75969
RAD54L2	5.66833	5.58119	4.93994	4.95725	5.28731	4.67806
RAD9A	2.56232	2.23152	2.32292	2.39592	1.68051	1.16313
RAE1	5.23719	5.28323	4.37585	4.83588	4.90622	5.05317
RAF1	5.88538	5.85714	5.66005	5.88362	6.2417	6.0712
RAI1	4.39433	4.06599	4.44363	4.21065	3.72361	3.36743
RAI14	5.63548	5.59345	5.65411	6.18148	6.27443	6.38139
RALA	4.24828	4.26336	4.70586	5.10244	4.45529	4.83825
RALB	4.12667	4.34852	3.60111	4.05983	4.01651	4.66831
RALBP1	4.49244	4.62838	4.57621	4.95842	5.81986	6.15234
RALGAPA1	4.02857	4.03556	2.53362	2.62785	3.6416	3.61984
RALGAPA1P	4.71629	4.78356	3.97023	3.86044	4.21975	4.32801
RALGAPA2	5.49475	5.31358	7.10042	7.2317	4.71799	3.86503
RALGAPB	6.70671	6.80042	6.26845	6.38204	6.14644	6.21437
RALGDS	3.19119	2.99249	3.26769	3.47801	2.98535	2.919
RALGPS1	0.800836	0.766044	0.674533	0.773887	0.549226	0.247587
RALGPS2	5.17511	5.01028	2.50299	3.05357	4.7277	4.81193
RALY	5.67107	5.5564	6.014	6.17395	5.39294	5.356
RAMP1	4.53813	4.78448	-3.85951	-3.10759	0.499649	1.67702

RAN	7.70945	7.79569	6.65458	7.26226	7.53942	7.93721
RANBP1	6.23076	6.05705	5.22524	5.53745	5.3699	5.46438
RANBP10	3.01346	2.97596	3.73086	3.85262	3.9754	3.55537
RANBP2	7.95107	8.11477	7.3933	7.6912	7.78184	7.98113
RANBP20P	0.164786	0.0763402	0.00729598	0.511853	0.524846	0.0514974
RANBP3	4.19952	4.1592	4.19477	4.42255	4.91899	4.61408
RANBP3L	-4.37417	-3.13966	6.52705	6.4878	-1.85245	-2.70579
RANBP6	4.84395	4.93962	4.53157	4.94713	5.98264	6.23835
RANBP9	3.89333	3.82143	4.03049	4.25274	4.73984	4.73457
RANGAP1	6.07116	6.09718	5.6117	6.13998	5.92111	5.92175
RANGRF	2.33251	2.1413	2.49627	2.9742	2.93254	3.30722
RANP1	5.289	5.3468	4.2285	4.84231	5.08243	5.46572
RAP1A	4.3295	4.45229	4.06439	4.14265	4.19133	4.14347
RAP1B	4.57636	4.86994	4.33886	4.52811	4.63551	5.03354
RAP1GAP	1.77668	0.980157	0.292311	0.520618	?	-4.70296
RAP1GAP2	5.3953	5.68775	0.893354	0.879925	0.597155	0.752791
RAP1GDS1	4.55062	4.70553	3.79662	4.15064	4.66588	5.04063
RAP2A	6.06784	6.03999	4.84989	5.02559	5.08438	4.98619
RAP2B	3.66505	3.74416	4.14515	4.4515	5.89243	5.78596
RAP2C	3.19271	3.31359	3.8235	4.0023	5.67453	5.34925
RAPGEF1	3.85647	3.6945	3.66039	3.89398	5.21081	4.89134
RAPGEF2	5.02329	4.99555	4.83536	4.9892	5.26263	5.10063
RAPGEF3	-1.1361	-0.75105	1.61437	2.01282	-2.572	-1.38442
RAPGEF4	-0.744656	-1.0363	-1.09576	-1.58485	2.57439	2.78515
RAPGEF5	2.10184	1.96763	-4.59508	-3.52222	-5.07051	-4.11926
RAPGEF6	5.55201	5.66149	5.73217	5.99034	5.80311	5.77429
RAPGEFL1	1.35081	1.24456	0.445928	0.95732	1.27637	0.615294
RAPH1	5.13489	5.05309	4.77842	4.61305	4.54281	4.59452
RARA	2.56439	2.27601	2.35178	2.44137	3.12774	2.81437
RARB	4.50426	4.62329	5.29422	5.3875	5.83657	6.03684
RARG	3.43792	3.20958	3.6651	3.85551	2.06763	1.80902
RARRES1	-0.703358	-0.485378	3.33797	3.56537	-0.572647	-1.61882
RARRES2	?	?	2.71735	2.67909	-2.13612	-2.12114
RARRES3	-2.89067	-2.1403	2.84162	3.36081	-2.26731	-0.706491
RARS	5.64604	5.81475	5.6957	6.23682	6.25916	7.13168
RARS2	4.12348	4.1296	3.24274	3.46672	4.15266	4.84114
RASA1	4.1548	4.21489	5.24819	5.59168	5.01749	5.017
RASA2	3.67488	4.23845	3.77503	4.07852	2.93588	3.34042
RASA3	4.60469	4.42208	3.843	4.16962	3.81238	3.81096
RASA4	0.123997	-0.571049	1.50915	1.55089	1.29149	0.245447
RASAL2	5.3887	5.38733	4.40519	4.57679	5.98755	5.73777
RASGRF2	0.54893	0.656176	1.7707	1.46864	0.381317	-0.280287
RASGRP1	2.42505	2.40445	1.32432	0.350701	1.3208	0.33778
RASL10B	2.2493	1.16656	-3.59683	-5.10387	-1.48999	-4.11926
RASL11A	-2.69815	-3.13966	2.64938	3.1224	-1.90489	-1.24695
RASSF1	3.07368	3.01794	2.30834	2.40629	2.90481	2.68451
RASSF2	0.334442	0.223769	2.27182	2.58118	3.48307	2.9972
RASSF3	3.94748	4.16468	3.81365	4.31952	3.52951	3.68982
RASSF4	6.19342	6.08885	4.40238	4.30161	3.76805	2.97277
RASSF5	0.44057	0.444137	2.70523	2.85118	-0.217212	-0.0627204
RASSF7	1.94841	1.15192	2.37873	2.75227	1.92467	2.21812
RASSF8	6.31093	6.49749	5.15855	5.46624	5.30366	5.35127

RASSF8-AS1	2.73764	2.52585	2.68993	2.48364	1.5829	0.965773
RAVER1	2.33221	1.82487	1.94429	1.89552	2.57037	2.6864
RAVER2	4.28889	4.31441	3.50124	3.57942	3.33262	3.28764
RB1	5.21903	5.44778	4.8249	5.0779	5.76148	5.77255
RB1CC1	6.6199	6.5841	6.3193	6.54692	6.17217	6.61716
RBAK	4.05915	4.01995	5.0891	5.01013	5.26643	5.05209
RBBP4	7.16825	7.06706	6.50487	6.54838	6.98298	6.97802
RBBP4P1	3.84342	3.68595	3.3029	3.37266	3.78729	4.16925
RBBP4P2	2.71896	2.6895	2.69248	2.62984	3.0057	2.81929
RBBP5	4.46019	4.59715	4.26769	4.63766	4.68674	4.78415
RBBP6	5.33124	5.12681	5.71167	5.68801	5.34142	4.96039
RBBP7	5.32319	5.13649	6.22522	6.49838	6.00204	5.92102
RBBP8	4.59439	4.64567	3.9509	4.26946	4.83254	5.33562
RBBP9	4.6623	4.96865	4.19152	4.59594	4.5661	5.16132
RBCK1	3.52733	3.16651	4.76702	5.00034	3.57809	3.76496
RBFA	3.71238	3.88576	3.0013	3.40877	2.46115	2.5925
RBFOX2	6.13617	5.92192	6.64718	6.53524	5.88247	5.54002
RBKS	-0.489437	-0.332268	-0.539469	0.267967	0.427241	1.40185
RBL1	5.22047	5.19884	4.42555	4.61735	4.58283	4.43665
RBL2	4.85575	4.85325	5.43641	5.61358	5.70886	5.71736
RBM10	4.58576	4.44002	4.32558	4.5527	4.11183	3.95822
RBM12	6.69854	6.71019	6.27198	6.36287	6.41371	6.36756
RBM12B	5.63791	5.64775	5.76351	5.74095	5.27443	4.94368
RBM12B-AS1	1.49572	1.38208	1.61716	1.69856	1.2586	0.560157
RBM14	5.08568	5.03619	4.68722	4.96416	4.41886	4.25195
RBM14-RBM4	2.52768	2.33786	2.5808	2.45538	1.85369	1.4239
RBM15	5.6545	5.66412	5.53626	5.6704	5.28535	4.9135
RBM15B	5.98461	5.83362	5.34485	5.38544	5.92823	5.27339
RBM17	4.9441	4.88952	4.4512	4.58515	5.11674	4.7371
RBM17P2	1.74538	1.68265	-0.573276	-1.0885	-0.280936	-1.67031
RBM17P4	0.627261	0.649983	-0.373552	-0.571723	0.553832	-0.209912
RBM18	2.76401	3.036	2.38864	3.05448	3.81016	4.55402
RBM19	4.78543	4.79213	3.90622	4.1809	3.87574	3.73326
RBM20	1.48583	1.49719	3.29221	3.47989	0.597155	0.719622
RBM22	3.88118	4.26507	3.93412	4.39897	4.57508	4.87164
RBM23	4.73721	4.43141	3.98926	4.1214	4.67699	4.54299
RBM24	-1.34118	-1.25306	1.53076	1.71775	3.24658	3.87824
RBM25	6.39861	6.34996	5.8594	5.93989	6.54948	5.84342
RBM26	5.64755	5.71411	5.89082	5.83567	5.59763	5.23921
RBM27	5.3315	5.38825	5.37035	5.58092	5.32204	5.25288
RBM28	5.53011	5.62177	5.04244	5.31251	5.38413	5.0351
RBM3	6.31754	6.4213	5.1052	5.49153	6.16759	6.37641
RBM33	5.49814	5.46455	5.68758	5.59894	5.08703	4.84402
RBM34	3.80422	3.68777	3.44947	3.70229	4.40374	5.05257
RBM38	1.98432	1.76037	1.47278	1.59227	3.5099	3.30405
RBM39	7.69903	7.62356	7.58541	7.53492	7.55521	7.41381
RBM4	5.62322	5.53109	5.50868	5.58782	5.43973	5.56739
RBM41	3.70499	3.61397	3.61738	3.77076	3.55764	3.51729
RBM42	3.36431	3.24452	3.54269	3.901	3.67731	3.79711
RBM43	1.22578	1.02102	1.95605	1.90801	2.52953	2.08772
RBM45	2.55832	2.76839	3.4729	3.74447	2.83155	3.44811
RBM47	-2.05464	-1.65505	2.42382	3.37901	2.04361	2.66833

RBM48	3.68569	3.79219	4.0774	4.19381	3.32695	3.08119
RBM4B	3.52378	3.54013	3.48206	3.44195	3.15398	2.86233
RBM5	6.01481	5.91203	6.07381	6.06722	6.47941	6.01996
RBM5-AS1	1.39138	1.55659	1.69735	1.80613	2.14171	1.25113
RBM6	5.92748	5.87207	5.31442	5.33159	6.3743	5.68918
RBM7	2.42409	2.18162	4.14476	4.25297	4.25203	4.10939
RBM8A	5.68468	5.33999	4.74146	4.72836	6.04079	5.53689
RBM8B	3.71645	3.33417	2.83336	2.76294	3.95693	3.35993
RBMS1	5.35167	5.43799	5.75472	5.95097	5.66924	5.50363
RBMS1P1	2.4077	2.6458	2.46601	3.08271	2.89584	2.94622
RBMS2	3.2035	2.97558	4.46085	4.7995	3.49499	3.27724
RBMS2P1	0.246931	0.335803	1.33675	1.90527	0.615699	0.609689
RBMS3	4.13696	3.91544	3.89995	3.60026	5.55854	5.14112
RBMS3-AS2	0.923035	0.603321	1.22592	0.491176	1.85935	0.650921
RBMX	7.50613	7.48779	7.08788	7.29652	7.45833	7.42444
RBMX2	3.3949	3.36115	3.34874	3.6178	4.13254	4.24639
RBMX2P5	-0.212365	0.170976	-0.261613	0.325997	0.729183	0.867383
RBMXL1	5.686	5.73637	4.82342	4.87021	5.12041	4.99626
RBMXP2	1.97196	2.09276	1.33895	1.67047	1.87748	1.80199
RBMXP4	0.907214	1.11223	0.597946	0.58323	1.28724	0.870132
RBPJ	5.79889	5.70532	5.58977	5.811	5.73721	5.36579
RBPMS	3.89484	3.37004	3.69656	3.51304	1.60651	1.17598
RBSN	4.79727	4.89413	4.26769	4.4245	5.35404	5.09418
RBX1	4.04154	4.15829	3.70978	4.22096	3.98354	4.34041
RC3H1	5.59583	5.44103	5.55075	5.57721	5.37928	4.91441
RC3H2	5.75572	5.87087	5.71658	5.98622	6.59416	6.77458
RCAN1	6.99602	6.6053	4.07975	4.51596	5.79527	5.83485
RCAN3	4.51037	4.22715	3.32018	3.62361	4.44363	4.26775
RCBTB1	3.90269	3.95185	3.99016	4.38501	3.58762	3.63758
RCBTB2	0.243547	0.775632	1.51695	1.85698	1.49455	2.13251
RCC1	5.73791	5.78258	4.00796	4.24413	5.67504	5.58036
RCC2	6.46015	6.34932	6.0641	6.2465	7.30194	6.87635
RCC2P6	1.06751	1.00913	0.671203	0.840967	1.93903	1.1519
RCCD1	3.66833	3.62859	3.63665	3.85963	0.134221	-0.662104
RCE1	3.56847	3.4396	3.27775	3.35823	3.20821	2.76869
RCHY1	2.77801	2.77078	2.25461	2.54166	4.16283	4.09616
RCL1	3.37507	3.459	2.19587	2.58467	4.18125	4.42642
RCN1	6.73126	6.46025	7.66845	7.99114	7.14807	7.00494
RCN1P2	5.128	4.93983	5.89254	6.12585	5.48645	5.39418
RCN2	6.36012	6.21773	5.2088	5.52098	5.59296	5.96349
RCN3	3.94838	3.70081	0.986462	0.902467	-5.65305	-4.70296
RCOR1	4.33503	4.35972	3.77357	3.57942	4.35719	3.58791
RCOR3	4.05078	4.04693	4.09608	3.94509	4.22691	3.93714
RDH10	4.85834	5.21735	3.73949	4.1347	3.80158	3.53993
RDH10-AS1	0.817271	0.807462	0.374299	0.394295	-0.624626	-0.976228
RDH11	6.50709	6.55114	5.70528	6.05258	6.36927	6.30615
RDH13	1.45069	1.51053	0.345975	0.617552	2.5266	2.23912
RDH14	3.28391	3.3267	2.30835	2.5494	3.24808	3.80306
RDX	6.25731	6.2878	6.49423	6.53413	7.32142	7.0327
REC8	0.133932	-0.158917	0.488988	0.399062	1.17894	0.293394
RECK	1.90457	1.95924	2.89062	3.22657	4.1235	4.39525
RECQL	5.58137	5.72708	5.24045	5.76637	5.07719	5.72305

RECQL4	4.05865	3.77283	4.09477	4.12711	3.48639	3.28889
RECQL5	3.05414	2.92086	3.74722	3.72406	3.50585	3.03813
REEP2	2.53934	2.52869	0.0645242	0.383121	2.30837	1.80111
REEP3	5.8318	5.83062	5.4818	5.79429	5.68036	5.22401
REEP4	3.1417	3.04891	3.32229	3.52054	2.46124	2.10066
REEP5	5.31535	5.84481	5.68769	6.42454	6.06678	7.0605
REL	4.67929	4.67774	3.84122	3.77129	3.69695	3.17337
RELA	4.39235	4.39357	3.67446	3.91215	3.86129	3.93353
RELB	3.10506	3.17921	0.914572	1.55655	0.871134	0.922708
RELL1	2.97577	2.98056	2.7835	3.11759	2.6422	2.33775
RELL2	1.42949	1.21761	2.48377	2.58798	2.1259	2.14753
RELT	3.99266	3.90674	3.02105	3.23103	3.22691	3.17593
REPIN1	4.83812	4.62661	6.0256	6.26296	5.65736	5.39
REPS1	3.72844	3.78328	3.79265	3.86747	4.16778	3.99036
REPS2	-0.52896	0.181128	2.29627	2.91354	-2.20023	-0.247123
RER1	5.46259	5.42753	5.55296	5.93475	6.5399	6.85287
RERE	3.93754	3.74416	4.27076	3.88834	4.69372	4.4481
REST	5.50945	5.51978	5.50777	5.46906	6.18242	5.80896
RETSAT	4.06661	3.98423	4.19569	4.66101	4.25481	4.39909
REV1	4.86418	4.95345	4.59713	4.49717	4.92624	4.93846
REV3L	5.54666	5.48922	5.52744	5.49969	5.82046	5.85855
REXO1	2.21529	1.70287	1.87112	1.95908	2.92827	2.37264
REXO2	2.93574	2.84255	4.40612	4.82854	4.13151	4.39394
REXO4	3.4918	3.38398	3.02554	3.43797	4.03378	3.89841
RFC1	5.37539	5.44855	5.19151	5.50264	6.29932	6.46726
RFC2	4.41009	4.41444	4.06796	4.55385	4.9119	5.05233
RFC3	5.56067	5.50835	4.59782	4.79127	4.97042	4.87636
RFC4	4.27101	4.33805	4.24063	4.56888	4.75279	4.95327
RFC5	5.26417	5.25638	3.72574	4.16072	4.4082	4.82759
RFFL	4.32456	4.33166	4.57498	4.8444	4.73541	4.60076
RFK	3.84201	3.92301	2.25944	2.78297	4.19612	4.18202
RFNG	2.92774	2.57321	2.68154	2.69353	3.85761	3.49752
RFT1	4.17624	4.1792	4.21522	4.56888	4.08452	4.26482
RFTN1	3.77361	3.9122	4.33807	4.53231	0.458718	0.541297
RFTN2	1.83233	1.3251	-0.659745	-0.938639	0.159978	-0.619042
RFWD2	4.68838	4.7545	4.33012	4.54567	4.37515	4.97364
RFWD3	6.08741	6.07654	5.53373	5.86737	5.04142	4.92354
RFX1	0.409538	0.265185	0.986462	0.632724	0.437814	0.719622
RFX2	1.49539	1.50874	1.89865	2.10036	1.69286	1.03481
RFX3	3.81169	3.93589	2.70827	2.29207	4.77795	4.72268
RFX5	5.61237	5.52158	4.56467	4.93965	5.23414	5.38205
RFX7	4.98478	4.89743	4.72784	4.54132	4.84052	4.67914
RFXANK	3.3513	3.01625	3.16305	3.24727	3.24897	3.57151
RFXAP	1.20183	1.09179	1.77651	1.35065	1.27637	0.50283
RGL1	4.15813	4.19178	4.81151	4.84318	4.4397	4.53407
RGL2	3.51443	3.39558	3.53562	3.60616	4.2526	4.52415
RGL3	0.776727	0.395227	0.73541	0.57776	0.69732	-0.151977
RGL4	0.536888	0.597374	0.579943	0.327239	-0.536373	-1.08738
RGMA	-0.74582	-1.24705	-2.01303	-1.30113	2.99945	2.5173
RGMB	5.3136	5.37846	3.4177	3.61519	6.05497	5.97524
RGMB-AS1	2.05536	2.23852	-0.999455	-0.693159	2.03129	1.65736
RGP1	4.85981	4.66707	4.58382	4.89749	6.10767	5.87003

RGPD3	0.590444	0.4456	-0.146011	0.0100637	0.242889	-0.115894
RGPD5	5.22533	5.16847	4.43856	4.13508	5.14637	4.91914
RGPD6	5.41198	5.2373	4.26669	4.45044	5.21153	4.73979
RGPD8	4.76495	4.5911	3.11716	2.86144	4.40777	4.30283
RGS10	1.54413	1.46201	1.7707	1.87419	0.954457	1.60621
RGS12	2.79278	2.64569	3.52728	3.34445	2.98715	2.80504
RGS14	0.159022	-0.122853	2.07389	2.36692	2.86913	3.41741
RGS16	3.99962	3.82602	-5.1783	-4.10635	-5.65305	-3.70484
RGS17	4.14471	4.16924	3.66979	3.88623	3.02269	2.79314
RGS19	2.35078	2.30866	1.46038	1.97866	1.89813	2.70269
RGS2	1.13386	0.894767	4.20341	3.17426	3.16941	3.5076
RGS20	2.08558	2.12214	1.42385	1.77384	0.86332	0.878317
RGS3	2.78071	2.19539	3.8235	4.44192	3.84251	4.31425
RGS4	-0.94393	0.456112	-5.1783	-6.09892	5.79782	6.92671
RGS5	1.74803	1.9295	1.84993	1.7241	1.84354	2.25331
RGS7	0.266817	-0.253233	-3.37468	-4.52056	1.43249	0.847941
RHBDD1	1.49078	1.23764	1.46038	1.65939	3.62046	3.38074
RHBDD2	3.8507	3.72079	4.12253	4.43517	3.93308	4.11983
RHBDD3	2.61154	2.42895	3.14341	3.17889	2.19146	1.92993
RHBDF1	3.18509	2.95079	3.73831	3.85984	2.44561	1.80111
RHBDF2	0.792839	0.920918	2.94319	3.35469	-0.450674	-0.799586
RHBDL2	2.20001	2.58121	1.16299	1.20195	-1.71976	-2.12114
RHBDL3	-1.8912	-2.00285	1.98141	2.26188	-2.65941	-2.8983
RHEB	4.56268	4.58502	4.34526	4.76506	5.51519	5.90058
RHEBL1	1.36705	1.54842	0.102	0.445854	-0.490197	-0.497069
RHEBP1	3.88193	3.79766	3.55567	4.06305	4.67128	5.41451
RHEBP2	2.45275	2.94418	2.18866	2.91259	3.49805	3.23722
RHNO1	4.03985	4.48407	3.89862	4.53502	3.50461	3.81685
RHOA	7.21978	7.15104	6.88307	7.18106	7.6284	7.66629
RHOB	3.60356	3.22541	2.53759	3.03584	7.19006	7.39049
RHOBTB1	2.17438	2.15922	2.66198	2.39501	4.09874	3.908
RHOBTB2	3.3901	3.49796	3.20389	3.45281	2.73291	2.84624
RHOBTB3	5.17588	4.9887	6.73594	7.0786	5.73067	5.76981
RHOC	6.77934	6.54023	5.77827	6.15384	7.21907	7.18062
RHOF	2.12549	2.08076	-0.737733	-0.54701	-1.04237	-1.05508
RHOG	3.21081	2.91221	2.65884	3.07357	3.52334	3.33221
RHOJ	4.47145	4.5678	-5.1783 ?		3.9754	3.77911
RHOQ	5.47876	5.34749	4.43447	4.66624	5.45507	5.62054
RHOQP1	1.48229	1.32608	-0.69519	-0.765692	0.184725	-0.22789
RHOQP2	1.30453	1.30354	0.25021	0.093479	1.45716	1.38312
RHOQP3	1.29276	1.18616	-0.0953502	0.555247	1.2	1.71934
RHOT1	4.32121	4.38227	3.90063	4.12908	4.60725	4.87438
RHOT1P1	-0.22289	-0.169477	-0.15956	0.147945	0.333893	0.53833
RHOT2	4.19952	3.80394	4.13049	4.16613	4.25183	4.21661
RHOU	2.29246	2.32833	-2.27595	-2.10822	-0.705893	-0.214706
RHPN1	1.43024	1.17383	-1.48269	-1.30113	0.416602	0.0483059
RHPN2	3.22102	3.22866	2.98123	3.22621	4.57431	4.42501
RIC1	4.89248	4.82824	5.04394	5.07945	6.12258	6.02044
RIC8A	5.79627	5.8602	5.87886	6.28375	5.78106	5.98938
RIC8B	4.19102	4.2201	3.41998	3.64083	4.23751	4.33754
RICTOR	5.28205	5.35789	6.07281	6.10784	5.41428	5.68122
RIF1	7.70897	7.76618	7.15977	7.27519	7.52317	7.66027

RILPL1	1.57599	1.45864	1.8513	1.57118	2.08163	1.26907
RILPL2	2.55596	2.23761	2.91617	3.19952	1.61119	1.46325
RIMBP3	-0.514596	-0.729289	0.968762	1.16125	-1.50594	-1.53035
RIMKLA	2.61381	2.46791	-2.09546	-2.30078	1.75757	0.293394
RIMKLB	-1.37679	-1.37502	4.42422	4.3302	2.69442	2.19114
RIMKLBP2	-0.026521	-0.122851	1.06446	1.1104	0.121175	-0.752286
RIMS3	1.33439	1.08411	1.13846	1.26626	0.871134	0.769095
RIN1	2.76617	2.71707	2.00632	2.53143	0.679613	1.33772
RIN2	3.43405	3.47525	4.74573	5.37449	4.55705	5.13798
RIN3	2.26672	2.04497	0.120378	0.57776	1.5246	1.67702
RING1	3.09209	3.20605	2.14741	2.46862	3.95715	4.13091
RINT1	4.40484	4.50267	4.07811	4.39736	4.74953	5.16436
RIOK1	3.61267	3.58693	2.3478	2.71117	4.0845	4.20765
RIOK2	3.60811	3.9216	4.05873	4.67857	3.90294	4.72127
RIOK3	4.61266	4.73682	4.20557	4.73116	4.71255	4.82563
RIPK1	3.82447	4.034	3.97761	4.37297	4.76386	5.00665
RIPK2	3.60812	3.57057	2.25944	2.72723	3.22539	3.62867
RIPK4	2.04241	2.06004	2.11571	2.46861	-5.07051	-3.52416
RIT1	4.27174	4.06071	4.51431	4.77762	5.15227	5.32463
RITA1	3.02715	2.95291	2.47827	2.89681	3.17889	3.21513
RLF	5.23239	5.22408	5.16187	5.36993	5.96082	5.97363
RLIM	6.49695	6.68394	6.46621	6.89416	7.20644	7.28933
RMDN1	3.98005	4.03376	4.14527	4.20185	4.58422	5.06832
RMDN2	2.44814	2.73684	0.120378	0.46112	1.2881	2.67267
RMDN3	4.18586	4.09652	3.48003	3.7288	3.58405	3.55539
RMI1	3.00136	2.9187	3.60602	3.60884	2.5805	2.36476
RMI2	2.96271	2.74418	2.88795	2.93283	0.969108	0.68568
RMND1	2.84202	2.9013	2.08796	2.47615	3.62971	3.95861
RMND5A	5.65047	5.85059	4.07385	4.64436	5.35158	5.43151
RMND5B	3.92156	3.86804	3.30068	3.34778	4.40904	4.26043
RMRP	11.2675	11.1141	11.2484	11.521	11.3558	12.0937
RN7SK	13.6472	13.3683	13.114	13.3388	13.2563	14.4542
RN7SKP162	1.2317	1.5759	0.558032	1.23995	1.58765	2.27058
RN7SKP176	2.14911	2.07443	1.74414	2.15134	1.61483	3.40163
RN7SKP180	-0.445832	-0.161839	-0.0550636	0.0722568	-0.955189	0.914679
RN7SKP203	5.72855	5.74327	5.53354	5.8551	5.31797	6.72825
RN7SKP255	3.75081	3.82722	3.6668	3.94749	3.61615	4.64468
RN7SKP71	5.83816	6.00095	5.69557	5.96455	5.63259	6.56786
RN7SKP80	5.71243	5.87519	5.31447	5.77749	5.49897	6.60345
RN7SKP9	4.09799	4.3232	3.60479	4.20416	4.52289	4.89774
RN7SKP90	-0.233552	0.15156	-0.57245	-0.0676833	-0.135304	1.15718
RN7SKP91	1.76038	1.55956	0.976408	1.65276	2.19769	2.22404
RN7SKP97	-1.08408	-1.2146	1.2174	1.14862	-0.594016	-1.18284
RN7SL1	14.342	14.1584	13.5182	13.9583	14.746	15.822
RN7SL128P	0.431345	1.04541	0.609642	0.906398	0.825753	1.59016
RN7SL396P	0.273456	0.638825	0.417877	0.465933	0.840451	1.58156
RN7SL4P	7.71352	8.05887	7.65946	7.90616	8.27759	8.69622
RN7SL5P	7.04343	7.44384	6.80193	7.18637	7.50909	8.48928
RN7SL674P	1.85429	2.07453	1.62847	1.94915	2.27799	3.29729
RN7SL767P	-0.033385	0.81078	-0.242767	-0.0284929	0.563685	1.46409
RNA5-8S5	9.29747	8.34173	8.79215	8.16054	8.71187	7.81189
RNASEH1	3.95549	3.91575	2.89718	3.43644	4.41647	4.57949

RNASEH1-AS1	1.87459	1.73194	0.355738	0.736735	1.15992	1.68564
RNASEH1P2	0.763148	0.825973	0.34864	0.700262	1.47148	1.30052
RNASEH2A	3.98743	4.02899	3.44947	3.83076	3.97993	4.2848
RNASEH2B	3.56301	3.57469	2.28002	2.375	3.3298	4.09418
RNASEH2C	4.56836	4.61859	4.30701	4.65602	4.22824	4.54062
RNASEK	4.01652	3.76202	3.71291	4.01451	4.86673	4.96045
RNASEL	2.80077	2.90567	2.73832	3.11518	2.0227	2.14505
RNASET2	0.367103	0.432061	1.01407	1.34506	2.212	2.30453
RND3	5.5485	5.56884	3.64777	4.65104	5.83702	6.86265
RNF10	4.70976	4.52228	4.53243	4.63768	5.23375	5.03475
RNF103	3.72078	3.69023	3.65926	3.66625	3.91025	3.75167
RNF11	5.07652	5.15966	5.16409	5.67579	5.78261	5.82369
RNF111	4.22498	4.20337	4.33217	4.36892	4.35579	4.02799
RNF113A	1.277	1.44523	2.28596	2.7312	3.27151	3.44887
RNF114	5.40845	5.40325	5.30982	5.84535	5.66919	5.63645
RNF115	4.58692	4.7398	4.95218	5.16941	5.4352	5.4493
RNF121	4.48825	4.61062	4.08208	4.22879	3.81745	3.69629
RNF123	3.95714	3.82614	3.43174	3.72634	4.44721	4.18994
RNF125	3.41206	3.43803	-4.59508	-4.10635	?	-5.69922
RNF126	1.99791	1.7171	1.41643	1.76157	3.10291	3.08676
RNF128	4.5201	4.85907	?	-5.10387	-6.64581	-5.69922
RNF13	4.40493	4.47045	4.82593	4.98677	4.47863	4.77816
RNF130	4.35279	4.17803	4.52194	4.61823	4.9566	4.97876
RNF135	2.17438	2.04497	2.46755	2.82488	1.77435	2.2062
RNF138	4.1763	4.29493	3.5989	3.74146	3.20169	3.05403
RNF138P1	0.714303	0.742153	2.24423	1.85082	0.0868381	0.507607
RNF139	4.10424	4.2937	3.64779	4.02688	4.92276	5.55184
RNF139-AS1	1.78476	1.89913	1.73537	1.79207	1.29974	0.97985
RNF14	4.38299	4.44762	4.35461	4.68628	4.96578	5.47189
RNF141	3.32744	3.19873	4.28179	4.40292	3.51811	3.59699
RNF144A	4.59496	4.32668	1.25116	0.761609	0.597155	0.578775
RNF144B	1.36705	1.23069	-3.18222	-2.64851	1.7868	2.0681
RNF145	6.49839	6.408	5.96127	6.40211	6.79275	6.94451
RNF146	3.14954	3.35255	3.53244	3.61568	3.89331	4.05483
RNF149	4.38107	4.74172	2.87177	3.31744	3.80729	3.87637
RNF150	4.92159	4.86358	1.61741	0.591699	4.94747	4.4031
RNF152	2.51038	2.66128	5.23379	4.88198	-1.65987	-2.70579
RNF157	3.84133	3.80281	0.385365	0.383121	4.17109	3.88899
RNF166	1.72721	1.41984	2.13842	2.36692	1.52955	1.15132
RNF167	4.18907	4.10654	4.05876	4.24435	5.16257	5.3475
RNF168	5.08266	5.03748	5.21573	5.35214	6.37958	5.90935
RNF169	6.24739	6.27419	5.49352	5.64675	5.14467	4.94672
RNF170	2.95913	3.05483	3.31832	3.28778	2.63432	2.87825
RNF175	-5.69162	-3.13966	2.02107	2.23992	0.0677351	-0.121607
RNF181	3.66833	3.75925	3.63572	4.20502	4.25781	4.97627
RNF182	3.77968	3.86246	-1.65952	-0.823182	4.90809	4.82465
RNF185	3.89527	3.91785	3.62532	3.93116	3.61775	4.07437
RNF187	4.64298	4.68626	4.44034	4.72959	4.75116	4.84787
RNF19A	5.25627	5.49713	4.52325	4.83697	5.51213	5.35843
RNF19B	2.84976	3.07243	2.20451	2.6225	3.30547	3.60389
RNF2	3.60812	3.8836	2.8193	3.16497	3.88078	4.27912
RNF20	5.33982	5.46047	4.867	5.23601	5.05608	5.04482

RNF213	7.72891	7.60915	8.08007	8.09908	7.83203	7.59266
RNF214	3.33958	3.27312	3.26483	3.37103	3.32002	3.11323
RNF215	3.57148	3.52325	3.34473	3.33304	2.75562	2.74472
RNF216	5.37223	5.27641	5.71388	5.80272	5.58926	5.28625
RNF216P1	3.20396	3.08641	3.75472	3.68812	3.45612	3.18446
RNF217	4.54093	4.31978	3.96588	4.18738	6.4583	5.81325
RNF219	4.20288	4.3612	3.83187	4.21441	3.3966	4.05761
RNF220	4.42975	4.17429	4.15279	4.41802	4.95975	4.71129
RNF24	4.3121	4.3386	6.74148	6.96602	5.01385	4.5712
RNF25	3.09739	3.2798	2.85284	3.53147	3.73094	4.06513
RNF26	4.30934	3.92409	4.59043	4.72722	3.87884	3.60389
RNF31	3.549	3.25949	3.05306	3.17986	3.89407	3.7623
RNF32	0.870345	0.883793	1.16142	0.942301	0.0959109	-0.478501
RNF34	4.53667	4.66777	3.82991	4.09396	4.54368	4.46974
RNF38	3.69644	3.4575	3.93541	3.92323	4.96858	4.62531
RNF4	5.16696	5.16196	4.75768	5.2413	5.99436	5.92091
RNF40	5.65863	5.62654	5.46711	5.8262	5.69282	5.38425
RNF41	4.65405	4.66514	3.43487	3.82928	4.61696	4.71429
RNF44	1.91565	1.74664	2.40521	2.49852	2.69945	2.56008
RNF5	3.0811	3.14017	2.63102	3.09001	4.33097	4.78142
RNF5P1	0.656094	1.03174	0.646494	0.982313	2.1481	2.48656
RNF6	4.42292	4.69499	4.71355	5.34641	4.96308	5.31425
RNF7	3.72642	3.70031	3.93454	4.24438	4.65974	5.13425
RNF8	3.93009	3.9233	3.25527	3.28134	3.93579	3.96571
RNFT1	3.26393	3.18992	2.88199	2.87369	3.71578	4.32117
RNFT2	3.584	3.81444	1.88797	2.17196	3.46897	2.97277
RNGTT	5.13382	5.15278	4.05846	4.22655	5.12637	5.07054
RNH1	4.91884	4.78567	5.16464	5.47189	4.68398	5.25183
RNMT	4.51158	4.44704	4.36143	4.43999	5.48594	5.48742
RNMTL1	2.78729	2.8148	2.68618	3.06185	3.364	3.42606
RNPC3	3.40145	3.38083	3.86531	3.81217	3.66749	3.5493
RNPEP	4.04872	4.08427	3.68695	4.19142	4.56911	4.82038
RNPEPL1	3.28819	2.68691	3.20018	2.99446	3.56368	3.70903
RNPS1	5.25993	5.29932	5.02043	5.16813	5.47049	5.22613
RNPS1P1	2.80884	2.73521	2.26774	2.53264	2.92289	2.96594
RNU1-1	6.6125	6.4312	6.52812	6.3927	6.57926	6.85869
RNU1-2	6.89745	6.85432	6.99507	6.91019	6.64332	6.9632
RNU1-27P	6.7597	6.89972	7.14611	6.95	6.7922	6.96023
RNU1-28P	6.66044	6.46355	6.52046	6.43593	6.61293	6.93439
RNU1-3	6.7543	6.52954	6.48938	6.31367	6.62629	7.03233
RNU1-4	6.84748	6.99164	6.99877	6.99843	6.64332	7.07237
RNU11	1.46083	1.44408	0.871828	0.89124	1.01217	1.17598
RNU12	3.74385	3.53574	3.57636	3.48736	2.8554	3.18203
RNU2-1	5.49874	5.50761	4.88793	4.95119	5.44791	6.35161
RNU2-2P	8.57217	8.46433	8.3861	8.34322	7.85194	8.47698
RNU4-1	5.42147	5.31111	4.74647	4.44959	4.50081	4.65083
RNU4-2	5.76443	5.77734	5.45716	5.43999	5.08452	5.71429
RNU5A-1	0.9009	1.09179	1.0357	1.12005	0.740643	1.1007
RNU5A-8P	0.81669	0.592522	-0.183372	-0.086319	0.539452	0.401903
RNU5E-1	1.44562	1.67161	1.77651	1.45345	-1.57243	-1.24695
RNU6ATAC	1.01523	0.955067	-0.161006	0.0811214	0.661695	0.752791
RNVU1-18	6.82767	6.59075	6.41731	6.45998	6.57099	6.91852

RNVU1-7	6.66035	6.43124	6.44993	6.34825	6.52407	6.80359
RNY1	3.71561	3.78508	1.0357	1.57771	3.84152	3.5003
ROBO1	7.07133	6.8449	6.55435	6.41668	6.41701	6.34109
ROBO2	1.26675	1.31856	-4.59508	-6.09892	?	?
ROBO3	2.36163	2.15923	2.60765	2.87417	-1.23372	-0.799586
ROCK1	5.5126	5.60907	5.31083	5.53176	5.52007	5.72175
ROCK2	7.13447	6.8499	6.11853	6.1809	6.44163	6.08851
ROGDI	1.37778	1.21668	2.13842	2.23549	1.57813	1.1007
ROM1	-0.00343481	-0.288791	2.00194	2.13309	1.17637	2.16667
ROMO1	4.24636	4.14818	3.55288	3.9826	4.26968	5.29961
ROR1	1.37242	1.18106	2.78803	3.01789	-0.00196773	-0.799584
RORA	0.452342	0.736899	2.02206	1.8512	0.273731	0.100762
RORB	0.219896	0.514521	-6.17309	-5.10387	1.29974	1.88577
ROS1	-0.654472	-1.00314	-3.01245	-3.71618	1.35654	1.36138
RP1-101D8.1	-1.74441	-1.93875	1.27595	1.38307	?	?
RP1-102E24.8	0.832372	0.581631	0.860939	0.786061	-2.75246	-3.38329
RP1-102K2.8	1.64112	2.00199	-2.41335	-3.47367	-3.62902	-3.34884
RP1-117O3.2	0.551767	0.492263	-0.109062	-0.0247734	0.0734775	-0.899103
RP1-121G13.3	-0.388639	0.111499	-0.028025	0.179244	0.737955	0.83733
RP1-136J15.5	-0.15637	0.0959109	0.0900811	0.538528	0.971869	0.834501
RP1-140K8.5	-0.0407343	0.635262	-0.659745	0.231137	1.23456	1.32676
RP1-145M24.1	1.03569	1.12967	-0.276471	0.414828	1.04711	1.53173
RP1-151F17.2	-0.0407343	0.0291348	0.976408	0.845438	0.147163	-0.0627204
RP1-152L7.5	-6.68418	-4.87366	3.13389	3.20179	2.09125	1.80111
RP1-159A19.3	2.38273	2.42323	0.734586	1.40168	2.82721	3.08498
RP1-159M24.1	3.14712	3.23472	1.69341	2.16411	2.84542	2.67091
RP1-179N16.3	0.399051	0.656176	0.636738	0.798133	0.339787	0.0747657
RP1-193H18.2	-0.144345	-0.00328298	1.04535	0.761609	1.19772	1.40185
RP1-203P18.1	0.406918	-0.120274	0.240949	0.407026	-0.41573	-0.174478
RP1-228H13.5	0.727258	1.60328	-0.428455	0.100749	0.270529	0.100762
RP1-232L22__B.1	0.382036	0.195688	1.98174	2.52139	0.511134	1.19147
RP1-239B22.5	2.21682	1.79457	-1.86109	-1.10853	1.1471	-0.00614162
RP1-241P17.4	2.84628	2.78688	2.6744	3.0173	4.03478	4.48183
RP1-253P7.4	0.50068	0.181128	0.904002	0.659431	1.66165	0.50283
RP1-261D10.1	0.271999	0.537236	0.681017	0.871	1.27292	1.58154
RP1-267D11.6	0.61206	0.666511	0.510982	0.625981	-0.0163223	-0.151977
RP1-273G13.2	1.29777	1.52319	0.830718	1.17704	1.14473	1.88351
RP1-278E11.3	3.79225	3.92024	3.36484	3.9162	4.56903	5.11921
RP1-283E3.4	0.4795	0.449905	1.52445	1.64698	1.37645	0.293382
RP1-283E3.8	2.3665	2.19998	2.7776	2.73848	3.07983	1.87967
RP1-292B18.1	1.87579	1.61954	0.881359	1.03591	2.32117	2.98368
RP1-305B16.3	0.301026	0.382745	-0.757028	-0.131402	0.518585	0.33778
RP1-39G22.7	1.86319	1.8681	1.44587	1.74918	1.08121	1.30456
RP1-68D18.2	-0.323873	-0.233871	1.01621	0.786061	0.871134	0.224139
RP1-68D18.4	-0.791952	-0.437121	0.625766	0.506155	0.0280033	-0.214706
RP1-78B3.1	2.98041	2.87701	3.17842	2.65937	1.32842	0.401903
RP1-79C4.4	1.17748	1.13712	-4.59508	-5.10387	1.33409	0.719622
RP1-89D4.1	1.96271	2.33642	1.68223	2.03877	2.96996	3.24907
RP1-95L4.4	-0.4048	0.0133408	0.0718862	0.384514	0.822543	1.10407
RP11-100N21.1	0.783172	0.803078	-0.0442914	0.15716	1.73964	2.13244
RP11-1012A1.7	0.345453	0.686872	-0.369766	-0.0297	1.4182	1.22672
RP11-1017G21.4	0.318739	0.278044	-0.0240632	0.32307	-0.165266	0.146525

RP11-1018J11.1	2.21393	2.28877	2.50245	2.92648	2.64748	2.90732
RP11-101E13.5	2.5727	2.6988	1.82685	1.98956	2.60628	1.89383
RP11-1020A11.2	1.08887	0.859151	1.37876	1.56363	1.51964	1.11352
RP11-1023L17.1	2.97627	3.11032	3.46811	3.07426	2.85071	2.80592
RP11-1023L17.2	2.37484	2.21978	3.00076	2.484	2.01748	1.01785
RP11-1024P17.1	-0.026521	-0.177293	0.488988	0.120113	-0.217212	0.578775
RP11-102M11.1	2.25328	2.28711	1.97562	2.16581	2.32165	2.46817
RP11-104N10.2	3.70819	3.57744	4.6206	4.53141	3.58168	2.47321
RP11-1069G10.1	0.769492	1.02308	-0.455418	-0.491178	0.951536	-0.00614162
RP11-106M3.3	2.11665	2.07103	2.18312	2.148	1.96445	1.6116
RP11-1070N10.5	2.05583	2.16288	-1.86109	-1.40802	-6.64581	-5.69922
RP11-107E5.3	3.14641	2.86134	0.308431	0.0811214	1.93963	1.96574
RP11-1094M14.11	1.8708	1.85911	1.37876	1.71138	2.21315	1.87827
RP11-1094M14.7	1.20429	1.31466	0.88509	1.35031	1.46231	1.821
RP11-1099M24.6	1.35351	1.45246	2.68783	2.42411	2.02627	1.71719
RP11-10K16.1	-0.222385	-0.195452	0.571444	0.822738	-1.13645	-0.31423
RP11-10N23.2	0.0692347	0.166625	0.571444	0.786061	0.0540566	-0.706491
RP11-10O17.1	0.104578	-0.525355	0.806728	0.769823	-0.792894	-1.47217
RP11-1100L3.7	2.5201	2.73181	0.527531	1.28366	1.41936	0.426565
RP11-110I1.12	0.0824028	0.357428	1.0357	0.82198	-0.878698	-1.70626
RP11-110J1.2	2.76329	3.1417	2.29186	2.64109	4.03354	4.18827
RP11-1110F20.1	1.67599	1.57061	-1.86109	-3.10759	-1.23372	-1.79933
RP11-1112J20.2	0.969521	1.07008	0.331545	0.763429	1.05494	0.826437
RP11-1114A5.4	0.031494	-0.346758	0.593449	0.469	0.785132	0.225707
RP11-111F5.2	0.572744	0.337677	-2.33716	-2.01929	0.62813	0.989873
RP11-111F5.4	1.21178	1.22823	-0.712341	-0.955449	1.21739	1.19795
RP11-111F5.5	0.0225596	0.366588	-1.97744	-1.64507	0.900475	1.2537
RP11-111M22.2	2.46332	2.73929	2.90659	2.81896	0.871134	0.560157
RP11-111M22.3	1.17133	1.08411	0.945825	0.82198	0.258664	0.33778
RP11-112J1.1	4.63564	4.64426	3.84478	4.21244	5.18914	6.2243
RP11-1148O4.1	0.382546	0.454376	0.432072	0.716894	0.811668	0.974683
RP11-114F3.2	1.57599	1.55557	1.04958	1.44969	1.12921	1.57065
RP11-114H7.1	2.30375	2.45532	1.61093	2.09577	3.15564	3.05823
RP11-114M5.1	0.563754	0.817312	0.54309	0.285473	0.441802	-0.577229
RP11-1151B14.4	-2.25047	-3.12884	3.91745	3.96671	3.05835	2.51808
RP11-115C21.2	0.640852	0.305445	0.355738	0.300663	0.384182	-0.121607
RP11-115J23.1	-4.37417	-1.46246	-2.48229	-1.93836	1.88661	2.50275
RP11-118D22.3	1.21144	1.36909	0.401532	0.805177	1.02038	0.752791
RP11-119F7.5	0.0152977	0.122235	0.292311	0.0612232	2.72987	1.30456
RP11-11N9.4	1.77262	1.65093	-3.37468	-6.09892	-0.250761	0.702755
RP11-120B7.1	-0.113985	-0.0726466	-0.0236246	0.245824	1.48047	2.19583
RP11-1212A22.1	1.24495	0.868663	1.30996	1.3973	0.900413	0.206406
RP11-1212A22.4	1.33195	1.38829	2.475	2.63856	1.04595	0.371581
RP11-1217F2.1	-0.244757	-0.40643	1.15068	1.18297	0.391537	0.731574
RP11-121C2.2	2.1713	2.39515	1.62387	1.45345	2.31412	2.2002
RP11-1246C19.1	0.159022	0.122235	0.544485	0.28359	0.32848	0.0213376
RP11-124N14.3	3.6804	3.93202	2.41509	2.91995	4.77313	5.00836
RP11-1280N14.3	0.845309	0.860653	1.59708	0.907306	1.52123	0.652014
RP11-128A6.3	-0.129081	0.537236	0.324373	0.89124	0.670686	-0.348991
RP11-12G12.7	0.312247	0.804012	0.976408	1.1104	1.2101	1.27061
RP11-12M9.3	2.32125	2.39381	1.7629	1.91487	2.87455	3.6539
RP11-1319K7.1	1.11417	1.25575	2.03086	1.80724	1.04049	1.76917

RP11-133N21.7	0.952035	0.840999	0.610974	0.659431	1.35654	1.03481
RP11-134E15.2	1.80832	1.75664	1.35001	1.45068	1.79808	2.39808
RP11-134K13.2	2.22543	2.92161	1.49311	2.78402	2.29628	3.03159
RP11-134L10.1	1.72491	1.35434	1.23723	1.21568	0.966873	0.628419
RP11-135F9.3	0.586404	0.548466	0.544485	0.968047	0.568596	0.769095
RP11-135N5.3	1.06916	1.00482	2.0405	2.08102	1.81545	0.80115
RP11-137J7.3	0.854012	0.880623	0.1513	0.381715	1.50546	1.85229
RP11-137N23.1	1.36654	1.54184	0.957142	1.13319	1.38209	1.95969
RP11-138A9.1	3.28534	3.05286	3.02593	3.1858	2.3397	2.15129
RP11-138A9.2	4.13854	3.94123	4.09724	4.17541	3.38681	2.80505
RP11-1399P15.1	0.207918	0.166625	0.904002	1.12005	-0.10558	-1.79933
RP11-1415C14.3	0.19502	-0.929951	-0.000639256	0.461413	0.815657	0.853445
RP11-1415C14.4	1.83054	2.02478	2.52699	2.42903	1.98066	2.07492
RP11-141B14.1	0.301026	0.656176	1.08332	1.15801	-0.450674	-0.38461
RP11-141M1.3	2.05249	1.80864	-0.861345	-1.20162	-0.660096	-1.12145
RP11-142E9.1	3.59554	3.55535	2.46035	2.44193	2.93588	2.00067
RP11-142L4.2	1.29939	1.67116	0.734022	1.44811	1.26702	1.94034
RP11-142L4.3	2.88638	3.16242	2.17683	2.88948	2.94086	3.51885
RP11-142O6.1	0.776727	0.537236	0.871828	0.724135	0.688494	-0.38461
RP11-144I2.1	1.00834	1.3251	0.73541	0.505972	-0.530833	-0.95156
RP11-144L1.8	0.655654	0.670278	0.0257942	0.148388	1.17977	1.00738
RP11-146N23.1	1.57024	1.70793	1.12497	1.61814	3.32685	3.64096
RP11-147L13.11	2.77294	2.98295	4.32651	4.59498	4.70557	4.71246
RP11-147L13.13	0.0298698	0.641842	1.7096	1.73259	1.75648	0.725785
RP11-147L13.15	-0.159773	-0.177293	0.355738	0.736735	0.317072	-0.121607
RP11-148K1.12	0.407581	0.49503	0.101516	0.576861	0.94152	0.529271
RP11-152C15.1	1.34981	1.42589	1.11933	1.23105	1.35236	2.10087
RP11-152N13.16	1.11663	1.33703	0.901209	1.02901	1.29328	0.697783
RP11-153M3.1	4.14325	4.02064	3.36947	3.65727	4.8483	4.77721
RP11-154J22.1	1.35601	1.11167	0.525317	0.313478	-0.299452	-1.00615
RP11-156E6.1	3.40814	3.4928	3.37298	3.66266	4.15266	3.6856
RP11-156P1.3	1.39795	1.16097	1.97391	2.0008	2.15711	1.73617
RP11-157K17.5	0.0692347	0.251507	0.966283	0.549472	0.0946955	-1.31405
RP11-157P1.4	0.849631	0.703827	-0.285618	-0.149916	0.804458	0.964835
RP11-158H5.7	1.69844	1.82258	1.06446	1.35882	-0.136604	-0.151977
RP11-158K1.3	1.25895	1.59224	0.714865	0.935792	1.89321	1.04787
RP11-158L12.4	1.00142	0.746683	0.882627	0.367002	0.282309	-0.247123
RP11-159D12.2	4.64369	4.80094	2.54781	2.7455	6.08192	5.32355
RP11-159D12.5	0.366521	0.343885	-13.054	-2.19718	1.72108	0.687733
RP11-159D12.6	1.52206	1.05497	0.816993	0.714962	1.2612	0.669254
RP11-159D12.8	2.77463	2.95712	2.81366	2.87986	2.87885	2.40704
RP11-159G9.5	1.28965	1.11464	1.70525	1.67911	1.12765	1.52214
RP11-159J3.1	-1.87859	-0.904524	2.05615	2.81484	3.44931	3.57952
RP11-15A1.3	-0.100139	-0.673205	-0.189953	-0.802935	1.65582	0.659285
RP11-15E18.5	-0.159773	0.358453	-0.723865	-0.154483	0.652647	1.13883
RP11-15H20.6	1.26097	1.12967	1.98141	1.74918	1.40582	0.785207
RP11-160E2.6	2.63051	2.18707	5.19899	5.09065	4.77078	5.66395
RP11-160O5.1	-0.840854	-0.734698	0.723445	1.15801	-1.53062	-0.121607
RP11-161H23.10	0.337586	0.166625	0.871828	0.266325	-0.302594	-0.899103
RP11-161H23.5	4.64021	4.77893	3.89622	4.28185	5.08712	5.09608
RP11-161I2.1	0.743937	0.645757	2.50299	1.82784	1.16629	0.937201
RP11-163E9.2	0.206781	0.0623428	0.203076	0.504386	-0.22112	-0.538691

RP11-166B2.3	0.399051	0.503023	0.138526	0.632724	0.107996	-0.183001
RP11-166D19.1	4.83376	4.22916	4.6976	4.97219	4.42249	4.34584
RP11-166P13.4	0.81669	0.896419	-0.47862	-0.823182	0.0831657	-0.95156
RP11-168J18.6	1.46684	1.75234	0.921132	1.15872	1.75194	2.12854
RP11-169F17.1	3.78674	4.1792	-5.1783	-6.09892	?	?
RP11-169K16.8	2.1026	1.79225	1.29451	1.74691	2.6617	2.69351
RP11-16C1.2	0.684702	0.645757	1.69915	1.35065	1.49454	0.463298
RP11-16E18.3	0.0152977	0.223769	0.386425	0.266325	-0.10558	-0.619042
RP11-16E23.4	1.36145	1.41733	1.67669	1.54029	1.55932	1.7118
RP11-16P6.1	1.26675	1.27867	1.46757	1.59857	1.57335	1.11352
RP11-170M17.2	2.77425	2.71415	2.56536	2.78577	3.30139	3.78901
RP11-170N16.3	0.00148521	0.305445	0.623914	0.57776	0.0677351	-0.38461
RP11-172F4.2	0.385398	0.34087	0.575361	0.615058	0.931902	1.50916
RP11-173E2.1	0.323716	0.465504	0.360926	0.65127	1.40936	1.29654
RP11-174G6.5	-0.95653	-0.576908	0.600346	0.571386	0.14325	-0.337454
RP11-175B9.2	0.169104	0.00445105	0.340141	0.0624671	0.121705	-0.120912
RP11-175B9.3	5.37412	5.33216	4.55305	4.85711	5.52175	6.30345
RP11-175O19.4	1.71034	1.80864	1.88797	2.11036	3.17574	2.88201
RP11-177G23.1	-0.632781	-0.253233	0.0260635	-0.353769	0.976371	0.293394
RP11-177H13.2	3.50292	3.63113	3.74087	3.67591	2.83723	2.36426
RP11-178C3.2	0.0193461	0.171822	0.123481	0.00424969	0.548338	0.594749
RP11-178H8.7	1.70183	1.70177	2.03357	2.25201	1.85478	1.92983
RP11-180C16.1	3.09805	3.24869	2.33944	2.4337	1.7319	1.19959
RP11-180M15.7	0.631914	0.697098	0.474784	0.57776	0.549226	1.08775
RP11-181C21.4	2.59211	2.58277	2.55371	3.04045	3.09603	3.59903
RP11-181E10.3	0.312247	0.223769	1.37111	1.08105	1.14064	0.247587
RP11-182N22.9	-0.379654	-0.0848585	0.329629	0.244936	0.664182	-0.247895
RP11-185E8.2	0.847885	0.775632	-3.18222	-1.93836	0.134221	0.650921
RP11-186B7.4	4.04633	4.0999	4.00401	4.6718	4.39018	4.73733
RP11-186N15.3	0.750529	0.456333	-0.558143	-0.606037	0.474981	-0.18313
RP11-187C18.3	0.750203	0.952378	-0.036844	-0.407419	2.43582	2.69596
RP11-188C12.2	0.694096	0.834307	1.32267	1.03644	0.244717	0.0920586
RP11-18B3.2	1.28767	1.41848	1.01028	1.50939	1.45367	1.71313
RP11-191L9.4	0.966312	1.29209	1.2174	1.25754	-4.65668	-5.69922
RP11-192H23.7	1.21983	1.13205	0.498925	0.793446	1.90736	1.02768
RP11-196G11.5	0.71885	0.794611	0.935482	0.798133	1.89046	0.443129
RP11-196G18.22	2.53734	2.73191	3.22967	3.37095	1.74034	1.43288
RP11-196G18.23	1.04244	1.01295	1.88259	1.72409	0.270529	1.0747
RP11-196I18.3	0.792606	0.948235	1.06846	1.12508	-1.42828	-1.07756
RP11-197N18.2	2.96181	2.84857	2.60677	2.99082	2.67996	2.65511
RP11-197N18.8	1.9303	1.67674	1.20884	1.0909	0.282309	-0.0627204
RP11-198M15.1	1.52239	1.46851	1.2469	1.5044	2.93969	3.53048
RP11-199F11.2	0.490837	0.988077	-0.300902	0.154078	0.57806	0.0747657
RP11-1H8.5	1.4671	1.49785	-1.67714	-1.32716	-0.63036	-0.775461
RP11-203B9.4	1.131	1.21518	1.53089	1.39068	1.82288	1.23844
RP11-204C16.4	2.23568	2.51445	2.54436	2.82995	2.3221	2.25081
RP11-206F17.2	2.11779	2.02679	2.83887	2.96401	3.3121	3.29724
RP11-206L10.2	0.525217	0.457405	0.602856	0.45133	0.481165	-0.152938
RP11-206L10.3	2.50217	2.41986	1.23462	1.18	0.894247	0.126629
RP11-206L10.9	1.11426	1.15052	0.799427	0.869627	0.596086	0.701469
RP11-209M4.1	0.00506937	0.334053	0.230387	0.370733	-0.119778	-0.414128
RP11-20B24.4	2.25425	2.22314	2.06505	2.13742	2.42538	2.24265

RP11-20I23.8	1.52061	1.40398	2.1357	2.01574	1.31517	0.382446
RP11-20O24.4	6.45376	6.33471	5.22529	5.77575	5.66096	6.65537
RP11-212I21.3	1.71045	1.9592	2.47827	2.54422	0.157716	-1.27836
RP11-212P7.1	1.19305	0.977851	0.986076	1.19969	2.16177	2.81831
RP11-212P7.2	2.11151	2.13911	2.17844	2.36692	2.23149	1.30042
RP11-212P7.3	1.2389	1.18256	0.858602	0.930283	1.58384	1.98279
RP11-214O1.2	?	-6.45019	1.13846	0.605504	2.46382	1.86317
RP11-215A21.2	1.53104	1.49186	0.323093	1.01781	0.786697	1.85521
RP11-216L13.19	0.531149	0.600945	0.791431	0.562142	0.297426	-0.224445
RP11-216M21.1	-0.395813	-0.655276	-1.37581	-1.46458	1.61585	1.61525
RP11-216N14.7	1.21983	1.44408	1.11115	0.535117	0.362161	-0.214706
RP11-219G10.1	-0.32867	-0.0533395	-0.663909	-0.281365	0.265101	0.745874
RP11-21K12.2	0.577131	0.357428	0.544485	0.520618	-0.0308197	-0.421131
RP11-21L23.2	1.7188	1.47383	2.24695	2.04601	-2.75246	-2.8983
RP11-220D10.1	0.708558	1.11619	-0.0214924	0.663554	2.19769	2.44678
RP11-223P11.3	0.415045	0.409755	0.56431	0.224151	-0.166613	-0.0926494
RP11-226L15.5	0.915689	0.831845	0.340141	0.367002	0.529601	-0.619042
RP11-226M10.3	0.61388	0.736899	0.191626	-0.380732	1.71476	0.68568
RP11-227G15.12	1.89528	1.9434	1.8045	2.00851	1.54653	0.644327
RP11-229D13.3	1.46083	1.39518	1.32432	0.989263	1.02625	-0.214706
RP11-229O3.1	0.206431	-0.669765	1.95685	1.40748	1.84602	1.47288
RP11-22B23.1	2.6134	2.52177	2.37896	2.1075	1.56533	0.609273
RP11-22H5.2	2.45575	2.81561	0.571444	0.367002	-1.57243	-1.89883
RP11-230F18.5	0.832372	0.756391	1.41643	1.4458	0.362161	-0.183001
RP11-231C14.4	1.71953	1.70021	2.31148	2.10273	1.66826	1.00398
RP11-231I16.1	1.06659	0.999495	1.40711	1.0449	0.230941	-0.400391
RP11-234A1.1	6.36209	6.30197	5.76509	6.31352	7.45519	8.40832
RP11-234B24.6	0.644142	1.13415	0.877776	1.78591	0.868165	0.857567
RP11-234N17.1	1.10373	1.31129	0.50854	1.32801	1.43985	2.12034
RP11-23J9.5	-6.68418	?	0.69635	0.884348	-0.198197	0.196959
RP11-240G22.5	0.857264	1.00482	0.324373	0.418147	-0.702262	-0.752286
RP11-242D8.1	0.649726	0.804012	1.00636	0.95732	-0.320293	-0.706491
RP11-244F12.3	3.79726	3.85954	2.82914	2.98441	1.72406	1.67742
RP11-244H3.4	1.17294	1.13553	1.09481	1.6095	1.90098	1.96205
RP11-249L21.4	-0.175597	0.168385	-0.147405	0.34593	0.33714	0.929988
RP11-24B13.2	1.43293	1.64614	-1.07804	-1.14589	-0.144696	-1.29623
RP11-251G23.2	-0.590352	-0.185202	0.425169	0.958828	0.270529	0.100803
RP11-251G23.5	-0.0124477	0.166625	-0.161006	0.248838	-0.0308197	0.126299
RP11-252A24.2	3.10171	3.20024	3.35545	3.32382	3.101	2.18634
RP11-252I13.1	0.593918	0.721898	0.799319	1.13264	0.75399	0.250792
RP11-253E3.3	2.49569	2.33807	0.793829	1.06115	0.416602	0.200291
RP11-254B13.1	1.58835	1.88906	0.841901	1.37753	1.99477	2.02959
RP11-255B23.1	1.00577	0.793746	0.8729	0.626187	1.22975	0.459558
RP11-257O5.2	1.82451	1.88591	1.61093	1.71775	1.5246	1.0747
RP11-258C19.7	1.85937	1.7171	1.36341	1.2921	1.10793	0.224139
RP11-259K5.2	1.4468	1.22268	0.549876	0.799908	0.72188	0.639343
RP11-259N19.1	0.649726	0.223769	0.355738	0.761609	1.33973	1.20023
RP11-260M2.1	0.200843	0.458812	0.524545	0.164439	0.644558	-0.0329731
RP11-261C10.5	0.674831	0.650002	0.326721	-0.0801947	-0.230037	-0.692947
RP11-262H14.3	0.965936	1.25473	-0.502869	-0.2132	-4.60883	-3.82041
RP11-262H14.4	1.30245	1.02201	-0.677147	-0.407182	0.796315	1.21704
RP11-263K19.4	0.0578453	0.171053	-0.0594904	0.0866072	1.94573	0.745177

RP11-264B17.3	0.326652	-0.0814672	0.569442	0.252028	-0.0488942	-1.41682
RP11-267M23.1	2.13699	2.1445	2.64304	2.38304	3.06931	1.62424
RP11-270C12.3	2.84669	2.97805	2.85481	3.16574	2.54657	3.76278
RP11-272L13.4	-0.207073	-0.354151	0.64945	0.476226	0.234612	-0.4586
RP11-274B21.14	2.12166	1.80678	2.47672	2.26703	2.26287	1.48727
RP11-274B21.2	1.84514	1.41139	2.05919	1.77634	2.03669	0.574828
RP11-274E7.2	2.62099	2.8812	2.93036	3.6051	2.4396	3.12058
RP11-274H2.3	-0.569597	-0.508457	1.37876	1.41477	1.37315	0.293394
RP11-274H2.5	0.878411	0.90354	2.70523	2.58467	2.68621	1.53173
RP11-278C7.1	3.1007	3.22873	2.66358	2.88617	3.13855	3.33224
RP11-278C7.5	0.476879	0.894007	0.0567222	0.236903	-0.552789	-2.60521
RP11-27I1.4	0.824548	0.813344	0.662041	0.82198	1.45867	1.2122
RP11-281O15.7	-0.123819	0.27422	-1.10134	-0.323834	0.802632	0.284775
RP11-282K24.3	2.57938	2.72451	3.27181	2.98129	-2.572	?
RP11-282O18.3	2.70465	2.59226	0.832128	1.03337	2.1463	0.92998
RP11-283I3.6	1.8934	1.90787	1.16526	1.32585	1.8195	1.18815
RP11-284F21.10	0.367103	0.357428	1.86635	1.63267	1.83555	1.42261
RP11-284F21.9	-1.74441	-1.25306	1.55122	1.27492	0.606556	0.126299
RP11-286H14.4	2.88128	2.66299	2.1418	2.56607	3.90275	3.60874
RP11-286N22.8	2.23497	2.0519	1.95862	2.2552	2.14378	2.53398
RP11-288C17.1	2.29896	2.33246	-0.455418	0.178848	0.428357	1.03481
RP11-288C18.1	1.82451	1.90787	1.26773	1.10068	1.68845	1.43288
RP11-288E14.2	1.45744	1.51996	1.00161	1.19758	1.57604	1.84385
RP11-288G3.4	-0.223192	0.123017	0.82777	1.28354	0.597155	0.560157
RP11-288H12.3	1.36579	1.045	1.93544	1.99448	2.22235	1.35942
RP11-288K12.1	1.31777	1.15192	1.48894	1.13916	0.606556	-0.280287
RP11-289H16.1	1.59189	1.37787	0.676042	0.374889	-0.557075	-0.762747
RP11-289I10.2	1.53819	1.60056	1.79153	2.26738	2.10382	2.13339
RP11-290L1.4	-0.0745444	0.0290358	0.332255	0.0121252	0.257011	-0.398979
RP11-294J22.7	1.55834	1.66131	1.71737	1.38307	1.20393	0.100762
RP11-295G20.2	1.74803	1.5651	-1.01332	-0.823182	0.706084	0.668401
RP11-295H24.3	1.85778	2.05521	1.0059	1.3287	2.065	2.05655
RP11-295P9.3	4.40096	4.44268	2.91696	2.7243	3.39767	2.95268
RP11-295P9.8	1.77694	2.12513	-1.57375	-1.66686	-1.33939	-2.08409
RP11-296E7.1	3.81117	3.73405	3.15552	3.43801	3.75729	3.55667
RP11-296P7.4	1.21781	1.48583	1.26018	1.88555	1.85542	1.64205
RP11-297L17.6	3.23073	3.41888	2.67682	3.07359	3.31388	3.51576
RP11-298C3.2	4.66107	4.48296	3.7929	3.88669	3.80681	3.92407
RP11-298J20.4	0.687473	0.371213	-0.184974	0.84835	0.644253	0.711178
RP11-299J3.8	1.75494	2.01142	2.16718	2.37052	2.28883	2.26831
RP11-29G8.3	2.44834	2.44692	1.87698	1.8029	1.6499	1.03157
RP11-2B6.2	2.06891	2.05644	2.26169	1.62741	1.90345	1.87852
RP11-2C24.3	0.278223	-5.05E-05	0.690024	0.438122	-0.224953	-0.308287
RP11-2C24.9	2.9104	3.03429	3.184	3.39241	3.09296	2.29016
RP11-301G21.1	0.480172	0.54587	-0.183372	0.476226	1.04134	1.35093
RP11-303E16.2	1.63187	1.9252	1.5647	2.20407	1.46901	1.96574
RP11-303E16.9	0.307778	0.395162	0.831123	0.47282	-0.853519	-2.23652
RP11-304F15.5	1.17868	1.24589	0.821359	1.17087	1.90661	2.03478
RP11-305B6.1	0.991993	1.43766	1.18467	1.78428	1.47987	1.38698
RP11-305M3.2	0.644604	0.758098	0.441367	0.739131	1.14881	1.65939
RP11-305O6.3	1.30659	1.7708	-0.183372	-0.177939	0.234612	-0.662104
RP11-307L3.2	1.29751	1.41474	1.57086	1.99549	2.24687	2.88751

RP11-307P22.1	2.0148	2.11323	1.22403	1.55834	2.31603	3.2132
RP11-308D16.2	-0.289526	-0.105152	0.747284	0.659431	-0.802092	-0.752286
RP11-30L15.4	0.743937	0.581631	0.945825	0.736735	-0.217212	-0.497071
RP11-30P6.1	0.688485	0.872033	1.16038	1.56009	1.92697	1.49039
RP11-312J18.5	4.21966	4.92031	3.51163	4.60076	4.16833	5.24658
RP11-313J2.1	4.71073	4.75761	0.242852	-0.158637	-5.06184	-5.66393
RP11-314A20.1	1.02845	0.812794	0.236009	0.572123	1.72393	2.95795
RP11-314B1.2	?	-4.13839	3.78081	3.73352	-3.6585	-3.38329
RP11-317N8.4	0.104914	0.305492	0.302723	0.282155	0.240791	0.0634339
RP11-320A16.1	0.961786	1.13699	0.886995	1.37338	1.59366	2.3843
RP11-321A17.3	1.35667	1.25884	0.208904	0.82198	1.78711	2.05486
RP11-321N4.5	0.556199	0.575932	-0.798642	-0.849703	0.123044	0.0372557
RP11-323I15.2	-0.0550876	-0.139207	0.163846	0.555384	-0.0816932	0.0213376
RP11-325E14.5	0.146525	0.370644	0.723934	0.999755	0.384182	0.33778
RP11-325K4.2	-1.00311	-1.38768	0.515561	0.871102	0.182336	0.225497
RP11-325P15.1	0.561702	0.550792	-0.348888	-0.257999	0.467165	0.324223
RP11-325P15.2	0.465421	1.00213	-0.0833124	0.216871	0.484128	0.967309
RP11-326A19.5	1.48595	1.40967	1.8361	2.33915	3.02968	3.32503
RP11-326I11.3	0.701877	0.265185	1.14744	1.33416	0.159978	0.270672
RP11-328C8.2	0.351413	0.417369	0.717684	0.853597	1.02613	0.508662
RP11-328C8.4	-1.36396	-1.89035	-1.44651	-2.24735	1.89854	1.55973
RP11-328J2.1	2.52734	2.52585	?	?	-6.64581	-4.70296
RP11-329A14.1	2.91203	2.63238	1.46804	1.60928	2.75506	3.11985
RP11-32B5.1	2.16715	2.20712	1.53762	1.8862	3.0825	2.72008
RP11-32B5.7	-0.224794	0.0562506	?	?	0.7149	0.0437225
RP11-331F4.5	1.61437	1.47863	2.46564	2.30151	1.01401	0.425116
RP11-332H14.2	2.41557	2.48554	2.45819	2.45994	1.988	1.50278
RP11-332M2.1	0.735617	0.581631	0.340141	0.445854	1.08121	0.50283
RP11-332P22.2	-0.525621	-0.373963	-0.686345	-0.0403337	1.44504	0.836183
RP11-333E13.2	2.22724	1.99854	2.44836	2.44384	2.83703	2.80278
RP11-333E13.4	-0.697001	-1.17487	0.21004	0.736735	0.041271	0.225559
RP11-334A14.2	1.60372	1.59597	1.94518	2.48315	2.96212	3.25753
RP11-334C17.5	1.39176	1.37811	2.0526	1.99795	0.69315	0.0747657
RP11-334E6.12	-4.41728	-6.80804	3.83892	4.06172	2.83983	2.70122
RP11-334L9.1	2.39582	2.42817	1.96469	2.38681	2.02109	1.24528
RP11-335F8.2	0.623342	0.86105	0.571444	0.968047	1.31761	1.11399
RP11-336K24.12	0.71885	0.559609	0.308431	0.89124	-0.705893	-1.12145
RP11-337C18.4	-2.52469	-2.6546	1.21851	2.05663	-2.48959	-2.53171
RP11-337C18.8	1.13083	0.733259	1.01326	0.906852	0.832404	0.807256
RP11-338I21.1	0.0289651	-0.0525343	0.474784	0.857026	-0.285108	-0.247123
RP11-33B1.1	2.28672	2.23417	4.96718	4.11353	1.91192	1.68771
RP11-340I6.6	1.75646	1.61476	1.81935	2.02799	2.12498	2.41416
RP11-341D18.7	2.8223	2.45884	2.35394	2.43217	2.74951	1.88363
RP11-342D11.2	0.00723857	0.344226	-0.485564	-0.28505	1.14509	0.682879
RP11-342K2.1	2.30656	2.23761	3.01615	2.72407	2.71474	1.12623
RP11-342K6.1	2.3535	2.37006	1.7995	1.8512	2.47413	2.46322
RP11-343C2.11	2.62137	2.46413	2.12491	2.35051	2.73904	3.46121
RP11-343C2.12	2.79977	3.00088	2.4438	2.77525	4.14621	4.28957
RP11-343H5.4	1.04729	1.17199	2.73467	3.19086	3.76088	4.73683
RP11-343L5.2	0.863225	0.822624	1.68687	1.65276	1.01923	0.560157
RP11-345J4.5	3.41381	3.60879	3.0684	3.62846	3.55089	4.37948
RP11-345K9.3	-0.697041	-0.790185	-0.115249	0.140752	0.493873	0.488032

RP11-345P4.9	-0.0124477	-0.39658	-0.117291	0.0612232	1.1812	0.53826
RP11-347C12.1	1.92862	1.55566	2.49656	2.47776	2.21481	0.206956
RP11-347E10.1	0.832372	1.18106 ?	?		-0.637731	-0.662104
RP11-347H15.2	0.0735324	0.589648	-0.487874	-0.288505	1.06793	1.29901
RP11-347I19.7	1.42176	1.50623	1.66896	1.618	0.929071	0.602675
RP11-347P5.1	-0.432112	-0.734698	0.893354	0.935626	0.210164	-0.848487
RP11-348P10.2	1.21386	1.29209	-0.825729	-0.786664	1.2101	0.597145
RP11-34P1.2	2.26288	2.38858	2.64388	2.91243	3.42191	3.99394
RP11-34P13.13	1.61955	1.51281	0.452448	0.590865	-0.436028	-1.46015
RP11-350G24.2	1.2776	1.87823	0.764966	1.33009	1.32953	1.20814
RP11-350J20.5	-0.373266	-0.135952	0.422427	0.766909	0.193229	-0.147122
RP11-351I24.3	-0.487563	-0.488532	-0.017899	0.509858	-0.828955	0.735843
RP11-352M15.2	2.85552	2.91002	2.5512	2.54582	2.21007	2.00755
RP11-355B11.2	1.85324	2.05873	1.52591	1.7894	1.963	2.12305
RP11-355O1.11	0.520176	0.697098	0.723445	0.549472	-0.0454657	-0.577229
RP11-356J5.12	-1.41331	-0.93902	-0.350462	0.176693	-0.0903144	1.3157
RP11-357N13.6	1.62288	1.38894	2.54781	2.53864	1.96907	1.27061
RP11-359B12.2	0.912435	0.892546	2.05182	2.09953	-0.135126	-0.150979
RP11-359E3.4	1.10833	1.16656	0.935482	0.89124	0.568596	-0.151977
RP11-35G9.3	1.07964	1.4166	-1.13466	-0.822638	-0.837001	-0.22179
RP11-362K14.5	1.30536	1.57951	2.16778	1.96625	1.86798	1.13315
RP11-363N22.3	0.733328	0.678757	0.615529	0.48943	0.188287	-0.747824
RP11-365D23.4	1.16702	1.28956	1.41542	1.77394	1.32987	1.77175
RP11-365H23.1	4.2117	4.23441	1.30837	1.89682	1.61585	2.02953
RP11-366L20.2	5.77317	5.60075	2.32398	2.34279	2.8365	2.49298
RP11-367E12.4	0.903995	1.03816	0.166381	0.660427	0.889536	0.974522
RP11-367G18.2	1.74049	1.89796	0.846931	1.50946	2.11391	2.83189
RP11-36C20.1	4.40088	4.49764	4.21156	4.76301	5.08994	5.61224
RP11-372E1.1	2.40379	2.67739	2.35998	2.7942	3.48854	3.49488
RP11-378G13.2	0.0243481	0.263611	1.09624	1.66826	2.19261	2.22743
RP11-378J18.8	1.22542	1.14987	1.56431	1.57745	1.18427	1.29068
RP11-379B18.5	-0.0695851	0.375701	1.75213	1.87767	-2.572	-4.11926
RP11-379F4.4	0.4521	0.766044	1.69336	1.42259	0.427241	0.176042
RP11-379H18.1	3.15579	2.92302	2.70218	2.63265	2.74697	2.22994
RP11-37B2.1	3.77564	3.54418	3.37298	3.09085	2.99969	2.58792
RP11-37C7.3	0.493914	0.414146	-0.0336419	0.300663	-0.381696	-0.799586
RP11-37J13.1	0.219054	0.653381	0.0638618	0.341257	0.683355	0.687948
RP11-380B4.3	0.847885	0.666511	1.78805	1.2041	0.469063	-0.752286
RP11-380G5.3	0.567857	0.107125	0.662041	0.476226	1.06767	0.719622
RP11-381O7.3	1.25993	0.579692	-1.70851	-1.33321	1.17645	0.64596
RP11-384B12.2	0.770753	1.02387	0.571444	0.606433	1.10793	0.968076
RP11-384B12.3	1.63634	1.95207	1.38097	1.76772	2.40345	2.46372
RP11-384K6.6	-1.49529	-1.38609	1.93928	2.0329	0.642092	-0.553748
RP11-386G11.10	3.56842	3.28623	3.20068	3.78039	4.95906	2.94351
RP11-386G11.5	0.440676	0.5903	-0.0986878	-0.322487	-0.229832	-0.416348
RP11-386I14.3	0.593526	0.874301	1.00203	1.41093	1.19705	1.01451
RP11-386J22.3	0.595618	0.318612	0.225978	-0.301307	-0.0602604	-0.899103
RP11-386M24.4	2.59889	2.51323	2.08486	2.4909	2.34432	2.50602
RP11-390E23.6	1.44562	1.42594	0.914572	1.07114	0.83161	0.578775
RP11-390K5.1	0.940933	0.826884	0.924678	1.29157	1.51046	1.85764
RP11-390P2.2	1.08887	1.31199	0.191626	0.578475	1.18935	0.769095
RP11-390P2.4	1.69327	1.52019	-0.659745	-0.938639	0.815493	0.224139

RP11-391L3.3	0.440559	0.370142	1.11115	0.810106	-0.802092	-1.24695
RP11-392O17.2	-0.175369	0.013012	1.02599	0.968047	-0.320293	-1.4584
RP11-392O18.2	0.503145	0.592522	0.138526	0.125175	0.688494	0.443129
RP11-392P7.1	-0.813933	-0.400153	-0.803249	-0.30984	0.938858	1.26406
RP11-393I2.2	0.301026	0.305445	0.747284	0.139221	0.839605	0.33778
RP11-393N4.2	0.901355	1.23813	0.794304	1.44625	2.3059	2.13075
RP11-394B2.1	0.52697	-0.805267	2.26295	1.82688	2.03058	1.93754
RP11-394B2.5	-0.306598	-0.140771	0.324373	0.430424	0.258664	0.422664
RP11-395G23.3	1.91933	2.41369	-0.428455	0.350701	0.749157	1.13883
RP11-395L14.17	2.20891	2.27806	2.13635	2.19299	2.44153	2.5744
RP11-397E7.4	0.291391	0.201521	-0.241062	0.651353	0.473641	0.803384
RP11-397P13.7	-0.0547804	0.292146	-0.300715	0.139221	1.00508	1.20023
RP11-398K22.12	2.52248	2.47915	0.350791	-0.557896	2.25649	1.63638
RP11-39C10.1	1.66476	1.61265	1.30099	1.63725	1.64424	1.34295
RP11-3D23.1	1.59888	1.69894	0.725235	1.30079	1.94369	1.70881
RP11-3J10.7	0.772426	0.843349	0.952975	1.0599	1.82089	1.81405
RP11-400F19.18	0.194844	0.137189	1.12031	1.02009	0.509706	-0.619042
RP11-400F19.6	1.71339	1.51337	1.90539	2.0714	2.4318	2.75846
RP11-400K9.4	0.80878	0.831845	1.68687	2.27921	-2.01588	-1.80567
RP11-401L13.4	2.62971	2.8895	2.14219	2.73548	3.13262	3.00102
RP11-403B2.7	-0.082776	0.111552	?	?	0.90952	0.31956
RP11-403I13.8	3.16014	3.00673	2.87395	2.78025	-1.49605	-2.20819
RP11-403P17.6	0.0199581	0.0853567	0.179664	0.332427	0.559433	0.799004
RP11-404J23.1	-2.69815	-3.46127	?	?	1.84354	2.15129
RP11-405A12.1	1.50287	1.62548	1.17015	1.6932	2.80787	2.73056
RP11-408P14.1	6.02619	5.86893	5.00528	5.35724	5.99089	6.4333
RP11-409C19.2	1.20554	1.55533	0.588603	1.11421	1.52005	0.978232
RP11-415J8.3	2.13734	1.88047	2.23035	2.07787	1.3528	0.600631
RP11-417F21.1	0.480917	0.503023	0.102	-0.0642959	0.351018	-0.421131
RP11-417L14.1	1.67735	1.85476	1.42238	2.08238	2.50273	2.06427
RP11-418J17.1	0.368265	0.184547	1.88534	1.77913	3.57531	3.40961
RP11-41O4.2	1.77262	1.2583	-2.37543	-2.40763	-1.80184	-2.70579
RP11-421E14.2	1.77668	1.87257	1.85542	1.48367	1.33409	0.615294
RP11-421F16.3	2.28666	2.61665	2.01617	2.22211	1.88661	2.06149
RP11-421L21.3	0.592043	0.680702	1.64306	1.72802	0.746072	1.09779
RP11-421N8.1	3.26422	3.2775	3.0879	3.4867	3.68533	4.2784
RP11-422P24.11	0.987481	0.6247	-0.229171	-0.154483	0.0402612	-0.619042
RP11-423E7.2	0.529821	0.419895	1.6494	1.68562	0.458718	-0.348991
RP11-423F24.3	1.07005	1.2583	0.933097	1.27044	1.85784	1.71776
RP11-423H2.1	3.9571	3.86089	3.27577	3.80075	4.8839	5.11088
RP11-423P10.2	2.73542	2.7123	2.18874	1.96302	2.3529	2.12145
RP11-424C20.2	3.69081	4.20537	2.58373	3.20963	2.94361	3.354
RP11-425L10.1	6.62275	6.47046	6.11862	6.54127	7.41558	8.48492
RP11-426C22.4	1.39683	1.46419	0.217243	0.843091	-6.64581	?
RP11-426D19.1	1.20786	1.12217	0.686917	0.120113	2.15028	0.752791
RP11-426J5.3	1.05586	1.12217	1.12031	0.857026	-1.26766	-1.61882
RP11-427L15.2	-0.611409	-0.253233	0.597917	0.520618	0.448309	-0.348991
RP11-428P16.2	0.934939	0.639288	0.386193	-0.103217	-0.635033	-0.707508
RP11-429P3.8	-0.289526	-0.158917	0.53081	0.57776	0.670686	0.315752
RP11-431J24.2	2.06111	1.95063	-3.85951	-4.08532	-5.65305	?
RP11-435F17.3	-0.0940992	0.152832	1.07423	1.09968	1.4578	1.35261
RP11-436H11.1	0.183582	-0.0665709	0.0432885	0.479944	1.54132	1.20646

RP11-437B10.1	3.26883	3.24556	3.47061	3.35287	3.75387	3.4303
RP11-438B23.2	-2.45038	-2.37465	?	?	1.06084	1.1637
RP11-438J1.1	1.24721	1.39654	0.408842	1.24196	2.06858	1.19613
RP11-438L7.3	2.39307	2.52162	1.89507	2.41658	2.00795	1.9956
RP11-439E19.10	1.85554	1.51447	-0.401986	-0.649181	-0.184426	-0.662104
RP11-43F13.1	1.55057	1.26572	-2.08749	-1.24133	0.865776	1.01203
RP11-43N16.4	0.049101	-0.0978662	0.125651	0.789562	0.807792	0.0927757
RP11-440D17.3	?	?	-1.01332	-0.616766	0.971023	1.14298
RP11-440L14.1	1.07578	1.10769	1.30838	1.39906	0.653152	-0.348934
RP11-441O15.3	0.171399	-0.292758	0.503064	0.28359	-0.200727	-0.662104
RP11-442H21.2	4.54672	4.62764	3.49429	3.64202	5.16852	4.23924
RP11-443B20.1	1.46455	1.50774	0.636738	1.12005	0.474036	-0.151977
RP11-443P15.2	-1.27248	-0.508457	4.6011	4.89819	1.37315	1.1007
RP11-444D3.1	1.66727	1.20254	0.893354	0.383121	1.49454	0.847941
RP11-444J21.2	-0.239491	-0.105152	-0.538101	-0.000171691	1.67113	1.56011
RP11-446E9.1	4.54019	4.69568	3.65543	4.2265	4.3668	5.46167
RP11-447D11.3	1.27825	1.28539	0.308431	0.334214	0.947083	0.90806
RP11-448A19.1	3.31634	3.24624	2.99639	3.00752	3.3565	2.57407
RP11-448G15.3	0.586404	0.635262	0.460428	0.646135	0.766027	0.560157
RP11-449H3.3	3.24789	3.00817	3.15287	3.53777	3.17848	3.72072
RP11-449J21.3	0.999603	0.930556	1.4447	1.34516	1.23165	0.66164
RP11-449L13.2	-0.0194058	-0.485378	2.47094	3.52817	-0.852709	-0.662104
RP11-449P15.2	1.13628	1.31416	2.54367	2.33358	0.407527	-0.0293981
RP11-44F14.6	-0.341358	-0.122851	0.723445	0.367002	0.210164	-0.151977
RP11-451F14.1	0.367103	0.107125	1.11115	1.08105	0.107996	-0.31423
RP11-452F19.3	1.01523	0.850103	0.308431	0.724135	2.27338	2.50762
RP11-452L6.5	2.37507	2.3592	2.53704	2.76985	1.61998	1.8437
RP11-452N17.1	4.23444	4.2236	3.7634	4.21111	4.48188	5.61662
RP11-454F8.2	0.183671	0.444137	0.610974	0.0612232	1.22238	0.993783
RP11-456J20.1	1.41332	1.65305	1.06297	1.69931	1.60616	1.99214
RP11-458D21.5	3.41799	3.39199	2.60537	2.59632	1.77034	1.27898
RP11-458J1.1	-0.00900304	0.040177	0.884879	0.900838	-0.0324241	-0.662104
RP11-45A17.4	-0.632781	-1.105	0.474784	-0.275776	1.90957	0.126299
RP11-45M22.2	2.29246	2.83866	3.02593	3.04852	2.1079	1.27061
RP11-45M22.3	0.00148521	0.107125	0.793829	0.139221	-0.594016	-1.12145
RP11-460B17.1	-6.68418	-3.65369	-3.85951	-3.52222	1.44825	1.88577
RP11-460N11.2	4.8662	4.78146	-2.27595	-2.62533	0.664974	1.19764
RP11-461A8.4	0.178339	0.19415	0.178198	-0.131011	2.37458	2.28105
RP11-463O12.5	4.59608	4.63784	3.75479	3.63123	4.06212	3.11451
RP11-464D20.2	1.47911	1.50199	1.46966	1.77467	2.2136	3.3004
RP11-464F9.1	0.177369	0.141995	0.664064	0.797918	1.38794	0.814657
RP11-466F5.8	1.41335	1.55739	-0.166577	-0.0304306	-0.648048	-0.505505
RP11-466H18.1	6.58274	6.51338	5.92436	6.33506	7.61215	8.36243
RP11-467L13.7	1.00834	0.822624	0.558032	0.158078	0.282309	-0.0918635
RP11-468E2.1	3.78145	3.78819	2.85326	3.22094	3.99248	4.06025
RP11-468E2.4	0.565968	0.579228	1.30347	0.920812	1.85871	1.10544
RP11-468O2.1	1.59304	1.59918	1.29295	1.81868	1.45544	1.17685
RP11-46C24.7	0.45076	0.265185	0.503064	0.549472	-0.356358	-0.899103
RP11-46D6.1	-0.972229	-1.47269	1.32663	0.981568	-0.202441	-0.694933
RP11-473I1.9	0.0778582	-0.0303378	1.50422	0.879196	-0.0389768	-0.0875065
RP11-473M20.16	0.792847	0.635262	0.64945	1.01017	0.909612	1.44307
RP11-473N11.2	3.85249	4.17125	3.12304	3.59839	3.83066	3.67691

RP11-474L11.5	0.41996	0.581631	-0.229171	-0.301307	0.222446	-0.662104
RP11-474P2.6	0.676033	0.804012	3.88525	3.77686	0.871134	0.878317
RP11-474P2.7	-0.255978	0.0450795	2.95603	2.69531	-0.23389	-0.619042
RP11-475C16.1	6.36839	6.28856	6.00623	6.33154	6.79671	7.44167
RP11-477D19.2	2.31176	2.24927	1.76096	1.7082	1.94683	1.31999
RP11-478B9.3	0.825997	1.24486	0.371838	0.880858	0.258664	0.100762
RP11-478C6.4	1.37793	1.48642	0.596248	1.22918	2.01934	2.43449
RP11-479G22.8	1.85554	1.96763	-0.659745	-0.616766	1.39501	1.18815
RP11-47I22.2	0.593382	-0.177293	-0.183372	-0.275776	1.87915	1.27788
RP11-480A16.1	0.990853	0.830166	2.37466	2.20507	2.4499	1.88847
RP11-480C16.1	-0.676496	-0.418273	-0.350462	0.535117	1.08794	0.80115
RP11-481C4.2	2.47336	2.5456	2.66823	2.5169	2.14061	1.40185
RP11-481J2.4	0.340858	0.0368339	0.351481	0.41512	0.837007	-0.169621
RP11-486G15.1	0.470938	0.331671	0.636738	0.619178	-0.10558	-0.38461
RP11-486O12.2	1.40468	1.33154	1.58789	1.47484	0.387782	-0.406704
RP11-488C13.1	4.23066	4.18243	4.06714	4.31653	4.98983	5.35806
RP11-488C13.5	0.640343	0.729818	0.0606147	-0.325416	0.405666	-0.151742
RP11-488L18.1	0.0654482	0.130048	-0.684793	0.202813	0.181103	0.242645
RP11-488L18.10	0.586404	0.746683	1.5714	1.30907	0.976371	-1.06257
RP11-488L18.4	3.8943	3.87812	4.06203	4.08962	3.84528	3.41483
RP11-490H24.5	4.55625	4.39028	5.07385	5.37646	6.27196	6.14673
RP11-495P10.1	0.759667	0.621234	0.524856	0.304371	0.0376493	-0.32357
RP11-495P10.10	2.14964	2.08077	1.99081	2.2323	2.6843	2.78589
RP11-497H16.9	0.972854	1.21625	1.6927	1.68021	2.21683	2.39295
RP11-498C9.15	3.56067	3.54418	3.11558	3.34601	3.49704	2.97277
RP11-49C9.2	1.88763	2.22642	1.9003	2.27996	2.85318	3.16946
RP11-49K24.6	1.16527	1.39684	-0.100017	0.447167	-1.80233	0.744308
RP11-49K24.8	1.85237	1.94888	0.395611	0.633543	-0.681421	-1.00454
RP11-49K24.9	1.3177	0.532397	1.78336	1.05792	?	?
RP11-4L24.4	3.01997	2.97528	0.802913	0.367002	2.39361	1.78597
RP11-500C11.3	0.146799	-0.00985836	0.0418738	0.199462	0.00824305	-0.0694034
RP11-500G22.5	0.531688	0.549699	0.152339	-0.0503948	0.475137	-0.274569
RP11-500M8.7	2.01094	2.28948	2.19288	2.6164	1.53567	2.07682
RP11-504P24.3	1.76604	2.02568	1.73665	1.45318	1.62155	0.959266
RP11-504P24.8	0.980194	0.683526	1.40161	1.01842	1.17836	0.288725
RP11-506K6.4	-0.45097	0.479758	0.174138	0.158078	2.59944	1.85556
RP11-507E23.1	2.82278	2.86379	2.4221	2.82335	3.57848	4.26897
RP11-507K2.6	0.80878	0.813344	0.0260635	0.317536	0.0946955	-0.280287
RP11-512F24.1	1.7257	1.60102	1.93811	2.32431	2.2989	1.51408
RP11-513I15.6	2.15837	1.95973	2.87488	3.05341	2.54008	2.41267
RP11-513M16.8	0.610039	0.477387	-0.909266	-0.825773	0.0499374	0.48553
RP11-516A11.1	2.88443	3.00596	3.64049	3.93259	5.30007	4.52187
RP11-517B11.7	0.71885	0.980157	0.571444	0.632724	1.44825	1.97981
RP11-517H2.6	0.592761	0.587231	1.20324	0.692302	0.74217	0.273313
RP11-517I3.2	-0.359059	-0.531913	-1.01332	-1.15432	1.28622	0.972384
RP11-51O6.1	7.1585	7.11936	6.38474	6.83818	7.3233	8.1095
RP11-521B24.4	1.44051	1.52019	0.488988	0.19507	1.40043	0.893261
RP11-524D16__A.3	-0.744656	-1.14061	-1.13882	-0.682344	1.45347	0.463298
RP11-525G13.2	0.758056	0.744497	-0.964455	-0.395433	-0.265199	-0.456694
RP11-527J8.1	0.231764	0.122235	0.045429	-0.108685	0.395063	-0.121607
RP11-52A20.2	0.470938	0.794611	1.07392	0.95732	0.234612	-0.848487
RP11-530N7.2	-1.27248	-1.41808	1.13846	1.16735	-0.852709	-0.214706

RP11-531A24.5	0.195852	0.407614	0.610974	0.505972	0.147163	-0.752286
RP11-533F5.2	0.0907586	0.342077	-0.499406	0.355129	-0.171115	1.30504
RP11-536K7.3	0.700511	0.259652	0.626691	0.418233	0.562709	0.295417
RP11-53I6.1	0.125995	-0.14707	-0.268132	-0.0669985	0.361701	0.951819
RP11-53O19.3	1.52978	1.78985	1.41643	1.30907	2.62047	2.32672
RP11-540B6.6	2.50306	2.40445	2.43488	2.31745	2.01919	1.34861
RP11-543B16.2	1.70051	1.67376	1.10822	1.70465	2.57526	3.12041
RP11-543P15.1	4.86446	4.78334	3.77334	4.19597	4.94944	5.9446
RP11-544A12.8	0.417952	0.546778	0.434134	0.129916	0.206993	-0.345445
RP11-545E17.3	0.768595	0.68698	0.0260635	0.0612232	0.234612	1.12623
RP11-545I5.3	0.415304	0.515591	1.42857	1.51972	1.03135	0.719061
RP11-546D6.3	2.41587	2.39101	1.72338	1.82773	1.78623	1.76351
RP11-547D13.1	0.255223	0.407614	0.571444	0.879925	0.578176	0.522196
RP11-54D18.4	2.7768	2.87512	2.06211	2.1894	3.57802	3.26971
RP11-54H7.4	2.10184	2.60056	0.646651	1.23007	2.67509	2.73624
RP11-54O7.3	?	-5.45651	0.371169	0.591699	1.08794	1.76905
RP11-550F7.1	2.92228	3.21128	1.973	2.66505	3.34555	3.59807
RP11-552F3.12	0.466392	-1.1369	0.140347	0.649735	0.289362	-0.229851
RP11-552F3.9	0.595055	0.81791	-0.20074	0.466914	0.0617624	-0.143752
RP11-552M11.4	1.04724	1.13005	0.984779	1.04243	1.84861	0.373921
RP11-552O4.2	1.17411	1.42837	0.80799	1.02146	0.339787	0.320496
RP11-553L6.5	0.980457	0.736899	3.33611	2.6856	3.66387	2.59708
RP11-555H23.1	0.520709	0.813919	-0.095919	0.399062	0.373208	0.126299
RP11-556K13.1	1.80664	2.19448	1.15214	1.53313	2.38142	2.5009
RP11-561B11.2	0.670333	0.384437	1.07985	-0.0787663	0.835746	1.45879
RP11-561O23.9	0.171399	0.331671	-0.229171	-0.301307	0.679613	-0.121607
RP11-561O4.1	0.319999	0.742291	-0.472385	0.412749	-0.284532	-0.301195
RP11-565F19.2	0.0425183	-0.177293	0.793829	0.999755	0.815493	-0.848487
RP11-566E18.1	2.84008	3.10891	2.55458	2.62589	2.55402	2.9515
RP11-566F5.1	-1.20691	-0.908293	0.132353	-0.255624	0.993819	0.707984
RP11-566K19.8	2.19982	2.07854	1.28812	2.14992	2.31695	2.75351
RP11-567G24.1	3.91286	4.12902	4.41431	4.87911	4.9085	4.75087
RP11-567M16.6	1.14018	0.996642	0.208904	0.317536	-0.660096	-1.4584
RP11-569G13.2	1.20786	2.06856	-5.1783	-6.09892	?	?
RP11-569G13.3	1.11475	2.12589	?	-6.09892	-6.64581	-5.69922
RP11-56B16.1	0.754905	0.930223	0.652528	0.824409	0.887346	0.650921
RP11-570P14.1	2.19867	2.21928	1.48161	1.62001	2.38697	2.26263
RP11-571I18.5	0.266817	-0.292758	0.597917	0.248838	2.21315	1.67702
RP11-572P18.1	7.18757	7.07936	6.91461	7.36183	7.48874	8.36296
RP11-573D15.9	2.03565	2.20606	2.35952	2.57066	2.9209	2.5787
RP11-574K11.24	1.32887	1.24456	1.662	1.52784	1.44302	0.847941
RP11-574K11.29	0.0127117	-0.0598588	0.724633	0.597031	0.765399	-0.205848
RP11-574K11.31	1.29368	1.03699	1.62984	1.32955	1.48115	0.257518
RP11-574K11.32	2.11087	2.23457	2.16095	2.30993	3.1361	2.78995
RP11-575F12.3	1.98394	2.59516	-6.17309	?	-5.65305	-4.11926
RP11-575G13.2	1.46586	1.33161	0.544485	0.535117	-0.0378982	-0.00614162
RP11-575L7.8	0.77295	0.24496	0.510608	0.655865	-0.211872	-1.36072
RP11-57H14.4	1.44562	1.49719	2.16964	2.25313	1.70604	1.61525
RP11-57K17.1	0.266817	0.0450795	-0.428455	-1.02108	0.947083	-0.183001
RP11-582E3.6	0.959191	1.12967	1.55798	1.86848	1.25263	0.702755
RP11-582J16.4	0.688395	0.946746	0.486828	-0.40892	-0.517858	-0.488546
RP11-582J16.5	4.71742	4.88595	3.07485	3.19199	3.06143	2.74716

RP11-585P4.5	-0.376978	-0.58	0.0260635	0.266325	0.351018	0.315752
RP11-587D21.1	1.99257	2.20806	1.55851	1.991	2.13963	2.5457
RP11-588H23.3	-3.37567	-3.73146	0.910963	0.711398	0.958806	0.455744
RP11-592N21.1	3.52341	3.71993	2.63113	3.02489	3.03899	3.55359
RP11-597D13.9	-0.145905	-0.373783	?	?	1.62229	1.15106
RP11-598P20.3	1.98042	2.19895	0.138526	0.334214	1.06084	0.176042
RP11-599J14.2	1.71691	2.0333	-5.02295	-4.67942	-6.64581	-4.73995
RP11-59C5.3	2.36351	2.09819	2.1644	2.22221	1.74794	1.1298
RP11-59D5__B.2	1.76038	1.88148	-4.18092	-4.52056	-4.65668	-2.70579
RP11-5N19.3	0.658554	0.491443	0.259532	-0.108685	-0.356358	-0.421131
RP11-600F24.7	0.61388	0.467978	0.73541	0.902467	-0.609852	-1.006
RP11-603J24.17	1.32138	1.24717	-0.862992	0.422556	0.760655	0.980895
RP11-603J24.7	4.95958	4.75902	3.72595	4.01364	3.48494	3.62224
RP11-603K19.1	1.01831	1.19515	1.36999	1.19196	0.411068	0.203514
RP11-606P2.1	0.92359	1.31199	0.711372	1.22214	2.27928	2.00067
RP11-609D21.3	-4.38616	?	2.13095	1.63899	2.05348	1.30314
RP11-60L3.1	-0.679236	0.591708	0.501465	1.13306	-2.21215	-0.716379
RP11-611O2.3	2.312	2.38867	-0.00507566	0.0789104	0.576048	1.02426
RP11-611O2.5	1.42514	1.48607	-1.72062	-0.304637	-2.25748	0.880599
RP11-617F23.1	0.338202	-0.0830877	2.64537	2.60789	0.319086	-0.71669
RP11-618G20.1	1.37958	1.39616	-0.920139	-0.641572	0.888063	2.33254
RP11-61F12.1	1.37242	1.54842	-0.0134653	0.0612232	-1.41201	-1.79933
RP11-61L23.2	1.81415	1.37808	-0.0969278	0.489872	2.4707	0.691436
RP11-61N20.3	1.27629	1.46815	0.890976	1.34031	2.59337	3.03679
RP11-620J15.3	0.312247	0.305445	1.19157	1.38307	0.807363	1.24753
RP11-624D20.1	0.410189	0.73754	0.641712	0.724135	0.185993	-0.121607
RP11-631M6.2	2.8104	2.75234	3.3149	3.13959	2.82882	3.11028
RP11-631M6.3	0.701877	0.850103	1.98141	1.38307	0.894341	0.100762
RP11-631N16.2	3.66833	4.1592	4.0477	4.30902	2.60179	1.92267
RP11-631N16.4	2.67162	2.77318	1.662	1.68562	0.0677351	0.100762
RP11-632C17__A.1	5.81998	5.65884	4.85898	5.20824	6.17943	6.81084
RP11-632K20.7	1.93719	1.89528	2.5333	2.88173	1.60906	1.09704
RP11-635L1.2	-0.791952	-0.629746	0.488988	0.491176	0.339787	-0.38461
RP11-641A6.5	1.24186	1.49406	0.475375	0.869982	0.832015	0.177892
RP11-641D5.1	5.52037	5.50062	5.27567	5.62865	6.31893	6.88113
RP11-644F5.10	1.84236	1.83411	1.25109	1.51442	0.293782	-0.326681
RP11-644F5.11	1.6585	1.2583	1.05494	1.46107	-0.0752099	0.33778
RP11-649A18.12	0.971707	0.667574	0.533504	0.428067	0.823676	0.572919
RP11-649E7.5	4.68657	4.87332	2.96422	3.70812	4.70119	4.89883
RP11-64B16.2	2.79189	2.79301	1.94021	2.35881	3.45723	3.07836
RP11-64K7.1	1.83441	2.38382	1.7216	2.23647	2.26041	2.44402
RP11-650L12.2	1.54757	1.45036	1.50394	1.73966	1.74258	2.2816
RP11-651P23.4	4.152	4.16664	3.38145	3.7847	4.0912	4.83048
RP11-658F2.8	2.48633	2.35666	3.20975	2.89933	2.58871	1.71503
RP11-65I12.1	4.56633	4.98019	-1.46921	-0.551532	0.10912	0.1904
RP11-65L3.3	1.55566	1.39945	-1.53563	-1.3165	1.09285	0.846955
RP11-660L16.2	0.727258	0.570657	-0.598354	-0.55404	0.222446	0.151378
RP11-661A12.7	-0.361118	-0.358883	0.0395736	-0.0539789	0.649349	0.218682
RP11-661A12.8	2.56147	2.44164	2.20631	2.49297	2.87111	2.92574
RP11-663P9.2	0.9387	1.00193	1.13304	1.6487	1.77195	2.33257
RP11-667K14.4	-1.72363	0.418589	0.665029	-0.299019	0.603777	-0.041579
RP11-66B24.4	6.25566	6.63193	2.40808	2.28525	-0.0239972	-0.725053

RP11-66B24.7	3.12345	3.39476	-2.86056	-3.30006	-6.47696	?
RP11-66B24.9	2.14014	2.52585	-2.37543	-2.40763	-4.65668	-5.69922
RP11-66N24.3	1.51061	1.54882	1.75929	1.70381	1.63542	1.06691
RP11-66N24.4	1.16506	1.05367	1.17221	1.13172	1.40716	0.60557
RP11-673C5.1	6.20266	6.15498	5.37516	5.51594	5.39218	5.71672
RP11-676M6.1	4.30095	4.21272	4.45845	4.85773	4.34577	4.4112
RP11-67L14.1	1.44562	1.67674	-0.229171	-0.086319	-2.65941	-2.12114
RP11-67L2.2	1.77668	2.02502	1.64306	1.51326	2.01919	2.08121
RP11-67L3.5	0.233041	0.278044	0.086118	0.11486	0.813902	0.820011
RP11-680G24.4	0.44982	0.688655	0.570346	0.868592	1.76047	0.711803
RP11-680H20.1	3.42422	3.50381	2.68001	3.13201	3.52369	4.30028
RP11-686D22.7	0.940144	0.556218	-1.71745	-1.95705	0.638129	0.269524
RP11-686D22.8	1.2305	0.825989	-1.04009	-2.73425	0.899245	0.695477
RP11-688I9.5	-0.569597	0.676782	?	?	0.134221	0.293394
RP11-690D19.3	0.133932	0.6247	1.99143	1.90801	1.71041	1.56011
RP11-697N18.3	2.26672	1.83639	1.5913	1.87419	0.0677351	-0.619042
RP11-69L16.5	5.37843	5.43823	4.75008	5.1442	4.75848	4.93101
RP11-6O2.3	1.5065	1.28763	-0.214925	-0.0148858	-0.197201	-0.978989
RP11-6O2.4	2.67549	2.30914	-0.483533	-0.665688	1.21926	-0.62585
RP11-700P18.1	3.5534	3.72379	2.22274	2.78837	3.53508	3.78631
RP11-701H24.4	2.63632	2.52301	?	-6.09892	0.578176	0.937201
RP11-705C15.2	1.35311	1.78912	2.63027	2.68885	1.83505	2.00221
RP11-705C15.3	1.28396	1.38268	2.49245	2.31325	1.7868	1.48315
RP11-706J10.2	-0.882238	-0.982774	?	?	1.67064	2.7534
RP11-708J19.1	0.312247	0.395227	0.259532	0.334214	-0.0454657	-0.95156
RP11-709A23.1	2.16203	1.97597	0.945825	0.761609	0.578176	-0.497071
RP11-70J12.1	-0.891472	-1.14061	-0.325375	-0.751045	0.909612	0.816919
RP11-70L8.4	3.22688	3.17749	3.25749	3.14844	3.74473	3.53259
RP11-713C19.2	0.440942	0.787917	-0.011668	0.535924	0.339787	0.36051
RP11-715J22.6	0.701877	0.559609	0.259532	0.300663	-0.338214	-1.06257
RP11-717F1.2	4.84421	4.6403	4.69457	4.51953	2.75219	1.77354
RP11-722G7.1	1.51696	1.69003	2.10247	2.10618	0.809291	-0.374118
RP11-726G1.1	4.26717	4.52549	1.5431	0.877446	-3.93681	-4.09509
RP11-727A23.5	2.0845	2.05612	2.34656	2.2749	1.93847	1.31864
RP11-728B21.3	1.58408	1.7559	0.122355	0.463309	0.979001	1.1872
RP11-72I8.1	-0.026521	-0.0197685	0.662041	0.28359	-0.338214	-1.12145
RP11-730G20.2	3.60431	3.33279	3.37313	3.43986	3.01054	3.03855
RP11-731C17.2	-0.0990262	-0.0876659	1.5647	1.30907	-0.572647	-1.18284
RP11-734E19.1	-0.651496	-0.237583	-0.620441	0.0318469	0.689729	1.12122
RP11-734K2.4	1.46586	1.50157	2.50998	2.40294	-0.356358	-0.609865
RP11-734K23.9	-1.57311	-1.07955	-0.386284	-1.20284	1.78121	-0.220525
RP11-737O24.3	1.51447	1.44577	3.02315	3.10388	2.34705	2.42577
RP11-73M18.10	0.131076	0.922586	-0.422033	-0.301449	0.423524	0.69447
RP11-73M18.2	0.826201	0.236744	-1.89446	0.473735	-0.147495	0.872726
RP11-73M18.6	0.243547	0.223769	0.120378	-0.301307	0.197777	-0.536593
RP11-73M18.7	0.996034	0.685025	0.272059	0.176795	1.11684	0.84004
RP11-73M18.8	1.88591	1.81795	1.26773	1.30061	2.1726	2.04819
RP11-742D12.2	2.53342	2.89691	-5.1783	-3.30006	-2.95876	-3.38329
RP11-745A24.1	-0.239491	-0.333394	-0.350462	-0.585061	0.901996	0.443129
RP11-74E24.2	3.09569	3.14327	2.54941	2.7878	2.8887	2.86853
RP11-752D24.2	0.0152977	0.0918691	0.747284	0.100749	-0.267833	-1.24695
RP11-752G15.4	-0.128497	0.201483	-0.255615	0.193355	0.278698	-0.22104

RP11-756P10.3	1.76129	1.75683	1.44522	1.88592	3.26596	3.46281
RP11-756P10.6	-0.312954	-0.130062	0.945473	1.47139	2.95174	3.03498
RP11-75A9.3	-0.549135	-0.734698	1.13846	1.12005	-0.121008	-0.151977
RP11-75C10.6	0.735617	0.840999	0.571444	0.139221	0.489522	-0.799586
RP11-75L1.2	4.76767	4.74341	4.73377	5.18947	5.59293	6.04872
RP11-761N21.2	6.09066	5.96456	4.99703	5.41319	6.08107	6.94615
RP11-762H8.2	0.0559314	-0.036446	-0.455418	-0.52367	1.65261	0.752791
RP11-766H1.1	0.0936819	0.0355113	0.623127	1.24258	1.33093	1.62891
RP11-767L7.1	-0.0407343	-0.46266	0.706632	0.798133	-0.490197	-0.799586
RP11-767N6.2	0.631914	0.766044	1.68687	1.30061	0.706084	0.0213376
RP11-76E16.2	1.44677	1.36775	0.641102	0.89714	1.72125	1.00218
RP11-76H14.2	0.846578	0.399204	0.749157	0.551019	0.450243	0.267883
RP11-778D9.4	4.04782	4.1136	3.35418	3.86447	3.2719	3.6816
RP11-77H9.2	1.10605	0.892508	0.92267	0.928306	1.14501	0.748521
RP11-77P6.2	0.784805	0.929533	0.503064	0.672606	-0.23389	-0.577229
RP11-78A19.3	-0.27139	-0.351176	1.21228	1.46439	1.33764	0.376813
RP11-791G16.4	1.58852	1.74831	0.671991	1.17217	1.39344	1.10913
RP11-792A8.4	0.42722	0.574751	0.446066	0.0290641	1.56848	1.07138
RP11-793H13.8	2.22794	1.86085	2.31646	2.21908	1.06998	1.42793
RP11-795H16.2	0.0711036	0.602628	-0.325375	-0.108685	0.599356	0.702755
RP11-796G6.1	2.46697	2.55514	2.52589	2.81583	3.70498	4.16177
RP11-797A18.5	1.00142	0.831845	0.759062	0.445854	0.185296	-0.536593
RP11-798G7.8	1.97337	2.08021	2.64938	2.36286	1.48439	0.68568
RP11-798M19.6	-0.191134	0.107125	0.120378	0.445854	0.757612	-0.247123
RP11-79H23.3	0.710384	1.27191	1.19157	1.95729	-2.13612	-1.79933
RP11-79P5.10	1.78661	1.55228	0.991289	1.19956	2.70417	3.13368
RP11-803D5.1	3.05247	2.83866	3.87852	3.66925	3.32126	1.81688
RP11-805J14.5	2.15551	2.02899	1.72018	2.00105	1.33287	2.20679
RP11-810K23.10	-6.68418 ?		0.623914	-0.40821	0.757612	0.522196
RP11-815I9.4	1.13537	0.611871	1.27058	1.31113	1.40933	0.726953
RP11-815J4.6	2.99663	3.10625	1.79418	2.09884	2.02315	1.94585
RP11-817I4.1	1.56305	1.81331	1.76487	1.72409	2.30549	1.76905
RP11-817I4.2	1.30659	1.3381	1.45314	1.42259	2.36761	1.33772
RP11-819C21.1	-0.791952	-0.790185	0.882627	0.902467	1.1211	0.769095
RP11-819M15.1	0.676574	0.854666	-0.303246	0.20137	0.579441	1.45063
RP11-81A1.6	2.18968	2.12963	2.17405	2.12001	1.29393	0.270672
RP11-822E23.8	-0.307901	-0.108884	-3.32027	-0.989151	0.339525	1.37558
RP11-823P9.3	-0.386559	-0.466987	1.15887	0.967611	0.327503	-0.4389
RP11-829H16.2	0.370388	0.592876	0.684774	0.994247	1.89106	1.88747
RP11-82H13.2	0.0316352	0.719131	-0.271435	0.0510519	1.12949	0.819783
RP11-832N8.1	1.95928	2.13508	1.55901	1.94335	1.67309	1.90264
RP11-834C11.4	1.31777	1.46201	-1.79072	-1.30113	-0.450674	0.0213376
RP11-83A24.2	2.08884	2.15188	2.44585	2.22658	2.03923	1.52391
RP11-843B15.4	0.430296	0.766044	0.156448	0.176693	1.01923	0.668401
RP11-848G14.5	1.41382	1.27675	2.44893	2.05243	2.79462	1.88889
RP11-849F2.7	-0.18843	-0.258677	0.732842	0.522116	1.15699	0.871576
RP11-849H4.2	1.31777	1.29209	1.6426	1.35065	0.210164	0.253699
RP11-849H4.4	2.9607	3.09816	3.01474	3.0579	2.30541	1.77308
RP11-849N15.3	1.61792	1.29635	-2.30935	-1.24688	0.0514974	-0.302441
RP11-863K10.4	1.13876	0.925605	0.609121	0.978166	1.40625	1.60376
RP11-863P13.3	0.462136	0.716051	-0.25649	0.46777	-6.64581 ?	
RP11-864I4.1	2.35383	2.50513	2.51476	2.80056	1.90808	1.3057

RP11-864N7.2	5.30296	5.29147	4.68793	5.06357	6.24436	7.17489
RP11-867G23.3	0.636116	0.376157	0.597488	0.519954	-0.077076	-0.520316
RP11-872J21.5	0.743937	0.727049	0.571444	0.57776	0.185296	-0.0341529
RP11-876N24.3	1.04917	0.920895	0.768146	1.00472	-0.729349	-1.99869
RP11-876N24.5	1.06916	0.859151	0.838911	0.698601	-0.374735	-1.06257
RP11-879F14.2	1.48029	1.70711	-0.00120949	0.107005	-2.85193	-2.8983
RP11-87H9.4	2.60699	2.62728	1.82773	1.70499	4.93307	4.19264
RP11-87H9.5	0.0289651	-0.233871	-1.59814	-2.30078	2.14384	1.52214
RP11-884K10.7	1.32887	1.39518	0.225978	0.158078	0.625168	0.847941
RP11-887P2.3	4.25971	4.17905	4.76694	5.09883	6.09696	5.73658
RP11-889L3.1	5.4417	5.42584	4.85072	5.35302	5.76685	6.15945
RP11-88I18.2	1.05066	0.845671	0.199688	0.412966	3.34288	2.85163
RP11-890B15.3	1.92665	1.9035	2.4312	2.48738	2.86522	2.95507
RP11-894P9.1	2.64522	2.52869	2.05967	2.03585	2.90384	1.62424
RP11-8H2.1	0.776727	0.97184	-0.509247	0.57776	0.529601	0.864232
RP11-90H3.1	3.56163	3.70172	3.36786	3.51823	3.58384	4.05176
RP11-90L20.2	0.684702	0.61405	0.64945	0.57776	-2.13612	-2.12114
RP11-92K2.2	4.97327	4.95589	4.19365	4.55887	4.89134	6.24284
RP11-932O9.10	0.558395	0.727049	0.636738	0.749225	-0.777433	-2.38405
RP11-936I5.1	0.563861	0.407614	0.56222	0.392724	0.285911	-0.499608
RP11-956A19.1	0.0289651	0.223769	-0.0541032	0.0410467	0.539452	-0.536593
RP11-958N24.1	-0.00348109	-0.479329	0.951901	1.25515	-0.284875	0.245252
RP11-95D17.1	1.9484	1.96763	0.174138	0.100749	1.56854	1.35942
RP11-95M15.2	1.53762	1.63237	0.768908	1.18402	1.32121	1.19776
RP11-966I7.2	-1.99808	-2.37465	0.416483	0.334214	0.901996	0.224139
RP11-96D1.10	-0.0537932	-0.476165	0.90411	1.10491	0.432275	-0.574854
RP11-96D1.11	-0.256202	0.0542233	0.712763	0.124566	0.0274089	0.439304
RP11-96D1.8	0.976371	1.34425	1.57048	1.69752	1.88935	1.04096
RP11-96H19.1	-0.791952	-0.818747	1.90397	1.68562	-0.615709	-0.899103
RP11-96K19.4	-0.470075	-0.233871	0.986462	0.773887	-0.932127	-2.00571
RP11-972P1.7	0.385928	0.285071	0.483891	0.470115	0.557601	-0.956507
RP11-97C16.1	0.470938	0.166625	-0.095919	-0.301307	0.246688	-0.0627204
RP11-97O12.6	0.133932	-0.122851	0.782333	0.317536	0.0812851	-1.31405
RP11-981G7.1	-0.998365	-0.214764	0.156448	0.020584	1.83157	1.66836
RP11-981G7.6	-1.94365	-2.06994	-1.72363	-1.30113	1.60651	1.00759
RP11-983P16.4	2.9056	2.6455	2.49185	2.45248	2.0298	1.62471
RP11-98I9.4	1.09678	1.00482	1.52608	1.69391	1.75043	1.61625
RP11-98J23.1	1.99914	1.7367	3.28702	2.86009	2.42525	1.30082
RP11-98J23.2	0.590147	0.502616	1.47686	1.21552	0.817975	1.61167
RP11-9E17.1	2.51525	2.30866	1.91979	1.98395	1.5394	1.42261
RP11-9L18.2	4.47518	4.57718	4.29826	4.62408	4.40376	3.97639
RP13-1032I1.7	1.94458	1.91676	1.47532	1.60136	1.92062	1.63198
RP13-104F24.1	-0.633667	-0.0472072	0.213055	0.466591	0.643902	0.432446
RP13-104F24.2	0.798084	0.514188	1.6564	1.83678	1.88479	1.11139
RP13-104F24.3	1.68293	1.70395	1.33783	1.49028	2.05016	1.73886
RP13-258O15.1	3.19787	3.20112	3.12224	3.41702	4.22986	5.18669
RP13-383K5.4	1.54639	1.80278	0.401532	0.927388	2.73519	3.24364
RP13-39P12.2	0.0811896	0.243279	0.812802	0.599089	0.718956	0.0963293
RP13-39P12.3	0.729453	0.576822	1.86742	1.7747	0.733996	1.46499
RP13-444K19.1	0.415705	0.453502	-0.287228	0.626224	0.73014	1.62833
RP13-516M14.8	0.458781	0.324626	1.35197	0.866006	1.58239	1.18945
RP13-735L24.1	3.27947	3.69494	-2.36195	-2.7648	-3.37228	-3.93762

RP13-88F20.1	2.09963	2.51729	2.55209	3.17234	2.98222	2.89425
RP13-890H12.2	-1.55256	-1.35527	-0.144387	-0.0380404	0.844603	0.896574
RP2	2.91931	3.05876	3.30029	3.81747	3.52705	3.63314
RP3-323A16.1	4.02652	4.07342	2.00938	2.56335	2.78063	2.84736
RP3-329A5.8	0.768595	0.868134	1.3479	0.724135	3.3098	2.10709
RP3-331H24.6	3.42892	3.00883	2.16522	2.05107	2.75544	2.47321
RP3-331H24.7	2.53215	2.36054	1.42385	1.2041	2.01214	1.92993
RP3-337O18.9	1.23322	1.25939	0.511063	0.764924	0.160275	-0.251049
RP3-340B19.2	1.93334	1.87709	1.99145	2.37194	2.28508	3.44289
RP3-347M6.2	1.53621	1.71933	1.37111	1.78092	1.72037	1.21301
RP3-368A4.5	3.14765	3.24521	4.36558	4.16818	4.44542	3.55292
RP3-368A4.6	3.47335	3.46048	4.62543	4.36081	4.37792	4.09903
RP3-394A18.1	4.14007	4.18599	5.12061	4.85212	4.09626	3.6397
RP3-405J10.3	1.78476	1.58158	2.00137	2.15329	0.587701	0.200291
RP3-417G15.1	5.44276	5.44692	5.09089	5.29373	6.19741	6.93273
RP3-425C14.4	1.66727	1.72206	2.0549	1.97335	2.69505	2.66833
RP3-425P12.4	2.91061	2.72897	1.98634	2.03587	2.06027	1.97056
RP3-426I6.2	-0.697341	-0.521923	-0.598354	-0.24681	0.776887	1.05084
RP3-428L16.2	-0.914271	-1.25306	-2.48229	-1.85786	4.69541	5.07138
RP3-437C15.1	2.66982	2.17285	1.24455	1.83046	2.62549	2.58879
RP3-449O17.1	1.60218	1.21219	1.40502	1.08021	-0.0172083	-1.06302
RP3-452M16.1	1.07717	1.36223	0.894899	1.45325	1.73236	2.3946
RP3-461F17.3	2.14519	2.26329	1.31632	1.84903	1.47668	2.41309
RP3-468K18.6	0.207918	0.357428	-0.428455	-0.40821	0.509706	0.100762
RP3-469D22.1	0.37669	-0.0181035	1.52439	2.38253	2.37699	3.27036
RP3-486I3.4	3.80801	3.79849	3.08212	3.59228	4.46507	5.16621
RP3-497J21.1	-0.138964	-0.84529	2.19887	2.63572	0.954018	1.17384
RP3-508I15.9	1.39869	1.37335	1.47445	1.35246	-0.401587	-1.42604
RP3-514P16.1	1.58436	1.74957	1.90794	2.51591	1.7091	2.05446
RP4-530I15.9	1.66407	1.53018	2.39494	2.55176	2.00474	1.25833
RP4-539M6.19	1.1009	0.803946	-1.84577	0.751524	0.31808	0.2885
RP4-555D20.1	0.87014	1.06117	1.45381	1.70941	1.15255	0.765238
RP4-555D20.2	2.73991	3.03491	3.53742	3.8356	3.43	3.06556
RP4-563E14.1	0.567789	0.635262	0.242852	0.0612232	-0.356358	-1.4584
RP4-564F22.5	1.07577	1.15925	-0.0541032	0.698601	0.270529	-0.577229
RP4-569M23.2	1.88215	1.9925	2.20451	1.69211	1.24663	1.1007
RP4-569M23.4	1.09539	0.559609	0.986462	0.724135	-0.267833	-0.577229
RP4-569M23.5	1.01134	0.813344	1.20023	0.631505	0.210164	-0.643045
RP4-575N6.2	-0.366535	-0.437066	0.0639033	0.263263	1.15534	1.52361
RP4-583P15.15	1.2347	1.04303	1.12883	1.30249	1.61995	0.872372
RP4-591C20.9	2.78273	2.45006	2.541	2.6856	1.5829	0.951558
RP4-591N18.2	0.430296	0.537236	0.386425	-0.154483	-0.510371	-1.70626
RP4-592A1.2	4.08682	4.17879	3.65277	3.97515	4.4606	4.76678
RP4-595K12.2	-0.470479	0.0648139	-0.663086	0.315218	-0.336315	1.07087
RP4-604A21.1	0.147945	0.253372	-0.325375	0.276794	1.63935	1.3217
RP4-614O4.13	-0.17598	-0.617481	0.990439	0.966807	0.1396	-0.799586
RP4-621F18.2	0.292735	0.896132	1.4209	1.60958	0.111525	0.159022
RP4-635A23.6	1.26097	1.09945	1.31637	0.989263	0.83161	0.483199
RP4-639F20.1	-0.129081	-0.105152	-0.455418	-0.201784	2.69505	2.82857
RP4-641G12.4	0.922145	1.27847	-0.0698743	0.579702	0.484056	0.457142
RP4-657D16.3	0.855192	1.11479	1.47626	1.50086	0.934698	-0.280422
RP4-665J23.1	1.15272	1.01295	1.66826	1.57771	-1.10542	-1.24695

RP4-669L17.10	1.66367	1.59445	0.90995	0.92448	0.535316	-0.785617
RP4-671O14.6	-0.359059	-0.531171	1.04535	1.03078	-4.33548	?
RP4-682C21.2	1.98932	2.02757	1.93381	2.04364	2.16661	2.09799
RP4-706A16.3	6.49913	6.43905	5.34947	5.86521	5.8881	6.55901
RP4-714D9.5	1.28396	1.42594	0.956086	0.786061	0.625168	0.359476
RP4-717I23.3	1.86324	1.75635	3.00383	2.93557	2.71256	2.01491
RP4-724E16.2	1.27756	1.61163	2.41999	2.05392	1.59937	0.586856
RP4-730K3.3	-0.144345	-0.140771	0.460428	0.317536	-0.00196773	-0.0918635
RP4-742J24.2	2.07471	1.87749	0.439049	0.244607	2.20391	1.82955
RP4-756G23.5	-0.367715	-0.11187	-0.0740401	0.485458	0.32633	0.393844
RP4-756H11.5	2.7534	2.72258	2.54008	2.83425	3.18965	3.50208
RP4-758J18.13	1.20183	1.33161	0.324373	1.0511	0.83161	0.863209
RP4-758J18.2	2.88293	2.85578	3.21537	3.47264	3.57165	3.68653
RP4-761J14.8	1.91145	2.2075	1.60282	1.00457	1.1698	1.82412
RP4-765C7.2	0.14672	0.489337	2.67482	3.17625	3.66494	4.58281
RP4-769N13.6	1.22687	1.37673	1.75204	1.65671	1.76384	1.95488
RP4-773N10.4	3.09736	3.05089	2.90857	3.12	2.67509	1.84355
RP4-775C13.1	4.25631	4.37875	2.83669	3.1979	4.35671	4.52766
RP4-778K6.1	0.549235	0.560001	0.218868	0.606044	1.94305	1.90064
RP4-781K5.2	0.782023	0.93946	1.45403	1.48894	1.61053	0.966822
RP4-785G19.2	1.28757	1.71789	0.47228	0.838573	1.5137	1.84288
RP4-791C19.1	0.736085	1.37774	-0.496037	-0.455228	0.458014	0.868292
RP4-798A10.2	1.15185	1.13173	1.44923	1.57534	0.99041	0.315578
RP4-800G7.2	2.22813	2.15152	1.96922	2.16324	1.6637	1.00248
RP4-814D15.1	0.85642	0.848502	1.30327	1.05971	0.804756	0.837935
RP4-814D15.2	0.634798	0.45982	1.07448	0.606243	-0.411902	-0.457667
RP5-1009N12.1	0.927995	1.03116	-0.177962	0.591067	0.650434	1.02717
RP5-1014D13.2	4.39496	4.42333	3.48728	3.59432	3.80587	3.41523
RP5-1021I20.5	1.23312	1.04232	0.126101	-0.146087	0.837354	-0.116012
RP5-1033H22.2	0.427338	0.144386	1.3278	1.52384	0.182107	-0.525621
RP5-1039K5.12	3.19157	3.24783	1.31734	1.0044	2.24788	1.77208
RP5-1039K5.19	7.55749	7.35837	3.16033	3.08416	2.70629	1.81126
RP5-1041C10.3	-0.113974	-0.139533	0.612258	0.619178	-0.216341	-1.53419
RP5-1042I8.7	0.159022	-0.214764	0.00643448	-0.086319	-0.0903144	0.270672
RP5-1056L3.3	1.26625	1.50054	1.91151	2.44798	2.8465	3.57843
RP5-1068E13.7	1.07577	0.988427	0.636738	1.01017	-0.594016	-1.18284
RP5-1074L1.4	3.70713	3.79218	3.42566	3.0933	3.19612	2.39659
RP5-1085F17.3	3.33159	3.31688	2.4312	2.53143	2.6135	1.55071
RP5-1086K13.1	0.243547	0.61405	-2.01303	-1.71605	2.2254	1.86317
RP5-1099D15.1	1.98393	1.93504	1.55127	1.82915	2.79671	2.66321
RP5-1112D6.7	-0.255978	-0.27286	-0.276471	-0.154483	0.749157	0.200291
RP5-1112D6.8	1.03142	1.17997	1.28056	1.07475	2.00881	1.48005
RP5-1126H10.2	0.85239	0.786111	1.05558	0.593469	0.718842	0.099376
RP5-1142A6.2	1.36334	0.936327	0.197891	0.207056	-0.276835	-0.395562
RP5-1142A6.9	2.31054	2.13523	1.86285	1.96861	1.20351	0.531988
RP5-1186N24.3	-0.494851	-0.855202	-0.205608	0.118413	0.827998	1.71308
RP5-821D11.7	1.66853	1.53968	2.06122	2.07328	1.07176	0.723305
RP5-827C21.1	0.580145	0.485035	-0.894282	0.0224033	0.750358	1.51616
RP5-849H19.3	0.460879	0.370142	0.610974	0.430424	0.0122397	-1.61882
RP5-850E9.3	2.94372	3.3056	4.88963	5.68741	3.51263	3.4542
RP5-854E16.2	0.536898	0.750426	0.374778	0.850223	0.713265	0.143694
RP5-857K21.11	4.06744	4.05482	4.57219	4.49665	4.87447	4.03306

RP5-857K21.6	8.35037	8.19948	9.18483	8.96354	8.84375	8.64566
RP5-857K21.7	3.64408	3.3982	4.38524	4.12357	4.5503	3.28757
RP5-867C24.1	-0.159773	-0.0598438	0.584741	0.334214	-0.0163223	-0.536593
RP5-881P19.7	-0.255978	-0.122853	1.0357	0.786061	-1.07506	-2.00571
RP5-884M6.1	1.61384	1.7515	-1.65952	-0.086319	-6.64581	?
RP5-890E16.2	1.34536	1.64571	0.517004	0.999755	-0.729347	-0.799586
RP5-894A10.2	0.684702	0.740617	-0.346777	-0.201784	0.449514	-0.183001
RP5-894A10.6	1.63187	1.59247	1.20023	0.989263	1.65713	1.24753
RP5-908M14.10	0.684702	0.727049	0.474784	0.476226	-0.200727	-1.24695
RP5-908M14.9	0.640852	0.548466	-0.757028	-0.716287	0.448309	0.315752
RP5-940J5.6	2.30097	2.40761	2.03924	2.10998	2.37777	2.03783
RP5-961K14.1	1.13354	1.48858	0.455607	1.06891	0.8595	0.584626
RP5-965G21.3	3.26292	3.49664	-2.27595	-1.42267	-0.797576	-1.70626
RP5-965G21.4	2.84782	3.13893	-1.93506	-1.30113	0.0263185	-0.421131
RP5-965G21.5	2.11472	2.041	-3.59683	-3.78492	-1.45047	-2.53597
RP5-965G21.6	1.88966	1.57611	-2.27595	-0.938639	-0.878698	-1.38442
RP5-967N21.11	0.900281	1.06853	0.980757	0.967383	0.86132	0.262565
RP5-984P4.6	1.95199	1.49719	-0.539572	-1.16509	?	?
RP5-991G20.1	1.87327	1.73733	2.68959	2.66382	1.69249	1.62367
RP6-109B7.2	-0.255978	-0.39658	0.27602	0.300663	-0.0163223	0.293394
RP6-159A1.2	1.66836	1.6861	1.19147	1.65477	1.65399	1.83816
RP6-218J18.2	3.4636	3.57547	3.09458	3.31302	3.51702	3.02833
RP6-99M1.3	0.558395	0.444137	0.571444	0.549472	0.782702	-0.899103
RP9	3.09215	3.0288	2.26014	2.3877	2.71692	2.66608
RP9P	1.34514	1.42048	1.79282	2.09005	1.4004	0.793488
RPA1	5.28821	4.55397	5.49332	5.14502	5.98603	5.11424
RPA2	4.89841	4.88255	3.07387	3.50581	4.781	5.24967
RPA3	3.88962	3.8702	3.98363	4.38298	3.77431	4.9281
RPAIN	3.92199	3.96902	4.21835	4.46621	4.98773	4.89697
RPAP1	3.70712	3.62197	3.46574	3.58117	3.88465	3.6618
RPAP2	4.12825	4.3453	4.65252	4.67744	4.63028	4.7981
RPAP3	4.81515	4.98423	4.35466	4.66182	4.32126	4.929
RPARP-AS1	2.16404	2.06778	1.37735	1.40658	1.40098	1.13384
RPE	4.87221	4.83349	5.42977	5.77842	5.58031	5.71716
RPEL1	1.49964	1.61693	1.89562	2.4247	2.2806	2.68603
RPF1	4.22423	4.29371	3.3749	3.89539	4.55157	5.19037
RPF2	4.26707	4.45362	3.17699	3.57982	5.77592	6.19408
RPF2P1	0.404107	0.887642	0.168	0.831091	2.08051	2.57017
RPGR	2.18052	2.30204	1.68687	1.8454	1.90957	2.14505
RPGRIP1L	3.39103	3.55813	4.3566	4.77152	4.42648	4.77003
RPIA	2.99267	2.82718	1.74725	1.9191	2.79503	2.83245
RPL10	7.13163	7.01035	6.49974	7.00441	7.75918	8.21368
RPL10A	6.98899	6.95949	6.06864	6.52772	7.34888	7.94608
RPL10AP2	1.91098	2.20097	1.25082	1.94294	2.49306	2.97023
RPL10AP6	5.24553	5.20412	4.29327	4.78931	5.55811	6.34317
RPL11	8.05327	7.93704	7.22434	7.60112	8.59921	8.91734
RPL12	5.96237	5.93303	5.05669	5.60496	6.81121	7.52229
RPL12P1	0.0253265	-0.0193546	-0.208589	-0.484296	0.645794	0.827941
RPL12P4	4.78803	4.72083	4.10816	4.49551	5.83069	6.36942
RPL13	7.21987	7.14735	6.46042	6.83573	7.05186	7.86249
RPL13A	7.13828	7.10632	6.25651	6.76167	8.22209	8.85933
RPL13AP25	1.80026	2.03674	1.69983	2.12049	3.23242	2.71534

RPL13AP5	8.17503	8.00057	7.76468	8.14319	9.20434	9.7923
RPL13AP7	0.583163	0.637026	-0.155724	0.69496	1.6418	1.52242
RPL13P12	6.4817	6.41732	5.86685	6.27505	6.5366	7.18019
RPL14	6.89963	6.91264	5.94813	6.43027	7.4175	7.89722
RPL14P1	6.85716	6.80908	5.92184	6.39651	7.35909	7.77026
RPL15	8.17582	8.03936	7.22388	7.58362	8.95861	9.49858
RPL15P3	6.35943	6.22896	5.41387	5.85434	7.08163	7.78384
RPL17	5.79383	5.72577	4.78168	5.23676	5.02408	5.85184
RPL17-C18orf32	3.91903	4.11595	2.84971	3.39693	3.26728	4.40982
RPL18	6.62639	6.43788	6.27565	6.77731	7.45841	8.04129
RPL18A	5.16731	5.09189	4.40384	4.68023	5.6634	6.14199
RPL18AP3	6.19322	5.94273	5.47361	5.90879	6.96923	7.64847
RPL18P13	0.973406	0.792556	-0.0748604	0.563939	1.15373	1.09503
RPL19	8.51361	8.46886	7.83339	8.32232	8.84607	9.26546
RPL21	3.70042	3.75567	3.505	3.93914	3.8182	4.44806
RPL21P119	1.50573	1.50726	1.42342	1.96811	2.10526	2.07118
RPL21P75	1.14075	2.59253	2.34614	2.36595	2.23739	2.75229
RPL22	5.91932	5.8915	5.32564	5.55824	6.49692	6.98259
RPL22L1	3.5524	3.60861	2.12028	2.57418	3.24658	4.71534
RPL23	7.78799	7.73564	7.21536	7.60414	8.34055	9.18581
RPL23A	5.91741	5.74877	5.53774	6.00426	6.56611	7.08734
RPL23AP2	2.75003	2.8374	2.70232	3.139	3.45908	3.95249
RPL23AP42	7.38227	7.32824	7.0752	7.51466	8.1443	8.58807
RPL23AP65	2.75439	2.52776	2.22105	2.77641	3.31267	3.95134
RPL23AP7	0.253336	0.705253	0.749817	0.90599	1.68869	1.91926
RPL23AP74	0.923317	0.976921	0.992239	0.883285	1.35372	1.80049
RPL23AP82	2.5492	2.62192	2.39077	2.64002	2.66116	3.16314
RPL24	5.99073	5.92461	5.86657	6.19431	6.2374	7.09359
RPL24P2	0.912236	0.792772	0.908567	1.38832	1.18672	1.89794
RPL24P4	4.7042	4.75398	4.59941	5.09411	5.11039	5.74363
RPL24P8	3.91856	3.8524	3.70843	4.15617	4.0793	5.10022
RPL26	7.20795	7.09626	6.95646	7.38982	8.18485	8.96667
RPL26L1	2.83619	2.81	3.00033	3.25909	1.86319	2.48264
RPL26P19	5.34112	5.3363	5.20938	5.60953	6.40836	7.28403
RPL26P30	2.03228	2.06626	2.32197	2.56456	1.37877	1.97347
RPL27	6.59562	6.45411	6.25605	6.73422	7.48502	8.25206
RPL27A	7.34717	7.27969	6.45629	6.84643	7.68833	8.24866
RPL28	6.03108	5.78323	5.55545	5.91689	6.79709	7.13587
RPL29	6.92919	6.66573	5.95438	6.20638	7.13678	7.96895
RPL29P11	1.29805	1.24416	0.354373	1.26193	1.79471	2.90262
RPL3	8.02951	7.87215	7.00247	7.37214	7.98849	8.26816
RPL30	6.98282	7.00081	6.68306	7.20546	7.85685	8.78857
RPL30P3	-0.376978	-0.253233	-0.568612	0.120113	0.732078	1.40185
RPL30P4	1.4816	1.59589	1.43909	1.93666	2.46061	3.50106
RPL31	7.3161	7.25971	6.40209	6.78266	7.77263	8.97454
RPL31P49	1.04504	1.29245	0.139469	0.91071	1.65597	2.71858
RPL31P63	0.776449	1.02698	0.0858869	0.393054	1.37672	2.30734
RPL32	7.47503	7.40289	6.52244	6.87327	8.07267	8.90936
RPL32P3	2.61834	2.78746	3.25735	3.33205	2.78264	2.19417
RPL34	6.27668	6.16264	5.82209	6.1043	6.59986	7.7211
RPL34P18	3.00767	2.98554	2.70998	3.04571	3.40768	4.50416
RPL34P27	0.0596183	0.00167256	-0.537837	-0.257399	0.434647	0.863153

RPL34P33	-0.186367	-0.313108	-0.762559	-0.375403	-0.0681914	1.04633
RPL35	6.54419	6.4562	6.01587	6.36966	7.07001	8.19677
RPL35A	6.31125	6.26759	6.35112	6.87964	7.65178	8.63675
RPL35AP21	-1.08408	-0.555757	-0.521987	-0.201784	0.482012	1.17598
RPL35P1	1.58531	1.43025	0.845743	1.40555	2.10967	3.27578
RPL35P2	2.38695	2.53653	1.95239	2.31637	3.16305	3.7789
RPL35P5	4.39806	4.47289	4.03192	4.32511	4.92544	5.96027
RPL36	6.22749	6.09217	5.54048	5.97889	7.48874	8.16768
RPL36A	3.2809	3.3348	3.04547	3.11972	4.06335	4.00195
RPL36AL	5.58805	5.37792	4.16188	4.41277	5.95531	6.0002
RPL37	7.42863	7.28769	7.15922	7.56632	8.30957	9.00757
RPL37A	7.96916	7.86987	7.5096	7.99427	8.66408	9.35092
RPL37AP1	2.30669	2.64264	2.27273	2.7611	3.41592	3.49098
RPL37P2	1.66047	2.00508	1.77188	2.46638	2.92606	3.26762
RPL37P23	4.12767	4.07002	4.03668	4.50486	5.14545	5.72766
RPL37P6	1.6402	1.72495	1.4028	2.07867	2.54071	3.35511
RPL38	6.20642	6.15735	5.79338	6.27567	7.51643	7.93981
RPL38P4	0.183671	0.358419	-0.161006	0.632724	1.68402	1.41227
RPL39	5.7536	5.61237	5.48289	6.01043	6.71224	7.92151
RPL39L	-5.69162	-5.45651	-0.0336404	0.300838	0.909612	1.76092
RPL39P3	3.89937	3.86837	3.78354	4.14661	4.93833	6.00889
RPL3P2	2.57656	2.79632	1.62951	2.22998	2.73996	2.72103
RPL3P4	6.68674	6.60845	5.77072	6.03284	6.72379	6.91302
RPL3P6	0.840145	0.938572	1.65571	1.40691	0.698645	0.270672
RPL3P7	2.22958	2.32922	1.32308	1.81795	2.3099	2.74064
RPL4	8.91094	8.88779	8.04367	8.53302	9.20517	9.70035
RPL41	8.82035	8.73243	7.73563	8.25884	9.03076	9.73352
RPL41P2	0.0797843	0.364606	-1.18873	-0.0132134	0.24468	1.15896
RPL4P3	1.04386	1.18651	0.276127	0.487404	1.31624	1.40828
RPL4P4	7.19866	7.20495	6.37096	6.87606	7.52471	8.09854
RPL4P5	4.65051	4.69668	3.81941	4.37914	4.99589	5.43
RPL4P6	0.108397	0.013012	0.662041	0.659431	-0.184426	-1.31405
RPL5	7.78773	7.625	7.22849	7.65032	8.1048	8.59448
RPL5P1	3.35284	3.31785	2.67327	3.32915	3.63405	4.14004
RPL5P17	1.5397	1.81426	1.10933	1.42263	1.80317	2.1569
RPL5P23	1.95668	1.98549	1.54855	1.94862	2.32542	2.81733
RPL5P29	0.218025	0.339103	-0.391995	0.0408504	0.829119	0.499619
RPL5P34	5.19475	5.06524	4.72183	5.16383	5.65911	5.98149
RPL5P4	3.41579	3.45686	3.08835	3.34535	3.87081	4.12768
RPL6	7.59063	7.45312	6.55783	6.8004	7.54189	7.84768
RPL6P27	7.73078	7.52944	6.6423	7.01589	7.63679	8.19603
RPL7	4.504	4.4931	4.08981	4.69756	5.26369	5.995
RPL7A	6.95086	6.80979	6.32128	6.72836	7.18659	7.49542
RPL7AP11	2.22579	1.95023	1.93764	2.05524	2.04931	2.38701
RPL7AP30	4.92578	4.80788	4.2721	4.68686	5.31516	5.54496
RPL7AP31	2.56511	2.46534	1.49078	1.9614	2.46718	2.50179
RPL7AP6	7.11326	6.98323	6.456	6.9499	7.40242	7.7947
RPL7AP66	2.23912	2.30817	1.64159	1.74722	2.09233	2.67742
RPL7L1	6.5439	6.70129	5.9036	6.54362	7.36155	7.77341
RPL7L1P8	0.999278	1.05834	-0.0382848	1.04348	1.72677	1.84426
RPL7P1	6.2196	6.15588	5.81354	6.38298	6.82101	7.9575
RPL7P21	1.90998	2.01282	1.69486	2.14052	2.60603	3.01229

RPL7P22	1.41973	1.76379	0.671167	1.59613	2.16123	2.59844
RPL7P23	-0.261281	0.191499	-0.0520721	0.297896	0.628475	1.00249
RPL7P9	7.59669	7.52235	7.27572	7.74436	8.28545	9.25565
RPL8	7.68001	7.60245	7.35625	7.82252	8.62839	8.89334
RPL9	4.43136	4.42574	2.77911	3.11481	3.92359	4.42499
RPL9P7	3.33625	3.61422	2.95615	3.29261	4.18554	5.16561
RPLP0	8.50543	8.44231	7.44093	7.87313	8.49081	9.18005
RPLP0P6	8.03138	8.02755	6.99403	7.49599	8.16788	8.78735
RPLP1	6.98857	6.66433	6.17672	6.4829	7.06583	7.61789
RPLP2	7.17496	6.97895	6.5377	6.86364	6.72294	7.3019
RPN1	7.19814	7.18317	7.36465	7.7696	7.01778	7.39222
RPN2	8.63458	8.53953	8.12544	8.46577	7.33654	7.15643
RPP14	4.52792	4.68562	3.65252	3.98128	4.70492	4.83728
RPP21	1.9552	1.8212	1.14492	1.72407	2.70348	3.33219
RPP25L	1.73557	1.49719	1.66826	2.08595	2.42451	3.28197
RPP30	3.69752	3.80042	3.15633	3.56183	3.73201	4.15592
RPP38	2.20425	2.30365	2.13995	2.46602	2.22323	2.57847
RPP40	1.46083	1.79928	1.52387	2.1296	2.74271	3.71113
RPPH1	11.0888	10.7731	11.2199	11.4596	11.4687	13.3141
RPRD1A	5.05663	5.3107	4.59373	4.69368	5.02923	5.18885
RPRD1B	4.85358	4.69326	4.42195	4.61058	3.96063	3.971
RPRD2	4.60756	4.54136	4.40425	4.5278	4.44559	4.36875
RPS10	4.99086	4.82894	4.3659	4.75968	5.78197	6.66112
RPS10-NUDT3	0.679991	0.751301	0.766197	1.35555	2.76851	1.74771
RPS11	7.70547	7.57334	7.22317	7.68281	8.35187	8.92697
RPS11P5	4.86146	4.91258	4.3872	4.83204	5.60761	5.91472
RPS12	5.87918	5.8215	5.28972	5.82913	6.99435	7.97205
RPS12P23	0.151002	0.478785	-0.102484	0.141027	1.32815	1.56766
RPS13	6.9033	6.85904	6.03669	6.54381	7.04116	7.88335
RPS13P2	5.12786	5.23801	4.51997	4.96119	5.30269	6.04306
RPS14	7.24709	7.11911	7.25091	7.70651	8.03235	8.9667
RPS15	5.57658	5.4184	4.83266	5.44577	6.5665	6.99196
RPS15A	6.90902	6.75311	6.70922	7.07722	7.12335	8.06743
RPS15AP1	2.0273	2.2207	1.99067	2.60544	2.39059	3.23965
RPS15AP11	0.875269	0.807165	0.770897	1.28173	0.919355	1.89296
RPS15AP12	1.22663	0.994109	0.976628	1.28719	1.6032	2.13055
RPS15AP24	1.74305	1.81444	1.59342	2.1201	1.94606	2.86371
RPS15AP38	2.98412	2.92997	3.05519	3.35555	3.42686	4.04124
RPS16	6.18596	5.95867	5.81123	6.06643	6.96822	7.29291
RPS17	8.19066	8.18108	7.47645	7.87172	8.61774	9.43081
RPS18	7.43018	7.4095	6.76374	7.27118	8.5289	9.33579
RPS18P12	0.52693	0.895194	0.237368	0.380829	1.64978	2.21685
RPS18P9	-0.0429175	-0.000222192	-0.453822	0.0686846	1.06055	1.0995
RPS19	7.7066	7.54235	6.69551	7.04154	7.93099	8.63782
RPS19BP1	4.65792	4.72636	3.83607	4.21423	4.45277	5.62026
RPS19P1	3.16961	3.36205	2.53037	2.83854	3.83305	3.81798
RPS19P3	1.06643	1.05166	0.141629	0.716122	1.75399	1.94362
RPS2	7.40462	7.45544	7.19526	7.62051	8.24591	9.01669
RPS20	7.24058	7.10666	7.23688	7.4863	7.57203	8.2971
RPS20P10	1.57882	1.49108	1.43995	1.73075	2.01825	2.71734
RPS20P14	3.56194	3.50948	3.6392	3.91012	4.43182	5.08206
RPS21	6.9136	6.8141	5.24602	5.60842	7.05875	7.89892

RPS21P4	2.98997	3.20518	1.41196	1.93685	3.21004	3.74995
RPS23	7.50857	7.39631	7.1132	7.593	8.31695	9.12052
RPS23P8	5.96426	5.88868	5.63388	6.11245	6.69533	7.47798
RPS24	7.40873	7.34408	6.89113	7.26575	8.26087	8.96335
RPS24P8	2.16484	2.49547	2.18792	2.19023	3.20072	4.12223
RPS25	6.74697	6.69434	6.20019	6.5322	6.86317	7.58039
RPS26	2.95491	3.03932	2.34885	2.48229	2.79521	3.33508
RPS27	3.35579	3.36636	2.46445	2.798	3.1584	4.26088
RPS27A	7.28589	7.22487	6.55953	6.90697	7.5108	8.1806
RPS27AP1	0.454534	0.59277	-0.454461	-0.0401527	0.200303	1.84282
RPS27L	5.59583	5.9991	4.02837	4.48269	5.4007	6.05897
RPS28	2.63673	2.44197	1.91092	2.33593	3.46384	3.90507
RPS28P7	3.53906	3.26185	2.93085	3.27467	4.44805	4.81098
RPS29	6.2244	6.03008	4.08458	4.52145	5.74118	7.09599
RPS2P46	5.13524	5.12078	4.85466	5.35572	5.88153	6.81881
RPS2P5	7.09633	7.14133	6.50904	6.96599	7.71931	8.37451
RPS2P55	3.83066	3.95239	3.76034	4.12557	4.80472	5.52735
RPS2P7	-0.0810582	-0.0275147	-0.567423	0.00288251	1.05477	1.28383
RPS3	8.34183	8.28876	7.57739	8.00929	7.65878	8.04792
RPS3A	6.20088	6.17174	5.80926	6.19801	6.36292	7.63568
RPS3AP26	6.05416	6.02149	5.59718	6.05017	6.22184	7.62278
RPS3AP5	2.07318	2.23517	1.69948	2.26951	2.34631	3.99533
RPS3AP6	7.33682	7.22659	6.87557	7.38573	7.5947	8.99867
RPS4X	8.03485	7.87419	7.47596	7.78237	8.7808	9.07078
RPS4XP1	1.1899	0.950244	0.794603	1.04857	1.87369	2.09069
RPS4XP11	2.84488	2.79863	2.47366	2.77616	3.54143	3.99381
RPS4XP13	0.460218	0.647462	0.178351	0.7564	1.37039	1.84142
RPS4XP14	0.869319	0.604698	0.284147	0.456627	1.01972	1.37511
RPS4XP16	0.0781999	0.434305	0.102819	-0.0412137	1.05999	1.19937
RPS4XP17	0.922441	0.999228	0.504824	0.80502	1.87869	1.83754
RPS4XP2	0.597927	0.713071	0.684891	0.973442	1.57617	1.56993
RPS4XP3	1.79414	1.79231	1.44745	1.88464	2.37403	2.74418
RPS4XP6	2.17125	1.94085	1.73914	2.08094	2.67858	2.9134
RPS4Y1	?	-6.45019	5.74794	6.15646	-6.64581	?
RPS5	7.00743	6.84118	6.18183	6.67384	7.59585	8.52735
RPS5P2	0.885949	0.850103	1.59787	1.38307	-0.777433	-2.00571
RPS6	8.07978	7.9902	7.2598	7.65077	9.59555	10.1835
RPS6KA1	5.10301	5.38616	3.5613	4.02431	3.94794	4.22992
RPS6KA2	1.2317	0.929533	0.674533	1.53507	-3.19956	-4.70296
RPS6KA3	6.40517	6.43458	5.36721	5.60067	5.0782	5.45378
RPS6KA4	3.31494	3.18462	2.88526	3.21085	3.27633	3.23581
RPS6KA5	3.53961	3.791	1.95028	1.91893	2.86887	2.95863
RPS6KB1	5.24976	5.27719	5.17616	5.48914	6.05091	5.89262
RPS6KB2	3.91194	3.59243	3.60927	3.75381	3.90384	4.03138
RPS6KC1	5.06204	5.16878	4.53587	4.76768	5.69419	5.99804
RPS6KL1	2.66944	2.51158	-0.539469	-0.616766	0.924716	0.702755
RPS6P25	-0.14695	-0.104488	-0.672391	-0.616087	1.30456	1.34689
RPS7	6.70318	6.65747	5.38423	5.78297	6.96818	7.93552
RPS7P1	7.31105	7.2304	6.06606	6.47622	7.67233	8.7641
RPS7P10	4.98007	4.84309	3.55768	4.00589	5.21327	6.41116
RPS7P11	4.2299	4.19558	3.23068	3.45553	4.61854	5.74329
RPS8	7.9312	7.88975	7.74672	8.15374	8.56567	9.17652

RPS9	6.59902	6.48583	5.93241	6.26742	7.11945	7.99746
RPSA	7.53444	7.5182	6.49888	6.89579	7.88989	8.61974
RPSAP15	3.67089	3.68138	2.64386	3.2454	4.1627	4.68146
RPSAP52	2.41207	2.33483	-0.539469	-0.55404	-0.0454657	0.224139
RPSAP54	4.34543	4.3053	3.58597	3.69685	4.38326	4.8322
RPSAP58	8.05199	7.98375	6.97801	7.42208	8.24357	9.0827
RPTOR	4.97786	4.58096	4.79053	4.55417	4.62747	3.84753
RPUSD1	2.96448	2.64569	2.44585	2.69853	2.89044	3.01781
RPUSD2	2.9138	2.9013	2.08796	2.25751	1.83157	2.18205
RPUSD3	4.47396	4.26336	3.68375	3.78447	4.36828	4.23139
RPUSD4	3.96545	3.92493	3.698	3.99589	3.79324	3.73906
RQCD1	5.4608	5.3959	5.25215	5.50771	5.65113	5.83967
RRAGA	4.23166	4.16741	3.99763	4.42255	6.35666	6.4585
RRAGB	1.69756	1.39518	2.32429	2.19497	2.6251	2.58792
RRAGC	3.55761	3.47085	3.25787	3.68557	4.14544	4.42254
RRAGD	3.83036	3.72698	-4.59508	-2.02079	-2.75246	-2.53597
RRAS	1.93394	1.70711	2.30433	2.80706	1.85935	2.15129
RRAS2	3.7974	3.75179	3.96142	4.46942	5.07172	5.21242
RRBP1	7.65325	7.5807	7.95364	8.0547	7.09701	6.46634
RREB1	4.61436	4.42818	4.70064	4.42255	4.17099	3.94074
RRM1	6.7715	6.81939	6.33439	6.74483	6.10432	6.44428
RRM2	6.27868	6.10036	5.69093	5.76863	6.40744	6.41848
RRM2B	4.78674	5.05678	4.18933	4.54759	4.64345	4.99631
RRM2P3	2.49702	2.63768	0.896497	0.98378	1.58256	1.28825
RRN3	4.67388	4.82782	4.16148	4.68234	5.00773	5.28628
RRN3P3	1.21798	1.20551	2.68801	2.66524	1.56363	0.725994
RRNAD1	2.5896	2.48301	2.64268	2.90688	2.76015	2.62383
RRP1	3.37238	3.30699	3.50472	3.79053	3.46897	3.2181
RRP12	5.17449	5.19446	3.79005	4.28588	5.4948	5.46071
RRP15	4.63798	4.80569	3.88121	4.10549	5.02487	4.74036
RRP1B	4.9918	4.93057	5.14905	5.42255	5.07441	4.53288
RRP36	4.67583	4.75755	4.22164	4.73254	5.69046	5.98582
RRP7A	4.50717	4.32833	5.12449	5.37446	4.00025	4.00422
RRP7BP	2.77447	2.75878	2.85078	2.9723	2.55789	1.95424
RRP8	4.57004	4.62462	3.89862	4.09452	3.90764	3.85362
RRP9	3.69216	3.50005	2.65569	3.17195	3.50081	3.95327
RRS1	3.69752	3.71831	2.35565	2.98657	3.63545	3.61935
RSAD1	4.66614	4.73191	3.36145	3.57066	3.94979	4.46821
RSBN1	4.13696	4.16012	3.89595	4.03965	4.24957	4.16672
RSBN1L	4.88728	4.6888	4.78658	4.70414	4.88849	4.56123
RSC1A1	2.1079	2.36917	1.92486	2.04798	2.29775	2.66873
RSF1	6.75926	6.61286	6.17734	6.12879	5.73902	5.56774
RSL1D1	6.32534	6.3625	5.66547	6.11757	6.60059	6.74087
RSL24D1	5.5128	5.4708	4.28978	4.90666	5.39996	6.25884
RSL24D1P1	1.44859	1.58854	0.360387	0.942225	1.37408	2.45826
RSL24D1P11	0.528041	0.593249	-0.563714	-0.486366	0.487208	1.12691
RSPH3	1.06916	1.14454	2.65569	2.86272	3.08116	3.22402
RSPH9	0.783608	0.477377	0.576571	0.58002	0.576319	0.16588
RSPRY1	4.10908	4.23151	4.89862	5.13374	5.58435	5.62698
RSRC1	4.16426	4.08845	4.63909	4.9838	5.45817	5.59234
RSRC2	5.90777	5.83292	5.45264	5.50633	5.83554	5.60047
RSRP1	4.93442	4.96175	5.06748	5.13693	6.17476	5.98949

RSU1	4.09045	4.3637	3.57802	4.36589	4.45668	5.11106
RTCA	4.08063	3.8903	3.75458	4.12597	4.45797	4.45819
RTCB	5.71507	5.75722	5.22904	5.80966	5.20347	5.62476
RTEL1	3.98711	3.65973	3.6992	3.80271	3.25335	2.54299
RTEL1-TNFRSF6B	2.71722	2.48282	2.48803	2.67095	1.9612	1.51724
RTEL1P1	-0.0252549	0.333229	0.906113	1.0038	-0.174024	-0.745912
RTF1	4.97157	4.96079	4.74202	4.93077	4.90049	4.72791
RTFDC1	5.61964	5.75388	5.06498	5.63222	5.93503	6.39354
RTKN	4.1589	3.85455	3.0453	3.18119	2.87692	2.69417
RTKN2	3.49814	3.53151	2.84715	2.74916	3.84252	3.76699
RTN2	0.61388	0.331407	0.860939	0.698601	0.529601	0.816919
RTN3	6.56133	6.63196	6.12987	6.33331	6.19335	6.22961
RTN3P1	3.31217	3.426	2.97574	3.0995	2.92034	2.93591
RTN4	6.53709	6.8038	6.95186	7.61596	7.11417	7.79759
RTN4IP1	1.59991	1.64571	1.87179	2.23074	1.87109	2.11985
RTP4	-2.17488	-1.76193	1.36341	1.55655	-1.07506	0.176042
RTTN	5.26743	5.21002	4.23749	4.37398	3.09291	2.83245
RUFY1	3.44924	3.37714	4.12852	4.4133	4.45883	4.33745
RUFY2	3.38348	3.6213	3.52941	3.62504	3.92116	4.06153
RUFY3	3.28677	3.51984	4.2759	4.23989	4.5399	4.58782
RUNDC1	3.50182	3.60054	3.44947	3.77076	4.3537	3.84018
RUNDC3B	0.604783	0.137189	0.558032	0.591699	-0.530833	-1.006
RUNX1	5.63127	5.65283	4.30129	4.49015	4.02443	3.31565
RUNX1T1	1.62738	1.40138	-5.1783	-5.10387	?	?
RUNX2	2.58864	2.55676	4.60356	4.76537	4.79964	4.36206
RUSC1	3.68938	3.37163	3.07879	3.37642	4.21315	4.1568
RUSC1-AS1	3.58992	3.69084	3.06427	3.14565	2.82164	2.7765
RUSC2	3.16667	2.96133	3.43303	3.72564	3.88657	3.73416
RUVBL1	4.95948	4.94566	4.6195	5.09736	4.71092	4.84113
RUVBL2	4.9022	4.7319	4.73309	5.10973	5.80218	5.99025
RWDD1	3.56051	3.64985	3.43253	3.81813	4.41507	5.23829
RWDD2A	2.40422	2.45006	1.83887	1.95189	1.98357	1.92993
RWDD2B	2.52563	2.62197	3.89462	4.28885	3.31841	3.84788
RWDD3	2.69895	2.76137	2.73147	2.87423	2.60984	2.9433
RWDD4	2.54774	2.7028	2.69062	3.17968	2.83944	3.33172
RWDD4P1	0.747147	0.618474	0.947995	0.700422	0.201358	-0.501884
RWDD4P2	1.38356	1.66283	1.11633	1.44209	1.22706	2.15937
RXRA	2.66725	2.40753	4.44675	4.80406	2.83755	2.96925
RXRB	3.2531	3.19767	3.36541	3.53578	3.62299	3.76221
RYBP	4.61436	4.58427	5.01553	5.32475	4.18752	3.8932
RYK	5.33455	5.32159	5.31817	5.39219	5.03005	4.99689
RYKP1	1.51704	1.6006	1.63208	1.96064	1.63957	1.17999
RYR2	1.82842	1.80864	-3.18222	-3.30006	-2.13612	-2.38405
RYR3	-0.0407328	0.305445	-1.13882	-0.751045	3.23299	2.58792
S100A1	1.19294	0.91173	-0.173663	-0.214791	-1.20407	-2.1163
S100A10	5.17594	5.43882	5.40892	5.95023	5.52735	5.53287
S100A11	4.99487	5.2131	5.47961	6.06045	5.04663	5.73004
S100A13	4.71797	4.69438	3.3238	3.79412	4.28992	5.36633
S100A16	5.66943	5.8686	2.76486	3.58987	6.16086	6.70981
S100A2	1.04917	0.996642	0.816542	1.69856	1.76598	2.52212
S100A3	1.53458	0.929533	1.08332	1.6921	0.362161	0.650921
S100A4	2.26383	1.89912	5.13275	5.60328	2.82754	3.22698

S100A6	8.50795	8.72685	7.96266	8.79957	8.96093	10.0975
S100B	4.61605	4.40055	?	?	1.06084	1.35942
S100PBP	4.59855	4.54207	4.48627	4.44349	4.65766	4.59019
S100Z	-0.113974	-0.354151	?	?	0.479334	0.0747657
S1PR1	-5.10914	-4.13839	0.386425	0.999755	3.4754	4.05816
S1PR2	4.25584	3.99658	2.06916	1.70499	1.9581	0.97985
S1PR3	1.87414	1.76118	5.57076	5.8098	4.34954	4.11178
S1PR5	1.12752	1.22371	2.13391	1.92462	-0.705893	-0.662104
SAA1	3.4895	1.75798	3.18558	3.74297	?	?
SAA2	1.11957	-1.63342	1.81804	2.89674	?	?
SAAL1	4.12189	4.24105	3.25319	3.64271	3.5196	3.83825
SAC3D1	2.27822	1.98839	1.37111	1.25754	1.2586	1.38079
SACM1L	4.94205	4.88256	4.6454	5.08899	4.54487	4.84307
SACS	7.91862	8.21524	6.54139	7.07079	7.8455	8.19211
SAE1	6.01534	6.02894	5.72561	6.09664	6.48415	6.80007
SAFB	5.15447	5.12489	4.66981	4.74474	5.15831	4.62837
SAFB2	4.31098	4.34455	4.04642	4.33894	4.9525	4.31184
SALL1	3.86698	3.79219	3.31831	3.28991	?	-4.70296
SAMD1	3.21382	2.88367	2.23012	2.31745	3.68399	3.2358
SAMD10	-0.413499	-0.354149	-1.229	-0.860651	0.997957	1.12623
SAMD11	-2.16599	-3.15088	-0.95233	-0.600125	1.93373	3.32305
SAMD12	3.01345	3.13148	1.82206	1.65939	1.64347	2.17595
SAMD15	0.62293	0.305445	-0.0134653	-0.0212303	1.06084	0.650921
SAMD4A	5.00352	4.53715	3.66822	3.76307	5.10745	3.93172
SAMD4B	4.08474	4.01895	4.21093	4.39797	4.29243	4.07955
SAMD5	2.62734	2.87924	-3.01245	-4.52056	3.09621	3.86883
SAMD8	4.75676	4.9439	4.72112	5.06921	5.45538	5.63811
SAMD9	3.99615	4.17468	5.93541	6.42573	5.68506	6.61862
SAMD9L	2.11792	2.77557	5.99762	6.41796	4.52021	4.94434
SAMHD1	4.39903	4.4973	5.08231	5.41953	5.7117	5.48358
SAMM50	4.50237	4.48893	4.23314	4.59668	3.8225	4.1759
SAP130	4.09371	3.98528	3.81929	3.99708	3.75541	3.49786
SAP18	4.68938	4.76919	4.60256	5.11218	4.98923	5.2605
SAP30	2.928	2.9529	1.83887	2.45242	3.47283	3.61746
SAP30BP	4.46079	4.40443	4.52469	4.79203	5.56368	5.59591
SAP30L	3.38174	3.51444	3.85265	4.1858	3.44035	3.6705
SAPCD2	4.08848	4.09689	2.64403	2.83237	3.25682	3.56201
SAR1A	5.87364	5.88009	6.18222	6.6658	6.6182	6.78614
SAR1B	5.64207	5.93166	6.11161	6.59132	5.90864	6.10461
SARAF	6.06327	6.22409	6.3979	6.82762	6.26541	6.50913
SARM1	2.56493	2.61803	3.63078	3.86576	2.73577	2.72679
SARNP	3.75266	3.74354	3.63214	3.86895	3.73849	4.36777
SARS	5.41787	5.54453	5.7215	6.34828	6.16309	6.15629
SARS2	2.54799	2.1973	1.86801	1.84433	2.37828	2.2729
SART1	4.44367	4.36924	3.96113	4.10791	3.96357	3.42515
SART3	5.74256	5.85851	5.0143	5.24155	5.19728	4.87259
SASH1	4.86223	4.96498	3.56131	4.01141	4.87204	4.72477
SASS6	4.23976	4.55466	3.81224	3.91769	4.1029	3.95684
SAT1	4.4411	3.92516	6.07297	6.31085	5.19493	4.9135
SAT2	3.01173	2.83178	3.60764	3.8219	3.71582	3.73207
SATB1	3.51281	3.25483	1.23438	0.698601	3.11777	2.54596
SATB2	4.10278	3.90161	2.92316	2.67251	2.88018	2.55694

SAV1	3.96597	3.84193	2.33876	2.51809	3.379	3.20842
SAYSD1	2.03904	2.23414	1.52387	2.03075	2.34813	2.48806
SBDS	4.68583	4.67675	4.20299	4.81309	5.23121	5.64336
SBDSP1	2.46979	2.39348	2.29485	2.94032	2.84771	3.25618
SBF1	5.15307	4.90866	5.59142	5.86539	4.61081	4.57983
SBF2	5.15267	5.26681	5.03497	5.02438	5.13672	5.13347
SBF2-AS1	2.16288	2.03866	2.96935	2.58992	1.23087	1.11924
SBNO1	6.68769	6.55321	5.68136	5.86046	6.25117	6.00229
SBNO2	4.03731	3.64306	3.54609	3.86702	4.11117	4.0058
SC22CB-1E7.1	3.90226	3.9259	3.21448	3.78208	4.31747	5.35309
SC5D	4.73867	4.62858	4.28611	4.6864	3.96632	4.28763
SCAF1	2.66725	2.39515	2.7909	2.87132	3.02619	3.26196
SCAF11	6.79276	6.79927	6.38182	6.34214	6.05589	5.99502
SCAF4	3.02203	2.87478	4.37489	4.10549	3.28221	2.86694
SCAF8	5.23287	5.12901	5.23666	5.20391	5.69047	5.48728
SCAI	3.40413	3.35603	3.64378	3.23432	3.52805	2.76193
SCAMP1	4.55177	4.21487	4.9795	4.53863	5.41948	4.59935
SCAMP1-AS1	0.255223	0.013012	0.996447	1.54942	1.06084	1.2122
SCAMP2	3.91839	3.74051	4.04168	4.38301	4.28073	4.20617
SCAMP3	5.21492	5.24279	4.47825	5.00099	5.7079	5.88807
SCAMP4	3.86679	3.43476	3.6743	3.24809	4.05729	3.5725
SCAMP5	3.2981	3.12962	1.25116	1.23107	0.0402612	-0.0341529
SCAND1	2.68367	2.25415	2.09721	2.25295	2.2161	3.16365
SCAND2P	3.79719	3.92629	3.75217	3.91801	4.30241	3.487
SCAP	5.38708	5.21442	5.00195	5.07044	5.11735	4.96038
SCAPER	4.07075	4.01593	3.95347	3.89539	3.06079	2.80504
SCARA3	-1.99808	-1.76193	3.28	3.64773	3.75226	3.28197
SCARB1	7.17285	6.80988	4.62625	4.39202	5.63573	4.91258
SCARB2	7.08377	6.55392	6.89397	7.04324	7.38671	6.97755
SCARF2	1.64525	0.570657	2.41268	2.12001	-1.45047	-1.89883
SCARNA10	7.33192	7.25793	7.34443	7.43431	6.45798	8.14532
SCARNA12	5.88891	5.6814	4.66276	5.05554	4.85318	6.00026
SCARNA13	5.32585	5.43686	4.23334	4.61254	4.39505	5.59348
SCARNA15	2.94229	3.12489	2.16584	2.32093	2.65781	2.6422
SCARNA16	2.07905	2.1973	1.42671	1.66737	2.20133	3.28501
SCARNA2	4.96936	4.86525	4.48537	4.88976	4.00858	5.54829
SCARNA21	0.577131	0.479758	1.27595	1.15801	0.740643	1.11352
SCARNA22	1.44051	1.82258	2.01126	1.95189	2.36761	2.46823
SCARNA5	5.88023	5.95421	5.45987	5.64521	5.04272	6.06435
SCARNA6	4.69323	4.76356	4.29017	4.60541	3.80219	4.54831
SCARNA7	5.64354	5.66997	5.51821	5.9974	5.83803	6.77431
SCARNA9	3.15267	3.00476	3.92761	4.16146	2.8073	2.61523
SCART1	0.399051	-0.0533185	0.102	-0.616766	0.661695	-1.06257
SCCPDH	5.2511	5.33197	3.22799	3.46293	4.05822	4.75271
SCD	8.91784	8.89212	9.22308	9.52718	10.3144	10.6889
SCD5	3.0186	2.85457	2.92239	2.98394	4.68343	4.66832
SCDP1	3.13338	3.21077	3.47759	3.85869	4.6263	4.58024
SCFD1	4.06993	4.32831	3.87852	4.14147	4.4926	5.33767
SCFD2	3.52733	3.57058	3.56969	3.71294	3.96721	4.1419
SCG2	4.30514	4.45601	3.7128	2.67582	4.66218	3.64864
SCHIP1	2.15757	2.16413	2.73245	2.21509	4.39445	4.52922
SCLT1	3.74342	3.57878	3.65863	3.50687	3.68418	3.77015

SCLY	3.96919	3.96383	2.78504	3.02804	3.75396	3.91286
SCMH1	3.99857	4.00314	3.98266	4.15004	3.60688	3.63936
SCML1	3.41076	2.89031	5.25681	5.01138	3.45184	2.56008
SCML2	1.80079	1.75635	-0.229171	-0.249455	1.86805	1.39136
SCN2A	2.02885	2.85378	-2.59248	-2.78325	0.107996	1.40185
SCN3A	-0.739418	0.418114	2.90884	2.52568	-4.07293	-1.28878
SCN4B	?	-6.45019	2.48182	2.35471	-6.64581	?
SCN8A	4.24269	4.24882	3.06203	3.14383	3.82954	3.60617
SCN9A	1.07224	2.45133	0.661713	0.697169	-1.20056	-0.102834
SCNM1	3.63869	3.68352	2.88923	3.35692	3.6706	3.74037
SCNN1A	-4.11163	-5.45651	0.9329	0.825761	1.96909	1.40161
SCO1	4.65791	4.56713	4.87515	5.06108	5.23828	5.33767
SCO2	2.86634	2.62016	2.3204	2.8454	2.28569	2.9985
SCOC	4.89386	4.80862	4.31532	4.6758	5.23301	5.51244
SCP2	4.1406	4.25509	5.23703	5.61405	4.6503	5.46518
SCPEP1	5.16972	5.18146	5.408	5.67621	4.74908	4.92537
SCRG1	3.71982	3.19	-0.375995	-0.55404	2.65711	2.22404
SCRIB	3.31075	3.12586	3.19369	3.18119	4.22156	4.59478
SCRN1	7.28734	7.30914	6.81541	7.41755	7.27191	7.61351
SCRN2	2.5055	2.23761	3.40144	3.57066	2.69064	2.84404
SCRN3	3.25947	3.16104	3.62543	3.77229	3.80729	4.26051
SCUBE1	-6.68418	-4.87366	3.43785	3.58972	?	?
SCUBE3	2.42764	2.39825	2.49948	2.1152	7.76043	7.35295
SCYL1	4.78581	4.44901	4.51004	4.89359	4.33396	4.07676
SCYL2	5.08749	5.19096	4.98939	5.3093	5.4866	5.75641
SCYL3	3.99293	4.02506	3.70593	3.82416	3.46155	3.61564
SDAD1	4.719	4.80723	3.80388	4.38027	5.37025	5.77798
SDAD1P1	0.874561	1.35813	0.650866	0.896373	1.86576	1.88999
SDAD1P2	0.601877	1.1438	0.349433	0.665666	1.42399	1.59309
SDC1	4.7444	4.53222	4.96871	5.13376	7.02447	7.5214
SDC2	3.90084	3.76597	2.70218	2.11519	4.73789	4.36742
SDC3	7.69449	7.72832	5.68335	5.81077	5.41817	4.96128
SDC4	6.84043	6.89112	5.40986	6.12658	5.12063	5.11425
SDCBP	5.04494	5.02287	6.22068	6.75437	5.42747	5.49479
SDCBP2-AS1	2.7804	2.69704	3.66297	3.89947	1.7406	1.53173
SDCBPP3	0.127686	0.297931	1.61917	2.06073	0.618483	0.675039
SDCCAG3	4.19877	3.8404	3.06544	3.23632	4.6591	4.35605
SDCCAG8	3.55917	3.71952	3.17507	3.41418	3.06445	3.56866
SDE2	4.39235	4.39357	3.8277	4.20065	4.5685	4.23286
SDF2	3.95823	4.034	4.22268	4.5305	5.09799	5.23286
SDF2L1	3.61493	3.32994	2.03082	2.30904	1.78266	1.62424
SDF4	5.40353	5.19759	5.38681	5.53682	5.66725	5.67536
SDHA	5.52892	5.57635	5.76468	6.21557	5.4491	5.43167
SDHAF1	1.88591	1.554	1.48185	1.60545	2.63662	3.20018
SDHAF2	3.76738	3.82399	3.49155	3.88944	3.71225	4.06563
SDHAF3	2.36702	2.40135	0.816542	0.935626	-0.23389	-0.183001
SDHAF4	1.40425	1.24456	0.156448	0.698601	1.03324	1.86317
SDHAP1	2.31652	2.19831	3.91404	4.04263	3.64008	3.10764
SDHB	4.51523	4.53855	4.45581	5.09573	5.68955	6.3681
SDHC	5.51982	5.61519	5.05952	5.2434	5.20253	5.57033
SDHD	3.04663	3.20723	4.49209	4.9665	5.22455	6.03581
SDK1	3.41855	3.21839	3.66354	3.52054	3.7816	3.41481

SDR39U1	1.70922	1.76545	1.10723	0.903023	1.97397	2.18698
SEC11A	5.92963	5.71321	6.16185	6.24282	6.38402	6.67807
SEC11B	2.19729	2.07148	2.69931	2.56139	2.66169	2.83631
SEC11C	4.18585	4.15185	2.49245	3.11035	1.69287	2.70269
SEC13	5.88587	5.85088	5.5264	6.22794	6.05005	6.35092
SEC14L1	5.19484	5.11829	5.33042	5.61935	6.86856	6.54716
SEC14L1P1	0.824548	0.877069	1.40981	1.485	1.90264	1.2356
SEC14L2	4.20557	4.13874	3.46106	3.92896	3.59789	3.25306
SEC16A	5.26537	5.10672	5.24061	5.2764	4.84249	4.82759
SEC22A	3.09689	3.17376	3.05249	3.40885	3.24052	3.91167
SEC22B	5.42238	5.46988	5.7776	6.18627	7.43287	7.81219
SEC22C	5.38616	5.39899	4.78378	5.03004	5.56484	5.42056
SEC23A	5.21793	5.8748	4.92624	5.9773	5.03011	6.13957
SEC23B	5.84516	5.90373	4.88659	5.36588	5.65003	5.82611
SEC23IP	5.33328	5.43575	5.04829	5.44815	6.2206	6.25762
SEC24A	5.60946	5.38881	5.44493	5.57142	5.50522	5.25889
SEC24B	4.64687	4.49569	4.70598	4.87202	4.58224	4.46196
SEC24C	5.55369	5.61567	5.33776	5.79243	6.49941	6.39762
SEC24D	4.41922	4.58222	5.12878	5.62035	4.50144	4.87823
SEC31A	5.94245	6.00376	5.95833	6.39952	6.04134	6.12774
SEC31B	1.09167	1.54648	2.0982	2.03746	2.87093	2.14433
SEC61A1	7.39887	7.47609	7.29207	7.68805	7.58356	7.58245
SEC61A2	2.79257	2.89917	2.19522	2.42552	2.13601	1.72827
SEC61B	3.99005	3.92086	3.75754	4.25859	3.72662	4.42383
SEC61G	4.07489	4.09939	4.31032	4.96929	4.63374	5.238
SEC62	5.65157	5.57366	6.36602	6.53527	6.54239	6.34754
SEC63	4.80209	4.70233	5.21427	5.24345	5.51509	5.36235
SEC63P1	2.4834	2.34255	2.86717	2.8692	3.06383	2.95553
SECISBP2	4.45889	4.42818	4.44583	4.46765	4.83703	4.87447
SECISBP2L	5.64521	5.59444	5.27845	5.46812	5.29459	5.34381
SECTM1	3.33297	3.19894	3.4729	3.68883 ?		-4.70296
SEH1L	4.11369	4.18198	3.74353	4.11222	4.95598	5.26194
SEL1L	6.20159	5.95156	5.48358	5.66141	6.0048	5.62027
SEL1L3	3.78573	4.027	4.95986	5.67336	6.17375	6.5813
SELENBP1	-6.68418 ?		2.48892	2.6157	0.652647	-0.0918635
SELK	4.37008	4.54035	2.94941	3.34603	3.80036	4.477
SELL	0.649726	0.920918	0.138526	1.04098	-4.65668	-3.12051
SELM	3.15423	2.87255	1.63029	1.94105	-3.19956	-3.38329
SELO	2.71686	2.18387	2.69188	2.73333	2.89856	3.02063
SELT	4.85865	4.81492	5.00634	5.3764	5.58966	6.0761
SEMA3A	4.93754	4.69071	4.38348	4.59766	7.85626	8.04917
SEMA3B	6.74721	6.69859	1.19157	1.81006	3.08621	1.46325
SEMA3C	6.08166	6.16649	8.55328	8.49194	8.35132	8.51389
SEMA3D	2.46835	2.29539 ?		-6.09892	-3.48878	-5.69922
SEMA3F-AS1	1.33537	1.22891	1.73094	1.59069	1.84222	1.15031
SEMA4B	3.76342	3.37163	4.08443	3.99314	4.89714	4.72894
SEMA4C	4.60126	4.13427	4.31232	3.95861	4.14784	3.6705
SEMA4D	2.04014	2.06074	3.99036	3.79213	1.73389	1.23011
SEMA4F	4.25145	3.77676	3.16521	3.03073	3.04878	2.31567
SEMA4G	1.33002	0.843775	1.28368	0.873404	0.518918	-0.910808
SEMA5A	2.49815	2.43199	5.7804	5.48169	1.02625	1.90802
SEMA6A	3.86501	3.90954	0.574276	0.111499	2.0315	1.61439

SEMA6B		1.19579	-0.58	0.860939	-0.0212303	3.74908	1.5971
SEMA6D		1.03569	1.37009	4.00011	4.11034	-6.64581	-5.69922
SEMA7A		2.13066	1.40138	2.25112	2.72407	5.17891	5.33359
SENP1		4.60983	4.5115	4.28911	4.39094	3.81441	4.00529
SENP2		4.12348	3.74783	4.74721	4.81597	5.50427	5.08036
SENP3		4.96436	4.95855	4.61755	5.00307	5.49162	5.8481
SENP5		5.41793	5.60455	5.71921	6.06827	6.59235	6.68878
SENP6		5.87172	5.85173	5.25058	5.16088	5.89428	5.7781
SENP7		3.46079	3.51586	4.22054	3.98788	3.31267	3.27057
SENP8		0.520176	0.367136	1.13668	1.35398	0.525437	0.978357
	Sep 15	5.97246	5.94894	6.26663	6.65874	6.22406	6.76215
SEPHS1		4.32907	4.33863	4.27413	4.45409	4.52885	4.36855
SEPHS1P4		0.226262	0.351775	0.396423	0.349773	0.442259	0.293029
SEPHS1P6		0.109053	0.0843501	0.065462	0.435607	0.535655	0.319525
SEPHS2		4.36633	4.10606	4.02104	4.23102	3.90574	3.93714
SEPN1		5.11798	4.92841	4.52843	4.19019	3.95575	3.83585
SEPP1		-4.05334	-3.61983	2.50742	2.62678	-5.65305	-4.70296
SEPSECS		2.84782	2.92733	2.32824	2.18582	3.17889	3.44304
SEPSECS-AS1		0.159022	0.223769	0.545464	0.646135	1.00508	0.522196
	Sep 01	0.0884944	-0.0118861	1.03517	1.00424	-0.362544	-1.28716
	Sep 10	4.81964	4.86734	4.97305	5.39946	3.91236	4.31047
SEPT10P1		0.914733	1.11659	1.14037	1.72385	0.210401	0.396094
	Sep 11	6.42179	6.58869	5.69988	6.01399	6.16643	6.02883
	Sep 02	7.39052	7.39847	7.12055	7.47034	7.92097	8.1961
SEPT2P1		1.18971	1.28539	1.06639	1.18756	1.48525	2.01441
	Sep 03	-5.69162	-5.45651	2.68685	3.21084	-3.48878	-2.8983
	Sep 04	-0.547413	-0.707737	2.88148	2.94552	3.1242	3.44358
	Sep 05	3.38702	2.98217	3.64934	3.66917	-1.65987	-2.70579
	Sep 06	1.24348	1.27867	4.03806	4.15796	1.39501	1.17598
	Sep 07	6.93522	7.07632	6.72185	7.13044	6.57392	7.04877
SEPT7P2		3.24909	3.23715	3.01828	3.0799	1.9365	1.70397
SEPT7P6		1.98405	2.42883	2.20748	2.39991	1.90107	2.26162
SEPT7P7		0.165481	0.643367	-0.00981478	0.852782	0.118027	0.849022
SEPT7P8		0.146525	0.68698	-0.00864596	0.266325	0.0717215	0.343908
	Sep 08	5.71888	5.43047	5.66132	5.74451	5.15711	4.78811
	Sep 09	8.11201	7.85365	6.14786	6.24662	7.27296	7.04585
SEPW1		3.65627	3.40752	2.65254	3.02046	3.78574	4.25906
SERAC1		2.6206	2.80629	2.29223	2.41082	4.8037	4.65742
SERBP1		7.77357	7.82739	7.23505	7.61141	8.28362	8.08189
SERBP1P1		4.71849	4.65524	4.02011	4.35593	5.0943	5.14827
SERBP1P5		5.69009	5.70808	4.95616	5.38069	6.15632	6.00305
SERBP1P6		3.86888	3.97347	3.28401	3.61454	4.34603	4.27902
SERF1A		1.76357	2.04011	2.45472	2.7166	1.70345	1.68974
SERF1B		2.6167	2.72259	3.2355	3.56775	2.87616	3.42652
SERF2		6.60408	6.50119	5.93578	6.41227	7.05763	7.47942
SERGEF		2.64523	2.55397	1.7294	2.11143	2.25259	2.43541
SERINC1		5.46519	5.77674	5.95889	6.56469	7.07735	7.59905
SERINC2		0.266817	-0.292758	2.60438	2.60199	-3.33694	-2.24661
SERINC3		6.92996	7.02032	7.01808	7.5046	7.00635	7.11142
SERINC5		2.81211	3.70604	4.94611	5.75503	7.67779	8.30216
SERP1		5.32502	5.35141	5.54147	6.00139	6.27861	6.61805
SERPINA1		-1.27248	-0.93902	1.37876	2.08102 ?	?	

SERPINA3	10.5928	10.5978	-0.375995	-0.55404	3.63661	1.90064
SERPINA5	5.96646	6.01035	-1.229	-1.20162	2.10458	1.23585
SERPINB1	1.52013	1.87703	-2.72315	-3.52222	-3.85089	-4.70296
SERPINB2	0.520176	1.73686	-1.79153	-0.616766	?	?
SERPINB5	5.06038	5.37516	-4.18092	-5.10387	-4.65668	-4.11926
SERPINB6	4.1147	4.374	5.08561	5.71415	4.88658	5.35465
SERPINB8	4.87847	5.00982	1.05494	2.43034	1.15992	1.95152
SERPINE1	3.72509	4.29037	2.30031	3.5864	8.71597	9.47276
SERPINE2	10.3605	9.40012	5.44719	6.09845	7.46518	6.96901
SERPINH1	7.11689	6.84738	6.7158	6.79849	4.37997	4.27486
SERTAD1	1.19579	1.60328	1.22592	1.83372	1.74486	2.80899
SERTAD2	4.14797	4.13334	3.28205	3.5022	3.76909	3.22698
SERTAD3	2.48543	2.42048	2.7721	3.1492	3.01941	3.24259
SERTAD4	6.17452	6.16925	2.37491	2.62928	1.97271	2.34315
SERTAD4-AS1	1.59558	1.74664	-0.861345	-1.20162	-1.65987	-0.848487
SESN1	1.87832	2.07035	1.50756	1.33124	3.11665	2.95779
SESN2	2.26094	1.98426	1.8609	2.62928	2.56611	2.15749
SESN3	2.6911	2.50871	1.43123	1.14862	5.89523	5.6152
SESTD1	4.03139	4.25825	5.48358	5.41228	5.19921	5.49724
SET	7.30465	7.67057	6.61773	7.21906	7.40899	7.81934
SETBP1	3.97951	3.7969	2.57804	2.14386	3.52951	3.23873
SETD1A	2.47012	2.09529	2.75558	2.74232	2.52458	1.73475
SETD1B	2.85552	2.61398	2.84163	2.78296	2.56128	2.63759
SETD2	6.48994	6.37872	6.47353	6.37625	6.5484	6.32152
SETD3	4.91378	4.9187	4.20449	4.63431	4.46832	4.6152
SETD4	2.60927	2.72204	3.45491	3.4928	3.03318	2.78915
SETD5	6.35245	6.22998	6.26768	6.26104	6.38356	5.82856
SETD6	3.79459	3.97677	3.28727	3.37432	4.6141	4.36063
SETD7	5.81808	5.89634	5.88189	6.41448	5.56338	5.82953
SETD8	4.55921	4.37556	4.0994	4.18634	4.52668	3.73449
SETD9	-0.517761	-0.258259	0.105584	0.68322	1.19526	2.17921
SETDB1	4.73006	4.69374	4.35262	4.38099	4.71449	4.51928
SETDB2	2.46081	2.58429	3.61737	3.75999	2.41117	2.84404
SETMAR	3.15579	2.87255	2.34006	2.2878	2.53692	2.65962
SETP14	5.06092	5.49379	4.44442	5.01115	5.09186	5.62404
SETX	7.04397	7.04779	6.71447	6.77746	6.70786	6.75475
SEZ6L2	3.43149	3.12024	3.35369	2.85408	-1.70566	-2.24661
SF1	5.49289	5.29287	5.15465	5.18118	5.00635	4.85553
SF3A1	5.63684	5.68275	4.86901	5.03132	4.92178	4.98146
SF3A2	2.41697	2.19181	1.95405	2.0561	2.48868	2.52212
SF3A3	6.25108	6.41221	5.21192	5.67565	6.10025	6.20271
SF3A3P1	2.33035	2.63992	1.69739	1.86251	1.95021	2.09118
SF3A3P2	2.59239	2.98988	1.51304	2.03284	2.47901	2.26316
SF3B1	7.78376	7.78617	7.30054	7.56039	8.0994	8.16478
SF3B2	7.26964	7.29758	6.33922	6.66993	6.41411	6.31286
SF3B3	7.12338	7.11034	7.23751	7.55785	7.79257	7.56737
SF3B4	4.57411	4.55882	3.69911	4.12111	4.05395	4.26483
SF3B5	3.97688	3.83406	3.64936	3.85262	5.18869	5.81782
SF3B6	4.7547	5.11735	3.6729	4.2553	5.672	6.94052
SFI1	3.25221	3.28367	3.41453	3.49298	2.63432	2.21812
SFMBT1	3.70293	3.95447	3.48507	3.77839	3.84903	4.06642
SFMBT2	-3.8896	-6.45019	0.711372	0.520618	2.9979	2.49787

SFPQ	7.74966	7.79924	7.38189	7.44223	7.54066	7.37759
SFR1	0.994464	1.42593	1.30837	1.64609	1.40043	1.97981
SFRP1	4.74798	4.87196	2.85814	3.97863	4.79346	5.33562
SFSWAP	5.17473	5.19715	4.50384	4.55472	4.5882	4.22992
SFT2D1	3.2782	3.33158	2.91451	3.15796	4.0676	4.31704
SFT2D2	7.44936	7.56842	6.3947	6.74697	7.20283	7.22432
SFXN1	5.72271	5.73282	6.14935	6.4448	6.70475	6.59534
SFXN2	2.98392	2.78271	2.53759	2.58815	3.02094	2.72792
SFXN3	4.70018	4.67286	3.84023	4.393	4.69834	4.76089
SFXN4	2.58864	2.48845	1.81651	2.19497	3.36206	3.7383
SFXN5	3.95197	4.12118	3.88256	4.15444	4.98175	4.84884
SGCB	4.62902	4.80686	4.21949	4.75148	4.73629	5.24965
SGCE	4.99091	4.74965	4.83189	5.241	5.65058	6.09982
SGK1	4.09939	3.6703	3.57304	3.82338	5.00414	5.09983
SGK223	-0.289526	-0.333394	3.44946	2.69209	1.51465	1.06153
SGK3	2.51417	2.52539	0.327503	0.806901	1.83787	1.84602
SGK494	1.74722	1.75719	2.25364	2.12524	1.65434	0.622499
SGMS1	3.93573	3.95184	3.19802	3.59853	3.22233	3.22993
SGMS1-AS1	0.833821	0.720427	1.24194	1.23577	-0.538103	-0.311564
SGMS2	2.0668	3.23701	2.86323	3.74786	3.00334	3.63136
SGOL1	3.75558	3.81123	3.33498	3.47532	3.47852	3.53608
SGOL2	5.12109	5.35772	4.55033	4.8219	5.23411	5.81929
SGPL1	4.59553	5.14955	4.08326	4.62249	4.76647	5.21065
SGPP1	3.51888	3.36529	3.19802	3.38102	3.48817	3.01439
SGPP2	-0.470075	-0.312932	0.225978	0.605504	0.688494	0.100762
SGSH	4.19498	3.83865	4.7772	4.87558	3.99253	4.00753
SGSM2	3.90006	3.82022	4.57251	4.55182	5.08631	4.64919
SGSM3	4.10422	3.67932	4.00509	4.07605	3.51667	3.15438
SGTA	4.46269	4.10034	3.90792	4.12238	5.1702	5.16211
SGTB	3.55402	3.64631	3.88759	4.32635	3.73962	3.68975
SH2B1	3.73123	3.53716	4.04409	4.14973	4.0777	3.68741
SH2B3	6.85816	7.48541	4.77211	5.25803	4.66667	4.768
SH2D4A	-1.20691	-1.41808	3.7398	3.93147	2.84749	2.85935
SH2D5	0.631914	0.107125	0.636738	1.25754	0.448309	0.126299
SH3BGRL	3.86029	4.08598	4.68606	5.11457	4.58997	4.96658
SH3BGRL2	2.91564	3.09558	-1.05395	-0.860651	3.29969	3.42514
SH3BGRL3	4.74776	4.62426	4.16212	4.67887	5.05825	5.32943
SH3BP1	2.7916	2.35754	3.13665	3.35041	1.78045	1.85636
SH3BP2	2.90084	2.68691	5.80905	5.6864	6.30801	5.12221
SH3BP4	6.04533	5.90511	5.40286	5.65645	6.63775	6.46509
SH3BP5	2.67552	3.11093	2.58377	2.83847	4.68737	4.37152
SH3BP5-AS1	3.442	3.43339	3.1002	2.9393	3.50697	2.73596
SH3BP5L	4.2123	4.11268	3.73086	4.10183	4.59826	4.68988
SH3D19	5.25881	5.1477	4.00063	4.184	4.82327	5.31587
SH3D21	1.01831	0.391262	1.26424	1.36745	3.13648	1.49167
SH3GL1	4.59972	3.98687	3.99334	3.94201	5.0141	4.49165
SH3GLB1	5.43694	5.48478	5.65052	5.87522	5.86544	6.13169
SH3GLB2	3.65957	3.6299	3.7113	3.79052	4.49514	4.45944
SH3KBP1	5.62594	5.77554	4.64698	5.36942	4.14865	4.29046
SH3PXD2A	6.65118	6.67111	4.20664	4.37398	3.89331	4.08281
SH3PXD2B	4.5892	4.27862	4.93217	4.89609	6.33985	5.81537
SH3RF1	3.85839	3.86918	2.87447	3.0586	3.69834	3.45315

SH3RF2	1.69327	1.93378	3.75164	4.11276	1.69727	1.75275
SH3RF3	-5.69162 ?		-0.825729	0.120113	2.13087	1.82469
SH3TC1	0.878411	0.736899	2.15634	2.02562	-4.07293	-3.12051
SH3TC2	5.38172	5.94996	3.35369	4.16729	1.55404	2.32121
SH3YL1	2.60243	2.63256	2.77938	2.43808	3.42317	2.6899
SHARPIN	2.21083	2.1146	3.0501	3.36691	2.81137	3.01439
SHB	2.63826	2.78271	2.53073	3.2131	4.97344	4.63092
SHC1	6.69763	6.53821	6.86027	6.99704	7.22812	7.38014
SHC1P2	0.0155547	0.109227	0.0655447	0.725427	0.956138	0.126973
SHC3	0.752201	0.775632	0.793829	0.749225	1.05399	0.650921
SHC4	3.06747	3.086	1.5647	1.81602	1.37866	1.34863
SHCBP1	4.86173	5.0768	4.85539	5.38002	5.96723	5.98284
SHFM1	5.61327	5.55055	5.63301	5.86379	5.35752	5.75423
SHFM1P1	0.0667987	0.36727	0.504386	0.28359	0.0946955	-0.00614162
SHISA2	-6.68418	-4.87366	4.47825	4.81375	1.17894	0.922708
SHISA4	2.72719	2.80862	1.79379	2.53143	3.08116	3.37011
SHISA5	5.63909	5.57469	5.20394	5.32684	6.39853	6.20468
SHKBP1	3.00828	2.86584	2.78227	3.20179	3.21314	3.56007
SHMT1	4.34985	4.31949	3.82543	4.08941	4.71033	4.85246
SHMT2	5.2264	5.23992	5.69618	6.32401	5.97602	5.98455
SHOC2	4.74024	4.73804	4.55541	4.83734	5.068	5.30453
SHOX2	3.02544	2.7742	3.70598	3.50238	3.99391	3.61746
SHPK	3.58424	3.43209	3.99652	4.221	3.93744	3.88541
SHPRH	5.01663	5.13702	5.34859	5.30327	5.56227	5.20994
SHQ1	4.1409	4.00067	3.68838	3.8582	4.09457	3.85743
SHROOM1	2.55596	2.31853	3.25111	3.5689	1.51465	1.29333
SHROOM2	4.26959	4.19625	-0.206091	0.120113	-5.65305	-3.70484
SHROOM3	3.87741	3.97594	2.35177	2.38304	4.81946	4.69629
SHROOM4	5.99227	6.10604	1.0162	1.03078	2.73202	2.677
SHTN1	4.90408	5.15231	4.38633	4.39399	5.6184	5.43175
SIAE	2.2245	2.25238	3.66194	3.75278	1.7024	1.50513
SIAH1	2.98845	3.08138	3.71915	3.64811	4.11652	3.63635
SIAH2	3.03537	2.83866	3.0477	3.12461	4.19748	3.96394
SIDT2	2.88777	2.82256	4.04409	3.90099	3.00147	2.77711
SIGIRR	2.24052	1.8681	-2.27595	-2.52305	-5.65305 ?	
SIGMAR1	5.17588	5.13149	4.65566	4.96127	5.09204	5.04648
SIK2	3.45406	3.38842	4.89988	5.05853	5.0142	4.83234
SIK3	3.52733	3.24452	3.74426	3.78447	4.38271	3.85743
SIKE1	4.87927	5.28239	4.62142	5.22711	6.0801	6.74988
SIL1	3.87444	3.59912	4.28386	4.51854	3.47905	3.89516
SIM2	-1.45077	-1.17713	2.56468	2.59854	-2.01588	-2.38405
SIMC1	1.27867	1.25908	3.56627	3.58799	-1.45047	-2.8983
SIN3A	5.38253	5.43474	5.56042	5.61499	5.06432	4.8603
SIN3B	3.89617	3.56506	3.85127	3.83075	4.6012	4.07136
SIPA1	3.75829	3.513	2.26358	2.60886	1.6435	1.65964
SIPA1L1	4.28248	4.11929	4.42933	4.68558	5.47411	5.35734
SIPA1L2	5.46108	5.3453	6.94186	6.88677	5.60619	5.47629
SIPA1L3	2.68247	2.51158	3.08794	2.86559	3.8235	3.24166
SIRPA	3.66271	3.33558	3.40652	3.4992	1.41349	1.27061
SIRT1	3.52612	3.45154	2.92761	3.1881	3.65823	3.88199
SIRT2	3.13064	3.025	3.39957	3.71295	4.42913	4.58098
SIRT3	2.43978	2.47169	3.05998	3.21278	2.20699	1.77713

SIRT5	1.18871	1.5593	1.67449	2.06112	2.01486	2.43796
SIRT6	1.43024	1.07637	0.882635	1.17662	1.65713	2.27058
SIRT7	3.88305	3.5938	3.80516	4.1067	4.83002	4.96747
SIVA1	4.15423	3.75268	3.0501	3.11034	2.88273	3.9135
SIX1	3.31495	3.51444	3.49771	3.66596	1.90957	2.14505
SIX4	4.07652	3.96446	3.92107	3.84973	3.67731	3.15748
SIX5	1.02487	0.6168	0.817713	1.0163	0.848782	1.19618
SKA1	4.03562	4.21575	2.75018	3.42677	2.59944	2.70269
SKA2	5.51043	5.3918	4.68424	5.08706	6.25036	6.27073
SKA2P1	1.31659	1.33506	0.775017	0.763454	2.43479	2.9462
SKA3	3.98039	4.1296	3.16077	3.54042	3.37723	3.57405
SKAP2	3.52973	3.74294	2.5273	2.96532	3.78986	4.18961
SKI	3.63631	3.57607	3.36915	3.27919	3.7169	3.69629
SKIDA1	0.987481	1.2583	-0.276471	-1.06415	3.34812	3.75066
SKIL	5.35892	5.59784	5.33856	5.26944	5.95506	5.70212
SKIV2L	4.43084	4.36608	4.43026	4.86702	5.34635	5.34721
SKIV2L2	5.54037	5.67504	5.77366	6.01407	5.95607	6.37741
SKP1	6.49121	6.5363	6.86645	7.23208	6.76748	7.26592
SKP1P1	2.9303	3.13957	3.35397	3.7578	3.29415	3.83433
SKP2	4.76749	5.00169	5.23275	5.52644	5.02312	5.33288
SLAIN1	4.40486	4.2845	-5.1783	-5.10387	4.11694	4.61633
SLAIN2	4.73656	4.87978	4.33513	4.68719	5.93817	5.85026
SLBP	4.72562	4.78626	4.07386	4.44865	5.17177	5.25617
SLC10A3	3.09046	2.92948	2.46395	2.76462	4.18909	4.60729
SLC10A7	3.13855	3.04495	2.97635	2.98657	2.33123	1.97981
SLC11A2	6.08595	6.024	5.2359	5.70895	5.56067	5.59192
SLC12A2	6.09431	6.55031	5.4298	5.7696	6.22978	6.39558
SLC12A4	4.4326	4.17951	4.73147	5.02302	4.94595	4.28563
SLC12A6	3.94932	3.862	1.87454	1.78258	2.83519	2.48607
SLC12A7	2.55596	2.88368	4.18933	4.35674	-3.85089	?
SLC12A8	-1.1752	-0.93902	4.98702	5.72009	2.81341	3.11984
SLC12A9	3.55595	3.38733	3.75899	3.92871	3.80423	3.95684
SLC13A4	0.784805	0.785149	0.996447	0.879925	0.909612	-0.199111
SLC14A1	5.24232	5.95789	2.24695	3.1391	-3.85089	-2.70579
SLC15A3	-2.61075	-2.87684	2.65653	2.74293	-0.510371	-2.24661
SLC15A4	3.72088	3.83062	3.84161	4.16962	3.94701	3.88574
SLC16A1	7.07873	7.15721	6.60496	6.91281	7.96221	7.71661
SLC16A1-AS1	3.3844	3.54839	3.3653	3.43614	3.34812	2.55539
SLC16A13	0.243547	0.581631	1.34008	0.902467	-0.490197	-0.799586
SLC16A2	1.25516	0.90354	4.41079	4.82115	5.50301	5.8085
SLC16A3	3.70388	3.20391	6.359	6.51207	7.50824	6.91446
SLC16A4	3.11048	3.16528	4.9361	5.44137	3.74272	4.0316
SLC16A6	0.86656	1.50328	0.657521	1.74785	-3.12253	-4.01143
SLC16A7	4.83765	5.01844	5.15409	5.55694	3.74163	3.84595
SLC16A9	-6.68418	-6.45019	0.662041	0.120113	3.74907	4.69949
SLC17A5	3.44175	3.32994	2.08328	2.74293	3.45083	3.32671
SLC18B1	3.59439	3.12774	2.57138	2.75537	3.86912	3.89876
SLC19A1	3.75742	3.66843	4.50113	4.6623	4.41035	4.08102
SLC19A2	4.1904	4.15462	2.92239	3.1603	3.22082	3.01439
SLC19A3	2.2493	2.14451	-4.18092	-4.52056	1.07446	0.951558
SLC1A1	2.31774	2.86134	2.34397	3.03329	3.05908	3.36206
SLC1A3	1.32333	2.02903	3.71432	4.19609	5.94819	6.05733

SLC1A4	7.18815	7.32832	2.72036	3.65106	2.88079	2.8932
SLC1A5	6.32268	6.21142	6.54271	6.81058	6.72286	6.50385
SLC20A1	7.64553	8.08634	6.48163	7.22814	6.83455	7.07273
SLC20A2	4.42181	4.574	3.53244	3.73509	4.38749	4.41869
SLC22A15	2.0986	2.21665	1.50302	2.31325	0.0540566	0.0747657
SLC22A17	-5.69162 ?		1.30837	1.31748	-3.19956	-3.38329
SLC22A18	0.334442	-0.0703893	2.05012	2.32582	0.679613	0.951558
SLC22A23	3.1696	3.24778	3.40155	3.5401	5.59883	5.02745
SLC22A4	1.55138	2.16426	2.39446	3.19406	0.0389839	0.615294
SLC22A5	3.65737	3.49135	3.529	3.58987	2.08454	1.493
SLC23A2	5.62901	5.32706	6.03624	5.91629	5.0552	4.16826
SLC24A1	3.10828	2.94443	2.88257	2.82487	2.06728	1.72794
SLC24A2	2.49075	2.27526	-3.18222	-4.52056	-5.07051	-5.69922
SLC25A1	4.31565	4.0149	3.91319	4.2232	4.46315	4.40181
SLC25A10	3.81853	3.49011	2.521	2.52873	3.77259	4.08979
SLC25A11	4.02776	3.96602	4.06411	4.35873	4.69791	5.17483
SLC25A12	3.76035	3.84093	3.38253	3.95591	4.13736	4.79611
SLC25A13	5.39829	5.39276	4.87377	5.14442	5.37821	5.48924
SLC25A14	1.2426	0.894767	2.07859	2.30482	3.00503	3.16057
SLC25A15	2.84783	2.80161	1.32432	1.56364	3.03319	3.32395
SLC25A16	3.02202	2.92733	3.23011	3.4108	3.71906	3.28764
SLC25A17	4.22574	4.22889	3.88793	4.11397	4.2682	4.70796
SLC25A19	2.17512	2.10664	1.05306	1.6065	3.36065	3.61141
SLC25A20	2.30936	2.47381	2.42382	2.89118	2.98535	3.51004
SLC25A22	3.06748	2.82718	2.95859	3.14856	3.20852	3.24789
SLC25A23	2.2696	2.07245	2.52039	2.67908	3.34531	3.42257
SLC25A24	4.70142	4.93085	5.13881	5.59378	4.32951	4.84743
SLC25A25	2.74834	2.64369	2.91482	3.10469	3.59829	3.60187
SLC25A25-AS1	1.39288	1.69588	0.590559	1.06042	-0.318274	-0.54425
SLC25A26	2.99305	2.97868	1.80624	2.00441	2.65771	2.93835
SLC25A27	1.56774	1.71211	3.16521	3.26838	-6.64581 ?	
SLC25A28	2.35621	2.29539	2.90129	3.24432	2.72987	2.43796
SLC25A29	4.04968	3.67705	3.48809	3.45235	3.23402	2.86223
SLC25A3	7.62364	7.57503	6.69336	7.10167	7.26414	7.60814
SLC25A30	2.89712	3.05483	3.40332	3.8273	3.10427	3.50274
SLC25A32	4.33705	4.49902	4.0861	4.56468	5.40675	5.57319
SLC25A33	1.66001	1.82241	1.53913	1.76315	2.90875	3.23989
SLC25A35	0.40609	0.381062	1.25045	1.28929	0.370901	0.423331
SLC25A36	5.33743	5.36131	6.66353	6.74817	5.35125	4.80206
SLC25A37	6.70657	6.66595	6.26329	6.25584	5.69614	4.99285
SLC25A38	3.71511	3.51	3.13754	3.28534	4.27641	4.31692
SLC25A39	4.32675	4.16103	4.41638	4.78219	5.08913	5.159
SLC25A3P2	1.44051	1.70209	0.53081	1.30061	1.56854	1.44307
SLC25A4	3.70925	3.68435	1.59787	1.90801	3.91428	3.77307
SLC25A40	3.74936	3.97414	4.26061	4.50349	3.4069	3.58606
SLC25A42	0.301026	-0.292756	1.65571	1.83372	1.99792	1.67702
SLC25A43	2.25513	2.24453	3.08093	3.64104	3.72769	3.89133
SLC25A44	5.37138	5.36168	4.30933	4.59679	5.42711	5.24383
SLC25A45	1.60474	1.7708	0.0645242	0.0612232	-0.852709	-1.61882
SLC25A46	5.02287	5.0734	5.49199	5.8355	5.69614	5.99543
SLC25A5	5.80249	5.79531	6.40021	6.86232	7.83955	8.31575
SLC25A5-AS1	-0.174498	-0.277805	1.00153	1.09644	1.28506	2.1241

SLC25A51	3.31739	3.40164	2.8494	2.96382	4.7379	4.61164
SLC25A53	1.43819	1.39497	0.703544	0.700404	1.83837	1.91296
SLC25A5P2	-1.10227	-0.732052	-0.167726	0.289032	1.07312	1.24581
SLC25A6	5.05706	4.76115	5.42609	5.70213	5.2715	5.429
SLC26A11	2.64966	2.50584	2.88257	2.98393	2.3397	2.27058
SLC26A2	9.42842	9.6708	5.71658	6.00525	4.91143	5.3486
SLC26A6	5.55209	5.19759	4.36337	4.59507	5.67396	5.06146
SLC27A1	2.69071	2.32507	2.95335	3.12927	3.45587	2.75273
SLC27A3	0.493668	0.101058	0.316204	0.321974	1.32582	0.982641
SLC27A4	4.18355	4.10699	2.68993	2.79506	3.76596	3.64864
SLC27A5	1.81322	1.4838	0.353583	0.4804	0.224645	0.737194
SLC29A1	4.53392	4.4129	3.45853	3.70334	4.0744	3.76699
SLC29A2	2.37388	2.32284	0.0510658	-0.158238	-1.11575	-1.53638
SLC29A4	-1.08408	-1.60444	2.4422	1.67257	0.0677351	-1.79933
SLC2A1	5.58023	5.86474	6.68121	7.24431	7.85117	7.3843
SLC2A1-AS1	-0.889271	-1.33321	1.47473	1.67911	-0.551587	-1.24695
SLC2A10	1.67164	1.31856	5.2443	5.19094	0.270529	0.33778
SLC2A11	2.11394	1.97952	3.23505	3.39924	2.04464	1.25862
SLC2A12	-1.16051	-1.09328	2.60646	2.25135	5.75726	6.25983
SLC2A13	3.37372	3.88145	0.986462	0.924662	0.799187	0.615294
SLC2A3	3.24928	2.66902	5.17219	5.27695	4.54008	3.54567
SLC2A3P1	0.249518	-1.43496	0.727668	0.513743	-0.386035	2.94751
SLC2A3P4	0.153053	0.266337	0.70794	0.712367	0.278413	-1.14402
SLC2A4RG	2.96981	2.9697	3.2045	3.5968	2.91523	3.54122
SLC2A6	1.85554	1.51448	2.84162	3.31534	1.19772	1.25912
SLC2A8	1.95558	1.85459	1.19157	1.39901	2.01214	2.25909
SLC30A1	4.68678	5.16057	3.13163	3.85551	5.19026	6.24347
SLC30A4	2.78163	2.89359	2.98031	3.48864	2.31933	2.64202
SLC30A5	4.52696	5.10492	4.95661	5.74952	5.01188	5.87317
SLC30A6	4.87692	4.8925	4.47379	4.66348	5.59883	5.54711
SLC30A7	5.71876	6.13263	5.79374	6.36437	6.30292	6.39591
SLC30A9	5.37638	5.45229	4.997	5.17599	6.2546	6.58848
SLC31A1	5.09614	5.03295	5.72035	6.14796	5.18282	4.78813
SLC31A2	1.8509	1.60319	1.1267	1.678	1.56335	1.71968
SLC33A1	4.56413	4.60989	4.75458	5.00751	5.10492	4.91256
SLC35A1	4.43401	4.44095	3.02682	3.2391	3.25361	3.99246
SLC35A2	4.10585	4.20514	3.27591	4.01787	3.3909	3.58561
SLC35A3	4.14953	4.40982	3.80232	3.83516	4.07523	4.0246
SLC35A4	4.65757	4.4964	4.49978	4.50608	5.09291	4.94463
SLC35A5	3.91194	3.9832	4.12254	4.51779	3.57451	4.15127
SLC35B1	5.16896	5.11929	4.6596	5.11517	4.75013	5.05649
SLC35B2	5.59955	5.45039	4.76339	5.12	6.1924	6.56445
SLC35B3	2.18052	2.22717	2.5273	2.6856	2.91901	3.28481
SLC35B4	5.59668	5.71426	5.18607	5.36537	5.35299	5.44493
SLC35C1	2.63184	2.47086	3.62865	4.13791	2.76176	3.12301
SLC35C2	5.31178	5.1715	4.72637	4.81078	5.01212	5.20317
SLC35D1	4.66833	4.96236	4.97508	5.48828	4.44559	4.94433
SLC35D2	2.9101	2.74418	2.2002	2.37903	4.3418	4.28197
SLC35E1	5.00568	5.21265	4.63185	5.13376	6.12596	5.96967
SLC35E2	1.25818	1.05613	1.98013	1.95124	2.02217	1.76166
SLC35E2B	5.37192	5.17992	5.45372	5.45149	5.98523	5.39274
SLC35E3	5.01559	4.77077	3.29424	3.35266	3.74057	3.4582

SLC35E4	2.15298	2.09952	1.65628	1.87455	1.06804	1.61526
SLC35F1	4.39634	4.07049	-3.37468	-2.93781	-2.48959	-2.53597
SLC35F2	3.41335	3.50725	4.08677	4.59076	5.71034	5.47131
SLC35F5	5.54379	5.73529	5.26097	5.5658	5.51648	5.78912
SLC35F6	5.07248	5.15261	5.55639	5.92149	5.37272	5.21772
SLC35G1	4.09451	4.09365	2.50649	2.64273	3.98354	3.97803
SLC35G2	2.83728	2.86101	3.4872	3.66615	2.8157	3.18136
SLC36A1	2.82645	2.59516	3.21308	2.95457	2.55645	1.97981
SLC36A4	3.52371	3.56091	5.1552	5.37296	3.85834	3.86693
SLC37A2	3.44685	3.39358	2.73064	3.53323	0.159978	-0.497071
SLC37A3	5.15655	5.2514	4.32129	4.73509	5.17258	5.45818
SLC37A4	4.21208	4.23175	3.87316	3.95002	3.88485	3.88386
SLC38A1	6.26216	6.54906	7.22718	7.97188	7.20869	7.26697
SLC38A10	5.367	5.14401	4.99016	5.13434	4.53382	4.80008
SLC38A2	8.13063	8.09549	9.52337	9.75713	8.53651	8.66211
SLC38A3	-2.79121	-6.45019	-1.48269	-2.30078	3.95348	1.8479
SLC38A6	3.42925	3.46927	3.58717	3.54683	3.03753	3.33637
SLC38A7	3.41986	3.10701	3.606	3.46293	4.32196	3.91716
SLC38A9	3.38838	3.49715	3.69758	3.99445	4.38135	4.84978
SLC39A1	6.45777	6.12709	5.97989	6.04343	6.41107	6.23394
SLC39A10	5.61691	5.70083	4.59044	4.80555	5.0933	5.81878
SLC39A11	1.00834	1.13712	2.91188	3.12	3.46381	3.99719
SLC39A13	3.14641	3.08598	3.72636	4.20632	3.88657	4.13091
SLC39A14	7.97279	8.13317	6.25081	7.07008	6.66906	6.92713
SLC39A3	3.18966	3.10701	2.2841	2.43422	3.22234	3.28481
SLC39A6	6.56816	6.71901	5.36914	5.81896	5.67562	5.85933
SLC39A7	6.28884	6.31085	6.32327	6.77567	6.84065	6.67192
SLC39A8	4.47272	4.65219	3.21094	4.40415	4.19457	5.03559
SLC39A9	5.98791	6.18348	5.33317	5.75923	6.49797	6.7991
SLC3A2	6.94115	6.83891	6.00599	6.60003	6.56683	6.17821
SLC40A1	?	?	-5.1783	-5.10387	2.62741	3.2358
SLC41A1	4.40091	4.33644	4.42101	4.67336	4.39767	4.09419
SLC41A2	2.87078	3.46493	1.82773	2.67254	3.15347	3.62644
SLC41A3	3.53813	3.46789	3.1828	3.13673	3.62162	3.41998
SLC43A1	1.35625	0.877069	1.8772	1.65939	-4.07293	-5.69922
SLC43A2	1.95916	1.14454	2.14741	2.22044	0.305574	-0.421131
SLC43A3	5.38925	5.48698	4.9779	5.72733	1.74539	1.99375
SLC44A1	6.14223	5.38381	5.02774	4.47048	5.50391	4.76496
SLC44A2	3.8107	3.09558	4.31533	4.05356	2.81543	2.07467
SLC44A3	-0.450968	-0.508457	0.191626	0.685662	0.384182	-0.497069
SLC44A5	4.03732	4.24279	-2.48229	-4.10635	3.13897	3.68989
SLC45A3	2.9698	2.81794	2.16964	2.0561	1.66615	1.28202
SLC45A4	0.62293	0.503023	2.38255	2.17196	3.27043	3.05151
SLC46A1	3.29832	3.41747	4.02537	4.2959	3.3314	3.76301
SLC46A3	1.12752	0.736899	3.19369	3.45341	2.42451	2.55069
SLC48A1	2.21383	2.37006	2.64304	2.89961	3.26599	3.5003
SLC4A1AP	4.57893	4.61142	3.425	3.73026	4.63347	4.79921
SLC4A1APP1	1.6226	1.58578	0.292911	0.638547	1.72853	1.93191
SLC4A2	6.10506	6.05142	5.93381	6.3049	6.70154	6.85854
SLC4A3	2.38308	2.05287	2.06916	2.15798	1.77435	1.54125
SLC4A7	7.68147	7.6604	7.59033	7.63113	8.09064	8.02374
SLC4A8	2.06581	3.11464	0.925065	2.32998	4.23223	5.38602

SLC50A1	4.11149	4.25826	3.35564	3.96932	4.441	5.08362
SLC51A	-0.315039	0.0304491	1.22897	1.2993	1.76848	1.32838
SLC52A2	4.12419	3.9393	3.71046	4.10152	4.81617	5.03274
SLC5A12	1.60929	1.53719	-4.18092	-5.10387	-6.64581	-4.70296
SLC5A3	9.9022	10.0237	10.2488	10.3596	8.61739	8.28265
SLC5A6	6.48581	6.52347	3.69556	3.81736	5.70161	5.54948
SLC6A6	6.38045	6.41667	6.97253	7.45819	8.39566	7.65041
SLC6A8	4.85403	4.2059	1.81086	1.56364	5.91976	4.55769
SLC6A9	2.7289	2.70627	2.53174	3.22151	2.32264	2.2002
SLC7A1	6.50241	6.67077	5.262	5.69489	5.77795	5.80453
SLC7A11	6.49981	6.88149	8.68278	9.08005	7.84481	7.67087
SLC7A11-AS1	1.34027	1.86201	4.40622	4.51379	2.33714	2.1399
SLC7A2	?	-5.45651	3.63186	3.60198	?	-5.69922
SLC7A5	7.77525	7.59059	5.3096	5.91792	7.16182	5.85336
SLC7A6	5.15063	5.56742	4.79026	5.2858	5.97701	6.119
SLC7A6OS	3.28212	3.27047	3.66982	3.74742	4.27646	4.33712
SLC8A1	0.989873	1.27651	1.41265	1.82421	2.53968	3.1968
SLC8B1	2.07573	2.04497	2.95091	3.29844	1.83956	1.67703
SLC9A1	3.01173	2.87032	3.23644	3.24873	2.80118	2.46823
SLC9A3R1	2.35622	2.31853	2.61739	2.86207	3.91138	3.79485
SLC9A3R2	1.61837	1.33161	2.32429	2.73353	4.47986	4.90523
SLC9A5	1.84067	1.74027	2.11073	2.01172	0.528621	0.653684
SLC9A6	3.08883	3.63386	3.92106	4.77991	4.35439	5.01777
SLC9A7	4.92615	5.12443	4.92105	5.42984	5.02092	5.02373
SLC9A8	3.80275	3.67797	4.33118	4.48081	3.03143	2.69844
SLC9A9	-1.37679	-1.60444	4.31632	4.68882	-6.64581	?
SLC9B2	2.32607	2.57334	0.925065	1.39106	1.62049	1.91536
SLCO1B3	-6.68418	-6.45019	?	-6.09892	1.27368	1.82067
SLCO2B1	-5.69162	-6.45019	4.43026	4.96062	?	?
SLCO3A1	3.96091	4.16832	4.22799	4.56977	3.51211	3.41464
SLCO4A1	2.41372	2.43333	4.12026	4.51942	2.16058	1.85761
SLCO5A1	-2.61075	-2.6546	-6.17309	-4.52056	2.72987	3.70691
SLF1	4.08963	4.25139	4.42089	4.4515	4.20928	4.67374
SLF2	5.10502	5.16239	5.46753	5.61526	6.18441	6.46292
SLFN11	3.9654	4.16959	-3.53313	-4.10635	5.69233	5.85064
SLFN12	3.6803	3.71954	1.5847	1.73041	3.62161	3.9135
SLFN5	5.5408	5.39628	6.69605	7.0204	5.3216	5.53108
SLFNL1	1.49179	1.24651	1.44713	1.27932	1.17889	-0.0395016
SLFNL1-AS1	1.85554	1.68846	1.71623	1.60018	1.3823	0.388983
SLIRP	4.35244	4.376	3.03556	3.52956	4.54336	5.61592
SLIT2	5.38672	5.02648	5.27693	5.19893	6.31567	6.43858
SLIT3	1.79016	1.59503	-6.17309	-4.10635	0.8704	2.0679
SLITRK4	0.792839	0.537236	0.871828	1.13916	-6.64581	?
SLITRK5	1.7314	1.78511	-6.17309	?	4.15746	3.5076
SLITRK6	3.74386	2.93804	0.156448	-0.786664	4.51897	4.14503
SLK	5.83256	5.99294	5.93149	6.1627	6.42162	6.64112
SLMAP	5.5216	5.74902	5.32129	5.59766	5.45667	5.4632
SLMO2	6.02476	6.20679	5.15461	5.71433	5.83075	6.06413
SLPI	?	-6.45019	5.42795	6.73704	?	-5.69922
SLTM	5.99852	6.01212	5.46527	5.62841	6.13957	5.7211
SLU7	4.42633	4.63188	4.53842	5.06297	4.94052	5.41157
SLX1A	2.14405	1.85185	1.84252	2.0249	2.04914	1.64257

SLX1A-SULT1A3	1.08681	1.09186	1.43063	0.188034	2.36334	1.29125
SLX1B	1.23793	1.40593	1.97042	2.09471	2.06284	2.4248
SLX1B-SULT1A4	1.68094	1.48511	1.35074	2.0498	1.54229	1.03659
SLX4	3.56301	3.41213	3.75899	3.51506	3.54793	3.10064
SLX4IP	1.76038	1.65613	3.66667	3.54581	2.8554	2.78515
SMAD1	2.30937	2.33483	1.60441	1.83372	1.76598	2.05486
SMAD2	7.12596	7.16285	5.91744	5.95016	5.51147	5.69469
SMAD3	7.23958	7.21813	6.87447	6.98359	6.05199	5.58905
SMAD4	6.14694	6.22781	5.26096	5.28625	4.51711	4.37009
SMAD5	6.33214	6.50262	6.34563	6.68011	6.03369	5.95053
SMAD5-AS1	1.04909	0.847652	0.96629	0.758005	-0.176802	-1.24306
SMAD6	1.37242	0.804012	3.71583	3.49851	2.29099	1.77713
SMAD7	2.64301	2.66902	1.46757	1.69856	1.90196	1.57873
SMAD9	-0.026521	0.013012	2.15635	1.97866	1.08794	0.100762
SMAGP	2.10474	2.25977	1.82968	2.10976	1.81131	2.21865
SMAP1	4.09355	4.18289	2.47803	2.72472	4.37733	4.53845
SMAP2	3.27821	3.66387	3.72186	4.42157	4.06335	4.48311
SMARCA1	4.91332	4.76174	6.11624	6.14474	5.97018	5.73049
SMARCA2	4.69269	4.67158	4.85402	4.76767	5.38543	5.39131
SMARCA4	5.39729	5.3078	5.20018	4.95456	5.73735	5.47194
SMARCA5	6.1847	6.29707	5.90938	6.1659	6.21909	6.46476
SMARCAD1	5.23719	5.12022	4.61333	4.68802	5.11159	5.08281
SMARCAL1	3.30654	3.26848	3.62383	3.74136	3.26599	3.34314
SMARCB1	4.86172	4.76235	3.83746	4.21196	4.56308	4.70585
SMARCC1	6.6318	6.61973	5.78332	5.95221	6.68721	6.65193
SMARCC2	5.58087	5.54995	5.58303	5.56747	4.73708	4.48072
SMARCD1	5.94721	6.04121	4.472	4.62079	4.78366	4.76394
SMARCD2	4.25146	4.9899	3.98139	4.69933	4.31123	5.2255
SMARCD3	2.8029	2.7155	3.47919	3.81302	2.05085	2.0676
SMARCE1	5.46054	5.2906	5.93756	5.93244	5.88016	5.52577
SMARCE1P5	0.0598398	0.184496	0.587576	0.835237	0.738699	0.241132
SMC1A	6.59743	6.16739	6.22192	6.05044	6.05199	5.10144
SMC2	6.24986	6.28638	5.51084	5.78674	5.64602	5.80897
SMC3	6.14106	6.17733	5.65253	5.85179	5.88045	6.00156
SMC4	7.17122	7.28581	7.44279	7.5594	7.33669	7.27368
SMC5	5.66467	5.70422	5.13783	5.14307	5.53004	5.34641
SMC6	5.12349	5.2254	4.66978	4.78447	5.25069	5.61914
SMCHD1	5.62115	5.73557	6.01459	6.11244	6.82022	6.93533
SMCO4	-3.11289	-3.87578	0.956094	1.31748	0.0540566	-0.151977
SMCR5	0.693311	0.635262	1.13846	0.82198	-0.490197	-1.38468
SMCR8	5.18522	5.14485	4.87081	4.94317	5.60532	5.11521
SMDT1	1.27825	1.14454	1.82213	1.95729	1.80324	2.21811
SMG1	7.14967	7.02479	7.0756	7.06177	7.16784	6.56347
SMG1P1	5.96811	5.99262	6.24419	6.2392	5.49008	5.35273
SMG1P2	2.68232	2.96051	3.2323	3.46979	2.7296	3.24477
SMG1P3	5.8364	5.80552	6.53773	6.48096	5.22724	5.01891
SMG1P4	3.25387	3.06906	4.12244	3.91876	3.0798	3.09544
SMG1P5	3.62969	3.45566	4.51954	4.25402	4.57026	4.52079
SMG1P6	2.2173	2.28988	1.87363	1.59098	1.65655	1.87239
SMG1P7	2.12042	2.13193	1.83114	1.77575	2.66538	2.48307
SMG5	6.30936	6.3631	5.76301	6.03518	6.81136	6.52511
SMG6	3.90552	3.75401	4.53322	4.41896	3.94815	3.51245

SMG7	5.30511	5.10557	4.96367	4.9773	5.37344	5.07463
SMG8	4.82887	4.78803	4.16631	4.41179	4.73308	4.93081
SMG9	4.00049	3.85456	3.44035	3.49665	4.09541	3.57638
SMIM10	0.207918	-0.0197685	1.97132	2.56714	2.75121	2.99373
SMIM10L1	4.10575	4.03298	3.74379	3.77594	4.0704	4.15931
SMIM11	1.98744	1.85459	2.4312	2.81004	2.19146	2.78515
SMIM12	4.1163	4.03301	3.41266	3.88553	4.46897	4.39788
SMIM13	4.61267	4.1032	3.60764	3.60369	5.60649	4.45567
SMIM14	2.39369	2.29872	3.9263	4.54401	3.66387	3.94254
SMIM15	4.68098	4.93897	4.34377	4.77764	5.51002	5.74585
SMIM19	2.30375	2.01696	1.96116	2.17659	1.97996	2.59708
SMIM20	2.43792	2.36371	1.5714	1.83372	3.01212	4.09419
SMIM3	1.39899	1.49719	4.07386	4.73822	5.22538	5.55948
SMIM4	2.09242	2.16098	1.80301	2.11589	2.49599	2.98409
SMIM5	-0.489437	-0.531907	0.610974	0.300663	0.305574	-0.619042
SMIM7	4.54567	4.50917	4.64819	4.57975	5.21668	5.52532
SMIM8	2.81466	2.77318	1.10193	1.02051	2.34813	2.40182
SMN1	4.21625	4.09457	3.97348	4.30035	4.45377	4.61487
SMN2	4.48252	4.46867	3.90694	4.29757	4.31587	4.58363
SMNDC1	3.83327	3.80511	3.93412	3.89258	4.30619	4.13249
SMO	4.44364	4.1445	4.04095	3.47088	-0.987612	-2.00571
SMOC1	-3.11289	-3.46127	1.04535	1.83956	-6.64581	?
SMOX	2.77261	2.4679	3.58632	4.08593	3.69724	3.69417
SMPD1	4.55888	4.41827	4.04529	4.30902	4.03925	4.14971
SMPD2	1.29511	1.28528	0.584424	1.11028	1.62967	2.25898
SMPD4	5.49149	5.35227	5.04862	5.22881	5.10631	4.77679
SMPDL3A	0.987481	1.27867	-0.401986	0.158078	1.87499	2.68561
SMS	5.1027	5.87048	4.41121	5.65396	5.15962	6.09826
SMTN	3.94699	3.94113	2.98514	3.31324	2.64931	2.87482
SMU1	5.19329	5.31871	4.65811	5.08745	6.46033	6.64003
SMUG1	4.27533	4.14633	3.58135	3.72089	3.85735	4.10385
SMURF1	5.21456	5.23094	5.16555	5.39403	4.93909	4.68878
SMURF2	4.88674	4.95966	5.85791	6.20028	6.08754	5.99933
SMYD2	4.8755	4.94653	3.78659	4.00231	5.11324	5.49109
SMYD3	2.95377	2.85909	3.08093	3.27919	3.34952	3.81685
SMYD4	3.42241	3.19358	4.19586	4.08593	3.53321	3.09095
SMYD5	3.53813	3.63913	3.09028	3.32997	4.06164	3.96747
SNAI1	1.83233	1.57061	1.90396	2.12481	-1.80184	-1.89883
SNAI2	-5.10914	-4.87366	3.13163	3.72563	2.01567	3.16365
SNAI3-AS1	-0.432112	-0.292758	1.39586	1.39901	-2.26731	-2.24661
SNAP23	4.79433	5.05997	4.2279	4.75612	4.5544	4.84956
SNAP25	3.09208	2.80161	2.47827	2.75846	-0.905167	-0.536591
SNAP29	3.84874	3.80158	3.72925	4.10786	2.92466	3.16653
SNAP47	4.06478	4.03224	4.02223	4.2484	4.61647	4.74669
SNAPC1	4.3615	4.15436	2.00286	2.2794	5.21217	5.43682
SNAPC2	2.05249	1.766	2.01617	2.57067	2.37037	2.30453
SNAPC3	3.99353	4.1758	4.42022	4.77152	5.83708	5.86271
SNAPC4	2.70181	2.52869	2.28817	2.28351	2.53199	2.48312
SNAPC5	3.01328	2.95094	2.78847	3.12028	3.49999	4.04618
SNAPIN	3.04912	3.14633	2.86361	3.1248	3.82753	4.54712
SNCAIP	-1.69862	-2.1403	4.13232	4.8146	-2.95876	-4.70296
SND1	7.23255	7.1215	6.83197	7.22348	6.76589	6.81766

SND1-IT1	1.97042	1.90695	1.99422	1.86899	0.874427	0.238163
SNED1	2.26782	2.36409	2.69395	2.63814	1.6881	1.09753
SNF8	4.99478	5.00781	4.85392	5.14138	4.98794	5.06529
SNHG1	6.46015	6.48351	5.75862	5.9826	5.84474	5.97276
SNHG10	2.6882	2.76955	0.899137	1.17055	2.21936	2.01096
SNHG11	2.8107	2.72204	2.05967	1.94648	1.34536	1.40185
SNHG12	4.70245	4.75035	3.38864	3.60063	5.02606	5.00873
SNHG14	4.55062	4.48796	2.30433	1.74295	3.95439	3.581
SNHG15	3.84685	3.92106	2.60139	3.127	4.02618	4.43874
SNHG16	8.01602	7.30931	5.99646	5.56179	8.41827	7.54865
SNHG17	4.84443	4.801	3.52556	3.67744	4.09791	3.88574
SNHG18	0.41996	0.151976	2.31634	2.375	-4.33548	-3.38329
SNHG19	1.45578	1.05291	0.386425	0.619178	1.11453	1.38079
SNHG20	3.10077	3.18924	2.19608	2.43755	3.4298	3.17235
SNHG21	1.54397	1.45202	0.733632	0.816206	1.64837	1.1755
SNHG22	2.74387	2.61398	2.14293	2.16745	0.706561	0.597145
SNHG25	0.619789	0.472394	-1.53701	-1.35543	1.02625	2.43792
SNHG3	6.71514	6.81897	5.23503	5.39451	6.89575	6.57086
SNHG4	3.72788	3.63904	2.1923	2.31293	3.536	3.16973
SNHG5	5.50579	5.55395	4.05965	4.64689	4.02531	4.51728
SNHG6	3.73918	3.67902	3.29747	3.51459	4.32253	4.79576
SNHG7	3.63871	3.58002	2.57559	2.96936	3.27729	3.71148
SNHG8	3.58632	3.57333	1.91453	2.40689	4.50648	5.2777
SNHG9	0.266817	-0.27286	-0.276471	-0.275776	0.210164	0.668401
SNIP1	3.90176	3.91544	3.28815	3.54222	4.05307	4.04984
SNN	2.69539	2.24453	3.47647	3.60198	3.32838	3.02121
SNORA12	1.50063	1.48556	1.62387	1.60545	1.22238	2.41742
SNORA21	1.30588	1.59951	0.789571	1.68581	-0.170264	-0.0360909
SNORA22	1.09539	1.45008	1.37111	1.17662	0.932212	0.816919
SNORA23	3.95017	3.96027	3.16963	3.1055	3.03492	3.63758
SNORA31	-0.651786	-1.49394	1.27945	1.28601	1.64113	0.850615
SNORA34	1.38311	1.33154	1.33222	1.42259	0.351018	0.443129
SNORA38	0.684702	0.6247	0.431281	0.685662	0.932212	1.06153
SNORA42	0.693311	0.467978	0.976408	0.978694	-0.932127	-1.18284
SNORA43	2.51121	2.59063	2.19558	2.18656	1.71424	2.47852
SNORA48	4.91857	4.74365	4.81274	5.13036	5.19247	5.60084
SNORA49	2.84782	2.88146	2.7909	2.52054	1.91336	1.76905
SNORA52	2.75739	2.86482	2.06398	2.31957	1.79152	2.77569
SNORA53	4.06247	4.21223	3.3302	3.52416	3.41784	4.91075
SNORA54	0.54893	0.794611	0.445928	0.773887	-0.393347	0.401903
SNORA57	2.90976	2.62439	1.43714	1.71633	1.85238	3.23118
SNORA61	0.950334	1.14338	-0.165909	-0.047249	0.542228	0.729453
SNORA63	4.24008	4.17813	4.78195	4.68285	5.01456	5.46369
SNORA68	2.34105	2.4184	1.83235	1.8508	1.95923	3.65875
SNORA70	3.16271	3.40111	3.33918	3.27962	2.89442	3.60364
SNORA71A	3.94206	3.94336	3.44583	3.66101	3.22539	3.42771
SNORA71C	1.43024	1.20963	0.355738	0.773887	0.479334	0.33778
SNORA71D	1.96983	1.86361	1.24279	1.35065	0.634389	1.70271
SNORA73A	8.59504	8.68801	7.90541	7.8963	8.37747	8.89619
SNORA73B	8.2961	8.33648	7.22585	7.26158	8.01695	8.72294
SNORA74	0.133932	-0.140771	-0.510905	-0.682344	0.0263185	0.805664
SNORA74A	4.02883	3.99453	3.2718	3.27272	3.86717	4.95772

SNORA74B	0.840145	0.467978	1.73537	1.30907	1.03324	1.99375
SNORA77	0.581891	0.761881	0.333859	0.390657	-1.05345	0.352273
SNORA79	2.91379	2.78508	2.12484	2.10065	2.04015	2.44305
SNORA7A	1.13418	1.32658	0.347348	0.806506	1.19146	2.54558
SNORA80A	0.345419	0.503023	0.584741	0.978694	-0.374735	0.769095
SNORA80B	-0.289526	0.122235	-1.05395	-0.55404	-0.637731	1.42261
SNORD10	3.64706	3.67401	3.75317	3.59183	3.27216	4.52258
SNORD13	0.897388	0.896388	1.30071	1.32958	1.50377	0.411415
SNORD15B	2.83426	2.80862	1.91979	2.03585	1.57335	2.13879
SNORD17	7.73925	7.53706	7.08968	6.95034	6.56428	7.56532
SNORD3A	12.0744	11.8043	12.206	12.4204	10.6212	12.78
SNORD3B-1	2.96165	3.49618	5.07954	5.36183	7.67103	10.0065
SNORD3B-2	2.0752	2.42545	5.25223	5.18583	7.5753	9.96497
SNORD3C	3.03015	3.5608	3.79223	3.90496	1.18649	3.01116
SNORD3D	8.64749	8.78681	8.89384	9.11293	8.28539	9.10008
SNORD94	1.04244	0.885949	0.401532	0.845438	0.134221	1.25912
SNORD97	2.86123	2.81765	2.76394	2.99226	2.9481	3.26315
SNPH	1.18361	1.14454	2.33219	2.74605	-2.33767	-1.53638
SNRK	4.41506	4.45097	4.07839	4.29482	4.62384	4.17073
SNRNP200	7.67512	7.71835	6.8314	7.01925	7.74048	7.5444
SNRNP25	1.81369	1.94682	2.88844	3.43851	3.31368	3.62428
SNRNP27	3.61606	3.50005	3.06203	3.38601	4.3411	4.29707
SNRNP35	2.84487	2.92538	2.18767	2.352	2.48691	2.3058
SNRNP40	5.42904	5.39363	4.58961	5.03927	5.21842	5.42643
SNRNP48	4.0633	4.18909	3.91845	4.03583	4.27337	4.10867
SNRNP70	5.45762	5.39552	5.58548	5.70214	6.07821	5.39329
SNRPA	5.02111	4.8985	4.46659	4.7327	4.85336	4.68876
SNRPA1	3.3441	3.29208	3.27281	3.42303	3.36403	3.11296
SNRPB	6.3554	6.2494	5.90749	6.4252	5.83379	6.35566
SNRPB2	7.65753	7.37472	4.65489	5.06358	4.81188	5.35653
SNRPC	5.11177	4.98596	4.4937	4.89631	5.77389	6.09072
SNRPCP2	-0.192533	0.0544873	-0.615747	-0.319134	0.749551	0.623595
SNRPD1	5.33263	5.33004	4.18392	4.56613	5.35333	5.5497
SNRPD2	6.20685	6.13287	5.37105	5.82042	6.66671	7.50726
SNRPD3	5.86436	5.96934	5.43615	5.81529	5.51781	5.64356
SNRPE	4.43372	4.18597	3.51483	3.81361	4.54615	4.80425
SNRPEP4	3.77741	3.57237	3.00558	3.30698	3.90783	4.81684
SNRPF	4.23541	4.25166	3.81623	4.24954	3.70583	3.84574
SNRPFP1	1.18922	1.40559	0.937307	1.33631	0.95561	0.635169
SNRPG	4.15922	4.18186	3.17334	3.45845	4.19047	5.03401
SNRPGP10	2.66143	2.66747	1.44097	2.00959	2.62275	3.55042
SNRPGP15	0.837959	0.553154	-0.352529	-0.149767	0.62728	1.0624
SNRPN	3.43544	3.3863	-11.1841	-6.09892	3.21621	3.37142
SNTA1	1.40948	0.766044	2.91977	2.87132	2.28515	1.97279
SNTB1	4.41393	3.83558	3.40481	3.65213	1.20791	0.648208
SNTB2	5.18177	5.0355	6.12528	6.43678	6.10792	5.59763
SNU13	5.50762	5.52867	4.8499	5.30796	5.43277	5.71532
SNUPN	3.55663	3.4545	2.50649	2.86639	2.97843	3.42667
SNW1	5.14478	5.28594	4.22246	4.62661	4.60822	4.90434
SNX1	6.41564	6.1514	5.65448	5.72485	5.38339	5.46508
SNX10	1.27825	1.67161	-1.93506	-1.06415	0.270529	1.3157
SNX11	3.6441	3.47232	2.68377	3.00492	3.80932	4.0263

SNX12	4.36701	4.52865	4.31133	4.86486	5.20429	5.68073
SNX13	6.03032	5.91272	6.31531	6.33309	5.79218	5.95683
SNX14	5.58565	5.67126	4.71795	5.02191	5.74753	6.50723
SNX15	1.08116	0.620305	0.937164	1.64655	1.54849	1.49207
SNX16	1.84784	1.7708	2.04532	2.14858	2.60648	2.73624
SNX17	5.37008	5.32852	4.64062	4.93689	5.67503	6.28702
SNX18	3.52008	3.5623	5.92042	5.98327	5.57241	5.21065
SNX18P12	0.570074	0.557836	1.8685	1.4727	-0.944924	-1.57156
SNX18P1Y	-0.871725	-0.641764	0.162519	-0.00228849	0.619385	0.220491
SNX19	5.33742	5.38964	6.07505	6.3367	6.62973	6.65302
SNX2	4.97024	4.85738	4.71734	5.05105	4.83852	5.26697
SNX21	4.18359	4.10781	3.85799	4.11323	3.13839	3.2077
SNX22	5.31617	5.03795	4.97728	5.1469	4.61172	4.67684
SNX24	3.61495	3.57259	2.4327	2.78903	3.22233	3.53407
SNX25	4.33308	4.37532	3.41549	3.62721	3.54612	3.70082
SNX27	5.13892	5.24149	4.568	4.85333	4.85931	4.93713
SNX29	2.76029	3.07049	4.52208	4.85616	3.28074	3.25618
SNX3	4.37035	4.31852	4.71883	4.9859	6.39213	6.61857
SNX30	4.95621	5.20966	3.74878	3.87071	4.32708	4.19565
SNX32	0.983802	1.14799	0.948309	1.14663	-0.0206536	-0.106816
SNX33	4.26271	3.9163	4.37258	4.53845	3.78908	3.50144
SNX4	3.79777	3.84549	3.75899	4.02174	4.22386	4.57984
SNX5	7.0405	6.98154	6.14129	6.33925	6.48485	6.61871
SNX5P1	1.93107	1.87384	1.08298	1.58907	1.5526	1.85887
SNX6	4.3829	4.42637	3.9808	4.40018	5.06172	5.68958
SNX6P1	-0.039389	0.331671	-0.0723326	-0.201784	0.135968	0.225324
SNX7	3.77462	3.98931	3.44751	3.82633	4.73201	5.53105
SNX8	3.52973	3.07825	3.62383	3.86127	4.62797	4.5076
SNX9	4.45925	4.61729	4.19654	4.63622	5.03032	5.28218
SOAT1	6.50987	6.35271	5.99703	6.39173	5.56614	5.59594
SOBP	1.78476	1.07637	3.54269	3.5022	-0.802092	-0.577229
SOCS2	3.13155	2.05907	-0.220845	0.049338	3.37205	3.62907
SOCS3	3.96803	2.85909	4.40799	5.06046	4.54792	4.85743
SOCS4	5.22201	5.31232	4.50734	4.79051	5.353	5.23944
SOCS5	3.63629	3.71332	5.74718	5.97431	5.84518	6.10745
SOCS6	5.83984	5.70988	4.3013	4.53952	3.95624	3.96923
SOCS7	4.69912	4.6926	3.95346	4.02431	5.04531	4.54121
SOD1	5.33746	5.41907	6.20181	6.80258	5.4821	6.07549
SOD2	8.29857	7.17832	5.50091	6.84934	5.92425	6.07002
SOD3	2.07902	0.766044	-3.85951	-5.10387	?	-4.70296
SOGA1	6.72375	6.6337	6.01184	6.01788	5.12839	5.21734
SOGA3	1.41816	1.59261	0.626738	0.930026	2.63554	3.20174
SOHLH2	4.6261	4.65024	-4.5955	-3.78492	-2.49202	-0.90064
SON	7.14768	7.06157	8.14331	8.09549	7.22306	7.04893
SORBS1	3.57472	3.46642	2.22589	2.08102	4.12515	3.51969
SORBS2	3.58979	3.01492	-2.72315	-1.15432	4.73748	4.882
SORBS3	2.38078	2.41874	2.59773	2.65605	2.00149	2.23582
SORD	4.40383	4.39634	4.68359	4.86681	3.58729	3.87386
SORD2P	2.18225	2.43032	1.90508	2.01674	2.21706	2.53804
SORL1	-6.68418	-6.45019	-2.01303	-1.35358	3.7764	4.29328
SORT1	6.68437	6.51786	5.17948	5.17249	7.64001	7.23066
SOS1	4.70443	4.61596	5.29323	5.2613	4.87349	4.807

SOS1-IT1	0.0559314	-0.39658	0.838911	0.383121	0.222446	-0.662104
SOS2	3.93754	4.029	3.81648	3.99576	3.97633	4.3087
SOWAHC	4.17973	4.61196	1.83331	2.16265	1.74911	1.76905
SOX11	4.85166	4.74599	3.21094	3.56005	-6.64581	-3.70484
SOX12	2.62736	2.41981	3.58796	3.53503	2.90765	2.35939
SOX13	1.96272	2.09942	2.1019	2.35062	4.5631	4.35127
SOX2	3.10989	3.02299	?	-6.09892	6.87944	6.51758
SOX2-OT	0.219896	-0.0197685	-4.59508	-4.52056	7.14495	7.20372
SOX21-AS1	-6.68418	-5.45651	?	?	3.64803	2.80505
SOX4	3.84201	3.51157	5.15465	4.88834	5.85291	5.55537
SOX5	6.4606	6.10819	2.50299	2.35471	4.64632	4.23139
SOX6	4.2078	3.72327	1.46757	0.491176	0.568587	-0.662104
SOX9	-5.10914	-5.45651	3.52901	3.38102	5.55885	4.9824
SP1	6.17805	6.11481	6.24327	6.43832	5.85194	5.642
SP100	4.57788	4.60352	4.63817	5.08927	5.23874	5.48341
SP110	2.33226	2.07292	3.36431	3.66451	3.60909	3.70512
SP140L	3.26612	3.43567	3.67561	3.99193	3.87408	3.6265
SP2	1.00142	0.746683	1.00636	0.810106	0.815493	0.100762
SP2-AS1	1.27825	1.23764	1.30034	1.21315	0.782702	0.50283
SP3	6.33277	6.38166	6.8792	7.06236	6.53409	6.86003
SP4	3.8999	3.66127	3.44947	3.36894	3.53936	3.08771
SPA17	0.941046	1.00378	2.46819	2.86847	0.925354	2.07964
SPACA6P	1.70984	1.64139	3.00521	3.06741	3.53468	2.78046
SPACA6P-AS	-0.448705	-0.466606	0.437782	0.839315	0.72181	-0.452211
SPAG1	1.80791	2.00891	1.84257	2.50408	2.93008	2.98461
SPAG16	1.72721	1.66131	2.80233	2.85697	2.56129	2.96925
SPAG17	-2.04797	-1.50354	-2.85671	-3.30006	6.34336	6.26181
SPAG4	-0.489437	-0.122851	2.05967	1.83372	-0.530833	-1.06257
SPAG5	6.03383	6.02892	6.19628	6.5891	6.38872	6.21463
SPAG5-AS1	1.89729	2.10556	2.11444	2.26503	2.71213	2.44976
SPAG7	3.51158	3.4899	3.80232	4.03838	4.31911	4.62867
SPAG9	6.79651	6.85694	6.28941	6.55205	7.13187	6.97451
SPARC	8.78374	8.91169	9.38758	9.74446	10.4937	10.4874
SPAST	4.53454	4.56437	3.42381	3.5476	4.57811	4.34584
SPATA13	5.6897	5.69669	4.30531	4.53321	4.6006	4.4097
SPATA2	2.94298	2.93162	2.84162	3.03328	2.79912	2.68561
SPATA20	3.53094	3.42133	4.64381	4.82264	1.72774	2.2475
SPATA24	0.0954519	-0.629746	0.102	0.19507	0.909612	0.97985
SPATA2L	1.26675	0.885949	-0.0541032	0.213217	0.293994	1.08775
SPATA33	2.38308	2.43502	2.12938	2.22211	1.84354	1.37014
SPATA5	5.30794	5.39279	4.867	4.95119	5.15788	5.14894
SPATA5L1	2.78877	2.74905	1.60441	1.94105	2.23755	2.18205
SPATA6	4.12269	4.16196	1.28413	1.25754	3.47668	3.38604
SPATA6L	-1.89035	-0.818747	-3.37629	-3.52222	1.07446	1.80111
SPATA7	0.7459	0.645757	1.2116	1.29296	0.73187	1.24809
SPATC1L	-0.632781	-1.00314	1.6807	1.57771	0.395063	-0.121607
SPATS2	4.40748	4.25654	4.56969	4.5724	4.20928	3.68345
SPATS2L	5.1199	5.22321	4.66243	5.20007	5.84177	5.93035
SPC24	4.72612	4.69639	4.48535	4.636	5.39939	5.4232
SPC25	3.03051	3.17559	2.38255	2.4799	2.58288	3.02461
SPCS1	5.25743	5.45337	4.646	5.04304	5.50977	6.08717
SPCS2	4.41128	4.5377	4.69687	4.90142	3.92474	3.58519

SPCS2P4	4.16059	4.16397	4.34192	4.75566	3.79594	3.8386
SPCS3	6.39334	6.43139	6.81275	6.92045	6.62494	6.6087
SPDL1	4.37037	4.60458	4.72411	5.10972	5.20232	6.05399
SPDYA	0.65172	0.829671	0.433387	0.0947225	1.97321	1.95062
SPDYE1	0.59807	0.481609	1.71481	1.33892	0.585837	-0.637756
SPDYE11	0.17291	0.61696	0.665829	0.909842	-0.178344	-0.764704
SPDYE16	0.366789	0.317107	1.11789	0.793896	0.608488	-0.448363
SPDYE2	1.6014	1.41758	2.1874	2.03928	1.501	0.0277769
SPDYE2B	0.308885	-0.285342	0.444487	0.732269	0.405796	-0.763256
SPDYE3	1.0704	1.28855	0.968732	1.05571	0.91873	0.725689
SPDYE5	1.22453	1.34381	1.61487	1.16693	0.327768	-0.46741
SPDYE6	1.35428	1.3195	1.45734	1.57599	1.46797	0.610303
SPDYE9P	-0.616698	0.0477338	0.876951	-0.0140887	0.0731621	-0.840009
SPECC1	-2.30606	-2.76144	6.25162	6.38926	7.4551	7.52107
SPECC1L	5.9836	5.69478	5.8053	5.74663	5.39508	4.76921
SPEG	2.27535	1.90454	2.49023	2.19953	4.2208	3.95541
SPEN	6.01859	5.96367	5.92172	5.80368	5.87957	5.69522
SPG11	5.17059	5.1602	5.07353	5.0601	5.60502	5.76406
SPG20	5.53156	5.64894	5.24222	5.60369	5.25255	5.5965
SPG21	4.24196	4.37948	4.40893	4.8388	4.6823	5.10947
SPG7	5.03595	4.98939	5.23801	5.27162	4.52826	4.03811
SPHK1	2.08884	1.92519	1.12031	1.54226	2.15349	2.20619
SPHK2	1.99287	1.23573	2.00607	1.99328	2.5041	2.29303
SPICE1	4.06993	4.09557	4.15632	4.03072	3.93028	3.49786
SPIDR	5.03647	4.82832	5.16686	5.23546	4.04619	4.04315
SPIN1	5.25148	5.25396	4.93606	5.16612	5.66272	5.87964
SPIN2A	-0.0190695	-0.31204	0.774621	0.206831	0.689362	0.00319923
SPIN2B	1.05233	0.903154	1.42138	1.5448	2.44797	2.80766
SPIN3	1.19579	1.35098	1.29226	1.36695	2.20079	2.22404
SPIN4	2.23758	2.62463	2.50998	2.46861	3.01212	2.61973
SPIRE1	3.39019	3.2336	3.95218	4.14008	4.7956	4.35633
SPIRE2	2.45575	2.28536	2.5273	2.67909	1.46901	1.13883
SPNS1	3.52832	3.22993	3.45158	3.27494	3.17878	3.23413
SPOCD1	1.02889	1.46339	-0.161006	1.04469	0.437814	1.32676
SPOCK1	6.02946	6.05656	2.8305	2.89118	5.67676	5.95326
SPON1	0.529821	0.498384	1.11905	1.16735	1.51833	1.25563
SPOP	4.81365	4.74721	4.47468	4.56713	4.52456	4.74243
SPOPL	4.48724	4.17984	3.89453	3.7731	5.59505	5.0285
SPP1	5.5258	6.17354	2.9713	3.61227	7.4775	8.3409
SPPL2A	5.41507	5.5847	4.2553	4.77639	5.68111	5.90785
SPPL2B	2.2493	1.8318	2.86088	2.93284	2.32555	2.14505
SPPL3	4.54529	4.49425	3.58796	3.59161	3.51835	3.67266
SPR	3.50548	3.35893	3.55458	3.80555	3.73522	4.17133
SPRED1	5.91309	6.00215	4.50955	4.73666	4.90764	4.75884
SPRED2	4.72613	4.65284	3.44947	3.22209	3.52705	3.36475
SPRED3	2.01862	2.08021	1.18285	1.51326	2.22845	1.60621
SPRN	1.39442	0.937164	1.38959	1.0165	1.5342	1.19821
SPRTN	3.8079	3.9672	3.53407	3.87112	3.8392	3.85791
SPRY1	0.567789	-0.177294	2.48892	1.52784	2.83155	1.95152
SPRY2	5.82543	5.47452	2.79662	2.63938	3.68288	3.3834
SPRY3	0.00148521	-0.292758	0.914572	1.37503	0.339787	0.200291
SPRY4	5.72928	5.73715	3.56467	3.50219	3.49071	2.85171

SPRY4-IT1	1.56774	1.72206	0.045429	-0.353769	-1.53062	-2.70579
SPRYD3	2.68463	2.61931	1.8772	2.1152	3.29389	3.32947
SPRYD4	2.42744	2.4014	3.39903	3.47695	3.25441	3.1778
SPRYD7	1.99094	2.06855	1.7995	2.57418	2.65484	2.61071
SPSB1	2.31216	2.09176	3.05966	3.4049	0.878914	0.832517
SPSB2	1.511	1.48556	2.08796	2.29633	1.27637	1.43288
SPSB3	1.66056	1.06086	1.86795	1.94293	1.6731	2.01507
SPTA1	-1.41331	-1.41808	3.47647	3.26839	?	?
SPTAN1	6.96828	7.00855	7.06691	7.25321	7.12131	6.82537
SPTBN1	8.63273	8.69649	8.28843	8.43886	8.53596	8.16941
SPTBN2	0.855575	0.766044	2.4164	2.3424	3.72231	3.65523
SPTLC1	4.39776	4.72292	4.16168	4.66941	4.05512	4.65248
SPTLC1P1	0.787207	1.19605	0.956302	1.38659	0.491607	0.938286
SPTLC2	5.64214	5.54135	4.35175	4.39599	5.11693	5.12854
SPTLC3	0.108397	0.151976	2.9839	3.71771	2.69945	3.32119
SPTSSA	3.79977	3.80978	4.06559	4.52143	4.45798	3.91534
SPTY2D1	4.50877	4.76833	4.45927	4.96164	4.66847	5.0224
SQLE	5.80176	5.94732	5.13727	5.58291	4.98937	5.47258
SQRDL	-2.45229	-3.29152	3.52025	4.47569	2.12008	3.15064
SQSTM1	7.44101	7.51392	8.78994	9.6815	8.40813	9.02009
SRA1	4.03332	4.0051	4.12711	4.4256	4.83185	5.18441
SRBD1	4.31214	4.21576	4.10072	4.27487	4.25481	4.47816
SRC	3.72928	3.80861	3.94576	3.93966	1.31128	1.46325
SRCAP	4.44928	4.04019	4.81399	4.39471	4.06184	4.12781
SRD5A1	3.85933	3.75026	4.41638	4.4515	4.55522	4.0465
SRD5A3	3.05681	3.48929	1.62155	2.07192	2.9117	4.14006
SREBF1	4.80623	4.50554	5.22449	5.34555	6.35279	6.63785
SREBF2	6.30841	6.15246	5.73893	5.76406	6.25445	6.08636
SREK1	6.58888	6.59494	6.93425	6.80474	7.10981	6.55496
SREK1IP1	4.24368	4.11707	4.04769	4.04092	4.67675	4.60389
SRF	3.64188	3.57469	2.92239	3.1835	4.2917	3.99025
SRFBP1	4.05327	4.00736	3.9286	4.19989	3.88023	4.22836
SRGAP1	4.36379	3.89054	3.74503	3.77674	3.66444	3.11347
SRGAP2	6.39779	6.12785	5.95114	6.0096	5.08812	4.72428
SRGAP2-AS1	1.57148	1.47246	1.75831	1.6021	0.978269	0.15694
SRGAP2B	5.76736	5.73872	4.8065	4.89489	4.87109	4.39096
SRGAP2C	5.84816	5.76582	4.94372	5.21281	6.23261	5.95452
SRGAP2D	3.43341	3.29781	2.32204	2.8289	2.77216	2.48243
SRGAP3	0.463706	0.55615	0.738145	0.452743	4.46136	3.97683
SRGN	3.68029	3.69702	6.41918	7.18468	-1.30241	-0.0341529
SRI	5.35041	5.41788	5.46168	6.05261	5.00456	5.3198
SRM	3.92389	3.59108	3.03565	3.40293	4.732	4.71849
SRP14	5.61196	5.52575	5.03135	5.33665	5.75795	6.10104
SRP14-AS1	-0.272655	-0.707737	0.445928	0.646144	-0.10558	-0.00614162
SRP19	4.48171	4.08728	4.23545	4.16942	5.05701	4.74089
SRP54	4.75161	4.82369	3.9145	4.34341	5.13817	5.26768
SRP68	4.9861	4.86526	4.81646	4.98656	5.13732	4.85839
SRP72	6.01998	6.07355	5.53234	5.92044	7.62388	8.10021
SRP72P1	0.176629	0.387385	-0.15641	0.0668814	1.90052	2.11161
SRP72P2	1.48583	1.71211	1.31637	1.66421	3.05197	2.8451
SRP9	6.07151	6.13344	6.42458	6.6724	7.01971	7.0411
SRP9P1	2.91246	2.763	2.99485	3.35931	3.80007	4.22077

SRPK1	6.17238	6.3023	5.91377	6.33634	6.13353	6.30204
SRPK2	5.41644	5.38304	5.15756	5.14643	5.11525	4.91576
SRPK2P	0.370443	0.139823	0.310538	0.0612232	0.417942	0.0747657
SRPR	5.85762	5.86805	5.91053	6.10061	6.25707	6.24347
SRPRB	5.11958	5.07029	5.08765	5.499	5.34496	5.71854
SRPX	5.43471	5.5396	3.69452	4.02302	3.84648	3.908
SRPX2	3.03733	2.91654	2.66511	3.0686	4.65256	4.74243
SRR	2.76024	2.65474	3.35717	3.57208	2.86336	2.94399
SRRD	4.10004	4.1979	2.93507	2.85759	3.67999	3.76256
SRRM1	5.95036	5.79243	5.7129	5.52516	5.96457	5.56585
SRRM1P3	0.609027	0.689415	1.24822	1.05392	0.691436	0.344079
SRRM2	7.27559	7.04882	7.79798	7.52659	7.26423	7.00389
SRRM5	0.397058	0.214684	-0.0203653	-0.13126	0.574741	-0.710831
SRRT	5.69884	5.59107	5.2443	5.49433	5.62537	5.26194
SRSF1	7.97011	7.99733	7.27323	7.52027	8.11307	7.84753
SRSF10	6.90587	6.86573	6.8769	6.90404	6.82254	6.53072
SRSF11	6.89054	6.77913	7.27013	7.15619	7.05308	6.34754
SRSF2	7.35837	7.39435	6.77029	7.03793	7.54844	7.22392
SRSF3	7.28441	7.28927	6.70388	6.99831	7.38667	7.67954
SRSF4	5.13815	5.15462	5.04588	5.21591	5.95809	5.39525
SRSF5	6.12507	6.1733	5.36095	5.55561	6.09499	5.79163
SRSF6	6.72376	6.69162	6.33827	6.46792	6.25501	6.30138
SRSF7	6.63043	6.66932	5.75715	6.09114	6.56383	6.80922
SRSF8	3.28534	3.5992	2.72338	2.97599	4.03665	4.13406
SRSF9	5.70144	5.62136	5.16158	5.4125	5.73505	5.60818
SRXN1	4.88333	4.8259	6.72305	7.23584	4.85855	5.03439
SS18	5.58256	5.91329	4.92172	5.49387	4.59176	4.6242
SS18L1	4.38306	4.42361	3.87987	3.82633	4.48434	3.88013
SS18L2	2.73979	2.61931	1.89865	2.1058	3.03869	3.79731
SSB	6.06147	6.13187	5.15183	5.54464	6.26716	6.46586
SSBP1	5.30793	5.26549	4.64381	5.06484	5.62749	6.20205
SSBP2	1.98744	1.47971	5.09026	5.02688	4.11858	4.02968
SSBP3	2.03857	1.62994	3.46136	3.28135	2.65781	2.28596
SSBP3-AS1	-0.768111	-0.734698	1.32432	0.989263	-0.136604	-0.752286
SSBP4	1.51232	1.02667	1.51478	1.52832	1.48496	1.41777
SSFA2	7.00131	6.89633	7.63585	7.66254	6.79142	7.0998
SSH1	6.91796	6.52067	5.63705	5.21872	4.41917	3.14816
SSH2	4.53152	4.31851	5.61695	5.50999	5.6213	5.14893
SSH3	2.31495	1.95924	2.58798	2.74293	1.53448	1.67702
SSNA1	3.45952	3.32832	2.56803	2.92735	3.18831	3.92991
SSPN	1.2076	1.63379	0.138526	0.563685	1.18103	1.18931
SSR1	6.94871	6.72094	6.76317	6.73706	7.37135	7.19808
SSR2	7.85508	7.77617	6.7224	6.98957	7.67214	7.7596
SSR3	6.77821	6.80016	6.92441	7.36556	7.22149	7.46361
SSR4	5.13669	5.02764	5.28451	5.71238	5.34326	6.05032
SSR4P1	0.701877	0.581631	1.0357	1.31748	-1.23372	-2.00571
SSRP1	7.00403	6.93331	6.57355	6.81812	7.27174	6.92611
SSSCA1	3.31049	3.17115	2.14919	2.50525	2.58806	2.92917
SSTR2	0.577131	0.707154	2.47044	2.49404	0.107996	-0.348991
SSU72	4.81021	4.69071	4.62623	4.89818	5.58702	5.63146
SSX1	1.8708	1.92155	-6.17309	-4.52056	?	?
SSX2B	3.45941	3.48905	-4.59508	-7.06267	?	?

SSX2IP	4.78825	4.92785	4.27999	4.51322	5.70108	6.06103
ST13	6.01417	5.80571	5.95935	6.18975	6.08958	5.71464
ST13P15	2.91131	2.84192	3.14216	3.17445	3.12226	2.32411
ST13P18	1.65547	1.63506	1.65745	1.8894	1.47629	1.56908
ST13P19	2.60161	2.55591	2.40987	2.76964	2.75546	2.26485
ST13P3	2.24351	1.9238	2.09495	2.22287	2.31469	1.76513
ST13P4	3.28614	3.12226	3.1104	3.43661	3.50645	3.36032
ST13P5	3.1131	3.04418	3.04279	3.3989	3.26771	2.88327
ST13P6	4.1307	3.91713	3.95816	4.18982	4.20869	3.84495
ST20	1.35544	1.22047	0.658664	0.822526	-0.450654	-0.169021
ST20-AS1	0.545168	0.288004	0.487353	0.905413	-0.158774	0.0799754
ST3GAL1	4.87268	4.52583	6.6404	6.46126	5.75564	5.34041
ST3GAL2	4.31284	4.24279	4.88256	4.98129	5.23185	5.1419
ST3GAL3	1.31777	1.45008	2.31235	2.44193	2.21315	2.03478
ST3GAL4	6.56813	6.62806	3.37298	3.73195	3.95256	4.10385
ST3GAL4-AS1	2.50795	2.80862	1.89332	2.13437	0.509706	0.668401
ST3GAL5	4.71983	4.71582	1.41643	1.38307	-0.302594	0.200291
ST3GAL6	3.72523	3.59505	3.4125	3.09575	2.55159	1.95152
ST5	3.51493	3.13051	4.63358	4.66172	4.57862	4.40321
ST6GAL1	4.15578	4.20337	2.11112	2.11519	-3.19956	-3.38329
ST6GALNAC2	3.06464	2.78499	0.838097	0.786596	3.55618	3.14188
ST6GALNAC3	3.22425	3.36052	-2.5977	-2.02079	2.72124	3.44811
ST6GALNAC4	2.2696	2.02502	2.8305	3.16963	2.97994	3.49786
ST6GALNAC5	-0.791952	-1.37502	-3.85951	-2.78591	2.00505	2.11348
ST6GALNAC6	2.95546	2.58064	3.99926	4.32567	3.11216	3.21367
ST7	3.47156	3.58969	2.71884	3.01312	3.50722	3.94466
ST7-OT4	0.773287	0.846762	0.823285	0.479717	-0.108509	-0.380936
ST7L	3.28778	3.56081	3.35906	3.58106	3.43335	3.82904
ST8SIA1	3.55948	3.04098	2.79948	2.43808	-2.07475	-1.89883
ST8SIA4	2.45322	2.40135	-3.59683	-4.52056	3.00147	2.75682
ST8SIA5	7.60357	7.71533	-2.72315	-2.20128	-3.48878	-2.70579
STAC	2.3233	2.88146	2.5512	3.19952	5.13531	5.36406
STAG1	5.44169	5.56818	4.79045	5.25724	4.5342	4.96445
STAG2	6.66084	6.63534	6.23801	6.53523	6.87831	7.30172
STAG3	2.48317	2.10491	2.9641	2.61113	1.77374	0.803723
STAG3L1	0.826835	0.609348	1.12068	0.95954	0.813566	0.514865
STAG3L2	3.39746	3.32471	3.45489	3.44558	3.32311	2.70333
STAG3L3	4.24032	4.10351	3.73905	3.84155	3.75065	3.25067
STAG3L4	2.41723	2.14923	2.37017	2.64253	2.61167	2.58477
STAG3L5P	3.73047	3.74088	3.73816	3.86854	3.40306	2.55778
STAG3L5P-PVRIG2	3.99132	3.78167	4.11864	4.25767	3.81947	2.66775
STAM	4.0558	4.06853	3.32823	3.57767	3.5888	3.44811
STAM2	3.61833	3.8936	3.60111	4.11155	4.24357	4.96394
STAMBP	4.83229	4.99708	4.23221	4.63431	5.74029	5.8266
STAMBPL1	3.1115	3.05286	2.47113	3.0611	3.22844	4.04148
STAP2	0.558395	0.407614	1.16526	1.48367	1.62974	2.03478
STARD10	2.50258	2.08535	2.28245	2.19872	0.242328	0.501964
STARD13	6.17452	5.80008	3.66906	3.80528	4.99662	4.64771
STARD13-AS	4.70597	4.33737	2.22361	2.23629	3.02939	2.65012
STARD3	3.50548	3.37005	3.59784	3.72248	3.94052	4.11026
STARD3NL	4.51402	4.55535	5.06617	5.378	4.49133	5.15669
STARD4	5.94266	5.76428	5.02759	5.33169	5.35746	5.34116

STARD4-AS1	3.55676	3.39864	3.68443	3.28771	3.12919	2.41104
STARD5	1.41307	1.5284	1.08657	1.52876	0.768417	0.615944
STARD7	6.76285	6.88537	6.03595	6.50604	6.98798	7.01067
STARD7-AS1	0.43312	0.013012	0.431281	0.689362	0.973156	0.95839
STARD8	1.48583	1.15192	-0.568612	-1.02108	1.08121	1.50278
STARD9	5.02758	4.84948	4.99885	4.65604	5.14502	4.25328
STAT1	5.41823	5.46569	5.72109	6.18189	7.09477	6.94479
STAT2	3.61153	3.53997	5.19206	5.66512	5.49512	5.073
STAT3	5.7915	5.76743	6.26407	6.62417	7.2494	6.9734
STAT5A	1.41857	1.57569	-2.04277	-1.15204	0.61744	0.875316
STAT5B	4.13875	4.13248	5.12445	5.42398	4.81786	4.76921
STAT6	-1.8912	-2.6546	3.05727	3.40095	-3.07415	-3.70484
STAU1	6.27066	6.31317	6.19094	6.45457	5.95917	5.92898
STAU2	3.9679	3.96656	4.53221	4.47689	4.7124	4.76926
STC1	2.64523	2.94655	6.41409	6.88994	5.78989	5.11187
STC2	4.77208	4.33724	5.25266	6.03901	5.4225	4.21065
STEAP1	2.40802	2.53722	2.24544	2.9237	-2.01588	-2.62443
STEAP1B	2.04425	1.92511	-3.51315	-2.60617	-2.572	-2.45262
STEAP2	1.66727	1.88591	4.45038	4.88624	-1.70566	-2.70579
STEAP3	3.92183	4.2045	4.49665	5.23357	4.74906	5.00128
STEAP3-AS1	0.210813	0.0722019	-0.0913485	0.637675	-0.167112	0.107233
STIL	5.46863	5.50112	5.49991	5.67006	4.95439	5.05232
STIM1	4.22645	4.23847	4.64302	4.88057	3.80014	3.42257
STIM2	4.40288	4.53997	3.62543	3.60884	4.0218	4.09419
STIP1	7.08762	7.04733	6.46444	6.80913	6.71702	6.50963
STIP1P3	3.18048	3.28031	2.6393	3.1093	3.16163	2.50459
STK10	5.0757	4.78449	4.79517	4.98919	4.29678	3.79314
STK11	3.77106	3.63579	3.72446	3.90486	4.61427	4.46801
STK11IP	3.03902	2.9801	2.48892	2.48364	3.40712	2.97629
STK16	2.96134	2.82405	3.28728	3.68225	3.38873	3.69265
STK17A	5.78898	5.93611	5.24482	5.57679	6.69848	7.49955
STK17B	3.69966	3.80861	4.82979	5.22264	4.96127	5.25183
STK19	2.0771	1.86128	2.17885	2.27525	2.97323	2.76542
STK24	5.458	5.38789	5.5319	5.56094	5.26678	4.82348
STK25	5.20781	5.01867	4.96894	5.18893	5.26158	5.03953
STK26	-4.37417	-4.87366	-0.455418	-0.154483	4.17968	4.00237
STK3	3.64596	3.57469	3.72787	3.84348	4.65234	4.85553
STK32A	-0.744656	-0.604659	0.242852	0.414828	3.83753	4.13406
STK32B	4.42114	4.4957	3.04048	3.38503	-2.01588	-4.70296
STK32C	0.50068	0.645757	1.66826	1.95189	1.32273	1.13883
STK33	-5.10914	-5.45651	-6.17309	-5.10387	2.60179	3.04483
STK35	4.25946	4.05776	4.78514	4.85188	3.97631	3.37544
STK36	4.11207	3.88068	4.36234	4.49662	4.54057	4.02931
STK38	3.80673	3.85683	4.48269	4.7761	4.79244	4.90983
STK38L	3.87267	3.84549	2.79376	2.79807	5.09122	5.31634
STK39	4.02373	4.23499	3.4108	3.80706	4.11035	4.43411
STK4	6.4335	6.37175	5.85402	6.18518	6.55926	6.2727
STK40	4.06909	3.12024	4.14177	3.57592	4.76175	3.22993
STMN1	7.42374	7.49737	6.55307	6.98998	7.50294	7.97762
STMN3	5.17546	4.72265	5.19316	5.3074	0.0540566	-0.121607
STOM	4.79477	4.98112	4.37966	4.9038	4.97451	4.89411
STOML1	1.86319	1.64048	2.44585	2.6856	2.44561	2.90432

STOML2	4.81219	4.78269	3.95348	4.39994	5.99522	6.40053
STON1	3.68563	3.09478	3.22765	3.18162	3.97336	3.53203
STON1-GTF2A1L	-1.30408	-1.72029	-3.1534	-3.33829	1.7068	2.0623
STON2	-3.2383	-3.29152	1.82773	3.01271	4.49956	4.58906
STOX2	3.67921	3.43953	1.95605	2.52418	0.499649	0.785207
STPG1	2.35929	2.28552	-1.01182	-0.61561	1.78782	2.27117
STRA13	4.19422	4.02197	3.05965	3.52052	4.84052	5.28124
STRA6	4.83819	5.64863	5.93674	6.30865	2.6844	4.08643
STRADA	3.78403	3.65511	3.83371	3.83008	4.22935	3.94239
STRADB	3.61027	3.42545	2.6739	2.97552	3.8367	4.08372
STRAP	6.24833	6.18222	5.48487	5.82568	6.02308	6.29456
STRBP	4.26768	4.15222	2.95701	2.85581	4.94219	4.82482
STRIP1	4.28462	4.31769	4.64776	4.9505	4.48561	4.4732
STRIP2	3.31355	2.96552	2.87177	3.1673	1.96176	1.8479
STRN	5.34118	5.32628	4.66899	4.91493	5.44852	5.23872
STRN3	4.33846	4.37871	3.73831	4.0933	4.61174	4.6309
STRN4	4.51828	4.30422	4.12963	4.28571	5.31619	5.06652
STS	2.64078	2.31525	0.174138	0.300663	2.73844	2.89692
STT3A	6.67214	6.74965	7.12765	7.47507	6.66863	6.66586
STT3B	7.30475	7.35856	6.98652	7.1813	7.42861	7.69907
STUB1	3.78789	3.6795	3.51744	4.0031	4.7925	5.15493
STX10	3.67596	3.60324	3.15187	3.37901	4.20077	4.36875
STX12	3.92205	3.97802	3.76046	4.20858	4.85489	5.21735
STX16	5.74484	5.69629	5.70904	5.78238	5.56465	4.94517
STX16-NPEPL1	-0.057105	-0.831628	1.30186	0.0881959	0.791256	-2.42864
STX17	3.23129	3.33771	3.72813	3.85775	2.96083	3.26483
STX18	3.11746	3.201	3.25104	3.64041	3.51773	3.64506
STX18-AS1	-1.05967	-0.971063	0.747044	0.316609	0.405666	0.0480548
STX1A	2.65203	2.69657	1.89889	2.54686	3.01654	3.228
STX2	3.99614	4.02599	3.40893	3.59523	3.48336	3.2677
STX3	4.3996	4.37478	3.52557	4.10789	3.91144	4.04147
STX4	2.62736	2.79926	3.03565	3.41278	3.00503	2.88948
STX5	3.26237	3.23586	3.12709	3.52416	3.09124	3.11984
STX6	5.40552	5.52818	5.16558	5.42248	5.23391	5.19005
STX7	3.2168	3.13148	3.78802	3.91493	5.47185	5.37476
STX8	2.31216	2.30535	2.21883	2.70817	2.97994	3.5293
STXBP1	2.37077	2.53223	2.99137	3.23684	0.643551	1.11352
STXBP3	4.6857	5.1013	3.88928	4.44095	4.51897	5.15203
STXBP4	4.16123	4.30119	4.26045	4.46102	4.3141	4.34856
STXBP5	4.39814	4.46047	4.12778	4.40274	5.47401	5.43596
STXBP5L	2.31774	2.11082	1.46037	1.24877	0.469063	-0.536593
STXBP6	2.31774	1.59788	-2.01303	-2.30078	-0.00196773	-0.421131
STYK1	-0.865939	-0.707737	1.48185	2.01532	-1.95932	-0.421131
STYX	3.87078	3.6507	2.9939	3.09456	4.2614	4.00238
STYXL1	2.76241	2.78271	3.08794	3.49851	2.89812	3.17899
SUB1	5.50319	5.63702	5.75959	5.93314	6.65625	7.25024
SUB1P1	0.0856014	0.284182	0.263046	0.431099	1.02454	1.78006
SUB1P3	0.598079	1.02906	0.688395	1.13916	1.68853	2.33982
SUCLA2	4.00111	4.13741	4.12453	4.57313	4.25351	4.64619
SUCLA2P1	-0.111491	-0.113369	-0.00422896	0.448436	0.255742	0.570298
SUCLG1	4.66121	4.70457	4.51343	4.98723	4.7071	5.41482
SUCLG2	4.73812	4.80272	3.98596	4.1844	4.16069	4.5682

SUCLG2-AS1	0.45076	0.592522	-0.206091	0.0811214	-0.705893	-0.280287
SUCLG2P2	3.19886	3.30709	2.55578	2.62998	2.56843	3.13575
SUCNR1	-5.69162	-5.45651	2.05012	0.46112	?	?
SUCO	5.8149	6.16308	5.41731	5.88973	6.01604	6.61857
SUDS3	5.09651	5.2424	4.46861	4.73171	4.72452	4.73721
SUDS3P1	1.96659	2.18056	1.28239	1.70049	1.74439	1.85592
SUFU	2.39104	2.08021	2.46754	2.45724	2.21929	1.80111
SUGCT	1.15272	1.12967	0.120378	-0.0212303	-0.374735	-0.247125
SUGP1	3.36432	3.31842	2.9406	3.19489	3.84165	3.61295
SUGP2	5.92346	5.8577	6.1663	6.16546	6.86988	6.36641
SUGT1	5.05663	5.19134	4.87785	5.16204	5.0954	5.42577
SUGT1P2	1.08887	1.2583	0.935482	1.04098	1.16629	1.1637
SULF2	-0.0407343	-0.654524	5.63795	6.42955	5.19345	5.67194
SULT1A1	-6.65375	-6.43535	4.19047	4.43454	-1.06106	-0.328835
SULT1A3	0.805243	0.923537	1.12769	1.35235	0.97	-0.21166
SULT1A4	-0.475403	-0.847656	0.874144	1.07003	0.959065	1.0084
SUMF1	4.14326	3.91761	3.3203	3.46293	3.96539	4.23873
SUMF2	6.3721	6.30004	5.74291	5.92036	5.67149	5.83217
SUMO1	5.46235	5.52935	4.92953	5.23323	6.13299	6.55819
SUMO1P3	3.07437	3.08099	2.6768	2.81805	3.73297	4.33209
SUMO2	5.63691	5.69354	5.66977	6.03169	6.92844	7.03275
SUMO2P1	2.26056	2.21649	2.06024	2.52088	3.63025	3.73434
SUMO2P17	0.683337	0.709688	0.547154	0.787533	1.88575	2.30064
SUMO3	4.37703	4.27355	4.44674	4.86485	5.63	5.89691
SUMO4	-0.129081	0.151976	0.793829	0.520618	1.17894	0.878317
SUN1	5.62068	5.74635	6.27855	6.56202	5.5632	5.63572
SUN2	6.04592	6.00885	5.62972	6.03484	5.64151	5.67573
SUOX	3.45954	3.3637	3.21736	3.32163	3.36482	3.22993
SUPT16H	7.08682	7.22233	5.72852	6.18982	6.85151	7.03857
SUPT20H	4.55946	4.6229	4.75275	4.75299	4.58832	4.43614
SUPT3H	1.16515	1.01295	0.503064	1.04098	1.27048	1.34861
SUPT4H1	4.39424	4.48674	3.47084	4.09555	5.10455	5.72955
SUPT5H	5.21679	5.26846	5.05904	5.39698	5.60646	5.69577
SUPT6H	6.30989	6.30495	6.5234	6.77914	7.02057	6.62867
SUPT7L	5.1259	5.18731	4.28734	4.54037	5.23239	5.1142
SUPV3L1	3.87361	3.8214	3.28	3.61227	4.07778	4.0565
SURF1	2.1957	2.23414	2.55795	3.02565	3.09124	3.67697
SURF2	1.96272	2.17017	0.805235	1.35049	2.68621	3.11347
SURF4	7.02241	6.98491	7.13969	7.62897	6.9607	7.18169
SURF6	4.24205	4.01494	3.38361	3.52142	4.60789	4.21661
SUSD1	1.26675	1.12217	2.66823	2.77382	1.35096	1.06153
SUSD2	-5.10914	-3.87578	3.69758	4.33308	-4.33548	-2.8983
SUSD5	5.38072	5.34651	3.04289	3.38102	-1.07506	-1.4584
SUSD6	3.03394	3.1774	2.83608	3.0611	3.24808	3.10386
SUV39H1	3.14807	3.11954	2.15915	2.29017	2.52521	2.49787
SUV39H2	4.20379	4.27954	3.75387	3.91148	4.10363	4.27385
SUV420H1	4.6857	4.46862	4.41266	4.24762	4.17968	3.94971
SUV420H2	1.40949	0.996642	1.47473	1.31748	1.45867	0.951558
SUZ12	5.89793	5.99637	5.2745	5.40493	5.76642	5.65687
SUZ12P1	2.79791	2.62696	3.39715	3.27461	3.097	2.70281
SV2A	5.79678	5.74197	5.66271	5.88907	6.33477	6.316
SV2C	3.03054	2.82257	-4.59508	-3.52222	-6.64581	?

SVBP	1.34536	1.51447	1.96116	2.00494	2.18833	2.64202
SVEP1	-1.74441	-2.46206	3.70825	2.94646	-3.33694	-3.70484
SVIL	5.97931	5.94169	1.33552	1.3642	5.61099	5.40735
SVIL-AS1	4.06891	3.96781	3.19912	3.2047	3.77683	3.64454
SVIP	3.80858	4.03592	0.416483	0.810106	2.47925	2.74862
SWAP70	4.50487	4.48333	4.85675	5.19037	5.19574	5.1419
SWI5	2.23168	2.08792	2.25112	2.375	2.12107	2.34315
SWT1	2.07572	1.89473	1.69915	1.69856	1.8195	2.23582
SYAP1	5.1507	5.28198	4.91647	5.30532	5.24318	5.40701
SYBU	1.62401	1.73692	-0.861345	-0.0426024	0.384182	0.993783
SYCP3	1.42171	1.65122	1.10541	0.996483	0.168257	-0.0211732
SYDE1	3.11149	3.24624	2.693	3.34443	3.63888	3.92446
SYDE2	2.66068	2.52301	3.56131	3.40095	2.73202	2.63759
SYF2	3.77259	3.80043	2.69301	3.29206	4.24358	4.73935
SYMPK	4.86459	4.76175	4.54013	4.63263	5.01608	4.75884
SYNC	3.23303	3.56018	1.19349	2.04338	3.09766	3.96358
SYNCRIP	7.43248	7.49652	6.19887	6.40565	7.48277	7.81226
SYNE1	5.23367	5.17218	3.76921	4.16729	7.15791	7.17938
SYNE2	7.18695	7.052	5.85812	5.55115	6.93446	6.67293
SYNE3	4.44748	4.37004	3.14066	3.5476	0.679613	0.560157
SYNGAP1	2.63047	2.43065	2.40987	2.21656	2.60959	1.97513
SYNGR1	3.55476	2.99659	1.68069	1.195	2.45605	2.23582
SYNGR2	5.12793	5.16782	3.641	4.01223	5.34699	5.45483
SYNJ1	2.98216	3.14818	4.21093	4.35367	3.52084	3.62421
SYNJ2	5.43597	5.7239	6.21923	6.30502	6.45575	6.33801
SYNJ2BP	5.16652	5.27132	4.52367	4.78739	5.48245	5.52692
SYNJ2BP-COX16	1.19307	1.20449	-0.171705	-0.000819684	1.23896	2.25594
SYNM	7.32206	7.10499	3.40898	3.50127	5.92007	5.46893
SYNPO	2.89151	2.82487	4.535	4.75069	3.10787	3.67699
SYNPO2	0.278306	0.318612	-0.350462	0.399062	-0.320293	-0.121607
SYNRG	4.84878	4.90347	5.12423	5.19894	5.0019	5.06311
SYPL1	5.85979	5.88889	5.40095	5.63218	5.57482	5.68453
SYS1	4.94367	4.90724	4.51871	4.65032	4.3943	4.53636
SYT1	3.59325	4.23063	-0.161006	1.04098	3.13249	4.39526
SYT11	5.83594	6.50797	3.90923	4.75844	6.84709	7.60019
SYT14	4.29455	4.31933	1.78229	2.25751	-4.65668	-5.69922
SYT15	-0.432112	-0.122853	1.40897	0.89124	-0.105579	-1.24695
SYTL2	1.83622	1.60328	3.60274	4.83295	2.34252	2.67267
SYTL4	2.63185	2.69198	1.96625	2.32998	3.55158	3.22107
SYTL5	4.25365	4.07921	-0.428455	-0.464785	3.84452	4.61857
SYVN1	4.17397	4.25144	3.73598	3.87054	3.45107	3.38076
SZRD1	5.66669	5.52477	5.71658	5.93404	6.81451	6.72706
SZT2	5.11151	4.8859	5.47146	5.57734	4.83437	4.43942
TAB1	3.35892	3.025	3.21094	3.38102	2.69725	2.85171
TAB2	4.76237	4.81444	5.1271	5.27809	6.4748	6.7174
TAB3	4.20027	4.1013	3.96623	3.98129	4.09039	3.74449
TACC1	5.8615	6.19963	6.96573	7.596	6.36222	6.6492
TACC2	2.19576	2.17017	2.11571	2.23549	1.84751	1.94436
TACC3	5.16433	5.1592	4.89861	5.03837	5.77195	5.4831
TACO1	3.29527	3.1882	3.16521	3.45724	3.87204	4.25905
TACR1	0.490837	0.746683	-0.095919	-0.131402	2.1112	2.45821
TADA1	3.48455	3.57471	2.78227	2.77076	3.0384	3.05484

TADA2A	3.71454	3.7356	2.72938	2.95187	3.00681	3.35127
TADA2B	3.25221	3.15187	2.76777	2.94375	3.96265	3.64202
TADA3	4.71032	4.62467	3.93793	4.18205	5.41969	5.8899
TAF1	4.91804	4.80801	5.14726	5.09838	5.39558	5.0412
TAF10	4.94179	4.958	4.33736	4.76399	4.69441	4.77518
TAF11	3.8821	3.82947	3.38062	3.47425	4.7701	4.86881
TAF12	2.04577	2.32181	2.53073	2.94375	3.50961	4.23873
TAF13	4.162	4.33481	3.18499	3.99839	5.20425	5.36474
TAF15	5.13731	4.96813	5.02287	4.89117	5.28347	4.54358
TAF1A	2.41468	2.41981	1.90926	2.42646	2.51213	3.05151
TAF1B	3.95377	3.95394	2.61739	2.54224	3.85637	4.17592
TAF1C	3.4858	3.28367	3.8221	3.90798	3.04187	2.53647
TAF1D	6.16057	6.08324	6.33253	6.36873	6.51943	6.82333
TAF1L	1.57451	1.62968	2.07026	1.80206	2.05277	1.78779
TAF2	5.02629	5.04047	4.99388	5.26457	5.71607	5.856
TAF3	2.40159	2.16288	2.4164	2.18121	2.55887	1.80111
TAF4	3.2063	3.11459	2.83885	2.67255	2.45864	2.06149
TAF4B	3.51281	3.5496	1.36342	1.59165	0.688494	1.3157
TAF5	3.09534	3.2514	2.32429	2.73039	3.10124	3.12934
TAF5L	4.32675	4.2744	3.51865	3.67907	4.53505	4.2848
TAF6	4.09618	3.95156	4.06903	4.18348	3.7291	3.66427
TAF6L	2.50578	2.19385	1.69751	1.81791	2.06829	1.9967
TAF7	5.98127	6.01465	5.37823	5.59247	6.0386	6.49295
TAF8	3.44302	3.40907	3.31633	3.64773	3.88175	3.8305
TAF9	4.91977	4.95769	4.67351	5.27552	5.49128	6.08484
TAF9B	4.00212	4.24823	4.32491	4.56624	5.52665	5.18345
TAGLN2	6.94799	6.96185	7.0774	7.44012	7.55041	7.63414
TAGLN2P1	4.51156	4.56804	4.82862	5.03882	5.0155	5.10006
TALDO1	5.74281	5.75965	6.7435	7.34041	5.44624	5.98544
TAMM41	2.30656	2.29206	1.98643	1.71775	2.63202	2.45821
TANC1	6.87601	6.60826	6.76465	6.73981	6.39619	5.97495
TANC2	5.32777	5.41442	4.69682	4.69206	6.42979	6.37473
TANGO2	3.16226	2.86609	3.50792	3.76499	2.99075	2.58722
TANGO6	3.39632	3.55366	3.11082	3.40362	3.87579	4.10596
TANK	3.97599	4.09653	3.85127	4.13672	4.45017	4.97188
TAOK1	6.65529	6.62275	7.11899	7.24439	7.42334	7.28481
TAOK2	4.87882	4.64175	4.84782	4.9031	4.69722	4.47816
TAOK3	4.08064	3.98836	3.5444	3.89539	3.62046	3.79114
TAP1	4.86848	5.09434	5.0269	5.38042	5.1389	5.84871
TAP2	4.3195	4.51662	5.07976	5.50177	5.38119	5.3093
TAPBP	5.84173	5.83544	6.71053	6.69691	5.86128	5.89829
TAPBPL	2.90674	3.21795	3.71612	4.11873	1.42497	1.86742
TAPT1	2.86126	3.43651	2.81366	3.48176	2.83355	3.28481
TAPT1-AS1	2.37562	2.74081	1.99303	2.18475	2.47885	3.12537
TARBP1	4.22721	4.39823	4.669	4.64104	4.28	4.5245
TARBP2	3.78976	3.5938	3.10419	3.31954	3.03144	3.47568
TARDBP	6.49228	6.40117	6.22807	6.43903	6.78499	6.28353
TARID	0.0201857	0.400494	1.94774	1.25102	1.08121	1.29333
TARS	6.27211	6.42663	6.0001	6.56801	7.17897	7.6079
TARS2	4.14405	3.92947	3.163	3.59853	3.92934	3.82661
TARSL2	2.53934	2.48845	1.51	1.96266	1.1211	1.35942
TAS1R1	0.824548	1.57611	-3.37468	-3.30006	-3.6585	?

TAS2R13	0.987481	1.06859	1.65571	0.761609	0.395063	0.224139
TAS2R14	1.76799	1.79458	2.05638	1.91684	1.1211	0.522196
TAS2R15P	1.09832	1.22997	1.44901	1.12963	0.362161	0.231764
TAS2R18	1.32333	1.31199	1.69303	1.32585	0.634389	-0.183001
TAS2R19	1.75577	1.76368	2.23132	1.8497	0.790972	0.592024
TAS2R20	0.908313	1.15925	1.70525	1.31748	0.405873	-0.31423
TAS2R30	2.24446	2.23323	2.709	2.47862	1.66981	1.11912
TAS2R31	1.36749	1.51561	1.85035	1.65844	0.534858	0.436236
TAS2R4	0.399051	0.822624	-1.53926	-1.25052	1.35096	0.965773
TAS2R46	0.789963	0.852766	1.51646	1.20393	0.0882502	-0.266838
TAS2R50	0.243547	0.370142	0.860939	0.46112	-0.338214	-0.848487
TAS2R64P	1.12426	1.14986	0.855049	0.662215	1.76434	1.41149
TASP1	4.22052	3.85682	2.63985	2.60199	3.61115	3.68989
TATDN1	2.80715	3.06861	2.49889	2.8141	3.72252	4.52593
TATDN1P1	1.50762	1.96319	1.44957	1.71585	2.49405	3.17467
TATDN2	4.91947	4.90059	4.35515	4.48995	4.92544	4.86629
TATDN2P2	2.51768	2.50295	2.16964	2.30482	3.57809	2.90432
TATDN3	1.96983	2.20606	1.6494	2.23992	2.14706	3.03813
TAX1BP1	6.52887	6.68651	6.42514	6.87895	6.14066	6.87239
TAX1BP3	3.83431	3.57397	3.27261	3.90416	4.34897	4.69407
TAZ	2.97158	2.77795	2.40895	2.56007	2.84749	2.43285
TBC1D1	5.00178	4.78508	4.23643	4.5458	6.03687	5.95416
TBC1D10A	2.51594	2.33316	3.276	3.58769	1.94219	2.2246
TBC1D10B	3.75048	3.60177	3.80482	4.05425	4.03722	3.86332
TBC1D12	3.23166	3.20249	2.46035	2.72407	2.92278	2.75682
TBC1D13	3.38041	3.307	3.32823	3.46483	3.92745	3.53884
TBC1D14	4.98528	4.88866	4.94908	5.19946	5.39431	5.13984
TBC1D15	4.82202	4.86806	3.99514	4.19722	3.72986	4.10706
TBC1D16	5.87434	5.53078	6.15043	6.11905	5.19301	4.36072
TBC1D17	2.37973	2.19447	2.53725	2.75124	3.16113	3.17132
TBC1D19	1.62738	1.35098	1.6494	1.79207	1.19149	1.71958
TBC1D2	0.640852	0.775632	3.15633	4.00491	0.549226	1.37014
TBC1D20	4.2346	4.21575	5.11739	5.38351	4.2682	3.84211
TBC1D22A	2.8186	2.69955	3.68993	3.85839	2.68842	2.61973
TBC1D22B	2.7408	3.08924	2.35804	2.66405	3.27999	3.35171
TBC1D23	4.41075	4.48332	4.64222	4.99642	4.69833	5.21064
TBC1D24	3.14954	3.12653	3.26219	3.2661	3.38837	3.16507
TBC1D25	2.40159	2.44406	1.63669	1.90242	2.3397	1.77713
TBC1D2B	4.45764	4.45824	4.51085	4.75613	4.28147	4.52451
TBC1D30	3.43663	3.57057	-3.59683	-3.30006	2.17891	2.69844
TBC1D31	3.49936	3.57333	3.62222	3.82781	3.24958	3.85933
TBC1D32	2.5006	2.58701	2.47113	2.41475	3.31124	3.54358
TBC1D3L	0.709573	0.458309	1.84771	1.67621	1.52452	0.660363
TBC1D4	4.86314	5.37637	3.72636	3.8926	5.61114	4.95149
TBC1D5	5.32294	5.46677	4.62301	4.93965	5.4853	5.76292
TBC1D7	2.63855	2.41368	2.45673	2.6191	2.97813	3.19415
TBC1D8	4.78122	4.83404	4.9795	5.21928	3.94885	3.54358
TBC1D8B	2.93392	3.09255	2.98139	3.27919	1.29393	1.81687
TBC1D9	3.78776	3.98216	3.5478	3.88269	4.44494	4.73933
TBC1D9B	5.44395	5.47488	5.91218	6.08252	6.28241	6.14776
TBCA	5.11559	5.0737	4.89506	5.18585	5.83276	6.14016
TBCAP1	1.97261	2.14573	1.92155	2.23039	2.93587	2.76273

TBCB	4.03923	4.08573	3.43643	3.87411	4.69935	4.92223
TBCC	2.69325	2.8455	2.38635	2.73039	3.46381	3.64643
TBCCD1	2.9588	3.00397	3.3418	3.57357	3.81463	4.0926
TBCD	4.91264	4.85919	4.56126	4.67804	4.94497	4.4632
TBCE	3.6857	3.70457	3.58135	4.01788	3.99576	4.16211
TBCEL	3.87172	3.92624	4.23327	4.4245	4.00769	4.34856
TBCK	3.23964	3.28968	3.97673	4.02795	3.61094	3.8489
TBK1	4.90911	5.01839	4.44061	4.70887	5.01735	5.61126
TBKBP1	1.57708	1.00482	1.71737	1.17662	-0.152369	-1.38442
TBL1X	2.92115	2.51445	4.62543	4.57064	2.49452	2.14505
TBL1XR1	6.91401	6.88792	7.5804	7.59038	7.15983	6.97475
TBL2	4.48827	4.59717	4.26665	4.6676	4.34601	4.21661
TBL3	2.97809	2.6561	2.80517	3.0859	2.68843	2.89692
TBP	2.77868	2.87032	2.36339	2.72723	3.76867	3.71536
TBPL1	2.9672	3.02634	2.98732	3.1377	4.49256	4.85353
TBRG1	3.69639	3.64286	3.99361	4.13851	3.72733	3.68968
TBRG4	5.47397	5.35971	4.85333	5.14678	4.98939	5.18049
TBX15	0.727258	0.419895	2.01126	2.1296	-1.75295	-2.12114
TBX19	0.207918	0.382745	-0.897866	-1.46458	1.37865	0.615294
TBX2	3.81169	3.1108	0.544485	0.445854	1.28225	0.176042
TBX3	3.31075	3.44404	5.49154	5.61567	4.93493	4.7771
TBXAS1	2.87078	3.06659	4.86291	5.20178	0.384182	0.878317
TCAF1	6.17164	5.96593	6.39226	6.43175	5.63484	5.29345
TCAF1P1	2.7544	2.51563	2.86034	3.04865	1.81602	1.48084
TCAF2	2.93241	2.73332	0.583721	0.901131	3.19578	1.67383
TCAIM	3.64135	3.6709	2.98891	3.41277	4.16126	4.50967
TCEA1	5.36351	5.52565	5.05041	5.52377	5.30394	5.2999
TCEA1P2	4.90889	4.98451	4.55242	4.97721	4.69516	5.14548
TCEA2	2.10829	1.71709	2.7909	2.83955	2.34533	2.39659
TCEAL1	2.06734	2.48519	2.09742	2.89957	2.57975	3.53835
TCEAL3	2.85059	2.60841	2.71676	3.13193	4.11447	3.8289
TCEAL4	4.28818	4.32994	4.02837	4.36589	4.9663	4.73207
TCEAL8	3.99789	4.00781	3.41267	3.75381	4.13491	4.65521
TCEANC2	3.64188	3.50726	3.84714	3.87131	3.72339	3.59707
TCEB1	4.1171	4.11832	4.04649	4.43128	5.14497	5.47257
TCEB2	4.56989	4.34633	4.39624	4.92571	4.5403	5.27978
TCEB3	6.25136	6.34056	5.44278	5.78065	6.93684	6.94588
TCERG1	5.71796	5.85371	6.17868	6.22404	6.12289	5.71481
TCF12	5.91079	5.93186	4.54864	4.50311	6.15126	6.31425
TCF19	4.18585	4.36978	4.49506	4.9444	5.03406	4.99546
TCF20	5.87432	5.85426	5.69566	5.74096	5.22651	4.86408
TCF25	4.75879	4.42282	5.01919	5.07479	4.51584	4.42899
TCF3	4.25293	3.99144	3.87852	3.75845	4.82702	4.3322
TCF4	5.51067	5.42284	5.90857	5.6498	5.83853	5.69841
TCF7	3.30514	3.09175	4.21735	4.09451	2.26748	1.51249
TCF7L2	3.04578	2.94177	-3.59683	-3.78492	3.45879	3.03509
TCFL5	3.28107	3.40753	2.95084	3.19494	3.25851	3.79479
TCHP	3.95079	4.00489	2.71677	3.03991	3.21391	2.7777
TCIRG1	2.21571	1.86596	4.37065	4.664	3.11334	3.33447
TCN2	-0.207251	0.151846	2.4164	2.74916	2.95428	3.53168
TCOF1	6.08001	6.06901	5.64619	5.92562	6.06228	5.82417
TCP1	6.76661	6.78746	6.4275	6.87123	7.9236	8.05816

TCP11L1	4.0691	4.20714	2.58143	2.88482	3.68073	3.77997
TCP11L2	1.10753	1.41423	1.87648	2.62038	2.14274	2.71921
TCP1P1	2.16975	2.45502	1.85142	2.45883	3.67313	3.32808
TCTA	2.5817	2.47381	2.78227	3.20633	3.01212	3.07137
TCTE3	1.03569	1.21668	0.138526	0.535117	0.643551	0.816919
TCTEX1D2	0.529821	0.819088	1.34008	1.46864	0.688485	1.75183
TCTN1	3.55429	3.27526	3.89305	3.786	2.49687	2.16366
TCTN2	3.37167	3.0826	3.41739	3.36439	2.13302	1.75275
TCTN3	4.49751	4.35493	4.62866	4.93694	4.68009	4.99633
TDG	4.47768	4.85889	3.63745	4.08413	4.26373	5.04741
TDGP1	1.53475	2.03435	0.799593	1.32965	1.50981	2.22354
TDO2	-3.11289	-1.76193	3.12254	3.15796	-0.00196773	1.37014
TDP1	4.51826	4.37871	3.04048	3.17658	4.06846	3.96394
TDP2	3.3897	3.45303	3.81083	4.36386	4.37928	4.71218
TDRD3	3.41075	3.35734	2.79376	2.68884	3.35928	3.60843
TDRD7	2.66725	2.95079	3.18061	3.81599	3.14544	3.70268
TDRKH	3.45558	3.43057	2.65192	2.92351	1.60666	1.63847
TEAD1	6.80979	6.86316	6.78929	6.94744	7.10738	6.88365
TEAD2	2.14643	1.9252	4.11108	3.92459	1.4996	1.38079
TEAD3	1.76039	1.15192	2.18282	1.99449	0.427252	-0.848487
TEAD4	2.87079	2.66129	1.67449	2.23104	0.983605	1.20023
TECPR1	3.30092	2.94655	3.4108	3.26621	3.7909	3.36475
TECPR2	3.906	3.80954	2.72338	3.1273	3.56183	3.47493
TECR	4.0016	3.74005	3.86691	4.04711	4.82909	4.55057
TECRP1	3.48003	3.35355	3.4292	3.44965	4.23429	4.12412
TEF	2.30093	2.16288	3.23433	3.41082	2.83555	2.7609
TEFM	2.59535	2.74898	3.04039	3.11913	3.05236	3.6464
TEKT4P2	1.9005	1.77904	2.24165	2.23051	2.71903	2.19842
TELO2	1.8934	1.78036	2.16523	2.4799	1.82755	2.27058
TEN1	1.60748	1.46515	0.735228	1.43871	2.63987	3.21934
TEN1-CDK3	0.0467285	0.0320021	0.338133	0.233495	0.806002	0.123838
TENM1	3.43974	3.41191	-0.629682	-0.786664	-2.65941	-3.38329
TENM2	-0.159773	0.151976	0.544485	0.350701	7.06964	7.27056
TENM3	6.79656	6.549	6.20916	5.97143	6.65259	6.26509
TEP1	3.55358	3.56229	3.0235	3.16497	3.06421	2.97629
TERF1	3.62286	3.72809	4.14244	4.18172	3.92258	3.80597
TERF1P4	-0.224503	0.0307175	0.531129	0.364539	0.361095	0.197652
TERF1P5	0.461822	0.263034	0.630284	0.58166	0.239936	0.450686
TERF2	2.71032	2.66127	3.0308	3.31114	4.01564	3.92446
TERF2IP	3.98928	4.66755	3.54592	4.57411	3.95687	5.45577
TERT	1.28965	0.71714	-2.27595	-2.64851	0.373208	-0.4586
TESK1	1.63418	1.46793	2.02213	2.30987	2.94941	2.43796
TESK2	2.04291	1.85173	2.2437	2.27644	1.06183	0.937209
TET1	3.70179	3.40444	4.01123	3.59853	5.4964	4.48804
TET2	4.92435	4.8432	5.938	5.72879	5.76492	5.44745
TET3	5.08595	4.80335	4.71508	4.54759	5.09583	4.17746
TEX10	4.98565	4.95341	4.44582	4.43709	4.35996	4.61859
TEX15	-6.68418	?	-2.27595	-3.52222	2.56129	2.72792
TEX2	5.96135	6.07992	4.98951	5.17136	6.10848	6.23285
TEX261	5.73061	5.67009	5.05163	5.36899	5.55226	5.46996
TEX264	4.36433	4.33562	3.6729	3.95995	4.1503	4.50273
TEX30	2.16513	2.22367	2.24695	2.67255	2.35093	3.12618

TEX9	1.61879	1.79623	0.244936	-0.431676	1.62325	1.9399
TF	2.75214	0.859151	-3.37468	-3.30006	-5.07051	-5.69922
TFAM	5.40056	5.27267	4.85812	5.20404	5.95689	6.17131
TFAP2A	2.55746	2.48554	3.89309	3.7785	3.57056	3.07728
TFAP2C	5.58689	5.46121	3.03323	3.80257	4.42714	4.40311
TFAP4	2.79878	2.45901	1.57806	1.96801	1.76178	1.92993
TFB1M	3.10835	3.1468	2.82146	2.82282	3.61969	3.60606
TFB2M	3.64841	3.70081	2.19153	2.71773	3.92183	4.73311
TFCP2	3.42891	3.39515	3.26769	3.39698	3.40173	3.33221
TFDP1	4.98537	4.96534	4.97847	5.34001	5.96991	5.82536
TFDP2	3.68463	3.76357	4.62383	4.66265	4.4638	4.55772
TFE3	4.69267	4.57334	4.3361	4.6037	4.2682	3.97277
TFEB	-0.323873	-0.418273	1.11115	1.25754	1.58765	1.67702
TFG	5.49536	5.40443	5.65961	5.74876	5.72958	5.47629
TFIP11	4.55664	4.5631	3.82229	4.34832	4.15073	4.39812
TFPI	4.39618	4.74286	6.36713	6.69686	4.17406	4.82757
TFPI2	6.57192	6.89674	3.1541	3.42645	0.0812851	-0.421131
TFPT	1.79578	1.71326	1.65179	2.43313	2.22585	2.40824
TFRC	8.36761	8.60884	8.44456	9.01013	9.69083	10.052
TGDS	2.67814	2.61131	1.87179	1.98923	2.4031	2.92992
TGFA	8.92855	9.10536	-0.897866	-1.06415	7.40824	6.92901
TGFB1	3.23671	3.13439	3.93283	3.65946	3.73085	3.36634
TGFB1I1	2.32884	2.41981	2.81084	3.20859	1.62974	1.88577
TGFB2	1.47088	1.36995	7.11255	7.11619	5.62297	5.15186
TGFB3	3.02175	3.04815	-0.0134653	-0.301307	3.28025	3.5027
TGFBI	8.35073	8.29767	10.12	10.2669	10.4823	10.1137
TGFBR1	5.54438	5.45638	4.07268	4.37801	4.05479	3.88013
TGFBR2	5.41955	5.72202	5.76992	5.88375	5.63286	6.67994
TGFBR3	3.89711	3.10321	4.11568	4.16264	-1.26766	-2.00571
TGFBRAP1	4.48208	4.50797	3.4531	3.92183	4.81288	4.66615
TGIF1	4.34531	4.28702	4.01737	4.34957	4.40105	4.61633
TGIF2	5.00087	4.84336	3.9491	4.03072	4.44581	3.78156
TGIF2-C20orf24	2.63392	2.54013	2.39576	2.2073	2.77316	2.3814
TGM2	4.96533	5.44892	3.24064	3.06104	-4.27913	-5.69922
TGOLN2	7.52434	7.41276	7.1329	7.33845	7.24469	7.19183
TGS1	4.98043	4.88937	4.53076	4.68567	4.27534	4.41088
THADA	5.31798	5.35133	4.7097	4.7653	5.21348	5.37675
THAP1	2.64966	2.65091	1.95605	2.27057	2.72341	2.99719
THAP10	0.587384	0.896272	0.864771	1.19464	0.372272	0.697436
THAP11	3.98479	4.02298	3.60764	3.95995	4.68176	4.78513
THAP2	1.34591	1.42655	1.60803	1.80736	1.85965	1.92977
THAP3	2.3431	2.11446	2.35185	2.57952	2.92962	3.30026
THAP4	3.88964	3.86695	3.60274	4.00491	4.41983	4.52089
THAP5	4.95025	5.1853	4.2848	4.6183	4.83473	5.03782
THAP6	2.759	2.79219	2.87492	3.02344	3.83583	3.94393
THAP7	3.93302	3.68905	2.9406	3.17658	2.64804	3.14816
THAP7-AS1	0.721075	0.64284	0.317675	0.149793	-0.428453	-0.105923
THAP8	-0.432112	-0.39658	0.503064	0.711424	0.983605	1.1007
THAP9	1.66805	1.44175	1.98417	1.73773	1.48992	1.52214
THAP9-AS1	3.52621	3.67324	3.99666	4.31801	4.39648	4.63205
THBS1	8.82173	9.17637	9.59822	9.92267	9.43202	9.12111
THBS2	10.7829	10.2727	-0.375995	-0.327299	6.40005	6.21402

THBS3	2.39082	2.16542	2.62146	2.54124	4.91301	4.58843
THEM4	3.99939	4.14111	2.82129	3.06194	1.53651	1.94872
THEM6	3.85071	3.67924	3.55288	3.64105	0.519683	1.03481
THEMIS2	0.930336	0.90354	1.02599	1.24877	0.799187	0.247587
THG1L	2.40946	2.2096	2.02595	2.16265	3.49577	3.07137
THNSL1	2.66506	2.69451	1.91453	2.17196	1.80324	2.2763
THOC1	3.9589	4.01697	4.11561	4.0994	4.5302	4.43539
THOC2	6.39984	6.40676	6.51602	6.6415	7.01911	6.87864
THOC3	3.50337	3.57661	4.17521	4.71032	3.84105	4.24199
THOC5	5.69616	5.61329	4.49419	4.70093	4.99923	4.8969
THOC6	2.53365	2.32352	1.88669	2.03923	1.84966	2.06958
THOC7	3.85742	4.02097	3.55795	3.93008	3.66162	4.44175
THOP1	4.41725	4.15737	3.9776	4.17773	4.67563	4.84787
THRA	3.24776	2.90544	4.69186	4.77861	4.73709	4.10605
THRAP3	6.8244	6.91082	6.60416	6.79261	7.18319	7.13688
THRAP3P1	0.937608	1.25223	0.782627	1.0909	1.48481	0.923271
THRB	1.75216	1.28539	3.19369	3.44959	3.89331	3.57638
THSD1	0.897937	1.60232	0.200567	1.19043	0.116924	0.920766
THSD4	4.09013	4.21046	-0.825729	-0.380732	2.16943	2.05486
THTPA	2.12329	2.18039	1.34461	1.19065	2.08838	2.20796
THUMPD1	5.00178	5.02147	4.8016	4.96798	5.03274	5.17973
THUMPD2	2.46584	2.42287	1.7995	1.99972	2.87497	3.29047
THUMPD3	5.08665	5.13064	4.28625	4.67209	5.11271	5.36898
THUMPD3-AS1	4.0242	4.12855	4.11436	4.24884	4.27143	4.08876
THY1	-0.651253	-0.413436	6.86341	7.08154	5.99471	6.23519
THYN1	3.13855	3.12775	3.0453	3.47614	3.5196	4.05317
TIA1	5.30444	5.21486	5.95952	5.78751	5.71527	5.42514
TIAF1	1.49022	1.16691	3.08413	3.13062	2.09623	1.36743
TIAL1	5.07074	5.10177	5.28357	5.14914	5.68896	5.46069
TIAM1	4.42571	4.79867	3.20665	3.36488	3.49197	3.6813
TIAM2	4.01848	3.76079	3.15513	3.28014	2.75606	2.30963
TICAM1	1.4199	0.955067	0.156448	0.82198	2.04015	2.01441
TICAM2	-0.546825	-0.394117	0.942631	0.91773	1.83571	1.56987
TICRR	5.50096	5.22594	5.52535	5.37977	4.57599	3.5673
TIFA	3.98987	4.18184	2.62384	3.046	3.21904	3.18852
TIGAR	4.14011	4.21398	2.2002	2.65273	2.42185	2.74038
TIGD1	2.77861	2.80009	2.67981	2.66055	2.0979	1.77336
TIGD2	3.34803	3.52298	3.55961	3.77687	1.92843	2.24167
TIGD5	2.30375	2.24453	2.25112	2.5059	2.10458	2.31567
TIGD6	1.73377	1.60874	2.38617	2.70805	2.11385	2.18124
TIGD7	2.61777	2.57609	1.81377	2.02464	2.15296	1.77954
TIMELESS	4.67648	4.68371	4.62625	4.90099	4.69283	4.5245
TIMM10	3.27534	3.11458	2.76193	3.33203	4.19687	4.73309
TIMM10B	4.58033	4.60795	4.1501	4.32976	4.30473	4.46445
TIMM13	3.90172	3.57196	2.94576	3.33618	4.59695	4.82661
TIMM17A	5.48699	5.51977	4.87583	5.3855	5.62625	6.22769
TIMM17B	3.25802	3.16469	3.27591	3.46851	3.27922	3.55537
TIMM21	3.8284	3.86583	3.31831	3.5968	2.75121	2.52692
TIMM22	3.24578	3.13875	3.38055	3.65544	3.84257	3.86896
TIMM23	4.69331	4.77835	4.03775	4.64848	5.31241	5.78008
TIMM23B	2.92206	2.84238	2.3394	2.43757	3.12906	2.84703
TIMM44	3.54171	3.24452	3.02322	3.27055	4.29671	4.25329

TIMM50	4.5524	4.59987	4.16962	4.58552	4.86373	5.09902
TIMM8A	2.95018	2.92302	2.24695	2.44961	3.65548	4.3329
TIMM8B	2.19734	2.28662	3.47292	3.76943	4.74566	5.68979
TIMM9	2.61324	2.57732	1.6023	1.64055	2.75563	3.15438
TIMMDC1	4.50822	4.41392	4.41485	4.73716	4.63638	5.0202
TIMP1	6.68859	6.69478	5.03624	5.30002	4.07271	4.42513
TIMP2	7.52805	7.2763	7.18817	7.09856	7.51651	7.60549
TIMP3	9.49528	9.27848	8.66406	9.12343	1.7406	1.58794
TIMP4	-6.68418 ?		3.19802	3.41082	1.22848	2.32121
TINAGL1	4.54408	5.01388	2.98139	3.60026	-1.57243	-0.577229
TINF2	3.42633	3.58972	2.79662	3.2176	3.88078	4.21661
TIPARP	5.80098	5.92459	5.84505	6.11729	3.74907	4.0515
TIPIN	2.44998	2.51038	2.12357	2.61082	2.62215	3.1289
TIPRL	5.38772	5.5033	4.49683	4.7738	5.51709	6.1675
TIRAP	2.56021	2.45611	2.22875	2.40645	2.36561	2.14457
TJAP1	3.36835	3.26848	3.19369	3.31744	3.58048	3.18507
TJP1	5.88188	5.88839	5.5974	5.94543	5.1414	4.89783
TJP2	5.09168	4.87087	3.70825	4.11155	3.33264	2.98329
TK1	5.27496	5.11458	5.31981	5.80779	5.46284	5.58905
TK2	2.49704	2.81051	2.84406	3.19276	3.15409	3.98802
TKFC	2.97752	2.93759	2.9708	3.09163	2.06149	2.25596
TKT	8.078	7.82318	8.32274	8.65687	9.94315	10.1846
TLCD1	1.88215	2.22717	0.503064	0.999755	2.30837	3.18811
TLCD2	0.847885	0.603321	3.05965	3.32372	1.53448	0.33778
TLDC1	4.08056	4.25368	4.45302	4.88465	3.31828	3.28481
TLDC2	-0.0570314	-0.444922	0.395699	0.32067	0.540434	-0.696653
TLE1	3.58837	3.38134	1.75127	1.26626	3.64067	3.46322
TLE1P1	0.703747	0.442121	-1.35947	-1.58485	0.553596	0.176042
TLE3	2.94117	2.6483	2.77068	2.83662	4.36344	3.8726
TLE4	4.75521	4.92355	5.28661	4.96191	2.84348	3.07137
TLK1	4.86115	4.80775	4.62785	4.54113	4.9071	5.09649
TLK2	4.55434	4.50156	4.19923	4.41681	4.83537	4.88567
TLK2P1	4.13015	4.1697	3.89895	4.08584	4.67261	4.54745
TLL1	-5.69162	-3.13966	2.69913	2.1152	-0.393347	0.270672
TLN1	6.80921	6.75828	7.0659	7.27527	8.30851	8.28252
TLN2	4.65179	4.88462	3.4108	3.44558	5.59627	4.98415
TLR1	-0.0220622	-0.436203	0.398701	-0.119261	1.24788	1.64876
TLR3	1.32887	1.43806	2.68376	3.40293	2.02271	2.78114
TLR4	4.72979	5.19981	5.26769	5.68477	5.8676	6.41287
TLR6	3.20884	3.16981	2.59189	2.65761	3.63865	3.48617
TLR7	2.25804	1.554	-6.17309	-5.10387	-4.65668	-4.11926
TM2D1	3.42762	3.4782	3.65725	3.82043	3.94979	4.16826
TM2D2	4.08228	3.98939	4.49331	4.69044	3.90953	4.14815
TM2D3	4.34258	4.3928	3.79089	3.86988	4.45669	4.50639
TM4SF1	4.48577	4.78924	6.97145	7.63859	5.10207	5.46944
TM4SF19	-0.908264	-0.805694	0.44687	1.18069	1.86521	2.188
TM7SF2	1.10187	0.885949	0.558032	0.749225	3.27633	3.57869
TM7SF3	5.24528	5.45899	4.52037	5.00165	4.80728	5.41611
TM9SF1	4.68295	4.61088	3.47606	3.70933	4.48648	4.56541
TM9SF2	6.33948	6.39434	6.73216	7.14752	6.67814	7.05586
TM9SF3	7.20569	7.33752	7.17759	7.39946	7.58195	7.66423
TM9SF4	6.10135	5.99529	6.04826	6.24483	5.86793	5.50211

TMA16	3.6275	3.83522	3.00877	3.25093	3.16968	3.51125
TMA16P2	0.518535	0.74491	0.0631578	0.176693	-0.141255	0.113434
TMA7	5.0428	5.02269	3.78051	3.92095	5.42808	5.92604
TMBIM1	4.79728	4.72264	5.32574	5.75111	4.77898	4.85838
TMBIM4	4.42569	4.57469	3.71584	3.98128	3.18831	4.51848
TMBIM6	8.3654	8.50147	8.41458	8.89802	8.17401	8.3157
TMC7	1.53298	1.59788	-0.995207	-0.860651	1.24663	0.702755
TMCC1	2.92846	2.63519	3.22587	2.95457	5.14299	4.61747
TMCC1-AS1	1.30659	1.49709	1.64927	1.82194	0.458435	0.560157
TMCC2	0.701877	0.676782	0.747284	1.60545	2.1852	2.44812
TMCO1	6.29212	6.33793	5.5153	5.83274	6.50245	6.91013
TMCO3	4.81416	4.89579	5.0308	5.47236	4.89044	5.27556
TMCO4	1.85554	1.74664	2.87718	3.53323	1.79093	2.75682
TMCO6	2.63632	2.43803	2.0549	2.10551	0.615896	0.702755
TMED1	3.24787	3.1848	3.06805	3.37383	4.10888	4.90497
TMED10	7.48143	7.56959	6.39975	6.83951	7.10065	7.15668
TMED10P1	1.1559	1.1081	-0.556039	-0.285012	-0.0965714	-1.09281
TMED2	7.23869	7.27133	6.31991	6.80131	6.87673	7.17506
TMED3	6.11886	6.05903	5.41826	5.88692	6.14019	6.88834
TMED4	6.09803	6.20418	5.94507	6.3835	6.00499	6.07137
TMED5	6.21125	6.06364	6.09254	6.1926	6.41793	6.11791
TMED7	6.73033	7.00215	6.72559	7.30717	6.82259	7.15317
TMED7-TICAM2	2.6431	2.61101	2.53113	3.0352	4.22984	4.13058
TMED8	5.48299	5.3074	4.04889	4.30902	5.18439	4.93352
TMED9	5.8507	5.78893	6.42266	6.76402	6.19359	6.51847
TMEM100	-0.891472	-1.0363	4.09375	3.65438	-5.65305	-5.69922
TMEM101	3.54171	3.45303	4.33314	4.64521	4.4983	4.66614
TMEM102	0.219896	0.525929	0.208904	0.445854	0.384182	0.359476
TMEM104	3.36161	3.10511	3.89462	3.98525	4.77381	4.46696
TMEM106A	1.48583	1.25145	2.66824	2.65273	1.53448	1.0747
TMEM106B	5.38273	5.36052	5.77429	5.82633	5.22309	5.23726
TMEM106C	6.32863	6.14701	5.09898	5.53592	5.50866	5.95551
TMEM107	2.71859	2.42803	3.63983	3.64481	2.4343	2.38795
TMEM109	5.61092	5.65741	5.34981	5.71172	4.59885	4.26339
TMEM11	2.65342	2.47674	2.87988	3.13535	3.41298	3.69249
TMEM110	3.96197	3.96045	3.72278	3.98924	4.48545	4.3142
TMEM110-MUSTN	1.32576	1.12909	1.30169	0.996063	1.28914	0.591402
TMEM115	4.95547	4.75877	4.19077	4.33113	5.06429	5.11895
TMEM116	2.49076	2.63783	2.93283	3.04346	0.83161	0.893261
TMEM117	2.48084	2.32833	-2.48229	-2.40763	0.351018	0.832517
TMEM120A	2.33711	2.12589	2.50649	2.96264	2.73844	3.19717
TMEM120B	4.28087	4.04635	3.56106	3.66094	4.34842	3.81685
TMEM123	5.46678	5.83667	7.25182	7.60831	6.30903	6.68159
TMEM126A	1.91565	1.766	2.7909	3.36488	2.66163	3.78514
TMEM126B	2.50795	2.66645	3.43303	4.04472	3.35928	4.2961
TMEM127	5.21529	5.21838	4.89261	5.18751	5.65624	5.68398
TMEM128	1.83233	1.8272	1.97132	2.31296	2.72557	3.01439
TMEM129	2.97158	2.8881	3.01861	3.25093	4.54974	4.91441
TMEM130	-5.69162	-5.45651	3.24902	2.87702	-4.07293	-4.70296
TMEM131	5.67079	5.57675	5.33856	5.38902	5.21237	4.99718
TMEM132A	6.03942	5.82524	5.56405	5.84513	3.47357	3.35817
TMEM132B	-5.69162	-6.45019	2.77938	2.73353	-5.65305	-4.11926

TMEM133	-1.48924	-1.41808	2.05967	2.13437	-3.85089	-3.38329
TMEM134	0.923035	1.08411	1.3479	1.38307	0.766027	1.02127
TMEM135	3.61493	3.5301	4.05846	4.12957	4.3537	4.90338
TMEM136	2.73138	2.91437	2.73833	3.04599	2.55402	2.7609
TMEM138	3.78736	3.98716	3.55735	4.0405	3.60418	3.99988
TMEM140	3.08528	3.04416	3.40927	3.38211	2.99063	2.91124
TMEM141	2.14403	2.04893	2.43001	2.7618	1.82187	3.18403
TMEM143	-0.0550876	-0.508457	0.914572	0.711424	0.0946955	-0.280287
TMEM144	1.19579	1.59247	-6.17309	-6.09892	2.78678	2.50275
TMEM147	4.21381	4.23238	3.75605	4.30585	4.61334	5.12062
TMEM147-AS1	2.77463	2.86808	3.08787	3.24432	2.73038	2.39133
TMEM14A	2.74594	3.12587	2.68993	3.23547	3.31123	4.41998
TMEM14B	4.4082	4.53615	4.53259	5.06158	4.88453	5.99852
TMEM14C	4.63295	4.80158	4.96303	5.54939	4.71906	5.85887
TMEM14D	0.775952	1.10272	0.528481	1.54388	1.14778	2.04648
TMEM14E	-0.840854	-0.333394	1.12941	0.935626	-0.0454657	-0.95156
TMEM150A	1.23171	1.18826	1.13846	1.40691	0.954457	0.736302
TMEM154	2.55832	3.30864	1.84441	2.99447	3.91712	5.19716
TMEM158	3.69001	4.18461	2.86633	3.80555	0.416602	1.55071
TMEM159	0.0824028	0.331671	0.636738	1.10068	-1.20056	-0.799586
TMEM161A	2.77869	2.282	2.82491	2.75537	3.51711	3.22107
TMEM161B	4.44547	4.50437	5.1511	5.14433	5.83644	6.03077
TMEM161B-AS1	2.14948	2.46495	2.94576	2.83363	4.18668	4.39785
TMEM161BP1	-0.912225	-0.354151	-0.316277	0.0846902	0.835067	1.1812
TMEM164	4.20103	4.18822	5.72561	5.92626	4.77011	4.61069
TMEM165	5.33778	5.37954	4.8547	5.19094	6.58093	7.06622
TMEM167A	5.70608	5.61888	6.28193	6.43461	7.03595	6.81691
TMEM167B	4.05013	4.16865	4.56857	4.9823	4.33575	4.76382
TMEM168	4.26091	4.27693	3.10419	3.09085	4.47411	4.65303
TMEM169	1.43024	1.70711	0.156448	0.383121	-0.23389	-0.31423
TMEM17	1.17749	1.13712	0.793829	0.902467	0.270529	0.752791
TMEM170A	4.14328	4.21749	3.87175	4.06702	3.2822	3.1325
TMEM170B	4.27174	4.30534	1.31637	1.07114	4.27337	3.78913
TMEM171	2.70821	2.77317	0.571454	1.4226	-2.95876	-3.12051
TMEM173	-2.69815	-3.13966	3.63025	4.32579	2.04015	2.10066
TMEM175	1.42508	1.15192	1.83331	2.08102	2.50461	2.31011
TMEM177	3.32744	3.37636	2.03082	2.25313	2.47925	2.54597
TMEM178B	2.88025	2.33806	-1.93506	-1.52346	-2.75246	-2.8983
TMEM179B	4.24084	4.33605	3.41447	3.92187	4.09878	4.24937
TMEM18	3.59337	3.43809	2.52648	2.74448	3.19188	3.44179
TMEM180	1.38311	0.996642	0.893354	1.32585	1.35096	1.65087
TMEM181	5.47895	5.51084	5.17512	5.35826	5.85267	5.5624
TMEM182	1.05586	1.27191	1.51	1.92462	1.01923	1.28202
TMEM183A	4.83071	4.89264	4.07373	4.42355	4.70002	5.29957
TMEM184B	4.76801	4.6424	4.78874	4.97531	4.45147	4.07301
TMEM184C	4.51706	4.54628	4.34493	4.66349	4.91947	4.81489
TMEM185A	3.6626	3.59939	3.77771	3.89587	3.84939	3.67349
TMEM185B	3.79076	3.71831	1.84993	1.79809	3.13086	2.85935
TMEM186	2.19229	2.27297	1.53076	2.02562	2.09793	2.36476
TMEM187	0.146525	0.382745	1.12031	1.10068	1.13416	1.57873
TMEM189	5.08125	5.16557	4.70379	5.30125	5.42142	5.33312
TMEM189-UBE2V1	2.19922	1.8217	1.43337	1.99483	2.45298	1.6764

TMEM19	4.18164	4.39455	3.80132	4.37095	3.5982	4.46774
TMEM192	3.72403	3.65739	4.19043	4.60111	3.99252	4.26769
TMEM198	1.54089	0.902992	1.27216	0.946514	-0.115923	-0.706491
TMEM198B	1.85007	1.54747	2.1034	2.07208	0.230953	0.444741
TMEM199	3.90769	4.00291	3.60266	4.07805	4.20603	4.59447
TMEM2	8.2225	8.26645	6.53112	6.54532	7.8525	7.26985
TMEM200C	?	?	-1.18321	-0.380732	1.13416	1.54125
TMEM201	3.33984	3.30864	2.93802	3.05105	3.53198	3.39131
TMEM203	3.7807	3.68436	3.20234	3.51139	3.79912	4.11665
TMEM205	3.43663	3.16835	3.42047	3.77654	2.91922	3.52803
TMEM206	3.95913	3.95817	3.26356	3.4515	3.76595	3.95328
TMEM208	3.66396	3.75994	3.58796	4.23769	4.06164	4.77809
TMEM209	5.06826	4.87187	4.56878	4.74508	5.37554	4.71952
TMEM214	4.43662	4.40211	4.40987	4.58378	5.08284	4.93623
TMEM218	3.24929	3.10891	3.35951	3.37498	2.62972	2.74038
TMEM219	3.60013	3.6113	3.41266	3.76462	3.55401	4.00236
TMEM222	4.03648	4.01592	3.53587	3.901	4.52207	4.51728
TMEM223	3.55421	3.6541	2.84999	3.14378	3.84049	4.44137
TMEM229B	2.26961	1.88148	1.53761	1.90243	-3.19956	-3.38329
TMEM230	5.281	5.17885	5.66749	5.92971	6.1685	6.66705
TMEM231	2.80972	2.90776	2.89322	3.08874	1.2586	1.35942
TMEM234	2.72574	2.77339	2.73638	2.93953	3.07111	2.98258
TMEM237	4.14716	4.31022	3.83215	4.14756	3.39028	3.86444
TMEM241	2.30937	2.25827	2.01126	2.24434	1.44301	1.02127
TMEM242	2.55596	2.97595	1.61741	1.92462	2.74058	3.11666
TMEM243	3.54775	3.60095	3.78268	4.05728	3.41549	3.52715
TMEM245	6.066	6.37655	6.23114	6.632	5.94654	6.36005
TMEM246	2.88964	2.93591	2.8305	3.20859	-5.65305	-5.69922
TMEM248	5.98061	6.01565	5.93348	6.20432	6.31692	6.20316
TMEM25	-1.94365	-1.65505	1.63669	1.67911	1.62049	1.67702
TMEM251	2.38732	2.35407	1.05494	0.773887	3.16068	3.24749
TMEM254	1.27825	1.09944	0.150482	0.195058	0.706084	0.68568
TMEM255A	2.18358	2.53435	1.7234	1.80409	0.373208	0.224139
TMEM256	2.12112	2.33155	2.73141	3.01269	2.09048	3.10383
TMEM256-PLSCR3	1.50063	1.60871	2.37995	2.81304	2.4091	2.39664
TMEM258	4.41336	4.35176	3.48827	4.01531	3.81077	4.41073
TMEM259	4.31144	4.16011	4.63185	4.70575	5.13813	4.81095
TMEM260	3.3302	3.22015	2.4747	2.53864	2.37862	2.55539
TMEM261	3.73034	3.57471	2.75312	2.92735	4.80423	5.87071
TMEM262	1.84957	1.72962	1.45565	1.56739	1.09564	0.381605
TMEM263	5.21801	5.33816	4.88928	5.28605	4.9785	5.01111
TMEM30A	7.85318	8.10741	7.08115	7.37702	7.71244	7.83787
TMEM33	6.23241	6.31336	5.60111	5.99607	7.07781	6.76648
TMEM37	-4.11163	?	2.29627	2.43422	-3.85089	-5.69922
TMEM38A	2.49569	2.47086	1.43123	1.39901	1.08121	1.33772
TMEM38B	3.52371	3.78507	3.36338	3.74759	3.4612	4.02799
TMEM39A	4.00049	4.14909	4.41172	4.80105	3.94886	4.36474
TMEM39B	2.21383	2.01291	2.58136	2.52781	2.42716	2.05486
TMEM41A	3.76852	3.76469	3.78247	3.88473	4.58707	4.27004
TMEM41B	4.93935	4.83176	5.55203	5.56536	5.2604	4.80701
TMEM42	2.15581	2.20606	1.90926	1.83957	2.23755	2.78515
TMEM43	6.25202	6.55553	5.39296	5.96631	5.42381	6.09374

TMEM44	1.98744	2.0956	3.64238	3.65355	2.76387	2.50029
TMEM44-AS1	0.520176	0.370142	1.12031	1.41867	1.03324	1.53655
TMEM45A	1.4458	0.832056	2.56143	2.89118	4.65685	3.31592
TMEM47	5.09329	4.38887	1.51695	2.0561	1.7191	1.41227
TMEM5	3.46834	3.60996	2.28817	2.71136	2.19458	2.9515
TMEM50A	5.19185	5.28571	4.74547	5.0864	5.31556	5.48839
TMEM50B	3.21125	3.08086	3.8088	4.09664	2.96536	3.18203
TMEM51	2.15269	2.06466	2.55795	2.63265	1.50463	1.46325
TMEM52B	-6.68418 ?		4.57552	4.66842	-6.64581 ?	
TMEM53	1.90708	1.79192	2.06045	2.01342	1.91844	1.97873
TMEM54	2.08453	2.17184	2.33427	2.83599	0.799543	1.93841
TMEM55A	1.8664	2.06325	3.06885	3.46608	2.71599	2.76858
TMEM55B	3.457	3.28199	2.23012	2.54224	3.14866	3.54596
TMEM56	4.00746	4.25206	3.77054	4.06476	4.16347	3.6927
TMEM56-RWDD3	1.04432	1.11567	1.29536	1.13007	0.499976	0.862868
TMEM57	4.21005	4.22277	4.36337	4.47988	4.23981	3.95506
TMEM59	6.35754	6.29786	6.57342	6.9591	6.62234	7.17762
TMEM60	2.82645	2.79691	3.00631	3.35266	2.91145	3.3781
TMEM62	2.83502	2.82117	2.54406	3.08614	3.3592	4.14758
TMEM63A	4.25074	4.21663	4.6853	4.66513	4.39089	3.70692
TMEM63B	3.84973	3.6945	3.34785	3.79656	4.38475	4.0398
TMEM64	4.37708	4.23498	5.32969	5.14559	5.33089	4.89226
TMEM65	3.60013	3.59108	4.73533	4.9294	4.72122	3.71745
TMEM67	2.82449	2.57883	3.31632	3.55117	3.49451	3.48804
TMEM68	3.9626	3.55494	3.02825	2.71739	2.71107	2.28211
TMEM69	4.18654	4.08784	3.60572	3.89372	3.95713	3.80081
TMEM70	2.81012	2.88987	3.11339	3.54402	3.76818	4.28186
TMEM75	2.28108	2.20959	2.00138	1.99448	1.5829	0.80115
TMEM79	1.7099	1.50298	2.02787	2.41845	1.22848	1.0747
TMEM80	1.98744	1.91222	3.15855	2.87986	2.45084	1.95865
TMEM81	0.81669	0.570657	0.64945	0.399062	0.499649	0.224139
TMEM86A	0.460879	0.060836	0.474784	0.924662	-1.65987	-1.38442
TMEM87A	5.25992	5.04489	4.91619	5.16848	5.83145	6.07785
TMEM87B	4.2522	4.66449	4.54098	5.1067	4.43244	5.11026
TMEM8A	2.90458	2.66128	4.05368	3.97065	4.14784	4.00064
TMEM8B	0.255223	-0.0876659	0.699196	0.591699	-0.43131	-0.706491
TMEM9	5.02158	4.62725	4.85743	4.91491	4.55276	4.63868
TMEM97	5.31796	5.32995	5.10325	5.22475	4.81445	4.72996
TMEM98	4.22943	4.14356	-1.59814	-1.40802	-6.64581	-5.69922
TMEM99	2.09535	2.02501	2.33613	2.56007	3.09624	3.14504
TMEM9B	3.39234	3.72817	3.67134	4.20405	4.12329	4.83727
TMF1	5.58266	5.65204	5.3716	5.64151	5.65997	5.94538
TMLHE	2.91682	2.8654	3.03518	2.89848	3.2624	3.62525
TMOD1	1.53871	1.59029	2.26342	2.63527	-1.64073	0.0904063
TMOD2	4.20479	4.53926	3.42195	4.0023	2.36207	3.5003
TMOD3	5.4576	5.64032	5.29016	5.66509	5.81714	5.8786
TMPO	7.20827	7.46307	6.40494	6.69402	6.23991	6.34414
TMPO-AS1	1.68424	1.29754	1.38521	1.35278	0.513319	0.292794
TMPOP2	1.17648	1.4568	0.373008	0.750092	0.147163	0.0758886
TMPPE	3.79177	3.47086	2.72338	2.46861	3.12641	2.59251
TMPRSS15	3.079	2.93162 ?	?		-1.90489	-1.18284
TMPRSS5	-1.20691	-1.2146	-2.37543	-2.20128	1.06767	1.43288

TMSB10	7.38338	7.65954	8.04609	8.65088	7.99894	8.60636
TMSB15B	1.80391	1.77364	2.28692	2.34484	1.2021	1.64739
TMSB4X	4.28446	4.26115	7.87297	7.95925	7.75411	8.62168
TMSB4XP2	-1.18099	-1.49126	-0.214083	-0.0562692	1.75452	3.18225
TMSB4XP4	-1.53729	-1.2173	0.842987	1.05611	1.01634	1.46405
TMSB4XP6	0.389192	0.856261	4.27171	4.08981	4.15939	4.75523
TMTC1	3.65075	3.22541	4.1214	4.1731	2.80322	2.65085
TMTC2	5.80324	6.47062	-0.568612	-1.06415	1.46385	1.6942
TMTC3	5.60611	5.99143	5.70557	6.00001	5.5473	6.47968
TMTC4	3.65517	3.98423	1.85542	1.96266	3.4754	3.83244
TMUB1	2.91011	2.60325	2.4164	2.68885	3.53445	3.51971
TMUB2	3.54647	3.46493	4.25214	4.52143	4.41115	4.5269
TMX1	6.05346	6.10649	5.25266	5.46953	6.64306	6.67559
TMX2	5.5288	5.58709	5.61705	6.21399	6.10278	6.39107
TMX2P1	3.0539	3.19676	3.15613	3.62961	4.11402	3.91561
TMX3	6.36412	6.55447	6.17978	6.43856	5.64888	5.71271
TMX4	6.15578	6.25974	5.76338	6.27621	5.84772	5.86313
TNC	10.1761	10.0723	4.64777	5.33825	9.62749	8.82762
TNFAIP1	4.77562	4.85457	4.54269	4.917	5.38543	5.4868
TNFAIP2	-0.791952	-1.65505	4.69222	4.8627	5.64803	5.6431
TNFAIP3	4.6241	4.61663	2.13842	2.44193	1.89813	1.4532
TNFAIP6	2.28962	2.47086	-2.09546	-1.52346	-0.217212	0.965773
TNFAIP8	3.92389	4.21574	3.07387	3.36691	2.49958	2.40182
TNFAIP8L1	3.61307	3.65067	2.29142	2.65578	3.53477	3.24449
TNFRSF10B	6.9892	6.73617	6.40003	6.41862	6.17181	5.38604
TNFRSF11A	1.95199	2.27526 ?		-6.09892	-6.64581 ?	
TNFRSF11B	2.0083	2.32507	9.41944	9.75895	-3.07415	-1.89883
TNFRSF12A	5.52068	5.48951	4.4942	5.3557	6.22657	6.81664
TNFRSF14	-0.507753	-0.142243	2.41982	2.53748	-1.30241	-0.0950158
TNFRSF19	7.0501	6.84846	3.39391	3.5864	3.39226	3.76902
TNFRSF1A	5.34872	5.05531	6.25861	6.28805	5.24545	5.13642
TNFRSF21	3.63965	4.17559	4.82209	5.04408	3.08621	3.95862
TNFRSF25	-0.413499	-0.513325	1.496	1.30394	1.69749	0.993783
TNFRSF9	2.6206	2.82718	3.54099	3.86845	1.53448	1.66836
TNFSF10	-3.5275	-3.46127	4.41079	3.9342	4.25855	4.44048
TNFSF12	0.132879	-0.142919	0.208904	0.296275	0.86336	1.48999
TNFSF9	2.02545	2.11838	-0.350462	0.724135	-6.64581	-5.69922
TNIK	5.81267	5.84663	1.43123	1.17662	5.96309	5.35937
TNIP1	4.63853	4.75813	5.21842	5.6523	5.3544	4.80108
TNIP2	2.90084	2.89691	2.83885	3.36285	4.16464	4.57731
TNK2	2.92326	2.66527	3.50661	3.37263	3.89829	3.59889
TNKS	5.19475	5.29302	5.51493	5.59687	5.11574	5.19702
TNKS1BP1	4.70925	4.56435	5.48137	5.59725	5.54976	5.1843
TNKS2	5.38439	5.40404	5.09026	5.30109	6.00679	6.12696
TNNT1	2.36971	2.42591	2.83608	3.35061	-0.217212	1.71958
TNPO1	7.43923	7.44158	7.92286	8.18795	8.38322	8.27847
TNPO1P1	1.10207	1.24377	1.27708	1.49472	1.2518	1.22575
TNPO1P2	1.78765	1.90917	2.23865	2.65519	2.5419	2.41988
TNPO1P3	1.31668	1.64711	1.78473	2.20013	2.20494	2.08504
TNPO2	5.31909	5.15645	4.47022	4.5123	5.33262	5.12141
TNPO3	6.04451	6.03679	5.53586	5.8457	6.07753	5.93036
TNRC18	3.85932	3.46341	4.9895	4.67414	3.19456	3.11024

TNRC6A	5.86196	5.8054	6.488	6.63282	6.04297	5.82709
TNRC6B	5.75238	5.78449	6.10331	6.15678	5.81767	5.70373
TNRC6C	3.71689	3.73281	3.5932	3.68597	3.43958	3.16299
TNS2	1.78692	0.871875	4.57085	4.46299	1.82535	1.1637
TNS3	7.09114	7.03055	8.55592	8.5967	6.71393	6.96573
TOB1	3.36814	3.03152	2.94242	3.14343	5.08999	4.89041
TOB2	3.86115	4.10203	3.96419	4.18474	4.22288	4.13895
TOE1	4.01449	3.86643	3.65446	4.12243	3.31553	3.27995
TOLLIP	3.10828	2.86808	3.40519	3.77839	3.08284	3.35938
TOM1	2.91011	2.58701	3.46933	3.83368	2.73416	2.82857
TOM1L1	3.53345	3.66653	3.36915	3.87485	3.953	4.51217
TOM1L2	3.63631	3.3732	3.96113	3.98788	5.63027	5.23065
TOMM20	7.07483	7.158	6.53398	6.94018	7.53636	7.58126
TOMM20P4	2.6111	2.74455	2.21286	2.65818	3.01074	3.33565
TOMM22	5.93818	5.99555	5.15355	5.56491	5.76332	5.95056
TOMM34	5.54111	5.59919	4.55625	5.10791	5.18791	5.33699
TOMM40	4.99287	4.82257	4.7235	5.04677	5.21084	5.05958
TOMM40L	3.74044	3.80234	3.25152	3.65099	3.55525	3.96846
TOMM5	5.19978	5.37258	4.53871	4.95658	6.67337	7.76699
TOMM6	5.10457	5.12215	4.00077	4.39494	5.50253	6.18984
TOMM7	5.79521	5.52536	4.58466	5.0236	5.1486	5.91808
TOMM70A	5.35127	5.48459	5.3804	5.81771	5.5883	6.01806
TONSL	2.99963	2.76116	2.90924	3.03073	3.02093	2.61973
TOP1	5.92775	5.82486	5.88289	5.87594	5.29932	5.19641
TOP1MT	3.0658	2.88367	2.47113	2.38705	3.37036	2.91168
TOP2A	7.89646	7.7791	8.18603	8.46481	8.02087	8.20288
TOP2B	6.82575	6.81628	5.82806	5.99739	6.35947	6.27448
TOP3A	4.56125	4.57126	4.56382	4.78977	5.09998	4.88853
TOP3B	3.50298	3.35962	3.27899	3.51119	3.07127	2.76791
TOPBP1	6.06579	6.0678	5.56801	5.88904	5.73868	5.88293
TOPORS	4.91613	4.99246	4.48712	4.65936	5.97634	6.26053
TOPORS-AS1	0.280885	0.0404015	-0.194807	-0.17462	0.82489	1.32287
TOR1A	4.79178	4.9497	4.16299	4.61055	4.61698	5.19037
TOR1AIP1	6.14717	6.35965	4.98513	5.42982	5.4732	5.65191
TOR1AIP2	7.90285	7.93369	6.78566	7.02543	8.0538	7.82332
TOR1B	4.41855	4.35812	3.74128	4.00621	4.40779	4.27912
TOR2A	1.76447	1.27191	1.60441	1.65939	0.909612	1.4532
TOR3A	4.13063	4.12773	4.31232	4.69449	4.02268	4.07792
TOR4A	-2.61075	-2.46206	0.460428	0.248838	2.69284	2.46322
TOX	-4.69535	-5.19307	0.497985	0.445854	1.98555	2.15113
TOX2	0.00148521	-0.158917	1.94062	1.81006	-4.65668	-4.70296
TOX4	4.79369	4.88961	4.13254	4.55094	4.9089	5.14708
TP53	3.68013	3.81443	4.35756	4.77914	4.60266	4.91329
TP53BP1	6.08112	6.10086	6.23829	6.35524	6.31178	6.37088
TP53BP2	5.25983	5.17286	4.9914	5.09329	5.05177	4.68881
TP53I13	3.10989	2.80161	3.07873	3.22664	2.51213	2.50275
TP53I3	2.2198	2.41944	1.62387	1.94104	2.19769	3.28764
TP53INP1	2.44559	2.27188	3.23222	2.80706	3.45082	3.06148
TP53INP2	2.24346	1.692	3.11111	2.85696	3.53201	3.49786
TP53RK	4.5079	4.32261	3.63027	3.9892	4.62624	4.50152
TP53TG1	0.0289651	0.265185	2.92762	3.55472	0.894341	2.24167
TP53TG5	0.388278	0.190299	0.0600474	0.39858	-0.174546	-1.268

TP73	1.3978	1.39254	0.463382	0.279602	1.6467	0.946282
TP73-AS1	-3.84359	-5.94425	3.38785	3.54979	3.60682	2.75424
TPBG	4.82983	4.82428	3.55458	4.20745	4.70599	4.58904
TPCN1	3.35214	2.70458	4.4729	4.49757	3.78573	3.52211
TPCN2	3.48579	3.46048	2.81084	3.23547	3.52705	3.48311
TPD52	3.95161	4.1931	1.48028	2.01739	5.40519	5.22768
TPD52L1	-0.721575	-0.655276	-2.5977	-2.64851	2.70383	2.80899
TPD52L2	6.0825	5.53043	5.44126	5.45009	5.96084	5.55771
TPGS2	4.69235	4.54956	4.66077	4.91469	5.18506	5.19729
TPI1	7.94217	8.00312	7.98872	8.56944	8.7099	8.79102
TPI1P1	6.64819	6.73396	6.8032	7.27643	7.36786	7.50243
TPK1	0.604783	1.09945	-2.48229	-2.02079	-0.490197	1.43288
TPM1	8.4433	8.62826	6.92217	7.23926	7.06772	7.22534
TPM2	4.00655	3.86021	5.91976	6.33166	5.07313	5.07382
TPM3	7.49398	7.66446	6.90689	7.44591	7.64325	7.8808
TPM3P8	0.829127	0.985989	0.432543	0.857033	1.06915	1.03466
TPM3P9	2.90713	2.94668	2.256	2.36872	1.86341	0.937774
TPM4	6.73723	6.94043	7.0507	7.51265	8.0829	8.18364
TPMT	3.33021	3.19894	2.99639	3.50956	4.74007	5.18743
TPMTP1	0.133932	0.318612	-0.229171	0.19507	1.9393	2.71061
TPP1	4.74799	4.31605	4.49331	4.241	2.75544	2.64644
TPP2	4.8313	4.85683	5.65449	5.97133	4.92886	5.26051
TPPP	0.989989	0.655718	1.15465	1.04644	-0.0605387	-1.12145
TPR	8.06248	8.12213	7.36505	7.61375	7.64706	7.65811
TPRA1	3.19726	2.88589	3.56802	3.97996	2.34533	2.28199
TPRG1	-2.89397	-2.6546	-0.568612	-1.20162	1.88661	2.28766
TPRG1L	3.40026	2.97386	3.22798	3.26186	4.63715	4.4769
TPRKB	3.75188	3.98197	3.07365	3.49031	3.9869	4.50143
TPRN	1.91565	1.53154	1.36341	1.65939	1.60182	1.81688
TPST1	4.68732	4.62196	5.28764	5.54267	4.79038	5.03053
TPST2	2.49569	2.56507	2.94577	3.03073	2.55402	2.52212
TPT1	8.78679	8.58045	8.34625	8.68157	9.04458	9.76939
TPT1-AS1	2.04569	1.81783	2.43218	2.4131	1.82471	0.903655
TPT1P4	1.5277	1.59982	1.25363	1.60375	1.84039	2.43875
TPT1P9	3.57607	3.70174	3.51676	3.82881	4.20634	4.56651
TPX2	7.43504	7.42949	6.91442	7.24095	6.72577	6.58421
TRA2A	4.93935	4.92624	5.27846	5.2176	5.0727	4.59363
TRA2B	7.30786	7.28007	7.36431	7.4135	7.67489	7.64298
TRABD	2.69969	2.43215	3.17135	3.17954	2.84802	2.46663
TRABD2A	-0.865939	-0.818747	2.53759	3.11518	-1.41201	-1.24695
TRABD2B	2.08193	2.51792	-3.18222	-3.52222	?	?
TRADD	0.470938	0.223769	2.11571	2.39103	2.55159	3.15438
TRAF1	2.95377	3.98112	-0.276471	1.14861	1.40043	2.23582
TRAF2	3.64077	3.46493	2.98641	2.98129	2.56129	2.46823
TRAF3	5.02459	5.05432	3.63666	3.93557	4.10206	4.01609
TRAF3IP1	3.43791	3.34772	3.35564	3.5022	3.56971	2.71956
TRAF3IP2	2.79678	2.63783	3.08234	3.21985	2.98792	3.12739
TRAF3IP2-AS1	0.693311	0.514521	0.976408	0.845438	1.19149	0.578775
TRAF4	5.07602	4.79858	4.1985	4.23704	5.42925	5.19615
TRAF5	3.95286	3.9241	3.38253	2.89399	3.38818	2.56942
TRAF6	2.67278	2.65782	2.6232	2.66799	3.52623	3.34442
TRAF7	4.38698	4.23142	4.55362	4.68635	5.00941	4.96474

TRAFD1	3.63297	3.61398	4.45491	4.96328	3.26005	3.47071
TRAIP	3.01173	3.05876	2.34006	2.56007	2.76806	2.94793
TRAK1	4.63631	4.57194	4.26562	4.20405	5.75751	5.38669
TRAK2	5.44444	5.3776	5.03315	5.39763	5.96968	6.37813
TRAM1	6.00676	6.01649	6.86186	7.41487	6.40682	6.73388
TRAM1L1	1.0158	1.07637	-0.252626	0.176693	-0.216981	0.0483059
TRAM2	6.40305	6.32525	7.2228	7.30611	6.84499	6.37842
TRAM2-AS1	1.07576	0.859151	1.07392	1.03078	0.782702	1.29334
TRANK1	3.54171	3.75752	4.46931	4.67743	4.112	4.45567
TRAP1	5.56013	5.34676	5.03137	5.26258	4.73759	4.50209
TRAPPC1	3.44175	3.3103	3.72637	3.92185	4.42316	4.3741
TRAPPC10	4.56106	4.6801	5.1461	5.41916	5.07718	4.88287
TRAPPC11	4.11549	4.08885	4.23116	4.39995	4.28074	4.60048
TRAPPC12	3.67705	3.66127	3.16078	3.22209	4.08367	3.96571
TRAPPC13	3.50435	3.4646	3.98029	3.98545	4.21387	4.60089
TRAPPC2	1.88904	1.9895	2.44447	2.60936	2.59623	2.08257
TRAPPC2B	2.1582	2.15797	1.81187	2.08149	2.69265	3.34616
TRAPPC2L	3.82448	3.70331	3.55288	3.87701	3.20697	3.75272
TRAPPC3	4.53932	4.54278	3.88121	4.39995	4.79859	5.17898
TRAPPC4	4.58777	4.82428	3.71888	4.22479	4.26073	4.91079
TRAPPC5	1.66378	1.34645	1.22363	1.62027	2.31135	3.21186
TRAPPC6B	3.40553	3.52867	2.67447	3.04092	3.7512	4.0465
TRAPPC8	4.94761	4.98756	4.41517	4.68213	3.75288	4.10699
TRAPPC9	2.97688	2.8903	3.52038	3.26186	2.61116	2.36476
TRDMT1	3.26959	3.17197	2.16522	2.15329	3.52332	3.44557
TRERF1	3.12429	3.04891	4.23853	4.18808	2.94703	2.72792
TREX1	2.35259	2.59746	2.84554	3.32993	2.93056	3.3033
TRHDE	2.46809	2.95866	2.69563	3.58582	-5.07051	-4.11926
TRHDE-AS1	1.51578	1.74795	2.25172	2.50345	-4.07293	-5.69922
TRIAP1	3.77158	3.74903	2.70219	3.03838	3.66836	4.20766
TRIB1	4.62735	4.51942	3.84576	4.34238	2.55887	2.1262
TRIB2	5.98369	5.6907	5.36769	5.05229	6.55472	5.97321
TRIB3	3.41727	3.31852	3.74573	4.44767	3.64004	3.83825
TRIM11	4.08637	4.01692	3.71734	3.92459	4.14219	3.95862
TRIM13	4.90836	4.92297	4.79324	4.80869	4.69435	4.70146
TRIM14	2.55832	2.64406	3.41824	3.47425	3.47713	2.99244
TRIM16	4.54013	4.48588	6.60843	6.75853	6.25601	6.1556
TRIM16L	3.63167	3.38358	6.49629	6.49655	6.19967	5.90718
TRIM17	0.0289651	-0.036446	0.956086	0.810106	0.32848	-0.0918635
TRIM2	7.03407	6.86192	4.67678	4.93418	7.67066	7.55677
TRIM21	1.60474	1.9035	2.94834	3.47048	2.4351	2.78915
TRIM22	2.22993	2.62992	4.14515	4.4049	3.89906	3.79711
TRIM23	3.68786	3.7827	4.04769	4.45151	4.34391	4.93533
TRIM24	4.3824	4.3732	3.75752	3.73666	4.77327	4.57058
TRIM25	5.46551	5.55393	5.14515	5.44479	5.91569	5.73311
TRIM26	3.68787	3.74051	4.11683	4.49201	4.46445	4.32118
TRIM27	5.13182	5.1324	4.54184	4.77915	5.32125	5.61295
TRIM28	6.47614	6.41902	5.9697	6.22039	6.74109	6.89943
TRIM3	3.6857	3.7969	2.49948	2.7677	2.46898	2.56476
TRIM32	3.92206	3.91219	3.59783	3.75845	3.72015	3.70479
TRIM33	5.65709	5.78182	5.59496	5.67376	5.59204	5.27484
TRIM34	0.438729	0.786655	2.53963	2.85412	0.444519	0.635782

TRIM35	3.43535	3.39046	2.76485	3.081	3.07947	3.10707
TRIM36	1.72301	1.53719	0.558032	1.0909	0.578176	0.541297
TRIM37	5.29791	5.25808	4.87306	5.17108	6.12637	6.16481
TRIM38	3.96002	3.80511	4.58796	4.86773	4.32125	4.31237
TRIM39	2.59044	2.6282	2.53531	2.66265	3.49508	3.1561
TRIM4	3.79578	3.79099	4.20233	4.49201	4.47798	4.58675
TRIM41	3.75583	3.77165	4.24357	4.44893	4.45573	3.99323
TRIM44	5.91746	5.88891	5.88491	5.94272	6.70122	6.41481
TRIM45	1.88966	1.96344	2.16079	1.86274	3.61116	3.54831
TRIM46	1.00834	1.05291	0.191626	0.95732	-0.637731	-0.799586
TRIM47	3.34267	3.65026	2.5839	3.23906	3.3176	3.92215
TRIM5	3.27391	3.4782	4.30331	4.57239	3.18361	3.36743
TRIM52	3.36195	3.50672	4.58814	4.60395	3.96999	3.35159
TRIM52-AS1	0.987037	0.813155	1.34468	1.28065	1.47696	0.889894
TRIM56	5.05997	5.0355	5.73421	5.85764	5.26561	4.77003
TRIM59	3.08482	3.28877	3.90206	3.94071	3.11624	2.61171
TRIM6	0.768697	1.23054	0.940264	1.59447	-3.51607	-3.99627
TRIM62	0.50068	0.503023	2.55795	2.6856	1.77435	1.56011
TRIM65	4.40617	4.50149	3.25319	3.61056	3.7427	3.8932
TRIM66	3.09859	2.9122	3.48358	3.40885	2.60414	1.90064
TRIM68	2.34805	2.53717	2.32429	2.62928	1.47928	1.43287
TRIM69	2.43022	2.38892	2.70523	2.93284	3.40981	3.9657
TRIM7	0.508388	0.623417	0.944011	1.61123	0.495327	0.941707
TRIM8	3.34941	3.13707	3.18061	3.48922	3.68288	3.38604
TRIM9	3.6943	3.16651	0.27602	-0.177939	3.61115	3.2358
TRIML2	3.8097	3.20782	-1.32519	-2.93781	-6.64581	-5.69922
TRIO	7.17559	7.35743	7.39888	7.66798	7.75666	7.49232
TRIOBP	4.99972	4.701	4.75755	4.80218	3.75437	3.85857
TRIP10	3.67039	3.64696	3.81209	4.14123	4.19595	4.38602
TRIP11	5.72	5.72321	5.11617	5.27103	5.5334	5.75999
TRIP12	7.58849	7.56989	7.60978	7.75579	8.29763	8.18599
TRIP13	5.16007	5.10225	4.01245	4.42547	5.43967	5.53348
TRIP4	2.5201	2.50295	2.541	2.64607	2.65711	3.06478
TRIP6	4.24343	4.1051	3.56467	4.02816	5.07227	5.59076
TRIQK	2.7082	3.0406	2.53759	3.02817	1.79915	2.57407
TRIT1	3.34533	3.24624	2.96877	3.01788	3.83754	3.86504
TRMT1	3.12906	2.99249	2.5273	2.72407	3.41646	3.32947
TRMT10A	1.57242	1.61934	1.7294	1.62592	1.96907	2.10709
TRMT10B	2.15581	2.19181	1.95605	1.96801	3.52952	3.23287
TRMT10C	4.21307	4.36844	3.22375	3.54042	3.81442	4.59136
TRMT11	3.17897	3.11647	2.59128	2.76155	3.79399	4.35397
TRMT112	6.03418	5.98829	4.74571	4.96977	5.80009	6.21966
TRMT112P6	3.02963	3.02248	1.74482	2.00268	2.8127	3.13003
TRMT12	3.28961	3.39046	2.69913	3.19267	3.74589	3.81292
TRMT13	3.76182	3.99447	3.31275	3.28205	3.83336	3.72017
TRMT1L	3.94747	4.10225	3.67446	3.85696	3.85045	4.48434
TRMT2A	4.42887	4.29264	3.8715	4.11684	3.50798	3.53988
TRMT2B	3.27534	3.36212	3.342	3.75536	3.74377	3.90062
TRMT44	2.97335	2.75148	2.47827	2.63265	2.85145	2.57407
TRMT5	4.18466	4.34435	3.20807	3.36445	3.48678	3.84942
TRMT6	4.16123	4.32748	3.59783	4.07729	4.99301	5.0101
TRMT61A	2.69754	2.37321	1.33222	1.79206	2.54182	2.90431

TRMT61B	2.97185	2.99321	2.35149	2.69362	2.80687	2.81694
TRMU	4.90824	4.87477	4.87514	5.11335	4.30041	4.16211
TRNAU1AP	3.29951	3.2887	2.71433	3.13435	3.56128	3.80108
TRNP1	1.82058	2.04497	-0.095919	0.672606	0.32848	0.560157
TRNT1	4.11809	4.11391	3.30105	3.50952	4.07113	4.35278
TRO	4.24254	4.11269	3.73236	3.73823	0.740643	0.650921
TROAP	4.79277	4.66572	4.10523	4.4068	3.56128	3.4607
TROVE2	6.78715	6.8768	6.02157	6.26032	6.83341	6.71027
TRPA1	-2.02658	-2.31171	3.70173	4.28295	?	?
TRPC1	2.59555	2.84094	4.00383	4.13314	2.45345	2.73208
TRPC3	-0.816197	-0.790185	2.57804	2.65937	-0.753191	-0.38461
TRPC4AP	5.69242	5.7192	5.69183	6.04721	5.57239	5.86215
TRPM2	1.09539	0.938098	-2.86056	-2.30078	?	?
TRPM3	2.25513	1.78985	1.25116	1.4458	2.48436	2.43285
TRPM4	0.776727	0.444137	1.20884	0.89124	-1.85245	-2.12114
TRPM7	6.20062	6.35409	5.76631	6.25253	6.00505	6.63284
TRPM8	2.92456	2.34096	-0.0336419	0.213217	-0.43131	-0.619042
TRPS1	5.57734	5.40095	2.16522	1.8512	8.14013	7.80365
TRPT1	2.64078	2.58429	2.41268	2.65937	2.82955	2.80505
TRPV1	0.979111	1.22321	0.118253	-0.000595956	1.55556	0.850631
TRPV4	3.36027	3.81095	-2.5977	-4.10635	?	?
TRRAP	6.70519	6.63638	7.00537	6.94725	6.16722	5.77811
TRUB1	3.7511	3.96551	3.51518	3.88553	4.32552	4.34177
TRUB2	4.68507	4.65131	3.60578	4.11008	4.66298	5.06508
TSC1	4.45066	4.42589	4.88324	4.9773	4.67451	4.38736
TSC2	4.01001	4.00985	4.38465	4.50588	4.90716	5.08852
TSC22D1	5.90194	5.87012	5.46528	5.61318	5.90958	5.68804
TSC22D2	3.55004	3.51729	5.34736	5.09634	5.42084	4.66832
TSC22D3	1.27251	1.12217	4.12596	4.43709	1.74486	1.99375
TSC22D4	2.9218	2.57334	2.71434	3.11626	3.42285	3.51003
TSEN15	4.29132	4.38471	3.72781	4.03916	4.12625	4.61792
TSEN2	3.56888	3.56208	3.11569	3.09085	3.0262	3.01097
TSEN34	3.88117	3.87032	3.95856	4.28455	4.55036	4.98588
TSEN54	2.57239	2.52301	2.65254	2.71773	2.82553	3.12301
TSFM	3.22539	3.12323	2.88446	3.39949	3.13338	3.51066
TSG101	4.14005	4.11347	3.40414	3.66487	4.52081	4.90013
TSGA10	0.356302	0.292146	0.558032	0.334214	1.95442	1.91536
TSHZ1	4.2287	4.0927	3.12936	3.29205	2.87692	2.66833
TSHZ2	1.06916	1.09179	1.30034	1.42696	-6.64581	-5.69922
TSHZ3	?	?	3.29221	3.33618	0.234612	0.893261
TSKU	4.3897	4.36608	4.78296	4.77381	1.58765	0.832509
TSLP	-2.30606	-1.87737	1.36341	1.42259	?	?
TSN	5.93686	6.07486	5.00134	5.49387	5.43243	5.8363
TSNARE1	0.62293	0.151976	0.0260635	0.367002	0.688494	1.12623
TSNAX	5.42607	5.68509	4.64485	4.99601	5.52543	5.66212
TSNAX-DISC1	0.461874	0.911331	1.36602	0.262385	-0.239256	-1.7239
TSPAN11	3.99179	3.96026	0.912765	0.659431	-3.19956	-2.70579
TSPAN12	2.72509	2.77557	1.48185	1.18585	-6.64581	-5.69922
TSPAN13	6.23126	6.34127	0.597917	1.40691	2.76806	2.50275
TSPAN14	4.55653	4.59514	4.20734	4.30902	4.5745	4.39788
TSPAN15	0.183671	0.444137	0.584741	0.749225	4.09039	4.80898
TSPAN17	3.62172	3.81909	4.69375	5.14206	4.54303	4.95861

TSPAN2	-4.37417	-3.65369	0.805235	1.04098	2.47669	2.5925
TSPAN3	6.95559	6.31725	6.38777	6.39126	6.55248	6.07748
TSPAN31	3.26988	3.17985	3.99051	4.17448	3.60445	3.7577
TSPAN33	3.00828	2.59786	0.225978	-0.616766	0.997957	0.0483059
TSPAN4	4.48641	3.898	4.8886	4.90867	2.93589	3.40181
TSPAN5	3.95733	4.05874	4.07267	4.25749	4.7293	5.21289
TSPAN6	4.18355	4.06756	3.44765	3.17427	4.79709	5.09419
TSPAN7	0.356302	0.514521	3.05249	3.70334	?	?
TSPAN9	2.9804	2.25485	2.81084	2.23104	3.77221	2.87826
TSPO	3.33847	3.26848	3.1828	3.7209	3.17416	4.52929
TSPYL1	4.44656	4.38386	4.74009	5.02024	5.99871	5.78363
TSPYL2	3.58169	3.4782	3.29828	3.32163	3.40443	2.46322
TSPYL4	3.00828	2.99249	3.3203	3.35674	4.20619	3.73207
TSR1	6.02777	6.10025	5.45129	5.90491	6.97246	6.79761
TSR2	3.50793	3.44705	2.77938	3.09085	3.03666	2.66833
TSR3	2.60616	2.53534	2.63543	2.95408	3.16277	3.37526
TSSC1	3.37238	3.36688	2.52039	2.86846	3.86226	4.4174
TSSC4	3.41986	3.26338	2.77649	3.32789	2.77433	3.48804
TSSK4	-0.144345	0.237698	0.00643448	0.020584	0.293994	-0.31423
TST	0.952028	0.71714	2.68993	3.25312	0.134221	0.633217
TSTA3	2.78474	2.76598	2.98641	3.41277	3.51959	4.31981
TSTD1	-2.45038	-3.29152	-3.85951	-3.30006	2.62279	3.39395
TSTD2	3.67611	3.66437	2.96378	3.00028	3.7655	3.55867
TTBK2	4.21207	4.10065	4.72192	4.84958	4.65905	4.37764
TTC1	4.05329	4.32586	4.53501	5.17657	5.50521	6.12577
TTC12	-6.68418	-6.45019	1.12031	1.39106	2.25296	3.05816
TTC13	4.56474	4.66384	3.76485	3.94644	3.93587	4.04483
TTC14	5.14051	5.28282	5.20717	5.17425	5.93611	5.93577
TTC17	5.24636	5.20115	5.66509	5.72879	6.83186	6.7365
TTC19	4.96516	4.94898	4.91803	5.10022	5.58475	5.78564
TTC21A	0.578089	0.525929	0.770744	0.430424	-0.852709	-1.18284
TTC21B	4.51588	4.49219	3.9025	3.84417	3.69503	3.59837
TTC23	3.92004	3.85644	3.70124	3.7299	3.87475	3.88482
TTC26	2.94298	2.83408	3.20234	3.27272	3.28074	3.03138
TTC27	3.70712	3.90456	1.97132	2.20407	3.96357	4.38868
TTC28	4.46113	4.41442	0.936727	0.604403	5.5526	5.2115
TTC28-AS1	2.90298	2.93836	2.75485	2.78393	2.95661	2.49602
TTC3	6.50514	6.51693	7.2971	7.36427	6.59713	6.49856
TTC30A	2.84283	2.91389	4.1083	4.31119	3.39661	3.19557
TTC30B	1.97543	2.05768	3.13486	3.23755	2.34753	2.67064
TTC31	4.15106	4.16285	3.28612	3.41082	3.47668	3.48804
TTC32	1.25516	1.40138	1.08332	1.32585	0.634389	0.832509
TTC33	3.23018	3.37794	2.98641	3.20406	3.37998	3.55068
TTC37	6.18546	6.20248	6.44012	6.65	6.55702	7.08421
TTC38	3.03394	2.95501	3.48182	3.65106	3.0436	3.17899
TTC39B	2.81859	2.91002	3.57304	3.74915	3.81744	4.30311
TTC39C	4.02903	3.97745	2.95669	3.35848	2.12183	2.56555
TTC3P1	4.27856	4.28245	5.25158	5.2516	4.50992	4.47641
TTC4	4.16356	4.08767	3.89542	4.3298	4.2703	4.54831
TTC5	3.12033	3.21025	2.09043	2.40689	2.90698	3.14816
TTC6	-2.1135	-2.76144	0.474784	0.476226	1.17263	0.293394
TTC7A	3.26033	3.17197	3.74057	3.97374	3.49135	3.02226

TTC7B	3.23461	2.97179	2.13842	1.80409	2.15677	1.57179
TTC8	3.1805	3.19358	2.46035	2.79807	2.30549	3.04818
TTC9C	2.93574	2.95501	2.89597	3.37701	3.03492	3.31843
TTF1	3.78171	3.82371	2.88526	2.9997	3.32126	3.49541
TTF2	5.44239	5.5375	4.8595	5.1852	6.40846	6.18468
TTI1	5.87618	5.92306	5.33856	5.40095	4.92977	4.94524
TTI2	2.9662	2.98117	2.39485	3.01822	2.91976	3.01878
TTK	5.31946	5.518	4.05846	4.50035	5.00947	5.79461
TTL	6.41301	6.64267	4.82348	5.26291	5.68452	5.81045
TTLL1	-0.0415864	-0.0520781	0.277259	0.788878	0.311712	0.592235
TTLL12	5.20631	4.94867	3.83745	4.31743	4.5467	4.57983
TTLL3	1.6894	1.64418	2.77242	2.72416	3.7446	2.8767
TTLL4	5.0233	4.95024	4.44217	4.61823	4.79449	4.28055
TTLL5	4.61661	4.57209	3.81929	3.95186	3.87394	3.83825
TTLL7	4.13222	4.21046	3.9864	4.1391	6.13997	5.78262
TTLL7-IT1	0.133932	0.292146	0.431281	0.19507	1.63893	0.68568
TTN	5.21324	5.10659	2.08605	2.45563	4.93402	4.77627
TTN-AS1	3.58148	3.44938	0.191891	0.184217	3.12254	3.30461
TTPAL	5.08105	5.19937	5.0001	5.23213	5.32053	5.35871
TTYH2	1.68032	1.49139	0.53081	-0.0426024	-0.324567	-0.752286
TTYH3	4.41663	3.98939	6.14035	5.80443	6.18125	6.24565
TUB	4.28818	4.37871	4.24692	4.34237	3.27043	3.47568
TUBA1A	6.03747	6.30321	6.53493	6.89983	8.74341	8.86979
TUBA1B	9.92472	10.0381	9.41806	9.97542	9.70816	9.86222
TUBA1C	8.13734	8.10182	6.98859	7.62625	8.27858	8.33019
TUBA3C	4.63788	4.43905 ?		-6.02121	-6.64581 ?	
TUBA3FP	-0.00349555	0.336957	1.89523	2.2849	0.17081	0.138277
TUBA4A	-0.533681	-0.305667	5.26429	6.05641	2.97013	2.73089
TUBAP2	4.42134	4.70571	4.06968	4.64473	4.41709	4.4804
TUBB	9.27684	9.35727	9.20738	9.5721	9.99939	9.87704
TUBB1	0.827591	1.06244	-0.175794	0.0735324	0.346066	0.13522
TUBB2A	4.21304	4.17516	2.37317	2.95691	4.96741	5.76103
TUBB2B	5.41998	5.55167	-0.113782	-0.0209405	4.81941	4.83118
TUBB3	5.92651	5.90549	4.28593	4.96984	4.90998	5.45057
TUBB4B	7.36155	7.36488	6.62521	7.15829	7.12925	7.29938
TUBB6	5.53309	5.47927	4.03923	4.63376	5.23917	5.38762
TUBB8P1	0.312247	0.432061	0.120378	0.213217	0.509706	-0.214706
TUBBP1	5.62714	5.75887	5.47076	5.89556	6.06846	6.12831
TUBBP6	-0.254178	0.345896	0.0463793	0.402449	0.872931	-0.120327
TUBD1	3.02884	3.00068	2.67447	2.71773	3.39226	3.4607
TUBE1	2.21284	2.6233	2.25025	2.57008	2.65385	2.83245
TUBG1	4.56343	4.43917	4.16493	4.57317	4.90863	5.06943
TUBG2	2.31571	2.34597	2.16631	2.21817	2.63644	2.10919
TUBGCP2	4.30959	4.34258	4.31306	4.65261	4.94575	5.14508
TUBGCP3	3.86792	3.94322	3.89962	3.97777	3.50837	3.38743
TUBGCP4	4.35125	4.31839	3.83587	3.96244	4.70865	4.71917
TUBGCP5	3.44939	3.60324	3.84576	4.07852	4.03491	4.05484
TUBGCP6	4.16819	3.83521	5.2147	5.21815	3.81035	3.29329
TUFM	6.55198	6.52922	6.14339	6.41885	6.80089	6.89484
TUFMP1	1.29532	1.71211	0.967515	1.46905	1.71497	1.50278
TUFT1	2.95914	3.21311	2.29223	2.74605	4.5637	4.29328
TUG1	6.7058	6.5237	7.04541	6.82725	6.43278	5.89967

TULP3	4.23016	4.05679	4.17732	4.28026	3.48434	2.92992
TULP4	5.15655	5.04097	4.82209	4.68233	6.6531	6.45125
TUSC2	3.57704	3.69954	2.46034	3.01788	4.5473	4.96836
TUSC3	5.94545	6.30159	4.46303	4.93624	4.64461	5.14425
TUT1	3.07111	2.98523	2.8574	2.78946	2.48541	2.57595
TVP23B	4.20329	4.47088	4.49407	5.05807	4.93833	6.10086
TVP23C	-0.889204	-0.484705	1.57644	1.82309	2.64532	1.98133
TVP23C-CDRT4	-13.2877	-4.9812	-0.0932079	-0.302769	2.09388	2.05952
TVP23CP2	0.624251	0.680837	0.890384	1.48141	1.40553	2.13276
TWF1	6.04817	6.26953	5.29864	5.77782	6.22212	6.54567
TWF1P1	4.20849	4.36113	3.43441	3.94732	4.34408	5.35461
TWF2	4.18434	3.99423	3.52998	4.05563	4.62971	5.38981
TWIST1	1.32333	1.57061	2.33219	2.64657	1.7191	1.6942
TWIST2	2.50794	2.61665	2.48538	3.0933	-4.33548	-5.69922
TWISTNB	5.48206	5.54908	4.78081	5.26131	5.04877	5.34517
TWSG1	4.16511	3.97803	4.99822	5.38351	5.20732	4.97452
TXLNA	6.41498	6.52795	5.62343	6.24541	6.7848	6.93826
TXLNG	5.01571	4.75646	3.78289	4.10963	4.11234	3.94352
TXLNGY	-6.68418	-4.45968	6.06351	5.90483	-6.64581	?
TXN	4.74045	5.23639	6.00759	6.3737	5.36368	6.7069
TXN2	4.31354	4.12023	3.53072	3.69369	4.41449	4.62643
TXNDC11	3.62511	3.61921	3.67438	4.29405	3.66836	3.67699
TXNDC12	5.71025	5.88259	5.21284	5.69669	5.95608	6.65504
TXNDC15	4.93844	5.10844	5.11568	5.49711	5.19065	5.8134
TXNDC16	3.15111	3.12024	2.85814	2.60543	2.27928	2.22404
TXNDC17	3.80121	3.89579	3.83654	4.116	5.19277	5.97553
TXNDC5	7.73625	7.40225	7.00593	7.2361	7.75956	7.75348
TXNDC9	4.61739	4.79407	4.21157	4.77207	4.49524	5.51785
TXNIP	4.1927	2.31525	7.31345	6.6645	5.09291	2.25909
TXNL1	4.89318	5.1071	4.45181	4.83158	3.94654	4.33983
TXNL4A	5.10303	5.21663	3.6651	4.09084	4.17098	4.4174
TXNL4B	2.54055	2.67671	2.503	2.83954	4.01155	4.25906
TXNP4	-1.00531	-0.435111	0.281241	0.861336	-0.405451	1.00063
TXNP5	-1.23932	-0.680082	0.63659	0.783088	-0.258907	0.466831
TXNP6	1.9002	2.48394	3.18821	3.65753	2.39604	3.81542
TXNRD1	7.24568	7.14391	8.98434	9.36502	7.80729	7.98857
TXNRD2	3.14798	3.12212	2.82211	3.02046	2.15989	2.49297
TXNRD3	1.46083	1.39528	2.26771	2.33413	-0.490195	-0.619042
TYK2	4.39631	4.30202	3.91055	4.04852	4.90906	4.62083
TYMP	1.06089	0.788502	0.610983	0.535117	-1.39952	-1.89883
TYMS	4.55101	4.40008	4.66331	4.77628	5.54438	5.79235
TYRO3	5.47137	5.12932	3.90303	3.98898	4.53041	4.3751
TYRO3P	1.91666	1.65417	1.67872	1.30204	0.994544	0.321212
TYRP1	-0.191134	-0.105152	1.60441	1.45345	-1.30241	-2.53597
TYSND1	3.31354	3.31852	2.59456	2.82192	2.70602	2.9972
TYW1	3.99942	4.01825	4.1303	4.29941	4.19774	4.05919
TYW1B	1.55537	1.38363	1.25888	1.19719	1.19992	0.539918
TYW3	4.86441	4.94025	3.95451	4.12547	4.64175	4.83539
TYW5	3.61841	3.63494	3.50982	3.4303	3.56758	3.60367
Telomerase-vert	2.17434	1.6805	2.57715	2.70328	2.48492	4.07744
U1	7.31778	7.53197	7.41069	7.26231	7.08072	7.44485
U2	9.09148	9.09537	8.4566	8.49119	9.05228	9.89109

U2AF1	4.64922	4.59989	5.66954	5.53028	4.59221	4.21486
U2AF1L4	0.514329	0.548466	1.23789	1.6725	1.93656	1.35077
U2AF1L5	3.28642	3.09342	3.23223	3.74616	2.8107	2.46913
U2AF2	6.07199	5.98784	5.20987	5.5605	6.80905	6.80008
U2SURP	6.5614	6.67105	7.07711	7.21932	6.41941	6.32913
U3	1.19936	0.963482	1.63029	1.64609	1.16629	2.19417
U47924.31	0.296217	0.390932	1.0812	1.06299	-0.578056	0.679955
U47924.6	1.29202	1.09948	1.42731	1.78745	1.21445	1.15092
U73166.2	1.9484	2.22367	0.138526	-0.000171691	-0.356358	-0.799586
UACA	5.57531	5.70456	6.68544	7.0175	5.79629	6.10184
UAP1	5.90316	6.00448	4.06203	4.81226	6.53693	6.95858
UAP1L1	3.15423	2.86584	3.22587	3.58988	3.24657	2.59708
UBA1	7.2861	7.17997	6.41355	6.73847	6.17768	6.09618
UBA2	6.25728	6.35531	5.76594	6.13256	7.00005	7.39465
UBA3	4.73188	4.96914	4.44219	4.92654	4.59406	5.08282
UBA5	4.25292	4.26971	4.2282	4.26777	4.54366	4.18652
UBA52	7.01604	6.99603	5.97991	6.5194	7.72096	8.27388
UBA6	5.82936	6.13218	5.25422	5.65189	6.82565	7.22992
UBA6-AS1	2.24706	2.18528	1.93906	2.0075	3.514	3.66635
UBA7	0.710384	0.676782	4.25214	4.46292	2.66163	2.41742
UBAC1	3.72403	3.7319	3.14291	3.54222	4.07355	4.39
UBAC2	4.50852	4.51013	4.44035	4.60369	4.7427	5.22622
UBALD1	1.36166	1.06859	0.53081	0.968047	0.774384	0.176042
UBALD2	3.72718	3.42742	1.55122	1.98395	2.42451	2.55539
UBAP1	3.71953	3.70583	3.59436	3.87701	5.69301	5.8669
UBAP1L	2.13699	1.88591	1.23438	1.27492	1.50965	0.50283
UBAP2	4.32259	4.3275	4.38348	4.60799	5.21812	4.99372
UBAP2L	6.39647	6.29909	5.75126	5.9111	6.0629	5.87213
UBASH3B	6.40698	6.44102	3.49771	4.19609	4.85735	4.63758
UBB	7.39336	7.24257	8.03072	8.64078	8.5335	8.91017
UBBP4	2.10263	2.2346	2.25254	3.05508	2.96491	2.35686
UBC	9.46086	9.59307	9.49216	10.0174	9.39078	9.94107
UBE2A	4.10343	4.13431	4.56641	4.90243	5.16019	5.46759
UBE2B	4.10504	4.00374	3.73534	3.96129	4.08703	3.92627
UBE2C	5.78649	5.67318	4.4729	4.91283	4.77327	4.69309
UBE2D1	2.62736	2.82948	3.29424	3.41865	2.70602	2.41742
UBE2D2	4.40092	4.68482	4.53758	5.11396	5.07228	5.44745
UBE2D3	6.56129	6.6901	6.44063	6.80783	6.71441	7.12462
UBE2D3P1	2.62586	2.91386	2.30606	2.81548	2.9027	3.31579
UBE2D3P2	1.08405	1.24562	0.796257	1.2829	1.44118	1.86093
UBE2D4	2.13125	2.35016	0.559678	1.17344	2.75783	3.31636
UBE2E1	5.10547	4.94761	4.22267	4.60455	5.33685	5.76647
UBE2E2	2.82449	2.82487	-3.37468	-2.02079	3.60061	3.90246
UBE2E3	4.03611	3.84331	4.44632	4.31893	4.744	4.86807
UBE2F	3.6921	3.71252	3.05986	3.4409	3.56972	3.73322
UBE2G1	4.58921	4.36051	4.70064	4.95388	5.15906	4.77205
UBE2G2	5.62649	5.66675	5.96399	6.09787	6.41413	6.13444
UBE2H	5.93728	5.78394	6.09892	6.42112	6.91559	6.85763
UBE2HP1	-0.486594	-0.489838	0.600412	1.13763	0.829752	0.383652
UBE2I	4.75727	4.61836	4.84782	5.07855	4.76946	4.51116
UBE2J1	4.95331	4.94918	3.79232	4.23436	5.35544	5.44175
UBE2J2	3.58284	3.50438	3.17623	3.44192	4.44363	4.48187

UBE2K	5.31109	5.30534	5.29776	5.2662	6.28053	6.07547
UBE2L3	6.05888	6.12615	5.75035	6.16481	5.71936	5.87086
UBE2L4	0.818515	0.994652	0.266433	0.754913	0.626701	0.607636
UBE2L6	2.90084	3.24797	5.00135	5.68435	3.3537	4.07793
UBE2M	4.03747	4.02889	3.68485	4.07845	5.10023	5.33137
UBE2MP1	2.81036	2.89717	2.21862	2.77401	3.96486	4.00616
UBE2N	5.72982	5.80235	4.77147	5.12622	5.58421	6.17016
UBE2O	4.77764	4.80511	5.12879	5.31638	5.76041	5.17516
UBE2Q1	5.08105	5.1477	4.05607	4.51869	5.2116	5.41158
UBE2Q2	3.67051	3.73314	3.36145	3.77991	3.89428	4.11983
UBE2Q2P1	0.61388	0.344613	2.65409	2.69174	0.847548	0.422664
UBE2Q2P2	1.0834	0.947539	1.33439	0.90032	0.855192	-0.451193
UBE2Q2P6	7.53838	7.47392	6.99958	7.51575	8.40427	9.92747
UBE2R2	4.18202	4.15645	4.38917	4.62249	5.58464	5.42192
UBE2S	4.18358	3.95441	3.74747	4.312	5.19076	5.40179
UBE2SP1	4.56475	4.32129	4.03214	4.63869	5.56801	5.51023
UBE2SP2	2.56775	2.45347	1.9443	2.42624	3.39412	3.90552
UBE2T	4.39763	4.48625	3.72186	4.11154	3.95624	4.56007
UBE2V1	5.49293	5.4645	5.31709	5.5236	5.67718	5.74051
UBE2V1P2	1.97103	2.04606	1.71054	2.19812	2.32565	2.48985
UBE2V2	5.1824	5.28951	4.8629	5.15795	5.08745	5.32395
UBE2W	4.09123	4.22277	4.39012	4.65355	4.95854	5.22337
UBE2Z	5.92889	5.9692	5.65527	6.06482	6.01483	6.23069
UBE3A	6.03015	6.11397	5.95137	6.21535	5.44559	5.65674
UBE3B	4.59124	4.51236	4.57569	4.7268	4.24822	4.13721
UBE3C	6.61044	6.6282	6.39864	6.72387	6.21296	6.12104
UBE3D	2.8107	3.14449	1.89332	2.02562	2.64119	2.88201
UBE4A	6.35277	6.44195	6.5669	6.85082	6.33582	6.39314
UBE4B	5.2493	4.99915	5.04649	5.02755	5.51368	5.02714
UBFD1	5.21099	5.79819	5.3144	6.01425	5.29258	5.91519
UBIAD1	2.97335	2.90567	2.81366	3.22209	4.12432	4.07628
UBL3	3.90547	3.70707	3.07622	2.95996	5.22918	5.06311
UBL4A	5.10019	5.03248	3.9573	4.33203	5.27268	5.61971
UBL5	4.9355	4.89133	4.01737	4.43367	5.57246	6.61613
UBL5P2	0.435309	0.851383	-0.0134303	0.37569	1.31046	1.94967
UBL7	3.96714	3.87478	2.99888	3.33825	4.01211	4.47939
UBL7-AS1	1.25688	1.27003	1.40799	1.44067	0.603074	1.06392
UBLCP1	3.74178	3.59785	4.1474	4.22321	4.35369	4.34176
UBN1	5.05371	5.20782	4.92826	5.06296	5.01696	4.63979
UBN2	5.13223	5.07534	4.86904	4.73195	5.30981	5.12538
UBOX5	2.07736	2.18168	3.01981	3.18502	2.12868	2.16511
UBP1	5.9216	5.78776	5.01184	4.91769	6.12124	5.9299
UBQLN1	5.98481	6.15735	6.11567	6.29734	6.07563	6.20355
UBQLN2	3.23166	3.25826	3.86768	3.91493	4.18596	4.46696
UBQLN4	5.14869	5.12682	3.64257	3.85003	4.4902	4.55063
UBQLN4P1	2.99116	2.89022	1.52581	1.76028	2.3135	2.26502
UBR1	5.50088	5.54804	5.15855	5.36993	5.41281	5.74912
UBR2	4.88494	4.96182	5.00876	5.42159	5.28581	5.60558
UBR3	5.78346	5.88087	5.99855	6.11154	6.10748	6.04231
UBR4	7.69696	7.69144	8.26551	8.41495	8.16623	7.47939
UBR5	6.60556	6.466	7.19462	7.24879	6.88157	6.65303
UBR5-AS1	0.370577	0.674569	1.27865	1.14795	0.800024	0.52311

UBR7	4.92569	4.96539	3.63186	4.00752	4.90897	4.90153
UBTD1	2.16512	1.74176	2.19587	2.28779	2.61582	2.41742
UBTD2	3.48083	3.67798	4.17513	4.31743	4.18909	4.26051
UBTF	5.29809	5.25482	4.96558	5.13013	5.66415	5.51243
UBXN1	4.95778	5.00118	4.08911	4.60456	4.54181	4.57521
UBXN11	2.74066	2.46648	2.76128	2.7468	1.86673	1.58814
UBXN2A	4.45763	4.12117	3.40893	3.15327	4.2719	3.71954
UBXN2B	3.41321	3.58316	3.46688	3.78013	4.49344	4.75132
UBXN4	6.13538	6.11059	6.48446	6.57901	6.41449	6.14696
UBXN6	3.59548	3.41238	3.56123	3.7967	4.48341	5.02203
UBXN7	5.41463	5.25394	6.09433	6.11999	6.28	5.99718
UBXN8	3.11951	2.91654	1.69303	1.89683	2.64119	3.0041
UCHL1	4.77259	5.12445	6.71544	7.31987	8.17865	8.47653
UCHL3	2.46025	2.5211	2.1651	2.75451	3.13873	4.03564
UCHL5	5.5187	5.62383	4.65363	4.93593	5.38096	5.45265
UCK1	3.76852	3.51301	3.20018	3.47614	3.87399	3.86314
UCK2	5.90644	5.88233	4.41709	4.70457	5.19876	5.20909
UCKL1	3.52715	3.4954	3.07324	3.52682	3.44144	3.83204
UCKL1-AS1	0.55616	0.51784	0.467582	0.441292	0.0754505	0.022517
UCN2	2.7492	2.80196	2.08568	2.5559	1.16493	0.764049
UCP2	2.27535	1.89913	1.6494	1.4458	-0.530833	-0.280287
UEVLD	3.20028	3.55536	3.53072	4.0243	3.6537	4.0246
UFC1	4.69592	4.42513	3.70674	3.94238	5.24768	5.48188
UFD1L	5.77734	5.82661	5.27479	5.67911	4.84278	4.92415
UFL1	4.08882	4.12399	4.35175	4.41571	4.44821	4.91442
UFM1	4.64244	4.69181	4.70967	5.16377	5.21772	5.44999
UFSP2	3.12609	3.19138	2.498	2.73766	3.43808	3.69664
UG0898H09	-6.68418	-6.45019	-6.17309	-5.10387	2.71691	2.46823
UGCG	5.28817	5.7466	5.5234	6.20602	4.71635	5.41675
UGDH	4.57476	4.51658	5.31037	5.98246	4.64225	4.79912
UGGT1	7.77871	7.78919	7.28887	7.53247	7.29334	7.14345
UGGT2	5.23533	5.28827	4.98576	5.13374	4.58225	4.81193
UGP2	6.69591	6.85131	4.77719	5.10001	5.17374	5.75423
UGT8	5.01256	5.27102	-4.59508	-5.10387	3.6605	3.60163
UHMK1	7.37556	7.8535	6.82086	7.53665	7.31473	8.30049
UHRF1	5.00494	5.50465	4.06118	4.70698	4.34028	4.53049
UHRF1BP1	4.4532	4.41366	4.14178	4.23547	5.33441	4.808
UHRF1BP1L	4.38904	4.6677	4.45579	4.91908	4.42052	5.10704
UHRF2	5.01114	4.95116	4.48427	4.37196	5.47646	5.26609
UHRF2P1	0.55218	0.648401	1.45478	1.42259	1.61434	1.34029
UIMC1	3.72508	3.77675	4.13388	4.30585	4.39632	4.35262
ULBP2	1.72869	1.359	1.87597	2.34141	2.72209	2.6808
ULBP3	2.15793	2.26114	0.525087	0.964606	1.75315	1.44652
ULK1	2.28678	1.87703	2.9406	2.86559	2.06763	2.10066
ULK2	-6.68418 ?		-1.72363	-1.93836	2.90384	2.18205
ULK3	3.6251	3.46345	2.72638	3.07605	3.55764	3.51487
ULK4	2.27822	2.11082	1.80519	2.02562	2.50963	2.50762
UMAD1	2.65365	2.69975	2.53934	2.78028	3.06713	3.26529
UMPS	4.2746	4.39357	3.87717	4.2727	4.13572	4.5269
UNC119	3.04239	2.96343	3.41453	3.70655	3.86814	3.35127
UNC119B	4.80072	4.84946	4.188	4.68358	4.04416	4.27912
UNC13B	3.49567	3.70457	2.08796	2.37096	5.56306	5.75478

UNC45A	5.19327	5.16034	5.07408	5.37779	5.11816	5.14784
UNC50	3.7449	3.64829	3.29018	3.43034	3.92465	4.15747
UNC5C	3.99701	3.43046	1.82773	1.68562	6.70476	6.63728
UNC80	0.54893	0.603321	-3.85951	-3.78492	1.44302	0.965773
UNC93B1	1.76289	1.83639	2.57433	2.98573	1.90036	2.65524
UNG	4.749	4.85287	3.37682	3.87843	4.06164	4.36742
UNK	3.79137	3.52847	3.35581	3.4089	3.75761	3.63707
UNKL	3.54563	2.62342	3.80244	3.07829	2.95264	1.43376
UPF1	4.58632	4.4877	4.33217	4.47519	5.04184	5.16826
UPF2	4.87315	4.91709	4.06322	4.21196	4.9781	4.85554
UPF3A	3.71169	3.58284	4.07382	4.03246	3.93848	3.71377
UPF3AP1	0.457689	0.0556122	0.424245	0.780814	0.504417	0.971847
UPF3AP2	0.529981	0.607437	0.833497	0.903254	1.43103	1.06577
UPF3B	3.05914	3.02901	3.15633	3.36488	3.96082	3.76292
UPK3BL	2.62694	2.48433	2.78621	2.79286	2.52286	1.71877
UPP1	4.24636	4.17559	3.39013	3.85262	4.54547	4.4481
UPRT	2.27535	2.34452	2.52039	2.59509	4.12513	4.27627
UQCC1	5.11989	5.18596	5.14738	5.49015	4.85882	5.18278
UQCC2	3.78213	3.77959	3.75763	4.11641	4.60758	5.01329
UQCC3	2.17481	1.95523	1.76003	1.81973	1.85624	2.49209
UQCR10	4.44429	4.21926	4.7316	5.0428	4.96949	5.45125
UQCR11	3.76137	3.70206	2.87176	3.2399	4.3453	5.57521
UQCRB	4.81247	4.86344	5.07632	5.40495	5.78533	6.81853
UQCRBP1	1.20622	1.37217	1.6338	2.022	2.452	2.86293
UQCRC1	6.41253	6.40461	5.57885	6.08283	6.97765	7.23339
UQCRC2	5.47021	5.48734	5.71544	6.11215	5.61318	6.04398
UQCRFS1	4.56863	4.44669	5.17698	5.4364	5.93931	6.26402
UQCRFS1P1	0.371592	0.473371	1.2225	1.62213	1.98798	2.36054
UQCRH	5.77805	5.80923	5.33465	5.88259	6.16723	7.10221
UQCRHL	5.63527	5.65278	5.22264	5.62384	6.05193	6.62136
UQCRQ	5.55416	5.55533	5.28865	5.71925	6.20711	7.32985
URB1	5.82491	5.84596	5.68529	5.87985	5.89377	5.63366
URB1-AS1	0.399051	0.122235	1.07392	0.946514	1.29393	1.58794
URB2	5.20065	5.17558	3.95985	4.24211	5.32232	5.27769
URGCP	4.58265	4.28555	4.48357	4.52631	4.33297	4.05985
URI1	4.74489	4.71767	4.77864	5.03646	5.71361	5.86835
URM1	4.28176	4.08211	3.59455	3.81894	4.04963	4.28763
UROD	5.18355	5.12492	5.56131	5.89432	5.10414	5.68291
UROS	3.55948	3.45154	3.94189	4.21084	3.95715	4.27342
USB1	2.94838	2.63783	3.87458	4.23658	4.98804	4.94702
USE1	1.69327	1.64571	0.904002	0.761609	2.3397	2.27058
USF1	4.4532	4.47305	4.26458	4.56448	4.4625	4.56357
USF2	4.03562	4.17829	3.2088	3.60026	4.04186	4.20767
USH2A	1.16516	0.963482	0.431281	0.020584	0.351018	0.483199
USMG5	4.57336	4.6877	3.53194	4.15152	5.4195	6.72514
USMG5P1	0.370466	0.353798	-0.176968	0.167101	1.52011	2.32248
USO1	5.33707	5.55569	5.46481	6.00552	6.40903	7.02555
USP1	6.27068	6.34323	5.69063	6.04975	5.95273	6.3714
USP10	5.74398	5.78064	5.761	6.02121	5.44035	5.21616
USP11	5.05288	5.09747	4.04409	4.38702	4.2413	4.3087
USP12	4.2584	4.49494	4.33931	4.97148	4.09869	4.75277
USP12PX	-0.322074	-0.138969	0.220503	0.564398	0.0184633	0.579238

USP13	4.6716	4.55604	4.74278	4.8526	5.06759	5.00924
USP14	4.37723	4.38809	5.0217	5.19892	5.40573	5.52449
USP15	5.59094	5.61329	4.88053	5.15385	4.96676	5.47071
USP16	4.53071	4.47645	4.97533	5.0085	5.06456	5.54296
USP18	0.457657	0.201132	1.65747	1.6769	2.69891	2.58792
USP19	5.26526	5.14077	4.31432	4.60541	5.44656	5.50881
USP20	2.37239	2.05287	2.52729	2.48364	2.49957	2.35939
USP21	3.59554	3.50005	3.00136	3.1835	2.90003	2.87072
USP22	6.68908	6.3877	7.13628	7.23199	7.69574	7.06845
USP24	6.59093	6.61663	6.55582	6.6717	6.34688	6.2982
USP25	4.101	3.99248	4.80586	5.001	4.6406	4.66178
USP27X	1.55834	1.28539	0.945825	1.21315	-0.374735	-0.848487
USP28	2.82841	2.69704	4.75972	4.99248	4.3312	4.24602
USP3	4.70057	4.7415	4.36215	4.41828	4.45068	4.38888
USP3-AS1	1.20116	1.29579	1.22408	0.690024	0.345135	-0.340028
USP30	2.36702	2.37321	2.43406	2.58118	2.06081	2.1388
USP31	5.07115	4.93214	4.44127	4.49664	5.31625	4.66069
USP32	5.63132	5.63949	5.03496	5.47411	6.12791	6.16316
USP32P1	-3.11163	-2.91325	-0.269587	-0.177761	2.31559	1.265
USP32P2	?	-12.9395	2.86146	2.51541	1.42538	0.773406
USP32P3	-5.63492	-6.40401	0.673448	0.73683	2.64022	2.36405
USP33	5.49565	5.73345	5.85606	6.14058	5.81693	6.27377
USP34	7.65994	7.57762	6.93387	7.03442	7.22896	7.18605
USP35	3.13697	2.94655	3.18934	3.34856	2.0946	1.41227
USP36	4.78674	4.63386	4.00383	4.10306	4.8245	4.29046
USP37	4.61998	5.03544	4.83439	5.22425	4.78042	5.18309
USP38	4.40944	4.43045	4.16409	4.48362	4.85096	5.21215
USP39	4.9225	4.86076	4.15521	4.52597	4.86422	4.94522
USP4	4.36566	4.38967	3.72787	3.9024	4.30691	4.25184
USP40	4.92114	4.65868	3.65253	3.67253	5.06376	4.72583
USP42	3.19119	3.10511	3.42011	3.23103	3.04359	2.67267
USP43	-2.45038	-2.6546	1.20023	1.52057	?	?
USP45	3.17897	3.55674	3.50122	3.54402	3.89043	3.98153
USP46	4.37506	4.42055	3.80232	3.89679	4.27926	4.20017
USP47	5.54201	5.50221	6.05545	6.29472	5.4638	5.56533
USP48	5.36633	5.35892	4.91779	5.09329	5.34741	5.19415
USP49	2.82355	2.76139	3.44257	3.21409	3.1113	2.27378
USP5	5.78472	5.59616	5.44764	5.78787	5.60851	5.50515
USP51	0.183671	0.237698	1.09265	1.26626	?	?
USP53	5.48331	5.39047	2.77649	2.73353	4.55278	4.44556
USP54	3.65717	3.8568	3.40962	3.65868	3.31553	3.38299
USP6NL	4.72191	4.6509	3.29626	3.47801	4.03926	3.78313
USP7	5.79531	5.79411	5.90841	6.1226	5.69845	5.67475
USP8	5.39999	5.45828	5.00602	5.24422	5.78653	6.04561
USP8P1	0.976195	1.11517	0.597917	0.948048	1.59855	1.29333
USP9X	7.06256	7.00878	5.7659	5.92007	7.1063	7.04228
USP9Y	?	-4.45968	5.27997	5.25036	-6.64581	?
USPL1	4.022	4.09844	3.97885	4.2299	4.30114	4.46945
UST	2.28108	2.27864	2.60439	2.88836	4.70874	5.15979
UTP11L	4.82888	4.79512	3.58632	3.85839	5.12595	5.21882
UTP14A	3.9984	4.12759	3.58774	4.08294	4.84257	5.28669
UTP14C	4.30868	4.37862	4.55275	4.73056	4.5351	4.5632

UTP15	4.20113	4.29952	4.18388	4.58389	4.64859	4.9379
UTP18	4.92524	4.96289	3.8771	4.3669	4.42979	4.9841
UTP20	6.79478	6.85315	5.06321	5.36335	5.68564	5.61802
UTP23	3.81605	3.91118	3.29567	3.61985	4.61433	4.68455
UTP3	4.52853	4.71581	3.34004	3.75845	5.13084	5.30101
UTP6	5.27017	5.41094	4.52362	4.99235	5.28546	5.80255
UTRN	6.01193	6.11363	6.26871	6.61033	8.53963	8.24801
UTY	?	-6.45019	4.73534	4.59853	-5.65305	-5.69922
UVRAG	4.2292	4.42503	3.74662	3.95726	3.10623	3.32119
UVSSA	2.66507	2.41981	2.60439	2.52418	3.04705	2.52692
UXS1	5.40876	5.91273	3.90393	4.5022	3.7512	4.19717
UXT	2.98696	2.8161	1.94951	2.35812	2.77832	3.29858
VAC14	3.6251	3.46641	3.74869	3.93966	4.52765	4.31704
VAC14-AS1	0.0559175	0.2376	1.02599	1.12963	1.45867	0.578775
VAMP1	2.30042	2.07731	3.43635	3.56191	2.66032	1.78067
VAMP2	1.49078	1.38894	2.56132	2.46483	2.21007	2.04819
VAMP3	4.55062	5.00169	5.03624	5.88764	5.35126	6.02672
VAMP4	2.89525	3.06659	3.12709	3.15796	2.3227	2.65085
VAMP5	0.243547	-0.214764	2.54441	2.96532	-5.07051	-5.69922
VAMP7	4.99615	4.98579	4.41265	5.01914	5.53934	5.95105
VANGL1	5.42343	5.48003	5.20611	5.4841	6.09582	6.08809
VANGL2	2.68895	2.10703	-6.17309	?	-6.64581	-5.69922
VAPA	5.15319	5.15413	5.8007	6.19069	6.66185	6.89808
VAPB	5.87218	5.76204	5.31383	5.31427	5.39532	5.23652
VARS	5.33777	5.02699	4.66117	4.89046	5.81414	5.74243
VARS2	3.64926	3.41274	2.61164	2.74699	3.6945	3.63698
VASH1	2.03099	1.66983	1.47289	1.51261	-2.08446	-2.50285
VASH2	1.63634	1.64047	2.2002	1.76772	-0.10558	-0.799584
VASN	-1.69862	-2.6546	3.55288	3.54939	0.172692	0.0483059
VASP	4.42375	4.57333	3.938	4.23324	5.01431	5.56765
VAT1	5.07158	4.83692	5.63105	6.10487	5.38509	5.11026
VAT1L	-4.37417	-2.6546	0.73541	1.60545	-2.85193	0.719622
VAV2	3.26094	2.83408	2.94577	3.16963	3.20388	3.0041
VAV3	0.735617	0.278722	4.02348	4.04472	-6.64581	?
VBP1	4.5689	4.54975	4.55373	5.00555	5.28328	6.10504
VCAM1	0.0559314	-1.41808	5.86088	5.71574	-0.0163223	-0.0918635
VCAN	7.73861	7.88743	6.59268	6.18176	9.48937	9.57547
VCL	6.75912	7.02879	7.429	7.79282	8.28189	8.18156
VCP	7.73303	7.17143	7.60938	7.52333	9.0649	8.2693
VCPIP1	4.61776	4.70079	5.32377	5.55161	5.19494	5.28267
VCPKMT	1.88966	1.96344	1.12031	1.38307	1.62974	1.86317
VDAC1	7.43007	7.60685	6.84197	7.39338	7.63522	7.76753
VDAC1P1	4.83764	5.05576	4.20037	4.82502	5.02124	5.09753
VDAC1P2	2.91079	3.10051	2.17088	2.89829	3.07452	3.40057
VDAC1P6	2.16047	2.23701	1.55124	1.94208	2.2804	2.30651
VDAC1P8	-1.13126	-1.26811	1.65311	1.71989	2.51377	2.11569
VDAC2	4.13293	4.17797	3.77145	4.31897	4.8491	5.18046
VDAC3	5.50088	5.66897	4.85607	5.40243	5.30434	5.92624
VDAC3P1	-0.0255147	0.433109	-0.252626	0.19507	-0.0590936	0.127052
VDR	2.38308	2.45901	2.61739	3.78143	1.60651	1.53173
VEGFA	4.49626	4.64476	5.0316	5.72116	6.99517	5.63508
VEGFB	3.37642	3.26508	2.89864	2.83076	3.00502	2.96925

VEGFC	3.77665	3.47967	3.4275	3.79505	2.66613	2.89692
VEPH1	-4.11163	-3.65369	-0.628724	-0.616766	0.885348	2.25166
VEZF1	6.14188	6.12033	5.08964	5.30892	6.56641	6.39112
VEZF1P1	0.540315	0.6515	-0.251212	0.0447438	1.204	0.946424
VEZT	4.89849	5.04395	5.12651	5.29311	5.1058	5.27199
VGF	2.09536	1.614	-1.86109	-1.46458	-2.33767	-1.38442
VGLL3	2.86317	2.85909	-0.659745	0.248838	-0.615709	-0.848487
VGLL4	5.47706	5.08853	4.29715	4.37484	5.48409	4.89049
VHL	5.63322	5.5343	4.4531	4.44479	5.30362	4.48803
VIM	9.34192	9.30194	7.98483	8.6496	10.3463	10.8048
VIM-AS1	0.604783	0.357428	-1.42826	-1.02108	0.0866072	0.560157
VIMP	4.68623	4.74171	3.97129	4.27163	4.38544	4.59479
VIPAS39	3.0658	3.12024	2.66824	2.86559	3.35092	3.55773
VKORC1	4.98347	4.8354	4.19804	4.55724	5.09884	5.0549
VKORC1L1	4.90313	4.85624	4.83744	4.95785	4.80676	4.63531
VLDLR	2.9138	2.96552	0.816542	0.82198	7.28524	7.21585
VMA21	6.05517	6.06388	5.69107	6.00588	7.29496	7.06332
VMAC	0.768595	0.331671	0.838911	0.978694	2.75544	2.08772
VMP1	8.31763	8.26596	8.7446	8.82733	9.41249	9.36111
VOPP1	5.66153	5.80901	6.14696	6.4676	6.38543	6.33385
VPRBP	5.13681	5.15575	4.27299	4.54327	5.25286	4.97456
VPS11	3.90362	3.84662	4.00506	4.331	3.84351	3.8188
VPS13A	6.45608	6.50509	5.99698	6.01336	6.1456	6.07868
VPS13B	5.35145	5.33237	6.30456	6.24981	6.08935	6.16632
VPS13C	6.75969	6.67128	7.0501	7.01423	6.57509	6.81215
VPS13D	6.40356	6.37101	6.28535	6.3815	6.28569	6.05648
VPS16	4.09811	4.06737	4.10573	4.44258	3.49244	3.62664
VPS18	4.10182	4.00883	3.56802	3.93283	4.14462	4.34585
VPS25	4.71137	4.58768	4.27488	4.54581	5.00591	5.50212
VPS26A	3.70818	3.65479	3.98766	4.13077	4.45408	4.77003
VPS26B	3.98838	4.00758	4.23511	4.39695	4.9681	4.85376
VPS28	3.50915	3.29037	3.19802	3.41277	3.44559	3.6813
VPS29	4.43767	4.54908	4.28509	4.56713	4.55401	4.96127
VPS33A	4.21179	4.30445	3.2529	3.67922	4.18515	4.49721
VPS33B	3.13223	3.06659	2.79663	3.07853	3.03318	3.06808
VPS35	5.68352	5.77792	6.15878	6.60174	6.78364	7.26664
VPS36	4.7277	4.96759	3.93542	4.11033	4.15027	4.43157
VPS37A	4.14003	3.94663	4.27636	4.49467	4.22679	4.20087
VPS37B	4.27247	4.0341	2.78763	2.95143	4.20899	4.10654
VPS37C	2.47835	2.45603	1.00636	1.18584	1.99076	1.78517
VPS39	4.92159	4.84605	4.74424	4.99118	5.34356	5.1222
VPS41	5.46707	5.56472	5.06737	5.32058	5.95049	6.2511
VPS45	4.84345	4.86246	4.46663	4.74993	5.02881	5.32601
VPS4A	5.09327	5.03095	4.94903	5.16226	6.25347	5.98416
VPS4B	5.30267	5.17828	3.75899	4.06983	4.10041	3.8876
VPS50	4.162	4.1592	4.52037	4.87914	3.91428	4.68882
VPS51	3.90169	3.62391	3.31918	3.63114	3.7077	3.86508
VPS52	3.81673	3.596	3.72963	3.85543	4.58407	4.48455
VPS53	4.12213	4.04142	4.7061	4.97219	5.55473	4.99305
VPS54	4.44302	4.43273	3.97886	4.09696	4.87496	5.18583
VPS72	4.26506	4.18172	3.38732	3.60869	4.2844	4.53407
VPS8	3.80673	3.90456	4.64857	4.70815	5.60001	6.03094

VPS9D1	1.63075	1.49321	1.65408	1.76137	1.37133	1.55905
VRK1	4.76443	4.84776	3.23222	3.63769	3.43508	4.14348
VRK2	3.22305	3.10169	2.78846	3.26501	-0.959851	-1.7242
VRK3	2.67417	2.65351	3.2754	3.42645	3.6274	3.52966
VSIG10	4.54508	4.59181	3.66647	3.95318	3.55812	3.76003
VSNL1	-0.721575	-0.681268	-0.482894	-0.154483	2.84353	3.62868
VTA1	4.46457	4.59853	4.32228	4.49015	5.74538	6.36304
VTI1A	3.72403	3.6703	4.03806	4.21084	4.07101	4.05483
VTI1B	4.82429	4.96345	4.01752	4.58643	5.34638	5.63673
VWA5A	1.7968	1.67161	-0.300715	-0.301307	-3.6585	-3.38329
VWA8	4.24416	4.27694	4.00507	4.09574	4.04619	4.16519
VWA9	4.49383	4.456	4.09725	4.26185	4.21004	4.43666
VWDE	-0.654472	-0.0197685	-1.93506	-1.52346	2.74484	3.01439
WAC	6.04931	6.08813	5.81276	5.97098	6.17158	6.08036
WAC-AS1	2.39767	2.47506	2.77695	2.98866	2.7152	2.72787
WAPAL	5.93047	5.93723	5.64144	5.82338	6.16287	6.09255
WARS	5.97439	5.73049	4.73807	5.37016	4.94158	5.04063
WARS2	2.27791	2.33081	2.47287	2.42698	4.53893	4.72791
WASF1	2.90825	2.89252	2.30031	2.43808	3.57809	3.18507
WASF2	5.17491	4.92761	4.91548	4.91024	5.21055	4.76109
WASF3	-4.37417	-4.13839	2.30835	2.28351	4.28294	4.17592
WASF4P	1.23489	0.958806	0.799726	1.02808	1.07239	0.479189
WASH1	2.17097	1.96751	1.64422	1.55761	2.96029	2.00976
WASH2P	2.34287	2.11509	2.69028	2.57915	2.32159	1.44916
WASH3P	2.4194	2.20316	2.80998	2.58228	2.89556	1.82963
WASH5P	5.42173	5.26281	5.40171	5.37998	5.5923	4.59582
WASH6P	3.0882	2.84557	3.91485	4.0846	3.3742	2.61159
WASH7P	1.10293	0.790113	2.85675	3.12704	2.97876	1.74947
WASL	4.92113	4.76957	4.67833	4.77839	4.80627	4.67698
WBP1	3.49087	3.40806	3.71167	3.77118	3.65256	3.9431
WBP11	5.39795	5.57292	4.75892	5.30684	4.55088	4.74535
WBP1L	3.92571	2.77318	4.1926	3.68721	5.4198	4.06477
WBP2	4.1002	3.9935	3.606	4.17541	4.31624	4.58445
WBP4	2.8485	2.88773	2.55433	2.75679	3.14325	3.46322
WBP5	4.26958	4.29788	4.72786	5.16378	4.75597	5.06641
WBSCR16	4.57645	4.32912	4.29119	4.6097	4.88706	5.02799
WBSCR22	4.78712	4.9968	4.50351	4.89863	4.94246	5.2124
WDFY1	4.917	4.99862	5.37871	5.81894	5.48178	5.66397
WDFY2	4.64632	4.68499	5.4321	5.27162	5.01696	4.55655
WDFY3	5.63546	5.59753	5.89439	5.89611	6.47783	6.13265
WDHD1	4.59324	4.73499	4.0247	4.28133	4.35581	4.5694
WDPCP	3.14695	3.09762	2.42024	2.42271	2.49989	2.42777
WDR1	6.5383	6.69098	6.05579	6.61973	7.85976	7.77025
WDR11	5.31414	5.30442	5.29454	5.5376	5.73186	5.75884
WDR12	4.87749	4.89405	3.4525	4.05432	5.08738	5.43007
WDR13	3.74283	3.60324	2.8554	2.97599	2.71256	3.00754
WDR17	2.21383	2.33159	2.21737	2.12001	2.76176	2.79712
WDR18	3.14954	2.78509	2.40521	2.4799	2.67509	2.89692
WDR19	3.58517	3.29204	3.8277	3.74603	3.77431	3.83244
WDR20	3.8755	3.99577	2.77264	2.94646	3.40309	3.42515
WDR24	1.3612	0.904912	2.10034	2.09715	1.24366	1.36832
WDR25	1.54993	1.40499	0.436045	1.05667	1.25689	1.29333

WDR26	5.79049	5.79336	6.48181	6.3865	6.8344	6.84641
WDR27	3.73971	3.75268	4.15521	4.08962	4.10704	3.79512
WDR3	6.25762	6.38749	4.88791	5.27487	7.31089	7.57784
WDR33	6.04427	5.93508	5.30856	5.40538	5.99923	5.85981
WDR34	3.6727	3.34772	3.02594	3.33825	3.48689	4.13877
WDR35	4.89662	5.03799	3.49066	3.70975	5.09499	5.28339
WDR36	6.04979	6.32527	5.88321	6.26349	5.74481	5.83147
WDR37	3.30233	3.20958	2.51346	2.6225	3.46123	3.22402
WDR4	2.52252	2.30204	2.75312	2.68235	3.01918	2.46322
WDR41	4.14238	4.23181	4.7766	5.06822	4.61659	5.21359
WDR43	6.01558	6.09005	4.46032	4.96997	5.95047	6.08076
WDR44	2.23758	2.50871	3.70825	3.81746	4.45018	4.56123
WDR45	3.72861	3.60347	3.45112	3.81222	3.37007	3.64528
WDR45B	5.5295	5.34083	4.92721	5.26531	6.86922	6.6237
WDR45BP1	1.08703	1.22426	0.777763	1.04772	2.66831	2.08545
WDR46	5.23865	5.27904	4.3043	4.82996	5.89594	6.06022
WDR47	3.62284	3.95921	3.65882	4.08962	3.8245	4.08445
WDR48	5.04196	5.17377	3.9026	4.16496	4.94099	5.20765
WDR5	4.13372	4.06409	3.74624	3.88818	4.13461	3.87218
WDR53	2.07573	2.19538	2.75018	3.0686	2.97631	3.17593
WDR54	3.35077	3.37004	3.16742	3.5476	4.02268	3.99546
WDR55	5.01071	5.11719	5.07829	5.363	5.63847	5.53363
WDR59	4.09533	4.08982	3.89862	3.95187	3.45603	3.17593
WDR5B	2.60699	2.70207	2.72037	3.01271	2.57811	2.63759
WDR6	6.65008	6.77595	5.64268	5.84452	6.70754	6.53222
WDR60	3.56184	3.47525	4.46931	4.31638	3.12596	2.43796
WDR61	4.89935	4.80682	4.27475	4.64338	6.61825	7.26479
WDR62	3.48207	3.2514	3.66039	3.76461	3.4754	3.04148
WDR66	1.42206	1.33107	0.521191	1.07646	0.680243	1.02127
WDR7	4.7979	4.84478	4.38861	4.48907	3.44235	3.51105
WDR70	3.9014	3.86731	3.98445	4.16158	4.10703	4.11283
WDR72	2.79278	3.03101	-6.17309	-5.10387	-2.95876	-3.12051
WDR73	4.60564	4.71168	4.62297	4.83717	5.02944	4.93225
WDR74	4.50124	4.62038	3.50688	3.888	4.05852	4.4342
WDR75	4.95871	5.06315	4.23958	4.52235	5.11981	5.83243
WDR76	4.65847	4.63715	4.34688	4.48456	4.53813	4.42899
WDR77	4.82835	4.90988	3.9787	4.51301	5.13119	5.38935
WDR81	3.60805	3.32343	3.38858	3.35913	3.4725	3.57171
WDR82	6.07404	6.0581	5.40597	5.56532	6.43173	6.23391
WDR82P1	1.16275	1.33299	0.670532	0.589505	1.61761	1.277
WDR83	1.45429	1.24373	0.896807	1.13204	1.66963	1.71498
WDR83OS	4.81016	4.87858	4.2798	4.72793	5.60142	5.9515
WDR89	3.40814	3.51872	2.12028	2.30482	3.3241	3.95149
WDR90	2.65978	2.35192	2.79448	2.93285	2.41229	1.65964
WDR91	3.55832	3.29525	3.18331	3.02501	3.18951	2.74524
WDR92	2.33062	2.61105	2.60344	2.88491	2.39068	2.28702
WDSUB1	1.84784	1.73686	1.43857	1.34243	1.92091	1.97981
WDTC1	3.63407	3.37636	3.07151	3.10307	3.70492	3.38604
WDYHV1	2.11472	1.63522	2.09262	2.1904	2.60883	2.65524
WEE1	5.12189	5.09222	5.03261	4.91145	5.36965	5.33357
WFDC21P	-3.37567	-2.76144	2.88919	3.85288	-2.75246	-2.53597
WFS1	3.00483	2.64045	3.9939	4.17078	3.04013	3.05484

WHAMM	2.76241	2.59628	2.47113	2.77948	2.51213	2.06149
WHAMMP2	-6.66845	-5.88982	1.66534	1.69972	-2.00106	-3.17887
WHAMMP3	?	-7.18483	1.91141	1.84334	1.02901	0.134221
WHSC1	6.96288	7.09113	6.61473	6.72364	7.18074	6.86409
WHSC1L1	5.96679	5.95433	6.08273	6.15992	5.95687	5.71787
WI2-1896O14.1	-1.99982	-2.29967	-1.42761	-0.123714	0.0173091	1.92559
WIBG	2.66667	2.86359	2.41268	2.77076	2.73416	2.88173
WIPF1	4.2346	4.036	3.14515	3.14148	4.36896	4.31704
WIPF2	3.53813	3.54978	3.86223	4.04473	4.88416	4.49907
WIPI1	2.87219	2.56055	3.28806	3.95792	2.73405	3.58861
WIPI2	5.01086	4.72574	5.08618	5.21309	5.15986	5.01095
WISP1	-6.42303	-4.19658	4.13835	4.89187	-0.66265	-3.12811
WIZ	2.43022	2.33483	2.693	2.70817	2.96174	3.05816
WLS	5.6211	5.83474	2.01126	2.56361	5.56544	5.88049
WNK1	6.7863	6.80549	6.73344	6.75746	6.31538	6.11783
WNK3	4.04827	4.26167	-1.59814	-2.20128	2.32554	2.27058
WNK4	3.44318	3.27994	-0.99927	-1.2905	-2.43335	-4.01094
WNT2B	2.03406	2.29291	2.03783	2.13041	4.12074	3.32638
WNT3	1.44051	0.570657	1.70525	1.64609	0.558944	0.247587
WNT5A	5.73189	5.3074	7.38537	7.71791	4.78779	5.31841
WNT5B	0.0824028	-0.177294	4.41917	4.74291	3.48943	3.96218
WRAP53	3.24952	3.36536	3.3381	3.60407	3.5763	3.57688
WRAP73	3.8507	3.58154	2.72338	2.84828	4.29315	4.19264
WRB	3.33191	3.35211	4.10255	4.38906	3.12454	3.54317
WRN	3.58515	3.6286	3.98012	4.20632	3.959	4.28622
WRNIP1	4.41008	4.39747	4.56633	4.53502	4.67397	4.5257
WSB1	5.23202	5.17469	6.98812	6.72089	6.69573	5.67374
WSB2	6.2825	6.46209	3.85996	4.43729	6.0251	6.64947
WSCD1	-5.10914	-4.13839	-3.59683	-3.52222	4.84005	5.02799
WTAP	5.95303	5.43969	4.80853	5.22624	5.81415	5.92599
WTIP	1.11475	0.195474	2.09262	1.71138	-0.510264	-1.70626
WWC1	2.65408	2.86808	1.51695	2.26188	3.46381	3.54831
WWC2	5.16479	5.19507	5.27219	5.44338	5.55823	5.296
WWC3	2.86317	2.9122	4.62945	4.83807	1.45867	0.965773
WWOX	1.27825	1.03706	2.16964	2.31745	1.31701	1.54125
WWP1	3.66336	3.64727	4.12777	4.12912	3.87139	4.05844
WWP1P1	0.454997	0.356752	0.742308	0.836538	0.556199	0.522116
WWP2	5.5366	5.15828	3.60927	3.77229	4.74109	4.5281
WWTR1	6.87846	6.87381	5.90921	5.91702	6.78007	6.59033
XAB2	3.6251	3.44103	3.18935	3.23103	3.60296	3.53407
XAF1	-10.9684	-3.87578	3.91537	3.94111	4.20784	3.70506
XAGE1A	4.28424	4.15791	?	-5.51725	-7.63143	?
XAGE1B	3.77821	4.12436	?	-5.51725	-7.63143	?
XBP1	5.9953	5.82742	5.51519	5.81002	5.11323	5.11505
XIAP	4.10825	3.9455	4.99719	4.97678	6.10678	5.62961
XK	-2.9975	-3.65369	?	?	1.18523	1.25912
XKR4	-0.865939	-2.00285	-6.17309	-5.10387	3.67284	3.6856
XKR6	0.96632	0.831845	1.45314	1.21315	1.22848	0.597145
XKR8	1.89714	1.62465	-0.482896	-0.380732	2.68177	2.74038
XPA	1.93757	1.9035	2.02107	2.01014	1.78266	1.6062
XPC	4.96482	5.17659	3.98141	4.48572	3.8618	3.84808
XPNPEP1	4.56007	4.62792	4.71734	5.039	4.76228	4.7414

XPNPEP3	4.31931	4.45008	4.53129	4.88935	4.28575	4.18897
XPO1	7.59989	7.65965	7.24529	7.45011	7.73011	7.98481
XPO4	5.52941	5.57578	5.05904	5.34804	5.45802	5.39264
XPO5	5.97089	5.97115	5.08383	5.23424	6.12854	5.85357
XPO6	5.70646	5.65187	5.61127	5.89243	5.84411	5.66934
XPO7	5.66664	5.67641	5.13727	5.41521	5.36133	5.50943
XPOT	6.6725	6.83278	5.52726	5.99905	6.67421	6.96696
XPOTP1	3.19069	3.45792	2.08223	2.63312	3.20644	3.45224
XPR1	5.48671	5.65511	5.34541	5.61226	5.49354	5.57579
XRCC1	3.9953	4.07399	3.49743	3.8683	3.37832	3.17593
XRCC2	5.46144	5.49097	5.29725	5.386	5.23941	4.42642
XRCC3	3.77041	3.51171	2.89177	3.11108	3.28167	3.07589
XRCC4	2.26094	2.43501	3.06441	3.15092	2.62972	3.6107
XRCC5	8.0605	8.10464	7.7221	8.09814	8.22531	8.74772
XRCC6	7.53717	7.58509	7.20586	7.64435	7.39132	7.62315
XRCC6BP1	0.171399	0.479758	1.43123	1.8454	-0.0454657	1.1637
XRCC6P2	4.59816	4.79757	4.36388	4.74169	4.54753	4.58834
XRN1	4.9817	5.0083	6.21761	6.47778	4.97267	4.88667
XRN2	7.16045	7.16375	6.54031	6.91062	6.36442	6.65448
XRRA1	3.64556	3.71574	4.28667	4.40324	2.24915	1.93955
XX-FW83563B9.5	1.20281	0.846995	1.13775	1.17718	1.51648	1.20387
XXYLT1	3.57587	3.43046	4.00877	3.89118	4.0659	3.62421
XXbac-B461K10.4	4.54207	4.24304	2.21759	1.86274	3.35984	2.96833
XXbac-B476C20.13	1.6585	1.52019	-2.01303	-2.10822	-0.250761	-0.706491
XXbac-B476C20.9	0.708364	0.547677	-0.558047	-0.688718	-0.0893947	-0.144621
XXbac-B562F10.11	1.77542	1.86239	1.79749	1.58472	1.0641	0.707304
XXbac-BPG246D15	2.14813	0.660637	-1.15985	-0.639809	-1.85672	-0.857059
XXbac-BPG248L24.	-0.207288	-0.809694	1.94184	2.22506	0.493535	0.639566
XXbac-BPG252P9.9	3.59577	3.5477	3.20359	3.78275	3.59845	3.4806
XXbac-BPG283O16	2.34259	2.47086	3.14516	3.0586	3.52827	3.66397
XXbac-BPGBPG55(	0.951647	0.713995	1.3456	1.29229	0.630899	-0.0694897
XXyac-YRM2039.3	2.17699	2.39893	2.6746	2.57082	2.46571	2.31071
XYLB	3.40684	3.50868	0.623914	0.857026	-5.07051	?
XYLT1	?	-6.45019	2.89174	3.29483	?	-5.69922
XYLT2	4.68083	4.45452	5.01185	4.9017	4.85292	4.5076
YAE1D1	3.18968	3.36212	3.08566	3.21913	3.15523	3.63091
YAF2	3.94296	4.04989	3.9276	3.81301	2.91334	2.71115
YAP1	4.53273	4.48624	6.1384	6.39251	5.59587	5.33493
YARS	5.93227	6.028	5.38299	5.93978	6.25928	5.99155
YARS2	3.74179	3.76477	2.78227	2.81599	2.84154	3.21513
YBEY	0.159022	0.305445	1.59787	1.56363	1.43248	1.48315
YBX1	7.45653	7.47747	7.23211	7.62976	8.10149	8.10453
YBX1P1	6.31241	6.23393	6.05775	6.4508	6.80241	6.84038
YBX1P10	5.55159	5.55863	5.28408	5.65055	6.14514	6.20684
YBX1P2	4.04931	4.10125	3.83815	4.11901	4.68531	4.54783
YBX3	6.25208	6.29818	5.27482	5.44839	5.35161	5.35665
YDJC	2.44559	2.31196	1.32432	1.87419	2.0879	2.54122
YEATS2	5.19301	5.19242	5.83291	5.96868	6.64282	5.96642
YEATS2-AS1	1.15398	1.48326	2.25983	2.18066	2.53701	1.51631
YEATS4	3.58053	3.68563	1.37111	1.86848	2.93961	3.49051
YES1	5.07151	4.94024	5.43598	5.68193	6.53805	6.47841
YES1P1	-0.460413	-0.160947	0.388686	0.340517	1.32961	1.30204

YIF1A	3.67123	3.55539	3.36267	3.93763	3.2116	3.95149
YIF1B	2.95377	2.80161	3.34786	3.81895	3.39226	4.16211
YIPF1	3.10505	3.01289	3.3768	3.54581	3.73736	3.92265
YIPF2	3.26018	3.13917	3.19682	3.54121	4.02726	4.43306
YIPF3	4.43341	4.52441	4.35369	4.89539	5.33156	5.42321
YIPF4	5.25512	5.37949	4.22268	4.49849	5.19571	5.47069
YIPF5	4.93302	4.96026	5.4787	5.84715	5.40242	5.62028
YIPF6	5.13656	5.3581	5.15799	5.67376	5.8492	6.3455
YJEFN3	-0.113528	-0.525299	0.511428	-0.0156352	0.81189	-0.682796
YKT6	5.69273	5.7836	5.65409	6.15239	6.41798	6.56121
YLPM1	5.5602	5.50209	5.06083	4.89725	5.09397	4.81158
YME1L1	6.30913	6.4581	5.87292	6.22919	6.72238	7.02125
YOD1	5.03231	4.97413	4.29004	4.39175	5.17726	4.91519
YPEL2	3.80275	3.41368	2.18282	2.23549	0.740643	0.401903
YPEL3	1.19257	0.355118	0.636738	0.333515	0.92024	0.428496
YPEL4	-0.865939	-1.14061	2.27592	1.86848	-0.756662	-1.38762
YPEL5	3.68678	3.66386	3.97382	4.37297	4.15747	4.28197
YRDC	4.08457	4.22723	2.40557	2.84665	4.73553	5.07224
YTHDC1	5.22426	5.19358	4.78226	4.92528	5.54853	5.11665
YTHDC2	5.12587	5.1569	4.8242	4.75844	5.24543	4.99891
YTHDF1	5.42148	5.42513	4.73011	5.10305	5.29896	5.02545
YTHDF2	6.1083	6.1309	5.41795	5.70978	5.91321	5.95608
YTHDF3	5.24049	5.29704	5.6214	5.70734	5.34109	5.2777
YWHAB	7.51434	7.34592	7.29047	7.47003	7.41483	7.33082
YWHABP2	1.13721	1.20254	0.783331	1.02093	1.04711	0.494395
YWHAE	7.07226	6.96168	7.37192	7.65896	8.03827	8.26506
YWHAEP1	-0.421059	0.0873949	0.0450934	0.418503	0.604603	0.832574
YWHAEP5	1.4047	1.39995	1.97192	2.27984	2.7312	2.28311
YWHAG	7.26181	7.2561	7.22413	7.61108	7.86892	7.84185
YWHAH	5.65383	5.69669	5.00754	5.41131	5.6551	5.52209
YWHAQ	7.33732	7.42913	6.89347	7.29626	8.20207	8.45284
YWHAZ	8.40249	8.42555	8.2913	8.65566	8.41153	8.36329
YWHAZP2	2.76813	2.86809	2.73859	3.17876	2.8459	2.75206
YWHAZP3	4.81365	4.92126	4.78474	5.09202	4.7108	4.90971
YWHAZP4	1.96837	1.85396	1.81395	2.07681	1.63768	1.93589
YWHAZP5	0.25141	0.935535	0.587528	1.01143	0.777014	0.709838
YY1	5.9036	5.95762	5.22374	5.48315	5.85339	5.84643
YY1AP1	4.8649	4.77114	4.47026	4.70274	4.95975	4.73183
YY2	0.541683	0.547302	1.55053	1.2369	0.660719	-0.0239546
Y_RNA	0.994464	1.23033	0.759062	0.973553	1.51223	0.993783
ZACN	2.339	1.83834	1.94187	2.1433	2.31971	1.32027
ZADH2	5.0335	5.42665	3.57136	4.04473	3.09458	3.76089
ZBED1	3.66724	3.53432	4.15187	4.37196	3.72231	3.60163
ZBED3	1.60359	1.44806	0.804582	0.964369	1.78209	1.31009
ZBED3-AS1	0.367997	0.395534	-0.349094	-0.260942	0.607749	-0.13007
ZBED4	5.18508	5.05383	5.01246	4.95726	4.45277	3.82076
ZBED5	4.43144	4.46922	4.40774	4.52579	4.01814	4.32945
ZBED5-AS1	-0.0541212	-0.527101	0.96902	1.19692	-0.611892	-0.799586
ZBED6	6.38569	6.32832	6.03098	6.11294	5.84195	5.66988
ZBED8	1.96983	2.10703	2.8305	2.7677	2.00859	2.3377
ZBED9	-0.094911	-0.13631	1.55784	1.67968	2.32682	3.18439
ZBTB1	4.44236	4.51154	4.30411	4.30797	4.47339	4.60161

ZBTB10	4.82201	4.87421	4.00753	3.76769	4.32126	3.84018
ZBTB11	4.93585	5.11104	4.70094	4.93835	4.78606	4.9199
ZBTB11-AS1	0.920949	0.863463	0.669635	0.998809	0.332725	0.200203
ZBTB12	0.855575	0.503023	0.558023	0.749131	0.246688	0.334168
ZBTB14	2.65408	2.66645	2.91188	2.97333	2.97813	3.23287
ZBTB17	2.29246	2.15923	2.85266	3.0611	3.03318	2.94075
ZBTB18	4.1828	4.19359	4.0821	4.12957	4.51212	4.60277
ZBTB2	3.46959	3.57607	3.24902	3.60198	4.16782	4.42771
ZBTB20	2.73663	2.33483	4.6581	4.14142	4.69316	3.92082
ZBTB21	4.2168	4.3928	4.37201	4.81384	3.90669	4.02799
ZBTB22	2.32053	2.33158	2.27182	2.60543	2.96357	2.86694
ZBTB24	3.55739	3.7039	3.19656	3.35537	4.18194	3.95658
ZBTB25	3.7549	3.95578	3.15067	3.1812	3.59569	3.17425
ZBTB26	3.15734	3.27186	2.88258	2.97599	2.75966	2.77307
ZBTB3	1.40425	1.29209	1.15638	1.27492	1.13416	1.29333
ZBTB33	3.79477	3.898	4.32723	4.62503	4.92133	5.26049
ZBTB34	3.8097	3.73313	3.27796	3.45341	3.24658	2.95507
ZBTB37	5.31457	5.41289	4.59043	4.38302	5.48339	5.40702
ZBTB38	6.87915	7.06033	6.3073	6.67883	6.38527	6.46101
ZBTB39	3.08883	3.24106	3.3653	3.47989	2.97813	2.66833
ZBTB4	4.14954	3.86919	4.99514	5.04788	5.47985	5.16979
ZBTB40	5.20779	5.01792	5.26924	5.20177	5.15466	4.58329
ZBTB41	4.14955	4.83232	4.07268	4.78522	4.44099	5.4831
ZBTB42	1.02889	0.676782	0.191626	0.176693	0.246688	1.00759
ZBTB43	3.92936	3.95394	3.83884	4.032	4.03924	3.66615
ZBTB44	5.3528	5.35733	4.33316	4.35468	5.14017	5.04399
ZBTB45	0.595618	0.292146	0.759062	0.698601	0.661695	0.443129
ZBTB46	2.36702	1.98012	3.4382	2.93808	1.10733	0.443129
ZBTB47	2.20482	2.05288	0.82777	1.35882	1.8195	2.70693
ZBTB48	3.30655	3.27862	2.82491	2.83076	3.6053	3.42515
ZBTB49	1.9303	2.02501	1.22592	1.37503	1.97271	1.73626
ZBTB5	3.6986	3.85456	3.50823	3.75226	4.87204	4.84306
ZBTB6	3.50548	3.72574	2.92239	3.15092	3.73201	4.11187
ZBTB7A	2.7251	2.73683	2.49948	2.53864	2.92278	2.9515
ZBTB7B	3.51281	3.25483	3.42381	3.62926	4.73843	4.54003
ZBTB8A	1.95541	2.08727	1.45086	1.89823	1.89112	2.08411
ZBTB8B	1.33811	1.16701	-0.686025	-1.10677	-0.45167	-0.669208
ZBTB8OS	2.95203	2.94664	2.72123	2.96645	3.82041	4.23989
ZBTB8OSP2	0.538836	0.498567	-0.0235484	0.782618	1.37552	2.11786
ZBTB9	2.58096	2.69635	2.04893	2.13094	3.53745	3.66479
ZC2HC1A	1.77595	1.98399	3.12937	2.97332	2.14061	1.87767
ZC3H10	3.31405	3.21415	2.66436	2.67961	2.47743	2.50386
ZC3H11A	7.50508	7.53521	7.04437	7.18746	7.26297	7.03419
ZC3H11B	3.26462	3.33418	2.73202	2.97881	3.1051	2.84136
ZC3H12A	3.7345	3.62594	3.45673	3.97465	2.36761	2.22404
ZC3H12B	-0.113974	-0.195907	0.53081	0.57776	0.766027	0.832509
ZC3H12C	3.70179	4.0139	4.3361	5.10366	3.79399	3.971
ZC3H13	5.9314	5.89142	5.72898	5.75921	5.74241	5.32533
ZC3H14	5.76181	5.78934	4.61955	4.90001	5.20805	5.35496
ZC3H15	5.47927	5.54066	4.4889	4.96529	5.93331	6.50853
ZC3H18	4.58401	4.47158	4.12595	4.16146	3.72014	3.61971
ZC3H3	1.69327	1.44408	2.11112	2.23104	1.48439	1.73626

ZC3H4	4.35007	4.09271	3.84852	3.71771	3.92651	3.81292
ZC3H6	3.13223	2.59786	3.96495	3.77992	3.0608	2.26485
ZC3H7A	5.0558	4.90402	4.7398	4.82264	4.70381	4.44809
ZC3H7B	4.99701	5.0305	5.37728	5.57328	5.66104	5.5856
ZC3H8	3.29952	3.30203	2.54441	2.44193	3.00325	2.98329
ZC3HAV1	5.40223	5.34731	5.67288	5.76577	6.16263	5.83774
ZC3HAV1L	1.60474	1.9252	?	-5.10387	-6.64581	?
ZC3HC1	3.81365	3.78745	3.26149	3.43614	3.68176	3.78313
ZC4H2	-3.69722	-3.00228	2.89063	2.99184	2.4351	2.47817
ZCCHC10	2.94475	3.00266	3.09028	3.29844	3.6582	3.98148
ZCCHC11	5.52671	5.56747	5.82839	5.71014	5.3295	5.20016
ZCCHC14	4.37573	4.26764	4.51084	4.32891	3.92373	3.6398
ZCCHC17	5.00781	4.85447	3.52472	3.93451	4.34015	4.71148
ZCCHC2	3.86602	3.86132	2.74426	3.38303	2.13413	2.28199
ZCCHC24	4.28861	4.20702	4.83431	5.14895	1.68713	1.9028
ZCCHC3	3.4392	3.3103	3.87311	3.91493	3.14866	2.80899
ZCCHC4	2.60243	2.58701	2.31634	2.38304	3.23299	3.24748
ZCCHC5	?	?	-1.42826	-1.15432	0.529601	1.17598
ZCCHC6	4.61438	4.67158	4.30532	4.50034	4.75911	4.75067
ZCCHC7	3.5828	3.65998	3.25735	3.38301	3.94608	4.14815
ZCCHC8	4.37237	4.33805	3.79089	3.92459	4.22462	4.07136
ZCCHC9	3.78171	3.75268	3.925	4.08961	4.35579	4.33631
ZCRB1	3.87589	3.86107	3.79171	3.97218	3.73655	4.17732
ZDBF2	4.76546	4.64306	4.89195	4.98921	5.98016	5.87589
ZDHHC12	2.49815	2.19539	2.34787	2.48738	2.48945	2.83245
ZDHHC13	3.55359	3.58837	3.35564	3.65438	3.45343	4.25763
ZDHHC14	2.63855	2.79691	1.71133	1.79809	1.90957	1.72795
ZDHHC16	3.82153	4.02197	4.13953	4.568	4.16305	4.55889
ZDHHC17	4.64577	4.75025	4.61656	4.74525	4.42846	4.38471
ZDHHC18	4.21006	4.24278	3.43486	3.83369	3.9979	3.94253
ZDHHC2	-3.5275	-3.29152	4.02715	4.26512	5.32694	5.72739
ZDHHC20	6.4391	6.48316	6.01023	6.33052	5.51725	5.69483
ZDHHC20P4	1.95366	2.1759	1.61918	2.1337	1.24388	1.69176
ZDHHC21	4.67106	4.89305	1.05494	1.15801	6.01762	5.57694
ZDHHC23	2.97158	3.33158	1.57806	2.1152	2.98895	3.47319
ZDHHC24	2.61404	2.47184	2.81754	3.17014	2.55418	2.87252
ZDHHC3	5.00912	5.08067	4.52297	4.97398	5.17889	5.18126
ZDHHC4	4.04911	3.71332	3.24692	3.36488	4.58934	4.92536
ZDHHC5	5.53841	5.44819	5.59248	5.78559	5.8943	6.0084
ZDHHC6	4.30413	4.52156	3.67816	4.04632	3.97282	4.55184
ZDHHC7	5.10867	5.25182	5.33954	5.76345	4.6616	4.38867
ZDHHC8	2.90084	2.51731	2.86088	2.84246	2.05396	1.64205
ZDHHC9	3.04239	2.98631	4.03927	4.00491	4.98893	4.70901
ZEB1	4.78938	5.22405	6.00353	6.10348	4.94437	4.89274
ZEB1-AS1	0.928548	0.776601	1.81623	1.60943	0.466152	0.0875715
ZEB2	6.53465	6.2694	3.6662	3.65024	6.00158	5.72487
ZER1	2.94658	2.67672	3.39768	3.28778	3.7512	3.45063
ZFAND1	3.45194	3.47085	3.39391	3.76615	4.21159	4.79313
ZFAND2A	1.43539	1.58704	2.56132	3.01788	1.8711	2.2475
ZFAND2B	2.31216	2.28536	2.91977	3.05608	3.73522	3.90062
ZFAND3	3.53094	3.35094	3.79519	4.01528	4.80932	4.40832
ZFAND4	0.870842	0.840999	-3.85951	-4.10635	0.362161	0.522196

ZFAND5	5.68564	5.68779	6.43107	6.61177	6.21049	6.13006
ZFAND6	4.86313	4.8036	4.82835	4.9759	5.03298	5.16976
ZFAS1	4.89798	4.88643	4.45121	4.84317	5.34144	5.95994
ZFAT	3.26568	3.29351	2.67004	2.75821	3.26394	3.23957
ZFC3H1	5.87804	5.90943	5.60449	5.50883	5.12673	4.99545
ZFHX3	4.50876	4.41051	5.15526	4.86248	4.23076	3.91545
ZFHX4	4.6279	4.60256	4.22692	3.99183	6.60072	6.47657
ZFP1	3.63072	3.58836	3.31233	3.46103	2.54915	2.80505
ZFP14	3.03902	3.0994	2.68993	2.6157	2.05739	2.00067
ZFP28	2.41728	2.55953	2.47113	2.45724	3.03666	2.79314
ZFP3	-0.569597	-0.27286	-0.935332	-0.682344	2.80118	2.61071
ZFP30	3.38758	3.38408	3.44026	3.31809	3.1363	3.19215
ZFP36	0.470938	-0.122851	1.42385	1.54942	0.924716	0.90806
ZFP36L1	4.0005	3.67158	5.38729	5.26134	4.23224	4.03475
ZFP36L2	3.70287	3.62196	1.20884	1.18584	3.37448	3.82467
ZFP37	1.95558	1.79457	2.11571	1.94105	1.73632	2.21217
ZFP41	2.07254	2.00777	2.8953	2.94727	2.43491	1.96037
ZFP62	3.61946	3.58972	4.09841	4.18465	4.26226	4.01439
ZFP64	3.14642	3.05088	2.40145	2.64941	3.03144	2.5317
ZFP69	1.00834	1.06077	1.15638	1.49854	-3.07415	-4.11926
ZFP69B	2.17746	2.88145	0.770744	1.02051	0.932205	1.03481
ZFP82	3.32744	3.53292	2.11571	2.12481	0.643551	0.443129
ZFP90	3.26093	3.23933	4.3768	4.31114	4.28073	4.53645
ZFP91	5.99728	6.29764	6.01306	6.48343	6.16247	6.37865
ZFPL1	2.81179	2.44547	2.05325	2.43603	2.31941	2.22973
ZFPM2	3.45826	3.57058	3.50122	2.92185	2.42451	1.65087
ZFPM2-AS1	3.01001	2.80629	3.65252	3.35061	3.39226	3.34314
ZFR	6.37438	6.39765	6.06084	6.24512	6.79633	6.73775
ZFX	5.13063	5.06169	5.56706	5.56256	5.25368	4.96393
ZFY	-6.68174	-5.45492	4.11709	4.21233	-6.64581	?
ZFYVE1	3.05247	2.71956	2.59127	2.33413	3.40847	2.70269
ZFYVE16	5.60383	5.5685	5.98482	6.11335	5.9736	6.20316
ZFYVE19	3.2903	3.29146	3.02005	3.28261	2.9924	3.17899
ZFYVE21	3.11471	2.68182	1.93024	1.9191	2.68843	2.2763
ZFYVE26	4.71981	4.73006	3.91845	4.07852	4.31482	4.60616
ZFYVE27	3.97687	4.08405	3.68991	4.16613	3.82049	3.9135
ZFYVE28	1.6408	1.44408	0.904002	1.42259	0.670686	0.633217
ZFYVE9	5.05356	4.99108	4.70002	4.85661	4.76226	4.70202
ZGPAT	3.93789	3.80228	3.36894	3.58447	3.73403	3.33767
ZGRF1	4.78422	4.90837	4.88524	5.01464	4.05651	4.27627
ZHX1	3.45314	3.46391	5.18863	5.45939	5.54233	5.9606
ZHX1-C8orf76	0.825818	0.708955	1.42741	1.42255	1.29972	1.39917
ZHX2	3.00655	2.77078	-0.11729	-0.860651	3.24958	2.73209
ZHX3	5.8479	5.60544	5.6305	5.57591	5.01199	4.48545
ZIC1	-1.61119	-1.55554	2.12938	1.8912	1.40043	0.522196
ZIC2	-5.69162	?	1.45314	1.77994	0.0122397	-0.4586
ZIC4	-4.11163	?	1.02599	1.12005	-0.267833	-1.24695
ZIC5	?	?	0.460428	0.786061	0.69732	-0.121607
ZIK1	3.13698	3.34772	2.43497	2.60886	3.97722	3.61522
ZKSCAN1	6.2359	6.37018	6.33239	6.38906	6.26706	6.15707
ZKSCAN2	3.11471	2.86583	3.23644	3.05357	3.23905	2.75682
ZKSCAN3	1.34869	0.987088	2.79877	3.01936	2.96039	2.96974

ZKSCAN4	1.57889	1.69788	1.8513	1.98622	2.22462	2.40632
ZKSCAN5	4.45954	4.37635	4.74573	4.9532	4.65765	4.7414
ZKSCAN7	-0.543543	-0.604659	2.10296	1.91921	-0.957246	-1.52941
ZKSCAN8	4.67868	4.74232	4.81648	4.85551	5.18792	4.99544
ZMAT2	5.03502	4.97836	5.22958	5.66744	5.39757	5.42013
ZMAT3	5.15226	5.17332	4.40237	4.7507	4.66274	4.7981
ZMAT5	1.94832	1.78414	2.01935	2.41626	1.82099	2.21691
ZMIZ1	4.59609	4.3437	4.7959	4.57153	4.21005	4.45944
ZMIZ2	3.51037	3.25312	3.94576	3.9586	3.55643	3.12618
ZMPSTE24	5.79151	6.1967	5.25993	5.91352	5.80244	6.36641
ZMYM1	4.00913	4.20603	4.3498	4.6097	4.50961	4.64422
ZMYM2	5.67622	5.58053	6.40094	6.4218	6.01409	5.78862
ZMYM3	4.43085	4.35253	4.89661	4.87985	4.91191	4.72165
ZMYM4	6.04689	5.97657	5.82717	5.95453	5.96958	6.14455
ZMYM5	3.14641	3.16469	2.72638	2.93284	2.72772	2.60618
ZMYM6	3.15899	3.32513	3.27807	3.54755	3.52549	3.51986
ZMYM6NB	1.69608	1.90862	2.20721	2.1643	1.9709	2.8419
ZMYND11	5.3205	5.26167	4.31432	4.52416	5.39259	5.34312
ZMYND19	3.46959	3.46642	2.98139	3.28992	3.26302	3.37544
ZMYND8	6.13272	5.78032	6.25214	5.88625	5.30076	4.78168
ZNF10	2.56302	2.70458	0.882627	0.749225	2.26452	2.14505
ZNF100	3.37433	3.4255	3.15807	2.98448	2.44069	1.95152
ZNF101	3.1417	3.1013	2.16964	2.34651	3.13411	3.40443
ZNF101P2	0.490837	0.479758	-0.375995	-0.649181	-0.0752099	0.293394
ZNF106	6.88244	6.97955	6.50843	6.80797	6.51911	6.46006
ZNF107	3.94747	3.78315	4.09491	3.94645	3.71037	3.25329
ZNF112	3.61492	3.66514	3.0186	3.21085	3.40039	3.48064
ZNF117	1.65386	1.51841	4.5357	4.02886	1.79992	1.19187
ZNF12	4.74594	4.70205	5.04828	5.11276	5.03883	4.98153
ZNF121	6.29666	6.23824	4.57552	4.70975	6.55032	6.16171
ZNF124	3.6716	3.56091	3.25111	3.23769	3.50711	4.25762
ZNF131	4.21305	4.45825	4.41545	4.49015	5.08494	5.17892
ZNF132	-0.129081	-0.790185	2.37873	2.6225	2.6251	2.61071
ZNF133	4.5213	4.51084	4.04169	4.12599	3.36344	3.38869
ZNF134	3.61267	3.81211	3.39957	3.56183	4.35299	4.22253
ZNF136	2.68894	2.61932	1.68687	1.53507	2.19769	2.08121
ZNF138	2.84589	2.82025	2.9939	2.92185	2.684	3.05816
ZNF14	2.18358	2.13709	1.70525	1.69856	1.9359	2.02802
ZNF140	3.10226	3.32907	0.92184	0.680027	2.86929	3.09764
ZNF141	3.03224	3.08983	-1.42826	-1.35358	2.41117	2.58792
ZNF142	3.8714	3.81465	3.63251	3.70028	4.01513	3.70188
ZNF143	3.34945	3.30212	3.49771	3.59853	3.5721	3.82272
ZNF146	6.21299	6.26071	5.30561	5.45847	6.58261	6.70679
ZNF148	5.52974	5.40635	5.52124	5.70254	5.59469	5.57173
ZNF154	1.40948	1.83639	1.63029	1.26626	1.5829	0.752791
ZNF155	0.804574	1.02048	1.85244	1.72972	2.86505	2.77691
ZNF16	1.9303	2.02099	2.10652	2.08102	2.16943	2.43796
ZNF160	3.89244	3.75994	3.96749	3.96932	4.67172	4.35667
ZNF169	1.52013	1.60865	1.30837	1.41477	1.62513	0.863209
ZNF17	2.58633	2.77317	2.53759	2.75226	3.12923	3.19717
ZNF174	2.14956	2.15923	1.96116	2.27489	2.0332	2.0415
ZNF175	3.18051	3.16469	3.26563	3.47614	3.58643	3.6107

ZNF18	1.41317	1.57805	2.07829	2.10497	2.02685	2.5782
ZNF180	3.4494	3.57333	3.00136	3.11518	3.48052	3.80109
ZNF181	2.39193	2.54488	2.75249	2.9413	2.8435	3.6322
ZNF182	3.03224	2.85458	2.03082	2.17196	2.05052	2.19417
ZNF184	3.26526	3.41213	2.42751	2.76462	3.08452	3.91715
ZNF185	1.59558	1.20963	-1.32519	-1.20162	-0.729349	-0.799584
ZNF189	3.42892	3.36846	3.75605	3.85262	3.2778	3.54122
ZNF19	0.626645	0.523773	1.67151	1.51793	1.27856	1.12271
ZNF192P1	-0.791952	-0.333394	1.25947	0.773887	0.606556	0.50283
ZNF195	4.02799	3.95712	3.80373	3.72722	3.64575	3.5293
ZNF197	4.31447	4.45854	4.28617	4.25542	4.38817	4.18202
ZNF2	2.18052	2.09942	1.88259	1.93012	2.30837	2.18812
ZNF20	0.928753	1.08415	0.793247	0.54209	0.586894	0.565188
ZNF200	3.17378	3.29455	2.89246	2.95953	2.92466	2.81686
ZNF202	3.43791	3.53856	2.8554	3.0686	3.01212	2.74862
ZNF204P	1.48086	1.50298	-1.01332	-1.35358	-4.65668	-3.38329
ZNF205	0.662588	0.3097	0.459589	0.850591	1.21438	1.2607
ZNF207	7.36701	7.41612	7.47678	7.5772	7.57082	7.41357
ZNF208	3.68345	3.6078	-3.59683	-4.51838	-6.64581	-5.69922
ZNF211	2.5536	2.52301	2.80801	2.99708	3.30548	3.29046
ZNF212	2.77766	2.73683	2.63027	2.58467	2.75755	2.63759
ZNF213	1.00142	0.785149	0.636738	0.786061	1.1471	1.02127
ZNF213-AS1	1.47588	1.46201	1.83887	2.01532	1.41654	1.08885
ZNF215	2.93936	3.06072	-6.17309	-5.10387	-6.64581 ?	
ZNF217	6.07119	6.07083	6.88544	6.78795	6.29307	5.82882
ZNF219	1.66808	1.30004	0.408907	0.272823	0.9837	0.84754
ZNF22	3.97878	4.08064	3.82178	4.03671	4.68452	5.25757
ZNF221	2.63185	2.78034	2.00632	1.88555	2.58288	1.67702
ZNF222	1.65121	1.67091	1.19801	1.32915	1.74313	1.93874
ZNF223	0.923035	1.21226	0.742619	0.524435	1.39134	0.740392
ZNF224	3.85602	3.78732	4.15941	4.07873	4.65984	4.04656
ZNF225	2.63856	2.77317	2.61415	2.56361	3.25408	2.99373
ZNF226	3.79477	3.84777	3.8249	3.63264	4.71418	4.53526
ZNF227	4.14727	4.24926	3.50164	3.61733	4.68554	4.68562
ZNF229	3.96357	3.96759	-0.825729	-1.02108	-2.07475	-3.12051
ZNF23	2.60952	2.56856	3.29326	3.49738	3.49093	3.0848
ZNF230	2.54411	2.28872	1.80519	1.76772	2.37862	2.33769
ZNF232	3.25199	3.32518	2.34846	2.53579	2.85304	3.12599
ZNF233	1.10187	1.33161	0.802367	0.414828	1.02625	0.702755
ZNF234	3.66506	3.81561	3.15856	3.19723	4.2139	3.86883
ZNF235	3.25179	3.45076	3.25694	3.15083	3.19341	2.77222
ZNF236	4.5219	4.39591	4.032	3.73979	2.85145	2.42258
ZNF239	1.43538	1.29875	1.20023	0.736735	3.20233	2.85554
ZNF24	6.08736	6.24842	5.37632	5.59855	5.49386	5.7886
ZNF248	3.39104	3.34612	3.70978	3.5724	3.47026	3.22993
ZNF25	2.5536	2.77557	2.31235	2.47239	2.48181	2.96925
ZNF250	1.99094	2.08792	3.24693	3.29632	2.31412	2.10066
ZNF251	2.53455	2.65351	3.58962	3.63095	3.64461	3.53169
ZNF252P	3.8949	3.95427	4.25902	4.40739	5.43927	5.79293
ZNF253	2.77765	2.57883	1.78229	1.64609	-1.26766	-1.79933
ZNF254	2.77148	2.83754	3.6506	3.44158	3.79811	3.71881
ZNF256	2.30656	2.45305	1.28413	1.28354	2.02971	1.89323

ZNF257	2.69968	2.85231	?	-4.52056	-6.64581	?
ZNF259P1	-0.0494525	0.123375	-0.284425	0.499588	1.26277	1.33874
ZNF26	4.23976	4.45973	3.32428	3.24211	4.04445	3.81292
ZNF260	4.2033	4.30781	3.843	3.83661	3.86023	3.73208
ZNF263	4.20271	4.22655	3.97009	4.10283	4.16651	4.10534
ZNF264	4.06745	3.93967	4.2511	4.32475	5.2027	4.72059
ZNF266	4.12667	3.90346	3.74277	3.68883	3.82652	3.31288
ZNF267	4.43982	4.46641	3.85539	4.12358	3.57811	4.04817
ZNF268	4.49135	4.48405	2.40895	2.1152	4.10124	4.18355
ZNF271P	3.81563	3.72574	3.88121	3.95052	3.56249	3.79512
ZNF273	2.49076	2.43501	2.62706	2.44193	1.91405	1.70271
ZNF274	2.50795	2.3413	2.44948	2.44193	3.89139	3.48064
ZNF275	2.92663	2.88811	3.06678	3.52961	3.45992	3.31287
ZNF276	3.17057	2.93011	3.22804	3.42399	2.96289	2.65294
ZNF277	3.30534	3.28505	2.59303	3.00275	3.09722	3.69203
ZNF28	4.03378	4.13465	2.70068	2.80029	3.38056	3.51522
ZNF280B	?	?	-0.69145	-1.30113	1.35654	0.443129
ZNF280C	1.99791	2.01291	3.08795	3.15561	3.86619	3.88947
ZNF280D	4.0936	4.25772	4.06769	3.94645	4.57652	4.92991
ZNF281	5.54762	5.45306	5.01062	5.11093	5.55856	5.18809
ZNF282	3.87078	3.73067	3.33611	3.49294	3.83853	3.88946
ZNF283	3.68462	3.96132	2.90395	2.83662	4.04098	4.18353
ZNF284	2.93019	3.06239	1.97684	2.00671	3.61749	3.02679
ZNF285	1.7716	1.69004	1.21518	0.867359	0.613211	0.502025
ZNF286A	4.99361	4.84515	4.947	4.97001	5.12305	5.19134
ZNF286B	2.00425	1.8661	2.58813	2.5635	2.94903	2.74334
ZNF287	1.51041	1.45008	2.31634	2.29207	1.80732	2.10709
ZNF292	6.13693	6.07823	6.09463	5.92528	6.74081	6.66151
ZNF3	4.73414	4.72653	4.74604	4.7104	4.8033	4.64817
ZNF30	1.76038	1.84096	1.91979	1.92462	1.82755	1.80111
ZNF300	4.31076	4.34531	3.98764	4.02432	3.48307	3.79512
ZNF302	4.11961	4.03089	4.64349	4.59364	4.27502	4.31534
ZNF304	3.44371	3.4156	3.28418	3.52662	3.51319	3.5641
ZNF316	3.37507	3.23412	3.27796	3.32372	4.00857	3.23287
ZNF317	4.07405	3.98837	3.65883	3.86988	4.73842	4.42127
ZNF318	4.52972	4.49499	4.70976	4.74136	4.78211	4.42128
ZNF319	1.02208	0.804012	2.22554	2.8968	2.33688	2.48312
ZNF32	2.63045	2.50169	2.06329	2.18003	2.8119	3.42822
ZNF320	4.24827	4.19587	3.15409	3.21584	3.16796	2.67541
ZNF322	3.8126	3.8695	4.0036	4.05333	4.49926	4.88138
ZNF324	3.18562	3.22076	2.59152	2.8482	3.28354	3.33701
ZNF324B	1.91804	1.90786	1.72919	1.71465	2.45876	2.50444
ZNF326	5.12945	5.08114	5.21253	5.06546	4.90668	4.2848
ZNF329	2.12112	1.94657	0.292311	0.383121	?	?
ZNF330	3.65737	3.82024	2.59785	3.07357	3.56731	3.67482
ZNF331	3.85263	3.81675	3.44766	3.26404	1.90577	1.18815
ZNF333	2.13066	1.9925	2.04532	1.88118	3.25855	2.75682
ZNF334	0.878411	0.479758	3.35176	3.11276	?	?
ZNF335	4.46958	4.33481	3.8866	3.88269	3.6251	3.17287
ZNF337	4.8745	4.86216	3.96871	3.84823	4.10868	3.53789
ZNF337-AS1	3.43972	2.99964	3.24912	2.83377	1.71668	1.37737
ZNF33A	4.24141	4.24926	4.081	3.86373	4.49305	4.33835

ZNF33B	3.64495	3.77017	3.2236	3.04673	3.98561	3.89412
ZNF34	0.870842	1.00482	0.925065	1.06115	1.40582	1.43288
ZNF341	0.847885	0.727284	0.641241	0.49591	0.394821	0.277616
ZNF343	4.16464	4.23932	4.1213	4.26621	3.45977	3.36743
ZNF345	0.510466	0.850103	1.04535	1.01017	0.529601	0.33778
ZNF346	2.69753	2.68945	3.57636	3.74915	3.59708	3.20018
ZNF347	4.30513	4.40132	3.63169	3.69369	3.15827	3.07137
ZNF35	3.84781	4.01287	2.78803	3.05608	3.88658	4.18202
ZNF350	1.70609	1.47971	1.12031	1.10068	2.0332	1.95152
ZNF354A	3.43524	3.43562	3.15872	3.10088	1.7443	1.68424
ZNF354B	2.69771	2.75535	3.57457	3.52943	2.38991	2.18911
ZNF354C	3.64736	3.55949	2.36424	2.52733	4.0421	3.42626
ZNF358	2.28078	2.02886	2.29153	2.29982	2.66375	2.74844
ZNF362	2.1558	2.48846	2.14741	2.27057	1.66615	1.99375
ZNF365	2.1083	2.58156	1.20023	2.24874	3.72554	4.18202
ZNF367	3.94026	3.57196	2.88257	2.98129	4.11776	3.79114
ZNF37A	4.81199	4.88033	4.26389	4.31848	4.5832	4.08118
ZNF37BP	4.99066	4.95078	4.39359	4.33825	3.74633	3.00754
ZNF382	2.34381	2.19558	0.650296	0.139221	2.37333	1.66836
ZNF383	2.03238	2.01567	1.38893	1.45216	2.27296	2.05264
ZNF384	4.3296	4.04057	3.72223	3.91983	3.93349	3.35688
ZNF385A	2.3316	2.18103	-0.539469	-0.649181	-1.61549	-1.31405
ZNF385D	-1.08408	-1.41808	2.01126	2.27921	1.85542	1.92993
ZNF394	3.79355	3.91211	3.91676	4.09294	3.85838	3.84402
ZNF395	4.11338	4.15243	4.69444	4.8803	4.34137	3.56712
ZNF397	3.77143	3.75583	3.68651	3.65855	2.99853	2.59569
ZNF398	4.31981	4.16285	3.95347	4.05607	4.15428	3.89876
ZNF404	-0.149034	-0.195908	-0.193947	-0.380859	1.70706	1.50237
ZNF407	5.08428	4.97906	4.49507	4.23547	3.67619	3.36475
ZNF408	1.88215	1.78036	1.39395	2.0711	2.1112	2.11348
ZNF41	3.20329	3.58427	2.11571	2.21762	2.4767	2.94792
ZNF410	4.62142	4.51424	3.59967	3.91793	4.54291	4.73989
ZNF415	2.68679	2.52585	0.770744	0.619178	0.723471	0.483199
ZNF416	1.78879	1.8681	1.25925	1.49854	2.43246	2.32121
ZNF417	3.7625	3.8148	3.55225	3.74659	3.93988	3.46301
ZNF418	2.28108	2.27864	-0.568612	-0.979274	1.93217	1.22407
ZNF419	3.12444	3.20642	3.05234	3.19597	3.75372	3.46981
ZNF420	2.58783	2.83507	1.53076	1.43037	2.63332	3.06782
ZNF425	-1.41331	-1.00314	0.782333	1.04098	0.0677351	0.293394
ZNF426	3.74074	3.71956	3.40893	3.63769	4.26746	4.0263
ZNF428	1.91381	1.81417	0.894573	1.21399	2.44544	2.61656
ZNF429	-0.323873	-0.604659	1.38638	1.42259	-3.85089	-4.70296
ZNF43	4.2703	4.31392	3.95602	3.95371	1.24663	1.12623
ZNF430	2.76241	2.86312	2.9938	2.97599	2.84749	2.49782
ZNF431	4.07676	4.06977	3.89862	3.80305	4.00855	3.19519
ZNF432	2.767	2.86592	2.43744	2.63646	3.39892	3.48983
ZNF433	0.969734	0.929518	1.06197	0.432126	-0.316268	-0.584023
ZNF436	3.87929	3.84548	3.87717	3.94508	6.64196	6.35559
ZNF436-AS1	2.17438	2.17017	2.41268	2.47615	3.12923	3.15127
ZNF438	1.51527	1.36374	1.7707	2.00494	1.93963	2.09421
ZNF439	3.00476	2.68409	1.98395	1.92597	-0.0720838	-2.12114
ZNF44	2.1854	2.16085	1.61093	1.79809	0.928829	0.247587

ZNF440	3.16826	2.94942	2.49419	2.45065	2.85352	2.53756
ZNF441	2.22278	2.36689	2.59784	2.6856	2.23149	2.28199
ZNF443	1.83366	1.85036	1.88933	1.90378	0.430478	0.507181
ZNF444	2.25514	2.04497	2.71131	2.44577	2.3397	1.74453
ZNF445	5.13893	5.22057	4.99575	5.01206	4.95715	4.57751
ZNF446	2.05756	2.05767	1.94821	2.05223	2.18965	2.33905
ZNF449	2.64966	2.77078	3.23011	3.2176	3.60413	3.34857
ZNF45	4.29737	4.33723	3.42381	3.51504	4.63488	4.48309
ZNF451	6.19993	6.13975	5.86188	5.99832	6.57385	6.45394
ZNF460	4.94631	5.15794	4.59352	5.01892	5.55886	5.75376
ZNF461	1.38311	1.07637	0.759062	0.505972	1.52955	1.55071
ZNF462	5.65141	5.73704	4.57872	4.52597	5.90213	5.28481
ZNF468	3.55997	3.34166	2.31951	2.20771	3.04148	2.80093
ZNF469	2.85359	2.50006	2.89597	2.39103	2.71473	1.56945
ZNF470	-0.489437	0.107125	1.91453	1.68562	3.9105	3.2358
ZNF471	1.85937	1.68184	2.46035	2.03585	0.384182	-0.4586
ZNF473	4.07806	3.96747	3.69266	3.84519	3.90552	3.67697
ZNF48	1.98393	1.8546	2.09262	2.36692	2.24057	2.39133
ZNF480	3.43535	3.47674	3.25527	3.29844	3.71255	3.87448
ZNF483	2.64522	2.36054	2.8933	2.85408	3.01389	2.63315
ZNF484	3.04408	3.03501	3.03322	3.03073	2.99075	3.47071
ZNF485	1.83233	1.7171	0.324373	0.672606	2.00505	2.2002
ZNF486	2.76649	2.71707	2.03567	2.23992	-4.33548	-4.11926
ZNF487	0.356302	0.137189	0.759062	1.02051	-0.374735	-0.0918635
ZNF488	-0.791952	-0.485378	1.89865	2.21762	?	?
ZNF490	1.50294	1.76345	1.34812	1.60099	1.89926	1.63729
ZNF493	2.72286	2.71613	3.06746	2.78296	1.2101	0.90806
ZNF496	4.70232	4.79099	4.34784	4.53954	4.95392	4.97188
ZNF497	0.0936683	0.329181	0.621918	0.943629	-0.803675	-0.732088
ZNF500	2.41	2.36704	2.52609	2.8287	2.17347	1.42757
ZNF501	1.38311	1.55956	-1.32519	-1.06415	0.270529	0.126299
ZNF503	-0.816197	-0.485378	0.809135	1.14767	1.48025	1.76277
ZNF506	3.19892	3.1601	2.07568	1.84955	2.44136	1.5228
ZNF507	4.85213	4.79513	5.12992	5.0288	4.91522	4.64973
ZNF510	3.92846	3.93375	3.2532	3.38703	3.71474	4.07136
ZNF511	2.83574	2.82116	2.30778	2.58511	3.72954	4.32385
ZNF512	4.05259	4.21351	4.31473	4.41368	4.41273	4.32408
ZNF512B	4.99414	4.68111	4.92812	4.86975	5.45424	4.96762
ZNF513	2.63577	2.38745	2.25948	2.4498	3.41255	3.19925
ZNF514	3.95107	3.83865	3.59126	3.24211	4.02531	3.56474
ZNF516	4.09776	4.09557	4.14739	3.96933	2.78056	2.54123
ZNF517	1.37242	1.3381	1.34008	1.32585	0.969108	0.863209
ZNF518A	3.08393	2.77079	2.65569	2.41475	4.48815	4.28905
ZNF518B	-6.68418	-6.45019	-6.17309	-6.09892	4.43771	4.23433
ZNF519	3.1927	3.36027	4.17196	3.95322	3.39208	2.99225
ZNF521	5.16197	4.86806	1.92503	2.08102	?	-4.70296
ZNF524	-0.676496	-1.41808	-0.482896	-0.250687	1.05886	1.44307
ZNF525	3.04251	3.05709	2.29397	2.4657	2.5152	2.4755
ZNF526	2.86888	2.87701	1.94578	2.39103	2.87497	2.6899
ZNF527	1.8401	1.72701	1.27595	1.39901	2.06081	1.71958
ZNF528	3.81169	3.71082	2.41268	2.19953	2.01567	1.28202
ZNF528-AS1	1.399	1.3764	0.82777	0.100749	-1.13645	-3.38329

ZNF529	3.31711	3.51005	2.43463	2.19953	3.90184	3.41483
ZNF530	2.70394	2.85231	2.38635	2.4799	3.19611	3.10064
ZNF532	6.10651	5.73731	5.48135	5.22194	3.77315	3.13257
ZNF536	-5.69162	?	?	-6.09892	0.585578	1.40996
ZNF542P	3.08032	3.10564	-0.861345	-0.767904	0.724676	0.0133836
ZNF543	2.74714	2.76463	2.55607	2.8612	3.76194	3.37782
ZNF544	4.31611	4.32293	3.93056	4.07069	5.306	5.16128
ZNF546	2.51282	2.58156	2.38255	2.27921	3.02444	2.74038
ZNF547	1.75791	1.70246	1.35721	1.42849	2.01726	1.82221
ZNF548	3.26015	3.31431	3.30445	3.51996	3.71564	3.49718
ZNF549	2.97462	3.08018	2.66989	2.61155	3.56373	3.40997
ZNF550	3.27717	3.54138	3.25625	3.46901	4.05796	3.60586
ZNF551	3.72259	3.86683	2.81366	2.89661	3.98082	3.77104
ZNF552	2.23002	2.35563	2.65531	2.64887	2.70765	2.65292
ZNF554	0.0425183	-0.79	0.488988	0.445854	0.258664	0.0483059
ZNF555	2.72826	2.53152	2.28288	2.23776	2.72241	2.74862
ZNF557	3.08556	2.99249	2.541	2.71773	3.88658	3.75885
ZNF558	3.35852	3.07939	3.45732	3.27348	4.06229	3.62644
ZNF559	2.92296	2.65819	2.76485	2.67233	3.10772	2.70248
ZNF561	3.8583	3.8342	3.17773	3.2791	4.29944	4.37336
ZNF562	5.18108	5.24311	4.11985	4.23038	5.49111	5.4002
ZNF564	2.39265	2.3694	1.68036	1.60327	1.85088	2.41366
ZNF565	-0.0847132	0.0889557	0.564085	0.637861	0.931585	0.346021
ZNF566	3.09007	3.09598	2.30719	2.65385	2.64134	2.47817
ZNF567	2.73555	2.72204	1.91453	1.8512	2.3841	2.22994
ZNF568	2.89898	2.98218	1.37111	1.36695	2.09793	1.96574
ZNF569	2.51038	2.58252	1.46757	1.51326	2.31412	2.65962
ZNF57	1.38827	1.46066	1.01989	1.21565	1.36312	1.55939
ZNF570	2.77868	2.78187	1.93544	2.01532	2.88659	2.83245
ZNF571	1.36731	1.49719	0.208904	-0.52367	1.05399	1.15132
ZNF573	2.03755	1.92123	0.598108	0.773887	1.92701	2.12484
ZNF574	2.35443	2.62381	2.40346	2.7489	2.43785	2.61415
ZNF576	2.28874	2.24267	2.09369	2.53828	2.72647	3.24981
ZNF577	2.33259	2.20634	1.25179	1.0909	2.50712	1.51249
ZNF579	0.460879	0.0918691	0.544485	0.445854	0.749157	1.12623
ZNF580	1.62935	1.56763	2.04698	2.08638	2.91389	2.59414
ZNF581	2.7939	2.71094	1.91797	2.19457	2.97398	3.11552
ZNF583	1.51528	1.38268	-0.973801	-1.02108	0.954457	0.832509
ZNF584	2.98567	2.90348	2.61415	2.85408	3.09958	3.06148
ZNF585A	2.59819	2.56382	1.80987	1.5745	2.38094	2.51867
ZNF585B	3.54452	3.44289	0.46802	0.57416	3.39438	2.96623
ZNF586	3.02575	3.05279	3.06446	3.2161	3.58307	3.50196
ZNF587	4.61422	4.62688	5.03233	5.14796	5.47744	4.97611
ZNF587B	3.95564	4.04993	3.73087	3.97984	4.67111	4.59097
ZNF589	4.58525	4.85457	2.96575	2.92284	3.3717	3.04342
ZNF592	4.45984	4.36548	4.71063	4.65877	5.32208	4.91689
ZNF593	2.65617	2.27498	1.15539	1.6772	2.63952	3.50687
ZNF594	3.51402	3.61397	3.85677	3.60884	3.83454	3.16673
ZNF595	3.56301	3.65089	2.16079	2.03585	3.08452	3.14504
ZNF596	-0.815382	-0.705156	0.683723	0.613192	-0.685081	0.229711
ZNF597	2.01175	1.96344	1.662	1.95189	2.53937	2.61523
ZNF598	3.60721	3.45282	3.24186	3.44289	3.98282	3.95407

ZNF599	1.23761	1.35738	1.81651	1.8512	1.66165	1.8479
ZNF600	2.03579	2.20538	1.48938	1.69726	1.58427	1.74374
ZNF605	3.99876	4.14355	1.8609	1.80406	2.74058	2.54105
ZNF606	2.50411	2.49672	2.4171	2.32582	1.7584	1.30456
ZNF607	2.62447	2.63709	1.89244	1.87327	1.84186	1.55071
ZNF608	4.50731	4.22453	6.12137	5.76498	5.19687	4.61069
ZNF609	5.64686	5.43311	5.91351	5.92561	5.94861	5.24602
ZNF611	2.71299	2.76887	2.64567	2.82131	2.75721	2.70713
ZNF613	1.74389	1.8272	1.28413	1.39901	1.85935	1.97981
ZNF614	3.5886	3.71448	2.74399	2.80935	3.53568	3.69841
ZNF615	2.61608	2.63256	1.95093	1.97335	2.43774	2.38606
ZNF616	2.82448	2.90129	2.24276	2.19498	2.31411	2.34858
ZNF618	3.3562	3.11459	4.30431	3.97863	2.62741	1.94436
ZNF619	1.21983	1.48556	1.81651	2.22211	1.65261	1.80902
ZNF620	0.377835	0.395227	0.0833836	-0.086319	3.10788	2.95863
ZNF621	4.31563	4.26677	4.08559	4.31638	4.8885	4.5221
ZNF622	3.8555	3.85908	3.04289	3.49665	3.8544	4.17286
ZNF623	4.37103	4.43727	4.60029	4.80779	4.81896	4.73416
ZNF624	1.75216	1.33809	2.73237	2.56007	2.15669	1.95865
ZNF625	1.83627	1.75993	1.1862	1.46148	1.94211	1.31846
ZNF625-ZNF20	0.785935	0.257373	0.53048	1.2691	1.45147	1.72953
ZNF626	1.38111	1.11463	0.558032	0.837653	-5.07051	-3.12051
ZNF627	2.76036	2.74661	2.71736	2.75227	2.82552	3.12619
ZNF629	4.05078	3.81444	4.65567	4.75613	3.59708	3.47816
ZNF638	7.19796	7.10012	6.69986	6.67049	7.02288	7.13051
ZNF638-IT1	2.85167	2.76838	2.9939	2.66267	2.46382	1.95865
ZNF639	4.08146	4.05875	4.23537	4.57942	4.93355	5.18658
ZNF641	2.18969	2.12963	1.41643	1.36695	1.92843	2.1262
ZNF644	6.38573	6.46529	5.81541	5.8366	6.45829	6.8246
ZNF646	3.75229	3.7618	3.98743	4.10152	3.89908	3.63922
ZNF649	2.8397	2.96016	1.86761	2.10781	2.50583	2.41699
ZNF652	5.12188	4.93376	5.02226	5.02943	4.84102	4.59363
ZNF653	-0.569597	-0.604659	-0.300715	-0.823182	1.1211	0.893261
ZNF654	4.02458	4.01996	4.4236	4.49757	4.17731	4.11026
ZNF655	6.26668	6.39305	6.61043	6.75259	6.67268	6.74132
ZNF658	3.03677	2.91771	2.53326	2.41217	2.31617	2.74912
ZNF658B	1.11046	0.872246	1.40347	1.2977	2.01667	2.55952
ZNF66	1.13683	0.635262	0.801349	0.445854	-4.33548	-3.70484
ZNF660	1.73006	1.32248	2.51327	2.33785	-1.95932	-1.53638
ZNF664	6.22214	6.20027	5.10185	5.30687	5.7155	5.63408
ZNF665	0.832372	0.868134	0.355738	0.350701	-2.26731	-1.79933
ZNF667	3.22573	3.36688	-0.117291	-0.154483	1.81139	1.1007
ZNF667-AS1	1.99426	1.91208	2.76442	2.82745	1.13291	1.25912
ZNF668	1.53033	1.36737	1.26776	1.26434	1.61261	1.94835
ZNF669	2.50061	2.47968	1.60441	1.9191	2.19146	2.52212
ZNF670	2.47659	2.35437	1.42847	1.62532	1.2101	1.493
ZNF671	1.43538	1.50298	1.53761	1.6394	2.66837	2.34858
ZNF672	3.40682	3.06072	2.49597	2.78903	3.21467	3.13878
ZNF674	1.75572	1.95954	2.5312	2.78993	1.85456	1.84477
ZNF674-AS1	1.48583	1.31856	1.8609	2.15329	0.894341	0.33778
ZNF675	2.88964	2.82025	3.01861	3.16497	3.02619	3.43795
ZNF677	3.31634	3.13272	-4.59508	-6.09892	-4.33548	-5.69922

ZNF678	3.89057	3.87812	3.1111	2.894	3.43772	3.63092
ZNF680	4.36161	4.49497	4.06533	3.88977	3.53691	3.47319
ZNF681	1.61548	1.36507	1.92503	1.95729	0.437814	0.293394
ZNF682	1.867	1.70209	1.15638	0.619178	-2.95876	-3.38329
ZNF684	1.66289	1.78985	0.986462	1.14862	1.33409	1.83247
ZNF687	3.90775	3.67681	3.83854	3.91729	4.1516	3.94918
ZNF688	0.434551	0.46244	0.693436	0.96902	-0.852709	0.293394
ZNF689	2.53455	2.34774	2.60439	2.70496	2.26748	2.38606
ZNF69	1.70609	1.3764	1.97132	1.69856	-0.777433	-1.53638
ZNF691	2.26093	2.25827	2.72037	2.81896	1.99434	1.94436
ZNF692	3.62172	3.52584	3.16963	3.26838	4.02092	3.41741
ZNF695	1.97233	1.64012	0.877603	0.606717	-5.07051	-2.70579
ZNF696	1.17749	1.26512	0.636738	0.711424	1.87887	1.70271
ZNF697	5.77563	5.83807	3.5478	4.0256	5.36966	5.37143
ZNF699	1.88101	1.54842	1.42378	1.52691	0.868813	0.650921
ZNF7	4.08763	4.11541	4.20538	4.26176	4.07294	3.99905
ZNF70	3.43149	3.30699	3.42381	3.63433	2.60179	1.95152
ZNF700	3.50057	3.31617	3.38444	3.26681	3.57368	3.67421
ZNF701	2.60891	2.50187	1.8459	1.99242	2.00849	2.24298
ZNF702P	3.7848	3.83719	2.02402	2.46483	-5.65305	-4.70296
ZNF703	-2.45038	-2.00285	1.36341	1.24877	0.172692	0.33778
ZNF704	4.01601	4.36687	-4.18092	-3.52222	-1.45047	-2.12114
ZNF706	4.32798	4.33333	4.26029	4.11118	3.99835	3.88935
ZNF707	1.40948	1.40138	1.61093	1.86274	1.71041	1.74453
ZNF708	2.80732	2.83408	2.59565	2.34651	2.82553	2.83633
ZNF709	1.68256	1.71843	1.50563	1.48367	0.594568	0.094182
ZNF71	2.11792	1.89913	2.23435	2.29633	2.64804	2.32672
ZNF710	2.04799	1.7886	2.81159	2.83453	2.19091	1.78697
ZNF713	0.150573	0.0961944	0.186754	-0.0382537	2.03003	1.27575
ZNF714	3.94747	4.0135	4.18024	4.29737	3.21774	2.82467
ZNF717	3.28676	3.14078	3.50472	3.27055	-6.64581 ?	
ZNF718	2.75419	2.83637	-2.5977	-2.20128	2.12434	1.9868
ZNF720	2.26349	2.12963	3.18457	3.02538	1.59706	1.3157
ZNF721	4.54311	4.62124	4.32261	4.12406	5.13006	5.05539
ZNF724P	3.50426	3.46584	2.58467	2.8337	3.20384	3.25907
ZNF726	1.93998	1.78083	1.57932	1.3406	1.37372	0.606982
ZNF730	1.03481	0.904473	1.28096	1.03517	-5.07051	-3.70484
ZNF736	4.87454	4.92559	4.37871	4.33825	2.76173	2.39659
ZNF737	1.3985	1.20963	1.88409	1.81997	-13.2876	-4.11926
ZNF738	2.10184	1.97257	2.12938	1.82784	1.00508	0.293394
ZNF74	3.25657	3.13707	3.12026	2.81599	2.58525	2.26485
ZNF740	4.9298	4.81907	4.05368	4.13905	4.37723	4.06147
ZNF746	3.37878	3.27036	4.0043	3.89998	3.27693	3.20147
ZNF747	2.50085	2.27565	1.95768	2.1	-1.39675	-2.23309
ZNF749	2.56947	2.56496	2.23001	2.26877	2.99608	2.74038
ZNF750	0.62293	0.756391	0.191626	-0.086319	-0.302594	-0.899103
ZNF75A	3.78476	3.67246	3.32513	3.26922	3.87091	3.46014
ZNF75D	2.88401	2.8881	3.66822	3.74676	3.5496	3.4582
ZNF76	3.19877	3.02901	3.35758	3.44	3.732	3.74037
ZNF761	3.16287	3.10193	2.83097	2.99116	2.80458	2.51104
ZNF764	1.42508	1.3381	1.46757	1.4458	0.652647	0.483199
ZNF765	3.24731	3.31657	3.13831	3.30105	3.25214	3.40149

ZNF766	3.24637	3.15187	2.6109	2.6157	3.18518	3.05484
ZNF767P	3.1948	3.12379	3.84385	3.91039	3.10056	2.43577
ZNF768	3.11064	3.32586	2.78722	3.12082	3.27619	3.68987
ZNF77	1.7928	1.87257	1.24279	1.39106	1.90577	1.78517
ZNF770	5.49536	5.62095	4.85607	5.02494	5.59027	5.3363
ZNF771	1.27904	1.07937	1.39	1.47986	1.23803	1.22235
ZNF772	3.26237	3.50149	2.8191	3.01439	4.44493	4.5987
ZNF773	1.76816	1.8619	2.14321	2.25492	3.24085	2.73774
ZNF774	1.28965	1.26512	1.30837	1.40691	1.80324	1.44308
ZNF775	1.14646	1.27191	3.0453	3.42451	1.2406	1.70271
ZNF776	3.97596	3.9454	3.85813	3.87799	4.69284	4.71219
ZNF777	2.09536	2.04497	2.15189	1.81601	2.09125	1.87827
ZNF778	4.2108	4.18371	3.43486	3.49108	3.81643	3.24749
ZNF780A	4.07359	4.03238	3.95986	3.90351	4.43743	4.53289
ZNF780B	4.73218	4.73681	4.60507	4.51064	5.41557	5.26031
ZNF782	2.28393	2.38891	2.59785	2.43422	2.15988	1.80902
ZNF783	3.73819	3.67683	2.84277	2.85661	2.95357	2.61239
ZNF785	2.66485	2.53049	2.83776	2.93046	-2.3604	-2.28791
ZNF786	2.5055	2.57334	2.02595	1.87419	2.15349	1.95865
ZNF787	1.96628	1.95924	1.8772	2.11036	2.72556	3.01097
ZNF788	3.97981	3.95491	2.9066	2.9149	1.79661	1.61494
ZNF789	3.47364	3.53302	3.75205	3.89579	3.35086	2.75682
ZNF79	2.37775	2.41061	1.71738	2.01014	1.5443	1.2122
ZNF790	2.32884	2.35095	1.40898	1.28354	2.64804	2.70269
ZNF791	4.34188	4.29205	3.85951	4.04472	4.78625	4.53525
ZNF792	2.02886	2.08021	3.43853	3.60713	2.01567	1.82469
ZNF799	1.84801	1.6895	1.10483	0.899121	0.525748	0.858315
ZNF8	4.06599	3.84126	3.61346	3.47111	4.25258	3.52094
ZNF800	5.26598	5.17919	4.52469	4.41472	4.71309	4.70902
ZNF804A	0.743937	0.822624	1.09265	1.27492	2.2254	2.85935
ZNF805	2.60109	2.37014	2.1677	2.28578	3.28193	2.95187
ZNF808	3.68784	3.58433	3.19767	3.10212	1.80795	1.2008
ZNF81	4.11471	4.1792	2.82771	2.7209	3.30547	3.32671
ZNF813	3.13353	3.12455	1.33489	1.318	-1.03019	-1.43954
ZNF814	4.92099	4.98542	4.51711	4.44186	5.20657	4.67902
ZNF815P	0.537316	0.105383	-0.496346	-0.243552	1.48044	1.36896
ZNF816	2.47346	2.51287	2.36327	2.43045	2.28914	2.86404
ZNF816-ZNF321P	0.192257	0.293253	1.00826	1.05979	1.31686	1.01711
ZNF821	0.454239	0.018506	0.854921	0.996114	1.08749	1.02445
ZNF823	1.82842	1.614	2.02596	2.26623	1.75757	1.82469
ZNF826P	1.38843	1.36374	0.292311	0.0612232	-6.64581	?
ZNF827	4.68946	4.56437	3.27386	3.39499	4.53812	4.09418
ZNF829	3.21381	3.2887	1.06446	0.833756	1.90196	1.97981
ZNF83	3.72298	3.7052	4.30432	4.08346	3.91588	3.66476
ZNF830	3.26526	3.47967	2.72037	3.16497	3.171	3.01097
ZNF836	0.784713	0.659979	0.82042	1.01122	1.22184	1.46322
ZNF839	3.0309	2.97906	1.90509	2.03228	2.08508	1.83021
ZNF84	4.74799	4.78507	3.86904	3.80406	4.55401	4.43795
ZNF841	3.26847	3.43245	3.37871	3.40181	3.5592	2.99263
ZNF844	2.53934	2.33218	2.43854	2.34325	0.997957	0.50283
ZNF845	3.48974	3.42688	2.87125	2.98854	3.15123	3.70903
ZNF85	2.5663	2.49136	2.25729	2.1904	0.91718	0.952773

ZNF850	3.94027	4.0695	2.23856	2.19953	2.38389	1.82469
ZNF852	2.12343	2.22717	1.91332	1.94094	1.80698	1.28103
ZNF853	-5.69162	?	1.48894	1.08105	-3.48878	-4.70296
ZNF860	2.63502	2.85004	-2.09546	-2.20128	1.81139	1.66836
ZNF862	1.88642	1.73084	2.6423	2.82211	1.36868	0.780436
ZNF865	0.430296	0.060836	0.208904	0.535117	0.847548	0.0747657
ZNF879	1.28965	1.3251	0.860939	0.0410467	-2.48959	-3.12051
ZNF880	2.48333	1.83639	1.90926	1.47617	0.159978	-1.18284
ZNF883	3.54766	3.74294	0.53081	0.476226	2.70164	3.01097
ZNF891	3.94543	4.19941	2.18852	2.16467	2.93386	2.55508
ZNF90	3.06254	2.98057	1.43603	1.90366	0.57918	1.76133
ZNF91	4.64298	4.41366	5.20863	5.06889	4.63571	4.43921
ZNF92	3.93638	3.79994	3.79231	3.74956	4.56366	4.52799
ZNF93	3.08311	3.26526	1.92802	2.12835	-1.07506	-0.662104
ZNFX1	6.16926	6.19907	6.17894	6.53456	6.32391	6.28491
ZNHIT1	4.68344	4.62186	4.54024	4.83006	4.74378	5.12015
ZNHIT2	1.11475	1.01295	-0.11729	0.19507	0.69732	1.00759
ZNHIT3	3.54668	3.60367	3.58738	3.91304	3.92161	4.59268
ZNHIT6	4.1187	4.32993	4.14066	4.42547	4.62162	4.53408
ZNRD1	2.27089	2.44871	2.14225	2.29721	2.7482	3.44097
ZNRD1-AS1	3.00751	2.87203	3.75534	3.65607	3.09799	2.82966
ZNRF1	2.92198	2.49678	3.63637	3.56491	2.46641	1.56011
ZNRF2	2.73571	2.61155	2.88477	2.63122	3.00912	2.78752
ZNRF3	3.52732	3.64306	3.39202	3.52234	2.90575	2.83633
ZPR1	4.33681	4.51438	4.04809	4.6203	5.58624	5.74134
ZRANB1	4.53041	4.4246	4.61766	4.8521	4.82457	4.80168
ZRANB2	5.75268	5.73109	6.24339	6.28652	6.00043	5.63621
ZRANB3	3.02202	2.71956	1.96116	2.12001	2.71256	3.02121
ZRSR1	-0.162472	0.41778	0.579006	0.944783	-0.749861	-2.17176
ZRSR2	1.31172	1.59087	1.30488	1.54091	1.24368	0.463298
ZSCAN12	3.14799	3.19715	3.3749	3.51871	0.823578	1.06153
ZSCAN16	1.38263	1.69247	1.08139	1.17529	2.42157	2.5173
ZSCAN16-AS1	-0.561392	-0.568099	0.0346524	0.319629	0.810591	0.847941
ZSCAN18	1.76447	1.55586	-1.35334	-1.86039	?	?
ZSCAN2	2.52215	2.33497	3.07315	3.20098	2.02486	1.62242
ZSCAN20	2.91011	2.99249	2.759	2.91355	3.23299	2.96925
ZSCAN21	2.91512	2.931	2.46237	2.78957	2.76134	2.99172
ZSCAN22	2.2018	2.15923	2.27592	2.24874	2.79708	2.18205
ZSCAN23	-0.413499	-0.27286	1.91453	1.6394	?	?
ZSCAN25	4.27246	4.31029	4.14626	4.37095	4.06931	4.05317
ZSCAN26	2.89898	3.01895	2.80963	3.0686	3.70312	4.06312
ZSCAN29	4.58741	4.65153	4.12936	4.13553	5.00676	4.73623
ZSCAN30	3.17887	3.38015	3.89225	3.90168	2.31312	2.07665
ZSCAN31	0.539412	0.318612	3.20018	2.76154	3.3298	3.20618
ZSCAN32	2.98029	2.86833	2.85227	3.0634	2.81116	2.85455
ZSCAN5A	1.66681	1.51716	1.33777	1.79049	1.58646	1.45229
ZSCAN9	2.57006	2.73192	2.70218	2.80706	3.77744	3.43795
ZSWIM1	3.16667	2.99454	3.07387	3.31744	2.87886	2.76902
ZSWIM3	1.84784	1.76119	1.5913	1.8454	1.46385	1.41227
ZSWIM4	2.56067	2.33158	2.17405	2.06112	3.07947	2.45317
ZSWIM5	0.676033	0.676628	0.584741	-0.0642959	2.50712	1.87827
ZSWIM6	3.79277	3.59649	4.64302	4.54042	4.5745	3.91899

ZSWIM7	2.39369	2.3773	1.91979	2.0561	3.07394	2.87776
ZSWIM8	4.5724	4.25241	4.51011	4.58505	5.16779	4.84011
ZUFSP	2.44049	2.49717	2.47113	2.67582	3.19144	3.4607
ZW10	2.7726	3.02299	4.29017	4.71613	3.87981	4.17287
ZWILCH	4.64913	4.96927	4.01535	4.56875	4.78079	5.73781
ZWINT	5.59639	5.52049	5.10533	5.39548	5.1686	4.96923
ZXDA	0.59986	0.408136	0.499231	0.392515	2.01449	1.95322
ZXDB	2.65306	2.70698	2.40248	2.78422	3.45516	3.08042
ZXDC	3.6727	3.70331	3.9509	3.94509	3.56971	3.08771
ZYG11A	2.07902	2.27188	?	-4.52056	-5.65305	?
ZYG11B	5.37906	5.47707	5.4964	5.79691	5.27596	5.3533
ZYX	4.66285	4.73989	3.83467	3.98657	4.49768	4.49541
ZZEF1	5.22565	5.17875	5.89362	5.87732	6.01936	5.63867
ZZZ3	5.79949	5.72419	5.67015	5.75959	6.35612	6.61383
bP-21264C1.1	-0.459286	-0.368926	1.2088	0.789354	-1.60029	-1.38442
bP-21264C1.2	1.32133	1.24121	2.24601	2.20668	-0.576704	-0.577229
bP-2171C21.6	-0.145242	-0.334076	0.914572	0.833424	1.43774	1.1007
bP-2189O9.2	0.254933	0.26265	2.11805	1.81052	-0.673562	-1.57134
pk	5.82932	5.89284	6.93909	7.30592	5.44387	5.33728
uc_338	0.34872	0.0217356	0.634575	0.705093	0.281028	-0.400336
