# Supplementary material for: The long non-coding RNA HOTAIRM1 promotes tumor aggressiveness and radiotherapy resistance in glioblastoma
Source: Cell Death Dis. 2021 Sep 28;12(10):885. doi: 10.1038/s41419-021-04146-0 (PMC8478910; doi:10.1038/s41419-021-04146-0)
Supplement: Supplementary file 14 — Supplementary Table 3 [file 41419_2021_4146_MOESM14_ESM.pdf]

Supplementary Table 3. Mass spectrometry results of transient *HOTAIRM1* knockdown cells

Data supplied as Log2 intensities

ID	LN-229_HOTAIRM1_KD	LN-229_control	T98G_HOTAIRM1_KD	T98G_control	U251_HOTAIRM1_KD	U251_control
A2M	23.7961	24.2484	20.9874	21.8376	24.5263	19.6764
AAAS	21.0028	21.4093	21.9546	22.0133	21.4531	21.2581
AAK1	20.1521	19.0896	19.113	18.7751	19.5256	19.2923
AAMP	18.2859	17.5486	18.3186	17.919	18.2106	19.1009
AAR2	18.2256	17.0338	17.9656	18.1982	16.6947	17.4465
AARS	24.9249	22.774	23.8972	23.4092	23.6321	24.7188
AARS2	20.3005	18.9527	19.5286	19.8007	19.1916	21.1651
AASDHPPT	19.2435	18.435	19.3625	18.8208	18.8118	19.0492
AASS	19.7797	20.343	20.1296	20.2994	20.105	20.1463
AATF	18.0309	19.4514	19.3122	19.5214	19.4427	18.9327
ABCB6	19.0761	17.5803	18.6887	19.2478	16.1452	18.9584
ABCB7	18.0416	19.2249	18.8478	18.9956	19.9263	19.2378
ABCC1	21.7664	20.9115	22.6724	23.0943	20.1247	20.4115
ABCC4	20.1448	17.3922	19.061	19.4672	16.2738	14.8332
ABCD3	20.7152	20.583	21.8341	21.8103	20.491	21.1906
ABCE1	23.7282	23.2568	23.4971	23.1871	23.9215	24.1421
ABCF1	19.8182	20.6761	19.4084	18.7626	20.7844	21.2962
ABCF2	20.0245	19.7535	20.6983	20.5713	20.1085	21.3692
ABCF3	19.3566	18.1994	18.776	18.9915	18.9064	20.612
ABHD10	21.3017	22.1339	22.1204	22.0633	22.3354	21.0212
ABHD11	20.4188	20.3011	21.3861	21.5546	20.4524	20.2456
ABHD12	20.8257	20.504	20.6276	21.0875	20.6896	20.6127
ABHD14B	19.225	19.0796	18.6864	18.8932	19.2182	20.0557
ABLIM3	18.8124	19.5443	17.0045	15.5612	20.4128	13.3227
ABR	17.8089	17.9075	18.4318	17.6137	18.799	19.0705
AC013461.1	18.7127	18.5323	19.0265	18.5328	19.5132	17.3768
ACAA1	21.6187	22.153	21.6172	21.8022	21.9257	22.3319
ACAA2	22.8125	22.1665	22.0531	22.3475	22.124	21.1992
ACACA	20.0134	20.214	21.1055	20.8865	20.6307	21.5537
ACAD8	21.0336	20.5175	19.9341	19.9711	20.8	20.4349
ACAD9	20.0819	20.4788	21.4202	21.2849	20.7161	19.9568
ACADM	24.0113	23.2541	23.7704	23.5907	23.7396	24.1142
ACADSB	20.7204	19.8947	20.9369	21.168	19.9709	21.0647
ACADVL	24.6323	24.2466	24.861	25.0692	24.399	24.2135
ACAT1	24.4307	23.8659	24.4069	24.7179	23.7886	23.7469
ACAT2	23.3492	21.552	21.8328	21.5623	21.4649	23.3567
ACBD3	21.4743	21.1724	21.4577	21.2153	21.8511	21.3696
ACIN1	22.0597	22.2294	22.0812	22.2529	22.2223	21.7356
ACLY	24.9424	25.1736	24.5684	24.4177	25.4569	25.781
ACO1	22.0551	23.06	21.4542	20.7756	23.7709	21.5579

ACO2	25.4374	24.4815	25.2355	25.3069	24.8406	24.559
ACOT1	21.471	20.9596	19.2869	19.8685	21.142	20.3219
ACOT7	23.9148	21.9422	23.2441	23.0282	22.5368	23.9407
ACOT8	20.1089	20.799	19.7738	19.754	20.2673	18.3876
ACOT9	21.5259	20.3501	21.0181	21.2916	20.6431	18.77
ACOX1	20.0364	19.5286	19.8286	20.3313	18.6492	20.0742
ACOX3	17.7828	17.5764	19.2548	19.2677	17.0565	17.8182
ACP1	22.2282	21.0478	21.2699	21.1311	21.6348	22.5451
ACP2	20.2988	21.208	19.8145	20.0719	21.0331	19.7677
ACSF2	19.9312	20.7814	20.6578	20.9057	20.2222	18.8488
ACSF3	18.4246	17.419	18.9046	19.3968	16.9276	17.2587
ACSL1	21.0785	20.2379	20.8442	20.6417	20.8557	17.4565
ACSL3	21.9468	23.0329	23.4505	23.7262	23.3187	23.5539
ACSL4	24.3207	24.3618	24.6122	24.3593	24.922	24.034
ACSL5	16.8745	18.6737	18.641	18.2118	19.7463	16.8334
ACSS1	19.2728	18.8198	19.6452	19.1923	19.3178	19.051
ACSS2	18.8424	14.0305	16.2151	15.551	14.8546	14.5305
ACTB	29.0856	28.9118	27.6962	28.2321	28.8405	28.7376
ACTBL2	22.5427	23.1985	22.831	22.0786	23.21	21.896
ACTC1	26.8743	26.0377	25.4368	26.2565	26.5273	26.5993
ACTL6A	22.2247	22.1064	22.4338	22.3769	22.0722	22.6051
ACTN1	27.5091	27.2805	26.189	26.1548	27.3437	26.6488
ACTN4	27.373	27.4897	27.5946	27.5927	27.5287	28.4069
ACTR1A	22.9323	22.3841	21.9544	21.9007	22.5283	22.4171
ACTR2	23.2729	23.5837	23.3411	23.3657	24.1593	24.1802
ACTR3	24.9867	24.9113	24.5446	24.5718	25.3106	25.272
ADAM17	19.5217	20.1666	19.7857	19.6957	20.2457	20.2495
ADAM9	17.9153	18.3758	18.5035	18.3194	17.0284	17.495
ADAR	22.1556	22.6764	23.0053	23.3126	22.7154	22.8091
ADD1	20.909	19.8814	20.9295	21.0123	19.1293	20.1996
ADD3	19.8287	18.7452	20.5096	20.1932	18.5603	18.0168
ADGRE5	16.3714	18.8441	18.5698	18.0389	18.4568	18.9359
ADH5	25.0812	23.7503	23.3092	23.0081	23.9888	23.9091
ADK	20.6658	19.2821	18.7013	18.189	20.2099	20.146
ADNP	19.5806	20.9011	20.1593	20.5252	20.8818	19.9696
ADPGK	18.7625	19.8549	20.0339	20.5336	19.9171	18.8443
ADPRHL2	18.9345	17.5555	18.0601	17.6246	17.5999	19.6167
ADRM1	21.0928	20.3425	20.7679	20.4989	20.4934	21.0483
ADSL	21.6994	21.1395	20.9936	20.7199	21.3878	22.4407
ADSS	23.3445	22.1214	22.1147	21.5851	22.813	23.5554
AFAP1	16.5625	18.044	15.9496	15.9601	15.5913	17.2699
AFG3L2	21.3645	21.6948	22.1537	22.1144	21.9586	21.8471
AGFG1	19.4842	17.5207	19.26	18.5923	18.0955	20.6882
AGK	22.6146	22.537	22.8293	22.8837	22.5285	23.2106
AGL	20.494	19.0307	18.735	18.7682	18.6238	19.3685
AGO2	20.6808	20.9319	20.8374	19.9512	21.5048	21.8291
AGPAT1	18.9125	18.6584	18.0192	18.5188	19.0767	18.3967
AGPS	23.651	23.498	24.4342	24.4365	23.5746	24.2872
AGRN	15.5709	17.2067	16.2063	16.9685	17.3173	15.5727
AGTRAP	17.4985	18.8824	19.2135	19.033	19.3249	16.0985
AHCTF1	16.5902	18.3972	17.938	17.8113	18.5185	16.0158
AHCY	24.0507	23.9086	24.2935	24.0781	24.0319	25.0436

AHCYL1	22.0383	20.6479	20.3593	20.2167	21.2811	21.1553
AHNAK	27.7118	26.7778	28.1183	28.1623	27.3843	25.892
AHNAK2	18.0279	16.7209	16.74	16.59	17.7556	17.7427
AHSA1	22.6471	21.791	21.2953	21.3431	22.2096	23.6437
AHSG	20.2662	17.2362	16.0226	16.5597	18.2434	13.7051
AIDA	22.6833	22.7954	22.3648	22.0449	23.2337	22.6547
AIFM1	23.1291	22.9655	23.6603	24.0173	22.8096	23.7071
AIM1	18.7582	16.5526	17.6857	16.0567	15.2952	16.6055
AIMP1	22.669	21.356	21.703	21.9016	21.6798	22.4239
AIMP2	23.0503	22.4332	22.3881	22.2995	22.208	23.1048
AIP	21.1371	20.6894	20.4568	20.3237	20.9542	20.8343
AK1	21.5111	19.5334	19.294	19.3764	19.9346	19.969
AK2	23.0585	23.7456	24.2318	24.58	23.6914	23.9211
AK3	22.5737	21.8356	22.1942	22.4056	22.0508	22.7859
AK4	21.1557	19.9135	20.2268	20.4665	19.9552	22.2183
AK5	17.494	17.4504	18.5817	18.1819	18.0801	17.2483
AKAP8	18.7356	19.0485	18.6623	18.7465	19.2725	19.5369
AKAP8L	18.8204	19.0345	18.4986	18.5861	18.9429	18.838
AKR1A1	21.2345	19.9302	19.456	19.5189	20.307	20.0954
AKR1B1	26.9814	26.5094	25.7655	25.4157	26.3416	24.3892
AKR1B10	23.6919	20.0383	24.6316	24.4146	19.9562	20.4004
AKR1C1	20.0617	16.4945	21.6698	21.2496	15.3305	16.035
AKR1C2	22.648	17.894	22.2103	22.4979	18.33	17.9656
AKR1C3	23.4622	17.8916	22.4926	22.5299	17.5441	21.066
AKR7A2	21.7382	20.3214	19.7331	19.7292	20.6548	20.9659
AL365273.1	19.6432	13.5177	13.6419	14.5513	12.8926	12.5845
ALB	25.6081	23.5976	21.1159	21.4855	23.8647	20.9132
ALCAM	20.7579	23.438	21.8558	21.8177	23.3432	21.527
ALDH16A1	20.5753	18.8611	17.9202	17.7123	19.4155	20.1981
ALDH18A1	23.7377	23.7525	24.6834	24.775	24.2328	24.6851
ALDH1A3	20.0997	20.3048	20.6149	20.6604	20.383	21.1938
ALDH1B1	19.8021	21.5686	21.2448	21.254	21.4192	21.9849
ALDH1L2	16.8007	18.7424	13.3588	14.2949	20.0037	15.1933
ALDH2	25.2713	22.6089	24.4373	24.7247	22.9483	22.4818
ALDH3A1	23.654	18.6037	20.2673	19.9371	18.5883	19.6998
ALDH3A2	22.663	20.2528	22.3146	22.3781	20.5586	21.1566
ALDH4A1	18.2931	18.7061	17.9657	18.5888	18.5973	16.6563
ALDH6A1	20.7116	20.1422	19.7388	19.7246	20.4252	20.4095
ALDH7A1	24.3728	23.4163	21.4926	21.7229	23.265	22.8361
ALDH9A1	22.4497	22.8272	21.111	21.4658	22.6167	22.3903
ALDOA	28.5415	26.9562	27.0282	26.9419	27.1088	28.1704
ALDOC	26.3	24.4006	24.4998	23.9608	24.9266	25.9306
ALG1	19.6632	20.2725	20.3183	20.2693	20.0604	18.1783
ALG2	16.6397	18.0614	17.1419	17.2945	15.616	13.6388
ALG5	18.8361	18.5175	19.95	19.9037	18.2753	18.9209
ALYREF	20.9761	21.7559	21.7553	22.0167	21.6456	22.6898
AMFR	19.5046	19.1203	20.326	20.0349	19.5109	19.418
AMIGO2	17.0146	16.5275	16.466	15.8886	17.1107	18.4034
AMPD2	20.3435	19.6352	19.9169	19.6124	20.351	20.7507
AMPD3	18.812	20.0911	19.0424	19.4009	22.3997	19.0834
ANAPC1	18.3739	17.0628	17.6752	17.4424	17.4885	17.2657
ANAPC4	17.1053	18.0158	18.2412	18.2051	18.0679	17.9496

ANAPC7	18.7856	18.7874	19.1052	18.8506	19.2742	19.3364
ANK3	11.8055	14.5034	16.2015	15.067	15.5629	16.1255
ANKFY1	20.7509	19.8546	20.8618	20.036	20.3428	19.7837
ANKHD1	17.4045	16.6454	16.8419	16.8628	16.4997	17.8714
ANKLE2	16.1686	16.2176	16.2561	17.3157	16.1624	14.9357
ANKRD52	16.6504	16.1732	15.6262	14.9349	16.0685	16.9005
ANLN	20.7599	19.7907	21.1887	20.4278	20.0285	19.7507
ANO10	15.9604	17.741	17.3667	17.6065	15.7358	15.422
ANO6	18.5605	18.4818	18.4308	18.456	18.8175	17.4256
ANP32A	18.3656	18.9951	19.0788	19.9251	18.9368	20.0012
ANP32B	22.3509	21.8638	21.8086	21.9249	21.9332	22.4058
ANP32E	21.8434	21.666	21.504	21.1304	21.7036	21.9867
ANPEP	21.6983	26.7327	21.3281	21.2826	27.2884	21.7359
ANTXR1	17.0217	16.9393	17.9449	18.1068	14.6995	13.8798
ANTXR2	16.1514	17.2666	15.046	14.2156	16.856	12.3711
ANXA1	26.6378	25.995	25.9468	25.8645	26.3814	27.2957
ANXA11	22.4525	21.7461	22.3374	21.8603	21.8628	22.2144
ANXA2	28.5516	28.4392	28.9478	29.157	28.2932	29.8117
ANXA4	22.4093	20.514	21.2734	20.8264	20.8294	21.3252
ANXA5	26.9199	26.9081	25.2955	25.1027	27.2611	25.95
ANXA6	25.4413	25.4066	25.534	25.2208	25.58	22.2095
ANXA7	22.6836	21.9886	21.5479	21.4782	22.408	22.4962
AP1B1	23.1263	22.9239	22.7147	22.75	23.0919	23.2562
AP1G1	22.7789	22.3409	22.6683	22.3569	23.1763	23.1313
AP1M1	21.6232	20.9772	21.3995	21.1014	21.5209	22.1936
AP1S1	16.8618	17.573	17.9168	17.849	19.3311	18.167
AP2A1	22.3407	23.4117	22.6497	23.2432	22.8021	23.1738
AP2A2	21.3438	21.5807	21.9689	21.6613	21.5757	20.3569
AP2B1	24.0473	24.0865	24.2894	24.1849	23.9507	24.1855
AP2M1	21.5749	22.396	21.8021	22.1791	22.6076	22.5031
AP2S1	21.2341	21.9364	21.2853	21.8725	21.4334	20.8836
AP3B1	21.5689	21.7507	21.3766	20.7051	22.5461	22.0271
AP3D1	19.432	20.5422	20.1092	19.8282	20.7478	20.2431
AP3M1	21.6429	21.3808	21.2312	20.9173	21.8491	21.7929
AP3S1	19.5342	19.5768	19.2175	18.9748	20.3036	18.7765
APEH	20.9219	20.2122	19.7682	19.5308	20.8196	22.063
APEX1	24.0332	23.3182	22.3946	22.4264	22.7606	22.9349
API5	22.2124	23.1513	22.7145	22.6996	23.2241	23.505
APIP	14.9609	14.3827	9.6483	14.1222	14.5331	9.08322
APLP2	18.9667	18.3131	19.0584	18.781	18.5219	19.1048
APMAP	23.8326	23.5219	24.3752	24.3274	23.6235	24.2484
APOA1BP	19.7658	17.7679	19.2174	19.1622	17.7868	19.7382
APOB	19.3939	18.8855	19.0964	18.7089	19.2171	19.6495
APOBEC3B	21.0679	20.8489	22.047	22.1332	20.764	19.2956
APOBEC3C	20.7499	20.113	21.0192	20.9316	20.0467	19.3432
APOL2	18.4014	19.3933	18.8969	18.9026	19.2143	17.6203
APOO	19.4266	18.6044	20.1789	20.2058	18.8712	19.3755
APP	16.8738	16.5701	17.15	16.9873	16.4832	16.8049
APPL1	16.9265	16.1069	16.1844	15.8076	16.4303	18.082
APRT	22.8382	22.2876	22.3175	22.3393	22.3796	22.7857
AQR	19.2461	20.2651	20.9001	20.9092	19.5288	19.1748
ARAP1	18.4012	19.6594	18.9627	18.6918	20.3452	19.2659

ARCN1	24.263	24.1311	23.8203	23.6226	24.6089	23.8247
ARF4	20.6907	22.1722	21.5903	21.7115	22.7138	22.095
ARF6	16.9173	18.8847	17.94	17.9499	18.4953	16.2563
ARFGAP1	19.4868	19.1038	19.2625	18.5049	19.9163	19.9512
ARFGAP2	16.7749	14.1561	14.0701	14.7627	13.6751	17.0576
ARFGEF1	15.1379	13.7149	14.8001	13.0157	15.9108	14.7161
ARFIP1	19.9643	18.7396	18.6333	18.0027	19.3281	19.6548
ARG2	16.7437	19.25	16.6429	16.1058	19.7467	18.9033
ARHGAP1	21.9833	22.3787	20.8663	20.8254	22.9672	22.8049
ARHGAP17	21.2195	20.1635	20.3235	19.9364	20.0553	19.739
ARHGAP18	17.8952	18.0943	16.487	15.169	19.553	19.1949
ARHGAP35	18.6143	15.9752	16.5135	15.8047	16.685	16.2433
ARHGDIA	24.6932	24.1301	22.8705	22.9977	24.2889	25.6068
ARHGEF1	20.7781	20.4479	19.8467	20.1746	20.8293	20.7205
ARHGEF2	20.2876	20.4302	19.9268	20.146	20.1949	20.7492
ARID1A	18.1238	19.0422	18.7606	18.9786	19.0942	18.307
ARL1	20.3634	21.3906	20.6442	20.6052	21.6399	20.4731
ARL2	18.777	18.6725	18.7045	18.9586	17.9303	17.8411
ARL3	20.0959	19.8648	20.1385	20.1771	20.2643	20.695
ARL6IP5	21.635	21.9466	21.3762	21.6444	22.0199	21.4002
ARL8B	19.3211	20.7428	19.8383	19.9661	21.2111	20.604
ARMC10	15.9375	15.4707	17.8774	18.2579	14.9568	18.0756
ARMC2	16.751	15.771	18.0545	16.7497	17.31	15.7486
ARMC6	18.4551	18.0522	17.3186	17.3262	18.2084	19.4788
ARMCX3	18.849	20.5562	18.6871	18.253	21.2361	18.8785
ARMT1	18.8479	18.1061	18.4407	18.4488	17.7892	18.8669
ARPC1A	22.0285	22.4034	21.643	21.7959	22.4822	23.0439
ARPC1B	23.3753	23.4732	23.0484	22.9187	24.096	23.3644
ARPC2	23.9154	23.9612	23.7341	23.4943	24.3968	24.4115
ARPC3	24.1781	24.3472	23.5014	22.8631	25.18	24.0432
ARPC4	23.9913	24.2796	24.1224	23.9626	24.7888	24.4315
ARRB1	16.4108	15.946	16.3859	16.5913	14.9351	14.2012
ARSA	19.2984	18.1302	18.5011	18.8281	15.1905	14.2783
ARSB	19.1303	19.5318	17.7805	17.2265	19.6829	16.7321
ASAH1	20.3787	19.9874	19.8069	19.9841	19.3907	19.7468
ASCC2	19.6019	19.0567	18.7094	19.244	19.069	19.3655
ASCC3	17.2802	17.3933	17.3694	16.9684	18.2049	18.0485
ASF1A	17.0221	16.4294	17.794	18.1052	15.3813	18.6412
ASF1B	18.7257	17.4131	18.6292	18.8339	16.6119	16.8948
ASH2L	20.2009	19.9563	20.4296	20.3034	19.3323	20.0648
ASL	19.3296	18.6905	17.7376	17.2762	19.4909	19.088
ASNA1	23.2823	22.5565	22.2737	22.4788	22.7096	23.0041
ASNS	20.6607	19.2464	20.5676	20.0815	20.513	21.9689
ASPH	23.2526	24.1728	24.5493	24.6642	23.9689	23.0044
ASPSCR1	19.1135	17.9764	16.2358	16.1739	18.1868	19.3674
ASUN	16.8537	17.1504	17.7372	17.8853	15.751	15.8197
ATAD1	20.2436	20.6293	20.569	20.7361	20.9008	20.2871
ATAD2	19.14	17.7255	19.5807	19.2507	17.1842	18.1428
ATAD3A	21.3811	21.5989	21.7525	22.1373	21.6717	21.8655
ATAD3B	20.5175	20.578	20.8258	21.1326	20.7602	21.3225
ATG3	20.7747	19.6216	19.2935	18.8708	20.114	20.2095
ATG5	14.6393	14.0721	14.4887	11.8637	14.7123	16.9878

ATG7	18.9401	20.8545	18.9459	18.5353	21.6145	19.8712
ATG9A	18.2642	17.5419	17.7078	17.7941	16.6491	18.2465
ATIC	25.343	24.6607	23.672	23.3867	25.0493	25.321
ATL2	19.5741	19.5008	20.3604	20.0169	19.5222	19.5797
ATL3	24.4784	24.3325	23.6975	23.6461	24.2402	23.0563
ATP13A1	23.4374	22.7379	22.547	22.4948	22.2562	24.1054
ATP1A1	24.4108	25.6692	25.6754	25.7827	25.6659	25.2527
ATP1A2	19.9474	21.0162	20.9258	20.6208	20.4382	19.7964
ATP1B1	19.7233	20.3219	22.0036	21.9036	20.6717	21.4599
ATP1B3	23.2414	24.6948	24.2785	24.2555	24.6215	23.9247
ATP2A2	24.5559	24.8903	24.5967	24.8996	24.92	23.9083
ATP2B1	20.0717	22.8485	20.1373	20.0591	22.5308	20.7364
ATP2B4	16.5032	16.7839	16.4928	16.2109	17.4395	15.3975
ATP2C1	19.2073	20.3459	19.4422	19.7765	20.2329	19.7707
ATP5A1	27.8629	27.8807	28	28.2058	27.841	27.1509
ATP5B	27.2846	27.1917	27.5235	27.8902	27.1493	26.8336
ATP5C1	23.0902	23.3773	23.6523	23.9483	23.3685	23.2605
ATP5F1	22.467	23.1437	23.3859	23.5734	23.2394	22.3217
ATP5H	22.3472	22.2324	22.644	22.8678	22.1553	21.8282
ATP5I	21.7707	21.9675	22.0627	22.2164	21.9686	20.9643
ATP5J2	20.4366	21.1457	21.0525	21.0787	21.2741	20.4333
ATP5L	22.1261	22.6293	23.0458	23.1103	22.7099	21.8229
ATP5O	23.9209	24.2699	24.3315	24.5743	24.1822	23.4654
ATP6AP1	20.3538	20.1168	19.761	19.8056	20.7064	20.4448
ATP6AP2	19.3756	19.1311	19.7188	19.4915	19.2936	19.8988
ATP6V0A1	19.7378	20.0308	19.0699	19.1618	20.0561	18.44
ATP6V0A2	18.0896	17.6727	17.2469	17.2759	17.8164	17.0302
ATP6V0D1	21.9376	22.0081	21.8152	21.9472	21.778	21.2727
ATP6V1A	24.1648	23.7438	23.2035	23.2931	23.5731	23.4985
ATP6V1B2	24.3112	23.9815	23.408	23.316	23.8574	23.7258
ATP6V1C1	20.8517	20.8685	20.9281	20.4027	20.9655	21.1629
ATP6V1E1	20.9584	21.1091	19.686	20.2987	20.7111	20.5219
ATP6V1F	15.9362	16.491	17.4467	17.2158	16.3159	15.924
ATP6V1H	20.8791	20.6722	20.0121	20.004	20.5533	20.294
ATPAF1	19.3443	19.0457	20.4643	20.1224	18.9219	19.6979
ATPAF2	18.0112	17.6698	18.1087	18.196	16.9651	16.8999
ATRX	16.0885	16.6077	16.9864	17.3644	16.4008	11.8893
ATXN10	21.1844	20.8931	21.0186	20.6959	21.013	21.5938
ATXN2	16.761	15.3541	16.6006	16.5287	16.1948	14.8807
ATXN2L	21.7249	20.6405	21.309	21.0053	21.2544	21.5728
AUP1	18.8855	18.4519	20.4279	20.5086	18.935	19.786
AURKB	19.7712	19.6531	20.4636	20.4206	19.5097	19.4612
AVL9	21.882	22.1561	20.4237	19.9413	22.7643	21.4211
AXL	18.4449	20.4428	20.5041	20.4805	20.524	20.3956
B2M	23.6204	23.7692	23.9743	24.1954	22.6936	21.9982
B4GALNT1	18.8016	22.3755	18.8347	18.8358	23.065	18.1081
B4GALT1	18.2023	18.3766	19.3364	18.7224	19.2177	20.6868
BAG2	21.1322	21.0061	21.9073	22.0351	20.6589	22.4581
BAG5	17.0522	16.4509	15.5486	14.3294	16.263	16.6492
BAG6	20.9595	20.4329	20.5164	20.3135	20.8154	20.7764
BAIAP2	15.7021	14.101	19.3038	19.1829	15.0373	18.9458
BAK1	16.7583	18.1922	17.6462	18.8333	17.1607	17.9895

BANF1	19.9095	19.907	19.3936	20.1195	19.7567	19.1055
BASP1	17.3005	20.2117	19.7794	19.4077	20.918	16.6022
BCAP31	21.2792	22.1527	21.5682	22.2199	22.1714	21.8527
BCAR1	19.3993	18.539	19.4391	19.3386	17.7663	19.4443
BCAS2	16.8782	18.4576	18.8928	18.8888	18.0511	17.6314
BCAT1	17.8313	21.4801	20.1076	19.9103	21.5725	20.9351
BCAT2	19.426	19.8863	20.9924	21.3953	19.4647	21.053
BCCIP	19.357	19.628	20.1388	19.7093	19.2483	20.2679
BCKDHB	18.4033	18.7351	18.7053	19.0239	18.1711	18.1345
BCL2L13	20.4139	18.8253	20.0153	19.9499	18.8437	19.6716
BCLAF1	21.8712	21.6881	22.169	22.3043	22.1234	21.8117
BCS1L	18.3679	19.3574	20.0809	20.1408	18.9723	19.6385
BDH1	15.6558	17.8703	17.6423	17.9598	17.3549	18.009
BET1	18.8924	19.3058	19.1048	19.4845	19.8943	17.1966
BID	15.9628	17.5786	18.359	18.2445	17.5283	17.7872
BIN1	20.3769	18.449	19.4781	19.2795	19.0617	17.4607
BLMH	21.8184	20.4017	21.1412	21.0257	20.6054	22.6522
BLVRA	23.7781	21.3157	22.1984	22.0215	21.3326	20.9678
BLVRB	22.6775	19.6526	21.6569	21.6278	19.9404	19.5512
BMP2K	17.4713	17.4435	17.6582	17.4731	16.9834	16.0841
BOP1	21.3361	22.1806	22.5964	22.4372	22.1045	22.7342
BPNT1	19.8443	18.6674	18.7096	17.8442	19.3032	20.0036
BRAT1	18.4404	18.3773	17.8394	18.0175	17.7264	19.3474
BRD4	17.9451	18.6431	18.7189	18.5581	19.0541	17.9778
BRE	18.7102	18.6484	18.3271	18.3992	18.3043	18.9474
BRI3	14.6612	16.6592	14.6128	14.8021	15.0181	NA
BRIX1	17.5429	18.6031	20.4424	19.9256	18.8579	20.5986
BROX	19.5506	18.5675	18.6244	18.224	19.4036	19.4981
BSG	23.8753	24.1944	23.6733	24.033	24.4176	24.6929
BTF3	21.5058	20.9241	21.6059	21.337	20.5214	22.0395
BTN3A3	18.6719	19.7131	18.652	18.6565	19.5015	17.07
BUB3	22.9796	23.0132	23.0337	22.8539	23.1336	23.0248
BUD31	19.918	20.5231	20.002	20.2501	20.4468	20.038
BYSL	22.1651	20.9608	21.0841	21.5294	20.7187	21.3638
BZW1	22.0662	21.6548	21.5909	21.0747	22.4414	22.5084
BZW2	20.6921	21.0597	21.4356	21.3782	21.1679	22.2697
C11orf68	20.566	19.889	19.3745	18.9772	20.1412	19.0972
C12orf10	21.1708	21.0487	20.5888	20.3464	21.4103	20.8067
C14orf1	14.8843	16.0128	15.6586	16.0414	15.7982	14.9885
C14orf159	17.0227	17.0626	15.1087	14.9014	16.5586	13.3384
C14orf166	21.8454	22.1088	21.5618	21.5384	22.0529	21.4224
C16orf58	18.0999	17.5451	19.3114	19.2258	17.7443	17.8718
C16orf62	15.8911	15.9848	15.8726	14.8807	14.2427	10.3691
C19orf70	19.9895	20.5351	20.4155	20.7804	20.117	20.1153
C1orf27	18.5947	18.7349	19.2797	19.1341	19.2281	18.9361
C1QBP	23.1933	23.3109	25.2988	25.2118	23.2923	24.9074
C21orf33	20.1694	18.726	19.5101	18.991	18.538	18.5254
C2CD2	13.6804	14.822	16.6172	16.8749	12.6354	14.0588
C3	17.9348	21.3989	20.2258	19.2743	21.1869	18.033
C7orf50	17.5158	17.1832	19.4691	19.7074	16.4779	20.7017
C8orf82	17.1622	17.2821	17.9566	17.9166	16.4517	17.6054
CA9	17.816	17.1921	17.5363	18.0655	17.6779	21.9527

CAB39	17.8422	19.1265	17.8042	18.1375	18.0251	17.4578
CACYBP	23.8509	23.5737	24.0782	23.8239	23.9847	25.0232
CAD	22.768	22.0053	21.7491	21.5562	22.5983	22.9905
CALD1	25.5792	23.2313	23.4896	24.0531	23.6256	24.3256
CALR	27.5888	28.0807	27.9056	27.9163	27.723	27.0741
CALU	23.7267	23.4103	23.7345	23.6974	23.225	22.6568
CAMK1	15.9012	15.3365	13.9615	15.2241	15.4758	16.6099
CAMK2D	18.0184	12.0613	11.4898	NA	13.949	16.5947
CAND1	24.4812	24.1577	24.1125	23.5809	24.4457	24.3156
CANT1	15.7255	16.967	17.3205	17.3768	17.5586	18.0085
CANX	27.2245	26.7883	27.9521	27.7924	26.917	26.766
CAP1	25.6053	24.5538	23.8797	23.5097	25.3602	25.6033
CAP2	23.7592	23.8962	23.2061	22.8336	24.5178	23.8712
CAPG	17.7453	20.8171	15.6338	15.6325	21.2243	16.7164
CAPN1	21.6301	20.8802	20.4898	19.9664	21.5648	19.6095
CAPN2	24.8439	24.4782	23.4886	23.1408	25.1303	24.6935
CAPN5	18.2529	18.8959	18.7339	18.5878	18.8503	18.056
CAPNS1	22.7806	22.9353	22.0589	21.7229	23.6405	22.6604
CAPRIN1	22.4418	22.2395	22.4824	22.6245	22.3799	23.0608
CAPS	20.7638	19.5737	19.0798	18.9343	20.3328	25.0153
CAPZA1	24.3033	23.8567	23.6572	23.5133	24.1872	24.4367
CAPZA2	23.32	22.9049	22.0593	22.0507	22.9183	22.8337
CAPZB	24.9238	24.7847	24.2267	24.0693	25.1476	24.9034
CARM1	21.6604	21.6479	21.4924	21.2612	22.1739	21.9713
CARS	22.1663	20.6818	21.1245	20.4801	21.641	21.9751
CARS2	19.0299	20.5168	20.5041	20.319	20.889	20.1415
CASK	20.9923	18.4777	18.53	18.9426	18.7652	19.0699
CASP1	22.7635	21.7439	21.294	21.4593	21.9019	21.1063
CASP4	17.943	17.2708	16.3603	15.7425	17.9857	15.6447
CASP8	16.4015	17.3177	15.9715	15.4134	16.754	15.0423
CAST	20.4507	17.3587	18.8462	17.9726	18.264	20.7838
CAT	22.8324	22.3356	22.3745	22.4436	22.1042	23.1993
CAV1	22.7741	24.7292	22.8985	23.0969	24.733	22.5535
CAV2	19.8337	20.2449	18.9329	18.0379	20.9382	19.211
CBFB	20.0353	19.0427	18.9183	19.4856	19.7183	20.6057
CBL	17.9505	16.8451	17.0042	17.1198	17.9643	16.7518
CBR1	24.2208	21.904	21.6337	21.6219	21.8984	23.6661
CBR3	22.5315	18.2842	19.6908	19.0599	18.9671	19.3887
CBS	18.4379	18.2125	20.03	19.275	18.7701	20.2883
CBX1	19.4174	19.783	20.25	20.1257	19.6735	19.4253
CBX3	23.2248	23.3734	23.567	23.5443	23.3459	23.6539
CBX5	21.577	20.8842	21.6378	21.7473	21.084	21.0765
CCAR1	21.3993	21.5981	21.4615	21.6356	21.5711	21.0136
CCAR2	23.3416	22.7588	23.527	23.6722	22.524	23.3613
CCBL2	19.8709	19.3399	18.3054	18.4524	19.5796	20.4527
CCDC124	18.9456	16.8256	17.9883	18.0858	17.1013	19.8239
CCDC134	19.6298	19.9123	20.2508	20.4976	19.7165	18.0136
CCDC22	20.1036	19.0403	19.4887	18.7651	19.9019	19.469
CCDC47	22.6902	22.6342	22.399	22.5309	22.698	23.1025
CCDC51	17.3138	17.7604	18.5334	18.6591	17.8856	18.9609
CCDC58	19.9792	19.7934	20.1288	20.4034	20.0516	20.0456
CCDC90B	16.9508	17.097	17.7551	17.8864	17.1186	14.3743

CCNH	17.0715	18.3953	17.6804	17.4189	18.0903	16.2398
CCNK	19.3661	20.1358	19.7138	19.5022	20.3043	19.9364
CCNYL1	17.7171	18.167	17.8496	17.5424	18.2765	16.7233
CCT2	25.7945	25.1924	25.4791	25.5907	24.9539	26.184
CCT3	26.3128	25.7695	25.9843	26.0036	25.9785	26.7843
CCT4	25.6421	25.2713	25.6098	25.5564	25.2645	26.5186
CCT5	25.4351	24.8094	25.1236	25.2194	24.7409	25.8888
CCT6A	25.8092	25.3086	25.6049	25.6632	25.2313	26.2792
CCT7	25.3563	25.084	25.2886	25.3369	25.1348	26.2399
CCT8	26.4078	25.8333	26.1346	26.0861	26.0582	26.842
CD109	22.7007	22.1336	22.6028	22.1506	22.1739	21.7853
CD151	20.703	21.5066	21.0353	21.034	21.5559	20.5509
CD276	20.0469	19.5346	19.5583	20.0042	19.2235	19.5681
CD2AP	17.1025	16.7089	17.3403	16.719	17.1435	17.983
CD2BP2	18.0117	17.9417	18.6035	18.8945	18.1465	18.4866
CD44	23.8442	24.0279	25.0242	24.8225	24.1741	23.6736
CD47	21.9839	22.9222	22.4537	22.6633	23.1819	21.8996
CD59	21.7318	23.1675	21.6119	21.7015	23.8312	21.5729
CD63	21.0089	21.4	18.7101	20.1904	20.9091	17.7245
CD74	14.251	15.1688	14.8168	13.4128	14.5942	14.6598
CD81	22.3574	22.9353	22.7398	23.0005	22.3216	21.5928
CD82	17.9731	20.2955	19.6012	18.1142	20.5898	15.5739
CD9	20.6626	18.067	20.8288	21.4509	19.2942	20.1429
CDC123	17.7129	17.2839	17.954	17.8459	17.7419	18.4919
CDC16	16.4092	15.2463	16.1737	15.9617	16.0405	15.7816
CDC23	20.0195	19.8575	19.9366	19.758	20.0875	19.7654
CDC27	16.7001	16.9713	18.0095	17.6403	16.3193	17.3832
CDC37	20.6611	21.2084	20.1765	20.2692	21.137	22.0479
CDC40	18.2451	19.1912	19.0226	19.4638	19.1389	19.5061
CDC42	22.8892	23.7824	23.7253	23.824	23.4958	23.8088
CDC42BPB	19.3397	19.8268	18.575	18.5409	20.0932	17.9137
CDC5L	21.4383	21.5879	22.1958	22.2822	21.7795	22.0921
CDC73	20.3277	20.4627	20.9069	20.8321	20.3173	20.5341
CDCP1	15.5218	21.397	13.4112	16.3124	22.3343	15.3503
CDH11	17.0224	15.8467	16.6535	16.779	15.8706	14.9967
CDH13	14.549	20.9162	17.8507	18.0497	21.4365	16.9048
CDH2	17.4248	18.1179	18.9298	18.7097	18.6438	18.562
CDH6	16.3488	11.9962	18.9949	18.8826	13.6658	16.2007
CDIPT	17.8728	17.2137	17.3981	18.3623	16.22	16.9621
CDK1	21.1815	20.1442	21.0411	20.7368	20.4147	21.8217
CDK11A	20.5166	21.1236	20.5672	20.432	21.3734	20.8717
CDK13	15.9875	17.9316	17.1543	17.2377	16.963	13.6922
CDK2	19.1078	18.3763	18.8562	18.911	18.038	19.0193
CDK4	21.2737	26.1402	20.9803	21.0174	25.9846	20.8954
CDK5	19.5765	18.7401	18.774	18.9272	18.8763	20.0657
CDK5RAP1	16.1615	15.9952	17.9132	17.3453	16.1571	16.353
CDK5RAP3	20.1919	20.2415	20.3779	20.2275	20.3927	19.7913
CDK6	26.5082	21.2058	21.8027	21.6015	21.8737	22.7233
CDK9	15.6541	18.6037	18.6567	18.406	18.7378	18.5653
CDKN2A	20.8732	20.077	11.7085	10.3823	20.251	12.0221
CDS2	16.9668	17.5923	18.949	18.9743	17.0312	16.2858
CDV3	19.2204	15.8142	17.5618	17.59	18.1754	18.8829

CEBPZ	15.7478	17.1099	17.7325	17.6079	17.8072	17.9804
CECR5	25.2897	23.4447	23.8346	23.6547	24.043	25.1455
CELF1	21.5015	21.6187	21.6815	21.7953	21.3101	21.6456
CENPB	21.4046	19.5564	22.2576	21.4621	18.8256	18.0269
CEP41	18.9971	17.3813	17.2931	17.5655	17.1444	16.731
CERS2	21.6958	20.9343	21.9128	21.9466	20.9763	21.3837
CES1	16.3557	16.3089	18.1226	18.3191	16.3984	16.8706
CFL1	26.5429	26.2798	25.7226	25.8442	25.9374	26.3881
CFL2	21.8792	22.2476	20.1681	20.1439	22.3148	21.1749
CHAF1A	16.8735	15.8669	17.7998	18.0027	14.757	16.4767
CHAF1B	17.0053	16.4029	17.4815	17.9754	15.9982	16.9992
CHAMP1	18.5309	18.5995	19.3413	19.494	18.9196	19.3551
CHCHD2P9	16.7426	12.6951	17.9459	17.9962	13.6755	16.8212
CHCHD3	21.64	21.638	21.8914	22.2242	21.8656	22.139
CHD1	NA	13.1641	16.8456	16.9851	15.3762	17.2819
CHD4	19.7346	21.7452	20.8835	21.1381	21.792	20.8025
CHERP	19.9761	20.826	20.221	20.3075	20.54	20.1576
CHID1	17.9242	18.9239	20.4844	20.6425	18.0167	19.3062
CHL1	19.5157	11.6164	13.721	13.5663	11.4459	11.9356
CHMP5	18.1275	16.6939	16.1222	15.6138	17.8664	19.1584
CHORDC1	21.6051	21.3609	22.3057	21.8843	22.3251	22.4421
CHST14	17.9007	18.4321	17.8751	18.3956	18.4174	17.4604
CHTF18	12.1837	13.5848	14.7701	14.596	11.2651	13.1879
CHUK	16.9039	13.5323	15.8173	14.9459	15.5268	18.134
CIAO1	18.5946	18.0556	18.2925	17.9106	18.3294	20.0056
CIAPIN1	21.5992	18.9276	20.6922	20.4004	19.6608	22.486
CIRBP	20.7803	20.7915	20.5176	20.6421	20.0653	20.6086
CISD2	21.5655	21.6696	22.1053	22.2197	22.0813	21.9358
CIT	16.2859	14.8195	16.4681	16.9794	15.3391	12.235
CKAP4	27.0527	28.09	27.1723	27.1632	28.4094	26.4988
CKAP5	20.7853	21.5914	21.0842	20.8048	22.0167	22.0366
CKB	23.1206	20.5964	20.173	20.4826	20.6302	24.8517
CKMT1A	15.9955	14.0086	16.7218	12.9574	14.6339	19.1367
CLASP1	19.4596	18.3421	19.6306	19.0078	18.5128	18.0599
CLCC1	18.3965	17.2344	18.9421	18.7088	17.0869	18.7751
CLCN7	18.06	18.3332	17.7046	18.3498	17.5887	17.3521
CLIC1	25.7774	25.1631	24.4116	24.1641	25.5876	26.0073
CLIC4	23.3968	23.5588	22.774	22.7401	24.1718	23.7794
CLINT1	21.0649	20.6188	21.0211	21.0427	21.4004	21.8384
CLIP1	18.6177	16.1266	16.1806	16.3132	16.5761	16.8253
CLIP2	19.2496	18.4234	18.068	17.986	18.7321	17.9624
CLPB	21.2705	21.7042	21.6126	21.5916	22.0317	21.2222
CLPP	18.9608	19.3359	19.319	19.8948	19.991	20.5968
CLPTM1	18.9756	19.7823	20.2549	20.175	19.4105	19.11
CLPTM1L	18.5204	19.3352	19.1154	19.1477	18.3866	19.6226
CLPX	21.9055	22.3307	22.456	22.6837	22.4534	22.4812
CLTA	19.2323	18.7268	18.226	18.3902	19.3332	19.6605
CLTC	28.5916	28.1713	27.9819	27.8682	28.3377	28.4978
CLU	18.6865	16.6669	19.1621	18.7422	17.5101	17.4214
CLUH	18.2644	17.9529	19.5157	18.9839	18.338	20.3336
CMAS	21.1561	22.0663	22.3264	22.3223	22.1635	21.2932
CMBL	21.1637	17.7813	20.4458	20.1259	18.1496	19.3351

CMPK1	19.7393	18.3457	18.7408	18.8782	17.1548	18.9537
CMTR1	19.5669	19.8758	19.0678	18.9294	19.4637	19.0952
CNBP	21.6865	21.5229	21.1765	21.3555	21.9064	21.7377
CNDP2	24.1926	22.6996	22.6902	22.6323	22.8263	22.0418
CNN2	22.4898	22.8805	20.8313	21.5438	22.274	22.2203
CNN3	22.4803	21.28	21.921	22.0087	20.8568	24.381
CNOT1	20.074	20.0619	20.1951	20.2395	20.1554	19.8039
CNOT7	17.8651	17.809	17.975	17.851	17.9025	16.0574
CNP	22.3043	22.7736	22.9129	23.2957	22.6613	23.2664
CNPY2	21.0828	21.1796	22.2185	22.3451	20.2762	20.3118
CNPY3	19.0373	18.4211	17.9145	18.0265	19.0403	18.7218
CNTNAP1	19.4706	21.8156	19.854	19.7176	21.6576	18.227
COASY	17.9972	17.3972	18.1973	18.1989	17.1404	18.7374
COG1	18.5265	17.1446	17.8141	17.4693	18.4892	18.4079
COG8	18.0029	17.6738	17.4483	16.5289	18.1906	17.8027
COIL	14.1487	16.4079	17.9247	17.544	16.9077	16.3601
COL11A1	14.3558	15.6607	15.9876	15.3058	14.9773	18.7881
COL12A1	19.1343	19.0646	19.7052	19.9868	19.1349	18.9142
COL18A1	18.222	17.269	18.942	19.8526	16.3077	19.8548
COL1A1	21.4228	24.0881	20.191	20.5096	22.119	20.2778
COL1A2	21.5543	24.018	21.389	22.4117	22.5556	21.1243
COL6A1	23.551	26.0486	23.2582	23.1494	25.3405	21.6162
COL6A2	21.8741	24.7415	21.4654	21.7547	23.8973	19.8321
COL6A3	24.4982	26.4953	27.1045	27.2334	25.3359	23.0893
COLEC12	17.0768	18.354	17.7638	17.0829	18.094	16.2743
COLGALT1	23.5131	24.9716	23.5362	23.5685	24.7398	24.3984
COMMD3	17.765	17.3239	17.1919	16.8947	17.5286	17.3649
COMMD4	17.7643	17.5218	18.0811	17.203	18.0596	18.0078
COMMD9	17.4261	17.4524	17.5008	17.7355	17.3902	17.497
COMT	24.0108	24.5993	23.8143	23.8897	24.2899	23.7167
COPA	24.2892	25.0367	25.1001	24.7976	26.0484	24.8955
COPB1	24.2563	24.472	24.1134	23.8471	25.1412	24.1244
COPB2	24.9884	25.2467	24.775	24.3353	25.7065	24.5889
COPE	23.3112	23.438	22.9387	22.8315	23.9749	23.0557
COPG1	24.115	24.3218	24.0877	23.7071	24.9291	23.8086
COPG2	20.0366	20.2752	20.0831	20.2168	20.3601	20.7224
COPS2	22.8904	22.518	22.6293	22.6257	22.7171	22.9484
COPS3	20.363	20.0686	19.3803	19.6806	19.9989	20.2171
COPS4	22.3587	21.7026	21.711	21.671	21.5974	21.9808
COPS5	21.9095	20.6521	21.1705	20.8506	20.7859	21.281
COPS6	22.4049	21.8981	21.5621	21.4754	22.1168	22.0551
COPS7A	21.2123	19.751	19.2024	18.8232	20.193	19.9848
COPS8	20.4126	20.7849	20.5048	20.1513	21.19	20.7707
COPZ1	21.1785	21.5438	20.717	20.89	21.8211	20.6958
CORO1B	23.3589	23.0074	22.7442	22.5716	23.2544	23.0327
CORO1C	24.5067	24.826	24.5589	24.1906	25.4948	24.8066
CORO2B	18.3449	16.2682	11.9097	11.2184	16.6152	12.6678
CORO7	18.3181	15.8282	14.8587	15.4634	16.2994	16.3232
COTL1	23.1183	22.8666	22.3377	22.1503	23.3166	23.7087
COX11	16.0911	17.7506	18.478	18.7545	16.9989	16.8687
COX4I1	21.9622	22.16	22.6271	23.131	22.3969	22.3706
COX5A	16.3034	17.3895	17.9156	18.2433	16.6826	15.2421

COX5B	21.2438	21.7308	21.6588	22.0253	21.8344	20.9339
COX6B1	18.0274	18.7962	19.9265	20.0676	18.4833	17.7764
COX6C	18.2118	19.8487	20.0349	20.4247	20.2508	19.0534
CP	18.8449	12.5355	15.3743	16.216	11.3884	13.1493
CPA4	17.159	19.0887	18.7132	17.8237	19.0851	18.1078
CPD	19.6022	19.6976	21.4713	21.6911	19.5227	20.2609
CPNE1	21.9251	22.7923	22.89	22.7754	22.8611	23.163
CPNE3	22.32	21.3073	22.1263	21.7771	21.5195	23.5312
CPOX	23.4032	22.5248	22.9675	23.3637	22.2337	22.0522
CPQ	19.7206	17.7563	17.5783	17.2575	17.2799	16.5864
CPS1	16.5444	17.7158	18.5362	18.3519	17.2576	14.8344
CPSF1	18.9	19.8933	19.8916	19.7904	20.0368	19.4156
CPSF2	20.9937	20.7796	20.7511	20.6968	20.5646	21.1678
CPSF3	21.0795	21.1783	20.8519	20.758	21.2018	21.4682
CPSF6	22.5814	22.3805	22.9766	23.5866	22.3249	22.8719
CPSF7	22.7932	23.1467	21.8017	22.1845	23.3369	22.1992
CPT1A	21.4864	19.6502	21.1792	20.6653	20.555	19.327
CPT2	21.1048	20.9555	21.1136	21.3979	21.176	20.697
CRAT	19.9943	17.2937	19.2375	19.3323	16.8837	19.2864
CRELD2	18.9446	18.2985	18.6217	18.4538	17.9306	15.9542
CRIP2	19.6938	19.3922	15.0211	14.9573	19.8048	19.8028
CRK	20.2605	19.129	18.9712	19.076	19.6381	20.6728
CRKL	20.5863	19.7624	20.0023	20.0467	19.9364	20.5418
CRNKL1	18.6532	19.4964	19.6167	19.6538	19.3602	19.3299
CROT	20.739	17.4522	21.1504	20.8783	17.2389	17.8467
CRTAP	23.5678	24.5093	24.4334	24.7632	24.0519	23.0656
CRYAB	20.7356	17.9091	16.8061	17.14	18.3233	22.0188
CRYZ	21.6592	22.8342	22.3539	22.2526	23.1107	23.1689
CS	24.7875	24.7075	25.1347	25.5537	24.8353	25.1315
CSDE1	24.0237	23.5799	23.8566	23.8264	23.6759	25.0907
CSE1L	24.1352	23.7618	24.9338	24.6718	24.1225	24.8554
CSNK1D	17.7378	17.5898	17.1696	17.2923	18.2062	18.8961
CSNK2A1	23.1353	23.2035	23.463	23.457	23.2373	23.0314
CSNK2A2	20.7351	20.5378	20.8825	21.162	20.5797	21.4431
CSNK2B	23.1753	22.5608	22.9283	22.7828	22.4414	22.9165
CSPG4	21.9507	23.6154	21.816	21.6219	23.679	22.0873
CSRP1	22.9684	22.9787	22.0892	22.0274	23.7933	24.355
CSRP2	20.2841	16.718	17.646	17.0741	17.2046	19.3805
CSTB	21.084	20.2284	19.4625	19.4856	20.79	20.3578
CSTF1	21.2086	21.668	21.2032	21.6271	21.2445	21.6148
CSTF2	20.7075	20.416	20.9621	20.9608	20.7932	21.6754
CSTF3	20.8646	21.2084	20.8897	20.8059	21.179	21.0386
CTAGE5	17.6012	16.5594	14.9597	15.3677	17.4599	17.1046
CTB-50L17.10	19.7171	19.5358	19.4707	19.482	20.4038	19.9362
CTBP1	22.335	21.5793	20.9892	21.3505	21.0366	21.8192
CTBP2	19.787	20.2975	20.7503	20.7536	20.2181	20.8733
CTDSPL2	15.1278	12.6091	16.4905	16.8422	13.7933	16.8005
CTNNA1	22.1731	22.3838	23.3133	23.1038	22.8567	23.7457
CTNNB1	22.204	22.4908	22.5824	22.6832	22.5403	22.8021
CTNNBL1	19.7855	20.6639	20.6883	20.6253	20.4028	20.1899
CTNND1	22.9978	23.2243	23.5727	23.5161	23.6456	22.8708
CTPS1	22.675	21.7281	22.4487	22.1684	22.3446	23.6287

CTR9	19.8177	20.5152	20.7115	20.6592	20.3473	19.9197
CTSA	22.4744	23.3417	23.1937	23.4057	23.1878	22.2439
CTSB	24.5108	25.7758	24.8835	25.0114	25.5963	24.4196
CTSC	24.3838	20.4278	20.9153	21.086	20.4878	22.2938
CTSD	27.6068	26.0703	26.8929	27.2726	25.844	26.6495
CTSK	16.4818	21.8711	16.6578	17.5046	20.9404	17.0939
CTSS	19.8774	22.9401	22.8889	22.6653	23.5426	19.5743
CTSZ	7.70227	19.0388	18.9361	19.5705	17.5969	8.27929
CTTN	22.7013	22.4559	22.6126	22.1787	23.0904	23.0758
CUL1	18.7588	19.4572	19.7446	19.5693	19.9783	20.597
CUL2	19.3792	19.7585	19.3052	19.0261	20.0927	19.4403
CUL3	19.7016	19.6781	19.6651	19.288	19.8181	19.3255
CUL4A	17.8593	17.8435	18.8718	18.463	17.5044	18.1368
CUL4B	20.9793	20.6973	21.6573	21.2778	21.0101	22.1998
CUL5	17.8317	16.7164	16.774	15.9844	16.8384	16.5453
CUTA	20.6282	20.4099	20.269	20.4496	20.1676	20.9426
CWC22	17.0085	18.2447	18.8796	18.9735	18.0776	17.5935
CWC27	17.9026	18.2176	19.1263	18.9422	17.4859	18.4679
CWF19L1	17.8487	18.5857	18.7617	18.3435	18.9477	19.0147
CYB5B	22.5673	21.6286	22.7984	23.1589	21.068	22.5627
CYB5R1	19.3255	20.6486	21.1676	21.1286	21.2346	20.4302
CYB5R2	18.9609	20.426	19.8645	19.116	20.7211	18.8988
CYB5R3	24.2472	24.3385	23.9735	23.88	24.5469	22.8277
CYC1	22.6819	23.0719	23.4782	23.7692	22.9816	23.2768
CYCS	23.6764	22.968	22.5766	23.3897	23.302	23.118
CYFIP1	21.6234	21.925	21.6307	21.3757	22.5429	22.4112
CYP2S1	18.3767	17.4583	19.4655	19.8326	17.0099	19.268
CYP4F11	16.8027	17.0929	21.0936	21.2885	17.3403	17.5014
CYP51A1	19.4303	20.8422	19.9795	20.0918	21.2792	19.6579
CYR61	18.393	20.2559	18.9649	18.7588	20.43	20.0541
DAB2	20.3629	21.1767	20.3054	20.1637	21.4397	19.4759
DAD1	21.3136	22.4989	22.0383	22.0778	23.1319	21.5861
DAG1	18.6468	18.4294	19.7839	19.5758	18.6488	19.8348
DAGLB	16.1662	17.6983	16.9581	16.755	17.3591	14.0947
DAP3	22.4027	22.7709	23.2815	23.2564	23.0534	23.3736
DARS	25.5379	24.282	24.0428	24.0225	24.6544	25.4335
DARS2	22.0443	22.3094	22.426	22.5793	22.4612	22.3069
DAXX	18.5638	18.7537	18.7376	19.3736	18.5257	19.2409
DAZAP1	22.3322	22.6934	22.3409	22.3796	22.7923	22.825
DBN1	22.8062	23.7368	23.7631	24.0183	23.9579	22.2991
DBNL	20.7602	19.4923	19.9997	20.0138	19.8826	20.4348
DBT	19.4736	19.7042	20.0832	20.3587	18.7704	19.4919
DCAF7	17.5696	16.2494	17.1138	17.183	16.0279	17.6652
DCBLD2	18.1581	20.8047	20.5868	20.1399	20.929	21.654
DCK	19.8536	19.065	18.3869	18.267	19.7065	19.6792
DCLK1	19.3357	14.57	14.4489	13.1719	15.9683	17.655
DCPS	20.8792	20.9214	19.7641	19.6561	20.7707	20.9481
DCTN1	22.6715	21.5317	21.9122	21.5285	21.8112	21.8339
DCTN2	22.5505	21.2328	21.7477	21.4385	21.5409	21.8098
DCTN4	19.4364	19.0966	18.4519	18.4097	19.3773	19.4705
DCUN1D1	19.6051	19.5186	19.1059	18.74	20.0194	20.2646
DCXR	19.9583	19.2111	20.1532	20.3527	18.9424	21.7879

DDAH1	19.7671	20.1758	18.6532	18.1883	19.9689	21.4719
DDAH2	19.6071	18.436	15.4166	15.8174	18.4245	18.0389
DDB1	25.2352	25.2108	24.8966	24.6343	25.4431	24.8657
DDB2	18.7921	18.566	17.731	17.791	18.6935	18.0032
DDOST	25.5409	26.3794	25.954	26.046	26.2407	25.1376
DDRGK1	19.402	19.2678	19.4339	20.0295	19.1439	19.5096
DDT	20.1495	19.3369	19.2412	19.6702	19.6658	18.7272
DDX1	25.0377	24.8249	24.2068	23.9986	25.0571	24.6951
DDX17	25.0566	25.1781	24.658	24.9868	25.0982	24.4769
DDX18	18.9677	20.4565	20.9744	20.5909	20.898	21.8046
DDX19A	22.3098	21.1912	21.3776	21.2622	21.5606	23.4239
DDX20	18.4672	18.4792	19.3915	19.5049	18.4645	19.2451
DDX21	21.1317	23.1373	22.741	22.385	23.1953	24.938
DDX23	21.2685	21.796	21.9146	21.8964	21.6467	21.3712
DDX27	19.4308	21.4579	21.6358	21.6802	21.1878	22.1439
DDX39A	22.2351	21.6593	21.251	21.3796	21.1489	21.4291
DDX39B	22.6536	23.3389	23.2544	23.773	22.9143	23.1314
DDX3X	23.8955	23.4288	23.1262	22.9181	23.5428	23.958
DDX41	19.811	19.4642	19.8327	19.6761	19.6255	20.1058
DDX42	23.1081	23.8376	23.5665	23.7184	23.6943	23.3252
DDX46	22.9222	23.0787	23.2295	23.438	22.8745	23.0162
DDX47	16.8911	18.8873	19.4245	19.315	18.6647	19.8183
DDX5	25.1577	25.2125	25.5442	25.5044	25.2396	25.9354
DDX50	18.0521	17.6097	18.5177	18.3918	17.3146	19.0413
DDX56	17.0075	19.3539	19.3513	19.4054	18.6822	19.9934
DDX6	23.3964	22.8747	23.5295	23.4997	23.0201	23.1659
DECR1	23.3021	23.1021	22.9999	23.3028	23.2253	22.3155
DEGS1	19.5451	20.0073	20.0843	20.5201	19.3229	19.6093
DEK	21.1461	20.6063	21.7277	21.5349	20.9791	22.5676
DERA	20.1388	18.8293	18.3008	18.1582	19.3469	18.9042
DERL1	19.8725	20.6936	21.0945	20.9731	21.1761	19.9416
DERL2	19.0406	19.9805	20.3138	20.4408	20.157	18.3253
DFFA	16.6495	15.6985	16.1888	16.0666	15.7494	18.1472
DFNA5	18.5038	20.4876	18.038	17.8274	21.3269	18.3442
DHCR24	21.3732	21.7212	23.0973	23.3017	21.8256	23.6437
DHCR7	20.7531	21.0772	21.8751	22.0529	21.5006	22.1197
DHODH	19.591	18.3961	20.6214	20.6046	18.63	21.2861
DHPS	16.5716	18.497	17.1033	16.6114	18.4937	18.6252
DHRS7	18.164	19.0442	19.1043	18.9507	17.6945	16.9621
DHX15	25.3988	25.301	25.0361	25.0072	25.1623	25.4576
DHX16	19.6474	19.435	20.4346	20.3016	19.0272	19.5085
DHX29	16.35	18.0114	17.7046	17.5838	17.6962	15.9131
DHX30	19.1925	20.3971	20.7748	20.7756	20.9965	21.9554
DHX36	16.5003	16.5084	17.7845	17.2822	16.5053	18.2451
DHX37	17.493	18.2988	17.9527	17.8535	18.203	18.1465
DHX38	21.3691	21.0437	21.4358	21.1713	21.308	21.1381
DHX8	14.8866	16.2857	16.4312	16.1595	16.3143	15.665
DHX9	25.5311	26.2095	26.3286	26.4008	26.233	26.0893
DIABLO	18.3776	18.2003	18.5424	18.7211	18.5257	18.1687
DIAPH1	21.426	20.6216	21.3678	20.8871	21.151	20.8544
DIDO1	17.9491	18.463	17.8363	18.4873	17.9826	17.3543
DIEXF	13.1717	16.715	17.174	16.7131	16.6385	17.8376

DIP2B	15.7555	16.2451	15.6779	15.7447	17.3553	16.3332
DIS3	19.8961	20.3426	20.2708	20.0396	20.5059	19.5223
DKC1	17.1778	18.4782	17.935	18.0845	18.6817	19.8524
DLAT	22.5012	22.217	22.5838	22.7825	22.2594	22.5443
DLD	24.1755	23.925	23.9428	24.4042	23.895	23.9404
DLG1	20.5811	19.8778	20.61	20.5159	19.5911	20.309
DLST	23.9573	24.0097	23.0632	23.0955	24.6163	23.6605
DNAAF5	17.7516	18.3387	19.3707	19.1625	18.0429	19.9216
DNAJA1	22.2213	21.468	21.9346	21.8113	21.1949	23.3953
DNAJA2	22.9024	21.962	22.6043	22.535	22.1326	23.4285
DNAJA3	21.7722	21.5001	22.1558	22.2784	21.5597	21.5005
DNAJB1	21.5449	21.3247	21.6046	21.531	21.504	23.8241
DNAJB11	21.9588	22.5236	22.443	22.8625	22.4289	22.502
DNAJB4	18.4399	19.9115	19.6768	18.9702	20.5838	20.261
DNAJB6	18.413	19.7757	19.3207	19.4502	19.2514	19.1758
DNAJC10	20.8327	22.3871	21.4455	20.8685	22.7422	21.3862
DNAJC11	20.0564	20.2374	21.0749	20.9943	20.6034	20.9529
DNAJC13	20.3369	21.2841	20.801	20.6867	21.784	19.7131
DNAJC19	16.8873	17.998	18.7913	18.8986	17.9189	18.7955
DNAJC3	22.0368	22.1979	21.8862	21.9866	22.1347	20.6447
DNAJC5	17.11	19.0024	18.9585	19.0041	18.8654	19.0057
DNAJC7	20.5474	20.685	20.6596	20.7073	20.4088	21.8207
DNAJC8	21.6696	21.9119	21.0053	21.3239	22.1452	21.6908
DNAJC9	18.6625	18.4277	18.4324	18.255	18.6738	19.8747
DNM1L	24.3504	22.9945	23.4711	22.9677	23.7596	24.9297
DNM2	22.8986	22.3487	21.8556	21.4472	22.3399	22.556
DNMT1	17.4077	18.5166	18.0111	17.6787	18.4907	18.5429
DNPEP	21.9539	20.4008	20.782	20.4166	20.729	20.5751
DNTTIP2	17.154	18.501	19.3372	19.3552	18.3279	19.1945
DOCK1	18.9124	18.0343	17.6418	17.4721	18.0744	17.6677
DOCK10	18.8892	21.5759	20.9837	21.1215	21.6469	20.142
DOCK7	20.806	20.7407	20.2533	20.0724	21.1091	20.0665
DPF2	18.6442	18.6538	18.7394	18.9679	18.8814	18.5778
DPH5	21.092	19.6255	20.2854	19.7945	20.2494	21.3812
DPM1	20.092	21.4972	21.6054	21.6266	21.0472	21.0003
DPP3	21.9434	19.9856	20.7797	20.5341	20.3173	21.8858
DPP4	12.5755	19.3367	12.6112	12.7151	18.9392	11.5432
DPP7	18.3582	18.476	18.2894	18.594	17.9993	18.3558
DPYSL2	26.5882	25.1386	24.5361	24.3816	25.0747	24.2295
DPYSL3	24.9254	23.0261	23.9584	23.6917	22.8901	23.0059
DRG1	20.1314	20.5453	20.3725	20.3944	21.023	21.6366
DRG2	19.7093	18.2409	18.6608	18.219	18.7304	20.0905
DSG2	15.8547	18.8253	16.4645	15.6936	18.5193	16.4939
DST	23.914	24.2497	23.8486	23.6774	24.3154	22.9994
DSTN	24.6003	23.3974	23.6911	23.8764	23.7235	24.4571
DTD1	17.6038	18.8383	19.8598	19.1385	19.2907	19.5206
DTX3	20.1514	24.3462	19.0417	18.7816	24.5461	19.5735
DTX3L	18.6194	16.8903	16.8681	16.6311	15.1652	17.5078
DTYMK	23.1269	22.5757	22.4152	22.425	22.7485	22.9358
DUSP3	19.103	18.3413	17.4478	17.3752	19.1319	19.0441
DUT	22.1376	21.1452	21.9251	21.7832	21.3396	22.2022
DYNC1H1	25.2721	25.2843	25.1423	24.9938	26.0526	25.2791

DYNC1I2	22.6384	21.8795	21.536	21.4597	22.1987	21.9069
DYNC1LI1	22.994	21.8115	22.005	21.2966	22.4817	22.8419
DYNC1LI2	22.9048	21.9415	21.7545	21.6413	22.529	22.4572
DYNC2H1	27.8271	28.5744	27.3476	27.2227	28.8338	28.0603
DYNLL1	20.57	19.2717	19.1755	19.476	19.6155	19.4896
DYNLT1	17.2734	17.2609	16.8206	16.0521	18.2891	15.0747
DYNLT3	19.7724	17.3492	16.6847	16.9168	17.8184	18.3247
DYSF	12.9902	13.6251	13.464	13.4284	13.1635	14.0186
EARS2	17.2196	18.8202	19.3503	19.7414	18.8487	19.1309
EBNA1BP2	19.3706	20.687	21.9324	21.8641	20.8699	21.977
EBP	19.6701	19.0973	19.4549	19.4927	19.6115	19.4606
ECD	15.7887	14.8363	17.6145	16.9056	15.2922	17.7789
ECE1	20.2507	21.3637	20.5112	20.6088	20.8091	19.9834
ECH1	23.4454	22.9078	22.7934	23.3425	22.3775	22.5429
ECHDC1	20.775	21.0645	20.5249	20.4589	21.222	21.8322
ECHS1	23.3852	23.0987	23.5845	23.9556	23.2639	23.8405
ECI1	20.1075	19.235	20.4265	20.8766	19.0369	18.6653
ECI2	21.4627	21.633	21.0244	21.1723	21.7339	20.8885
ECSIT	18.3849	18.8066	18.4454	18.7501	18.9026	18.2936
EDC3	19.6992	18.5573	19.7776	19.455	19.0035	20.1325
EDC4	21.9419	20.6246	22.1253	22.0852	20.8557	22.3255
EDEM3	17.7022	18.3961	17.952	17.6354	19.0923	18.1527
EDIL3	21.098	19.3388	20.4883	20.7051	19.6108	20.2805
EEA1	22.9505	22.5741	22.1907	22.1446	22.9508	21.2053
EED	17.3375	18.0927	18.8697	19.2104	17.536	17.8952
EEF1A1	28.3845	27.0073	27.0964	27.0988	26.9244	28.57
EEF1A2	18.3615	16.2611	17.6386	18.0166	17.1844	19.5198
EEF1B2	23.1425	22.873	23.1038	22.8075	23.3398	23.7323
EEF1D	25.0982	23.8844	24.7883	24.8063	24.3475	25.6044
EEF1E1	19.9364	18.9128	19.7458	19.7033	17.799	19.39
EEF1G	26.8917	25.9838	26.6534	26.5569	26.4441	26.8877
EEF2	28.1771	27.7849	27.5682	27.2918	28.3272	29.5403
EFHD2	22.2387	21.918	22.1871	22.0993	22.2264	23.1069
EFR3A	17.6678	18.4211	17.721	17.6374	17.9422	14.8218
EFTUD1	17.4289	18.4342	19.0103	18.3775	17.929	18.2339
EFTUD2	24.4791	25.0419	25.2037	25.2279	24.9136	25.0529
EGFR	22.1166	23.3465	23.7138	23.6966	23.6291	21.6346
EHD1	22.6166	23.0794	22.8439	22.7395	23.645	23.1837
EHD2	21.9838	22.5215	21.8793	22.3621	21.8665	21.6296
EHD4	20.4745	20.9733	20.9902	20.4208	21.7664	22.3876
EIF1	19.7861	18.8686	20.0903	19.8774	18.9258	20.3612
EIF1AY	20.2852	20.746	20.4902	20.6021	20.533	20.4372
EIF2A	21.9531	21.8637	22.5371	22.2622	22.3451	23.1519
EIF2AK2	21.7311	21.1079	21.7344	21.3761	20.9714	21.8987
EIF2B1	20.5692	20.5709	20.3688	19.9528	21.1421	21.766
EIF2B2	18.0629	18.0907	17.1491	16.791	18.2882	17.8628
EIF2B3	21.2055	19.8594	20.2362	20.0358	20.3126	20.9544
EIF2B4	19.0668	18.929	19.3549	18.9196	19.6455	20.7431
EIF2B5	20.2797	19.2934	19.6034	19.3833	19.6999	20.2735
EIF2S1	23.9602	23.3805	23.4568	23.4756	23.7726	24.0759
EIF2S2	23.1534	22.5173	22.7323	22.5294	23.0103	23.4264
EIF2S3	24.2193	23.6687	24.0858	23.9466	24.1805	24.8213

EIF3A	24.9355	24.6061	24.9199	24.7718	24.9861	25.0189
EIF3B	24.9675	24.4519	24.9298	24.563	24.8466	25.2475
EIF3C	23.7497	23.5881	24.0014	23.8588	23.9527	24.3007
EIF3D	23.3399	22.8255	23.2408	23.1111	22.9975	23.8174
EIF3E	23.2359	23.2043	23.5766	23.3788	23.4792	24.0674
EIF3F	23.4721	23.0755	23.5203	23.292	23.3032	23.8626
EIF3G	23.4382	22.7523	23.0449	22.8965	23.0589	23.7773
EIF3H	22.4863	21.8562	22.2248	22.2104	22.1937	22.9136
EIF3I	23.7753	23.5471	23.7573	23.7355	23.5239	24.3604
EIF3J	21.592	21.1732	21.2983	21.2509	21.3421	22.0176
EIF3K	22.9574	22.7239	23.0365	23.0466	23.1255	23.5622
EIF3L	23.5907	23.495	23.8562	23.7211	23.8871	24.2986
EIF3M	22.7711	22.9137	23.2491	23.0865	23.2252	23.5502
EIF4A1	25.6861	25.118	25.3251	25.2385	25.7699	26.5495
EIF4A2	21.0301	21.3762	20.0213	20.3786	20.8249	21.8809
EIF4A3	24.2929	24.6358	24.0591	24.2184	24.0818	23.7924
EIF4B	20.6117	20.1681	20.3201	20.4568	20.1133	20.1585
EIF4E	22.0435	21.4711	21.6933	21.6829	21.7207	22.1516
EIF4E2	18.8998	18.4945	18.4448	18.73	17.8578	18.3967
EIF4G1	23.7656	23.516	24.1218	24.0596	24.0122	24.5696
EIF4G2	23.2405	22.803	23.3833	23.2027	22.9239	23.3858
EIF4G3	18.2152	16.6471	17.5257	16.9716	16.9376	17.1064
EIF4H	22.9963	21.0647	22.4791	22.2362	21.3434	23.164
EIF5	23.6157	22.7869	23.9944	23.2791	23.3715	24.478
EIF5A	24.1809	24.5639	24.2751	24.3752	24.6298	25.229
EIF5B	23.6779	23.5557	22.7148	22.8886	23.7361	22.8632
EIF6	23.3282	23.5705	23.9118	23.9653	23.7531	24.3417
ELAC2	19.9095	20.125	21.1579	21.1291	19.6151	21.4273
ELAVL1	24.2909	24.7065	24.6526	24.8267	24.4505	24.7855
ELMO2	19.8323	20.3126	19.8289	19.7198	20.5227	19.8894
ELP2	18.7088	18.0639	17.9108	17.524	18.9116	18.7851
EMC1	22.2924	22.4973	23.5859	23.2917	22.6327	22.9382
EMC10	17.251	17.0636	16.3463	17.7407	15.8825	17.3597
EMC2	20.7891	20.5417	20.7319	20.8407	20.4676	21.2122
EMC3	18.1252	18.0275	19.1742	18.8741	18.4248	18.6533
EMC7	21.2855	21.317	22.0525	21.8061	21.1246	21.3816
EMD	21.7932	21.8762	22.0585	22.3479	21.8649	21.7977
EMG1	19.781	21.8469	21.882	21.8411	21.4711	21.7008
EMILIN1	16.7392	19.338	9.23836	NA	18.3862	9.12702
EML1	20.8844	18.4561	17.4595	17.379	19.1983	16.9817
EML2	19.826	18.0496	15.3597	15.7895	18.9565	14.7223
EML4	19.6997	19.4728	19.3833	18.7871	20.2017	20.4865
EMP3	18.113	16.4876	17.9176	17.5811	15.2394	14.3119
ENAH	22.4174	19.1621	20.0936	19.9776	20.0619	22.2739
ENDOD1	17.3584	19.911	16.5032	16.0264	20.2262	17.7461
ENDOG	14.4726	15.0479	7.65401	14.2898	12.2486	10.9373
ENG	22.4181	23.8112	23.0633	23.1789	23.2969	21.1395
ENO1	29.4688	28.0481	27.5813	27.6185	28.3134	29.6184
ENO2	22.505	18.1997	19.1194	18.7964	18.567	20.9223
ENOPH1	18.1767	16.9009	15.1327	16.0476	15.95	17.5932
ENPP1	17.3986	19.2625	16.4718	16.614	18.508	17.6473
EPB41	15.069	15.3776	18.7879	18.4823	15.0728	17.0345

EPB41L2	22.8654	22.3373	21.6332	21.7401	22.1906	22.9042
EPB41L3	16.7032	21.4301	18.3818	17.7171	21.8574	17.7647
EPDR1	22.5852	20.7471	22.5525	22.7721	21.0612	21.6787
EPHA2	22.0448	22.1584	22.3156	21.9242	22.4243	23.0107
EPHB2	18.4973	16.1	18.3633	17.9834	15.7362	15.9845
EPHX1	27.7759	24.082	27.4791	27.5277	24.0803	25.67
EPN1	15.3725	16.6897	15.933	16.5805	16.6303	17.9112
EPN2	15.7395	16.976	15.0517	15.3319	16.851	15.5866
EPRS	25.6295	24.7292	24.7783	24.6216	25.4172	25.2521
EPS15	20.1205	19.2269	20.2886	19.7035	19.9559	19.6836
EPS15L1	19.692	18.821	19.7571	19.3487	19.2843	19.8575
EPS8	20.3228	17.2672	18.7535	18.4782	18.5661	19.4424
ERAL1	19.3162	20.198	20.7838	21.0192	20.6614	21.5157
ERAP1	23.549	22.3033	22.9913	23.0574	22.1478	21.6174
ERAP2	20.4934	24.4822	23.9607	23.6813	24.4161	20.8962
ERBB2IP	20.6922	20.2545	20.499	20.3497	20.0027	19.4663
ERGIC1	20.8396	21.7896	22.1079	21.9958	21.9276	21.0395
ERGIC2	18.8521	19.5486	19.4521	19.4462	19.355	18.4709
ERGIC3	19.0918	19.8178	20.1709	20.0045	19.6127	18.8531
ERLEC1	19.8637	18.0285	18.5686	18.2638	19.0131	19.4184
ERLIN1	19.8209	20.3475	20.3588	20.6086	20.31	20.0953
ERLIN2	21.5188	21.5429	21.3103	21.4014	21.0409	21.4981
ERMP1	16.9978	17.0894	17.9208	18.4237	16.0288	17.0168
ERO1A	24.1117	24.0032	23.3949	22.9305	23.9649	22.8519
ERP29	24.3116	24.4617	24.0825	24.3974	24.2388	24.2399
ERP44	24.2201	23.7191	23.7595	23.9055	23.9389	23.5042
ESD	23.0916	22.0359	21.653	21.4155	22.436	23.0576
ESF1	20.8293	20.1311	18.2193	18.2328	20.6486	19.9159
ESYT1	23.2096	23.1949	23.5158	23.4307	23.1027	22.9639
ESYT2	21.166	20.7882	21.7857	21.6522	20.6165	19.4837
ETF1	23.8057	22.9421	23.2994	22.9754	23.2424	24.2014
ETFA	23.4351	23.4662	24.3602	24.6987	23.0818	23.3659
ETFB	23.598	23.5567	24.3765	24.6939	23.5886	23.6223
ETFDH	18.5598	18.0231	17.4222	16.9182	17.1316	16.8029
ETHE1	22.2614	21.3261	21.19	21.5047	21.8827	20.1495
EWSR1	22.0311	21.58	21.7124	22.0581	21.9878	21.5843
EXOC1	18.5327	18.247	18.0887	18.665	18.417	19.4411
EXOC2	20.2584	20.4461	19.4837	19.6217	20.2359	19.987
EXOC3	19.3531	18.1453	18.337	17.8219	18.4288	18.8332
EXOC4	21.2697	20.3859	20.1638	20.0823	20.5323	20.7456
EXOC5	19.3887	18.6251	17.8675	17.176	18.9717	18.5659
EXOC6B	19.2142	19.1864	18.3275	18.1113	19.0943	18.3579
EXOC7	20.9629	19.9909	18.7295	18.5688	19.9637	20.0643
EXOC8	19.7236	19.1005	19.1647	18.7029	19.2513	19.3899
EXOG	16.8006	17.9427	17.7993	17.1703	18.3815	18.0122
EXOSC1	18.5885	20.2277	20.2486	20.5303	20.1506	19.9739
EXOSC10	19.1271	21.0706	20.8223	20.7519	20.9293	20.8123
EXOSC2	20.6058	21.1242	21.4375	21.3825	20.9716	21.2328
EXOSC3	18.1338	19.0528	19.8092	20.0284	18.9027	19.9613
EXOSC4	16.0884	18.3137	18.5449	18.6341	17.5899	17.7323
EXOSC5	20.8237	21.3964	21.5363	21.7647	21.1128	21.4162
EXOSC6	20.1473	21.0362	21.3462	21.492	20.929	21.1281

EXOSC7	20.5236	21.117	21.2784	21.495	20.5645	21.0204
EXOSC8	19.1185	19.5983	20.2902	20.143	19.1725	20.6158
EXOSC9	20.0691	20.8985	21.1178	21.2551	20.7333	21.1314
EXT2	15.1591	16.1202	16.7705	16.778	15.437	15.7126
EZH2	17.7767	18.6807	19.7135	19.6627	19.3872	20.047
EZR	23.3286	21.3572	22.5743	21.898	21.8744	24.5227
F11R	16.5526	17.6109	17.6698	17.3158	18.4228	18.1747
F3	18.7198	19.1529	18.9637	18.7191	18.5436	17.9691
FADS2	22.262	22.7446	23.4559	23.2725	23.1373	21.7624
FAF1	20.7848	19.6264	17.4065	17.8141	19.8531	17.9335
FAF2	20.8216	21.6326	22.3291	22.6747	21.5274	21.8287
FAH	21.168	19.7678	20.3572	20.01	20.1103	20.4279
FAHD1	20.3344	20.1747	19.8496	20.194	19.9523	18.682
FAHD2A	20.8048	21.4642	20.0713	20.1119	21.4563	21.0249
FAM114A1	21.7519	21.1895	19.4833	18.9599	21.5985	19.0039
FAM114A2	17.6236	17.3102	16.3455	16.2966	18.213	17.8032
FAM120A	21.7679	21.4229	21.5419	21.3537	21.8583	21.701
FAM126A	17.7643	17.6061	16.698	16.6485	17.5658	15.701
FAM129A	21.5899	20.7927	20.3511	19.7627	22.0027	22.1328
FAM129B	23.3268	23.2	22.4979	22.5577	23.3653	23.3216
FAM160B1	17.767	16.8627	15.3433	15.4155	16.8442	18.0707
FAM208A	16.4019	16.7481	17.2201	16.8765	16.5206	15.3523
FAM21C	17.9672	17	16.9602	16.622	17.079	15.5125
FAM234A	17.0688	16.0304	17.7134	17.8489	15.665	16.3492
FAM3C	18.9919	19.9203	20.4206	20.2698	20.1754	20.7811
FAM49B	20.9592	21.0341	21.5181	21.2568	21.3893	21.8567
FAM50A	20.8838	19.8289	19.7459	19.6182	19.5465	20.4878
FAM91A1	20.1771	20.6002	19.9671	19.9941	20.6858	20.2862
FAM98A	20.9521	21.0707	20.2565	19.9669	21.5343	21.5126
FAM98B	19.9655	20.1355	19.6312	19.4901	20.1187	19.6171
FANCI	15.9911	16.2176	18.1202	17.3062	17.3718	16.3481
FAP	18.5099	22.4209	18.1202	17.7658	22.3369	18.143
FAR1	16.1234	17.193	19.3546	19.021	18.1028	18.7615
FARP1	20.3789	18.9802	18.3944	18.455	18.7445	14.1076
FARSA	21.3656	20.5566	20.8536	20.8436	20.9391	22.0937
FARSB	23.5181	23.1347	23.209	23.0692	23.5501	24.1233
FASN	25.424	25.5451	26.0584	25.8937	25.7635	27.5934
FASTKD2	17.7515	18.2748	19.0133	18.9223	19.0584	19.6545
FASTKD5	18.6481	19.0907	20.0885	20.1005	19.0781	19.8632
FBL	23.3071	24.0555	24.2657	24.2192	24.1039	24.167
FBXO22	17.8054	16.7604	18.2572	17.6353	17.6121	18.6013
FDFT1	20.6269	19.6499	20.6582	21.0005	19.849	20.4076
FDPS	20.6923	20.5205	19.5988	20.0106	20.6345	20.4688
FDXR	20.6571	18.8554	18.927	19.4165	19.0186	19.1245
FECH	17.9583	17.8643	18.496	19.0711	17.6307	17.4094
FEN1	23.3144	23.6888	23.393	23.1393	23.6263	23.7018
FERMT2	23.2142	22.8355	21.1683	21.0183	22.9771	22.0313
FGF2	18.4608	21.7182	18.4433	18.3035	22.1911	17.9884
FH	23.9426	23.8728	24.1867	24.4176	24.1956	24.1986
FHL1	24.042	21.8706	21.5082	21.3628	22.204	21.525
FHL2	23.7114	22.2507	20.6946	20.448	23.1545	20.579
FHOD1	19.1361	19.5394	19.078	18.534	20.0981	20.5227

FIP1L1	18.9216	17.0827	18.3623	18.5216	17.4958	18.9626
FKBP10	23.247	24.0813	23.6691	23.5673	24.033	20.6408
FKBP14	18.561	19.962	19.844	19.8412	20.0669	17.814
FKBP15	18.7314	17.6806	18.4182	17.8754	18.1538	17.5166
FKBP2	24.4006	23.1322	22.7654	22.8735	23.2044	21.9388
FKBP3	20.2158	19.4727	18.6231	18.8977	19.9088	19.4935
FKBP4	24.2368	23.4097	24.1754	23.8079	23.4271	24.3076
FKBP7	18.6683	18.748	20.4853	20.3868	17.5213	16.7682
FKBP8	20.1324	20.895	20.6407	21.1103	20.352	20.6358
FKBP9	24.1217	23.8315	23.5258	23.7355	23.3975	21.8566
FLAD1	16.6261	14.0015	16.2132	15.5663	14.3201	16.0319
FLII	23.1089	22.7235	22.1212	21.6416	22.9516	22.3162
FLNA	28.8083	28.0913	27.7845	27.7548	28.7218	28.0735
FLNB	25.0212	24.9825	25.4428	25.4652	25.5414	26.5978
FLNC	23.2649	25.5267	23.3542	23.3672	25.8733	21.6456
FLOT1	25.7455	25.9529	25.2933	25.1189	26.4154	25.8994
FLOT2	21.8242	21.7798	22.0723	22.4262	21.4355	22.5122
FMNL3	13.6714	18.3541	16.4047	16.2701	19.0938	17.2062
FMR1	20.3268	20.3936	20.3541	20.4176	20.1204	20.3866
FN1	23.6979	27.6231	23.1645	23.7587	26.3215	22.7644
FNDC3A	17.6474	18.2839	17.7567	17.1994	18.365	18.0901
FNDC3B	24.9003	24.2677	23.9477	23.3195	24.4505	24.7906
FNTA	21.1134	20.0712	19.5997	19.2751	20.5171	20.2931
FOCAD	18.4075	18.3834	19.0642	18.2879	16.9495	15.8065
FOXK1	18.7626	19.0422	19.4238	19.5533	18.4826	18.6624
FOXRED1	14.4424	14.6573	17.9948	18.2974	13.7856	17.2147
FRYL	16.9776	16.4436	16.5779	16.0041	17.4149	17.6508
FSCN1	25.3529	25.3331	23.2122	23.2257	25.6348	23.9505
FSTL1	19.5629	20.3573	18.1486	18.2759	20.1154	17.867
FTH1	22.7323	22.4391	22.0059	22.0131	22.8459	21.6543
FTL	23.7597	21.7815	22.7399	23.0328	21.8528	21.7829
FTO	21.771	19.8883	20.1932	19.954	19.9039	21.7002
FTSJ3	19.4594	20.4723	20.672	20.2248	20.6777	21.4891
FUBP1	22.9512	23.0166	23.1499	23.3674	23.3142	22.7741
FUBP3	22.2402	21.9414	22.1691	22.1612	22.3117	22.8065
FUCA1	21.2675	21.0117	21.6403	21.234	21.3102	21.3857
FUCA2	17.8605	15.6945	16.0975	17.4418	14.9088	16.5945
FUS	22.8151	22.5126	22.7024	23.1458	22.185	22.4105
FUT8	15.6978	19.0088	15.3461	15.2647	18.9299	14.8891
FXR1	22.7736	22.7096	23.256	23.3151	22.6999	23.2865
FXR2	20.2198	19.8225	19.476	19.7363	19.5664	20.0505
G3BP1	24.4293	23.6608	25.1181	24.783	24.6507	24.7049
G3BP2	22.1755	21.8601	21.7757	21.7866	21.833	22.7852
G6PD	27.7891	24.7459	26.3095	26.2514	25.059	27.2011
GAA	20.7435	20.0928	20.3224	20.7454	19.6987	17.4363
GABARAP	16.1412	17.4218	16.6986	16.3958	15.9194	14.3164
GABARAPL2	16.8982	16.7126	16.8385	17.0727	15.5652	15.6559
GABPA	17.5165	17.6184	18.0868	18.384	17.3835	17.7921
GADD45GIP1	17.4673	18.332	18.0257	18.4884	18.7939	18.7591
GAK	19.4708	19.4994	19.3474	19.4887	19.6454	19.0613
GALE	19.0145	17.6442	17.8417	16.6856	18.3114	19.591
GALK1	20.247	19.0878	19.4855	18.8007	19.0639	19.2921

GALNS	18.9452	18.7922	19.9192	19.9631	17.9925	17.9462
GALNT1	18.1442	17.7007	17.3807	17.588	17.9564	16.7321
GALNT2	22.6353	23.7552	23.4989	23.7829	22.9581	22.9305
GALNT7	19.3832	18.9171	20.5312	20.1287	19.0963	19.8936
GANAB	27.5315	27.6865	27.4926	27.5495	27.5618	26.5439
GAPDH	30.6257	29.3758	29.0814	28.885	29.5305	29.9497
GAR1	20.613	21.6872	21.4781	21.4941	21.7505	21.839
GARS	24.4107	23.9068	23.5565	23.1264	24.6972	24.4253
GART	22.9555	22.0438	22.9797	22.432	22.4578	23.5304
GATAD2B	19.9534	18.6625	20.3034	20.5117	18.6281	20.4226
GATB	15.8641	15.5678	16.7685	16.4753	14.0297	15.1416
GBA	22.6769	22.8045	22.3793	22.5571	22.3824	22.1355
GBAS	20.7935	20.4205	20.1887	20.9834	20.109	21.1331
GBE1	23.7034	22.6846	22.1216	22.1339	23.0387	23.4776
GBF1	20.6001	20.4896	20.4973	20.0131	21.2924	20.2194
GBP1	18.2838	17.5815	17.5399	16.6358	18.795	17.9388
GCAT	15.2606	16.449	16.9778	17.7288	16.0811	15.749
GCDH	15.8495	17.7699	17.6651	17.7313	16.8232	16.2443
GCLC	21.3396	17.0181	20.5064	19.9443	17.2414	20.0639
GCLM	22.0485	20.3159	21.2821	21.4741	21.3631	22.0288
GCN1	20.8814	21.3236	21.7097	21.5059	22.1838	22.7253
GDI1	23.6486	21.6367	20.5719	20.1454	22.6033	23.2125
GDI2	25.1283	23.8795	23.4222	23.1541	24.5351	25.4236
GEMIN4	19.1542	19.2737	20.1216	19.8017	19.8582	20.6373
GEMIN5	20.5569	20.2802	21.8336	21.4998	20.6111	21.1472
GFAP	13.4068	13.5475	13.9549	13.5057	13.7038	18.4315
GFER	15.236	11.3976	14.6336	15.5066	13.0675	11.9805
GFM1	22.6415	22.8458	23.8452	23.698	23.1566	23.6176
GFM2	17.8638	18.6348	20.4735	20.4122	19.0554	18.8153
GFPT1	23.3919	22.9728	22.9076	22.1795	24.4348	23.767
GFPT2	24.7472	26.703	22.7905	23.835	26.8687	24.0436
GFRA1	12.7175	10.6781	17.3954	18.5693	11.1522	19.6911
GGCT	20.531	19.4653	19.3308	19.1088	19.7625	20.976
GGCX	16.9931	18.9958	18.6701	18.6343	18.6292	16.3768
GGH	21.2427	22.1564	22.3707	22.7154	21.8488	21.2778
GGT5	13.3955	13.9835	17.9625	17.7538	13.349	12.2471
GGT7	17.5794	17.0141	18.0466	17.9814	16.9605	16.566
GIGYF2	18.6724	18.0703	18.9278	18.9667	17.8662	18.5372
GINS4	17.9892	17.816	18.7308	18.2754	18.4207	17.8404
GIPC1	18.7475	19.5574	19.44	19.8341	17.7903	20.6839
GIT1	18.0344	16.7881	17.6034	17.4238	17.1428	18.3809
GLA	20.4747	20.3067	21.5952	21.7174	19.4848	20.1465
GLB1	21.402	22.1384	21.4999	21.8367	21.7803	21.3043
GLE1	15.9709	17.8161	17.5213	17.6865	17.9041	17.5824
GLG1	23.1232	23.1942	23.6092	23.6409	23.0923	22.2535
GLIPR1	17.2845	17.8976	17.9794	17.7002	18.2966	18.1796
GLIPR2	19.4371	17.7522	18.2998	19.2925	17.5278	20.4557
GLMP	16.2111	19.9917	16.3459	15.8576	19.7784	16.5625
GLO1	22.7506	21.4733	21.2833	21.3303	21.7143	23.1977
GLRX3	22.911	21.3129	22.6263	22.329	21.8367	23.4851
GLS	23.2014	24.1021	23.6641	23.8407	23.9672	23.6985
GLUD1	24.6499	23.9779	24.4001	24.6385	24.3584	24.482

GLYR1	19.2008	20.2951	20.0941	20.3066	20.031	19.7423
GMDS	19.0796	16.4023	17.4805	17.1945	16.3288	19.5731
GMPPA	20.9241	21.2293	20.2814	20.2104	21.539	20.6878
GMPPB	20.6597	20.3915	19.505	19.2433	21.0471	20.3183
GMPS	23.026	22.704	23.1393	22.8882	22.8094	23.1701
GNA11	19.6708	20.696	19.9238	20.2414	20.495	20.201
GNA13	20.763	20.9421	21.3749	21.6334	20.6718	20.6924
GNAI1	18.512	18.6545	19.409	19.4954	18.6152	16.2188
GNAI2	23.0567	24.845	24.0037	24.0623	24.8871	24.3955
GNAI3	21.741	22.1332	22.0702	22.1906	22.3667	22.4038
GNAO1	19.2408	10.6909	14.0724	16.2158	12.5366	9.81329
GNAQ	18.0607	19.2949	18.35	18.6914	19.0367	17.7109
GNAS	21.7539	23.6082	22.9954	23.1947	23.6354	22.6139
GNB1	22.9915	23.4444	22.4595	22.9486	23.246	23.2433
GNB2	23.1565	23.5751	22.7994	23.1948	23.5076	22.4942
GNB2L1	26.555	26.7904	26.6661	26.6354	26.9612	27.2387
GNB4	19.3383	20.2252	19.9849	20.2302	18.7048	18.5947
GNE	18.5499	18.5396	18.603	18.3804	19.3039	19.1484
GNG12	19.0337	19.9973	17.7631	18.7673	20.3518	18.4001
GNL1	21.1739	20.3954	19.6944	19.4759	20.8143	21.5013
GNL3	18.9569	21.1237	21.4969	21.1464	21.2645	21.2192
GNPAT	17.7203	18.1638	18.895	18.8037	18.1291	17.1986
GNPDA1	24.5296	24.6357	24.4984	24.9043	24.6124	23.84
GNS	23.0852	23.906	22.844	23.2185	23.8245	22.6184
GOLGA2	19.3624	19.0674	18.9533	18.9545	19.407	18.4111
GOLGA3	19.3722	18.292	18.5574	17.4488	19.2628	18.3697
GOLGA4	18.2322	19.1784	18.1331	18.0027	19.6639	17.7483
GOLGB1	21.0628	21.1857	21.2084	21.1126	21.4421	20.7621
GOLIM4	18.5377	18.9048	18.6065	18.7137	18.8018	16.9079
GOLPH3	18.6405	18.3558	19.4622	19.3801	18.5143	19.4532
GOLT1B	20.2378	20.7702	20.9031	20.5621	20.7104	19.1208
GOPC	18.4004	17.3358	17.9397	17.3622	17.7136	17.5147
GORASP2	20.7824	20.5431	21.153	20.9424	20.3723	20.4754
GOT1	23.0514	21.3004	21.6879	21.4649	21.7019	22.9436
GOT2	25.6709	25.1284	26.1111	26.3201	25.1865	26.4322
GPAT3	19.379	18.3819	20.6693	20.4613	18.976	18.6911
GPAT4	16.5059	15.3959	16.4838	16.8123	16.042	16.9717
GPC1	22.6311	20.1421	22.0519	22.2073	20.3626	21.6757
GPC4	17.4176	18.711	18.8302	19.163	18.879	22.9421
GPD1L	18.3201	16.5769	16.7549	16.6322	16.6795	18.1129
GPD2	24.6745	23.6386	24.2725	24.3271	23.8741	24.458
GPI	26.841	26.646	26.3375	26.1619	26.8856	27.0547
GPKOW	17.9323	14.8926	18.7765	18.5137	13.8948	17.9126
GPM6A	18.0426	12.4074	13.1654	13.5644	12.946	17.9296
GPNMB	20.4084	21.8542	18.5705	18.4442	21.2837	17.4237
GPS1	22.3173	21.5818	21.4707	21.4054	22.0778	22.5866
GPT2	15.9292	13.57	18.4851	18.3311	14.4455	18.3206
GPX1	18.796	19.5119	13.1837	12.3479	19.5957	15.4098
GPX8	19.9929	21.6157	21.4483	21.1998	22.1213	20.3088
GRAMD1A	10.7465	14.4866	16.3602	16.4346	12.9362	13.6063
GRB2	20.7014	20.2791	21.049	20.6017	20.9229	21.8779
GRHPR	22.2535	20.4901	20.9383	21.2272	20.7262	22.3188

GRN	18.3884	19.6649	17.8692	17.9519	19.4742	15.8554
GRPEL1	22.2255	22.3071	22.7932	22.8669	22.5531	22.8628
GRSF1	21.8923	21.6281	22.0743	22.1146	21.8102	22.5134
GRWD1	20.7412	20.7666	21.106	20.8535	20.8283	21.6299
GSK3A	19.1211	17.2322	16.4739	16.6196	17.8013	19.3574
GSK3B	19.6007	19.4019	18.2198	17.8953	20.0713	19.2265
GSN	24.3854	22.8135	21.6741	21.4971	23.0803	21.7004
GSPT1	23.7295	22.4143	22.5004	22.0533	22.557	23.3696
GSR	25.0932	20.9195	23.381	23.2169	21.3317	23.3094
GSS	22.4114	21.0269	20.67	20.6452	21.1051	21.3032
GSTK1	21.3834	21.8099	21.2663	21.7836	21.8022	21.2994
GSTM1	20.1182	16.686	14.4461	15.6103	16.8916	16.0874
GSTM2	18.2764	16.9539	15.659	16.203	15.556	15.048
GSTM3	24.2216	18.9892	21.9531	21.7779	18.5057	22.7093
GSTM4	19.0892	18.0083	18.0706	17.6523	17.1009	16.9572
GSTO1	23.6719	24.4951	23.0749	22.8829	25.3425	24.5275
GSTP1	26.0436	23.7125	24.9234	25.0905	24.0964	24.6108
GTF2B	16.2601	18.0621	17.8099	17.5035	17.7535	16.8982
GTF2F2	17.6874	16.7588	18.7925	18.3549	16.7892	19.825
GTF2H1	15.8111	16.775	16.4386	16.7329	16.0972	12.1504
GTF2I	23.7137	23.621	24.2968	24.2906	23.4606	23.2905
GTF3C1	15.4301	17.1203	17.8809	17.7109	16.3527	17.2071
GTF3C3	18.7041	19.0718	19.9266	19.9007	18.7822	19.5466
GTF3C4	18.6808	18.9674	19.7064	19.4563	19.0264	19.2697
GTF3C5	17.9638	18.1424	18.5478	18.214	17.7057	18.5043
GTPBP10	15.788	17.84	17.7789	17.8304	18.2904	17.7823
GUF1	13.9077	14.6242	13.6422	14.482	12.2669	15.3254
GUSB	19.3534	18.6693	18.8347	19.1617	18.7778	18.2058
GXYLT1	16.7514	17.6102	18.0778	17.6429	18.1308	15.4086
GYS1	19.2796	18.9644	20.1925	19.4042	19.3305	20.7619
H1FX	19.2433	19.4625	19.3374	19.4461	18.8479	17.5752
H2AFY	21.9721	22.576	23.3763	23.1371	23.2253	23.512
H2AFZ	23.4859	23.5865	23.829	23.7832	23.7906	23.8019
H3F3A	20.488	22.3272	22.3895	22.7089	20.9192	20.4489
H6PD	18.454	19.5756	17.8647	18.3352	19.2414	16.2174
HACD3	21.9132	22.5027	22.8367	23.0767	22.3577	22.5903
HACL1	16.9236	19.6211	18.4411	18.3247	20.0487	19.4891
HADH	23.4619	22.8754	23.0865	23.3642	22.6543	21.3756
HADHA	25.1545	24.6232	24.7568	25.0342	24.5405	24.5548
HADHB	24.6081	24.1441	23.8731	24.1243	24.2656	24.0059
HARS	23.0915	22.2792	22.7431	22.4477	22.7075	23.9726
HARS2	17.4494	17.2304	19.3509	19.1832	16.8219	17.2894
HAT1	21.4924	21.0314	21.3116	20.9828	20.8795	21.9491
HBA1	19.0645	16.8378	14.3712	14.5708	17.6021	14.9731
HBD	21.2471	19.5635	17.9139	17.9756	20.0733	18.8665
HBS1L	19.0084	18.9167	18.7092	18.484	19.64	20.4632
HCCS	19.4752	19.49	19.9382	20.768	18.9205	20.8262
HCFC1	22.8212	22.6419	23.1123	23.2107	22.5872	23.1611
HDAC1	21.8575	21.6489	22.0059	22.2699	21.5736	21.7139
HDAC2	17.79	18.6164	18.509	18.7994	16.905	17.2974
HDAC3	18.587	18.4726	17.3746	17.2798	18.4876	19.2659
HDAC6	15.3672	14.0431	14.9524	13.9635	12.1581	11.6548

HDGF	22.4669	22.661	21.8531	21.5973	22.8879	22.6701
HDLBP	24.739	24.7733	24.4741	24.5708	24.9631	24.1407
HEATR1	18.3789	20.0102	20.9611	20.806	19.9982	20.3181
HEATR3	17.2288	17.9056	17.9421	17.8299	17.4657	16.9997
HEBP1	21.3601	21.2995	21.6662	21.9253	21.2528	20.4729
HECTD1	17.4343	17.3166	15.6254	15.375	17.7243	15.5281
HEXA	21.7132	22.2823	22.4722	22.521	21.9473	21.8731
HEXB	24.448	25.1562	25.2774	25.2031	24.9047	24.4502
HGS	20.9131	20.7604	20.1675	20.2115	21.434	20.7001
HIBADH	23.5392	22.8903	23.727	23.8022	23.1022	21.8552
HIBCH	20.6528	20.687	20.5711	20.6127	20.7365	20.5537
HIGD1A	17.4548	17.8827	18.4237	18.8151	16.7218	16.3973
HINT1	20.9165	20.3113	19.8193	20.1837	20.6029	20.6302
HINT2	19.868	19.2929	20.3622	20.7823	19.4381	18.6962
HIP1	21.467	20.3041	20.9957	20.7802	20.3353	19.1004
HIP1R	15.0917	16.9303	15.5537	16.6073	15.7437	15.8443
HIST1H1B	16.7639	19.7311	17.3919	17.567	19.4794	17.8402
HIST1H1C	19.4719	20.8706	19.983	19.5624	20.8897	20.0688
HIST1H2AE	17.8155	19.9412	17.296	20.5693	19.5674	17.2046
HIST1H2BJ	24.8331	24.8819	25.2161	25.3685	24.8037	24.9827
HIST1H2BK	23.3436	23.8415	23.8542	23.5607	24.1267	23.5493
HIST1H3D	21.8383	22.7469	23.5881	24.1196	20.9338	21.4654
HIST1H4D	27.531	28.1288	28.8032	28.7644	28.3752	28.4768
HIST2H2AC	19.5782	21.2767	20.9958	20.4716	21.9255	17.1804
HIST2H3A	21.5422	23.2028	24.0545	23.9261	21.4037	20.8747
HK1	24.2009	25.5121	24.9731	25.1417	25.8525	25.6141
HK2	21.9748	21.3705	21.3902	21.2987	21.2731	21.8481
HLA-A	19.7929	16.2497	20.8598	21.766	16.0597	19.7063
HLA-A	20.088	20.6019	18.2739	19.9713	20.2242	18.1842
HLA-A	16.1641	16.8639	16.0802	16.6107	17.004	16.9274
HLA-A	17.3603	17.5857	9.24914	NA	16.3412	13.1345
HLA-B	15.3531	17.6398	13.3971	15.6214	17.281	19.8945
HLA-B	19.5409	20.1881	22.7302	23.055	19.9729	19.0421
HLA-B	18.3952	15.4462	15.2087	14.8701	15.6887	14.1475
HLA-DRA	22.1195	21.7485	21.7231	21.4831	21.6981	22.312
HLA-DRB1	18.2982	17.5109	18.7049	18.2789	18.314	19.9516
HM13	22.7786	22.9874	23.6285	23.5958	23.6702	23.21
HMBS	17.7715	16.581	17.5312	17.673	15.2729	16.5956
HMG20A	17.8315	18.3579	18.7066	19.3701	17.7484	17.1393
HMGA2	15.4225	19.298	13.6302	13.3716	19.3445	13.2474
HMGB1	23.5383	22.78	22.5328	22.439	22.7912	23.5987
HMGB2	19.0931	18.0265	16.8615	16.7106	18.3809	18.4405
HMGCL	20.9463	21.2566	20.4812	20.6859	21.0314	19.3786
HMGCS1	20.1041	18.2948	19.5633	18.9801	19.0634	20.3567
HMOX1	20.3171	20.6551	20.2176	20.3892	19.3922	21.8035
HMOX2	19.6442	19.9332	20.4601	20.5376	19.4551	19.5206
HNRNPA0	24.2804	24.4835	24.694	25.0325	23.9861	24.3947
HNRNPA1	26.833	27.1706	27.0794	27.3596	26.9006	26.9953
HNRNPA2B1	28.1286	28.178	28.3034	28.5508	28.1575	28.2055
HNRNPA3	25.9974	26.2347	26.5279	26.6835	25.9779	25.9547
HNRNPAB	23.4353	23.3343	23.6551	23.9556	23.3835	22.9393
HNRNPC	26.1507	26.631	26.2378	26.5355	26.6276	26.4907

HNRNPD	24.6967	25.0428	24.6382	24.8945	24.9932	24.7379
HNRNPDL	23.9453	23.9626	24.1284	24.3672	23.8002	23.8897
HNRNPF	23.9861	24.1386	23.8576	24.1919	23.9541	24.4254
HNRNPH1	24.4306	24.6176	24.8933	25.2408	24.0896	24.382
HNRNPH2	21.2133	21.8487	20.768	21.0874	21.682	21.4245
HNRNPH3	24.2547	23.9039	24.1605	24.4213	24.0224	23.4893
HNRNPK	27.3652	27.2987	27.4705	27.606	27.1761	27.5149
HNRNPL	25.1422	25.4726	25.2106	25.4986	25.2556	25.2133
HNRNPLL	20.8781	21.174	20.9186	20.7038	20.9505	20.7153
HNRNPM	25.119	25.1266	25.0752	25.5071	24.862	25.3496
HNRNPR	24.6325	24.7852	24.7914	24.8143	24.7018	25.1006
HNRNPU	26.2192	26.409	26.8052	26.9531	26.3362	26.7473
HNRNPUL1	23.3275	23.5967	23.6556	23.8349	23.4708	23.4838
HNRNPUL2	24.5629	24.9806	24.4391	24.509	25.0009	24.306
HOOK3	18.0291	15.7508	16.5821	16.3609	15.9008	16.3467
HP1BP3	18.7191	18.8744	17.2905	15.8131	19.5778	18.867
HPCAL1	22.3186	22.502	22.8812	22.7972	22.4947	21.6953
HPRT1	22.9473	22.1035	22.0903	21.8094	22.6623	23.9856
HRSP12	18.831	19.3538	20.2005	20.2284	19.1323	19.1091
HS1BP3	19.027	17.485	17.4528	17.3158	16.8884	17.0246
HS71A	25.7346	24.4689	25.3461	25.6467	24.4409	25.9329
HSD17B10	24.7956	23.4555	24.3462	24.6331	23.5047	24.4125
HSD17B11	18.8801	19.3818	18.3155	18.2725	19.1364	16.9347
HSD17B12	19.66	21.0716	18.5295	18.5	20.8467	20.6133
HSD17B4	25.1312	25.66	25.1199	25.3225	25.5649	24.6176
HSD17B7	19.3752	19.6668	19.5437	19.8849	20.096	19.5295
HSDL1	16.6927	17.8492	19.2354	19.375	17.2898	17.3865
HSDL2	21.9725	21.158	21.966	22.1209	20.7864	20.8145
HSP90AA1	27.4316	26.9091	26.7627	26.5852	27.2639	28.4917
HSP90AB1	27.5156	27.1376	27.6628	27.45	27.7096	29.0892
HSP90AB4P	21.1169	20.1879	21.4828	21.3751	20.892	22.9815
HSP90B1	28.7708	28.3443	28.3482	28.2106	28.4878	27.5431
HSPA13	21.2227	20.2028	20.6047	20.4794	20.7856	19.9824
HSPA14	18.6448	18.282	18.619	18.4823	17.9038	19.0004
HSPA2	22.6499	18.518	18.6546	18.2285	18.8825	19.049
HSPA4	25.5255	25.2491	25.4158	25.1875	25.4769	25.8811
HSPA4L	21.5626	20.3843	20.7398	21.0658	20.4012	21.7071
HSPA5	29.4254	29.2132	29.2894	29.2031	29.5068	28.6324
HSPA8	28.293	27.9033	28.0305	28.0473	28.0785	28.624
HSPA9	26.5246	26.4284	27.2221	27.4562	26.8375	27.1627
HSPB1	26.0015	25.5057	26.1072	25.942	25.4107	26.469
HSPB11	21.2121	20.3593	20.7637	20.6085	20.6029	20.9412
HSPB6	17.8731	17.6111	17.1666	17.2535	16.7726	16.7546
HSPBP1	20.0812	19.7444	19.2613	19.3531	19.6117	20.8146
HSPD1	26.9968	27.0679	27.8056	28.2835	26.9365	28.5866
HSPE1	23.161	23.6544	24.3754	24.714	23.7215	24.6965
HSPH1	24.6853	23.5095	24.7746	24.4563	23.7659	25.4247
HTATSF1	20.6137	20.472	20.439	20.4968	20.6937	20.493
HTRA1	19.4749	18.2491	11.1619	13.1042	15.6595	10.6744
HTRA2	18.2218	18.5331	18.6813	19.065	18.7605	18.5202
HUWE1	19.5005	18.3841	18.7494	18.8435	18.055	18.8315
HYOU1	25.9382	25.7144	26.0789	26.1565	25.821	25.1816

IARS	24.1766	23.425	23.6322	23.1508	24.0201	23.6992
IARS2	22.7451	22.6675	23.0308	23.007	22.8597	23.1014
IBA57	15.0495	14.611	16.4741	17.4281	14.974	15.7921
ICAM1	19.0507	23.2313	19.5296	19.45	23.9532	18.9492
ICT1	18.0931	18.5243	19.0207	18.9628	19.3798	19.3971
IDE	21.8961	20.2014	20.8917	20.3843	20.851	21.6742
IDH1	24.9813	23.3744	23.4754	23.1718	23.4354	23.8471
IDH2	24.5549	24.1104	25.4302	25.9098	23.942	22.2038
IDH3A	23.5899	22.6644	23.6696	23.7142	22.8868	24.0872
IDH3B	22.0315	20.9922	22.1749	22.2883	21.3668	22.5862
IDH3G	21.8356	20.1175	21.4927	21.7173	20.1556	22.1898
IDI1	19.5115	18.5013	18.3152	18.5632	18.3112	19.1445
IER3IP1	19.1123	20.0001	19.8109	19.7161	20.0822	18.302
IFI16	21.4959	23.425	21.4301	21.5215	23.2269	21.342
IFI35	18.3874	18.6685	17.8854	17.5465	18.6706	18.3495
IFIT1	19.3162	18.1566	15.9054	16.3942	17.8028	17.1349
IFIT3	20.919	19.0854	18.9865	18.3809	18.9279	18.4762
IFIT5	19.0362	17.3432	17.0209	17.1142	16.1484	18.4977
IFRD1	17.09	14.7484	16.2548	16.2072	16.2082	16.7956
IGF2BP2	18.6162	22.8167	21.9431	21.9748	23.1007	22.7634
IGF2BP3	22.0866	23.8122	22.8621	22.5276	24.0754	21.0631
IGF2R	23.2369	24.4826	23.694	23.753	24.877	23.386
IGFBP7	18.5195	19.4399	18.2546	18.783	19.7918	22.123
IK	19.4846	19.6109	20.2142	20.0821	19.6794	21.003
IKBIP	22.2953	23.1903	22.1711	22.2415	23.7679	21.5082
IKBKAP	18.0773	15.9296	17.8612	17.271	16.9902	18.7768
IL6ST	19.6268	18.3046	19.3181	18.7279	18.9386	19.2447
ILF2	24.7092	25.872	25.3905	25.738	25.7161	25.9357
ILF3	24.1028	24.983	24.6781	24.8723	24.8758	25.1474
ILK	20.6512	20.8958	19.0883	19.3798	21.471	19.58
ILKAP	20.5512	20.6446	19.9511	20.2333	20.4859	20.3898
ILVBL	20.3526	20.277	20.5968	20.7245	20.1264	21.0445
IMMT	24.4323	24.6447	24.7054	24.9681	24.7689	24.882
IMPA1	20.6043	19.1334	19.6933	19.0558	19.9504	21.7537
IMPAD1	19.9239	20.0439	20.3467	20.2068	19.9717	19.5902
IMPDH1	19.9067	18.365	19.9289	19.689	19.7428	19.8924
IMPDH2	24.2372	23.9163	23.9958	23.763	24.3054	26.0095
INF2	20.5121	20.4798	20.1318	20.0666	20.82	18.5333
INPP5K	16.8889	17.6068	17.4444	17.0571	17.7333	18.035
INPPL1	16.66	16.6119	16.7534	16.6425	17.2251	15.6776
INTS1	17.1366	17.9581	18.9004	19.0693	17.4277	16.934
INTS3	19.6036	20.3185	20.5573	20.5809	19.8568	19.4737
INTS4	17.1416	18.0963	18.73	18.5005	17.5954	17.0777
INTS5	15.7614	17.2026	18.2072	17.9667	15.917	15.8945
INTS6	12.1216	15.4614	15.734	15.6729	15.2698	13.4481
INTS9	16.7388	17.2653	17.6008	17.8181	14.7229	16.1994
IPO11	18.4253	18.2392	18.7889	17.9529	18.7078	18.6067
IPO4	21.7307	21.6694	20.9128	21.215	21.8943	22.4844
IPO5	24.4212	24.4554	24.0862	23.6354	24.876	24.1654
IPO7	24.3957	23.4079	23.9427	23.6016	23.7946	24.8242
IPO8	17.1618	15.8051	17.7368	17.3227	15.9	16.2456
IPO9	21.7874	20.9197	20.9211	20.548	21.4299	21.0434

IQGAP1	24.7438	24.8988	24.6458	24.0008	25.6361	25.6748
IQGAP2	12.2251	8.12856	18.124	17.4145	NA	8.71614
IQGAP3	12.5799	12.7926	14.2222	12.8801	13.1603	14.6208
IRF2BP2	19.829	20.3143	20.0232	20.3081	19.6619	19.8454
IRF2BPL	19.6648	17.761	16.0861	16.9158	17.3537	17.4601
ISG15	21.197	20.5015	19.3483	19.3629	19.008	20.0471
ISOC1	23.1471	18.8708	19.3621	19.7743	18.544	19.8844
ISOC2	20.3264	20.263	20.7877	20.8901	20.109	20.3177
IST1	19.2642	19.297	18.8208	19.0189	19.2661	20.428
ITCH	19.1571	18.4686	19.85	19.4027	18.9256	18.9354
ITGA11	20.0952	19.5188	18.9418	18.9451	20.0344	20.3544
ITGA2	22.9874	21.5036	22.6009	22.6587	22.1117	21.4396
ITGA3	24.6451	25.4671	24.8954	25.5802	25.7646	24.8503
ITGA4	18.1544	17.2574	15.9189	15.7507	18.088	17.5685
ITGA5	21.4744	23.3009	21.3575	21.2724	24.0096	20.2236
ITGA6	19.7751	20.5546	20.2728	19.5408	20.6573	19.9836
ITGA8	19.8805	17.2675	17.2194	18.5716	17.085	16.6229
ITGAV	23.3463	23.4846	22.8419	22.768	23.3982	22.5624
ITGB1	24.6632	26.3624	24.4762	24.7173	26.2762	24.251
ITGB3	20.6638	18.6055	20.4017	20.0453	19.6221	19.5656
ITGB4	19.4745	19.6062	18.9819	19.27	20.0091	22.3177
ITGB5	19.6709	21.0997	19.6197	19.2486	20.2605	18.1222
ITIH2	19.65	17.615	15.8176	15.6768	18.3727	15.2595
ITIH3	19.2342	19.2043	17.6797	18.0394	18.3839	15.9723
ITM2B	19.9243	19.2494	19.5581	19.6107	18.6627	20.6709
ITM2C	18.2798	18.0992	18.9146	19.5071	16.9439	18.158
ITPA	21.4839	20.8871	20.3167	20.4497	20.9989	21.6886
ITPK1	17.3224	16.8253	14.3896	14.7479	16.0417	16.2783
ITPR3	16.9973	17.3503	17.6841	17.1488	16.7847	15.1048
ITSN2	19.5152	19.2447	17.7512	18.2276	19.355	19.6333
IVD	19.5214	20.1317	19.5574	19.9293	19.9378	19.1393
IVNS1ABP	20.3155	20.359	20.4226	20.3521	20.6198	20.6664
JAK1	16.8919	17.5937	17.909	18.3975	16.9772	15.173
JMJD6	18.5432	19.1333	19.2745	18.894	18.8235	19.3561
JUP	15.1828	15.444	15.1198	14.9954	14.7429	14.3835
KANK2	20.2625	19.27	18.1971	18.3068	19.2381	17.4028
KARS	24.7555	23.6065	24.4148	24.2766	24.0762	24.6898
KCMF1	17.1591	13.8274	16.4263	16.1167	12.854	15.8229
KCTD12	24.426	21.5536	21.406	21.2549	21.9731	22.6696
KCTD5	14.6013	15.857	16.6893	16.8066	13.0275	13.906
KDELC1	19.2024	20.8044	20.1136	20.2083	20.1422	19.1338
KDELC2	21.429	22.458	21.9799	22.3522	21.5383	20.3524
KDELR1	20.0555	19.9481	19.9273	20.0988	19.2521	18.7249
KDELR2	19.4238	20.8084	21.1011	20.9126	20.0645	20.2376
KDM1A	20.831	21.2591	21.3866	21.453	20.7648	20.7472
KDM2A	16.3234	17.1279	18.2948	18.6975	17.531	16.6229
KDM3B	19.3469	19.3739	19.5275	19.5695	19.1009	18.3388
KDSR	18.7355	19.7846	18.8183	18.5735	19.822	16.4848
KHDRBS1	24.1495	23.9687	23.9101	24.3264	23.815	24.1882
KHSRP	24.2867	24.8613	24.2237	24.6477	24.6138	24.7695
KIAA0196	18.8105	18.4292	18.7502	18.4648	19.0473	18.3815
KIAA0368	20.3974	20.5225	20.9215	20.3855	20.9162	20.6312

KIAA0391	17.2764	19.1527	18.0944	18.4542	19.3636	19.2158
KIAA1033	17.4498	17.6598	16.4896	15.7531	18.066	15.5401
KIAA1429	17.7514	19.0164	18.7543	18.8358	18.5682	17.6785
KIAA1468	18.9705	17.4391	16.3842	15.7894	18.1346	16.1998
KIAA1524	20.0786	18.672	19.7948	18.7477	18.9635	20.7657
KIAA1715	20.7425	19.7318	20.8042	20.485	20.4615	19.275
KIAA2013	19.968	19.8894	19.6088	19.5264	19.8208	20.9297
KIF11	18.6787	18.3176	18.5531	18.1109	18.7925	17.9214
KIF15	15.7744	16.3831	16.7718	15.8927	15.7171	15.7731
KIF1BP	19.7888	18.7521	19.5157	19.2251	19.7297	20.5181
KIF20A	19.863	18.8899	19.5746	19.2108	18.9451	19.6994
KIF23	19.889	19.4827	20.731	20.4388	19.7544	20.043
KIF2A	19.39	19.1106	19.7238	19.4094	19.5541	19.9352
KIF2C	19.0825	16.6458	19.2016	18.5917	17.5001	19.2454
KIF4A	16.6429	15.3984	16.6783	15.2345	15.6967	16.6495
KIF5A	11.121	18.4477	9.47599	11.1956	19.6685	12.7892
KIF5B	23.8027	22.6469	22.3628	21.9805	23.2595	23.295
KIFC1	17.0422	14.6041	15.4588	15.3784	14.6338	16.0789
KIRREL	17.9341	18.2616	18.0647	18.3376	18.6086	17.6721
KLC1	19.4086	18.1638	16.4619	16.2249	19.2856	18.9951
KLC2	19.8126	18.2599	18.2984	17.7688	19.0129	19.2369
KPNA1	20.5245	20.3181	20.3441	20.0501	20.1914	20.246
KPNA2	23.1145	23.6063	23.9951	24.1741	23.4951	24.8661
KPNA3	21.2069	21.085	21.6762	21.4892	20.9237	21.9789
KPNA4	21.6149	21.952	21.6884	21.5086	21.3459	21.7659
KPNA6	21.2688	21.0015	21.8151	21.1242	22.1407	21.3553
KPNB1	25.3951	25.2921	25.1779	25.2826	25.3538	26.0984
KRT1	22.1499	23.3685	22.3345	22.9627	23.788	23.7818
KRT10	21.788	22.3066	21.9181	21.9596	22.8984	23.4935
KRT14	11.7404	15.5	14.4629	13.4324	12.7554	19.7488
KRT16	16.5359	16.6075	16.4801	16.6734	17.2854	19.1052
KRT17	19.4191	18.7314	18.7184	19.0984	19.2482	21.41
KRT18	21.5391	21.3029	21.091	20.9538	21.5755	21.0243
KRT2	20.4981	21.2298	20.6008	20.5572	21.5669	21.8408
KRT5	16.1663	15.2142	14.645	15.0796	16.244	17.4181
KRT6A	16.7902	15.9763	17.0935	16.4141	16.1824	20.6635
KRT8	22.8403	21.9596	21.9496	22.1736	22.565	22.9589
KRT80	13.761	13.1618	13.3054	13.1019	14.707	13.2718
KRT81	16.7581	17.902	17.8501	17.8358	18.3777	21.9364
KRT9	20.1305	21.8701	20.4308	21.3399	22.0004	21.1496
KRTCAP2	17.9703	20.1864	19.5746	19.6924	20.1052	18.2749
KTN1	23.8419	23.4162	22.8728	22.9891	23.5387	23.1347
KYNU	24.7384	20.9012	23.1415	22.4525	21.2581	19.5475
L1CAM	14.8553	15.8303	16.3447	16.2097	15.6081	14.1518
L2HGDH	17.8193	16.2987	18.0086	18.5072	15.4961	18.0077
L3HYPDH	18.5697	17.4681	14.7419	13.6149	18.2056	19.327
LACTB	19.214	22.0682	21.9235	22.2909	21.7076	21.651
LACTB2	13.2649	15.4426	13.452	12.1495	15.5156	16.8239
LAMA4	19.3845	18.6055	18.7748	18.9652	18.6029	16.783
LAMB1	26.2312	23.91	24.2329	24.2566	24.1866	22.8111
LAMB2	17.854	18.4333	18.6732	17.9106	17.6834	17.9625
LAMB3	13.4564	13.9153	14.6247	13.541	17.527	11.9518

LAMC1	23.0348	23.4351	23.7394	23.4056	23.7261	21.6921
LAMP1	22.0107	21.6771	21.6987	20.8106	22.3391	21.5383
LAMP2	23.0059	22.6668	23.0081	22.4218	22.9012	23.2453
LAMTOR1	21.1848	21.4091	20.5668	21.0065	21.3898	20.8409
LAMTOR2	20.6117	20.918	19.761	19.579	21.3713	20.304
LANCL1	21.391	21.0723	22.0672	21.7908	21.4397	21.2147
LANCL2	17.5906	17.5709	17.2145	17.075	18.4667	18.2582
LAP3	25.5108	24.0923	23.8641	23.7363	24.0923	24.9758
LARP1	21.1019	20.3231	21.5567	21.1515	20.9846	21.278
LARP4	17.3251	16.7326	18.0904	18.1152	15.9803	17.679
LARP4B	17.4895	16.8426	17.3978	17.6242	17.7832	18.6356
LARS	24.1802	23.5075	23.8275	23.436	24.0871	24.3811
LARS2	19.1785	19.4515	19.3055	19.5076	19.5827	19.7669
LAS1L	19.2215	20.3441	21.0064	21.08	20.5424	20.6799
LASP1	22.7454	22.7698	22.5747	22.2503	23.4279	22.7055
LBR	20.7265	20.7579	20.997	21.1795	21.3049	21.0028
LCN2	10.5532	13.9969	11.1886	12.5544	15.0732	NA
LCP1	22.5017	20.8851	19.6227	21.4868	22.1544	22.678
LDHA	27.6595	26.7923	26.9722	26.7788	26.9344	23.4068
LDHAL6B	19.2183	19.4974	18.8527	18.8849	20.0713	19.9287
LDHB	26.4159	25.9688	26.0555	25.893	26.3397	26.7931
LDLR	18.2701	20.0094	19.0918	18.4787	20.1435	18.2684
LEMD2	18.076	19.433	18.5467	18.7318	19.3959	18.1693
LEMD3	17.5955	16.908	17.873	17.8146	17.196	17.5342
LETM1	22.5583	22.3111	22.6589	22.7751	22.3757	23.4465
LGALS1	25.7369	26.4757	26.2679	26.6857	26.6385	25.1207
LGALS3	23.4854	22.7408	21.8962	21.7526	22.8238	20.4687
LGALS3BP	23.2163	22.8323	23.234	23.4318	22.1394	23.9591
LGALS8	19.2667	18.3022	19.3039	19.0651	19.6329	18.786
LGMN	19.2155	19.3053	19.6546	19.7846	19.3475	19.7871
LIG1	18.649	18.1736	18.3937	18.2956	17.8317	18.7614
LIG3	17.3736	17.5819	18.4184	18.8386	16.9509	18.2513
LIMA1	24.0196	23.0757	23.3072	23.2817	23.0237	22.7506
LIMCH1	18.8312	12.97	16.1605	17.0525	13.7582	18.8988
LIMS1	20.4865	20.7428	20.6516	20.0606	21.4254	21.4599
LIN7C	19.531	18.4749	19.2774	19.4698	18.3023	18.9575
LIPA	18.8983	21.5125	20.6303	20.7523	20.8825	18.9715
LLGL1	19.5869	19.6377	18.7749	18.8161	20.1286	18.3498
LMAN1	23.6471	24.0717	23.5399	23.5851	24.8968	22.078
LMAN2	24.1686	24.3781	24.0187	24.0991	24.4324	24.3705
LMAN2L	20.2127	20.5058	21.1923	21.379	20.0388	20.0296
LMF2	19.3509	19.3261	19.6041	19.7448	19.0294	17.5436
LMNA	27.8531	28.3426	27.8033	28.0841	28.2042	27.4394
LMNB1	25.4424	24.7991	25.9152	26.0016	24.8482	25.5958
LMNB2	24.5645	24.6753	24.1639	24.6935	24.527	24.6979
LMO7	20.0037	18.8405	18.4833	18.1228	19.1293	17.9695
LNPEP	20.7711	20.4153	20.8515	20.4469	20.603	20.2193
LONP1	23.0868	22.9951	23.2229	23.3369	22.7613	23.3449
LONP2	17.8498	14.7715	17.4499	17.0367	14.7948	17.0751
LOX	16.4289	19.9317	19.0247	17.8423	19.9197	13.668
LOXL2	21.1608	21.5542	21.1368	21.2818	20.3469	20.4092
LPCAT1	19.3614	20.2914	20.6711	20.9043	20.2671	20.5812

LPCAT2	13.2585	19.1579	17.7823	15.7407	20.205	19.4233
LPCAT4	14.8583	16.6997	16.8436	16.2962	16.1135	17.9278
LPP	17.8841	15.442	15.629	16.0911	15.9957	16.9344
LPXN	15.9291	21.3531	17.9262	17.0561	22.4705	16.1353
LRCH3	17.1674	17.0568	18.2259	17.7156	16.6286	17.3802
LRP1	20.7389	23.1573	20.4302	21.107	22.877	19.0814
LRP10	17.8457	17.8631	15.5281	15.3386	17.9042	13.9593
LRPAP1	21.2451	20.9619	20.5354	20.9074	20.747	20.7635
LRPPRC	24.9039	25.2859	26.0435	26.134	25.411	25.7939
LRRC1	15.6161	17.0381	17.5753	17.4217	17.4252	18.365
LRRC15	18.7282	22.9409	18.3318	18.5	23.1448	18.1885
LRRC40	19.425	18.4439	19.1854	18.482	19.3255	19.0015
LRRC47	22.8814	22.1649	22.2549	22.197	22.4288	23.6415
LRRC58	12.3773	14.2163	17.4548	16.888	14.6109	16.6873
LRRC59	23.8195	24.89	24.4171	24.3222	24.937	24.9697
LRRC8C	15.6597	17.5372	17.2939	17.9224	15.8597	15.9315
LRRFIP1	20.5221	17.0907	18.9532	18.2593	18.9565	19.3186
LRWD1	16.905	17.8614	18.2533	18.3218	17.7569	18.587
LSG1	19.4396	20.068	20.3185	20.2229	20.0416	20.5827
LSM12	21.1486	20.5241	20.84	20.5692	20.7501	21.2478
LSM14B	18.0344	17.1219	17.4497	17.6315	16.3968	18.2266
LSM2	21.0657	21.2065	20.8545	21.1782	20.8219	21.0408
LSM7	19.7527	19.8887	19.958	19.792	19.9868	20.2393
LSM8	18.432	18.7106	19.1926	19.6152	17.9538	18.7283
LSS	21.8436	22.5514	22.5547	22.6638	22.5676	21.0309
LTA4H	22.357	21.1845	21.1665	20.9791	21.706	22.4333
LUC7L	18.4578	18.4395	17.5792	17.7593	17.7951	17.6551
LUC7L2	21.2776	20.8751	20.2443	21.1814	20.825	21.4574
LUC7L3	20.3519	20.0834	20.26	20.3536	19.9616	20.4269
LUZP1	19.0596	18.8804	18.9652	19.3625	18.5628	19.0929
LYAR	13.7817	12.7188	13.5545	13.0256	12.8712	17.9965
LYPLA1	20.4546	20.4867	20.8904	20.7859	20.9068	21.0057
LYPLA2	17.9999	18.1145	18.3089	17.9757	16.97	19.196
M6PR	21.2485	21.8542	21.4832	21.6748	22.0588	21.864
MACF1	20.6831	20.6438	20.5468	20.7038	20.8948	20.4814
MAD1L1	18.9219	19.3041	19.2108	19.3546	18.821	19.1487
MAD2L1	18.4308	17.2536	18.0378	17.9622	17.0256	19.9121
MAGEA1	10.9555	12.5748	10.1974	11.8247	12.5589	13.4173
MAGEA10	2.37828	9.36221	10.3996	-0.752766	6.36649	12.6883
MAGEC2	13.8153	13.4113	15.3121	14.8939	14.046	15.0032
MAGED2	18.406	18.2733	19.6764	18.9259	18.8638	19.5024
MAGOH	21.9469	22.9185	22.5992	22.8423	22.6711	22.7038
MAGT1	18.8058	19.0552	18.3093	18.8463	19.5532	18.8008
MAK16	15.9222	17.8244	18.339	18.5335	17.9008	19.3128
MALSU1	17.0617	17.9651	18.3432	18.2462	18.3848	17.5638
MALT1	19.7107	17.6486	17.2398	17.02	17.4212	19.4872
MAN1A1	16.2846	18.7384	15.4249	14.5988	18.9514	17.2901
MAN1B1	19.848	20.3703	20.5465	20.2091	19.9137	19.0175
MAN2A1	17.9081	21.2726	19.2054	19.4463	21.0871	19.1707
MAN2B1	20.3106	20.6845	20.2444	20.7273	20.0923	20.1661
MANF	22.2857	22.5419	23.0717	23.0494	22.4153	22.3017
MAP1A	21.134	19.0753	17.8598	17.6177	20.0953	17.2091

MAP1B	25.0323	23.7328	24.1398	23.6001	24.9166	25.3644
MAP1S	17.399	17.2408	16.0514	15.6427	17.232	16.2046
MAP2K1	19.2561	18.4596	17.8799	17.838	19.108	19.952
MAP2K2	18.0935	17.5924	16.8538	16.8445	17.8793	19.618
MAP2K3	15.0602	18.5195	15.5057	16.5393	18.8411	16.9482
MAP4	23.2403	21.7207	22.3644	22.1174	22.6395	23.8147
MAP4K4	21.2046	21.3076	21.1423	21.2903	21.8989	21.1052
MAPK1	22.4377	21.4493	20.914	20.4729	22.4734	21.8936
MAPK14	18.8554	18.2445	17.9163	17.9347	17.348	17.9233
MAPRE1	23.4193	22.542	23.2293	23.2341	22.9734	24.1525
MAPRE2	19.0443	16.6386	17.8639	17.8424	15.9453	18.5328
MARCH5	17.9091	18.468	18.8511	18.5645	18.1619	18.6655
MARCKS	22.2396	20.8873	21.1681	20.508	21.4134	22.0461
MARS	22.5515	21.6161	22.3472	22.2259	22.3713	23.407
MAT2A	23.9518	22.6029	23.2397	22.5112	23.642	23.4455
MAT2B	20.2055	18.7857	19.3814	18.0447	20.5917	18.2812
MATR3	25.5811	25.8401	25.9182	26.0937	25.7722	25.5799
MAVS	20.9485	20.5012	22.4167	22.3825	20.882	21.0763
MBD3	16.8692	17.8025	17.9439	18.2089	16.9997	17.4304
MBLAC2	16.5649	17.3602	17.9102	18.11	16.7037	17.4779
MBNL1	21.5376	20.5297	21.1711	21.1179	20.688	21.2507
MBOAT7	19.4424	20.8765	20.737	20.7277	21.2215	19.9575
MCAM	21.2433	19.9304	20.4125	20.6515	20.4675	20.4148
MCAT	16.1708	16.9877	18.6606	18.9846	16.7766	17.6148
MCCC1	19.237	18.9258	20.2187	20.0092	18.9507	19.6169
MCCC2	21.2009	21.9534	22.4107	22.5028	22.0268	21.9482
MCM2	23.8566	23.4786	23.2927	23.2666	23.2436	23.4717
MCM3	24.1089	23.7558	23.6563	23.6611	23.5723	23.9302
MCM4	23.7825	23.2185	23.0424	23.2815	22.8415	23.4
MCM5	23.3819	22.9944	23.1034	23.1586	22.9524	22.809
MCM6	24.465	23.9633	23.7637	23.7391	23.7437	23.9473
MCM7	24.564	24.0278	23.6785	23.9027	23.7307	24.0587
MCMBP	18.7501	20.0827	19.2079	18.8378	19.787	18.8533
MCTS1	21.418	20.6523	21.2438	20.8039	21.3162	21.8013
MCU	18.0114	18.3659	18.2103	18.369	18.1629	18.8622
MDC1	18.6587	19.3372	19.22	19.4125	19.3063	18.7156
MDH1	24.5019	23.9174	23.2984	23.1975	24.4807	25.0263
MDH2	26.8336	26.3472	26.815	27.2988	26.2231	27.0714
MDN1	16.7341	17.5632	16.7413	17.2489	17.5051	16.2971
ME1	20.0298	18.5258	18.1223	17.6402	18.738	18.223
ME2	22.0503	21.39	21.9116	21.9503	21.9776	21.5722
ME3	12.6963	15.6534	12.0839	11.5752	14.1416	9.14313
MECR	12.9336	14.6359	16.9084	16.58	11.6244	13.7556
MED1	17.3688	17.3406	18.1366	17.9464	17.1831	15.4286
MED14	10.6411	15.6899	16.7853	16.8073	15.6232	16.3142
MED17	15.2655	16.5392	16.2299	17.1387	14.7658	16.9517
MED23	15.8202	16.4437	17.0542	17.2846	15.1815	15.9383
MEMO1	19.014	17.6564	17.5452	16.1828	17.5908	19.348
MESDC2	22.3797	22.5049	23.0169	22.908	22.5364	22.2756
MET	13.8202	17.2321	18.6226	17.9768	17.2283	16.5968
METAP1	22.0201	21.8604	22.0822	22.2159	22.0052	22.1565
METAP2	21.9987	21.1217	20.5859	19.9951	21.2604	21.5928

MFF	18.2405	17.0735	18.3418	18.6663	17.1173	18.2412
MFN1	15.8376	15.3203	17.9714	17.9841	14.9319	15.6643
MGAT1	19.0244	20.9264	19.9445	20.1952	20.5435	18.8229
MGAT2	18.6939	19.8788	18.633	18.6644	20.3418	18.8908
MGEA5	20.5627	19.1427	19.5481	18.5284	20.7475	20.2329
MGLL	17.5908	18.0029	17.1201	16.7306	18.9688	17.3948
MGST1	22.6101	21.9147	23.3633	23.3681	22.4752	23.1654
MGST3	19.5219	19.9124	19.6378	18.8563	20.9563	18.0894
MIA3	20.0247	20.1829	20.3807	20.4477	20.7447	19.1901
MICU1	16.7671	17.4773	18.2781	18.2264	16.2835	16.7945
MICU2	17.6702	17.4235	17.6032	18.0453	16.586	17.6417
MID1	16.2553	15.8678	16.0089	15.8159	16.3614	18.4428
MIER1	16.9473	17.0172	17.223	17.3129	17.5309	17.056
MIF	24.6935	23.2491	23.3572	23.253	23.4521	18.9552
MINA	18.5039	19.4782	20.3484	20.0052	19.2672	18.2197
MINPP1	19.4086	20.0886	20.4843	20.6415	19.0229	21.0648
MIPEP	18.3581	18.3729	19.4894	19.7223	17.8496	18.8156
MKI67	19.8728	19.6408	21.3437	21.3692	20.3486	19.6879
MKLN1	19.1498	17.8757	18.202	17.8688	18.2712	17.4024
MLEC	22.5337	22.4775	22.621	22.5016	22.5349	21.6122
MLH1	15.3388	15.557	16.1082	15.6297	15.6279	16.4344
MLLT4	19.0171	17.9632	17.5943	17.5074	17.9798	17.1554
MME	20.6128	23.6367	20.0971	19.6982	23.2524	19.5759
MMP1	17.293	21.6409	17.1085	17.4054	22.7313	17.3218
MMP14	23.1088	22.8055	20.2886	20.6784	22.5952	19.6667
MMP2	20.2012	21.244	20.5188	20.2407	20.7815	17.835
MMP3	14.3365	19.0949	15.0655	14.8041	20.9738	14.3818
MMS19	19.1839	19.5578	19.3628	19.1879	19.6006	20.4145
MNAT1	17.9429	18.6415	18.6952	18.5745	19.0306	18.031
MOB1B	19.0873	18.5493	18.8672	18.4278	19.49	19.5993
MOB4	18.6045	17.911	18.2058	17.5406	18.0993	18.0069
MOGS	21.7195	23.2573	22.6233	22.7421	23.5679	23.5329
MON2	18.3691	17.2011	17.7019	16.3097	18.3965	16.7233
MOSPD2	19.7961	19.601	18.9793	18.6579	19.7738	19.0024
MOV10	20.4347	20.822	21.1206	21.0585	21.1836	20.9616
MPDU1	20.3189	20.803	20.7114	20.9166	20.8633	20.7287
MPHOSPH8	15.2364	17.4571	16.6255	17.0971	17.2798	15.6281
MPP1	18.9029	18.3284	17.6424	18.1262	18.0044	16.1164
MPP5	13.0579	17.1642	16.9626	16.3193	16.7236	16.1261
MPP6	18.3197	19.6307	21.0216	21.0787	19.9178	20.5719
MPRIP	20.8455	21.2547	20.7399	20.4982	21.3819	20.0862
MPST	20.4797	20.3804	20.6857	21.3326	19.8465	19.4956
MPZ	16.6145	16.3708	16.2349	17.7955	16.7925	16.722
MPZL1	20.6541	20.4152	20.2163	20.497	20.8676	19.4939
MPZL2	23.3741	20.1902	22.7175	22.483	20.7435	23.4093
MRC2	22.7458	22.8666	21.9994	22.3748	22.2538	19.7208
MRE11A	21.1066	21.1541	21.4352	21.2197	21.3513	20.288
MRGPRF	17.679	19.6874	14.7885	14.7218	18.6541	15.9176
MRI1	17.8302	15.217	15.484	15.4439	16.1748	19.6755
MRPL1	19.8098	19.8651	19.7119	19.7276	20.2319	19.851
MRPL10	18.3042	18.5005	20.0451	19.8666	17.6235	19.6757
MRPL11	19.2828	20.3158	19.6122	19.8791	20.3389	19.7148

MRPL13	20.1305	21.0281	20.6656	21.0599	21.2095	20.359
MRPL15	19.8995	20.6533	20.8915	21.2372	20.8538	20.9627
MRPL16	16.627	17.7609	17.1808	17.6375	17.7991	17.7064
MRPL17	18.3163	19.2973	19.634	19.8552	19.0773	18.8247
MRPL19	20.713	20.9805	21.1922	21.206	20.9974	21.247
MRPL21	19.6451	20.6817	20.3014	20.6045	20.3314	20.415
MRPL23	18.1473	18.8592	18.8575	19.1321	18.4567	17.0522
MRPL24	20.3025	20.4068	20.7605	20.5964	20.2713	20.8367
MRPL28	18.6007	19.8111	20.1959	20.4287	19.1183	18.8155
MRPL3	18.4216	19.2419	19.7415	19.2981	19.4274	19.6721
MRPL37	21.6284	22.2386	22.1812	22.2094	22.3048	22.1451
MRPL38	20.9532	21.4335	21.152	21.3589	21.773	21.3735
MRPL39	20.2997	20.7404	21.0887	21.2905	20.6594	20.5733
MRPL4	19.3788	19.8427	19.8647	20.1224	19.796	20.0654
MRPL41	18.3275	18.8088	19.4236	19.2761	19.2577	18.9936
MRPL44	18.5376	19.2465	19.4093	19.8732	19.2426	19.6017
MRPL45	21.5855	21.691	22.2637	22.3568	21.8386	22.0991
MRPL46	18.4649	18.6012	19.8092	19.7412	18.756	18.2646
MRPL47	18.3231	18.9125	19.6384	19.3488	19.1401	18.9764
MRPL48	18.2512	18.5193	19.1958	19.6312	18.7676	18.5214
MRPL49	18.9674	19.2137	19.8532	19.8056	19.545	19.1474
MRPL53	18.7364	18.5135	19.7018	19.8204	18.4934	19.2531
MRPL9	17.2896	18.3374	18.7279	18.6937	18.5244	18.7477
MRPS11	14.3319	15.174	16.5967	17.2252	13.2862	14.6072
MRPS16	18.6651	19.7022	20.0884	20.3541	19.8034	19.8124
MRPS17	19.2098	19.55	19.6159	19.8226	19.2989	19.6994
MRPS18B	16.4572	17.3744	18.2275	18.1713	17.4636	17.7818
MRPS2	19.6381	20.2196	20.727	20.7731	20.5689	21.0983
MRPS22	21.1257	21.4206	21.9762	22.0663	21.3061	21.7294
MRPS23	20.6038	20.8913	21.6476	21.7397	20.9806	21.5491
MRPS25	18.4131	18.3317	19.1598	19.2858	18.4269	18.8686
MRPS26	17.1519	17.297	17.4736	18.3808	17.428	18.0445
MRPS27	20.9067	20.8486	22.146	22.3927	20.7566	21.9924
MRPS28	18.2831	18.6854	19.4793	19.5602	17.7502	19.0279
MRPS30	19.6953	20.3019	20.5529	20.6929	20.0081	19.8005
MRPS31	17.9814	17.7255	19.0011	19.1288	17.7177	19.0056
MRPS34	18.2124	19.3314	20.3808	20.1266	19.5734	20.1821
MRPS35	20.2231	20.203	20.9732	21.1494	20.4302	20.4616
MRPS6	19.5842	19.875	20.8417	21.1768	20.0143	20.1091
MRPS7	19.1859	19.6213	20.1806	20.4587	19.9099	20.6552
MRPS9	18.3404	18.0073	17.8133	18.6381	18.3075	18.7879
MRTO4	20.1951	21.221	21.8169	21.8476	21.4968	22.3558
MSH2	21.6202	21.264	21.7951	21.5635	21.1533	21.8178
MSH6	21.5679	21.0786	21.6472	21.3082	21.2129	21.5201
MSN	25.7814	25.781	25.7098	25.5548	26.4362	25.9188
MSTO1	17.481	17.2356	16.2713	15.2995	18.1513	16.0303
MT2A	18.6549	19.399	16.0813	15.5575	21.0933	19.4883
MTA1	18.1406	19.1352	18.8896	18.8624	18.6012	18.8384
MTA2	20.2767	21.2075	21.0979	20.9434	21.3673	21.6358
MTA3	17.7372	18.0348	18.6258	18.716	18.3408	17.4598
MTAP	20.9281	21.1227	21.063	20.6582	20.9797	23.4171
MT-ATP6	20.2585	20.7577	21.0735	21.0299	20.7148	20.1007

MTCH2	21.4413	21.6398	22.4928	22.4765	21.6737	22.0885
MT-CO2	20.6542	22.2821	22.7415	23.1086	22.4547	20.8615
MTDH	22.0814	21.2322	22.9812	22.7472	21.4738	22.5595
MTERF3	14.4896	14.7207	16.0756	16.3117	13.8453	16.3406
MTFP1	16.6673	17.2036	17.498	17.9081	16.768	15.0838
MTFR1L	17.2375	15.9665	17.473	17.3926	14.7966	17.425
MTHFD1	25.5621	23.6522	23.3711	22.9714	24.1964	24.6857
MTHFD1L	21.3815	20.9847	21.1699	21.626	21.0668	22.5675
MTHFD2	21.6064	21.1254	21.9275	21.4355	21.9685	21.9341
MTOR	17.2082	18.3274	18.1058	18.0306	18.0987	17.5671
MTX1	18.9679	19.7818	20.0814	20.4143	19.5184	18.6641
MTX2	18.958	19.5257	20.3275	20.524	19.0092	19.1369
MUT	21.2946	21.6562	21.2591	21.0939	21.4993	22.076
MVB12A	17.3144	16.8308	15.7608	16.0998	16.3738	18.443
MVP	25.9238	25.2263	24.9559	24.5576	25.5984	23.9259
MX1	22.7523	20.4485	20.2855	20.509	20.6676	21.5061
MX2	14.373	15.6768	16.4589	15.9786	13.9151	12.8081
MYADM	21.1725	22.252	21.6283	21.3383	22.6393	19.9854
MYBBP1A	17.9249	20.3043	20.3993	20.2849	20.3406	22.2388
MYDGF	23.6919	24.1625	23.8898	23.8012	24.0401	23.9832
MYH10	21.2099	20.2458	22.2802	22.5933	20.6239	20.2344
MYH9	27.7295	28.16	26.9067	26.9219	28.7026	27.8245
MYL12A	23.216	23.8968	23.1834	23.0841	24.3018	23.6021
MYL6	23.9131	24.1871	23.4291	23.2682	24.6509	24.084
MYLK	22.5416	22.1203	21.2273	20.8712	22.16	21.0382
MYO18A	19.3743	19.4142	20.9782	21.1524	19.1302	20.9042
MYO1B	20.3048	23.8244	20.302	20.2521	23.8215	21.7511
MYO1C	24.4161	25.1167	24.891	24.8571	25.1535	24.6614
MYO1D	NA	15.9721	7.23992	10.2407	15.7429	11.0705
MYO1E	18.3712	18.119	18.8051	17.8868	19.0269	19.7513
MYO5A	15.3976	14.0921	16.0226	16.0135	15.4493	16.2906
MYO6	19.6381	19.0501	17.4832	17.3865	19.5379	17.8713
MYOF	24.6033	25.5365	25.2076	25.1243	25.5625	24.9433
NAA10	19.7108	18.4893	19.2392	18.9154	19.4726	20.3562
NAA15	21.4024	21.0392	21.568	21.3471	21.8249	22.3161
NAA35	16.3341	16.3873	15.2029	13.7028	17.4077	16.9116
NAA50	20.5894	19.7425	20.2684	19.6962	20.5285	20.6078
NACA	22.7105	22.0992	22.6937	22.2777	22.5624	23.5333
NACC1	17.8719	18.0683	17.7418	17.5351	17.1043	18.7677
NADK2	19.5693	20.2026	20.1995	20.4282	20.4112	19.0863
NAE1	20.2135	19.6774	19.379	18.8515	19.0236	19.8921
NAGK	21.9551	20.8176	19.4072	19.3197	21.3675	20.4949
NAGLU	20.0338	21.2874	19.9165	19.8727	20.6477	18.3536
NAMPT	24.9134	26.0638	24.8963	24.4159	27.4491	24.4112
NANS	22.2165	21.4019	21.292	20.9576	21.749	21.4787
NAP1L1	23.8119	23.0961	23.5804	23.5452	23.3794	24.3438
NAP1L4	23.0337	22.4448	22.4087	22.1583	22.8784	22.1855
NAPA	23.5726	23.1404	22.9268	22.9572	23.1831	23.3173
NAPG	19.6358	18.7527	19.6718	19.0248	19.1159	19.1397
NARS	24.7814	23.8737	23.7697	23.3197	24.6856	24.2856
NARS2	16.185	17.2335	18.0793	17.9957	17.429	17.0643
NASP	21.4607	21.2624	21.9207	21.8931	21.0761	21.2213

NAT10	17.7778	20.5693	20.8525	20.82	20.3667	20.5708
NBAS	18.3523	19.1061	18.3042	18.239	19.0802	17.3843
NBN	17.5376	17.1523	17.5832	18.213	17.987	17.3043
NCAPD2	20.2523	19.983	20.8238	20.6136	20.6265	21.0567
NCAPG	19.9893	19.1385	19.9031	19.4324	19.2108	20.336
NCAPG2	18.6416	17.3978	19.6093	19.4814	17.058	17.0142
NCAPH	19.4938	17.8374	17.9809	18.2582	18.1595	19.6634
NCAPH2	14.455	15.0451	16.4816	16.2573	14.3591	13.3216
NCBP1	21.4269	21.9555	22.2722	22.154	21.83	21.9416
NCBP2	18.6392	19.2741	19.383	19.501	18.927	18.6211
NCBP3	18.1187	18.2903	19.4607	19.4096	18.4391	19.1309
NCEH1	22.8861	23.6038	23.2392	22.7366	24.5377	22.3675
NCKAP1	21.1736	20.7654	20.2545	20.11	21.1689	21.3149
NCKIPSD	17.825	15.9914	17.4516	16.6452	16.7301	18.7493
NCL	26.548	27.0055	27.1907	27.142	26.8674	27.2353
NCLN	20.898	22.1069	22.0102	22.1319	22.1538	21.6412
NCOA5	19.8813	19.981	19.8383	20.0766	19.4657	19.4924
NCOR1	18.0095	16.3071	18.6744	18.6835	16.7558	16.7804
NCSTN	21.8278	21.9696	21.294	21.1657	21.8532	20.7443
NDRG1	20.2376	18.9317	18.1793	19.0367	18.4945	18.8202
NDRG3	19.6001	19.2513	19.8342	19.9433	19.1533	20.3232
NDUFA10	20.7245	21.1759	21.358	21.4926	20.3841	20.5246
NDUFA11	20.8539	20.9735	20.8565	21.1505	21.1547	20.7143
NDUFA12	16.8638	18.7126	18.0774	18.7351	18.9923	15.9868
NDUFA13	20.2466	20.8324	20.9812	21.2764	20.5377	20.0197
NDUFA4	20.3428	21.2125	22.2069	21.9545	20.7869	20.6603
NDUFA5	19.344	19.4289	20.0217	19.8161	19.5295	19.1644
NDUFA6	19.9213	19.6394	20.7864	20.7826	19.4118	18.6333
NDUFA8	20.519	20.6841	21.1795	21.4078	20.4533	20.0796
NDUFA9	18.5891	19.9235	20.3844	20.3757	19.5262	19.7753
NDUFAF2	18.4014	19.1437	20.1486	20.4154	19.4152	18.606
NDUFAF4	18.7515	19.3655	19.3619	19.4079	19.7598	19.7234
NDUFAF7	17.5427	18.2697	19.336	19.5344	18.3556	20.4288
NDUFB1	19.5079	19.4446	19.2415	19.409	19.6219	18.7402
NDUFB10	21.3228	21.1699	21.5892	21.914	20.9844	20.7125
NDUFB11	19.3593	19.9451	20.7993	20.7286	19.8491	19.2152
NDUFB3	19.8591	19.8757	20.2759	20.3971	20.1664	19.6326
NDUFB4	18.3561	18.6016	18.8522	19.1945	19.1055	17.3198
NDUFS1	23.361	23.1312	23.3348	23.6359	23.3961	22.9056
NDUFS2	22.1493	22.2309	22.6113	22.6347	22.4424	22.6168
NDUFS3	23.106	23.1892	23.3418	23.5405	23.1993	23.1706
NDUFS5	18.647	18.5734	18.5371	19.0992	18.933	18.1184
NDUFS8	19.6677	19.9007	20.3258	20.5103	19.5764	19.5296
NDUFV1	22.6774	22.6746	22.7965	23.0401	22.8728	21.82
NDUFV2	20.4045	20.8318	20.8835	21.1998	20.9863	19.3691
NEDD4	17.9758	17.3446	17.8798	17.5954	17.5792	17.8438
NEDD4L	15.1321	14.0265	12.9827	13.3066	13.8364	11.3218
NEDD8	19.6122	19.4359	19.34	19.4289	19.1141	20.3245
NEFL	14.6168	15.9539	12.288	13.6372	17.6675	20.9996
NEK9	20.0521	17.8253	16.6192	16.5355	18.8081	17.5982
NELFA	15.9721	17.3845	16.965	16.7802	16.6694	18.147
NELFCD	17.9359	18.2213	18.1912	18.2388	17.5378	18.5486

NEO1	19.9302	17.6131	17.8938	18.3713	16.9185	15.563
NES	24.5549	22.9819	22.1434	22.4091	23.2129	26.2534
NEU1	20.925	21.6829	21.857	21.9085	21.5119	21.7092
NF2	17.7166	18.1092	17.7131	17.9104	17.8031	19.0572
NFIC	17.839	17.4422	17.6454	17.615	16.2674	18.1438
NFKB1	19.7561	20.2947	19.0294	19.1729	20.7062	18.6424
NFKB2	16.6868	18.8682	16.7714	15.3851	19.8426	17.1065
NFS1	17.2508	17.6495	17.7747	18.0759	18.1737	17.576
NFYC	17.8941	17.9121	17.7534	17.9633	17.821	17.2354
NIF3L1	20.761	20.0966	20.0028	19.9605	20.1662	20.0626
NIFK	18.2834	17.825	17.7999	17.663	18.0664	19.1951
NIP7	19.653	20.5352	21.3806	21.3225	20.4093	20.7718
NIPSNAP1	20.2586	20.9211	18.7324	18.82	20.8108	20.513
NIPSNAP3A	20.8339	22.1103	20.8307	21.146	21.8729	20.8464
NIT1	18.881	19.1994	18.5353	19.0607	19.2543	18.0204
NIT2	21.3388	20.4748	20.5995	20.7607	20.6772	20.5418
NKRF	20.1237	19.8376	20.4588	20.3811	20.4418	21.0541
NLN	21.2486	20.7737	21.5166	21.1988	20.4983	21.5581
NMD3	18.0943	17.1451	19.63	18.8157	17.8165	19.2951
NME1	22.8586	22.511	22.6525	22.5224	23.1639	23.9598
NME1-NME2	23.5317	23.0163	22.545	22.7424	23.0403	23.7191
NME2P1	17.3481	14.6784	17.7802	18.1463	16.6187	19.8328
NMT1	22.0749	21.9593	22.3505	22.0283	22.5338	23.0172
NNMT	22.8421	22.1891	21.8874	21.5178	22.4253	21.1409
NNT	23.019	21.9541	23.3703	23.5624	22.158	22.5593
NOA1	17.9725	17.4286	18.0036	17.8818	17.8437	19.8368
NOC2L	20.0792	20.0738	20.3598	20.5386	20.3003	20.8944
NOC4L	18.4887	19.3133	18.9483	18.8436	19.5504	19.4531
NOL10	16.77	19.0743	18.7217	18.6596	18.7836	19.5383
NOL11	19.5816	20.3363	20.8273	20.7125	20.6812	21.3669
NOL6	19.8663	20.6031	21.2667	21.0209	19.9588	20.6827
NOL9	13.9061	15.6231	16.1244	16.2429	14.0288	16.4355
NOLC1	17.4222	17.2984	20.4326	20.4323	17.5488	20.8322
NOMO1	12.7304	14.8507	15.4905	15.2025	13.2391	11.3581
NOMO3	20.2463	20.9535	21.1962	20.861	20.5381	20.2596
NONO	24.0493	24.1233	24.5326	24.7788	24.044	24.0486
NOP16	15.0031	15.6174	17.5267	17.6107	15.164	16.3914
NOP2	16.3684	19.7928	18.5821	18.0486	19.537	18.4074
NOP56	17.3059	19.4652	19.0323	18.9149	19.6626	20.5557
NOP58	18.0938	20.3808	20.4791	20.3194	20.4739	21.3473
NOSIP	19.2696	18.463	19.9059	19.8351	18.1992	19.7889
NOTCH2	13.6673	17.0994	16.3367	16.5541	17.1595	13.2203
NPC1	20.0821	20.0423	20.5877	20.5164	20.3285	17.8055
NPC2	21.7439	22.2889	20.6823	21.2458	22.0375	21.2266
NPEPPS	24.0782	23.3826	23.6082	23.3236	23.7577	24.2951
NPLOC4	22.3588	22.326	22.8043	22.8757	22.4215	23.3306
NPM1	24.8271	24.8823	26.1825	26.7539	25.0923	26.1445
NPM3	17.2496	18.1913	18.761	19.37	18.2396	18.906
NPTN	21.7724	22.9008	22.1454	22.0459	22.9508	21.5269
NPTX1	17.8083	13.3567	15.5336	13.9757	12.7157	13.6225
NQO1	28.091	24.325	25.6039	25.5926	24.6323	26.4063
NR2C2	18.1375	17.2747	18.2727	19.21	17.625	19.0404

NR2F1	14.3008	15.3481	17.2648	17.736	14.0649	14.6364
NRAS	21.6717	22.329	21.9951	22.0939	22.3386	22.4235
NRBP1	20.3491	19.4513	19.1237	19.5026	19.1132	20.8103
NRCAM	12.6685	NA	12.8833	14.546	NA	17.4788
NRDC	21.517	20.8627	21.1708	20.4051	21.5726	21.7008
NRP1	21.2522	22.1108	20.1818	19.6147	22.4267	18.9545
NRP2	18.9523	21.1468	18.5714	18.6474	21.487	21.8113
NSA2	17.1253	18.0834	18.9582	18.716	18.4949	19.4968
NSDHL	23.0319	23.5826	23.5646	23.5885	23.3149	23.0789
NSF	23.7345	24.442	23.8934	23.7085	24.7894	24.084
NSFL1C	20.6001	18.8446	20.8975	20.7749	19.2758	20.1616
NSMCE4A	14.6977	13.9895	15.7253	14.8185	13.5107	14.8312
NSUN2	22.0693	22.1163	22.384	22.0713	22.0667	22.959
NT5C2	20.1288	19.0478	19.726	19.839	19.5417	20.9878
NT5DC1	20.9834	19.5746	19.1216	18.8625	19.6344	20.2857
NT5DC2	21.0337	22.4701	21.1211	21.2656	22.8969	21.4458
NT5DC3	20.7841	19.4236	19.942	19.3764	20.0857	21.4286
NT5E	23.4722	26.8543	23.3914	22.9013	27.3804	22.564
NTMT1	18.243	17.4479	17.1907	17.3718	17.8269	18.9451
NTPCR	20.2939	20.0724	20.5487	20.5428	20.1452	19.5302
NUBP1	20.3496	17.9215	18.1668	18.2822	18.3636	19.2172
NUBP2	19.6988	18.7627	18.7988	18.625	18.8039	19.3427
NUCB1	18.3559	17.8717	17.8904	18.3118	17.9198	17.6114
NUDC	22.6971	21.251	22.3821	21.7338	21.9416	23.8933
NUDCD1	19.5352	19.8527	20.0181	19.4837	19.6734	20.0087
NUDT16	15.9122	17.3763	16.1108	16.2356	17.4496	15.4033
NUDT19	17.4044	18.2285	19.2677	19.5551	17.6741	17.7311
NUDT21	22.7664	23.5794	23.4705	23.5306	23.1916	22.6549
NUDT5	22.8036	22.42	21.5064	21.2898	22.7484	23.19
NUFIP2	20.5294	17.5627	20.3326	19.8083	18.0554	20.8686
NUMA1	23.921	24.5031	24.639	24.7864	24.6471	23.0528
NUMB	17.3719	18.1944	17.76	17.6201	18.149	16.668
NUP107	20.8993	20.8914	21.5944	21.4943	20.5423	21.106
NUP133	21.888	22.2804	22.3609	22.3028	22.1899	21.3787
NUP153	21.1963	21.1713	21.7852	21.7825	21.4127	21.0396
NUP155	22.6085	23.3804	23.6406	23.6871	23.2873	23.111
NUP160	22.2391	22.5081	22.6127	22.4443	22.3165	22.388
NUP188	20.3573	19.6653	20.2266	19.9931	19.7686	20.142
NUP205	21.8145	22.5918	23.0799	22.9786	22.8964	22.6252
NUP210	15.1408	14.797	15.1303	15.9486	16.3878	20.1049
NUP214	21.2234	21.5025	21.3383	21.1452	21.1735	21.1118
NUP35	20.1421	19.9254	20.5116	20.6637	20.092	20.4769
NUP37	19.9489	20.31	20.851	20.5982	19.986	20.3472
NUP43	20.9665	21.1147	21.2625	21.1264	20.9805	21.3148
NUP50	21.2384	20.9429	21.4928	21.8568	20.7766	21.2817
NUP54	21.0432	21.1503	21.3736	21.3032	20.8013	21.2136
NUP58	20.4752	20.7137	20.6902	20.8135	21.11	21.1321
NUP62	19.7854	19.534	19.9841	19.958	19.4923	19.9974
NUP85	20.7942	21.0452	21.3941	21.4693	20.8339	21.5088
NUP88	20.4273	21.1157	20.9469	21.1907	21.1317	21.0144
NUP93	23.4746	23.8599	23.7703	23.764	24.0421	24.228
NUP98	22.0099	22.1565	22.6132	22.6482	22.343	22.0825

NUTF2	20.6788	20.4219	20.2482	20.1358	20.7188	21.6869
NXF1	20.5066	21.032	20.8976	20.8413	20.5427	21.0172
OAT	24.7257	24.0554	25.4378	25.0798	24.6377	23.998
OBSCN	16.3088	15.6102	17.4875	16.8531	16.6613	16.4679
OCIAD1	20.9604	20.2113	20.4949	20.5617	20.1054	20.4996
OCIAD2	18.092	19.7433	19.6063	19.3489	20.2919	23.4216
OGDH	23.8653	23.6615	23.7296	23.6828	23.5178	23.3566
OGFOD1	18.4864	17.4708	18.9073	17.9752	16.8883	20.055
OGFR	20.9106	21.755	20.7779	20.5178	21.6988	20.9992
OGT	19.9336	20.6953	19.9322	19.6822	21.1439	19.852
OLA1	23.4865	21.401	23.6171	23.1212	21.8187	23.6776
OPA1	22.6905	22.7276	23.3084	23.1603	22.9826	23.634
OPA3	17.5689	16.756	17.1668	17.4758	15.7522	18.034
OPTN	17.5728	15.9222	15.9346	15.1397	14.2658	14.3701
ORC2	15.3246	16.7319	17.6332	17.3927	16.3769	17.3715
ORC3	17.2551	18.5308	18.8271	18.9167	18.2761	18.7802
ORC4	15.6127	16.7186	17.059	17.5916	16.8302	17.8623
OS9	20.1149	23.2809	19.7843	20.6058	24.1593	20.5697
OSBP	20.8744	20.6409	20.6682	20.1452	21.0298	20.4436
OSBPL10	16.5743	14.14	15.6414	14.7002	14.2446	19.3478
OSBPL11	13.7493	15.7614	14.2678	14.3418	13.6313	11.0084
OSBPL1A	19.6266	20.3134	18.4323	18.732	19.4864	15.0719
OSBPL8	19.5244	20.1239	19.3945	19.3627	20.0811	18.7818
OSBPL9	19.2907	19.2766	19.2639	18.9646	19.2236	19.5124
OSGEP	17.917	17.1195	15.5244	14.9801	17.3091	16.4316
OSMR	15.2293	14.2417	17.4108	17.1211	15.0198	14.1048
OSTC	20.411	21.7344	21.2893	21.4844	21.7053	19.751
OTUB1	23.0007	22.1195	21.7304	21.5066	22.5775	23.1878
OVCA2	19.834	19.1482	20.0359	19.9893	19.7923	20.3305
OXA1L	16.8356	17.8924	18.3271	18.3642	18.2334	18.515
OXCT1	21.5043	21.0185	20.46	21.0371	21.1148	20.3985
OXSR1	20.6031	21.2102	20.0866	19.5227	21.7614	21.8736
P3H1	23.207	24.125	24.0407	24.202	23.7396	22.4189
P3H2	19.7104	21.9712	23.0255	23.3691	21.1886	21.091
P3H3	19.7845	21.9224	21.1255	21.0755	21.8778	20.3199
P3H4	18.7568	20.7289	20.9815	21.0796	20.4214	19.0261
P4HA1	24.9664	25.4203	24.9437	25.1402	24.7514	24.8617
P4HA2	24.4186	26.4382	24.5077	24.8352	25.9836	24.3348
P4HB	27.7655	28.157	27.7345	27.8794	27.9512	27.2666
PA2G4	25.0481	23.9513	24.5776	24.4667	24.4176	25.6229
PAAF1	19.3453	16.6278	19.7944	19.3804	16.9343	19.4472
PABPC1	25.5219	25.2851	25.2825	25.1591	25.5351	26.0711
PABPC4	22.6996	22.6984	22.274	22.3674	22.876	23.325
PABPN1	22.2736	22.5571	22.3697	22.4675	21.7923	21.9649
PACSIN2	22.6194	21.3711	21.7769	21.5	21.7646	21.7486
PACSIN3	17.055	17.3053	19.2743	19.2435	17.2736	18.64
PAF1	20.716	20.9834	21.8338	21.6756	21.1137	21.4466
PAFAH1B1	22.2291	20.9515	20.5201	20.1898	21.7757	22.1774
PAFAH1B2	21.5382	20.5021	20.437	20.1153	21.492	20.8874
PAICS	25.199	24.2869	24.583	24.311	24.6462	27.3824
PAIP1	20.7811	19.3143	20.0524	19.9296	19.5544	21.1538
PAK1IP1	14.3474	18.0747	17.7881	17.3963	17.0887	19.0696

PAK2	22.7228	21.7374	21.8629	21.4089	22.6268	23.387
PALLD	23.2431	21.4956	20.5498	20.5415	22.2499	22.3027
PAPOLA	18.1675	18.4003	17.2255	16.0248	19.015	18.5529
PAPSS1	20.5986	21.0833	19.8589	19.5868	21.0352	19.7412
PAPSS2	21.0397	22.4558	21.4233	20.8104	23.1427	19.7532
PARK7	25.1817	24.1749	23.647	23.7738	24.4695	25.2546
PARN	18.9241	18.8085	19.3214	19.5656	18.83	18.7211
PARP1	22.7055	23.7914	23.7088	23.818	23.4092	24.1805
PARP14	19.2222	18.3469	18.0398	17.7446	17.5097	17.9763
PARP4	18.108	16.722	17.6913	17.0649	17.5768	16.7271
PARP9	18.2379	17.0118	16.3446	15.6135	14.9858	17.9489
PARVA	21.2181	21.3802	20.1227	19.7319	21.8269	20.1055
PAWR	10.7678	11.8249	13.3876	13.2827	7.19606	13.457
PBDC1	19.3591	18.5312	18.6673	17.636	19.5827	20.3016
PBK	21.3015	20.2104	20.0649	20.3337	20.4529	20.9066
PBRM1	15.3626	17.9135	17.734	17.807	17.6983	14.9841
PBXIP1	17.8198	18.7887	19.7186	19.8449	18.6103	19.0742
PC	23.739	23.1663	23.5175	23.5187	23.0439	21.8429
PCBP1	24.4842	23.9767	23.8662	23.895	24.1866	25.1401
PCBP2	24.0945	23.7545	24.1797	24.0278	23.83	24.5717
PCCB	18.0356	18.159	18.6067	19.0094	18.0187	17.4639
PCK2	21.211	19.333	20.6026	20.5874	20.4034	20.53
PCMT1	21.0227	20.2932	20.3784	20.2992	20.5624	21.3636
PCNA	25.0503	24.3395	24.4162	24.618	24.4278	24.8066
PCOLCE	18.4239	18.2761	15.393	16.9049	16.0198	14.4273
PCYOX1	23.0265	22.8798	22.659	22.711	22.6082	22.2973
PCYT1A	21.435	21.2911	21.1421	21.1044	21.5619	22.1039
PDAP1	18.187	14.4965	16.2934	16.0841	15.7287	18.661
PDCD11	12.2833	17.8454	17.0469	16.6194	16.9568	18.0654
PDCD4	16.3119	19.1228	18.5415	17.9867	20.5428	17.9361
PDCD6	22.076	21.425	21.9155	21.8016	21.6442	22.5006
PDCD6IP	24.3524	24.1605	23.5877	23.0951	24.7892	24.773
PDCL3	18.0894	17.0044	17.8725	17.6846	17.3741	19.9735
PDE12	20.0986	19.9468	19.9212	19.9081	20.158	22.1901
PDE1C	14.8444	15.0811	15.7246	15.7614	14.8284	20.9465
PDE5A	22.182	17.5501	21.1239	21.19	17.276	17.359
PDF	20.2154	18.3493	19.3735	19.7346	18.9406	19.7742
PDGFRA	17.1345	17.9082	9.96694	NA	15.3881	12.94
PDGFRB	21.8936	22.0369	22.1766	22.1781	21.5441	20.3516
PDHA1	21.4374	20.3252	21.7948	22.0192	20.6221	21.6192
PDHB	23.0579	22.7917	23.3833	23.6273	23.1219	23.5237
PDHX	20.8873	20.6811	21.338	21.1711	20.6516	21.2654
PDIA3	27.7569	27.6483	27.6183	27.8777	27.5979	26.8101
PDIA4	27.6851	27.0797	27.3972	27.4318	27.3212	26.7691
PDIA5	20.3083	22.6459	20.2255	20.4475	22.5826	21.0899
PDIA6	26.8244	27.1391	26.7421	26.9764	26.6919	26.5944
PDK1	18.5017	18.3535	18.8798	19.2739	18.2987	19.4914
PDK3	17.1413	11.4544	16.4853	16.4274	10.6609	14.211
PDLIM1	24.7626	23.6716	21.5029	21.0665	23.9938	22.7386
PDLIM4	19.9402	22.0049	20.0794	19.9704	22.4336	22.6466
PDLIM5	21.9763	20.5122	18.729	18.1859	21.5953	21.9921
PDLIM7	22.8266	23.4476	22.5114	22.4485	23.8557	23.234

PDP1	19.4003	19.2316	19.7151	19.4876	19.108	20.555
PDPR	22.4611	22.2277	22.7956	22.7664	22.7	23.1816
PDS5A	18.7395	20.6255	20.2285	20.4338	20.8524	20.3273
PDS5B	16.0489	18.1772	17.8785	18.1026	18.6292	17.7818
PDXDC1	20.3134	18.3281	19.1315	18.6847	19.1589	18.3988
PDXK	19.9006	19.2302	19.3177	19.1433	18.3458	18.6161
PEA15	17.3193	15.7562	14.522	14.6918	16.2407	17.3626
PEBP1	24.27	22.782	22.2106	22.3922	23.0879	23.8848
PEF1	19.8202	20.0425	19.9556	19.6355	20.2751	19.9483
PEG10	15.0523	14.6512	14.2859	14.8107	13.1191	18.4255
PELO	19.0493	19.1842	19.3217	18.9567	19.7671	19.3387
PELP1	20.4124	21.7017	21.7472	21.6673	21.6611	20.9923
PEPD	22.6798	21.4664	20.467	20.2403	21.6385	22.078
PES1	19.1991	19.701	20.0654	19.6439	19.6665	20.7417
PFAS	21.3676	21.2233	21.2411	20.8667	21.4817	22.3012
PFDN2	18.9825	18.3734	18.9933	18.3218	19.0529	19.9355
PFDN5	19.0427	18.2397	18.9654	18.5137	18.1757	19.1965
PFKFB3	9.96497	12.5194	11.5018	11.1611	12.7116	17.1464
PFKL	23.869	21.9513	22.9746	22.791	22.3007	22.6656
PFKM	21.5051	20.0119	20.8623	20.2498	20.7254	21.0764
PFKP	24.9102	23.4874	23.7931	23.4683	24.1507	24.8602
PFN1	27.2772	27.1038	26.3428	26.2712	27.4658	28.0076
PFN2	21.0902	20.0845	21.0583	21.1922	20.7959	21.5694
PGAM1	26.4893	25.26	25.2966	25.4577	25.3497	26.9596
PGAM5	19.1606	18.9465	19.5863	20.2692	19.4108	20.1524
PGD	24.6131	23.395	25.3982	25.3056	23.9612	25.7928
PGK1	28.3084	26.5921	26.5046	26.5044	26.7855	28.8707
PGLS	23.8253	23.1487	21.9647	21.8911	23.2916	22.5929
PGM1	23.4676	22.1178	22.4597	22.2725	22.5696	22.9954
PGM2	20.5512	20.1443	19.8036	19.254	20.7911	21.8884
PGM2L1	19.9503	17.6045	14.8375	14.2772	18.8122	20.9259
PGM3	18.9376	18.5263	16.3308	15.2754	19.5959	18.5759
PGM5	18.8244	14.7611	16.931	15.6871	15.0092	13.3283
PGP	21.1139	22.6216	20.993	20.9652	22.34	20.487
PGRMC1	20.5249	22.27	22.3704	22.3203	22.5895	22.5713
PGRMC2	22.6176	22.9613	22.4787	22.5509	23.0216	22.3763
PHB	25.3843	26.0835	26.2622	26.4485	25.9162	26.0781
PHB2	24.9397	25.5917	25.7682	26.0775	25.2364	25.6846
PHC2	18.7869	17.0618	19.0043	18.9598	16.143	18.4571
PHF14	16.331	16.7459	18.0849	18.1522	16.6266	15.4652
PHF5A	20.9039	20.9474	20.3118	20.84	21.0334	20.3732
PHGDH	25.4122	23.2044	24.7315	24.2704	24.5259	26.0916
PHLDA1	18.5418	18.5463	19.2891	19.1399	18.8806	17.7127
PI4K2A	18.1975	19.4347	19.9614	20.2132	18.8744	18.7422
PI4KA	14.5894	16.5584	16.5027	15.6679	15.9306	13.3274
PICALM	21.5481	22.2328	21.5869	21.7102	22.2481	21.3285
PIGK	18.2555	19.1418	19.6456	19.4886	18.9722	17.274
PIGS	19.3463	20.4961	20.9034	20.9626	20.31	20.2393
PIGT	21.5741	21.6573	21.9945	21.922	21.8372	21.9077
PIGU	16.7132	17.6087	18.6156	18.9727	17.3908	17.2272
PIK3R4	15.2461	17.3584	17.3124	16.9144	17.797	16.7805
PIN1	18.4738	15.6864	17.4806	17.2841	16.6413	19.4756

PIP4K2A	18.8881	18.409	18.491	18.5782	18.2821	19.0377
PIP4K2C	20.9504	25.559	20.8615	20.7399	25.9355	21.4203
PIR	21.5662	17.2039	20.5299	20.5705	17.2371	20.0738
PITPNB	22.5688	21.7413	21.5953	21.1368	22.2737	22.4325
PITRM1	21.4363	22.3684	21.8918	21.9355	22.6987	21.777
PKM	30.9736	28.9994	29.4072	29.104	29.3672	30.6017
PKMYT1	16.2654	10.8331	15.9328	16.0809	NA	9.78379
PKN1	16.9041	15.1554	16.9022	16.0113	15.4043	17.0037
PKN2	20.6542	19.9459	20.6625	20.3646	20.2967	21.0223
PLA2G15	18.7251	19.5567	19.9635	20.6058	19.6718	20.6479
PLA2G4A	20.9009	18.0375	18.7151	18.8087	18.698	18.36
PLAA	19.1196	19.8135	20.3738	20.17	20.2746	22.3608
PLAT	11.9063	12.6452	14.6418	15.3539	10.3846	16.9843
PLAU	17.61	16.7042	17.9879	16.7923	17.0658	16.9719
PLBD2	22.4776	22.6563	21.7927	22.2036	22.3672	21.1689
PLCB4	12.8127	15.8289	13.4812	14.3891	15.3694	12.6888
PLCG1	20.1522	19.0463	20.2314	20.1064	19.1944	20.2697
PLD3	21.3129	22.2651	21.8148	21.8104	21.5991	20.4722
PLEC	28.4369	27.6642	28.3374	28.5718	27.7218	26.9911
PLIN3	23.8414	24.3247	22.9872	22.8892	24.7581	24.2119
PLOD1	23.2057	24.8865	23.9628	24.2505	24.1992	24.539
PLOD2	24.4848	25.806	25.7457	25.6216	25.448	24.9144
PLOD3	24.0339	24.7578	24.1831	24.3603	24.3441	24.6058
PLP2	21.5443	20.6464	21.7346	21.7324	21.1387	20.4266
PLPP1	22.4766	19.2206	20.9793	21.3106	18.8114	19.5531
PLPP2	17.7785	20.1521	19.1558	18.9486	19.9207	19.5569
PLPP3	19.1832	20.3135	18.4953	17.3384	20.8747	17.7781
PLRG1	21.0801	21.3395	21.1311	20.9191	21.2167	21.3291
PLS3	24.8553	24.2953	23.7435	23.4612	25.0803	25.0787
PLSCR1	19.0563	19.4878	18.3895	18.6193	17.9221	17.9069
PLXNB2	20.9968	21.5452	21.0794	21.4868	20.7777	20.1202
PML	22.3681	23.3372	22.7845	22.7976	23.0538	21.8059
PMPCA	21.029	21.5217	21.7055	22.0971	21.1578	22.185
PMPCB	21.7237	22.4518	22.6894	23.0014	22.0434	22.9737
PMVK	17.9682	15.9512	16.468	16.1636	15.3543	17.5594
PNKP	18.4426	19.27	16.9092	17.691	18.1914	18.6037
PNN	21.5266	21.5173	21.8691	21.9392	21.7661	21.8033
PNO1	15.2274	17.4572	18.1298	17.8379	16.8097	18.4374
PNP	21.4121	21.3759	19.9034	19.3174	22.4774	21.6549
PNPLA6	17.3473	19.6646	17.4615	17.5057	19.2375	17.4276
PNPO	13.6932	15.1136	16.3165	16.1903	13.0383	13.9613
PNPT1	22.2106	22.3824	23.1817	23.0762	22.5886	23.3704
PODXL	15.3381	19.8258	16.1374	15.7676	21.765	18.1882
POFUT1	22.7272	22.2624	22.4329	22.8005	21.9587	21.5846
POFUT2	20.8492	20.8641	21.6219	21.8895	20.4092	20.7874
POGZ	16.4665	18.1695	17.3342	17.5986	18.2266	16.5486
POLA2	18.3899	17.1867	18.6052	18.5816	17.2888	17.9059
POLD1	18.3664	19.6569	19.8835	19.7851	19.5743	19.5497
POLD2	19.1944	19.3881	19.3252	19.5843	19.147	18.7414
POLDIP2	21.0455	21.2338	21.809	22.0609	21.2501	21.9165
POLDIP3	19.3902	18.9975	19.7194	19.7089	19.0664	20.4389
POLR1A	18.1844	21.1069	20.1784	20.1614	21.7405	20.257

POLR1B	15.8175	16.625	17.9283	17.7278	14.9755	18.1246
POLR1C	19.9809	20.4553	20.8058	20.8811	19.9466	21.0885
POLR2A	20.7463	21.2758	21.1087	21.167	21.9051	21.3017
POLR2B	19.7632	20.5208	20.6831	20.7174	20.8547	21.8541
POLR2C	18.6679	18.8616	18.7058	19.0461	18.202	19.1324
POLR2E	17.4684	18.5378	18.0591	19.0075	18.5571	17.9357
POLR2G	18.4295	18.0779	18.5168	18.6757	18.7595	18.8708
POLR2H	19.1606	19.3131	18.5179	18.8709	19.2869	19.9461
POLRMT	15.6889	15.8924	17.0366	16.9401	16.7953	18.8171
PON2	22.0122	22.4463	22.8425	23.0348	22.2483	21.7198
POR	24.2469	23.1077	24.1517	24.2267	23.1304	23.7476
PPA1	23.4756	21.344	22.5467	22.0758	21.6295	24.2463
PPA2	22.2559	22.3959	21.7951	21.9469	22.4215	21.5636
PPAT	20.3417	18.8104	19.3003	18.6182	19.6173	22.6073
PPFIBP1	19.5781	19.6865	19.4831	19.022	20.1731	19.0167
PPHLN1	16.074	16.5332	16.1679	16.6399	16.495	16.8313
PPIA	27.9586	27.751	27.4481	27.5609	27.7788	27.7517
PPIB	26.5582	27.3375	26.975	27.2593	27.2338	26.6394
PPIC	21.988	22.3724	21.8207	21.9127	21.8335	21.2836
PPID	20.0397	19.4731	20.4623	19.9748	19.5887	20.9851
PPIE	19.9847	20.684	20.3013	20.7779	20.4554	20.2469
PPIF	22.7654	24.7513	24.0419	24.4835	24.7309	24.1929
PPIG	18.6825	19.524	20.8916	19.3194	18.9408	18.2214
PPIH	19.814	18.9631	20.1222	20.3737	18.9788	20.7054
PPM1A	19.289	17.5332	15.097	13.8369	18.0118	17.8158
PPM1F	21.1508	20.1888	19.5407	19.5055	20.3079	20.5212
PPM1G	22.0403	21.1093	21.1854	21.0546	21.267	22.9419
PPME1	19.3445	18.7208	18.91	19.2682	17.9179	19.3717
PPOX	18.2552	19.4899	18.4671	18.471	19.3705	18.649
PPP1CB	21.7354	21.7179	21.7485	21.548	21.8628	22.3903
PPP1R10	20.144	19.2167	19.5449	19.5447	19.7002	19.7101
PPP1R12A	19.284	18.5707	19.1236	19.1715	19.0284	18.1273
PPP1R18	18.7193	18.8697	18.7309	19.0189	18.4003	18.4933
PPP1R7	21.5178	20.1943	19.6986	19.6013	20.3747	20.8566
PPP1R8	20.2027	19.6538	19.9638	19.7072	19.245	19.8356
PPP2CA	22.3698	21.3934	21.3316	21.4238	21.7631	22.3701
PPP2R1A	24.238	23.1762	23.0081	22.9821	23.3427	24.0893
PPP2R1B	19.9194	19.893	19.3532	19.12	20.5005	19.7494
PPP2R2A	21.618	20.4524	20.1701	20.0782	21.0258	21.5317
PPP2R4	21.2848	19.5917	19.424	19.4439	19.9834	21.6073
PPP2R5C	16.9895	17.5375	16.432	16.3495	17.1264	17.4257
PPP2R5E	19.2406	18.8795	18.3505	18.0705	19.0502	19.7099
PPP3CA	20.5816	20.1636	19.7848	19.65	20.4079	19.8552
PPP4C	21.7177	21.3789	21.2939	21.2324	21.2139	21.3273
PPP4R1	18.5125	18.2815	17.6726	17.3071	18.0106	18.4916
PPP4R3A	18.9835	19.5401	19.5024	19.8507	18.6687	19.3513
PPP4R3B	17.456	17.3455	18.1049	17.365	17.5403	17.3885
PPP5C	21.5005	20.0758	20.0951	20.0378	20.5826	21.6404
PPP6C	19.6753	19.6084	19.7374	19.6297	19.4769	19.51
PPP6R3	18.0416	17.2817	18.6062	18.1622	17.4463	18.316
PPT1	21.3801	21.7092	21.1919	21.7816	21.5948	22.0783
PPT2	18.0803	19.7549	19.2713	19.6856	19.3949	19.331

PPWD1	18.5859	19.4169	19.3052	19.6588	18.9527	19.0661
PRC1	15.5557	14.1518	15.8454	16.0122	13.0746	14.1002
PRCP	21.1211	21.0339	21.0362	21.5579	20.6045	19.8555
PRDX1	27.9187	25.8357	27.6489	27.5524	26.3223	28.7601
PRDX2	24.2056	22.2442	21.8667	21.8913	22.4348	24.4269
PRDX3	24.6736	24.4115	25.0976	25.1217	24.6861	24.8264
PRDX4	24.9772	23.5964	24.5816	24.5512	23.3236	24.108
PRDX5	24.2683	24.4215	23.7119	23.6735	24.681	23.422
PRDX6	25.4264	24.6298	24.8369	24.4586	25.0471	25.2888
PREB	16.6554	16.6331	16.8776	17.8852	16.4993	16.6966
PREP	22.0452	20.8155	20.0788	19.7954	20.9412	22.9378
PRIM1	18.1766	17.2924	18.3137	18.2515	17.9431	19.089
PRKAA1	19.6893	19.1666	19.0666	18.7421	20.1387	20.4813
PRKACA	20.568	20.8851	20.7942	20.3609	20.9525	21.7732
PRKACB	18.9995	18.6363	18.7189	18.7657	19.2361	18.9318
PRKAG1	19.5837	18.753	18.6691	18.2851	18.5813	19.6007
PRKAR1A	23.8569	22.7748	23.2727	23.0293	22.9513	23.2798
PRKAR2A	21.2669	21.6197	20.4757	20.5505	21.883	22.5024
PRKCA	18.7794	19.3194	17.9187	18.8793	18.4425	18.9014
PRKCD	14.949	17.2322	17.0562	16.7858	17.2042	14.1089
PRKCDBP	19.1725	21.3888	19.3194	19.0531	21.9696	17.7199
PRKCI	17.4245	16.979	18.064	17.4429	17.9494	17.9637
PRKCSH	25.6838	25.158	25.2148	25.2924	25.364	25.6981
PRKDC	24.1029	24.5719	24.9888	25.369	24.5386	24.4779
PRKRA	21.1593	20.387	21.3517	21.1604	20.2574	19.9936
PRMT1	24.5337	24.9886	24.6981	24.6382	24.7669	25.5729
PRMT3	17.6059	15.4087	16.5766	16.6028	15.361	17.5224
PRMT5	22.6503	22.3663	22.0625	22.0795	22.9355	23.4914
PROCR	21.1425	20.7486	21.2624	21.4372	20.5088	19.2275
PRPF19	24.3655	25.0609	24.7823	25.0628	24.9006	24.5793
PRPF31	20.255	20.5397	20.4439	20.5532	19.2508	20.1208
PRPF38A	20.1741	20.3955	20.7291	20.8909	20.3585	20.795
PRPF38B	18.5951	19.9286	18.5652	18.717	20.6603	17.9559
PRPF4	22.2994	22.5423	22.5598	22.6359	22.3785	22.6302
PRPF40A	21.6079	21.7875	22.0884	22.042	21.9248	21.1946
PRPF4B	16.7079	18.4608	17.3761	17.9958	18.0674	17.7174
PRPF6	21.1262	21.9437	22.0489	22.1757	22.1887	22.2635
PRPF8	23.3994	23.9507	24.1892	24.2348	24.0425	23.9476
PRPS2	21.1684	21.7451	20.737	20.6929	22.2696	20.761
PRPSAP1	19.069	18.6573	18.9767	18.5382	18.9101	19.4657
PRRC1	21.2151	20.7444	20.8915	20.4593	21.5364	20.5272
PRRC2A	19.3694	19.4581	17.9418	18.5141	19.7758	18.6819
PSAP	22.0455	22.092	22.1428	22.2124	22.1845	23.3871
PSAT1	21.3962	19.3207	21.1218	21.3424	19.8972	23.5475
PSIP1	20.376	20.2405	20.0609	20.2169	19.849	21.0029
PSMA1	24.611	24.1365	24.0279	24.0035	24.5374	24.3835
PSMA2	23.1994	23.0096	22.7727	22.7392	22.8116	22.811
PSMA3	23.6225	23.2252	22.9898	22.7577	23.753	23.3969
PSMA4	24.0316	23.2491	23.4365	23.1834	23.4352	23.785
PSMA5	24.9614	24.9011	25.1041	24.6595	24.6485	24.7443
PSMA6	24.4712	23.8971	23.6165	23.5596	24.2396	24.2676
PSMA7	25.1868	24.4456	24.5219	24.4775	24.8962	25.1047

PSMB1	24.6895	24.19	23.7916	23.6354	24.6174	24.3922
PSMB10	21.5078	19.6629	19.6368	19.777	18.9133	17.7426
PSMB2	23.359	22.6361	22.6305	22.6699	22.9995	23.0614
PSMB3	22.4334	21.8155	21.7273	21.8976	21.2068	21.6965
PSMB4	22.3908	21.4225	21.5475	21.6031	22.1344	22.1345
PSMB5	21.9881	22.5744	21.9414	21.9676	23.2427	23.2648
PSMB6	21.4431	21.9264	21.3896	21.2566	22.7088	22.6157
PSMB7	21.8955	22.1185	21.6266	21.3433	22.681	22.1788
PSMB8	22.7948	21.0115	21.6827	21.5089	21.0203	20.4997
PSMB9	22.7277	21.2474	21.0092	21.287	21.0977	19.2861
PSMC1	22.6622	22.9218	22.4359	22.5762	23.7057	23.8342
PSMC2	24.7667	24.4236	24.3756	24.1755	24.9377	25.1033
PSMC3	23.7825	23.0565	22.797	23.0676	23.3559	23.718
PSMC4	24.2864	23.9397	23.979	23.8887	24.3806	24.5705
PSMC5	23.6669	23.0377	23.5482	23.4229	23.4716	24.2214
PSMC6	24.0227	23.4764	23.2129	23.2348	23.8453	23.9095
PSMD1	24.0962	23.99	24.2471	24.0724	24.4051	24.6291
PSMD10	20.0525	18.2032	19.689	19.1906	19.1003	20.4542
PSMD11	24.3012	23.9467	24.3171	24.2494	24.3342	24.9304
PSMD12	22.7779	22.8802	22.9022	22.7972	23.3262	23.5827
PSMD13	24.0983	23.9624	24.1503	23.9918	24.2528	24.4772
PSMD14	22.5803	22.2369	22.4405	22.4266	22.7484	22.9824
PSMD2	24.5632	24.6435	24.6061	24.3974	25.3075	25.3256
PSMD3	22.0904	22.3059	22.7178	22.3978	23.082	23.3715
PSMD4	22.8937	22.6987	22.5899	22.7503	23.1032	23.2072
PSMD5	22.2681	21.3734	21.0451	20.7213	21.7785	22.3215
PSMD6	23.4955	23.3243	23.4264	23.2856	23.7154	23.9896
PSMD7	23.2083	23.346	23.2558	23.1303	23.3984	23.4932
PSMD8	23.2433	22.9689	23.326	23.2542	23.3712	23.6097
PSME1	25.101	23.0135	22.2969	22.0107	23.2493	23.1282
PSME2	24.2752	22.8113	22.0349	21.8086	22.2784	22.0814
PSME3	21.5595	21.8956	21.606	21.6442	21.4192	22.8029
PSME4	12.7654	14.2834	15.3704	15.269	15.5885	17.3764
PSMF1	20.386	18.7286	19.9086	19.1831	19.4488	19.8488
PSMG1	20.0773	18.5621	19.5699	18.9061	19.2083	19.9099
PSMG3	18.2474	18.0043	17.9652	17.9429	17.5698	18.4678
PSPC1	22.5793	21.9933	22.1065	22.3864	21.5211	22.378
PTBP1	25.6576	26.274	26.0929	26.1018	25.838	26.0876
PTBP3	19.0125	19.0285	19.0498	19.2009	18.9907	18.703
PTCD1	14.2037	15.4373	16.5158	17.1032	15.4677	17.3046
PTCD3	19.2131	19.7656	20.4475	20.9686	19.8115	20.2572
PTDSS1	17.3579	17.3654	17.6846	17.6279	16.8103	17.4697
PTGES2	20.3217	20.5443	20.9887	20.986	20.3302	20.6661
PTGES3	22.6838	21.7196	23.0096	22.7724	22.3656	23.5473
PTGFRN	23.2106	18.8896	21.3422	20.7364	19.0397	20.2473
PTGR1	23.6583	20.7209	23.3342	23.0708	20.8423	20.5817
PTGS2	18.889	18.5676	15.4839	14.3295	19.3637	14.9893
PTK7	21.0121	22.275	22.0339	22.3594	21.2161	20.9285
PTPMT1	13.9494	14.1273	16.1937	17.1379	11.6132	15.7033
PTPN1	22.252	22.4104	23.3692	23.1524	22.7094	22.6121
PTPN11	19.9606	18.9546	18.8746	18.2596	20.1236	20.0422
PTPN23	18.7163	18.8742	18.3925	17.6207	19.8469	18.9606

PTPRF	13.5233	14.0375	15.1732	15.9912	13.6323	13.7483
PTPRK	18.7967	18.7344	17.8796	18.1688	19.201	17.6995
PTRF	23.0315	23.504	22.8113	22.8456	23.8489	23.0239
PTRH2	20.1318	21.2365	21.4246	21.4929	21.3871	21.9378
PTTG1IP	17.0011	18.5273	15.4801	17.3449	17.6788	13.7864
PTX3	11.2873	17.2256	16.4261	15.3585	16.1735	9.98585
PUF60	24.2419	24.1744	24.158	24.3211	24.0873	24.5144
PUM3	16.2481	18.5561	18.8901	18.481	18.076	19.8686
PURA	21.3681	20.7801	20.8136	20.2737	21.4472	20.5549
PURB	21.5617	20.6149	21.0231	20.6416	21.7571	20.7668
PUS1	18.1693	18.8964	19.096	19.4588	19.1099	19.4586
PUS7	17.8074	17.6862	18.1601	17.9537	17.4239	17.694
PVRL2	20.4563	20.9566	20.3151	20.4479	20.6095	20.4639
PWP2	18.9419	20.6025	21.8229	21.6053	20.5758	21.6844
PXDN	20.7566	21.6013	19.4431	20.1063	21.3718	19.8305
PXN	19.2183	18.6426	18.3506	18.4757	18.6976	18.9486
PYCARD	17.0465	15.6596	19.3907	19.3389	14.2984	12.0362
PYCR1	19.8533	19.542	20.9394	21.1805	19.6853	20.0493
PYCR2	21.3076	21.0047	21.3478	21.8723	21.3334	21.6512
PYGB	24.9081	23.3012	24.1055	23.6864	23.9852	26.3664
PYGL	21.295	20.2686	20.4535	20.2678	20.8515	22.233
PZP	23.1916	23.6352	19.5094	20.4362	23.6463	17.7378
QARS	24.1502	24.0914	24.242	24.0934	24.4697	25.1417
QDPR	19.657	19.0386	18.1125	18.3482	19.3853	19.5175
QKI	21.6082	21.3494	21.0852	21.0899	21.5405	22.5424
QRSL1	17.2785	16.4651	17.1307	17.0936	17.1233	16.541
QSOX2	20.6414	21.0583	21.8318	21.5527	21.4698	21.2361
QTRT1	17.099	17.1319	17.2066	17.1893	17.6911	17.7201
RAB10	22.3452	21.9983	21.9174	22.0153	22.4351	22.3094
RAB11B	24.4461	24.8802	24.601	24.8777	24.7607	24.691
RAB11FIP5	14.5101	18.0303	17.4652	17.3768	17.4407	14.5211
RAB12	18.9132	19.1358	19.5591	19.6016	18.9378	20.1615
RAB13	21.274	22.504	21.4048	21.6037	22.8072	21.4294
RAB14	22.3303	22.0162	22.3884	22.3302	22.4728	22.1137
RAB18	21.588	21.4014	21.7612	21.629	21.5406	20.9396
RAB1A	22.4138	22.7104	22.6968	22.8534	22.7338	21.6915
RAB1B	22.5995	22.8703	22.5094	23.0193	22.4345	21.3425
RAB21	21.6023	21.9959	21.1888	21.3078	22.066	21.0828
RAB22A	17.2396	18.3101	18.0579	18.2316	17.5569	17.9172
RAB23	18.2401	18.2855	17.3917	18.0517	18.7206	18.38
RAB27A	19.9324	20.2378	20.5726	20.0365	20.4119	20.3365
RAB2A	24.4435	24.2917	24.8705	24.9152	24.2877	23.5849
RAB31	20.1531	20.659	19.9732	20.0473	20.4953	19.2818
RAB32	19.3423	21.439	21.0461	20.2418	22.0432	21.3964
RAB34	18.9444	21.2798	21.3748	21.227	20.9228	20.5551
RAB35	23.0857	20.4962	20.7893	21.3528	19.517	19.8224
RAB3GAP1	23.37	23.8885	23.0762	22.9052	22.6768	22.7321
RAB3GAP2	19.8608	19.6487	18.8153	18.2101	20.3312	19.6383
RAB5A	19.2334	20.1694	19.9419	20.1102	19.4837	19.3235
RAB5B	20.0085	19.7153	20.6422	20.3164	19.7826	18.0341
RAB5C	22.6994	23.0175	23.3369	23.5898	22.664	22.9303
RAB6A	21.7578	21.8526	21.9665	21.9122	22.1295	21.5294

RAB6C	19.3503	19.7732	19.0029	18.7489	19.6286	18.1918
RAB7A	25.3874	25.9316	25.0059	25.2115	25.8592	25.5591
RAB8A	19.4334	20.1159	20.0638	20.3868	20.0411	20.3708
RAB8B	18.3827	20.6538	20.1567	20.6086	20.7252	19.7699
RAB9A	16.8468	14.9606	15.9274	15.7131	14	13.9363
RABAC1	20.0421	20.2327	20.4001	20.3446	20.2861	19.5027
RABL3	17.6019	17.8139	17.5568	18.295	17.2147	17.2496
RAC1	21.6067	22.7473	22.2171	22.7546	23.3204	23.4519
RAC2	15.8271	21.0095	21.5031	21.1388	22.0037	16.0075
RACGAP1	19.1655	19.3126	20.202	20.3036	19.2923	19.1272
RAD21	20.9019	20.2876	21.3873	21.4958	20.281	20.4647
RAD23A	17.2849	15.8685	15.2361	14.8883	14.2947	16.6158
RAD23B	21.4065	20.9748	20.7295	20.6427	21.4054	21.9213
RAD50	21.5549	21.2267	21.6978	21.6331	21.3554	20.791
RAE1	21.2012	21.7999	21.8668	22.0251	21.7795	21.6122
RAI14	20.746	21.2395	19.849	19.508	21.7048	19.2589
RALA	20.7442	22.764	22.6367	22.7074	22.9448	21.5344
RALB	17.5843	21.0385	19.6626	19.9731	21.3083	18.2713
RALY	23.5396	23.1715	23.9291	24.3548	23.2794	23.3257
RAN	26.2405	25.9625	25.8987	25.7549	26.1115	26.4462
RANBP1	22.3433	21.7523	22.0174	21.6405	21.7428	21.7537
RANBP2	21.8993	22.9637	22.462	22.6042	23.1275	21.737
RANBP6	16.4602	17.1034	16.2219	15.9153	16.4573	18.1893
RANBP9	18.7775	18.2152	18.7701	18.8456	18.3289	18.6717
RANGAP1	23.5111	23.4851	23.6303	23.5679	23.7617	23.837
RAP1B	18.9341	20.9699	21.2689	21.3436	21.6006	20.9913
RAP1GDS1	20.9829	20.6388	19.5788	19.077	21.0168	20.7593
RAP2B	18.7615	19.9635	19.1016	19.619	18.7074	19.6282
RAPH1	19.399	17.3365	16.6428	17.4276	16.7574	16.8491
RARS	24.9755	24.3113	24.6504	24.4181	24.8932	25.4399
RARS2	16.6744	17.4246	17.2841	17.5647	16.4479	17.4645
RASA1	18.1791	15.43	17.2726	16.0299	16.4274	16.5936
RASA3	17.8725	19.5945	19.0327	18.7453	19.8858	17.2736
RAVER1	21.3084	21.4753	20.6897	20.7696	21.5519	21.8941
RB1	18.7322	15.5545	17.4573	16.6153	16.0616	17.9676
RBBP4	22.6315	22.898	22.6328	22.8393	22.7405	22.9179
RBBP5	19.3992	19.1183	19.6686	20.1557	18.8897	19.3462
RBBP7	21.675	22.0289	22.2938	22.474	21.5717	21.4794
RBFOX2	19.2	18.9734	19.5642	19.2316	18.8921	18.7968
RBM10	20.0169	19.5168	20.6813	20.9606	19.6766	19.0613
RBM12	21.1588	21.1343	20.8028	20.7743	21.0003	21.5271
RBM12B	19.9171	20.0617	20.1783	20.1497	20.3142	20.5764
RBM14	23.0995	22.7326	23.4835	23.8649	22.6959	23.9474
RBM15	20.2605	19.9324	20.6352	20.8208	20.2074	20.3307
RBM17	20.2052	20.0699	20.437	20.6919	19.8865	20.7869
RBM22	18.1641	18.2796	18.4326	18.4099	17.2839	18.0885
RBM25	22.0671	22.1072	21.9257	22.2222	22.0973	22.0481
RBM26	18.0782	17.2369	18.4162	18.2416	18.031	17.7751
RBM27	17.3566	16.3603	18.4596	18.4387	15.0978	17.5747
RBM3	22.1191	21.8392	21.4679	21.3027	21.9149	22.0756
RBM39	23.2398	22.9116	23.0686	23.3687	22.6285	23.0952
RBM4	22.0222	22.37	22.6571	22.7551	22.1951	22.9137

RBM42	19.0766	18.9518	19.8787	19.7746	18.8284	19.785
RBM45	17.4158	17.4016	18.6338	18.5124	16.4682	16.5225
RBM5	17.1534	17.9998	17.5794	17.9112	17.655	17.3238
RBM8A	20.4421	20.7064	20.9114	21.0063	20.9256	21.2271
RBMS1	19.8335	20.2127	19.9307	19.6555	20.9659	19.8495
RBMX	21.8394	21.7081	21.5757	21.9458	22.0758	22.2513
RBPJ	19.079	19.7422	18.4564	18.2898	19.7633	18.48
RBX1	18.0463	18.1986	18.2606	18.3133	18.5132	17.9902
RCC1	23.1782	23.47	23.2265	23.5629	23.1082	24.2466
RCC2	23.7838	23.7973	23.6453	23.7532	23.5869	25.0627
RCE1	16.0824	16.9897	17.3421	17.0088	16.7	16.4067
RCL1	16.3952	18.5844	19.1228	18.7321	19.4052	19.8
RCN1	23.0685	23.6877	23.5101	23.4209	23.6595	22.4128
RCN2	18.9462	18.565	19.8848	19.8908	17.9359	19.2623
RCN3	16.3892	19.7312	16.0761	16.3666	19.8476	15.2947
RCOR1	21.1391	21.6702	21.0529	20.4977	21.6089	21.0908
RDH11	20.508	21.4163	20.2339	20.6416	21.4153	20.6332
RDX	23.3458	22.5364	23.0213	23.0382	22.612	23.4938
RECQL	23.7384	23.8241	23.4132	22.9291	24.2458	23.2082
REEP5	21.7363	20.7867	21.0724	21.5267	21.5113	21.2931
RELA	19.504	18.756	17.7835	16.8533	19.5393	19.2567
REPS1	16.6423	17.1939	17.0102	17.1252	17.5336	17.2605
RETSAT	17.3128	18.3667	17.5507	17.4365	16.8777	16.7399
REXO2	20.9501	21.4169	20.2568	20.3951	21.8996	20.2529
RFC2	19.2528	19.475	19.4783	19.8019	19.6806	20.341
RFC3	26.9429	25.9316	25.3112	25.1044	26.487	26.9407
RFC4	21.4918	21.1742	21.346	21.4572	21.384	22.0685
RFC5	22.1944	21.4365	21.3363	20.9827	22.2042	22.4078
RFT1	15.3418	16.7713	17.8283	17.6223	16.7649	16.1065
RFTN1	20.1206	22.7058	20.9723	21.3372	22.4679	16.939
RFX1	13.0691	16.1921	15.5335	15.463	15.629	14.1321
RHEB	21.9724	21.2952	21.69	21.6458	21.6614	22.3718
RHOA	20.2529	20.5651	21.1471	21.4346	20.1355	21.062
RHOC	20.51	21.6648	20.9796	20.9257	21.8035	22.1344
RHOG	20.27	22.2859	21.5518	21.7228	22.4263	21.7583
RHOT1	17.8539	17.0793	19.3442	18.9112	17.9877	19.064
RHOT2	19.6393	20.1886	20.8669	21.1978	20.2211	20.9562
RIC8A	19.72	19.4216	19.409	18.8329	19.6731	19.2866
RIF1	19.367	19.2684	20.5296	20.6817	19.8071	19.9986
RIN1	15.4115	16.8302	14.9226	14.8803	15.7459	12.8292
RING1	17.5667	16.7555	16.7173	17.2464	17.0482	17.1438
RINT1	18.5485	19.1558	18.3172	18.1921	19.4545	18.8054
RIPK1	18.9232	18.4787	18.3949	17.4559	19.2922	19.285
RMDN1	19.8994	19.3476	19.9668	19.8808	19.6403	19.9204
RMDN3	20.1108	19.3811	20.9966	20.8572	19.5251	20.2649
RNF114	18.3945	17.1169	19.103	18.2404	17.3126	19.3385
RNF20	18.4062	18.4859	18.7287	18.3756	18.5301	18.8412
RNF213	18.814	18.8835	19.3047	19.3498	18.6737	19.3025
RNF40	19.3338	19.1719	19.3599	19.1889	19.5841	19.0144
RNGTT	17.5792	16.7155	16.8987	16.5529	16.7385	17.6082
RNH1	25.3635	24.1223	24.114	24.1152	24.3717	24.2301
RNMT	19.1317	20.5889	19.2846	19.4449	20.4132	20.3821

RNPEP	23.315	21.6567	21.0341	20.5177	22.2119	22.3029
RNPS1	20.0162	20.2218	20.0992	20.6818	20.1331	20.0101
ROCK1	18.6084	17.0907	16.9141	16.2757	17.9887	17.0428
ROCK2	19.8186	18.4832	18.5522	18.1468	19.024	18.5642
RP2	21.5334	21.8845	21.9647	21.5656	21.9492	22.1054
RPA1	23.1948	22.5701	22.191	21.9695	22.4441	23.3184
RPA2	19.3348	19.1081	19.2565	19.4042	17.4881	18.0273
RPA3	20.5897	20.2618	19.8343	19.9888	20.2046	20.8008
RPAP3	18.3898	17.8863	18.1657	17.5538	18.4511	18.5195
RPE	19.0459	17.6552	18.7006	18.4041	17.8217	19.1177
RPF2	14.9333	16.2165	15.1707	14.5623	16.373	17.2693
RPIA	15.9684	15.7032	16.0195	15.3758	15.3921	18.4404
RPL10	21.0243	21.6673	21.6009	21.8943	22.2606	22.4867
RPL10A	20.9687	21.4578	21.6294	21.4521	22.0211	23.027
RPL11	23.4526	23.9087	23.8988	23.8388	24.0435	24.311
RPL12	25.013	25.6508	25.4337	25.5924	25.8805	26.0626
RPL13	21.293	22.6999	22.9579	22.6825	23.2723	23.7927
RPL14	20.6223	22.0008	21.1954	20.9988	22.0326	22.3891
RPL15	21.3755	22.0613	22.2013	21.9915	22.1891	22.9901
RPL17	21.2151	22.1562	21.7641	21.6179	21.8432	22.8157
RPL18	19.3009	20.3493	19.7666	19.797	20.3664	21.4537
RPL18A	22.2398	23.0819	23.0832	22.8065	23.7222	23.8816
RPL19	19.0491	19.0382	18.7769	18.4431	19.6448	20.2604
RPL21	21.234	22.32	22.0171	21.9996	22.6118	23.3823
RPL22	22.314	23.2555	23.4725	22.9955	23.8715	24.2157
RPL23	21.4808	21.8111	22.0849	22.2091	22.4436	23.4073
RPL23A	22.2628	23.0013	23.0476	23.0121	23.383	23.8967
RPL24	21.4586	22.0882	21.6701	21.4905	22.3919	22.6927
RPL26	21.3583	22.5678	21.9025	21.814	22.9929	23.367
RPL27	22.3886	23.566	23.4589	23.1394	24.0543	24.62
RPL27A	22.6443	23.4508	23.9059	23.4944	24.0557	24.4386
RPL28	17.781	18.5883	18.5706	18.4902	18.9228	20.0419
RPL3	22.1954	22.8295	22.9899	22.7152	23.2624	23.9945
RPL30	23.2	23.8794	23.9041	23.5861	24.2043	24.7748
RPL31	19.5262	20.8091	20.2173	20.4848	21.106	21.4808
RPL32	18.1738	17.9558	19.2235	19.2705	18.1797	19.5519
RPL35A	20.9181	21.5905	21.7564	21.2999	22.3767	22.7507
RPL36A	18.6957	19.0812	19.1183	19.1671	19.8208	20.4386
RPL37A	19.1867	20.7451	20.8641	20.5624	21.1696	21.587
RPL38	21.5366	20.9667	22.3843	22.1611	21.1516	23.4224
RPL4	24.2263	24.73	24.6004	24.412	24.8512	25.4902
RPL5	23.5535	24.2386	24.0965	23.7729	24.559	24.6057
RPL6	22.1577	22.5879	21.7786	21.8212	22.8409	23.3714
RPL7	21.2382	21.8976	21.8541	21.7164	22.4875	23.5199
RPL7A	21.8883	22.9593	22.874	22.6514	23.4471	24.1857
RPL8	21.0245	22.2047	21.6485	21.6181	22.5589	23.0365
RPL9	21.8073	22.6502	22.8333	23.0101	21.9774	23.6081
RPLP0	25.7061	25.7749	25.9075	25.7968	26.1973	26.8301
RPLP1	21.4044	21.6454	22.3256	22.3927	21.6558	22.334
RPLP2	19.8952	20.6073	20.5213	20.4601	20.9443	20.7793
RPN1	27.3907	27.3331	27.1106	27.0712	27.5933	27.1644
RPN2	25.1744	26.2679	25.7951	25.8498	26.1884	24.9293

RPP30	19.4715	20.4746	20.7815	20.6856	20.2095	20.4804
RPP40	15.3753	16.678	17.7824	17.6275	15.1115	17.0619
RPRD1B	21.0762	20.6714	21.5546	21.4698	20.7667	20.8955
RPRD2	19.9078	20.4683	20.1402	20.0814	20.6352	20.5688
RPS10	24.5786	24.4499	24.5424	24.6775	24.712	25.2338
RPS11	22.3722	23.3116	22.7955	22.9633	23.6929	24.1385
RPS12	24.0137	24.1583	24.5452	24.3831	24.357	24.481
RPS13	22.1989	23.299	23.2995	22.9893	23.6017	24.2274
RPS14	23.0506	23.3194	23.4981	23.3834	24.1317	24.7309
RPS15	17.2872	17.6057	18.5468	19.267	17.0891	17.8416
RPS15A	23.2716	23.6949	23.9964	24.0097	24.1444	24.4638
RPS16	23.2872	24.0364	23.8335	23.7675	24.6913	24.9683
RPS17	22.0111	22.7712	22.2478	22.2949	22.8185	23.5312
RPS18	22.5982	23.4203	23.5918	23.2422	23.801	24.094
RPS19	23.2181	23.5639	23.3586	23.2788	23.9274	24.3252
RPS2	24.0557	24.6751	25.0012	24.7651	25.1975	25.8118
RPS20	23.68	24.1457	23.8133	23.6871	24.4836	24.8809
RPS21	20.5242	20.6378	20.6938	21.1949	21.4082	21.6571
RPS23	22.0824	22.4799	22.7238	22.5897	22.7164	23.3919
RPS24	20.6363	21.5625	21.46	21.4546	20.6325	21.5041
RPS25	22.8192	23.7122	23.7543	23.6561	24.2177	24.7247
RPS26	21.7259	22.1431	22.1725	21.968	22.4424	22.8543
RPS27	21.6081	21.4669	21.4661	21.8792	22.2954	22.528
RPS27A	26.7859	26.7755	26.7808	26.8788	26.9801	27.1985
RPS28	21.7271	21.8275	21.735	21.7343	21.8775	21.8423
RPS29	17.773	18.5514	18.4736	18.4213	19.4894	18.8466
RPS3	25.6061	25.9366	26.0075	25.9076	26.2913	26.8851
RPS3A	23.9462	24.6939	24.5755	24.4602	25.1896	25.716
RPS4X	23.2959	24.0547	23.9899	24.1039	24.3303	25.1717
RPS5	22.1868	23.1354	22.8563	22.7776	23.6061	23.9355
RPS6	21.8389	22.0591	22.5504	21.8687	22.3941	23.5834
RPS6KA1	18.0668	13.9333	16.8053	16.2166	13.9654	17.4249
RPS6KA3	19.4059	15.5619	17.1103	16.4553	16.2098	18.0364
RPS7	22.7391	22.8232	23.2067	22.9496	22.6178	23.4248
RPS8	23.4422	24.3009	24.3824	24.1923	24.6429	24.9735
RPS9	20.5943	21.9341	22.5659	22.0115	22.2971	23.5717
RPSA	26.0517	26.0371	25.9234	26.0055	26.3989	26.7333
RPTOR	16.7018	16.4384	16.6382	16.37	15.1087	16.6128
RPUSD3	13.6009	15.2484	16.3409	17.0553	15.715	17.8181
RRAD	15.3651	20.1644	13.4597	14.08	21.8694	14.8132
RRAGA	19.5028	19.7202	18.4921	18.5828	19.3242	18.3319
RRAGC	19.601	18.95	17.9888	17.8226	18.8267	18.8078
RRAS	20.0908	21.5477	20.3039	20.3727	21.4418	19.3353
RRAS2	18.3198	20.446	19.0525	19.7158	19.5848	18.9667
RRBP1	24.2052	25.0554	24.6624	24.9333	25.3153	23.5844
RRM1	22.5371	20.7892	21.929	21.2944	22.1809	22.6961
RRM2	21.3244	19.1586	19.357	19.1101	19.5067	21.1113
RRN3	15.9628	16.0107	15.3767	14.5104	15.5671	14.6322
RRP1	12.7568	17.2753	17.3393	17.2875	17.0537	18.0847
RRP12	15.9488	18.4999	17.6838	17.2049	18.3216	18.943
RRP15	17.4809	18.1538	19.1458	19.1978	18.3853	19.5655
RRP8	17.1447	18.9651	18.6057	18.9353	18.7768	18.843

RRP9	19.0002	19.7957	19.7891	19.6641	19.3397	19.7838
RRS1	18.5477	20.0506	20.7592	20.5458	20.1865	21.0743
RSF1	17.3834	18.5407	18.9561	19.0105	18.2183	17.2267
RSL1D1	18.4068	19.8736	20.832	20.1097	19.9021	21.5917
RSU1	22.0829	22.1436	20.504	20.115	22.3819	20.7231
RTCA	19.5175	19.2216	19.0738	18.5213	19.2925	19.8665
RTCB	23.6177	23.4314	23.1575	23.0181	23.8966	23.4624
RTN4	25.3713	25.4486	25.47	25.1664	26.0479	25.0577
RTN4IP1	17.8243	17.7351	18.2966	18.3978	17.0658	16.9129
RUFY1	19.8654	19.1405	19.7853	19.7194	19.8289	20.4894
RUVBL1	24.6364	23.9648	24.378	24.3379	24.0954	24.3404
RUVBL2	24.2279	23.7019	24.0857	23.966	23.9174	24.0896
S100A10	19.4629	20.234	20.9253	21.3624	19.8239	19.2043
S100A11	21.3666	21.127	20.0855	20.2568	21.8784	20.5173
SAAL1	17.1645	17.7285	17.3459	16.5726	16.6634	17.5549
SACM1L	19.7167	20.5572	19.9416	20.2216	20.1676	19.6176
SAE1	22.2895	21.879	21.7125	21.4789	22.1307	22.5283
SAFB	21.823	22.2495	22.0606	22.1763	22.335	21.8308
SAFB2	19.551	19.8122	20.4319	20.2755	20.1667	19.6363
SAMD4B	17.1334	15.1393	16.2167	15.9052	15.4075	15.755
SAMD9	13.4413	12.9796	16.1413	14.9646	14.7417	14.7053
SAMHD1	21.9129	20.8611	21.4658	20.9807	20.6168	21.6819
SAMM50	22.6198	23.1682	23.4194	23.5271	23.2495	22.9628
SAP18	21.2574	21.7767	21.8455	22.3592	21.4807	20.9081
SAP30BP	17.5693	17.9158	17.8747	18.1806	18.1193	18.8653
SAR1A	17.2418	17.7425	17.3571	17.7674	18.131	18.0645
SARM1	16.8819	16.5395	16.9422	16.9927	15.025	13.6754
SARNP	21.6196	21.3249	21.665	21.8513	21.5154	21.5661
SARS	23.9406	22.3039	22.5392	21.9232	23.1097	23.6539
SARS2	20.1094	20.4525	20.1391	20.74	20.1121	19.9756
SART1	21.1566	19.8771	21.5532	21.3457	20.3621	21.9164
SART3	21.6394	21.768	21.5758	21.3401	21.6129	21.0034
SBDS	21.3581	20.6489	20.3094	20.1452	20.8683	21.3613
SCAF4	19.8449	19.6637	20.5223	20.462	19.617	19.4221
SCAMP2	19.7925	19.8384	20.4602	20.8181	20.2099	20.0801
SCAMP3	22.0484	21.931	21.9986	22.1328	22.2792	22.9827
SCARB2	23.344	23.1699	22.5088	22.195	22.5107	21.9562
SCCPDH	20.7253	20.5233	20.2021	20.4125	20.8995	20.5252
SCD	18.1009	18.2182	20.0076	20.3955	17.4082	19.8942
SCFD1	22.145	22.1201	20.8281	21.0062	21.695	20.6528
SCFD2	17.2479	18.4669	17.4239	16.5729	18.0884	16.3214
SCP2	22.9919	22.4194	22.6506	22.7449	22.6408	21.2531
SCPEP1	21.0736	20.3712	21.0166	21.1735	20.444	20.2627
SCRIB	19.6353	20.1087	20.3708	20.6008	20.0562	20.1921
SCRN1	22.5742	22.4631	20.8106	20.5633	23.1273	22.3196
SCYL1	19.9318	20.2655	19.515	19.0885	21.0468	19.4318
SDC1	16.7016	19.4353	18.5664	18.9071	20.239	20.8814
SDCBP	20.592	21.2783	19.3318	19.5415	19.2141	17.6269
SDF2	17.8384	19.5426	19.1148	19.5472	18.852	19.5384
SDF2L1	21.1015	21.3598	20.9929	21.2413	20.8267	19.8531
SDF4	16.9496	18.127	19.2224	19.1899	17.3348	18.13
SDHA	24.0072	23.4882	24.1355	24.1056	23.7981	23.1391

SDHB	22.6368	22.2154	22.4596	22.6175	22.3796	21.7512
SDHC	19.6971	19.6927	20.66	20.4662	20.1145	19.3175
SEC11A	21.4708	21.9581	22.4483	22.4658	22.16	21.6676
SEC13	22.1251	21.9831	21.6634	21.3125	22.459	21.4641
SEC22B	22.0752	23.4012	22.3559	22.8456	23.9016	23.5603
SEC23A	23.9805	23.6552	23.2421	22.6221	24.6751	22.4064
SEC23B	20.7888	20.6352	21.4372	21.2606	20.4989	22.4468
SEC23IP	21.8413	21.3362	21.5724	20.8665	22.1799	22.5049
SEC24A	20.9009	20.65	20.9726	20.3764	21.4654	20.7863
SEC24B	19.2745	19.0983	19.454	18.9643	19.2981	18.5498
SEC24C	22.2857	22.2461	22.0681	21.7247	22.9527	22.8808
SEC24D	21.397	21.1217	20.463	19.6934	22.2222	19.7199
SEC31A	24.1949	23.7524	23.0344	22.5748	24.627	23.1109
SEC61A1	21.0244	22.602	21.3886	21.6053	22.6521	20.0157
SEC61B	19.2016	20.5584	19.8172	20.1641	21.3618	20.0291
SEC62	20.4751	20.9027	20.1567	20.5397	21.1257	20.8787
SEC63	21.9126	21.7975	21.4336	21.299	22.3772	21.7231
SEH1L	20.4788	21.04	20.6236	20.713	20.9163	20.7427
SEL1L	20.1937	20.8297	20.0725	19.8837	20.4874	19.5363
SELO	15.2895	16.8562	14.7818	15.5522	16.7215	15.3672
SEMA3A	15.729	13.9843	14.965	13.8762	15.4889	18.0606
SEMA3C	17.7239	17.3017	20.0372	20.0316	17.5902	19.9459
SEMA7A	17.9038	21.9811	17.7731	17.1131	22.1167	19.9959
SENP3	20.138	20.105	20.3958	20.6244	20.1898	19.5399
SEPHS1	21.1719	20.7429	19.8141	19.6888	20.461	20.4891
SEPT10	20.2539	19.5336	20.5147	19.9383	20.6417	18.6636
SEPT11	22.6354	21.5039	20.9076	20.6956	22.1063	21.3045
SEPT2	25.2984	24.3722	24.156	23.97	24.8417	24.1473
SEPT7	24.6178	23.7836	23.4157	23.0708	24.1514	23.4691
SEPT8	21.8202	20.2056	21.1328	21.0798	20.3018	20.7806
SEPT9	24.3716	23.0811	23.3246	23.2252	23.3256	24.1649
SERBP1	21.2342	18.8245	20.7248	20.7028	20.3657	22.2248
SERINC1	18.2266	17.5051	17.0091	16.5999	18.2839	17.2325
SERPINA3	18.9088	19.4454	18.0462	18.9308	19.7557	19.5004
SERPINB2	20.0564	25.1056	19.933	19.5233	27.1968	20.8902
SERPINB5	17.1764	17.8186	16.9102	17.0035	17.6877	17.4221
SERPINB6	24.182	21.8015	21.866	21.5392	21.9637	22.1497
SERPINB8	19.2272	19.8147	17.045	16.6975	20.2655	16.5228
SERPINE1	18.07	21.1635	17.5782	17.5887	21.9566	20.0661
SERPINE2	19.6316	20.231	19.7102	19.9269	19.4401	19.9653
SERPINH1	26.8481	28.2621	27.2454	27.5168	27.524	24.7005
SET	19.9741	19.8455	20.8931	20.2233	20.326	20.6593
SETD3	19.759	19.2468	19.1405	18.604	19.6281	19.6498
SF1	20.7437	21.1452	21.0728	21.2591	20.7765	20.7696
SF3A1	24.4123	24.3057	24.5531	24.5365	24.4258	24.3
SF3A2	21.1565	21.364	20.9823	21.0182	20.9505	21.0689
SF3A3	24.6579	24.1256	23.8537	24.0433	24.0663	24.2823
SF3B1	23.0669	23.9947	24.2163	24.3625	24.185	24.2075
SF3B2	22.8465	22.7393	23.1128	23.2723	22.9409	22.9698
SF3B3	24.7969	25.2087	25.3221	25.2561	25.3164	25.4458
SF3B4	21.1118	20.7985	21.0703	21.11	20.8686	21.2171
SF3B6	21.3727	21.6708	21.4247	21.8425	21.5754	21.5549

SFPQ	25.091	25.7814	25.2995	25.6053	25.6418	25.2374
SFXN1	22.9817	23.3936	23.8536	24.2404	23.6488	23.6973
SFXN3	21.671	22.2609	21.4175	21.2919	22.505	21.0736
SGF29	15.5785	15.5885	16.2884	16.3293	17.0488	14.2813
SGPL1	17.6526	16.007	16.7318	16.3109	17.5147	16.5461
SGSH	20.8253	21.1552	20.8678	21.1245	21.5716	20.9705
SGTA	19.4074	18.3102	18.9793	18.8908	18.2644	20.2705
SH3BGRL3	20.1044	20.0492	18.819	19.2492	20.2651	19.3739
SH3BP4	19.0902	20.2412	19.8418	19.984	20.2558	20.1888
SH3GLB1	20.3279	19.7883	19.5541	19.0825	20.0295	19.7835
SH3KBP1	18.9317	17.0038	17.9058	17.5244	17.5419	17.2801
SH3PXD2B	19.1265	19.3821	16.6521	16.6001	19.6243	19.5016
SHC1	18.2103	16.5833	18.3003	18.2404	15.9852	18.5149
SHCBP1	18.52	16.426	18.0918	17.4505	17.1037	18.1646
SHMT1	21.6414	18.7787	19.5499	19.347	19.4069	21.6093
SHMT2	25.3364	25.4029	25.9061	26.0638	25.5474	25.4285
SHTN1	14.215	13.9795	15.1416	14.0723	12.7692	18.9478
SIGLEC10	17.2139	15.6718	15.2915	15.0729	16.459	17.4257
SIGMAR1	18.8943	20.2265	20.2117	20.2274	20.1776	18.8018
SIL1	18.6737	20.2346	20.2528	20.178	19.9761	17.959
SIN3A	17.3931	18.1466	19.0944	19.1815	17.4107	17.917
SIPA1L1	19.3069	17.8404	17.9804	18.0215	18.1228	17.4006
SIRT3	17.0053	16.9329	17.4354	18.3003	16.2507	16.337
SKIV2L	19.7006	18.5315	19.3172	19.3301	19.3129	19.357
SKIV2L2	22.2181	22.9084	23.3777	23.2745	22.9552	23.2151
SKP1	20.7073	20.8509	21.112	20.8697	20.7152	21.1013
SLC12A2	19.396	18.3622	18.5115	18.3618	19.2942	18.9901
SLC12A4	17.2498	17.6342	16.9042	16.9552	18.0264	16.548
SLC12A7	21.0814	14.7918	17.1133	17.1932	15.4138	15.11
SLC12A9	18.3365	17.8842	16.7691	17.2009	17.4594	16.6706
SLC14A1	18.0663	11.9382	12.4918	10.5409	NA	7.03647
SLC16A1	20.5214	21.5401	20.9475	20.9675	21.2358	21.7381
SLC16A3	20.9507	21.445	20.9244	21.0995	21.4653	21.8332
SLC1A3	17.1581	17.1017	16.0305	16.1819	15.1397	16.8555
SLC1A4	19.8814	17.4375	18.2686	17.846	17.504	18.3294
SLC1A5	23.3552	22.8711	23.5004	23.4264	22.8937	23.2132
SLC25A1	19.9944	21.0043	20.6301	21.3245	20.9693	20.4835
SLC25A10	19.8658	19.8487	21.4436	21.8721	19.9844	21.9118
SLC25A11	22.462	22.7463	23.1215	23.0526	22.5662	22.7
SLC25A12	22.2356	22.6669	22.0374	22.2125	22.987	22.1949
SLC25A13	22.5972	23.4166	23.4756	23.73	23.3968	23.2879
SLC25A17	15.0385	14.3771	17.2018	16.8349	14.4714	16.0298
SLC25A22	17.5383	19.1321	20.0541	20.5806	18.1758	19.188
SLC25A24	21.0999	22.0272	22.5174	22.5139	22.3783	20.1016
SLC25A3	22.978	24.0233	23.5432	24.0604	24.526	23.3722
SLC25A32	15.0005	16.7971	18.2802	18.1688	16.4596	17.0328
SLC25A4	19.061	17.6252	18.793	18.737	17.5789	19.9195
SLC25A5	24.6856	24.8389	25.5787	25.7072	25.3623	26.0332
SLC25A6	23.4399	22.6485	23.7432	24.0623	22.9765	23.1299
SLC26A2	19.4432	18.4347	18.6659	18.6293	18.6468	18.8728
SLC2A1	22.4777	22.542	23.8505	23.5047	22.3431	23.581
SLC30A1	19.8665	19.3837	18.296	17.0241	20.2726	18.8321

SLC30A7	21.4039	21.6985	21.8071	21.5023	22.5545	21.2804
SLC35B2	20.2269	20.6702	19.9597	19.9811	20.8252	20.7381
SLC35F6	18.1366	18.1183	18.1374	18.0714	18.0915	16.5379
SLC38A2	21.8913	21.6916	22.041	21.7205	21.94	21.5104
SLC39A14	19.4489	20.7263	19.7265	19.4472	21.8548	19.2642
SLC39A7	18.4819	19.7359	19.63	19.7718	20.0815	17.8101
SLC3A2	26.0837	23.3797	25.9076	25.6383	24.1245	25.6567
SLC44A1	18.6089	19.0327	17.4427	18.523	17.6534	18.2875
SLC4A7	17.6784	18.1391	19.1915	18.8895	17.9954	19.6331
SLC5A3	20.3171	19.1986	19.9367	20.4655	18.5296	18.2876
SLC6A6	17.5881	18.2013	19.1284	18.6523	18.4861	18.7792
SLC7A11	19.3773	16.8274	18.6318	18.3444	17.4362	18.1232
SLC7A5	21.005	16.8039	20.7923	20.4735	17.1732	21.2806
SLC9A3R1	12.3728	18.6719	17.5414	17.264	19.4187	19.2002
SLC9A3R2	19.9433	19.0637	20.0581	19.8602	19.6715	21.2894
SLFN5	18.1126	20.8316	20.9289	20.3136	21.612	19.6047
SLIRP	21.1601	21.8005	21.7183	22.1148	21.8514	21.3767
SLK	18.0826	17.7759	17.1498	17.2274	18.1829	17.6843
SMAD3	18.0151	17.5829	19.3314	19.3217	18.5677	18.5519
SMARCA1	19.9571	19.3492	20.8302	20.7382	19.7513	21.5657
SMARCA4	14.9908	15.7961	17.1522	16.1339	15.8533	17.3198
SMARCA5	21.0023	21.7661	21.9154	21.5213	22.1399	21.8745
SMARCB1	20.2942	20.3634	20.7024	20.7829	20.3125	20.7896
SMARCC1	20.5687	21.4269	21.0324	21.3956	21.0636	21.6966
SMARCC2	21.3276	21.202	21.4705	21.7343	21.3321	20.3103
SMARCD1	17.7184	18.9525	18.4228	18.8435	18.9797	19.2339
SMARCE1	20.3179	20.5789	20.5919	20.9974	20.4982	20.8484
SMC1A	22.3527	22.1994	22.3192	22.5034	22.3253	22.1013
SMC2	23.5725	23.3186	23.0287	22.4868	23.7042	23.1563
SMC3	22.9234	22.3071	22.9546	22.9792	22.6318	22.5161
SMC4	22.3514	21.2958	21.9792	21.7326	21.9025	21.7819
SMC6	15.6315	16.181	17.184	17.1455	16.0519	15.5838
SMCHD1	21.9177	21.8153	21.7093	21.7604	21.9475	21.3849
SMPD4	19.263	19.7187	20.0046	19.7165	20.1257	17.9492
SMS	21.6217	19.348	20.69	19.7733	19.3672	20.2904
SMU1	22.8267	23.1944	23.3547	23.4992	22.8443	23.6444
SND1	26.129	26.2358	26.3471	26.2523	26.6245	26.002
SNRNP200	24.5936	25.1789	25.3663	25.5556	24.9827	24.767
SNRNP40	20.9431	21.364	21.8856	21.9228	21.1335	21.4364
SNRNP70	23.1343	23.4285	23.2593	23.512	23.6225	23.9062
SNRPA	21.1711	19.5865	21.5676	21.8276	18.7607	21.1166
SNRPA1	22.0319	22.3979	22.4966	22.5793	22.464	22.501
SNRPB	23.8784	23.9018	24.0648	24.1892	23.9066	24.0295
SNRPB2	19.9915	20.076	20.7257	20.831	19.8639	20.0775
SNRPD1	21.7337	23.1669	22.6499	22.8073	22.9578	22.6189
SNRPD2	23.4355	23.9301	23.651	23.7622	23.8643	23.7177
SNRPD3	22.8384	23.8667	23.3895	23.7019	23.6975	23.4709
SNRPE	21.1467	21.7309	22.0861	22.683	21.2673	21.2432
SNTB2	20.8552	20.0011	19.7926	19.9928	20.1104	20.3388
SNU13	21.1032	21.9257	21.353	21.9478	21.8444	21.4671
SNW1	18.3534	16.9206	18.5982	18.8722	16.4292	17.6874
SNX1	20.5106	19.1538	20.133	19.6409	19.8174	20.4514

SNX12	19.5445	18.0411	18.466	18.1524	18.8272	19.0399
SNX17	19.6424	19.2611	17.7386	18.4641	19.4978	18.9077
SNX18	21.2652	19.9218	20.5265	20.5123	19.8787	20.702
SNX2	21.9582	21.2228	20.6102	20.1653	21.5602	21.9211
SNX27	20.4084	20.4449	19.7277	19.4387	21.0155	20.0599
SNX3	21.4921	21.0357	20.4756	20.8436	21.1391	21.967
SNX4	19.318	18.6231	17.7499	17.95	18.4974	18.8599
SNX5	20.8489	19.4662	20.3809	19.9928	20.1601	20.534
SNX6	20.3839	19.3385	18.0549	17.8382	19.5783	19.7972
SNX8	18.391	18.6679	18.8992	19.1613	18.3226	18.4407
SNX9	21.9127	21.023	20.5314	20.0909	21.7143	20.5273
SOAT1	20.3113	21.5181	21.1942	21.2782	21.4425	20.1533
SOD1	21.5573	19.9051	21.076	20.8511	20.2961	20.7786
SOD2	23.9912	27.9486	24.0124	23.4705	28.0084	22.9335
SON	21.4196	21.9953	21.7633	22.5508	21.7505	21.0867
SORD	19.1579	17.1313	19.7173	19.7325	16.6783	20.0502
SORT1	18.9872	19.3843	19.0386	18.8147	19.655	20.2649
SOX10	14.6754	15.1058	13.5197	13.9546	16.1218	13.8271
SP100	18.0666	20.033	18.9346	18.7179	19.8474	17.8902
SPAG9	21.3904	20.4493	20.6681	20.5461	21.2861	20.414
SPANXB1	16.5466	17.9551	16.6319	16.668	18.1339	16.5986
SPARC	22.0397	23.0447	22.8051	22.8943	22.2015	22.8812
SPATA5	19.382	19.0275	18.8019	18.9389	19.2939	19.7151
SPATA5L1	17.1885	16.4236	16.754	16.4265	17.3829	18.5922
SPC25	18.2454	17.0756	16.9764	17.0122	17.4645	18.0169
SPCS1	17.0948	17.8455	18.6087	18.9886	18.2119	18.3434
SPCS2	21.2171	21.2341	21.5161	21.8071	21.2809	21.0151
SPCS3	20.6607	21.8338	21.5737	21.814	21.8803	21.1322
SPECC1	17.502	14.7941	18.0185	18.0387	15.1506	17.9601
SPECC1L	20.0311	18.3708	19.3417	19.7388	18.1544	18.7717
SPG20	18.5439	17.7803	17.9344	17.1219	18.622	18.9938
SPINT1	10.9518	8.46163	7.81832	5.16006	10.0899	12.3916
SPNS1	18.474	19.2154	18.3795	18.8504	18.1319	17.635
SPPL2A	19.5452	19.7488	17.8618	17.6785	19.7321	18.2972
SPR	17.6396	17.5852	19.1744	19.5752	17.0334	18.8274
SPRYD4	18.9308	18.8968	18.6015	19.1578	18.3522	18.048
SPRYD7	18.8068	18.7145	19.03	18.7206	18.7656	17.9356
SPTAN1	24.3048	25.4863	24.9071	25.0684	25.6543	24.6418
SPTBN1	23.952	25.387	24.8034	25.0155	25.4987	24.615
SPTLC1	21.286	21.0594	21.0226	21.2529	20.9368	20.2433
SPTLC2	16.5924	19.482	18.0483	18.0827	18.7979	18.219
SQLE	17.7686	16.0432	17.1254	17.1913	16.8235	15.038
SQRDL	24.0037	23.0759	23.633	23.2705	24.1049	21.5971
SQSTM1	25.736	24.5902	25.6864	25.5037	25.2949	24.9824
SRI	20.4886	19.9387	20.9741	20.891	20.1833	20.5752
SRM	22.3415	21.4035	21.2194	20.9833	21.9126	22.7822
SRP14	21.4746	21.5279	21.0373	21.6405	21.4441	21.3505
SRP54	22.0577	22.0928	21.7825	21.305	22.2732	22.6523
SRP68	22.7494	22.5085	22.1912	22.0101	22.8846	23.0956
SRP72	22.482	22.2795	22.2869	22.175	22.4844	23.1427
SRP9	21.2453	21.3531	21.5968	21.701	21.4198	21.6852
SRPRA	19.7115	22.5635	21.2254	21.2647	22.9612	21.753

SRPRB	23.3621	24.0816	23.6874	23.6297	24.3948	23.3923
SRR	17.1296	15.738	17.1357	16.894	13.4338	15.008
SRRM1	20.8259	21.6799	21.6526	21.8822	21.6224	21.6592
SRRM2	21.7183	21.1143	21.8453	22.2963	21.4501	21.4142
SRRT	22.7115	22.7935	22.8543	22.8712	22.7374	22.5355
SRSF1	24.9625	25.0967	25.4341	25.5435	25.2959	25.1006
SRSF10	20.8197	20.7987	20.7444	20.9955	21.0797	21.0667
SRSF11	20.2488	20.0704	20.577	20.712	20.0962	19.8636
SRSF2	21.0768	21.2955	20.8562	21.0847	21.569	21.3791
SRSF3	23.716	24.5421	24.0557	24.1216	24.4734	23.566
SRSF5	21.8801	22.0037	22.1018	22.0755	22.3257	21.9427
SRSF6	21.9755	22.6614	22.2026	22.652	22.8999	21.9518
SRSF7	23.2075	23.4808	23.3874	23.5432	23.6801	23.4117
SRSF9	22.9118	22.9837	23.5492	23.2948	23.0714	23.3104
SSB	24.0597	24.1523	23.8334	23.6352	23.6531	24.3279
SSBP1	22.2698	22.9907	22.9183	23.1064	23.3292	22.9898
SSFA2	15.3323	15.9269	16.8697	16.7964	16.1748	14.5005
SSR1	21.7176	22.6473	22.6666	23.0087	22.5384	22.3503
SSR3	20.1754	21.1642	20.8489	21.3344	21.3525	20.5764
SSR4	23.7108	24.472	24.5203	24.7541	24.4243	24.0207
SSRP1	24.1492	24.5596	24.3632	24.2129	24.7351	24.4885
ST13P4	23.3593	22.3722	23.0624	22.8415	22.4632	23.1299
ST3GAL1	14.1099	16.2656	17.9326	18.0427	18.3938	14.0161
STAG1	18.0229	18.0224	18.0204	17.7573	18.4408	17.9093
STAG2	19.2311	19.1187	20.0008	19.8289	19.2748	19.1659
STARD9	17.1069	16.5499	16.7222	15.3029	17.1246	16.0766
STAT1	24.4334	23.8905	23.0589	22.8225	23.5839	23.9463
STAT2	15.6824	16.9894	15.6537	15.1123	15.0685	13.9721
STAT3	22.212	21.476	21.7432	21.6314	21.4827	22.895
STAU1	20.9781	19.9611	21.5868	21.6584	20.5189	21.8394
STEAP2	14.5951	17.0063	14.5749	14.4519	18.1745	14.1015
STEAP3	17.5744	21.1462	18.7401	18.6563	21.146	17.7336
STIM1	19.5646	18.177	19.3827	19.4437	18.4242	18.1515
STIP1	24.6827	24.0908	24.656	24.52	24.4725	25.1242
STK24	18.7756	18.0762	18.4098	18.6756	16.4491	17.6118
STMN1	22.5393	22.2227	21.2656	20.9587	22.5048	22.5114
STOM	22.4708	20.8936	19.7616	20.2504	20.787	19.696
STOML2	22.5539	22.5562	22.5237	22.796	22.362	23.5853
STRA6	15.4628	14.9809	20.7629	20.57	15.8635	16.6251
STRAP	23.9324	23.6139	23.8001	23.7886	23.8252	24.7195
STRN	19.6641	19.577	18.6611	18.4922	19.9698	19.8616
STT3A	22.2119	23.3987	23.2149	23.3423	23.6871	21.8995
STT3B	20.565	21.8804	21.4592	21.4299	22.1548	21.1594
STX12	20.7878	21.1315	20.2789	20.1207	21.5773	20.7523
STX16	16.7319	17.1045	17.3257	17.665	16.76	17.734
STX18	17.8255	17.6445	17.3521	17.0979	16.8147	15.3092
STX6	19.2363	19.1316	19.6298	18.6471	18.856	19.9513
STX7	19.1825	20.2617	19.1764	19.7424	20.3082	21.1115
STXBP1	20.3074	20.2765	19.7744	19.3904	20.8869	18.5907
STXBP2	16.5112	15.8149	15.766	15.893	16.2559	19.157
STXBP3	20.6587	20.3151	20.4167	20.1619	20.699	19.7607
SUB1	21.2123	21.1063	21.2027	21.0648	21.7905	22.1249

SUCLA2	22.4761	21.6765	22.4457	22.496	21.9487	21.9556
SUCLG1	23.0539	23.0702	22.8904	22.9741	23.3247	22.7507
SUCLG2	22.3176	22.5298	21.7666	21.9419	22.8326	21.8948
SUDS3	16.7303	16.8998	17.0429	17.1603	16.8337	16.3497
SUGP1	15.8827	15.5579	17.0609	16.6274	16.5186	17.0159
SUGP2	16.4558	15.7314	17.6775	17.6247	16.1774	17.6182
SUGT1	21.8795	21.2439	21.596	21.1335	21.7795	22.0807
SUMF2	20.863	22.3242	20.7724	21.149	21.2303	20.573
SUMO1	20.0727	20.6067	20.4899	20.6149	20.8983	21.2574
SUN1	17.6719	17.038	17.9066	17.631	17.592	16.6602
SUN2	22.2922	22.6558	22.2545	22.3776	22.1771	21.9003
SUPT16H	24.0608	24.3477	23.9093	23.7142	24.573	24.3457
SUPT5H	20.934	21.1738	21.0683	21.0155	21.108	20.613
SUPT6H	19.6064	20.1509	20.1671	20.1352	19.5258	18.8595
SUPV3L1	18.8958	18.985	19.1086	19.0413	19.7141	19.723
SURF1	16.9419	16.1837	18.2313	18.2811	14.443	16.8029
SURF4	21.9884	22.244	22.3199	21.8496	21.2245	20.6139
SUSD2	14.2351	14.2489	20.8173	20.1871	14.8097	14.9508
SVIL	15.4066	17.5342	15.0311	14.6742	17.0831	15.3533
SWAP70	20.0496	19.0452	18.872	18.8226	19.7051	19.5587
SYMPK	18.4731	19.623	19.9927	20.024	19.1108	19.5759
SYNCRIP	25.4914	25.4134	25.1449	25.0866	25.4423	26.0465
SYNJ2BP	19.7929	19.7455	19.5715	19.5266	20.0957	19.0982
SYNM	24.7007	23.3011	23.5355	22.7406	22.8602	24.2093
SYNPO	21.4626	22.4459	20.8745	20.9354	22.8372	20.3728
SYPL1	23.4841	23.393	22.942	22.71	23.5994	22.4887
TAB1	18.2737	17.1449	16.9408	16.8085	17.6035	17.4628
TACC1	20.1865	18.353	19.9944	19.306	18.8677	18.8163
TACO1	20.9304	20.9569	21.3259	21.5016	21.0933	21.3718
TAF15	21.1235	22.087	20.3401	21.3733	21.9312	21.3118
TAF6	17.166	17.6348	18.3711	18.8313	16.7218	17.7416
TAGLN	19.1951	19.3609	18.5209	17.9853	19.305	18.8274
TAGLN2	25.3891	24.5019	24.5156	24.3801	24.5632	25.4487
TALDO1	26.1435	24.2931	25.6807	25.462	24.8316	24.8755
TAMM41	16.2159	17.7857	16.7542	17.7271	17.3445	18.5847
TAP1	20.0048	19.2408	19.8472	20.1628	19.1014	18.1633
TAP2	19.9651	18.7835	19.3643	19.489	18.9845	18.0936
TAPBP	20.4973	20.462	20.11	20.8724	20.3108	19.401
TAPBPL	16.9667	17.7143	17.5326	17.1709	18.0391	14.9814
TARDBP	22.8268	23.1731	23.1333	23.308	22.5335	22.875
TARS	23.8696	22.7729	23.3198	22.8255	23.5171	24.6329
TARS2	20.331	20.2954	20.1812	20.3151	21.0924	21.1652
TAX1BP1	17.6425	16.0647	16.3338	15.3442	17.2492	15.3225
TBC1D13	18.197	18.2985	17.4858	18.102	18.2479	19.247
TBC1D15	19.0237	16.7496	17.0334	16.1865	18.2222	18.0132
TBC1D17	18.5181	17.8879	19.1653	18.971	18.5524	19.1155
TBC1D23	18.553	19.3001	18.6184	18.5411	19.3704	17.9112
TBC1D9B	17.8032	17.9868	16.8213	16.2722	17.8112	17.3781
TBCB	21.6922	20.655	19.6486	19.6993	21.2631	21.8043
TBCD	19.3218	19.1421	19.9155	19.4938	19.7561	20.4507
TBCE	19.139	19.1893	19.2361	18.6456	18.4949	19.0551
TBK1	18.0677	17.0714	17.6512	17.3126	18.0994	18.7265

TBL1XR1	22.4043	22.1476	22.6035	22.6461	22.3357	22.4335
TBL2	20.8724	21.4074	21.3475	21.5633	21.6682	21.3059
TBL3	22.0437	22.7831	23.4223	23.2803	22.7997	23.3725
TBRG4	19.4213	20.4542	21.2606	21.4682	19.7722	20.5757
TBXAS1	16.1769	15.0854	17.6938	17.7103	14.1053	13.2544
TCAF1	17.7201	18.7445	19.3019	18.9058	18.8307	18.4809
TCEA1	21.4611	20.5108	21.0755	20.8749	21.1403	21.1455
TCEB1	20.9448	20.3256	20.4062	20.5084	20.665	20.8425
TCEB2	21.3377	20.8811	20.81	20.6641	21.225	21.1588
TCERG1	21.5532	21.9924	21.9229	22.0542	22.1556	21.4862
TCF25	18.0176	18.3091	18.1364	19.0087	16.8186	17.0132
TCIRG1	17.8141	18.4705	18.1548	17.7976	18.046	14.199
TCOF1	18.9017	17.2807	19.5877	19.8829	17.6525	19.568
TCP1	25.8888	25.4323	25.6635	25.6519	25.3343	26.3348
TCP11L1	16.0992	14.7754	14.5527	14.3435	17.1415	14.5309
TCTN3	13.9133	11.7753	15.2057	14.7632	8.29861	12.6451
TECR	21.8058	21.989	22.2114	22.2673	21.8477	22.4903
TELO2	14.0901	15.178	16.9333	16.1787	15.0665	16.3373
TERF2	14.9672	16.0062	15.6896	15.4622	16.991	15.5373
TEX10	12.5406	17.8047	17.0518	16.9449	17.7889	17.0341
TEX264	19.3279	19.347	18.6875	18.8852	19.57	18.8148
TF	23.4864	21.1333	23.0833	22.6902	20.558	22.67
TFB1M	18.1498	18.3644	19.1652	18.9618	18.5814	20.0077
TFCP2	19.5353	18.8167	18.8823	18.9428	19.2316	19.4991
TFG	21.1135	20.6994	20.7497	20.3769	21.2971	21.2929
TFIP11	15.9613	14.8954	16.7315	17.3018	14.8973	17.0987
TFRC	25.4947	25.7689	27.0681	26.8373	26.0083	27.1344
TGFB1	19.4404	17.5174	18.3328	18.538	16.8096	17.7026
TGFB1I1	18.2818	19.0852	16.5487	15.9624	20.2032	14.6608
TGFBI	21.6764	22.6866	22.5929	22.6851	21.2575	22.66
TGM2	20.4146	22.6634	19.9321	20.1008	23.0883	20.0751
THBS1	21.4238	22.2624	21.6616	21.5756	21.7216	20.5503
THBS2	20.1597	19.0109	19.7257	19.7162	19.9089	20.2364
THEM6	17.366	17.8452	19.3633	19.5417	18.1583	17.4663
THG1L	16.0942	16.9761	17.0315	17.0094	16.3655	17.7703
THOC1	19.6774	20.6687	20.7196	20.8357	20.9136	20.119
THOC2	20.2389	21.0739	21.2728	21.3494	20.9056	20.7937
THOC3	22.6474	19.1615	22.828	22.3039	19.4819	20.2986
THOC5	19.36	20.1422	19.9306	20.1241	20.0041	19.2301
THOC6	20.4802	20.9456	20.5907	20.9282	20.446	20.8224
THOP1	20.9807	20.4863	20.1066	19.9622	20.65	21.3024
THRAP3	21.8314	21.7145	21.9999	22.3209	22.1447	21.799
THUMPD1	19.2872	18.7643	18.112	17.8592	18.4814	18.3114
THUMPD3	16.0409	16.3336	16.0615	14.8112	14.2059	16.204
THY1	21.3595	23.3702	24.2212	24.3681	22.7452	22.4746
TIA1	19.5771	19.4434	18.9526	19.089	19.3971	19.1386
TIAL1	21.3416	21.999	21.2951	21.5611	21.7811	21.7343
TIGAR	17.6836	16.5896	16.2999	16.3252	16.2533	16.8878
TIMM17B	18.7933	18.5394	19.1977	19.3116	19.3115	19.6277
TIMM21	16.9295	17.8918	18.1944	18.2375	18.3344	18.3823
TIMM22	18.3434	18.1708	18.4251	18.3746	17.9786	17.9994
TIMM23	18.808	18.8836	19.6604	19.9537	18.6026	19.3665

TIMM44	22.0065	21.7657	22.2839	22.5367	21.8857	22.7413
TIMM50	22.1976	21.7162	22.4351	22.4768	21.7106	22.2355
TIMMDC1	13.1702	15.8702	17.4521	16.8605	14.3607	16.9053
TIMP3	20.322	17.3381	19.5918	19.2661	16.0453	16.6916
TIPRL	20.2073	19.4836	19.3742	19.0762	19.5798	20.278
TJP1	21.2621	20.7268	21.4032	21.2301	21.2871	19.68
TJP2	17.9337	17.9541	18.4293	17.8999	18.4755	14.7848
TK1	19.7546	17.0509	19.8392	19.6146	16.2695	19.3491
TKT	28.2375	26.4832	27.2023	27.048	26.4849	29.3771
TLN1	26.1029	26.0603	24.8398	24.9345	26.5289	26.0533
TM9SF2	21.8995	22.4731	22.1883	22.1922	22.4248	21.9435
TM9SF3	22.0068	22.5416	22.2625	22.1372	22.7984	22.0524
TM9SF4	21.5729	21.9669	21.7531	21.9435	21.7105	21.1825
TMCO1	20.4836	20.486	21.274	21.6762	20.5267	20.5856
TMED10	23.2838	23.3467	22.8795	23.1836	23.6558	22.8471
TMED2	20.8378	20.8827	20.0492	20.6999	21.0179	20.185
TMED4	18.9308	19.5443	19.132	19.184	19.725	19.0967
TMED7	22.6519	22.7924	22.7298	22.7325	23.0998	22.2035
TMED9	20.0797	21.613	19.9553	20.3525	21.808	20.0256
TMEM106B	16.9202	18.3166	17.2115	17.3946	17.8838	15.8648
TMEM109	21.7014	22.7924	22.8311	22.9063	22.8887	21.7378
TMEM11	19.645	19.9506	20.505	20.7104	20.11	20.1733
TMEM126A	17.8881	17.5158	18.7055	18.998	17.493	17.2938
TMEM132A	14.2257	18.7307	17.9712	17.6898	19.5807	14.6955
TMEM14C	18.6965	19.204	20.4908	20.1907	18.4611	17.7703
TMEM165	20.7091	22.2441	20.5569	20.327	22.8281	21.575
TMEM167A	21.0558	21.7343	21.5887	21.357	22.1061	20.5281
TMEM189	19.6356	20.6389	19.565	19.1499	20.5784	19.6345
TMEM192	18.5736	18.6024	18.785	18.5292	18.3928	17.6522
TMEM2	17.9923	18.9821	18.3485	18.5158	18.6691	20.0075
TMEM214	17.9925	20.4414	19.5912	19.3863	20.9821	20.5274
TMEM245	16.7252	16.7594	17.4987	17.4716	17.6931	15.8016
TMEM33	21.0006	20.8518	21.2439	20.9707	21.0543	21.5295
TMEM43	22.9915	23.5557	23.2698	23.1785	23.7325	21.2438
TMEM55B	18.6191	19.3403	17.3495	17.9921	19.1472	17.2611
TMEM56	13.7498	11.2237	12.4984	11.6137	15.1779	13.1691
TMEM87A	20.091	19.9482	19.7565	19.7661	19.9634	19.9659
TMEM97	19.0498	19.8524	19.8368	20.3443	20.3326	19.9621
TMF1	15.2886	16.0753	16.9319	16.8287	16.436	13.9312
TMLHE	17.0811	16.9114	16.4031	17.3903	16.5997	17.3539
TMOD3	21.9624	21.6072	21.0013	21.0034	21.7434	21.4018
TMPO	22.8233	21.442	22.5413	22.6599	21.6402	21.9922
TMPO	22.5492	22.3305	21.6322	21.7643	22.7812	21.4114
TMTC3	17.9947	18.6229	18.1085	17.649	18.0642	17.0736
TMUB1	17.9448	18.3776	18.1706	17.7391	18.5855	19.1942
TMX1	23.5402	23.5274	22.9305	23.2058	23.6454	23.715
TMX2	20.1825	20.0205	21.146	20.9952	19.5173	20.8031
TMX3	21.9509	21.3645	21.028	21.0068	21.8098	20.2629
TMX4	20.6305	20.1037	20.0157	20.0324	19.8922	19.1031
TNC	21.8162	24.4207	19.9042	19.9442	23.5283	21.8575
TNFAIP2	18.3833	19.9895	18.0107	17.6324	19.2485	18.7113
TNFAIP8	13.2557	17.4085	15.5311	15.4766	17.3112	14.3613

TNFRSF10B	17.9559	19.7733	19.4385	19.8999	18.801	17.7124
TNFRSF11B	13.8919	15.405	19.2875	19.4005	12.3178	11.9596
TNKS1BP1	19.5528	19.1884	18.8622	18.8746	19.4658	18.4396
TNPO1	23.8659	24.1832	24.218	24.0697	24.3489	24.5205
TNPO2	18.1903	16.4305	16.3457	16.1207	16.1381	18.3664
TNPO3	22.3475	20.9915	21.4552	20.9736	20.9317	21.7057
TNS3	23.5329	21.8635	22.7666	22.5574	21.9271	21.3493
TOE1	18.4836	18.5958	19.4718	19.9915	18.7126	19.1059
TOLLIP	20.8439	20.6406	20.1339	20.1947	20.3775	19.4638
TOM1	20.0768	20.613	18.8912	18.9821	20.6597	18.0727
TOMM22	19.8203	20.278	21.2687	21.5459	19.5033	19.5399
TOMM34	20.4797	20.6392	21.9498	21.8914	21.089	22.6192
TOMM40	22.9599	22.9938	23.8473	23.9795	22.8865	23.6311
TOMM5	18.1472	18.6934	18.2482	17.7547	18.7329	17.3587
TOMM7	19.6375	19.8907	19.8221	19.8401	20.5489	19.6286
TOMM70A	22.2744	22.2003	22.7542	22.8945	22.4353	22.4467
TOP1	18.5208	19.6911	19.1933	19.0296	19.9892	20.3235
TOP2A	20.1152	19.8207	20.8335	20.4456	20.2753	20.4265
TOP2B	15.9166	19.5244	16.9756	16.9072	19.3697	17.1501
TOR1A	18.3122	18.4185	18.3805	18.2863	17.8008	17.5083
TOR1AIP1	23.0388	22.7539	22.8554	22.7876	22.97	22.8728
TOR1AIP2	19.7233	19.9944	20.0609	20.0492	20.2244	20.3749
TOR1B	19.8682	18.9059	18.9828	19.1634	18.4318	19.1475
TOR3A	17.0957	15.8654	17.594	17.4037	15.6476	15.3663
TP53	16.1373	15.553	18.972	19.756	14.8195	18.5309
TP53BP1	23.2146	23.3456	22.7376	22.5617	23.9807	23.1136
TP53I11	20.6411	16.5694	17.7398	17.6773	17.2621	17.6607
TP53I3	19.1919	18.6941	15.8268	16.0941	19.4213	16.6483
TPD52	15.9538	9.8538	13.8737	13.645	13.6661	19.4694
TPD52L2	20.1319	20.8036	20.7817	20.9255	20.7497	21.7916
TPI1	28.1696	26.643	26.7956	26.6636	26.7344	27.7937
TPM1	18.7436	18.5015	18.4829	17.8169	18.6966	17.1492
TPM3	21.7123	21.067	21.3901	21.2088	21.6781	21.0297
TPM4	22.7445	22.0538	21.7559	21.0967	22.5113	20.1748
TPP1	22.2625	24.2169	21.61	22.2546	23.8109	19.8111
TPP2	23.0158	22.0437	22.7944	22.3884	22.6196	21.8941
TPR	23.351	23.52	23.7017	23.9321	23.7259	23.2056
TPRKB	18.885	18.0663	17.5044	17.5557	18.0044	18.0492
TPT1	21.5304	20.4628	20.4585	21.1774	21.1818	22.2882
TPX2	18.111	12.6571	18.5583	18.4746	14.1148	17.7331
TRA2A	21.7176	22.1832	22.132	22.0663	22.0274	21.1588
TRA2B	23.5085	23.9035	23.8369	24.1322	23.2447	23.3305
TRAP1	23.0324	22.8697	24.1151	24.4273	23.1691	23.2939
TRIM16	21.9879	20.6789	21.5039	21.0942	20.5573	21.9013
TRIM22	18.5017	19.1336	18.9023	18.7798	19.3008	14.358
TRIM24	14.986	13.8343	16.0502	15.1089	13.46	14.2556
TRIM25	22.4118	21.5892	21.8591	21.4182	21.8734	22.4028
TRIM27	16.1762	17.0116	17.7502	17.5962	17.4061	16.3916
TRIM28	24.9608	24.4248	24.8526	24.9949	24.3693	24.937
TRIM3	17.9747	17.6222	16.5218	15.9242	17.7118	16.1878
TRIM47	19.956	16.5508	17.3919	16.7673	16.0837	18.7232
TRIO	18.0656	19.1206	17.7859	17.5609	19.8247	18.2556

TRIOBP	20.4901	19.9011	19.1448	19.1443	20.0016	17.9917
TRIP10	18.5581	19.6625	18.8349	18.2557	19.5262	18.2831
TRIP11	18.1232	18.3059	17.6373	17.4652	18.2481	15.9706
TRIP12	19.0298	21.2653	20.5148	20.3504	21.5349	20.2532
TRIP13	20.9656	19.8082	20.1879	20.1987	20.3523	22.1846
TRIP4	18.2082	17.5317	17.5597	16.8183	18.2351	18.3516
TRIP6	22.285	20.8372	20.4798	20.2495	20.9712	22.428
TRMT1	15.9565	17.5179	16.139	15.4281	17.5204	17.9279
TRMT10C	21.4745	22.1039	21.9269	22.0674	22.343	22.5622
TRMT112	16.7677	16.716	17.9693	17.6696	16.1619	18.1822
TRMT1L	15.7567	17.2888	16.5503	16.6461	16.8497	16.3236
TRMT6	18.8192	18.5287	18.4448	18.6719	19.0106	19.1432
TRMT61A	18.135	18.5458	18.2081	18.0684	18.3232	19.1447
TRMU	14.9804	16.1658	16.8818	17.6493	16.1011	15.508
TRNT1	19.3026	20.2989	18.6604	18.9135	20.3871	20.0136
TROVE2	21.1983	20.7681	19.4895	19.5274	20.8643	21.2139
TRRAP	13.0431	13.8756	15.8594	16.1413	14.5111	14.6574
TSFM	20.6744	20.5054	21.3407	21.3707	21.0694	20.7814
TSG101	20.419	20.7458	19.7777	19.665	20.7927	20.817
TSN	21.1829	21.2012	20.8051	20.4764	21.5485	21.2812
TSPAN4	17.837	20.6419	20.6762	20.7475	19.205	16.5583
TSPYL1	12.4614	12.0208	13.9213	11.3773	12.7535	13.5357
TSR1	17.3341	18.0975	19.075	18.768	18.5663	19.9164
TSSC1	17.9486	16.6599	16.3887	15.9523	17.0638	18.4894
TST	20.8011	21.1919	22.2192	22.1911	21.4335	19.0744
TTC1	18.74	17.5408	19.054	18.5205	18.4226	19.4677
TTC37	16.9245	16.0377	17.4747	17.2532	16.717	16.9683
TTC4	18.8084	18.2791	18.9894	19.2019	18.7945	19.4839
TTI1	16.4108	16.4558	18.2903	18.0476	16.7675	17.5265
TTI2	15.1784	15.5052	16.2137	16.0272	14.1674	15.1738
TTLL12	22.5409	21.4162	22.3332	21.9833	21.2529	23.1544
TTN	21.0295	21.3474	20.0929	20.9471	21.8087	21.426
TTYH3	17.5335	21.9875	16.2063	18.3983	20.9292	15.9511
TUBA1C	22.8221	21.4457	21.5679	21.8923	21.7908	23.7836
TUBA3D	18.9471	19.8234	18.4517	18.1849	20.4043	19.1002
TUBA4A	22.5387	21.6256	22.6953	22.1598	21.9783	20.9116
TUBAL3	19.6521	16.0138	18.1707	19.0058	16.7511	20.38
TUBB	27.4319	26.2109	25.9919	25.7548	26.5965	27.5028
TUBB2A	21.1945	17.7411	16.2216	15.8586	18.3984	20.6984
TUBB2B	18.2087	16.8219	16.3737	16.1819	17.8649	18.966
TUBB3	22.168	20.8404	20.7829	20.7851	20.7384	21.6114
TUBB6	20.4904	23.3589	20.1061	19.9796	23.5155	21.7614
TUBG1	19.8101	19.2366	20.0168	19.7458	19.7095	20.1373
TUBGCP2	18.1047	17.3405	18.0361	17.1043	17.6369	18.4707
TUFM	25.8254	25.1213	25.6635	25.9971	25.1828	26.0669
TWF1	22.4445	21.8909	21.9005	21.7049	22.254	22.26
TWF2	22.3905	22.6974	22.2261	21.8565	23.3292	23.4706
TXLNA	22.1658	22.429	22.3295	21.748	22.5802	23.1912
TXN	23.9006	21.0726	22.977	22.6314	21.8412	22.5493
TXNDC12	19.222	19.2685	20.2756	20.4908	18.7938	20.0651
TXNDC17	20.9694	20.0086	20.4953	20.4931	20.3601	22.4899
TXNDC5	25.5212	25.3852	25.3201	25.6622	25.0304	25.6555

TXNDC9	18.5318	18.6015	19.0535	18.9703	18.8924	18.8614
TXNL1	23.6289	22.4195	22.3327	22.0232	23.0695	22.1305
TXNL4A	14.6257	16.3353	16.921	16.7138	14.0544	14.5163
TXNRD1	26.0397	23.889	23.7741	23.6086	24.5654	23.547
TXNRD2	18.2765	18.5596	17.2863	17.6348	18.0278	15.932
TYMS	20.0106	18.0664	19.5727	19.4041	18.0772	21.2127
U2AF1	21.6455	21.5274	20.9157	21.2993	21.7338	21.5989
U2AF2	24.4482	24.1826	24.371	24.3641	23.9558	24.6639
U2SURP	21.0487	21.7802	21.7652	21.6327	21.7847	21.6736
UACA	18.8309	17.7931	19.0094	18.8631	17.8985	17.3716
UAP1	20.8752	21.0896	20.051	19.9366	21.507	22.758
UAP1L1	20.4394	18.0658	17.3788	17.3264	16.9513	17.8271
UBA1	26.6273	25.3058	25.4007	25.1039	25.5492	25.426
UBA2	23.3442	22.5999	22.198	22.3932	22.6895	23.6748
UBA3	21.117	20.6275	20.4823	19.9524	20.8591	20.9671
UBA6	22.301	21.693	20.2195	19.9167	22.3773	22.2541
UBAP2L	21.2912	19.9019	20.9018	20.8609	20.292	21.6521
UBASH3B	14.9567	15.5768	13.9335	14.6517	14.1541	14.6102
UBE2C	18.0214	16.3767	18.2097	17.9062	16.1185	18.4699
UBE2D3	19.4961	19.7861	19.4754	19.5795	18.3695	18.7739
UBE2H	19.3955	20.0591	19.6899	19.4424	20.7221	20.5026
UBE2I	21.9074	22.01	21.9017	21.9145	22.2629	21.7916
UBE2K	22.7552	21.7387	21.1879	20.8525	22.4053	23.3914
UBE2L3	22.9806	22.1809	21.8922	21.725	22.2557	22.51
UBE2M	22.3152	21.6731	21.3006	21.0424	22.272	22.8918
UBE2N	23.5548	22.8098	22.7038	22.5143	23.1208	23.5035
UBE2O	20.6443	19.8919	20.7835	20.6911	20.4941	21.3415
UBE2S	19.3714	18.5566	19.3543	19.0111	19.2811	20.2019
UBE2V1	21.0735	20.0493	19.708	19.5236	20.9894	21.5137
UBE3A	19.3631	18.7551	18.7683	18.478	17.9269	19.066
UBE4A	20.3843	20.0685	20.2877	20.5401	20.1745	19.893
UBLCP1	21.131	19.9329	20.3061	20.0408	19.7143	20.5181
UBQLN4	19.6761	18.2681	19.6245	19.4384	19.3544	19.2005
UBR4	21.1959	20.5046	20.9757	20.6194	21.1865	20.8164
UBR5	16.2477	15.8308	18.1739	18.0389	16.7677	16.4111
UBTD1	15.3246	17.5522	17.0412	17.4584	16.3746	16.5432
UBTF	18.6509	19.8634	19.9799	19.8289	19.7693	19.8907
UBXN1	20.7742	18.6607	19.8556	19.347	19.3743	20.4122
UBXN4	21.3133	20.9717	20.8218	21.2064	21.1727	20.7172
UBXN6	18.5204	19.028	17.0387	17.2815	18.6817	18.6265
UBXN7	17.3988	17.3816	17.8836	17.8274	17.2365	18.3174
UCHL1	23.1907	22.3056	22.6811	22.7254	23.2329	25.0417
UCHL5	22.7216	21.8812	23.0537	22.5392	22.4035	23.0744
UCK2	19.3748	19.2744	19.2644	18.6341	19.2708	19.9429
UFD1L	21.5562	21.3656	21.5781	21.8821	21.356	22.2905
UFL1	21.35	21.7961	21.3831	21.413	22.0683	21.3943
UGDH	25.2938	24.3271	24.235	24.0498	24.5947	23.4753
UGGT1	24.2531	24.7708	24.6094	24.5307	24.7574	23.2961
UGP2	23.6627	23.7164	22.2908	21.7104	23.9266	23.1378
UHRF1	20.0747	19.5295	20.4075	19.8868	20.4693	19.3982
UMPS	21.4255	21.4124	21.4964	21.1741	21.7363	21.7691
UNC45A	21.7399	21.8254	21.1178	20.8093	22.0886	21.2388

UPF1	24.1539	23.7536	23.4865	23.4223	24.0705	24.0327
UPP1	20.7895	18.9289	18.3364	17.8078	19.5145	20.2216
UQCR10	20.1601	20.4605	20.9211	20.912	20.6533	20.8412
UQCRB	19.0244	19.0425	19.8467	19.9137	19.2825	18.5018
UQCRC1	24.6217	24.5705	24.9904	25.2626	24.4976	24.3176
UQCRC2	24.018	24.1414	24.6567	24.7968	24.356	23.6418
UQCRFS1	22.5951	22.4272	23.1987	23.4512	22.6569	22.7301
URB1	14.2872	15.8357	17.9972	17.6614	15.5097	15.2692
URB2	16.248	17.4498	18.442	18.4683	16.8887	17.5926
URGCP	13.5713	15.2964	13.587	14.2356	15.1402	14.1442
UROD	20.6657	20.6387	20.4335	20.4116	20.6773	20.4736
USMG5	21.0477	20.9126	21.6922	21.567	21.6222	21.6311
USO1	22.8678	22.9523	22.2076	21.8309	23.8934	22.8619
USP10	22.5035	22.0082	22.6919	22.5628	22.3756	21.9073
USP14	22.4728	21.3507	21.9934	21.3906	22.2505	23.0593
USP15	19.9774	18.1658	18.6669	17.5558	18.1803	18.6572
USP19	13.9512	14.8177	15.4266	14.427	11.4993	14.1378
USP24	20.37	20.0215	19.2251	19.2733	20.172	20.4356
USP33	18.0929	17.7179	18.2724	18.0855	18.0839	18.0117
USP39	18.555	20.3567	20.258	20.2495	19.6802	19.8627
USP47	19.8543	19.2601	19.4793	19.1226	18.5061	19.0085
USP5	23.822	22.8617	22.5945	22.3577	22.8624	23.1355
USP7	23.2067	22.9766	23.0714	22.918	22.9303	22.4617
USP9X	21.7325	20.4103	20.3136	20.252	20.9951	21.1697
UTP14A	14.837	16.4694	17.8587	17.6683	16.9634	18.3247
UTP15	17.385	18.1423	19.1457	18.9934	18.3569	19.6455
UTP18	17.3493	18.9972	19.707	19.716	19.2378	20.0339
UTP3	12.4632	16.641	17.0428	16.7312	14.1902	16.0492
UTP4	19.486	20.2434	21.3479	21.1023	20.4948	21.6404
UTP6	17.8001	18.1533	19.224	19.1495	18.2051	19.8176
UTRN	21.3066	21.5705	21.2234	21.1592	21.337	21.536
VAC14	19.9698	20.9435	19.6363	19.5994	20.7441	20.5078
VAMP3	21.822	21.9075	21.7369	21.7579	22.3563	21.5286
VAMP7	19.9337	20.3241	19.9522	19.8689	20.3279	20.0438
VAPA	23.0967	23.9697	23.3951	23.5472	24.1633	23.6503
VAPB	21.8616	22.4619	22.0984	22.6631	22.4883	21.9794
VARS	22.8132	23.0795	23.2603	23.0809	23.7993	24.7056
VASN	19.6782	19.694	19.9872	20.1435	18.3674	17.7823
VASP	21.5362	21.923	20.3635	20.3004	22.3693	21.6114
VAT1	22.8727	22.742	22.4005	22.5125	22.9005	22.2842
VAV2	15.6203	13.2891	14.6711	13.133	11.2026	14.9944
VBP1	21.0088	20.1279	20.8044	20.3482	20.4285	21.3281
VCAM1	17.6369	16.1696	17.9235	18.7206	14.03	14.3037
VCL	25.9858	24.9625	24.4161	24.0551	25.5203	26.0145
VCP	26.7527	26.8873	26.639	27.1522	26.3102	28.4412
VDAC1	26.8779	26.3808	27.2764	27.3747	26.4291	26.9158
VDAC2	26.2041	25.7276	26.2472	26.2587	25.6656	26.3187
VDAC3	24.5725	24.2885	24.8146	25.0573	24.2155	24.326
VIM	31.6631	32.0129	30.389	30.4929	32.0228	31.6202
VKORC1L1	19.6401	19.9482	20.025	20.3986	19.8821	19.1561
VLDLR	12.5533	11.9888	11.9149	12.7275	13.9385	18.5325
VPS11	16.8606	16.4183	16.887	16.1539	16.6698	15.9524

VPS16	17.991	17.9334	18.1096	17.9129	17.1969	16.9205
VPS18	19.2843	19.2242	18.5873	18.0199	19.5359	18.8396
VPS25	17.0562	15.7062	15.6092	15.8568	12.9037	15.4842
VPS26A	21.5803	20.8742	20.8966	20.6147	21.5575	21.619
VPS26B	19.7828	19.812	19.5534	19.9079	18.8535	20.1878
VPS29	21.1334	20.4505	19.9989	20.1319	21.0185	21.0092
VPS33A	18.4131	18.0925	17.9476	17.4991	17.9276	17.6483
VPS33B	17.4385	18.0362	17.7223	17.5033	18.5462	18.1032
VPS35	23.8447	23.1141	23.3779	23.035	23.4052	23.8417
VPS41	16.9098	17.0321	16.5909	16.4434	15.2749	16.2392
VPS45	20.0826	20.2827	20.392	20.4843	20.1631	20.7286
VPS4A	19.5275	18.3471	18.9673	19.0255	18.588	20.5617
VPS4B	18.3525	17.8809	16.5548	16.4768	17.2536	17.6434
VPS51	17.5708	17.2002	17.9885	17.3593	17.0246	17.8263
VTA1	20.4609	19.47	19.4303	19.4929	19.5824	21.7436
VTN	17.7759	16.6682	16.397	16.9677	17.2103	16.0557
VWA5A	20.4796	16.1583	16.3579	16.7302	16.639	15.4923
VWA8	17.3788	16.4816	17.4815	17.0924	15.7888	16.7221
WAC	14.1424	12.5802	16.7361	16.988	15.2233	15.8467
WARS	26.5203	24.4812	23.6771	23.4237	24.905	24.095
WBP11	20.0267	19.3645	20.3235	20.0488	19.8805	19.9999
WBSCR16	18.7359	19.5547	19.2126	19.2752	20.4205	19.3952
WDFY1	20.5473	21.1457	20.6967	20.3053	21.5965	20.6945
WDHD1	18.884	18.4356	17.9499	17.4154	18.5031	17.7307
WDR1	26.6582	25.9361	24.9717	24.7733	26.4397	26.4393
WDR11	19.4842	20.0017	20.2761	19.9842	20.4768	20.1959
WDR12	20.9561	21.152	21.2663	21.4077	20.9463	21.6975
WDR18	20.594	21.4324	20.5566	20.9958	21.8613	21.0836
WDR26	21.0485	21.557	20.3899	20.3148	21.7759	20.5585
WDR3	16.7654	19.1682	20.5701	20.2169	19.1418	20.9616
WDR33	17.4417	17.3046	17.2122	17.7514	17.7409	18.1029
WDR36	19.9421	20.409	21.5184	21.5359	21.0149	21.6298
WDR43	19.0674	21.0337	21.3789	21.2099	21.3226	21.5501
WDR45B	18.7164	18.4057	18.3918	18.1398	18.6012	19.5467
WDR5	20.6897	21.1824	21.3326	21.4458	20.7218	20.703
WDR55	17.3283	19.1409	18.3648	18.1045	19.3812	17.7701
WDR6	16.6023	18.2041	17.3583	16.8573	18.9448	19.0374
WDR61	21.2869	20.7151	21.547	21.3839	20.274	22.4197
WDR74	16.6842	17.9579	19.004	19.1081	17.6482	19.2383
WDR75	21.5937	21.1632	21.4964	21.1635	21.4948	21.9926
WDR77	22.726	21.8741	21.8821	21.1161	22.1892	22.7408
WDR82	19.9231	20.3453	19.9576	20.141	20.2545	19.8517
WDR92	18.3948	18.1349	17.6363	17.8132	18.1527	18.9791
WFS1	16.8671	18.0562	18.4406	18.6222	18.9272	16.6182
WRNIP1	17.5741	17.3004	16.9599	17.3512	14.8687	16.4367
WTAP	16.8149	17.4609	18.032	18.3268	17.8422	17.1529
WWP2	13.461	12.5591	14.9886	14.9956	12.3556	15.6524
XAB2	18.6487	19.5838	19.7012	19.737	18.9381	18.8584
XPNPEP1	22.4148	20.3944	20.6802	20.2524	21.0539	21.3693
XPNPEP3	19.1709	19.0313	20.6757	20.4137	19.4997	18.8468
XPO1	24.1825	23.8953	24.4861	24.2181	24.1314	24.5959
XPO4	16.9349	16.0544	17.821	17.2542	17.2383	17.4184

XPO5	20.5596	19.9976	20.658	20.5038	20.1309	21.6231
XPO6	15.6568	14.8932	16.2761	15.2244	15.5565	13.4894
XPO7	19.1067	17.8017	18.9399	18.578	18.939	19.7384
XPOT	21.126	20.8915	20.3772	19.799	21.2989	21.4432
XRCC1	18.8848	18.6228	18.7333	18.6938	19.1497	19.0926
XRCC5	27.0182	26.6299	26.242	26.2453	26.6298	26.3194
XRCC6	26.1425	26.2326	26.0075	25.8795	26.203	26.1724
XRN2	22.3687	23.1201	23.752	23.8497	23.2699	23.1308
YARS	23.8137	22.8404	22.9628	22.5682	23.8187	24.3444
YARS2	19.2879	20.4009	19.9447	20.4503	20.6563	20.281
YBX1	21.5962	21.1902	21.7083	21.4697	22.007	22.3841
YBX3	18.0059	18.1983	18.4555	17.9156	18.9252	18.5698
YEATS2	14.2491	13.269	15.9588	16.256	10.6408	13.8591
YES1	18.2146	19.1578	18.4639	18.804	19.2403	19.5422
YIPF5	17.8305	19.0305	17.4716	18.0748	19.428	17.0053
YKT6	19.4527	19.4823	18.8263	18.3999	19.9248	19.9435
YLPM1	19.7616	20.026	19.3916	19.686	19.9757	19.1841
YME1L1	20.099	20.3861	20.9892	21.2863	20.5637	21.5764
YRDC	14.9999	14.7454	15.4047	14.419	15.4788	17.8759
YTHDC1	18.2586	18.7745	18.1088	18.2899	18.8887	18.4578
YTHDF2	18.8256	18.6495	19.3779	18.8512	18.6921	19.6749
YTHDF3	19.0286	13.0211	18.4656	18.4527	15.587	19.1577
YWHAB	23.7147	23.1816	23.7526	23.6507	23.2474	24.1457
YWHAE	25.7231	24.8449	24.7811	24.7153	25.1164	25.8372
YWHAG	24.0328	23.0176	23.4576	23.344	23.4789	23.9536
YWHAH	22.7787	21.8355	21.3489	21.3431	22.1588	22.193
YWHAQ	25.4088	23.9107	24.0537	23.8086	24.1217	25.3302
YWHAZ	26.8087	25.1551	25.6827	25.498	25.6124	26.2414
ZC3H11A	16.5338	16.4295	18.7534	18.3901	17.3356	18.2546
ZC3H14	18.7817	16.9627	18.6832	18.6546	16.9459	18.1344
ZC3H15	20.2271	20.1074	19.6014	18.9665	20.3755	21.0764
ZC3H4	15.5094	9.51791	16.0514	16.3307	9.90282	14.3205
ZC3HAV1	22.5335	20.699	21.7958	21.889	21.1947	22.2448
ZFPL1	20.2039	20.4012	19.709	19.9526	20.5379	19.1478
ZFR	19.5604	20.1207	20.3027	20.1028	20.4721	20.2909
ZGPAT	19.7141	19.6637	20.9446	20.7717	20.0942	20.0216
ZMPSTE24	21.5094	20.907	21.6473	21.3909	21.2922	20.6567
ZMYND8	18.583	18.8756	19.8762	19.9242	18.8446	19.1718
ZNF207	21.8203	21.4715	21.6984	21.6977	21.7469	22.2116
ZNF326	20.76	20.2468	20.4867	21.0133	20.0846	20.4435
ZNF622	15.4722	16.8535	17.9776	17.7846	16.6099	18.0754
ZNF638	15.4214	17.2573	16.8537	17.0788	18.211	16.9941
ZNHIT6	11.4697	-1.72406	NA	NA	NA	12.1054
ZPR1	17.83	17.0802	16.5132	16.7368	17.771	18.7713
ZRANB2	19.0929	19.3944	20.456	20.5533	19.9624	20.26
ZW10	18.3998	18.7413	19.0714	18.4615	19.114	18.9049
ZWILCH	16.7331	13.4278	16.0493	14.7844	14.481	16.0092
ZYX	22.9618	23.2666	21.7662	21.9564	23.8985	23.042
