# Supplementary material for: The long non-coding RNA HOTAIRM1 promotes tumor aggressiveness and radiotherapy resistance in glioblastoma
Source: Cell Death Dis. 2021 Sep 28;12(10):885. doi: 10.1038/s41419-021-04146-0 (PMC8478910; doi:10.1038/s41419-021-04146-0)
Supplement: Supplementary file 15 — Supplementary Tables 4-8, 10-12 [file 41419_2021_4146_MOESM15_ESM.pdf]

Supplementary Table 4

GSEA based on RNA sequencing upon *HOTAIRM1* siRNA-mediated knockdown in glioblastoma cells. Positively enriched genesets in the *HOTAIRM1* knockdown cells are listed according to their normalized expression score values. NES: normalized expression score; NOM: nominal; FDR: false discovery rate.

GeneSet details	NES	NOM *p*-value	FDR *q*-value
CADHERIN SIGNALING PATHWAY_PANTHER PATHWAY_P00012	2.11899	<0.001	0.16766
LIMB DEVELOPMENT_GOBP_GO:0060173	2.01167	0.00238	0.30982
APPENDAGE DEVELOPMENT_GOBP_GO:0048736	1.99275	<0.001	0.25373
APPENDAGE MORPHOGENESIS_GOBP_GO:0035107	1.93417	<0.001	0.34565
HISTONE-LYSINE N-METHYLTRANSFERASE ACTIVITY_GOMF_GO:0018024	1.92291	<0.001	0.30706

Supplementary Table 5

GSEA based on RNA sequencing upon *HOTAIRM1* siRNA-mediated knockdown in glioblastoma cells. Top 10 negatively enriched genesets in the *HOTAIRM1* knockdown cells are listed according to their normalized expression score values. NES: normalized expression score; NOM: nominal; FDR: false discovery rate.

**GeneSet details**	**NES**	**NOM *p*-value**	**FDR *q*-value**
RUNX1 REGULATES TRANSCRIPTION OF GENES INVOLVED IN DIFFERENTIATION OF HSCS_REACTOME DATABASE ID RELEASE 61_8939236	-2.22344	<0.001	<0.001
REGULATION OF MITOTIC CELL CYCLE_REACTOME DATABASE ID RELEASE 61_453276	-2.17473	<0.001	<0.001
ACTIVATED PKN1 STIMULATES TRANSCRIPTION OF AR (ANDROGEN RECEPTOR) REGULATED GENES KLK2 AND KLK3_REACTOME_R-HSA-5625886.1	-2.16665	<0.001	3.36E-04
DEPOSITION OF NEW CENPA-CONTAINING NUCLEOSOMES AT THE CENTROMERE_REACTOME_R-HSA-606279.1	-2.16189	<0.001	2.52E-04
RESPIRATORY ELECTRON TRANSPORT, ATP SYNTHESIS BY CHEMIOSMOTIC COUPLING, AND HEAT PRODUCTION BY UNCOUPLING PROTEINS._REACTOME_R-HSA-163200.1	-2.15902	<0.001	2.01E-04
APC C-MEDIATED DEGRADATION OF CELL CYCLE PROTEINS_REACTOME DATABASE ID RELEASE 61_174143	-2.15268	<0.001	4.19E-04
APC C:CDC20 MEDIATED DEGRADATION OF MITOTIC PROTEINS_REACTOME_R-HSA-176409.2	-2.15107	<0.001	3.59E-04
ACTIVATION OF APC C AND APC C:CDC20 MEDIATED DEGRADATION OF MITOTIC PROTEINS_REACTOME DATABASE ID RELEASE 61_176814	-2.15100	<0.001	3.14E-04
NUCLEOSOME ASSEMBLY_REACTOME_R-HSA-774815.1	-2.15021	<0.001	2.79E-04
RNA POLYMERASE I PROMOTER OPENING_REACTOME_R-HSA-73728.2	-2.14531	<0.001	2.51E-04

Supplementary Table 6

GSEA results using proteomic data upon *HOTAIRM1* siRNA-mediated knockdown. Top 10 positively enriched genesets in the *HOTAIRM1* knockdown cells are listed according to their normalized expression score values. NES: normalized expression score; NOM: nominal; FDR: false discovery rate.

**GeneSet details**	**NES**	**NOM *p*-value**	**FDR *q*-value**
CLASS I MHC MEDIATED ANTIGEN PROCESSING & PRESENTATION_REACTOME_R-HSA-983169.3	2.30708	<0.001	5.23E-04
GOLGI VESICLE TRANSPORT_GOBP_GO:0048193	2.21344	<0.001	0.005067
REGULATION OF PHOSPHATASE ACTIVITY_GOBP_GO:0010921	2.19807	<0.001	0.005149
ANTIGEN PRESENTATION: FOLDING, ASSEMBLY AND PEPTIDE LOADING OF CLASS I MHC_REACTOME DATABASE ID RELEASE 61_983170	2.1921	<0.001	0.004262
PROTEIN UBIQUITINATION INVOLVED IN UBIQUITIN-DEPENDENT PROTEIN CATABOLIC PROCESS_GOBP_GO:0042787	2.12129	<0.001	0.010577
ER TO GOLGI ANTEROGRADE TRANSPORT_REACTOME_R-HSA-199977.3	2.12091	<0.001	0.008901
ANTIGEN PROCESSING: UBIQUITINATION & PROTEASOME DEGRADATION_REACTOME DATABASE ID RELEASE 61_983168	2.11776	<0.001	0.007933
TRANSPORT TO THE GOLGI AND SUBSEQUENT MODIFICATION_REACTOME_R-HSA-948021.2	2.10455	<0.001	0.008475
GOLGI-TO-ER RETROGRADE TRANSPORT_REACTOME DATABASE ID RELEASE 61_8856688	2.07161	<0.001	0.01416
REGULATION OF DEPHOSPHORYLATION_GOBP_GO:0035303	2.05964	9.29E-04	0.015143

Supplementary Table 7

GSEA using proteomic data upon *HOTAIRM1* siRNA-mediated knock-down. Top 10 negatively enriched genesets in the *HOTAIRM1* knockdown cells are listed according to their normalized expression score values. NES: normalized expression score; NOM: nominal; FDR: false discovery rate.

**GeneSet details**	**NES**	**NOM *p*-value**	**FDR *q*-value**
RIBOSOME BIOGENESIS_GOBP_GO:0042254	-3.206222	<0.001	<0.001
RNA PROCESSING_GOBP_GO:0006396	-3.094729	<0.001	<0.001
RRNA PROCESSING_GOBP_GO:0006364	-3.088324	<0.001	<0.001
RRNA PROCESSING_REACTOME_R-HSA-72312.3	-3.081031	<0.001	<0.001
MAJOR PATHWAY OF RRNA PROCESSING IN THE NUCLEOLUS AND CYTOSOL_REACTOME_R-HSA-6791226.3	-3.06467	<0.001	<0.001
RRNA PROCESSING IN THE NUCLEUS AND CYTOSOL_REACTOME_R-HSA-8868773.2	-3.03982	<0.001	<0.001
RRNA METABOLIC PROCESS_GOBP_GO:0016072	-3.029373	<0.001	<0.001
RIBONUCLEOPROTEIN COMPLEX BIOGENESIS_GOBP_GO:0022613	-2.961836	<0.001	<0.001
NCRNA PROCESSING_GOBP_GO:0034470	-2.935496	<0.001	<0.001
STRUCTURAL CONSTITUENT OF RIBOSOME_GOMF_GO:0003735	-2.858148	<0.001	<0.001

Supplementary Table 8

GSEA of integrated proteogenomic datasets obtained upon *HOTAIRM1* siRNA-mediated knock-down in glioma cells. The genesets printed in red color are mitochondria-related genesets. The genesets are listed according to the RNA sequencing *q*-values. RNA-seq: RNA sequencing; NES: normalized expression score; NOM: nominal; FDR: false discovery rate.

GeneSet details	RNA-seq NES	RNA-seq NOM *p*-value	RNA-seq FDR *q*-value	Proteo-mics NES	Proteo-mics NOM *p*-value	Proteo-mics FDR *q*-value
MITOCHONDRIAL INNER MEMBRANE_GOCC_GO:0005743	-2.108	<0.001	0.00014	-2.072	<0.001	0.01962
STRUCTURAL CONSTITUENT OF RIBOSOME_GOMF_GO:0003735	-2.100	<0.001	0.00017	-2.858	<0.001	<0.001
MITOCHONDRIAL TRANSLATION ELONGATION_REACTOME_R-HSA-5389840.1	-2.092	<0.001	0.00019	-2.306	<0.001	0.00112
RIBOSOMAL SUBUNIT_GOCC_GO:0044391	-2.141	<0.001	0.00021	-2.715	<0.001	<0.001
MITOCHONDRIAL TRANSLATION INITIATION_REACTOME DATABASE ID RELEASE 61_5368286	-2.082	<0.001	0.00022	-2.345	<0.001	0.00061
MITOCHONDRIAL TRANSLATION_REACTOME DATABASE ID RELEASE 61_5368287	-2.074	<0.001	0.00023	-2.330	<0.001	0.00074
RIBOSOME_GOCC_GO:0005840	-2.073	<0.001	0.00023	-2.707	<0.001	<0.001
MITOCHONDRIAL TRANSLATIONAL ELONGATION_GOBP_GO:0070125	-2.075	<0.001	0.00023	-2.307	<0.001	0.00109
TRANSLATION_REACTOME_R-HSA-72766.3	-2.073	<0.001	0.00024	-2.206	<0.001	0.00472
TRANSLATION_GOBP_GO:00064	-2.068	<0.001	0.00024	-2.084	<0.001	0.01834

12						
MITOCHONDRIAL TRANSLATION TERMINATION_REACTOME_R-HSA-5419276.1	-2.063	<0.001	0.00028	-2.337	<0.001	0.00065
ORGANELLE INNER MEMBRANE_GOCC_GO:0019866	-2.058	<0.001	0.00033	-2.066	<0.001	0.02018
MITOCHONDRIAL TRANSLATION_GOBP_GO:0032543	-2.043	<0.001	0.00038	-2.225	<0.001	0.00370
PEPTIDE BIOSYNTHETIC PROCESS_GOBP_GO:0043043	-2.050	<0.001	0.00039	-1.994	<0.001	0.03187
LARGE RIBOSOMAL SUBUNIT_GOCC_GO:0015934	-2.045	<0.001	0.00039	-2.524	<0.001	<0.001
TRANSLATIONAL ELONGATION_GOBP_GO:0006414	-2.046	<0.001	0.00040	-2.289	<0.001	0.00142
MITOCHONDRIAL TRANSLATIONAL TERMINATION_GOBP_GO:0070126	-2.039	<0.001	0.00042	-2.3571	<0.001	0.00048
AMIDE BIOSYNTHETIC PROCESS_GOBP_GO:0043604	-2.030	<0.001	0.00046	-1.935	<0.001	0.04255
ORGANELLAR RIBOSOME_GOCC_GO:0000313	-2.025	<0.001	0.00051	-2.288	0.0021	0.00142
MITOCHONDRIAL GENE EXPRESSION_GOBP_GO:0140053	-2.011	<0.001	0.00062	-2.278	<0.001	0.00158
MITOCHONDRIAL RIBOSOME_GOCC_GO:0005761	-2.002	<0.001	0.00070	-2.263	<0.001	0.00199

Supplementary Table 10

List of top significantly upregulated proteins following *HOTAIRM1* stable knockdown in LN-229 glioblastoma cells. The proteins are listed according to their *q*-values.

Protein ID	Description	Fold change	*p*-value	*q*-value
L1CAM	Neural cell adhesion molecule L1	1.74303	2.01E-06	0.00362
LCN2	Neutrophil gelatinase-associated lipocalin	12.0809	1.11E-05	0.00385
HSD17B10	3-hydroxyacyl-CoA dehydrogenase type-2	1.91749	6.58E-06	0.00385
AKR1C3	Aldo-keto reductase family 1 member C3	1.99863	2.18E-05	0.00522
CALB2	Calretinin	1.85151	5.60E-05	0.01011
KYNU	Kynureninase	1.91012	0.00011	0.01627
DYNLRB1	Dynein light chain roadblock-type 1	1.58320	0.00012	0.01642
ANKRD52	Serine/threonine-protein phosphatase 6 regulatory ankyrin repeat subunit C	3.11078	0.00021	0.02369
RAP1GAP2	Rap1 GTPase-activating protein 2	1.64261	0.00024	0.02369
PDLIM4	PDZ and LIM domain protein 4	1.64951	0.00048	0.03625
INF2	Inverted formin-2	1.62381	0.00047	0.03625
AKR1B1	Aldose reductase	1.75732	0.00059	0.04126
GAK	Cyclin-G-associated kinase	1.75092	0.00096	0.04641
SLC2A1	Solute carrier family 2, facilitated glucose transporter member 1	1.57233	0.00091	0.04641
L3HYPDH	Trans-L-3-hydroxyproline dehydratase	1.53126	0.00099	0.04641
DDT	D-dopachrome decarboxylase	1.64757	0.00109	0.04672

Supplementary Table 11

List of top significantly downregulated proteins following *HOTAIRM1* stable knockdown in LN-229 glioblastoma cells. The proteins are listed according to their *q*-values. Transglutaminase 2 (TGM2) is used for further assays and is highlighted.

Protein ID	Description	Fold change	*p*-value	*q*-value
TMEM87A	Transmembrane protein 87A	-6.44115	1.28E-05	0.00385
MYO6	Myosin-VI	-3.57295	1.00E-05	0.00385
RFTN1	Raftlin	-1.84698	4.56E-06	0.00385
NGFR	Nerve growth factor receptor	-15.5604	2.31E-05	0.00522
**TGM2**	**Transglutaminase 2**	**-1.62376**	**3.20E-05**	**0.00641**
HAT1	Histone acetyltransferase type B catalytic subunit	-1.61425	0.00011	0.01627
SLC4A7	Sodium bicarbonate cotransporter 3	-2.20409	0.00024	0.02369
PKN2	Serine/threonine-protein kinase N2	-1.69435	0.00032	0.02888
C16orf58	Chromosome 16 open reading frame 58	-2.21576	0.00037	0.03146
SORT1	Sortilin	-2.08654	0.00059	0.04126
UBR1	E3 ubiquitin-protein ligase UBR1	-1.57988	0.00070	0.04252
HSPA14	Heat shock 70 kDa protein 14	-2.13546	0.00091	0.04641

Supplementary Table 12

List of microRNAs predicted to bind to *HOTAIRM1* and the mRNAs of one or more of the 12 downregulated proteins identified upon *HOTAIRM1* knockdown in LN-229 cells. microRNAs are ranked based on *p*-values. *TGM2* is highlighted in the table. miRNA: microRNA; mRNA: messenger RNA; TCGA: The Cancer Genome Atlas.

miRNA	mRNA	TCGA correlation	*p*-value
hsa-miR-17-5p	**TGM2**	-0.397026	3.29E-05
hsa-miR-93	**TGM2**	-0.395785	3.50E-05
hsa-miR-95	**TGM2**	-0.354698	0.0002368
hsa-miR-20b	**TGM2**	-0.343056	0.00038901
hsa-miR-328	**TGM2**	-0.342861	0.00039218
hsa-miR-106a	**TGM2**	-0.327445	0.00073486
hsa-miR-20a	**TGM2**	-0.321762	0.00091882
hsa-miR-20b	RFTN1	-0.317927	0.00106573
hsa-miR-130b	**TGM2**	-0.315417	0.00117314
hsa-miR-17-5p	RFTN1	-0.310938	0.00138963
hsa-miR-93	RFTN1	-0.305739	0.001686
hsa-miR-153	RFTN1	-0.296443	0.00236182
hsa-miR-153	**TGM2**	-0.295234	0.00246571
hsa-miR-95	RFTN1	-0.285786	0.0034302
hsa-miR-92	SORT1	-0.28524	0.00349507
hsa-miR-106b	**TGM2**	-0.281732	0.00393889
hsa-miR-106a	RFTN1	-0.253882	0.00966134
hsa-miR-95	SLC4A7	-0.25275	0.0100009
hsa-miR-25	NGFR	-0.252134	0.0101904
hsa-miR-101	**TGM2**	-0.24385	0.0130574
hsa-miR-598	**TGM2**	-0.239846	0.0146794
hsa-miR-92	**TGM2**	-0.234186	0.0172689
hsa-miR-328	SORT1	-0.232079	0.018329
hsa-miR-20a	RFTN1	-0.224141	0.0228423
hsa-miR-328	RFTN1	-0.223122	0.0234852
hsa-miR-25	**TGM2**	-0.219973	0.0255714
hsa-miR-17-5p	SORT1	-0.217921	0.0270137
hsa-miR-148b	SLC4A7	-0.205092	0.0376912
hsa-miR-130b	SORT1	-0.203905	0.0388369
hsa-miR-130b	RFTN1	-0.201252	0.0415039
hsa-miR-106b	RFTN1	-0.198482	0.0444489
hsa-miR-328	HAT1	-0.1969	0.046208

hsa-miR-20a	SORT1	-0.196052	0.0471739
hsa-miR-148b	RFTN1	-0.194955	0.0484485
hsa-miR-106a	SORT1	-0.194221	0.0493178
