# Supplementary material for: The long non-coding RNA HOTAIRM1 promotes tumor aggressiveness and radiotherapy resistance in glioblastoma
Source: Cell Death Dis. 2021 Sep 28;12(10):885. doi: 10.1038/s41419-021-04146-0 (PMC8478910; doi:10.1038/s41419-021-04146-0)
Supplement: Supplementary file 16 — Supplementary Table 9 [file 41419_2021_4146_MOESM16_ESM.pdf]

Supplementary Table 9. Mass spectrometry results of stable LN-229 *HOTAIRM1* knockdown cells
Data supplied as Log2 intensities

ID	LN-229_control	LN-229_control	LN-229_control	LN-229_control	LN-229_control	LN-229_HOTAIRM1_KD	LN-229_HOTAIRM1_KD	LN-229_HOTAIRM1_KD	LN-229_HOTAIRM1_KD
AAAS	20.2707	20.0783	20.2281	20.1135	20.0942	20.1536	20.6406	20.3122	20.3552
AAGAB	17.6663	17.0309	17.4114	17.262	17.5227	16.1273	16.958	17.9081	17.51
AAK1	17.865	17.4153	17.3982	17.3919	17.2845	17.0373	17.657	17.6172	17.9443
AAMP	18.4515	18.2325	18.4887	18.4097	18.0909	18.0543	18.3055	18.1718	18.271
AAR2	19.4322	18.8333	18.7869	19.0179	19.0206	19.1339	19.0521	18.8584	18.7929
AARS	22.1567	22.098	22.0975	21.8621	21.9425	21.8565	22.0762	22.0603	22.0607
AARS2	17.1108	17.6265	17.7677	17.5017	17.6652	17.1859	17.6826	17.4313	17.5711
AARSD1	17.4513	17.2727	16.9285	16.882	17.3279	16.2446	16.6043	16.9875	16.7124
AASDHPPT	17.5614	17.8649	18.1514	17.977	18.0368	18.0058	17.8814	17.8439	17.7687
AASS	17.5613	17.805	17.7738	17.4612	17.2856	17.1547	17.8981	17.39	17.3529
AATF	17.4786	18.2297	17.4855	17.6062	17.6245	16.3923	17.5642	17.3761	17.227
ABCB6	18.0897	17.7693	17.3536	17.7866	17.1946	16.718	17.1812	17.1677	17.1689
ABCB7	17.7559	17.6342	17.6613	17.6056	17.5093	17.2331	17.7127	17.5104	17.9313
ABCC1	16.9508	17.991	18.476	16.9534	19.0042	17.2	17.9608	18.6345	18.2048
ABCD3	19.5934	21.8682	19.4256	19.5755	19.1766	19.1624	19.5673	19.0886	19.2144
ABCE1	22.5646	22.5373	22.5579	22.5111	22.3183	22.2164	22.7736	22.407	22.5842
ABCF1	19.4344	19.5852	19.4591	19.3458	18.8525	19.4295	19.2642	19.5144	19.3027
ABCF2	21.0286	20.8668	20.8339	20.6697	20.6977	20.7797	20.7828	20.6957	21.1179
ABCF3	18.5307	18.9513	19.0496	18.6996	18.6522	18.654	18.7082	19.0327	19.119
ABHD10	19.975	19.998	19.8874	19.612	19.6173	19.6614	20.0365	19.6247	19.7041
ABHD11	18.2972	17.8886	18.1341	17.4964	17.8399	18.0518	18.6408	18.4499	18.4084
ABHD12	19.2667	19.159	19.2445	19.0701	19.069	19.472	19.9858	19.0976	19.2175
ABHD14B	18.913	18.5617	18.8039	18.7821	18.7288	18.8722	18.7176	18.9307	18.6655
ABI1	18.8012	18.7405	18.6684	18.7688	18.5576	18.8425	18.7462	18.6975	18.8246
ABR	19.1311	19.0967	19.1768	19.1341	18.5326	18.9874	19.6998	19.49	19.6512
ACAA1	19.0736	19.0478	18.6454	18.872	18.9285	18.1179	18.8641	18.9122	18.6769
ACAA2	18.7813	19.015	18.9557	19.0671	19.0966	19.1874	19.0111	18.8585	18.868
ACACA	18.8626	18.4276	18.886	18.2945	18.3278	18.8262	19.4906	19.0367	19.159
ACAD9	18.9279	18.1975	18.5254	18.5797	18.4079	19.0408	18.8441	18.6309	18.6695
ACADM	21.9486	21.8902	21.7361	21.4832	21.4019	21.3595	21.57	21.4444	21.6066
ACADSB	19.8036	19.7828	19.8745	19.8995	19.4138	19.7786	19.5798	19.9559	20.1143
ACADVL	21.9171	21.7424	21.6106	21.6274	21.6279	21.7612	22.0421	21.6411	21.7654
ACAT1	18.6728	18.6031	18.7436	18.3107	18.4981	17.7671	18.4073	18.1087	18.1148
ACAT2	21.1871	20.9658	20.7784	20.8098	20.8712	20.3654	20.8885	20.6134	20.7426
ACBD3	19.0722	19.2195	19.6441	19.4403	19.3448	19.3466	19.6976	19.3149	19.6893
ACIN1	21.3035	21.1486	21.1244	21.1519	20.8603	20.9571	21.3336	21.1893	21.3689
ACLY	21.7215	21.5281	21.4824	21.4766	21.2425	21.5196	22.0833	21.7609	21.8702
ACO1	20.5695	20.6481	20.4341	20.7776	20.5666	20.0469	20.4318	20.6001	20.6533
ACO2	21.2524	21.4578	21.1264	21.0654	20.9151	21.2266	21.569	21.2691	21.6625
ACOT1	19.0417	19.0262	19.3871	19.1197	18.9929	18.8757	19.4006	19.0419	19.2177
ACOT7	22.2444	22.0321	22.056	22.0371	21.9626	22.3216	22.3005	22.1691	22.3144
ACOT8	17.9123	17.649	17.8532	17.7004	17.7247	17.9504	18.3764	18.1407	18.0624
ACOT9	18.5695	17.9613	18.361	18.0696	18.3545	17.965	18.8737	18.5963	18.4208
ACOX1	19.6059	19.465	19.0127	19.1068	19.0818	18.1407	18.7096	18.7197	18.8624

ACP1	21.6768	21.8408	21.9542	21.8347	21.8663	21.9736	22.1306	21.7157	21.804
ACSL1	21.0513	21.088	20.7944	20.9395	20.7495	20.8432	21.1355	20.9222	20.9865
ACSL3	22.3696	22.4171	22.5031	22.3749	22.2242	22.3208	22.779	22.5807	22.7984
ACSL4	18.7237	18.4619	18.1241	18.3105	18.2563	18.3026	18.3787	18.4167	18.3844
ACSS1	20.3723	20.4843	20.46	20.22	20.0736	20.5863	20.8631	20.536	20.8021
ACSS2	17.7504	17.4999	17.7752	17.8448	17.5238	17.4424	17.1961	17.7493	17.5747
ACTB	29.277	28.8595	29.0482	29.0463	28.9532	29.2471	29.4416	29.0394	29.0789
ACTL6A	21.7197	21.847	21.9802	21.7386	21.7194	21.5448	21.7551	21.3166	21.7725
ACTN1	24.1303	23.9528	23.9178	23.6596	23.6555	23.7747	24.1185	24.0333	23.9083
ACTN4	24.4606	24.2864	24.2812	24.2824	23.9326	24.2466	24.4606	24.3321	24.5721
ACTR10	16.9833	17.9435	16.902	16.9224	16.7639	16.7786	16.401	16.637	16.3717
ACTR1A	20.8616	20.705	20.6712	20.56	20.6067	20.8911	20.8256	20.6339	20.7262
ACTR2	22.7509	22.0382	22.5968	22.6665	22.4107	22.4575	22.7709	22.5529	22.8327
ACTR3	22.8615	22.5473	22.6675	22.7562	22.6275	22.5764	22.7228	22.6758	22.5689
ACY1	19.0091	18.6754	18.7912	18.7818	18.5153	18.8391	19.0021	19.0849	18.2399
ADAR	21.8386	21.3819	21.3956	21.3991	21.4285	21.3322	21.2033	21.5015	21.5748
ADD1	18.879	18.2212	18.3837	18.3163	18.2787	18.4852	19.0293	18.7459	18.6225
ADD3	18.5204	18.6317	18.4717	18.5524	18.3571	18.0329	17.8758	18.2973	18.3314
ADH5	22.4609	22.1299	22.1927	22.2119	22.0594	22.0917	22.1917	22.1736	22.0249
ADK	21.7978	20.8303	21.3748	22.2086	21.575	21.7821	21.4535	21.507	21.6883
ADNP	19.7295	19.3866	19.3609	19.5098	19.2681	19.308	19.7058	19.5676	19.6308
ADPGK	18.6183	19.1762	19.0641	18.6062	19.2183	19.0875	19.713	19.4654	18.9897
ADPRHL2	17.9037	17.8052	17.8213	17.6776	17.6398	18.0362	17.8706	17.8254	17.4656
ADRM1	21.5651	21.645	21.8251	21.4805	21.6554	21.3998	21.7462	21.3606	21.5625
ADSL	21.7911	21.4216	21.4833	21.4548	21.2657	21.4364	22.0937	21.6334	21.3634
ADSS	21.3701	21.3075	21.4561	21.2659	21.1638	21.0585	21.2662	20.9494	21.0479
AFAP1	17.5287	16.0129	16.6414	17.0969	16.8119	17.2978	18.0108	16.6986	17.761
AFG3L2	18.361	18.4177	18.4917	18.1948	18.2859	18.5957	18.8601	18.4991	18.2239
AGA	19.757	20.0035	19.0981	19.5192	19.7956	19.8336	19.9307	19.4045	19.6267
AGAP3	16.1945	16.9296	16.7122	16.441	16.3536	15.9534	16.5963	16.8693	16.9493
AGFG1	18.9831	18.3601	18.9888	18.9287	18.6297	18.7664	18.7695	18.2772	18.5331
AGK	22.2888	21.6877	21.5814	21.9907	21.4106	22.0757	21.4135	22.4171	22.2836
AGL	17.8503	17.827	17.9895	17.6402	17.8122	17.1735	17.9971	17.9247	17.8528
AGPAT9	20.5083	19.4239	20.2994	20.5434	20.1027	20.3489	20.4691	20.6717	20.9465
AGPS	20.4207	20.0226	20.1027	19.979	19.8021	19.941	19.9599	20.1204	20.1923
AHCTF1	16.6928	16.5038	16.6124	16.1841	16.4535	16.7705	17.1022	16.7952	16.7933
AHCY	23.9758	23.4198	24.1749	24.0435	23.9907	24.229	24.6258	24.1245	24.228
AHCYL1	21.1959	22.8062	21.2974	21.3471	21.1821	21.3712	21.7761	21.3021	21.6034
AHNAK	24.1812	23.9109	23.8986	23.8746	23.6438	23.8004	23.8301	23.7283	24.0051
AHNAK2	21.1172	21.0201	20.9921	21.0972	20.7116	21.3463	21.3239	21.3055	21.666
AHSA1	21.3715	21.0808	21.2187	21.3138	21.2039	21.2984	21.2735	21.2804	21.3681
AHSG	17.2003	17.3625	17.9427	18.2116	18.3985	18.7307	18.7225	16.3233	18.2959
AIFM1	19.0226	18.8887	18.8728	18.9839	18.9105	19.0198	18.605	19.2237	19.0943
AIMP1	22.1253	21.6966	21.93	21.8061	21.6544	21.9188	22.1194	21.8533	21.9493
AIMP2	21.9385	21.7109	21.7005	21.7632	21.648	21.9857	22.0666	21.6916	21.936
AIP	21.5589	21.1184	21.3231	21.5554	21.1953	21.5452	21.5927	21.5062	21.6009
AK1	20.4552	20.58	20.9214	20.5527	20.5114	20.7853	20.9074	20.6289	20.7016
AK2	22.2256	21.1098	22.3007	22.1538	22.1376	22.5211	22.8104	22.3067	22.5304
AK3	18.7816	19.5092	19.443	19.6733	19.5981	19.7748	19.9865	19.5451	19.8131
AK4	17.8322	17.9948	17.9302	17.7643	18.0199	18.3847	18.5235	18.0087	18.2467
AK6	17.385	19.0882	18.2308	18.5784	18.8402	18.1252	18.8915	18.5922	18.7949
AKAP1	17.5936	18.0591	18.3578	17.3	18.2863	18.2559	18.1655	18.0012	17.9761
AKAP12	22.1933	21.4894	21.8247	21.9977	21.7914	21.6587	22.2353	21.6491	21.8767
AKAP8	17.3447	17.3948	16.9047	16.3952	16.6445	16.449	16.3527	16.9651	16.8346
AKR1A1	19.0337	19.1508	18.9182	18.9799	18.9597	19.1764	19.3383	19.0965	19.2399
AKR1B1	21.9911	21.9815	22.0231	22.2059	22.131	22.8712	23.2775	22.5807	22.7902

AKR1C3	18.3144	17.8427	18.1635	18.0833	17.915	19.0412	19.1023	19.1235	18.9841
AKR7A2	19.7217	19.4289	19.2737	18.9855	19.1845	19.2248	19.3938	19.6647	19.6171
AKT3	19.3152	18.7989	18.8975	18.5731	18.2481	18.4318	18.5824	18.8933	18.8415
ALB	23.6473	22.8948	23.7115	24.2191	23.6903	23.2829	23.6929	22.8422	23.0122
ALDH16A1	18.6913	19.397	18.5185	18.5889	18.2531	18.4013	18.6091	18.3663	18.4047
ALDH18A1	21.0862	20.5328	20.9775	20.9916	21.0924	21.371	22.0357	21.286	21.4804
ALDH1A3	24.8235	24.6968	24.6898	24.6142	24.6303	24.5416	24.5777	24.5945	24.3404
ALDH1B1	18.9522	18.394	18.2638	18.6896	17.9664	18.8107	18.7669	19.029	19.3342
ALDH3A2	18.3783	19.3961	19.8607	18.6221	19.6525	18.6811	20.0136	18.0947	18.8074
ALDH5A1	18.626	18.6124	18.424	18.5463	18.3361	18.1728	18.4913	18.5578	18.4786
ALDH7A1	21.5986	21.4785	21.5905	21.4826	21.38	21.1839	21.42	21.2414	21.145
ALDH9A1	20.9953	20.6696	21.1749	20.8836	20.9721	21.0925	21.256	20.6804	20.992
ALDOA	25.803	25.7619	25.7394	25.881	25.7997	26.2092	26.1169	25.9502	25.9675
ALDOC	23.7622	23.6191	23.6869	23.7898	23.3334	23.4589	22.8708	23.5151	23.635
ALG1	16.6647	16.8451	16.8641	16.8322	16.8009	17.1251	16.7536	16.8508	16.4599
ALYREF	21.5541	20.9186	21.1543	21.6786	21.3637	21.4493	21.3226	21.3504	21.4255
AMPD2	18.6065	18.9797	18.8788	18.4394	18.3673	18.3373	18.4837	18.4405	18.5387
ANAPC1	17.4084	17.4837	17.8948	16.893	17.783	17.7757	17.8091	17.2272	17.0215
ANAPC5	18.4858	17.7094	17.4592	17.5874	17.7998	17.4524	17.7238	17.9841	17.7343
ANAPC7	19.1161	18.4352	18.9454	19.3157	18.8002	18.8503	19.1277	18.4963	18.9192
ANKFY1	16.6204	16.6999	16.6554	16.6434	16.8468	16.9098	16.6216	16.6022	16.298
ANKLE2	18.0503	18.2159	18.3343	18.4184	18.1748	18.6887	18.6499	18.5334	18.7396
ANKRD28	18.5992	18.5236	18.586	18.4899	18.5445	18.3903	18.6021	18.4707	18.3892
ANKRD52	16.2633	15.8642	15.9593	15.7204	15.9431	17.2376	18.2597	17.6146	17.2376
ANLN	19.0892	18.9358	19.2058	18.9917	19.0653	19.2959	19.2425	19.1536	18.749
ANP32A	19.6471	19.0374	20.0787	19.8773	20.2865	20.9483	21.1201	19.9629	19.9959
ANP32B	21.1934	21.1357	21.2203	21.3404	21.0785	21.5403	21.2012	21.2591	21.2632
ANP32E	22.0365	21.9017	21.8219	21.9115	21.8769	21.7782	21.9202	21.6387	21.7059
ANXA1	24.5766	24.2399	24.7297	24.5278	24.6737	24.7408	25.0626	24.2995	24.5677
ANXA11	21.2963	21.2847	21.1758	21.5442	21.3681	21.4093	21.6756	21.2618	21.5098
ANXA2	26.865	26.1036	26.7024	26.7306	26.6252	26.8751	27.0859	26.7334	26.9354
ANXA4	19.666	19.5458	19.473	19.4971	19.4142	19.4008	19.3439	19.2355	19.3761
ANXA5	24.7986	24.9179	24.8757	24.6788	24.5474	24.7538	24.9276	24.695	24.7631
ANXA6	21.4994	21.1081	21.376	21.3241	21.0364	21.2868	21.4958	21.2606	21.2446
ANXA7	21.2153	20.7299	20.8392	20.6299	20.5229	20.5081	20.8619	20.4871	20.6316
AP1B1	20.7594	20.7061	20.9424	20.8116	20.7178	20.8956	21.2133	21.0529	21.2684
AP1G1	20.4601	20.2268	20.3715	20.118	20.1671	19.9298	20.6076	20.1968	20.5111
AP1M1	20.8432	20.6037	20.5183	20.6208	20.5212	20.5453	20.8362	20.6335	20.653
AP1S1	18.5836	18.9847	18.2238	18.156	17.9769	18.5537	18.5206	18.67	18.7938
AP2A1	20.6273	20.5566	20.7599	20.4189	20.4131	20.4926	20.8128	20.6339	20.7046
AP2A2	19.3546	19.2135	19.0672	19.1708	19.0171	18.9218	19.1076	18.9418	19.1302
AP2B1	21.0718	20.9564	20.828	20.8918	20.8741	20.6959	20.9558	20.9289	21.0194
AP2M1	19.9341	19.6608	19.6944	19.8013	19.6489	19.813	20.1718	19.8012	19.8041
AP2S1	19.9283	20.4814	20.5559	20.308	20.2161	20.3722	20.3064	20.1853	20.2067
AP3B1	19.7302	19.9394	19.9524	20.0384	19.7254	19.4741	20.1183	19.8884	19.9556
AP3D1	20.3236	19.7242	19.1956	19.1243	18.9554	18.4382	18.933	19.3388	19.2446
AP3M1	19.9347	19.7049	19.9594	19.8145	19.5965	19.8358	20.2866	19.9524	19.9754
AP3S1	18.5171	18.5068	18.5337	18.488	18.3404	18.6043	18.7324	18.6139	18.5528
APEH	20.6766	21.0884	21.2096	20.9687	20.9971	21.0793	21.1715	20.9938	21.2116
APEX1	21.8105	24.0342	21.2678	21.572	21.112	21.5917	21.5004	21.5557	22.045
API5	21.5952	21.9925	22.0037	21.7859	21.7084	21.4406	21.5176	21.6632	21.7633
APIP	13.7404	17.1436	17.2913	17.4301	17.3914	17.1839	16.7538	17.0711	16.6345
APMAP	23.4462	23.5412	23.5592	23.3078	23.2619	23.3407	23.7533	23.3188	23.591
APOA1BP	19.864	19.7112	19.5543	20.2901	19.4344	19.5097	19.2323	19.4049	19.6539
APOBEC3B	20.8007	20.8663	20.5191	20.4881	19.9634	20.3132	20.7362	20.7982	20.9256
APOOL	16.1328	16.6473	16.5934	16.4732	16.0465	15.3932	15.3625	16.1988	15.7745

APP	16.3745	16.0997	15.7539	15.7973	16.2041	15.7683	16.5656	16.118	16.3441
APPL1	19.1506	19.0217	19.0403	19.0607	18.933	18.8553	19.5297	19.0544	19.1112
APRT	21.9901	21.6489	22.0357	21.9392	21.934	22.6035	22.9473	22.2463	22.2761
AQR	19.7897	19.7934	19.9224	19.5879	19.649	19.4564	19.855	19.7217	19.7329
ARAF	18.1358	17.7906	17.8749	17.9449	17.6983	17.9183	17.5593	18.0485	17.9924
ARAP3	16.0032	16.948	16.6431	16.215	16.0891	16.0907	17.0777	16.4509	17.0105
ARCN1	21.8862	21.7284	21.8042	21.513	21.6113	21.8498	22.0146	21.6487	21.7458
ARF1	22.3158	25.6912	21.8947	22.3064	21.8619	22.3749	21.7335	22.3287	22.096
ARF4	21.8963	22.0109	22.1965	22.1107	22.0331	22.111	21.9177	22.0433	22.2528
ARF5	19.2837	19.5021	19.3957	19.4295	19.3752	19.7369	19.5335	19.7159	19.5125
ARF6	19.7352	20.1998	20.2903	20.1594	20.555	20.6215	20.2973	20.5055	20.1551
ARFGAP1	19.3766	19.0827	19.1788	19.0331	18.9712	19.0912	19.4706	19.0139	19.0528
ARFGAP3	18.5157	18.0075	18.6995	18.2691	18.4583	17.9552	17.8315	18.1296	17.5347
ARFGEF1	15.9465	17.2485	16.7071	17.2711	16.8581	16.9206	16.5799	16.5008	16.4166
ARFGEF2	17.2041	17.1774	17.2997	17.0746	16.7352	16.3177	16.4578	16.5686	17.2347
ARFIP1	18.8402	19.0988	18.8827	19.0963	18.8942	18.8271	18.8867	19.018	19.3551
ARFIP2	17.6861	16.8849	16.9761	17.4088	17.3327	17.4862	17.77	17.4302	17.7596
ARHGAP1	20.3265	20.6265	20.5459	20.5548	20.4518	20.6391	21.0674	20.4607	20.7855
ARHGAP35	17.0164	16.9059	17.1886	17.0024	17.0209	16.5237	17.3493	17.0804	16.8216
ARHGAP5	15.3404	15.9085	16.0756	15.7702	15.9723	15.8202	15.8613	16.2868	16.3906
ARHGDIA	23.7888	23.2975	23.6157	23.5743	23.3739	24.0347	24.2633	23.9046	24.0383
ARHGEF1	21.339	21.9557	21.978	21.1566	22.1616	22.7733	20.604	21.5695	21.2849
ARHGEF11	15.4991	16.6138	16.4332	16.3072	16.3719	16.428	16.3946	16.5143	15.9675
ARHGEF2	19.9039	19.5237	19.9454	20.0984	19.8443	19.7252	20.133	19.8189	19.9943
ARIH1	18.7692	19.1992	19.2275	18.9579	19.0473	19.2218	19.714	19.1327	19.3153
ARIH2	15.5805	18.6073	16.2624	15.8862	16.3903	16.4408	17.8499	16.5921	15.5809
ARL1	20.7768	20.9263	21.0143	20.7571	20.7339	20.5789	20.6551	20.6074	20.6408
ARL2	19.1686	19.2459	19.5629	19.305	19.1469	19.0904	18.5854	18.9109	18.8104
ARL3	20.3643	18.9446	18.884	18.8404	18.7306	19.0977	19.4351	18.816	18.8313
ARL6IP5	20.2808	20.515	20.2379	20.4214	20.1218	20.6944	20.7982	20.5594	20.3653
ARL8B	19.7108	19.4963	19.9742	19.6446	19.8916	20.3328	20.1332	19.7043	19.7325
ARMC6	22.3874	21.956	21.9039	22.3402	22.3517	22.397	22.2702	22.6792	21.8086
ARMCX3	18.7344	18.7057	18.5314	18.3919	18.5232	18.8251	19.084	18.689	18.8267
ARPC1A	20.3877	20.2952	20.4576	20.3245	20.3868	20.0264	20.7108	20.3064	20.2594
ARPC1B	21.5252	22.7341	21.3219	21.6368	21.4311	21.6628	21.8841	21.582	21.8618
ARPC2	22.519	22.5581	22.6545	22.2509	22.3036	22.4534	22.7354	22.4662	22.4873
ARPC3	20.236	20.1488	19.9883	19.9813	19.9398	19.8713	20.0715	20.0643	20.0068
ARPC4	23.0706	23.0987	23.0731	22.8968	22.9329	23.0983	23.3107	22.9345	23.0997
ARPC5	21.583	21.6457	21.573	21.5553	21.6762	21.5057	21.9692	21.4237	21.6469
ARPC5L	19.7449	19.2796	19.2445	19.3907	19.418	19.0672	19.3418	19.4341	19.5494
ASAH1	18.1559	18.3761	18.3547	18.0404	18.3299	17.7994	17.9363	17.627	17.4223
ASCC2	15.8111	16.8147	17.0885	16.296	16.6082	16.1707	16.3133	16.8553	16.5288
ASCC3	15.6793	16.6144	16.509	16.7305	16.56	15.0617	16.0666	16.4421	16.2143
ASF1B	19.2404	19.1573	19.4611	19.6575	19.0956	19.8263	19.8663	19.3074	19.5223
ASH2L	18.1394	18.1025	17.9747	18.0795	17.9197	17.9675	17.2081	17.7594	17.7514
ASNA1	20.2276	19.2494	19.8758	20.5362	19.6483	20.6554	20.8259	20.7501	20.4423
ASNS	22.905	22.9684	22.9881	22.9172	22.8432	23.1974	23.6075	23.0816	23.3624
ASPH	19.9065	19.7676	19.6211	19.7128	19.6963	19.3278	19.787	19.8739	19.7822
ASPSCR1	18.6067	18.0421	18.2936	18.2093	18.1145	18.6479	18.7675	18.3222	18.1055
ATAD1	19.3182	19.2809	19.091	19.288	19.0422	19.0377	18.6438	18.8834	19.0456
ATAD2	20.2305	19.9203	20.6274	19.8076	20.2643	20.6032	21.5324	20.7146	20.1177
ATAD3A	21.6399	21.2428	21.1246	21.1657	21.0833	21.2748	21.4072	21.1565	21.185
ATAD3B	20.6971	20.5629	20.6497	20.7916	20.6365	20.9298	20.7611	20.5066	20.6335
ATG3	18.8064	18.8628	18.825	18.9708	18.8906	18.642	18.3592	18.1687	18.1581
ATG5	17.2329	17.7412	17.2815	17.0281	17.2738	17.608	17.298	17.3292	17.768
ATG7	17.682	17.7915	17.995	17.7998	17.8708	17.3643	18.3643	18.0564	17.9117

ATG9A	16.7688	17.1838	17.2621	17.0424	17.233	16.9719	17.3996	17.2949	16.8752
ATIC	23.246	23.0536	23.1376	22.962	22.9901	23.0161	23.3512	23.0222	23.2143
ATL2	20.1631	20.0901	20.0294	19.8435	19.8766	20.2885	20.3886	20.1382	20.2985
ATL3	21.9039	21.6031	21.5401	21.5619	21.3789	21.0689	21.4571	21.4117	21.4529
ATP13A1	19.2945	19.0158	19.3936	18.8171	19.0111	18.9212	19.4861	18.8273	18.914
ATP1A1	23.6581	23.5091	23.6826	23.4441	23.2447	23.4894	23.9936	23.4669	23.8372
ATP1B1	20.89	20.7901	20.4821	20.8051	20.2096	20.6398	21.1033	20.6797	20.7464
ATP1B3	22.3989	21.637	22.2642	22.4523	22.2927	22.6118	22.8776	22.3808	22.579
ATP2A2	22.5296	22.4486	22.3693	22.3101	22.1627	22.0052	22.2594	22.13	22.3068
ATP2B1	18.2075	17.8929	18.1606	19.438	17.9406	18.381	17.7353	18.1199	18.0663
ATP5A1	24.4401	24.2672	24.2844	24.038	24.108	24.2956	24.6898	24.383	24.4415
ATP5B	24.2103	24.1845	24.0065	24.2602	23.9202	24.0735	23.8159	23.9159	24.1309
ATP5C1	21.6951	21.5978	21.9264	21.6721	21.5773	21.7923	22.0688	21.7524	21.7796
ATP5F1	20.9426	20.9952	20.8776	20.4911	20.7094	20.5493	21.2336	20.9062	20.9679
ATP5H	21.1711	21.243	21.1892	21.2067	20.9353	21.2755	21.3661	21.1268	21.4238
ATP5I	19.3057	19.1168	19.3497	19.0454	19.1314	18.9429	19.3342	19.2918	19.0898
ATP5J2	20.0043	20.231	20.0545	19.8554	20.0114	19.5453	20.0147	20.115	20.0346
ATP5L	21.3749	21.2607	21.0097	21.0831	20.9813	21.1132	21.448	21.1979	21.4162
ATP5O	22.2297	22.2281	22.2907	22.0765	21.9162	22.3238	22.5846	22.3806	22.3608
ATP6V0A1	19.5377	19.4999	19.4418	19.417	19.3702	19.5483	20.0096	19.6998	19.9042
ATP6V0A2	16.5425	16.7694	16.6197	16.7282	16.4348	15.3282	16.5761	16.6632	16.5534
ATP6V0D1	20.5528	20.4951	20.1941	20.3156	20.1433	20.7335	20.7909	20.5616	20.6856
ATP6V1A	20.6766	20.7524	20.7916	20.4721	20.5278	19.872	20.3198	20.4133	20.3111
ATP6V1B2	21.9492	21.5295	21.5986	21.5352	21.5277	21.5398	21.754	21.5563	21.5013
ATP6V1C1	19.0001	19.0229	18.8984	18.8417	18.9718	19.2665	18.8855	18.6758	18.5498
ATP6V1E1	20.1879	20.1872	20.3592	19.9502	20.0394	20.5334	20.0254	19.9872	20.205
ATP6V1H	19.6392	19.9359	19.8831	19.6252	19.6137	19.4813	19.8837	19.7636	19.7452
ATRIP	21.2881	21.4241	21.4147	21.0245	21.1593	21.1452	21.2143	21.3179	21.4734
ATXN10	20.499	20.1844	20.1948	20.0227	20.1504	20.1429	20.6146	20.2448	20.379
ATXN2	14.5636	16.2478	16	16.3771	15.7357	16.1405	16.3591	16.1141	16.9136
ATXN2L	20.4764	20.2618	20.3966	20.479	20.4657	20.816	20.8449	20.5913	20.9198
AUP1	20.232	20.1964	19.9826	20.2047	19.9863	20.0974	20.4421	20.0507	20.1
AURKB	19.0588	18.8595	19.2083	18.9904	18.8055	19.178	19.3484	19.1773	19.1306
AXL	20.1494	20.3047	20.3843	20.1387	20.0346	19.8369	20.2834	19.8513	20.1989
B2M	20.6531	20.7255	20.6443	20.541	20.8848	20.9345	21.2847	20.541	20.9048
BAG2	19.6225	19.5408	19.35	19.2467	19.0766	19.3563	19.1117	18.9952	19.0404
BAG5	17.8017	17.6525	17.7031	17.465	17.3892	17.3099	17.4841	17.7216	17.741
BAG6	20.271	20.186	20.2568	20.0567	20.1436	20.0798	20.469	20.2102	20.3088
BAIAP2	18.6013	18.7516	18.7698	18.3123	18.5564	18.5477	19.1024	18.7392	18.782
BANF1	21.1737	20.0839	20.558	21.1003	20.5744	20.995	20.6417	20.4286	20.9258
BAX	20.1241	19.8232	19.7475	19.7017	19.83	20.2215	19.6078	19.8445	19.8154
BCAP31	21.3712	19.2865	21.7794	21.4382	21.3192	21.7769	21.8986	21.1951	21.5127
BCAR1	16.6147	17.9576	18.2373	18.2703	17.8737	18.5949	18.6071	18.2169	18.0725
BCAS2	18.4085	17.9742	17.7658	18.3343	17.8923	17.7559	17.0736	17.6292	17.894
BCAT1	18.428	18.2567	18.1111	18.4565	18.5396	16.9864	18.636	18.7383	18.2032
BCAT2	18.4403	17.6929	18.3585	18.282	18.3808	18.6765	18.4619	18.5417	18.7998
BCCIP	20.706	20.6425	20.4951	20.6946	20.302	20.82	20.9671	20.5308	20.657
BCL2L13	18.5691	18.5403	18.2686	18.3921	18.34	18.2505	18.0666	18.0055	17.9578
BCLAF1	21.0649	21.3479	21.248	21.3944	21.0497	20.9907	20.8376	20.828	21.2287
BCS1L	18.7898	18.4878	18.49	18.3476	18.4758	18.3994	18.2534	18.533	17.9275
BID	17.606	17.2838	17.1439	17.1467	16.9514	16.5752	17.0051	16.8116	16.8867
BIN1	17.907	18.5932	18.0561	17.9913	18.1946	16.9043	17.685	18.0159	17.8544
BIRC6	17.1633	17.2474	17.2156	17.0191	16.7759	16.8538	16.6601	16.6742	16.3704
BLMH	19.9605	19.9865	19.5826	19.9517	19.752	19.2604	19.2858	19.6368	19.7666
BLVRA	21.3182	20.5493	20.9626	21.1424	21.0251	21.1822	21.5977	21.2708	21.2267
BLVRB	19.525	19.294	18.9509	19.3816	19.1029	19.5993	18.5133	19.2708	18.9903

BOP1	21.5288	20.9241	21.4763	21.6657	21.2928	20.974	21.242	21.453	21.3952
BPNT1	20.1982	19.855	20.4926	20.1563	20.163	20.0366	20.3677	20.1558	20.0261
BRAT1	17.7444	18.0602	18.223	18.0027	17.886	18.015	18.4714	17.8512	18.1517
BRD4	19.3689	18.7406	18.6752	18.8594	18.4551	18.7325	18.7062	18.6526	18.7627
BRD8	17.4908	17.7376	17.5522	17.6496	17.4928	17.4523	17.3934	17.7535	17.92
BRE	18.801	18.4289	18.1683	18.0987	18.0661	17.8757	18.1314	18.1668	17.9588
BRIX1	20.7273	20.7715	20.8855	20.5737	20.7495	20.5775	20.6719	20.5838	20.4047
BROX	19.0092	18.8788	19.17	18.9412	18.9824	18.7668	19.0168	18.8469	18.7013
BSG	21.4113	21.094	21.2928	21.4121	21.1492	21.2984	21.0156	21.147	21.4725
BTAF1	17.3324	17.3157	17.3949	17.316	17.4652	17.106	17.581	17.2528	17.3066
BTF3	21.3674	21.1277	21.2124	21.2771	21.0881	21.3028	20.8519	20.8336	20.9184
BTF3L4	19.4025	18.8549	19.3431	19.2096	19.3336	19.0561	19.2254	19.0082	19.4534
BUB3	21.4131	20.9713	21.6124	21.5882	21.0792	21.3605	21.5436	21.4452	21.505
BUD31	20.3459	19.9342	20.2605	20.1848	20.1771	20.6044	20.4413	19.9154	20.1915
BYSL	20.0093	19.9088	19.5935	19.5863	19.4483	19.6585	19.6513	19.7677	19.5329
BZW1	23.0825	23.0192	23.0537	22.8311	22.751	22.9494	22.901	22.8254	23.0151
BZW2	21.3262	21.4839	21.3901	21.2046	21.1665	21.4093	21.6538	21.2682	21.4075
C11orf68	19.5812	18.8673	19.2295	19.246	19.1137	19.5104	19.7523	19.5588	19.2785
C12orf10	21.6449	21.5249	21.6408	21.3944	21.386	21.7446	22.063	21.481	21.6216
C14orf166	21.1939	20.9941	21.0644	20.9363	20.9639	21.0214	21.1001	20.962	21.0446
C16orf58	15.6758	16.2964	16.3832	16.0143	15.9347	14.6129	14.8575	14.997	15.1849
C18orf8	16.8995	16.8755	16.7666	16.9287	16.3376	16.8736	16.5622	17.0296	16.986
C19orf10	23.0543	23.0903	23.2053	22.8652	23.0181	23.1008	23.4582	23.0547	23.2087
C1QBP	24.0855	23.8519	23.6787	24.0564	23.8827	23.5942	23.3331	23.605	24.113
C21orf59	16.6042	17.0883	16.7335	17.2582	16.942	15.6037	16.2912	16.7731	16.7516
C22orf28	21.872	20.612	21.6983	22.0245	21.683	21.7115	21.8373	21.6882	22.0493
C4orf27	17.7124	17.448	17.3551	17.5859	17.5566	17.3465	17.3541	17.4141	17.0236
C6orf211	16.3291	16.6994	16.0645	16.5697	16.4355	16.773	15.5103	16.3558	16.0426
C7orf50	19.2943	19.2544	18.8427	19.1076	18.7173	19.0924	18.6935	19.1776	19.0522
C9orf114	18.0794	17.6405	18.0706	17.7913	17.8526	17.7453	18.642	17.8442	17.4296
CAB39	19.3055	19.3286	19.4804	19.1521	19.148	19.3107	19.6805	19.2625	19.1579
CACYBP	24.0614	23.8502	23.9434	23.7566	23.7909	23.3959	23.8785	23.4633	23.5065
CAD	21.6723	20.9967	21.7166	21.6459	21.5751	21.3725	21.8397	21.6102	21.6452
CADM4	20.6388	20.6643	20.6449	19.9999	20.8325	19.8638	19.9767	20.0937	20.1597
CALB2	19.2012	18.9948	19.4272	19.0419	19.0649	20.14	20.1347	19.9462	19.9178
CALD1	21.4109	20.9375	21.2283	21.4176	21.3474	21.246	21.2017	21.1619	21.419
CALM1	21.2567	21.0892	21.2085	21.3445	21.3066	21.4131	22.1699	21.2942	21.8734
CALR	25.5656	25.2639	25.1745	25.3132	25.1759	25.2285	25.2907	25.3162	25.5371
CALU	21.6662	22.3626	22.2356	22.4888	22.2589	22.4593	22.9365	22.3991	23.111
CAMK1	16.5912	16.3738	17.2683	16.4192	16.9935	16.8505	17.3871	16.7888	16.1548
CAMK2D	19.4508	19.2164	19.4261	19.4154	19.0745	19.3342	19.7613	19.4438	19.4702
CAMK4	18.7843	18.3679	18.2799	18.4312	18.2915	18.105	18.6801	18.1747	18.009
CAMSAP2	15.7932	15.8967	15.9039	16.2875	15.0605	15.8731	15.9817	15.5313	15.5513
CAND1	22.7769	21.8429	22.3759	22.5332	22.2565	22.2678	22.4667	22.4534	22.447
CANX	24.6247	24.4305	23.9682	24.4895	24.0043	23.8118	23.7036	24.0256	24.2322
CAP1	23.2661	21.8978	23.0283	22.9601	22.7866	22.9305	22.9167	22.8656	22.908
CAP2	19.7109	19.3023	19.0047	19.4769	18.9485	18.8063	18.6998	18.9224	19.0781
CAPN1	21.1097	20.8243	20.854	20.8644	20.7009	20.7881	21.0428	20.9788	21.024
CAPN2	22.6837	22.3686	22.593	22.4428	22.2955	22.3997	22.7903	22.4616	22.4393
CAPNS1	21.7925	22.1738	22.0022	21.903	21.5514	21.8411	21.8524	21.7151	22.0548
CAPRIN1	22.1851	22.2275	22.1871	21.9514	21.8718	21.8827	22.3069	21.9009	22.288
CAPS	21.6169	21.8172	22.015	22.0038	21.9672	22.1421	22.3082	21.6741	22.0636
CAPZA1	22.9741	22.5865	22.6482	22.7331	22.5677	22.6917	22.6842	22.6482	22.7663
CAPZA2	21.7342	21.2935	21.0265	21.3442	21.1741	21.3362	21.315	21.2799	21.1878
CAPZB	23.1661	23.0678	23.1731	22.8532	22.7493	23.0158	23.2168	22.9362	23.1533
CARM1	19.0599	18.3081	18.931	18.7778	18.6422	18.9173	19.3874	18.8421	19.1547

CARS	20.9774	20.9219	21.0652	21.0086	20.8178	20.9027	20.8706	20.8749	21.0629
CARS2	18.799	18.6991	19.0167	18.7392	18.84	19.1563	19.5922	18.9493	19.0217
CASP2	17.2477	17.4412	17.3062	17.4684	17.2887	17.3909	17.3323	17.3127	17.1984
CASP3	19.7453	19.3955	19.3535	19.5531	19.0901	19.1408	18.273	18.9404	18.9371
CASP6	15.9248	16.7117	17.0195	16.3891	16.5809	16.8764	17.2339	16.9628	16.5625
CAST	18.4618	18.2362	18.6707	18.2225	18.2243	17.7499	17.8168	17.8419	17.7299
CAT	21.7159	21.6745	21.5859	21.6127	21.3747	21.8413	21.7319	21.4348	21.6444
CBFB	20.2325	20.1129	20.0799	20.3906	20.0563	20.6214	20.387	20.3019	20.7673
CBL	17.6039	17.3102	17.2302	17.409	16.9519	16.867	17.3501	16.7513	17.4321
CBR1	19.7718	19.4704	19.7855	19.3151	19.5009	19.7182	19.9667	19.4295	19.3172
CBR3	19.7462	19.4975	19.7031	19.653	19.4393	19.5805	19.3705	19.4033	19.52
CBX1	20.574	19.5816	20.3735	20.6034	20.442	20.071	20.4619	20.203	20.497
CBX3	23.053	22.5399	23.2748	23.2676	23.2714	23.4442	23.6656	23.0361	23.4098
CBX5	20.5474	20.6955	20.6799	20.6465	20.3899	20.704	20.7597	20.4178	20.7388
CCAR1	20.063	19.9819	20.1172	19.8791	19.7484	19.9069	20.1888	19.9683	20.0702
CCDC12	17.7747	16.1229	17.8126	17.829	17.8111	18.407	18.7018	18.0631	18.3232
CCDC124	19.3985	19.0821	19.0366	19.0249	18.8137	19.4116	19.4579	19.0613	19.3002
CCDC22	18.6967	18.318	18.7308	18.2208	18.4141	18.65	19.3368	18.7909	18.7867
CCDC47	19.6042	18.6197	19.1815	19.6574	19.2151	19.4755	19.0726	19.33	19.6438
CCDC51	18.8086	18.8151	18.8931	18.3948	18.7129	18.6498	18.8439	18.7673	18.5578
CCDC6	18.9282	18.498	18.2497	18.4402	18.2036	18.4158	18.4872	18.1784	18.6513
CCDC86	19.3559	18.664	18.5738	18.8299	18.4163	18.6748	18.8475	18.8758	19.2313
CCNA2	17.7209	17.1166	16.9483	16.9024	16.6059	16.8273	16.1902	16.6071	16.9853
CCNH	18.1646	17.7704	17.9269	17.844	17.6929	17.7977	18.1042	18.1387	18.1049
CCNK	19.5939	19.782	19.6939	19.4558	19.5951	19.7102	20.1907	20.0445	19.9165
CCT2	24.1367	22.8435	24.0127	23.9521	24.0267	24.0072	24.3667	23.8624	24.0072
CCT3	24.5751	24.3046	24.382	24.3486	24.2531	24.1738	24.2864	24.2528	24.2841
CCT4	23.8234	24.1491	23.8785	24.0806	23.9787	23.9022	24.2303	23.8727	24.0725
CCT5	24.2519	23.7411	23.9429	23.8868	23.9055	24.0727	24.1751	23.9806	24.0322
CCT6A	23.6592	23.7422	23.6308	23.551	23.4579	23.356	23.688	23.4323	23.573
CCT7	24.0471	23.9812	24.017	23.9205	23.7389	23.7916	23.9609	23.7952	23.9122
CCT8	23.9996	24.0202	23.9596	23.7239	23.6897	23.9195	24.0505	23.7701	23.9772
CD109	22.6508	21.818	21.9557	21.6376	21.6696	21.1916	21.955	21.6984	21.6158
CD151	20.4253	19.884	20.3945	20.29	20.2687	20.2783	20.8177	20.5049	20.7961
CD276	19.0717	18.4773	18.6409	18.4881	18.8556	18.4285	18.7626	18.7862	18.6156
CD2AP	19.6283	19.6397	18.935	19.1304	18.6102	18.5844	18.9534	19.5107	19.2404
CD2BP2	17.3496	17.0794	17.1015	17.511	17.2291	16.9345	17.2323	17.4045	17.3485
CD3EAP	18.3997	18.3844	18.1172	18.5124	18.0391	18.3637	18.1005	18.1814	18.458
CD44	23.4716	23.2396	23.3377	23.179	23.1927	23.1406	23.2804	23.0343	23.1898
CD59	21.7782	20.6754	21.6905	21.7391	21.723	22.2881	22.5066	21.7539	22.3847
CD63	20.5202	16.0829	20.3759	20.7024	20.3834	20.668	20.4856	20.2606	20.7966
CD74	21.0895	21.4431	20.7825	20.7925	20.5494	20.8577	20.8569	20.7633	21.1121
CD81	20.7433	20.2881	20.3485	20.2564	20.2059	20.3308	20.5823	20.6317	20.4703
CDC123	17.5434	17.8631	17.9568	17.8259	17.7689	16.0831	17.6071	17.6916	17.0601
CDC16	18.4748	18.4204	18.3612	17.9747	18.0144	17.7325	17.664	18.1359	17.5847
CDC20	19.0502	19.2801	18.9185	19.1106	18.9398	19.3305	19.3749	19.2371	19.3376
CDC23	18.7378	18.829	18.6075	18.433	18.5184	18.3026	18.2442	18.6209	18.5175
CDC27	18.581	17.8158	18.3143	18.2612	18.1196	17.8714	18.2664	18.2099	18.324
CDC37	22.2731	22.1798	22.0644	22.2692	21.8166	22.3947	21.8357	21.9396	22.1625
CDC40	17.602	17.6355	17.6831	17.4314	17.9022	17.0215	17.8868	17.1947	17.4551
CDC42	21.9763	22.1727	21.9863	21.9617	22.1285	21.6389	21.5279	21.7771	21.6791
CDC45	18.0603	18.8808	18.9897	19.313	18.0571	18.8265	18.5118	18.5045	18.4495
CDC5L	21.2946	20.9672	21.0491	21.0676	20.912	21.127	21.3603	20.9802	21.28
CDC73	20.4498	20.3871	20.6651	20.1338	20.1567	20.0143	20.3361	20.0095	20.0167
CDCA8	17.7189	17.5232	17.8052	17.9859	17.1472	17.7648	16.9672	17.2096	17.4191
CDH13	18.5506	18.4536	18.1308	18.6703	18.5999	18.1487	18.9251	18.8945	18.7691

CDH2	19.9299	20.0767	20.3136	20.2955	20.2446	20.1459	19.9042	19.8464	20.0376
CDIPT	17.5609	18.1493	18.067	18.1492	17.6394	17.5441	16.9949	17.6125	17.54
CDK1	21.329	21.3081	21.6059	21.4251	21.249	21.2875	21.451	21.3027	21.4493
CDK11B	19.5498	20.0696	20.0158	19.8153	19.6856	19.517	20.0258	19.577	19.8705
CDK12	17.9623	17.9407	17.8823	17.8711	17.7766	17.9377	17.9729	18.1555	17.9936
CDK2	20.2112	19.9467	20.1652	19.6948	19.9653	20.2328	20.5284	20.019	19.8716
CDK5	20.3675	20.6386	20.441	20.0831	20.3108	20.581	20.9076	20.4037	20.4386
CDK5RAP1	16.5659	16.6577	16.149	16.264	16.3848	16.3856	16.4549	16.4078	16.1195
CDK5RAP3	17.8529	17.9967	17.8165	17.788	17.8624	17.618	17.8674	17.5874	17.4495
CDK6	21.1755	21.2331	21.0969	21.2582	20.7528	20.6164	20.7587	20.6475	21.0372
CDK9	18.6985	19.0422	18.9995	18.8061	18.5157	18.9546	19.0174	18.8517	18.8586
CDV3	19.6193	19.0458	19.0384	19.7547	19.1191	19.5898	19.6701	19.4104	19.4933
CEBPZ	19.1498	18.4212	18.684	18.5908	18.5249	18.2295	18.6593	18.5951	18.7483
CECR5	19.5763	22.7842	18.9039	19.2732	19.8555	19.2871	19.2614	19.5246	19.7755
CELF1	20.4515	20.5858	20.632	20.6999	20.4937	20.8282	20.6211	20.6039	20.7568
CEP170	18.1663	24.4873	18.8759	19.2358	18.7863	18.904	18.8428	18.7261	19.059
CERS2	21.6595	21.4857	21.448	21.5564	21.1718	21.4984	21.6656	21.3564	21.425
CFL1	26.125	26.0129	26.1969	26.1406	26.1651	26.4908	26.3481	25.9404	25.8393
CFL2	19.5244	19.2378	19.5535	19.6858	19.5423	19.8858	19.5757	19.3053	19.4182
CGGBP1	20.6632	20.1571	20.9669	20.3986	20.698	21.0984	21.4285	20.7376	20.7645
CHAF1A	18.3016	18.1648	18.4267	18.1812	17.9576	17.9306	18.3329	17.9411	18.1868
CHAMP1	20.0795	19.9504	19.9321	19.8855	19.7978	20.1493	20.2482	20.0905	20.0932
CHCHD2	19.6893	19.1113	19.2148	19.2721	19.0854	19.4617	20.0188	19.5596	19.597
CHCHD3	21.2356	21.7271	20.953	21.1145	20.9727	21.3264	21.3471	21.1816	21.5137
CHD1	18.2634	17.8415	17.7478	18.0521	17.8083	17.7107	17.7955	18.0045	18.2583
CHD1L	18.28	18.0685	18.1844	18.0483	17.9632	17.9543	18.3385	18.194	18.09
CHD4	20.8984	20.8956	20.7977	20.6438	20.7143	20.6744	21.2277	20.7035	20.8771
CHERP	19.251	19.3499	19.5398	19.5211	19.1586	18.9531	19.3734	19.3207	19.6653
CHID1	16.5935	17.3669	17.5039	17.6313	17.3643	16.4377	16.1231	17.6779	17.4618
CHMP3	18.1976	18.1351	17.4517	17.8192	17.725	17.7953	17.9217	18.1103	17.357
CHMP4B	18.677	17.8082	18.2895	18.6072	18.3684	18.5344	19.0237	18.5628	18.8275
CHMP5	17.5122	17.1422	16.8913	17.9256	16.4881	17.992	17.3986	17.2107	17.9552
CHMP7	17.2609	17.1484	17.3422	17.0348	16.8419	16.6076	16.3633	17.2108	17.348
CHORDC1	19.8912	18.9715	19.6427	19.7893	19.6775	19.9157	19.5927	19.5709	19.9156
CHP1	20.3244	20.1585	20.2245	19.9624	20.1163	20.3497	20.9169	20.4359	20.651
CIAO1	20.0832	20.0319	19.7851	19.8838	19.8115	19.8508	20.1418	19.9875	20.1294
CIAPIN1	20.3011	20.7141	20.5695	20.665	20.3221	20.8863	21.1723	20.5924	20.827
CIRBP	19.9068	19.5554	19.5471	19.5995	19.55	19.7299	19.45	19.6675	19.8938
CIRH1A	20.1018	20.4643	20.1144	20.0822	20.0255	20.0866	20.2735	20.1984	20.5525
CISD2	20.5739	20.6528	20.7857	20.3881	20.4633	20.6233	20.9566	20.5555	20.7659
CIT	18.1096	17.3942	17.879	18.203	18.2497	18.0873	18.2018	17.7165	18.092
CKAP4	23.3747	23.1226	23.2647	22.8195	23.118	22.8476	23.063	22.7965	22.7421
CKAP5	19.0063	19.2955	19.2558	19.0674	19.0416	18.7298	19.4095	19.3271	19.1948
CLASP1	20.6187	20.6947	20.5403	20.4356	20.4323	20.1293	20.6506	20.5278	20.8023
CLASP2	18.1704	17.5876	17.4048	17.2379	17.1937	17.4896	17.5219	17.5814	17.648
CLCC1	17.35	17.5112	16.4194	17.5897	16.928	15.3319	15.506	16.9728	16.9795
CLIC1	23.3698	23.2658	23.2625	23.1523	23.1819	23.3457	23.7232	23.1382	23.4008
CLIC4	22.2728	22.9944	22.1855	22.1032	22.0534	21.8861	22.1985	21.8212	21.9199
CLINT1	19.653	19.8771	19.92	20.4008	20.102	20.1041	19.6801	19.7168	19.978
CLIP1	17.4878	17.7054	17.5298	17.4959	17.461	17.181	17.2439	17.7815	17.9576
CLMN	17.9132	18.1656	17.9579	17.8293	17.8894	17.4973	17.4597	17.79	17.747
CLNS1A	18.7144	19.6473	19.4964	19.8227	19.7619	18.2336	18.2242	19.6072	19.5166
CLPB	20.5703	20.2694	20.3739	20.5842	20.4268	20.6234	20.9431	20.5525	20.7637
CLPTM1	18.4809	15.6826	18.5857	18.5969	18.7369	18.7198	18.6577	18.2392	18.4165
CLPTM1L	18.2259	18.3256	17.9614	18.0158	18.057	16.2115	17.3064	17.4635	16.8763
CLPX	20.0659	20.142	20.2163	20.0764	19.7286	20.0478	20.4909	19.9999	20.174

CLTA	19.0438	18.9167	19.534	19.4337	19.137	19.6964	20.1503	19.1476	19.7978
CLTC	24.7753	24.3812	24.6702	24.5434	24.3069	24.2738	24.6514	24.469	24.5244
CLUH	20.6078	20.2583	20.2186	20.018	19.9635	19.9102	20.2646	19.9637	20.1323
CMAS	20.2722	20.0097	19.9912	20.0035	19.8601	19.9179	20.3983	20.0861	20.3167
CMBL	16.1501	16.2346	15.7891	16.5348	16.4577	15.0123	15.6204	17.256	17.6774
CMPK1	21.5063	21.4105	21.5914	21.4024	21.4097	21.6166	21.6487	21.4874	21.3518
CMSS1	18.0929	18.0327	18.0044	17.8453	17.7654	19.3721	18.1632	17.8671	17.9292
CMTR1	17.8679	17.5876	17.8967	17.6651	17.7233	17.5552	18.0754	17.8893	17.7147
CNBP	19.6441	17.5082	19.5045	19.5615	19.4897	20.089	19.9344	19.9794	20.186
CNDP2	21.705	21.4602	21.4025	21.2842	21.258	21.4879	21.7136	21.3589	21.4192
CNN2	20.4418	19.679	19.7641	19.7163	19.3689	19.5649	18.5803	19.2513	19.1334
CNN3	22.3831	22.3943	22.2369	22.3962	22.1692	22.0621	21.9306	21.9582	22.0231
CNOT1	18.2465	18.3188	18.2724	18.208	18.2897	18.2845	18.6141	18.3983	18.3597
CNP	21.6716	21.9884	21.7939	21.6829	21.6224	21.9453	22.1208	21.6169	21.9552
CNPY2	22.1429	21.9174	22.1773	22.326	22.3102	22.5803	22.6363	22.1377	22.4242
CNPY3	20.0589	22.5908	19.8053	19.7562	19.661	19.6398	19.6572	19.5287	19.7783
CNTNAP1	17.6502	17.8714	17.91	17.4279	17.9333	17.5794	18.7778	17.831	18.1792
COASY	18.5502	18.3996	18.3231	18.1908	18.3556	18.1743	18.2077	18.0221	18.1055
COG4	17.9486	18.4963	18.9978	18.4342	18.8399	18.0319	19.1881	17.8366	18.642
COIL	17.6913	17.9071	17.9852	17.8648	17.8783	17.8312	18.3096	17.757	17.7976
COL4A3BP	17.5871	17.4391	18.1554	17.8889	18.1028	18.457	18.1968	17.0481	16.5691
COLGALT1	20.4966	20.2508	20.1997	19.9207	19.9441	19.5641	19.7353	19.6917	19.8188
COMMD3	18.9548	19.0164	18.7821	18.5964	18.477	19.2736	18.6199	19.0455	18.7221
COMMD4	18.8326	19.0172	18.2527	19.0092	18.4638	17.6666	17.6695	18.4869	18.5305
COMT	21.0926	24.7863	20.4211	20.8137	20.694	22.5043	20.6957	20.8853	20.9687
COPA	22.5795	22.4901	22.6457	22.5702	22.4298	22.566	23.0037	22.5299	22.7146
COPB1	22.098	22.0066	22.2998	22.1681	21.8223	22.067	22.4761	22.2056	22.2709
COPB2	21.8149	21.608	21.6789	21.8004	21.5941	21.568	22.1604	21.8358	22.0328
COPE	21.4134	21.3686	21.4188	21.2382	21.0413	21.2074	21.4121	21.197	21.3544
COPG1	21.3355	21.0474	21.1115	21.1585	20.9209	21.1405	21.4044	21.1275	21.2058
COPG2	20.7151	20.5971	20.4727	20.315	20.1475	20.2443	20.8472	20.4336	20.5572
COPS2	21.0928	20.7943	20.7077	20.666	20.512	20.7594	20.8621	20.6286	20.8051
COPS3	20.7474	20.5847	20.5713	20.4753	20.5921	20.5258	20.0664	20.6371	20.6434
COPS4	20.9738	20.6644	20.5997	20.814	20.6637	20.8195	20.9999	20.6927	20.5213
COPS5	20.8911	20.9046	20.8649	20.7841	20.7379	20.7379	21.2317	20.833	20.9102
COPS6	20.9115	20.9285	20.7826	20.4909	20.557	20.9712	21.1357	20.7265	20.9716
COPS7A	19.146	19.2386	18.921	19.24	18.744	18.5929	18.8527	18.8477	18.9505
COPS7B	17.3256	19.0428	17.3066	17.675	17.29	18.0667	18.3419	18.0284	18.4429
COPS8	20.3401	20.3069	20.2772	20.1696	20.2314	20.6375	20.397	20.2376	20.3212
COPZ1	20.96	19.6054	20.7103	20.2682	20.6592	21.1128	20.9003	20.8227	20.8559
CORO1A	16.9226	16.7769	16.3199	16.7196	16.3527	16.5583	15.915	16.7523	16.9004
CORO1B	21.1106	20.9178	21.0933	21.0977	21.0395	21.2334	21.1522	20.852	20.9713
CORO1C	22.7	22.5653	22.5474	22.5337	22.5447	22.7454	22.4928	22.826	22.6977
COTL1	23.8698	23.8866	23.9469	23.8849	23.673	24.0958	24.103	23.9706	23.9875
COX4I1	22.4858	22.1618	22.3537	22.5386	22.1767	22.7079	22.6554	22.6531	23.0939
COX5A	19.3354	19.5166	19.1444	19.2274	19.3561	19.9302	19.5467	19.5387	19.4943
COX5B	20.5536	20.9225	20.4564	20.7916	20.2372	20.7193	21.0552	20.5844	21.1914
COX6B1	18.3387	18.5332	18.342	18.9089	18.6458	18.8682	19.2067	18.7371	19.1946
CPD	16.832	17.0402	16.8755	17.0757	16.9424	16.3506	16.5103	16.4623	16.8086
CPNE1	22.8097	22.6211	22.5713	22.6714	22.3979	22.3032	22.4458	22.2619	22.3641
CPNE3	21.3575	21.5317	21.3114	21.4291	21.2405	21.3558	21.5886	21.3564	21.5463
CPOX	20.6868	20.6154	20.5978	20.4725	20.4099	19.9325	20.5378	19.957	20.1397
CPSF1	18.9074	19.0481	19.0509	19.0034	18.7487	18.8682	19.0495	18.9767	19.1489
CPSF2	19.2344	19.1422	19.551	18.6143	19.0698	19.4687	19.6468	19.502	19.7116
CPSF3	20.1594	20.2565	20.0351	20.1356	20.1679	20.0466	20.5962	20.2228	20.1935
CPSF3L	17.9484	17.8907	17.9479	17.7668	17.9914	17.8784	18.3971	17.8494	17.9124

CPSF6	22.0354	21.9215	21.8545	21.9164	21.726	21.7693	22.1073	21.8453	22.1909
CPSF7	20.0857	19.8103	20.1671	20.0789	19.9638	20.2515	20.5398	20.1283	20.2008
CPT1A	21.6252	21.6049	21.8569	21.1835	21.5509	21.7119	22.2295	21.716	21.7202
CPT2	19.3039	19.255	19.2931	19.2874	19.161	19.3015	19.658	19.3737	19.722
CRELD2	17.5645	16.9569	17.95	17.6081	17.9964	17.0393	17.6716	17.9185	17.9418
CRIP2	19.4069	18.6754	18.8052	18.8192	18.9275	19.5027	19.3131	18.9043	18.9881
CRK	21.0262	21.1334	20.7411	20.9627	20.8618	19.3372	20.3518	20.8767	20.7987
CRKL	20.1017	19.8954	19.8089	19.7781	19.864	19.646	19.9555	19.8108	19.888
CRNKL1	19.2765	19.2149	19.2216	19.0376	19.1684	19.1011	19.411	19.1907	19.3083
CRTAP	20.4792	20.3149	20.9473	20.7894	20.6405	20.8179	21.1167	20.7421	21.0582
CRYZ	21.8637	21.7225	21.5868	21.6032	21.5446	21.7296	21.8655	21.5946	21.6563
CS	23.3755	23.3482	23.5172	23.1659	23.1652	23.5075	24.0742	23.5777	23.7512
CSDE1	21.8821	21.7823	21.9282	21.7297	21.6313	21.5275	22.0728	21.7453	21.9125
CSE1L	23.8412	23.8079	23.8903	23.7649	23.4482	23.5989	23.9616	23.7466	23.8596
CSK	19.1073	19.2484	19.2187	19.0223	18.8972	18.405	19.2385	18.9686	19.0957
CSNK1A1	19.0503	18.9833	19.2145	18.8964	18.9791	18.6828	18.8744	18.7947	18.7915
CSNK1D	16.2454	18.7675	16.7808	15.1463	16.3545	16.6714	16.728	17.0996	15.7162
CSNK2A1	22.0246	21.7356	21.8203	21.7134	21.5601	21.8871	22.2916	21.8744	21.9971
CSNK2A2	19.8931	20.078	20.0051	19.9649	19.8669	20.1718	20.1497	20.0106	20.0789
CSNK2B	21.4795	20.791	20.9922	21.2846	20.8834	21.1995	21.0851	21.3102	21.5867
CSPG4	21.8003	21.0433	21.4778	21.7899	21.628	22.2635	22.507	21.8847	22.1193
CSRP1	23.1947	22.354	22.7512	22.8878	22.6634	23.0839	23.4039	23.1066	23.228
CSRP2	21.3492	21.2343	21.564	21.1446	21.0989	21.1875	21.0063	20.8814	20.8911
CSTB	19.6818	19.7901	20.0705	20.1888	20.1267	20.5474	20.726	20.1631	20.4797
CSTF1	20.5607	20.4766	20.7021	20.4317	20.5571	20.4531	20.8678	20.6076	20.7207
CSTF2	20.8406	20.4546	20.721	20.8273	20.7587	20.8324	21.2907	20.7398	20.9992
CSTF3	19.7366	19.9639	19.9469	19.9137	19.5297	19.4342	19.7034	19.6521	19.9597
CTBP1	20.6768	20.2459	20.244	20.3332	20.1199	20.1966	20.3429	20.3225	20.4464
CTBP2	20.2995	19.6679	19.6068	19.7405	19.5423	19.5803	20.0029	19.6626	19.8674
CTDP1	17.9332	17.8497	18.2497	18.0107	17.7444	18.0595	18.5592	18.0809	18.2826
CTHRC1	18.8816	18.9254	19.3323	18.9554	18.8104	19.0253	19.4428	19.041	18.9533
CTNNA1	21.8443	21.9586	21.5757	21.5898	21.5326	21.6326	21.773	21.6837	21.7617
CTNNAL1	16.0792	16.1004	16.2961	16.2492	16.3402	14.7809	15.5852	15.6663	14.8058
CTNNB1	21.1118	19.7797	20.5781	21.0159	20.6926	20.8915	21.0713	20.9318	20.9784
CTNNBL1	20.3069	20.2599	20.155	20.0094	20.1452	20.1839	20.3017	20.1393	20.1622
CTNND1	22.107	21.994	22.1534	22.1028	22.0842	22.364	22.6409	22.0169	22.182
CTPS1	22.5205	22.2884	22.3479	22.3615	22.1099	22.4012	22.7913	22.3779	22.5897
CTR9	19.8418	19.4082	19.5536	19.4387	19.5205	19.5455	19.8818	19.5894	19.253
CTSA	21.5014	21.4671	21.4245	21.209	21.2076	21.2612	21.6878	21.6234	21.6757
CTSB	21.2389	21.0807	21.2014	21.0998	20.7505	20.8698	21.0057	20.8547	21.0869
CTSC	20.4461	20.2432	20.4641	20.5716	20.5473	20.5371	20.4943	20.5806	20.6764
CTSD	21.9179	22.4555	21.9634	22.0533	21.7246	22.0579	22.5687	22.2489	22.6884
CTTN	21.1468	20.8774	20.9687	21.181	21.0146	21.1748	21.0471	21.1629	21.5065
CUL1	19.6852	19.4807	19.8951	19.8931	19.673	19.9007	19.9867	19.7016	19.775
CUL2	19.3661	18.9047	19.503	19.6078	19.4905	19.177	19.3153	19.2468	19.3344
CUL3	19.0679	19.4059	19.1198	19.2007	19.0855	19.0958	19.3606	19.2499	19.4566
CUL4A	17.6897	18.5429	17.7146	17.9211	17.7382	17.3968	18.4769	18.315	18.4609
CUTA	19.6904	19.7059	19.6939	19.826	19.8606	19.8544	19.5982	19.6942	20.2502
CWF19L1	18.8576	18.9412	19.0275	18.8653	18.8922	18.9321	19.3292	19.0589	18.9757
CYB5A	19.1295	19.5119	18.557	19.109	18.8975	19.2591	18.6735	18.4938	18.9599
CYB5B	20.8171	20.2435	20.4334	20.787	20.4304	20.8475	20.413	20.5474	20.4854
CYB5R1	18.5566	18.6822	18.246	18.5787	18.3032	18.3087	18.2487	18.3289	18.2848
CYB5R2	21.6154	21.2101	21.283	21.435	21.2487	22.0053	21.66	21.4	21.3755
CYB5R3	23.5279	22.9526	22.7992	22.6712	22.5299	22.6683	22.6008	22.7664	22.9946
CYC1	22.175	22.1193	22.2649	21.9459	22.2334	22.34	22.8612	22.3786	22.2855
CYCS	22.034	22.1308	22.0481	21.9778	21.889	22.2138	22.1886	22.0341	21.9908

CYFIP1	20.3979	20.5283	20.5723	20.484	20.5488	20.3267	20.7946	20.4632	20.7419
CYP51A1	18.684	17.7111	18.7989	18.8958	18.5373	19.2834	19.3599	18.7341	19.1696
DAG1	19.2428	18.9085	18.9177	19.0559	18.9558	18.7589	18.4214	18.498	18.6402
DAK	18.4212	18.3389	18.299	17.9305	18.0196	18.1011	18.4969	17.9948	17.8351
DAP3	20.3171	20.4148	20.2568	20.1369	20.1213	20.3952	20.6537	20.2034	20.552
DARS	23.0412	22.8078	22.7661	22.6909	22.7504	22.7363	22.9439	22.6822	22.8104
DARS2	21.2938	21.0737	21.0842	21.1525	21.0957	21.0512	20.9977	20.8601	20.8922
DAXX	19.3146	18.9062	19.0283	19.0878	18.9876	18.6817	18.9656	18.7186	18.5934
DAZAP1	21.9394	21.5638	21.6746	21.5066	20.7632	21.2017	21.598	21.7475	21.7544
DBN1	20.7751	20.8929	20.7011	20.8978	20.5656	20.228	20.8558	20.7092	20.8966
DBNL	20.8442	20.6477	20.4573	20.4811	20.2732	20.5322	20.4762	20.4705	20.7863
DCBLD2	18.6654	18.3053	18.6405	18.5289	18.3635	18.2538	18.561	18.3724	18.3748
DCK	19.1798	19.2206	19.2007	18.8841	18.84	18.972	19.0027	18.4869	18.2214
DCP1A	18.2481	18.4358	18.1113	17.9356	17.7729	17.8142	18.0042	17.8824	17.9212
DCPS	20.0657	19.9579	20.2832	19.861	19.9181	19.9612	20.2529	19.7631	19.8439
DCTN1	20.2293	19.9328	20.1294	20.3782	20.044	19.9921	20.5781	20.3476	20.3857
DCTN2	20.36	20.2123	20.3856	20.2369	20.1033	20.3227	20.5994	20.3488	20.338
DCTN3	18.2215	19.6136	18.0622	18.2692	18.1914	18.3322	18.0316	18.243	18.0903
DCTN4	18.793	18.2909	18.4357	18.2615	18.3429	18.1143	18.8062	18.3556	17.9002
DCUN1D1	18.2058	18.4843	18.3686	18.4915	18.1328	18.5566	17.9799	18.2374	18.2797
DCXR	20.9603	20.9119	20.9963	20.97	20.8151	20.5689	20.9232	20.7171	20.8018
DDB1	23.003	22.9797	22.9664	22.8069	22.7755	22.5952	22.8834	22.7435	23.0094
DDB2	20.5474	20.3853	20.4124	20.3086	20.1639	20.1101	20.3358	20.1358	20.3286
DDOST	23.1769	23.1328	23.1006	22.8328	22.8172	23.1109	23.3744	23.0175	23.0292
DDRGK1	17.91	16.7298	17.9674	17.7362	17.889	18.3063	17.927	17.7717	17.8905
DDT	21.0162	20.781	20.8266	21.0979	20.8354	21.4943	21.9656	21.3621	21.7052
DDX1	22.1902	22.3543	22.3217	22.1091	22.1359	22.2843	22.4612	22.1435	22.2915
DDX17	22.9068	22.7777	22.7295	22.8109	22.5253	22.6517	23.0849	22.7389	22.8319
DDX18	21.0226	20.8472	20.8889	20.8072	20.7499	20.5081	20.7538	20.6189	20.6104
DDX19A	22.159	20.7731	22.6238	22.5193	22.3417	22.4506	22.1051	22.0847	22.5556
DDX20	19.4312	19.0129	19.1467	19.1211	18.9835	19.0891	19.3028	19.1053	19.267
DDX21	21.7694	22.1283	22.2541	21.8376	21.7316	21.754	22.1474	21.6587	21.747
DDX23	20.4641	20.4616	20.585	20.353	20.2134	20.3646	20.6931	20.336	20.1914
DDX24	17.1639	17.4631	17.3629	17.1161	17.084	17.2442	17.4265	16.9774	16.9433
DDX27	21.2496	21.3915	21.4388	21.2164	21.0319	20.977	21.3622	21.175	21.2223
DDX39A	22.0913	21.6828	21.699	21.8357	21.5699	21.648	21.3397	21.6183	21.5019
DDX39B	22.798	22.2572	22.4006	22.5307	22.1941	22.4197	22.0244	22.4042	22.2345
DDX3X	22.9172	23.1636	23.572	23.1368	23.2114	23.2758	23.6185	23.2939	23.3098
DDX41	19.6308	19.3225	19.5995	19.0056	19.175	19.541	19.5475	19.4768	19.1546
DDX42	21.2572	21.4264	21.1208	21.1709	21.0924	20.9869	21.1066	20.9775	21.2895
DDX46	22.6821	22.7039	22.6892	22.5112	22.4532	22.658	22.9705	22.5907	22.7104
DDX47	19.9319	19.8345	19.8235	19.833	19.6663	19.1736	19.6266	19.7272	19.4984
DDX5	24.1782	24.0876	24.1516	24.073	23.9622	23.9947	24.4161	24.0847	24.1996
DDX50	17.8354	16.6028	18.3955	18.3466	17.8546	18.2466	18.5624	17.9536	17.6993
DDX51	18.0512	19.3531	17.8051	17.8009	17.8015	18.0063	18.2993	17.8043	17.882
DDX54	18.9528	18.6803	18.5708	18.8282	18.78	19.3307	19.4658	18.8245	18.856
DDX56	19.8335	20.3038	20.2479	20.0622	19.966	19.9796	20.5681	20.1429	20.0393
DDX6	21.6688	21.435	21.3578	21.1779	21.2031	21.5587	21.8067	21.6545	21.6624
DECR2	13.2773	14.2982	14.4842	15.607	13.282	13.1634	?	11.4362	13.7922
DEK	21.3187	21.2139	21.0897	21.2011	20.8583	21.2231	20.9242	21.1289	21.3978
DEPDC7	19.0763	18.8701	18.8599	18.7707	18.8332	18.9356	19.2462	19.0324	18.7724
DERA	18.4484	18.1198	18.2671	18.5473	18.1809	17.9612	18.4984	18.3586	18.244
DFFA	19.1364	18.631	18.5358	18.5566	18.6319	18.6399	18.8107	18.5703	18.4117
DFNA5	18.5156	18.1893	18.6449	18.2067	18.2827	18.2747	18.9041	18.462	18.4586
DGCR14	17.7165	18.8564	18.3654	18.6658	18.2821	18.4209	18.7531	18.4086	18.4817
DHCR24	20.3082	20.5153	20.3894	20.2949	20.3209	20.6568	20.8203	20.7735	20.8038

DHCR7	19.0876	20.0462	19.0916	19.1847	19.0702	18.8344	19.2097	18.7611	19.145
DHFR	20.3063	19.0642	20.274	20.2414	20.0226	20.2135	20.4125	19.8378	20.1912
DHPS	19.029	18.602	18.7062	18.5335	18.4665	19.2276	19.1606	19.3209	19.5017
DHRS7	18.8713	19.1805	18.8323	18.8088	18.8624	19.073	19.2311	18.9353	18.9398
DHTKD1	16.8654	17.8236	17.6734	18.1261	17.6838	16.6615	17.1925	16.6533	17.0814
DHX15	22.6342	22.8055	23.0091	22.6664	22.6236	22.413	22.9342	22.6455	22.7779
DHX16	19.4442	19.1662	19.1643	19.0521	19.2934	19.1854	19.3259	19.2365	19.2888
DHX29	16.974	17.1748	17.2345	17.0322	17.0993	16.7086	17.6126	16.9853	16.8708
DHX30	20.2214	20.3009	20.2893	20.1386	20.0515	20.0394	20.4248	19.9492	20.0627
DHX36	18.4547	18.5665	18.5058	18.1495	18.5166	18.2256	18.4655	18.4769	18.0613
DHX38	18.8971	18.4069	18.4023	18.4374	18.3746	17.1902	18.3048	18.3059	18.1904
DHX40	16.3211	16.5269	15.595	16.5612	16.9165	6.86874	14.6042	16.2372	14.7951
DHX9	24.4528	24.3744	24.2941	24.1702	24.0305	23.9987	24.502	24.1348	24.4779
DIABLO	23.2136	23.4705	23.4874	23.367	23.1261	23.4652	23.6539	23.3197	23.5717
DIAPH1	20.427	20.0757	20.1803	20.0243	19.9101	19.9308	20.3697	19.9955	20.0228
DIDO1	18.7022	18.9206	18.5008	18.2888	18.899	18.8029	18.9252	18.621	18.8644
DIEXF	18.0188	18.6794	18.8297	18.4477	18.622	17.9543	18.7841	18.441	18.5602
DIMT1	18.7254	18.368	18.4065	17.8101	18.1768	17.8791	18.7021	18.1299	18.2727
DIP2B	17.4057	17.9351	17.8609	17.5846	17.3061	16.3251	17.6629	17.4113	17.5411
DIS3	20.0738	20.4658	20.4255	20.1647	20.0598	19.8799	20.313	20.1755	20.3662
DKC1	19.7085	19.5224	19.8561	19.8855	19.7127	19.3994	19.7465	19.4873	19.4436
DLAT	18.2966	17.3819	18.2607	19.0287	18.4367	18.5013	18.7508	18.4054	18.5301
DLD	21.8948	21.6476	21.7664	21.6514	21.6134	21.6722	21.9794	21.8881	21.9473
DLST	21.4002	21.796	21.6838	21.5129	21.6517	21.7962	21.9208	21.7006	21.9004
DNAJA1	22.1129	21.4141	22.1434	21.9456	21.7705	22.0038	22.1299	21.9534	21.9644
DNAJA2	20.8273	20.5183	20.8348	20.5343	20.6072	21.0269	21.8387	20.7597	20.8074
DNAJA3	20.3629	20.2112	20.2289	20.0239	20.0014	20.5358	20.7366	20.4729	20.4169
DNAJB1	21.7269	21.7921	21.8344	21.6763	21.5022	22.1274	22.3738	21.8317	22.0952
DNAJB11	21.1884	20.6849	20.8907	20.6158	20.7411	21.0176	20.8855	21.0058	21.0676
DNAJB6	19.5448	19.2615	19.1997	19.1591	18.9578	19.3158	19.5348	19.2258	19.535
DNAJC10	15.7456	16.9146	16.1093	16.0925	16.4647	15.4271	16.0735	16.1787	16.1004
DNAJC11	19.0449	18.9864	18.9563	18.743	18.786	18.6145	18.7014	18.675	18.9693
DNAJC13	18.2738	17.7214	17.8607	17.6636	17.8058	17.1661	18.1879	17.8996	17.7777
DNAJC3	19.0964	19.6066	19.1992	19.1035	19.0632	19.2145	19.082	19.2034	19.138
DNAJC5	18.4325	17.8006	17.8431	18.2173	17.9196	17.1275	17.3668	17.8696	17.8967
DNAJC7	20.5084	20.6483	20.3636	20.3019	20.0883	20.0851	20.2549	20.2025	20.4857
DNAJC8	21.5225	21.9982	21.3739	21.6799	21.5026	21.7982	22.0039	21.6239	21.8819
DNAJC9	20.2695	21.7739	20.0834	20.2162	20.1339	21.3097	20.1254	20.1341	20.1091
DNM1L	21.1114	21.0211	21.0683	20.831	20.7739	21.083	20.8568	20.7881	20.8634
DNM2	19.7035	19.4814	19.5514	19.5819	19.055	19.2699	19.6974	19.6414	19.6294
DNMBP	13.8737	14.0206	14.193	12.9598	14.0021	14.4307	13.8112	14.7757	13.3501
DNMT1	19.7075	19.5417	19.6831	19.5242	19.2047	19.3527	19.5011	19.299	19.3754
DNTTIP2	19.4242	18.6617	18.9472	18.6008	18.9674	19.3428	19.5271	19.2176	18.8687
DOCK5	18.6561	19.2255	18.8924	19.0124	18.8596	18.4827	18.7815	18.958	19.1461
DOCK7	18.9456	18.9845	19.0277	18.918	18.9031	18.1559	18.828	18.5863	18.8091
DOK1	17.6942	17.4788	17.9998	17.6463	17.8565	17.8741	17.6187	18.0075	17.3834
DPH5	19.175	19.2579	19.1719	19.2269	19.4845	19.818	19.08	19.4333	18.7358
DPM1	21.6696	21.4167	21.2619	21.1738	21.2068	21.0001	21.3066	21.1637	21.2268
DPP3	21.1763	21.7206	20.8715	20.678	20.6562	20.8087	21.1442	20.791	20.918
DPYSL2	23.6065	23.2054	23.5455	23.2267	23.3114	23.085	23.3829	23.0433	23.0424
DPYSL3	21.6944	21.872	21.8062	21.7703	21.4736	22.0633	22.2589	21.9477	22.1248
DRAP1	19.22	19.5218	19.3118	19.4381	19.2031	19.1671	19.4943	19.3183	19.5347
DRG1	20.1668	20.444	20.4828	20.218	20.3695	20.4092	20.8564	20.4198	20.2743
DSG2	19.2053	18.7872	18.8597	18.9047	18.7025	17.7544	18.5339	18.3973	18.5468
DSP	18.9143	?	13.3494	14.0956	12.8203	4.54654	7.6474	11.6136	11.8312
DSTN	22.4557	21.1749	22.4561	22.5323	22.217	22.6017	22.4031	22.3261	22.4992

DTD1	21.0373	20.8565	21.0448	20.7886	20.5561	20.8092	21.0138	21.2802	21.1355
DTYMK	21.2908	21.595	21.3423	21.2791	21.1269	21.4036	21.6598	21.3242	21.354
DUS3L	15.767	16.5201	17.1581	17.5185	17.2476	16.5008	16.7311	16.935	16.6266
DUSP12	18.6568	18.6017	18.8262	18.6151	18.4048	18.8949	18.7287	18.5975	18.7738
DUT	22.8997	22.6004	22.7948	22.8416	22.7815	22.7888	22.5176	22.6387	22.7745
DYNC1H1	21.7126	24.3149	21.5313	21.4922	21.4129	21.5962	21.8644	21.7084	21.759
DYNC1I2	21.4182	21.2101	21.1752	21.4683	21.1347	21.2183	21.2017	21.3136	21.5136
DYNC1LI1	21.3832	20.9677	21.2816	21.2748	21.1829	21.021	21.2763	20.9721	21.207
DYNC1LI2	20.5106	20.3949	20.5411	20.3807	20.5516	20.9272	21.1113	20.7213	20.7998
DYNLL1	21.927	20.7604	21.8337	21.979	21.7507	22.2493	21.9905	21.962	22.0845
DYNLRB1	19.5003	19.2449	19.0983	19.4625	19.3623	20.0256	20.0126	20.0255	19.9222
DYNLT1	17.7882	17.7871	17.8799	18.1604	17.9874	18.5695	18.2548	18.154	18.1391
EBNA1BP2	20.4364	19.3001	20.2001	20.39	20.1012	20.4762	20.5366	20.0999	20.2126
EBP	17.784	16.8021	17.4502	17.8476	17.7111	17.6324	17.8038	17.8866	18.0557
ECE1	20.4971	20.327	20.3326	20.365	20.1537	19.9206	20.4973	20.0871	20.1535
ECH1	21.8151	21.7431	21.7719	21.6478	21.4793	21.5994	21.7675	21.6158	21.8045
ECHDC1	18.697	18.6266	18.2068	18.8759	18.4214	17.3898	17.4255	18.0444	17.8311
ECHS1	21.1422	21.4116	21.0924	21.1785	21.1146	21.3527	21.6336	21.022	21.4012
ECM29	20.5539	20.2868	20.3499	20.2208	19.9803	20.0037	20.4914	20.1502	20.3105
ECSIT	15.5919	15.1913	15.4459	15.7772	15.5993	15.7654	15.5859	15.4879	15.1992
EDC3	18.9523	18.9155	18.668	18.3046	18.5799	18.6877	19.4295	18.7231	18.8502
EDC4	19.975	19.7608	19.7346	19.6297	19.5778	20.0099	19.9942	19.7138	19.7152
EEA1	19.3531	18.7783	19.3202	19.4441	19.1706	18.8468	19.0677	18.8568	19.2106
EED	17.2353	16.5756	16.5067	16.0724	16.234	16.4087	16.7417	16.4766	15.8369
EEF1A1	27.9784	27.567	27.7861	27.6832	27.6999	27.3739	27.6828	27.4197	27.5505
EEF1B2	24.1122	24.1965	24.2756	24.3728	24.1752	24.3197	24.544	24.1703	24.4702
EEF1D	24.5668	24.6134	24.8738	24.5599	24.5889	24.5567	24.9585	24.5756	24.7656
EEF1E1	16.9284	15.4227	16.2222	17.34	16.5492	17.4214	16.8993	17.1887	17.0873
EEF1G	25.2146	25.3018	25.2498	25.2148	25.1012	25.0962	25.4977	25.2571	25.2901
EEF2	26.1927	26.1021	26.3759	26.1155	25.9726	26.0671	26.4936	26.1257	26.2422
EFHD2	20.7432	20.5343	20.6864	20.4399	20.7105	20.7896	21.2623	20.7951	20.8534
EFTUD1	17.1292	17.8666	17.6605	17.7451	16.8718	16.8677	18.1618	17.0493	17.3958
EFTUD2	22.322	22.3013	22.4112	22.1736	22.0454	22.0327	22.64	22.2709	22.4622
EGFR	17.9242	17.832	17.5448	17.6861	17.288	17.2756	18.0327	17.5301	17.8003
EHBP1L1	17.4999	17.5896	17.8216	17.6555	18.0312	17.0107	18.0357	17.404	17.0001
EHD1	21.6789	21.7149	21.7025	21.6048	21.3358	21.7838	22.0201	21.6136	21.8505
EHD4	21.6372	20.9753	21.6154	21.5288	21.3668	21.7929	22.3252	21.7109	21.8082
EIF1	21.1867	20.972	20.7439	21.3354	20.7608	20.706	20.7226	20.669	20.812
EIF1AY	19.9741	19.828	19.9948	19.7434	20.3979	20.8075	20.6682	20.1412	19.7755
EIF2A	20.5499	20.7792	20.7905	20.5695	20.5583	20.5114	20.8478	20.5571	20.7105
EIF2AK2	19.9978	19.9643	20.0319	19.9941	19.6504	19.6572	19.6066	19.7768	19.99
EIF2AK4	19.1497	19.1478	19.2607	18.7934	19.0163	17.8585	18.743	18.4115	18.412
EIF2B1	20.915	20.876	21.2076	21.0658	20.7845	20.9749	21.3316	20.9719	20.9071
EIF2B2	18.7027	18.6545	18.7035	18.54	18.7989	19.2116	19.2705	18.7839	18.9107
EIF2B3	20.0808	19.6412	19.7554	19.8339	19.7951	19.9129	19.7777	19.8374	19.6704
EIF2B4	19.4109	19.561	19.3136	19.4431	19.7114	19.5698	19.6753	19.5175	19.4614
EIF2B5	20.106	19.9376	19.8617	20.1415	20.0675	19.7201	19.7984	19.7055	20.0588
EIF2C2	19.9606	19.9707	20.4577	19.2726	20.4046	20.6759	19.8873	20.3103	19.8446
EIF2S1	22.6109	22.4823	22.8706	22.7394	22.6365	22.9647	23.1459	22.7427	23.0387
EIF2S2	22.1006	22.2155	22.2805	22.1708	22.1808	22.5259	22.7268	22.333	22.5453
EIF2S3	22.7374	22.8679	22.6021	22.8685	22.521	22.8453	22.9961	23.0092	23.0621
EIF3A	22.5489	22.1523	22.634	22.489	22.3448	22.419	22.5943	22.407	22.5857
EIF3B	23.1442	22.8511	22.9211	22.7606	22.6128	22.7231	22.7217	22.9	23.0009
EIF3C	22.4374	22.2636	22.4561	22.146	22.1717	22.2333	22.5564	22.3568	22.4905
EIF3D	22.4789	22.2967	22.2483	22.3226	22.1034	22.114	22.1309	21.9999	22.1977
EIF3E	22.1228	21.6607	22.2224	22.0047	22.041	22.1298	22.3775	22.0198	22.3457

EIF3F	22.734	22.5073	22.5227	22.5833	22.4165	22.3085	22.4135	22.4409	22.4051
EIF3G	21.9072	21.8071	21.9218	22.1023	21.7313	22.139	21.6587	21.7736	22.0913
EIF3H	22.5312	22.8341	22.3471	22.2589	22.1432	22.2445	22.5798	22.2024	22.4873
EIF3I	22.8861	22.28	22.301	22.4329	22.1571	22.2484	22.0649	22.4092	22.3745
EIF3J	21.9428	21.2305	21.4526	21.7499	21.39	21.4773	21.4714	21.4137	21.8091
EIF3K	22.5976	22.6798	22.6817	22.5853	22.4588	22.5721	22.6793	22.5273	22.5785
EIF3L	22.1095	20.7389	22.1713	22.3715	22.1413	22.0978	22.2514	21.9935	22.171
EIF3M	22.0494	22.0716	22.1372	22.0213	21.8507	21.8869	22.0429	21.9182	21.9434
EIF4A1	25.123	25.0639	24.972	25.0857	24.7981	24.9482	25.2605	25.042	25.1945
EIF4A2	20.3999	20.4672	20.1573	20.3968	20.126	19.9691	20.341	20.3833	20.3257
EIF4A3	22.1818	22.029	22.1234	22.1182	21.9497	22.0866	22.0326	22.0399	22.2314
EIF4B	21.8622	19.987	21.8206	21.8803	21.6974	22.2318	22.5787	21.8731	22.2334
EIF4E	21.6611	21.7163	21.8189	21.6434	21.3275	21.571	21.5943	21.5379	21.801
EIF4G1	21.4855	22.0222	21.5294	21.5532	21.4601	21.5387	21.7548	21.4841	21.7556
EIF4G2	21.6984	21.5833	21.5203	22.0155	21.4955	21.4193	21.3928	21.1401	21.572
EIF4G3	17.6323	17.1726	17.223	16.9463	16.7646	16.82	16.8407	16.8622	16.8375
EIF4H	22.2664	21.9066	21.4188	21.7295	21.5026	21.7943	22.2279	22.1371	22.26
EIF5	22.4934	22.2524	22.3101	22.2203	22.0531	22.232	22.3538	22.3048	22.3853
EIF5A	24.4536	24.2027	24.3983	24.3861	24.1515	24.3931	24.3374	24.0829	24.1119
EIF5B	21.8489	21.7354	21.5459	21.6845	21.4799	21.4869	21.4181	21.5095	21.6848
EIF6	23.5356	23.4828	23.5985	23.6716	23.4376	23.7071	23.8362	23.5172	23.8932
ELAC2	19.0594	19.2345	19.2568	19.1095	18.9557	18.5911	19.2522	19.0791	19.342
ELAVL1	22.6842	22.5744	22.6236	22.5906	22.3782	22.6373	22.9299	22.561	22.6332
ELMO2	18.3631	17.9836	17.9517	18.218	18.0926	18.1705	17.7245	18.0403	17.4871
ELP3	17.7325	17.7291	17.7078	17.7055	17.6242	15.8141	17.2621	17.6397	16.9338
EMC1	20.2762	19.9048	20.0928	20.498	20.3284	20.1493	20.4354	20.2228	20.4007
EMC10	18.185	17.1462	17.1007	17.2676	17.6923	16.2205	17.6413	17.7574	17.6897
EMC2	18.7509	18.7955	18.8669	18.6754	18.5991	18.7312	18.5525	19.001	19.1349
EMC3	21.0429	19.6401	18.6523	18.6479	18.3118	18.7993	18.476	18.8631	18.5872
EMC7	20.4299	20.2021	20.1176	20.2244	20.1403	20.2872	20.4036	20.3335	20.1513
EMD	20.3338	19.2832	20.3458	20.5002	20.3351	20.5453	20.9108	20.3023	20.8575
EMG1	22.0031	21.6135	21.8661	21.6622	21.7192	21.7996	21.8793	21.7221	21.7536
EML2	17.8015	15.8255	16.7282	16.835	17.0989	16.9229	16.9674	17.3141	17.1407
EML4	18.1171	18.4764	18.7114	18.484	18.2574	18.3109	18.481	18.2997	18.6228
ENAH	19.7913	18.3711	19.8931	19.2497	19.8702	19.8766	20.5144	19.9334	20.1998
ENDOD1	17.6164	16.9418	17.0281	17.3659	16.9615	17.3521	17.1432	16.9926	17.1233
ENO1	26.7595	26.5987	26.8233	26.6516	26.4845	26.8506	27.0947	26.8406	26.7791
ENO2	19.1731	19.3042	19.5149	19.1096	19.3917	19.6335	19.8399	19.5431	19.3918
ENOPH1	20.1844	19.77	19.5541	19.8856	19.5941	19.3214	18.935	19.4719	19.6865
EPB41L2	21.2476	20.8846	21.0239	20.8158	20.8295	20.8194	21.0277	20.8959	20.8486
EPHA2	19.3545	19.398	19.2357	19.1225	18.8542	19.0422	19.3557	19.3027	19.548
EPHX1	21.4545	21.408	21.2312	21.1831	21.224	21.3863	21.6251	21.5063	21.4261
EPRS	22.1248	21.8619	22.0501	22.0078	21.9964	22.0476	22.3249	22.0893	22.0766
EPS15	17.5521	17.6993	17.6013	17.6511	17.5404	17.501	17.7826	17.883	18.1307
EPS15L1	18.7926	18.7907	18.6715	18.6109	18.4221	18.8339	18.82	18.7624	18.8925
EPS8	20.1885	19.9425	20.0807	19.9988	19.9166	19.1909	19.7927	19.6222	19.7376
ERAP1	21.2588	19.849	21.6672	21.5991	21.7744	21.727	22.1959	21.4438	21.861
ERBB2	17.9513	17.4878	16.752	17.6224	16.6806	17.4857	17.7794	18.1754	18.4386
ERC1	18.8297	18.2612	18.5641	18.0916	18.1856	18.3339	17.8037	18.4936	18.618
ERCC6L	18.1955	17.8831	17.7685	17.1123	17.3744	17.5667	17.3341	17.5035	17.3638
ERGIC1	19.6855	19.7504	19.8525	19.7924	19.7052	19.6606	19.846	19.5859	19.5682
ERGIC2	18.2922	18.3483	18.3135	18.5945	18.3087	17.7968	18.0927	18.2902	17.7053
ERGIC3	19.3164	19.1806	19	19.0342	19.0865	18.9854	19.1551	19.1846	19.1893
ERH	20.5709	20.4978	20.5386	20.7038	20.5596	20.3874	20.5518	20.3366	20.9924
ERLEC1	18.6609	18.1927	18.1995	18.3066	18.1432	18.2007	17.9471	18.04	17.9788
ERLIN1	18.5492	16.5678	18.01	18.3024	18.2535	18.9221	18.5908	18.3759	18.6365

ERLIN2	19.5036	17.5777	19.3022	19.4901	19.2683	19.4737	19.4101	19.4274	18.9328
ERO1L	21.0297	20.4467	20.7323	20.9612	20.806	20.8324	21.2676	21.007	21.341
ERP29	23.7594	23.6595	23.6233	23.4567	23.4231	23.7684	24.1532	23.5773	23.7255
ERP44	22.2028	21.8844	21.7917	21.5988	21.7197	21.9081	22.2769	21.9788	22.0347
ESD	21.0059	19.0068	20.8801	20.9255	20.8528	20.6991	21.2077	20.9868	21.1291
ESF1	17.6412	17.4286	17.1664	17.3995	17.3471	17.1496	17.7762	17.4961	17.3135
ESYT1	21.8402	21.5597	22.0507	21.6965	21.7961	21.662	22.1357	21.8741	22.0924
ESYT2	19.1633	18.967	19.233	19.087	19.1308	19.1417	19.0627	18.9883	18.916
ETF1	22.4387	22.3685	22.3703	22.2663	22.0213	22.0019	22.2677	22.0839	22.3718
ETFA	22.2272	22.1147	22.3057	21.9942	21.8821	22.201	22.3306	22.1481	22.0722
ETFB	22.0435	21.8379	22.0736	22.0684	21.9359	22.2423	22.3414	21.8823	22.0759
ETHE1	19.9333	19.9389	19.9438	19.969	19.8149	20.0545	20.2954	19.9382	20.1607
EWSR1	21.828	21.9639	22.048	21.8828	21.7972	22.21	22.4527	21.9073	22.104
EXOC1	18.1403	18.2425	18.2981	18.0254	18.22	17.9336	17.6176	17.9027	17.8148
EXOC2	19.2535	18.763	18.7327	18.3592	18.6157	18.7956	19.2164	18.8293	18.7513
EXOC3	18.4883	18.3881	18.1916	18.3168	17.9718	18.0143	18.4706	18.4047	18.4242
EXOC4	17.3823	17.4817	17.8536	17.2695	17.3205	17.0281	18.0882	17.7941	17.8321
EXOC5	17.557	17.3391	17.646	17.3775	17.1455	16.9927	17.2884	17.4223	17.6076
EXOC7	18.111	18.1019	17.9673	18.0482	18.1517	17.749	18.1245	17.8033	17.9631
EXOC8	16.7161	17.4927	17.153	17.0822	16.9316	17.8183	17.2569	16.9389	17.4433
EXOSC1	17.9744	17.86	18.2899	18.3075	17.8848	18.4066	18.4688	18.3812	18.3566
EXOSC10	20.5348	20.4914	20.4392	20.4247	20.2009	20.1895	20.3748	20.5399	20.368
EXOSC2	21.1591	20.5826	20.5054	20.6489	20.4888	20.5067	20.8541	20.6424	20.6661
EXOSC3	20.0053	19.5302	19.5105	19.579	19.7218	19.6977	18.746	19.8569	19.4218
EXOSC4	19.2645	19.6096	19.1172	19.1911	18.9569	19.2719	19.6759	19.294	19.53
EXOSC5	19.4832	19.684	19.6258	19.5863	19.527	19.7796	20.0934	19.5755	19.8248
EXOSC6	19.7095	21.6746	19.3831	19.8432	19.5424	19.8005	19.8028	19.8614	20.111
EXOSC7	21.0492	21.2644	20.7669	20.8003	20.4889	20.9455	20.9161	20.7054	21.2047
EXOSC8	20.3085	20.2359	20.3357	20.0994	20.1263	20.3975	20.3485	20.4699	20.7233
EXOSC9	21.0305	20.8488	20.7493	20.6487	20.5925	20.6061	21.0878	20.9053	21.0268
EZR	21.6925	21.8636	21.6381	21.5645	21.3683	21.5692	21.7237	21.5176	21.7721
FABP5	19.9815	12.6959	11.8071	12.858	12.546	11.2668	9.98971	11.4127	13.2684
FADS2	19.195	19.108	18.8964	18.8345	18.7225	18.8651	19.2608	18.9936	19.0076
FAF2	20.1875	19.9415	20.0886	19.9042	20.1361	19.9102	20.4325	20.0537	20.2324
FAH	16.928	17.0595	17.1254	17.3345	17.415	17.5562	18.1432	17.5701	17.5954
FAHD1	18.1227	18.2645	17.9156	17.9716	17.9433	18.2144	17.8024	17.904	18.0925
FAHD2A	19.1909	18.9364	18.9974	19.2485	18.9707	19.6118	19.6342	19.4979	19.6191
FAM114A1	17.0009	17.0751	18.1895	17.298	17.388	17.3634	17.65	17.2245	17.384
FAM115A	18.8817	18.8751	19.2045	19.0456	19.0971	19.0613	19.3507	19.1832	19.1856
FAM120A	19.8874	19.9472	20.0013	19.5113	19.7356	19.6259	20.1282	19.9707	19.8554
FAM120C	19.2811	18.7907	18.3981	18.6275	17.7192	18.2676	19.1367	18.871	18.8755
FAM126A	16.9981	17.5261	17.4997	17.4133	17.7132	17.7505	17.031	17.282	17.1505
FAM129A	21.4638	21.3988	21.3505	21.089	21.0853	20.9459	21.5038	20.9929	21.1079
FAM129B	21.0919	20.9619	21.0673	20.8921	20.8564	21.1358	21.4842	21.0822	21.1315
FAM162A	20.1915	20.262	20.1709	19.8533	19.863	19.741	19.9905	19.9714	19.9341
FAM20B	17.7777	17.3148	17.4253	17.4674	17.2109	17.2509	17.2615	17.2645	17.2094
FAM21C	17.2887	17.1813	17.6023	17.2874	17.3182	17.772	18.2101	17.5973	17.2344
FAM3C	20.1086	20.3077	20.2965	20.0453	20.0844	20.1022	19.9996	19.8624	19.8246
FAM49B	20.3987	20.2788	20.3026	20.2493	20.1528	19.9549	20.082	19.8556	19.8689
FAM50A	20.1457	19.7695	20.0401	19.9417	19.9993	20.1822	20.3065	20.0028	19.9011
FAM91A1	16.881	17.2775	17.3144	17.1753	17.4742	17.0404	17.7913	17.4526	17.3602
FAM96B	18.2269	17.232	17.5239	17.379	17.2556	17.4707	17.298	18.0008	18.5273
FAM98A	20.4865	20.4069	20.4531	20.5433	20.3265	20.4684	20.311	20.4496	20.595
FAM98B	19.7525	19.6694	19.7828	20.0261	19.6942	19.6737	19.6461	19.6648	19.9267
FANCD2	17.8853	17.9937	18.0115	18.2939	17.8321	17.0145	18.0697	18.1347	18.242
FANCI	19.7248	19.7813	19.6047	19.7511	19.555	19.5798	20.1204	19.7167	19.8709

FARSA	20.7194	20.1149	20.6774	20.7065	20.3031	20.3574	20.8773	20.6083	20.9832
FARSB	22.2178	21.6192	22.2815	22.0976	22.215	22.3374	22.5489	22.2817	22.3326
FASN	24.1591	23.6869	23.5206	23.6271	23.4464	23.735	24.0543	23.7444	23.688
FASTKD2	19.0628	18.968	18.9289	18.5924	19.1244	18.6593	18.9633	18.5066	18.7602
FAU	21.4231	21.8309	21.6452	21.9903	21.8251	21.9011	22.34	21.8329	21.8517
FBL	22.5751	22.101	22.3659	22.3159	22.1602	22.0948	22.5907	22.2954	22.2708
FBXO22	20.487	20.5031	20.84	20.6963	20.4585	20.5049	20.908	20.3723	20.6494
FBXO7	18.5286	18.1376	18.4171	18.2585	18.151	18.3475	18.4896	18.3507	18.2144
FDPS	22.0565	21.5287	21.8188	22.0268	21.567	22.0476	21.9102	21.9958	22.049
FEN1	22.5497	22.4955	22.5461	22.3386	22.3888	22.5387	22.6822	22.2993	22.3162
FERMT2	22.1141	22.4378	22.3346	22.5631	22.3955	22.4183	22.7792	22.4223	22.5369
FH	21.6144	21.5627	21.601	21.9053	21.4118	21.5296	21.3	21.4914	21.824
FHL2	20.2806	20.0579	20.5078	19.9962	20.2328	20.5839	21.0159	20.4238	20.4889
FHOD1	18.5825	18.3728	18.3058	18.3346	18.2277	18.219	18.2305	18.1672	18.0487
FIP1L1	18.8078	18.8141	18.7668	18.9938	18.6497	19.0466	19.1245	19.0365	19.0843
FIS1	18.5601	19.0836	18.844	19.0174	19.1056	19.4369	18.6217	18.7914	18.218
FKBP10	21.0217	21.2923	21.2082	21.1486	20.9245	20.6269	20.6519	20.8566	21.2738
FKBP11	19.7719	19.9795	19.6863	19.7182	19.5371	20.2523	20.2343	20.2117	20.1777
FKBP1A	21.6425	21.139	21.9685	21.8772	21.5648	22.2647	21.8304	21.8929	22.1853
FKBP2	21.7623	21.6876	21.8003	21.8591	21.4987	21.7542	21.8746	21.5573	21.7487
FKBP3	20.7725	20.535	21.1028	20.8445	20.6509	21.2866	21.2893	20.8033	21.0063
FKBP4	22.5028	21.5137	22.4525	22.3231	22.0966	22.5523	23.0176	22.3782	22.4942
FKBP8	18.5319	18.2864	18.5038	18.5247	18.0963	18.4161	18.511	18.676	18.6334
FKBP9	20.1095	20.0256	20.2099	19.9899	19.9455	20.4134	20.568	20.2978	20.2957
FLAD1	17.5497	18.0541	18.0506	18.0121	17.9835	17.9423	18.0492	18.0791	17.6964
FLII	19.4204	19.4975	19.4807	19.6335	19.5151	19.6102	20.3582	19.6762	19.8023
FLNA	24.4388	24.216	24.2882	24.3997	24.1331	24.3239	24.4859	24.2289	24.4586
FLNB	22.1916	21.8509	21.9079	21.8694	21.7582	22.0412	22.3086	21.9422	21.9292
FLOT1	18.2944	18.5609	18.8794	18.711	18.7086	18.8492	18.926	18.9899	19.14
FLOT2	19.6081	19.4753	19.7063	19.3014	19.5649	19.3058	19.6965	19.6107	19.5894
FLRT3	17.3898	18.1651	18.297	18.0427	17.9015	17.5932	18.3574	17.8689	17.7459
FLT1	18.2598	18.2051	17.9588	18.1491	17.8261	17.5743	18.2009	17.7751	18.0502
FMNL3	16.4089	16.5668	16.6416	16.9372	16.9377	17.7687	16.6316	17.0319	16.2218
FMR1	18.4321	18.2525	18.345	18.2593	18.3943	17.536	17.8874	18.0703	17.9866
FN3KRP	19.4465	19.3621	19.5377	19.3182	18.9906	18.6697	19.0664	18.6691	18.9678
FNBP1L	18.8892	18.5287	18.4583	18.587	18.0208	18.0792	18.0212	18.4282	18.3922
FNDC3B	17.373	17.8213	18.4416	17.9024	17.618	17.4813	17.6359	17.2858	17.6361
FNTA	18.6484	18.9517	18.9624	18.7672	18.6101	18.8998	19.0749	19.1003	19.0289
FOXK1	18.7123	18.6506	18.56	18.794	18.4772	17.9844	18.8469	18.7531	18.5753
FOXRED1	17.5537	17.7947	17.2145	17.407	17.318	16.9364	16.9995	17.6487	17.5249
FOXRED2	14.4276	15.8647	15.4698	15.2422	15.5937	15.2987	15.882	14.8763	15.4742
FRYL	16.6268	17.0311	16.9786	17.0249	16.8812	17.1939	16.6	16.8059	16.2641
FSCN1	23.6158	23.1932	23.3813	23.4454	23.2979	23.1825	23.5833	23.3495	23.4688
FTH1	18.2646	19.1471	18.8443	17.2404	18.8126	19.6398	19.8115	19.4765	19.6342
FTO	14.3339	15.5128	15.2277	15.564	15.1403	15.9316	15.146	15.7842	15.8014
FTSJ1	17.335	16.8902	16.808	17.0113	16.7044	16.7895	16.6332	16.8723	17.2215
FTSJ3	20.1394	19.8835	20.2991	20.089	20.1642	19.7787	20.3565	20.2434	20.0665
FUBP1	22.8907	22.9526	22.8474	22.8676	22.8001	23.2195	23.4623	22.8449	23.2744
FUBP3	20.5046	20.1756	20.2546	20.9231	20.5583	20.3496	20.4972	20.5263	20.527
FUS	22.7093	22.1312	22.9635	22.6364	22.4903	23.041	23.4224	22.5894	22.7642
FXR1	22.26	21.844	21.7516	22.0075	21.7831	21.7598	21.9191	21.906	21.8281
FXR2	19.4916	19.2528	19.0951	19.4783	19.2641	19.4631	19.0928	19.2354	19.4369
G3BP1	23.7831	23.5376	23.6185	23.6372	23.4631	23.6993	23.9735	23.5848	23.7082
G3BP2	19.8262	19.3553	19.4627	20.0274	19.7275	19.9648	19.7938	19.6459	20.1602
G6PD	22.9338	22.5422	22.8667	23.067	22.7437	23.3491	23.4277	23.1537	23.485
GAA	16.0707	16.8586	16.6553	16.5402	16.993	16.0126	16.0481	16.7939	16.5286

GABARAP	12.8821	14.8679	15.7369	16.5831	16.1955	15.3016	12.6793	15.4555	14.6511
GABPA	18.4365	18.5623	18.3545	18.364	18.4935	18.3435	18.6608	18.4854	18.8652
GADD45GIP1	16.2111	17.36	16.7552	16.726	16.0964	16.6298	16.438	16.7456	17.0869
GAK	19.2179	18.9	19.2875	19.2548	19.3777	19.7264	20.1422	19.8759	20.3183
GALE	18.4863	19.0963	18.7817	18.9289	18.5335	18.4908	17.8496	18.665	18.9665
GALK1	20.0605	19.2535	19.6759	19.1732	19.3869	19.7538	20.1959	19.8342	20.013
GALNT2	21.7355	21.4722	21.4442	21.3035	21.347	21.2526	21.4319	21.3207	21.3466
GANAB	24.0379	24.0571	24.1803	23.9254	23.8107	23.801	24.1664	23.9194	24.1103
GAPDH	28.9387	28.6733	28.7987	28.6731	28.6378	29.2063	29.2142	28.9884	28.8799
GAPVD1	19.7816	18.6332	19.858	19.927	19.8046	19.9716	20.2959	19.678	19.9471
GAR1	20.5011	20.9303	20.5064	20.6289	20.696	20.8659	20.8028	20.725	20.674
GARS	22.2667	21.8124	21.9006	21.9094	21.8505	22.0265	22.4136	22.0797	22.0592
GART	22.0799	21.3025	22.2411	21.9275	21.9455	22.0445	22.61	22.0451	22.1278
GATAD2B	20.2244	19.1064	19.89	20.0568	19.8988	20.1167	20.4159	19.8409	19.9501
GBA	20.7647	20.0382	20.143	20.1615	19.7559	19.9759	19.7379	20.0353	20.3732
GBAS	19.9333	18.9345	19.6993	19.9258	19.7828	19.8017	20.0117	19.9052	20.0675
GBE1	19.7747	19.6953	19.5533	19.4502	19.4644	19.7362	19.7647	19.6215	19.6849
GBF1	18.0311	18.069	17.8195	17.8644	17.736	17.7198	18.3609	18.1428	18.4311
GCDH	17.6915	18.2478	18.0973	18.3933	17.881	17.9646	17.431	18.1453	17.9199
GCLC	19.1186	18.927	18.5511	18.8055	18.4119	18.5723	18.6813	18.8071	18.9345
GCLM	21.2132	20.3849	21.2147	21.3748	21.1572	21.1385	21.5276	21.0725	21.1044
GCN1L1	21.2809	20.902	20.827	20.7624	20.8145	20.5528	20.932	20.6485	20.7089
GDI1	21.1016	21.1852	21.1747	21.122	20.8653	21.2878	21.2521	21.3568	21.6401
GDI2	23.045	22.7233	22.5515	22.5381	22.5248	22.8333	22.7716	22.6011	22.6916
GEMIN4	18.8706	20.1196	19.1145	18.9511	19.5089	18.9538	19.9114	19.2364	18.8386
GEMIN5	19.0018	18.9206	18.9606	18.8202	18.7441	18.3711	18.9986	19.0217	18.972
GFM1	20.5643	20.4104	20.2631	20.3423	20.0642	19.8996	20.1005	19.9832	20.2405
GFM2	17.1975	16.8801	17.1744	17.1199	16.9153	16.7034	17.4232	17.0423	17.0458
GFPT1	21.4211	21.3441	21.5037	21.2772	21.418	21.5806	21.9664	21.6064	21.7601
GGCT	21.014	20.8259	21.0824	20.9425	20.8768	20.7307	20.7829	20.4945	20.4137
GGH	21.3297	21.4026	21.4983	21.2927	21.3938	21.4759	21.6691	21.3979	21.5558
GGT7	14.3423	16.4396	16.6962	16.7089	16.8982	17.5331	16.7645	16.967	17.0279
GIGYF2	20.2857	19.9292	20.3362	20.1165	19.9981	20.3283	20.6472	20.0934	20.4073
GINS3	17.1264	17.526	17.2654	17.2624	16.9834	18.3543	18.1847	17.7626	17.781
GINS4	19.8628	19.6458	19.6038	19.375	19.5615	19.8843	20.0888	19.6611	19.5321
GIPC1	20.4724	20.2619	20.3117	20.3668	20.1169	20.1512	20.3716	20.1476	20.4148
GIT1	17.9145	17.1812	17.2849	16.7384	16.9117	16.9946	17.2769	17.2506	16.5096
GIT2	14.3208	15.3918	16.0598	16.6165	15.6908	15.3094	13.8955	15.5275	14.7506
GLA	19.3957	18.5515	18.8358	18.8822	18.0891	18.6434	18.4897	18.7479	18.9293
GLB1	19.6913	19.238	19.2765	18.9467	19.0819	19.2923	19.6229	19.1832	19.0072
GLG1	20.2608	20.1341	20.3087	20.0527	19.9953	19.5487	20.0351	19.6503	19.7096
GLO1	22.5172	22.4422	22.6192	22.395	22.3539	22.685	22.8062	22.6019	22.6868
GLOD4	20.6867	20.3572	20.9217	20.7121	20.4187	20.9615	21.3109	20.885	20.9657
GLRX3	21.5123	21.2245	21.2061	21.2275	21.2741	21.4628	21.5005	21.4619	21.335
GLS	21.1868	20.8292	20.9987	20.8706	20.7532	20.9063	21.567	21.0004	21.0208
GLUD1	21.5769	21.0368	21.326	21.1623	20.8816	20.6769	21.1133	21.0046	21.073
GLYR1	18.2597	18.2283	18.4683	18.078	18.148	18.2401	18.2606	18.1958	18.1283
GMDS	18.7525	18.4485	18.2665	18.4629	18.1785	18.2927	18.4387	18.377	18.7349
GMPPA	19.4588	19.2648	19.4964	19.2569	19.3004	19.2915	19.4795	19.3313	19.1025
GMPPB	20.3285	19.7583	20.3354	20.3874	20.3379	20.4183	20.5493	20.3048	20.4852
GMPR2	17.5755	17.671	17.6241	17.3983	17.4139	17.5987	17.9131	17.6665	17.5824
GMPS	21.5173	21.5775	21.5974	21.7383	21.5538	20.7738	21.7238	21.5854	21.7556
GNA11	19.1471	18.1183	19.0289	18.8838	18.9587	19.239	19.4244	19.2608	19.4257
GNA13	17.9097	17.4604	17.5072	17.4945	17.2956	16.3102	16.3283	17.5753	17.7947
GNAI2	22.0116	21.8499	21.7482	21.9427	21.558	21.9859	22.0335	22.0427	22.2901
GNAI3	19.9867	19.6641	19.358	19.7052	19.2615	19.0154	19.2022	19.2975	19.3435

GNAQ	18.3277	18.291	18.2379	18.574	17.9875	17.4564	17.4724	17.969	18.0003
GNAS	20.6799	21.2396	21.2159	20.9897	20.961	21.2577	21.5555	21.2162	21.3822
GNB1	20.895	19.4058	20.7607	20.7939	20.6246	20.7601	20.8707	20.5785	20.8672
GNB2	21.1776	20.4169	21.0119	20.5893	20.9936	21.4893	21.5396	21.2507	21.3576
GNB2L1	25.3593	25.0429	25.2289	25.0789	25.1745	25.2852	25.4008	25.1343	25.2347
GNE	19.1184	18.8455	18.8652	18.482	18.6742	18.5113	18.8704	18.9963	18.6809
GNL1	19.0955	18.5386	18.7046	18.7454	18.4771	18.8823	19.1872	18.7509	18.7218
GNL2	18.7745	18.7688	18.745	18.9037	18.5171	18.2179	18.6327	19.0393	18.8432
GNL3	21.2797	21.2843	21.3405	21.0709	21.0233	21.0218	21.4926	21.2081	21.2065
GNPDA1	20.8389	21.5124	21.1134	21.3106	21.1926	21.373	21.4012	20.8198	21.2402
GNPNAT1	19.0037	18.4975	18.5825	18.6524	18.735	18.7374	18.6537	18.7631	18.6235
GNS	18.6222	18.5788	18.2459	18.2402	18.181	18.4068	18.1063	18.4252	18.9505
GOLGA2	17.5862	17.8164	17.7587	17.7068	17.6625	17.9809	18.0239	17.9923	17.8257
GOLGA3	17.4017	17.565	17.6959	17.9208	18.0531	17.4725	17.5654	17.4061	18.0293
GOLGA4	17.235	17.1388	17.3549	17.0988	17.0132	16.9092	17.3031	17.4714	17.5816
GOLGB1	13.6447	17.0887	17.2887	16.6254	16.9726	16.8326	17.2378	16.6781	16.6727
GOLPH3	18.2079	17.9641	17.9682	17.9703	17.8834	17.7837	18.2243	17.8403	18.1193
GOLT1B	19.1696	19.0041	19.0464	18.8431	19.1063	18.8792	18.684	18.6805	18.2604
GORASP2	19.6913	18.8501	19.4324	19.5141	19.3863	19.5474	19.5252	19.4347	19.5067
GOT1	21.1615	20.5903	21.1314	21.1985	20.8452	20.9919	21.3784	21.1753	21.3579
GOT2	23.2251	22.7215	23.0744	23.0284	22.9244	23.1536	23.3792	23.0615	23.288
GPATCH4	18.9064	18.3214	18.435	18.3624	18.233	18.5464	18.603	18.7347	18.4181
GPC1	19.9305	22.0696	19.7974	19.7089	19.5507	20.045	20.4163	19.8152	20.1407
GPD1L	17.8948	19.0386	16.8745	16.6567	16.8982	16.4628	16.4944	16.7839	16.5615
GPD2	20.9508	21.1809	21.36	20.9895	21.2647	20.999	21.2938	21.0359	21.0556
GPHN	18.3908	17.7389	18.1295	18.2731	18.1717	18.7063	19.0969	18.3717	18.6425
GPI	23.218	23.0459	23.7098	23.7518	23.6424	24.1182	24.3622	23.932	24.1455
GPKOW	18.3691	18.2724	18.2128	18.3844	17.6299	17.9215	18.0534	18.0236	18.3733
GPN1	18.9945	18.7971	19.0218	18.9786	18.8685	19.3315	19.1156	18.9059	18.7897
GPRC5A	21.0746	20.9422	20.7712	21.0596	20.6972	20.8571	20.8951	21.0083	21.5176
GPS1	20.8601	20.932	21.3084	20.9707	20.9649	20.678	21.2864	21.0003	21.1307
GRB2	20.1362	20.0846	20.4406	20.2298	20.0719	20.05	20.6733	20.1251	20.4244
GRHPR	19.3456	18.8986	19.2619	18.9407	18.9254	19.1461	19.7092	19.3976	19.4424
GRIPAP1	17.045	17.944	16.2316	16.2184	16.8086	16.613	16.861	16.6578	16.9377
GRPEL1	19.2225	19.0053	18.8314	19.005	19.0595	17.9803	19.3581	18.967	19.2063
GRSF1	20.7738	20.8654	20.5673	20.7574	20.6631	19.8638	20.7074	20.4972	20.6757
GRWD1	21.3064	21.0098	21.1598	20.781	20.7654	21.0756	21.4188	21.0633	21.0015
GSK3B	18.5917	18.7542	18.4026	18.891	18.7103	18.6544	19.0598	18.525	18.6563
GSN	21.5522	20.8462	21.371	21.3484	21.385	21.9649	21.8274	21.8605	21.7456
GSPT1	21.5566	21.6466	21.5152	21.4967	21.3886	21.419	21.4265	21.3842	21.6588
GSR	21.9196	21.3792	21.7184	21.7377	21.592	21.9641	22.3043	22.0436	22.0205
GSS	21.8165	21.3048	21.5515	21.2226	21.4772	21.8071	22.251	21.5943	21.5684
GSTK1	20.1523	19.9098	19.9076	19.8095	19.6897	20.1699	20.0688	20.107	20.3775
GSTM3	20.7911	21.6442	20.6637	20.3894	20.3295	20.5307	20.6754	20.3595	20.4906
GSTO1	21.2976	20.7395	21.2859	21.4878	21.2533	21.7216	21.8199	21.4635	21.6794
GSTP1	25.2845	24.7271	25.0259	25.0859	24.8534	25.4325	25.7728	25.2703	25.2824
GSTT2	19.5181	19.6192	20.008	19.1892	19.4542	19.0648	19.8823	19.5857	19.6129
GTF2B	18.6686	17.4032	18.1549	18.1089	18.3448	18.4469	18.8942	18.1076	18.2029
GTF2F1	19.5488	19.2555	19.4161	19.3576	19.2097	19.3077	19.2526	19.2223	19.3388
GTF2F2	19.4207	19.7932	19.4964	19.6715	19.2599	19.3236	19.1219	19.4573	19.6737
GTF2H1	16.8876	16.9233	16.6574	16.7023	16.8132	17.2636	16.4146	16.5774	16.6765
GTF2H2	17.9597	17.7946	17.8808	17.4477	17.6859	17.6611	18.1097	17.6714	17.8895
GTF2I	21.0166	20.9738	20.8584	20.7638	20.933	20.8105	21.5533	21.0211	21.1938
GTF3C1	18.2656	18.2908	18.3376	18.1505	18.0955	18.1947	17.8274	18.1699	18.0872
GTF3C2	17.5438	18.5767	19.1651	19.0753	18.98	18.9508	19.3757	19.1517	19.0434
GTF3C3	18.1051	18.1377	18.3347	17.8624	17.8794	17.5865	18.1063	17.9618	18.2048

GTF3C4	19.172	18.8129	19.2032	19.271	19.2179	19.2253	19.3608	19.054	19.1386
GTF3C5	18.3523	18.6561	18.5717	18.8896	18.7427	18.9002	18.7779	18.625	18.2106
GTPBP10	16.8909	17.0597	16.7869	16.0696	16.7297	16.9864	17.0365	16.833	16.7735
GTPBP4	19.471	19.4643	19.4335	19.429	19.2406	18.9569	19.457	19.4376	19.4545
H1FX	19.7359	19.6559	19.58	19.8773	19.2788	19.7257	20.0551	19.6615	19.739
H2AFY	22.799	22.7236	22.6004	22.4367	22.4473	22.7545	22.8674	22.365	22.4198
H2AFZ	23.0733	22.9454	22.9181	22.957	22.9274	23.0777	22.8462	22.8553	23.1371
H3F3A	20.9243	20.8093	21.1084	19.5487	21.4191	22.1678	19.1276	21.0793	20.8772
HACL1	19.7196	19.6451	19.7372	19.3997	19.3158	19.4598	19.8033	19.4817	19.7312
HADH	20.1055	20.188	20.3017	19.8107	20.3027	19.8168	19.8699	20.0736	20.041
HADHA	21.9915	22.3991	22.2963	22.3267	22.2617	22.4405	22.9028	22.2678	22.6301
HADHB	22.8736	22.9439	22.7602	22.8039	22.6632	22.7367	22.9295	22.6876	22.9463
HARS	22.7331	22.6972	22.7932	22.5157	22.552	22.5172	22.9502	22.4867	22.6756
HAT1	20.6818	20.7101	20.6998	20.6513	20.429	20.0509	19.8922	19.7543	20.0768
HAX1	18.9989	18.6718	18.6785	18.7706	18.5671	18.7243	19.0656	18.6914	18.8798
HBA1	19.8003	19.5261	19.8803	20.2526	19.7625	19.8603	20.1837	19.5457	20.0483
HBS1L	18.215	18.4396	18.4683	18.6685	18.4233	18.1767	18.3221	18.2029	17.8968
HCCS	18.0887	18.1136	18.0137	17.9659	18	17.9643	18.016	18.0507	18.2598
HCFC1	21.2887	21.1921	21.7033	21.2704	21.1471	21.6902	21.8284	21.2536	21.8575
HDAC1	21.3622	20.8998	21.0418	21.0075	20.7443	21.2209	21.0493	21.1246	21.3632
HDAC2	21.8767	21.4603	21.6324	21.4737	21.1827	21.2107	21.162	21.3986	21.587
HDGF	23.4217	23.2866	23.2478	23.4736	23.2617	23.1308	23.1877	22.9717	23.4524
HDGFRP2	19.2108	18.2114	19.2787	19.1348	19.2079	19.3341	19.3782	19.292	19.5525
HDHD2	18.0989	17.7651	17.7645	17.6791	17.9574	17.3132	17.8435	17.5685	17.3864
HDLBP	22.3188	21.8477	22.123	22.2437	22.2545	22.6374	23.3363	22.403	22.4822
HEATR1	20.0833	20.1796	20.1946	19.7564	19.869	19.7117	20.5884	20.1222	20.3866
HEATR2	18.6698	18.9002	19.3191	18.7488	18.8019	19.1622	19.5035	18.8896	18.9636
HECTD1	16.9841	16.7543	16.6131	16.9692	16.7107	16.3276	17.1586	17.075	16.9572
HELLS	17.381	17.6456	17.6875	17.4637	17.3521	16.7268	16.6433	17.0755	17.2345
HEXA	20.4068	20.1603	20.2431	20.2988	20.2514	20.0225	20.205	20.4104	20.6053
HEXB	21.3617	21.1751	21.0203	21.1028	21.0176	20.6724	21.1336	21.096	21.2598
HEXIM1	19.1311	18.1307	18.3955	18.9915	18.2563	18.0467	17.5586	18.3188	18.7922
HGS	20.3441	19.6552	20.4742	20.5926	20.1213	20.1947	20.5112	20.2342	20.5133
HIBADH	19.4438	19.3325	18.8182	19.2814	18.8637	19.0037	18.4184	19.2017	19.281
HIBCH	19.0258	18.9851	18.8565	18.99	18.7872	18.4853	18.9744	18.7052	18.6685
HINT1	19.2932	16.8099	19.1642	19.1024	18.9195	20.369	21.307	19.9789	19.9902
HINT2	19.6951	19.8136	19.681	19.7618	19.8413	20.2311	20.5319	19.7483	20.1446
HIP1	17.6317	18.041	17.9141	18.3771	17.8586	17.8866	17.9262	17.7356	18.0636
HIST1H1C	21.7111	22.1572	22.2318	22.4638	22.0054	22.2967	22.2873	21.8424	22.1595
HIST1H2AA	21.3402	21.313	21.1929	21.2967	21.233	15.6859	20.5577	21.7302	21.02
HIST1H2AB	17.9602	15.9737	17.8391	16.9879	17.7839	20.4272	22.883	17.7156	17.672
HIST1H2AH	20.1218	19.4486	20.8951	19.3459	20.2295	22.7277	24.9226	20.7915	19.7025
HIST1H2BJ	24.7906	24.9053	24.9069	24.857	24.8684	24.7517	25.2561	24.8603	25.0527
HIST1H2BK	25.9767	22.6121	25.8051	26.0683	25.9646	26.127	26.4615	26.0601	26.1873
HIST1H3A	22.8917	22.8324	23.1118	24.2386	23.3681	22.4312	21.7341	23.0618	23.2664
HIST1H4A	28.0553	28.4033	28.2635	28.083	27.9718	28.1784	28.501	28.2109	28.3125
HIST2H3A	19.5733	21.9477	22.4389	23.5623	22.8845	18.8509	20.1023	22.6384	22.3299
HJURP	15.4243	14.3694	15.8746	15.8964	15.5289	15.8222	16.5504	16.3988	16.9311
HK1	21.4071	21.5445	21.4907	21.3903	21.3359	21.3342	21.6827	21.3168	21.4849
HK2	20.8952	21.4055	20.8378	20.7463	20.7592	20.9878	21.0595	20.7874	20.9285
HLA-A	23.1939	23.0908	23.1441	23.0174	22.7263	23.1602	23.3746	23.1213	23.3792
HLA-B	18.3932	17.7029	18.1267	17.9576	18.1915	18.5414	18.5762	18.0389	17.9935
HLA-DPA1	22.3187	22.0877	22.0553	22.2118	21.8892	21.8495	21.9504	22.1164	21.8967
HLA-DRA	24.1286	23.5072	23.5838	23.3764	23.6431	23.6554	23.5999	23.6724	23.3255
HLA-DRB1	24.0469	24.3971	23.8156	23.8645	23.6819	23.8453	23.9955	23.7091	24.008
HM13	23.3501	23.3366	23.3977	23.1931	23.1597	23.0396	23.4017	23.1994	23.3882

HMBS	18.1105	17.7924	17.5571	18.0753	17.5542	17.0814	17.2357	17.692	17.7456
HMGA1	22.0166	21.2643	21.6552	22.3547	21.8675	22.2474	22.1631	22.0294	22.4695
HMGB1	22.9381	19.139	22.4743	22.9691	22.7184	23.0284	22.8138	22.4741	22.7829
HMGB2	19.8991	17.9715	19.5495	19.9737	19.508	19.9317	19.227	19.4496	19.8887
HMGB3	20.475	20.6401	20.7516	20.4964	20.4923	20.5967	20.2032	20.3592	20.3403
HMOX2	18.0267	17.6442	17.8481	17.8121	17.4649	17.9674	17.0251	17.6969	17.203
HNRNPA0	24.2495	24.0109	23.9089	23.95	23.8914	24.1428	24.1401	24.0821	24.1467
HNRNPA1	26.2739	25.5899	25.9831	26.1341	25.9263	25.9389	25.564	25.9853	26.1915
HNRNPA2B1	26.647	26.0418	26.1193	26.456	26.0706	26.2204	25.9826	26.2789	26.4405
HNRNPA3	24.9084	24.6456	24.737	24.6915	24.5196	24.429	24.3918	24.6487	24.7714
HNRNPAB	22.1927	21.9334	21.8028	21.9743	21.9626	22.1627	22.4327	22.1695	22.16
HNRNPC	25.0742	24.4509	25.1452	25.0104	24.997	25.1183	25.3867	24.9963	25.0711
HNRNPD	24.0605	23.982	23.9563	24.017	23.9093	24.2072	24.5585	24.1381	24.1168
HNRNPF	23.1584	22.3838	23.1114	23.3492	23.1648	23.5522	23.62	23.2318	23.5784
HNRNPH1	24.7757	24.3838	24.5944	24.4141	24.2812	24.5026	24.5667	24.5556	24.7046
HNRNPH2	21.0343	20.657	20.8135	20.7012	20.497	20.6699	20.6308	20.8716	21.0127
HNRNPH3	23.188	23.1048	23.1577	22.9696	23.0076	23.1327	23.4026	23.1583	23.1834
HNRNPK	25.5245	25.4595	25.395	25.2935	25.2797	25.5614	25.8372	25.4228	25.5467
HNRNPL	24.085	24.0675	23.9432	23.7868	23.6956	23.8611	24.2382	24.008	24.2261
HNRNPM	24.5029	24.2217	24.2852	24.2088	24.0375	24.3688	24.7913	24.4129	24.4706
HNRNPR	23.1567	22.6521	23.0286	22.9206	22.8763	22.9179	22.959	22.9017	22.9136
HNRNPU	25.1392	24.8114	24.6951	24.7631	24.7896	24.2894	24.8067	24.9096	24.8693
HNRNPUL1	21.789	21.7935	21.7854	21.8291	21.5164	21.4359	21.6024	21.3392	21.5043
HNRNPUL2	22.9131	22.8035	22.7101	22.7564	22.5255	22.4722	22.6911	22.6309	22.8413
HNRPDL	23.2446	23.2505	23.1971	23.2612	23.1773	23.3791	23.5862	23.2932	23.4025
HNRPLL	19.2258	18.9488	19.1284	18.9032	18.9231	18.9405	19.2193	19.219	18.8133
HOOK3	19.254	19.2062	19.7158	19.4009	18.8985	19.2856	19.6247	19.1856	19.4794
HPCAL1	21.7781	21.7299	21.6414	21.7586	21.633	21.9732	22.224	21.8707	22.2151
HPRT1	20.5886	20.5002	20.4675	20.5641	20.3536	20.4518	20.5723	20.6181	20.4562
HRNR	18.9832	13.768	13.3354	12.5507	14.3061	13.2271	13.2117	14.1899	13.9549
HSD17B10	22.0697	21.9609	21.9915	21.7829	21.8171	22.8185	23.031	22.7904	22.8147
HSD17B11	18.9409	18.761	18.8217	18.5108	18.6025	19.1219	18.8604	18.9493	19.1178
HSD17B12	18.6415	18.4619	18.3692	17.9727	18.3983	17.3187	18.2169	18.286	17.8372
HSD17B4	21.8797	21.5034	21.8067	21.7343	21.5522	21.6682	21.9987	21.7277	21.7027
HSDL1	18.4643	18.2097	18.3902	17.85	17.9831	17.5728	18.0314	17.8604	17.665
HSDL2	18.5653	18.4652	18.5453	18.5932	18.3755	18.6152	18.9164	18.8039	18.7939
HSP90AA1	26.2837	25.6702	26.0786	26.0939	25.9864	26.246	26.5057	26.2088	26.3853
HSP90AA4P	17.8851	18.038	17.2681	19.0025	17.327	17.6148	14.843	17.3425	18.1317
HSP90AB1	26.8557	26.6206	26.7542	26.6406	26.5085	26.6525	27.0126	26.5932	26.7941
HSP90B1	25.9629	25.6296	25.6646	25.6388	25.6468	25.6181	25.9914	25.638	25.717
HSPA13	18.8601	18.3374	18.3487	18.5236	18.1192	18.6534	18.7962	18.7791	18.8471
HSPA14	17.3315	18.1046	18.2466	18.0699	17.7611	16.8404	16.6384	17.0261	16.728
HSPA1A	24.4781	24.3918	24.5494	24.3341	24.2821	24.5457	24.7618	24.41	24.4725
HSPA4	23.5846	23.5512	23.5946	23.4532	23.261	23.2134	23.6818	23.4683	23.4934
HSPA4L	20.5377	20.7378	20.6317	20.4632	20.5123	20.3483	20.7666	20.4266	20.6845
HSPA5	25.7387	25.4902	25.7786	25.4966	25.5156	25.6179	26.1837	25.6536	25.8306
HSPA8	26.6909	25.6166	26.6275	26.4643	26.3224	26.6028	26.8731	26.5759	26.7228
HSPA9	24.5381	24.2346	24.2906	24.217	24.1005	24.5586	24.7579	24.2433	24.4458
HSPB1	24.9227	24.7854	24.7724	24.5891	24.6297	24.8452	25.0956	25.0014	24.9409
HSPB11	20.9384	20.6512	20.5102	20.8574	20.3672	20.6185	20.3695	20.6146	20.5697
HSPBP1	20.5438	20.257	20.2829	19.9715	20.099	20.2075	20.3753	20.2093	20.034
HSPD1	25.8018	25.3173	25.375	25.2474	25.2671	25.8111	26.0203	25.4774	25.508
HSPE1	23.9286	24.0984	23.8888	24.142	24.0395	24.3032	24.6364	24.1435	24.4767
HSPH1	23.0016	22.6403	22.5851	22.632	22.3759	22.2882	22.5945	22.5258	22.5091
HTATSF1	18.9682	19.0698	18.6631	18.9441	18.6202	18.1294	18.3869	18.4539	18.4121
HUWE1	18.755	18.7466	18.4766	18.5135	18.3561	18.7483	19.0122	18.9198	19.1537

HYI	16.9777	17.3473	16.9561	16.9163	15.3878	16.1563	15.4784	16.8286	17.0374
HYOU1	22.9773	23.012	23.1012	22.8236	22.9026	22.9513	23.3129	23.0101	23.1906
IARS	22.1555	21.3734	21.8653	21.9681	21.7487	21.8708	22.2116	21.8178	22.0424
IARS2	21.1672	21.2489	21.284	21.2037	21.1224	20.9659	21.1764	20.9918	21.1454
ICAM1	21.0011	21.0892	21.1143	20.8833	20.8927	21.4681	21.648	21.3267	21.5757
IDE	19.9091	19.4418	19.706	19.7435	19.6931	19.7172	20.0879	19.8133	20.1751
IDH1	21.0645	20.4645	21.2116	20.9839	20.9435	20.9198	21.23	20.8955	20.901
IDH2	20.6057	20.4658	20.4025	20.3003	20.3817	20.2464	20.693	20.4959	20.3476
IDH3A	21.5303	21.6849	21.7306	21.5626	21.2568	21.5998	21.671	21.5058	21.7605
IDH3B	19.7316	19.1773	19.4659	19.2241	19.3091	19.317	19.7589	19.5744	19.6673
IDH3G	20.325	20.1271	20.114	20.0757	19.8804	19.9916	20.0916	20.0588	20.0938
IER3IP1	18.872	18.735	18.6002	19.1613	18.3759	18.9649	18.6434	18.9059	18.4268
IFI16	19.8951	19.7882	20.0258	19.7188	19.7144	19.9503	20.3696	19.8102	19.7986
IFI35	18.1318	17.7998	17.4962	17.5715	17.6047	17.5064	17.9462	17.6348	17.409
IFITM1	18.5468	19.0975	18.7346	19.1503	18.7298	18.3189	17.9898	18.385	18.7236
IGBP1	18.3027	18.4919	18.6955	18.5476	18.315	18.3616	19.0141	18.2912	18.3778
IGF2BP2	20.9241	20.7941	20.6204	20.5118	20.565	20.8258	21.1534	20.8173	21.2501
IGF2R	20.5173	20.3469	20.5994	20.3599	20.1213	20.2295	20.4479	20.0307	20.5813
IGSF8	17.5957	17.3466	17.2916	17.6503	17.225	18.1096	18.3346	18.1267	17.7188
IK	18.9052	19.4015	19.0637	19.3327	18.8123	18.9749	18.0576	18.9752	19.6032
IKBIP	20.3187	20.527	20.3958	20.4917	20.2506	20.3036	20.6605	20.4067	20.5886
IKBKAP	19.4307	19.2549	19.0624	19.0037	18.9849	19.2018	19.6091	19.4498	19.5193
ILF2	24.3035	24.1648	24.2684	24.1244	24.0144	24.0263	24.3442	24.1478	24.341
ILF3	22.9505	23.024	23.0688	22.9611	22.5575	22.7476	23.0581	22.7978	23.041
ILK	19.798	18.5443	19.6246	19.8318	19.5382	19.8564	19.7488	19.6085	20.0032
ILKAP	20.0653	19.5278	20.2117	19.6508	19.8987	20.0844	20.4518	19.9819	20.0144
ILVBL	19.749	19.6011	19.5094	19.6966	19.4331	19.2933	19.6015	19.5601	19.8369
IMMT	22.6333	22.351	22.8538	22.5877	22.7388	22.3715	23.1024	22.4571	22.734
IMP3	18.1791	18.2794	18.3798	18.2131	17.977	18.2244	18.2876	18.2028	18.488
IMP4	18.8091	18.2708	18.5214	18.4213	18.3675	18.4732	18.059	18.3163	17.7088
IMPA1	20.2389	20.1655	20.0494	20.1742	19.9193	19.8319	19.6789	19.7955	19.9239
IMPDH1	19.713	19.6544	19.793	19.4725	19.5602	19.9154	20.1866	19.7044	19.6477
IMPDH2	23.7784	23.4453	23.4796	23.391	23.3205	23.2351	23.7148	23.464	23.5586
INCENP	17.2864	17.3982	17.4304	18.1066	17.3483	18.0531	18.2736	17.7567	18.6301
INF2	19.1801	19.0798	19.0869	19.2821	18.9311	19.7633	20.1114	19.6147	19.7562
INPP5F	16.0298	15.7888	16.8546	16.7801	16.3371	15.1378	14.8423	15.6895	15.4708
INPPL1	18.3883	18.1889	18.3625	18.2009	18.1025	18.2912	18.5866	18.1353	18.2442
INTS1	16.9446	17.2655	17.402	17.1866	17.0484	16.6937	17.4872	17.3722	17.4458
INTS3	19.3901	19.1936	19.4227	19.3849	19.2626	18.7721	19.7116	19.3122	19.3929
INTS4	17.0443	16.8772	17.0162	16.5733	16.7389	16.3397	16.8329	16.8412	16.8964
INTS5	16.9954	16.9285	17.0473	17.0123	17.0234	16.8467	17.318	16.8869	16.9294
IPO11	18.2499	18.276	18.1916	18.0159	18.3973	17.7858	18.2676	18.047	18.212
IPO4	20.7902	21.0203	20.6153	20.6907	20.5568	20.1276	20.6815	20.4215	20.4918
IPO5	22.9162	22.7424	22.7708	22.5881	22.5335	22.5033	22.9685	22.7789	22.8986
IPO7	20.6192	20.4673	20.8179	20.5743	20.6635	20.1305	20.5765	20.3214	20.3314
IPO8	18.478	19.0783	18.5483	18.2506	18.4058	18.0355	18.8055	18.4857	18.6616
IPO9	21.3581	21.1129	21.362	21.3247	21.1033	21.075	21.4699	21.3387	21.4931
IQGAP1	23.5351	22.2453	22.3215	22.3293	22.1503	22.0023	22.2203	22.2642	22.2599
IQGAP3	19.0667	18.9711	19.1716	19.0699	18.9141	18.6589	18.8391	18.5986	18.8123
IRF2BP1	18.473	18.8605	18.7586	18.5297	18.3669	18.7942	19.0415	18.4619	18.5681
IRF2BP2	20.3423	19.7401	19.9195	20.0507	19.9137	20.1596	20.169	19.822	19.9097
IRGQ	19.7108	20.562	19.9661	19.6399	19.7394	19.8939	20.2418	19.6737	20.0235
ISOC1	19.6786	19.7453	19.7511	19.5746	19.5302	20.1209	20.3679	19.9362	20.3743
ISY1	19.0533	19.5374	19.2397	19.2287	19.1238	19.5284	19.581	19.2275	19.4328
ISYNA1	16.0804	16.5076	16.5389	17.1139	15.7829	14.3528	13.2072	15.3906	14.8148
ITCH	18.8526	19.5016	18.5172	18.5043	18.2034	18.3269	18.9656	18.6185	18.9069

ITGA2	19.9381	19.1957	19.4874	19.0362	19.1265	18.7502	19.3951	19.3537	19.382
ITGA3	21.4783	21.3844	21.394	21.2249	21.1261	21.1009	21.6255	21.4253	21.6763
ITGA4	17.8236	17.222	17.2608	17.2396	17.1615	16.9933	17.2433	17.6963	17.3896
ITGA5	20.0496	19.8913	19.9484	19.8302	19.5622	19.6296	20.1265	20.0147	20.2198
ITGA6	20.3004	20.4303	20.8239	20.23	20.3506	20.038	20.3026	20.1611	20.1871
ITGAV	20.6857	20.5313	20.4207	20.5593	20.4054	20.2431	20.7969	20.4813	20.384
ITGB1	23.4631	23.2153	23.2715	23.1779	23.0684	23.4075	23.655	23.4774	23.4941
ITGB3	19.9524	19.8692	19.8219	19.8605	19.7544	19.9671	20.3443	20.0728	20.407
ITGB8	19.8974	19.7103	19.5664	19.4416	19.5062	20.1333	20.6653	19.5622	19.6826
ITPA	19.9764	19.9334	20.2161	20.0253	20.0554	20.0158	20.1214	19.9923	19.864
ITPK1	19.0103	18.4131	19.1002	18.7831	18.6652	19.6319	19.9706	19.7572	19.3472
ITPR3	18.3337	17.9734	17.9958	17.8311	17.8117	18.1933	18.5105	18.3135	18.1904
IVD	19.5582	19.6957	19.1464	19.1561	19.3135	19.5323	20.0729	19.3777	19.3295
JMJD6	19.079	19.4562	18.9329	19.2958	18.8913	18.5747	18.418	19.0028	19.0195
KARS	23.1281	23.1105	23.1243	23.1046	22.9349	22.7848	23.2468	22.9174	23.0618
KAT7	18.9349	18.271	18.6705	18.6175	17.9763	18.2745	18.3609	18.6514	18.6558
KBTBD3	18.1723	24.7532	17.5492	17.6666	17.7102	17.8605	18.5234	18.1807	18.4301
KCMF1	16.3537	16.3042	16.0694	15.8572	16.2741	13.0319	15.3823	16.3881	16.1181
KCTD5	18.109	17.1551	17.4419	17.2182	17.5885	17.2693	17.5931	17.6823	17.568
KDELC1	16.3063	16.499	16.775	16.8548	16.7979	16.3011	15.6968	16.2445	15.6591
KDELR1	20.1759	20.1468	20.1536	19.7947	20.1885	19.9141	19.0234	19.9743	19.61
KDM1A	20.0523	20.1235	20.3684	20.1825	20.272	20.1288	20.6491	20.4108	20.3754
KDM2A	19.3762	18.3287	18.3895	18.0817	18.1002	17.7154	18.1866	17.9997	17.9548
KDM3B	17.9625	17.8259	17.5804	17.8803	17.628	17.4077	17.443	17.6341	17.4551
KDM5D	16.2971	16.268	16.1466	15.7389	16.4107	16.5328	16.1167	16.3124	15.5888
KDSR	19.2392	19.0385	19.2256	19.0575	19.0375	18.6056	18.9261	18.9332	18.9573
KHDRBS1	23.1544	23.0494	22.6588	22.9297	22.6273	22.7547	22.7472	22.6637	22.9629
KHSRP	23.0818	22.5785	23.1969	23.2537	23.0647	23.3379	23.4093	23.1652	23.2944
KIAA0020	19.1419	19.3145	19.6328	19.2424	19.368	18.3946	19.9637	19.4759	19.1481
KIAA0196	18.5939	18.3362	18.1921	18.5301	18.1842	17.8322	17.9318	17.7404	17.9641
KIAA0391	17.4741	17.4846	17.5255	17.2051	17.2697	17.6679	17.7499	17.4975	17.3138
KIAA1279	19.0867	18.6663	18.7159	18.6973	18.6838	18.6534	19.1723	18.7666	18.9458
KIAA1429	18.0936	18.2715	18.4644	17.9063	18.3571	18.2516	18.4853	18.378	17.9377
KIAA1468	17.3134	16.9122	17.2891	17.1056	17.0455	17.1029	17.5044	17.343	17.4389
KIAA1524	17.7351	17.7838	17.6768	18.3928	17.8372	17.6776	17.451	17.5773	17.6528
KIAA1967	21.6244	21.0849	21.6487	21.613	21.367	21.2266	21.511	21.1403	21.3817
KIAA2013	18.084	17.6617	17.9379	17.724	17.9801	17.4342	18.1157	18.0698	17.9094
KIF11	18.5885	18.7732	18.5765	18.5082	18.3101	18.1337	18.6067	18.5686	18.6562
KIF15	17.9579	17.2901	17.5257	18.1617	17.7878	17.7437	17.5241	17.9471	17.9328
KIF20A	18.0119	17.7323	17.7615	17.7193	17.8592	15.9687	17.1953	17.1596	17.1882
KIF23	18.6664	19.1534	19.139	19.1451	18.9046	18.5079	19.0231	18.9229	19.0344
KIF2A	18.8238	18.8363	18.6949	18.6386	18.4507	18.1738	18.5032	18.3989	18.7036
KIF2C	18.5714	18.4159	18.311	18.3926	18.1657	18.1811	18.1485	18.3357	18.3773
KIF4A	17.4202	18.1866	18.3256	17.4362	17.7214	17.6895	17.8956	17.8122	17.9822
KIF5B	21.5596	21.2303	21.2445	21.3839	21.1696	21.4937	21.9152	21.5142	21.6874
KIFAP3	14.9512	15.835	15.8002	16.3962	15.8474	14.8657	14.8537	15.3872	14.9236
KLC1	20.1515	20.3525	20.1215	20.2789	19.8683	20.2704	20.1173	20.1108	20.5178
KLC2	19.8419	19.356	19.8415	19.7911	19.742	20.0375	20.1939	19.9399	20.1624
KPNA1	18.5533	19.0559	18.6464	18.8616	18.8631	18.6134	18.8506	18.5892	18.9502
KPNA2	22.4153	22.4821	22.6165	22.3035	22.2614	22.2898	22.5309	22.3675	22.5851
KPNA3	20.3238	19.7518	20.0669	20.0559	19.9765	19.8922	20.248	19.8854	20.1212
KPNA4	20.4843	19.6322	20.0398	19.8288	19.9666	20.0401	19.9093	19.7672	19.8323
KPNA6	21.3289	21.3498	21.3321	21.1834	21.1902	21.3302	21.6947	21.5329	21.6021
KPNB1	24.0535	24.0621	24.0016	24.0593	23.6329	23.5919	23.4568	23.7237	24.0981
KRT1	26.7607	20.7552	21.0741	22.0683	21.2554	21.5791	21.7466	21.099	21.1075
KRT10	24.8755	21.1975	21.478	21.3062	21.0877	21.2231	21.0292	21.0891	21.1323

KRT14	22.4517	16.5842	16.6019	16.979	16.7086	16.3744	17.3511	16.542	16.6052
KRT18	17.0927	17.0103	16.9649	17.7172	17.3099	16.8617	16.9675	17.7617	17.5447
KRT2	23.3553	20.0915	20.0155	20.1063	19.9393	20.6258	21.1129	20.52	22.2727
KRT5	21.2824	14.8194	14.6649	13.4372	14.199	16.1066	14.4478	14.1739	14.4654
KRT6C	21.3663	15.1479	13.4911	15.4429	14.5676	13.9941	14.9861	13.8073	14.9163
KRT9	26.5721	19.2654	19.2188	20.7848	19.7685	20.1008	20.7644	19.8747	20.2949
KRTCAP2	18.7172	19.0584	18.7156	18.8787	18.4508	18.3806	18.7258	18.2864	18.8302
KTN1	20.7887	20.6163	20.5262	20.5539	20.3519	20.8173	21.1879	20.6586	20.5539
KYNU	20.2475	20.0756	20.1686	20.1045	20.0359	20.9204	21.4077	21.0838	20.8285
L1CAM	18.1462	18.0486	17.9617	17.9794	17.9531	18.7825	18.9352	18.8309	18.7289
L2HGDH	15.8882	16.5881	16.3085	16.4269	16.6139	16.369	16.2795	16.6357	16.3765
L3HYPDH	19.5848	19.5677	19.3713	19.3913	19.4379	20.3623	20.1844	19.9379	19.8567
LAMA4	17.3562	16.6897	17.0312	17.3399	17.0289	16.6516	16.7822	16.8788	16.6822
LAMB1	20.1952	20.1712	19.8062	19.9295	19.8714	19.5413	19.8329	19.7972	20.1651
LAMC1	19.2153	19.1932	18.8435	19.0785	18.8795	18.3474	18.733	18.6536	18.7605
LAMP1	20.8832	20.148	20.8202	21.0529	20.9193	20.894	21.3066	20.8777	21.2753
LAMP2	20.1086	19.9721	20.1276	20.1505	20.0033	19.8624	20.4619	20.0863	20.5754
LAMTOR1	20.5513	20.3845	20.4107	20.3175	20.3227	20.5666	20.4831	20.2887	20.3033
LAMTOR2	19.6013	19.6563	19.4947	19.486	19.3393	19.0802	19.0994	19.3709	19.5022
LANCL1	20.3707	20.4109	20.4029	20.3713	20.2947	20.7123	20.5321	20.438	20.4814
LANCL2	19.8139	19.903	19.6879	19.8333	19.6468	19.6686	19.5076	19.574	19.6968
LAP3	22.0027	21.9981	22.0818	21.8869	21.8681	22.1171	22.355	21.8441	22.0479
LARP1	18.9101	19.155	19.1936	19.1216	18.9418	18.9224	19.2281	19.1557	19.1557
LARP4	19.7658	19.867	19.8509	20.0619	19.8623	20.037	20.0793	19.8435	20.0741
LARP4B	17.872	17.6424	17.6528	17.6675	17.9385	18.4188	17.7732	17.4825	17.2087
LARS	21.6559	21.6558	21.5538	21.3778	21.2974	21.296	21.588	21.4781	21.4678
LAS1L	19.0893	18.811	18.8391	18.8592	18.6261	18.819	19.0317	18.6712	18.9121
LASP1	20.0659	20.8418	19.988	20.2466	19.9015	20.1393	20.1237	19.7978	20.2109
LCN2	14.6788	15.3566	14.965	16.2416	15.7879	18.8601	18.9424	18.9697	19.2304
LDHA	26.2043	26.1962	26.0597	26.1873	26.0699	26.5387	26.7502	26.3209	26.4823
LDHB	25.678	25.6935	25.6613	25.459	25.4002	25.5774	25.8569	25.5707	25.7416
LDLR	17.6243	17.4172	17.7597	17.5533	17.4739	17.4327	18.0393	17.8554	18.06
LEMD2	18.1919	18.1376	17.525	18.0772	17.3789	17.8823	17.418	17.9099	18.4287
LEO1	20.2499	20.046	19.9166	19.9458	19.822	19.6684	19.9199	19.8977	19.796
LEPRE1	20.6252	20.0939	20.5984	20.5621	20.1897	20.3116	20.8088	20.3692	20.538
LEPREL2	19.2087	18.8323	19.0802	18.7652	18.9369	18.5597	18.549	18.7218	18.5369
LEPREL4	18.5719	18.4958	18.358	18.4634	18.0207	18.0042	18.0467	18.248	18.2027
LETM1	20.0127	19.9669	19.7122	19.8931	19.7256	19.7853	20.1819	19.8801	20.1298
LGALS1	24.327	24.7568	24.3954	24.6745	24.4885	25.0354	25.321	24.8562	25.1489
LGALS3	20.052	20.2642	20.0366	20.1846	20.0312	20.1632	20.5392	20.5374	20.559
LGALS3BP	19.0944	19.4028	19.2781	18.9577	19.2341	19.5484	19.8155	19.5789	19.6797
LGALS8	17.7691	17.6471	17.8859	17.6717	17.724	17.4547	18.0927	17.7145	17.4725
LGMN	15.2677	15.8415	15.5333	16.5853	15.4306	16.1962	13.9731	15.2132	15.167
LIG1	17.1767	17.5788	17.4523	17.7812	17.2987	17.2901	16.5941	17.4654	17.5238
LIG3	19.4809	19.6476	19.6983	19.5136	19.5501	19.5554	19.3562	19.3812	19.4061
LIMA1	21.832	22.3198	21.6625	21.3372	21.4485	21.2581	21.4443	21.2716	21.2822
LIMS1	19.5478	19.5878	19.7269	19.6379	19.4289	19.5724	19.3725	19.3667	19.694
LIN7C	21.6501	22.3097	21.9294	21.9699	21.9205	22.1038	21.5272	21.9049	22.2854
LIPA	19.31	18.5577	18.7085	18.6067	18.7403	16.9069	18.8191	18.6446	18.7995
LMAN1	22.1242	21.7715	21.6411	21.8251	21.3833	21.6572	21.7355	22.0184	22.2507
LMAN2	22.2761	21.9448	22.1844	22.1158	22.1103	22.2897	22.5648	22.1397	22.5222
LMAN2L	18.9415	18.9987	18.8076	18.321	18.4991	18.7056	18.9768	18.8628	18.9211
LMCD1	18.3371	17.8428	18.4228	18.3101	18.1713	18.7688	18.9188	18.7218	18.5538
LMNA	26.0806	25.8168	26.0785	25.8452	25.7273	26.064	26.3064	25.9658	26.2088
LMNB1	23.5926	23.7357	23.6265	23.5706	23.2522	23.221	23.5606	23.3885	23.781
LMNB2	21.6864	22.6737	22.1282	22.162	22.009	21.9545	22.3094	21.9631	22.2141

LMO7	19.2087	18.5876	18.907	18.8121	18.9874	19.0127	19.3316	18.7525	19.0777
LNPEP	18.3309	18.2012	18.0762	18.0236	17.971	17.7158	18.2328	18.2567	18.1546
LONP1	20.9882	20.7932	20.6997	20.4372	20.8213	20.7112	21.2161	20.7811	20.7519
LOXL2	18.9048	18.5404	18.5367	18.4617	18.4677	18.4886	18.5994	18.5814	18.54
LPCAT1	18.1627	18.5272	18.5316	18.492	18.4913	18.7158	18.8377	18.5739	18.5515
LPP	19.3791	19.1836	18.8344	19.2212	18.6035	18.4936	17.8723	18.1677	18.9524
LRCH1	17.6558	17.6554	17.8232	17.6302	16.9711	16.8611	17.3362	17.6312	17.5726
LRPAP1	19.591	19.1072	19.3711	19.3659	19.1892	19.7105	19.6336	19.3623	19.2083
LRPPRC	22.5528	22.3174	22.4497	22.2625	22.3555	22.2646	22.9037	22.5575	22.7763
LRRC1	14.8374	15.2396	15.8251	14.7853	14.886	14.2624	15.1188	14.8548	14.6376
LRRC40	18.9695	18.9935	18.9906	18.7442	18.6015	18.8973	19.0405	18.9227	18.8913
LRRC47	21.029	20.2189	20.8483	20.7333	20.6809	20.6912	21.059	20.7655	20.8274
LRRC59	23.0228	22.8794	22.8134	22.6801	22.5095	22.9632	23.2002	22.7644	22.8424
LRRFIP1	18.895	18.3581	18.6669	18.5717	18.4841	18.8666	19.0908	18.7906	19.1686
LRWD1	19.8664	19.5985	19.6497	19.4808	19.3456	19.2129	19.7199	19.9452	19.7779
LSG1	18.2627	18.423	18.4989	18.5755	18.4752	18.5208	19.0353	18.6213	18.8643
LSM1	18.8599	18.8605	18.8111	18.9181	19.0396	18.727	18.8772	18.587	18.7207
LSM12	19.9358	19.5782	19.6597	19.6333	19.5135	20.0112	20.1534	19.9207	19.9791
LSM14A	16.3623	16.1128	17.4995	17.9153	17.1917	16.9934	17.3132	17.446	17.9591
LSM2	20.6393	20.6587	20.447	20.3632	20.347	20.4584	20.3308	20.3476	20.5712
LSM4	19.5257	19.5129	19.7784	19.5929	19.3635	19.4135	19.3258	19.5404	19.2062
LSM6	19.5739	14.3986	19.5134	19.1027	19.7664	19.8715	20.0925	19.5849	19.6873
LTA4H	21.4507	21.4186	21.3193	21.2512	21.0868	21.3078	21.4516	21.3958	21.4265
LUC7L2	21.8263	21.3695	21.373	21.4022	21.1695	21.5328	21.2861	21.349	21.4314
LUC7L3	20.483	20.4751	20.5286	20.502	20.3628	20.1238	20.731	20.5059	20.7944
LUZP1	19.1503	18.828	18.9971	18.757	18.5909	18.8813	18.6916	18.9124	19.0719
LYPLA1	20.747	20.2892	20.7155	20.6725	20.6042	21.1531	21.2444	20.9181	21.0849
LYPLA2	19.7598	19.211	19.486	19.7796	19.5197	19.5895	19.5167	19.468	19.4622
M6PR	21.6005	21.9215	21.5828	21.7545	21.7026	21.7608	22.108	21.7292	21.8503
MACF1	18.8906	18.5754	18.7542	18.4829	18.6745	18.7794	19.1075	18.9137	18.9248
MACROD1	20.0897	19.2642	19.6961	19.7	19.5722	19.887	19.9682	19.7996	19.9194
MAD1L1	22.708	19.4816	23.0034	23.0873	22.8828	22.0147	22.7303	22.6377	23.1587
MAD2L1	19.657	19.8164	19.6004	19.514	19.5738	20.0082	19.6468	19.4573	19.6264
MAGEA1	17.3917	17.7603	17.6243	17.5681	17.5405	17.0529	17.3968	17.3036	17.1287
MAGEA10	21.4429	21.2126	21.1875	21.4713	21.1063	20.9494	21.2242	21.7185	21.1646
MAGEA3	18.7464	18.4195	18.6357	18.7251	18.4578	18.3027	18.5647	18.1629	18.4292
MAGEA6	19.5184	18.8212	19.456	19.4055	19.3927	19.1852	19.4704	19.5068	19.3829
MAGED2	20.9544	20.7855	20.8041	20.7453	20.6496	20.7214	20.5811	20.668	20.7424
MAGOH	22.7991	22.8011	22.7172	22.664	22.8483	22.6788	22.885	22.6783	22.7269
MAGT1	20.081	19.8027	19.75	19.6493	19.5209	19.3986	19.037	19.259	19.516
MAK16	17.2263	17.6149	17.3252	17.3133	17.39	17.4338	17.5128	17.4445	17.0428
MALSU1	16.7375	15.7424	16.8034	16.4368	16.5452	16.7546	17.1088	16.9146	16.6911
MALT1	18.3364	17.7488	17.5163	17.7852	17.349	17.5959	17.8176	17.6503	17.8529
MANF	22.2164	22.1326	22.0315	22.1371	21.8383	22.2033	22.1021	22.2329	22.3561
MAP1B	21.7028	21.5201	21.5122	21.3584	21.2857	21.4831	21.7097	21.4165	21.6307
MAP1LC3B2	20.6935	21.0145	20.9006	20.987	20.7463	21.0567	20.7387	20.7666	20.733
MAP1S	17.4678	17.4477	17.713	17.5126	17.6629	17.5588	18.0985	17.8399	18.0751
MAP2K1	17.4749	18.5489	17.8598	17.9556	17.6315	17.9013	18.099	17.5775	17.9814
MAP2K2	18.7606	18.2479	18.8644	18.8084	18.8218	18.9235	19.5655	18.8341	18.9895
MAP2K3	15.7512	15.7096	16.0273	17.1039	16.0351	16.9446	16.7666	16.8439	16.4499
MAP4	22.1265	22.035	22.0458	21.9822	21.9163	22.1107	21.9233	22.1004	22.4224
MAP4K4	18.6873	18.7367	18.5988	18.5594	18.3959	18.2932	18.476	18.5294	19.072
MAPK1	22.0128	22.0724	22.1183	22.0789	21.8206	21.8535	22.0399	21.8135	22.0644
MAPK14	19.0936	19.2033	18.9982	18.9303	18.959	19.1612	19.2947	19.0558	18.8197
MAPRE1	22.8176	22.6069	22.5971	22.5439	22.4453	22.9422	22.9805	22.7536	22.7164
MARCH5	18.6931	17.2141	18.5656	18.2623	18.4347	17.9233	18.8382	18.4402	18.4967

MARCKS	20.5254	20.8316	20.5013	20.5735	20.5133	20.3532	20.8659	20.3918	20.6637
MARS	21.6437	21.5421	21.4515	21.3991	21.4883	21.6368	22.0207	21.5348	21.5946
MAT2A	23.1195	23.1292	22.8987	23.0393	22.8052	22.9982	23.085	22.8132	23.0673
MAT2B	20.519	19.8386	20.248	20.1795	20.1715	20.2644	20.568	20.118	20.1763
MATR3	23.7082	23.6071	23.5662	23.662	23.331	23.1062	23.2819	23.2826	23.5653
MAVS	19.8373	19.4163	19.8055	19.7759	19.8145	20.2472	20.5672	20.1274	20.1701
MBD3	17.7566	17.735	17.1486	17.559	17.2567	17.0349	17.2534	17.0474	17.2667
MBNL2	20.863	21.0261	20.9742	20.7972	20.8467	21.1363	21.153	20.9205	20.8985
MBOAT7	19.8996	20.0505	19.8813	19.7607	19.7641	19.5562	20.1055	19.7044	20.1047
MCAM	22.1152	22.1397	21.6835	21.9572	21.5758	21.566	21.6242	21.3974	21.7885
MCCC2	19.0561	18.917	18.8239	18.8525	18.8292	18.8478	19.3352	18.8093	18.9256
MCM2	23.1683	22.8795	23.113	22.91	22.7446	22.3485	22.8824	22.8264	22.77
MCM3	23.1263	22.7705	22.6911	22.8092	22.6411	22.5784	22.9844	22.7278	22.8992
MCM4	23.1528	22.9398	23.0639	22.8465	22.7346	22.674	22.8561	22.7252	22.897
MCM5	22.4547	21.3827	22.7396	22.4281	22.5038	22.5803	22.6157	22.3391	22.3331
MCM6	22.6311	22.8438	22.785	22.4575	22.5489	22.433	22.6264	22.4666	22.8698
MCM7	23.1227	23.059	23.0318	22.8967	22.896	22.969	23.3449	23.0253	23.135
MCMBP	21.617	22.0453	22.2195	21.8193	22.0344	22.4636	21.9163	22.0798	22.0906
MCTS1	17.9019	18.1072	17.7069	18.2534	17.4809	17.3726	17.1289	17.7136	17.6609
MDC1	18.3158	17.711	17.9195	17.9416	17.7643	18.1831	18.2065	18.2078	18.1266
MDH1	23.0554	22.974	23.0934	23.1316	23.1443	23.1646	23.3315	23.095	23.3432
MDH2	24.9194	24.5874	24.4886	24.5714	24.3268	24.7923	25.0301	24.7804	24.9258
MDN1	17.2124	17.3467	17.4863	17.1546	17.4581	16.8411	17.6806	17.2237	17.136
ME1	20.0627	19.5685	19.6559	19.4341	19.5226	19.8188	19.8209	19.9774	19.6569
ME2	21.5474	21.7208	21.5047	21.6216	21.3764	21.5913	21.8734	21.6395	21.9709
MECR	15.7126	16.0224	16.1438	16.5247	16.2282	16.3596	15.2143	16.4744	16.6337
MED14	16.8422	17.0765	17.1245	16.8259	16.9955	16.7447	17.428	16.7721	17.1971
MED20	17.8355	17.4342	17.4413	17.6817	17.2582	17.9819	17.7701	17.79	17.7761
MED23	15.4229	15.9693	15.9136	15.6706	15.7879	15.4581	15.2397	15.5539	15.0101
MEMO1	18.4694	19.6057	19.8621	19.5013	19.5659	19.0002	19.5549	19.6142	19.7302
MEN1	16.2831	16.2702	16.2952	16.5237	16.9159	17.2618	17.8767	16.7556	17.1841
MEPCE	17.8941	17.0864	17.7988	18.2661	18.0273	17.9979	17.9793	17.7754	18.0684
MESDC2	20.7024	20.8224	20.6442	20.358	20.3641	20.6097	20.5134	20.6162	20.8188
METAP1	21.1167	20.6681	20.855	20.6431	20.5094	20.5729	20.7245	20.8664	20.8265
METAP2	21.5465	20.9495	21.6223	21.5667	21.6665	21.5833	21.5471	21.7391	21.5001
METTL13	19.6489	19.1221	19.2051	19.1034	19.082	18.9338	19.1798	19.2493	18.7422
METTL16	19.016	18.5524	18.4348	18.595	17.9379	18.0815	17.8198	18.3601	18.6674
MFAP1	19.5174	19.5913	19.4507	19.59	19.3574	19.3121	19.6218	19.3265	19.6617
MGEA5	17.906	18.0108	18.1024	18.0711	18.1153	17.972	18.2722	18.1025	18.0034
MGLL	17.9989	17.626	17.7727	17.7959	17.9125	15.0329	17.6336	17.742	17.0159
MGME1	18.3427	18.3255	18.2351	18.1569	18.1211	18.3281	18.1296	17.9822	18.0386
MGST1	21.5035	21.7529	21.3243	21.5087	21.3413	22.0145	22.0464	21.6959	22.1028
MGST3	20.3114	20.8018	20.6193	20.3742	20.7006	20.368	19.9106	20.0604	20.4197
MIA3	17.4172	17.5443	17.3463	17.1632	17.3108	17.6332	17.7814	17.6221	18.1134
MICAL3	18.7637	18.5772	18.5692	18.4331	18.314	18.4432	18.8328	18.7984	18.7629
MICALL1	18.4495	17.8044	18.504	18.2369	18.4851	18.3932	19.0693	18.7484	18.7986
MIF	24.5583	24.2171	24.304	24.4538	24.0043	24.6972	24.7014	24.5437	24.651
MINA	18.1549	18.2482	18.3781	17.4229	17.9719	17.1167	17.6836	17.8936	17.9811
MINPP1	17.6568	17.35	17.3621	17.3248	17.5569	19.4189	16.9625	17.3845	17.2547
MIPEP	17.8665	18.0706	18.0089	18.2436	17.8616	18.0993	18.0157	18.0662	18.186
MKI67	18.365	18.4913	18.3532	18.355	18.2256	18.415	18.6096	18.0319	18.2508
MKI67IP	18.8819	18.9301	18.6537	18.5839	18.457	18.4931	18.1216	18.5818	18.7598
MLEC	21.7946	21.6963	21.9165	21.7368	21.4754	21.3308	21.6063	21.4524	21.7994
MLH1	15.842	16.4803	16.0363	16.1957	17.4589	16.3757	16.7777	16.0871	16.7046
MMS19	19.8432	19.4674	19.5235	19.5269	19.4213	19.3359	19.5844	19.3654	19.216
MMTAG2	16.4135	17.8663	18.0934	18.1463	17.2389	17.9296	18.1008	16.9618	18.2217

MNAT1	19.4498	19.5949	19.3829	19.1071	19.3025	19.556	19.4976	19.0369	19.5906
MOB1B	20.324	20.3225	20.4445	20.1458	20.1173	19.9308	20.1967	19.9152	20.0573
MOB4	17.6748	17.3499	17.09	16.6612	17.088	15.9558	17.5871	16.9301	16.603
MOCS2	15.802	16.4219	16.227	17.0184	16.865	16.249	15.6855	16.4561	15.5392
MOCS3	17.1965	17.5757	17.8835	17.3928	17.379	18.2124	17.6521	17.5955	17.9646
MOGS	21.6278	21.4591	21.4857	21.308	21.1321	21.4827	21.5508	21.4471	21.4023
MON2	18.5477	18.2799	18.2957	18.0394	17.8685	17.7878	18.4012	18.024	18.3911
MORF4L1	18.7097	18.8752	18.875	18.8386	18.5096	19.0927	19.1526	18.9882	18.9129
MOV10	18.4236	17.8794	18.2795	18.2763	18.048	18.148	18.3653	18.2801	18.2038
MPDU1	20.059	19.8952	19.8025	19.6897	19.7151	19.6056	19.6746	19.671	19.457
MPHOSPH10	16.9295	17.4549	17.7469	18.0913	17.8814	17.697	16.8307	17.5028	17.7107
MPHOSPH8	16.6006	16.887	16.8174	16.4557	16.7127	16.9652	17.0122	16.9982	16.4414
MPP5	15.4648	16.8137	16.4974	16.4848	17.0353	16.8465	16.9457	16.4422	16.655
MPP6	18.7002	19.5986	19.5147	18.6741	18.6756	19.9622	20.3671	19.8664	20.1037
MPRIP	17.4712	17.4914	17.6198	17.5288	17.3779	18.0152	17.6999	17.9467	18.0755
MPST	20.6921	20.6189	20.1718	20.7339	20.6951	18.5965	19.6874	20.583	20.0596
MRC2	16.2892	16.5617	16.4467	16.195	16.4145	14.1798	16.6309	16.3081	16.3671
MRE11A	18.5664	18.4019	18.4641	18.524	18.1653	18.1796	18.5623	18.4811	18.5095
MRI1	18.7084	18.4046	18.5365	18.5795	18.4506	18.6095	18.2746	18.6266	18.621
MRPL1	18.9728	19.0337	19.1504	19.5146	19.0715	19.1778	19.5378	19.1255	19.0995
MRPL10	19.1761	19.1323	19.1266	18.5369	18.8679	18.572	18.9315	18.9571	18.8757
MRPL11	19.0783	19.2042	19.5102	19.0401	19.0818	19.2666	19.5331	19.0839	19.1908
MRPL13	19.8762	19.7103	19.7225	19.5977	19.6376	20.1399	20.2363	19.9695	20.0902
MRPL15	18.4327	18.7616	18.1729	18.0223	18.2091	18.9211	19.1359	18.7736	18.9072
MRPL16	18.4492	18.6714	19.1103	19.1314	19.2621	19.3776	19.5526	18.5861	19.214
MRPL17	19.6946	19.5162	19.4189	19.3942	19.2359	19.5861	19.8551	19.4033	19.4771
MRPL21	19.5323	18.6673	19.506	19.7372	19.612	19.9945	19.8308	19.5138	19.4876
MRPL24	19.4934	18.0471	19.5303	19.7356	19.5196	19.7535	20.11	19.6612	19.9077
MRPL28	16.8412	17.4148	17.6951	16.9554	17.2461	17.2303	17.942	17.5689	17.5954
MRPL3	19.2854	19.1984	18.9138	19.1384	19.0523	19.0374	19.099	19.0663	18.8882
MRPL37	20.2673	19.9535	20.0886	20.0824	19.9156	19.9478	20.2253	20.2321	20.2819
MRPL38	19.5026	19.5347	19.4745	19.3075	19.0513	19.4742	19.9569	19.6277	19.5873
MRPL39	19.5356	19.4363	19.38	19.1333	19.2781	20.0221	19.284	19.1522	19.0252
MRPL4	19.9631	19.8892	19.838	19.8093	19.5932	19.955	20.295	19.8903	19.7855
MRPL44	18.9585	18.8373	18.781	19.0266	18.7062	18.8277	18.8704	18.9876	18.8353
MRPL45	20.3216	20.3967	20.2729	20.3886	20.3123	20.1619	20.0897	19.9367	19.9378
MRPL47	18.1993	18.0759	17.8354	18.0985	18.0859	18.0895	17.7889	18.3719	18.2397
MRPL48	18.1399	18.508	18.8097	18.5779	18.245	18.2225	18.8183	18.6047	18.9061
MRPL49	18.8363	18.7341	18.5282	18.7755	18.5866	18.5091	19.3116	18.7688	18.8811
MRPS10	18.8066	18.4169	18.4796	17.9707	18.0768	18.4217	18.3328	18.222	17.8729
MRPS17	19.5817	19.165	19.031	19.3277	19.1597	19.7371	19.6237	19.6671	19.0758
MRPS2	19.3551	19.9265	19.5267	19.3874	19.4537	19.5545	19.9153	19.5257	19.6988
MRPS22	19.8902	20.1455	19.9484	19.9115	19.7908	19.9553	19.8677	19.9578	20.1396
MRPS23	20.1072	20.1697	19.9441	19.9222	19.6186	19.9439	20.2063	19.8995	19.9523
MRPS27	18.7112	18.0936	18.444	17.866	18.4302	18.8265	18.9151	18.5843	18.1114
MRPS28	19.2649	18.2031	19.3886	19.7934	19.4611	19.6895	19.8979	19.527	19.816
MRPS30	19.3786	18.5204	19.0913	18.7588	18.69	18.8234	18.9168	18.6416	19.0073
MRPS34	19.623	19.5768	19.7188	19.2682	19.3493	19.3513	19.8149	19.6434	19.6477
MRPS35	19.0418	18.7777	18.8536	18.9948	18.8689	18.7044	18.8225	18.9394	19.0104
MRPS6	19.3612	19.1583	19.1589	19.0882	18.9831	19.3247	19.1724	19.2217	19.2079
MRPS9	17.8308	17.8938	18.0142	17.6589	17.6336	17.3452	17.9888	17.9668	17.9903
MRTO4	20.9441	21.1116	20.8665	21.1211	20.8024	21.1228	20.8618	20.883	21.0017
MSH2	21.01	21.055	21.1989	20.9697	20.8729	20.5452	21.0813	20.8259	20.9299
MSH6	21.3782	21.6876	22.2419	21.1985	21.9134	21.2102	22.0863	21.1005	21.3316
MSI2	20.0796	19.6382	19.6756	19.7454	19.8494	19.9248	19.5807	19.8222	19.6336
MSN	22.8147	23.3278	22.8331	22.6751	22.615	22.9223	23.1945	22.8799	23.2016

MSTO1	19.0337	17.3742	19.2855	19.2116	19.4638	19.3289	19.9812	19.4649	19.4738
MTA1	18.1724	17.9783	18.0246	17.8815	17.8093	17.9588	18.1457	18.1779	18.2247
MTA2	21.4924	21.7677	21.286	21.2077	21.0811	21.1833	21.5883	21.2346	21.561
MTAP	20.7184	20.311	20.3403	20.3774	20.3537	20.6485	20.6693	20.4084	20.3061
MTCH2	20.9988	21.4661	21.6321	20.8601	21.7336	21.1675	22.3421	20.8623	21.0734
MT-CO2	21.6742	21.6661	21.4921	21.5766	21.3798	21.6969	22.2173	21.7974	21.9085
MTDH	19.9665	19.8882	19.8631	19.7876	19.6133	19.5793	19.7384	19.8051	19.9516
MTHFD1	22.523	22.5563	22.8016	22.382	22.3898	22.5936	23.0237	22.6163	22.8466
MTHFD1L	22.3362	20.2163	22.0744	22.0835	22.0081	22.167	22.5481	22.0903	22.5558
MTHFD2	22.406	22.3812	22.2897	22.2803	22.3321	22.4451	22.7525	22.3228	22.495
MTIF2	16.5021	16.7052	16.9774	16.0944	16.5336	16.2205	16.6437	16.8248	16.3851
MT-ND4	17.6922	17.6031	16.6026	17.4702	17.7987	17.3126	16.6021	17.3822	17.7243
MTOR	18.1223	17.9481	17.8325	17.6559	17.8163	17.7179	17.6668	17.7277	17.6171
MTPN	21.3483	21.3564	21.3892	21.6349	21.5639	21.9463	22.3246	21.6398	22.0234
MTX1	18.8744	18.3832	18.5785	18.6228	18.2649	18.5933	18.7219	18.7005	18.5052
MTX2	18.2326	18.3668	18.1813	18.1408	18.0382	18.2011	17.4991	17.9421	18.1212
MUT	18.0891	17.5558	17.8823	17.9751	17.7531	17.8744	18.1071	18.0128	18.0366
MYADM	20.3305	20.5057	20.3221	20.4056	20.243	20.3751	20.6925	20.3043	20.4715
MYBBP1A	20.0328	20.046	20.3122	20.1942	19.8425	20.0821	20.3993	19.9776	20.2855
MYD88	15.2173	14.9177	15.4185	15.2392	14.8762	16.0157	15.0642	15.2719	15.233
MYH10	21.1913	20.7522	20.8083	20.5988	20.6763	20.6741	20.9485	20.5315	20.6902
MYH9	24.2292	24.2411	24.1723	24.097	24.0772	24.1745	24.3297	24.0206	24.1321
MYL12A	22.9222	22.974	22.7958	22.9905	22.7892	22.9349	23.0762	22.945	23.315
MYL6	22.9135	23.0209	23.0234	22.951	22.8763	22.9854	23.4447	23.0194	23.3619
MYO18A	17.4032	17.4038	17.371	16.7946	16.5266	16.8224	16.1194	16.9298	16.9596
MYO19	16.4489	17.3034	17.8193	16.2121	16.9384	16.1332	18.18	16.0968	16.4922
MYO1B	19.1209	19.6753	19.8247	19.5802	19.2638	19.4043	20.0372	19.4034	19.5678
MYO1C	21.5886	21.8335	21.568	21.5674	21.1816	21.4123	21.9046	21.645	21.7755
MYO1E	17.7415	17.4661	17.9815	18.0159	17.9885	17.4179	17.7448	17.8125	18.0692
MYO5A	16.1352	16.5662	16.7309	16.3478	16.5957	15.2167	16.6864	16.5122	16.346
MYO6	16.3974	16.2034	16.3233	16.5603	16.1664	14.8984	14.2823	14.6076	14.1839
MYOF	22.1637	21.9962	21.9871	21.8606	21.9549	21.6752	22.2027	21.8922	21.813
NAA10	18.5655	17.9739	17.5887	18.6749	17.5261	17.5192	16.382	17.5141	18.4475
NAA11	16.6013	16.7708	16.2193	17.5879	15.8907	15.8845	13.9506	15.622	15.9181
NAA15	21.1156	21.1609	21.4631	21.1241	21.1948	20.8852	21.3498	20.9598	21.1938
NAA25	17.4212	18.3498	18.106	18.013	17.7979	18.1827	18.0565	18.1108	18.4572
NAA38	18.7639	19.1379	19.0005	19.1466	18.8457	19.2355	19.0649	19.3707	18.9627
NAA50	20.6739	20.7235	20.778	20.7479	20.612	20.91	20.931	20.5932	20.6572
NACA	22.8965	22.8388	22.9699	22.8732	22.8729	22.743	23.1817	22.8025	22.9879
NACC1	17.588	18.521	18.2112	18.4567	18.3266	18.4491	18.6599	18.314	18.5027
NAE1	19.9444	19.9552	19.944	20.0442	20.0275	19.9726	19.8653	20.1473	20.0572
NAGK	18.6124	19.176	19.4364	19.0361	19.0357	18.8188	18.9712	18.8479	18.8763
NAMPT	23.2348	23.0261	23.0322	23.047	22.9496	23.0025	23.2768	23.0947	23.1373
NANS	20.9707	20.6109	20.5254	20.4944	20.401	20.744	21.0377	20.7788	20.9342
NAP1L1	23.1753	23.022	23.0715	23.0744	22.8702	23.0028	22.9293	23.044	23.0523
NAP1L4	21.9753	22.1717	22.0621	22.2836	21.9847	22.2159	22.3994	22.0845	22.2079
NAPA	21.4794	20.9106	20.9444	20.8485	20.7187	20.8557	21.0322	20.9527	20.9871
NAPG	17.6611	18.5085	18.8091	18.3648	18.4453	18.392	18.3499	18.0989	18.4397
NARS	23.7818	23.5024	23.5597	23.9538	23.5426	23.4122	23.6381	23.6931	23.5736
NARS2	17.9387	18.3006	17.7398	18.1152	17.6665	17.6169	16.9376	17.5484	17.9552
NASP	21.5118	20.7747	21.6355	21.9537	21.9193	22.0915	22.4607	21.7028	22.0494
NAT10	18.6734	19.0781	18.9045	18.623	18.9839	18.6926	19.2989	18.8332	18.7576
NBN	17.49	18.1754	17.4389	17.7323	17.7591	17.2098	17.9292	17.5117	17.831
NCAPD2	21.175	21.0148	21.2107	20.9104	20.9637	20.8424	21.2835	20.863	21.0531
NCAPD3	17.4514	17.4611	17.6046	17.6623	17.4399	17.4218	17.3455	17.4426	17.5225
NCAPG	22.4382	22.3408	22.262	22.5256	22.4165	21.8827	22.2617	22.1536	22.7087

NCAPG2	18.3316	18.3777	18.2998	18.3448	18.3889	17.944	18.5029	18.1175	18.1423
NCAPH	20.0107	21.7213	19.9387	20.2375	19.7438	19.7098	19.8385	19.7155	19.8513
NCBP1	22.9053	21.4587	22.9473	22.994	22.8448	23.018	22.7007	22.8886	23.1512
NCDN	19.1286	19.423	19.3473	19.2945	19.1667	19.1195	19.2652	19.3277	19.079
NCEH1	21.9717	22.0887	22.9036	21.5551	22.6381	22.8726	22.5367	22.7793	22.2042
NCK2	17.7852	17.2312	17.8875	17.5106	17.5696	17.6422	18.1798	17.9081	18.2329
NCKAP1	19.7551	19.6344	19.7652	19.5063	19.7564	19.4375	19.7059	19.6649	19.5693
NCL	25.3468	25.6456	25.5987	25.4878	25.2239	25.3185	26.008	25.4335	25.7068
NCLN	20.4844	20.4373	20.3538	20.3014	20.1055	20.3396	20.4926	20.2725	20.2031
NCOA5	19.3204	18.3848	18.8653	19.2418	18.5535	19.3393	18.6782	18.8265	19.1554
NCSTN	19.928	19.7777	19.812	19.611	19.5675	19.4426	20.0015	19.5812	19.8158
NDRG1	19.8247	19.7923	19.678	19.9305	19.4656	19.6616	18.7643	19.4811	19.6869
NDRG3	20.5945	20.676	20.6409	20.4265	20.4864	20.8116	21.2657	20.4904	20.5602
NDUFA11	19.2366	19.5098	19.2127	19.0398	19.3231	20.071	19.4519	19.4739	19.5581
NDUFA13	19.4283	19.4287	19.376	19.0089	19.2837	19.5377	19.9381	19.4983	19.592
NDUFA4	21.7381	21.2042	21.2278	21.213	21.146	21.3892	21.3764	21.4644	21.3634
NDUFA5	20.0425	19.646	19.5753	19.6822	19.5007	19.8599	19.8342	19.7296	19.7694
NDUFA8	19.7657	18.8604	19.3816	19.5739	19.2438	19.7815	20.047	19.6968	19.7326
NDUFA9	19.9431	19.6125	19.442	19.5193	19.6447	18.9725	19.636	19.5387	19.606
NDUFAF3	18.2928	17.1131	18.3427	17.8368	18.2081	18.5033	18.7224	18.5327	18.5345
NDUFAF7	18.249	18.2963	18.0463	18.1515	17.9385	18.0824	18.5677	18.283	18.631
NDUFB10	20.4139	20.2303	20.0616	19.6789	19.9856	20.1645	20.462	19.9404	19.847
NDUFB11	20.4961	19.9804	20.1692	20.2189	20.1916	20.3168	20.2836	20.3312	20.6751
NDUFB3	20.0408	20.4603	20.169	20.1128	19.9331	20.2335	20.22	20.1262	20.4702
NDUFS1	20.4569	20.1861	20.2606	20.4199	20.2224	20.1304	20.3598	20.383	20.4813
NDUFS2	20.1573	20.5668	20.5909	20.3633	20.5046	20.2275	20.4406	20.1165	20.4029
NDUFS3	21.4666	21.393	21.2265	21.2002	21.0948	21.232	21.4997	21.359	21.522
NDUFS5	19.0967	19.9479	19.6692	19.5478	19.3953	19.7241	20.0108	19.6427	19.8824
NDUFS8	18.5247	18.7934	18.4606	18.488	18.5099	17.6292	18.1595	18.7548	18.2905
NDUFV1	21.6056	20.9324	20.9061	20.5275	20.7666	20.731	21.1368	20.9618	20.9926
NDUFV2	19.577	19.4617	19.4652	19.2427	19.5473	20.004	20.3687	19.8489	19.9907
NECAP1	18.1868	18.4842	18.2994	18.2732	18.2546	18.573	19.0552	18.4728	18.5307
NECAP2	18.2706	18.8213	18.0758	18.348	17.8598	17.7592	17.1758	17.7466	18.2759
NEDD4L	18.6804	18.4936	18.7928	18.5661	18.5451	18.5858	18.9123	18.7224	18.5226
NEDD8	19.9311	19.537	19.9407	19.5488	20.0635	20.0521	19.9968	19.7471	19.5499
NELFA	17.6681	17.3202	17.5549	17.0835	17.2766	17.2617	17.9524	17.3327	17.6134
NELFB	19.0975	19.1182	19.0165	19.0924	19.0308	18.1213	19.1073	18.9517	18.5119
NELFCD	18.9284	18.9953	19.1269	18.87	18.9156	18.9982	19.1639	19.1716	19.0267
NELFE	17.9162	16.9276	16.874	17.0798	16.5104	17.2247	17.0349	17.4448	17.7378
NES	24.2206	24.1463	24.063	23.9504	23.8777	23.7551	24.0666	23.7819	24.1676
NEU1	18.1985	18.5227	18.3345	18.8697	18.0794	18.8024	19.3081	18.9966	19.0991
NFIC	19.0385	18.2295	19.4153	19.345	18.8813	18.1719	18.8331	18.7247	18.7815
NFKB1	19.8477	19.4107	19.7014	19.544	19.559	20.032	20.0104	19.9028	19.7418
NFKB2	20.2498	22.7434	20.2317	20.1117	20.4403	20.4831	20.816	20.5742	20.5747
NFS1	19.0346	17.5578	18.3064	18.5658	18.0261	18.4831	18.6066	18.0398	18.5019
NFYC	17.6439	16.7775	17.9297	17.7533	17.9597	17.9042	18.1176	17.9077	17.6553
NGFR	16.3788	16.9156	16.8364	16.7012	16.8838	13.0903	11.6293	12.6969	13.7169
NHLRC2	19.6249	19.435	19.3997	19.4842	19.3696	19.3894	19.6225	19.212	19.3282
NHP2	20.1786	20.1096	19.8631	19.863	19.6918	20.0977	20.2669	19.903	19.8451
NHP2L1	22.1265	21.865	22.1048	22.2121	21.7594	22.2304	21.9359	21.9105	22.284
NIF3L1	19.6372	19.8289	20.116	19.2671	19.6892	19.5182	19.5333	19.4037	19.8843
NIP7	20.6833	20.6844	20.8822	20.5576	20.5518	20.7697	21.1842	20.7584	20.7219
NIPSNAP1	18.975	19.5256	19.128	19.6614	19.3928	19.6797	19.4912	19.6254	19.6795
NIT1	18.7101	18.6242	18.5904	18.2597	18.4916	18.7956	18.8388	18.8283	18.7223
NIT2	20.1037	19.9284	20.0016	20.075	19.9478	20.0123	19.9537	19.9114	19.8707
NLE1	19.4023	19.0784	19.1344	19.1934	18.9672	19.1826	19.0431	19.1325	19.1409

NLN	20.2363	19.957	19.9206	19.8203	19.9066	20.2598	20.2043	20.0515	19.9868
NMD3	19.0432	19.4637	19.3157	19.2652	19.5024	18.8694	19.2774	19.0187	19.2655
NME1	23.845	23.9655	23.882	23.997	23.6551	23.8289	23.8145	23.8921	24.0803
NME2	24.7119	24.7839	24.4304	24.6225	24.5504	24.7442	24.8902	24.5071	24.6417
NME3	17.8809	17.9925	17.587	17.5303	17.6315	18.0383	18.4425	18.0788	18.28
NMT1	20.6372	20.6551	20.485	20.4496	20.4597	20.6118	20.7383	20.3692	20.4361
NNMT	18.6282	18.9702	18.8999	19.1866	18.597	18.2911	18.0035	18.4371	18.6385
NNT	20.3048	20.5423	20.5156	20.4426	20.1255	19.9849	20.5582	20.2154	20.4309
NOB1	18.53	18.7946	18.8654	18.7046	18.6121	18.7126	18.8132	18.6299	18.6085
NOC2L	20.1803	20.714	20.452	20.4731	20.3938	20.2483	20.7273	20.4183	20.6149
NOC4L	18.4877	18.4854	18.3708	18.0825	18.172	18.0626	18.1539	18.0249	17.9026
NOL10	17.5908	17.4362	17.9624	17.3104	17.7314	17.5532	18.3088	17.4504	16.973
NOL11	19.4845	19.3907	19.3709	19.2664	19.057	19.6266	20.067	19.5572	19.5501
NOL6	20.146	19.6737	20.3518	20.4871	20.2939	20.2408	20.6965	20.3661	20.5875
NOL8	13.0547	14.9032	15.0671	14.5716	14.4122	14.8813	14.6075	14.9656	13.9328
NOLC1	20.9646	20.0574	20.8959	21.0467	20.9831	21.5205	21.7765	21.0436	21.3274
NOM1	16.3984	16.6413	14.974	15.6215	16.2327	15.3313	16.2223	16.2387	16.03
NOMO3	20.5415	21.0147	21.1306	21.0498	20.6345	20.8779	21.0411	20.8191	21.0296
NONO	23.2458	24.4196	22.8666	23.1085	22.5375	22.8981	22.589	22.8568	23.1793
NOP14	17.9551	17.5926	17.4146	17.3406	17.4755	17.2091	17.0179	17.7106	17.7757
NOP16	19.4467	19.5672	19.4616	19.4035	19.266	19.3127	19.6041	19.4114	19.396
NOP2	19.95	20.0171	19.8737	19.9842	19.7071	19.8146	19.8994	19.8329	19.8398
NOP56	21.1547	20.2588	21.5277	20.5369	20.422	20.8413	21.6896	21.1546	21.0751
NOP58	20.8595	20.7809	20.6989	20.5627	20.3497	20.5861	20.6889	20.5868	20.7907
NOP9	17.6111	17.1379	17.3992	17.6044	17.4349	16.7108	17.6039	17.3656	17.4779
NOSIP	19.8048	19.433	19.5253	19.3558	19.3354	19.4413	19.5503	19.3007	19.292
NPC2	18.9239	18.7241	18.6674	19.3446	19.0932	19.6394	19.4034	19.4384	19.9618
NPEPPS	21.5601	21.5185	21.4571	21.2869	21.2027	21.3246	21.6891	21.4847	21.5711
NPLOC4	20.9241	20.3736	20.6258	20.553	20.3463	20.6961	20.6838	20.8058	20.8511
NPM1	24.7322	24.9064	25.2249	24.0754	25.2225	25.0959	25.4757	24.5416	24.9942
NPM3	20.3974	19.4092	20.2633	20.3721	20.3313	20.6783	20.2772	20.3452	20.5486
NPTN	19.8534	19.9772	20.1872	20.0281	20.0415	20.032	20.2326	19.832	20.1001
NQO1	24.0659	23.5273	23.8076	23.9011	23.5279	23.9786	24.2478	24.1418	24.2575
NR2F2	18.3544	23.0239	18.5817	18.8624	18.4714	18.9906	18.7933	18.5452	19.0058
NRD1	23.5329	23.5565	23.5295	23.4147	23.3485	23.6393	24.1135	23.6099	23.5794
NRP1	17.9612	18.115	17.9741	18.1097	17.4858	17.6642	18.1141	17.9891	18.2241
NSA2	19.6209	19.4852	19.0124	19.4632	19.0711	18.8662	19.2177	19.515	19.187
NSDHL	20.4476	20.4096	20.4181	20.3213	20.1974	20.2039	20.4884	20.2192	20.2291
NSF	21.4237	21.0414	20.8235	20.7196	20.7519	20.1522	20.7507	20.4988	20.6631
NSFL1C	20.7521	20.438	20.5499	20.5567	20.5427	21.0369	21.02	20.7396	20.8197
NSUN2	21.4448	21.297	21.1494	21.1854	20.9059	21.2091	21.5416	21.3402	21.4883
NT5C	16.8408	17.3077	17.2734	17.1508	17.2503	17.1269	17.0733	17.23	17.2919
NT5C2	16.9098	17.2016	17.4269	18.2291	17.343	16.5301	17.1964	17.269	17.0982
NT5DC1	17.6229	17.9151	17.7668	17.7756	17.9175	17.1216	17.3048	17.4623	17.5172
NT5DC2	19.7498	20.1154	20.0432	19.6453	19.9194	19.8642	20.3493	19.9307	20.1226
NT5E	21.2692	20.9487	21.0743	20.8424	20.8581	21.2477	21.6003	21.3162	21.395
NTMT1	18.4444	17.6588	17.7874	17.837	18.0651	18.6299	18.161	18.1795	17.8705
NTPCR	17.8346	18.4338	18.3333	18.2599	18.1852	18.2466	18.4125	18.4728	18.2535
NUBP1	18.9047	19.0768	18.9599	19.1181	18.8939	19.2095	19.3038	18.9993	18.9171
NUBP2	18.8354	18.97	18.8412	19.0439	18.6855	18.8611	18.5732	18.6775	18.8088
NUCB1	18.7516	19.9597	18.8076	19.3871	18.4677	18.9606	18.2139	18.5681	19.2437
NUCKS1	19.5524	18.9697	18.8713	19.1755	18.5887	18.3438	17.5595	18.7354	18.97
NUDC	22.935	22.9807	22.8826	22.8929	22.6074	22.3057	22.0519	22.6335	23.0261
NUDCD1	19.7995	20.0432	20.0352	20.0437	19.8269	19.6103	20.0282	19.7738	19.7739
NUDCD2	16.7302	17.7204	17.5691	18.2292	17.7766	17.7089	17.6133	17.7937	18.1076
NUDCD3	18.0807	17.6347	17.5923	18.1219	17.4628	16.8005	16.8998	17.5252	17.7055

NUDT15	16.177	16.1188	12.6057	16.5841	16.5814	16.4684	15.7646	15.853	16.8408
NUDT21	22.2982	22.0813	22.4274	21.9261	21.9252	21.8255	22.2752	22.117	22.281
NUDT4	19.2549	19.2341	19.3787	18.9391	19.0836	19.5367	19.7875	19.2341	19.4949
NUDT5	21.7294	21.6718	21.7589	21.5258	21.6112	21.624	21.9392	21.6188	21.5852
NUFIP2	19.8204	19.8781	19.9509	19.7331	19.7439	19.6886	19.9333	19.8775	19.9661
NUMA1	21.4549	23.668	21.0426	21.1947	21.1524	21.3306	21.614	21.1978	21.4749
NUP107	19.3742	19.7839	19.7419	19.7069	19.7632	19.8388	20.0243	19.702	19.8551
NUP133	19.6844	19.9566	19.3402	19.3731	19.314	19.3477	19.7191	19.2272	19.5336
NUP153	20.0643	20.1982	20.1595	20.0873	19.7419	20.1445	20.566	20.133	20.2095
NUP155	20.9619	20.7877	20.6488	20.6346	20.6635	20.372	20.8461	20.6788	20.6307
NUP160	19.6145	20.4581	19.8878	20.1222	20.2839	20.0002	19.8273	20.0332	19.9864
NUP188	21.8286	22.013	21.9197	21.6861	21.7071	22.2623	21.1005	21.9483	21.7104
NUP205	21.2029	21.0073	21.2732	21.0518	20.918	20.8773	21.2867	20.7725	21.047
NUP214	19.1884	18.7072	18.7023	18.9743	18.3097	19.137	18.8668	18.7793	19.1975
NUP35	20.0008	19.7551	19.9461	19.9865	19.8589	20.0748	19.9428	19.9683	20.0026
NUP37	20.1633	19.5594	19.7739	19.587	19.6695	19.9462	20.2322	20.0994	20.1202
NUP43	20.3559	20.0423	20.2233	19.9605	19.9424	20.2283	20.6125	20.1031	20.128
NUP50	20.8415	21.2769	20.5347	20.7293	20.5476	20.5273	20.4284	20.5739	20.8982
NUP54	19.6125	19.4358	19.4045	19.1253	19.3366	19.3368	19.6543	19.3289	19.3321
NUP62	18.9087	18.816	18.8048	18.8513	18.8364	18.3344	18.1679	18.9234	18.6506
NUP85	19.2623	19.4364	19.2474	19.1719	19.1901	20.0867	19.2251	19.1367	19.2181
NUP88	19.6909	19.8917	19.9261	19.6867	19.4773	19.6736	20.0379	19.813	19.9787
NUP93	20.8939	21.1354	21.0286	21.0921	20.965	20.8639	21.4411	21.1621	21.536
NUP98	21.1165	20.9325	20.9727	20.8856	20.8638	20.9061	21.0855	20.8311	20.9914
NUPL1	20.3459	19.9965	20.1855	19.8622	19.8441	19.5832	19.9648	19.9775	19.9604
NUTF2	19.6349	19.7844	19.608	20.1632	19.6123	19.4809	18.1936	19.6425	19.4992
NXF1	20.0015	20.0625	19.9716	19.6901	19.6157	19.5825	20.0265	19.7641	19.9386
OAT	21.7415	21.6363	21.8553	21.3532	21.504	21.2956	21.8925	21.5015	21.5201
OCIAD1	20.8907	19.4421	20.9792	20.6872	21.0233	20.9907	21.2889	20.8819	20.9661
ODR4	18.8981	18.4844	18.602	18.4912	18.3655	18.0082	18.4906	18.3785	18.6832
OGDH	20.3007	21.3557	20.0597	20.4147	19.7857	20.174	20.1523	19.954	20.5113
OGFOD1	18.8202	18.4618	18.7047	18.9316	18.841	19.0156	18.2161	18.7505	18.2929
OGFR	19.6919	19.3192	19.5885	19.4859	19.4996	19.6426	19.8117	19.6082	19.5955
OGT	19.4088	19.482	19.5111	19.0591	19.0321	19.2509	19.5628	19.2551	19.6233
OLA1	22.2414	22.1476	22.2552	22.148	21.9357	21.7612	22.3662	22.0867	22.0965
OPA1	21.0081	20.6458	20.6787	20.4465	20.3196	20.4727	20.5702	20.5401	20.6692
ORC1	13.6603	20.0193	14.4998	14.9185	15.094	16.7133	15.6742	15.6955	15.1255
ORC2	17.5837	17.6762	17.3988	17.7012	17.2056	16.3355	17.0059	17.7102	17.2023
ORC3	18.2128	17.5098	17.9796	17.7528	17.9108	17.1059	18.2644	17.9212	17.5311
ORC4	18.4545	18.1956	18.6128	18.4764	18.5913	18.2757	19.0065	18.5242	18.1869
ORC5	18.2989	18.1975	17.8897	17.6244	17.4935	17.277	17.447	17.4431	17.5833
OSBP	18.5989	18.4668	18.3545	18.6627	18.2618	18.5878	18.694	18.6323	18.7504
OSBPL10	19.4596	18.9285	19.1451	19.0544	19.0084	18.8388	19.2627	19.1762	19.0283
OSBPL3	18.2185	16.4494	17.792	18.4502	18.1467	18.468	18.8535	18.3231	18.9566
OSBPL8	18.3906	18.4427	18.461	18.5258	18.3851	18.102	17.936	18.5423	18.3779
OSBPL9	18.1219	17.9548	18.1207	18.0479	18.0279	17.4359	17.3826	17.8283	17.4245
OTUB1	21.9615	21.297	21.9362	21.9396	21.8384	21.9487	22.2409	21.8651	22.0474
OVCA2	19.6064	19.498	19.894	19.4818	19.3679	19.7472	19.6796	19.6249	19.6245
OXCT1	19.5042	19.3375	19.33	19.5413	18.7162	18.9118	18.3844	18.9024	19.2774
OXSM	17.2892	17.4364	17.1684	17.1191	17.0904	17.0692	17.703	16.8758	17.0563
OXSR1	19.9336	19.7474	19.8083	19.8064	19.649	19.5488	19.4876	19.6398	19.7196
P4HA1	21.4207	21.214	21.1834	21.0603	20.9935	21.2003	21.3821	21.0124	21.2278
P4HA2	19.221	18.9763	18.4092	18.8451	18.8567	19.204	19.0891	19.0736	19.0796
P4HB	25.2277	24.9415	24.9639	24.9612	24.8154	24.9812	25.1679	25.0034	25.0843
PA2G4	23.9496	24.1756	23.8821	24.1038	23.824	23.9323	23.9905	23.9733	24.3573
PABPC1	24.1034	23.8167	24.0218	23.8824	23.6973	23.6813	24.2048	23.914	23.9958

PABPC4	22.5004	22.3793	22.319	22.3471	22.2155	22.2135	22.4681	22.3018	22.4509
PABPN1	20.8573	20.5708	20.5963	20.325	20.6154	20.6611	21.0072	20.7434	20.5912
PACSIN2	21.2839	20.6976	21.179	21.3041	21.137	21.3133	21.4726	21.182	21.5338
PACSIN3	20.1077	19.6341	19.9472	19.7355	19.8181	19.8956	20.0222	19.7118	19.9223
PAF1	20.2596	20.3645	20.0143	20.0904	20.0503	19.8702	20.0512	19.9192	20.2323
PAFAH1B1	20.4001	21.2785	20.7106	20.7034	20.5229	20.6111	20.8804	20.6697	20.9173
PAFAH1B2	21.1495	20.9264	20.947	20.8281	20.9548	21.0313	21.2194	20.9059	20.8396
PAFAH1B3	20.77	20.0329	20.8495	20.8347	20.8531	20.7871	21.1224	20.6647	20.9073
PAICS	23.3569	23.2065	23.1482	23.1799	22.9688	23.2735	23.2137	23.1803	23.299
PAIP1	16.7606	17.5296	17.0909	17.1229	16.7298	15.0836	15.1711	16.5002	16.0195
PAK1	18.944	19.0163	18.9171	19.0474	18.8104	19.2739	19.3548	19.0884	19.0116
PAK2	20.5929	23.164	20.6335	20.0288	20.1737	20.4543	20.813	20.2667	19.9843
PAPOLA	19.7437	19.9386	20.0071	20.0287	19.6398	19.8286	20.2701	19.8486	19.9477
PAPSS1	20.8375	20.5054	20.4056	20.4469	20.3904	20.0999	20.5586	20.3566	20.1814
PAPSS2	20.1744	20.6188	20.633	20.4294	20.447	20.1713	20.6499	20.2257	20.4712
PARK7	23.402	23.5438	23.5679	23.6344	23.3928	23.9029	24.0637	23.4326	23.6388
PARP1	21.8833	21.6736	21.8494	21.5857	21.5468	21.3017	21.8441	21.6493	21.6683
PARVA	20.0493	19.5397	19.9938	19.8237	19.7637	19.8113	20.2043	19.8993	19.9342
PAWR	20.9526	20.9638	20.5763	20.8	20.8402	20.8289	20.6812	20.6976	20.9158
PBDC1	20.7009	21.0564	21.1266	20.9986	20.8808	20.7088	21.2176	20.8647	20.8453
PBK	21.1795	21.0599	20.8506	21.0118	20.7216	20.6436	20.5937	20.4228	20.4875
PBRM1	20.2037	21.9884	20.1302	20.0493	19.958	20.4952	20.6744	20.4768	20.8976
PC	20.7636	20.5952	20.7217	20.458	20.5537	20.564	21.1483	20.6189	20.7539
PCBP1	23.2662	23.0217	22.9818	23.0313	22.8477	23.2781	23.3897	23.1433	23.4193
PCBP2	24.0457	23.4861	23.6492	23.6542	23.6048	23.5037	23.8398	23.7957	23.7056
PCBP3	20.2745	19.8305	19.4062	20.1487	19.3287	20.1038	19.3566	20.3553	20.0813
PCK2	19.0103	18.8894	18.7433	18.7025	18.5106	18.7904	19.3545	19.0541	19.0857
PCMT1	19.9446	19.8155	19.8405	20.1959	20.0319	20.2557	20.385	19.7442	20.1449
PCNA	24.1418	23.8034	24.0307	23.9865	24.0068	24.1339	24.5124	24.019	24.178
PCNP	20.4291	20.185	20.4446	20.6079	20.1689	20.0415	20.5564	20.0257	20.287
PCYOX1	19.7507	19.3917	20.3282	20.3029	20.3689	20.194	20.6087	20.1873	20.3494
PCYT1A	20.2024	19.7918	19.6773	19.8588	19.5597	19.7738	19.8825	19.7298	19.6464
PDAP1	19.7885	18.4742	18.8245	19.8632	19.1597	19.559	19.3972	19.7405	19.8329
PDCD11	16.8071	17.8078	17.9954	17.3995	17.388	17.1505	17.8203	17.4425	17.2217
PDCD6	22.0359	21.8777	21.9361	21.6837	21.9205	21.783	22.0693	21.8812	21.6355
PDCD6IP	21.5563	21.7731	21.8504	21.5581	21.608	21.7549	22.1437	21.758	21.893
PDCL3	20.7833	20.704	20.3632	20.6651	20.2238	20.1182	20.1928	20.3857	20.6139
PDE12	20.5465	20.3343	20.3231	20.3247	20.1882	19.8907	19.9447	19.9409	20.0146
PDE1C	20.042	19.9732	20.1005	20.0362	19.8796	19.6632	19.8897	19.7643	20.018
PDHA1	20.4162	20.2831	20.2145	20.164	19.9248	19.6642	19.8938	20.0913	20.3854
PDHB	20.7411	20.7525	20.7442	20.636	20.767	20.8464	21.0234	20.7822	20.7223
PDIA3	24.729	24.8065	24.6899	24.7251	24.5958	24.7941	24.9784	24.5469	24.9238
PDIA4	24.4963	24.3745	24.4217	24.2498	24.1905	24.4796	24.8151	24.3837	24.5616
PDIA6	24.5335	24.4937	24.1615	24.2712	24.0952	24.0194	24.3441	24.1501	24.4907
PDLIM1	20.4749	20.2222	20.2442	20.3529	20.0482	20.2787	20.2691	19.8993	20.1959
PDLIM4	22.1714	21.8553	22.1341	22.1334	22.0638	22.7964	23.1087	22.6011	22.6682
PDLIM5	19.7743	19.4015	19.5876	19.5138	19.4524	19.3659	19.6819	19.317	19.3469
PDLIM7	21.1101	21.0288	20.8979	20.8731	20.7246	20.9988	21.1173	20.9087	21.043
PDP1	14.2055	17.3587	17.4261	17.4259	17.1424	16.4555	16.9422	17.1869	16.5571
PDS5A	20.1325	20.007	20.005	19.8696	19.8688	19.6002	20.1229	19.8142	19.883
PDS5B	18.1911	17.7686	18.0238	17.9225	18.16	17.7928	18.0359	18.002	17.8503
PDXK	19.7928	19.0504	19.6543	19.3971	18.9983	20.375	20.3782	20.1061	19.824
PDXP	18.9619	18.6208	18.7685	18.8977	18.5666	18.8819	18.9391	18.6843	18.6658
PEA15	18.7691	18.2223	18.309	18.4794	18.2417	18.217	17.601	18.1867	17.9155
PEBP1	23.6548	23.4821	23.2851	23.5891	23.4276	23.8072	23.5387	23.4454	23.3298
PEF1	19.2823	19.6656	19.2592	19.4551	19.2799	18.931	18.7305	18.8467	19.1409

PELO	17.8266	17.5865	17.6848	17.7014	17.7462	18.1084	18.1328	18.056	18.028
PELP1	20.2783	20.0132	19.9855	19.9931	19.7771	19.8027	20.0384	20.1008	19.9372
PEPD	20.2315	20.2184	20.3264	20.2499	20.0593	20.4362	20.9825	20.5913	20.4288
PES1	20.4318	20.5642	20.4628	20.3427	20.4794	20.3555	20.6831	20.5862	20.6308
PEX11B	18.0917	18.2564	18.2073	18.2246	18.0682	17.7741	18.5351	18.1995	18.1135
PFAS	21.1721	21.2276	21.2355	21.1382	21.18	21.4139	21.7529	21.2331	21.4306
PFDN2	20.6491	20.0906	20.4272	20.7074	20.4302	20.7153	21.0406	20.4509	20.8795
PFDN5	19.2882	19.0192	19.2594	19.4387	18.9984	19.1455	19.3249	18.9835	19.3597
PFKL	20.4171	20.104	20.1197	20.0477	20.0015	20.3144	20.5385	20.3918	20.2971
PFKM	20.7179	20.5526	20.9122	20.8918	20.7088	20.8305	21.0466	20.6979	20.8825
PFKP	22.5155	22.6362	22.4976	22.4699	22.2196	22.3963	22.1229	22.4175	22.5505
PFN1	26.8142	26.9493	26.9909	26.873	26.8294	26.967	26.806	26.8212	26.9685
PFN2	20.8381	19.6724	20.9505	20.8813	20.8037	20.9346	20.781	20.8635	21.023
PGAM1	24.2244	23.8752	23.8488	23.9749	23.7281	24.3289	24.2885	24.0886	24.2992
PGAM5	20.5957	20.3947	20.7274	20.5743	20.6753	20.8221	21.0255	20.755	20.7954
PGD	23.5643	23.6531	23.6149	23.8028	23.5995	24.0681	24.3493	23.8862	24.0979
PGK1	25.4044	24.9942	25.1095	25.0173	25.034	25.1814	25.3507	25.06	24.9041
PGLS	21.2535	21.0567	21.0199	21.178	20.8758	21.4943	21.647	21.1699	21.4189
PGM1	21.3323	21.3314	21.2792	21.3405	21.0354	21.3285	21.5763	21.3641	21.3816
PGM2	18.646	19.4174	18.7217	18.9605	18.0057	18.0613	18.2301	18.4839	18.7328
PGM3	19.4118	19.5452	19.4405	19.5341	19.5018	19.6332	19.8483	19.1413	18.9829
PGP	19.7838	19.9547	19.948	18.5724	20.0724	18.8391	18.7989	20.0836	19.6714
PGRMC1	19.7825	19.5173	19.299	19.6143	19.2089	19.1599	19.4813	19.365	19.4908
PGRMC2	19.8977	20.1695	20.0224	19.962	19.8076	20.2316	20.3678	19.9127	20.1802
PHB	24.6439	24.7737	24.5392	24.4224	24.3378	24.6864	24.9393	24.6344	24.7785
PHB2	24.5798	24.2308	24.2486	24.3054	24.1035	24.2083	24.4656	24.3037	24.2728
PHC2	19.3101	19.021	19.0958	18.9209	18.9711	18.8496	18.8893	18.8123	19.1792
PHF5A	18.3524	18.1277	18.7781	18.2741	17.6314	18.878	19.2455	18.5186	18.7474
PHGDH	23.798	23.513	23.6019	23.4685	23.3884	23.7988	23.9767	23.7234	23.8242
PHPT1	18.5402	18.3057	18.8061	18.4304	18.5703	18.6461	18.385	18.3455	18.3303
PI4K2A	18.5781	18.0977	18.1883	18.3774	18.0673	18.3853	18.2176	18.3589	18.5263
PI4KA	14.364	15.0782	15.1354	0.91432	15.4716	5.25395	14.0371	15.1696	14.383
PICALM	18.9612	19.0891	18.8229	18.9681	18.9288	18.5153	18.9717	19.1384	19.2867
PIGK	17.3539	17.2533	16.6497	16.8813	16.6531	16.0306	15.9393	17.1727	17.4052
PIGS	17.4192	17.5854	17.25	16.1123	16.8119	16.3269	17.0316	16.9498	17.4964
PIGT	20.0764	21.2801	20.42	20.4879	20.517	20.6665	21.1354	20.5531	20.7728
PIGU	16.0578	16.6248	16.5282	17.2597	16.8558	13.0724	15.0214	16.5389	16.0611
PIH1D1	19.6903	19.2751	19.2407	19.3109	19.3713	19.6947	19.7813	19.3992	19.5406
PIK3R4	17.9526	17.3067	19.5209	17.5999	19.6662	17.7207	20.2265	17.6807	17.9946
PIN1	18.1177	18.3595	17.9298	18.6673	17.8158	18.3275	17.6777	18.1626	17.9491
PIP4K2B	17.772	18.0803	17.9572	17.7823	17.9995	18.4975	18.77	18.3704	18.3013
PIP4K2C	16.5861	16.9981	16.9637	16.9744	17.1094	16.7484	16.4743	16.8095	16.4411
PIR	19.4671	19.9117	19.405	19.6181	19.2741	19.0453	19.4032	19.3436	19.3914
PITHD1	19.6455	19.5772	19.5955	19.7015	19.5263	19.959	19.5025	19.5346	19.6327
PITPNA	17.2093	14.944	16.5613	16.8962	16.099	16.5192	14.851	16.89	16.8392
PITPNB	21.6487	21.5981	21.7968	21.5135	21.4001	21.736	21.9411	21.5592	21.5986
PITRM1	19.6608	19.627	19.6745	19.5466	19.5279	19.7218	20.212	19.8238	19.9005
PKM	27.4569	27.4498	27.5579	27.3467	27.2486	27.5632	27.8756	27.4367	27.5778
PKN2	18.6399	18.4908	18.3021	18.653	18.2309	17.6857	17.5131	17.7577	17.854
PLA2G15	14.2728	15.0083	14.8362	15.0079	14.5808	12.8962	10.9702	13.934	12.4547
PLA2G4A	16.0505	15.8138	15.9021	15.0633	15.3811	14.0955	14.9747	15.7831	14.9458
PLAA	19.6927	19.5979	19.5802	19.721	19.4004	19.5392	19.572	19.4682	19.6307
PLAT	18.201	17.6408	17.6955	17.6238	17.2827	18.4915	18.7163	18.6112	18.4618
PLCB3	17.5516	17.2246	17.584	17.2203	17.1691	17.5437	17.0423	17.4173	17.4407
PLCG1	21.0007	20.6447	20.7268	20.4174	20.4167	20.6451	20.8591	20.1627	20.324
PLCH1	21.1971	21.7391	21.3014	21.2745	20.9503	20.3015	20.201	21.182	21.3971

PLD3	19.2466	18.8534	18.9116	18.8084	18.8037	18.4103	18.7949	18.9138	18.9789
PLEC	23.5363	23.3649	23.2397	23.2925	23.2024	23.125	23.2977	23.1938	23.3365
PLIN3	21.7602	21.5065	21.5992	21.5025	21.3085	21.5733	21.6299	21.4091	21.763
PLK1	18.0667	18.7474	18.6428	18.4771	18.4299	18.7307	18.9729	18.7319	18.6718
PLOD1	20.0742	19.8766	20.062	19.8922	20.0019	19.7313	20.1222	19.8198	19.8229
PLOD2	19.2874	19.3384	19.4219	18.97	19.1876	18.9514	19.4298	19.1896	19.0846
PLOD3	21.5153	21.4962	21.5361	21.3361	21.2161	21.0679	21.4281	21.1724	21.3492
PLP2	20.2289	19.9941	20.1486	19.9352	20.029	20.4243	20.6724	20.3838	20.4887
PLRG1	20.4171	20.4518	20.2519	20.374	20.1555	19.8229	20.4093	20.0569	20.1448
PLS1	19.0393	18.4079	17.9371	17.7273	17.6312	17.6368	18.187	18.135	18.2496
PLS3	19.8136	19.6454	19.9696	19.5313	19.5227	19.5403	19.8851	19.3946	19.6155
PLXNB2	17.2003	17.1833	17.3428	16.9756	17.0399	17.0012	17.358	17.2836	16.9969
PML	19.5237	19.3972	19.4643	19.2609	19.1088	19.7398	19.6459	19.8014	19.8238
PMM2	19.2854	19.3795	18.8343	19.0429	19.1405	19.4386	19.6091	19.1151	19.4652
PMPCA	19.6767	19.6254	19.6267	19.5179	19.4924	19.1687	19.3212	19.3554	19.4675
PMPCB	19.9277	19.781	19.9387	19.8446	19.8641	19.8416	20.6661	19.7304	20.1115
PMVK	18.4792	18.9447	19.2024	18.5858	18.7551	18.6489	18.8555	18.618	18.5198
PNN	21.2487	21.0178	20.9137	21.0751	20.9595	21.0327	21.431	21.0021	21.1941
PNO1	18.2318	18.9683	18.3034	18.808	18.9466	19.1353	18.356	18.8009	18.008
PNP	18.1697	17.811	17.9782	18.0241	17.8883	18.0952	17.869	18.081	17.7585
PNPO	20.3653	20.5125	20.5414	20.4392	20.2767	20.3867	20.4509	20.3335	20.4867
PNPT1	20.8798	21.2468	20.9907	20.9897	20.9779	20.8592	21.5583	20.9179	21.2183
PODXL	21.1986	21.6839	21.412	21.3986	21.3025	21.8058	21.8516	21.8945	22.2879
POFUT1	21.5369	21.1483	21.3136	21.0519	21.0299	21.3118	21.1418	21.069	21.2446
POGZ	17.741	18.5034	18.5253	18.3169	18.5602	18.3253	18.5659	18.5351	18.3196
POLA2	19.8422	19.5098	19.565	19.3991	19.5416	19.4611	19.5505	19.2712	19.2335
POLD1	20.0606	22.5496	20.6388	20.7699	20.4935	20.1521	20.4892	20.1035	20.3646
POLD2	18.1404	17.347	18.1472	18.2553	18.3211	18.4856	18.5115	17.5449	18.3397
POLDIP2	19.8384	20.2474	19.8637	19.727	19.7593	20.0345	20.4731	19.9891	20.0254
POLDIP3	19.8189	19.6215	19.8776	19.7315	19.666	19.4805	19.8091	19.6805	19.6535
POLR1A	20.2429	19.8286	19.7733	19.9751	19.679	19.8231	19.8616	19.9174	19.9138
POLR1B	16.9721	16.8891	16.6258	17.2939	16.6563	14.3149	15.5599	16.7385	16.66
POLR1C	19.9818	19.5843	19.7275	19.6146	19.4294	19.7838	19.9114	19.6956	19.887
POLR2A	19.7015	19.6531	19.8859	19.6204	19.5115	19.7645	19.9519	19.7825	19.9024
POLR2B	20.4329	20.3461	20.4557	20.2874	20.2862	20.2293	20.7705	20.3749	20.5298
POLR2C	18.3993	18.1608	18.505	18.3649	18.3218	18.0684	18.9824	18.475	18.3433
POLR2E	19.5634	19.7705	19.7521	19.7878	19.5276	19.8178	19.8598	19.7609	19.7554
POLR2G	19.8011	19.5825	19.6266	19.5925	19.2752	19.5274	19.7472	19.5176	19.8494
POLR2H	20.9299	21.3619	20.551	20.6717	20.7259	20.9382	20.9598	20.6215	21.0592
POLR3C	19.1118	19.1015	18.8278	19.1486	19.1539	18.947	19.0578	18.7849	18.8263
POLR3F	19.2633	19.4146	19.4175	19.279	19.0871	19.2872	19.3905	19.3174	19.4399
PON2	19.7991	20.1428	20.0808	20.124	19.8864	20.1918	20.1883	20.2043	20.1451
POR	21.2053	21.1123	20.9358	20.9281	20.7445	21.116	21.5173	21.1418	21.4624
PPA1	23.0337	22.6036	22.8616	22.4966	22.565	22.996	23.0742	22.9518	22.6723
PPA2	17.1101	17.3254	17.2488	16.7707	17.454	17.5712	17.0782	17.6684	16.7096
PPAN	18.0559	18.0763	18.2763	17.9011	17.8338	18.1641	18.4445	18.0542	17.7317
PPAT	20.0423	19.7559	19.6778	19.6949	19.5583	19.7811	20.1721	19.841	20.0188
PPFIA1	17.1002	17.0201	17.0885	17.2771	17.2348	17.3019	17.8644	17.527	17.3913
PPFIBP1	18.3532	18.1189	18.5803	18.2337	18.0476	18.5266	18.4728	18.2721	18.3853
PPIA	26.5591	26.5669	26.5303	26.6633	26.313	26.9888	27.0723	26.7636	27.0088
PPIB	25.5983	25.539	25.6267	25.3322	25.2635	25.2521	25.7096	25.3592	25.5951
PPIC	19.9747	19.897	20.1439	19.8945	19.7557	19.6507	20.0394	19.8007	19.9072
PPID	19.9857	20.2862	19.9204	20.2734	19.9555	19.636	19.6744	19.6604	19.9134
PPIE	19.6219	19.4775	19.3822	19.3572	19.6167	20.0033	19.6869	19.4558	19.552
PPIF	21.6257	21.6885	21.4161	21.4945	21.4749	21.9017	22.2683	21.671	21.832
PPIG	20.2508	20.1378	19.6829	19.3278	19.1585	19.9081	19.595	19.7678	19.6872

PPIH	19.1688	19.3635	19.0465	19.3098	19.192	19.6957	19.7063	19.0522	19.624
PPIL2	18.4866	18.017	17.8852	18.356	18.0882	18.0238	18.0375	18.1511	18.3819
PPIL3	18.3889	18.7119	18.8396	18.8728	18.5984	18.8414	18.5147	18.3756	18.8845
PPIL4	19.1292	18.9855	19.0294	19.2	19.0723	18.6685	18.8806	19.0231	18.7223
PPM1A	19.7114	19.1271	18.8163	19.427	18.8992	19.4232	19.5566	19.4943	19.4095
PPM1F	20.9277	20.7806	20.9037	20.7921	20.7246	20.6423	21.0316	20.8305	20.6765
PPM1G	21.9391	21.8454	21.7806	21.7563	21.6041	21.665	21.8555	21.559	21.8253
PPME1	20.7717	20.3781	20.7003	20.3638	20.3709	20.7526	20.834	20.5115	20.4999
PPOX	17.0991	17.5524	17.6606	17.0672	17.2651	16.6939	17.3314	17.1442	17.1092
PPP1CA	20.9414	20.7463	20.6157	20.7196	20.5487	20.5889	20.2652	20.6214	20.9045
PPP1CB	21.7495	20.9324	21.6979	21.4044	21.3225	21.5396	21.7308	21.5185	21.5018
PPP1CC	19.123	15.5949	18.7089	18.9947	19.0439	19.2653	19.5983	18.8294	19.1038
PPP1R10	18.1673	18.4371	18.3386	18.37	18.0103	18.0213	18.4224	18.231	18.3679
PPP1R12A	18.7167	18.8082	19.0767	18.4111	18.9497	18.7951	19.2164	18.536	18.8951
PPP1R14B	19.1346	18.7497	18.8097	19.0843	18.8109	19.3471	19.0625	18.5925	18.908
PPP1R18	17.338	17.0301	17.4303	17.4537	17.1665	17.6056	17.6233	16.761	17.2351
PPP1R7	19.5345	19.6158	19.9787	19.3774	19.5991	19.7143	20.1991	19.6342	19.7202
PPP1R8	19.8365	19.5138	19.4627	19.6711	19.3067	19.5475	19.7767	19.4341	19.576
PPP1R9B	18.2379	17.7977	17.8909	17.9014	17.7818	17.3917	19.0673	17.733	17.777
PPP2CA	20.3123	20.1872	19.9744	20.1478	19.8282	20.2414	20.0973	20.3297	20.1826
PPP2CB	17.0887	18.1452	17.1685	17.3503	16.7025	16.886	16.9397	16.9308	16.6476
PPP2R1A	22.5422	22.9392	22.7142	22.4105	22.1061	22.1908	23.0503	21.9465	22.3328
PPP2R2A	20.1027	19.441	20.2814	20.3124	20.141	19.8737	20.4287	20.189	20.2254
PPP2R4	21.1658	20.4214	20.5915	20.4475	20.4097	20.3947	20.7883	20.5977	20.6258
PPP2R5C	18.4832	18.352	18.2246	18.2095	17.8318	18.0144	18.4843	18.2039	18.2996
PPP2R5D	17.5512	17.6105	17.8677	17.5952	17.5934	17.0029	17.5344	17.5126	17.387
PPP2R5E	16.5681	17.2114	16.7635	16.8721	16.9852	16.4414	16.3979	16.7041	16.2491
PPP3CA	20.2861	19.47	19.6748	20.0566	19.5759	19.5667	19.6623	19.5098	19.7975
PPP4C	20.6676	20.1449	20.1608	20.1325	20.1702	19.8058	19.8704	20.1727	20.1212
PPP4R1	15.5231	15.3199	15.9009	16.0206	15.1452	14.899	15.375	15.5956	15.3453
PPP4R2	18.9862	18.919	18.7334	18.8817	18.9629	18.6875	19.0445	18.6652	18.9189
PPP5C	20.5549	20.4275	20.2192	20.4426	20.1358	19.9146	19.8342	20.1356	20.3359
PPP6C	20.0173	20.1168	20.2367	20.0519	20.0968	20.3659	20.758	20.2305	20.3629
PPP6R3	19.3468	19.475	19.2781	19.4034	19.19	18.7795	18.6949	19.3737	19.5269
PPT1	20.8474	20.262	20.889	20.8658	20.6678	20.8442	20.8106	20.9013	20.9918
PPT2	20.0709	19.8008	19.9923	19.6294	19.5731	19.4788	19.7085	19.8648	19.7228
PQBP1	20.3106	19.845	19.835	20.1177	19.7424	20.4607	20.5361	20.088	20.2832
PRAF2	20.5	19.4085	19.853	18.8826	19.6567	19.6367	19.5269	19.7424	19.9427
PRC1	16.777	17.0438	16.7813	16.5657	17.0346	15.9066	16.619	17.2287	17.1969
PRCC	16.2919	16.2298	17.7681	17.1886	17.0367	17.6448	16.8418	16.9903	17.031
PRDX1	25.9539	26.0246	25.9384	25.9546	25.7724	26.1256	26.1593	25.9798	25.9879
PRDX2	22.9452	23.0128	22.9737	22.7866	22.8356	23.1516	23.475	22.9942	23.1161
PRDX3	22.3918	22.4868	22.2926	22.274	22.2869	22.3618	22.4709	22.1967	22.3624
PRDX4	21.744	21.3979	21.6997	21.546	21.5218	21.7809	22.0234	21.698	22.0004
PRDX5	23.5797	23.5034	23.5218	23.6311	23.461	23.4838	23.6848	23.3713	23.547
PRDX6	24.7884	24.7948	24.6302	24.6927	24.5938	24.7765	24.9466	24.7509	24.8325
PREB	17.008	17.153	17.4112	17.6968	17.487	17.8985	17.8872	17.5187	17.391
PREP	20.1164	20.4287	20.6567	21.4405	20.5777	19.6023	20.0576	20.1007	20.0901
PREPL	17.2023	17.0944	16.7159	16.7706	16.9238	16.7211	17.0351	17.19	16.5744
PREX1	15.5829	14.1587	16.4192	16.3606	16.5767	15.4603	15.4163	15.5864	14.9202
PRIM1	18.284	17.5301	17.7889	17.0346	17.7896	17.8544	17.9061	17.7105	17.6903
PRIM2	16.7885	16.908	16.8293	16.687	16.7076	16.3663	16.5209	16.6814	16.5759
PRKAA1	18.8019	19.1206	19.2443	18.9191	19.1687	19.2533	19.3286	19.1088	18.9457
PRKACA	19.5733	19.438	19.2743	19.4382	19.2071	19.2817	18.6418	19.122	19.1226
PRKAG1	19.3558	19.2608	19.3011	19.0801	19.0708	18.9065	19.0454	18.9209	18.8637
PRKAR1A	21.1657	21.0836	20.9735	21.0402	20.7792	21.1901	20.8792	20.9987	21.0492

PRKAR2A	20.5439	21.7747	20.5872	20.8177	20.7332	20.767	20.8948	20.605	20.8333
PRKCD	20.4091	20.1633	19.9366	20.0532	19.689	19.8181	20.0934	19.9075	19.9193
PRKCDBP	20.0108	19.4914	19.7737	19.6163	19.3296	19.4937	19.5983	19.436	19.5474
PRKCI	17.8435	17.3014	17.4322	17.5255	17.0263	16.4086	17.1412	16.9431	17.1565
PRKCSH	23.5098	23.7522	23.5993	23.8112	23.4458	23.426	23.4026	23.4277	23.8569
PRKD1	16.8734	16.7271	16.4791	16.4573	16.3878	17.1087	15.9055	16.341	16.1184
PRKDC	22.5711	22.3246	22.1716	22.2153	22.1475	22.0827	22.3964	22.1922	22.2804
PRKRA	16.9695	16.8557	17.8918	17.5064	17.7649	16.8095	16.7014	17.0725	16.939
PRMT1	22.9208	22.8872	22.7437	22.7967	22.7021	22.7314	22.9543	22.662	22.8438
PRMT3	17.5716	17.5161	18.3154	17.9791	17.2808	16.4564	17.2655	17.2994	17.2179
PRMT5	21.2127	21.0231	20.7895	21.1046	20.9602	20.8802	20.9143	20.8719	21.0596
PROCR	20.5883	20.538	20.5304	20.4141	20.4532	20.585	20.8553	20.8288	20.7911
PRPF19	23.3727	23.3073	23.4953	23.281	23.1797	23.3176	23.4163	23.1213	23.2898
PRPF31	20.2073	19.8709	19.708	19.7896	19.7313	19.5697	19.3082	19.6285	19.6118
PRPF38A	20.4003	20.2249	20.0563	20.083	20.0176	20.0511	20.3212	19.8845	20.155
PRPF38B	17.9646	18.0768	17.6606	17.7857	18.1004	16.5604	17.4515	18.0168	17.7362
PRPF4	20.9949	20.4997	20.5855	20.4122	20.4519	20.4771	20.5996	20.5779	20.2539
PRPF40A	20.4054	20.2281	20.4671	20.2083	20.173	20.4566	21.092	20.5338	20.52
PRPF4B	16.9696	16.9997	16.9108	17.0592	16.7688	17.8886	18.1821	17.8255	19.6919
PRPF6	21.5579	21.4553	21.3891	21.4328	21.2121	21.6704	21.654	21.4058	21.6468
PRPF8	24.3646	24.5135	24.2745	23.7081	24.3887	23.8851	23.6527	23.8945	24.0339
PRPS1	21.0976	20.8352	20.7644	20.7125	20.7378	20.7546	21.0202	20.7373	20.4573
PRPS2	20.2717	20.0473	20.066	19.9058	19.9174	20.0516	20.2354	20.0529	19.6122
PRPSAP1	20.128	20.0235	19.7481	20.1046	19.9048	19.7981	19.9669	19.6064	19.7565
PRPSAP2	17.7975	17.5533	17.5289	17.5904	17.5515	16.2875	17.5977	17.7317	17.2856
PRRC1	20.0243	19.6048	19.7267	19.7869	19.7005	19.7522	19.905	19.8638	19.7675
PRRC2A	18.3235	18.0512	17.9355	17.9789	17.8142	18.1555	18.1938	18.0784	18.2666
PRRC2C	21.1707	21.2677	21.1929	20.408	21.1276	21.0785	21.5441	20.711	21.2192
PRTFDC1	18.8595	16.8855	17.4129	16.5005	17.1164	18.2259	16.9182	16.8741	16.4062
PSAP	20.8979	20.8769	20.7419	20.9481	20.7102	20.9993	21.2131	20.9652	21.3492
PSAT1	22.3125	22.1063	22.3909	22.3994	22.2169	22.5791	22.865	22.4649	22.753
PSIP1	20.064	19.9934	19.8596	19.961	19.9392	20.1471	19.9942	19.8536	19.6947
PSMA1	23.3553	22.7512	23.2228	23.2159	23.0744	23.3974	23.5127	23.3114	23.4177
PSMA2	21.7308	21.8054	21.6662	21.8403	21.6324	21.9447	21.1047	21.5502	21.6852
PSMA3	21.5581	20.4765	21.5769	21.4229	21.5067	21.6477	21.7826	21.5474	21.5451
PSMA4	22.2416	22.4118	22.6201	22.4925	22.3575	22.6485	22.9551	22.2884	22.4163
PSMA5	22.7753	22.6512	22.7736	22.6367	22.5221	22.9468	22.8541	22.8587	22.92
PSMA6	23.3028	22.7641	23.2085	23.2314	23.1529	23.406	23.7748	23.2794	23.428
PSMA7	23.6228	23.1652	23.9153	23.8268	23.7507	23.8764	24.1485	23.783	23.9313
PSMB1	23.7384	23.5919	23.564	23.4585	23.3609	23.6219	23.7403	23.5979	23.7224
PSMB2	22.253	21.5036	21.9295	21.9965	21.8756	21.2516	22.2807	21.7245	21.9764
PSMB3	21.7295	21.5166	21.3532	21.655	21.3684	21.2664	21.3936	21.4978	21.4148
PSMB4	22.2769	21.0014	21.9367	22.1865	21.8021	22.424	22.3791	22.112	22.5066
PSMB5	22.5169	22.6041	22.4523	22.4547	22.3373	22.4943	22.7757	22.4296	22.5817
PSMB6	21.9099	21.4729	21.8601	22.0657	21.8112	22.153	22.1754	21.928	22.2603
PSMB7	22.2662	22.2894	22.11	22.09	21.8744	22.3386	22.4706	22.0871	22.373
PSMB8	19.8676	20.1144	20.0819	20.0397	19.9797	20.005	20.5537	20.2881	20.2949
PSMB9	20.0675	20.2444	20.1391	19.9203	19.9634	19.9087	20.5165	20.2029	20.0336
PSMC1	22.5892	21.9844	22.4745	22.5332	22.3714	22.5727	22.7254	22.5536	22.503
PSMC2	22.9502	23.0237	22.9088	22.7182	22.6168	22.9894	23.1926	22.6971	22.8445
PSMC3	22.5252	22.5521	22.5376	22.4743	22.0071	22.4875	22.1973	22.3663	22.5659
PSMC4	22.4844	22.3512	22.5689	22.2772	22.1826	22.5029	22.6825	22.3755	22.4001
PSMC5	22.131	22.087	22.0151	22.0097	21.7558	21.911	21.8805	21.919	21.9965
PSMC6	22.376	22.2491	22.3356	22.4718	22.3811	22.5109	22.8683	22.4056	22.6353
PSMD1	22.9266	22.6441	22.9313	22.6425	22.5629	22.7415	22.901	22.7025	22.7143
PSMD10	19.3908	18.9843	18.5142	19.6349	18.4795	18.2873	18.3547	18.09	18.6107

PSMD11	22.1221	21.4221	22.2125	22.0683	21.8411	22.0987	22.336	22.0469	22.242
PSMD12	21.7863	21.3535	21.6997	21.7663	21.5826	21.3045	21.8072	21.7543	21.8976
PSMD13	22.6625	22.2452	22.9965	22.6956	22.827	22.6275	23.0847	22.8687	22.7216
PSMD14	22.7378	22.7363	22.6046	22.5485	22.4301	22.6009	22.7977	22.4255	22.7309
PSMD2	22.5599	22.2947	22.5013	22.3093	22.2907	22.0946	22.6347	22.4572	22.479
PSMD3	22.0087	21.9598	22.1371	21.9201	21.8047	21.8188	22.2974	21.8543	22.1539
PSMD4	21.9706	21.7259	22.0068	21.9254	21.8546	21.9915	22.3568	21.9451	21.9899
PSMD5	20.3922	20.4249	20.2496	20.0869	19.9926	20.2827	20.5607	20.1671	20.5037
PSMD6	22.0916	22.0811	22.209	22.0191	21.7815	21.9074	22.0457	21.942	22.1174
PSMD7	21.9369	21.4001	21.6516	21.5545	21.6229	21.6926	21.8042	21.7127	21.6322
PSMD8	22.0506	21.7773	22.1536	22.1768	22.1074	22.4769	22.696	22.1823	22.4138
PSMD9	18.9574	19.138	19.7387	19.8504	20.0097	20.1875	20.3225	19.6698	20.0463
PSME1	22.0954	22.0543	22.1835	22.1184	21.9827	22.3937	22.5716	22.2013	22.4274
PSME2	21.3454	21.2521	21.5655	21.3249	21.2897	20.647	21.4372	21.3809	21.4814
PSME3	22.3418	22.321	22.6974	22.2433	22.3359	22.392	22.844	22.4021	22.557
PSME4	18.0483	18.0864	18.1495	17.951	18.0547	17.3345	18.276	18.1254	17.9777
PSMF1	19.9867	19.9815	19.8687	19.8136	19.6361	20.115	20.1642	19.9829	20.0233
PSMG1	18.5847	19.3277	19.2514	18.9902	19.2832	18.7644	19.3733	19.1782	19.1694
PSMG2	18.46	20.7909	20.9722	21.6322	21.5872	21.2884	21.5016	20.3663	21.0452
PSMG3	16.6578	17.1485	17.9546	17.4765	17.4295	17.5623	17.1665	17.318	16.7173
PSPC1	22.0885	21.9521	22.078	21.7371	21.673	21.8349	21.992	21.8016	21.7686
PSPH	20.9921	20.6744	20.8661	20.919	20.6549	20.8028	20.7998	20.7831	20.7641
PTBP1	23.4376	23.1055	23.4188	23.3632	23.5439	23.8056	23.7953	23.3887	23.1556
PTBP2	17.9437	17.7334	17.8501	17.5045	17.5809	17.5177	18.0905	17.7149	18.1058
PTCD1	16.7585	17.3521	17.4423	17.388	17.114	16.9591	17.3269	17.4036	17.0488
PTCD3	19.2118	20.5768	19.1389	19.0832	19.0427	18.9416	19.5821	19.2856	19.3743
PTDSS1	18.0207	17.9034	17.621	17.6524	18.1553	17.374	17.9727	17.8528	17.8296
PTGES2	19.4617	19.1099	19.0857	19.0245	19.0805	18.6747	19.3088	19.4254	19.0579
PTGES3	22.8877	22.7183	22.9638	23.0515	22.8184	22.9682	23.3244	23.0115	23.2918
PTGFRN	19.9997	20.3857	20.1178	20.1373	20.2412	20.1565	20.7328	20.4757	20.6287
PTGR1	17.4585	17.1752	17.2947	17.1701	17.5873	17.4435	17.7081	17.6315	16.6046
PTK2	17.8457	17.7536	17.5715	17.3655	17.5369	17.4789	17.8968	17.2414	17.5055
PTK7	19.1783	18.9875	19.0907	18.6987	19.1104	18.2246	19.1074	18.3218	18.3777
PTMA	17.0045	17.0431	16.782	17.2616	17.4711	17.4614	18.3188	16.474	17.8173
PTPLAD1	21.0177	21.0778	20.8758	20.6594	20.8955	20.6857	20.8278	20.9521	21.109
PTPN1	22.0526	21.5466	21.7327	21.4289	21.3417	21.1689	21.6417	21.5846	21.617
PTPN11	19.8155	20.7866	19.6205	19.6752	19.4758	19.1584	19.5121	19.5015	19.5988
PTPN12	18.7066	18.8074	18.9962	18.7199	18.5407	18.991	19.1265	18.9704	19.1609
PTPN23	24.0213	24.4613	24.6539	24.1013	24.6603	24.3824	24.9097	24.0779	24.7061
PTPRF	20.4388	20.0996	20.1793	20.0454	20.0609	20.0353	20.5059	20.1443	19.7942
PTRF	20.846	20.4609	20.7579	20.5926	20.5646	20.3367	20.2376	19.8285	19.9143
PTRH2	20.328	19.8494	20.2824	20.3093	20.3248	20.5812	20.7202	20.2237	20.3239
PUF60	22.7265	22.214	22.5422	22.3968	22.0406	22.3301	22.4829	22.452	22.7405
PURA	20.7181	20.4067	20.4623	20.4353	20.3764	20.5031	21.1029	20.5448	20.3421
PURB	20.6474	20.4066	20.5951	20.5631	20.5509	20.4892	20.8999	20.5156	20.6153
PUS1	19.6293	19.0919	19.0962	18.9394	18.8818	19.1813	19.4268	19.1747	19.286
PUS7	19.2156	18.9075	19.2494	19.3482	18.9701	19.1065	19.6673	19.1305	19.3609
PWP1	18.2557	18.3893	18.628	18.485	18.4975	18.1886	18.2709	18.1312	18.3377
PWP2	19.6728	19.5782	19.3158	19.4769	19.554	19.0113	19.6033	19.4726	19.419
PXMP2	15.4827	16.1547	16.5341	16.651	16.4483	15.8377	16.3125	16.5765	16.419
PXN	19.3368	19.7461	18.7043	19.37	18.8061	19.3359	18.677	18.7937	19.3423
PYCR1	20.7995	20.6215	20.7436	20.4707	20.409	20.3396	20.5057	20.1007	20.4878
PYCR2	20.1085	22.7281	20.218	19.9864	19.8069	20.5254	20.8649	20.1476	20.4718
PYGB	25.6825	25.4374	25.4457	25.478	25.2613	25.2952	25.7164	25.5303	25.6744
PYGL	20.5383	20.359	20.5066	20.2224	20.2409	20.3157	20.6104	20.4216	20.3886
QARS	22.4069	22.4358	22.3585	22.3328	22.198	22.1103	22.5222	22.1984	22.3475

QDPR	19.4919	19.2257	19.499	19.3415	19.3001	19.1354	19.4454	19.3408	19.3329
QIL1	19.7454	19.986	19.8836	19.7834	19.559	19.7081	19.9096	19.7823	20.1075
QKI	20.6978	19.8881	20.4997	20.7182	20.5414	20.7873	20.4925	20.6061	20.8448
QRICH1	18.8489	18.5113	18.7664	18.3591	18.7419	18.2644	18.903	18.6745	18.0753
QSOX2	18.5804	18.9379	18.6737	18.8109	18.2939	17.7364	17.9065	18.729	19.1529
QTRTD1	19.5956	19.2926	19.3817	19.0271	19.4578	19.0992	19.3989	19.4002	18.8019
RAB10	21.2557	21.0623	21.1536	21.1308	21.3088	20.766	21.478	21.217	21.2514
RAB11B	23.3067	23.4841	23.483	23.3213	23.4059	23.3954	23.7902	23.2588	23.2751
RAB13	22.5936	22.7516	22.6416	22.6627	22.411	22.8393	22.9461	22.7978	23.2358
RAB14	21.5585	20.9535	21.1892	21.1896	21.1066	21.2702	21.196	21.1226	21.5239
RAB18	21.0708	21.2323	21.3473	21.1181	21.0961	21.3866	21.7285	21.4172	21.6469
RAB1B	22.338	22.2103	22.0256	22.1423	22.0486	22.402	21.9219	22.2828	22.1743
RAB21	21.4055	21.5806	21.7568	21.5983	21.7028	21.4768	21.8784	21.5844	21.5997
RAB22A	19.1234	19.4835	19.1278	19.2126	18.8497	18.8293	18.9898	18.5468	19.0406
RAB2A	21.5335	21.5296	21.3313	21.3769	21.3098	21.5613	21.7112	21.4731	21.6602
RAB34	19.9238	20.5391	19.5471	20.4279	19.7723	20.209	19.2213	19.7531	20.4727
RAB35	20.0753	19.3762	19.7533	19.9821	19.9186	20.3641	19.6947	20.0729	19.8597
RAB3D	17.0274	17.1167	16.9603	16.6321	17.3796	16.5668	16.5742	16.9012	16.6552
RAB3GAP1	21.5397	21.3212	21.2109	21.5523	21.4495	19.431	21.4071	21.6489	21.0018
RAB3GAP2	19.0163	19.9292	19.6735	19.7286	19.6605	19.8942	20.2181	20.2547	20.4849
RAB5A	19.8811	19.4612	19.1588	19.5123	19.1351	19.3227	18.2493	19.3617	18.8278
RAB5B	18.4419	17.6234	17.3676	18.2551	17.299	18.3644	17.6552	18.2816	17.8428
RAB5C	21.7422	21.1261	21.2876	21.457	21.3685	21.7305	21.5113	21.5994	21.4527
RAB6A	21.6878	21.6491	21.7346	21.7256	21.4553	21.7719	21.6654	21.5558	21.6798
RAB6C	18.8262	19.1709	18.7967	18.4737	18.578	18.3713	18.2041	18.9206	18.7442
RAB7A	23.1028	23.3031	23.519	23.1809	23.1194	23.1512	23.3893	23.0997	23.1983
RAB8A	21.3356	19.1885	18.6898	18.8211	18.9962	19.286	19.1556	19.1862	19.0886
RAB8B	17.7067	18.3849	17.8076	17.6446	17.8456	18.2448	18.579	17.5736	18.0148
RABAC1	19.3134	19.3463	19.4153	19.0631	18.9974	19.2204	19.7555	19.3555	19.4591
RABGGTA	17.4526	18.2106	18.0195	17.9003	17.7516	17.8796	18.2572	17.92	18.3484
RABGGTB	19.4045	19.5017	19.2238	19.2301	19.0492	19.2298	18.9857	19.1847	19.4996
RAC1	22.6012	22.1527	22.7515	22.6048	22.5475	23.2248	23.2571	22.7559	23.0033
RACGAP1	18.5802	18.5332	18.767	18.4598	18.2436	18.3383	19.1325	18.403	18.6792
RAD18	16.5568	17.0013	17.697	17.4609	17.0042	17.0964	17.1801	16.5041	16.7226
RAD21	20.8822	20.5539	20.748	20.4228	20.6679	20.4412	21.0034	20.5714	20.6042
RAD23A	17.173	17.3944	17.3995	17.4921	17.9487	16.9045	16.0929	17.4336	17.1794
RAD23B	20.5679	21.0306	21.1062	21.2381	20.9854	20.8339	21.2967	20.985	21.3096
RAD50	17.1427	17.8281	17.7865	17.6204	17.6383	17.252	18.1569	17.7187	17.8272
RAE1	20.7043	20.7397	20.8352	20.6664	20.4681	20.5634	20.4829	20.5109	20.6067
RAI14	17.4005	17.8743	17.9634	17.3538	17.2427	17.513	18.274	17.7344	17.8503
RALA	20.4984	20.6507	20.9344	21.1314	21.115	21.0864	21.4493	20.8037	21.2191
RALB	19.237	19.0355	19.356	18.7377	19.2195	18.9769	19.7335	18.969	19.1292
RALY	22.6004	22.3095	22.7052	22.4547	22.2449	22.3277	22.5532	22.3409	22.5883
RAN	25.59	25.5802	25.4557	25.4921	25.1864	25.4857	25.5878	25.4233	25.5102
RANBP1	22.892	22.4194	22.6575	22.7067	22.7377	22.8551	23.1156	22.7159	22.7912
RANBP2	21.1821	20.9382	20.9422	20.8431	20.477	20.796	20.8631	20.9659	21.1787
RANBP6	16.9362	16.9268	16.6385	17.4067	16.8315	16.4983	16.6764	16.8909	16.7431
RANBP9	18.1656	17.6279	17.7954	17.4662	17.8544	17.9476	18.0289	17.9705	17.7125
RANGAP1	21.8124	21.943	21.8999	21.7815	21.5781	21.5771	21.9577	21.5978	21.713
RAP1A	20.3479	20.2952	20.0415	20.4599	19.8171	20.4194	19.8111	20.6127	20.8141
RAP1B	21.816	21.5999	21.5313	21.6709	21.1821	21.2382	20.6972	21.3958	21.4738
RAP1GAP2	20.5987	20.6269	20.5562	20.688	20.6306	21.2801	21.6268	21.067	21.3705
RAP1GDS1	20.0362	19.8315	19.9707	19.8923	19.5636	19.6767	19.9599	19.8452	19.7415
RAPH1	17.8128	17.8839	17.9904	17.7749	17.7225	17.7558	17.796	17.9155	18.1171
RARS	22.0402	22.0503	22.1922	21.9434	21.8269	21.6792	22.0135	21.5804	21.7583
RASAL2	16.016	17.0428	16.5432	16.6658	16.5414	16.4741	16.6919	15.9448	16.4004

RAVER1	20.4529	20.005	20.1319	20.2835	20.0312	20.1356	20.4717	20.1429	20.2397
RB1	18.9931	18.1447	18.3876	18.1323	18.2129	17.9577	18.168	18.611	18.8827
RBBP4	23.2836	22.4456	22.6068	23.0423	22.7502	22.9623	23.2184	22.9119	23.1343
RBBP7	21.5303	21.1152	21.2639	21.2117	21.2	21.1487	21.3332	21.0802	21.31
RBFOX2	18.229	18.8189	18.7565	19.0589	18.6277	18.8134	19.336	19.0144	18.8555
RBM10	20.3598	20.2786	20.1609	20.2552	20.2562	20.5593	20.4913	20.3133	20.3406
RBM12	20.857	20.6879	20.9657	20.6614	20.7356	20.7616	21.2833	20.9188	20.8991
RBM12B	18.7748	19.0877	18.8918	18.8392	18.7422	18.438	18.6325	18.7687	19.0104
RBM14	21.9199	21.5759	21.7879	22.0194	21.8353	21.9333	22.2041	21.694	21.9025
RBM15	18.3346	18.8374	18.9094	18.5492	18.4903	18.715	19.0452	18.5767	19.0592
RBM17	19.9887	17.8087	20.6468	19.8716	18.7541	19.275	19.3014	19.2164	20.5165
RBM19	17.3013	16.8562	16.8026	16.4802	17.0821	16.8722	17.5307	17.1878	17.185
RBM25	21.2665	20.9068	21.1015	20.9605	21.0902	21.1379	21.6184	21.1426	21.7994
RBM26	18.0993	18.2349	18.1708	18.1975	17.9407	18.1067	18.5126	18.2054	18.4227
RBM28	16.8752	17.2705	17.0494	16.6539	16.686	16.571	17.2889	17.2196	17.0494
RBM3	21.491	21.1872	21.2706	21.5008	21.2836	21.023	20.7805	21.2521	21.0925
RBM39	21.9347	21.7612	21.7599	21.6604	21.5988	21.7581	22.0371	21.8227	21.9077
RBM4	21.8636	21.58	21.3937	21.5225	21.4356	21.6523	21.8514	21.5822	21.8218
RBM42	19.0803	19.1289	19.2384	18.8854	18.8935	18.9268	19.3893	18.769	18.9433
RBM5	16.7094	17.1511	17.349	17.0592	17.2476	17.6525	17.2075	17.5299	17.985
RBM6	18.5596	18.144	18.4719	18.1471	18.1389	18.2395	18.5483	18.0859	18.1308
RBM8A	21.6455	21.3413	21.5969	21.683	21.3592	21.4378	21.3888	21.4952	21.911
RBMX	20.5247	20.8642	20.2981	20.5798	20.4918	20.6802	20.5984	20.3831	20.8627
RCC1	22.1943	22.3484	22.2631	22.1089	22.0185	22.2923	22.6664	22.361	22.4187
RCC2	22.5692	22.1992	22.4448	22.2293	22.184	21.9406	22.1832	22.118	22.0673
RCL1	19.1213	19.0973	18.8276	18.9098	18.7521	18.9334	19.1306	19.0014	19.0304
RCN1	21.9147	21.833	21.9588	21.9317	21.4151	21.5756	22.1227	21.7094	22.1265
RCN2	20.0258	19.5798	19.6987	19.8476	19.6978	19.4295	19.4107	19.5559	19.494
RCN3	18.4194	19.5492	19.1656	19.5714	19.5713	19.419	19.4666	19.4748	20.2409
RCOR1	19.3767	19.1317	18.861	19.0711	18.6641	18.7776	18.8729	18.9149	18.8793
RDH10	18.8271	18.4657	18.6292	18.4858	18.3562	18.7327	19.1307	18.8058	18.5277
RDH11	18.2579	19.0474	20.2035	19.8355	19.951	20.3948	20.791	20.2226	20.6045
RDX	20.1788	19.9243	19.9699	20.4292	19.9831	19.7568	20.1164	20.0457	20.1097
RECQL	22.1112	22.0316	22.0487	22.0483	21.7257	21.8585	22.0324	21.9097	22.0646
REEP5	20.7391	20.8274	20.6044	20.8109	20.2611	20.7482	20.4823	20.5666	20.887
RELA	19.4203	19.3446	19.0956	19.214	18.9828	18.9795	19.3071	19.1283	19.3313
REPS1	17.151	17.1941	17.5716	16.9496	17.0145	16.5904	17.2808	16.4398	16.3672
RER1	19.9099	19.9611	19.9279	19.906	19.7084	19.2254	19.8027	19.8241	19.8856
RETSAT	17.827	18.8644	19.1807	18.6408	18.4187	18.3603	18.4056	18.2899	18.43
RFC2	19.9473	19.7127	20.4857	20.191	19.7859	20.1033	20.3043	19.9153	20.1841
RFC3	20.1229	19.8403	20	19.9152	19.7205	20.0123	19.4193	19.6869	19.7909
RFC4	20.3941	20.104	20.2119	20.1508	19.9977	20.1291	20.428	20.155	20.1827
RFC5	19.6594	19.3879	19.6019	19.4723	19.3753	19.2357	19.697	19.3476	19.4597
RFTN1	18.1666	18.2803	18.0238	18.1216	17.9382	17.1759	17.291	17.2188	17.1981
RHEB	21.1227	20.4895	20.9111	21.1673	21.0249	21.2193	21.3397	21.198	21.4464
RHOA	22.0673	21.8534	21.9432	21.626	21.6065	21.8328	21.5163	21.7811	22.1133
RHOC	21.6	21.1989	21.4977	21.1854	21.5083	21.8003	22.1732	21.908	21.8998
RHOG	20.226	18.8694	19.7995	19.2585	19.226	19.9614	19.448	19.3361	19.011
RHOT1	18.3187	18.2746	18.4164	18.1802	18.1912	18.1408	18.7436	18.3317	18.3209
RHOT2	19.9969	19.9465	19.7139	19.8078	19.508	19.48	19.777	19.6813	19.7719
RIC8A	19.6716	19.3698	19.4439	19.3562	19.4439	19.4949	19.6783	19.5281	19.0397
RIF1	19.2475	19.0801	19.1188	18.8824	18.8618	18.6017	18.9312	18.7752	18.8407
RIN1	17.4355	17.836	17.57	17.6365	17.6193	16.9918	17.9419	17.1403	17.1231
RING1	17.6033	18.2728	17.6277	17.6022	17.5724	18.1926	18.4505	17.9752	18.1551
RIOK1	18.3162	18.2815	18.8069	18.2489	18.3056	18.699	18.7795	18.2753	18.4596
RMDN3	19.9143	19.5148	19.5533	19.1936	19.4511	19.5619	19.9747	19.6321	19.4149

RNASEH2A	18.8179	19.1171	18.5572	18.6948	18.4618	18.8621	19.0679	18.7525	18.7949
RNF114	20.2469	19.9338	20.1266	19.8051	19.9897	19.982	20.192	19.7095	20.0064
RNF20	24.1613	24.2421	24.1481	24.1547	23.5705	23.3251	22.3583	23.5134	23.8242
RNF213	16.9201	17.3194	17.5854	16.9699	17.1582	17.4021	17.8279	17.6713	17.2698
RNF40	20.6202	20.6402	20.6341	20.4552	20.3784	20.2708	20.6256	20.4731	20.6841
RNGTT	18.3716	18.3157	18.2197	18.172	17.9093	18.0541	18.0436	18.1698	18.0821
RNH1	22.7694	22.8559	22.9357	22.9721	22.8006	23.0012	23.2729	22.7049	22.9045
RNMT	18.4641	17.9955	18.4795	17.9645	18.1087	17.6414	18.2106	18.3056	17.9294
RNPEP	20.8392	20.5857	20.5958	20.7605	20.3698	20.4897	20.7464	20.5959	20.5349
RNPS1	20.2296	20.4379	19.9614	20.3316	20.0343	20.3079	20.3961	20.302	20.4667
ROCK2	20.0973	20.117	20.174	20.0205	19.5579	19.6004	19.8637	19.6512	20.0189
ROMO1	20.8927	20.4968	20.053	20.9713	19.713	20.4978	20.6554	20.8561	20.8933
RPA1	21.6491	21.6665	21.4823	21.4663	21.2933	21.3148	21.7385	21.6419	21.746
RPA2	19.3637	19.2262	19.6423	19.6898	19.7688	19.1351	19.4617	19.9123	19.6083
RPA3	21.2095	21.293	21.244	21.3506	21.1287	21.2689	21.0725	20.9464	21.2423
RPAP1	17.579	17.4742	17.3646	17.346	17.253	16.959	17.5012	17.3748	17.4547
RPAP3	19.3067	19.0992	19.2094	19.0103	19.0033	18.8087	19.3188	19.1244	19.292
RPF2	16.5511	16.8259	16.4801	16.4845	16.7277	16.354	15.9387	16.1686	15.7899
RPIA	18.6472	18.1088	18.9268	18.5097	18.3975	18.8367	18.7119	19.102	18.8035
RPL10	22.2151	22.0203	22.0848	22.143	21.7049	21.74	21.9245	22.163	22.1521
RPL10A	23.2926	23.7737	23.4956	23.4358	23.2776	23.4658	23.4781	23.147	23.3859
RPL11	23.672	23.536	23.5047	23.5857	23.3954	23.5797	23.7314	23.4714	23.6925
RPL12	25.0509	25.0675	25.0893	25.0319	24.9144	25.0737	25.2574	24.9338	25.1268
RPL13	24.3092	24.3278	24.412	24.2379	24.1154	24.2737	24.477	24.2295	24.3049
RPL13A	23.3167	23.0431	23.3893	23.5671	23.5363	23.4265	23.7554	23.4062	23.4943
RPL14	21.6699	21.3028	21.6305	21.8111	21.8617	21.918	21.7539	21.847	21.6336
RPL15	23.542	23.7489	23.6894	23.6415	23.5246	23.3399	23.5803	23.6078	23.5912
RPL17	23.0902	22.6659	23.187	23.0308	23.0292	23.3034	22.9236	22.9802	22.8994
RPL18	21.5402	20.7036	21.3551	21.3609	21.4454	21.3354	21.8399	21.6753	21.5097
RPL18A	23.6685	23.7107	23.6894	23.5544	23.4128	23.6626	23.8248	23.6849	23.8094
RPL19	20.749	20.3836	20.7924	20.9248	20.6698	20.6466	21.1583	20.8128	20.6847
RPL21	23.0339	22.8741	23.0378	23.2716	22.9679	23.0579	23.4257	23.149	23.4416
RPL22	23.7852	24.2933	24.2201	24.0882	24.0451	24.0694	24.2523	24.1421	24.277
RPL23	23.2635	23.3605	23.2021	23.0615	22.8711	23.3038	23.037	23.0914	23.1805
RPL23A	23.1998	23.5135	23.1272	23.3794	23.1783	23.6062	23.7164	23.3628	23.3475
RPL24	22.5567	22.4751	22.4051	22.4068	22.1913	22.4265	22.2562	22.3695	22.6797
RPL26	22.8878	23.0128	23.1559	22.9183	22.9993	23.0297	23.2679	22.9266	22.9944
RPL27	24.2383	23.917	24.2205	23.9808	24.0682	24.0285	24.2317	24.0431	24.1279
RPL27A	24.2948	24.4788	24.4268	24.27	24.1397	24.417	24.2905	24.3514	24.5025
RPL28	21.8749	21.506	21.8574	22.093	22.1493	22.207	22.4158	22.1766	22.4235
RPL3	23.2083	21.4138	23.0982	23.2168	23.211	23.6314	23.5721	23.2686	23.5264
RPL30	23.8237	23.6516	23.6671	23.5233	23.3677	23.4738	23.6207	23.6605	23.5971
RPL31	21.5776	20.3814	21.5897	21.757	21.7304	21.8409	22.0391	21.6353	21.9334
RPL32	21.6166	21.8575	21.6367	21.6334	21.4272	21.701	21.8029	21.7161	21.8721
RPL34	22.6481	22.333	22.8436	23.3714	23.0722	23.1385	24.0764	23.3549	23.6825
RPL35	19.2668	19.312	19.8739	19.938	20.0421	19.3052	19.9769	19.683	19.7947
RPL35A	23.0433	23.4314	23.0255	23.0543	22.8625	22.9516	22.7004	23.0047	23.2054
RPL36	21.7241	21.7515	21.7485	22.0597	22.1244	22.0467	22.2328	22.1056	22.2014
RPL37A	22.105	21.8853	21.8621	22.0353	22.0109	22.0234	22.1043	21.9995	22.154
RPL38	22.8184	22.3839	22.5408	22.5801	22.3245	22.672	22.6851	22.6694	22.4838
RPL39	19.1277	16.7713	18.7906	19.9436	18.8907	20.0367	19.2247	19.8795	19.792
RPL4	24.0739	23.8053	23.98	23.8811	23.8287	23.7944	24.4083	23.9692	24.0059
RPL5	24.1539	23.8125	24.1012	23.8226	23.7946	23.9457	24.2231	23.9649	24.032
RPL6	22.0302	21.3787	22.3039	22.1945	22.4434	22.2305	22.9191	22.2355	22.2101
RPL7	23.4813	23.0516	23.5376	23.5992	23.4903	23.3934	23.6958	23.4616	23.5302
RPL7A	23.5932	23.6912	23.879	23.575	23.3549	23.8008	24.0021	23.7256	23.8368

RPL7L1	18.163	18.5101	18.3001	18.1054	18.079	17.5214	18.3682	18.2572	18.1505
RPL8	22.5654	21.1133	22.4934	22.6448	22.506	22.7722	22.7159	22.5046	22.593
RPL9	22.9849	22.8655	23.0431	22.8192	22.8424	23.0586	23.4436	22.9786	22.8739
RPLP0	24.908	24.6934	24.7025	24.617	24.5035	24.5595	24.8339	24.6447	24.7914
RPLP1	21.9522	21.5817	21.5282	22.0892	22.1334	21.1594	21.9741	21.46	21.98
RPLP2	21.7742	21.4812	21.514	21.7385	21.3074	21.6613	21.8636	21.3994	21.9216
RPN1	23.2299	23.0933	23.3408	23.017	23.0034	22.8523	23.3528	23.1199	23.2082
RPN2	23.0887	22.7751	22.862	22.7374	22.6598	22.3276	22.7644	22.8389	22.8997
RPP30	19.9585	19.9039	19.8888	19.7792	19.6928	19.7127	20.2535	19.8657	19.8161
RPP40	17.5961	17.5135	17.3432	17.4627	17.253	15.5329	17.2912	17.0596	16.5386
RPRD1B	20.4646	20.5173	20.834	20.3596	20.6207	20.425	20.5196	20.2268	20.1354
RPRD2	19.1325	18.9936	19.0976	18.9795	18.914	19.3776	19.3694	18.8448	19.139
RPS10	23.6884	23.5861	23.4221	23.4699	23.5478	22.9952	23.1885	23.2784	23.0949
RPS11	23.5572	24.8147	23.5284	23.414	23.4563	23.8251	23.866	23.5339	23.4678
RPS12	24.5228	24.5878	24.6649	24.582	24.5634	24.817	24.9077	24.6489	24.6159
RPS13	23.3187	22.202	23.6368	23.7473	23.3719	23.6742	23.401	22.9628	22.8297
RPS14	24.1747	24.4133	24.1813	24.1222	24.0533	24.275	24.0436	24.049	24.4273
RPS15	21.0253	19.0475	20.7835	21.3226	20.6574	20.9863	20.7886	20.9579	21.2798
RPS15A	24.3605	24.4002	24.5929	24.2265	24.2425	24.513	24.6781	24.399	24.4565
RPS16	24.1561	23.9539	23.9828	24.081	24.0034	24.1918	24.4656	23.978	24.1104
RPS17L	22.9823	22.239	22.7486	23.0076	22.76	23.0502	23.0938	22.8561	22.9847
RPS18	23.2159	22.6072	23.403	23.4942	23.1513	23.6345	23.3978	23.2117	23.4333
RPS19	23.5709	22.9086	23.7013	23.5043	23.4717	23.8736	23.9051	23.5802	23.618
RPS2	24.4128	24.5514	24.4451	24.4076	24.2336	24.3401	24.4826	24.3074	24.3649
RPS20	21.987	22.6424	21.9922	22.0933	22.0537	21.7923	21.8107	21.7643	22.069
RPS21	21.9734	20.9645	22.2949	22.5335	22.4115	22.3951	22.5477	22.0848	22.7046
RPS23	22.691	22.4445	22.7249	22.7822	22.5974	22.3238	22.7805	22.6167	22.7438
RPS24	22.1835	22.2523	22.1726	22.2222	22.2145	22.407	21.7354	22.0391	21.9262
RPS25	23.3319	23.0771	23.3318	23.0652	22.9684	22.9876	23.5862	23.2468	23.2313
RPS26	22.7888	22.5268	22.5521	22.7078	22.6363	22.2261	23.0342	22.7557	22.8346
RPS27	23.0494	22.9248	22.9531	22.8427	22.6126	23.1824	23.1302	23.0662	23.0803
RPS27A	25.8587	24.8346	25.8413	25.7257	25.8562	25.8975	25.8366	25.652	25.7299
RPS28	23.0416	22.969	22.9369	22.9451	22.9057	23.2005	23.1864	22.8687	23.1703
RPS29	21.1721	20.5854	21.239	20.7233	21.1741	21.7316	22.3747	21.5081	21.2559
RPS3	25.1813	25.2611	25.1293	25.1134	24.8873	25.0458	25.3466	25.0588	25.2262
RPS3A	24.4127	24.2629	24.3971	24.2142	23.9343	24.3475	24.52	24.3671	24.3354
RPS4X	23.4659	23.0969	23.6826	23.7778	23.6483	23.6637	23.8264	23.5698	23.7396
RPS5	23.6103	23.5588	23.4351	23.1862	23.3313	22.3304	23.6062	23.3285	23.019
RPS6	23.964	23.9875	23.9631	23.9676	23.782	24.009	23.9986	23.9839	24.2001
RPS6KA1	20.2789	20.0577	20.3114	20.115	20.1469	20.2791	20.7679	20.453	20.2708
RPS6KA3	19.2352	18.7546	18.9218	19.0432	18.7792	19.041	19.3511	19.0258	18.9292
RPS7	23.5691	22.5618	23.396	23.7844	23.5911	23.6274	23.5886	23.5158	23.806
RPS8	24.5745	24.6387	24.5079	24.5193	24.4603	24.6908	24.9833	24.6798	24.8489
RPS9	22.9248	22.9114	22.9446	22.9332	22.8672	23.0986	23.2255	23.027	22.9648
RPSA	24.7867	23.5144	24.5951	24.7796	24.71	24.901	25.0638	24.7115	24.801
RPTOR	13.5448	17.7355	18.0165	17.9547	17.8826	16.4367	16.9945	17.2545	17.1228
RRAGA	19.24	19.1784	19.2437	19.0652	18.9962	19.1716	19.3787	19.2643	19.3047
RRAGC	19.0171	18.8396	18.8189	18.7001	18.6458	18.2863	18.9944	18.6486	18.5638
RRBP1	22.2232	22.2398	22.0255	22.0821	22.0338	22.2561	22.5082	22.2441	22.6727
RRM1	21.4113	21.5291	21.3699	21.5136	21.3599	21.1237	21.2138	21.2007	21.3799
RRM2	21.975	21.8344	22.015	21.7464	21.6201	21.6222	21.7781	21.6483	21.6519
RRP12	18.7357	18.7554	18.7501	18.6261	18.3185	18.0971	18.6323	18.5487	18.1508
RRP15	19.697	19.5888	19.7105	19.5485	19.3387	19.5442	19.8433	19.5757	19.8102
RRP9	18.3596	17.6151	17.9562	17.9999	18.2684	17.9245	17.6553	17.9578	17.6343
RRS1	19.7881	19.7339	19.5662	19.8393	19.5567	19.3149	19.5115	19.6208	19.4875
RSF1	19.3232	21.4439	19.232	19.3625	19.0373	19.2549	19.7044	19.1521	19.2582

RSL1D1	20.8859	20.9202	20.9559	20.9135	20.8005	20.7869	20.8833	20.6887	20.8827
RSU1	19.5485	19.6337	19.5417	19.6246	19.4979	18.5649	19.5312	19.3199	19.4451
RTCA	19.5774	19.5194	19.5295	19.5631	19.4623	19.5594	19.7123	19.604	19.374
RTF1	19.052	19.0536	19.3396	19.2135	19.136	19.1663	19.4555	19.3594	19.4816
RTFDC1	18.0651	17.9361	18.0678	18.1848	18.0721	18.7391	17.8439	17.9653	17.7847
RTN3	19.8014	21.2506	19.5597	19.8547	19.5995	19.7491	19.9655	19.6468	19.9555
RTN4	23.5837	23.3501	23.7186	23.5619	23.6109	23.3177	23.6309	23.2714	23.3838
RUNX1	20.1209	20.5346	20.4197	20.596	20.2462	20.6203	20.565	20.205	20.4126
RUVBL1	22.8174	22.8714	22.7507	22.7466	22.66	22.7509	23.1797	22.6738	22.9296
RUVBL2	22.407	22.4911	22.3243	22.3412	22.0981	22.194	22.4041	22.1233	22.6229
S100A10	24.1151	23.7632	23.7819	23.7932	23.6901	23.9038	24.5179	24.1328	24.1358
S100A11	21.2842	20.4616	20.8543	21.1748	21.1254	20.8938	21.1949	20.5101	21.2871
S100A13	20.0719	19.1797	19.7883	20.0421	20.0075	20.2516	20.1085	20.1926	20.428
S100A6	22.4828	21.756	22.3569	22.657	22.3397	23.1492	22.8955	22.6112	23.2175
SAAL1	19.326	19.3428	19.3088	19.2702	19.2549	20.2978	19.5612	19.1437	19.2727
SACM1L	19.7237	19.3903	19.6015	19.3668	19.3358	19.6678	19.8661	19.673	19.6444
SAE1	21.1005	21.4967	21.3958	21.323	21.0235	21.1502	21.2562	21.0131	21.2953
SAFB	20.9219	21.0123	21.3358	21.1451	21.3359	21.6158	21.8323	21.4015	21.715
SAFB2	19.814	19.758	19.7158	19.8622	19.6977	19.9832	19.9476	19.7686	20.0708
SAMHD1	19.1716	18.8713	19.1141	19.0398	18.8765	19.1181	19.2549	19.3615	19.2569
SAMM50	21.1445	21.0331	21.2434	20.8082	20.8049	20.6613	21.3368	20.858	21.1248
SAP18	19.3114	19.4011	19.611	19.5698	19.6934	19.5863	18.9805	19.3896	19.2585
SAP30BP	23.2654	23.4024	22.364	23.1088	22.6774	22.6643	21.5661	22.5869	22.8337
SAR1A	20.8488	21.1092	20.8567	20.8541	21.0302	21.1807	21.1122	20.9135	20.9538
SAR1B	16.7854	16.5574	16.8944	16.2595	16.7491	16.0359	16.7475	17.2479	16.0622
SARNP	21.2844	21.0976	21.3054	21.1	21.0231	21.3624	21.5408	21.0657	21.4826
SARS	21.5742	21.4708	21.4704	21.4952	21.1852	21.306	21.3158	21.4806	21.5334
SARS2	18.0177	18.5998	18.7225	18.4629	18.3912	18.6924	19.082	18.4482	18.7502
SART1	20.3022	19.9289	20.3158	20.281	20.267	20.4249	20.5619	20.4668	20.7871
SART3	20.7434	20.6996	20.567	20.6169	20.5208	20.3007	20.546	20.5325	20.5847
SBDS	21.0377	20.6982	21.1171	20.6566	20.6736	20.9768	20.8845	20.6835	20.5737
SCAF11	18.8303	17.9509	17.7986	18.2922	17.7715	17.103	17.5905	17.8904	17.8244
SCAMP2	19.9725	19.2501	19.6304	20.2477	19.4138	20.0049	19.9913	19.8055	19.8945
SCAMP3	21.4709	19.5628	21.5815	21.543	21.5752	21.9842	22.0905	21.587	21.772
SCARB2	18.9106	20.0732	18.7297	19.0317	18.6809	16.7938	18.1712	18.7465	18.466
SCCPDH	20.1652	20.2357	20.3516	20.1382	20.0363	20.4811	20.9066	20.529	20.3703
SCFD1	19.4679	19.2533	19.228	18.9748	19.0201	19.2758	19.4248	19.1943	19.3111
SCP2	20.4408	20.4784	20.8958	20.7723	20.8208	20.9336	21.2276	20.4886	20.9554
SCPEP1	16.6984	17.3584	17.009	16.6738	16.9748	17.8732	16.9876	17.3191	16.9109
SCRIB	18.1737	18.2791	17.9014	17.998	17.8254	17.5814	17.6583	18.0127	17.588
SCRN1	21.78	21.5873	21.4034	21.6299	21.4608	21.679	21.6633	21.4976	21.5055
SCYL1	19.3704	19.221	19.1791	19.1091	18.9716	19.006	19.4955	19.1641	19.0277
SDF2L1	20.9323	20.5175	20.8605	20.7926	20.6755	20.9149	20.7045	20.8564	20.979
SDHA	20.9395	20.684	20.8047	20.9356	20.5145	20.4635	20.647	20.618	20.8186
SDHB	20.052	19.7996	20.1572	19.7372	19.6567	19.9221	19.9621	19.8699	20.1784
SDHC	18.9875	18.7061	18.956	18.8007	18.6647	18.6917	18.8152	18.5971	18.894
SDHD	17.871	18.2274	18.1214	17.9377	17.5473	18.2137	17.1314	17.8286	17.8991
SEC11A	21.0183	21.2646	21.265	20.8168	20.7341	20.9301	21.3109	21.0844	21.4013
SEC11C	19.428	19.5119	19.1354	19.8753	19.5306	19.5095	19.6426	18.9	19.0288
SEC13	21.4388	20.8838	20.8209	21.1494	20.8843	21.1639	21.3754	21.1106	21.3292
SEC14L2	18.5576	17.8926	18.8236	18.9679	18.9656	19.548	19.4496	19.4335	19.4311
SEC22B	20.6476	20.6361	20.937	20.534	20.6099	20.7544	21.0065	20.7183	20.9343
SEC23A	20.4347	20.0538	20.1165	19.9879	20.1203	19.9346	20.2169	19.952	19.8393
SEC23B	21.0391	20.4974	21.2307	21.0532	20.8351	21.1366	21.0579	20.9936	21.2144
SEC23IP	20.3812	20.1746	19.9153	20.1916	19.7999	19.9392	19.8162	20.2217	20.3531
SEC24A	19.5133	19.2259	19.1018	19.2091	19.0108	18.3854	19.2382	19.3408	19.2832

SEC24B	18.2987	18.2268	18.3351	18.2298	18.081	17.8025	18.2842	18.2055	18.3073
SEC24C	21.4149	21.2131	21.2685	21.1065	21.1542	21.4272	21.8723	21.4193	21.3611
SEC24D	18.5284	18.404	18.4398	18.2921	18.0937	17.7931	18.3455	18.2894	18.0882
SEC31A	21.5614	21.0082	21.8251	21.294	21.3424	21.8753	22.2926	21.6688	21.7376
SEC61A1	23.0895	21.3171	23.2914	23.359	23.1061	23.2113	23.3524	22.9914	23.4859
SEC61B	20.5572	19.7972	20.5864	20.5937	20.2673	20.6445	20.8754	20.4959	20.9251
SEC63	19.7275	20.1329	20.2157	19.7818	20.2176	20.2109	20.0516	20.0251	20.1321
SEH1L	19.7806	19.7295	19.6425	19.6664	19.4678	19.4378	19.4542	19.7853	19.7264
SEL1L	19.4161	18.7286	18.8619	18.4655	18.8163	18.0372	18.3599	18.3175	18.547
SENP3	18.5823	18.2659	18.4942	18.1477	18.5735	18.4428	18.8322	18.6059	18.586
SEPHS1	19.701	19.6487	19.7003	19.5078	19.4024	19.6975	19.6636	19.2894	19.4163
Sep 10	18.3706	18.623	18.7079	18.8046	18.2805	18.4594	18.9283	18.758	19.1191
Sep 11	21.4772	20.7075	20.9917	20.9904	20.7196	20.9381	20.5124	20.7499	21.0527
Sep 02	22.4127	22.241	22.2723	21.9035	22.1236	22.1194	22.4472	22.205	21.9474
Sep 07	22.0238	22.0491	22.0205	21.7611	22.0204	21.8174	21.932	21.9699	22.1224
Sep 08	20.5524	20.209	20.0696	20.2454	20.1184	20.1462	20.1803	20.1112	20.1419
Sep 09	22.8684	22.8725	23.0122	22.8291	22.7148	23.0783	23.3421	22.836	23.148
SERBP1	21.6036	21.2346	21.1419	21.8603	21.4122	21.7195	20.9154	21.2485	21.5626
SERPINA3	19.5772	21.0328	19.3023	19.0599	19.1712	19.5703	19.8036	19.7856	19.8027
SERPINB5	22.3751	22.4044	22.3293	22.4185	22.1069	22.481	22.6904	22.4974	22.5772
SERPINB6	20.9575	20.5733	20.8143	20.6693	20.4239	20.9247	21.0164	20.8134	21.0473
SERPINB8	20.0081	19.69	19.7608	19.7765	19.4821	19.8028	19.8501	20.0234	20.2079
SERPINE2	20.5572	20.0973	20.6288	20.2771	20.5332	20.9315	21.3295	20.6967	20.3858
SERPINH1	24.5838	24.2325	24.3236	23.9225	24.2301	24.0601	24.5269	24.0248	23.995
SET	22.7487	22.3196	23.113	22.9627	22.8688	23.7227	24.1045	23.3255	23.392
SETD3	14.428	14.6217	15.1177	14.0584	14.5529	14.0353	13.7442	15.1434	14.9259
SF1	21.1578	19.6649	20.6661	20.8379	20.809	20.9446	20.7445	20.7904	20.5667
SF3A1	22.9907	22.6408	22.6123	22.7998	22.5426	22.8253	23.0808	22.826	23.0443
SF3A2	20.6467	19.7532	20.4838	20.546	20.256	20.0404	20.1236	20.2677	20.5869
SF3A3	22.5095	22.002	22.6636	22.3311	22.3478	22.7172	23.1195	22.5846	22.6791
SF3B1	22.4394	22.0351	22.2296	22.2388	22.1748	22.5033	22.9963	22.1759	22.2401
SF3B14	20.6095	20.3985	20.2423	20.0049	20.3104	20.5026	20.4059	20.5452	20.1874
SF3B2	22.3918	21.4284	22.2996	22.5381	22.36	22.5415	22.5925	22.2167	22.4995
SF3B3	23.4659	23.0539	23.0192	22.9346	22.786	22.6024	23.0249	22.8969	23.1033
SF3B4	21.8619	21.5618	21.6793	21.6376	21.6193	21.7232	21.5507	21.8649	21.7969
SF3B5	18.5476	18.4732	18.9139	18.9836	18.7674	18.6545	18.1119	18.5262	19.0743
SFPQ	24.6194	24.21	24.5329	24.2548	24.2222	24.2124	24.5318	24.332	24.4682
SFT2D2	16.9539	17.257	17.0049	16.8862	17.3641	17.7981	17.4288	18.0672	17.5782
SFXN1	21.201	21.3719	21.1804	21.1166	21.165	21.3373	21.5795	21.1769	21.3556
SFXN3	20.3564	20.5057	20.3844	20.2465	20.1619	20.1934	20.4079	20.3751	20.4486
SGK196	17.0103	16.7038	17.2995	17.7042	17.4889	17.3725	16.6274	16.0873	16.2891
SGPL1	16.9326	17.5325	17.3664	17.4063	17.6024	16.8079	17.9095	18.0122	17.7653
SGTA	20.1523	20.1072	20.2899	20.104	19.8865	20.0313	20.3086	20.107	20.2424
SH3BGRL3	21.4564	21.3048	21.3841	21.5395	21.2747	21.3746	21.1095	21.3454	21.4463
SH3BP1	17.6453	17.8338	17.5941	17.6902	17.477	17.5762	17.7933	17.6325	17.7759
SH3BP4	18.8641	18.7711	18.8508	18.6233	18.4662	18.4824	18.937	18.4369	18.5817
SH3GL1	19.4615	19.0399	19.0075	19.4331	18.9429	19.501	19.3263	19.7625	19.6239
SH3GLB1	19.7984	19.4807	19.8519	19.6974	19.4652	19.6064	19.6037	19.3485	19.5335
SH3KBP1	19.6814	21.9087	19.6952	18.9957	19.9155	20.1185	19.5729	19.373	19.5814
SHC1	17.3696	17.2219	17.7674	17.6386	17.6378	16.8054	16.437	17.5485	17.2671
SHMT1	20.1829	19.8295	20.0519	19.9239	19.7484	20.1361	20.3785	20.0853	20.072
SHMT2	22.8105	22.7873	22.6572	22.5451	22.4344	22.6038	22.8384	22.5112	22.7557
SIN3A	18.0112	18.3246	18.2408	18.0584	17.9437	17.9327	18.5745	18.2499	17.9439
SKIV2L	18.1015	18.102	18.1639	18.3672	18.0251	18.2131	18.6413	18.2505	18.4822
SKIV2L2	20.8945	21.0646	21.0749	20.8964	20.8808	20.9126	21.4232	20.9488	21.1481
SKP1	21.172	20.764	21.0899	21.1209	21.0102	20.8367	20.9545	21.1886	21.379

SLC12A2	19.1096	18.9458	18.6844	18.7695	18.9392	18.7411	19.246	18.7817	18.9202
SLC12A4	16.5231	16.9507	16.7652	16.8803	16.4705	16.7003	17.1926	17.0845	16.6075
SLC12A9	16.9307	16.009	16.0347	16.2606	15.9711	15.4952	15.8727	15.6276	15.2816
SLC16A1	17.6031	17.8932	17.5182	19.8392	17.8166	19.6328	16.9989	17.3007	17.2248
SLC1A4	22.6441	22.4193	22.3451	22.4643	22.284	22.3729	22.655	22.5269	22.8679
SLC1A5	22.0891	22.2051	22.0179	21.9458	22.075	21.1689	21.7638	21.7424	21.647
SLC25A1	19.8581	19.6524	19.5753	19.4597	19.4014	19.8031	19.974	19.6842	19.7441
SLC25A10	20.8939	21.2479	20.7252	20.6984	20.5507	21.3939	21.6189	21.2301	21.5723
SLC25A11	21.0037	20.9661	20.8517	20.6363	20.6102	20.7027	21.0249	20.7481	21.0492
SLC25A12	17.7836	18.6834	18.7247	18.2618	18.1343	18.0722	18.5422	18.5269	18.4855
SLC25A13	21.5548	21.5401	21.553	21.6826	21.4225	21.6252	22.0749	21.5624	21.9268
SLC25A22	20.1729	19.7436	20.0696	20.034	20.0103	20.3087	20.7891	20.3881	20.3757
SLC25A24	19.9294	20.1558	20.0737	19.7916	19.6219	20.4329	19.9522	19.9417	20.3333
SLC25A3	22.9502	22.6478	23.0259	23.0127	22.8094	22.7032	22.6375	22.6251	22.8714
SLC25A4	20.3286	20.0264	19.9853	19.86	19.8283	19.6537	19.6789	19.5934	19.6457
SLC25A5	23.7699	23.5324	23.5694	23.3235	23.5198	23.6217	24.2122	23.7283	23.6403
SLC25A6	22.4014	22.1993	22.0381	21.9445	21.9288	22.2052	22.4286	22.2323	22.2509
SLC26A2	20.524	20.4051	20.4339	20.2367	20.3015	20.2583	20.5427	20.3683	20.5171
SLC29A1	18.2362	18.3406	18.1699	18.0704	18.1577	17.328	17.9599	17.9475	17.9495
SLC2A1	20.9383	21.1688	21.4063	21.2568	21.3779	21.8185	22.0241	21.6861	22.0015
SLC30A7	20.1752	19.6073	20.2784	20.2957	20.2497	19.9672	20.3934	20.0416	19.8277
SLC35B2	19.8014	20.0923	19.5183	19.4068	19.416	19.9121	20.4972	19.824	20.0324
SLC39A7	16.5858	16.753	16.5362	16.1841	16.6641	16.1479	17.9506	17.3839	17.0165
SLC3A2	24.5838	24.3299	24.5475	24.4058	24.2311	24.7218	25.2707	24.9157	24.9804
SLC4A1AP	19.4082	18.7252	19.3569	19.1636	19.0667	18.9539	19.2864	19.0518	19.1054
SLC4A2	17.3022	17.933	17.8004	17.4353	17.445	18.205	18.3702	18.1809	18.3523
SLC4A7	17.5378	17.3353	17.6827	17.3927	17.6651	16.1073	16.5436	16.7748	16.1045
SLC5A3	18.9873	19.456	18.5972	18.4568	18.5014	18.592	18.561	18.5821	18.7614
SLC7A1	18.2693	18.3394	18.3366	18.1129	18.1312	18.5851	18.2039	18.475	17.9605
SLC7A5	22.0317	21.6065	22.111	22.0553	22.0638	22.3244	22.5307	22.2734	22.7061
SLC9A3R1	18.129	17.9593	18.53	18.6584	18.234	18.5725	19.0251	18.3195	18.8632
SLC9A3R2	16.9394	13.8135	15.7914	17.146	16.3664	14.4038	15.8971	15.7413	16.7245
SLIRP	20.4333	19.4639	20.6272	20.3338	20.4407	20.6777	21.14	20.5299	20.3437
SLK	19.0446	19.3463	19.0258	19.2524	18.6647	18.5773	18.3938	18.981	19.2524
SMAD3	20.8105	21.0625	20.5717	20.5282	20.439	20.7583	20.9065	20.5424	20.803
SMAD4	18.9548	20.06	19.04	19.3096	18.7796	19.1774	19.3706	18.8415	19.0454
SNRPGP15	20.4609	20.7997	20.7506	20.5463	20.844	21.4325	21.2843	20.9109	20.8425
SMAP1	18.4665	18.5009	18.557	18.5477	18.5316	18.4397	18.7558	18.4176	18.738
SMARCA1	17.1166	17.9766	17.6425	17.5117	17.3161	17.9541	18.0809	17.8118	17.9462
SMARCA4	17.0782	17.8015	16.5527	16.5441	16.1657	16.7089	17.0481	16.7922	17.1974
SMARCA5	20.2675	19.9262	20.1792	20.0654	20.16	20.2027	20.7446	20.1987	20.3986
SMARCAD1	17.6859	17.6411	17.2726	17.4435	17.4887	16.3772	17.1542	17.4189	17.1262
SMARCB1	20.1387	19.9098	19.8949	19.9695	19.8814	20.0408	19.9683	19.8994	20.178
SMARCC1	19.0204	18.8504	18.7822	18.8726	18.8015	18.7035	18.8142	18.83	18.6669
SMARCC2	19.6839	19.7569	20.1351	19.6886	19.737	19.7835	20.3172	20.0976	20.2187
SMARCD1	19.2542	19.4258	19.6485	19.2086	19.1984	19.5196	20.0429	19.2628	19.5233
SMARCE1	19.277	18.6119	19.5303	19.5483	19.3092	19.6914	19.6853	19.1018	19.5337
SMC1A	21.2987	20.9288	21.1525	21.1692	21.0786	21.0693	21.1345	21.0742	21.2555
SMC2	21.4952	21.4629	21.3634	21.2616	21.1886	21.1489	21.496	21.2011	21.378
SMC3	21.3623	21.2633	21.1559	21.2717	20.9345	20.6589	21.1727	20.9599	21.2044
SMC4	21.5414	21.4312	21.3462	21.3399	21.0985	21.1284	21.4342	21.2029	21.415
SMCHD1	17.496	18.1981	17.9322	18.0186	18.1025	18.3886	18.7286	18.2771	18.3813
SMEK1	17.1252	17.4734	17.0591	16.8929	16.9254	16.0077	17.0774	16.87	17.2056
SMEK2	17.5783	17.8125	17.6569	17.5468	17.5589	17.1889	18.153	17.5218	17.6329
SMG9	16.6133	16.7956	16.4828	17.5861	17.2131	15.1781	16.0414	16.5694	16.7189
SMN1	23.1132	18.6279	22.882	23.1574	22.6445	23.6577	22.9673	22.9006	23.3558

SMNDC1	17.0819	16.883	17.057	17.5396	16.6133	17.7207	15.375	17.0242	17.4606
SMPD4	18.5234	18.6529	18.6414	18.4086	18.5577	18.6081	18.8477	18.7047	18.6568
SMS	21.5531	21.1795	21.5257	21.5486	21.3878	21.622	21.6681	21.4898	21.6534
SMTN	16.5795	16.492	17.4704	16.9366	16.6828	16.8933	16.9444	16.4491	16.6718
SMU1	21.4776	21.4147	21.4917	21.3097	21.1886	21.3529	21.5831	21.4145	21.4769
SND1	23.5699	23.6116	23.5509	23.4789	23.3376	23.6511	23.8792	23.6954	23.9225
SNRNP200	22.7832	22.0425	22.4114	22.4031	22.3774	22.1886	22.6385	22.2455	22.4218
SNRNP40	20.8905	20.7846	20.7802	20.9154	20.5813	20.9881	20.8854	20.682	20.8397
SNRNP70	21.64	21.6278	21.4137	21.5888	21.3598	21.5854	21.9293	21.447	21.5748
SNRPA	21.942	21.4767	21.5533	21.6069	21.3846	21.6747	20.9448	21.4906	21.6269
SNRPA1	21.4214	21.6083	21.6188	21.5704	21.4289	21.9931	22.0945	21.7126	21.8142
SNRPB	23.4079	23.4704	23.5074	23.2181	23.1223	23.1837	23.3599	23.4324	23.5372
SNRPB2	21.0262	20.9144	20.7697	20.9475	20.9274	20.7278	20.5394	20.7511	20.7168
SNRPC	22.1454	21.6923	21.4817	21.6543	21.0851	21.4995	21.5807	21.5485	21.7426
SNRPD1	22.6382	22.4264	22.7867	22.202	22.5108	22.3845	22.908	22.6654	22.7997
SNRPD2	23.7398	23.5424	23.5201	23.4019	23.4033	23.6572	23.4748	23.5448	23.6532
SNRPD3	22.8023	23.0433	23.1227	22.8919	22.6798	22.9338	23.0672	22.8156	23.0983
SNRPE	22.5589	22.0259	22.0408	21.9644	21.7413	21.8913	21.9866	21.8779	22.007
SNRPF	20.3351	19.8381	20.1608	20.2914	20.003	20.3587	20.5012	20.2005	20.6652
SNW1	19.4642	19.3257	19.4401	19.5624	19.4013	19.6307	19.2488	19.0118	19.3417
SNX1	20.8718	20.8768	20.8088	20.6631	20.6711	20.5686	20.9356	20.525	20.7943
SNX12	19.4443	19.9208	19.9692	19.9956	19.8914	20.1172	20.3039	19.9211	20.3746
SNX17	17.2865	17.6417	17.6475	17.8669	17.7023	17.791	17.601	17.6302	17.758
SNX2	22.2902	22.2269	22.7111	22.6369	22.3464	22.5727	22.8468	22.1747	22.2404
SNX27	19.2368	18.5882	19.0192	18.9712	18.7498	19.1284	19.7184	19.3774	19.4737
SNX3	20.5743	20.5633	20.9098	20.6608	20.546	20.7538	20.8885	20.5744	20.6933
SNX4	17.9226	17.7408	17.6844	17.4336	17.4481	17.8095	17.9314	17.9784	17.9065
SNX5	21.1408	21.065	21.1682	20.84	20.7843	20.7449	20.9593	20.8727	21.1311
SNX6	18.6196	18.6041	18.093	18.0065	17.9509	18.7361	18.4656	18.3879	18.4416
SNX9	19.1546	19.006	19.1809	19.0474	18.8467	18.6812	19.0718	19.012	18.9843
SOAT1	19.3857	19.8486	19.3981	19.3572	19.332	19.3725	19.71	19.4961	19.4744
SOD1	21.5656	21.4442	21.467	21.671	21.31	21.602	21.8107	21.3781	21.9237
SOD2	23.1296	22.0311	22.8155	22.9899	22.8965	22.2732	22.3287	22.5345	22.561
SON	19.6759	19.4691	19.374	19.4932	19.4283	19.5283	19.6077	19.3332	19.5782
SORD	20.1494	19.6926	20.1359	20.1683	19.9328	20.1738	20.4523	20.0137	20.3245
SORT1	18.4203	18.7418	18.6525	18.6463	18.4082	17.0314	17.5162	17.5711	17.9323
SOX10	20.7657	20.0542	20.8515	20.5369	20.6608	20.9168	21.1305	20.7675	20.9693
SP100	18.764	18.3502	18.4793	18.1068	18.3348	18.3475	18.6174	18.5541	18.1172
SPAG9	19.5985	19.1189	19.3657	19.1255	19.1091	19.2195	19.2216	19.1367	19.0008
SPATA5	18.8789	18.4425	18.7777	18.5602	18.6353	18.6609	18.993	18.7137	18.3239
SPATA5L1	18.0632	18.0207	18.1643	18.1122	17.7622	18.0182	18.3343	18.0509	18.062
SPATS2L	20.0278	20.1282	19.7048	20.0703	19.6735	19.7233	19.7558	19.6791	19.95
SPC24	17.1583	17.4536	17.3984	17.2417	17.4575	17.0581	17.3818	17.6388	17.7141
SPC25	19.6019	19.714	19.5502	19.3396	19.5199	19.6495	19.6294	19.6033	19.7038
SPCS1	18.8985	18.9047	18.8063	19.0432	18.8964	18.881	18.7682	18.6599	19.0757
SPCS2	20.7573	21.0042	20.8842	20.9652	20.8019	20.8529	20.9591	20.8043	20.9937
SPCS3	21.0014	20.3852	20.7912	20.5618	20.7366	20.8981	21.3284	20.7318	21.0649
SPECC1L	19.0857	19.0423	18.2794	18.0895	19.0024	18.1933	19.1348	19.0043	18.6887
SPG20	19.1776	19.0597	19.2385	18.9876	19.1114	19.1643	19.6744	19.3043	19.7027
SPG21	15.3017	16.2042	15.5385	15.3617	15.7344	14.2998	12.1704	13.8378	14.1416
SPIN1	16.6304	16.6075	16.8732	16.2711	16.9216	15.1639	16.8458	16.1828	16.3113
SPR	19.0354	20.4586	18.917	18.771	18.5645	19.2487	20.0086	19.3404	19.7781
SPTAN1	22.118	21.597	21.5929	21.6492	21.4845	21.4432	21.827	21.7413	21.7983
SPTBN1	22.026	22.2669	22.0949	22.1134	22.1129	21.9342	22.3574	21.7943	22.0139
SPTLC1	17.8376	18.1816	18.013	17.6248	18.0582	17.1205	18.0336	17.9077	17.9799
SPTLC2	16.9437	16.4417	16.4178	16.7553	16.6904	16.8237	16.6919	16.8277	16.5555

SQSTM1	19.7969	18.9395	19.6091	19.1929	19.0747	20.2807	20.8793	19.8666	19.5827
SRC	17.5893	17.2609	17.6316	18.1212	17.4466	17.2527	17.3037	17.6504	17.8486
SRGAP2	17.5347	17.7317	17.1777	17.3722	17.2951	16.8445	17.3193	17.4068	17.8454
SRI	21.9489	22.0935	21.8029	21.7264	21.6257	21.8941	22.0292	21.5889	21.7926
SRM	21.6603	21.6347	21.5716	21.5598	21.413	21.7201	22.0408	21.6699	21.7046
SRP14	21.2196	21.2585	20.9327	21.3079	20.997	21.1481	21.0723	20.9651	21.2079
SRP19	19.5456	19.417	18.9874	19.5341	19.3118	19.7725	19.3537	19.5732	19.4911
SRP54	21.3172	21.0937	21.0942	21.0154	20.9071	20.418	21.1488	21.0257	20.9288
SRP68	20.4275	20.2775	20.2829	20.0206	20.1249	19.8763	20.4649	20.3054	20.3553
SRP72	21.2039	20.5503	20.8558	20.9976	20.9856	20.9041	21.028	20.9855	20.9467
SRP9	22.4789	22.0598	21.8823	22.1206	21.7465	22.0782	22.3642	22.2213	22.1436
SRPK1	18.6057	18.3779	18.5009	18.3764	17.893	17.9542	18.0592	17.9827	17.6903
SRPR	19.8229	19.5589	19.8086	19.6021	19.5847	19.2423	19.5787	19.3548	19.5995
SRPRB	21.4968	21.5576	21.7352	21.4222	21.4019	21.3903	21.5497	21.354	21.6109
SRRM1	20.0361	19.7494	19.6688	19.8209	19.6027	19.7986	19.8015	19.6761	19.8724
SRRM2	21.7987	20.9068	21.6595	21.8812	21.6892	22.0172	22.1927	21.8142	22.0641
SRRT	21.5257	21.0664	21.3487	20.9777	21.0214	21.1465	21.3528	21.1989	21.1161
SRSF1	23.7126	23.6635	23.5728	23.8232	23.5432	23.7113	23.9292	23.6779	23.8531
SRSF10	20.0427	19.6483	19.465	20.5151	19.4906	18.907	19.953	19.8027	19.8631
SRSF11	20.7256	20.2392	20.3807	20.665	20.5091	20.7909	21.0142	20.5535	20.8219
SRSF2	21.5259	22.2582	21.3437	21.5979	21.5147	21.7784	22.1461	21.6102	22.0434
SRSF3	23.3349	23.6245	23.5005	23.109	23.3503	23.4639	24.2283	23.5286	23.4912
SRSF4	18.7251	18.7996	18.4206	19.0147	18.2784	18.6289	18.1398	18.8486	18.7794
SRSF5	20.8472	21.7102	21.1202	21.4206	20.9069	20.7147	20.4396	21.1968	21.4517
SRSF6	21.6419	20.2405	21.1554	21.3667	21.3628	21.7115	21.9468	21.3607	21.7243
SRSF7	22.8705	22.8625	22.8103	22.8513	22.5846	22.8195	23.2664	22.8474	23.0142
SRSF9	21.7168	21.7735	21.6957	21.8829	21.6943	21.7218	21.878	21.7518	21.8822
SSB	22.6517	22.5176	22.2906	22.5331	22.3563	22.602	22.326	22.503	22.2693
SSBP1	22.5916	22.6222	22.7947	22.7347	22.5423	22.7867	22.912	22.7867	23.1935
SSR1	21.4222	21.3771	21.3826	21.4385	21.2066	21.4545	21.7167	21.3387	21.5215
SSR3	19.986	19.8898	19.6165	20.1311	19.2596	19.9383	19.8268	20.1686	20.4355
SSR4	21.9765	21.998	22.1929	22.1996	22.1658	21.8367	22.4935	22.3318	22.378
SSRP1	22.8052	22.7697	22.7912	22.67	22.6399	22.7122	23.1566	22.8179	22.8492
SSSCA1	19.5956	19.3267	19.2172	19.6028	19.142	19.3926	18.8442	19.213	19.4027
SSX2	18.6542	18.3441	18.3512	18.1467	17.9729	18.2054	18.2939	18.1601	18.489
ST13	23.198	23.3799	23.4013	23.4471	23.3467	23.5632	23.8769	23.5491	23.7769
ST3GAL4	14.041	15.8549	15.9094	14.7346	16.0007	15.955	15.1029	15.8764	15.5651
STAG2	18.9264	18.7171	18.9413	19.2363	19.0828	18.5842	18.5833	18.6425	18.408
STAM	19.3599	18.9377	19.1975	18.8782	18.8759	18.8249	19.23	18.878	19.1476
STARD13	19.3484	18.7081	18.7637	18.6441	18.4035	18.6567	18.9591	18.7429	18.8502
STAT1	20.6365	20.0409	20.4185	20.075	20.1057	20.1301	20.4129	20.1229	20.1094
STAT3	20.1157	19.5792	20.0912	20.1673	19.6946	19.4157	19.8716	19.7195	19.8809
STAU1	20.9627	20.7833	21.2255	21.0742	20.859	21.1236	21.5034	20.9161	21.2043
STIM1	18.6451	18.4751	18.1236	18.3366	18.2304	18.1642	18.2137	18.2711	18.3699
STIP1	24.0492	23.3686	23.9584	23.8967	23.8057	23.8826	24.0136	23.7284	23.8099
STK24	19.8715	19.8883	19.9973	19.9591	19.6649	19.5268	19.7053	19.6425	19.6925
STK39	18.1521	18.4327	18.4065	18.2889	18.2744	17.7238	17.8305	18.3	18.0878
STMN1	22.7201	22.8159	22.9337	22.6336	22.6413	22.6929	22.9407	22.4735	22.7913
STOM	17.4874	16.6827	16.9432	16.7581	17.6181	18.0483	18.3224	18.1333	18.2569
STOML2	21.171	20.2178	20.9152	21.0612	20.7995	20.6781	21.1099	20.9426	21.3565
STRA6	18.2305	18.5263	18.3869	18.169	18.3119	18.9318	19.618	18.7974	19.0811
STRAP	22.4675	22.4693	22.1486	22.5505	22.1538	22.0928	22.1312	22.1213	22.3687
STRN	19.3721	18.7325	18.9099	18.709	18.572	18.9606	19.0682	18.6983	18.7607
STT3A	21.3576	21.5141	21.1963	21.179	21.0384	20.8936	21.5323	21.2398	21.3262
STT3B	20.716	20.8201	20.6466	20.6426	20.6006	20.5283	20.8009	20.8498	21.0682
STUB1	21.6165	21.7515	21.7826	21.729	21.4847	21.6155	21.9524	21.326	21.6871

STX12	18.0973	17.1706	18.3273	19.1061	18.5927	19.1488	18.8481	17.6815	18.0748
STX6	16.1857	16.4641	16.1026	17.5157	16.4098	15.9327	15.2741	16.3545	16.4019
STXBP1	21.3536	20.5049	22.4276	20.2729	21.5891	22.3654	22.9739	22.2958	21.5876
STXBP3	17.8276	17.6971	17.7756	17.7395	17.6588	17.8527	18.218	18.1333	18.0672
SUB1	21.2452	21.2391	21.0501	21.3659	20.6474	20.9317	20.4458	21.0694	21.2559
SUCLA2	20.2319	20.3979	20.0958	19.9576	19.9773	20.18	20.4649	20.168	20.5125
SUCLG1	20.5003	20.704	20.5574	20.5833	20.3647	20.5199	20.825	20.4649	20.7378
SUCLG2	21.1855	20.9527	21.0581	20.7358	20.8444	20.895	21.0565	20.8406	20.8476
SUGP1	16.9164	17.7515	17.6434	17.7365	17.3792	17.8046	18.0944	17.9722	17.9952
SUGT1	21.5824	20.8086	21.218	21.1859	21.067	21.4267	21.1471	21.0126	21.2716
SUMF2	18.9975	18.6866	18.9513	18.8853	18.7581	19.3153	19.1556	19.2386	19.3528
SUN1	17.3532	17.2625	17.2387	16.9823	17.1109	16.6831	17.2272	16.8228	16.9038
SUN2	19.577	19.2509	19.1623	19.5688	19.2005	18.9317	19.3406	19.6419	19.4177
SUPT16H	22.801	22.5654	22.3896	22.5426	22.4213	22.0856	22.702	22.4757	22.6193
SUPT5H	20.4626	19.8552	19.5863	20.0833	19.4594	20.0617	20.1991	19.9433	19.9855
SUPT6H	20.5766	20.5762	20.5829	20.4129	20.4369	20.494	20.1263	20.7543	20.4389
SUPV3L1	18.2908	18.5504	18.8372	18.8618	18.4019	18.712	18.7953	18.6091	18.7418
SURF4	20.2666	20.3542	19.9413	20.1593	20.0898	19.6471	19.6652	20.2101	20.2117
SURF6	17.8202	17.7053	17.8396	18.0156	17.8558	17.9542	18.0691	17.631	17.9222
SVIL	16.8974	16.8245	16.7058	16.4085	16.2546	15.8286	14.5393	15.5533	15.7685
SYAP1	17.128	16.7895	17.3669	17.8775	17.5275	18.259	17.7922	17.6126	17.2587
SYMPK	18.981	19.3559	18.7844	19.0554	19.4257	19.0693	19.7865	19.4023	19.3081
SYNCRIP	24.1253	23.8937	23.8514	23.8109	23.645	23.3832	23.5554	23.5089	23.4453
SYNJ2BP	18.7692	19.031	19.0916	19.0465	18.9098	19.3231	19.2381	19.0352	18.9167
SYNM	21.8105	21.0118	21.007	20.9224	20.7828	20.6295	21.0933	20.5158	20.8016
SYPL1	22.6552	22.4502	22.3348	22.3305	22.2405	21.9205	22.3102	21.9119	22.1455
TAB1	17.4646	17.4548	17.8361	17.1685	17.7636	17.911	18.3087	17.7348	17.8549
TACO1	19.4434	19.0366	19.3772	19.4014	19.1971	19.4586	19.7678	19.4153	19.3319
TAF15	20.1674	19.6881	20.2409	19.5272	19.868	20.4626	21.0361	20.4254	20.3526
TAF9B	18.2691	18.7714	18.57	18.5648	18.3491	18.321	18.5553	18.4377	18.5517
TAGLN2	23.6493	23.5603	23.6674	23.7384	23.316	23.5883	23.5934	23.6326	23.9193
TALDO1	24.0368	23.2519	23.8768	23.8246	23.7002	24.3074	24.4126	24.07	24.2048
TAOK1	18.2129	17.7746	17.9324	17.448	17.4371	17.5689	18.2754	17.7529	17.938
TAP1	19.0125	18.4827	18.4027	18.742	18.1893	19.1602	19.0181	19.5678	19.3376
TAP2	17.5515	17.6221	17.4549	17.3914	17.2697	16.532	17.2536	17.3797	17.5236
TAPBP	18.7229	14.6907	18.4946	18.4284	18.573	19.2479	19.0181	18.7558	19.1062
TARDBP	21.7202	21.6254	21.4962	21.5947	21.4464	21.9514	21.9018	21.511	21.7899
TARS	22.4866	22.1923	22.2654	22.231	22.0927	22.2287	22.4211	22.2986	22.3801
TARS2	22.1911	19.9917	20.3048	19.8905	20.0531	20.0555	20.3608	19.7028	19.9022
TBC1D15	19.4838	19.2315	19.32	19.1232	19.2469	19.0062	19.6082	19.2658	19.5184
TBC1D5	18.877	18.4234	18.8892	18.8494	18.9045	18.9524	19.3441	18.8856	18.8296
TBCA	19.3188	18.9477	19.11	19.506	19.188	19.6912	19.1645	18.9801	19.2761
TBCB	19.5239	19.1784	19.3994	19.5487	19.628	19.6281	19.5983	19.7123	19.7452
TBCD	19.8188	19.6116	19.8522	19.6628	19.4081	19.3919	19.8788	19.5978	19.7255
TBCE	17.9797	17.8213	17.9374	18.6904	18.306	17.1206	17.7893	17.8233	17.9466
TBK1	24.2239	24.4047	24.5831	24.2619	24.1581	23.9493	24.3495	24.1657	24.5583
TBL1XR1	20.1363	20.0725	20.0759	20.0565	19.7714	20.0161	20.0982	20.1035	20.149
TBL2	18.9401	18.6669	18.7336	18.6936	18.7918	18.8454	18.9873	19.0346	18.9836
TBL3	19.993	19.8152	19.6906	19.3667	19.6216	19.3803	19.6939	19.7477	19.5838
TBP	16.3725	17.0586	16.6153	16.9232	16.9576	16.729	16.8632	16.4975	16.5317
TBRG4	20.0579	19.5206	19.2423	19.1367	19.3797	19.4339	19.5676	19.3151	19.3942
TCEA1	21.3216	20.9313	21.2401	21.2372	21.0474	21.2893	21.2904	20.9683	21.2825
TCEB1	21.0605	19.1925	20.9978	21.5473	21.2607	21.4223	21.2132	21.0211	21.3022
TCEB2	20.8312	19.7948	20.7225	20.7171	20.6195	20.9952	21.1956	21.0526	21.1493
TCERG1	20.8906	20.6813	20.7654	20.4866	20.5377	20.8864	21.2502	20.7263	20.9133
TCOF1	19.4231	19.3449	19.1165	19.4388	19.0751	19.6796	19.6727	19.4129	19.5844

TCP1	23.8546	23.7604	23.6963	23.5376	23.3899	23.5166	23.8821	23.5747	23.6354
TECR	20.9752	20.4305	20.8658	20.4885	20.7364	20.3445	21.2365	20.7608	20.8794
TELO2	17.5795	17.5677	17.8651	17.4716	17.8948	17.419	17.8356	17.6483	17.4732
TEX10	17.7943	17.8918	17.8445	17.5754	17.6271	16.729	17.9706	17.5893	17.4576
TEX264	18.2871	16.1907	18.4035	18.4896	18.0534	18.5454	18.8419	18.4296	18.7909
TFAP2A	17.4238	14.9517	17.3078	16.5326	17.4347	16.0767	16.5343	16.1336	14.9515
TFB1M	15.4911	16.3758	16.6085	16.7983	16.4422	16.2159	16.8742	16.3048	16.1713
TFB2M	19.3756	19.3115	19.6645	19.247	19.191	19.6941	19.5052	18.9645	19.0075
TFCP2	19.034	18.7263	19.0336	18.9685	18.8112	18.8651	18.9473	19.0922	18.9303
TFG	21.0017	20.5413	20.9524	21.2482	21.037	20.9867	21.5174	20.857	21.2114
TFRC	23.0409	23.0439	22.9534	22.9129	22.7264	23.1207	23.4362	23.1846	23.2804
TGM2	21.139	20.8833	21.0875	21.0062	20.9102	20.1876	20.4566	20.2797	20.2998
TGOLN2	17.5664	17.6328	17.2075	17.1693	17.1149	17.0193	17.0803	17.0588	17.3679
THBS2	18.5668	19.097	18.5832	18.5144	18.6315	19.0888	19.215	18.8066	19.0756
THEM4	18.7415	18.6228	18.7953	18.7052	18.6441	18.7027	18.7394	18.5354	18.2585
THEM6	20.0232	19.7233	19.557	19.1865	19.2057	19.6696	19.7281	19.5167	19.9153
THOC1	18.0417	18.3607	18.1412	18.1812	18.1507	18.3233	18.7563	18.5831	18.385
THOC2	18.0326	18.2428	17.9573	17.8226	18.149	17.6113	18.282	18.0368	17.8696
THOC3	18.0057	18.5201	18.3375	18.3984	17.758	17.9574	17.4705	18.0411	17.8621
THOC5	20.3097	19.5768	18.9782	19.0263	18.8914	19.2526	19.0943	19.3253	19.5623
THOC6	18.6264	18.2441	18.2418	18.0972	17.9943	18.3204	18.4253	18.0675	17.8251
THOP1	22.4869	22.6425	22.8337	22.3857	22.3581	22.4439	22.6389	22.4869	22.6973
THRAP3	21.0943	20.4995	21.2416	21.2817	21.1891	21.3889	21.1416	21.0895	21.4994
THUMPD1	18.6773	18.5171	18.5265	18.5128	18.4974	18.454	18.6144	18.4939	18.6124
THUMPD3	19.303	19.2716	19.3877	19.1661	19.1261	19.1388	19.7089	19.2712	19.1718
THYN1	17.9561	18.0939	18.2702	18.0811	17.8496	18.2628	18.6239	18.2781	18.3885
TIA1	16.3843	15.4337	16.8505	11.7288	15.3752	16.2662	17.7517	16.5515	16.0946
TIAL1	21.1054	20.1758	20.9866	20.9035	20.9804	21.3249	21.5617	20.9416	21.0544
TIGAR	20.4854	20.4036	20.0705	20.3534	20.1098	20.0231	20.1319	20.257	20.3738
TIMM44	21.2459	21.0492	20.9364	20.8054	20.7544	20.7684	20.9067	20.8956	21.0075
TIMM50	20.8656	20.4652	20.3745	20.5235	20.4697	20.8338	20.857	20.8601	20.6086
TIMMDC1	16.4731	16.7699	16.5318	16.7389	16.9694	15.9386	15.2187	16.595	16.259
TIMP3	20.465	20.0643	20.0254	19.9158	19.7506	19.8978	20.2402	20.1811	19.9898
TIPRL	20.534	20.4817	20.3457	20.419	20.26	20.3659	20.4731	20.2139	20.2869
TJP1	19.4806	19.2356	19.2596	19.2649	19.3726	18.6459	19.7006	19.3832	19.6342
TJP2	19.5536	19.5874	19.5383	19.3009	19.1052	19.1913	19.2549	19.3145	19.4222
TK1	20.1495	19.8328	19.8382	19.9218	19.8619	19.6794	19.7405	19.9	19.662
TKT	25.7838	25.6407	25.5826	25.5389	25.3107	25.7856	26.0546	25.7632	26.1088
TLE3	19.1899	18.8625	19.0089	19.0918	18.9771	18.9962	19.3798	19.1091	19.3738
TLN1	21.5427	21.713	21.6761	21.6844	21.5911	21.1174	21.7653	21.5486	21.6976
TM9SF1	19.4894	19.3109	19.3854	19.211	19.0798	19.1433	19.425	19.3909	19.5769
TM9SF2	21.0538	20.7182	20.8917	20.9995	20.9354	20.7599	20.8895	20.9614	20.9762
TM9SF3	21.2414	20.9661	21.0411	21.1113	21.1308	20.8471	21.3242	20.9765	21.2322
TM9SF4	21.7784	21.422	21.1841	21.565	21.1928	21.0635	20.9834	21.2431	21.3135
TMCO1	20.758	21.2337	20.538	20.6434	20.4146	20.6227	20.7543	20.6374	20.4939
TMED10	21.9173	22.0427	22.0551	22.0417	22.0062	22.3341	22.2398	22.1264	22.2724
TMED2	20.3568	18.805	20.2201	20.4203	20.1022	20.569	20.5117	20.4122	20.8403
TMED7	21.9326	21.3962	21.3296	21.5378	21.1895	21.3242	21.5083	21.4696	21.6625
TMED9	20.775	20.5007	20.5523	21.0737	20.5168	20.9905	21.0676	21.0332	21.0999
TMEM14C	18.4096	18.4568	18.0414	18.3176	18.1672	16.7682	16.644	18.0138	17.862
TMEM165	20.3822	19.9937	19.7216	20.2091	19.8294	20.0426	19.5817	20.0688	20.1565
TMEM167A	20.8264	21.131	21.3728	21.0215	20.7676	20.7728	21.1484	20.7739	20.9217
TMEM189	19.093	19.1063	19.2526	18.9441	19.3803	19.2697	19.6264	19.5662	19.1964
TMEM2	21.0521	21.277	21.2216	21.1205	21.2935	21.2154	21.5919	21.038	21.2119
TMEM223	14.1673	?	14.485	15.3678	15.9034	15.4639	16.0756	15.1161	15.9549
TMEM33	20.6373	20.4598	20.9314	20.9277	20.6155	20.3709	20.7537	20.5533	20.5361

TMEM43	21.5237	21.53	21.4057	21.5884	21.4109	21.3501	21.5642	21.2648	21.4051
TMEM87A	17.566	17.829	17.8113	17.4879	17.994	14.6528	15.0826	15.7645	14.7015
TMEM97	18.4381	18.8306	18.6079	18.3654	18.6697	18.904	19.5147	18.7703	19.0332
TMOD3	20.8818	20.7719	20.742	20.6782	20.7	20.9036	21.3285	20.7897	20.9395
TMPO	21.9721	21.8233	21.9038	22.0551	21.8517	22.0132	22.4452	21.8017	22.0783
TMPO	21.7869	22.0363	21.9314	21.8608	21.508	21.7254	21.7117	21.7807	22.0154
TMX1	21.4703	20.9065	21.5496	21.5999	21.2777	21.3725	21.5151	21.1356	21.32
TMX2	18.2755	18.037	18.3813	18.3979	18.0518	18.2333	18.2165	18.1499	18.1689
TMX3	19.0807	18.9288	18.8143	18.7971	18.9125	18.3736	18.1631	19.0531	19.1239
TMX4	18.9956	19.3102	19.2677	19.0032	19.0203	18.8387	18.8318	18.9671	19.0722
TNC	19.0605	19.0553	18.5056	18.6381	18.8703	19.0189	19.8692	19.4455	19.7457
TNFAIP8	15.8739	16.3464	16.1741	16.2416	16.3702	15.2086	15.5675	16.2222	16.0872
TNKS1BP1	15.6486	15.7648	16.6725	16.6433	16.3646	17.7042	17.7361	17.002	17.0042
TNPO1	22.741	22.6569	22.6744	22.5136	22.5725	22.4883	22.9691	22.4161	22.5525
TNPO2	19.1112	19.1505	19.1834	19.2648	18.7376	18.8848	18.3186	18.7424	19.0466
TNPO3	20.3398	20.8032	20.5705	20.4455	20.6322	20.4845	20.8878	20.6666	20.6562
TNS3	20.5039	19.9535	20.518	20.7931	20.6959	20.9199	20.9438	20.6378	20.9709
TOE1	18.7868	19.2295	19.5608	19.7108	19.4084	19.5014	19.4512	18.7196	19.3049
TOMM20	20.3888	20.515	20.5916	20.2741	20.5052	20.611	20.5514	20.3284	20.2532
TOMM22	21.0699	20.6179	21.1604	20.819	20.9002	20.8252	21.0952	20.8892	21.1452
TOMM34	22.2569	21.9824	22.0432	21.8125	21.6012	21.9484	22.2224	21.8158	21.7895
TOMM40	22.0716	21.7729	21.5786	21.7463	21.5836	21.4865	21.7425	21.724	21.892
TOMM70A	21.4409	20.3685	20.989	21.1847	20.8711	21.147	21.238	20.944	21.2819
TOP1	19.0094	19.4473	19.505	19.0127	18.9423	19.2253	19.721	19.3536	19.2806
TOP2A	19.3043	19.6451	19.4429	19.4501	19.2129	19.294	19.4955	19.5255	19.944
TOP2B	18.2442	18.5403	18.4682	18.3678	18.331	17.7688	18.3006	18.3244	18.2567
TOR1A	18.4921	18.2199	17.9321	17.575	17.9293	16.9711	17.6664	17.8239	17.2087
TOR1AIP1	21.8002	21.8356	21.6022	21.6717	21.5799	21.6046	21.6244	21.8265	22.0096
TOR1B	17.9762	17.1045	17.5064	17.7631	17.4412	16.6684	16.9785	16.2383	16.5981
TP53BP1	19.1729	18.844	18.6166	18.7877	18.831	19.1921	19.3018	18.9967	19.2365
TP53RK	18.3307	17.9898	17.9211	17.7726	17.9618	18.1333	17.9598	17.979	17.854
TPBG	17.5335	17.9991	17.9695	17.7959	17.7245	17.3696	17.7669	17.6034	17.8224
TPD52	19.9393	19.7851	19.5411	19.7809	19.5886	18.9091	18.6712	19.3879	19.5068
TPD52L2	22.3882	22.2143	22.1238	22.1744	21.9025	21.9186	22.0439	21.9269	22.1508
TPI1	25.7364	25.3242	25.4789	25.5532	25.321	25.6377	25.5976	25.7187	25.6415
TPM1	19.886	19.5201	19.7841	20.2139	19.9197	19.5125	19.542	19.273	19.6455
TPM3	22.1206	21.4355	22.1589	22.5648	22.2064	22.1066	21.8688	21.8756	22.1704
TPM4	19.91	19.1276	19.7115	19.8944	19.4969	19.8812	19.9818	19.5903	20.1397
TPP1	21.1215	21.4991	21.5476	21.4149	21.2128	21.3086	21.7792	21.407	21.5432
TPP2	18.7846	19.6348	19.547	20.1335	19.4796	19.9986	20.0195	19.7961	20.2669
TPR	22.0789	21.5708	21.6307	21.809	21.608	21.9976	22.0491	21.7317	21.8688
TPRKB	18.1382	18.2636	17.9989	17.9482	17.7138	18.0426	18.3057	17.9651	18.0157
TPT1	23.6329	23.3773	23.4279	23.6322	23.2834	23.5056	23.2634	23.2788	23.3726
TRA2A	20.6848	20.8254	20.6922	20.8337	20.7976	20.665	20.9542	20.7679	20.8478
TRA2B	22.3508	22.0093	22.277	22.4117	22.4771	22.3798	22.2614	22.3101	22.2687
TRAP1	22.2522	22.2099	22.1698	22.0748	21.9748	22.1006	22.5547	22.233	22.5127
TRAPPC3	19.9055	20.1238	20.4289	19.9158	20.2515	20.7839	20.0585	20.0701	19.9352
TRIM24	17.9272	17.785	17.8714	17.31	17.4453	17.681	18.0485	17.4707	17.7297
TRIM25	21.5676	21.4386	21.4175	21.3268	21.4173	21.2929	21.5092	21.2893	21.4108
TRIM26	15.7332	16.2069	16.4205	16.0282	16.3839	16.666	16.6237	16.5383	16.6124
TRIM28	24.0608	23.6478	23.5866	23.7352	23.4282	23.4407	23.5028	23.6998	23.9538
TRIM47	19.1903	18.4461	19.1607	19.3046	18.9099	19.8209	20.0119	19.5533	19.7957
TRIM56	16.572	16.7754	17.2817	17.144	16.8767	16.9939	17.3302	17.0418	17.3934
TRIO	16.6736	16.5149	16.679	16.1789	16.7379	16.6929	17.308	17.117	16.77
TRIOBP	18.1834	17.3343	18.4561	18.5902	18.4611	18.339	18.7272	18.1952	18.4308
TRIP10	16.6768	17.9914	19.267	17.7521	19.4751	16.4196	17.4255	17.4226	18.2753

TRIP12	18.8926	18.841	18.6871	18.6334	18.5796	18.8185	19.2059	18.6036	18.8762
TRIP13	20.8827	20.6955	20.9106	20.8947	20.8789	20.9928	21.2453	20.7677	20.9548
TRIP6	20.8532	20.7098	20.8693	20.5419	20.5627	20.5205	20.6303	20.5824	20.7859
TRMT1	17.3746	17.6227	17.7004	17.5954	17.5829	17.2364	17.4518	17.6154	17.4979
TRMT10C	19.2064	19.1955	19.2667	19.0067	19.0622	19.3487	19.6126	19.4329	19.2915
TRMT112	20.9822	20.9139	20.9345	20.9661	20.7942	21.0048	21.223	20.8535	21.064
TRMT1L	15.9105	15.6365	15.5901	15.6551	15.5597	14.9249	15.143	16.1022	15.5304
TRMT6	17.7114	17.8821	17.6761	17.5336	17.5535	18.5206	18.2454	17.5707	17.5799
TRNT1	19.2117	18.8733	19.1737	19.0282	18.8274	19.1565	19.4436	19.1163	19.4096
TROVE2	20.1293	19.7289	20.0056	20.2201	20.159	20.0369	19.807	20.0967	20.04
TRRAP	17.0359	17.178	17.3488	17.0881	17.1368	17.0143	17.7299	17.4885	17.689
TSFM	20.2306	20.146	19.8344	19.9973	19.8389	20.038	20.1372	20.0148	20.253
TSG101	20.0031	20.0346	20.0645	19.7835	19.8542	19.6811	20.0938	19.8422	20.0197
TSN	21.7945	21.6419	21.5389	21.2712	21.4252	21.5071	21.7992	21.4246	21.4265
TSNAX	19.0646	19.2343	18.8631	18.596	18.9645	17.9236	18.389	18.7705	18.6328
TSR1	20.204	19.9296	20.1095	20.06	19.8295	19.6819	20.055	19.7903	19.9665
TSSC4	16.8065	17.3196	17.3709	17.7489	17.3332	17.3086	17.9598	17.2177	17.9342
TSTA3	17.1251	16.6918	16.7379	16.7768	16.5985	16.3578	15.4746	16.4269	16.3748
TTC1	17.5014	17.3708	17.4498	17.6868	17.2619	17.9252	17.7663	17.4031	18.0265
TTC37	17.7994	17.6184	18.1582	17.8017	17.7376	17.6687	18.0718	18.0389	17.8285
TTC4	19.7869	19.7011	20.1193	19.6862	19.7534	19.7886	19.8365	19.8293	19.8164
TTI1	19.9563	20.8602	20.1603	20.8746	20.2545	19.9966	20.4988	20.2704	20.6322
TTI2	16.7502	17.7967	17.861	17.8643	17.4845	17.5301	17.3684	17.7029	17.2216
TTLL12	21.8079	21.4749	21.3889	21.3187	21.2442	21.3821	21.1873	21.3541	21.3574
TUBA1B	25.2463	24.9523	24.8708	25.0274	24.5336	24.8832	24.8092	25.273	25.1265
TUBA1C	23.6011	23.196	23.2881	23.4428	23.0527	23.4686	22.5696	23.4105	23.1301
TUBA3C	21.0532	21.1779	21.383	20.8348	20.9636	19.5643	20.4854	20.5811	20.3671
TUBB	26.2944	25.9179	25.9113	25.9273	25.9424	26.0866	26.3053	26.124	25.8371
TUBB2B	22.2662	20.9745	22.0284	22.0071	21.8281	21.9621	21.7301	21.8605	21.8555
TUBB3	22.5606	22.6383	22.4347	22.4387	22.2403	22.538	22.3264	22.1105	22.7224
TUBB6	21.2994	21.1725	21.1195	21.2298	20.9989	21.3783	20.6574	21.0756	21.6372
TUBG1	20.7057	20.507	20.5336	20.4149	20.3253	20.5695	20.6046	20.562	20.3157
TUBGCP2	18.1832	18.0693	17.9081	18.0946	17.987	17.9581	18.3567	18.1114	18.1774
TUBGCP3	18.643	19.0637	18.9361	18.5957	19.4293	18.7877	20.1969	19.4403	19.1415
TUFM	23.6672	23.7315	23.757	23.5946	23.4188	23.4932	23.7102	23.4252	23.7115
TWF1	20.3274	20.7403	20.5485	20.3406	20.6051	20.6559	20.7926	20.442	20.1119
TWF2	21.5604	21.265	21.3541	21.4144	21.3913	21.4363	21.236	21.3263	21.3857
TXLNA	21.1906	20.8361	21.0498	21.2921	21.3269	21.1314	21.258	21.0939	21.179
TXLNG	17.3652	17.8326	18.0634	17.9286	17.991	17.9149	17.6557	17.3215	18.2099
TXN	23.3049	23.4861	23.1899	23.7308	23.0845	23.4226	22.6165	23.5344	23.6652
TXNDC17	21.4041	21.5758	21.7939	21.7636	21.6007	21.8706	21.8954	21.5544	21.6918
TXNDC5	22.5634	22.7296	22.6228	22.6114	22.5995	22.7811	22.7134	22.723	22.8301
TXNDC9	19.0518	18.8594	19.112	19.0963	19.078	18.8281	18.987	18.8941	19.1363
TXNL1	22.75	22.7067	22.6686	22.6968	22.457	22.7729	22.9045	22.7323	23.0102
TXNRD1	22.3826	21.977	22.0652	22.1602	22.1988	22.5424	22.5976	22.3806	22.3091
TYMS	20.707	20.8547	20.8462	20.6743	20.6432	20.6131	20.8451	20.6331	20.3991
U2AF1	21.8344	20.6414	21.642	21.8111	21.7683	21.9455	21.9089	21.5562	21.5216
U2AF2	23.305	23.2101	23.151	23.21	23.0597	22.9803	23.3531	23.1279	23.3755
U2SURP	20.9538	21.5528	21.1234	20.9913	21.0238	20.9716	21.1886	20.9377	20.9907
UACA	16.6093	17.1103	17.2905	16.9869	16.827	16.9261	17.3194	17.0787	16.9834
UAP1	21.7732	21.598	21.658	21.5035	21.1443	21.0752	20.9676	21.1997	21.4417
UAP1L1	18.4826	18.2612	18.4401	18.669	18.3192	18.245	18.6392	18.4125	18.3536
UBA1	23.8981	23.8636	24.139	23.7914	23.7283	23.895	24.4658	24.0716	24.1506
UBA2	21.3676	21.618	21.5078	21.3419	21.3313	21.135	21.6282	21.2421	21.3474
UBA3	20.7933	20.669	20.6325	20.5203	20.3489	20.4713	20.4806	20.4827	20.7089
UBA5	17.4706	16.4338	16.9499	17.0095	17.0251	17.4357	17.6554	17.0941	17.503

UBA6	19.7678	19.6489	19.9265	19.7157	19.6361	19.5265	20.0374	19.7312	19.7084
UBAP2L	20.8206	20.3044	20.7936	20.8104	20.7849	20.8138	20.8561	20.3864	20.7595
UBASH3B	18.9365	18.9783	19.1433	19.1507	19.0055	18.8745	19.0526	19.1419	19.1004
UBE2C	20.8669	20.5676	20.516	20.5036	20.2669	20.3401	20.4134	20.3842	20.3071
UBE2D3	22.1134	22.1275	22.3629	22.0833	21.869	22.241	22.4753	22.2165	22.4229
UBE2H	19.6545	19.6832	19.7077	19.7691	19.6091	19.967	19.931	19.739	19.7793
UBE2I	21.2855	21	21.1259	21.1838	20.9587	21.4008	21.4977	21.2194	21.2993
UBE2K	22.0189	21.8157	21.9515	21.8143	21.6751	21.8543	22.0813	21.812	22.0651
UBE2L3	23.0968	22.8718	22.926	23.108	22.9094	23.1599	23.2357	22.8959	22.9932
UBE2M	22.2303	22.287	22.2587	22.2005	21.9305	22.1747	22.462	22.1638	22.2689
UBE2N	23.3058	23.1941	23.2161	23.1735	22.9967	23.3246	23.3125	23.1857	23.2007
UBE2O	19.937	19.4364	20.161	20.1956	19.9141	20.3334	20.5165	20.0087	20.2758
UBE2S	18.8928	18.6758	18.5317	18.8161	18.4559	18.7685	18.7378	18.3759	18.1131
UBE2T	20.9287	20.9475	20.8277	20.802	20.489	20.553	20.3527	20.6703	20.9031
UBE2V2	22.8161	22.8152	23.2474	23.1207	23.0337	23.2166	23.1065	23.0248	23.2228
UBE3A	17.6988	17.6503	17.5835	17.3777	17.6534	17.4447	16.9924	17.345	16.7777
UBE4A	18.4562	18.701	18.3751	18.2088	18.0226	17.8116	18.2095	17.9905	18.2741
UBFD1	18.5892	18.745	18.8304	19.0452	18.631	18.3087	17.8772	18.0914	18.2741
UBLCP1	18.8048	18.9049	19.083	18.7783	18.5379	18.0969	18.3037	18.5128	18.9308
UBQLN4	18.3947	18.2807	18.3964	18.4394	18.0849	18.4351	18.6963	18.0181	18.5565
UBR1	17.0315	17.401	17.228	17.061	17.2988	16.358	16.7211	16.6908	16.4071
UBR4	20.0872	19.8774	19.9288	19.9	19.8753	20.0938	20.3179	20.1066	20.2095
UBR5	16.5753	16.4865	16.5914	16.4496	16.6682	16.749	16.6782	16.7938	16.442
UBR7	20.822	20.6611	19.0697	19.2493	20.6526	20.2598	19.5406	18.99	19.0319
UBTF	19.8318	19.8307	19.9931	19.8881	19.9102	19.8679	19.7574	19.7903	19.717
UBXN1	19.7367	19.465	19.2055	19.915	19.4239	19.6009	19.3199	19.1373	19.6093
UCHL1	22.4168	22.2206	22.3329	22.6878	22.3297	22.7583	22.5684	22.4737	22.8346
UCHL3	18.0903	18.0885	18.2887	18.2825	17.712	18.4691	18.0417	18.1237	18.6777
UCHL5	23.0088	22.974	22.7099	22.3832	22.4449	22.1555	22.3831	22.4189	22.7694
UCK2	20.5185	19.9746	19.8783	20.1028	19.8496	20.2616	20.2992	20.3185	20.0687
UFC1	19.5294	19.4026	19.5218	19.2958	19.3612	19.5175	19.6931	19.1438	19.3183
UFD1L	21.6562	21.5041	21.5425	21.4638	21.2333	21.5843	21.6612	21.55	21.7931
UFL1	16.7	15.9241	15.577	15.7816	16.9742	14.8529	15.0455	16.754	15.0357
UGDH	20.9965	20.0138	20.481	20.3419	20.4389	20.9161	21.2308	20.9708	20.9209
UGGT1	21.7093	21.9991	21.4557	21.7277	21.5342	21.391	21.6893	21.4469	21.7752
UGP2	22.9946	23.1493	22.9687	23.0223	22.7301	23.2842	23.6874	23.3763	23.548
UGT8	18.1809	18.2598	18.264	17.9801	18.1791	17.8398	18.0869	18.2369	18.048
UHRF1	20.7626	20.3581	20.2701	20.2555	20.2314	20.023	20.3772	20.2937	20.0705
UMPS	20.7801	20.4198	20.5195	20.3127	20.3833	20.3774	20.8481	20.5475	20.4746
UNC119B	18.2603	17.7811	18.0237	18.0889	17.7173	18.0059	18.4196	18.1349	17.8129
UNC45A	20.3062	20.2469	20.4678	20.1009	20.1069	20.1308	20.5636	20.2138	20.3303
UNG	20.2752	20.2854	20.117	20.149	20.0393	20.2238	19.8336	19.6443	20.1178
UPF1	21.8083	21.3438	21.5335	21.4436	21.1775	21.5743	22.3094	21.7116	21.6061
UPP1	20.2586	19.8512	20.1016	20.3481	20.1212	20.7684	20.0223	20.2424	20.4677
UQCRB	20.3515	20.2821	20.3293	19.9755	20.0791	20.173	20.1786	20.2728	20.3991
UQCRC1	22.651	22.458	22.461	22.3443	22.4673	22.7525	22.9413	22.6568	22.6481
UQCRC2	22.0649	21.975	22.0208	21.8292	21.9126	22.0413	22.5358	22.1276	22.3493
UQCRFS1	20.0818	19.9758	19.7808	20.3246	20.0148	20.0718	19.7826	19.9007	20.201
UQCRQ	20.1039	19.0688	20.0337	19.862	19.8533	20.2826	20.5312	20.1743	20.2244
URB1	17.4524	17.757	17.737	17.5633	17.5678	17.2515	17.4271	17.5456	17.4191
URB2	18.4108	18.3768	18.3234	18.4455	17.838	17.835	18.16	18.5011	18.4545
UROD	20.2499	19.2115	20.0886	20.0959	19.8903	20.4546	20.558	20.1962	20.3406
USO1	20.4195	20.4167	20.5242	20.3453	20.2876	20.3703	20.9707	20.4956	20.5941
USP10	20.409	20.2418	20.2085	20.0371	20.0178	20.1758	20.8688	20.281	20.6173
USP11	19.1623	19.0562	18.9284	18.98	18.9185	18.4611	18.5039	18.8461	18.7594
USP14	20.6388	20.0601	20.3844	20.4396	20.2499	19.6568	19.6561	19.7779	19.8818

USP15	18.7856	18.5691	18.4709	18.1207	18.374	18.5416	19.3904	18.6514	18.535
USP19	20.7025	20.2982	20.218	20.4504	20.3469	20.4948	20.3747	20.5339	20.6028
USP39	19.1319	19.211	19.6278	19.5505	19.2206	19.0454	19.2954	19.245	19.4515
USP4	17.612	17.3401	17.3901	17.4173	17.1122	17.1382	16.4488	17.1133	16.9808
USP48	19.4652	18.7666	19.9033	18.9362	19.8144	17.8007	19.1296	19.9381	20.1419
USP5	21.2662	21.2871	21.1056	21.202	20.9387	21.3482	21.6855	21.2511	21.5584
USP7	20.3326	20.0616	20.0512	20.0149	20.031	20.2544	20.704	20.1659	20.2901
USP8	16.7458	17.4519	17.625	17.7059	17.3377	17.3524	17.346	17.4828	17.2353
USP9X	18.2102	18.3252	18.3573	17.86	18.0899	18.0346	18.5069	18.1173	18.4879
UTP14A	18.586	18.5431	18.3055	17.8946	18.2225	17.8352	18.4384	18.3757	18.7462
UTP15	19.2118	18.95	19.0933	18.8221	19.0156	18.7882	19.4568	19.0159	19.0757
UTP18	19.8205	23.0259	19.755	19.727	19.7716	19.9607	19.892	19.5679	19.6096
UTP20	17.3362	17.5854	17.3749	17.1184	17.1814	16.6737	17.1255	17.1619	16.9427
UTP3	17.0611	16.0606	16.3568	16.2416	17.0553	16.389	14.2902	16.1541	15.8463
UTP6	18.067	18.3622	17.2182	18.2309	18.1333	17.0415	17.6669	17.7815	17.7366
VAC14	16.9374	16.8735	16.9649	18.2994	17.079	18.3295	16.5837	16.6656	16.6299
VAMP3	18.6754	18.3576	18.1796	18.1764	17.8999	18.0598	16.8532	18.2137	17.9601
VAMP7	19.5617	19.5241	19.6887	19.3686	19.6681	19.65	19.889	19.415	19.4184
VAPA	20.98	20.7326	20.7145	20.7427	20.1743	20.6034	20.6905	20.6823	20.5509
VAPB	18.9506	19.158	19.5634	19.2552	19.6704	20.1767	20.658	19.7125	19.8152
VARS	22.0488	21.6554	21.4658	21.5833	21.4114	21.2739	21.5756	21.3927	21.6579
VASP	20.3224	20.1236	19.7642	20.046	19.6377	20.0079	19.7148	19.8104	19.9464
VAT1	20.7205	20.7945	20.9643	20.6883	20.6647	21.0136	21.2568	20.8152	21.1259
VBP1	20.8749	20.4456	20.8415	20.5414	20.6623	21.0906	21.0539	20.854	21.1202
VCL	20.9458	20.7943	20.8581	21.0326	20.7886	21.0874	21.3831	21.2333	21.3905
VCP	24.2158	24.185	24.0134	24.004	23.9099	23.8128	24.3408	24.0271	24.3074
VDAC1	24.8531	24.771	24.6963	24.6253	24.6112	24.8091	25.0059	24.7092	24.9513
VDAC2	23.7761	23.4687	23.6061	23.5817	23.4481	23.5037	23.7942	23.4786	23.669
VDAC3	23.2691	22.9473	22.9878	22.8314	22.7414	22.7134	23.1515	22.8733	23.0137
VIM	28.2667	28.2607	28.2699	28.0771	28.0242	28.0359	28.094	28.0866	28.2869
VKORC1	20.8203	21.3159	21.0574	21.2239	21.001	20.8756	21.1699	20.8021	21.2232
VKORC1L1	16.2905	17.7805	17.3814	17.6761	17.4272	17.7831	17.8053	17.529	17.5344
VPRBP	18.0477	17.696	17.7635	17.8806	17.4699	17.4251	17.7062	18.1413	17.8495
VPS11	18.5669	16.6866	16.3199	16.8107	16.4919	16.7908	16.6685	16.7777	16.5212
VPS18	18.1434	17.929	17.6502	17.874	17.691	17.8511	18.0961	17.8628	17.9719
VPS25	17.0102	17.1544	17.6514	17.5103	17.3748	16.629	17.467	17.6175	16.6214
VPS26A	20.0795	20.1157	20.1478	19.9788	19.9863	20.0641	20.278	20.174	20.4018
VPS29	20.2425	20.4443	20.2272	20.3195	20.1438	20.1821	20.4655	20.1463	20.6072
VPS33A	17.2131	17.3106	17.4641	17.2349	16.9995	17.0538	17.6131	17.0406	16.8763
VPS35	21.1978	21.226	21.0244	21.0427	21.0411	20.9093	21.3153	21.1183	21.3521
VPS37B	18.8678	18.6719	18.0253	18.663	18.2927	18.7658	18.0848	18.2849	18.3633
VPS45	17.9569	18.7234	17.9441	17.5675	17.9527	17.9264	18.2683	17.8705	17.5173
VPS4A	19.0352	19.1595	19.2332	18.7304	18.7344	19.0671	19.3121	18.7729	19.0466
VPS4B	20.6274	20.3791	20.4623	20.4116	20.1683	20.7958	20.5453	20.4898	20.3789
VPS51	17.2105	17.1949	17.0608	17.1089	16.8972	16.0372	16.9202	16.7301	16.601
VPS53	16.4198	17.0546	17.3476	17.0543	17.2167	17.9105	17.6433	17.2731	16.6863
VRK1	18.883	18.8831	18.8685	18.9293	18.6913	18.8969	18.494	18.7607	18.8472
VWA9	17.7875	17.4418	17.9928	17.6675	17.6558	17.8127	18.6861	18.0535	17.7023
WAPAL	17.586	17.088	17.3993	17.747	17.6771	16.6095	17.1867	17.5054	17.111
WARS	22.22	21.953	21.7455	21.7066	21.6778	22.3646	22.1672	22.1738	22.0697
WASF2	18.3003	18.8432	18.2427	19.2158	18.4444	17.4672	17.2949	18.0959	18.0172
WASL	16.3493	17.4115	17.5572	17.4111	17.0333	17.0665	17.022	16.7792	17.0376
WBP11	20.4356	20.1258	20.2253	20.2137	20.0082	19.8003	20.1482	20.1238	20.018
WBSCR16	19.0401	18.9883	18.9346	18.8744	18.8183	18.9637	19.3177	18.7835	19.181
WBSCR22	18.9863	18.7529	18.5868	18.8025	18.6711	18.5992	18.3279	18.4693	18.8447
WDHD1	19.1218	19.172	19.366	19.2447	18.9621	19.0503	19.6765	19.3019	19.6073

WDR1	23.4884	23.173	23.2844	23.282	23.0479	23.0265	23.313	23.1004	23.1303
WDR11	18.3335	17.934	17.9656	18.0282	18.195	17.5184	18.1495	17.9264	18.0146
WDR12	20.2357	20.2235	20.0775	20.0695	19.7994	19.7237	20.1401	19.6812	19.9818
WDR18	18.915	17.9638	18.6575	19.0987	19.0153	19.289	18.8628	18.8169	19.0393
WDR26	17.634	18.0103	17.3242	17.8069	17.8124	17.5924	17.656	17.3817	17.552
WDR3	19.3675	19.2144	19.4613	19.0671	19.279	18.7752	19.2206	19.1603	18.8076
WDR36	19.4172	19.8259	19.974	19.567	19.7634	19.6746	20.4283	19.7572	19.5937
WDR43	20.6443	20.2593	20.6577	20.4571	20.259	20.3132	20.883	20.6409	20.6905
WDR46	17.804	17.8321	16.6013	16.8205	15.651	17.6377	16.898	17.4817	17.7044
WDR5	19.5972	19.3948	19.4954	19.4423	19.2955	19.1126	19.3513	19.3844	19.3403
WDR55	17.1076	17.1506	18.0016	17.6608	17.4073	17.3062	16.9997	16.9552	17.3252
WDR6	18.8617	18.4212	18.5064	18.2859	18.099	18.1852	18.8199	18.6852	18.3722
WDR61	20.9345	20.5506	20.4157	20.4627	20.4665	20.544	20.5741	20.5062	20.477
WDR70	18.0931	18.2373	18.0424	18.0734	17.7199	17.7157	18.2331	17.9405	18.0319
WDR73	16.0182	16.3325	16.0909	15.3036	15.8052	15.9052	16.0915	15.3107	15.8816
WDR74	19.2996	19.1603	19.0371	19.5627	19.1162	19.4407	19.0597	19.2081	19.0319
WDR75	19.5831	19.7436	19.8015	19.6775	19.5622	19.4096	20.079	19.8598	19.9085
WDR77	22.0554	21.3595	21.4556	21.4363	21.2276	21.1427	21.4913	21.3119	21.6415
WDR82	19.5086	18.8128	19.634	19.4398	19.4931	19.8146	20.3608	19.5753	19.3807
WFS1	17.5738	17.4617	17.3647	17.2269	17.0092	16.4442	17.0195	16.9044	17.1886
WNK1	18.8753	18.2075	18.5526	18.374	18.1045	18.0486	17.8396	18.2527	18.358
WRNIP1	16.6125	17.5639	17.4524	16.6533	17.4282	17.2272	17.4852	17.2858	17.1848
WTAP	17.0287	17.5327	17.6449	17.5957	17.635	17.6736	16.9085	17.3375	17.7319
XAB2	18.9599	19.3572	19.0403	19.1947	19.0946	18.774	19.2708	19.323	19.4629
XPNPEP1	19.5989	19.8001	19.8288	19.8214	19.5316	19.9227	19.6626	19.7819	19.8068
XPNPEP3	17.917	18.0732	17.9819	18.1385	17.8259	17.4124	17.9105	17.9101	18.0445
XPO1	22.727	22.6645	22.7684	22.7228	22.6331	22.423	22.6294	22.7014	22.5339
XPO5	20.4129	20.2304	20.3241	20.1409	20.0469	19.9956	20.5709	20.2939	20.3199
XPO7	20.3884	20.2138	19.9506	20.213	19.8643	19.7479	20.2203	20.0366	19.9909
XPOT	20.2734	20.4772	20.3531	20.2836	20.3118	19.9093	20.6704	20.3185	20.4967
XRCC1	20.362	20.0798	20.0649	20.0033	19.9005	20.0323	20.4305	20.0652	20.1878
XRCC4	17.7163	16.0803	16.9776	16.8709	17.3004	17.3809	16.8599	17.2388	16.5499
XRCC5	24.4728	24.2378	24.5728	24.6673	24.3525	24.2658	24.7595	24.0714	24.119
XRCC6	24.5387	24.5776	24.4915	24.5184	24.3575	24.5112	24.562	24.4404	24.5803
XRN2	21.696	21.8603	21.805	21.3991	21.6909	21.6986	22.1719	21.704	21.9013
YARS	22.3007	22.5942	22.4622	22.3436	22.2414	22.2928	22.4044	22.3525	22.5577
YARS2	20.4903	20.3109	20.2101	19.9656	19.783	20.0105	20.6277	20.09	20.2575
YBX1	22.7798	20.126	22.3958	22.7439	22.5308	22.743	22.6046	22.3302	23.0276
YBX3	20.0071	18.3297	19.9917	19.8511	19.3788	19.7047	19.8149	18.8978	20.1437
YIPF3	19.0956	19.1675	18.7758	19.0757	18.4029	18.6849	17.8466	19.08	19.1671
YIPF6	15.1916	17.3768	16.5726	17.6646	17.1154	16.4639	15.8594	16.8187	17.2362
YKT6	21.7935	21.5789	21.409	21.7073	21.1421	21.1262	21.0641	21.1265	21.6165
YLPM1	19.8726	19.3722	19.2341	19.5211	19.178	19.4361	19.4061	19.5056	19.7644
YME1L1	19.0232	18.8224	19.2188	19.0547	18.8611	19.0178	19.3946	18.7775	19.1187
YRDC	19.2668	19.4076	19.2445	18.9475	18.9453	19.4458	19.1455	19.1192	19.2944
YTHDC1	17.5935	17.7264	18.155	18.2555	18.15	17.8784	18.1429	18.0595	18.2818
YTHDF2	19.8331	19.683	19.8077	19.61	19.4284	18.9486	19.2499	19.5344	19.7464
YWHAB	23.5091	23.7807	23.2521	23.5457	23.341	23.3748	23.2117	23.3299	23.7978
YWHAE	23.8671	21.6069	23.4823	23.8023	23.5497	23.2601	23.3819	23.4485	23.6267
YWHAG	22.6495	22.6608	22.7458	22.866	22.874	22.8882	23.3169	22.8492	23.304
YWHAH	21.9893	21.795	21.9675	22.2023	21.968	22.0103	22.006	21.9133	22.3926
YWHAQ	23.363	23.4546	23.1351	23.0683	23.2358	22.3561	22.6517	22.9234	23.2239
YWHAZ	25.8311	25.6109	25.5823	25.5654	25.4642	25.6354	25.8019	25.6191	25.7348
ZBTB34	20.9245	20.9783	20.5921	19.9046	20.8454	19.9485	20.7082	19.7684	19.9032
ZC3H11A	18.8294	17.923	18.4613	18.7424	18.3746	18.5975	18.5551	18.8151	18.8347
ZC3H13	18.5452	17.8999	17.3067	17.5128	17.0171	17.1017	17.0201	17.2697	17.1957

ZC3H14	19.1089	18.7952	19.4867	19.4866	19.1203	18.8756	18.8463	19.0418	19.259
ZC3H15	20.0961	19.8633	19.9314	19.7064	19.6424	19.8093	19.887	19.7801	20.0009
ZC3H18	18.257	17.0091	17.5848	18.4072	17.8064	18.0999	17.644	17.8705	18.3776
ZC3H4	19.226	17.2042	17.028	17.0375	16.9785	17.5006	16.8288	17.5334	17.5913
ZC3H7A	16.7857	16.6396	16.5635	16.132	16.0366	16.2699	17.204	16.6183	16.9169
ZC3HAV1	20.2763	20.2058	20.4008	20.0183	19.9357	20.186	20.3015	20.0155	20.1172
ZCCHC8	18.4423	20.5439	17.3057	17.3411	17.1284	17.0152	18.3881	17.4952	17.8055
ZFPL1	19.0188	19.1488	19.43	19.0083	19.0937	19.0747	19.2508	19.2649	19.2569
ZFR	19.5689	19.5546	19.5257	19.4344	19.196	19.5042	19.8193	19.3754	19.557
ZGPAT	20.0178	20.2696	20.0264	20.47	19.9716	19.4028	19.1606	19.5368	19.9599
ZMPSTE24	21.1682	21.0984	21.0897	21.0581	20.7959	20.5691	20.8617	20.7521	20.9382
ZMYND8	18.5238	18.4842	18.4492	18.441	18.3716	18.4959	18.7452	18.5312	18.1964
ZNF207	21.0543	21.025	21.0428	21.4335	21.0265	20.9019	21.1981	21.2108	21.4887
ZNF259	19.8917	17.5371	18.9154	19.7361	19.1106	20.0778	19.5712	19.8839	19.9358
ZNF326	19.6409	18.4734	19.5677	19.4798	19.4868	19.5032	19.5748	19.264	19.6355
ZNF622	19.5908	19.4509	19.6172	19.6629	19.5759	19.9082	19.3399	19.654	19.4872
ZNF638	17.4763	17.5887	17.6086	17.2928	17.2606	17.4795	17.6701	17.5963	17.8375
ZRANB2	19.8365	20.176	19.8562	19.9823	19.6859	20.3254	20.4938	19.9212	20.2134
ZYX	22.4171	21.5646	22.3421	22.5041	22.3395	22.978	23.0834	22.639	22.8275
