# Supplementary material for: The long non-coding RNA HOTAIRM1 promotes tumor aggressiveness and radiotherapy resistance in glioblastoma
Source: Cell Death Dis. 2021 Sep 28;12(10):885. doi: 10.1038/s41419-021-04146-0 (PMC8478910; doi:10.1038/s41419-021-04146-0)
Supplement: Supplementary file 17 — author contribution form [file 41419_2021_4146_MOESM17_ESM.pdf]

DECLARATION OF CONTRIBUTIONS TO ARTICLE

ADMC

Manuscript Number: CDDIS-20-2007

Journal Name: *Cell Death & Disease* (the 'Journal')

Proposed Title of the Contribution:

The long non-coding RNA HOTAIRM1 promotes tumor aggressiveness and radiotherapy resistance in glioblastoma (the 'Contribution)

Author(s):

Ulvi Ahmadov, Daniel Picard, Jasmin Bartl, Manuela Silginer, Marija Trajkovic-Arsic, Nan Qin, Alina Marie Winkelkotte, Marlen Melcher, Maike Langini, Lena Blümel, Viktoria Marquardt, Anja Stefanski, Sasha Steltgens, Christina Hassiepen, Anna Kaufhold, Frauke-Dorothee Meyer, Annette Seibt, Lara Kleinesudeik, Anika Hain, Carsten Münk, Christiane Brigitte Knobbe-Thomsen, Alexander Schramm, Ute Fischer, Gabriel Leprivier , Kai Stühler, Simone Fulda, Jens Siveke, Felix Distelmaier, Arndt Borkhardt, Michael Weller, Patrick Roth, Guido Reifenberger, Marc Remke

(the 'Authors')

For all *CDDis* articles, each person named as an author in the published version must be able to show he or she has contributed substantially to the article.

Authorship credit should be based on 1) substantial contributions to conception and design, acquisition of data, or analysis and interpretation of data; 2) drafting the article or revising it critically for important intellectual content; and 3) final approval of the version to be published. Authors should meet conditions 1, 2 and 3.

Any person who cannot be shown to have made a substantial contribution to the article cannot be listed as an author in the final version. The name of any person who is deemed to have made a minor contribution can, however, appear in the Acknowledgments section of the article.

Please complete the table below to indicate the contributions of all named authors to the manuscript.

Author Full Name:	Specification of Contribution to the Manuscript:
Ulvi Ahmadov	Conception and design, acquisition of data, and analysis and interpretation of data, drafting the article and revising it
Daniel Picard	Conception and design, acquisition of data, and analysis and interpretation of data, drafting the article and revising it
Jasmin Bartl	Conception and design, and analysis and interpretation of data, drafting the article and revising it
Manuela Silginer	Design, acquisition of data, and analysis and interpretation of data
Marija Trajkovic-Arsic	Contributing the manuscript with important intellectual content
Nan Qin	Contributing the manuscript with important intellectual content
Alina Marie Winkelkotte	Contributing the manuscript with important intellectual content
Marlen Melcher	Design, acquisition of data, and analysis and interpretation of data
Maike Langini	Design, acquisition of data, and analysis and interpretation of data
Lena Blümel	Design, acquisition of data, and analysis and interpretation of data
Viktoria Marquardt	Contributing the manuscript with important intellectual content
Anja Stefanski	Design, acquisition of data, and analysis and interpretation of data
Sasha Steltgens	Contributing the manuscript with important intellectual content

DECLARATION OF CONTRIBUTIONS TO ARTICLE

ADMC

Manuscript Number: CDDIS-20-2007

Journal Name: *Cell Death & Disease* (the 'Journal')

Proposed Title of the Contribution:

The long non-coding RNA HOTAIRM1 promotes tumor aggressiveness and radiotherapy resistance in glioblastoma (the 'Contribution)

Author(s):

Ulvi Ahmadov, Daniel Picard, Jasmin Bartl, Manuela Silginer, Marija Trajkovic-Arsic, Nan Qin, Alina Marie Winkelkotte, Marlen Melcher, Maike Langini, Lena Blümel, Viktoria Marquardt, Anja Stefanski, Sasha Steltgens, Christina Hassiepen, Anna Kaufhold, Frauke-Dorothee Meyer, Annette Seibt, Lara Kleinesudeik, Anika Hain, Carsten Münk, Christiane Brigitte Knobbe-Thomsen, Alexander Schramm, Ute Fischer, Gabriel Leprivier , Kai Stühler, Simone Fulda, Jens Siveke, Felix Distelmaier, Arndt Borkhardt, Michael Weller, Patrick Roth, Guido Reifenberger, Marc Remke (the 'Authors')

For all *CDDis* articles, each person named as an author in the published version must be able to show he or she has contributed substantially to the article.

Authorship credit should be based on 1) substantial contributions to conception and design, acquisition of data, or analysis and interpretation of data; 2) drafting the article or revising it critically for important intellectual content; and 3) final approval of the version to be published. Authors should meet conditions 1, 2 and 3.

Any person who cannot be shown to have made a substantial contribution to the article cannot be listed as an author in the final version. The name of any person who is deemed to have made a minor contribution can, however, appear in the Acknowledgments section of the article.

Please complete the table below to indicate the contributions of all named authors to the manuscript.

Author Full Name:	Specification of Contribution to the Manuscript:
Christina Hassiepen	Contributing the manuscript with important intellectual content
Anna Kaufhold	Acquisition and analysis of data
Frauke-Dorothee Meyer	Acquisition and analysis of data
Annette Seibt	Acquisition and analysis of data
Lara Kleinesudeik	Contributing the manuscript with important intellectual content
Anika Hain	Contributing the manuscript with important intellectual content
Carsten Münk	Contributing the manuscript with important intellectual content
Christiane Brigitte Knobbe-Thomsen	Contributing the manuscript with important intellectual content
Alexander Schramm	Contributing the manuscript with important intellectual content
Ute Fischer	Contributing the manuscript with important intellectual content
Gabriel Leprivier	Contributing the manuscript with important intellectual content
Kai Stühler	Contributing the manuscript with important intellectual content
Simone Fulda	Contributing the manuscript with important intellectual content

DECLARATION OF CONTRIBUTIONS TO ARTICLE

ADMC

Manuscript Number: CDDIS-20-2007R

Journal Name: *Cell Death & Disease* (the 'Journal')

Proposed Title of the Contribution:

The long non-coding RNA HOTAIRM1 promotes tumor aggressiveness and radiotherapy resistance in glioblastoma (the 'Contribution)

Author(s):

Ulvi Ahmadov, Daniel Picard, Jasmin Bartl, Manuela Silginer, Marija Trajkovic-Arsic, Nan Qin, Marietta Wolter, Jonathan K. M. Lim, David Pauck, Alina Marie Winkelkotte, Marlen Melcher, Maike Langini, Lena Blümel, Viktoria Marquardt, Felix Sahm, Anja Stefanski, Sasha Steltgens, Christina Hassiepen, Anna Kaufhold, Frauke-Dorothee Meyer, Annette Seibt, Lara Kleinesudeik, Anika Hain, Carsten Münk, Christiane Brigitte Knobbe-Thomsen, Alexander Schramm, Ute Fischer, Gabriel Leprivier , Kai Stühler, Simone Fulda, Jens Siveke, Felix Distelmaier, Arndt Borkhardt, Michael Weller, Patrick Roth, Guido Reifenberger, Marc Remke (the 'Authors')

For all *CDDis* articles, each person named as an author in the published version must be able to show he or she has contributed substantially to the article.

Authorship credit should be based on 1) substantial contributions to conception and design, acquisition of data, or analysis and interpretation of data; 2) drafting the article or revising it critically for important intellectual content; and 3) final approval of the version to be published. Authors should meet conditions 1, 2 and 3.

Any person who cannot be shown to have made a substantial contribution to the article cannot be listed as an author in the final version. The name of any person who is deemed to have made a minor contribution can, however, appear in the Acknowledgments section of the article.

Please complete the table below to indicate the contributions of all named authors to the manuscript.

Author Full Name:	Specification of Contribution to the Manuscript:
Jens Siveke	Revising the manuscript critically for important intellectual content; and doing the final approval of the version to be published
Felix Distelmaier	Revising the manuscript critically for important intellectual content; and doing the final approval of the version to be published
Arndt Borkhardt	Revising the manuscript critically for important intellectual content; and doing the final approval of the version to be published
Michael Weller	Revising the manuscript critically for important intellectual content; and doing the final approval of the version to be published
Patrick Roth	Revising the manuscript critically for important intellectual content; and doing the final approval of the version to be published
Guido Reifenberger	Revising the manuscript critically for important intellectual content; and doing the final approval of the version to be published
Marc Remke	Revising the manuscript critically for important intellectual content; and doing the final approval of the version to be published
Marietta Wolter	performed experiments for the manuscript revision
Jonathan K. M. Lim	performed experiments for the manuscript revision
David Pauck	performed experiments for the manuscript revision
Felix Sander	performed experiments for the manuscript revision

Please complete the table below to indicate the contributions of all named authors to the figures.

Figure 1:

DP acquired and analyzed the data, and prepared all the panels. AB, MW, PR, GR and MR did the revision and the final approval

Figure 2:

panel A-D: UA generated the data; UA, DP and JB designed and prepared all the panels. AB, MW, PR, GR and MR did the revision and the final approval

Figure 3:

panel A: AK and AS generated the data, and DP and ML analyzed the data, KS interpreted the data, DP prepared the panel; panel B: MM generated the data, MM and UA analyzed and prepared the panel, FD interpreted the data; panel C: UA generated the data, UA, DP and JB prepared the panel. AB, MW, PR, GR and MR did the revision and the final approval

Figure 4:

panel A-C: UA generated the data, UA and DP designed and analyzed the data; panel D-E: MS generated and analyzed the data, UA, DP, JB and MS prepared the panels. AB, MW, PR, GR and MR did the revision and the final approval

Figure 5:

panel A: UA generated the data, NQ, UA and DP designed and analyzed the data; panel B-C: LB and UA generated the data, UA and DP analyzed the data; panel C: UA and DP prepared the panel. AB, MW, PR, GR and MR did the revision and the final approval

Figure 6:

panel A-C: UA generated the data; UA, DP and JB designed and prepared all the panels. AB, MW, PR, GR and MR did the revision and the final approval

Signed for and on behalf of the Author(s):	Print Name:	Date:
Marc Remke	Marc Remke	26-06-2020
